# Bony labyrinth morphology clarifies the origin and evolution of deer

**DOI:** 10.1038/s41598-017-12848-9

**Published:** 2017-10-13

**Authors:** Bastien Mennecart, Daniel DeMiguel, Faysal Bibi, Gertrud E. Rössner, Grégoire Métais, James M. Neenan, Shiqi Wang, Georg Schulz, Bert Müller, Loïc Costeur

**Affiliations:** 1 Nathurhistorisches Museum Basel, Augustinergasse 2, 4001 Basel, Switzerland; 2 ICTA-ICP, Edifici Z, c/de les columnes s/n, Universitat Autònoma de Barcelona, 08193 Cerdanyola del Vallès, Barcelona, Spain; 3 Departamento de Ciencias de la Tierra, Área de Paleontología.Universidad de Zaragoza, Pedro Cerbuna 12, 50009 Zaragoza, Spain; 4 Fundación ARAID, Zaragoza, Spain; 5 Museum für Naturkunde Berlin, Leibniz Institute for Evolution and Biodiversity Science Invalidenstraße 43, 10115 Berlin, Germany; 6 Bayerische Staatssammlung für Paläontologie und Geologie, Richard-Wagner-Strasse 10 80333 Munich, Germany; 7 CR2P - Centre de Recherches sur la Paléobiodiversité et les Paléoenvironnements, UMR 7207, Muséum National d’Histoire Naturelle, CNRS, UPMC, Sorbonne Universités. MNHN, CP38, 8 rue Buffon, 75005 Paris, France; 8 Oxford University Museum of Natural History, Parks Road, Oxford, OX1 3PW United Kingdom; 9 Institute of Vertebrate Paleontology and Paleoanthropology, Chinese Academy of Sciences, 142 Xizhimenwai Street, Beijing, 100044 China; 10 University of Basel, Biomaterials Science Center, Department of Biomedical Engineering, Gewerbestrasse 14, 4123 Allschwil, Switzerland

## Abstract

Deer are an iconic group of large mammals that originated in the Early Miocene of Eurasia (ca. 19 Ma). While there is some consensus on key relationships among their members, on the basis of molecular- or morphology-based analyses, or combined approaches, many questions remain, and the bony labyrinth has shown considerable potential for the phylogenetics of this and other groups. Here we examine its shape in 29 species of living and fossil deer using 3D geometric morphometrics and cladistics. We clarify several issues of the origin and evolution of cervids. Our results give new age estimates at different nodes of the tree and provide for the first time a clear distinction of stem and crown Cervidae. We unambiguously attribute the fossil *Euprox furcatus* (13.8 Ma) to crown Cervidae, pushing back the origin of crown deer to (at least) 4 Ma. Furthermore, we show that Capreolinae are more variable in bony labyrinth shape than Cervinae and confirm for the first time the monophyly of the Old World Capreolinae (including the Chinese water deer *Hydropotes*) based on morphological characters only. Finally, we provide evidence to support the sister group relationship of *Megaloceros giganteus* with the fallow deer *Dama*.

## Introduction

Deer (Cervidae) are a family of antlered ruminants and, with 55 extant species, are one of the most diverse groups of artiodactyls. They are adapted to inhabit numerous climatic zones and ecotones on all continents, with the exception of Oceania and Antarctica^1,2^. In the last decade, molecular analyses have provided new input for a relative consensus of the phylogeny of extant taxa^2–7^. However, morphology-based analyses that allow the integration of extinct taxa and lead to a deeper understanding of the evolutionary history of the whole group do not always yield similar results for cervid phylogenetics^8–11^. While the distinction between the extant subfamilies Capreolinae and Cervinae is morphologically distinguishable by the condition of the lateral digits (distal part preserved only or “telemetacarpal” vs. proximal part preserved only or “plesiometacarpal”, respectively), the phylogenetic position and the genus-level relationships of many fossil species are still debated (see Grubb^12^ and Croitor^13^ for Plio-Pleistocene taxa). This has a strong effect on the ages used to calibrate deer phylogeny and, indeed, ruminant phylogeny as a whole. A classic uncertainty is the origin of the crown clade, as molecular-based approaches use the oldest occurrence of the extant *Muntiacus* (7 to 10 million years ago (Ma), Late Miocene^3,7,14^), whereas palaeontologists embrace *Euprox furcatus* (a Middle Miocene deer dated ca. 13.8 Ma, 4 to almost 7 million of years (My) older than the earliest *Muntiacus*) as the oldest crown deer and even as a member of the Muntiacinae^15–18^. Resolving these incongruences is therefore crucial for understanding the origin and evolutionary history of deer.

Antlers are classic morphological features of deer, and are paramount characters for the inclusion of fossil representatives in phylogenetic analyses^19^, having appeared in the fossil record ca. 19 Ma^9,16,20–23^. Antlers are deciduous cranial appendages that have developed a wide array of morphologies throughout the history of deer; from tiny multitomous antlers in *Lagomeryx* to small pointed spines in *Pudu* and huge spiked shovel-shaped structures in the extinct “Irish Elk”, *Megaloceros*, and the recent moose, *Alces*. Despite their good fossil record, studies based on antlers and other body parts have left many issues in deer phylogeny unresolved. The bony labyrinth (i.e., the organ of hearing and balance) has been recently shown to be an informative structure reflecting phylogenetic relationships in accordance with molecular-based hypotheses in various mammal groups e.g.^24–30^. Because molecular data for extinct species are unavailable, reconstructing the three-dimensional (3D) morphology of this structure throughout the history of a lineage thus has the potential to solve key questions regarding the origins and diversification of clades.

Here we reconstruct the bony labyrinth of 29 extant (*N* = 12) and extinct (*N* = 17) deer species spanning the 19 Ma of their evolutionary history (Supplementary data 1). We use a 3D geometric morphometric approach and the most comprehensive cladistic analysis (including fossil deer) to tackle phylogenetic issues at all levels of the tree. Stem taxa as well as key fossil species for the main tribes are included. Our analysis *i*) unquestionably separates stem from crown cervids; *ii*) sets the origin of crown deer earlier than previously proposed by molecular phylogenetic analyses and, in line with palaeontological data, confirms the position of *Megaloceros* in the *Dama* lineage; *iii*) adds data to the peculiar morphological disparity of New World cervids; *iv*) recalibrates the phylogenetic tree of cervids; and *v*) confirms the high potential of the bony labyrinth for resolving conflicting phylogenies in mammals, such as the phylogenetic position of the inermous *Hydropotes*.

## Results

### Principal Component Analysis of stem and crown Cervidae

**Figure 1:**
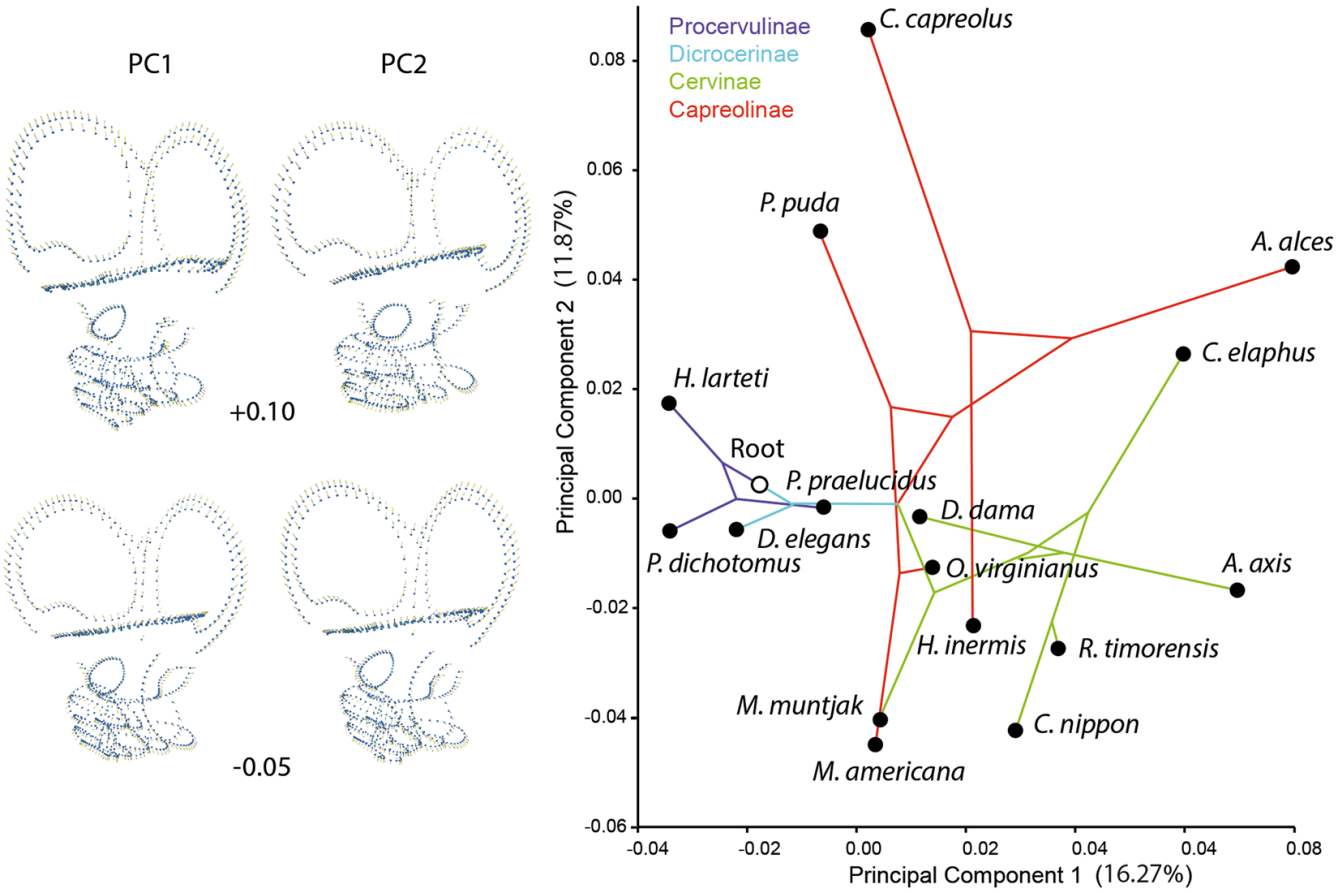
Principal component analysis (PCA) based on the 3D coordinates of the cervid bony labyrinth morphology with superimposed phylogenetic tree. PC shape variation is highighted by the hypothetical bony labyrinth shapes at the extreme scores −0.05 and +0.10 on PC1 and PC2.

Shape variation of the bony labyrinth among cervids is presented in Fig. 1. The p-value resulting from the permutation test, which tested the influence of the phylogenetic signal on the shape variation, is not significant (p-value = 0.3586). This indicates that we cannot reject the hypothesis that overall shape variation, across the tips of the tree is random. Nevertheless, the morphospace occupied by the bony labyrinth morphology of stem Cervidae differs from those of crown Cervidae along Principal Component 1 (PC1), with no overlap being observed between these groups (Fig. 1). The morphospace of extant Capreolinae is larger than that of the stem Cervidae and the extant Cervinae, indicating a higher variation in the shape of the bony labyrinth in the former clade (Fig. 1). In addition, there is no overlap between the Old World (*Capreolus capreolus*, *Hydropotes inermis*, and *Alces alces*) and the New World (*Odocoileus virginianus*, *Mazama americana*, and *Pudu puda*) Capreolinae, with the American ones being shifted toward stem Cervidae along the PC1 (Fig. 1).

### Canonical Variate Analysis of subfamilies

**Figure 2:**
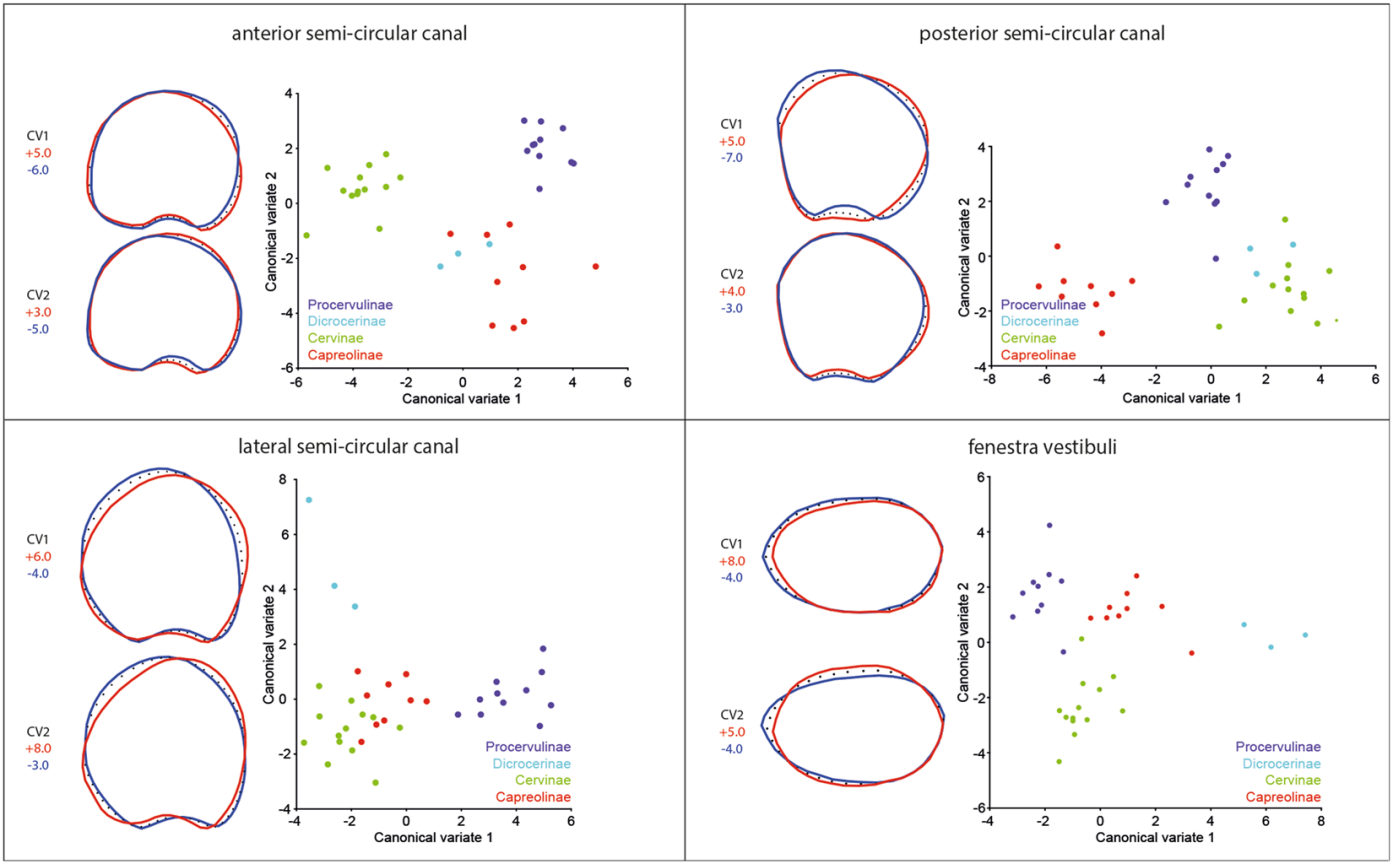
Canonical variate analyses (CVA) of the different structures of the bony labyrinth (semi-circular canals, fenestra vestibuli) to maximise the similarities among subfamilies (Procervulinae, Dicrocerinae, Cervinae, and Capreolinae). The red outline corresponds to the CV shape variation at the positive extreme score and the blue outline to the negative extreme score.

The Canonical Variate Analysis (CVA) statistically highlights discrimination between deer subfamilies based on bony labyrinth structures (Supplementary data 2). The discrimination of the Dicrocerinae is systematically less-pronounced than the other sub families due to the low number of specimens (Supplementary data 2). Nevertheless, the six CVA applied to specific areas of the cervid bony labyrinth show graphically the phylogenetically relevant structures allowing distinction between subfamilies when possible (Fig. 2). The following characters are continuous based on deduced measurements. Supplementary data 2 shows how the various measurements and ratios have been performed and Supplementary data 3 explains the terminology used in the following text and the cladistics analyses based on the bony labyrinth sole. Canonical Variate 1 (CV1) of the anterior semi-circular canal analysis allows a clear distinction of Procervulinae, Dicrocerinae, and Capreolinae from Cervinae, with the former having a rounded structure and the latter a squarer canal (Fig. 2). CV2 separates Cervinae and Procervulinae from Capreolinae and Dicrocerinae by a more anteriorly ovoid anterior canal, with the exception of *Dama dama* and *Metacervocerus philisi* (second morphotype) which show a more rounded canal. Moreover, Cervinae and Procervulinae have a more flattened anterior ampulla (Fig. 2). The posterior semi-circular canal is ovoid posteriorly in the Capreolinae, rounded in Procervulinae, and ovoid anteriorly in Dicrocerinae and Cervinae (except for *Rusa timorensis*). This canal is wider than high only in Procervulinae (Fig. 2). The posterior ampulla is strongly rounded in Capreolinae, rounded in Procervulinae, and flattened in Dicrocerinae and Cervinae (with the exception of *R. timorensis*). The lateral semi-circular canal is ovoid posteriorly in Capreolinae and Cervinae, while it is ovoid anteriorly in Procervulinae and Dicrocerinae (Fig. 2). Similarly, the lateral ampulla is flattened in Capreolinae and Cervinae and rounded in Procervulinae and Dicrocerinae. The fenestra vestibuli is elongated and narrow in Cervinae (stapedial ratio above 1.557), while it is more rounded in the remaining Cervidae (except in *O. virginianus*). The beginning of the first turn of the cochlea is narrow in Capreolinae and is rather enlarged in the other Cervidae. The thickness of the cochlear first turn is asymmetrical in Cervinae, while it remains symmetrical in the remaining Cervidae. The coil of the cochlear second turn is tightened in Cervinae and enlarged in the other Cervidae. Finally, the second turn of the cochlea is flattened in the Capreolinae and thick in the other Cervidae.

### Cladistic analyses

**Figure 3:**
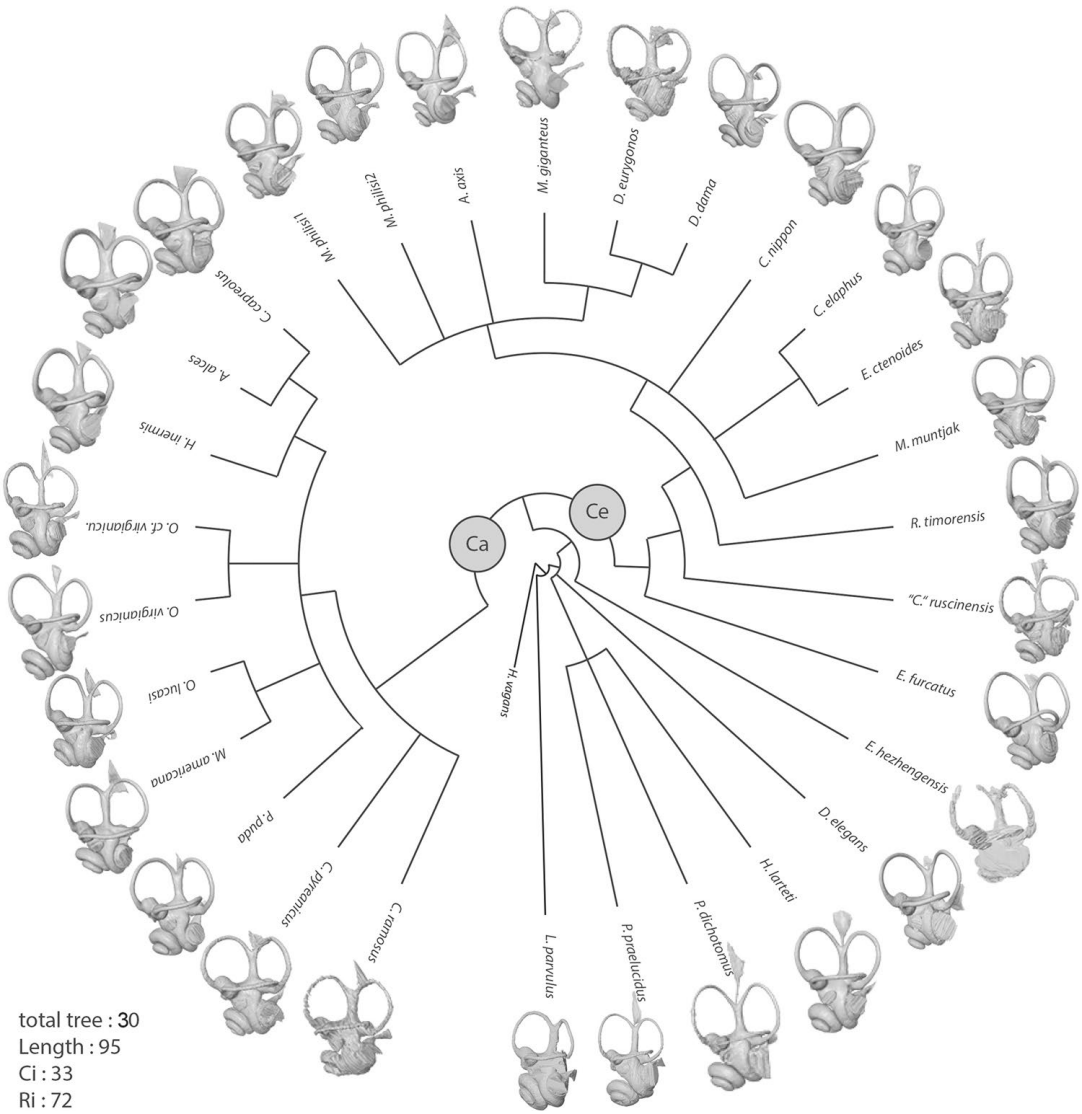
Phylogenetic tree of selected Cervidae based on 26 characters of the bony labyrinth (see Supplementary Information 3 for the description of the characters and character states and for the phylogenetic tree with the associated characters) using maximum an Euristic analysis and the Nelson consensus compromise (collaps + consensus) (Ci = 0.33, Ri = 0.72).

Supplementary data 3 depicts the characters and character states on each node. The Nelson consensus compromise (collapse + consensus) tree of 30 most parsimonious trees shows a highly homoplasic and well-structured result (Consistency Index = 0.33, Retention Index = 0.72, see Fig. 3). Precise description of the topology of the tree and character distribution are found in Supplementary data 3.

### Calibrated tree

**Figure 4:**
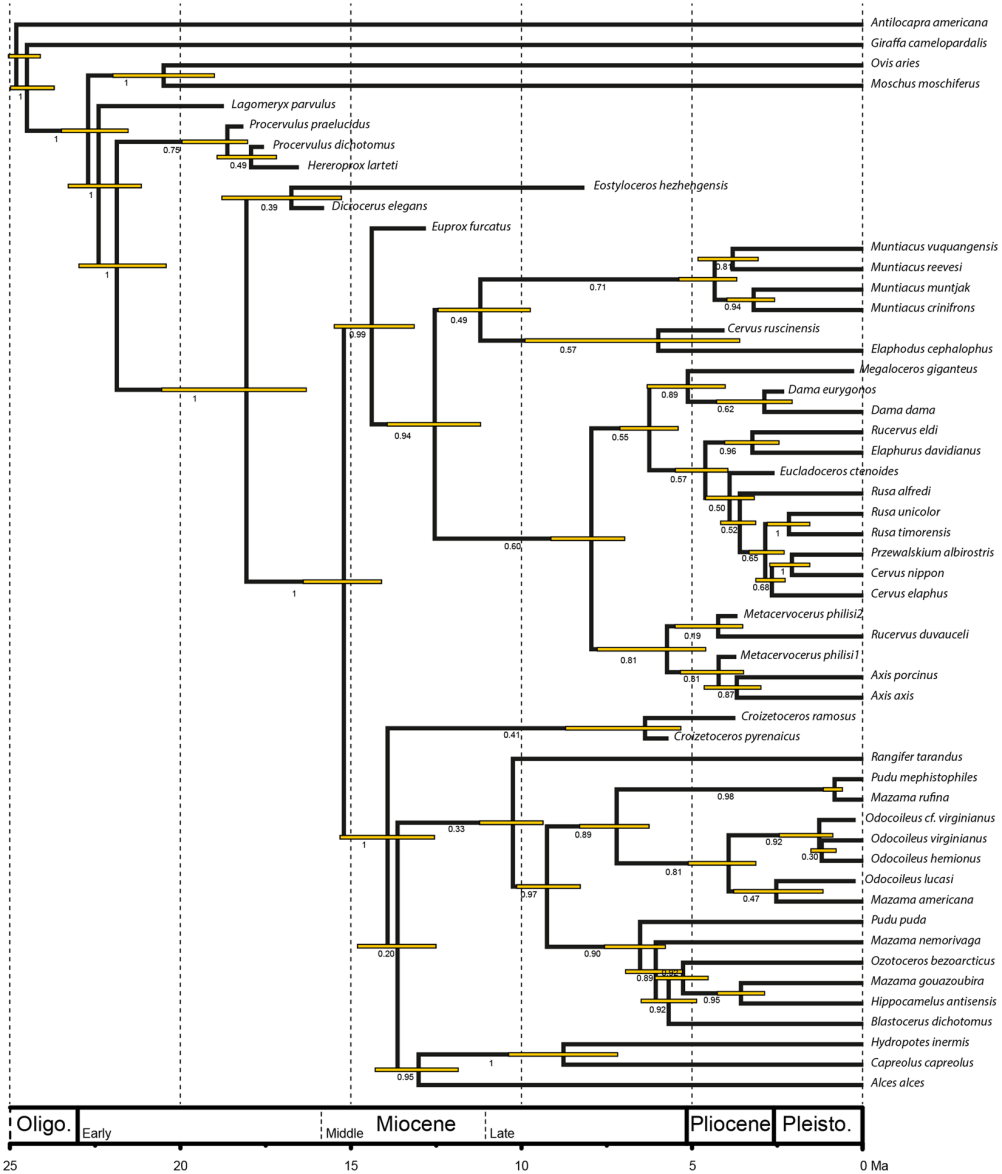
Calibrated ruminant tree (see Supplementary Information 4 for the method and the results).

Despite the relatively small size of the dataset, visual inspection of the logs in Tracer v.1.6 showed that none of the analyses appeared to be reaching stationarity, with fluctuating parameters and extremely small effective sample sizes for some parameters. We believe this is because of the small size of the morphological dataset (26 characters, compared to a molecular dataset of >16,000 base pairs for most taxa), from which molecular rates had to be calculated for the whole tree. The uncertainty in the tree is also indicated by the very low posterior probability values on nodes that were not constrained. Nevertheless, repeated runs of 10 millions replications consistently yielded the same topology and similar divergence dates (even when certain parameters were changed, such as using a BDSKY tree model). This shows that the results presented here, while beset with uncertainty, are at least consistently reproducible with the current dataset. Figure 4 is one of the resulting calibrated trees. Precise description of the results are found in Supplementary data 4.

## Discussion

The tree is structured by different groups of characters depending on the taxonomic level considered. All the characters of the cochlea (1, 6, 7, 24, 25, 26) contribute to the basic structure of the tree (main clades: *Lagomeryx*/more derived Cervidae; stem Cervidae/crown Cervidae, Capreolinae/Cervinae). Mennecart and Costeur^29^ and Costeur *et al*.^31^ revealed the ossification sequence of the bony labyrinth in different foetal stages of ruminants (Tragulidae and Bovidae). With the exception of structures that open in the brain cavity (vestibular aqueduct/endolymphatic sac and cochear aqueduct), the bony labyrinth mainly ossifies within three weeks, around mid gestation (Costeur *et al*.^31^). The cochlea and thus the pars cochlearis of the petrosal bone is the first part to ossify in the bony labyrinth^31^. The ossification timing differs from the developmental timing of the membranous inner ear (only known in humans) where the anterior, posterior, and lateral semicircular ducts are formed in that order, before the final coiling of the cochlea^32^. Nevertheles, the anterior canal (17 and 18) is the first fully ossified canal^29^. It contributes in structuring the tree at the subfamily level (i.e., Capreolinae and Cervinae). Terminal clades (*O. lucasi* + *M. americana*, *O. virginianus* + *O*. cf. *virginianus*, *E. ctenoides* + *C. elaphus*, *M. giganteus* + *D. eurygonos* + *D. dama*) are principally defined by characters of the endolymphatic sac (4, 9) and vestibular aqueduct (3, 8) both ossifying at last in the bony labyrinth^29,31^. The cochlear aqueduct still continues to grow after birth (Costeur *et al*.^31^). Characters of this structure (10, 11) are shown to distinguish closely related species (e.g. *M. giganteus*/*D. dama* lineage, *A. axis*/*M. philisi*, *M. americana*/*O. lucasi*). We show here the importance of reconstructing these structures precisely in a phylogenetic context.

Despite extensive data on well-studied fossils such as those of the putative Middle Miocene Muntiacini *E. furcatus*, calibrations of the origin of crown deer performed by molecular biologists often rely on fossil specimens attributed to extant genera (e.g. *Muntiacus* ^3,7,14^). Our identification of *E. furcatus* as a member of stem Cervinae allows us to recalibrate the cervid tree, as well as the timing of cladogenesis within the whole family (see Fig. 4). Our results indicate that crown Cervidae originated at least ca. 4 to 8 My earlier than previously estimated (from 8.5 Ma^14^ to 10.7 Ma^7^) at ca. 15.2 Ma. Similarly, the origin of the cervid subfamilies at crown level is suggested to be older than previously estimated. With an appearance at ca. 12.5 Ma for crown Cervinae and ca. 13.6 Ma for crown Capreolinae, respectively (Fig. 4), we set the origin of these clades ca. 4 to 5 My earlier (previous estimations lie between 7 and 9 Ma^3,7,33^) in the late Middle Miocene. Unfortunately, the cervid fossil record remains scarce for this period, especially in Asia where they originated. *Cervavitus* and *Cervocerus* from the Late Miocene (10 and 7 Ma, respectively) of Asia, the oldest known members of these clades, are either discussed as potential stem capreolines and cervines^9^ or even crown taxa^34^. Similarly, the estimation of the origin and diversification of the extant tribes is pushed back from 2 to 4 Ma in comparison to previous studies (see Fig. 4), occurring during the latest Miocene and Pliocene from ca. 6.5 to 3 Ma. This corresponds to a global climatic transition between the warm Late Miocene towards the onset of the Northern hemisphere glaciations^35^. In strong agreement with Duarte *et al*.^36^ and Hassanin *et al*.^7^, we find evidence for an early origin of the South American deer (early Late Miocene) long before the formation of the Panamian Isthmus. The oldest evidences of New World Cervidae are *Eocoileus gentryorum*, *Odocoileus* sp. and *Bretzia pseudalces* from the late Hemphillian, ca. 5 Ma^37,38^. These ruminants are rare and uncommon in Pliocene faunas^39^. However, their lineages were very successful, giving birth to several fossil genera (e.g. *Navahoceros*, *Sangamona*, *Agalmoceros*, *Morenelaphus*) and to the entire diversity of the extant South American Capreolinae^1,37^.

As a result of the cervid tree branch calibration, we propose new ages at different hierarchical levels of the ruminant tree, which are in some instances confirmed by earlier studies. The divergence of crown Pecora is estimated between 25 and 24 Ma, which corresponds to a peak of diversification of inermous pecoran ruminants of uncertain taxonomical affinities, first in Asia and then in Europe and North America^40,41^.

There is little consensus for the identification of the oldest stem deer^8,9,16,20,21^. It is mostly related to the presence of antlers in general and to their morphology. An array of Early and Middle Miocene Old World cervids bore branched cranial appendages similar to extant antlers but with a different morphology (among others, no beam and no burr)^19^. The identification of diagnostic characters for antlers has posed many problems. Evidence for deciduousness and regeneration of early “antlers”, fundamental features unique to deer today, add to the complexity^16,19,21^. In addition, some extant deer can *i*) lack antlers (Chinese water deer), *ii*) possess highly reduced antlers (tufted deer), or *iii*) show very tiny unbranched antlers (the *Pudu*). It stresses the high phenotypic plasticity and possible morphological convergences of this type of cranial appendage. “Protoantler” bearing taxa have often been hypothesised to be in their own family closely related to Cervidae, as for *Lagomeryx* or *Ligeromeryx* (Lagomerycidae e.g.,^23^), in different stem deer subfamilies, such as *Procervulus* and *Heteroprox* (Procervulinae^16,20^), and *Dicrocerus, Acteocemas*, or *Stephanocemas* (Dicrocerinae^20^), or even in crown deer, as again in *Lagomeryx* e.g.,^8^. Our phylogenetic analysis provides strong support that *Lagomeryx*, Procervulinae, and Dicrocerinae are not crown Cervidae, as preliminarily shown in Mennecart *et al*.^30^. However, the sister position of *L. parvulus* to all other cervids under study retrieved here prevents us from drawing any firm conclusion on its membership to the Cervidae lineage. Our shape analysis combined with the phylogenetic hypothesis unveil the modifications of the bony labyrinth that occur in deer evolution, such as thicker cochleas (character 6) and bulged lateral ampullas (character 21) in stem deer, becoming thinner and more flattened, respectively in crown species.

A surprising result is the stem position of the Late Miocene *E. hezhengensis*, which markedly differs from the general acceptance of a phylogenetic position deeply rooted within the Muntiacinae^18,20,42–44^—though all these analyses are mostly based on the shape of antlers. In agreement with our hypothesis, a recent phylogenetic analysis^18^ also places *Eostyloceros blainvillei* in an intermediate position between *D. elegans* and *Muntiacus*, the only crown cervid considered by the authors, thus preventing any firm conclusion on its crown or stem position. In addition, Deng *et al*.^44^ suggest a paraphyly of the genus *Eostyloceros* and exclude *E. hezhengensis* and all the species of *Eostyloceros* from the crown Cervidae. Despite the results of their phylogenetic analyses, the authors still consider *Eostyloceros* as a Muntiacinae due to its antler shape^18,44^. Our results confirm the published phylogenetic trees but firmly excludes *Eostyloceros* from crown Cervidae, and questions the widespread use of antler characteristics in phylogeny. Thus, stem deer may well have been still present during the Late Miocene in Asia.

Most molecular systematic analyses use the first appearance of the living muntjac’s genus *Muntiacus* (*Muntiacus noringenensis* at 9 Ma^43^) to calibrate the origin of crown deer e.g.,^3,7,14^. However, palaeontologists have proposed *Euprox* as the earliest crown deer— or even nested within Muntiacinae^15–18,20,42,43,45^. The first appearance of the genus (*Euprox minimus*) at ca. 14.5+/− 0.3 Ma is recorded in the Middle Miocene Austrian locality of Göriach^46^. Our phylogenetic analysis unambiguously proposes the hypothesis of *E. furcatus* as a stem Cervinae. Several characters (a rounded posterior ampulla, an elongated fenestra vestibuli, and a tightly coiled second cochlear turn) shared by *E. furcatus* and all the other analysed Cervinae support this hypothesis. Interestingly, its purported relationship with *Muntiacus* is not recovered. The single common character linking *Euprox* to other Muntiacinae is the strong backward inclination of the antlers’ pedicles also observed in *Eostyloceros* ^16,20^. This character is thus symplesiomorphic for crown deer. More recent phylogenetic analyses mentioned above^18,44^ do not seem to confirm the existence of a Muntiacinae clade that includes both *Euprox* and *Eostyloceros*, which is here (*E. hezhengensis*) excluded from Muntiacinae too and even from crown Cervidae. Several characters used as synapomorphies for Muntiacinae in these works have to be considered as symplesiomorphic for the crown Cervidae (e.g., true burr or centripetal mineralization of the antlers) and should not be used to define a subfamily or a tribe. *E. furcatus* is thus nested at the base of Cervinae pushing back the calibration of crown Cervidae by at least 4 My.

Our data provide new input for both the understanding and support of previous hypotheses on Plio-Pleistocene deer^13^. Dong^47^ considers the enigmatic Early Pliocene “*C*.” *ruscinensis* as a probable stem Cervidae, sister taxon to the poorly diagnosed Pliocervinae (see below and Croitor^13^). The result of our phylogenetic analysis places it within the stem Cervinae, in a more derived position than *E. furcatus*. The phylogenetic position of *E. ctenoides* within Cervini has often been discussed e.g.,^48^. Croitor^13^ indicates that species of this genus probably do not belong to the lineage of *Przewalskium-Rusa-Cervus* but does not conclude on any closer affinities with other deer taxa. In addition, even if *Eucladoceros* is not included into the Megacerines (sensu Vislobokova^49^), a close relationship with this group has been proposed^10^. Symplesiomorphic characters and a common evolutionary trend towards large size and heavy antlers could account for this^49^. Here we support close affinities of *E. ctenoides* from Senèze (in France) with *C. elaphus*. However, the well-defined *Rusa*-*Cervus* lineage is not clearly recovered in our analysis. Indeed, the insular species *R. timorensis*, which is considered to be more basal in our phylogenetic analysis, and *C. nippon*, included in a basal polytomy that includes all the Cervinae except *R. timorensis*, does not directly cluster with *C. elaphus*. Also, the position of *R. timorensis* and *C. nippon* in the bony labyrinth morphospace is very similar, distant from that of *C. elaphus*. *C. nippon* mainly differs from *C. elaphus* based on characters of the endolymphatic sac. Although nothing is yet known about how the bony labyrinth of ruminants evolves in an insular context (e.g. *R. timorensis* and possibly *C. nippon*), pressure release is known to induce significant morphological changes in the sense organs^50^. Accordingly, significant changes in the inner ear and bony labyrinth are also expected to occur. *M. philisi* is considered by Croitor^51^ as a sister taxon of the extant genus *Axis* based on cranial characteristics, while Valli^48^ and Pfeiffer^10^ found a closer relationship with *Dama* based on postcranial bones. Breda & Lister^52^ even attributed this species to the genus *Pseudodama*. The shape and position of the endolymphatic sac in *M. philisi* specimens is very similar to the conditions observed in *Axis*, being triangular in shape and starting at the level of the common crus. The relationships of *Metacervocerus* with *Dama* or *Axis* are not solved in our phylogenetic analysis and more than one species attributed to *Metacervocerus* could be present in Senèze. Indeed, the two *Metacervocerus* bony labyrinths under study display significant differences in orientation of the vestibular aqueduct recalling the *Dama* lineage. *Croizetoceros pyreanicus* and *Croizetoceros ramosus* have a very similar bony labyrinth, thereby confirming a close relationship^13,20,47^. They cluster together and are placed in a sister position to the crown Capreolinae. Their phylogenetic position has been discussed together with that of the extinct Pliocervinae because some raised arguments for the pliocervine *Damacerus* being their ancestor^13^. The Pliocervinae, observed to be holometacarpal deer (i.e., lateral digit full retention, a plesiomorphic cervid character), have a long history of debated affinities and could be a polyphyletic group containing early representatives of the Cervinae lineage, as well as Capreolinae and stem Cervidae (see e.g., Grubb^12^, Dong^34^, Croitor^13^ for competing opinions). In contrast, *Croizetoceros* is a plesiometacarpal deer^48,53^, but possible convergences in the reduction of the lateral digits of the foot and hand may have occurred^13,15^. *C. pyreanicus* emerges therefore as one of the oldest recognised Capreolinae, being already present in the Late Miocene of Spain^13,20^. In addition Croitor^13^ synonymises Pliocervinae with Capreolinae thus leaving a tribe Pliocervini within the Capreolinae subfamily, thus adding to the complex picture of the affinities of Plio-Pleistocene deer. A definitive conclusion about *Croizetoceros* cannot be proposed here because of the small number of characters involved in its position.

Excluding the enigmatic dromomerycine *Surameryx acrensis* (from the Late Miocene of Bolivia/Brazil, ca. 9 Ma^54^), Capreolinae are the only ruminants that have colonised South America. This could partly explain their prominent morphological and ecological diversity^1,37^. At least 12 species of South American Capreolinae are currently described, which constitutes over 20% of the whole diversity of Cervidae^2,36^. The basal polytomy of New World Capreolinae reflects the huge shape diversity of the bony labyrinth highlighted in Fig. 1. Bony labyrinth morphology of Old World Capreolinae differs from that of New World ones. This morphological shift of the bony labyrinth may be related to the release of ecological pressure or an ecological readjustement after the colonisation of America. The evolution of South American deer with the presence of ambush predator only “fosters low rates of reproduction, tiny young, low neonatal investment, and small adult body size” (sic. Geist^1^), that recalls insular evolutionary pressure and release of ecological constraints. Based on DNA data, *Mazama* seems to be a polyphyletic genus^2,7,36,55^. Interestingly, the species *M. americana* is part of the *Odocoileus* lineage, which coincides with earlier works^7,14,36^. Escobedo-Morales *et al*.^55^ and Heckeberg *et al*.^2^ even found evidence that some *Mazama* species are deeply nested within Odocoileini. In our tree *M. americana* clusters with the investigated specimen of *O. lucasi* from the Pleistocene (skull described by Rössner^56^) adding some more weight to the previous hypotheses. *O. virginianus* and *O*. cf. *virginianus* cluster together but some differences can be observed between the two specimens. Contrary to Heckeberg and Rössner^57^, we cannot support the view that they represent the same species and we suggest instead a sister relationship for them.

All the Old World Capreolinae (*H. inermis*, *C. capreolus*, and *A. alces*) cluster together, as already evidenced by numerous DNA analyses e.g.,^2,3,6,7,58^. The Chinese water deer *H. inermis* is the only antlerless deer, but with enormous upper canines instead. Its phylogenetic position has long been discussed since morphology, behaviour, and molecular markers led to contrasting hypotheses e.g.,^2,7,8,11,58–60^. Its primitive morphology and certain behavioural features (also found in tragulids, a sister group to pecoran ruminants) have led many of the aforementioned authors to propose *Hydropotes* as the sister taxon to antlered Cervidae. Only recent molecular data-based analyses unambiguously relate it to the Old World Capreolinae, close to the roe deer *C. capreolus* ^2,3,6,7,58^. Its telemetacarpal condition and its post-glenoid foramen support this hypothesis and strongly suggests that antlers were secondarily lost in the Chinese water deer. Our phylogenetic analysis places the bony labyrinth of *H. inermis* among those of the crown Capreolinae (which is supported by six characters) as a sister taxon of the Old World Capreolinae *A. alces* and *C. capreolus*. The latter monophyletic clade is supported by three characters (a symmetrical endolymphatic sac, a curved cochlear aqueduct, and an undulating posterior canal), with *Hydropotes* being excluded from the *Alces-Capreolus* group by a shorter cochlear aqueduct only. The bony labyrinth adds to the very few morphological features that relate *Hydropotes* to the Old World Capreolinae and confirms the results obtained from molecular data.

The phylogenetic position of the giant deer *M. giganteus* within the Cervinae is corroborated by both morphological and molecular analyses e.g.,^1,5,6,61,62^. The oldest Megacerine (and therefore crown Cervini) corresponds to the Late Miocene *Praesinomegaceros* (Tortonian, more than 7.25 Ma^49,62^). There is a growing consensus on the relationships of *Megaloceros* and the fallow deer *Dama* based on molecular and morphological data^1,5,6,63^. Some results had been obtained from these approaches that linked it to the *Cervus* lineage^10,61^, e.g., Kuehn *et al*.^61^ suggested that *M. giganteus* is conspecific to *C. elaphus*, but this result is probably due to modern contamination of the DNA samples. In our analysis, *Dama* species and *Megaloceros* share a similar cochlear aqueduct and endolymphatic sac and cluster together. Our results also support that *D. eurygonos* from Val d’Arno (ca. 1.5 Ma) belongs to an archaic lineage of fallow deer as suggested by Croitor^51^.

## Material and Methods

### Material

We used 49 specimens (see Supplemenatry data 1 for information about their origin, age, host institution, and scanning parameters) including Miocene (25 specimens from 7 species), Pliocene (2 specimens from 2 species), and Pleistocene (10 specimens from 8 species) deer from Europe, Northern America, and Asia, and extant (12 specimens from 10 genera and 12 species) deer. Our dataset therefore comprises more than half of the current cervid diversity (for a total of 17^64^, 18^59^, or 19^7^ genera). The fossil species were selected in order to encompass two of the oldest known ruminants possessing deciduous cranial appendages (*Lagomeryx parvulus* and *Procervulus praelucidus* ^22^) and most of the Plio-Pleistocene genera, which are part of the Cervidae radiation during glacial episods. The nomenclature of Plio-Pleistocene fossils here used follows Croitor^51^. The terminology of the bony labyrinth follows Ekdale^26^, Macrini^27^, and Mennecart & Costeur^28^. The 3D data are available from the corresponding author on reasonable request.

### Geometric morphometrics analyses

Left bony labyrinths were preferably selected when available. If not, the right one was included for analysis using the reflect application of Landmark Editor 3.6 software^65^. Digitizing of the specimens was also performed using this software. 77 curves of 10 semi landmarks and 1 landmark were digitised on the surface of the specimens following the protocol described in Mennecart and Costeur^29^. Shape variation in bony labyrinth morphology (disparity and similarity) was studied using the geometric morphometrics methods implemented in MorphoJ 1.06d software^66^. Principal component analysis is used to visualise the overall shape variation among specimens. It encompasses 3 species of Procervulinae (11 specimens), 1 species of Dicrocerinae (3 specimens), 6 living species of Capreolinae (7 specimens), and 6 living species of Cervinae (6 specimens) for which we have a phylogenetic control^7,30^. We included several specimens of fossil species as a control on the taxonomy and to increase the number of observations in the predefined groups of the following ordination method. A permutation test^67^ was performed to test the presence or absence of a phylogenetic signal in the overall shape of the bony labyrinth (randomised rounds: 1000), that could be assimilated to phenetics^29^. Mennecart & Costeur^29^ demonstrated, using this methodology, that intraspecific variability is lower than interspecific variation, and may be phylogenetically informative. This observation has been confirmed in many mammal groups (e.g. cetaceans^68,69^; primates^70^; primitive artiodactyls^71^) except for sloths, which exhibit extremely slow locomotion^72^.Thus, the hypothetical specimen mean (consensus of all specimens from a species) was used for the phylogenetic test to not artificially decrease the homoplasy. The phylogenetic tree was created using Mesquite 3.04 software^73^. Klingenberg & Gidaszewski^67^ remind us that geometric morphometrics results cannot be directly used as phylogenetic characters. An ordination method and splitting the shape into a set of multiple characters are methods already used (see Klingenberg & Gidaszewski^67^ for an exhaustive review). Mennecart & Costeur^28^ and Mennecart *et al*.^30^ have shown that morphological characters of the bony labyrinth may be phylogenetically significant when used separately in a cladistics analysis. Moreover, Costeur *et al*.^31^ have demonstrated the timing of the bony labyrinth ossification in an ontogenetic series of a pecoran ruminant. The different structures ossify diachronously and may be independent^31^. We should also highlight that the ecological impact on the morphology the bony labyrinth, if existing, is limited because all the studied ruminants possess a similar kind of locomotion and live in relatively similar environments. Mennecart & Costeur^29^ and Costeur *et al*.^31^ already pointed out that the open structures (i.e. the endolymphatic sac and the cochlear aqueduct) suffer from a strong intraspecific allometric effect due to continuous ossification of these structures long after birth (contrary to the rest of the bony labyrinth). Thus, these regions are not analyzed to avoid a Pinocchio effect. Similarly to Billet *et al*.^25,74^, we observed a weak allometric effect on bony labyrinth shape (see Supplementary data 5). The allometric effect is mainly based on the semi-circular canal proportions in comparison to the entire bony labyrinth. In our dataset, the centroid size may predict 8.5% of the total shape changes and there is a highly significant covariation between the bony labyrinth shape and the centroid size (p-value < 0.0001). However, the centroid size is not predictive for the bony labyrinth shape (R^2^ = 0.6672) and is not influenced by phylogeny (p-value = 0.2586). Since our data matrix for the phylogeny uses the semi-circular canal shape individually, and not the ratio between the canals and the bony labyrinth, the allometric effect does not affect our dataset. Thus, the semi-circular canals, the fenestra vestibuli, and the cochlea were treated here as separate and independent structures using an ordination method (CVA). This method was applied to maximise the between-group variation, relative to within-group groups according to the specified chosen grouping variable; here the well-defined cervid subfamilies Procervulinae, Dicrocerinae, Cervinae, and Capreolinae. Shape differences expressed along the axes are scaled morphological distances relative to within group variation. These shape differences are used as morphological characters in our cladistics matrix as additional characters to the matrix proposed by Mennecart and Costeur^28^ and coded for all the studied specimens (fossil and extant). We have included fossil specimens of well-known subfamilies (2 Capreolinae and 5 Cervinae), that were not in the PCA since their precise phylogenic position is still under discussion, to increase the number of observation per subfamilies (9 Procervulinae, 3 Dicrocerinae, 13 Cervinae, and 11 Capreolinae). Nevertheless, the low number of Dicrocerinae specimens may influence its morphospace relative to the other subfamilies. All statistical results are shown in the Supplementary data 5.

### Cladistics analysis

A cladistic analysis based on a matrix of 26 characters (see Supplementary data 3) of the bony labyrinth was also performed to test the phylogenetic power of the bony labyrinth: 12 characters of the semi-circular canals, 7 on the vestibule and associated structures, and 7 characters on the cochlea. New characters expand the matrix of Mennecart and Costeur^28^ and are derived from the CVA (see Supplementary data 2, 3, 5, and 6). 29 species of antlered ruminants are included in these analyses (see Supplementary data 1) to test the phylogenetic relationships of the fossil *L. parvulus*, *E. furcatus*, *Eostyloceros hezhengensis*, “*Cervus*” *ruscinensis*, *Croizetoceros pyreanicus*, *Croizetoceros ramosus*, *Metacervoceros philisi*, and *Megaloceros giganteus* within the Cervidae lineage and find new characters to characterise Cervidae tribes. The earliest artiodactyl *Homacodon vagans* from the Eocene of North America was chosen as outgroup^75,76^. The small number of character states (60) in comparison to the number of taxa (30) cannot provide a robust relationship. We are testing here the relevance of the bony labyrinth solely as a tool for phylogeny and why this structure should be systematically used in character matrices. The analysis was performed using WinClada^77^. All characters were unordered and equally weighted. We ran a heuristic search (1000 maximum trees to keep, 5 replications), which resulted in 30 most parsimonious trees of 91 steps (retention index Ri: 0.35; consistency index Ci: 0.74) and used the Nelson consensus compromise (collaps + consensus) to optimise the graphic result (95 steps; Ri 33: Ci: 72). For each node, the list of non-ambiguous synapomorphies is provided in Supplementary data 3.

### Calibrated tree

The complete mitochondrial genome matrix of Hassanin *et al*.^7^ was pruned down to include just cervids plus a few outgroup representatives of the remaining pecoran families. Cytochrome *b* gene data was added for *Megaloceros giganteus* (Genbank Accession number AM182644)^6^. After some tests of the combined morphological and molecular datasets, the early fossil artiodactyl *Homacodon vagans* (~47 Ma) was not included in the analysis because there is far too much of a temporal and phylogenetic gap between it and the next taxa for which morphological data was scored (*Lagomeryx parvulus, Procervulus prealucidens*, ~18 Ma). Its inclusion or exclusion did not significantly affect divergence estimates for the critical part of the tree (Pan-Cervidae), which, as detailed below, were largely controlled by the prior setting for the age of Pecora. The 34 partitions of the mtDNA data used in Hassanin *et al*.^7^ were analyzed in Partition Finder v.1.1^78^ using the greedy search and GTR + G model. Best partitioning scheme indicated eight partitions. The morphological dataset comprised a ninth partition. Analysis xml files from the morphological and molecular data matrices were set up using Beastmaster^79^ and R^80^. The GTR + G model was used for all molecular partitions, the MK model for morphology. The SABDSKY treemodel was used, which allows for fossil tips to be ancestral nodes (though experimental runs with BDSKY produced similar results, not shown). Two main analyses were run. 1) A minimal constraint analysis in which the root age (Pecora) was set to a uniform distribution of 19-25 Ma (dubbed the ‘Min Constraint’ analysis), and only a single topological constraint forcing all extant fossil cervids and stem cervids to be monophyletic; and 2) an analysis with the same root prior plus many constraints (‘All Constraints’ analysis) following previous the topology of previous molecular studies as well as the parsimony analysis based only on the inner ear characters in this paper. This analysis constrained pecoran relationships with *Antilocapra* branching off first, followed by Giraffa, Bovidae + Moschidae, then Cervidae e.g.,^7^, *Lagomeryx* basal to all other pan-cervids, *Euprox furcatus* was united with the Cervinae, and *Croizetocerus* spp. with the Capreolinae. As in the ‘Min Constraint’ analysis, root age was set to a uniform 19–25 Ma distribution. A third experimental analyses was run same as the Min Constraint analysis, but with a wider prior on the root (19–35 Ma, ‘Old Pecora’ analysis). All analyses used the included fossil taxa as tip dates, with age assigned as a range based on the uncertainty of that taxon’s first appearance datum. This information is given for each taxon in a separate table (Supplementary data 4). The phylogenetic analysis was run in BEAST v.2.2.1^81^ for 10 million generations. The morphological character matrix was separately analyzed using PAUP* 4^82^ using a heuristic search of 1000 replicates with TBR and a random addition sequence. Bootstrap was run for 100 replicates, each holding a single TBR replicate.

### Fossil host institutions

SNSB-BSPG – Staatliche Naturwissenschaftliche Sammlungen Bayerns - Bayerischen Staatssammlung für Paläontologie und Geologie, Munich (Germany); MNHN – Muséum National d’Histoire Naturelle, Paris (France); NMB – Naturhistorisches Museum Basel (Switzerland); ICP – Institut Català de Paleontologia Miquel Crusafont, Barcelona (Spain); IVPP – Institute of Vertebrate Paleontology and Paleoanthropology, Chinese Academy of Science, Beijing (China); CCEC – Musée des Confluences, Lyon (France).

## Electronic supplementary material

Supplementary Information

Dataset 1-5
