# Supplementary material for: Bony labyrinth morphology clarifies the origin and evolution of deer
Source: Sci Rep. 2017 Oct 13;7:13176. doi: 10.1038/s41598-017-12848-9 (PMC5640792; doi:10.1038/s41598-017-12848-9)

Bony labyrinth morphology clarifies the origin and evolution of deer

Bastien Mennecart[1]*, Daniel DeMiguel[2], Faysal Bibi[3], Gertrud E. Rössner[4], Grégoire Métais[5], James M. Neenan[6], Shiqi Wang[7], Georg Schulz[8], Bert Müller[8], Loïc Costeur[1]

[1] *Nathurhistorisches Museum Basel, Augustinergasse 2, 4001 Basel, Switzerland*

[2] *ICTA-ICP, Edifici Z, c/de les columnes s/n, Universitat Autònoma de Barcelona, 08193 Cerdanyola del Vallès, Barcelona, Spain*

[3] *Museum für Naturkunde Berlin, Leibniz Institute for Evolution and Biodiversity Science Invalidenstraße 43, 10115 Berlin, Germany*

[4] *Bayerische Staatssammlung für Paläontologie und Geologie, Richard-Wagner-Strasse 10, 80333 Munich, Germany*

[5] *CR2P - Centre de Recherches sur la Paléobiodiversité et les Paléoenvironnements, UMR 7207, Muséum National d'Histoire Naturelle, CNRS, UPMC, Sorbonne Universités. MNHN, CP38, 8 rue Buffon, 75005 Paris, France*

[6] *Oxford University Museum of Natural History, Parks Road, Oxford, OX1 3PW, United Kingdom*

[7] *Institute of Vertebrate Paleontology and Paleoanthropology, Chinese Academy of Sciences, 142 Xizhimenwai Street, Beijing 100044, China*

[8] *University of Basel, Biomaterials Science Center, Department of Biomedical Engineering, Gewerbestrasse 14, 4123 Allschwil, Switzerland*

Supplementary data 1. Specific information of the scanned specimens. The anatomy corresponds to the scanned section. NMB 213 and NMB 583 are partial skeletons attributed at first to *Metacervocerus philisi*.

Genus	species	anatomy	locality	locality age (Ma)	host institution	inventory number	instution scanner	Subfamily
Lagomeryx	*parvulus*	skull	Sandelzhauzen (Ge)	ca. 16	BSPG	1959II16856	BSPG	Lagomerycinae
Procervulus	*praelucidus*	petrosal	Wintershof West (Ge)	ca. 18	BSPG	1937II23339	BMC	Procervulinae
Procervulus	*praelucidus*	petrosal	Wintershof West (Ge)	ca. 18	BSPG	1937II23340	NMB	Procervulinae
Procervulus	*praelucidus*	petrosal	Artenay (Fr)	ca. 16.5	NMB	SO652 left	NMB	Procervulinae
Procervulus	*praelucidus*	petrosal	Artenay (Fr)	ca. 16.5	NMB	SO652 right	NMB	Procervulinae
Procervulus	*praelucidus*	petrosal	Artenay (Fr)	ca. 16.5	NMB	SO3521	NMB	Procervulinae
Procervulus	*dichotomus*	skull	Rauscheröd (Ge)	ca. 16.5	BSPG	1979XV555	BSPG	Procervulinae
Heteroprox	*lartetii*	petrosal	Steinheim (Ge)	ca. 13.5	NMB	Sth1343a	NMB	Procervulinae
Heteroprox	*lartetii*	petrosal	Steinheim (Ge)	ca. 13.5	NMB	Sth2390b	NMB	Procervulinae
Heteroprox	*lartetii*	petrosal	Steinheim (Ge)	ca. 13.5	NMB	2391a	NMB	Procervulinae
Heteroprox	*lartetii*	petrosal	Steinheim (Ge)	ca. 13.5	NMB	2394a	NMB	Procervulinae
Heteroprox	*lartetii*	petrosal	Steinheim (Ge)	ca. 13.5	NMB	2394b	NMB	Procervulinae
Heteroprox	*lartetii*	petrosal	Sansan (Fr)	ca. 15	MNHN	Sa9956	MNHN	Procervulinae
Dicrocerus	*elegans*	skull	Sansan (Fr)	ca. 15	MNHN	SA10150	MNHN	Dicrocerinae
Dicrocerus	*elegans*	skull	Sansan (Fr)	ca. 15	NMB	Ss5293	NMB	Dicrocerinae
Dicrocerus	*elegans*	petrosal	Sansan (Fr)	ca. 15	MNHN	Sa9954	MNHN	Dicrocerinae
Dicrocerus	*elegans*	petrosal	Sansan (Fr)	ca. 15	MNHN	Sa9957	MNHN	Dicrocerinae
Dicrocerus	*elegans*	petrosal	Sansan (Fr)	ca. 15	MNHN	Sa10193	MNHN	Dicrocerinae
Dicrocerus	*elegans*	petrosal	Sansan (Fr)	ca. 15	MNHN	Sa9911	MNHN	Dicrocerinae
Dicrocerus	*elegans*	petrosal	Sansan (Fr)	ca. 15	MNHN	Sa10191	MNHN	Dicrocerinae
Dicrocerus	*elegans*	petrosal	Sansan (Fr)	ca. 15	MNHN	Sa9801	MNHN	Dicrocerinae
Euprox	*furcatus*	skull	Eppishausen (Ge)	ca. 13	Hiller	Slg. Hiller Es	NMB	Stem Cervinae
Euprox	*furcatus*	skull	Abocador de Can Mata (Sp)	ca. 12	ICP	IPS 4444-1	MNCN	Stem Cervinae
Euprox	*furcatus*	skull	Steinheim (Ge)	ca. 13.5	NMB	Sth.2394c	NMB	Stem Cervinae
Eostyloceros	*hezhengensis*	skull	Goajiashan (Ch)	ca. 8	IVPP	V19059	IVPP	stem Cervidae
Muntiacus	*muntjak*	skull	unknown	0	NMB	C.2408	NMB	Cervinae
„Cervus"	*ruscinensis*	skull	Perpignan (Fr)	4.0-5.0	CCEC	Pp148	NMB	Cervinae
Eucladoceros	*ctenoides*	petrosal	Senèze (Fr)	ca. 2	NMB	Se1797	NMB	Cervinae
Eucladoceros	*ctenoides*	petrosal	Senèze (Fr)	ca. 2	NMB	Se981	NMB	Cervinae
Eucladoceros	*ctenoides*	petrosal	Senèze (Fr)	ca. 2	NMB	Se557	NMB	Cervinae
Cervus	*elaphus*	skull	unknown	0	NMB	11147	NMB	Cervinae
Rusa	*timorensis*	skull	Lamontjong (Id)	0	NMB	3657	NMB	Cervinae
Cervus	*nippon*	skull	unknown	0	NMB	6106	NMB	Cervinae
Dama	*dama*	skull	Kleinriehen (Ch)	0	NMB	3186	NMB	Cervinae
Dama	*eurygonos*	petrosal	Val d'Arno (It)	ca. 1.5	NMB	V.A.1642	NMB	Cervinae
Megaloceros	*giganteus*	skull	Westown Naul (Ir)	13.7-12.6 ka	NHML	M2326	NHML	Cervinae
Axis	*axis*	skull	Zoo Basel (Ch)	0	NMB	C.3718	NMB	Cervinae
Metacervocerus	*philisi1*	petrosal	Senèze (Fr)	ca. 2	NMB	Se583	NMB	Cervinae
Metacervocerus	*philisi2*	skull?	Senèze (Fr)	ca. 2	NMB	Se213	NMB	Cervinae
Croizetoceros	*pyreanicus*	skull	Perpignan (Fr)	4.0-5.0	CCEC	Pp116	NMB	?Stem Capreolinae
Croizetoceros	*ramosus*	skull	Etouaires (Fr)	ca. 3	NMB	P.r.r.65	NMB	?Stem Capreolinae
Odocoileus	*virginianus*	skull	unknown (Ve)	0	NMB	9872	NMB	Capreolinae
Odocoileus	*lucasi*	skull	Missouri (USA)	2.6-0	BSPG	2012I77	BSPG	Capreolinae
Odocoileus	*cf. virginianus*	skull	Michigan (USA)	2.6-0	BSPG	2015I37	BSPG	Capreolinae
Mazama	*americana*	skull	unknown (Br)	0	NMB	2315	NMB	Capreolinae
Pudu	*puda*	skull	unknown (Cl)	0	NMB	C2209	NMB	Capreolinae
Hydropotes	*inermis*	skull	Zoo Basel (Ch)	0	NMB	9892	NMB	Capreolinae
Capreolus	*capreolus*	skull	unknown	0	NMB	6212	NMB	Capreolinae
Alces	*alces*	skull	unknown	0	NMB	2198	NMB	Capreolinae

Supplementary data 2. Canonical Variate analyses on the different regions of the bony labyrinth (semi-circular canal, oval window, cochlea not shown). The results show the variation of the studied structure depending of the considered clade subfamilies (Procervulinae, Dicrocerinae, Cervinae, and Capreolinae).

Statistical results are provided in dataset1

1 Canonical Variate analyses of the anterior semi-circular canal landmarks.

Along CV1, the value -1.5 permits to separate the Cervinae from the other Cervidae due to a squarer canal. Along CV2, the value -0.5 permits to separate the Cervinae and Procervulinae from the Dicrocerinae and Capreolinae due to an ovoid anteriorly antero-upper part.

anterior semi circular canal

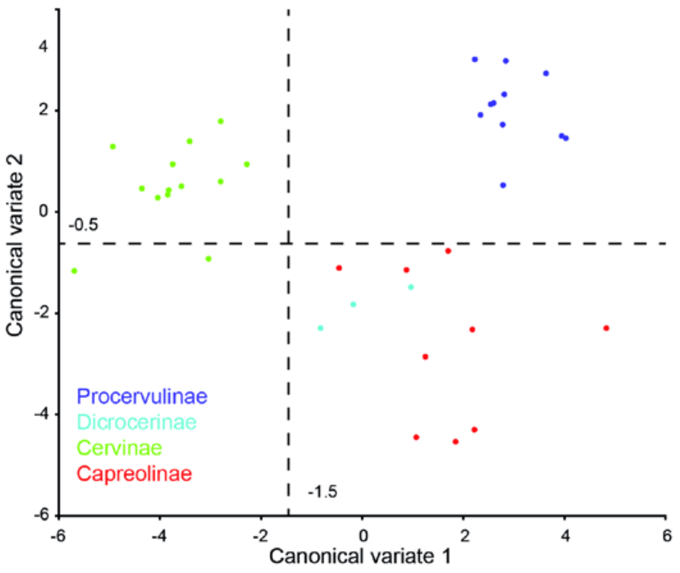

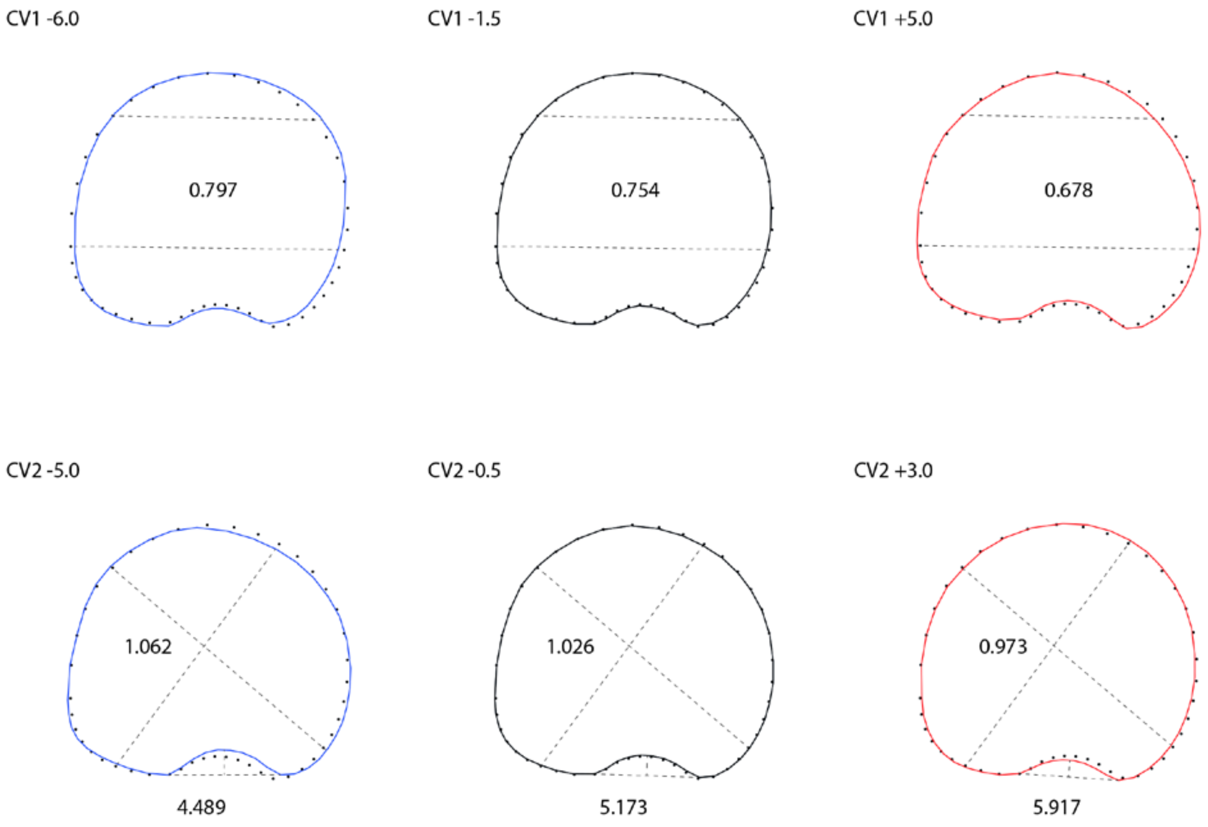

2 **Canonical Variate analyses of the posterior semi-circular canal landmarks.**

Along CV1, the values lower -2.0 permits characterise the Capreolinae (ovoid posteriorly posterior semi-circular canal and well rounded posterior ampulla), between -2.0 and +1.0 the Procervulinae (rounded posterior semi-circular canal and rounded posterior ampulla), and bigger than +1.0 the Cervinae and Dicrocerinae (ovoid posteriorly anteriorly semi-circular canal and flattened posterior ampulla). Along CV2, the value +1.5 permits to separate the Procervulinae from the other Cervidae due to a higher than wide posterior semi-circular canal.

posterior semi circular canal

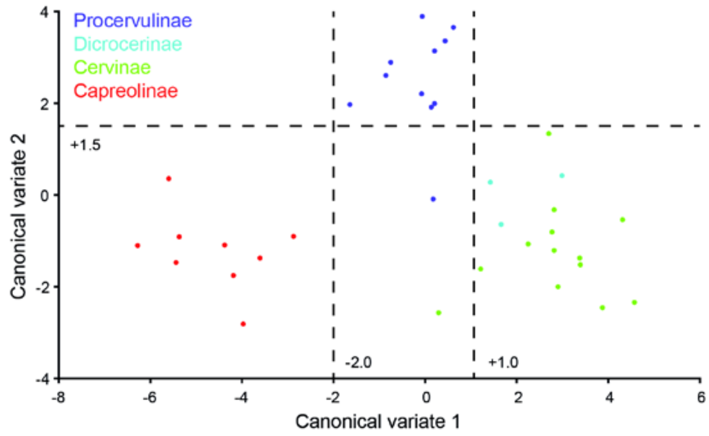

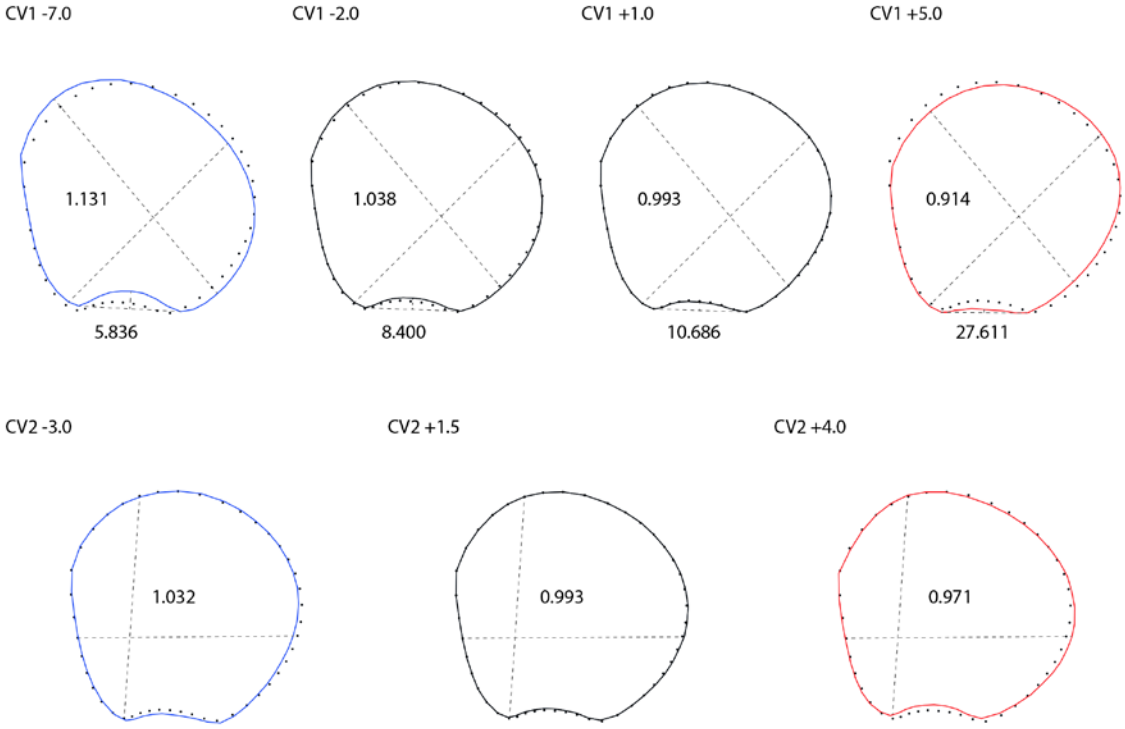

3 **Canonical Variate analyses of the lateral semi-circular canal landmarks.**

Along CV1, the value +1.5 permits to separate the Procervulinae from the other Cervidae due to a ovoid anteriorly canal and a flattened lateral ampulla.

lateral semi circular canal

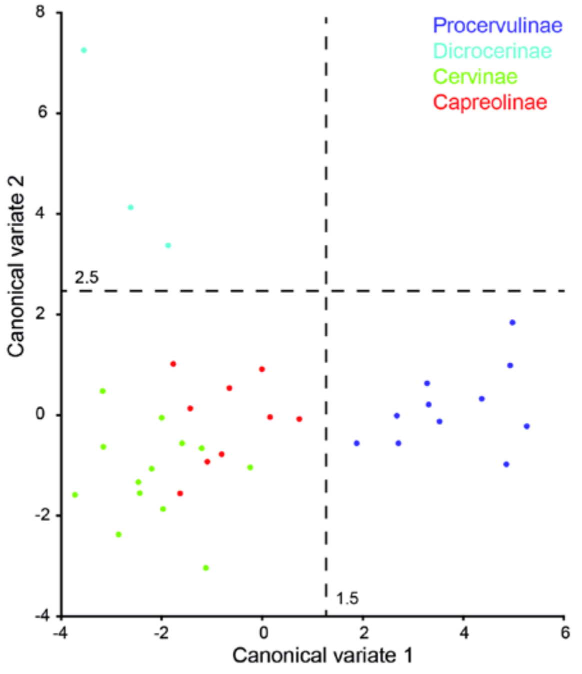

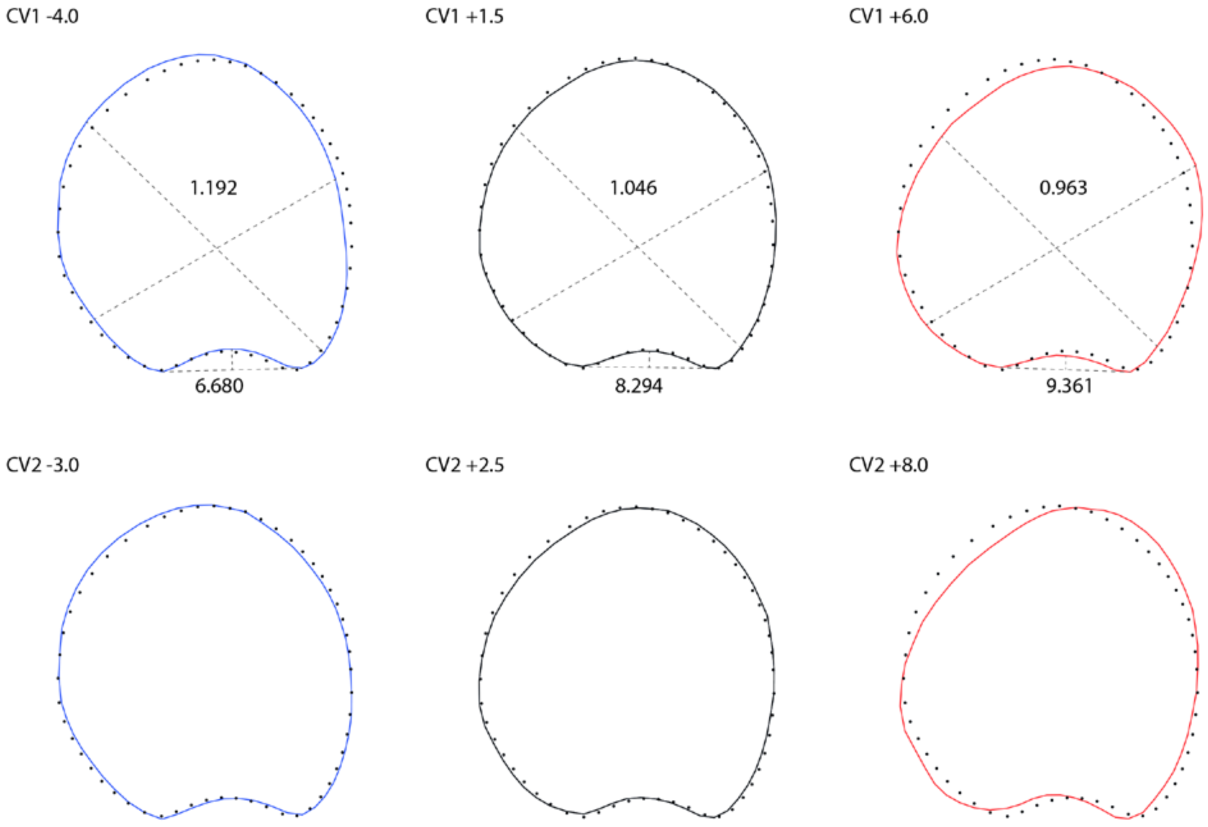

4 **Canonical Variate analyses of the Fenestra vestibuli landmarks.**

Along CV2, the values lower than +0.0 permits to separate the Cervinae from the other Cervidae due to a more elongated fenestra.

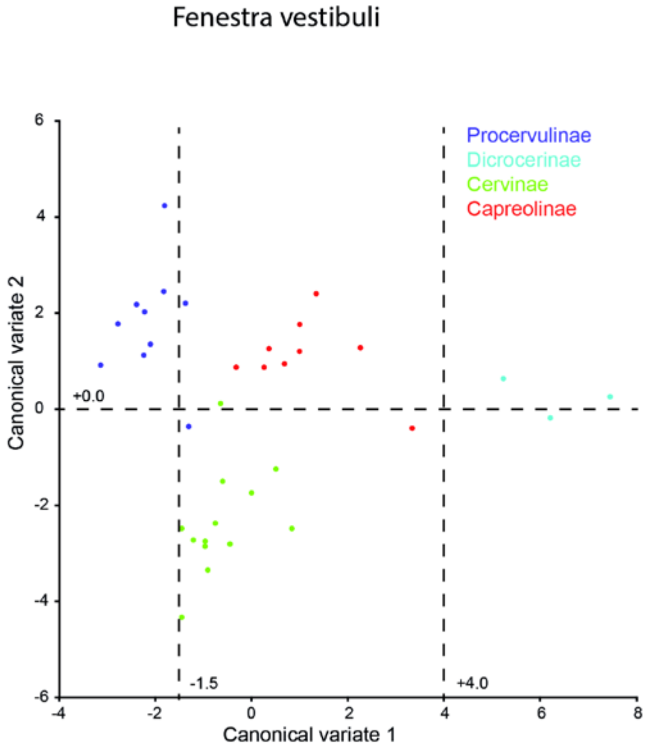

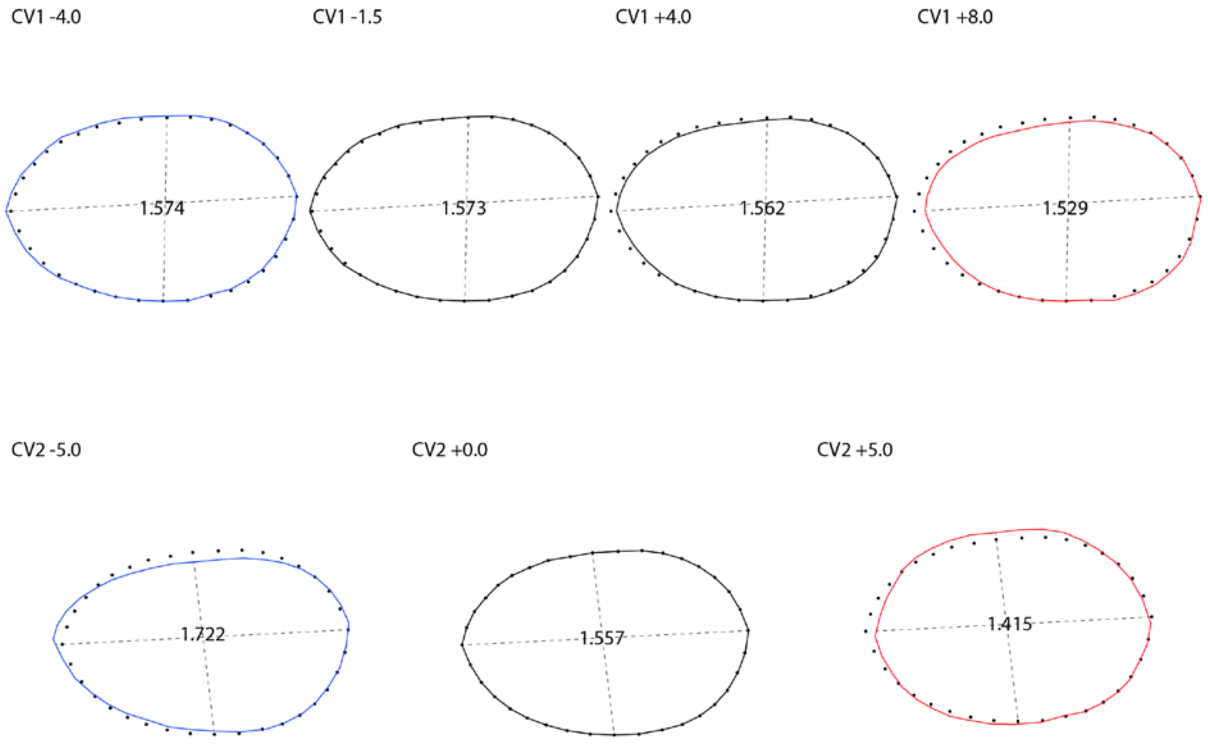

5 **Canonical Variate analyses of the cochlea thickness landmarks.**

Along CV1, the value -1.0 permits to separate the Cervinae from the other Cervidae due to a clear asymmetrical first turn of the cochlea. Along CV2, the value -1.0 permits to separate the Capreolinae from the other Cervidae due to an enlarged second turn of the cochlea.

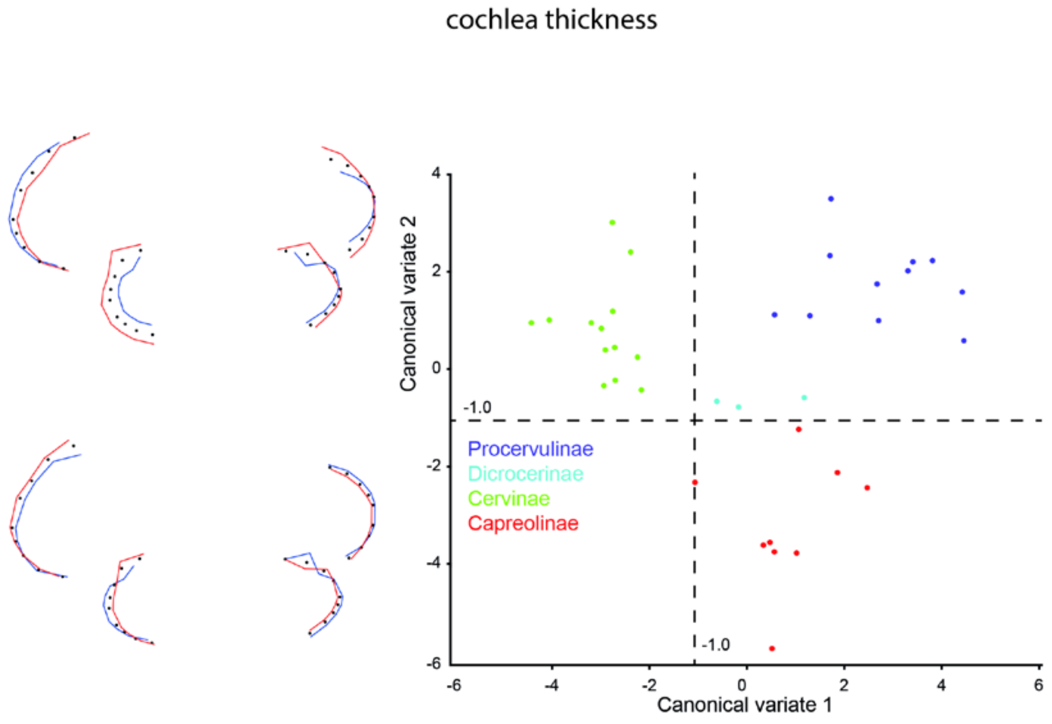

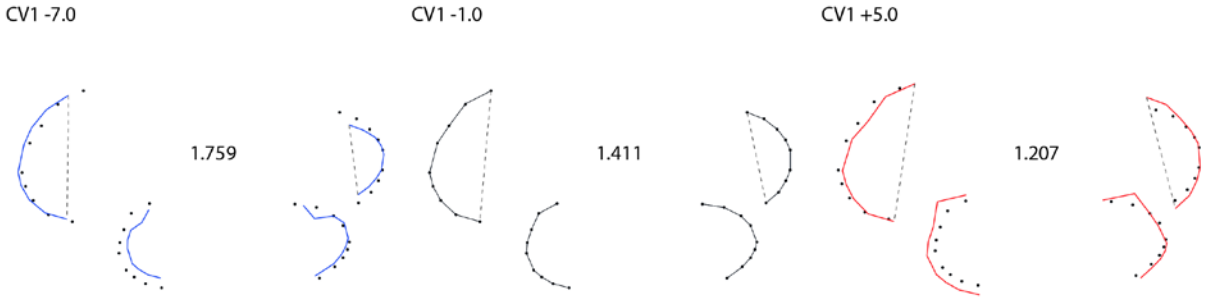

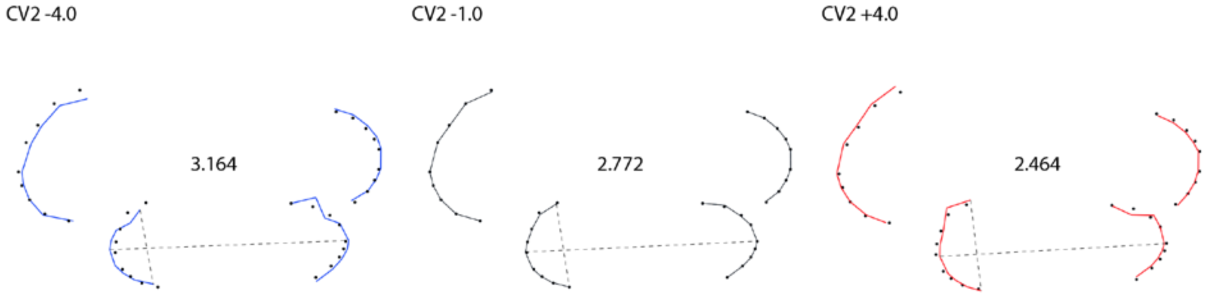

6 **Canonical Variate analyses of the first turn of the cochlea landmarks.**

Along CV1, the value -1.0 permits to separate the Cervinae from the other Cervidae due to a tightened second turn. Along CV2, the value +1.5 permits to separate the Capreolinae from the other Cervidae due to first turn width ratio starting narrow.

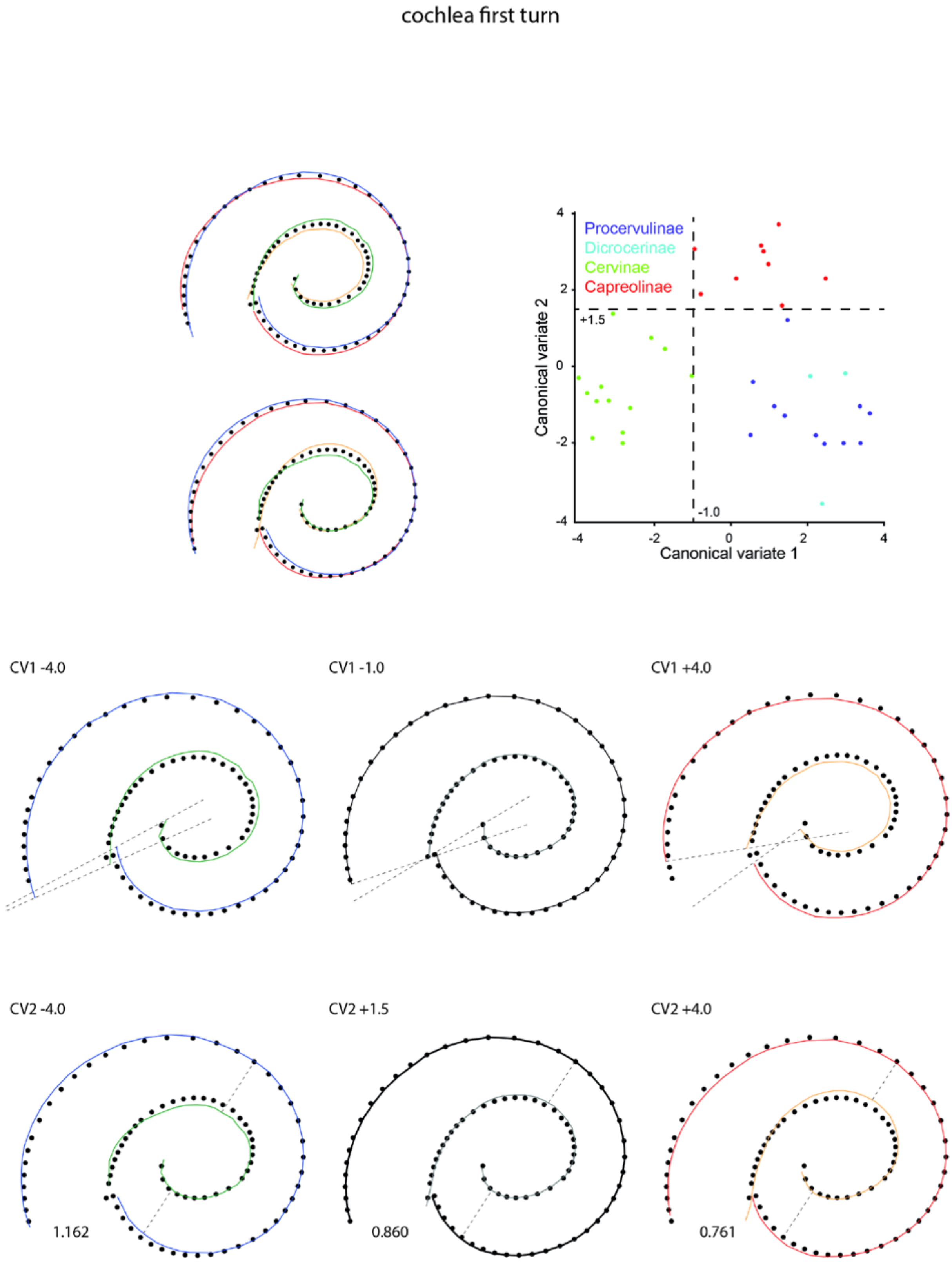

Supplementary data 3. Data matrix (nexus format) and character list used in the cladistics analyses with associated resulting consensus tree.

List of characters

Characters 1–7, 13 follow Ekdale 2013, Macrini et al. 2013, and Mennecart et al. 2016. The others are original.

1 Number of cochlear turns: equal or less than two (0); between two and three (1); equal or more than three (2)
2 Insertion of the lateral semi-circular canal in the vestibule towards the posterior ampulla: low in posterior ampula (0); high dorsally between posterior ampula and commun crus (1); high in posterior ampula (2); anterior to posterior ampula in vestibule (3)
3 Length of vestibular aqueduct: less than the common crus (0); same as common crus (0); longer than common crus (2)
4 Shape of the endolymphatic sac: knob like (0); straight and funnel like (1); triangular in shape (2); pouch like (3)
5 Fusion of lateral semi-circular canal with posterior ampulla: absent (0); partial to complete fusion (1)
6 Relative thickness of basal cochlear turn: thick (0); thin (1)
7 Section of cochlear aqueduct: flat (0); ovoid to circular (1)
8 Vestibular aqueduct: straight (0); curved (1)
9 Endolymphatic sac: symetrical (0); asymetrical (1)
10 Cochlear aqueduct: short (0); long (1)
11 Cochlear aqueduct: curved (0); straight (1)
12 Shape anterior semi-circular canal: round (0); flat anteriorly (1)
13 Shape of posterior canal: straight (0); undulating (1)
14 Shape of posterior ampulla: well rounded (ratio<8.400) (0); rounded (8.400<ratio<10.686) (1); flattened (ratio>10.686) (2)
15 Shape of posterior semi-circular canal: ovoid posteriorly (ratio>1.038) (0); rounded (1.038>ratio>0.993) (1); ovoid anteriorly (ratio<0.914) (2)
16 Height of posterior semi-circular canal: higher than wide (ratio<0.993) (0); wider than high (ratio>0.993) (1)
17 Shape of the anterior semi-circular canal: squared (ratio>0.754) (0); rounded (ratio<0.754) (1)
18 Anterior semi-circular canal antero-upper part: ovoid posteriorly (ratio>1.026) (0); ovoid anteriorly (ratio<1.026) (1)
19 Anterior ampulla: rounded (ratio<5.173) (0); flattened (ratio>5.173) (1)
20 Shape of the lateral canal: ovoid posteriorly (ratio>1.046) (0); ovoid anteriorly (ratio<1.046) (1)

21 Lateral ampulla: flattened (ratio>8.294) (0); rounded (ratio<8.294) (1)

22 Fenestra vestibuli: elongated (ratio>1.557) (0); rounded (ratio<1.557) (1)

23 Cochlea second turn coil: enlarged (0); tightened (1)

24 Cochlea first turn width ratio: start enlarged (ratio>0.860) (0); start narrow (ratio<0.860) (1)

25 Cochlea first turn thickness: symmetrical (ratio<1.411) (0); asymmetrical (ratio>1.411) (1)

26 Cochlea second turn thickness: enlarged (ratio<2.772) (0); flattened (ratio>2.772) (1)

References

Ekdale, E. G. Comparative Anatomy of the Bony Labyrinth (Inner Ear) of Placental Mammals. *PLoS ONE* **10(8)**, e0137149 (2013).

Macrini, T. E., Flynn, J. J., Ni, X., Croft, D. A., & Wyss, A. R. Comparative study of notoungulate (Placentalia, Mammalia) bony labyrinths and new phylogenetically informative inner ear characters. *J. Anat.* **223**, 442–461 (2013).

Mennecart, B. *et al.* The petrosal bone and bony labyrinth of early to middle Miocene European deer (Mammalia, Cervidae) reveal their phylogeny. *J. Morphol.* **277**, 1329–1338 (2016).

Nexus File of the character matrix

```
#nexus
...
begin data;
  dimensions ntax=30 nchar=26;
  format datatype=standard missing=? gap=-;
  matrix
Homacodon_vagans              0000-0-0001002001100110001
Lagomeryx_parvulus            000?100010000???11101?0001
Procervulus_praelucidus       11210000000001111111110000
Procervulus_dichotomus        11210000000001111111110000
Hereroprox_larteti            11210000000101111111110000
Dicrocerus_elegans            12121000010002201001010000
Eostyloceros_hezhengensis     1112?0000110?020??????0000
Euprox_furcatus               12121110011001111100101000
Muntiacus_muntjak             12121110011002200110001010
Cervus_ruscinensis            1221111011100011101000?1110
Metacervocerus_philisi_1      12130111111002200110001010
Metacervocerus_philisi_2      12130111111002200000001010
Axis_axis                     12131111110002200110001010
Eucladoceros_ctenoides        12211110011002200110001010
Cervus_elaphus                12211110011012200110001010
Cervus_nippon                 12121110111002200110001010
Rusa_timorrensis              12021110111001100110001010
Dama_eurygonos                12030111010002200110001010
Dama_dama                     12030111010002200000001010
Megaloceros_giganteus         12031111011002200110001010
Croizetoceros_pyrenaicus      12120110111000201100110000
Croizetoceros_ramosus         021201101110002011?0??0?00
Alces_alces                   12010110000010001000010101
Capreolus_capreolus           12120110000010001000010101
Odocoileus_virginianus        12210110011100001000000101
Odocoileus_cf._virginianus    12110110011100001000010101
Odocoileus_lucasi             12130111111100001000010101
Pudu_puda                     12220110111000001000010101
Hydropotes_inermis            10121100010010001000010101
Mazama_americana              12031111100000001000010101
                              [----+--10|----+--20|----+-]
  ;
end;
```

Phylogenetic tree of the Deer based on the above mentioned character matrix

For each node the list of the nonambiguous synapomorphies is given and each synapomorphy is represented by a black circle (strict synapomorphy) or an open white circle (homoplasic synapomorphy). The upper numbers indicate the character number and the lower numbers the states for these characters.

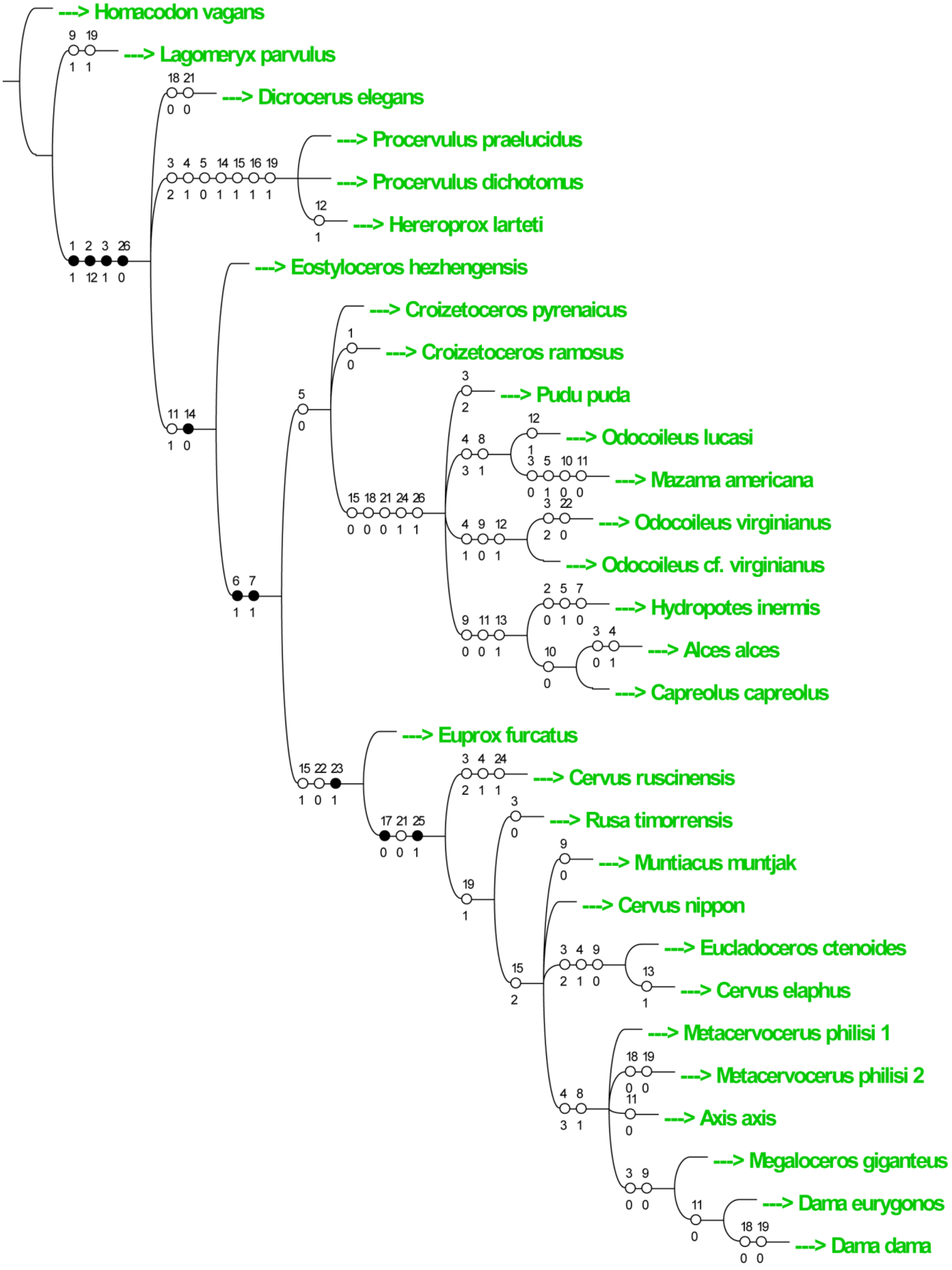

Lagomeryx parvulus is the sister taxon of the other antlered taxa (Figure 3). *L. parvulus* differs by 6 unambiguous plesiomorphic characters from the other species analysed, such as a short cochlea (1^0) with an enlarged second turn (26^0), a lateral semi-circular canal entering low in the posterior ampula (2^0), and a short vestibular aqueduct (3^0). Procervulinae, consisting of *Procervulus* species and *Heteroprox larteti*, form a monophyletic clade well supported by the combination of 7 non-ambiguous, but homoplasic, characters (3^2: elongated common crus; 4^1: straight and funnel like endolymphatic sac; 5^0: no fusion of lateral semi-circular canal with posterior ampulla; 14^1: rounded posterior ampulla; 15^1 & 16^1: rounded and wide posterior semi-circular canal; 19^1: flattened anterior ampulla). *Dicrocerus elegans* and Procervulinae are placed in an unresolved trichotomy with all crown cervids and *Eostyloceros hezhengensis* (Figure 3). The latter is excluded from the crown Cervidae due to the absence of the two autapomorphies of the crown Cervidae: 6^1 (thin basal turn of the cochlea) and 7^1 (ovoid to circular section of the cochlear aqueduct). The inclusion of *Croizetoceros* species within the crown Cervidae, and especially within Capreolinae, is weakly supported by one homoplasic character (5^0: no fusion of lateral semi-circular canal with posterior ampulla). The stem Cervinae position of *E. furcatus* is confirmed by (at least) three apomorphies of Cervinae (15^1, 22^0, 23^1). The more derived, but rather enigmatic "*Cervus*" *ruscinensis* is the sister taxon to the crown Cervinae (sharing 17^0, 21^0, 25^1 with the crown Cervinae) (Figure 3). The crown Capreolinae and Cervinae are well-supported by 6 and 7 non-ambiguous characters respectively of the cochlea (Capreolinae: 24^1,26^1; Cervinae: 22^0, 23^1, 25^1) and semi-circular canals (Capreolinae: 5^0, 15^0, 18^0, 21^0; Cervinae: 15^1, 17^0, 21^0) (Figure 3). A basal polytomy is observed within the crown Cervinae and crown Capreolinae. These clades are mainly supported by the open structures (endolymphatic sac, vestibular aqueduct) and the cochlear aqueduct supports closely related species apomorphies. *H. inermis*, *C. capreolus*, and *A. alces* constitute a monophyletic group (Figure 3) supported by not less than three non-ambiguous homoplasic characters (9^0 symetrical endolymphatic sac, 11^0 curved cochlear aqueduct, and

13^{1} undulating posterior canal). *H. inermis* is separated from *A. alces* and *C. capreolus* by a shorter cochlear aqueduct (10^{0}). *Odocoileus* cf. *virginianus* and *O. virginianus* cluster together (three homoplasic characters: 4^{1} triangular endolymphatic sac, 9^{0} symetrical endolymphatic sac, 12^{1} flat anteriorly anterior semi-circular canal) and are separated from the clade including *Odocoileus lucasi* and *M. americana* (two homoplasic characters: 4^{3} pouch like endolymphatic sac, 8^{1} curved vestibular aqueduct). *Muntiacus muntjak* is placed in a clade with *Cervus* species and *R. timorensis* as sister taxon due the homoplasic and highly variable character of the posterior semi-circular canal shape (15). As in the Capreolinae terminal clades (e. g. *Mazama americana* and *Odocoileus lucasi*), the Cervinae terminal clades are supported by the shape of the endolymphatic sac (4, 9) and vestibular aqueduct (3, 8). *Eucladoceros ctenoides* and *Cervus elaphus* form a clade (Figure 3) supported by three non-ambiguous homoplasic characters (3^{2}, 4^{1}, 9^{0}). The two specimens of "*Metacervocerus philisi*" are included in a basal polytomy with *Axis axis* and a well-supported clade formed by *M. giganteus*, *Dama eurygonos*, and *D. dama* (3^{0}, 9^{0}; Figure 3).

Supplementary data 4. Calibration points of the calibrated phylogenetic tree of the Cervidae.

Tree files are loaded in a separate file due to the size of the information
Dataset3 corresponds to the file All constrain tree.
Dataset4 corresponds to the file minimum constrain tree.
Dataset5 corresponds to the old Pecora tree.

Methods

In the Min Constraint analysis, the root age of Pecora sits at its maximum allowed limit (95% range 25-23.4 Ma), *E. furcatus* falls out of crown cervid, and the 95% age range estimate for Cervidae is 17.9-14.2 Ma (median 16 Ma). The All Constraints analysis, which is constrained to give a similar topology as the parsimony analyses (with *Euprox* allied to the Cervinae), gives a similar root age to that in the Min Constraints analysis, and a similar age for crown cervids (16.4-14.1 Ma, median 15.2 Ma). This shows that the inclusion of *Euprox furcatus* within the Cervidae changes little in the divergence estimates, which clearly push for the oldest possible ages across the tree. The Old Pecora analysis dated the node age for Pecora to 35-25.7 Ma, and crown Cervidae to 25-16 Ma (median 22.8 Ma) again showing that ages across the tree were largely determined by the root prior. Interestingly, the Old Pecora analysis placed (though with very low support) *Euprox furcatus* in the Cervinae, as in the parsimony analyses of the morphology-only dataset, and without need for topological constraints as in the All Constraints analysis. This indicates that the placement of *Euprox furcatus* is strongly affected by the rates of evolution calibrated by the morphological dataset.

Deer Calibrations

Genus	Species	FAD Locality / MN Zone	FAD	FAD Max	FAD Min	LAD	LAD Max-Min	Notes	Reference
„Cervus"	*ruscinensis*	MN15		5	3.5	MN15	5.0-3.5		Palombo & Valli 2003-2004
Alces	*alces*					Extant	0		
Axis	*axis*					Extant	0		
Capreolus	*capreolus*	MN20				Extant	0		Palombo & Valli 2003-2004
Cervus	*elaphus*	MN20?-MN21				Extant	0		Palombo & Valli 2003-2004
Cervus	*nippon*					Extant	0		
Croizetoceros	*pyreanicus*	Venta Del Moro		7.1	5.3			NOW database.	Also Gentry et al. 1999; Palombo & Valli 2003-2004
Croizetoceros	*ramosus*	La Calera		4.2	3.4			Now database oldest site.	
Dama	*dama*					Extant	0		
Dicrocerus	*elegans*	MN5		17	15.2			Now database. Only a single loc dated 20-17 but many dated 17-15.2, so this was chosen.	
Eucladoceros	*ctenoides*	MN16b		3	2.5	MN18	1.8-0.6		Palombo & Valli 2003-2004
Euprox	*furcatus*	MN 6		15.2	12.5			Now database. Two doubtful occurrences from MN5, but many localities from MN 6.	
Eustyloceros	*hezhen-gensis*			ca. 8	ca. 8	ca. 8	ca. 8	only one specimen known	
Heteroprox	*laterii*			18	15.2			NOW database. Single oldest dated to 18-17 Ma, but many dated to 17-15.2, so 18-15.2 chosen.	
Homacodon	*vagans*	Central Great Plains CP34D, USA		46.9	46.3			NOW database. Only locality for this species in the NOW database.	(1998). Evolution of Tertiary Mammals of North America: Volume 1, Terrestrial Carnivores, Ungulates, and Ungulate-like Mammals. Cambridge University Press.
Hydropotes	*inermis*					Extant	0		
Lagomeryx	*parvulus*	MN4		20	18			NOW database. Many locali-ties, oldest among these dated to 20-18 Ma.	
Mazama	*americana*					Extant	0		
Megaloceros	*giganteus*	Reilingen, Germany		0.301	0.242			NOW database. Oldest loc with most precise age range.	Ziegler & Dean (1998). Mamma-lian fauna and biostratigraphy of the pre-Neandertal site of Reilingen, Germany. Journal of Human Evolution
Metacervoce-rus	*philisi*	Apolakkia		4.2	3.4			Now database.	
Muntiacus	*muntjak*					Extant	0		
Odocoileus	*virgianicus*					Extant	0		
Odocoileus	*lucasi*					Extant	0		
Odocoileus	*sp.*					Extant	0		
Procervulus	*dichotomus*	Numerous, see notes		20	17			NOW database. Many localities, oldest among these dated to 20-19 Ma, but the vast majority to 18-17 Ma. So a wide range chosen to allow for 20-17 Ma.	Numerous localities in NOW database.
Procervulus	*praelucidens*		MN3	20	18			NOW database. Many locali-ties, oldest among these dated to 20-18 Ma.	
Pseudodama	*eurygonos*	Farneta & Pirro, Italy		3.4	1.95			NOW database. Oldest 2 locs for 'Axis' eurygonos.	"di Stefano, Petronio & Sardella (2005). Large bovids and cervids from latest Villafranchian-Gale-rian faunas of Italy. Quaternaire, hors série 2 (): 95-102. Palombo (2005). Middle Pliocene – Late Pleistocene herbivore guilds of Italy. Quaternaire, hors série 2 (): 123-136. "
Pudu	*puda*					Extant	0		
Rusa	*timorensis*					Extant	0		

Nodes age

	all constrain	range (95%)	min constrain	range (95%)	old Pecora	range (95%)
crown Ruminantia	24.79	25.0-24.1	24.66	25.0-23.4	31.36	35.0-25.7
Cervidae/Bovoidea	22.69	23.5-21.5	24.66	25.0-23.4	31.36	35.0-25.7
Crown Cervidae	15.2	16.4-14.1	15.99	17.9-14.18	20.62	25.2-16.3
Crown Cervinea	12.55	13.9-11.2	13.11	15.2-11.0	15.91	19.0-11.9
Crown Cervini	7.96	9.1-7.0	8.38	9.8-7.1	10.07	12.1-8.1
Crown Muntiacini	11.21	12.5-9.7	11.58	13.8-9.9	?	?
Crown Capreolinae	13.63	14.8-12.5	14.55	16.6-12.7	17.85	20.9-13.8
Crown Capreolini	13.02	14.3-11.9	13.85	15.9-12.0	16.98	19.9-13.1
Crown Odocoileini	10.26	11.2-9.4	11.13	12.9-9.6	13.51	15.9-10.4

Supplementary data 5. Results of the Principal Component and Canonical Variate Analyses.

All the information are in Dataset2.

Supplementary data 6. Different studied structures defined in Supplementary data 3 for the fossils not formally affiliated yet to a subfamily.

1 Semi circular canals and Fenestra vestibule landmarks with associated character measurements of *Homacodon*, *Lagomeryx*, and *Eostyloceros.*

	Homacodon vagans	*Lagomeryx parvulus*	*Eostyloceros hezhengensis*
Anterior canal	0.664 1.098 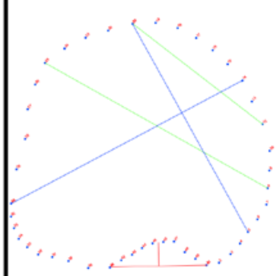 4.074	0.622 1.020 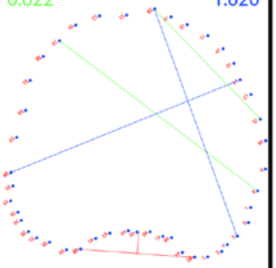 5.328	
Lateral canal	1.190 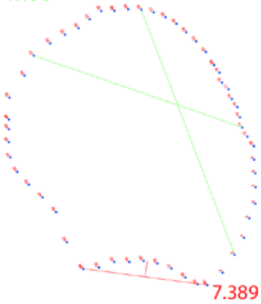 7.389	1.367 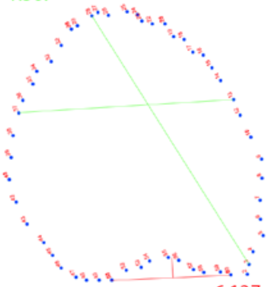 6.137	
Posterior canal	1.109 0.800 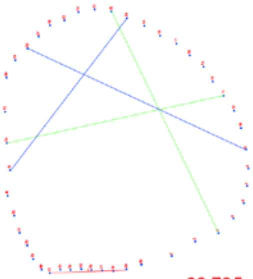 23.735		0.908 0.928 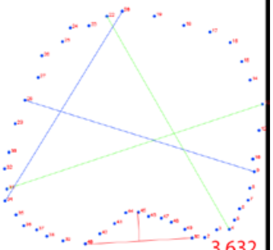 3.632
Fenestra vestibuli	1.396 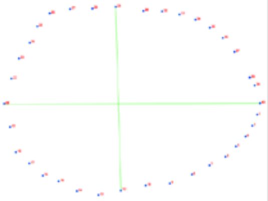		

2 Cochlea landmarks with associated character measurements of *Homacodon*, *Lagomeryx*, and *Eostyloceros.*

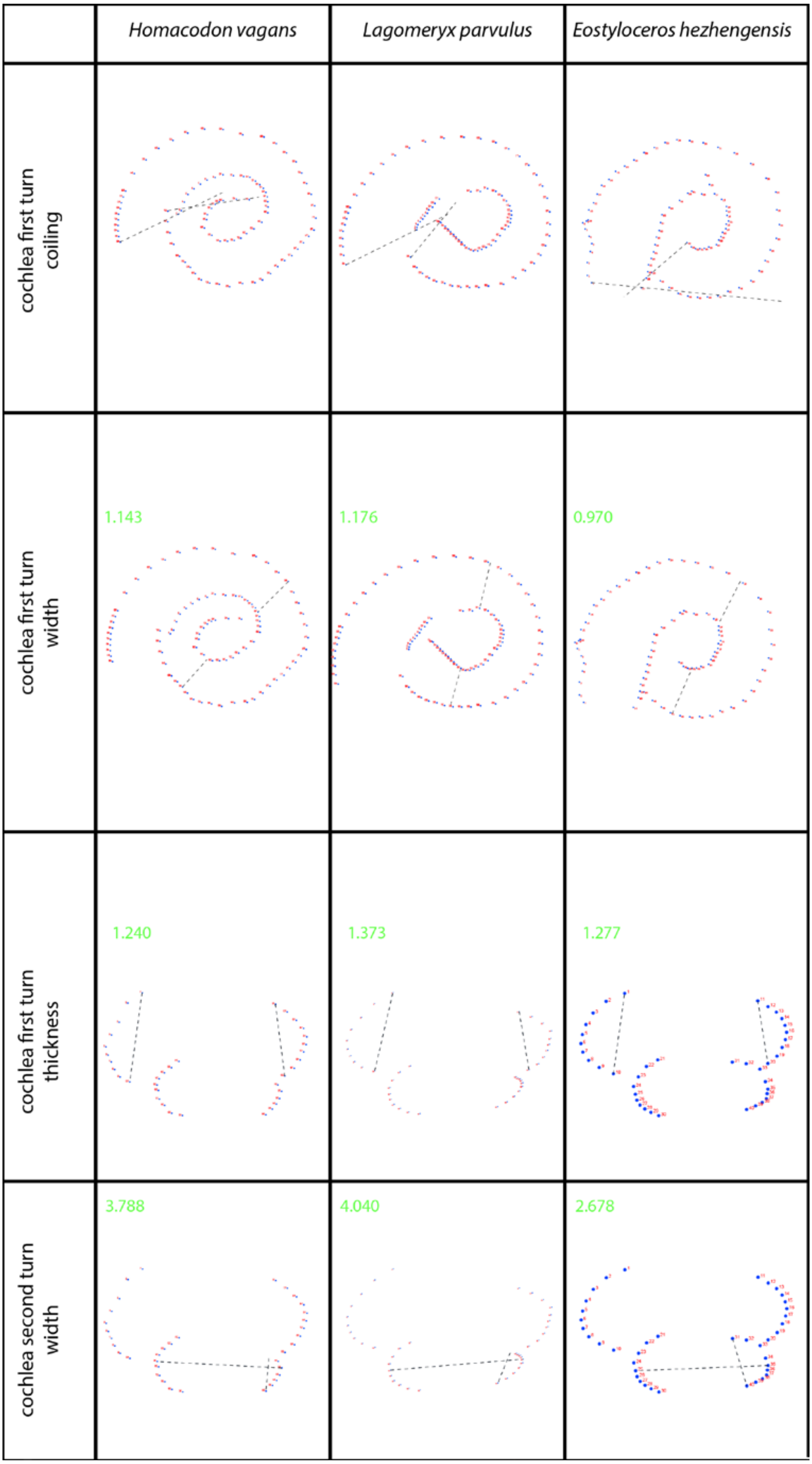

3 Semi-circular canals and Fenestra vestibule landmarks with associated character measurements of *Euprox*, *Croizetoceros*, and "*Cervus*".

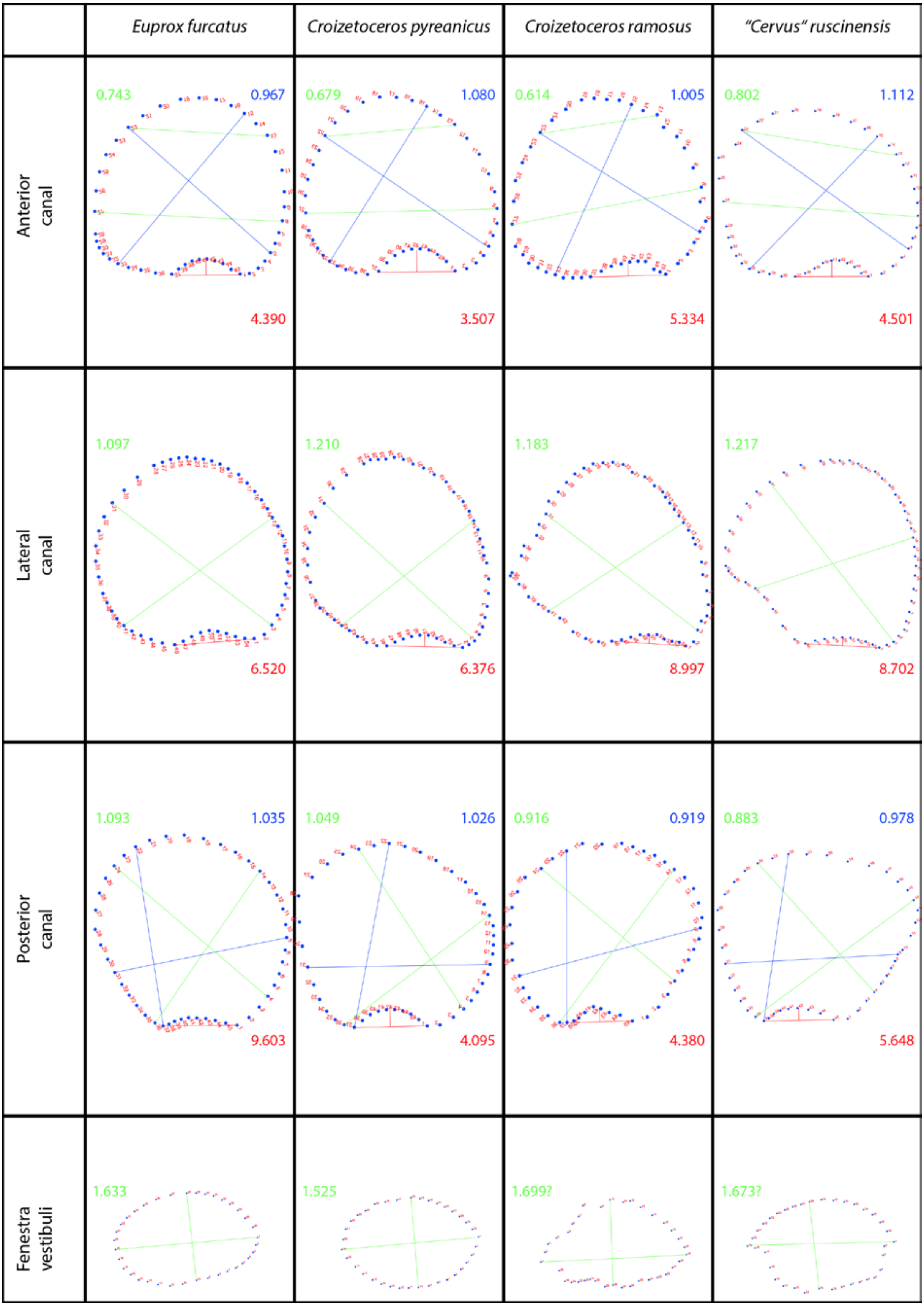

4 Cochlea landmarks with associated character measurements of *Euprox*, *Croizetoceros*, and “*Cervus*”.

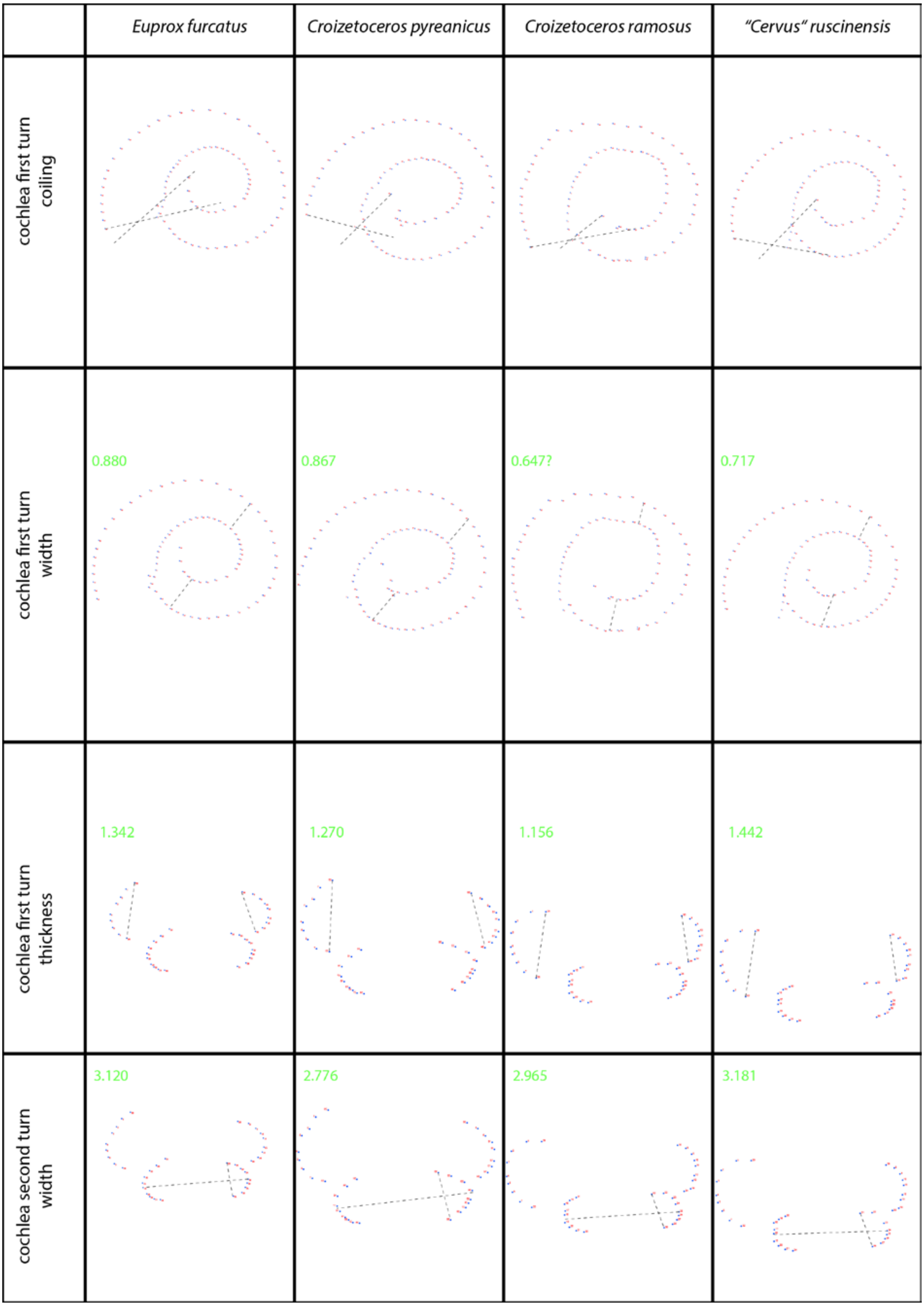

Supplement: Supplementary file 1 — Supplementary Information [file 41598_2017_12848_MOESM1_ESM.pdf]
