# Supplementary material for: Bony labyrinth morphology clarifies the origin and evolution of deer
Source: Sci Rep. 2017 Oct 13;7:13176. doi: 10.1038/s41598-017-12848-9 (PMC5640792; doi:10.1038/s41598-017-12848-9)
Supplement: Supplementary file 2 — Dataset 1-5 [file 41598_2017_12848_MOESM2_ESM.zip › dataset3.pdf]

```
#NEXUS
begin taxa;
	dimensions ntax=53;
	taxlabels
	Alces_alces
	Antilocapra_americana
	Axis_axis
	Axis_porcinus
	Blastocerus_dichotomus
	Capreolus_capreolus
	Cervus_elaphus_NC007704
	Cervus_nippon_centralis_NC006993
	Cervus_ruscinensis
	Croizetoceros_pyrenaicus
	Croizetoceros_ramosus
	Dama_dama_dama
	Dicrocerus_elegans
	Elaphodus_cephalophus_N008749
	Elaphurus_davidianus
	Eucladoceros_ctenoides
	Euprox_furcatus
	Eostyloceros_hezhengensis
	Giraffa_camelopardalis_angolensis_NC012100
	Hereroprox_larteti
	Hippocamelus_antisensis
	Hydropotes_inermis
	Lagomeryx_parvulus
	Mazama_americana_1
	Mazama_gouazoubira
	Mazama_nemorivaga_1
	Mazama_rufina
	Megaloceros_giganteus_AM182644
	Metacervocerus_philisi_1
	Metacervocerus_philisi_2
	Moschus_moschiferus
	Muntiacus_crinifrons_NC004577
	Muntiacus_muntjak_NC_004563
	Muntiacus_reevesi_NC008491
	Muntiacus_vuquangensis
	Odocoileus_hemionus
	Odocoileus_lucasi
	Odocoileus_cf._virginianus
	Odocoileus_virginianus_1
	Ovis_aries_NC001941
	Ozotoceros_bezoarcticus
	Procervulus_dichotomus
	Procervulus_praelucidus
	Przewalskium_albirostris
	Dama_eurygonos
	Pudu_mephistophiles
	Pudu_puda
	Rangifer_tarandus_NC007703
	Rucervus_duvauceli
	Rucervus_eldi
	Rusa_alfredi
	Rusa_timorensis
	Rusa_unicolor_NC008414
;
end;
```

begin trees;
	tree TREE1 = [&R] (((((((((Alces_alces[&height=5.942704552876331E-
7,height_95%_HPD={0.0,1.2069940567016602E-
6},height_median=5.960464477539062E-
7,height_range={0.0,1.6093254089355469E-
6},length=12.996578238775506,length_95%_HPD={11.722114562988281,14.245163
917541504},length_median=13.016486167907715,length_range={7.8045229911804
2,15.1128511428833},rate=0.009035467355969756,rate_95%_HPD={0.00784401419
100052,0.010372223903613607},rate_median=0.00899880345411578,rate_range={
0.007237981572762113,0.011459119521782855},!rotate=true]:13.016487,(Capre
olus_capreolus[&height=5.859245153047274E-
7,height_95%_HPD={0.0,1.2218952178955078E-
6},height_median=5.662441253662109E-
7,height_range={0.0,1.6391277313232422E-
6},length=8.81292583018581,length_95%_HPD={7.192226886749268,10.382258415
222168},length_median=8.777616500854492,length_range={6.524768829345703,1
1.173799514770508},rate=0.011848184766722211,rate_95%_HPD={0.009685684757
699231,0.014098009441981798},rate_median=0.011717394852452528,rate_range=
{0.008714140946256365,0.01648615698031696},!rotate=true]:8.777995,Hydropo
tes_inermis[&height=5.874324890074337E-
7,height_95%_HPD={0.0,1.2218952178955078E-
6},height_median=5.662441253662109E-
7,height_range={0.0,1.6391277313232422E-
6},length=8.809073664694429,length_95%_HPD={7.192226886749268,10.38225841
5222168},length_median=8.774261474609375,length_range={3.8631510734558105
,11.173799514770508},rate=0.012105307153806751,rate_95%_HPD={0.0094997869
21795343,0.01424290181721694},rate_median=0.012032260950050402,rate_range
={0.009248078561269416,0.01665677915761964},!rotate=true]:8.777995)[&heig
ht=8.815891328220284,height_95%_HPD={7.192227937281132,10.382259212434292
},height_median=8.777996003627777,height_range={6.524768978357315,11.1737
99743875861},length=4.194636102825562,length_95%_HPD={2.7063605785369873,
5.760099411010742},length_median=4.147654056549072,length_range={0.647877
2163391113,6.612398624420166},posterior=0.996005326231691,rate=0.01121501
2571950103,rate_95%_HPD={0.007384600837203447,0.014726132233648062},rate_
median=0.011064844743877596,rate_range={0.00660638621196075,0.01749335051
9001964},!rotate=true]:4.238491)[&height=13.02441180135283,height_95%_HPD
={11.857097700238228,14.28611834347248},height_median=13.016487136483192,
height_range={11.157182186841965,15.112851440906525},length=0.72966884730
73544,length_95%_HPD={0.21748627722263336,1.2996273040771484},length_medi
an=0.7180008888244629,length_range={0.017111416906118393,2.06259155273437
5},posterior=0.952063914780293,rate=0.010417734570736204,rate_95%_HPD={0.
006417925386352346,0.01474830155965057},rate_median=0.010196671933118238,
rate_range={0.004625244658880038,0.018774864746420275},!rotate=true]:0.61
5881,(((((Blastocerus_dichotomus[&height=5.829866948263464E-
7,height_95%_HPD={0.0,1.1101365089416504E-
6},height_median=5.662441253662109E-
7,height_range={0.0,1.5497207641601562E-
6},length=5.698129934969342,length_95%_HPD={4.880300998687744,6.546792030
334473},length_median=5.699440002441406,length_range={0.813406765460968,7
.0695695877075195},rate=0.01285267753590266,rate_95%_HPD={0.0106526172146
91937,0.014850378988164634},rate_median=0.012818724359326772,rate_range={
0.008871520326825562,0.016758341474579035},!rotate=true]:5.698876,((Hippo
camelus_antisensis[&height=5.932219568550085E-
7,height_95%_HPD={1.1920928955078125E-7,1.1026859283447266E-
6},height_median=5.811452865600586E-
7,height_range={0.0,1.475214958190918E-
6},length=3.5494605905504106,length_95%_HPD={2.8025660514831543,4.4139823
91357422},length_median=3.568859577178955,length_range={0.116672389209270
48,5.118407249450684},rate=0.011776010288397077,rate_95%_HPD={0.009446362
573364455,0.01437760994035024},rate_median=0.01177492007683959,rate_range

={0.007662947714959743,0.015962243241564224},!rotate=true]:3.582883,Mazam
a_gouazoubira[&height=5.941148360210846E-
7,height_95%_HPD={1.1920928955078125E-7,1.1026859283447266E-
6},height_median=5.811452865600586E-
7,height_range={0.0,1.475214958190918E-
6},length=3.595538674753293,length_95%_HPD={2.892051935195923,4.370753765
106201},length_median=3.5847156047821045,length_range={0.5264249444007874
,5.118407249450684},rate=0.011839292862760925,rate_95%_HPD={0.00948561260
8509969,0.014527687541675839},rate_median=0.011826389595265397,rate_range
={0.008010001695058402,0.01675478475586681},!rotate=true]:3.582883)[&heig
ht=3.6098826082998694,height_95%_HPD={2.8920525312423706,4.27422137139365
1},height_median=3.5828831493854523,height_range={2.8025666773319244,5.11
8407428264618},length=1.7055182540684604,length_95%_HPD={1.11158382892608
64,2.324831247329712},length_median=1.678325355052948,length_range={0.401
36420726776123,3.3004698753356934},posterior=0.948069241011984,rate=0.011
812040688019394,rate_95%_HPD={0.007505982319264106,0.015742040959219675},
rate_median=0.011615920620317688,rate_range={0.006674650271705944,0.02058
25241079295 6},!rotate=true]:1.695402,Ozotoceros_bezoarcticus[&height=6.00
230423721191E-7,height_95%_HPD={5.9604644775390625E-
8,1.1324882507324219E-6},height_median=5.960464477539062E-
7,height_range={0.0,1.5944242477416992E-
6},length=5.29136367215615,length_95%_HPD={4.509182929992676,6.1598844528
19824},length_median=5.282581329345703,length_range={1.0906246900558472,6
.773534774780273},rate=0.010503766138782244,rate_95%_HPD={0.0086366961294
76793,0.012315945745403588},rate_median=0.010473942153118542,rate_range={
0.007703704917762499,0.01315743328083059},!rotate=true]:5.278284)[&height
=5.320626696497518,height_95%_HPD={4.543949898332357,6.100172816775739},h
eight_median=5.278284918516874,height_range={4.383197009563446,6.77353575
0806332},length=0.40624205833785454,length_95%_HPD={0.12217432260513306,0
.7138513326644897},length_median=0.38702628016471863,length_range={0.0639
5573914051056,1.0013667345046997},posterior=0.9214380825565912,rate=0.010
798476824258841,rate_95%_HPD={0.006977833789453845,0.014755544600336},rat
e_median=0.01058128542880327,rate_range={0.005095573333530186,0.019547834
52044306},!rotate=true]:0.420591)[&height=5.72127198468644,height_95%_HPD
={4.880301415920258,6.504075527191162},height_median=5.698876276612282,he
ight_range={4.800343334674835,7.069570057094097},length=0.404918991917267
77,length_95%_HPD={0.13423529267311096,0.7203856110572815},length_median=
0.3865680545568466,length_range={0.09379556030035019,1.3779090642929077},
posterior=0.9161118508655126,rate=0.01077855585107635,rate_95%_HPD={0.006
698566230416691,0.014351556921232758},rate_median=0.0106790575313788,rate
_range={0.004950350961707795,0.017777941341786063},!rotate=true]:0.37898,
Mazama_nemorivaga_1[&height=5.934247002997665E-
7,height_95%_HPD={0.0,1.0924413800239563E-
6},height_median=5.662441253662109E-
7,height_range={0.0,1.3709068298339844E-
6},length=6.119205754225486,length_95%_HPD={5.294653415679932,6.998454570
770264},length_median=6.083005428314209,length_range={2.6167633533477783,
7.588428497314453},rate=0.012976075396904504,rate_95%_HPD={0.011011645583
359042,0.015466300044828811},rate_median=0.012972455071000165,rate_range=
{0.009985463622129322,0.016863537469767435},!rotate=true]:6.077856)[&heig
ht=6.123725408584072,height_95%_HPD={5.294654309749603,6.9622387290000916
},height_median=6.077856425195932,height_range={5.1633604764938354,7.5884
2920511961},length=0.45987269080000087,length_95%_HPD={0.1323462277650833
,0.7763292789459229},length_median=0.4417170435190201,length_range={0.029
590066522359848,1.0215283632278442},posterior=0.8948069241011984,rate=0.0
10967992280046947,rate_95%_HPD={0.007091222525460792,0.014982999058974052
},rate_median=0.010763025404241972,rate_range={0.005537240061116443,0.018
46962678030812},!rotate=true]:0.457081,Pudu_puda[&height=5.86018763661146
4E-7,height_95%_HPD={2.9802322387695312E-8,1.0728836059570312E-
6},height_median=5.960464477539062E-

7,height_range={0.0,1.4156103134155273E-
6},length=6.566745926314759,length_95%_HPD={5.735774040222168,7.578143596
64917},length_median=6.5403666496276855,length_range={2.8138833045959473,
8.247387886047363},rate=0.010507915975981821,rate_95%_HPD={0.008696445688
170745,0.012256263876152695},rate_median=0.010470356364249273,rate_range=
{0.007751541299449168,0.013772254650602457},!rotate=true]:6.534937)[&heig
ht=6.581966285406546,height_95%_HPD={5.794845327734947,7.579628152772784}
,height_median=6.534937251359224,height_range={5.630484163761139,8.025159
180164337},length=2.6984225163783773,length_95%_HPD={1.9786244630813599,3
.618433952331543},length_median=2.6781692504882812,length_range={0.640485
1078987122,4.030714511871338},posterior=0.9014647137150466,rate=0.0132505
92773069919,rate_95%_HPD={0.009524331360889907,0.017088735291325364},rate
_median=0.013057739635933947,rate_range={0.00788367697591274,0.0201677912
0245831},!rotate=true]:2.724544,(((Mazama_americana_1[&height=5.953927160
415565E-7,height_95%_HPD={1.1920928955078125E-7,1.1175870895385742E-
6},height_median=5.811452865600586E-
7,height_range={0.0,1.4603137969970703E-
6},length=3.2122824703964508,length_95%_HPD={1.5280674695968628,4.5988397
5982666},length_median=3.414017677307129,length_range={0.7158862352371216
,5.632106781005859},rate=0.011665908625217637,rate_95%_HPD={0.00874102266
6085566,0.014717273846927633},rate_median=0.011700746097953687,rate_range
={0.00763049741176024,0.016954552646918895},!rotate=true]:2.54982,Odocoil
eus_lucasi[&height=5.98643678815579E-
7,height_95%_HPD={1.1920928955078125E-7,1.1473894119262695E-
6},height_median=5.960464477539062E-
7,height_range={0.0,1.4603137969970703E-
6},length=2.7875849471021983,length_95%_HPD={0.03164291754364967,5.088521
0037231445},length_median=2.6708192825317383,length_range={0.031642917543
64967,8.719322204589844},rate=0.010497021967573838,rate_95%_HPD={0.006708
3893689343045,0.014631867389372673},rate_median=0.010356661807511396,rate
_range={0.005232830596426181,0.018144108967786086},!rotate=true]:2.54982)
[&height=2.5194295371893065,height_95%_HPD={1.182255670428276,3.794416319
578886},height_median=2.5498201176524162,height_range={0.7158865928649902
,4.317578740417957},length=1.3839254486547854,length_95%_HPD={0.028739117
085933685,2.559396266937256},length_median=1.4075247049331665,length_rang
e={2.106949978042394E-
4,3.465005397796631},posterior=0.4673768308921438,rate=0.0109072403948658
45,rate_95%_HPD={0.006925549707986088,0.015155144658775746},rate_median=0
.010888386508019128,rate_range={0.006125200824425341,0.019155520122024795
},!rotate=true,!color=#ff0000]:1.394934,((Odocoileus_hemionus[&height=5.9
64122026831349E-7,height_95%_HPD={1.4528632164001465E-
7,1.169741153717041E-6},height_median=5.811452865600586E-
7,height_range={0.0,1.385807991027832E-
6},length=0.9906804853115235,length_95%_HPD={0.055256616324186325,1.53969
46668624878},length_median=1.1235393285751343,length_range={0.01600930094
718933,2.082707166671753},rate=0.009276692191663497,rate_95%_HPD={0.00641
9460019428826,0.012893690294364761},rate_median=0.009171288800362887,rate
_range={0.004319274286441181,0.016761252113253598},!rotate=true]:1.219867
,Odocoileus_virginianus_1[&height=5.939954996590172E-
7,height_95%_HPD={8.940696716308594E-8,1.1175870895385742E-
6},height_median=5.811452865600586E-
7,height_range={0.0,1.4007091522216797E-
6},length=1.1041374630561365,length_95%_HPD={0.3938829302787781,1.5830373
764038086},length_median=1.1427751779556274,length_range={0.0792511850595
4742,2.0663790702819824},rate=0.011977520766242849,rate_95%_HPD={0.008280
367842983284,0.015370369618170525},rate_median=0.011831117935304735,rate_
range={0.0069861579876147484,0.020021252989847926},!rotate=true]:1.219867
)[&height=1.227662173641986,height_95%_HPD={0.7972208242863417,1.53774475
3062725},height_median=1.2198671698570251,height_range={0.797220824286341
7,2.0029754266142845},length=0.9836647995627144,length_95%_HPD={6.9057050

8595556E-
4,2.870384693145752},length_median=0.7397340536117554,length_range={6.905
70508595556E-
4,3.147207736968994},posterior=0.30492676431424764,rate=0.010118458227501
955,rate_95%_HPD={0.006407762007288195,0.013883090781071847},rate_median=
0.010029988986921127,rate_range={0.0055755209057847,0.01662976513613206},
!rotate=true,!color=#ff0000]:0.073343,Odocoileus_cf._virginianus[&height=
5.940028628118624E-7,height_95%_HPD={1.1920928955078125E-
7,1.1175870895385742E-6},height_median=5.736947059631348E-
7,height_range={0.0,1.3709068298339844E-
6},length=1.1505422252100135,length_95%_HPD={0.01600930094718933,3.210626
6021728516},length_median=0.9455312490463257,length_range={0.016009300947
18933,7.063737869262695},rate=0.010652165074359848,rate_95%_HPD={0.006771
952831567657,0.015060579582815347},rate_median=0.01044508370815096,rate_r
ange={0.003908816865335434,0.019161962658348363},!rotate=true]:1.29321)[&
height=1.4414544394594238,height_95%_HPD={0.893643343821168,2.46211662143
46886},height_median=1.293210543692112,height_range={0.8338168263435364,3
.9941663034260273},length=2.1589090190653137,length_95%_HPD={0.4305913448
33374,3.2844443321228027},length_median=2.3107361793518066,length_range={
0.006511873565614223 5,3.938591718673706},posterior=0.9227696404793608,rat
e=0.0096857832652076 9,rate_95%_HPD={0.006892676665648396,0.0129291247208 7
1975},rate_median=0.009663869820193793,rate_range={0.005477903591278420 4,
0.015890043502955677},!rotate=true]:2.651544)[&height=4.004811543472731,h
eight_95%_HPD={3.1467990577220917,5.124562740325928},height_median=3.9447
54269439727,height_range={2.9365534484386444,7.330965355038643},length=3.
22728196692233,length_95%_HPD={2.0817906856536865,4.4503254890441895},len
gth_median=3.2623497247695923,length_range={0.4118765592575073,4.96734666
8243408},posterior=0.8149134487350199,rate=0.01004679029220707,rate_95%_H
PD={0.00745119891902825,0.013032841038309317},rate_median=0.0099199889596
95996,rate_range={0.006365511326423813,0.015402059292370044},!rotate=true
]:3.275291,(Mazama_rufina[&height=5.948099031171373E-
7,height_95%_HPD={5.9604644775390625E-8,1.1175870895385742E-
6},height_median=5.811452865600586E-
7,height_range={0.0,1.5422701835632324E-
6},length=0.8572572766593627,length_95%_HPD={0.6140080690383911,1.1908696
88987732},length_median=0.8423610925674438,length_range={0.03164291754364
967,1.579636812210083},rate=0.010897153923612018,rate_95%_HPD={0.00723393
7820383788,0.014382573948847016},rate_median=0.010736408643551212,rate_ra
nge={0.00527789304699359,0.018542375948133 09},!rotate=true]:0.843897,Pudu
_mephistophiles[&height=5.949934393901641E-
7,height_95%_HPD={5.9604644775390625E-8,1.1175870895385742E-
6},height_median=5.811452865600586E-
7,height_range={0.0,1.5422701835632324E-
6},length=0.8591067740388622,length_95%_HPD={0.6140080690383911,1.1908696
88987732},length_median=0.8424170613288879,length_range={0.15573991835117
34,1.579636812210083},rate=0.010238733286847709,rate_95%_HPD={0.006554884
099560483,0.013520523569061216},rate_median=0.01018863380535722,rate_rang
e={0.005459201735571594,0.017119491042761056},!rotate=true]:0.843897)[&he
ight=0.8641934972496241,height_95%_HPD={0.6140084266662598,1.172957301139
8315},height_median=0.8438979834318161,height_range={0.528859905898571,1.
5796369314193726},length=6.162464186244252,length_95%_HPD={4.533933639526
367,7.8116254806518555},length_median=6.320677280426025,length_range={0.0
88247150182724,8.07140064239502},posterior=0.9760319573901465,rate=0.0114
47197477542316,rate_95%_HPD={0.009309433268690456,0.013603065132106336},r
ate_median=0.011398581507177015,rate_range={0.008225603036026203,0.019676
86908854223},!rotate=true]:6.376147)[&height=7.230687683209614,height_95%
_HPD={6.274102717638016,8.302842238917947},height_median=7.22004504501819
6,height_range={5.739544376730919,8.941856697201729},length=2.04718207058
21235,length_95%_HPD={1.2122483253479004,2.923841714859009},length_median
=1.996545135974884,length_range={0.10778253525495529,3.492797613143921},p

osterior=0.8894806924101198,rate=0.01011543430243099,rate_95%_HPD={0.0068
9814714932891,0.01339755700088517 8},rate_median=0.01003868018888534,rate_
range={0.005514948176081298,0.017081385582988745},!rotate=true]:2.039436)
[&height=9.279364845978643,height_95%_HPD={8.287421464920044,10.146888032
55558},height_median=9.25948102772235 9,height_range={7.834977447986603,10
.68770346045494},length=1.1387604505851352,length_95%_HPD={0.585632085800
1709,1.8317389488220215},length_median=1.137093424797058,length_range={0.
0678568035364151,2.155306816101074},posterior=0.9653794940079894,rate=0.0
1148366758057 4324,rate_95%_HPD={0.007667971230628979,0.016026334609411948
},rate_median=0.011284372730000745,rate_range={0.006528552849547599,0.020
523815048357843},!rotate=true]:0.998238,Rangifer_tarandus_NC007703[&heigh
t=5.966790588436209E-7,height_95%_HPD={0.0,1.141335815191269E-
6},height_median=5.960464477539062E-
7,height_range={0.0,1.519918441772461E-
6},length=7.367466698950045,length_95%_HPD={3.4000632762908936,10.8649787
90283203},length_median=7.117804527282715,length_range={3.400063276290893
6,11.560518264770508},rate=0.009748294119878617,rate_95%_HPD={0.007369248
596862674,0.011923707134546088},rate_median=0.009745661778712056,rate_ran
ge={0.005392604277563631,0.01567003705871491},!rotate=true]:10.257718)[&h
eight=10.2860468134374,height_95%_HPD={9.376895189285278,11.2312166988849
64},height_median=10.257719039916992,height_range={9.033702936023474,11.5
6051853299141},length=2.95690030655063,length_95%_HPD={0.8252476453781128
,4.441131114959717},length_median=3.1553750038146973,length_range={0.0741
356611251831,4.933319091796875},posterior=0.33422103861517977,rate=0.0127
07394032458099,rate_95%_HPD={0.009131215312466766,0.01741834345201748},ra
te_median=0.01252781379038525,rate_range={0.007501331813192555,0.01914375
31633397},!rotate=true,!color=#ff0000]:3.374649)[&height=13.6311827316831
79,height_95%_HPD={12.509042426943779,14.802709549665451},height_median=1
3.632368005812168,height_range={12.000705659389496,15.355463534593582},le
ngth=1.067391259297291,length_95%_HPD={0.14491364359855652,1.930475473403
9307},length_median=1.0324138402938843,length_range={0.003413809696212411
,2.607797622680664},posterior=0.19840213049267644,rate=0.0108473294768102
97,rate_95%_HPD={0.006922221977741097,0.015226892181112095},rate_median=0
.010757957515255451,rate_range={0.006600380417182955,0.018598098966504915
},!rotate=true,!color=#ff0000]:0.291585,(Croizetoceros_pyrenaicus[&height
=5.829927807537245,height_95%_HPD={5.304703578352928,6.711183479055762},h
eight_median=5.7018815274350345,height_range={5.300120204687118 5,6.999535
068869591},length=0.6419425041826514,length_95%_HPD={0.0,2.23097515106201
17},length_median=0.3256840407848358,length_range={0.0,7.424014568328857}
,rate=0.010801369078087375,rate_95%_HPD={0.007187573407661719,0.015861170
365571584},rate_median=0.010652000177041513,rate_range={0.005558150282069
748,0.02078925629330467 5},!rotate=true]:0.688529,Croizetoceros_ramosus[&h
eight=3.763668965986966,height_95%_HPD={3.4012838546186686,4.148857422173
023},height_median=3.746121644973755,height_range={3.400063633918762,4.19
896918162703 5},length=1.916088775841218,length_95%_HPD={0.0,4.16386318206
7871},length_median=1.9335191249847412,length_range={0.0,9.21120738983154
3},rate=0.010800784335199699,rate_95%_HPD={0.006886970699552545,0.0147189
47173079948},rate_median=0.010737662434470623,rate_range={0.0051958612376
07247,0.021602117116700438},!rotate=true]:2.644289)[&height=6.65024555779
6861,height_95%_HPD={5.342511236667633,8.714125543832779},height_median=6
.390410289168358,height_range={5.342511236667633,12.745914928615093},leng
th=5.458277294677487,length_95%_HPD={0.02106008119881153,9.08622455596923
8},length_median=6.035485744476318,length_range={0.02106008119881153,10.5
93926429748535},posterior=0.41145139813581894,rate=0.010792291548825092,r
ate_95%_HPD={0.006759233165935492,0.015680571706029493},rate_median=0.010
57791739865104 9,rate_range={0.004856127878522685,0.01954851676097654},!ro
tate=true,!color=#ff0000]:7.533543)[&height=13.97256583989317,height_95%_
HPD={12.553294837474823,15.313687592744827},height_median=13.923953324556
35,height_range={12.073503836989403,16.70235649496317},length=1.226702279
531547,length_95%_HPD={0.037547916173934937,1.9751942157745361},length_me

dian=1.262813687324524,length_range={0.026843665167689323,2.5399360656738
28},posterior=1.0,rate=0.011171308817994009,rate_95%_HPD={0.0076063499031
223435,0.015466848126286644},rate_median=0.011020593623326774,rate_range=
{0.006299438412611392,0.019484122287360184},!rotate=true]:1.279124,((((((
Axis_axis[&height=6.046252958873379E-
7,height_95%_HPD={2.9802322387695312E-8,1.1324882507324219E-
6},height_median=5.960464477539062E-
7,height_range={0.0,1.471489667892456E-
6},length=3.789325222035699,length_95%_HPD={2.99931001663208,4.6708784103
393555},length_median=3.717561960220337,length_range={2.7333500385284424,
5.266963005065918},rate=0.010306358278474526,rate_95%_HPD={0.007929259946
236038,0.01277233563408906},rate_median=0.010317151755439848,rate_range={
0.006925827157619196,0.014753077699821794},!rotate=true]:3.705024,Axis_po
rcinus[&height=6.043786690204582E-7,height_95%_HPD={2.9802322387695312E-
8,1.1324882507324219E-6},height_median=5.960464477539062E-
7,height_range={0.0,1.471489667892456E-
6},length=3.7934483799255005,length_95%_HPD={2.99931001663208,4.672191619
873047},length_median=3.730372428894043,length_range={2.7333500385284424,
5.266963005065918},rate=0.011180077326399265,rate_95%_HPD={0.008585144284
355738,0.014011090151863614},rate_median=0.011106769808262588,rate_range=
{0.00735891812144271,0.01652851601447768},!rotate=true]:3.705024)[&height
=3.7709525325291886,height_95%_HPD={2.99931070022285,4.6597514152526855},
height_median=3.7050245627760887,height_range={2.733350788243115,5.266963
569447398},length=1.2522107736117694,length_95%_HPD={0.007931490428745747
,2.0892159938812256},length_median=1.3515031337738037,length_range={0.007
931490428745747,3.2739992141723633},posterior=0.8695073235685752,rate=0.0
10588549275006845,rate_95%_HPD={0.006636832657431679,0.014524819130833875
},rate_median=0.010470601739461603,rate_range={0.005467376159062189,0.018
519812086361772},!rotate=true]:0.532234,Metacervocerus_philisi_1[&height=
3.74874011489716,height_95%_HPD={3.4000855684280396,4.15682889521122},hei
ght_median=3.718257997184992,height_range={3.4000855684280396,4.198444321
7515945},length=0.8629063360032873,length_95%_HPD={0.0,2.6196787357330322
},length_median=0.5772008895874023,length_range={0.0,5.8790788650512695},
rate=0.010768368677457488,rate_95%_HPD={0.007114314724440242,0.0151205418
50695127},rate_median=0.010617741491566149,rate_range={0.0056122391162946
25,0.018798059665194152},!rotate=true]:0.519)[&height=4.33472614203768,he
ight_95%_HPD={3.5075006932020187,5.354872941970825},height_median=4.23725
8240580559,height_range={3.5075006932020187,6.530801037326455},length=1.0
4046396373355,length_95%_HPD={0.013019784353673458,1.8899140357971191},le
ngth_median=1.1176114082336426,length_range={0.013019784353673458,2.43779
5639038086},posterior=0.3129161118508655,rate=0.01050482236524218,rate_95
%_HPD={0.007063702628079829,0.014302604240131246},rate_median=0.010252377
61019157,rate_range={0.006300526179352145,0.022093300096006945},!rotate=t
rue,!color=#ff0000]:1.510681,(Rucervus_duvauceli[&height=6.01814896706615
7E-7,height_95%_HPD={0.0,1.1324882507324219E-
6},height_median=5.960464477539062E-
7,height_range={0.0,1.4454126358032227E-
6},length=4.836720313594122,length_95%_HPD={3.402686357498169,6.148852348
327637},length_median=4.953930377960205,length_range={3.402686357498169,6
.9207963943481445},rate=0.008847143165774857,rate_95%_HPD={0.006761550199
932521,0.010923947470067903},rate_median=0.008827316856742722,rate_range=
{0.005872069449061739,0.01256584958262467},!rotate=true]:4.25125,Metacerv
ocerus_philisi_2[&height=3.7291939767591744,height_95%_HPD={3.40112726204
09727,4.144232168793678},height_median=3.6848804280161858,height_range={3
.400303840637207,4.199918493628502},length=1.5846616985416164,length_95%_
HPD={0.0,4.09199333190918},length_median=1.3631781339645386,length_range=
{0.0,7.7519378662109375},rate=0.011177859721546307,rate_95%_HPD={0.006635
327547329538,0.015378556360011527},rate_median=0.011016695568791991,rate_
range={0.005573756115351396,0.020142149070726274},!rotate=true]:0.56637)[
&height=4.356688792988254,height_95%_HPD={3.53179326467216,5.512199111282

8255},height_median=4.251250565052 0325,height_range={3.4536321982741356,5
.949619710445404},length=0.9654130536307405,length_95%_HPD={0.03668623417
6158905,2.2000272274017334},length_median=0.8943178653717041,length_range
={0.036686234176158905,2.872870683670044},posterior=0.1904127829560586,ra
te=0.010114959170990048,rate_95%_HPD={0.006209005151766339,0.013508541303
638932},rate_median=0.009973607821242295,rate_range={0.005718658234115745
,0.017227104846075255},!rotate=true,!color=#ff0000]:1.496689)[&height=5.9
08613810238022,height_95%_HPD={4.61352101713419,7.788421485573053},height
_median=5.747939735651016,height_range={4.138533271849155,9.3495378345251
08},length=1.9479259183370945,length_95%_HPD={0.1393240988254547,3.128755
569458008},length_median=2.090397357940674,length_range={7.82895192969590
4E-
4,3.710559368133545},posterior=0.810918774966711,rate=0.01009192560121916
8,rate_95%_HPD={0.006376144322647774,0.013508717761574373},rate_median=0.
010004862891916398,rate_range={0.004793661968568883,0.017611645394711835}
,!rotate=true]:2.21339,((((((Cervus_elaphus_NC007704[&height=5.9647199923
49254E-7,height_95%_HPD={8.940696716308594E-8,1.1399388313293457E-
6},height_median=5.736947059631348E-
7,height_range={0.0,1.5832483768463135E-
6},length=2.6631869050697703,length_95%_HPD={2.372208595275879,3.16955757
1411133},length_median=2.623727798461914,length_range={2.111531972885132,
3.8094322681427},rate=0.01102834279024877,rate_95%_HPD={0.007891395528533
561,0.013887777375059307},rate_median=0.011111863150533469,rate_range={0.
0059509049929298,0.01491199520929868 5},!rotate=true]:2.678731,(Cervus_nip
pon_centralis_NC006993[&height=5.9574281458263E-
7,height_95%_HPD={8.940696716308594E-8,1.1473894119262695E-
6},height_median=5.811452865600586E-
7,height_range={0.0,1.5348196029663086E-
6},length=2.1407270323579386,length_95%_HPD={1.5678907632827759,2.7402057
64770508},length_median=2.0924792289733887,length_range={1.37137246131896
97,3.059450149536133},rate=0.011296704082762472,rate_95%_HPD={0.007952883
187458314,0.013850793254904778},rate_median=0.011317779391766257,rate_ran
ge={0.007049744286315131,0.016177313436603873},!rotate=true]:2.092479,Prz
ewalskium_albirostris[&height=5.9574281458263E-
7,height_95%_HPD={8.940696716308594E-8,1.1473894119262695E-
6},height_median=5.811452865600586E-
7,height_range={0.0,1.5348196029663086E-
6},length=2.1407270323579386,length_95%_HPD={1.5678907632827759,2.7402057
64770508},length_median=2.0924792289733887,length_range={1.37137246131896
97,3.059450149536133},rate=0.012653802643855615,rate_95%_HPD={0.009230638
340452972,0.015904830476857285},rate_median=0.012657846761932781,rate_ran
ge={0.007628692979351751,0.018992594400301804},!rotate=true]:2.092479)[&h
eight=2.1407276281007532,height_95%_HPD={1.567891500890255,2.740205794572
83},height_median=2.092479884624481,height_range={1.3713729828596115,3.05
94508424401283},length=0.6972569629014252,length_95%_HPD={0.3734540343284
607,1.040212631225586},length_median=0.6797759532928467,length_range={0.1
7185397446155548,1.4808536767959595},posterior=1.0,rate=0.011341559590501
865,rate_95%_HPD={0.007015410859117657,0.0156305146042757 5},rate_median=0
.011251559249128316,rate_range={0.005848479486425263,0.020658842880482007
},!rotate=true]:0.586252)[&height=2.69144668933677,height_95%_HPD={2.2893
926054239273,3.1497233361005783},height_median=2.678731694817543,height_r
ange={2.1115327700972557,3.8094327673316},length=0.1942103828619403,lengt
h_95%_HPD={0.06446869671344757,0.35276585817337036},length_median=0.18637
514114379883,length_range={0.0058118924498558044,0.4663824737071991},post
erior=0.6830892143808256,rate=0.010949132871939379,rate_95%_HPD={0.006817
779051150043,0.01449965947625413},rate_median=0.010762797334352332,rate_r
ange={0.005944975749849903,0.01838108776787632},!rotate=true]:0.194441,(R
usa_timorensis[&height=5.957657566167583E-
7,height_95%_HPD={1.043081283569336E-7,1.1362135410308838E-
6},height_median=5.811452865600586E-

7,height_range={0.0,1.4901161193847656E-
6},length=2.2187175695175494,length_95%_HPD={1.5681899785995483,2.8130683
89892578},length_median=2.1811411380767822,length_range={1.54946267604827
88,3.544837713241577},rate=0.009290433910496967,rate_95%_HPD={0.006762975
287941206,0.012047601864087638},rate_median=0.009294507637325217,rate_ran
ge={0.00532722705951679,0.013495495302400044},!rotate=true]:2.179531,Rusa
_unicolor_NC008414[&height=5.957459148575122E-
7,height_95%_HPD={1.043081283569336E-7,1.1362135410308838E-
6},height_median=5.811452865600586E-
7,height_range={0.0,1.4901161193847656E-
6},length=2.2187522745005457,length_95%_HPD={1.5966317653656006,2.8329434
394836426},length_median=2.1811411380767822,length_range={1.5494626760482
788,3.544837713241577},rate=0.008487924600177716,rate_95%_HPD={0.00607340
1250668142,0.010779777461993076},rate_median=0.008479486247052867,rate_ran
ge={0.0052058202269371,0.013644794450581},!rotate=true]:2.179531)[&height
=2.21729833771182,height_95%_HPD={1.5681905895471573,2.813068985939026},h
eight_median=2.179531916975975,height_range={1.549463152885437,3.54483813
04740906},length=0.8169561835510708,length_95%_HPD={0.4098624587059021,1.
2275129556655884},length_median=0.8025556802749634,length_range={0.073096
1486697197,1.6045894622802734},posterior=0.9973368841544608,rate=0.009407
648156044284,rate_95%_HPD={0.005980447334011671,0.013139654333060611},rat
e_median=0.00923328714706458,rate_range={0.004672402801420311,0.021375729
339682983},!rotate=true]:0.693641)[&height=2.884814334876626,height_95%_H
PD={2.505319371819496,3.3186377487145364},height_median=2.873173071071505
5,height_range={2.2754486799240112,3.996087059378624},length=0.7171283299
742709,length_95%_HPD={0.31146350502967834,1.1738356351852417},length_med
ian=0.7048469483852386,length_range={0.004380523692816496,1.5591543912887
573},posterior=0.6498002663115846,rate=0.010789987471826934,rate_95%_HPD=
{0.006653740100865183,0.01477864626989589},rate_median=0.0106828316140967
6,rate_range={0.005682708704696125,0.021810159205125898},!rotate=true]:0
.74441,Rusa_alfredi[&height=5.939719375699124E-
7,height_95%_HPD={0.0,1.087784767150879E-
6},height_median=5.885958671569824E-
7,height_range={0.0,1.4901161193847656E-
6},length=3.713164257146071,length_95%_HPD={2.6134262084960938,4.56121778
4881592},length_median=3.6845784187316895,length_range={2.502282857894897
5,5.147170543670654},rate=0.010438092804859732,rate_95%_HPD={0.0081968666
82174082,0.01262707306090916},rate_median=0.010478864644205721,rate_range
={0.007362402647889792,0.013952498533148701},!rotate=true]:3.617582)[&hei
ght=3.629875829944145,height_95%_HPD={3.151222236454487,4.177852511405945
},height_median=3.6175826713442802,height_range={3.0165005922317505,4.471
48622572422},length=0.7712165209345329,length_95%_HPD={0.3449566066265106
,1.1984186172485352},length_median=0.7609741389751434,length_range={0.010
885544121265411,1.5698094367980957},posterior=0.5193075898801598,rate=0.0
10929315805297934,rate_95%_HPD={0.007472660195219823,0.015527392185707825
},rate_median=0.010913866391383868,rate_range={0.006011249547187 45,0.0179
16982867784176},!rotate=true]:0.298657,Eucladoceros_ctenoides[&height=2.6
49946984949602,height_95%_HPD={2.5000277161598206,2.937023401260376},heig
ht_median=2.5996751189231873,height_range={2.5000277161598206,2.999502569
437027},length=0.6936549350309521,length_95%_HPD={0.0,3.4782872200012207}
,length_median=0.12321680784225464,length_range={0.0,9.524391174316406},r
ate=0.010997849313883543,rate_95%_HPD={0.006816931505600665 5,0.0154321539
48970904},rate_median=0.010703494972577303,rate_range={0.0055825191296779
36,0.01916893413863322},!rotate=true]:1.316564)[&height=3.939387480150409
,height_95%_HPD={3.1930766254663467,4.616280265152454},height_median=3.91
62393622100353,height_range={3.1538113951683044,5.147170722484589},length
=0.797777297083663,length_95%_HPD={0.4490777552127838,1.2296525239944458}
,length_median=0.7839510142803192,length_range={0.12613487243652344,1.342
6545858383179},posterior=0.5033288948069241,rate=0.010638723152205785,rat
e_95%_HPD={0.007440872088936008,0.014640029771830432},rate_median=0.01037

8889429986557,rate_range={0.005968491394083462,0.019924079569547885},!rot
ate=true]:0.710282,(Elaphurus_davidianus[&height=5.927287273421467E-
7,height_95%_HPD={5.9604644775390625E-8,1.1026859283447266E-
6},height_median=5.662441253662109E-
7,height_range={0.0,1.475214958190918E-
6},length=3.2752576607680033,length_95%_HPD={2.4895541667938232,4.0746788
97857666},length_median=3.247847557067871,length_range={2.289959907531738
3,4.664518356323242},rate=0.008225597913592269,rate_95%_HPD={0.0060787205
32259118,0.010222102020055806},rate_median=0.008164253111419172,rate_rang
e={0.005249346475612345,0.012674785789946016},!rotate=true]:3.248873,Ruce
rvus_eldi[&height=5.930641770844017E-
7,height_95%_HPD={1.1920928955078125E-7,1.171603798866272E-
6},height_median=5.662441253662109E-
7,height_range={0.0,1.475214958190918E-
6},length=3.2728503198026817,length_95%_HPD={2.4895541667938232,4.0600452
4230957},length_median=3.248898506164551,length_range={2.2899599075317383
,4.664518356323242},rate=0.010387441129155071,rate_95%_HPD={0.00794012154
8900283,0.0129462429628474 4},rate_median=0.010256883621107226,rate_range=
{0.006903636035614603,0.015262932048631868},!rotate=true]:3.248873)[&heig
ht=3.2773407432512127,height_95%_HPD={2.4690880328416824,4.06004604697227
5},height_median=3.2488731890916824,height_range={2.2899603843688965,4.66
4519116282463},length=1.274730273161841,length_95%_HPD={0.581896245479583
7,2.03542160987854},length_median=1.257765769958496,length_range={0.06307
30539560318,2.9726388454437256},posterior=0.9560585885486018,rate=0.00945
170853600062,rate_95%_HPD={0.005769476155542032,0.01313490291920324},rate
_median=0.009351510704296382,rate_range={0.004917795563375261,0.019401774
016055764},!rotate=true]:1.377648)[&height=4.703577104966919,height_95%_H
PD={3.9679659008979797,5.5020333006978035},height_median=4.62652167677879
3,height_range={3.8193585835397243,6.5550002455711365},length=1.566236245
7981243,length_95%_HPD={0.9033262729644775,2.2545154094696045},length_med
ian=1.5400516986846924,length_range={0.4033570885658264,3.114277362823486
3},posterior=0.5739014647137151,rate=0.009699943632516684,rate_95%_HPD={0
.006560656308277485,0.013163682351009745},rate_median=0.00954667924898835
8,rate_range={0.005492222507339262,0.018100576030678297},!rotate=true]:1.
638836,((Dama_dama_dama[&height=6.02032846030835E-
7,height_95%_HPD={0.0,1.1026859283447266E-
6},height_median=5.662441253662109E-
7,height_range={0.0,1.5348196029663086E-
6},length=2.9165214146183587,length_95%_HPD={1.952228307723999,4.66769695
2819824},length_median=2.6972897052764893,length_range={1.952228307723999
,6.201586723327637},rate=0.011618656576278858,rate_95%_HPD={0.00812467238
0610947,0.014852775792135226},rate_median=0.011613385008948783,rate_range
={0.006542775167026293,0.017227104846075255},!rotate=true]:2.898614,Dama_
eurygonos[&height=2.41783665967651,height_95%_HPD={1.9522290080785751,3.2
03091189265251},height_median=2.3184208273887634,height_range={1.95041784
6441269,3.397843226790428},length=0.4452315578979692,length_95%_HPD={0.0,
1.720126748085022},length_median=0.1816006451845169,length_range={0.0,4.5
50197601318359},rate=0.010887731355641724,rate_95%_HPD={0.006380678930183
888,0.014944991277324094},rate_median=0.010656293080718806,rate_range={0.
005392604277563631,0.02069280869843818},!rotate=true]:0.580193)[&height=3
.0322067637158585,height_95%_HPD={2.085716634988785,4.295782858505845},he
ight_median=2.89861420635134,height_range={1.9964041109196842,5.892182897
776365},length=2.2598178983537545,length_95%_HPD={0.5498784780502319,3.90
97769260406494},length_median=2.2938960790634155,length_range={0.07753012
329339981,4.4645915031433105},posterior=0.6178428761651131,rate=0.0115740
76065077989,rate_95%_HPD={0.007854616866471672,0.015659516226214587},rate
_median=0.01133234974453753,rate_range={0.006925549707986088,0.0189370547
95833467},!rotate=true]:2.24082,Megaloceros_giganteus_AM182644[&height=0.
27122759557270676,height_95%_HPD={0.24430974572896957,0.29970091581344604
},height_median=0.2708228677511215,height_range={0.2421422302722931,0.300

97822844982147},length=4.8743233306112685,length_95%_HPD={3.4859983921051
025,6.456793785095215},length_median=4.884158611297607,length_range={1.75
85480213165283,7.648420333862305},rate=0.01100536177463825,rate_95%_HPD={
0.007552147538198647,0.014338580650926863},rate_median=0.0108896365039826
12,rate_range={0.005778368127414527,0.01846962678030812},!rotate=true]:4.
868611)[&height=5.154969805007568,height_95%_HPD={4.0437198877334595,6.32
8430882655084},height_median=5.139434260316193,height_range={3.4977082936
09321,6.971606183797121},length=0.9971243217120416,length_95%_HPD={0.1100
0196635723114,1.9356508255004883},length_median=0.9782798290252686,length
_range={0.006792180705815554,2.6507272720336914},posterior=0.889480692410
1198,rate=0.010979577573211345,rate_95%_HPD={0.00703640253134705,0.015072
589119967254},rate_median=0.010748080505714288,rate_range={0.004950350961
707795,0.02278723629122121},!rotate=true]:1.125923)[&height=6.29700363398
6885,height_95%_HPD={5.4230897799134254,7.124143574386835},height_median=
6.265357628464699,height_range={5.144484039396048,7.942700881976634},leng
th=1.6561948406295135,length_95%_HPD={0.8406364917755127,2.41837811470031
74},length_median=1.6135260462760925,length_range={0.16540661454200745,3.
334547281265259},posterior=0.5539280958721704,rate=0.009916615576169905,r
ate_95%_HPD={0.006577089681973236,0.0137847001153846},rate_median=0.00983
5173292890291,rate_range={0.004793661968568883,0.018337394188617042},!rot
ate=true]:1.695972)[&height=8.006243080297017,height_95%_HPD={6.987832918
763161,9.14283213019371},height_median=7.961329601705074,height_range={6.
5988695323467255,11.858412511646748},length=4.498660309556831,length_95%_
HPD={2.320310592651367,5.909894943237305},length_median=4.60330057144165,
length_range={0.30556511878967285,6.548364162445068},posterior=0.60319573
90146472,rate=0.009096218629383122,rate_95%_HPD={0.006805683606447274,0.0
11653638108662163},rate_median=0.008991621473143618,rate_range={0.0063060
02596981167,0.013855472195475088},!rotate=true]:4.584199,((Elaphodus_ceph
alophus_N008749[&height=5.887826587186355E-
7,height_95%_HPD={0.0,1.1380761861801147E-
6},height_median=5.960464477539062E-
7,height_range={0.0,1.4603137969970703E-
6},length=7.756860227940403,length_95%_HPD={3.520810842514038,11.72063636
7797852},length_median=7.590379238128662,length_range={2.7550225257873535
,13.339049339294434},rate=0.010596383100032313,rate_95%_HPD={0.0078927223
40963484,0.013801941954115191},rate_median=0.01059855625882915,rate_range
={0.005582048642446789,0.01592772705816991},!rotate=true]:6.00308,Cervus_
ruscinensis[&height=4.121884690223915,height_95%_HPD={3.501530349254608,4
.861120596528053},height_median=4.063543569296598,height_range={3.5015303
49254608,4.999523177742958},length=3.744678864945049,length_95%_HPD={0.0,
9.12176513671875},length_median=3.269943952560425,length_range={0.0,11.63
4778022766113},rate=0.011582480693065084,rate_95%_HPD={0.0075482316076418
81,0.016319124954676554},rate_median=0.01127862850548829,rate_range={0.00
6488909677678691,0.020142149070726274},!rotate=true]:1.939537)[&height=6.
411735529771997,height_95%_HPD={3.6195297837257385,9.903097242116928},hei
ght_median=6.0030809212476015,height_range={3.6195297837257385,12.1696503
83293629},length=4.536561095947835,length_95%_HPD={0.48948925733566284,7.
52872896194458},length_median=4.812227725982666,length_range={0.043680034
57784653,8.39008903503418},posterior=0.5699067909454061,rate=0.0109831878
08249998,rate_95%_HPD={0.007304816738283093,0.01458443364341139},rate_med
ian=0.010833508241436368,rate_range={0.006722050188145297,0.0183691141027
10544},!rotate=true]:5.207703,((Muntiacus_crinifrons_NC004577[&height=5.8
75716138427727E-7,height_95%_HPD={1.1920928955078125E-
7,1.1473894119262695E-6},height_median=5.811452865600586E-
7,height_range={0.0,1.4156103134155273E-
6},length=3.221818782359401,length_95%_HPD={2.5228517055511475,3.96642494
20166016},length_median=3.194323778152466,length_range={2.426220417022705
,4.583507061004639},rate=0.010687741982616617,rate_95%_HPD={0.00804402867
1770436,0.013207167694303622},rate_median=0.010614539358830758,rate_range
={0.006785688665595418,0.01588462376776357},!rotate=true]:3.21246,Muntiac

us_muntjak_NC_004563[&height=5.871450160189808E-
7,height_95%_HPD={1.1920928955078125E-7,1.1473894119262695E-
6},height_median=5.811452865600586E-
7,height_range={0.0,1.4156103134155273E-
6},length=3.2309494828098466,length_95%_HPD={2.5365452766418457,3.9664249
420166016},length_median=3.2049899101257324,length_range={2.4262204170227
05,4.583507061004639},rate=0.012450000043296288,rate_95%_HPD={0.009593084
12319484,0.015177389035202354},rate_median=0.01243599239132903,rate_range
={0.007951079663281985,0.016806855132015},!rotate=true]:3.21246)[&heigh
t=3.2407648819314336,height_95%_HPD={2.598727114032954,3.9940969944000244
},height_median=3.212460272014141,height_range={2.426221337169409,4.58350
7910370827},length=1.112263743204742,length_95%_HPD={0.5666841864585876,1
.7969391345977783},length_median=1.0964758396148682,length_range={0.02285
7069969177246,2.309025764465332},posterior=0.9374167776298269,rate=0.0112
34425505937725,rate_95%_HPD={0.006804937052426302,0.015543882078839768},r
ate_median=0.011073697175960304,rate_range={0.005950675503031915,0.020724
64283682881 3},!rotate=true]:1.140694,(Muntiacus_reevesi_NC008491[&height=
5.891892597694371E-7,height_95%_HPD={0.0,1.0728836059570312E-
6},height_median=5.811452865600586E-
7,height_range={0.0,1.5944242477416992E-
6},length=3.804437235096958,length_95%_HPD={2.5529727935791016,4.78338527
6794434},length_median=3.7795510292053223,length_range={2.500791311264038
,5.7144622802734375},rate=0.009771220240812301,rate_95%_HPD={0.0074556963
39059461,0.01212061046831333},rate_median=0.009730737236966559,rate_range
={0.006414976753397949,0.014295408146235906},!rotate=true]:3.825829,Munti
acus_vuquangensis[&height=5.865311615923035E-
7,height_95%_HPD={0.0,1.0561197996139526E-
6},height_median=5.811452865600586E-
7,height_range={0.0,1.5944242477416992E-
6},length=3.805912217509731,length_95%_HPD={2.8285038471221924,4.93880128
8604736},length_median=3.7846181392669678,length_range={2.507093429565429
7,5.85165548324585},rate=0.010116575789823713,rate_95%_HPD={0.00755342920
5901302,0.012666577379000702},rate_median=0.010060391825158596,rate_range
={0.006300526179352145,0.013772846044428374},!rotate=true]:3.825829)[&hei
ght=3.870463929900564,height_95%_HPD={3.0805417420342565,4.84073007106781
},height_median=3.8258295813575387,height_range={2.8519956953823566,5.673
448391258717},length=0.5152996092847867,length_95%_HPD={0.127865776419639
6,0.949535310268402 1},length_median=0.4935186356306076,length_range={0.00
9416886605322361,1.4426429271697998},posterior=0.8095872170439414,rate=0.
010124973121046885,rate_95%_HPD={0.006138147478883847 6,0.0137899670555169
14},rate_median=0.010017535224757975,rate_range={0.004917795563375261,0.0
20939205076688 6},!rotate=true]:0.527325)[&height=4.404937070819225,height
_95%_HPD={3.706449471414089,5.38424801081419},height_median=4.35315447673
2016,height_range={3.5519158244132996,5.914797410368919},length=6.0905228
944725955,length_95%_HPD={1.3175551891326904,8.473470687866211},length_me
dian=6.467314004898071,length_range={0.001489252899773419,9.2325935363769
53},posterior=0.7057256990679095,rate=0.010317267025222778,rate_95%_HPD={
0.008091728167595574,0.013206838454880788},rate_median=0.010203679699214 6
27,rate_range={0.006399662621690009,0.014969161353252098},!rotate=true]:6
.857629)[&height=11.214163895480157,height_95%_HPD={9.74709689617157,12.4
45240996778011},height_median=11.210783779621124,height_range={9.18913090
2290344,13.22970899194479},length=1.4820996366543313,length_95%_HPD={0.57
46485590934753,2.4771628379821777},length_median=1.458625078201294,length
_range={0.0166355948895216,2.9768826961517334},posterior=0.48601864181091
875,rate=0.010643057102201307,rate_95%_HPD={0.006807209105348067,0.014587
617254025138},rate_median=0.010385719606967438,rate_range={0.005500903885
8964045,0.018741894889841643},!rotate=true,!color=#ff0000]:1.334744)[&hei
ght=12.527705724325585,height_95%_HPD={11.20258041843772,13.9329356104135
51},height_median=12.545528165996075,height_range={10.32661384344101,14.9
94893744587898},length=1.869819485251772,length_95%_HPD={0.67164474725723

27,3.3328142166137695},length_median=1.813430905342102,length_range={0.12
281385064125061,3.9648666381835938},posterior=0.9414114513981359,rate=0.0
09898052995593683,rate_95%_HPD={0.006240122680691591,0.013231770562194744
},rate_median=0.009795611109312522,rate_range={0.005626472783998645,0.017
42449258954885},!rotate=true]:1.842273,Euprox_furcatus[&height=12.9148016
46011123,height_95%_HPD={12.500022768974304,13.643206773325801},height_me
dian=12.801772058010101,height_range={12.500022768974304,14.8662599474191
67},length=1.4294001081507013,length_95%_HPD={0.1982085108757019,2.677040
3385162354},length_median=1.3974800109863281,length_range={0.0,3.60767650
60424805},rate=0.012049494572013715,rate_95%_HPD={0.007143817264299l704,0
.017560707890148915},rate_median=0.011729993051367455,rate_range={0.00595
3114613288365,0.024471680183577173},!rotate=true]:1.586029)[&height=14.40
7856365327456,height_95%_HPD={13.143351458013058,15.485922574996948},heig
ht_median=14.38780115172267,height_range={12.950626634061337,16.391258517
28022},length=0.7951198496426913,length_95%_HPD={0.0105875413864851,1.612
50901222229},length_median=0.7451868057250977,length_range={0.00733062112
7039194,3.0403287410736084},posterior=0.9946737683089214,rate=0.010762537
661726163,rate_95%_HPD={0.00684185958059007,0.01487214710105264 6},rate_me
dian=0.010628729546385177,rate_range={0.005642222395537096,0.022093300096
006945},!rotate=true]:0.815276)[&height=15.199268119424717,height_95%_HPD
={14.102531924843788,16.39080621302128},height_median=15.203077644109726,
height_range={13.57583338022232,17.173164799809456},length=2.460129512332
568,length_95%_HPD={0.04051026701927185,5.104713439941406},length_median=
2.3390603065490723,length_range={0.025425877422094345,7.249337196350098},
posterior=1.0,rate=0.013167098645126336,rate_95%_HPD={0.00830433898673399
8,0.01774696218543308},rate_median=0.013011500835084339,rate_range={0.006
342218049990022,0.021221706766106803},!rotate=true]:2.851259,(Dicrocerus_
elegans[&height=15.87507097073443,height_95%_HPD={15.200415596365929,16.8
07630136609077},height_median=15.780582517385483,height_range={15.2004155
96365929,16.995032727718353},length=1.3639993156069585,length_95%_HPD={0.
0,3.5406908988952637},length_median=1.1656882762908936,length_range={0.0,
6.1026716232299805},rate=0.011061407341194766,rate_95%_HPD={0.00661166897
0796473,0.015085525645748138},rate_median=0.010947469671876021,rate_range
={0.0054778330421697856,0.018980562279719376},!rotate=true]:0.958606,Eost
yloceros_hezhengensis[&height=8.104094366899682,height_95%_HPD={7.1318249
106407166,8.976631447672844},height_median=8.163010470569134,height_range
={7.000117406249046,8.996598780155182},length=8.750573132866709,length_95
%_HPD={6.253302574157715,11.446269035339355},length_median=8.567869186401
367,length_range={5.527644634246826,14.313974380493164},rate=0.0106290654
4792446,rate_95%_HPD={0.006894759310550989,0.014675538812823486},rate_med
ian=0.0105312811246608 9,rate_range={0.0057759601552361435,0.0227872362912
2121},!rotate=true]:8.576178)[&height=16.903291841293367,height_95%_HPD={
15.274116456508636,18.77222865819931},height_median=16.739188596606255,he
ight_range={15.274116456508636,20.921831369400024},length=1.6649609759567
165,length_95%_HPD={0.004773970227688551,4.143695831298828},length_median
=1.3001934885978699,length_range={0.004773970227688551,6.775301933288574}
,posterior=0.3914780292942743,rate=0.011038576637187675,rate_95%_HPD={0.0
06541940202707861,0.01512218526476385},rate_median=0.010871938918214535,r
ate_range={0.005072335491533211,0.019247942620309173},!rotate=true,!color
=#ff0000]:1.315148)[&height=18.22674298857096,height_95%_HPD={16.30211514
979601,20.533152014017105},height_median=18.05433663725853,height_range={
15.81038365047425,22.29130107164383},length=3.5579235217089367,length_95%
_HPD={1.3112807273864746,5.589343547821045},length_median=3.6563940048217
773,length_range={0.0513087622821331,6.464219570159912},posterior=0.97336
88415446072,rate=0.01468335186068078,rate_95%_HPD={0.010472219287164923,0
.019503307524401665},rate_median=0.014640029771830432,rate_range={0.00748
9830278240485,0.022599306412304163},!rotate=true]:3.79249,((Hereroprox_la
rteti[&height=16.51446604140065,height_95%_HPD={15.27439945936203,17.8844
28024291992},height_median=16.51890578866005,height_range={15.20359861850
7385,17.993229374289513},length=1.5476037018973325,length_95%_HPD={0.1514

6711468696594,2.937694787979126},length_median=1.4479256868362427,length_
range={0.0,4.71018123626709},rate=0.010926924488421402,rate_95%_HPD={0.00
680056987798605,0.015175861061161726},rate_median=0.010713113141921122,r
ate_range={0.004564789670462965,0.019503307524401665},!rotate=true]:1.400
531,Procervulus_dichotomus[&height=17.677175399834773,height_95%_HPD={17.
00049076974392,18.760976143181324},height_median=17.540222190320492,heigh
t_range={17.00049076974392,19.983597327023745},length=0.39717680612887774
,length_95%_HPD={0.0,1.230303168296814},length_median=0.2758913040161133,
length_range={0.0,2.364839792251587},rate=0.010982063640534499,rate_95%_H
PD={0.006345438165806608,0.015206353671245 81},rate_median=0.0108008944143
78346,rate_range={0.005912824983091931,0.021070797821841476},!rotate=true
]:0.379215)[&height=17.973847562032187,height_95%_HPD={17.175438970327377
,18.91396559216082},height_median=17.919437223579735,height_range={17.106
069050729275,19.455583035945892},length=0.591544623165769,length_95%_HPD=
{0.002230706624686718,1.53494131565094},length_median=0.4932640790939331,
length_range={0.002230706624686718,2.5344886779785156},posterior=0.492676
43142476697,rate=0.010763303013620391,rate_95%_HPD={0.006918603008504216,
0.014747089579896313},rate_median=0.010583288337170572,rate_range={0.0053
41969950368749,0.021375729339682983},!rotate=true,!color=#ff0000]:0.69297
1,Procervulus_praelucidus[&height=18.275541091306565,height_95%_HPD={18.0
00181443989277,19.003287814557552},height_median=18.1419506594538 7,height
_range={18.000181443989277,19.992634758353233},length=0.22269057201851872
,length_95%_HPD={0.0,0.8683903813362122},length_median=0.0974753499031066
9,length_range={0.0,2.227649211883545},rate=0.010939787832160082,rate_95%
_HPD={0.007098218847243259,0.015448430274147575},rate_median=0.0108389790
93306337,rate_range={0.004319274286441181,0.019639680265498508},!rotate=t
rue]:0.470457)[&height=18.75635957725107,height_95%_HPD={18.0202109813690
2,19.938027560710907},height_median=18.612408006563783,height_range={18.0
0908997654915,20.707210399210453},length=3.075445094572759,length_95%_HPD
={1.425382137298584,4.5902323722839355},length_median=3.1418871879577637,
length_range={0.27071523666381836,5.230633735656738},posterior=0.75233022
63648469,rate=0.01202091531072598,rate_95%_HPD={0.008068123972586603,0.01
7561195052050303},rate_median=0.01173754467917139,rate_range={0.005895491
859971152,0.021447819171794776},!rotate=true]:3.234418)[&height=21.780612
006591422,height_95%_HPD={20.403723992407322,22.954346910119057},height_m
edian=21.846826143562794,height_range={19.339435428380966,23.471204370260
24},length=0.5518011481503642,length_95%_HPD={0.0017773122526705265,1.489
2374277114868},length_median=0.42647162079811096,length_range={0.00177731
22526705265,2.704744577407837},posterior=1.0,rate=0.011288250474081463,ra
te_95%_HPD={0.007007388879448335,0.015900901133844823},rate_median=0.0111
41325549007451,rate_range={0.005834558794667721 5,0.02016779120245831},!ro
tate=true]:0.533751,Lagomeryx_parvulus[&height=18.80378862981763,height_9
5%_HPD={18.003005146980286,19.84419621527195},height_median=18.7138812467
45586,height_range={18.000445291399956,19.999947622418404},length=3.52862
4524924155,length_95%_HPD={2.040778636932373,4.867641925811768},length_me
dian=3.5391945838928223,length_range={1.2251975536346436,5.56208086013793
95},rate=0.012070774303053243,rate_95%_HPD={0.007125301405662899,0.016882
292125930645},rate_median=0.011928491622043182,rate_range={0.006339251082
732072,0.024199032422356586},!rotate=true]:3.666696)[&height=22.332413154
741786,height_95%_HPD={21.12979121506214,23.26729776710272},height_median
=22.380576968193054,height_range={20.515851497650146,23.83285953104496},l
ength=0.3269060050736004,length_95%_HPD={3.9948977064341307E-
4,0.9492372274398804},length_median=0.23709100484848022,length_range={3.9
948977064341307E-
4,1.9388442039489746},posterior=1.0,rate=0.010932553119220789,rate_95%_HP
D={0.006483909677687053 5,0.014993895215376385},rate_median=0.010755141351
747582,rate_range={0.005249509868188782 4,0.020607268318515667},!rotate=tr
ue]:0.30661,(Moschus_moschiferus[&height=5.974614132101463E-
7,height_95%_HPD={0.0,1.5497207641601562E-
6},height_median=5.122274160385132E-

7,height_range={0.0,2.0265579223632812E-
6},length=20.434855205876215,length_95%_HPD={18.990917205810547,21.960294
723510742},length_median=20.476552963256836,length_range={18.031776428222
656,22.55040740966797},rate=0.008514075723702828,rate_95%_HPD={0.00748765
3773754676,0.009677740149720507},rate_median=0.008500145017318585,rate_ra
nge={0.007014154117683652,0.010576186211359884},!rotate=true]:20.476553,O
vis_aries_NC001941[&height=5.974614132101463E-
7,height_95%_HPD={0.0,1.5497207641601562E-
6},height_median=5.122274160385132E-
7,height_range={0.0,2.0265579223632812E-
6},length=20.434855205876215,length_95%_HPD={18.990917205810547,21.960294
723510742},length_median=20.476552963256836,length_range={18.031776428222
656,22.55040740966797},rate=0.011899624219291869,rate_95%_HPD={0.01045709
2744126479,0.013242454991199094},rate_median=0.011925543775304984,rate_ra
nge={0.009904381678140485,0.013971787702467212},!rotate=true]:20.476553)[
&height=20.434855803337626,height_95%_HPD={18.990918286144733,21.96029584
109783},height_median=20.476553082466125,height_range={18.03177759051323,
22.550408005714417},length=2.2244633564777603,length_95%_HPD={1.110813617
7062988,3.5336647033691406},length_median=2.1719441413879395,length_range
={0.7582273483276367,4.736240386962891},posterior=1.0,rate=0.010535327222
395061,rate_95%_HPD={0.007105249177090734б,0.014607734958291058},rate_med
ian=0.010374726833297982,rate_range={0.005640429147009795,0.0187844987246
87508},!rotate=true]:2.210634)[&height=22.65931915981539,height_95%_HPD={
21.516587018966675,23.46516693686135},height_median=22.68718671798706,hei
ght_range={20.84463855624199,23.960269331932068},length=1.75681325440083,
length_95%_HPD={1.107163667678833,2.549954414367676},length_median=1.7181
379795074463,length_range={0.7221366167068481,2.9180281162261963},posteri
or=1.0,rate=0.013535371738651438,rate_95%_HPD={0.008769573070020135,0.017
81288924021212б},rate_median=0.01332012565784661l,rate_range={0.008103708
295555267,0.021559499229637333},!rotate=true]:1.790208,Giraffa_camelopard
alis_angolensis_NC012100[&height=5.933343272869501E-
7,height_95%_HPD={0.0,1.4957040548324585E-
6},height_median=5.364418029785156E-
7,height_range={0.0,1.8104910850524902E-
6},length=24.41613182088189,length_95%_HPD={23.68043327331543,24.95532417
2973633},length_median=24.477394104003906,length_range={22.63677406311035
,24.979618072509766},rate=0.00917091485497491,rate_95%_HPD={0.00821306802
6911704,0.010287146658995454},rate_median=0.009145673316608746,rate_range
={0.007786141167260808,0.011569558408238076},!rotate=true]:24.477394)[&he
ight=24.416132414216218,height_95%_HPD={23.680433750152588,24.95532417297
3633},height_median=24.47739487886429,height_range={22.636775225400925,24
.97961882315576},length=0.27756866986755424,length_95%_HPD={3.08343122014
77587E-
4,0.8154094219207764},length_median=0.20460245013237,length_range={3.0834
312201477587E-
4,1.6219549179077148},posterior=1.0,rate=0.010773442062431879,rate_95%_HP
D={0.006713074370436776,0.014940289912530769},rate_median=0.0106887844690
19469,rate_range={0.005249509868188782 4,0.018891785270392918},!rotate=tru
e]:0.311268,Antilocapra_americana[&height=5.857491947601385E-
7,height_95%_HPD={0.0,1.4975666999816895E-
6},height_median=5.140900611877441E-
7,height_range={0.0,1.8775463104248047E-
6},length=24.69370049833458,length_95%_HPD={24.08601951599121,24.99977684
020996},length_median=24.78866195678711,length_range={23.095870971679688,
24.99977684020996},rate=0.0105339416208986,rate_95%_HPD={0.00947514997860
2129,0.011788413763856 88},rate_median=0.010554371705960922,rate_range={0.
0088299231464464,0.012183241262501018},!rotate=true]:24.788662)[&height=2
4.693701084083774,height_95%_HPD={24.086019545793533,24.99977770447731},h
eight_median=24.788662552833557,height_range={23.095871955156326,24.99977
770447731},length=0.0,posterior=1.0,rate=1.0,!rotate=true];

```
end;

begin figtree;
	set appearance.backgroundColorAttribute="Default";
	set appearance.backgroundColour=#ffffff;
	set appearance.branchColorAttribute="User selection";
	set appearance.branchColorGradient=false;
	set appearance.branchLineWidth=2.0;
	set appearance.branchMinLineWidth=0.0;
	set appearance.branchWidthAttribute="Fixed";
	set appearance.foregroundColour=#000000;
	set appearance.hilightingGradient=false;
	set appearance.selectionColour=#2d3680;
	set branchLabels.colorAttribute="User selection";
	set branchLabels.displayAttribute="posterior";
	set branchLabels.fontName="sansserif";
	set branchLabels.fontSize=9;
	set branchLabels.fontStyle=0;
	set branchLabels.isShown=true;
	set branchLabels.significantDigits=2;
	set layout.expansion=0;
	set layout.layoutType="RECTILINEAR";
	set layout.zoom=0;
	set legend.attribute="height";
	set legend.fontSize=10.0;
	set legend.isShown=false;
	set legend.significantDigits=4;
	set nodeBars.barWidth=4.0;
	set nodeBars.displayAttribute="height_95%_HPD";
	set nodeBars.isShown=true;
	set nodeLabels.colorAttribute="User selection";
	set nodeLabels.displayAttribute="height_95%_HPD";
	set nodeLabels.fontName="sansserif";
	set nodeLabels.fontSize=14;
	set nodeLabels.fontStyle=0;
	set nodeLabels.isShown=false;
	set nodeLabels.significantDigits=2;
	set nodeShape.colourAttribute="User selection";
	set nodeShape.isShown=false;
	set nodeShape.minSize=10.0;
	set nodeShape.scaleType=Width;
	set nodeShape.shapeType=Circle;
	set nodeShape.size=4.0;
	set nodeShape.sizeAttribute="Fixed";
	set polarLayout.alignTipLabels=false;
	set polarLayout.angularRange=0;
	set polarLayout.rootAngle=0;
	set polarLayout.rootLength=100;
	set polarLayout.showRoot=true;
	set radialLayout.spread=0.0;
	set rectilinearLayout.alignTipLabels=false;
	set rectilinearLayout.curvature=0;
	set rectilinearLayout.rootLength=100;
	set scale.offsetAge=0.0;
	set scale.rootAge=1.0;
	set scale.scaleFactor=1.0;
	set scale.scaleRoot=false;
	set scaleAxis.automaticScale=true;
	set scaleAxis.fontSize=8.0;
	set scaleAxis.isShown=true;
```

```
	set scaleAxis.lineWidth=1.0;
	set scaleAxis.majorTicks=5.0;
	set scaleAxis.origin=0.0;
	set scaleAxis.reverseAxis=true;
	set scaleAxis.showGrid=false;
	set scaleBar.automaticScale=true;
	set scaleBar.fontSize=10.0;
	set scaleBar.isShown=false;
	set scaleBar.lineWidth=1.0;
	set scaleBar.scaleRange=3.0;
	set tipLabels.colorAttribute="User selection";
	set tipLabels.displayAttribute="Names";
	set tipLabels.fontName="Helvetica";
	set tipLabels.fontSize=10;
	set tipLabels.fontStyle=0;
	set tipLabels.isShown=true;
	set tipLabels.significantDigits=4;
	set trees.order=false;
	set trees.orderType="increasing";
	set trees.rooting=false;
	set trees.rootingType="User Selection";
	set trees.transform=false;
	set trees.transformType="cladogram";
end;
```
