# Supplementary material for: Bony labyrinth morphology clarifies the origin and evolution of deer
Source: Sci Rep. 2017 Oct 13;7:13176. doi: 10.1038/s41598-017-12848-9 (PMC5640792; doi:10.1038/s41598-017-12848-9)
Supplement: Supplementary file 2 — Dataset 1-5 [file 41598_2017_12848_MOESM2_ESM.zip › dataset4.pdf]

```
#NEXUS
begin taxa;
	dimensions ntax=53;
	taxlabels
	Alces_alces
	Antilocapra_americana
	Axis_axis
	Axis_porcinus
	Blastocerus_dichotomus
	Capreolus_capreolus
	Cervus_elaphus_NC007704
	Cervus_nippon_centralis_NC006993
	Cervus_ruscinensis
	Croizetoceros_pyrenaicus
	Croizetoceros_ramosus
	Dama_dama_dama
	Dicrocerus_elegans
	Elaphodus_cephalophus_N008749
	Elaphurus_davidianus
	Eucladoceros_ctenoides
	Euprox_furcatus
	Eostyloceros_hezhengensis
	Giraffa_camelopardalis_angolensis_NC012100
	Hereroprox_larteti
	Hippocamelus_antisensis
	Hydropotes_inermis
	Lagomeryx_parvulus
	Mazama_americana_1
	Mazama_gouazoubira
	Mazama_nemorivaga_1
	Mazama_rufina
	Megaloceros_giganteus_AM182644
	Metacervocerus_philisi_1
	Metacervocerus_philisi_2
	Moschus_moschiferus
	Muntiacus_crinifrons_NC004577
	Muntiacus_muntjak_NC_004563
	Muntiacus_reevesi_NC008491
	Muntiacus_vuquangensis
	Odocoileus_hemionus
	Odocoileus_lucasi
	Odocoileus_cf._virginianus
	Odocoileus_virginianus_1
	Ovis_aries_NC001941
	Ozotoceros_bezoarcticus
	Procervulus_dichotomus
	Procervulus_praelucidus
	Przewalskium_albirostris
	Dama_eurygonos
	Pudu_mephistophiles
	Pudu_puda
	Rangifer_tarandus_NC007703
	Rucervus_duvauceli
	Rucervus_eldi
	Rusa_alfredi
	Rusa_timorensis
	Rusa_unicolor_NC008414
;
end;
```

begin trees;
tree TREE1 = [&R] ((((((((Alces_alces[&height=5.577540710430776E-
7,height_95%_HPD={0.0,1.2032687664031982E-
6},height_median=5.364418029785156E-
7,height_range={0.0,1.7415732145309448E-
6},length=13.82966064613129,length_95%_HPD={11.985352516174316,16.0688743
5913086},length_median=13.840312004089355,length_range={3.271181106567383
,17.14572525024414},rate=0.008480892317930162,rate_95%_HPD={0.00704848589
1891054,0.010043254544287443},rate_median=0.008450419988388325,rate_range
={0.006466566708753162,0.011121744544032678},!rotate=true]:13.849115,(Cap
reolus_capreolus[&height=5.557202907203487E-
7,height_95%_HPD={0.0,1.2060627341270447E-
6},height_median=5.364418029785156E-
7,height_range={0.0,1.6987323760986328E-
6},length=9.483540075279267,length_95%_HPD={7.756155014038086,11.30814170
8374023},length_median=9.48104190826416,length_range={6.521266937255859,1
2.708541870117188},rate=0.01098334542772972,rate_95%_HPD={0.0089674614453
06722,0.01359636510298353},rate_median=0.010787204340432757,rate_range={0
.007959992428642012,0.01623309811135909},!rotate=true]:9.481042,Hydropote
s_inermis[&height=5.557202907203487E-
7,height_95%_HPD={0.0,1.2060627341270447E-
6},height_median=5.364418029785156E-
7,height_range={0.0,1.6987323760986328E-
6},length=9.483540075279267,length_95%_HPD={7.756155014038086,11.30814170
8374023},length_median=9.48104190826416,length_range={6.521266937255859,1
2.708541870117188},rate=0.011288682317560401,rate_95%_HPD={0.008925971357
46915,0.013613715938488374},rate_median=0.01115358959092327,rate_range={0
.00813066422522165,0.016331374395185227},!rotate=true]:9.481042)[&height=
9.483540630999556,height_95%_HPD={7.756155848503113,11.308142006397247},h
eight_median=9.481042919796892,height_range={6.521267414093018,12.7085433
30430984},length=4.399703542338548,length_95%_HPD={3.1364941596984863,6.1
13718032836914},length_median=4.321672439575195,length_range={1.365278959
274292,7.2953572273254395},posterior=1.0,rate=0.010639962678431758,rate_9
5%_HPD={0.007440381825095351,0.013874123563350334},rate_median=0.01044688
4616342857,rate_range={0.006467082299907305,0.017079651330796922},!rotate
=true]:4.368072)[&height=13.897126651403259,height_95%_HPD={11.9853534400
4631,15.885249137878418},height_median=13.849115401506424,height_range={1
0.687738493084908,17.145725458860397},length=0.7481397730291952,length_95
%_HPD={0.23099423944950104,1.2961093187332153},length_median=0.7252182960
510254,length_range={0.0988176017999649,2.0117228031158447},posterior=0.9
707057256990679,rate=0.009828224954935785,rate_95%_HPD={0.006507184648274
533,0.01396591678094083},rate_median=0.009615246994224703,rate_range={0.0
04789531529264687,0.020523700707092314},!rotate=true]:0.696008,(((((Blast
ocerus_dichotomus[&height=5.44842046213655E-
7,height_95%_HPD={0.0,1.0728836059570312E-
6},height_median=5.066394805908203E-
7,height_range={0.0,1.5031546354293823E-
6},length=6.144376236811459,length_95%_HPD={5.185646057128906,7.466375827
789307},length_median=6.13586950302124,length_range={0.4089604616165161,8
.281837463378906},rate=0.011807479641023121,rate_95%_HPD={0.0099153823890
71921,0.014212556362480316},rate_median=0.011806028603703703,rate_range={
0.00821696000680479,0.014824634007981372},!rotate=true]:6.161222,((Hippoc
amelus_antisensis[&height=5.410005731139823E-
7,height_95%_HPD={8.940696716308594E-8,1.1324882507324219E-
6},height_median=5.066394805908203E-
7,height_range={0.0,1.5031546354293823E-
6},length=3.79382912598342,length_95%_HPD={2.828721761703491,5.0333027839
660645},length_median=3.833460807800293,length_range={0.3411862254142761,
5.3029351234436035},rate=0.010954850191611484,rate_95%_HPD={0.00851926323
4832675,0.0137175093562691},rate_median=0.010835749874277205,rate_range={

0.007673567263695495,0.015759456615454012},!rotate=true]:3.865041,Mazama_
gouazoubira[&height=5.402093829640427E-
7,height_95%_HPD={8.754432201385498E-8,1.1324882507324219E-
6},height_median=5.066394805908203E-
7,height_range={0.0,1.5031546354293823E-
6},length=3.8300786964997116,length_95%_HPD={2.940380096435547,4.90776348
1140137},length_median=3.838912010192871,length_range={0.0259243529289960
86,5.3029351234436035},rate=0.010970199284415954,rate_95%_HPD={0.00861006
6648822436,0.0136891005395903},rate_median=0.010926533378256461,rate_rang
e={0.007053533693655889,0.016465761924347 17},!rotate=true]:3.865041)[&hei
ght=3.8983284405722154,height_95%_HPD={3.1423037350177765,4.9077638238668
44},height_median=3.8650414552539587,height_range={2.672665148973465,5.30
29357402119786},length=1.884059192799152,length_95%_HPD={1.22499763965606
7,2.6177515983581543},length_median=1.8491728901863098,length_range={8.33
5892925970256E-
4,3.41335391998291},posterior=0.9027962716378163,rate=0.01077903813963674
,rate_95%_HPD={0.0072589998426126 7,0.013991284405354756},rate_median=0.01
0659848532819177,rate_range={0.005948306177985992,0.022417551035589064},!
rotate=true]:1.876537,Ozotoceros_bezoarcticus[&height=5.475417655810823E-
7,height_95%_HPD={0.0,1.1175870895385742E-
6},height_median=5.066394805908203E-
7,height_range={0.0,1.5348196029663086E-
6},length=5.714927122492924,length_95%_HPD={4.762259006500244,7.110921859
741211},length_median=5.722018718719482,length_range={0.15707972645759583
,7.756762504577637},rate=0.009713921767494832,rate_95%_HPD={0.00765917292
6731748,0.011474301679737132},rate_median=0.009762019234889386,rate_range
={0.006766806246105164,0.01377469490209627},!rotate=true]:5.741578)[&heig
ht=5.801869990100125,height_95%_HPD={4.922914549708366,7.062218448380008}
,height_median=5.741578862071037,height_range={4.588151901960373,7.756763
264536858},length=0.4302816538757197,length_95%_HPD={0.1169787347316742,0
.770111083984375},length_median=0.4138015806674957,length_range={0.029735
030606389046,1.2170523405075073},posterior=0.8561917443408789,rate=0.0100
53461306427576,rate_95%_HPD={0.006240442887243693,0.013905919762801673},r
ate_median=0.009880889054998104,rate_range={0.004474373691818895,0.018579
56097586794},!rotate=true]:0.419644)[&height=6.235599276809026,height_95%
_HPD={5.252673864364624,7.409113436937332},height_median=6.16122283041477
2,height_range={4.979055505245924,8.281838163733482},length=0.43106131258
014685,length_95%_HPD={0.1788252294063568,0.7695376873016357},length_medi
an=0.40377962589263916,length_range={0.002883315086364746,1.0618505477905
273},posterior=0.8308921438082557,rate=0.010238075430198184,rate_95%_HPD=
{0.006953906334938987,0.014509721441724424},rate_median=0.010115781632400
733,rate_range={0.004719784172624163 5,0.018746878736951997},!rotate=true]
:0.449018,Mazama_nemorivaga_1[&height=5.467766177401532E-
7,height_95%_HPD={0.0,1.0952353477478027E-
6},height_median=5.066394805908203E-
7,height_range={0.0,1.5518744476139545E-
6},length=6.481132368035069,length_95%_HPD={5.523904800415039,8.159452438
354492},length_median=6.561125755310059,length_range={0.37968555092811584
,8.632019996643066},rate=0.011994083052181748,rate_95%_HPD={0.00971430718
0861179,0.014207139400050703},rate_median=0.011960145888490127,rate_range
={0.007769861689701355,0.015875776031446912},!rotate=true]:6.61024)[&heig
ht=6.6715542198010604,height_95%_HPD={5.572344928979874,7.849050834774971
},height_median=6.610240697860718,height_range={5.25576550886035,8.632019
996643066},length=0.4887164714410133,length_95%_HPD={0.17476040124893188,
0.8460468053817749},length_median=0.47446851432323456,length_range={0.115
56248366832733,1.331616759300232},posterior=0.7749667110519307,rate=0.010
287752329424457,rate_95%_HPD={0.007040612014831435,0.014595128026042885},
rate_median=0.010135339066152438,rate_range={0.004847303234377308,0.01680
526998262613 4},!rotate=true]:0.485168,Pudu_puda[&height=5.433787164692525
E-7,height_95%_HPD={0.0,1.0691583156585693E-

6},height_median=5.346955731511116E-
7,height_range={0.0,1.519918441772461E-
6},length=7.033065865582696,length_95%_HPD={5.832897186279297,8.476876258
850098},length_median=7.064578056335449,length_range={1.6106239557266235,
9.122953414916992},rate=0.009704875911564333,rate_95%_HPD={0.007792385427
407426,0.011509157496761487},rate_median=0.009667708023898498,rate_range=
{0.00713710802478757,0.013467885093622385},!rotate=true]:7.095408)[&heigh
t=7.159436297860818,height_95%_HPD={6.095402857288718,8.457834422588348},
height_median=7.09540830552578,height_range={5.637380074709654,9.12295371
2940216},length=2.793667469681057,length_95%_HPD={1.9882147312164307,3.70
34425735473633},length_median=2.770855188369751,length_range={0.094300605
35669327,4.007585525512695},posterior=0.7430093209054593,rate=0.012665977
600998935,rate_95%_HPD={0.009405624038154363,0.016489394960700017},rate_m
edian=0.012469107824291361,rate_range={0.008575192805152648,0.01962211379
429648},!rotate=true]:2.825433,(((Mazama_americana_1[&height=5.5053105062
25079E-7,height_95%_HPD={0.0,1.0728836059570312E-
6},height_median=5.047768354415894E-
7,height_range={0.0,1.430511474609375E-
6},length=3.563500671745458,length_95%_HPD={1.758615493774414,5.138854503
631592},length_median=3.7393412590026855,length_range={0.8246783018112183
,5.506061553955078},rate=0.01104414591222292,rate_95%_HPD={0.008255291232
868686,0.014275306318731584},rate_median=0.010896813704882628,rate_range=
{0.0074373564520537234,0.017560727136191622},!rotate=true]:2.650048,Odoco
ileus_lucasi[&height=5.476663966313471E-
7,height_95%_HPD={0.0,1.1026859283447266E-
6},height_median=5.066394805908203E-
7,height_range={0.0,1.430511474609375E-
6},length=3.0223991085989734,length_95%_HPD={0.025924352928996086,6.30510
5209350586},length_median=2.828721761703491,length_range={0.0259243529289
96086,11.807319641113281},rate=0.010017152886787046,rate_95%_HPD={0.00660
4365966225355,0.013696105210811316},rate_median=0.009908820724146145,rate
_range={0.005001618449468156,0.01947315033439314},!rotate=true]:2.650048)
[&height=2.6896029233094385,height_95%_HPD={1.1646322049200535,4.16305613
5177612},height_median=2.6500483751296997,height_range={0.824678719043731
7,4.887968964874744},length=1.3845095245991474,length_95%_HPD={0.00471415
0447398424,2.7892096042633057},length_median=1.3195997476577759,length_ra
nge={0.004714150447398424,3.5273420810699463},posterior=0.380825565912117
2,rate=0.010389443326114823,rate_95%_HPD={0.006718688433701923,0.01374094
2073279678},rate_median=0.010219961370417387,rate_range={0.00578821455649
2813,0.020493114542000507},!rotate=true,!color=#ff0000]:1.431672,((Odocoi
leus_hemionus[&height=5.443221301159086E-
7,height_95%_HPD={1.043081283569336E-7,1.1026859283447266E-
6},height_median=5.00120222568512E-
7,height_range={0.0,1.430511474609375E-
6},length=1.072323114165854,length_95%_HPD={0.030262188985943794,1.654618
263244629},length_median=1.17887282371521,length_range={0.001930134254507
7205,2.048544406890869},rate=0.00887718583866685,rate_95%_HPD={0.00559349
8968671994,0.01185446228766989},rate_median=0.008721277601296222,rate_ran
ge={0.0047197841726241635,0.01705447839773158},!rotate=true]:1.279498,Odo
coileus_virginianus_1[&height=5.454188523554899E-
7,height_95%_HPD={1.1920928955078125E-7,1.1026859283447266E-
6},height_median=4.991888999938965E-
7,height_range={0.0,1.430511474609375E-
6},length=1.1821686767746065,length_95%_HPD={0.38518527150154114,1.681123
3758926392},length_median=1.2172393798828125,length_range={0.090550497174
263,2.0187039375305176},rate=0.011324851238169882,rate_95%_HPD={0.0077187
95145985666,0.014862264053135538},rate_median=0.011216317004640134,rate_r
ange={0.005831411634147212,0.018509537691316538},!rotate=true]:1.279498)[
&height=1.2975894147613352,height_95%_HPD={0.9067143052816391,1.681124150
7530212},height_median=1.2794986963272095,height_range={0.763537943363189

7,2.004227936267853},length=1.4240659170588184,length_95%_HPD={0.01326203
066855669,3.2826502323150635},length_median=1.174748420715332,length_rang
e={4.2693119030445814E-
4,3.702378034591675},posterior=0.4194407456724368,rate=0.009394697466062
37,rate_95%_HPD={0.006199691506665298,0.012644932686205222},rate_median=0.
009376787615972281,rate_range={0.005302112765334191,0.015226277023041307}
,!rotate=true,!color=#ff0000]:0.144556,Odocoileus_cf._virginianus[&height
=5.470799796373773E-7,height_95%_HPD={5.21540641784668E-
8,1.1026859283447266E-6},height_median=4.991888999938965E-
7,height_range={0.0,1.4491379261016846E-
6},length=1.633009755839941,length_95%_HPD={0.001930134254507720 5,4.87171
745300293},length_median=1.115286946296692,length_range={0.00193013425450
77205,14.814472198486328},rate=0.009923452263695133,rate_95%_HPD={0.00640
2142719436363,0.01383168326280276},rate_median=0.009744101815476844,rate_
range={0.004847303234377308,0.017426816743941528},!rotate=true]:1.424055)
[&height=1.6087334095941004,height_95%_HPD={0.886891208589077,3.038240551
9485474},height_median=1.4240551367402077,height_range={0.788579851388931
3,4.119017779827118},length=2.242831733901343,length_95%_HPD={0.486494481
5635681,3.579911470413208},length_median=2.4019768238067627,length_range=
{0.0010336097329854965,4.678702354431152},posterior=0.8402130492676432,ra
te=0.009328575989661376,rate_95%_HPD={0.006507184648274533,0.012922997658
410906},rate_median=0.009146394481499 49,rate_range={0.005050954732685793,
0.01846238985843989},!rotate=true]:2.657665)[&height=4.191973987759481,he
ight_95%_HPD={3.0704915039241314,5.508062034845352},height_median=4.08172
0024347305,height_range={2.8027843236923218,8.337641716003418},length=3.5
63816167045665,length_95%_HPD={2.0750460624694824,4.7722697257995605},len
gth_median=3.6155145168304443,length_range={0.1310727596282959,5.53346586
227417},posterior=0.6191744340878829,rate=0.009161129585145832,rate_95%_H
PD={0.006910832208828456,0.01209894346550339},rate_median=0.0090095175721
3525,rate_range={0.005902530268882146,0.013928334460384117},!rotate=true]
:3.695729,(Mazama_rufina[&height=5.412060438318007E-
7,height_95%_HPD={0.0,1.1324882507324219E-
6},height_median=4.991888999938965E-
7,height_range={0.0,1.4901161193847656E-
6},length=0.9355732102924276,length_95%_HPD={0.6486279368400574,1.2729293
10798645},length_median=0.9213104248046875,length_range={0.16345521807670
593,1.5007199048995972},rate=0.009999643636892918,rate_95%_HPD={0.0068435
12287187579,0.013306486538858723},rate_median=0.009903001973992244,rate_r
ange={0.005065153351802831,0.015759456615454012},!rotate=true]:0.922699,P
udu_mephistophiles[&height=5.411663603133084E-
7,height_95%_HPD={0.0,1.1324882507324219E-
6},height_median=4.991888999938965E-
7,height_range={0.0,1.4901161193847656E-
6},length=0.9335589080096561,length_95%_HPD={0.6646963357925415,1.2849451
303482056},length_median=0.9205701351165771,length_range={0.0617456287145
6146,1.5007199048995972},rate=0.009408504979642771,rate_95%_HPD={0.006178
663818979883,0.012524378722817175},rate_median=0.009488446684450902,rate_
range={0.004685554937754828,0.014898654417062202},!rotate=true]:0.922699)
[&height=0.9425135597076388,height_95%_HPD={0.6696320176124573,1.26878488
06381226},height_median=0.9226990342140198,height_range={0.59953552484512
33,1.5007206201553345},length=6.438626299713987,length_95%_HPD={2.6409318
447113037,8.720088005065918},length_median=6.738439559936523,length_range
={0.0560602992773056,9.15097427368164},posterior=0.96271637816245,rate=0.
01063579487638148,rate_95%_HPD={0.008077235769816 52,0.013028851411036187
},rate_median=0.010565414264929365,rate_range={0.007094805133848455,0.017
05447839773158},!rotate=true]:6.85475)[&height=7.764701386775265,height_9
5%_HPD={6.596453458070755,9.176267087459564},height_median=7.777448534965
515,height_range={5.176331162452698,9.72471871972084},length=2.1923868379
86028,length_95%_HPD={1.341854214668274,3.1657278537750244},length_median
=2.1256027221679688,length_range={1.0836124420166016,4.286848068237305},p

osterior=0.7123834886817576,rate=0.009472539809008262,rate_95%_HPD={0.006
29422594231101,0.012972571213662935},rate_median=0.009295712226099002,ra
te_range={0.004746122550913741,0.016054426173595947},!rotate=true]:2.1433
92)[&height=9.943819173797811,height_95%_HPD={8.386760920286179,11.369870
364665985},height_median=9.920840868726373,height_range={7.93692719936370
85,13.074255257844925},length=1.2111415783486639,length_95%_HPD={0.650935
709476471,1.8669682741165161},length_median=1.1930173635482788,length_ran
ge={0.04450744763016701,2.672390460968017 6},posterior=0.8948069241011984,
rate=0.010824685629977186,rate_95%_HPD={0.007829567276761911,0.0151538654
51280862},rate_median=0.0106345388100545,rate_range={0.005370703483120626
,0.018977202951609637},!rotate=true]:1.206065,Rangifer_tarandus_NC007703[
&height=5.416053592366291E-7,height_95%_HPD={0.0,1.1644442565739155E-
6},height_median=5.066394805908203E-
7,height_range={0.0,1.6540288925170898E-
6},length=9.725059892540765,length_95%_HPD={3.411525011062622,12.62761688
2324219},length_median=10.796330451965332,length_range={0.932463467121124
3,14.217392921447754},rate=0.00916906780557118,rate_95%_HPD={0.0074033359
06642351,0.011204088762018154},rate_median=0.009150966360006708,rate_rang
e={0.004692155003066161,0.013576895204874866},!rotate=true]:11.126905)[&h
eight=11.194038219980445,height_95%_HPD={9.59487470984459,12.933081895112
991},height_median=11.126905873417854,height_range={8.946266621351242,14.
814472615718842},length=3.3464866476652793,length_95%_HPD={2.269361734390
259,4.420532703399658},length_median=3.3445186614990234,length_range={0.6
517525315284729,5.015898704528809},posterior=0.7163781624500666,rate=0.01
2390487362503252,rate_95%_HPD={0.009101603970665102,0.016236812424135928}
,rate_median=0.012154446256344785,rate_range={0.005426989128464773,0.0188
7662880722147},!rotate=true]:3.418218)[&height=14.567879305753364,height_
95%_HPD={12.700046002864838,16.617788791656494},height_median=14.54512375
5931854,height_range={11.389385025948286,17.700868278741837},length=1.354
2938688053534,length_95%_HPD={0.6142029762268066,2.210564613342285},lengt
h_median=1.3323888778686523,length_range={0.1379552185535431,3.0046837329
8645},posterior=0.6924101198402131,rate=0.010699000852535095,rate_95%_HPD
={0.006861669737077561,0.014485979568822761},rate_median=0.01049841457557
6702,rate_range={0.005938256041385749,0.01772105431542115},!rotate=true]:
0.371169,(Croizetoceros_pyrenaicus[&height=5.913437465272648,height_95%_H
PD={5.300205498933792,6.79284299723804},height_median=5.832580462098122,h
eight_range={5.300205498933792,6.964351937174797},length=0.89271116945610
45,length_95%_HPD={0.0,3.1668386459350586},length_median=0.50693804025650
02,length_range={0.0,9.259564399719238},rate=0.01011737523202106,rate_95%
_HPD={0.006111687507442587,0.013664738986807047},rate_median=0.0099830227
54300755,rate_range={0.00450624432479737,0.01714758104265916 6},!rotate=tr
ue]:0.866264,Croizetoceros_ramosus[&height=3.803167381805085,height_95%_H
PD={3.4319418370723724,4.188684940338135},height_median=3.809131205081939
7,height_range={3.401016429066658,4.199305638670921},length=2.67664687325
07427,length_95%_HPD={0.0,5.408016204833984},length_median=2.532274007797
241,length_range={0.0,10.98276138305664},rate=0.009955308276489994,rate_9
5%_HPD={0.006313215170939472,0.013593745719599464},rate_median=0.00984531
8575786783,rate_range={0.005129805702974475,0.017079651330796922},!rotate
=true]:2.889714)[&height=7.048575895579708,height_95%_HPD={5.414039909839
63,9.832863342016935},height_median=6.698844790458679,height_range={5.314
659625291824,14.828569412231445},length=7.603262242753414,length_95%_HPD=
{2.1786580085754395,12.421000480651855},length_median=7.9242119789123535,
length_range={0.33194810152053833,13.887935638427734},posterior=0.6564580
559254327,rate=0.010005956286079573,rate_95%_HPD={0.006219142003180 84,0.
013925772187524188},rate_median=0.009832953128433845,rate_range={0.004258
949028824671,0.018094283403453322},!rotate=true]:8.217448)[&height=14.986
648428311543,height_95%_HPD={13.232730274670757,17.572569355368614},heigh
t_median=14.916293010115623,height_range={12.040356993675232,18.301524661
48138},length=1.1499208400728402,length_95%_HPD={0.07757480442523956,2.07
5737476348877},length_median=1.2223025560379028,length_range={0.007874594

070017338,2.5557026863098145},posterior=0.31424766977363516,rate=0.010493
886086550228,rate_95%_HPD={0.006865494116813895,0.013597959320502},rate_m
edian=0.010458237994167612,rate_range={0.006049610200321226,0.01823429216
2025767},!rotate=true,!color=#ff0000]:1.073657,((((Axis_axis[&height=5.40
0239090192146E-7,height_95%_HPD={0.0,1.1064112186431885E-
6},height_median=4.917383193969727E-
7,height_range={0.0,1.4603137969970703E-
6},length=4.014037169724425,length_95%_HPD={3.1264379024505615,5.23322296
1425781},length_median=3.9114787578582764,length_range={2.884299993515014
6,6.156908988952637},rate=0.009673129283712495,rate_95%_HPD={0.0066638392
436147035,0.01233467418318399},rate_median=0.009631084316789973,rate_rang
e={0.0056391918504903734,0.013775961485700233},!rotate=true]:3.886429,Axi
s_porcinus[&height=5.383187578339998E-
7,height_95%_HPD={0.0,1.1026859283447266E-
6},height_median=4.917383193969727E-
7,height_range={0.0,1.564621925354004E-
6},length=4.011690314695457,length_95%_HPD={3.026088237762451,5.035771846
77124},length_median=3.920743703842163,length_range={2.8842999935150146,6
.158487319946289},rate=0.010424837078797157,rate_95%_HPD={0.0071301485823
00314,0.013120666453105733},rate_median=0.010427661514182852,rate_range={
0.0059927769429661305,0.015411620068237224},!rotate=true]:3.886429)[&heig
ht=3.9782478789872275,height_95%_HPD={3.0460987389087677,4.99097503721714
},height_median=3.8864290826022625,height_range={2.884300246834755,6.1569
09465789795},length=1.2805882132063742,length_95%_HPD={0.0056549059227108
955,2.1543831825256348},length_median=1.397998571395874,length_range={0.0
056549059227108955,3.8200266361236572},posterior=0.7416777629826897,rate=
0.010209561587442871,rate_95%_HPD={0.006266480368697505,0.013250507820903
41},rate_median=0.010112213066580136,rate_range={0.004994414664545471,0.0
1821167032886015},!rotate=true]:2.166567,((Rucervus_duvauceli[&height=5.3
15930215045364E-7,height_95%_HPD={0.0,1.1175870895385742E-
6},height_median=4.991888999938965E-
7,height_range={0.0,1.4938414096832275E-
6},length=5.100132909500488,length_95%_HPD={3.400620937347412,6.781627655
029297},length_median=5.22664737701416,length_range={3.400620937347412,7.
776570796966553},rate=0.008326731321449982,rate_95%_HPD={0.00620015963492
0041,0.010730434651080402},rate_median=0.008301916587120694,rate_range={0
.004838873425096357,0.012272365949042177},!rotate=true]:4.185547,Metacerv
ocerus_philisi_1[&height=3.7417241315735796,height_95%_HPD={3.40006578713
65547,4.130782466381788},height_median=3.72116606682539,height_range={3.4
000657871365547,4.195499092340469},length=0.9412220580803761,length_95%_H
PD={0.0,3.0118677616119385},length_median=0.5997194051742554,length_range
={0.0,5.6778435707092285},rate=0.010045685995755694,rate_95%_HPD={0.00634
7022819231006,0.0135890270387463},rate_median=0.00983871655242016,rate_ra
nge={0.005150943588962271,0.017350127393675436},!rotate=true]:0.464382)[&
height=4.375440613275749,height_95%_HPD={3.461904790252447,5.857402443885
803},height_median=4.185547582805157,height_range={3.461904790252447,6.43
2261684909463},length=1.1194786769825789,length_95%_HPD={0.10638877749443
054,2.6262338161468506},length_median=1.0680207014083862,length_range={0.
014291783794760704,3.6460163593292236},posterior=0.13981358189081225,rate
=0.009806924945579384,rate_95%_HPD={0.006519696710192882,0.01331470740572
9544},rate_median=0.009799154349327793,rate_range={0.006187225008112526,0
.015146849570741555},!rotate=true,!color=#ff0000]:0.690102,Metacervocerus
_philisi_2[&height=3.7269870502516347,height_95%_HPD={3.400279011577368,4
.1210610792040825},height_median=3.701601393520832,height_range={3.400279
011577368,4.1993845999240875},length=1.6667107675322512,length_95%_HPD={0
.0,4.217978000640869},length_median=1.4137623310089111,length_range={0.0,
9.629117012023926},rate=0.010469406743071676,rate_95%_HPD={0.007001728905
798535,0.01457786481718723},rate_median=0.010338896795289435,rate_range={
0.005491832410243426,0.017513698596637577},!rotate=true]:1.174048)[&heigh
t=4.970187224878598,height_95%_HPD={4.028474284335971,6.196170195937157},

height_median=4.875649392604828,height_range={3.6919525861740112,6.684915
855526924},length=0.7086775453825449,length_95%_HPD={0.011881908401846886
,1.8090473413467407},length_median=0.5922111868858337,length_range={0.011
881908401846886,2.5746634006500244},posterior=0.1158455392809587З,rate=0.
009857218470006795,rate_95%_HPD={0.006069785600399215,0.01371084462887205
3},rate_median=0.009826708129615514,rate_range={0.005316851344366978,0.01
4662287346690015},!rotate=true,!color=#ff0000]:1.177347)[&height=6.157786
04287548,height_95%_HPD={4.874856369569898,7.982382148504257},height_medi
an=6.0529964286834,height_range={4.462609946727753,9.514259338378906},len
gth=2.2031943543614014,length_95%_HPD={0.28268054127693176,3.288308620452
881},length_median=2.3033130168914795,length_range={0.09193116426467896,4
.231899261474609},posterior=0.8242343541944075,rate=0.009555902435487313,
rate_95%_HPD={0.006537161558322756,0.012540866708431532},rate_median=0.00
954145106753019,rate_range={0.004980583056966109,0.01654062771901984},!ro
tate=true]:2.32444,((((((Cervus_elaphus_NC007704[&height=5.34408846166302
3E-7,height_95%_HPD={0.0,1.0728836059570312E-
6},height_median=4.842877388000488E-
7,height_range={0.0,1.6167759895324707E-
6},length=2.6684767279263024,length_95%_HPD={2.4341251850128174,3.1344478
130340576},length_median=2.5865983963012695,length_range={2.1707706451416
016,3.536275863647461},rate=0.010170837669295722,rate_95%_HPD={0.00702553
12202635945,0.01272262601017307},rate_median=0.010184022102306336,rate_ra
nge={0.0057691155306525185,0.014639535192875017},!rotate=true]:2.664607,E
ucladoceros_ctenoides[&height=2.607240963841316,height_95%_HPD={2.5000904
351472855,2.915844112634659},height_median=2.5469441674649715,height_rang
e={2.5000904351472855,2.999230317771435},length=0.4428951642496755,length
_95%_HPD={0.0,2.6631152629852295},length_median=0.001689409022219479,leng
th_range={0.0,8.519149780273438},rate=0.010040599204744557,rate_95%_HPD={
0.006315278223896939,0.013824126513687881},rate_median=0.0099317897072686
43,rate_range={0.005236485066045247,0.018233203654867285},!rotate=true]:0
.117663)[&height=2.7144167068618117,height_95%_HPD={2.505980968475342,3.0
162705779075623},height_median=2.66460738517344,height_range={2.505980968
475342,3.536276690661907},length=0.5499749247471548,length_95%_HPD={0.083
40331166982651,1.1907765865325928},length_median=0.5078592002391815,lengt
h_range={0.012051178142428398,1.562893033027649},posterior=0.234354194407
45672,rate=0.010553989959210995,rate_95%_HPD={0.006931630075590033,0.0140
29732802842882},rate_median=0.010311934237250076,rate_range={0.0063855210
07744358,0.01668543790042156},!rotate=true,!color=#ff0000]:0.516959,(Cerv
us_nippon_centralis_NC006993[&height=5.318442987837394E-
7,height_95%_HPD={2.9802322387695312E-8,1.087784767150879E-
6},height_median=4.917383193969727E-
7,height_range={0.0,1.5050172805786133E-
6},length=2.3448310774588554,length_95%_HPD={1.8494967222213745,3.0958194
732666016},length_median=2.321629762649536,length_range={1.62220132350921
63,3.3213987350463867},rate=0.010349198486766764,rate_95%_HPD={0.00755249
6434629405,0.01330886098998868},rate_median=0.010234768929870913,rate_ran
ge={0.006733484951472816,0.01602031406537653},!rotate=true]:2.321629,Prze
walskium_albirostris[&height=5.318442987837394E-
7,height_95%_HPD={2.9802322387695312E-8,1.087784767150879E-
6},height_median=4.917383193969727E-
7,height_range={0.0,1.5050172805786133E-
6},length=2.3448310774588554,length_95%_HPD={1.8494967222213745,3.0958194
732666016},length_median=2.321629762649536,length_range={1.62220132350921
63,3.3213987350463867},rate=0.011515295250823777,rate_95%_HPD={0.00871190
0268361385,0.014648914180456668},rate_median=0.011477452284143886,rate_ra
nge={0.007892560190123427,0.01765148929681555},!rotate=true]:2.321629)[&h
eight=2.3448316093031543,height_95%_HPD={1.8494975566864014,3.09581969678
40195},height_median=2.32162993401289,height_range={1.6222024708986282,3.
3213991299271584},length=0.737452439934055,length_95%_HPD={0.454715222120
28503,1.1403177976608276},length_median=0.7198293209075928,length_range={

0.15192851424217224,1.3739314079284668},posterior=1.0,rate=0.010617518281
586897,rate_95%_HPD={0.006620012385208451,0.014373921286055025},rate_medi
an=0.010542693004763504,rate_range={0.005686393325383533,0.01719622257664
832},!rotate=true]:0.859936)[&height=3.230033385338962,height_95%_HPD={2.
75563345849514,3.8643665462732315},height_median=3.181565970182419,height
_range={2.609894171357155,4.287816561758518},length=0.21044723607134982,l
ength_95%_HPD={0.07425450533628464,0.39712268114089966},length_median=0.2
0303812623023987,length_range={0.009968668222427368,0.5547885298728943},p
osterior=0.6830892143808256,rate=0.010038048868370264,rate_95%_HPD={0.006
789158425328646,0.014091467293661591},rate_median=0.00989599376450335,rat
e_range={0.004758695990149947,0.016883589273190558},!rotate=true]:0.18388
7,(Rusa_timorensis[&height=5.383300738373198E-
7,height_95%_HPD={0.0,1.0617077350616455E-
6},height_median=4.917383193969727E-
7,height_range={0.0,1.5497207641601562E-
6},length=2.3793223297865826,length_95%_HPD={1.790930986404419,3.07909393
31054688},length_median=2.3524386882781982,length_range={1.49564790725708
,3.250823974609375},rate=0.008686309372223585,rate_95%_HPD={0.00648014929
6933763,0.01122871376012697},rate_median=0.008634792718493087,rate_range=
{0.005685636396216374,0.013555879143744952},!rotate=true]:2.352438,Rusa_u
nicolor_NC008414[&height=5.383300738373198E-
7,height_95%_HPD={0.0,1.0617077350616455E-
6},height_median=4.917383193969727E-
7,height_range={0.0,1.5497207641601562E-
6},length=2.3793223297865826,length_95%_HPD={1.790930986404419,3.07909393
31054688},length_median=2.3524386882781982,length_range={1.49564790725708
,3.250823974609375},rate=0.007887087693036431,rate_95%_HPD={0.00568637611
2824239,0.010025913734557012},rate_median=0.007830492115040527,rate_range
={0.0050430776573797504,0.012953614297788511},!rotate=true]:2.352438)[&he
ight=2.3793228681166565,height_95%_HPD={1.7909318506717682,3.079094648361
206},height_median=2.3524389415979385,height_range={1.4956482648849487,3.
2508242651820183},length=0.9128151750278853,length_95%_HPD={0.49508026242
256165,1.333738088607788},length_median=0.8955161571502686,length_range={
0.3040589988231659,1.7476530075073242},posterior=1.0,rate=0.0085622505066
5296,rate_95%_HPD={0.00518694900374249,0.011749533109264567},rate_median=
0.008456701180219304,rate_range={0.004367275450141353,0.0156165552997219}
,!rotate=true]:1.013014)[&height=3.4260234918996164,height_95%_HPD={2.868
39316226542,4.088961660861969},height_median=3.3654531836509705,height_ra
nge={2.677476041018963,4.431025080382824},length=0.7593404337060983,lengt
h_95%_HPD={0.4513625204563141,1.2339198589324951},length_median=0.7410867
214202881,length_range={0.0034111991990357637,1.3838533163070679},posteri
or=0.7190412782956058,rate=0.010024643617073187,rate_95%_HPD={0.006129400
479625728,0.013450027844593064},rate_median=0.009941851086038409,rate_ran
ge={0.004862237469226099,0.01669781458888479},!rotate=true]:0.742431,Rusa
_alfredi[&height=5.397019454726972E-
7,height_95%_HPD={0.0,1.084059476852417E-
6},height_median=4.917383193969727E-
7,height_range={0.0,1.6093254089355469E-
6},length=4.037032731839725,length_95%_HPD={3.1785831451416016,4.99128055
5725098},length_median=4.008796691894531,length_range={2.5084404945373535
,5.48317289352417},rate=0.009710412436393093,rate_95%_HPD={0.007418293170
805351,0.011824442436281857},rate_median=0.009649087308525199,rate_range=
{0.006546116051555109,0.013055796442212489},!rotate=true]:4.107884)[&heig
ht=4.162984196571686,height_95%_HPD={3.4362361431121826,5.021559715270996
},height_median=4.107884217053652,height_range={3.1486548110842705,5.4831
73184096813},length=0.8376967869054487,length_95%_HPD={0.4195183515548706
,1.2776271104812622},length_median=0.8107578158378601,length_range={0.159
91447865962982,1.8408539295196533},posterior=0.7696404793608522,rate=0.01
0169641480225994,rate_95%_HPD={0.006693378925299402,0.014182066926015943}
,rate_median=0.010122868295729684,rate_range={0.004619755467313812,0.0171

9471348231698},!rotate=true]:0.839762,(Elaphurus_davidianus[&height=5.415
038252342368E-7,height_95%_HPD={5.9604644775390625E-
8,1.0915100574493408E-6},height_median=4.936009645462036E-
7,height_range={0.0,1.434236764907837E-
6},length=3.4950554935973432,length_95%_HPD={2.654917001724243,4.47763967
5140381},length_median=3.471733570098877,length_range={2.4475066661834717
,4.968967437744141},rate=0.007787127330812435,rate_95%_HPD={0.0056908902
62709362,0.00991562921781754},rate_median=0.007801902410944025,rate_rang
e={0.005087408783445589,0.011397264949577051},!rotate=true]:3.474853,Ruce
rvus_eldi[&height=5.417716889840596E-
7,height_95%_HPD={5.9604644775390625E-8,1.0915100574493408E-
6},height_median=5.066394805908203E-
7,height_range={0.0,1.434236764907837E-
6},length=3.4924115963210753,length_95%_HPD={2.4895036220550537,4.3428535
46142578},length_median=3.472076416015625,length_range={2.148807764053344
7,4.968967437744141},rate=0.009708770778888983,rate_95%_HPD={0.0072849021
57499029,0.012339321083891778},rate_median=0.009646317429875883,rate_rang
e={0.006068123608248864,0.015707487816671365},!rotate=true]:3.474853)[&he
ight=3.5018198288030837,height_95%_HPD={2.654917359352112,4.4660641402006
15},height_median=3.474853515625,height_range={2.4475076124072075,4.96896
86596393585},length=1.385977401725412,length_95%_HPD={0.7227749228477478,
2.035771131515503},length_median=1.3526767492294312,length_range={0.01248
8292530179024,2.4079091548919678},posterior=0.9813581890812251,rate=0.008
757523782187903,rate_95%_HPD={0.005569144942853426,0.012155652529345156},
rate_median=0.008646616447211139,rate_range={0.004847303234377308,0.01448
5907038043061},!rotate=true]:1.472792)[&height=4.989612890614285,height_9
5%_HPD={4.077529683709145,5.986001372337341},height_median=4.947645813226
7,height_range={3.7441102862358093,6.388523608446121},length=1.6384292566
48224,length_95%_HPD={0.9349312782287598,2.3369176387786865},length_media
n=1.6220749020576477,length_range={0.10209784656763077,2.8750357627868652
},posterior=0.796271637816245,rate=0.009275486287912131,rate_95%_HPD={0.0
06046407003168468,0.012304852819212404},rate_median=0.009104053151792807,
rate_range={0.005280066484028559,0.015312918825194557},!rotate=true]:1.70
3232,((Dama_dama_dama[&height=5.306410821019542E-
7,height_95%_HPD={0.0,1.0728836059570312E-
6},height_median=4.76837158203125E-
7,height_range={0.0,1.4901161193847656E-
6},length=3.1557176390913293,length_95%_HPD={1.9500055313110352,5.4983253
47900391},length_median=2.918220281600952,length_range={1.950005531311035
2,7.188061714172363},rate=0.01093862207704929,rate_95%_HPD={0.00760452951
1667045,0.013935227735633824},rate_median=0.010904787372266264,rate_range
={0.006598625867479521,0.016072773414390562},!rotate=true]:3.056746,Dama_
eurygonos[&height=2.460313750123346,height_95%_HPD={1.9500062763690948,3.
244893567636609},height_median=2.376410335302353,height_range={1.95000627
63690948,3.393092103302479},length=0.599374982919585,length_95%_HPD={0.0,
2.3339340686798096},length_median=0.2725480794906616,length_range={0.0,4.
634920597076416},rate=0.010194920386979124,rate_95%_HPD={0.00657477413995
1298,0.014205806268665353},rate_median=0.009970749797464606,rate_range={0
.004956979541699514,0.018515208480919285},!rotate=true]:0.680336)[&height
=3.1762062695206605,height_95%_HPD={1.9920442700386047,4.72466503828764},
height_median=3.0567463636398315,height_range={1.9920442700386047,6.47188
0525350571},length=2.4142771621668104,length_95%_HPD={0.1401882618665695 2
,3.899284601211548},length_median=2.4586684703826904,length_range={0.0199
04188811779022,5.289116859436035},posterior=0.6724367509986684,rate=0.010
769046856562867,rate_95%_HPD={0.00739702328100402,0.01405796198914086},ra
te_median=0.010687280858186930,rate_range={0.006684891349354608,0.01769915
8336643008},!rotate=true]:2.371693,Megaloceros_giganteus_AM182644[&height
=0.27183340715197174,height_95%_HPD={0.24517804384231567,0.30004340782761
574},height_median=0.27126872539520264,height_range={0.2420474998652935,0
.3009360730648041},length=5.184309374952761,length_95%_HPD={3.69866871833

80127,7.065951347351074},length_median=5.171185493469238,length_range={1.
692101716995239З,8.232963562011719},rate=0.010278400224657187,rate_95%_HP
D={0.007466194202190536,0.013487012140457702},rate_median=0.0102312546972
82968,rate_range={0.006126318503291306,0.015986330713873526},!rotate=true
]:5.157171)[&height=5.475330809558117,height_95%_HPD={4.218425095081329,6
.750216938555241},height_median=5.428439617156982,height_range={3.5984567
47829914,7.598667085170746},length=1.0877083105989835,length_95%_HPD={0.0
563915632665157З,1.9014519453048706},length_median=1.081177592277526 9,len
gth_range={0.004166356753557920 5,2.7394943237304688},posterior=0.90146471
37150466,rate=0.01020074605749928,rate_95%_HPD={0.006431677863859540 6,0.0
13798480910790391},rate_median=0.010079791233635825,rate_range={0.0048140
05261719071,0.01764780157254672},!rotate=true]:1.222439)[&height=6.671560
830441532,height_95%_HPD={5.635592460632324,7.938624799251556},height_med
ian=6.650878295302391,height_range={5.2356984578073025,8.514343589544296}
,length=1.695571597332933,length_95%_HPD={0.8488796353340149,2.6442658901
2146},length_median=1.6679978370666504,length_range={0.08067397773265839,
3.60947847366333},posterior=0.7456724367509987,rate=0.009545385293450708,
rate_95%_HPD={0.006268084178919З795,0.013128161355477218},rate_median=0.0
09370762427177 5,rate_range={0.004980583056966109,0.015221049061677506},!r
otate=true]:1.726558)[&height=8.44049382201766,height_95%_HPD={7.06218338
0126953,9.825208380818367},height_median=8.377436585724354,height_range={
6.534320920705795,11.854884386062622},length=4.778143410987043,length_95%
_HPD={3.087130069732666,6.412862300872803},length_median=4.8219251632690 4
3,length_range={1.036341667175293,7.202353000640869},posterior=0.81358189
08122503,rate=0.00858952305957619,rate_95%_HPD={0.006328304335221872,0.01
1023723399740793},rate_median=0.008443641607080946,rate_range={0.00565549
5708765059,0.013812580121038883},!rotate=true]:4.730075,((Elaphodus_cepha
lophus_N008749[&height=5.283352526583115E-
7,height_95%_HPD={0.0,1.1730007827281952E-
6},height_median=4.805624485015869E-
7,height_range={0.0,1.475214958190918E-
6},length=8.735374812597282,length_95%_HPD={3.5159711837768555,12.8030385
97106934},length_median=9.805761337280273,length_range={2.879371881484985
4,14.370221138000488},rate=0.01005702422760053,rate_95%_HPD={0.0072055995
01558352,0.012393851685311295},rate_median=0.009972072727918481,rate_rang
e={0.006129537663755 46,0.01857956097586794},!rotate=true]:6.389773,Cervus
_ruscinensis[&height=4.124312003887866,height_95%_HPD={3.5002323538064957
,4.867226630449295},height_median=4.065888790413737,height_range={3.50023
23538064957,4.99691104888916},length=5.24109486355006,length_95%_HPD={0.0
,12.201981544494629},length_median=4.4475016593933105,length_range={0.0,1
9.10773468017578},rate=0.010826745566840872,rate_95%_HPD={0.0066487550779
84462,0.015078554340596571},rate_median=0.010645189782141127,rate_range={
0.004692155003066161,0.01914218173670361},!rotate=true]:2.323885)[&height
=6.6426349321061915,height_95%_HPD={3.6640032827854156,10.766430854797363
},height_median=6.389773307484575,height_range={3.5884139835834503,12.981
137737631798},length=4.806529919764494,length_95%_HPD={0.1808433532714843
8,7.970863342285156},length_median=5.037539958953857,length_range={0.1621
8627989292145,9.294123649597168},posterior=0.4620505992010652,rate=0.0104
35424975421218,rate_95%_HPD={0.006653086255428483,0.01329008826314504},ra
te_median=0.01041555045849828,rate_range={0.006242976379499231,0.01727412
5642340887},!rotate=true,!color=#ff0000]:5.190367,((Muntiacus_crinifrons_
NC004577[&height=5.334283842348039E-
7,height_95%_HPD={0.0,1.1026859283447266E-
6},height_median=4.76837158203125E-
7,height_range={0.0,1.426786184310913E-
6},length=3.5787473823354343,length_95%_HPD={2.7949984073638916,4.4188947
677612305},length_median=3.552689552307129,length_range={2.52120900154113
77,5.302539825439453},rate=0.009675588533006738,rate_95%_HPD={0.007413649
641926466,0.011852216462055584},rate_median=0.0096159515520128,rate_range
={0.006546116051555109,0.013817218574438146},!rotate=true]:3.571731,Munti

acus_muntjak_NC_004563[&height=5.335542553950215E-
7,height_95%_HPD={0.0,1.1026859283447266E-
6},height_median=4.76837158203125E-
7,height_range={0.0,1.426786184310913E-
6},length=3.579896281784606,length_95%_HPD={2.8129799365997314,4.41195631
0272217},length_median=3.5621068477630615,length_range={2.513707637786865
2,5.302539825439453},rate=0.011227639116968401,rate_95%_HPD={0.0089538244
70158482,0.014064131807274005},rate_median=0.011171291905932319,rate_rang
e={0.0074548845786563955,0.01613732858329011},!rotate=true]:3.571731)[&he
ight=3.5932023753333673,height_95%_HPD={2.873696118593216,4.4119565486907
96},height_median=3.571731150150299,height_range={2.6272087395191193,5.30
25402426719666},length=1.2358316259966187,length_95%_HPD={0.7716102600097
656,1.735483169555664},length_median=1.218642234802246,length_range={0.16
88033491373062,2.4225800037384033},posterior=0.96271637816245,rate=0.0104
38364259371115,rate_95%_HPD={0.007009260004339764,0.013826897026967579},r
ate_median=0.010326904179736416,rate_range={0.005589409955935913,0.019158
31032100543},!rotate=true]:1.243917,(Muntiacus_reevesi_NC008491[&height=5
.434900163375238E-7,height_95%_HPD={0.0,1.1324882507324219E-
6},height_median=5.066394805908203E-
7,height_range={0.0,1.519918441772461E-
6},length=4.285936086377831,length_95%_HPD={3.348567485809326,5.164355278
015137},length_median=4.280540466308594,length_range={2.5646321773529053,
5.975196838378906},rate=0.008752836028767776,rate_95%_HPD={0.007071951367
068699,0.01069490264686341},rate_median=0.008707890603115864,rate_range={
0.005682001644552976,0.011987152633975803},!rotate=true]:4.297676,Muntiac
us_vuquangensis[&height=5.432047910483606E-
7,height_95%_HPD={0.0,1.1324882507324219E-
6},height_median=5.066394805908203E-
7,height_range={0.0,1.519918441772461E-
6},length=4.268611745415293,length_95%_HPD={3.33628249168396,5.3274674415
58838},length_median=4.262481212615967,length_range={2.506932497024536,5.
975196838378906},rate=0.009021252474423109,rate_95%_HPD={0.00731211108651
6611,0.011353412365287361},rate_median=0.008968322870320634,rate_range={0
.006090719986379756,0.013368843691681969},!rotate=true]:4.297676)[&height
=4.317695161755169,height_95%_HPD={3.528955101966858,5.108476012945175},h
eight_median=4.297676861286163,height_range={3.281723141670227,5.97519713
640213},length=0.5223339442028142,length_95%_HPD={0.16892053186893463,0.9
011130332946777},length_median=0.5022076368331909,length_range={0.0314879
6781897545,1.8360538482666016},posterior=0.914780292942743,rate=0.0096452
60405114424,rate_95%_HPD={0.006134426106018596,0.013158202472604942},rate
_median=0.009487458217693588,rate_range={0.004474373691818895,0.017773289
576015212},!rotate=true]:0.517972)[&height=4.851384320509598,height_95%_H
PD={4.150394693017006,5.696265997597948},height_median=4.81564848870039,h
eight_range={3.7554650343954563,6.5075923800468445},length=6.395432370279
659,length_95%_HPD={3.087993860244751,9.350780487060547},length_median=6.
557168960571289,length_range={0.07179883122444153,9.975756645202637},post
erior=0.8748335552596538,rate=0.010125466311475283,rate_95%_HPD={0.007489
7524440696575,0.012908577830722833},rate_median=0.01004090385977471,rate_
range={0.006782521509570261,0.01613732858329011},!rotate=true]:6.764492)[
&height=11.63213050800096,height_95%_HPD={9.902521908283234,13.6981477737
42676},height_median=11.580140054225922,height_range={9.234806895256042,1
4.91204059123993},length=1.59397033380379,length_95%_HPD={0.7244415879249
573,2.8076725006103516},length_median=1.5365985035896301,length_range={0.
0036451411433517933,3.6366169452667236},posterior=0.5033288948069241,rate
=0.010033102120345277,rate_95%_HPD={0.006598392073260062,0.01382980780210
7418},rate_median=0.009887359754730898,rate_range={0.0053126691802725355,
0.01887662880722147},!rotate=true]:1.527372)[&height=13.175779506987622,h
eight_95%_HPD={10.966548204421997,15.16206020116806},height_median=13.107
511818408966,height_range={10.538610711693764,16.17154124379158},length=2
.3680793377596348,length_95%_HPD={0.6121572256088257,3.751033067703247},l

ength_median=2.4689388275146484,length_range={0.0012064240872859955,4.195
015907287598},posterior=0.7283621837549934,rate=0.009505662150949463,rate
_95%_HPD={0.006233320727368551,0.01308645922251122},rate_median=0.0092754
15747195018,rate_range={0.0056200672372101555,0.01737760812403828},!rotat
e=true]:2.882438)[&height=15.953010683822303,height_95%_HPD={14.180547237
39624,17.865455865859985},height_median=15.989949524402618,height_range={
12.876885056495667,18.80690374970436},length=2.4176234203492863,length_95
%_HPD={6.72731184749864E-
5,5.366854190826416},length_median=2.222494125366211,length_range={6.7273
1184749864E-
5,7.541028022766113},posterior=0.34753661784287615,rate=0.010600835945924
866,rate_95%_HPD={0.007785006642359574,0.014343004295111006},rate_median=
0.01063215731046691,rate_range={0.006331847878394572,0.017492592283677926
},!rotate=true,!color=#ff0000]:0.512721,Eostyloceros_hezhengensis[&height
=8.04408588896387,height_95%_HPD={7.104992389678955,8.994356215000153},he
ight_median=8.060691222548485,height_range={7.000348806381226,8.999920018
017292},length=8.129708963211938,length_95%_HPD={2.4836199283599854,12.96
942138671875},length_median=8.416043281555176,length_range={0.0,15.850427
627563477},rate=0.010098190775036863,rate_95%_HPD={0.0059858998456573,0.0
13691361580014409},rate_median=0.009917286502227902,rate_range={0.0051242
20992333772,0.017299490037949557},!rotate=true]:8.441979)[&height=16.5304
2010597012,height_95%_HPD={14.388900637626648,19.5879340544343},height_me
dian=16.50267058610916,height_range={12.930301010608673,20.73454517126083
4},length=2.5059999779802267,length_95%_HPD={0.043354056775569916,5.28350
9731292725},length_median=2.2812716960906982,length_range={0.043354056775
569916,8.260150909423828},posterior=0.2969374167776298,rate=0.01084483340
9530474,rate_95%_HPD={0.006676638264840894,0.014374297640333876},rate_med
ian=0.01073520848531424,rate_range={0.006676638264840894,0.01883607833553
2726},!rotate=true,!color=#ff0000]:1.648718,Euprox_furcatus[&height=13.56
5619918575743,height_95%_HPD={12.500370889902115,14.915273576974869},heig
ht_median=13.44284150749445,height_range={12.500370889902115,15.195730626
5831},length=4.6167887407894455,length_95%_HPD={0.0,9.398566246032715},le
ngth_median=4.145200729370117,length_range={0.0,11.735779762268066},rate=
0.010723296677991883,rate_95%_HPD={0.006981514625845279,0.014529251817542
43},rate_median=0.010569150372811349,rate_range={0.005359965164401016,0.0
19102922318579525},!rotate=true]:4.708547)[&height=18.325099094538018,hei
ght_95%_HPD={15.52496087551117,21.152880772948265},height_median=18.15138
81534338,height_range={14.96552911400795,24.034597516059875},length=2.375
641988658839,length_95%_HPD={0.07574501633644104,4.939699649810791},lengt
h_median=2.3555397987365723,length_range={0.026479098945856094,6.70345687
8662109},posterior=0.3621837549933422,rate=0.01073816473744363,rate_95%_H
PD={0.006852188620920948,0.014439862106948692},rate_median=0.010618492694
306805,rate_range={0.006370463384658679,0.01685868754157856},!rotate=true
,!color=#ff0000]:2.581691,Dicrocerus_elegans[&height=15.977204965926818,h
eight_95%_HPD={15.200605243444443,16.851840496063232},height_median=15.92
926362156868,height_range={15.200605243444443,16.997463762760162},length=
3.097522646027832,length_95%_HPD={0.0,6.760280609130859},length_median=2.
9592833518981934,length_range={0.0,8.90046501159668},rate=0.0105939228078
66972,rate_95%_HPD={0.0068598470140010075,0.014702950035059333},rate_medi
an=0.010374700022252752,rate_range={0.005993286284574233,0.01962968044114
5624},!rotate=true]:4.803816)[&height=20.721284326478635,height_95%_HPD={
17.888621389865875,23.421387881040573},height_median=20.733079612255096,h
eight_range={16.923654824495316,24.15935829281807},length=3.0082809563626
656,length_95%_HPD={0.5252452492713928,5.9948883056640625},length_median=
2.8731424808502197,length_range={0.01104823313653469,7.251901149749756},p
osterior=0.8721704394141145,rate=0.011237415171837467,rate_95%_HPD={0.007
210154752788237,0.015397251954643877},rate_median=0.010983251704547161,ra
te_range={0.006231373517699952,0.019174688743110038},!rotate=true]:3.4075
18,(((Hereroprox_larteti[&height=16.558441522818143,height_95%_HPD={15.24
0358263254166,17.818665124475956},height_median=16.55591982603073,height_

range={15.20108911395073,17.994988664984703},length=1.7010212550845032,le
ngth_95%_HPD={0.17507611215114594,3.273437738418579},length_median=1.6423
430442810059,length_range={0.047169968485832214,7.365030288696289},rate=0
.010062339725157549,rate_95%_HPD={0.006477371734276616,0.014085849307150587
7},rate_median=0.009949304407827294,rate_range={0.005031736142793427 5,0.0
17503946173157786},!rotate=true]:0.829964,Procervulus_dichotomus[&height=
17.81511675830142,height_95%_HPD={17.00019732117653,19.140838861465454},h
eight_median=17.661935716867447,height_range={17.00019732117653,19.983864
4862175},length=0.46645182209562885,length_95%_HPD={0.0,1.447016239166259
8},length_median=0.31237316131591797,length_range={0.0,4.49439001083374},
rate=0.010092195909172564,rate_95%_HPD={0.006074376617680745,0.0136497390
98282905},rate_median=0.010009052477870243,rate_range={0.0051281024179345
61,0.0170217389850558},!rotate=true]:0.0)[&height=17.517647882832982,heig
ht_95%_HPD={17.00068047642708,18.45523653179407},height_median=17.3858842
99874306,height_range={17.00068047642708,19.230565667152405},length=0.992
9907881552736,length_95%_HPD={0.11461999267339706,1.9191257953643799},len
gth_median=0.93672776222229,length_range={0.009220913983881474,3.47517561
9125366},posterior=0.19440745672436752,rate=0.010078040213715557,rate_95%
_HPD={0.006522263150780039,0.01476094829497533 4},rate_median=0.0098543487
0949598,rate_range={0.005525008175695848,0.016547064409789074},!rotate=tr
ue,!color=#ff0000]:1.420492,Procervulus_praelucidus[&height=18.3653795924
37414,height_95%_HPD={18.00030791759491,19.196784049272537},height_median
=18.22685533761978,height_range={18.00030791759491,19.926715448498726},le
ngth=0.28861007564020147,length_95%_HPD={0.0,1.1286689043045044},length_m
edian=0.14995437860488892,length_range={0.0,4.399961471557617},rate=0.010
287535356557435,rate_95%_HPD={0.006404176486279913,0.014058354727913922},
rate_median=0.010100206383779708,rate_range={0.005278262805438528,0.01935
9336610580813},!rotate=true]:0.579521)[&height=18.92710878054181,height_9
5%_HPD={18.034059561789036,20.1660328656435},height_median=18.80637612938
881,height_range={18.016340345144272,21.267756044864655},length=4.1504843
294816585,length_95%_HPD={1.975986361503601,6.1783318519592285},length_me
dian=4.267324447631836,length_range={0.7695871591567993,6.652685165405273
},posterior=0.8095872170439414,rate=0.01120667453720287,rate_95%_HPD={0.0
07670938485010582,0.01539979079977704},rate_median=0.011035991774608022,r
ate_range={0.00641429104043684,0.02034811872301709},!rotate=true]:3.89083
5,Lagomeryx_parvulus[&height=18.82213600796769,height_95%_HPD={18.0049784
70504284,19.842469301074743},height_median=18.76317198574543,height_range
={18.001397907733917,19.995738089084625},length=4.259900157683381,length_
95%_HPD={2.0998692512512207,6.271116733551025},length_median=4.3829431533
81348,length_range={1.0426384210586548,6.798763275146484},rate=0.01124829
7496993466,rate_95%_HPD={0.007849296885584198,0.016016865191312506},rate_
median=0.011042955769292017,rate_range={0.006062430382355765,0.0192920430
5151817},!rotate=true]:3.93404)[&height=22.65155115956102,height_95%_HPD=
{20.85664788633585,24.476619832217693},height_median=22.697211623191833,h
eight_range={19.896968603134155,24.689150959718972},length=1.321974682399
6565,length_95%_HPD={0.005135416518896818,3.0285837650299072},length_medi
an=1.1695653796195984,length_range={0.005135416518896818,4.19781589508056
6},posterior=0.5725699067909454,rate=0.010691876047027978,rate_95%_HPD={0
.006789141131835433 5,0.014938645637046466},rate_median=0.0105443774975428
24,rate_range={0.005564793376089943,0.01947315033439314},!rotate=true]:1.
443386)[&height=24.03326765347809,height_95%_HPD={22.909507136791945,24.9
55934330821037},height_median=24.140597641468048,height_range={21.3031129
98604774,24.990265490778256},length=0.42856721903162437,length_95%_HPD={6
.479003350250423E-
4,1.1721330881118774},length_median=0.3197910785675049,length_range={6.47
9003350250423E-
4,2.9041976928710938},posterior=1.0,rate=0.010191453976803301,rate_95%_HP
D={0.006328304335221872,0.014095023850122386},rate_median=0.0100841130138
77983,rate_range={0.00531870504929507,0.01762453681997733},!rotate=true]:
0.519768,((Antilocapra_americana[&height=5.112485042444558E-

```
7,height_95%_HPD={0.0,1.430511474609375E-
6},height_median=4.172325134277344E-7,height_range={0.0,1.9073486328125E-
6},length=19.666785556371615,length_95%_HPD={16.765857696533203,22.172338
485717773},length_median=19.722116470336914,length_range={15.061622619628
906,23.542560577392578},rate=0.012865800450283611,rate_95%_HPD={0.0106741
90999474089,0.015014452308735658},rate_median=0.012737141435033754,rate_r
ange={0.0099222462407149,0.016733872436406866},!rotate=true]:19.722116,Gi
raffa_camelopardalis_angolensis_NC012100[&height=5.112485042444558E-
7,height_95%_HPD={0.0,1.430511474609375E-
6},height_median=4.172325134277344E-7,height_range={0.0,1.9073486328125E-
6},length=19.666785556371615,length_95%_HPD={16.765857696533203,22.172338
485717773},length_median=19.722116470336914,length_range={15.061622619628
906,23.542560577392578},rate=0.010967255186865846,rate_95%_HPD={0.0092525
7192305694,0.012689765204787365},rate_median=0.010837743790374242,rate_ra
nge={0.008880540273974056,0.015081995291274781},!rotate=true]:19.722116)[
&height=19.66678606762012,height_95%_HPD={16.765859127044678,22.172338485
717773},height_median=19.722116857767105,height_range={15.061622738838196
,23.542560935020447},length=3.1281563116612032,length_95%_HPD={1.62650167
94204712,4.631968021392822},length_median=3.039447784423828,length_range=
{1.1144084930419922,8.02236270904541},posterior=1.0,rate=0.01046443293705
1047,rate_95%_HPD={0.007056339102481924,0.014409214704184048},rate_median
=0.010330958436834297,rate_range={0.005356562382640807,0.0187018535373949
56},!rotate=true]:3.00591,(Moschus_moschiferus[&height=5.354223550903734E
-7,height_95%_HPD={0.0,1.430511474609375E-
6},height_median=4.76837158203125E-7,height_range={0.0,1.9073486328125E-
6},length=20.525633160506043,length_95%_HPD={17.812545776367188,23.041633
60595703},length_median=20.55584144592285,length_range={15.99475479125976
6,23.81341552734375},rate=0.008443374665795214,rate_95%_HPD={0.0071054443
64454797,0.009828671371764611},rate_median=0.008388900339611639,rate_rang
e={0.006653882223236014,0.01136473236832637},!rotate=true]:20.555841,Ovis
_aries_NC001941[&height=5.354223550903734E-
7,height_95%_HPD={0.0,1.430511474609375E-
6},height_median=4.76837158203125E-7,height_range={0.0,1.9073486328125E-
6},length=20.525633160506043,length_95%_HPD={17.812545776367188,23.041633
60595703},length_median=20.55584144592285,length_range={15.99475479125976
6,23.81341552734375},rate=0.011787346486136724,rate_95%_HPD={0.0099839207
03253409,0.013787455651876807},rate_median=0.011681160911481917,rate_rang
e={0.009237456283327457,0.015317448793977766},!rotate=true]:20.555841)[&h
eight=20.5256336959284,height_95%_HPD={17.812546953558922,23.041634887456
894},height_median=20.55584144592285,height_range={15.994755506515503,23.
81341552734375},length=2.2566533038286014,length_95%_HPD={0.9542951583862
305,3.665925979614258},length_median=2.1843624114990234,length_range={0.4
625324308872223,5.162487983703613},posterior=1.0,rate=0.00987239440870730
5,rate_95%_HPD={0.006417073045650391,0.013527262099768648},rate_median=0.
009783895926515301,rate_range={0.00516699309647919,0.01925944335255517},!
rotate=true]:2.172185)[&height=22.609331849827775,height_95%_HPD={20.2253
64476442337,24.690062999725342},height_median=22.728026866912842,height_r
ange={18.8434097468853,24.907302618026733},length=1.880567297162833,lengt
h_95%_HPD={0.008676440455019474,3.904873847961426},length_median=1.766872
763633728,length_range={0.008676440455019474,5.7765374183654785},posterio
r=0.8988015978695073,rate=0.01006607886747896,rate_95%_HPD={0.00647650324
2955231,0.013583063098161681},rate_median=0.009885981952507106,rate_range
={0.004685554937754828,0.01620969756489109},!rotate=true]:1.932339)[&heig
ht=24.495908289710798,height_95%_HPD={23.382965832948685,24.9982221722602
84},height_median=24.660366117954254,height_range={21.54206907749176,24.9
98222172260284},length=0.0,posterior=1.0,rate=1.0,!rotate=true];
end;

begin figtree;
	set appearance.backgroundColorAttribute="Default";
```

```
	set appearance.backgroundColour=#ffffff;
	set appearance.branchColorAttribute="User selection";
	set appearance.branchColorGradient=false;
	set appearance.branchLineWidth=2.0;
	set appearance.branchMinLineWidth=0.0;
	set appearance.branchWidthAttribute="Fixed";
	set appearance.foregroundColour=#000000;
	set appearance.hilightingGradient=false;
	set appearance.selectionColour=#2d3680;
	set branchLabels.colorAttribute="posterior";
	set branchLabels.displayAttribute="posterior";
	set branchLabels.fontName="Helvetica";
	set branchLabels.fontSize=10;
	set branchLabels.fontStyle=0;
	set branchLabels.isShown=true;
	set branchLabels.significantDigits=2;
	set
colour.scheme.posterior="posterior:InterpolatingContinuous{{true,false,0.
0,0.5},#-2536895,#-16777216}";
	set layout.expansion=0;
	set layout.layoutType="RECTILINEAR";
	set layout.zoom=0;
	set legend.attribute="height";
	set legend.fontSize=10.0;
	set legend.isShown=false;
	set legend.significantDigits=4;
	set nodeBars.barWidth=4.0;
	set nodeBars.displayAttribute="height_95%_HPD";
	set nodeBars.isShown=true;
	set nodeLabels.colorAttribute="User selection";
	set nodeLabels.displayAttribute="height_median";
	set nodeLabels.fontName="sansserif";
	set nodeLabels.fontSize=10;
	set nodeLabels.fontStyle=0;
	set nodeLabels.isShown=false;
	set nodeLabels.significantDigits=2;
	set nodeShape.colourAttribute="User selection";
	set nodeShape.isShown=false;
	set nodeShape.minSize=10.0;
	set nodeShape.scaleType=Width;
	set nodeShape.shapeType=Circle;
	set nodeShape.size=4.0;
	set nodeShape.sizeAttribute="Fixed";
	set polarLayout.alignTipLabels=false;
	set polarLayout.angularRange=0;
	set polarLayout.rootAngle=0;
	set polarLayout.rootLength=100;
	set polarLayout.showRoot=true;
	set radialLayout.spread=0.0;
	set rectilinearLayout.alignTipLabels=false;
	set rectilinearLayout.curvature=0;
	set rectilinearLayout.rootLength=100;
	set scale.offsetAge=0.0;
	set scale.rootAge=1.0;
	set scale.scaleFactor=1.0;
	set scale.scaleRoot=false;
	set scaleAxis.automaticScale=true;
	set scaleAxis.fontSize=8.0;
	set scaleAxis.isShown=true;
	set scaleAxis.lineWidth=1.0;
```

```
	set scaleAxis.majorTicks=5.0;
	set scaleAxis.origin=0.0;
	set scaleAxis.reverseAxis=true;
	set scaleAxis.showGrid=false;
	set scaleBar.automaticScale=true;
	set scaleBar.fontSize=10.0;
	set scaleBar.isShown=false;
	set scaleBar.lineWidth=1.0;
	set scaleBar.scaleRange=3.0;
	set tipLabels.colorAttribute="User selection";
	set tipLabels.displayAttribute="Names";
	set tipLabels.fontName="Helvetica";
	set tipLabels.fontSize=10;
	set tipLabels.fontStyle=0;
	set tipLabels.isShown=true;
	set tipLabels.significantDigits=4;
	set trees.order=false;
	set trees.orderType="increasing";
	set trees.rooting=false;
	set trees.rootingType="User Selection";
	set trees.transform=false;
	set trees.transformType="cladogram";
end;
```
