# Supplementary material for: Bony labyrinth morphology clarifies the origin and evolution of deer
Source: Sci Rep. 2017 Oct 13;7:13176. doi: 10.1038/s41598-017-12848-9 (PMC5640792; doi:10.1038/s41598-017-12848-9)
Supplement: Supplementary file 2 — Dataset 1-5 [file 41598_2017_12848_MOESM2_ESM.zip › dataset5.pdf]

```
#NEXUS
begin taxa;
	dimensions ntax=53;
	taxlabels
	Alces_alces
	Antilocapra_americana
	Axis_axis
	Axis_porcinus
	Blastocerus_dichotomus
	Capreolus_capreolus
	Cervus_elaphus_NC007704
	Cervus_nippon_centralis_NC006993
	Cervus_ruscinensis
	Croizetoceros_pyrenaicus
	Croizetoceros_ramosus
	Dama_dama_dama
	Dicrocerus_elegans
	Elaphodus_cephalophus_N008749
	Elaphurus_davidianus
	Eucladoceros_ctenoides
	Euprox_furcatus
	Eostyloceros_hezhengensis
	Giraffa_camelopardalis_angolensis_NC012100
	Hereroprox_larteti
	Hippocamelus_antisensis
	Hydropotes_inermis
	Lagomeryx_parvulus
	Mazama_americana_1
	Mazama_gouazoubira
	Mazama_nemorivaga_1
	Mazama_rufina
	Megaloceros_giganteus_AM182644
	Metacervocerus_philisi_1
	Metacervocerus_philisi_2
	Moschus_moschiferus
	Muntiacus_crinifrons_NC004577
	Muntiacus_muntjak_NC_004563
	Muntiacus_reevesi_NC008491
	Muntiacus_vuquangensis
	Odocoileus_hemionus
	Odocoileus_lucasi
	Odocoileus_cf._virginianus
	Odocoileus_virginianus_1
	Ovis_aries_NC001941
	Ozotoceros_bezoarcticus
	Procervulus_dichotomus
	Procervulus_praelucidus
	Przewalskium_albirostris
	Dama_eurygonos
	Pudu_mephistophiles
	Pudu_puda
	Rangifer_tarandus_NC007703
	Rucervus_duvauceli
	Rucervus_eldi
	Rusa_alfredi
	Rusa_timorensis
	Rusa_unicolor_NC008414
;
end;
```

begin trees;
tree TREE1 = [&R] (((((((Alces_alces[&height=6.331974206578733E-
7,height_95%_HPD={0.0,1.4901161193847656E-
6},height_median=5.960464477539062E-
7,height_range={0.0,2.3245811462402344E-
6},length=16.698065492797795,length_95%_HPD={13.12072467803955,20.0920429
22973633},length_median=16.956602096557617,length_range={3.03278803825378
4,21.12135887145996},rate=0.007113182487062975,rate_95%_HPD={0.0055707107
929106455,0.008919587256113902},rate_median=0.006992709373328593 5,rate_ra
nge={0.005091932868934647,0.01107449103402797}]:16.765215,(Capreolus_capr
eolus[&height=6.195852459064334E-
7,height_95%_HPD={0.0,1.3113021850585938E-
6},height_median=5.960464477539062E-
7,height_range={0.0,2.0265579223632812E-
6},length=11.321254439450868,length_95%_HPD={8.355048179626465,14.0240182
87658691},length_median=11.426297187805176,length_range={4.12703561782836
9,15.240692138671875},rate=0.00925519728075686 5,rate_95%_HPD={0.006925654
0191592,0.012125070127718083},rate_median=0.009058112743094001,rate_range
={0.006280372906492149,0.015682278308269102}]:11.344868,Hydropotes_inermi
s[&height=6.196348998924175E-7,height_95%_HPD={0.0,1.3113021850585938E-
6},height_median=5.960464477539062E-
7,height_range={0.0,2.0265579223632812E-
6},length=11.34211651240536,length_95%_HPD={8.480232238769531,14.04759693
145752},length_median=11.432365417480469,length_range={6.795718193054199,
15.240692138671875},rate=0.00951715483807115,rate_95%_HPD={0.007227435335
9607435,0.012411957254230766},rate_median=0.009275620776258717,rate_range
={0.0064977842220223835,0.016617379902730902}]:11.344868)[&height=11.3430
09098379843,height_95%_HPD={8.46598157286644,14.043275833129883},height_m
edian=11.433399721980095,height_range={6.795718550682068,15.2406927347183
23},length=5.397048684304592,length_95%_HPD={3.3313939571380615,7.6140856
74285889},length_median=5.335847854614258,length_range={0.458182305097579
96,10.295797348022461},posterior=0.9930023325558147,rate=0.00875121558133
8076,rate_95%_HPD={0.006030411741286242,0.012040761480809105},rate_median
=0.008602568026798605,rate_range={0.004607307177707192,0.0153357334434836
97}]:5.420347)[&height=16.749076151926946,height_95%_HPD={13.139220356941
223,19.912191348150373},height_median=16.97878685221076,height_range={11.
191850155591965,21.12135946750641},length=0.8943159359681602,length_95%_H
PD={0.25991350412368774,1.5934585332870483},length_median=0.8476467728614
807,length_range={0.03496889770030975,2.716387987136841},posterior=0.9576
807730756415,rate=0.008302420378285205,rate_95%_HPD={0.005239671848002672
,0.01155201668001647},rate_median=0.008154028897961423,rate_range={0.0042
72074211266174,0.016457741430424565}]:0.889345,(((((Blastocerus_dichotomu
s[&height=6.246166687893381E-7,height_95%_HPD={0.0,1.1920928955078125E-
6},height_median=5.960464477539062E-
7,height_range={0.0,2.3655593395233154E-
6},length=7.116111678080941,length_95%_HPD={4.854650497436523,9.458734512
329102},length_median=7.31281852722168,length_range={0.05397239699959755,
9.60708236694336},rate=0.010001769226979927,rate_95%_HPD={0.0076992514356
65066,0.012727387338598085},rate_median=0.009883578865838108,rate_range={
0.006073266982510938,0.016352424602354542}]:7.405314,((Hippocamelus_antis
ensis[&height=6.322726927532714E-7,height_95%_HPD={8.940696716308594E-
8,1.2516975402832031E-6},height_median=5.960464477539062E-
7,height_range={0.0,2.384185791015625E-
6},length=4.548538774110424,length_95%_HPD={3.107394218444824,5.884088993
07251},length_median=4.603091239929199,length_range={0.0648970752954483,6
.963600158691406},rate=0.009263962673400145,rate_95%_HPD={0.0068427143474
19154,0.012039613859823568},rate_median=0.009108914066184012,rate_range={
0.005019332885596687,0.01610510078687898}]:4.642086,Mazama_gouazoubira[&h
eight=6.320144920261537E-7,height_95%_HPD={5.9604644775390625E-
8,1.2218952178955078E-6},height_median=5.960464477539062E-

7,height_range={0.0,2.384185791015625E-
6},length=4.527623594108868,length_95%_HPD={3.0731890201568604,5.88408899
307251},length_median=4.591820240020752,length_range={0.01518864557147026
,6.963600158691406},rate=0.009257582618559678,rate_95%_HPD={0.00696927921
5594312,0.012233453717310406},rate_median=0.009113965856613152,rate_range
={0.005341599518046855,0.01530456951900953}]:4.642086)[&height=4.62223809
0129279,height_95%_HPD={3.497459292411804,5.863076776266098},height_media
n=4.614778518676758,height_range={2.703029841184616,6.963600908406079},le
ngth=2.2311858069165056,length_95%_HPD={1.4225980043411255,3.192190885543
8232},length_median=2.210233688354492,length_range={0.011151861399412155,
4.1985182762146},posterior=0.9253582139286904,rate=0.009103064070463822,r
ate_95%_HPD={0.006143689240728944,0.012623389652459006},rate_median=0.008
912897124696204,rate_range={0.004861180969848838,0.015320397127065425}]:2
.247018,Ozotoceros_bezoarcticus[&height=6.344729850071947E-
7,height_95%_HPD={0.0,1.1920928955078125E-
6},height_median=6.034970283508301E-
7,height_range={0.0,2.1457672119140625E-
6},length=6.730223475271753,length_95%_HPD={5.043807029724121,8.682343482
971191},length_median=6.833737373352051,length_range={0.04224954918026924
,9.15800952911377},rate=0.008212196486841083,rate_95%_HPD={0.006394663724
11521,0.010391025129491673},rate_median=0.008094135424838346,rate_range={
0.005381335863358655 5,0.013447626955240684}]:6.889104)[&height=6.85992399
1169992,height_95%_HPD={5.292174369096756,8.408079236745834},height_media
n=6.853628128767013 5,height_range={4.208789795637131,9.055038183927536},l
ength=0.5167236803653233,length_95%_HPD={0.1513012051582 3364,0.9272544980
049133},length_median=0.49481964111328125,length_range={0.023037059232592
583,1.442014217376709},posterior=0.8687104298567144,rate=0.00846711154131
1256,rate_95%_HPD={0.005485061222472248,0.011867310569431553},rate_median
=0.0082799960945648,rate_range={0.004311544774988059,0.01708408994837167}
]:0.51621)[&height=7.367734791097463,height_95%_HPD={5.727415069937706,9.
002465069293976},height_median=7.357928365468979,height_range={4.66017854
2137146,9.607083261013031},length=0.5171109939322391,length_95%_HPD={0.15
528011322021484,0.8941926956176758},length_median=0.4939457029104233,leng
th_range={0.05339677259325981,1.5075832605361938},posterior=0.80906364545
15162,rate=0.00850579515280934,rate_95%_HPD={0.005415339285494204,0.01182
4250677834958},rate_median=0.008312690808195978,rate_range={0.00402033590
0498515,0.01666811448620408}]:0.524399,Mazama_nemorivaga_1[&height=6.2550
39234513927E-7,height_95%_HPD={0.0,1.2516975402832031E-
6},height_median=5.960464477539062E-
7,height_range={0.0,2.205371856689453E-
6},length=7.732911632729273,length_95%_HPD={5.819642066955566,9.751957893
371582},length_median=7.8793158531188965,length_range={0.0260637868195772
17,10.201858520507812},rate=0.010056774137432358,rate_95%_HPD={0.00772269
047393221,0.012675729165531086},rate_median=0.009899978308623023,rate_ran
ge={0.005159325003141464,0.016277337635215 47}]:7.929713)[&height=7.870962
0465795584,height_95%_HPD={6.06382966786623,9.472409099340439},height_med
ian=7.873183101415634,height_range={5.0325922928750515,10.117512792348862
},length=0.5909245755262077,length_95%_HPD={0.22025762498378754,1.0405348
539352417},length_median=0.5662918090820312,length_range={0.0452107749879
3602,1.6892880201339722},posterior=0.7504165278240587,rate=0.008654189769
535282,rate_95%_HPD={0.005513023590227888,0.012124861939359445},rate_medi
an=0.008499587693177504,rate_range={0.0040407827511481954,0.0183116151685
72257}]:0.581851,Pudu_puda[&height=6.252208957312829E-
7,height_95%_HPD={0.0,1.2516975402832031E-
6},height_median=5.960464477539062E-
7,height_range={0.0,2.384185791015625E-
6},length=8.384976162786526,length_95%_HPD={6.3752055168151855,10.4389114
37988281},length_median=8.479503631591797,length_range={0.855310440063476
6,10.85654354095459},rate=0.008186985608021204,rate_95%_HPD={0.0064986291
75822944,0.010481771868264203},rate_median=0.00805174418074598,rate_range

={0.005691534540666302,0.01331073745141898}]:8.511564)[&height=8.45897757
144038,height_95%_HPD={6.60040009021759,10.276647716760635},height_median
=8.464537784457207,height_range={5.468471135944128,10.853541553020477},le
ngth=3.370317022757977,length_95%_HPD={2.280214548110962,4.69983100891113
3},length_median=3.3373501300811768,length_range={0.01546996459364891,5.6
64191246032715},posterior=0.7354215261579473,rate=0.010523715372170346,ra
te_95%_HPD={0.007208189279931747,0.014125104984023243},rate_median=0.0103
76168200190277,rate_range={0.006212542266882612,0.018494752552767297}]:3.
529213,(((Mazama_americana_1[&height=6.239633261028587E-
7,height_95%_HPD={0.0,1.1920928955078125E-
6},height_median=5.960464477539062E-
7,height_range={0.0,2.1457672119140625E-
6},length=4.168041954216104,length_95%_HPD={1.73759925365448,6.0668702125
54932},length_median=4.356235504150391,length_range={0.8592497110366821,7
.040121078491211},rate=0.009299107359148384,rate_95%_HPD={0.0067097305270
94867,0.012271054920045087},rate_median=0.00912420957393827,rate_range={0
.005675107689541621,0.01516803125992378}]:6.515438,Odocoileus_lucasi[&hei
ght=6.230073171568912E-7,height_95%_HPD={0.0,1.1920928955078125E-
6},height_median=5.960464477539062E-
7,height_range={0.0,2.425163984298706E-
6},length=3.636815215497343,length_95%_HPD={0.002262458438053727,7.651970
863342285},length_median=3.3281569480895996,length_range={0.0022624584380
53727,17.047039031982422},rate=0.008399529835605815,rate_95%_HPD={0.00542
0705142221024,0.011625598537929889},rate_median=0.008216388120750179,rate
_range={0.004452788816668585,0.016942857792329756}]:6.515438)[&posterior=
0.3842052649116961,rate=0.008903734880349923,!color=#ff0000]:1.080829,((O
docoileus_hemionus[&height=6.246475231167616E-
7,height_95%_HPD={5.9604644775390625E-8,1.1920928955078125E-
6},height_median=5.960464477539062E-
7,height_range={0.0,2.305954694747925E-
6},length=1.3577752481436205,length_95%_HPD={0.026571456342935562,1.99625
40864944458},length_median=1.4856512546539307,length_range={0.00128340267
11061597,2.5153982639312744},rate=0.007366392671437209,rate_95%_HPD={0.00
4778251918927688,0.01008833802615617},rate_median=0.007193417920603234,ra
te_range={0.004100107474982272,0.014844848351160927}]:3.613504,Odocoileus
_cf._virginianus[&height=6.225043280976982E-
7,height_95%_HPD={0.0,1.1622905731201172E-
6},height_median=5.960464477539062E-
7,height_range={0.0,2.246350049972534E-
6},length=2.1338555926611935,length_95%_HPD={1.8026713223662227E-
4,6.469006538391113},length_median=1.421639084815979,length_range={1.8026
713223662227E-
4,18.26127052307129},rate=0.008399786138906238,rate_95%_HPD={0.0053603639
17100964,0.01201559443434823},rate_median=0.008205522938359494,rate_range
={0.004213974741020325,0.016486086253950247}]:3.613504)[&posterior=0.2832
3892035988,rate=0.007542087196411573,!color=#ff0000]:0.22535,Odocoileus_v
irginianus_1[&height=6.244071570928129E-
7,height_95%_HPD={5.9604644775390625E-8,1.2032687664031982E-
6},height_median=5.960464477539062E-
7,height_range={0.0,2.246350049972534E-
6},length=1.4622010063381243,length_95%_HPD={0.607652485370636,2.13451528
54919434},length_median=1.5041465759277344,length_range={0.11843741685152
054,2.469646692276001},rate=0.009390558365489866,rate_95%_HPD={0.00644866
1277907415,0.012765723575445703},rate_median=0.009167180683547067,rate_ra
nge={0.005095377554328371,0.018311615168572257}]:3.838854)[&height=1.8965
884928579635,height_95%_HPD={1.005492627620697,3.4502662271261215},height
_median=1.6891238689422607,height_range={0.9443507641553879,5.56287783384
3231},length=2.6740361833124497,length_95%_HPD={0.4579732418060303,4.2963
77658843994},length_median=2.80169677734375,length_range={0.0293899551033
9737,5.203842639923096},posterior=0.7527490836387871,rate=0.0078424320527

70427,rate_95%_HPD={0.005260101635842918,0.010546890054957183},rate_media
n=0.007655309775653842,rate_range={0.004159462053457545,0.013795442983792
873}]:3.757414)[&height=5.05159418035387,height_95%_HPD={3.51901715993881
23,6.776657372713089},height_median=4.943226158618927,height_range={3.017
290472984314,11.156096458435059},length=4.248365175119351,length_95%_HPD=
{2.3266918659210205,6.217705726623535},length_median=4.284366369247436 5,l
ength_range={0.0373515859246254,7.177684307098389},posterior=0.5924691769
410196,rate=0.007673423709977846,rate_95%_HPD={0.0052910219252397 5,0.0103
55959330777199},rate_median=0.007543018489510217,rate_range={0.0047577313
98009011,0.013547131901625114}]:2.517484,(Mazama_rufina[&height=6.2838172
10687639E-7,height_95%_HPD={0.0,1.2516975402832031E-
6},height_median=5.960464477539062E-
7,height_range={0.0,2.205371856689453E-
6},length=1.0474330779857934,length_95%_HPD={0.6871339678764343,1.4402228
593826294},length_median=1.0547362565994263,length_range={0.0014576193643
73386,1.8196324110031128},rate=0.008767033687150066,rate_95%_HPD={0.00591
0598244421379,0.012079759913778708},rate_median=0.008562498825379537,rate
_range={0.004769434266470307,0.017294956284399664}]:1.060589,Pudu_mephist
ophiles[&height=6.281586709026227E-
7,height_95%_HPD={0.0,1.2516975402832031E-
6},height_median=5.960464477539062E-
7,height_range={0.0,2.205371856689453E-
6},length=1.0431927178014733,length_95%_HPD={0.6871339678764343,1.4546890
258789062},length_median=1.052632212638855,length_range={1.80267132236622
27E-
4,1.8196324110031128},rate=0.008328679316349709,rate_95%_HPD={0.005689837
64596118,0.011566397691204626},rate_median=0.008132641261345329,rate_rang
e={0.004760832914190944,0.015729636753380613}]:1.060589)[&height=1.060074
6590011478,height_95%_HPD={0.7219478487968445,1.4127212762832642},height_
median=1.0582618117332458,height_range={0.5730948150157928,1.819633245468
1396},length=7.683894316094454,length_95%_HPD={2.2829806804656982,10.7851
22871398926},length_median=8.143707275390625,length_range={0.001818560063
8389587,11.169742584228516},posterior=0.9460179940019994,rate=0.008866337
101994687,rate_95%_HPD={0.006441148929768074,0.011372500609458906},rate_m
edian=0.008715914612521851,rate_range={0.004691837961373579,0.01446794021
4005326}]:9.053163)[&height=9.296860434137422,height_95%_HPD={7.086719155
3115845,11.530152384191751},height_median=9.314250066876411,height_range=
{5.969786286354065,12.879915781319141},length=2.577815188831723,length_95
%_HPD={1.4180494546890259,3.7446584701538086},length_median=2.52705168724
06006,length_range={0.1272279918193817,5.300347805023193},posterior=0.732
0893035654782,rate=0.008090091221150586,rate_95%_HPD={0.00516885192574820
8,0.01123142171961003},rate_median=0.007908817471845178,rate_range={0.004
471722647207824,0.01418565267455178}]:1.927025)[&height=11.88155805908681
,height_95%_HPD={9.477073311805725,14.308456063270569},height_median=11.9
95279625058174,height_range={8.01294618844986,15.124253869056702},length=
1.467159663349587,length_95%_HPD={0.73214191198349,2.234426736831665},len
gth_median=1.4296636581420898,length_range={0.16200189292430878,3.4396092
891693115},posterior=0.9083638787070977,rate=0.009010569997414812,rate_95
%_HPD={0.005999304111474837,0.012693819920360231},rate_median=0.008798322
430164356,rate_range={0.004051605976268534,0.017164141060722313}]:1.43692
3,Rangifer_tarandus_NC007703[&height=6.2320579732821E-
7,height_95%_HPD={0.0,1.3113021850585938E-
6},height_median=5.960464477539062E-
7,height_range={0.0,2.4065375328063965E-
6},length=12.337252219690159,length_95%_HPD={5.2391357421875,16.120422363
28125},length_median=13.11894416809082,length_range={1.3085812330245972,1
7.28158950805664},rate=0.007725220113866871,rate_95%_HPD={0.0058978941157
46893,0.009972285349679677},rate_median=0.007582535935175822,rate_range={
0.004217802441491726 5,0.012655723166739321}]:13.4777)[&height=13.36212112
2344627,height_95%_HPD={10.401543509215117,15.8813702464103 7},height_medi

an=13.513618379831314,height_range={8.733571350574493,17.149386698380113}
,length=4.142641083175288,length_95%_HPD={2.746177911758423,5.86839771270
75195},length_median=4.1613969802856445,length_range={0.03748710826039314
,7.020548343658447},posterior=0.7390869710096635,rate=0.00995331714062658
,rate_95%_HPD={0.007008449902833988,0.013827388862481138},rate_median=0.0
09724049436588347,rate_range={0.005832338007228342,0.016081043184720176}]
:4.17686)[&height=17.587138596868478,height_95%_HPD={13.806722700595856,2
0.892878651618958},height_median=17.86014711856842,height_range={11.55166
8763160706,21.86394512653351},length=1.6323766082214082,length_95%_HPD={0
.680438220500946,2.734360694885254},length_median=1.604248046875,length_r
ange={0.012017258442938328,3.6588010787963867},posterior=0.70809730089970
01,rate=0.00901940630267713,rate_95%_HPD={0.005883238681370788,0.01228984
3899439115},rate_median=0.008864092087677878,rate_range={0.00466599650806
1202,0.0186710076769011}]:1.974991,(Croizetoceros_pyrenaicus[&height=6.12
11124849906655,height_95%_HPD={5.301047593355179,6.901580274105072},heigh
t_median=6.088644277304411,height_range={5.300421297550201,6.999853849411
011},length=1.9931192609825172,length_95%_HPD={2.5165820261463523E-
4,6.293680191040039},length_median=1.3218039274215698,length_range={2.516
5820261463523E-
4,16.137556076049805},rate=0.008406432740688564,rate_95%_HPD={0.005233038
782625452,0.011620872132508977},rate_median=0.008245296356982026,rate_ran
ge={0.003927668164749973,0.015359160436679667}]:2.080482,Croizetoceros_ra
mosus[&height=3.8008724063467225,height_95%_HPD={3.42895644903183,4.18957
382440567},height_median=3.7981629371643066,height_range={3.4000049233436
584,4.199835949577391},length=4.0075002243022055,length_95%_HPD={0.004245
981108397245,8.242035865783691},length_median=3.5749247074127197,length_r
ange={0.004245981108397245,15.098678588867188},rate=0.008453789243345368,
rate_95%_HPD={0.005468359265185455,0.01182046505617884},rate_median=0.008
252236817576305,rate_range={0.004550555106693743,0.01614877594265984}]:4.
400722)[&height=8.024499262210323,height_95%_HPD={5.426042288541794,12.01
106210052967},height_median=7.518055122811347,height_range={5.33219628781
08025,18.402876883745193},length=9.150173594204988,length_95%_HPD={0.2562
578320503235,15.580187797546387},length_median=9.657908916473389,length_r
ange={0.01159396767616272,22.187028884887695},posterior=0.847050983005664
7,rate=0.008376637420460934,rate_95%_HPD={0.005305051205958324,0.01168895
7252092036},rate_median=0.008160572098163645,rate_range={0.00395717644893
447,0.016821892288071013}]:11.427957)[&posterior=0.3382205931356215,rate=
0.011236336080577485,!color=#ff0000]:3.200232,(((((((Axis_axis[&height=6.
249429546353233E-7,height_95%_HPD={0.0,1.1920928955078125E-
6},height_median=5.960464477539062E-
7,height_range={0.0,2.562999725341797E-
6},length=4.742780754304814,length_95%_HPD={3.5032832622528076,6.09802532
1960449},length_median=4.733974456787109,length_range={2.737508535385132,
7.021327972412109},rate=0.008243617551794844,rate_95%_HPD={0.006042658890
089902,0.010941047458160684},rate_median=0.008075353064560645,rate_range=
{0.004860591955114405,0.013893810361426616}]:4.840885,Axis_porcinus[&heig
ht=6.273524773424917E-7,height_95%_HPD={0.0,1.1920928955078125E-
6},height_median=5.960464477539062E-
7,height_range={0.0,2.562999725341797E-
6},length=4.702881219743769,length_95%_HPD={3.505742311477661,6.087549686
431885},length_median=4.690741062164307,length_range={2.4075963497161865,
6.789316654205322},rate=0.008859662069453192,rate_95%_HPD={0.006426077958
067139,0.011730998359078197},rate_median=0.008702706516011691,rate_range=
{0.005046080065160267,0.014237807402092514}]:4.840885)[&height=4.72112178
7854314,height_95%_HPD={3.443831082433462,6.0980260372161865},height_medi
an=4.726817287504673,height_range={2.8727364987134933,6.694447040557861},
length=1.6470758522144187,length_95%_HPD={0.08214535564184189,2.739773273
4680176},length_median=1.7166396975517273,length_range={0.001347930054180
324,3.9628217220306396},posterior=0.7057647450849717,rate=0.0085457911363
3102,rate_95%_HPD={0.005414149142101585,0.01193657283033127},rate_median=

0.008356915780352914,rate_range={0.004103774368846106,0.01527923175877572
2}]:1.973831,Metacervocerus_philisi_1[&height=3.805427502718428,height_95
%_HPD={3.440278574824333,4.199862721376121},height_median=3.8037415146827
698,height_range={3.400203377008438,4.199862721376121},length=1.711117780
218476,length_95%_HPD={0.00253248936496675,4.370080471038818},length_medi
an=1.3994522094726562,length_range={2.4198800019803457E-
5,10.93099594116211},rate=0.008353860667861994,rate_95%_HPD={0.0054539211
62899573,0.011797347851854949},rate_median=0.008155623693325181,rate_rang
e={0.0035367718772063585,0.01688210275855068 7}]:3.009289)[&posterior=0.31
556147950683106,rate=0.007584801645821531,!color=#ff0000]:0.699101,Rucerv
us_duvauceli[&height=6.244899395975709E-
7,height_95%_HPD={0.0,1.1920928955078125E-
6},height_median=5.960464477539062E-
7,height_range={0.0,2.384185791015625E-
6},length=6.199739329936464,length_95%_HPD={3.8940823078155518,8.21860504
1503906},length_median=6.335719108581543,length_range={3.449235200881958,
9.272561073303223},rate=0.007162213616931 6505,rate_95%_HPD={0.00521785648
84208705,0.009207844288138912},rate_median=0.007025411121625674,rate_rang
e={0.004526672434146644,0.011610352013668647}]:7.513817)[&posterior=0.201
5994668443852,rate=0.007542087196411573,!color=#ff0000]:0.491288,Metacerv
ocerus_philisi_2[&height=3.756517332347874,height_95%_HPD={3.401876211166
382,4.146205205470324},height_median=3.7329275608062744,height_range={3.4
00838017463684,4.199808239936829},length=2.368931830067796,length_95%_HPD
={2.0225926709827036E-
4,5.927083969116211},length_median=1.9361306428909302,length_range={2.022
5926709827036E-
4,12.593661308288574},rate=0.008666554650 82413,rate_95%_HPD={0.0056051491
97560573,0.01213087805820919},rate_median=0.008503551380026836,rate_range
={0.004507076309658205,0.016812683428460276}]:4.248588)[&height=7.2435651
21426125,height_95%_HPD={5.4272080063819885,9.283992439508438},height_med
ian=7.16828852891922,height_range={4.778569877147675,12.028089463710785},
length=2.705268217424619,length_95%_HPD={0.5608909130096436,4.18877792358
3984},length_median=2.8506362438201904,length_range={0.008760055527091026
,5.43519926071167},posterior=0.7387537487504166,rate=0.007978021862574968
,rate_95%_HPD={0.005396445953108217,0.010873927294761082},rate_median=0.0
07764653600970508,rate_range={0.004201104623306919,0.01520439292531951}]:
3.045332,((((((Cervus_elaphus_NC007704[&height=6.250509908470154E-
7,height_95%_HPD={8.940696716308594E-8,1.2218952178955078E-
6},height_median=5.960464477539062E-
7,height_range={0.0,2.562999725341797E-
6},length=3.0596179459262633,length_95%_HPD={2.519177198410034,3.79054737
09106445},length_median=2.9911537170410156,length_range={2.32590723037719
73,4.335023880004883},rate=0.008769819401547727,rate_95%_HPD={0.006095909
32651775,0.011373955069309868},rate_median=0.008617053385556137,rate_rang
e={0.004945241693520118,0.013935541111552 29}]:5.480091,Eucladoceros_cteno
ides[&height=2.706874888573102,height_95%_HPD={2.5000884234905243,2.96004
82434034348},height_median=2.686877742409706,height_range={2.500088423490
5243,2.99950347840786},length=0.7070894079466348,length_95%_HPD={1.326356
1413623393E-
4,2.728088140487671},length_median=0.3117855489253998,length_range={1.326
3561413623393E-
4,12.926254272460938},rate=0.008530582077177249,rate_95%_HPD={0.005668372
988616684,0.012243630750371767},rate_median=0.008331099555881901,rate_ran
ge={0.003977837865328894,0.017685767944241124}]:2.773217)[&height=2.95560
86869380778,height_95%_HPD={2.5191781520843506,3.4726979732513428},height
_median=2.915374740958214,height_range={2.510887950658798,4.2425204664468
765},length=0.7819748123074092,length_95%_HPD={0.11308451741933823,1.4693
549871444702},length_median=0.7660358250141144,length_range={0.0017556885
723024607,2.1582508087158203},posterior=0.659780073308897,rate=0.00868530
4109842426,rate_95%_HPD={0.006119002153371801 5,0.012112738890456443},rate

_median=0.008502536549114828,rate_range={0.004723907313033803,0.015563186
27917192}]:0.515949,(Cervus_nippon_centralis_NC006993[&height=6.250453271
892391E-7,height_95%_HPD={8.940696716308594E-8,1.2218952178955078E-
6},height_median=5.960464477539062E-
7,height_range={0.0,2.562999725341797E-
6},length=2.68841332691743,length_95%_HPD={1.910875678062439,3.3609888553
619385},length_median=2.697084903717041,length_range={1.5638352632522583,
3.925201892852783},rate=0.008989285753712798,rate_95%_HPD={0.006635363776
689809,0.01198238171973315},rate_median=0.008783015441288485,rate_range={
0.005929707794425339,0.015577496928069994}]:2.688661,Przewalskium_albiros
tris[&height=6.249180888501547E-7,height_95%_HPD={8.940696716308594E-
8,1.2218952178955078E-6},height_median=5.960464477539062E-
7,height_range={0.0,2.562999725341797E-
6},length=2.688195652343638,length_95%_HPD={1.9143801927566528,3.36098885
53619385},length_median=2.697514533996582,length_range={1.563835263252258
3,3.925201892852783},rate=0.010050787661066218,rate_95%_HPD={0.0073564465
29353849,0.013359727060544255},rate_median=0.009857229050119232,rate_rang
e={0.006505335781689734,0.016126439211696052}]:2.688661)[&height=2.687140
7566919356,height_95%_HPD={1.9134810268878937,3.3609890788793564},height_
median=2.696458101272583,height_range={1.563835933804512,3.92520287632942
2},length=0.8759215029985971,length_95%_HPD={0.45313721895217896,1.319751
3818740845},length_median=0.870094507932663,length_range={0.0040749986656
01015,1.801549434661865 2},posterior=0.996334555148284,rate=0.008946431368
440077,rate_95%_HPD={0.005709703753322628,0.012607137455707513},rate_medi
an=0.008669336734584542,rate_range={0.004079032074121974,0.01913505922112
364}]:3.307379)[&height=3.7235620694335094,height_95%_HPD={3.060749679803
8483,4.42534813284874},height_median=3.7103251963853836,height_range={2.6
524953693151474,4.9178639352321625},length=0.2440658687131306,length_95%_
HPD={0.0710362046957016,0.43442678451538086},length_median=0.234299898147
583,length_range={0.009100762195885181,0.6660426259040833},posterior=0.70
54315228257247,rate=0.008325476933798684,rate_95%_HPD={0.0054448812489543
38,0.011478737700935219},rate_median=0.00814048093893348,rate_range={0.00
3805318832712914,0.015714800487590955}]:0.172172,(Rusa_timorensis[&height
=6.255927817808028E-7,height_95%_HPD={5.9604644775390625E-
8,1.1920928955078125E-6},height_median=5.960464477539062E-
7,height_range={0.0,2.562999725341797E-
6},length=2.7382662771384503,length_95%_HPD={1.9707154035568237,3.5347940
921783447},length_median=2.743037223815918,length_range={1.52846884727478
03,4.1144514083862305},rate=0.007560724755286457,rate_95%_HPD={0.00547464
60353723315,0.009893253855168038},rate_median=0.007412367525240081,rate_r
ange={0.004735581140592425,0.013397138763250566}]:2.738266,Rusa_unicolor_
NC008414[&height=6.255927817808028E-
7,height_95%_HPD={5.9604644775390625E-8,1.1920928955078125E-
6},height_median=5.960464477539062E-
7,height_range={0.0,2.562999725341797E-
6},length=2.7382662771384503,length_95%_HPD={1.9707154035568237,3.5347940
921783447},length_median=2.743037223815918,length_range={1.52846884727478
03,4.1144514083862305},rate=0.006896015120824053,rate_95%_HPD={0.00505236
7366372376,0.009079929893580436},rate_median=0.006747284488537073,rate_ra
nge={0.004475225133875186,0.010994516918200983}]:2.738266)[&height=2.7382
669027312323,height_95%_HPD={1.9707159399986267,3.5347947776317596},heigh
t_median=2.7430376037955284,height_range={1.5284692645072937,4.1144524812
69836},length=1.07523310704356,length_95%_HPD={0.6240957975387573,1.62566
6856765747},length_median=1.0515555143356323,length_range={0.056599900126
457214,2.1180248260498047},posterior=1.0,rate=0.007365131765236431,rate_9
5%_HPD={0.004468865878380224,0.010129622422386226},rate_median=0.00718845
411788167,rate_range={0.004030961112775788,0.017439695807600086}]:3.42994
5)[&height=3.9645878518794038,height_95%_HPD={3.242888957262039,4.7137530
44605255},height_median=3.95462965965271,height_range={2.886509120464325,
5.075617551803589},length=0.9299343944038159,length_95%_HPD={0.4948616325

855255,1.4012144804000854},length_median=0.9088397026062012,length_range=
{0.023206263780593872,1.8273087739944458},posterior=0.7297567477507497,ra
te=0.00831224100210786,rate_95%_HPD={0.00515293573371771,0.01135094798020
009},rate_median=0.008158160481849797,rate_range={0.004865887702959966,0.
015439946605072672}]:0.678843,Rusa_alfredi[&height=6.294921907637099E-
7,height_95%_HPD={0.0,1.1920928955078125E-
6},height_median=5.960464477539062E-
7,height_range={0.0,2.5033950805664062E-
6},length=4.6951587320128825,length_95%_HPD={3.4505856037139893,5.7609658
24127197},length_median=4.73189115524292,length_range={2.6526200771331787
,6.399649620056152},rate=0.008365199355470403,rate_95%_HPD={0.00648915786
58631,0.010791579707119048},rate_median=0.008219276042504838,rate_range={
0.00548664823445307,0.01266817975340523}]:6.847055)[&height=4.87453965326
2861,height_95%_HPD={3.9313080608844757,5.754188179969788},height_median=
4.87763287872076,height_range={3.443804830312729,6.3996498584747314},leng
th=1.0291290747218054,length_95%_HPD={0.5924600958824158,1.56326508522033
7},length_median=0.9997484385967255,length_range={0.03712661564350128,2.0
18094062805176},posterior=0.769076974341886,rate=0.008281511305471926,rat
e_95%_HPD={0.005596995358683462,0.011347518974771093},rate_median=0.00812
7281477047004,rate_range={0.004515784656739972,0.015459410803799428}]:0.7
95685,(Elaphurus_davidianus[&height=6.20609868514674E-
7,height_95%_HPD={0.0,1.1920928955078125E-
6},height_median=5.960464477539062E-
7,height_range={0.0,2.6226043701171875E-
6},length=4.118880334833152,length_95%_HPD={2.970374345779419,5.230352878
570557},length_median=4.099576473236084,length_range={2.390920639038086,5
.952535629272461},rate=0.006650437365553592,rate_95%_HPD={0.0047902861757
46532,0.0087048506188186},rate_median=0.006560953235489493,rate_range={0.
004279534310099477,0.01193033324138607}]:4.121776,Rucervus_eldi[&height=6
.206390402314397E-7,height_95%_HPD={0.0,1.1920928955078125E-
6},height_median=5.960464477539062E-
7,height_range={0.0,2.6226043701171875E-
6},length=4.1152457981973996,length_95%_HPD={2.966909646987915,5.23892974
8535156},length_median=4.096084117889404,length_range={2.4110729694366455
,5.952535629272461},rate=0.008266280115732771,rate_95%_HPD={0.00599753602
7950784,0.010725890281431618},rate_median=0.00811981326062181,rate_range=
{0.004916893139374591,0.013820768227063013}]:4.121776)[&height=4.11764233
1540671,height_95%_HPD={2.967202305793762,5.218398332595825},height_media
n=4.096716284751892,height_range={2.4110734909772873,5.9525365978479385},
length=1.6049449441145363,length_95%_HPD={0.8701802492141724,2.4223558902
74048},length_median=1.5732391476631165,length_range={0.00573617033660411
8,3.082709550857544},posterior=0.9803398867044318,rate=0.0076055096482468
71,rate_95%_HPD={0.0047743437855159885,0.010463306495766269},rate_median=
0.007464073907581712,rate_range={0.003914633587305687,0.01345791620605015
}]:3.520965)[&height=5.887678287110725,height_95%_HPD={4.872263729572296,
7.07412326335907},height_median=5.906876690685749,height_range={4.1368640
66123962,7.691569805145264},length=1.9995607415815322,length_95%_HPD={1.1
511614322662354,3.0288474559783936},length_median=1.9880099892616272,leng
th_range={0.008238435722887516,3.797262191772461},posterior=0.76507830723
09231,rate=0.007590031143924525,rate_95%_HPD={0.005014644068932453,0.0102
5611561398444},rate_median=0.007451829904763088,rate_range={0.00403619518
6713107,0.01387231566953979}]:1.639163,((Dama_dama_dama[&height=6.2362122
75959073E-7,height_95%_HPD={0.0,1.1920928955078125E-
6},height_median=5.960464477539062E-
7,height_range={0.0,2.3245811462402344E-
6},length=4.115560907953702,length_95%_HPD={2.2365686893463135,6.84477615
3564453},length_median=3.824819326400757,length_range={1.984546422958374,
8.8663330078125},rate=0.009293007459267665,rate_95%_HPD={0.00625130306336
08024,0.0123343477949503},rate_median=0.009196678138397533,rate_range={0.
004914482078380704,0.01564750090353516}]:4.264517,Dama_eurygonos[&height=

2.6386813565193017,height_95%_HPD={1.9726578891277313,3.343396358191967},
height_median=2.6226801574230194,height_range={1.9500113725662231,3.39876
61004066467},length=1.3293543972667885,length_95%_HPD={8.494301000609994E
-
4,3.58817458152771},length_median=1.0303858518600464,length_range={8.4943
01000609994E-
4,8.669622421264648},rate=0.008462917212117905,rate_95%_HPD={0.0054214122
78348755,0.012163032701906917},rate_median=0.008224569445962433,rate_rang
e={0.004002568392174907,0.017592833555028133}]:1.625836)[&height=3.795943
3927926254,height_95%_HPD={2.2365696132183075,5.83311062645912},height_m
edian=3.619321569800377,height_range={1.9845470786094666,7.84412663802504
5},length=2.878946347918573,length_95%_HPD={0.4625248908996582,5.06022739
4104004},length_median=2.8996949195861816,length_range={1.189192989841103
6E-
4,6.514043807983398},posterior=0.8400533155614796,rate=0.0090575328391743
33,rate_95%_HPD={0.006019819077788677,0.012218369102270133},rate_median=0
.008872539863090684,rate_range={0.004688930030797844,0.01622546571385398}
]:2.453453,Megaloceros_giganteus_AM182644[&height=0.2717483931633868,heig
ht_95%_HPD={0.24229294061660767,0.29827718436717987},height_median=0.2717
5262570381165,height_range={0.2420099973678589,0.30099663883447647},lengt
h=6.110936277391751,length_95%_HPD={3.468184471130371,8.542262077331543},
length_median=6.2336273193359375,length_range={1.9095087051391602,10.0510
80703735352},rate=0.008673881789566741,rate_95%_HPD={0.005984108006764024
,0.011898816979627332},rate_median=0.008489637595250007,rate_range={0.004
370315726769648,0.017340588884472718}]:6.446222)[&height=6.54745966643990
85,height_95%_HPD={4.842783868312836,8.425430297851562},height_median=6.5
56260496377945,height_range={3.5005390644073486,9.628274205839261},length
=1.2549418559490209,length_95%_HPD={0.09522423893213272,2.43107533454895}
,length_median=1.2008858919143677,length_range={0.0018009290797635913,4.1
36171340942383},posterior=0.8267244251916028,rate=0.008566498400542697,ra
te_95%_HPD={0.005462173147179726,0.01179611493339293},rate_median=0.00844
6955960515186,rate_range={0.004131121591722676,0.01640066884791576}]:2.56
3933)[&height=7.97602453116793,height_95%_HPD={6.594090983271599,9.559737
861156464},height_median=7.965953230857849,height_range={5.68185114860534
7,11.605508387088776},length=2.0631918559729594,length_95%_HPD={1.1137074
23210144,3.112330198287964},length_median=2.064791440963745,length_range=
{0.03636801615357399,4.443547248840332},posterior=0.6901032989003666,rate
=0.007852758338244035,rate_95%_HPD={0.005358417273302604,0.01074300196786
671},rate_median=0.0077135864919264125,rate_range={0.004332041695510287,0
.014675117230275632}]:1.768534)[&height=10.141309442939885,height_95%_HPD
={8.125263273715973,12.058844462037086},height_median=10.07335657812655,h
eight_range={7.136469632387161,15.990851610898972},length=5.8754543786691
06,length_95%_HPD={3.5553059577941895,8.31005859375},length_median=5.9596
97246551514,length_range={0.13305772840976715,9.782716751098633},posterio
r=0.7837387537487505,rate=0.007100299378408812,rate_95%_HPD={0.0051114797
475600385,0.009436663943143594},rate_median=0.006966822946643371,rate_ran
ge={0.004051605976268534,0.012149507408396699}]:6.146885,((Elaphodus_ceph
alophus_N008749[&height=6.247922858216026E-
7,height_95%_HPD={0.0,1.3113021850585938E-
6},height_median=5.960464477539062E-
7,height_range={0.0,2.652406692504883E-
6},length=10.472059301518074,length_95%_HPD={3.9617056846618652,15.672436
714172363},length_median=11.27826976776123,length_range={3.22531175613403
3,17.68516731262207},rate=0.00843735454630942,rate_95%_HPD={0.00610477788
9746966,0.011062399854495614},rate_median=0.008332435458035088,rate_range
={0.004287530414814082,0.01395833626645244}]:12.81755,Cervus_ruscinensis[
&height=4.190895822243673,height_95%_HPD={3.5011012256145477,4.9012099951
50566},height_median=4.156034886837006,height_range={3.5011012256145477,4
.998523473739624},length=6.986992211302472,length_95%_HPD={1.187150992336
683E-

4,15.912616729736328},length_median=6.4611992835998535,length_range={1.18
7150992336683E-
4,28.970930099487305},rate=0.0089579380515443,rate_95%_HPD={0.00556725051
7950024,0.01243660182061277},rate_median=0.008737779845001317,rate_range=
{0.004020335900498515,0.017069023482657146}]:8.626655)[&height=7.87748314
1869967,height_95%_HPD={3.7168329656124115,13.110656931996346},height_med
ian=7.320184409618378,height_range={3.5426772832870483,15.874403581023216
},length=6.001068608628967,length_95%_HPD={0.42891407012939453,10.8320875
16784668},length_median=6.121089458465576,length_range={0.003008091589435
935,13.20677661895752},posterior=0.5131622792402533,rate=0.00872717832057
9105,rate_95%_HPD={0.005782590676364952,0.011930156505885334},rate_median
=0.008501129873937262,rate_range={0.004540261897372661,0.0165847892499979
8}]:3.109104,((Muntiacus_crinifrons_NC004577[&height=6.263035514356425E-
7,height_95%_HPD={0.0,1.2516975402832031E-
6},height_median=5.960464477539062E-
7,height_range={0.0,2.6635825634002686E-
6},length=4.1033890913900075,length_95%_HPD={3.0730292797088623,5.2986884
117126465},length_median=4.1080474853515625,length_range={2.5469884872436
523,6.09855842590332},rate=0.008476360217587593,rate_95%_HPD={0.006310907
047736976,0.011008131534352603},rate_median=0.00832782508285272,rate_rang
e={0.005360983703574331,0.014220475206219142}]:4.115801,Muntiacus_muntjak
_NC_004563[&height=6.26567105483124E-
7,height_95%_HPD={0.0,1.259148120880127E-
6},height_median=5.960464477539062E-
7,height_range={0.0,2.6635825634002686E-
6},length=4.107589169606809,length_95%_HPD={3.059515953063965,5.264712810
516357},length_median=4.1080474853515625,length_range={2.5469884872436523
,6.09855842590332},rate=0.00980839475648695,rate_95%_HPD={0.0074218610545
49713,0.012828888520422405},rate_median=0.009625967037912969,rate_range={
0.006038862309695865,0.01610819712042196}]:4.115801)[&height=4.1202649598
49722,height_95%_HPD={3.0621763058006763,5.243072994053364},height_median
=4.117502581328154,height_range={2.5469887256622314,6.098559141159058},le
ngth=1.4518264491994461,length_95%_HPD={0.8393861055374146,2.252097129821
7773},length_median=1.4218288660049438,length_range={0.006848551332950592
,2.8584651947021484},posterior=0.9653448850383206,rate=0.0086881516797742
09,rate_95%_HPD={0.0057484879345353936,0.011905916247915219},rate_median=
0.008556849699891156,rate_range={0.004256984404737523,0.01525679156224866
6}]:1.467886,(Muntiacus_reevesi_NC008491[&height=6.317118791059083E-
7,height_95%_HPD={0.0,1.2516975402832031E-
6},height_median=5.960464477539062E-
7,height_range={0.0,2.78279185295105E-
6},length=4.9246874541213055,length_95%_HPD={3.5635101795196533,6.3818740
84472656},length_median=4.9227190017700195,length_range={2.61012196540832
5,7.120263576507568},rate=0.007703658848121864,rate_95%_HPD={0.0055686177
65726763,0.009778847235044281},rate_median=0.0075705259110108464,rate_ran
ge={0.004907771128717793,0.013007622990242664}]:4.969445,Muntiacus_vuquan
gensis[&height=6.303370455767952E-
7,height_95%_HPD={0.0,1.255422830581665E-
6},height_median=5.960464477539062E-
7,height_range={0.0,2.78279185295105E-
6},length=4.9250242140483,length_95%_HPD={3.56742787361145,6.476093292236
328},length_median=4.924849510192871,length_range={2.607113838195801,7.12
0263576507568},rate=0.007891928050926322,rate_95%_HPD={0.0057727243680942
66,0.010100923432534077},rate_median=0.007796108954410166,rate_range={0.0
05088575043449394,0.012845734771713026}]:4.969445)[&height=4.983719275799
9995,height_95%_HPD={3.693301200866699,6.2281287014484406},height_median=
4.959811743348837,height_range={3.045691967010498,7.120264302939177},leng
th=0.6296118861810478,length_95%_HPD={0.1719183325767517,1.12199175357818
6},length_median=0.6085394024848938,length_range={0.008806493133306503,1.
9441077709197998},posterior=0.8983672109296901,rate=0.008103227289935407,

rate_95%_HPD={0.005046475520685253,0.011290904384758754},rate_median=0.00
7927265087839305,rate_range={0.004114979422155239,0.01851720045505872}]:0
.614241)[&height=5.669137369623495,height_95%_HPD={4.434058975428343,6.98
85283857584},height_median=5.689498379826546,height_range={3.612073604948
8187,8.023730885237455},length=7.867743989371293,length_95%_HPD={3.017250
0610351562,10.93833065032959},length_median=8.216299533843994,length_rang
e={0.0037598286289721727,12.31779670715332},posterior=0.8470509830056647,
rate=0.008213996764131566,rate_95%_HPD={0.006001298991075677,0.0105654825
15285328},rate_median=0.008061114749229648,rate_range={0.0049642883032210
24,0.01579572195111302}]:10.342968)[&posterior=0.48717094301899366,rate=0
.007248738150553372,!color=#ff0000]:1.270667)[&height=15.764367596224643,
height_95%_HPD={11.875276133418083,19.008002400398254},height_median=15.9
09135580062866,height_range={10.764184653759003,21.541824877262115},lengt
h=2.7855181695050923,length_95%_HPD={0.523643195629119 9,4.859714031219482
},length_median=2.846444606781006,length_range={0.020218953490257263,6.40
8545970916748},posterior=0.7300899700099966,rate=0.00792285634764827 9,rat
e_95%_HPD={0.005184332293338834,0.01121782618846438},rate_median=0.007769
732088540654,rate_range={0.004137393162566592,0.015266450615978736}]:4.82
1496,Euprox_furcatus[&height=13.683226582129079,height_95%_HPD={12.502662
420272827,15.001313179731369},height_median=13.595493197441101,height_ran
ge={12.500477969646454,15.19966672360897},length=7.010408158116452,length
_95%_HPD={0.41104015707969666,15.182784080505371},length_median=6.1860399
24621582,length_range={0.025349240750074387,20.411664962768555},rate=0.00
8958737729024634,rate_95%_HPD={0.005542482094134211,0.012629665718191711}
,rate_median=0.008711596588046093,rate_range={0.0038790266463455472,0.018
77177545074372}]:8.335591)[&posterior=0.3078973675441519,rate=0.008176634
88383876,!color=#ff0000]:0.810966)[&height=20.776394182385665,height_95%_
HPD={16.272213578224182,25.20773559808731},height_median=20.6193214058876
04,height_range={15.275856733322144,30.63388979434967},length=3.979728651
717453,length_95%_HPD={0.022652961313724518,8.629430770874023},length_med
ian=3.525653839111328,length_range={0.022652961313724518,13.3550586700439
45},posterior=0.5041652782405864,rate=0.008553799526980025,rate_95%_HPD={
0.005639630357636303,0.011932468580631045},rate_median=0.0083874348762185
08,rate_range={0.004449878027705865,0.01515865228578687}]:2.912238,(Dicro
cerus_elegans[&height=16.057370272619128,height_95%_HPD={15.2294820547103
88,16.916373416781425},height_median=16.03677448630333,height_range={15.2
00024074874818,16.999312043190002},length=5.313700979666832,length_95%_HP
D={0.015281043946743011,12.027491569519043},length_median=4.7620329856872
56,length_range={0.003593274625018239,17.501066207885742},rate=0.00869124
0074646986,rate_95%_HPD={0.005453346291526118,0.012360124267946377},rate_
median=0.008480386351456584,rate_range={0.004615216758155607,0.0166092486
59454522}]:5.684889,Eostyloceros_hezhengensis[&height=8.033291954952775,h
eight_95%_HPD={7.10384076833725,8.995205342769623},height_median=8.053827
330470085,height_range={7.000031273812056,8.999871730804443},length=11.15
847502429608,length_95%_HPD={3.1796979904174805,18.712289810180664},lengt
h_median=10.930937767028809,length_range={0.06446578353643417,25.51661491
394043},rate=0.008378938107287 81,rate_95%_HPD={0.005336814914192803,0.011
877788598404795},rate_median=0.008187622848211642,rate_range={0.003914633
587305687,0.016452651987426542}]:13.708967)[&height=19.39457078119775,hei
ght_95%_HPD={15.325478166341782,24.149130880832672},height_median=18.9722
47183322906,height_range={15.325478166341782,29.519454941153526},length=5
.094415748917193,length_95%_HPD={0.04627513512969017,11.175586700439453},
length_median=4.593052387237549,length_range={0.030577663332223892,17.944
04411315918},posterior=0.5268243918693769,rate=0.008475022509784252,rate_
95%_HPD={0.00578637676240639,0.012131129138248029},rate_median=0.00826242
2222399736,rate_range={0.00395717644893447,0.016819286128019747}]:3.99976
3)[&height=24.881028272990534,height_95%_HPD={19.356338620185852,30.66117
812693119},height_median=24.64549446105957,height_range={16.4782533347606
66,33.94686084985733},length=4.796995888938209,length_95%_HPD={0.01751506
3285827637,9.317309379577637},length_median=4.756028652191162,length_rang

e={0.002760556759312749,13.323931694030762},posterior=0.8040653115628124,
rate=0.008710593975350171,rate_95%_HPD={0.005857526923083069,0.0122908166
6428871},rate_median=0.008487007389476763,rate_range={0.00450619105764084
6,0.01669388226273478}]:4.154136,(((Hereroprox_larteti[&height=16.7569914
7892673,height_95%_HPD={15.421298623085022,17.99891296029091},height_medi
an=16.830309092998505,height_range={15.200075566768646,17.99891296029091}
,length=2.8984405392211743,length_95%_HPD={0.4133201241493225,5.170518875
12207},length_median=2.764653205871582,length_range={0.15984001755714417,
9.494277954101562},rate=0.008467878400988671,rate_95%_HPD={0.005494345447
097164,0.011796614947714229},rate_median=0.008279457906330433,rate_range=
{0.004260559961018851,0.016573293598832556}]:3.138226,Procervulus_dichoto
mus[&height=18.478234795212476,height_95%_HPD={17.063472848385572,19.8128
32221388817},height_median=18.48058795928955,height_range={17.00769773125
6485,19.99564090371132},length=1.1326984193810128,length_95%_HPD={5.71679
0910810232E-
4,2.96565318107605},length_median=0.9098553657531738,length_range={5.7167
90910810232E-
4,6.663582801818848},rate=0.008356482444518027,rate_95%_HPD={0.0052515456
63387964,0.011601592487202524},rate_median=0.008209760511685819,rate_rang
e={0.00445029804793328,0.017191225383662315}]:1.416983)[&posterior=0.3942
0193268910364,rate=0.004507410397708198,!color=#ff0000]:0.387562,Procervu
lus_praelucidus[&height=18.87330505183555,height_95%_HPD={18.010817170143
127,19.85983408242464},height_median=18.8062686920166,height_range={18.00
0287234783173,19.99857123196125},length=0.8866658876249167,length_95%_HPD
={1.1276798613835126E-
4,2.493586778640747},length_median=0.6651625633239746,length_range={1.127
6798613835126E-
4,7.101812362670898},rate=0.008400825662864998,rate_95%_HPD={0.0051936776
24432284,0.011720899754923093},rate_median=0.008217720487789626,rate_rang
e={0.00403626093028806,0.01676172430878023}]:1.409475)[&height=20.2789144
5768811,height_95%_HPD={18.344093322753906,22.3034525513649},height_media
n=20.118676238693297,height_range={18.06476590037346,26.5803941488266},le
ngth=6.7683263732447045,length_95%_HPD={1.9529798030853271,12.65939712524
414},length_median=6.596865177154541,length_range={0.19324377179145813,15
.158257484436035},posterior=0.9956681106297901,rate=0.009111487822421958,
rate_95%_HPD={0.005797809529117106,0.013096143606860216},rate_median=0.00
890588042117978,rate_range={0.00395717644893447,0.019029558908470665}]:7.
030068,Lagomeryx_parvulus[&height=18.917571459622152,height_95%_HPD={18.0
04553109407425,19.868001960217953},height_median=18.85825228691101,height
_range={18.000565379858017,19.99888837337494},length=8.11369098658881,len
gth_95%_HPD={2.8884785175323486,13.619062423706055},length_median=7.90941
6675567627,length_range={1.2758781909942627,16.64752197265625},rate=0.009
194863090562128,rate_95%_HPD={0.005878390992831844,0.013150681940378238},
rate_median=0.008921612202393294,rate_range={0.0043002364064983955,0.0188
9637007805051}]:8.395276)[&height=26.453200842278115,height_95%_HPD={21.5
09845703840256,31.323611825704575},height_median=26.30029994249344,height
_range={19.42029893398285,33.95524537563324},length=3.5920392768469243,le
ngth_95%_HPD={0.0029539386741816998,7.86846923828125},length_median=3.339
1106128692627,length_range={0.0029539386741816998,11.691393852233887},pos
terior=0.6624458513828724,rate=0.008860160744189986,rate_95%_HPD={0.00563
4679022266903,0.012429298068077797},rate_median=0.008630674799068544,rate
_range={0.004701037926273471,0.017780934677125937}]:2.58331)[&height=29.8
96157833524917,height_95%_HPD={24.924467265605927,34.66712172329426},heig
ht_median=30.168813824653625,height_range={20.9578601680696,34.8281816593
3713},length=0.9177196521829375,length_95%_HPD={7.913250738056377E-
5,2.6938953399658203},length_median=0.6614949703216553,length_range={7.91
3250738056377E-
5,6.278171062469482},posterior=1.0,rate=0.008501383840700375,rate_95%_HPD
={0.005322397385254121,0.011760521638067943},rate_median=0.00833274991487
8515,rate_range={0.003927668164749973,0.01666079031510433},!rotate=true]:

1.103342,((Antilocapra_americana[&height=6.275881252679676E-
7,height_95%_HPD={0.0,1.5795230865478516E-
6},height_median=5.438923835754395E-
7,height_range={0.0,2.816319465637207E-
6},length=25.236296758616778,length_95%_HPD={19.49710464477539,31.5048389
43481445},length_median=25.18645477294922,length_range={15.18188953399658
2,34.85188674926758},rate=0.010188061378339521,rate_95%_HPD={0.0076688186
40236864,0.012644089509272559},rate_median=0.01004272272257694,rate_range
={0.0071113167051442,0.01676172430878023}]:25.236673,Giraffa_camelopardal
is_angolensis_NC012100[&height=6.266955948699023E-
7,height_95%_HPD={0.0,1.5795230865478516E-
6},height_median=5.401670932769775E-
7,height_range={0.0,2.6421621441841125E-
6},length=25.2365122426156,length_95%_HPD={19.49710464477539,31.504838943
481445},length_median=25.18645477294922,length_range={15.181889533996582,
34.932682037353516},rate=0.008676835493914013,rate_95%_HPD={0.00670096227
534455,0.011060020393417271},rate_median=0.008557964535359123,rate_range=
{0.006048612586915903,0.014352827351483717}]:25.236673)[&height=25.219042
7382213,height_95%_HPD={19.648996353149414,31.504839539527893},height_med
ian=25.178624212741852,height_range={15.181890338659286,34.85188939142972
},length=3.868801735046252,length_95%_HPD={1.8996480703353882,6.071964263
916016},length_median=3.7752525806427,length_range={1.1823267414001748E-
4,8.292577743530273},posterior=0.9980006664445185,rate=0.0084618040200165
5,rate_95%_HPD={0.005594714774538437,0.011782206557745716},rate_median=0.
00829434037644365,rate_range={0.004290186172385258,0.015363221938228748}]
:3.940537,(Moschus_moschiferus[&height=6.266170685942651E-
7,height_95%_HPD={0.0,1.5795230865478516E-
6},height_median=5.438923835754395E-
7,height_range={0.0,2.5331974029541016E-
6},length=26.374696572356843,length_95%_HPD={21.212656021118164,32.582523
345947266},length_median=26.51662826538086,length_range={15.2051534652709
96,34.20582962036133},rate=0.006697374043043748,rate_95%_HPD={0.005223120
7645809154,0.008478844922440889},rate_median=0.006563223204379957,rate_ra
nge={0.004842772189106313,0.011359660868169778}]:26.381418,Ovis_aries_NC0
01941[&height=6.266009310488203E-
7,height_95%_HPD={0.0,1.5795230865478516E-
6},height_median=5.438923835754395E-
7,height_range={0.0,3.0994415283203125E-
6},length=26.380559453802164,length_95%_HPD={20.840375900268555,32.341106
41479492},length_median=26.51662826538086,length_range={15.20515346527099
6,34.700714111328125},rate=0.009325590963877826,rate_95%_HPD={0.007255826
502291573,0.011802966744599906},rate_median=0.009167686980552339,rate_ran
ge={0.006255426209383347,0.01628807267836834}]:26.381418)[&height=26.3440
1853206505,height_95%_HPD={21.221687391400337,32.56534433364868},height_m
edian=26.484943389892578,height_range={15.205154336988926,34.205831446684
9},length=2.7095015396571305,length_95%_HPD={1.0860764980316162,4.6774358
74938965},length_median=2.6297647953033447,length_range={0.01033725496381
5212,6.939298152923584},posterior=0.9926691102965678,rate=0.0080947746748
98145,rate_95%_HPD={0.00511139281046749,0.0114255860218631},rate_median=0
.007921693596133912,rate_range={0.004383250752083582,0.016511987513906864
}]:2.795791)[&height=28.57075615691008,height_95%_HPD={23.135632276535034
,34.367544651031494},height_median=28.715479336678982,height_range={18.66
1694526672363,34.77469253540039},length=2.331071075832343,length_95%_HPD=
{0.01967843435704708,4.678918838500977},length_median=2.2209771871566772,
length_range={1.7454533372074366E-
4,8.413496017456055},posterior=0.7817394201932689,rate=0.0081441012876287
48,rate_95%_HPD={0.004938832035513719,0.011093839231073864},rate_median=0
.007972361396206697,rate_range={0.004257847686942377 5,0.0178709700290633 1
8},!rotate=true]:1.82229)[&height=30.99950030637331,height_95%_HPD={25.71
8061700463295,34.993153899908066},height_median=31.363483667373657,height

```
_range={21.912464022636414,34.99877715110779},length=0.0,posterior=1.0,ra
te=1.0,!rotate=true];
end;

begin figtree;
	set appearance.backgroundColorAttribute="Default";
	set appearance.backgroundColour=#ffffff;
	set appearance.branchColorAttribute="User selection";
	set appearance.branchColorGradient=false;
	set appearance.branchLineWidth=2.0;
	set appearance.branchMinLineWidth=0.0;
	set appearance.branchWidthAttribute="Fixed";
	set appearance.foregroundColour=#000000;
	set appearance.hilightingGradient=true;
	set appearance.selectionColour=#2d3680;
	set branchLabels.colorAttribute="User selection";
	set branchLabels.displayAttribute="posterior";
	set branchLabels.fontName="Helvetica";
	set branchLabels.fontSize=9;
	set branchLabels.fontStyle=0;
	set branchLabels.isShown=true;
	set branchLabels.significantDigits=2;
	set layout.expansion=0;
	set layout.layoutType="RECTILINEAR";
	set layout.zoom=0;
	set legend.attribute="height";
	set legend.fontSize=10.0;
	set legend.isShown=false;
	set legend.significantDigits=4;
	set nodeBars.barWidth=4.0;
	set nodeBars.displayAttribute="height_95%_HPD";
	set nodeBars.isShown=true;
	set nodeLabels.colorAttribute="User selection";
	set nodeLabels.displayAttribute="height_95%_HPD";
	set nodeLabels.fontName="Abadi MT Condensed Extra Bold";
	set nodeLabels.fontSize=11;
	set nodeLabels.fontStyle=0;
	set nodeLabels.isShown=false;
	set nodeLabels.significantDigits=2;
	set nodeShape.colourAttribute="User selection";
	set nodeShape.isShown=false;
	set nodeShape.minSize=10.0;
	set nodeShape.scaleType=Width;
	set nodeShape.shapeType=Circle;
	set nodeShape.size=4.0;
	set nodeShape.sizeAttribute="Fixed";
	set polarLayout.alignTipLabels=false;
	set polarLayout.angularRange=0;
	set polarLayout.rootAngle=0;
	set polarLayout.rootLength=100;
	set polarLayout.showRoot=true;
	set radialLayout.spread=0.0;
	set rectilinearLayout.alignTipLabels=false;
	set rectilinearLayout.curvature=0;
	set rectilinearLayout.rootLength=100;
	set scale.offsetAge=0.0;
	set scale.rootAge=1.0;
	set scale.scaleFactor=1.0;
	set scale.scaleRoot=false;
	set scaleAxis.automaticScale=true;
```

```
	set scaleAxis.fontSize=10.0;
	set scaleAxis.isShown=true;
	set scaleAxis.lineWidth=1.0;
	set scaleAxis.majorTicks=5.0;
	set scaleAxis.origin=0.0;
	set scaleAxis.reverseAxis=true;
	set scaleAxis.showGrid=false;
	set scaleBar.automaticScale=true;
	set scaleBar.fontSize=11.0;
	set scaleBar.isShown=false;
	set scaleBar.lineWidth=1.0;
	set scaleBar.scaleRange=4.0;
	set tipLabels.colorAttribute="User selection";
	set tipLabels.displayAttribute="Names";
	set tipLabels.fontName="Helvetica";
	set tipLabels.fontSize=10;
	set tipLabels.fontStyle=0;
	set tipLabels.isShown=true;
	set tipLabels.significantDigits=4;
	set trees.order=false;
	set trees.orderType="increasing";
	set trees.rooting=false;
	set trees.rootingType="User Selection";
	set trees.transform=false;
	set trees.transformType="cladogram";
end;
```
