# Supplementary material for: Bony labyrinth morphology clarifies the origin and evolution of deer
Source: Sci Rep. 2017 Oct 13;7:13176. doi: 10.1038/s41598-017-12848-9 (PMC5640792; doi:10.1038/s41598-017-12848-9)
Supplement: Supplementary file 2 — Dataset 1-5 [file dataset2.pdf › dataset2.pdf]

New Procrustes fit:
Dataset 'sans Heteroprox Sansan'

686 landmarks in 3 dimensions.
The dataset contains 40 observations, of which 39 are included for analyses.

Average shape:

Lmk.	Axis 1 (x)	Axis 2 (y)	Axis 3 (z)
1	-0.04018963	0.02601720	-0.00829446
2	-0.02472170	-0.00999658	-0.02626000
3	-0.02620229	-0.01155833	-0.02731205
4	-0.02811814	-0.01241408	-0.02847990
5	-0.03012956	-0.01277627	-0.02952987
6	-0.03217640	-0.01277808	-0.03048387
7	-0.03431093	-0.01250291	-0.03135141
8	-0.03658549	-0.01181548	-0.03217753
9	-0.03900781	-0.01077522	-0.03292248
10	-0.04148604	-0.00921988	-0.03357816
11	-0.04396528	-0.00715217	-0.03410467
12	-0.04660935	-0.00426948	-0.03454398
13	-0.04862549	-0.00118795	-0.03456864
14	-0.05012741	0.00187721	-0.03424789
15	-0.05113588	0.00488964	-0.03354687
16	-0.05177938	0.00785586	-0.03256681
17	-0.05200051	0.01081657	-0.03123153
18	-0.05178507	0.01377696	-0.02954892
19	-0.05105413	0.01674095	-0.02750532
20	-0.04964714	0.01956767	-0.02514332
21	-0.04760274	0.02178350	-0.02203152
22	-0.04516068	0.02320381	-0.01917451
23	-0.04256102	0.02392190	-0.01655579
24	-0.03988260	0.02400733	-0.01414844
25	-0.03715229	0.02351700	-0.01185803
26	-0.03416126	0.02234169	-0.00982951
27	-0.03096158	0.02038076	-0.00812308
28	-0.02761379	0.01781314	-0.00654688
29	-0.02414981	0.01453542	-0.00530847
30	-0.02249807	0.01287936	-0.00500238
31	-0.02110586	0.01123495	-0.00508588
32	-0.02003923	0.00960955	-0.00558048
33	-0.01926827	0.00801410	-0.00644813
34	-0.01878711	0.00644363	-0.00768291
35	-0.01856216	0.00487914	-0.00926214
36	-0.01854807	0.00330400	-0.01117362
37	-0.01874521	0.00163716	-0.01342760
38	-0.01941107	0.00021176	-0.01609276
39	-0.02043534	-0.00017249	-0.01768853
40	-0.02161636	-0.00042991	-0.01922863
41	-0.02260442	-0.00098427	-0.02064226
42	-0.02339109	-0.00182504	-0.02190453
43	-0.02396115	-0.00295892	-0.02301504
44	-0.02436440	-0.00436722	-0.02401182
45	-0.02451661	-0.00609195	-0.02483906
46	-0.02450455	-0.00806962	-0.02549565
47	-0.01959915	-0.01434425	-0.02646535
48	-0.02203034	-0.01540425	-0.02780162

49	-0.02450550	-0.01611570	-0.02902604
50	-0.02697682	-0.01648274	-0.03011422
51	-0.02940651	-0.01656161	-0.03109227
52	-0.03176766	-0.01637886	-0.03200177
53	-0.03411014	-0.01591473	-0.03280222
54	-0.03641387	-0.01519923	-0.03354178
55	-0.03869333	-0.01423132	-0.03422925
56	-0.04093101	-0.01293751	-0.03478304
57	-0.04236787	-0.01070290	-0.03463774
58	-0.04490974	-0.00979536	-0.03571985
59	-0.04662105	-0.00806140	-0.03597865
60	-0.04819371	-0.00620147	-0.03614272
61	-0.04963499	-0.00425950	-0.03623319
62	-0.05087398	-0.00216837	-0.03614123
63	-0.05193311	0.00003467	-0.03593389
64	-0.05280371	0.00237345	-0.03554049
65	-0.05354148	0.00480469	-0.03506106
66	-0.05403572	0.00698906	-0.03439476
67	-0.05432676	0.00910558	-0.03363023
68	-0.05444526	0.01116539	-0.03276806
69	-0.05437205	0.01316142	-0.03178395
70	-0.05411853	0.01509059	-0.03070653
71	-0.05374276	0.01701393	-0.02956298
72	-0.05310627	0.01881712	-0.02823965
73	-0.05228817	0.02056659	-0.02683016
74	-0.05128648	0.02223594	-0.02531429
75	-0.04995436	0.02369198	-0.02379582
76	-0.04850992	0.02496249	-0.02216691
77	-0.04696153	0.02601570	-0.02041757
78	-0.04530372	0.02685566	-0.01853381
79	-0.04353813	0.02745372	-0.01653382
80	-0.04165074	0.02782277	-0.01440027
81	-0.03961546	0.02796121	-0.01205367
82	-0.03754169	0.02798994	-0.00971105
83	-0.03534537	0.02814002	-0.00728795
84	-0.02477122	0.00501421	0.02175783
85	-0.02667659	0.00349954	0.02309360
86	-0.02898467	0.00253263	0.02438014
87	-0.03130615	0.00187708	0.02544039
88	-0.03356350	0.00135654	0.02625373
89	-0.03579678	0.00101411	0.02688807
90	-0.03802920	0.00084138	0.02733142
91	-0.04026841	0.00080832	0.02762892
92	-0.04252573	0.00100666	0.02775544
93	-0.04477956	0.00139447	0.02768438
94	-0.04741396	0.00206035	0.02717218
95	-0.04975249	0.00299031	0.02637406
96	-0.05179761	0.00410714	0.02531047
97	-0.05350126	0.00545169	0.02399500
98	-0.05498748	0.00688826	0.02247194
99	-0.05623695	0.00850166	0.02072107
100	-0.05723106	0.01026201	0.01871871
101	-0.05794933	0.01222676	0.01648175
102	-0.05830295	0.01437178	0.01403204
103	-0.05819068	0.01697023	0.01097654
104	-0.05746601	0.01922287	0.00821768
105	-0.05618542	0.02114788	0.00583436
106	-0.05449687	0.02269715	0.00374637
107	-0.05238070	0.02388083	0.00194189
108	-0.04988131	0.02471511	0.00029192

109	-0.04686551	0.02522558	-0.00103194
110	-0.04335035	0.02534782	-0.00203398
111	-0.03936672	0.02512439	-0.00240349
112	-0.03568140	0.02344320	-0.00192029
113	-0.03239093	0.02183422	-0.00094125
114	-0.02946633	0.02033347	0.00015911
115	-0.02690823	0.01888570	0.00122617
116	-0.02469640	0.01746833	0.00234308
117	-0.02277672	0.01605808	0.00361756
118	-0.02116874	0.01462996	0.00520422
119	-0.02006882	0.01338846	0.00726017
120	-0.01985721	0.01284631	0.00941836
121	-0.02054360	0.01293056	0.01090969
122	-0.02148602	0.01308848	0.01237953
123	-0.02233034	0.01290668	0.01382455
124	-0.02304694	0.01239416	0.01524598
125	-0.02356847	0.01140780	0.01661810
126	-0.02397743	0.01020563	0.01793412
127	-0.02423397	0.00872204	0.01919526
128	-0.02429063	0.00676117	0.02041495
129	-0.01885969	0.00314389	0.02391888
130	-0.02104108	0.00226817	0.02519060
131	-0.02341246	0.00153074	0.02614263
132	-0.02575832	0.00088606	0.02701368
133	-0.02808297	0.00032440	0.02778260
134	-0.03038464	-0.00014352	0.02846918
135	-0.03268559	-0.00052604	0.02907834
136	-0.03499356	-0.00081493	0.02960584
137	-0.03731371	-0.00101071	0.02999346
138	-0.03962365	-0.00110886	0.03026721
139	-0.04197812	-0.00094765	0.03036993
140	-0.04421380	-0.00067296	0.03037541
141	-0.04631982	-0.00023392	0.03010544
142	-0.04830681	0.00029733	0.02967585
143	-0.05016104	0.00095470	0.02904829
144	-0.05188724	0.00176887	0.02822964
145	-0.05352021	0.00270351	0.02726008
146	-0.05502212	0.00377299	0.02608761
147	-0.05640705	0.00497564	0.02475214
148	-0.05785087	0.00654345	0.02305293
149	-0.05900423	0.00816003	0.02127774
150	-0.05989906	0.00981781	0.01943951
151	-0.06058246	0.01151603	0.01756529
152	-0.06101905	0.01326128	0.01563628
153	-0.06118744	0.01501681	0.01361603
154	-0.06108291	0.01679481	0.01152064
155	-0.06068393	0.01859898	0.00933955
156	-0.05993978	0.02039747	0.00709937
157	-0.05858836	0.02248393	0.00480411
158	-0.05682784	0.02420455	0.00271012
159	-0.05475221	0.02564445	0.00081361
160	-0.05242635	0.02685797	-0.00099254
161	-0.04970792	0.02771483	-0.00258681
162	-0.04665760	0.02828727	-0.00401703
163	-0.04322613	0.02850737	-0.00526731
164	-0.03940909	0.02854701	-0.00639373
165	-0.03534537	0.02814002	-0.00728795
166	-0.02494121	-0.01798474	-0.01138842
167	-0.02588848	-0.01926227	-0.01126242
168	-0.02711900	-0.02031355	-0.01064669

169	-0.02832017	-0.02121365	-0.00981865
170	-0.02948340	-0.02203217	-0.00879377
171	-0.03068286	-0.02271531	-0.00753556
172	-0.03188657	-0.02337081	-0.00603646
173	-0.03310631	-0.02391995	-0.00428553
174	-0.03431150	-0.02438346	-0.00230654
175	-0.03558327	-0.02463352	-0.00008365
176	-0.03648799	-0.02469937	0.00153409
177	-0.03733255	-0.02462610	0.00313692
178	-0.03819142	-0.02310792	0.00453986
179	-0.03881625	-0.02402984	0.00623687
180	-0.03944035	-0.02353381	0.00773684
181	-0.03999985	-0.02290194	0.00921917
182	-0.04047310	-0.02213859	0.01067590
183	-0.04088644	-0.02124164	0.01211193
184	-0.04122585	-0.02018559	0.01351244
185	-0.04145849	-0.01886225	0.01506942
186	-0.04151183	-0.01749291	0.01643501
187	-0.04140181	-0.01613745	0.01769425
188	-0.04106203	-0.01470694	0.01859130
189	-0.04056915	-0.01326832	0.01942298
190	-0.03987577	-0.01177842	0.01996346
191	-0.03897836	-0.01023811	0.02028442
192	-0.03789444	-0.00867503	0.02042377
193	-0.03661804	-0.00706379	0.02031977
194	-0.03466588	-0.00497949	0.01996338
195	-0.03282407	-0.00310521	0.01930858
196	-0.03107059	-0.00151388	0.01833047
197	-0.02941863	-0.00013073	0.01712388
198	-0.02789130	0.00105256	0.01569184
199	-0.02646131	0.00206351	0.01401534
200	-0.02512690	0.00276490	0.01205206
201	-0.02381589	0.00337729	0.00987999
202	-0.02269879	0.00370270	0.00744825
203	-0.02202019	0.00349807	0.00550297
204	-0.02153429	0.00299764	0.00357395
205	-0.02115350	0.00236035	0.00178138
206	-0.02079581	0.00157957	0.00012055
207	-0.02042041	0.00063391	-0.00142894
208	-0.02007801	-0.00051027	-0.00293243
209	-0.01980198	-0.00184800	-0.00437470
210	-0.01975239	-0.00338144	-0.00573196
211	-0.02018054	-0.00499496	-0.00669700
212	-0.02095845	-0.00647661	-0.00678867
213	-0.02190582	-0.00794519	-0.00678787
214	-0.02280858	-0.00940541	-0.00690954
215	-0.02350233	-0.01085804	-0.00724475
216	-0.02398389	-0.01228248	-0.00779858
217	-0.02427575	-0.01371074	-0.00857149
218	-0.02441041	-0.01516560	-0.00955721
219	-0.02447797	-0.01666188	-0.01066949
220	-0.02285841	-0.01973534	-0.01645663
221	-0.02430683	-0.02092568	-0.01573087
222	-0.02570480	-0.02199643	-0.01483342
223	-0.02704311	-0.02297848	-0.01377707
224	-0.02834269	-0.02387529	-0.01260806
225	-0.02956792	-0.02465570	-0.01127936
226	-0.03078016	-0.02534568	-0.00985919
227	-0.03194503	-0.02597478	-0.00832200
228	-0.03305856	-0.02651586	-0.00664731

229	-0.03415564	-0.02700414	-0.00486570
230	-0.03521425	-0.02734381	-0.00296159
231	-0.03626115	-0.02758234	-0.00111916
232	-0.03724068	-0.02763811	0.00071587
233	-0.03817704	-0.02755426	0.00251107
234	-0.03901363	-0.02728116	0.00430976
235	-0.03981779	-0.02685779	0.00607423
236	-0.04054536	-0.02623723	0.00783544
237	-0.04122838	-0.02549029	0.00956499
238	-0.04183719	-0.02454831	0.01127890
239	-0.04244994	-0.02335779	0.01315399
240	-0.04294801	-0.02213114	0.01486890
241	-0.04326264	-0.02084483	0.01641766
242	-0.04338431	-0.01948222	0.01782565
243	-0.04333736	-0.01808134	0.01905619
244	-0.04313404	-0.01662441	0.02014900
245	-0.04278623	-0.01513624	0.02109136
246	-0.04222833	-0.01357153	0.02185844
247	-0.04153735	-0.01197167	0.02248995
248	-0.04049102	-0.01006778	0.02305702
249	-0.03928938	-0.00820488	0.02330860
250	-0.03796771	-0.00636435	0.02328537
251	-0.03651065	-0.00456827	0.02297350
252	-0.03495301	-0.00280012	0.02239983
253	-0.03334204	-0.00095167	0.02166066
254	-0.03139691	0.00061820	0.02040948
255	-0.02935971	0.00222956	0.01895549
256	-0.02746479	0.00399514	0.01735474
257	-0.01429166	-0.00892709	0.00093796
258	-0.01372105	-0.00918555	0.00159076
259	-0.01306167	-0.00945976	0.00213038
260	-0.01227741	-0.00964513	0.00249955
261	-0.01144131	-0.00979170	0.00277368
262	-0.01062993	-0.01003973	0.00289484
263	-0.00970603	-0.01032878	0.00286477
264	-0.00876750	-0.01065235	0.00279203
265	-0.00761806	-0.01087577	0.00302877
266	-0.00665465	-0.01135229	0.00210841
267	-0.00540507	-0.01142825	0.00164206
268	-0.00430202	-0.01158409	0.00115997
269	-0.00339268	-0.01168110	0.00058476
270	-0.00262580	-0.01178412	-0.00006785
271	-0.00201977	-0.01194026	-0.00078587
272	-0.00154390	-0.01208635	-0.00162097
273	-0.00120083	-0.01213311	-0.00256951
274	-0.00106519	-0.01216578	-0.00360206
275	-0.00113088	-0.01214604	-0.00468272
276	-0.00183850	-0.01241420	-0.00551416
277	-0.00265959	-0.01253313	-0.00617685
278	-0.00351355	-0.01258494	-0.00667530
279	-0.00442310	-0.01261656	-0.00697135
280	-0.00536353	-0.01261173	-0.00711088
281	-0.00637683	-0.01255378	-0.00709473
282	-0.00739726	-0.01245893	-0.00687095
283	-0.00853912	-0.01241156	-0.00651426
284	-0.00966257	-0.01228291	-0.00599712
285	-0.01069452	-0.01193965	-0.00545986
286	-0.01164412	-0.01157672	-0.00485375
287	-0.01241503	-0.01121411	-0.00418658
288	-0.01307004	-0.01084595	-0.00348389

289	-0.01359696	-0.01050445	-0.00272737
290	-0.01399599	-0.01014402	-0.00189735
291	-0.01425261	-0.00980371	-0.00099343
292	-0.01433420	-0.00946224	-0.00003466
293	0.02607512	0.01938346	0.01600332
294	0.02834744	0.01822344	0.01833614
295	0.03017497	0.01717915	0.01999493
296	0.03275087	0.01475370	0.02154200
297	0.03368364	0.01323131	0.02151613
298	0.03451224	0.01140859	0.02101029
299	0.03500645	0.00913816	0.01970761
300	0.03724933	0.01685585	-0.01064848
301	0.03936587	0.01619731	-0.01094942
302	0.04107947	0.01550309	-0.01112910
303	0.04226804	0.01456862	-0.01106126
304	0.04353667	0.01233647	-0.01049340
305	0.04376146	0.01096717	-0.00999853
306	0.04359829	0.00934796	-0.00929014
307	0.02062679	0.00407936	-0.01859154
308	0.02112305	0.00241424	-0.01984352
309	0.02173305	0.00095088	-0.02054481
310	0.02239731	-0.00027687	-0.02081284
311	0.02391575	-0.00224069	-0.02027915
312	0.02481930	-0.00310748	-0.01953625
313	0.02586741	-0.00390432	-0.01838664
314	0.03194738	0.00458940	0.01063055
315	0.03350722	0.00345668	0.01182814
316	0.03463386	0.00240630	0.01255414
317	0.03547280	0.00142540	0.01287432
318	0.03652043	-0.00056679	0.01247252
319	0.03678731	-0.00169530	0.01176599
320	0.03688746	-0.00296556	0.01067397
321	0.03842241	0.00597501	-0.00649350
322	0.04085759	0.00446792	-0.00693500
323	0.04241856	0.00367909	-0.00737104
324	0.04364790	0.00191390	-0.00723066
325	0.04372900	0.00099346	-0.00694657
326	0.04358837	-0.00005349	-0.00651792
327	0.04313832	-0.00133030	-0.00589201
328	0.02821793	-0.00279703	-0.01138605
329	0.02850622	-0.00447040	-0.01309749
330	0.02903410	-0.00588910	-0.01393952
331	0.03015524	-0.00776267	-0.01422129
332	0.03077867	-0.00843919	-0.01382350
333	0.03150929	-0.00906261	-0.01311884
334	0.03236337	-0.00960814	-0.01196273
335	-0.01196402	-0.00142648	0.01798819
336	-0.01007625	-0.00036248	0.01592389
337	-0.00776172	0.00111692	0.01321134
338	-0.00489105	0.00171477	0.01196008
339	-0.00181373	0.00138293	0.01342961
340	0.00058083	0.00232851	0.01288081
341	0.00301124	0.00351860	0.01240645
342	0.00538578	0.00501497	0.01184315
343	0.00772142	0.00673054	0.01142565
344	0.01005228	0.00845388	0.01151546
345	0.01167134	0.00913039	0.01157257
346	0.01324762	0.00984822	0.01153165
347	0.01476617	0.01056040	0.01140772
348	0.01623669	0.01120813	0.01117598

349	0.01765408	0.01184123	0.01082082
350	0.01903618	0.01248771	0.01039468
351	0.02040121	0.01310322	0.00986697
352	0.02169263	0.01375376	0.00914541
353	0.02291945	0.01438649	0.00833395
354	0.02500460	0.01497153	0.00677076
355	0.02678813	0.01537011	0.00511429
356	0.02829795	0.01559345	0.00340809
357	0.02950922	0.01561380	0.00164192
358	0.03046405	0.01544792	-0.00012532
359	0.03122907	0.01511476	-0.00190708
360	0.03175733	0.01456402	-0.00371414
361	0.03193065	0.01370633	-0.00550345
362	0.03181056	0.01294277	-0.00754175
363	0.03125124	0.01181883	-0.00913668
364	0.03054134	0.01119747	-0.01047894
365	0.02968510	0.01028220	-0.01152446
366	0.02868806	0.00944304	-0.01225418
367	0.02760510	0.00859882	-0.01271252
368	0.02642144	0.00773595	-0.01294269
369	0.02513701	0.00682407	-0.01292880
370	0.02373266	0.00589793	-0.01265579
371	0.02218479	0.00508690	-0.01199337
372	0.02054281	0.00327916	-0.00980650
373	0.01926654	0.00239096	-0.00763586
374	0.01859071	0.00158572	-0.00555386
375	0.01839114	0.00115669	-0.00363508
376	0.01872837	0.00065460	-0.00181414
377	0.01965920	0.00010041	-0.00002582
378	0.02098082	-0.00023529	0.00177226
379	0.02322905	-0.00028163	0.00347442
380	0.02539327	0.00118926	0.00436503
381	0.02815888	0.00122741	0.00410622
382	0.02975832	0.00216646	0.00349958
383	0.03106713	0.00268133	0.00271276
384	0.03187629	0.00291793	0.00175404
385	0.03243700	0.00284067	0.00074886
386	0.03284852	0.00250826	-0.00028830
387	0.03319213	0.00176307	-0.00155578
388	0.03271270	0.00105301	-0.00313848
389	0.03122742	0.00063040	-0.00428593
390	0.03060301	-0.00063412	-0.00428351
391	0.02975173	-0.00158303	-0.00376001
392	0.02925480	-0.00188506	-0.00318083
393	0.02944181	-0.00228421	-0.00280118
394	0.03000982	-0.00284093	-0.00236000
395	0.03114435	-0.00385478	-0.00201845
396	0.03261377	-0.00496263	-0.00166812
397	0.03553424	-0.00584550	-0.00132215
398	0.00189134	0.01397731	0.01979273
399	0.00413514	0.01337079	0.01966598
400	0.00661825	0.01336651	0.01948902
401	0.00906578	0.01363383	0.01912299
402	0.01144045	0.01418048	0.01859591
403	0.01381234	0.01485489	0.01785819
404	0.01622853	0.01575907	0.01688643
405	0.01867679	0.01683836	0.01574801
406	0.02120794	0.01799273	0.01445089
407	0.02386989	0.01913620	0.01297916
408	0.02635737	0.02001029	0.01070581

409	0.02854943	0.02055482	0.00836929
410	0.03045723	0.02076256	0.00598275
411	0.03203013	0.02070825	0.00350756
412	0.03326030	0.02039725	0.00100052
413	0.03416414	0.01985796	-0.00161086
414	0.03474208	0.01906199	-0.00428004
415	0.03493189	0.01796991	-0.00698171
416	0.03486075	0.01678068	-0.00989740
417	0.03378154	0.01539988	-0.01207310
418	0.03256369	0.01406269	-0.01390189
419	0.03119224	0.01280433	-0.01539830
420	0.02971812	0.01154864	-0.01652530
421	0.02811385	0.01035193	-0.01730523
422	0.02638899	0.00914476	-0.01772136
423	0.02451275	0.00797617	-0.01779000
424	0.02253512	0.00677668	-0.01748031
425	0.02052902	0.00550397	-0.01678195
426	0.01774946	0.00391005	-0.01437406
427	0.01572422	0.00253004	-0.01187828
428	0.01439818	0.00137051	-0.00933582
429	0.01372226	0.00048572	-0.00674175
430	0.01364263	-0.00005019	-0.00407566
431	0.01435816	-0.00045324	-0.00134201
432	0.01578036	-0.00060202	0.00145761
433	0.01793402	-0.00054303	0.00432232
434	0.02067664	-0.00009280	0.00729206
435	0.02324703	0.00103689	0.00820576
436	0.02553776	0.00215497	0.00877426
437	0.02760020	0.00317878	0.00898049
438	0.02946269	0.00404107	0.00881711
439	0.03109365	0.00476539	0.00827320
440	0.03251118	0.00539006	0.00736228
441	0.03371380	0.00589949	0.00607666
442	0.03474005	0.00624268	0.00439952
443	0.03558481	0.00635778	0.00236851
444	0.03597460	0.00622831	0.00054200
445	0.03618660	0.00596098	-0.00114361
446	0.03623864	0.00563770	-0.00270847
447	0.03611769	0.00522050	-0.00413470
448	0.03584827	0.00469371	-0.00542291
449	0.03538852	0.00408779	-0.00657612
450	0.03474590	0.00341468	-0.00760543
451	0.03394118	0.00263557	-0.00850332
452	0.03296718	0.00176439	-0.00925269
453	0.03183877	0.00085052	-0.00965079
454	0.03075942	0.00006383	-0.00982166
455	0.02978318	-0.00069114	-0.00974626
456	0.02885536	-0.00134246	-0.00944482
457	0.02800226	-0.00194728	-0.00888468
458	0.02723146	-0.00252160	-0.00804517
459	0.02662196	-0.00312888	-0.00690382
460	0.02619934	-0.00377312	-0.00549063
461	0.02602548	-0.00449099	-0.00387478
462	0.02685781	-0.00428709	-0.00204830
463	0.02779526	-0.00431276	-0.00063495
464	0.02874517	-0.00438592	0.00045633
465	0.02976851	-0.00448682	0.00118439
466	0.03076663	-0.00442430	0.00177127
467	0.03201837	-0.00456289	0.00173564
468	0.03385362	-0.00486975	0.00114177

469	0.03569464	-0.00592021	-0.00032946
470	-0.01528484	-0.00452944	0.01266387
471	-0.01448692	-0.00619296	0.01222407
472	-0.01341474	-0.00771134	0.01173751
473	-0.01210657	-0.00900275	0.01138437
474	-0.01072574	-0.01022997	0.01129146
475	-0.00920304	-0.01132971	0.01138387
476	-0.00760060	-0.01238931	0.01168296
477	-0.00592026	-0.01343036	0.01221550
478	-0.00409477	-0.01435300	0.01291185
479	-0.00214145	-0.01515344	0.01378711
480	-0.00031542	-0.01558613	0.01486985
481	0.00141159	-0.01586157	0.01600826
482	0.00309745	-0.01590435	0.01716361
483	0.00471515	-0.01574029	0.01834268
484	0.00626197	-0.01538234	0.01956802
485	0.00775584	-0.01483860	0.02083012
486	0.00920099	-0.01402956	0.02210861
487	0.01058703	-0.01297150	0.02340746
488	0.01193385	-0.01167170	0.02466454
489	0.01316108	-0.01013355	0.02583566
490	0.01439619	-0.00850340	0.02680474
491	0.01563763	-0.00683735	0.02765451
492	0.01690563	-0.00510292	0.02826762
493	0.01815191	-0.00328540	0.02871973
494	0.01942388	-0.00139100	0.02898458
495	0.02071591	0.00059001	0.02906627
496	0.02201675	0.00264574	0.02893732
497	0.02331974	0.00477497	0.02855652
498	0.02531371	0.00775242	0.02760721
499	0.02727749	0.01039003	0.02623775
500	0.02923639	0.01266041	0.02451154
501	0.03121388	0.01459151	0.02249503
502	0.03322536	0.01621768	0.02018019
503	0.03526407	0.01750716	0.01751069
504	0.03732698	0.01845619	0.01446825
505	0.03928773	0.01896784	0.01098015
506	0.04114650	0.01901687	0.00710007
507	0.04274754	0.01848692	0.00243043
508	0.04355914	0.01751695	-0.00197945
509	0.04361316	0.01624169	-0.00593516
510	0.04314554	0.01482514	-0.00941049
511	0.04215660	0.01327028	-0.01243297
512	0.04076283	0.01163182	-0.01511062
513	0.03892735	0.00988445	-0.01748241
514	0.03656610	0.00796995	-0.01950200
515	0.03362571	0.00587371	-0.02103518
516	0.03099868	0.00423600	-0.02180275
517	0.02845238	0.00267111	-0.02205300
518	0.02604881	0.00120235	-0.02184135
519	0.02380900	-0.00016194	-0.02121984
520	0.02172062	-0.00145373	-0.02030375
521	0.01976510	-0.00266800	-0.01900621
522	0.01792823	-0.00380521	-0.01733423
523	0.01622524	-0.00486140	-0.01528144
524	0.01470160	-0.00580866	-0.01282573
525	0.01374623	-0.00671011	-0.01037161
526	0.01315234	-0.00744938	-0.00791587
527	0.01291472	-0.00806054	-0.00555697
528	0.01291688	-0.00848522	-0.00315468

529	0.01327142	-0.00880784	-0.00088023
530	0.01387188	-0.00899764	0.00139352
531	0.01477266	-0.00909783	0.00364489
532	0.01600150	-0.00899478	0.00585892
533	0.01760177	-0.00871101	0.00796650
534	0.01938902	-0.00815952	0.00993521
535	0.02128150	-0.00742048	0.01149709
536	0.02321312	-0.00657581	0.01271941
537	0.02518362	-0.00561769	0.01362125
538	0.02721400	-0.00450125	0.01412735
539	0.02928365	-0.00337291	0.01445786
540	0.03141422	-0.00202938	0.01431616
541	0.03361561	-0.00059381	0.01387272
542	0.03582896	0.00094347	0.01291860
543	0.03815187	0.00211273	0.01142564
544	0.04007704	0.00301603	0.00962032
545	0.04160189	0.00365699	0.00766026
546	0.04281931	0.00407494	0.00558339
547	0.04373083	0.00424515	0.00339112
548	0.04436328	0.00421684	0.00102445
549	0.04466204	0.00396006	-0.00153914
550	0.04450543	0.00341643	-0.00429570
551	0.04384858	0.00256844	-0.00712523
552	0.04293305	0.00160103	-0.00937581
553	0.04174659	0.00056975	-0.01122920
554	0.04042460	-0.00048767	-0.01272248
555	0.03896913	-0.00159080	-0.01386252
556	0.03739605	-0.00272174	-0.01469660
557	0.03565776	-0.00388725	-0.01520295
558	0.03376937	-0.00511046	-0.01541984
559	0.03169380	-0.00636261	-0.01521285
560	0.02952656	-0.00762273	-0.01449492
561	0.02764029	-0.00894852	-0.01320450
562	0.02617846	-0.01001954	-0.01163580
563	0.02513674	-0.01087365	-0.00996952
564	0.02445399	-0.01155181	-0.00826565
565	0.02407653	-0.01210816	-0.00649903
566	0.02399903	-0.01254804	-0.00463897
567	0.02428258	-0.01284851	-0.00264503
568	0.02502343	-0.01283135	-0.00054730
569	0.02623584	-0.01255681	0.00144603
570	0.02813325	-0.01192660	0.00333155
571	0.03002150	-0.01113727	0.00444209
572	0.03162717	-0.01043283	0.00498321
573	0.03296394	-0.00982515	0.00513280
574	0.03421689	-0.00929755	0.00500667
575	0.03550573	-0.00882718	0.00450555
576	0.03688673	-0.00838327	0.00332144
577	0.03765293	-0.00795708	0.00095624
578	-0.00459464	-0.00587182	-0.01376945
579	-0.00273155	-0.00533095	-0.01292065
580	-0.00073844	-0.00525005	-0.01198809
581	0.00096144	-0.00532449	-0.01082752
582	0.00239149	-0.00555646	-0.00942288
583	0.00344767	-0.00581869	-0.00774452
584	0.00413085	-0.00619832	-0.00572999
585	0.00465469	-0.00674562	-0.00355166
586	0.00496190	-0.00743293	-0.00114576
587	0.00530971	-0.00842767	0.00132359
588	0.00632525	-0.00996943	0.00363567

589	0.00775361	-0.01135201	0.00574620
590	0.00927329	-0.01240332	0.00770064
591	0.01084308	-0.01304152	0.00955831
592	0.01244059	-0.01324235	0.01135775
593	0.01412970	-0.01307055	0.01307867
594	0.01589284	-0.01250478	0.01474550
595	0.01771554	-0.01152557	0.01636447
596	0.01959295	-0.01013040	0.01790716
597	0.02147653	-0.00832849	0.01914559
598	0.02321275	-0.00639859	0.02011143
599	0.02482942	-0.00439306	0.02076766
600	0.02638890	-0.00237011	0.02108538
601	0.02793764	-0.00035993	0.02103826
602	0.02948326	0.00165308	0.02062584
603	0.03110900	0.00362327	0.01982939
604	0.03287009	0.00549295	0.01867936
605	0.03474645	0.00721475	0.01722359
606	0.03684689	0.00870022	0.01467667
607	0.03870447	0.00976526	0.01206565
608	0.04027808	0.01045594	0.00939671
609	0.04154846	0.01077441	0.00662729
610	0.04249368	0.01075060	0.00379266
611	0.04311396	0.01036542	0.00085822
612	0.04337671	0.00968176	-0.00218105
613	0.04321973	0.00872695	-0.00534707
614	0.04258291	0.00756473	-0.00860296
615	0.04160361	0.00631417	-0.01104767
616	0.04033954	0.00510050	-0.01310012
617	0.03892060	0.00383960	-0.01476117
618	0.03728818	0.00258201	-0.01602953
619	0.03551095	0.00126180	-0.01690153
620	0.03355573	-0.00009085	-0.01738905
621	0.03143395	-0.00149031	-0.01749612
622	0.02914230	-0.00294965	-0.01722661
623	0.02669279	-0.00447318	-0.01656660
624	0.02458627	-0.00592058	-0.01527932
625	0.02282270	-0.00712027	-0.01375202
626	0.02143926	-0.00814776	-0.01202402
627	0.02042435	-0.00900083	-0.01010367
628	0.01980654	-0.00970951	-0.00798694
629	0.01958789	-0.01032255	-0.00567488
630	0.01976566	-0.01080192	-0.00316231
631	0.02032862	-0.01118711	-0.00043071
632	0.02127070	-0.01130018	0.00247031
633	0.02265745	-0.01111651	0.00462887
634	0.02409988	-0.01067787	0.00643708
635	0.02560583	-0.01005549	0.00787273
636	0.02720520	-0.00929719	0.00893692
637	0.02889807	-0.00845870	0.00964467
638	0.03069440	-0.00750450	0.00999093
639	0.03260157	-0.00644598	0.00997519
640	0.03464823	-0.00534510	0.00963019
641	0.03678053	-0.00410615	0.00891382
642	0.03834466	-0.00335566	0.00785596
643	0.03967383	-0.00275624	0.00667716
644	0.04075996	-0.00230913	0.00536742
645	0.04161654	-0.00201976	0.00393782
646	0.04224815	-0.00189686	0.00239469
647	0.04264769	-0.00191587	0.00071319
648	0.04284856	-0.00212817	-0.00108748

649	0.04276893	-0.00244863	-0.00303274
650	0.04241955	-0.00289195	-0.00509175
651	0.04183543	-0.00349458	-0.00663639
652	0.04112522	-0.00414939	-0.00794542
653	0.04027032	-0.00482138	-0.00900584
654	0.03932087	-0.00556541	-0.00982470
655	0.03825551	-0.00634064	-0.01040354
656	0.03708499	-0.00718414	-0.01074707
657	0.03581856	-0.00810185	-0.01085039
658	0.03445033	-0.00907979	-0.01071391
659	0.03295812	-0.01008576	-0.01032627
660	0.03178287	-0.01100637	-0.00937166
661	0.03082946	-0.01175756	-0.00835265
662	0.03010725	-0.01236422	-0.00728055
663	0.02963288	-0.01285379	-0.00616350
664	0.02940491	-0.01320486	-0.00500057
665	0.02939212	-0.01341430	-0.00378288
666	0.02962966	-0.01349129	-0.00250939
667	0.03010733	-0.01341183	-0.00118663
668	0.03079837	-0.01316560	0.00019503
669	0.03189963	-0.01276166	0.00093701
670	0.03288750	-0.01228901	0.00145003
671	0.03383133	-0.01185655	0.00176518
672	0.03469615	-0.01140162	0.00186930
673	0.03550762	-0.01092103	0.00176100
674	0.03628995	-0.01037687	0.00141192
675	0.03704273	-0.00970455	0.00076688
676	0.03752573	-0.00867927	-0.00017363
677	-0.00912801	0.01390921	0.00010090
678	-0.01263726	0.01690472	-0.00145608
679	-0.01640844	0.01952211	-0.00290289
680	-0.01996464	0.02199208	-0.00413412
681	-0.02319218	0.02441795	-0.00520379
682	-0.02631276	0.02659332	-0.00607386
683	-0.02950097	0.02846677	-0.00681212
684	-0.03234726	0.03017782	-0.00719287
685	-0.03517994	0.03172123	-0.00744531
686	-0.03794887	0.03293702	-0.00756263

Procrustes sums of squares: 0.33329314860522935
Tangent sums of squares: 0.33016425735427646

Data matrices in this dataset:
   - Evolution Cervidae final, raw data
   - sans Heteroprox Sansan, centroid size
   - sans Heteroprox Sansan, Procrustes coordinates

New Procrustes fit:
Dataset 'Sans les Indet.'

686 landmarks in 3 dimensions.
 The dataset contains 40 observations, of which 36 are included for analyses.

Average shape:

Lmk.	Axis 1 (x)	Axis 2 (y)	Axis 3 (z)
1	-0.04036362	0.02592451	-0.00811150

2	-0.02486511	-0.00981714	-0.02627340
3	-0.02630368	-0.01137480	-0.02731431
4	-0.02818995	-0.01223046	-0.02847610
5	-0.03017741	-0.01259941	-0.02952329
6	-0.03220235	-0.01261672	-0.03047543
7	-0.03431632	-0.01234278	-0.03134544
8	-0.03656948	-0.01172608	-0.03215652
9	-0.03897484	-0.01071316	-0.03289171
10	-0.04144477	-0.00924722	-0.03353796
11	-0.04391420	-0.00725860	-0.03406058
12	-0.04660985	-0.00439912	-0.03449515
13	-0.04870323	-0.00138086	-0.03453497
14	-0.05020484	0.00168057	-0.03420577
15	-0.05127422	0.00468345	-0.03352928
16	-0.05194588	0.00763278	-0.03255234
17	-0.05218638	0.01059840	-0.03122656
18	-0.05198752	0.01357426	-0.02955756
19	-0.05126707	0.01655038	-0.02751849
20	-0.04987035	0.01940290	-0.02515643
21	-0.04786808	0.02164297	-0.02204558
22	-0.04544731	0.02307481	-0.01919084
23	-0.04287669	0.02382276	-0.01656435
24	-0.04021532	0.02392447	-0.01415795
25	-0.03749705	0.02347117	-0.01185990
26	-0.03451674	0.02226598	-0.00987187
27	-0.03131650	0.02036366	-0.00814552
28	-0.02795494	0.01785428	-0.00653419
29	-0.02446861	0.01459152	-0.00530442
30	-0.02274492	0.01293029	-0.00496445
31	-0.02130127	0.01127595	-0.00504291
32	-0.02018054	0.00965077	-0.00552535
33	-0.01937887	0.00805876	-0.00639792
34	-0.01887320	0.00649494	-0.00763445
35	-0.01863959	0.00493692	-0.00923185
36	-0.01861014	0.00336333	-0.01114849
37	-0.01881318	0.00170255	-0.01342681
38	-0.01950246	0.00028609	-0.01612496
39	-0.02051828	-0.00009089	-0.01770052
40	-0.02170450	-0.00033282	-0.01922434
41	-0.02269031	-0.00088248	-0.02062383
42	-0.02347663	-0.00171594	-0.02187741
43	-0.02404150	-0.00284644	-0.02298066
44	-0.02444990	-0.00424358	-0.02398036
45	-0.02463759	-0.00594249	-0.02481416
46	-0.02463999	-0.00790540	-0.02549130
47	-0.01971082	-0.01429760	-0.02654986
48	-0.02207603	-0.01533880	-0.02788349
49	-0.02452514	-0.01601771	-0.02907491
50	-0.02697985	-0.01635938	-0.03012478
51	-0.02938783	-0.01643944	-0.03108082
52	-0.03172828	-0.01627283	-0.03197942
53	-0.03405324	-0.01585177	-0.03278889
54	-0.03635762	-0.01517846	-0.03352073
55	-0.03864152	-0.01423175	-0.03417591
56	-0.04088868	-0.01300501	-0.03472953
57	-0.04298360	-0.01153854	-0.03527308
58	-0.04489822	-0.00994488	-0.03566984
59	-0.04665507	-0.00824501	-0.03596318
60	-0.04822977	-0.00641306	-0.03612856
61	-0.04969089	-0.00450700	-0.03623035

62	-0.05095120	-0.00244136	-0.03614721
63	-0.05204945	-0.00025523	-0.03596464
64	-0.05297543	0.00205276	-0.03560948
65	-0.05371134	0.00447999	-0.03512109
66	-0.05419957	0.00667575	-0.03445464
67	-0.05451852	0.00881152	-0.03371248
68	-0.05466005	0.01088919	-0.03286446
69	-0.05459579	0.01289528	-0.03188451
70	-0.05437111	0.01483793	-0.03081159
71	-0.05400202	0.01676676	-0.02966597
72	-0.05338614	0.01857604	-0.02833334
73	-0.05259955	0.02033943	-0.02692452
74	-0.05160894	0.02199484	-0.02538766
75	-0.05032088	0.02348538	-0.02386987
76	-0.04889424	0.02477045	-0.02222484
77	-0.04734848	0.02583566	-0.02045486
78	-0.04568356	0.02668656	-0.01854892
79	-0.04389958	0.02729224	-0.01651489
80	-0.04196674	0.02768918	-0.01436050
81	-0.03993372	0.02788108	-0.01206327
82	-0.03778278	0.02786411	-0.00962310
83	-0.03550002	0.02805675	-0.00713649
84	-0.02475516	0.00500360	0.02164341
85	-0.02663734	0.00345875	0.02300711
86	-0.02893429	0.00246871	0.02432777
87	-0.03125222	0.00179496	0.02541824
88	-0.03351854	0.00126713	0.02624892
89	-0.03576266	0.00091725	0.02689810
90	-0.03801045	0.00073757	0.02735358
91	-0.04026958	0.00071849	0.02765156
92	-0.04255395	0.00091244	0.02778557
93	-0.04484174	0.00133713	0.02770046
94	-0.04746711	0.00203698	0.02715615
95	-0.04978796	0.00300011	0.02632534
96	-0.05182017	0.00415271	0.02524752
97	-0.05352649	0.00550637	0.02392747
98	-0.05500355	0.00695445	0.02240050
99	-0.05624612	0.00856683	0.02065234
100	-0.05722574	0.01032876	0.01866341
101	-0.05794862	0.01226252	0.01643623
102	-0.05829858	0.01437348	0.01400590
103	-0.05820941	0.01695260	0.01097585
104	-0.05748421	0.01920360	0.00826470
105	-0.05622342	0.02109539	0.00589852
106	-0.05456535	0.02262365	0.00381355
107	-0.05245204	0.02378773	0.00203401
108	-0.04992802	0.02461653	0.00047559
109	-0.04691429	0.02511807	-0.00083014
110	-0.04338655	0.02524868	-0.00180378
111	-0.03939899	0.02500765	-0.00219625
112	-0.03571669	0.02334225	-0.00172767
113	-0.03242682	0.02173363	-0.00074659
114	-0.02951264	0.02020953	0.00028187
115	-0.02694310	0.01876207	0.00131585
116	-0.02471578	0.01734922	0.00240066
117	-0.02278449	0.01593180	0.00362812
118	-0.02116758	0.01449232	0.00516745
119	-0.02005743	0.01321894	0.00717437
120	-0.01982002	0.01265052	0.00928625
121	-0.02048050	0.01276028	0.01082980

122	-0.02140577	0.01295381	0.01235614
123	-0.02221766	0.01279049	0.01383239
124	-0.02290441	0.01229057	0.01525892
125	-0.02344077	0.01132780	0.01664227
126	-0.02388330	0.01016281	0.01795521
127	-0.02417959	0.00871239	0.01919274
128	-0.02428154	0.00676757	0.02037126
129	-0.01889190	0.00302862	0.02394466
130	-0.02110260	0.00214561	0.02522712
131	-0.02349788	0.00139899	0.02617422
132	-0.02586912	0.00076067	0.02701963
133	-0.02819728	0.00019760	0.02778945
134	-0.03049387	-0.00028099	0.02849185
135	-0.03278938	-0.00066872	0.02911548
136	-0.03508840	-0.00096192	0.02966257
137	-0.03739207	-0.00114914	0.03004513
138	-0.03968475	-0.00124508	0.03031424
139	-0.04199260	-0.00107408	0.03043188
140	-0.04419000	-0.00078425	0.03037969
141	-0.04626033	-0.00034011	0.03007118
142	-0.04822308	0.00018904	0.02966283
143	-0.05005434	0.00084689	0.02904747
144	-0.05177324	0.00165080	0.02827374
145	-0.05339725	0.00256569	0.02734367
146	-0.05488949	0.00361482	0.02622050
147	-0.05627996	0.00479674	0.02496187
148	-0.05780268	0.00638624	0.02325633
149	-0.05900770	0.00801934	0.02146877
150	-0.05993875	0.00967549	0.01961293
151	-0.06065928	0.01137019	0.01772820
152	-0.06111308	0.01310885	0.01578557
153	-0.06132017	0.01486941	0.01377255
154	-0.06123036	0.01664026	0.01167593
155	-0.06084204	0.01843626	0.00949995
156	-0.06010631	0.02023352	0.00726995
157	-0.05876156	0.02231800	0.00499395
158	-0.05702064	0.02406532	0.00291125
159	-0.05495415	0.02551484	0.00103142
160	-0.05263172	0.02673985	-0.00076846
161	-0.04991975	0.02759773	-0.00236395
162	-0.04685180	0.02816012	-0.00379531
163	-0.04340435	0.02836941	-0.00505424
164	-0.03957295	0.02841727	-0.00620094
165	-0.03550002	0.02805675	-0.00713649
166	-0.02499274	-0.01797812	-0.01144966
167	-0.02594114	-0.01926394	-0.01127705
168	-0.02715963	-0.02032120	-0.01063659
169	-0.02835612	-0.02123163	-0.00977690
170	-0.02953381	-0.02203539	-0.00869693
171	-0.03072663	-0.02273138	-0.00741166
172	-0.03191706	-0.02340512	-0.00589307
173	-0.03311180	-0.02396404	-0.00412300
174	-0.03431553	-0.02442274	-0.00211313
175	-0.03556056	-0.02468571	0.00012660
176	-0.03649211	-0.02473268	0.00178126
177	-0.03733966	-0.02465161	0.00340695
178	-0.03820179	-0.02302148	0.00481856
179	-0.03880320	-0.02403014	0.00653744
180	-0.03941172	-0.02351355	0.00803519
181	-0.03995039	-0.02286350	0.00951174

182	-0.04039727	-0.02207565	0.01095243
183	-0.04079079	-0.02116340	0.01237129
184	-0.04109684	-0.02011810	0.01374697
185	-0.04130890	-0.01876998	0.01527170
186	-0.04134585	-0.01740235	0.01661532
187	-0.04122223	-0.01601967	0.01784617
188	-0.04086897	-0.01457793	0.01871371
189	-0.04035602	-0.01312194	0.01951769
190	-0.03964819	-0.01161107	0.02003250
191	-0.03873596	-0.01005176	0.02032875
192	-0.03763316	-0.00846149	0.02042424
193	-0.03633865	-0.00682257	0.02029050
194	-0.03442252	-0.00477936	0.01989045
195	-0.03261396	-0.00294886	0.01921052
196	-0.03090104	-0.00137572	0.01823817
197	-0.02927633	-0.00002425	0.01701181
198	-0.02777946	0.00114102	0.01557634
199	-0.02638943	0.00209796	0.01386781
200	-0.02507999	0.00277584	0.01189396
201	-0.02383005	0.00335102	0.00971822
202	-0.02268630	0.00367457	0.00728109
203	-0.02198686	0.00350864	0.00526205
204	-0.02150943	0.00305574	0.00328699
205	-0.02109940	0.00246504	0.00146270
206	-0.02070629	0.00172063	-0.00021211
207	-0.02031526	0.00079952	-0.00175701
208	-0.02001998	-0.00032600	-0.00321626
209	-0.01977998	-0.00165156	-0.00460495
210	-0.01972435	-0.00318700	-0.00591164
211	-0.02012169	-0.00482420	-0.00683380
212	-0.02086359	-0.00632844	-0.00688795
213	-0.02179806	-0.00781622	-0.00684819
214	-0.02269357	-0.00929351	-0.00694264
215	-0.02340306	-0.01076302	-0.00724541
216	-0.02389958	-0.01220369	-0.00778537
217	-0.02423287	-0.01365489	-0.00854983
218	-0.02439367	-0.01512932	-0.00955153
219	-0.02446461	-0.01665275	-0.01071370
220	-0.02301658	-0.01983875	-0.01657561
221	-0.02449962	-0.02103703	-0.01580409
222	-0.02591209	-0.02210145	-0.01482174
223	-0.02725720	-0.02308326	-0.01371343
224	-0.02855787	-0.02398036	-0.01249724
225	-0.02979391	-0.02477324	-0.01114796
226	-0.03097478	-0.02545093	-0.00967183
227	-0.03210955	-0.02607016	-0.00809480
228	-0.03319008	-0.02659988	-0.00638774
229	-0.03426191	-0.02708908	-0.00460141
230	-0.03533030	-0.02742900	-0.00265247
231	-0.03638312	-0.02766692	-0.00077013
232	-0.03734717	-0.02770520	0.00110545
233	-0.03826857	-0.02760746	0.00292934
234	-0.03908166	-0.02730438	0.00475011
235	-0.03985656	-0.02685237	0.00652661
236	-0.04056376	-0.02621526	0.00828375
237	-0.04119816	-0.02541774	0.01001183
238	-0.04177051	-0.02445017	0.01171237
239	-0.04234573	-0.02326985	0.01357121
240	-0.04280543	-0.02204921	0.01527783
241	-0.04307339	-0.02075208	0.01680627

242	-0.04316928	-0.01938416	0.01818845
243	-0.04309833	-0.01796702	0.01938745
244	-0.04288469	-0.01648776	0.02045101
245	-0.04251190	-0.01496819	0.02134714
246	-0.04194658	-0.01335978	0.02208120
247	-0.04122537	-0.01171653	0.02264389
248	-0.04013103	-0.00977920	0.02317404
249	-0.03891342	-0.00789103	0.02339685
250	-0.03757997	-0.00603269	0.02333239
251	-0.03612316	-0.00421851	0.02298515
252	-0.03457502	-0.00244218	0.02236291
253	-0.03298520	-0.00059142	0.02156995
254	-0.03104639	0.00094796	0.02023291
255	-0.02905013	0.00254424	0.01870258
256	-0.02719004	0.00426947	0.01701131
257	-0.01420492	-0.00895315	0.00083756
258	-0.01362852	-0.00923638	0.00155711
259	-0.01293438	-0.00949925	0.00213629
260	-0.01213240	-0.00968167	0.00251142
261	-0.01129125	-0.00982879	0.00280732
262	-0.01045304	-0.01007004	0.00295865
263	-0.00950672	-0.01035670	0.00294027
264	-0.00854636	-0.01069007	0.00289594
265	-0.00740862	-0.01091971	0.00315727
266	-0.00644379	-0.01139151	0.00217907
267	-0.00519335	-0.01148261	0.00169384
268	-0.00408083	-0.01161007	0.00118547
269	-0.00317717	-0.01171258	0.00058195
270	-0.00244467	-0.01181043	-0.00011340
271	-0.00186863	-0.01198125	-0.00087273
272	-0.00139618	-0.01214156	-0.00173581
273	-0.00106708	-0.01221241	-0.00271789
274	-0.00097424	-0.01225575	-0.00380308
275	-0.00109211	-0.01226381	-0.00495813
276	-0.00181558	-0.01255486	-0.00585019
277	-0.00265436	-0.01267661	-0.00655561
278	-0.00352794	-0.01272289	-0.00709354
279	-0.00444229	-0.01278634	-0.00742448
280	-0.00539185	-0.01279159	-0.00757620
281	-0.00639964	-0.01275603	-0.00755145
282	-0.00743214	-0.01262899	-0.00732805
283	-0.00859772	-0.01256264	-0.00696748
284	-0.00973407	-0.01239425	-0.00642912
285	-0.01078608	-0.01202259	-0.00585542
286	-0.01173719	-0.01165365	-0.00519610
287	-0.01251652	-0.01124299	-0.00448940
288	-0.01318365	-0.01085834	-0.00376242
289	-0.01370455	-0.01048911	-0.00298549
290	-0.01408853	-0.01012381	-0.00212894
291	-0.01431493	-0.00976411	-0.00119754
292	-0.01435066	-0.00937240	-0.00019292
293	0.02587333	0.01926473	0.01617969
294	0.02816657	0.01805224	0.01850146
295	0.03003528	0.01703775	0.02018269
296	0.03267677	0.01459136	0.02173532
297	0.03362073	0.01306809	0.02168953
298	0.03446001	0.01126160	0.02116429
299	0.03496819	0.00901716	0.01983755
300	0.03707364	0.01699636	-0.01060991
301	0.03923838	0.01638373	-0.01093459

302	0.04097023	0.01570535	-0.01111726
303	0.04219610	0.01478278	-0.01105536
304	0.04350500	0.01254245	-0.01048141
305	0.04375876	0.01115959	-0.00997934
306	0.04362935	0.00951276	-0.00926276
307	0.02070917	0.00418236	-0.01852877
308	0.02123998	0.00252670	-0.01979348
309	0.02187883	0.00107518	-0.02051511
310	0.02256028	-0.00014793	-0.02081252
311	0.02411657	-0.00211887	-0.02029710
312	0.02503308	-0.00299290	-0.01955280
313	0.02608090	-0.00378639	-0.01840111
314	0.03185011	0.00463990	0.01061681
315	0.03345782	0.00346569	0.01181123
316	0.03461084	0.00242664	0.01257250
317	0.03548554	0.00143281	0.01291878
318	0.03657419	-0.00059275	0.01250653
319	0.03685028	-0.00172227	0.01179215
320	0.03695813	-0.00299166	0.01069417
321	0.03846695	0.00676464	-0.00647510
322	0.04109393	0.00486686	-0.00675629
323	0.04276408	0.00408199	-0.00718175
324	0.04411850	0.00221221	-0.00702164
325	0.04422334	0.00123872	-0.00674590
326	0.04409352	0.00014020	-0.00634048
327	0.04365107	-0.00119541	-0.00575169
328	0.02812671	-0.00288162	-0.01147439
329	0.02834146	-0.00458265	-0.01324353
330	0.02884814	-0.00600743	-0.01412604
331	0.02997272	-0.00791211	-0.01445270
332	0.03060502	-0.00858125	-0.01405170
333	0.03135400	-0.00917384	-0.01333860
334	0.03225444	-0.00968688	-0.01218141
335	-0.01172028	-0.00127986	0.01812014
336	-0.00981966	-0.00017310	0.01596653
337	-0.00748292	0.00135292	0.01318677
338	-0.00467901	0.00176626	0.01257339
339	-0.00160492	0.00166516	0.01345472
340	0.00073520	0.00261184	0.01288804
341	0.00309907	0.00379113	0.01238177
342	0.00544885	0.00525394	0.01189193
343	0.00775190	0.00691334	0.01155331
344	0.01000933	0.00859471	0.01167153
345	0.01159026	0.00924354	0.01172904
346	0.01313523	0.00992465	0.01169330
347	0.01462754	0.01061938	0.01153682
348	0.01607257	0.01123832	0.01130270
349	0.01746758	0.01184949	0.01094656
350	0.01883896	0.01248093	0.01049223
351	0.02019423	0.01308261	0.00995225
352	0.02149643	0.01371791	0.00923701
353	0.02272847	0.01433868	0.00841663
354	0.02481547	0.01491058	0.00685853
355	0.02656942	0.01530765	0.00518982
356	0.02808847	0.01552710	0.00350573
357	0.02930815	0.01554265	0.00175550
358	0.03028629	0.01537772	-0.00000438
359	0.03107232	0.01504573	-0.00178924
360	0.03160693	0.01450721	-0.00360661
361	0.03177200	0.01365817	-0.00540187

362	0.03160557	0.01293630	-0.00744600
363	0.03106170	0.01175631	-0.00900205
364	0.03038092	0.01111919	-0.01031657
365	0.02952562	0.01019383	-0.01133570
366	0.02853786	0.00936300	-0.01204035
367	0.02748432	0.00852688	-0.01249330
368	0.02632847	0.00767429	-0.01272849
369	0.02507221	0.00679246	-0.01271952
370	0.02370023	0.00589785	-0.01246631
371	0.02219163	0.00511297	-0.01183295
372	0.02060253	0.00332031	-0.00966574
373	0.01936455	0.00241102	-0.00757266
374	0.01871548	0.00159697	-0.00553696
375	0.01852484	0.00115230	-0.00365728
376	0.01884631	0.00065988	-0.00185211
377	0.01974608	0.00010857	-0.00008481
378	0.02094316	-0.00003790	0.00176350
379	0.02291868	0.00010279	0.00337593
380	0.02528540	0.00115883	0.00421022
381	0.02774686	0.00133632	0.00399910
382	0.02960979	0.00217368	0.00353866
383	0.03088601	0.00273921	0.00281149
384	0.03169197	0.00299553	0.00187416
385	0.03231657	0.00294339	0.00088867
386	0.03279191	0.00259206	-0.00014395
387	0.03321028	0.00185201	-0.00142195
388	0.03280723	0.00113424	-0.00303939
389	0.03138479	0.00071611	-0.00423056
390	0.03074004	-0.00050220	-0.00429013
391	0.02978969	-0.00125837	-0.00387234
392	0.02934388	-0.00169521	-0.00329803
393	0.02954951	-0.00213744	-0.00293272
394	0.03011139	-0.00272617	-0.00249838
395	0.03123281	-0.00373416	-0.00218899
396	0.03270543	-0.00474862	-0.00183261
397	0.03581692	-0.00584704	-0.00153825
398	0.00175150	0.01384106	0.01986431
399	0.00395592	0.01323955	0.01977547
400	0.00636728	0.01320706	0.01958638
401	0.00877127	0.01345148	0.01923816
402	0.01114184	0.01399185	0.01872838
403	0.01349436	0.01464576	0.01796054
404	0.01589219	0.01557216	0.01697272
405	0.01835448	0.01668598	0.01584368
406	0.02091562	0.01787523	0.01456537
407	0.02362854	0.01904545	0.01310844
408	0.02615073	0.01994385	0.01081755
409	0.02836713	0.02052226	0.00846297
410	0.03029835	0.02075309	0.00605305
411	0.03188309	0.02071802	0.00358688
412	0.03312215	0.02044034	0.00105844
413	0.03402449	0.01992269	-0.00155966
414	0.03459416	0.01916495	-0.00424869
415	0.03481121	0.01814049	-0.00701032
416	0.03466972	0.01691722	-0.00986995
417	0.03359050	0.01554510	-0.01201557
418	0.03238080	0.01422233	-0.01382808
419	0.03102669	0.01296553	-0.01530783
420	0.02957444	0.01170741	-0.01642472
421	0.02800219	0.01049636	-0.01719415

422	0.02631005	0.00928329	-0.01761252
423	0.02448131	0.00808574	-0.01767455
424	0.02254429	0.00687127	-0.01737555
425	0.02058327	0.00558651	-0.01669829
426	0.01785690	0.00399632	-0.01437669
427	0.01585703	0.00261997	-0.01195234
428	0.01453051	0.00146287	-0.00946739
429	0.01384650	0.00056285	-0.00691465
430	0.01375780	-0.00001375	-0.00427298
431	0.01440182	-0.00041373	-0.00156825
432	0.01576558	-0.00059723	0.00122092
433	0.01780654	-0.00054486	0.00410058
434	0.02045436	-0.00015461	0.00707657
435	0.02296861	0.00095982	0.00803691
436	0.02522707	0.00206162	0.00864778
437	0.02727785	0.00306918	0.00889625
438	0.02911712	0.00395986	0.00878205
439	0.03075297	0.00470902	0.00828189
440	0.03219306	0.00534578	0.00740889
441	0.03341590	0.00592685	0.00616181
442	0.03451745	0.00625965	0.00452302
443	0.03543575	0.00641256	0.00252476
444	0.03585532	0.00629171	0.00071914
445	0.03609805	0.00604791	-0.00094565
446	0.03618289	0.00573616	-0.00249520
447	0.03608863	0.00534430	-0.00390653
448	0.03584725	0.00484086	-0.00518997
449	0.03540758	0.00424762	-0.00634779
450	0.03479334	0.00358544	-0.00738212
451	0.03401438	0.00281988	-0.00829128
452	0.03307100	0.00195507	-0.00905581
453	0.03197989	0.00104425	-0.00949076
454	0.03092847	0.00026206	-0.00970118
455	0.02998050	-0.00049538	-0.00966793
456	0.02907338	-0.00114972	-0.00940987
457	0.02824415	-0.00176644	-0.00889215
458	0.02748598	-0.00234658	-0.00810671
459	0.02687795	-0.00295596	-0.00702761
460	0.02643400	-0.00358347	-0.00567919
461	0.02622359	-0.00427971	-0.00412512
462	0.02697931	-0.00414388	-0.00228177
463	0.02783022	-0.00416996	-0.00083607
464	0.02872124	-0.00424154	0.00027241
465	0.02970001	-0.00433671	0.00101924
466	0.03066197	-0.00426093	0.00164923
467	0.03189605	-0.00440755	0.00162636
468	0.03374501	-0.00469686	0.00105143
469	0.03562753	-0.00567469	-0.00040085
470	-0.01405999	-0.00448353	0.01199846
471	-0.01327624	-0.00615359	0.01155577
472	-0.01218013	-0.00765257	0.01108889
473	-0.01089008	-0.00894656	0.01074745
474	-0.00952228	-0.01014643	0.01065119
475	-0.00802525	-0.01122904	0.01074947
476	-0.00648290	-0.01228996	0.01106501
477	-0.00526464	-0.01342736	0.01160294
478	-0.00284469	-0.01445149	0.01218824
479	-0.00235071	-0.01505638	0.01362634
480	-0.00057922	-0.01551150	0.01468553
481	0.00112223	-0.01579763	0.01578951

482	0.00277592	-0.01587528	0.01692713
483	0.00435767	-0.01575872	0.01807645
484	0.00588584	-0.01544568	0.01926245
485	0.00736047	-0.01496314	0.02049256
486	0.00878267	-0.01424004	0.02174220
487	0.01016040	-0.01325788	0.02300620
488	0.01150151	-0.01205326	0.02424520
489	0.01272474	-0.01063365	0.02541270
490	0.01394615	-0.00912507	0.02639944
491	0.01517522	-0.00756413	0.02728012
492	0.01643602	-0.00591284	0.02795602
493	0.01767138	-0.00416505	0.02847710
494	0.01893487	-0.00230851	0.02881471
495	0.02021383	-0.00038022	0.02899355
496	0.02150517	0.00165100	0.02897907
497	0.02279615	0.00377396	0.02871005
498	0.02479946	0.00683549	0.02788249
499	0.02678725	0.00955162	0.02661579
500	0.02876705	0.01190745	0.02497364
501	0.03075035	0.01392402	0.02302300
502	0.03276864	0.01564003	0.02077317
503	0.03480122	0.01701378	0.01814617
504	0.03685017	0.01804085	0.01513511
505	0.03885990	0.01867420	0.01169836
506	0.04071137	0.01880678	0.00783131
507	0.04234804	0.01842942	0.00319138
508	0.04319392	0.01758835	-0.00120791
509	0.04329672	0.01643156	-0.00516137
510	0.04283799	0.01510102	-0.00863886
511	0.04191714	0.01363323	-0.01167814
512	0.04058038	0.01206258	-0.01437005
513	0.03881765	0.01036889	-0.01677530
514	0.03653839	0.00849577	-0.01883607
515	0.03369222	0.00641862	-0.02043009
516	0.03121893	0.00480826	-0.02122917
517	0.02881560	0.00326752	-0.02153818
518	0.02653170	0.00180376	-0.02142990
519	0.02438529	0.00042885	-0.02093315
520	0.02235112	-0.00087884	-0.02012587
521	0.02043778	-0.00211728	-0.01893960
522	0.01862180	-0.00329883	-0.01740436
523	0.01691967	-0.00439601	-0.01547014
524	0.01539708	-0.00537614	-0.01311932
525	0.01436649	-0.00635815	-0.01077700
526	0.01369976	-0.00717745	-0.00841675
527	0.01337480	-0.00782977	-0.00607145
528	0.01334378	-0.00834772	-0.00375622
529	0.01362223	-0.00870699	-0.00147973
530	0.01416921	-0.00895092	0.00078057
531	0.01500797	-0.00907364	0.00304707
532	0.01619837	-0.00902670	0.00527835
533	0.01774937	-0.00877777	0.00743715
534	0.01943712	-0.00824749	0.00936803
535	0.02124334	-0.00752661	0.01093038
536	0.02308198	-0.00671600	0.01217647
537	0.02497056	-0.00579067	0.01311554
538	0.02693106	-0.00470307	0.01367410
539	0.02893472	-0.00360428	0.01407307
540	0.03101090	-0.00229582	0.01403004
541	0.03316389	-0.00088888	0.01367675

542	0.03533969	0.00060278	0.01283318
543	0.03767007	0.00181293	0.01145107
544	0.03961497	0.00276158	0.00973062
545	0.04116412	0.00344369	0.00784756
546	0.04240633	0.00390778	0.00583676
547	0.04334771	0.00412907	0.00370463
548	0.04403160	0.00415911	0.00139968
549	0.04438067	0.00396289	-0.00110620
550	0.04430687	0.00347775	-0.00380985
551	0.04372093	0.00269044	-0.00660298
552	0.04285048	0.00178208	-0.00882628
553	0.04174237	0.00079350	-0.01068087
554	0.04049535	-0.00023208	-0.01218732
555	0.03909850	-0.00130016	-0.01334704
556	0.03759087	-0.00240096	-0.01421729
557	0.03592539	-0.00354340	-0.01477669
558	0.03411289	-0.00474780	-0.01505795
559	0.03211970	-0.00599507	-0.01494660
560	0.03003332	-0.00725745	-0.01433042
561	0.02818582	-0.00857227	-0.01319741
562	0.02673746	-0.00964840	-0.01175889
563	0.02568626	-0.01052689	-0.01021597
564	0.02497676	-0.01122878	-0.00860865
565	0.02455074	-0.01182021	-0.00692180
566	0.02440537	-0.01230005	-0.00512194
567	0.02459443	-0.01265729	-0.00317997
568	0.02522170	-0.01269135	-0.00109534
569	0.02616218	-0.01232520	0.00112710
570	0.02799792	-0.01164154	0.00307416
571	0.02985354	-0.01086215	0.00428979
572	0.03149247	-0.01011763	0.00487646
573	0.03286284	-0.00952532	0.00507522
574	0.03415731	-0.00902859	0.00498691
575	0.03571407	-0.00844504	0.00434290
576	0.03719703	-0.00815875	0.00316565
577	0.03796199	-0.00783864	0.00091595
578	-0.00472278	-0.00591580	-0.01389955
579	-0.00282152	-0.00534411	-0.01307654
580	-0.00077831	-0.00526381	-0.01217037
581	0.00097025	-0.00534827	-0.01103326
582	0.00242991	-0.00558909	-0.00963486
583	0.00351485	-0.00586547	-0.00795511
584	0.00433010	-0.00629561	-0.00603422
585	0.00486325	-0.00686536	-0.00381865
586	0.00519077	-0.00760052	-0.00137754
587	0.00556083	-0.00865338	0.00112401
588	0.00646828	-0.01013170	0.00343617
589	0.00783613	-0.01149684	0.00551644
590	0.00930897	-0.01254350	0.00744219
591	0.01084713	-0.01319676	0.00926903
592	0.01241892	-0.01342319	0.01104797
593	0.01408775	-0.01328703	0.01276525
594	0.01583373	-0.01277122	0.01443718
595	0.01763102	-0.01183438	0.01607692
596	0.01947566	-0.01048279	0.01764952
597	0.02140151	-0.00861850	0.01893748
598	0.02317182	-0.00663277	0.01995184
599	0.02480619	-0.00457297	0.02065572
600	0.02639007	-0.00251694	0.02101676
601	0.02795184	-0.00048046	0.02101025

602	0.02950971	0.00154632	0.02063967
603	0.03113609	0.00352040	0.01987287
604	0.03288489	0.00539669	0.01874626
605	0.03474481	0.00711491	0.01730788
606	0.03686200	0.00862248	0.01474500
607	0.03872174	0.00971562	0.01211694
608	0.04029211	0.01043822	0.00941802
609	0.04158210	0.01077047	0.00664789
610	0.04252638	0.01077335	0.00380530
611	0.04314034	0.01041931	0.00085880
612	0.04337970	0.00977831	-0.00218957
613	0.04322285	0.00884295	-0.00533685
614	0.04260484	0.00769363	-0.00858368
615	0.04161695	0.00644682	-0.01099706
616	0.04035395	0.00523362	-0.01302552
617	0.03893777	0.00397600	-0.01467091
618	0.03733841	0.00270040	-0.01593965
619	0.03557586	0.00137918	-0.01681177
620	0.03364114	0.00002475	-0.01730930
621	0.03156233	-0.00139408	-0.01743314
622	0.02931854	-0.00288202	-0.01718814
623	0.02690239	-0.00442226	-0.01654399
624	0.02483326	-0.00585611	-0.01531786
625	0.02310872	-0.00708084	-0.01384842
626	0.02174057	-0.00812270	-0.01217845
627	0.02073371	-0.00900456	-0.01031061
628	0.02008726	-0.00970652	-0.00824582
629	0.01984304	-0.01036388	-0.00598328
630	0.01997215	-0.01087333	-0.00350758
631	0.02047542	-0.01124309	-0.00083346
632	0.02133162	-0.01140695	0.00203385
633	0.02270881	-0.01122814	0.00426263
634	0.02414832	-0.01080825	0.00614223
635	0.02564284	-0.01017136	0.00763857
636	0.02724265	-0.00941665	0.00876399
637	0.02893980	-0.00857386	0.00952246
638	0.03074246	-0.00759938	0.00990687
639	0.03266598	-0.00653779	0.00993561
640	0.03473008	-0.00542265	0.00962073
641	0.03688195	-0.00415521	0.00892264
642	0.03844788	-0.00340503	0.00785551
643	0.03978015	-0.00279195	0.00666462
644	0.04087124	-0.00232931	0.00534496
645	0.04173090	-0.00202117	0.00390837
646	0.04236048	-0.00186649	0.00235601
647	0.04276548	-0.00186371	0.00067766
648	0.04297459	-0.00204918	-0.00111624
649	0.04290986	-0.00235173	-0.00304896
650	0.04257675	-0.00277653	-0.00509931
651	0.04199268	-0.00337785	-0.00662625
652	0.04128121	-0.00403213	-0.00792095
653	0.04042464	-0.00471125	-0.00897988
654	0.03946348	-0.00545763	-0.00981281
655	0.03840840	-0.00624549	-0.01039310
656	0.03724789	-0.00710525	-0.01074771
657	0.03598539	-0.00803769	-0.01087718
658	0.03462609	-0.00903747	-0.01077708
659	0.03312538	-0.01004584	-0.01042879
660	0.03197561	-0.01098321	-0.00948970
661	0.03103462	-0.01173975	-0.00848024

662	0.03033323	-0.01236701	-0.00741586
663	0.02986373	-0.01285649	-0.00629548
664	0.02963806	-0.01321170	-0.00513194
665	0.02963912	-0.01344038	-0.00390085
666	0.02985399	-0.01349280	-0.00262084
667	0.03031734	-0.01340983	-0.00128204
668	0.03100931	-0.01318364	0.00011827
669	0.03213870	-0.01275362	0.00090662
670	0.03314645	-0.01225541	0.00146206
671	0.03410651	-0.01180367	0.00179509
672	0.03498578	-0.01133088	0.00191744
673	0.03582410	-0.01084690	0.00182995
674	0.03664555	-0.01030458	0.00150342
675	0.03744968	-0.00965418	0.00083948
676	0.03793364	-0.00861659	-0.00017299
677	-0.00929247	0.01399275	-0.00004503
678	-0.01283930	0.01696123	-0.00155635
679	-0.01665438	0.01954362	-0.00296851
680	-0.02025991	0.02198157	-0.00416642
681	-0.02350131	0.02441735	-0.00522117
682	-0.02664614	0.02660005	-0.00607178
683	-0.02988727	0.02846567	-0.00678561
684	-0.03277505	0.03017638	-0.00712933
685	-0.03566587	0.03171160	-0.00735360
686	-0.03850607	0.03290508	-0.00743352

Procrustes sums of squares: 0.3657145180346275
Tangent sums of squares: 0.35793716368340484

Data matrices in this dataset:
- Evolution Cervidae final, raw data
- Sans les Indet., centroid size
- Sans les Indet., Procrustes coordinates

New Procrustes fit:
Dataset 'Sans les Indet.'

686 landmarks in 3 dimensions.
The dataset contains 40 observations, of which 36 are included for analyses.

Average shape:

Lmk.	Axis 1 (x)	Axis 2 (y)	Axis 3 (z)
1	-0.04036362	0.02592451	-0.00811150
2	-0.02486511	-0.00981714	-0.02627340
3	-0.02630368	-0.01137480	-0.02731431
4	-0.02818995	-0.01223046	-0.02847610
5	-0.03017741	-0.01259941	-0.02952329
6	-0.03220235	-0.01261672	-0.03047543
7	-0.03431632	-0.01234278	-0.03134544
8	-0.03656948	-0.01172608	-0.03215652
9	-0.03897484	-0.01071316	-0.03289171
10	-0.04144477	-0.00924722	-0.03353796
11	-0.04391420	-0.00725860	-0.03406058
12	-0.04660985	-0.00439912	-0.03449515
13	-0.04870323	-0.00138086	-0.03453497
14	-0.05020484	0.00168057	-0.03420577

15	-0.05127422	0.00468345	-0.03352928
16	-0.05194588	0.00763278	-0.03255234
17	-0.05218638	0.01059840	-0.03122656
18	-0.05198752	0.01357426	-0.02955756
19	-0.05126707	0.01655038	-0.02751849
20	-0.04987035	0.01940290	-0.02515643
21	-0.04786808	0.02164297	-0.02204558
22	-0.04544731	0.02307481	-0.01919084
23	-0.04287669	0.02382276	-0.01656435
24	-0.04021532	0.02392447	-0.01415795
25	-0.03749705	0.02347117	-0.01185990
26	-0.03451674	0.02226598	-0.00987187
27	-0.03131650	0.02036366	-0.00814552
28	-0.02795494	0.01785428	-0.00653419
29	-0.02446861	0.01459152	-0.00530442
30	-0.02274492	0.01293029	-0.00496445
31	-0.02130127	0.01127595	-0.00504291
32	-0.02018054	0.00965077	-0.00552535
33	-0.01937887	0.00805876	-0.00639792
34	-0.01887320	0.00649494	-0.00763445
35	-0.01863959	0.00493692	-0.00923185
36	-0.01861014	0.00336333	-0.01114849
37	-0.01881318	0.00170255	-0.01342681
38	-0.01950246	0.00028609	-0.01612496
39	-0.02051828	-0.00009089	-0.01770052
40	-0.02170450	-0.00033282	-0.01922434
41	-0.02269031	-0.00088248	-0.02062383
42	-0.02347663	-0.00171594	-0.02187741
43	-0.02404150	-0.00284644	-0.02298066
44	-0.02444990	-0.00424358	-0.02398036
45	-0.02463759	-0.00594249	-0.02481416
46	-0.02463999	-0.00790540	-0.02549130
47	-0.01971082	-0.01429760	-0.02654986
48	-0.02207603	-0.01533880	-0.02788349
49	-0.02452514	-0.01601771	-0.02907491
50	-0.02697985	-0.01635938	-0.03012478
51	-0.02938783	-0.01643944	-0.03108082
52	-0.03172828	-0.01627283	-0.03197942
53	-0.03405324	-0.01585177	-0.03278889
54	-0.03635762	-0.01517846	-0.03352073
55	-0.03864152	-0.01423175	-0.03417591
56	-0.04088868	-0.01300501	-0.03472953
57	-0.04298360	-0.01153854	-0.03527308
58	-0.04489822	-0.00994488	-0.03566984
59	-0.04665507	-0.00824501	-0.03596318
60	-0.04822977	-0.00641306	-0.03612856
61	-0.04969089	-0.00450700	-0.03623035
62	-0.05095120	-0.00244136	-0.03614721
63	-0.05204945	-0.00025523	-0.03596464
64	-0.05297543	0.00205276	-0.03560948
65	-0.05371134	0.00447999	-0.03512109
66	-0.05419957	0.00667575	-0.03445464
67	-0.05451852	0.00881152	-0.03371248
68	-0.05466005	0.01088919	-0.03286446
69	-0.05459579	0.01289528	-0.03188451
70	-0.05437111	0.01483793	-0.03081159
71	-0.05400202	0.01676676	-0.02966597
72	-0.05338614	0.01857604	-0.02833334
73	-0.05259955	0.02033943	-0.02692452
74	-0.05160894	0.02199484	-0.02538766

75	-0.05032088	0.02348538	-0.02386987
76	-0.04889424	0.02477045	-0.02222484
77	-0.04734848	0.02583566	-0.02045486
78	-0.04568356	0.02668656	-0.01854892
79	-0.04389958	0.02729224	-0.01651489
80	-0.04196674	0.02768918	-0.01436050
81	-0.03993372	0.02788108	-0.01206327
82	-0.03778278	0.02786411	-0.00962310
83	-0.03550002	0.02805675	-0.00713649
84	-0.02475516	0.00500360	0.02164341
85	-0.02663734	0.00345875	0.02300711
86	-0.02893429	0.00246871	0.02432777
87	-0.03125222	0.00179496	0.02541824
88	-0.03351854	0.00126713	0.02624892
89	-0.03576266	0.00091725	0.02689810
90	-0.03801045	0.00073757	0.02735358
91	-0.04026958	0.00071849	0.02765156
92	-0.04255395	0.00091244	0.02778557
93	-0.04484174	0.00133713	0.02770046
94	-0.04746711	0.00203698	0.02715615
95	-0.04978796	0.00300011	0.02632534
96	-0.05182017	0.00415271	0.02524752
97	-0.05352649	0.00550637	0.02392747
98	-0.05500355	0.00695445	0.02240050
99	-0.05624612	0.00856683	0.02065234
100	-0.05722574	0.01032876	0.01866341
101	-0.05794862	0.01226252	0.01643623
102	-0.05829858	0.01437348	0.01400590
103	-0.05820941	0.01695260	0.01097585
104	-0.05748421	0.01920360	0.00826470
105	-0.05622342	0.02109539	0.00589852
106	-0.05456535	0.02262365	0.00381355
107	-0.05245204	0.02378773	0.00203401
108	-0.04992802	0.02461653	0.00047559
109	-0.04691429	0.02511807	-0.00083014
110	-0.04338655	0.02524868	-0.00180378
111	-0.03939899	0.02500765	-0.00219625
112	-0.03571669	0.02334225	-0.00172767
113	-0.03242682	0.02173363	-0.00074659
114	-0.02951264	0.02020953	0.00028187
115	-0.02694310	0.01876207	0.00131585
116	-0.02471578	0.01734922	0.00240066
117	-0.02278449	0.01593180	0.00362812
118	-0.02116758	0.01449232	0.00516745
119	-0.02005743	0.01321894	0.00717437
120	-0.01982002	0.01265052	0.00928625
121	-0.02048050	0.01276028	0.01082980
122	-0.02140577	0.01295381	0.01235614
123	-0.02221766	0.01279049	0.01383239
124	-0.02290441	0.01229057	0.01525892
125	-0.02344077	0.01132780	0.01664227
126	-0.02388330	0.01016281	0.01795521
127	-0.02417959	0.00871239	0.01919274
128	-0.02428154	0.00676757	0.02037126
129	-0.01889190	0.00302862	0.02394466
130	-0.02110260	0.00214561	0.02522712
131	-0.02349788	0.00139899	0.02617422
132	-0.02586912	0.00076067	0.02701963
133	-0.02819728	0.00019760	0.02778945
134	-0.03049387	-0.00028099	0.02849185

135	-0.03278938	-0.00066872	0.02911548
136	-0.03508840	-0.00096192	0.02966257
137	-0.03739207	-0.00114914	0.03004513
138	-0.03968475	-0.00124508	0.03031424
139	-0.04199260	-0.00107408	0.03043188
140	-0.04419000	-0.00078425	0.03037969
141	-0.04626033	-0.00034011	0.03007118
142	-0.04822308	0.00018904	0.02966283
143	-0.05005434	0.00084689	0.02904747
144	-0.05177324	0.00165080	0.02827374
145	-0.05339725	0.00256569	0.02734367
146	-0.05488949	0.00361482	0.02622050
147	-0.05627996	0.00479674	0.02496187
148	-0.05780268	0.00638624	0.02325633
149	-0.05900770	0.00801934	0.02146877
150	-0.05993875	0.00967549	0.01961293
151	-0.06065928	0.01137019	0.01772820
152	-0.06111308	0.01310885	0.01578557
153	-0.06132017	0.01486941	0.01377255
154	-0.06123036	0.01664026	0.01167593
155	-0.06084204	0.01843626	0.00949995
156	-0.06010631	0.02023352	0.00726995
157	-0.05876156	0.02231800	0.00499395
158	-0.05702064	0.02406532	0.00291125
159	-0.05495415	0.02551484	0.00103142
160	-0.05263172	0.02673985	-0.00076846
161	-0.04991975	0.02759773	-0.00236395
162	-0.04685180	0.02816012	-0.00379531
163	-0.04340435	0.02836941	-0.00505424
164	-0.03957295	0.02841727	-0.00620094
165	-0.03550002	0.02805675	-0.00713649
166	-0.02499274	-0.01797812	-0.01144966
167	-0.02594114	-0.01926394	-0.01127705
168	-0.02715963	-0.02032120	-0.01063659
169	-0.02835612	-0.02123163	-0.00977690
170	-0.02953381	-0.02203539	-0.00869693
171	-0.03072663	-0.02273138	-0.00741166
172	-0.03191706	-0.02340512	-0.00589307
173	-0.03311180	-0.02396404	-0.00412300
174	-0.03431553	-0.02442274	-0.00211313
175	-0.03556056	-0.02468571	0.00012660
176	-0.03649211	-0.02473268	0.00178126
177	-0.03733966	-0.02465161	0.00340695
178	-0.03820179	-0.02302148	0.00481856
179	-0.03880320	-0.02403014	0.00653744
180	-0.03941172	-0.02351355	0.00803519
181	-0.03995039	-0.02286350	0.00951174
182	-0.04039727	-0.02207565	0.01095243
183	-0.04079079	-0.02116340	0.01237129
184	-0.04109684	-0.02011810	0.01374697
185	-0.04130890	-0.01876998	0.01527170
186	-0.04134585	-0.01740235	0.01661532
187	-0.04122223	-0.01601967	0.01784617
188	-0.04086897	-0.01457793	0.01871371
189	-0.04035602	-0.01312194	0.01951769
190	-0.03964819	-0.01161107	0.02003250
191	-0.03873596	-0.01005176	0.02032875
192	-0.03763316	-0.00846149	0.02042424
193	-0.03633865	-0.00682257	0.02029050
194	-0.03442252	-0.00477936	0.01989045

195	-0.03261396	-0.00294886	0.01921052
196	-0.03090104	-0.00137572	0.01823817
197	-0.02927633	-0.00002425	0.01701181
198	-0.02777946	0.00114102	0.01557634
199	-0.02638943	0.00209796	0.01386781
200	-0.02507999	0.00277584	0.01189396
201	-0.02383005	0.00335102	0.00971822
202	-0.02268630	0.00367457	0.00728109
203	-0.02198686	0.00350864	0.00526205
204	-0.02150943	0.00305574	0.00328699
205	-0.02109940	0.00246504	0.00146270
206	-0.02070629	0.00172063	-0.00021211
207	-0.02031526	0.00079952	-0.00175701
208	-0.02001998	-0.00032600	-0.00321626
209	-0.01977998	-0.00165156	-0.00460495
210	-0.01972435	-0.00318700	-0.00591164
211	-0.02012169	-0.00482420	-0.00683380
212	-0.02086359	-0.00632844	-0.00688795
213	-0.02179806	-0.00781622	-0.00684819
214	-0.02269357	-0.00929351	-0.00694264
215	-0.02340306	-0.01076302	-0.00724541
216	-0.02389958	-0.01220369	-0.00778537
217	-0.02423287	-0.01365489	-0.00854983
218	-0.02439367	-0.01512932	-0.00955153
219	-0.02446461	-0.01665275	-0.01071370
220	-0.02301658	-0.01983875	-0.01657561
221	-0.02449962	-0.02103703	-0.01580409
222	-0.02591209	-0.02210145	-0.01482174
223	-0.02725720	-0.02308326	-0.01371343
224	-0.02855787	-0.02398036	-0.01249724
225	-0.02979391	-0.02477324	-0.01114796
226	-0.03097478	-0.02545093	-0.00967183
227	-0.03210955	-0.02607016	-0.00809480
228	-0.03319008	-0.02659988	-0.00638774
229	-0.03426191	-0.02708908	-0.00460141
230	-0.03533030	-0.02742900	-0.00265247
231	-0.03638312	-0.02766692	-0.00077013
232	-0.03734717	-0.02770520	0.00110545
233	-0.03826857	-0.02760746	0.00292934
234	-0.03908166	-0.02730438	0.00475011
235	-0.03985656	-0.02685237	0.00652661
236	-0.04056376	-0.02621526	0.00828375
237	-0.04119816	-0.02541774	0.01001183
238	-0.04177051	-0.02445017	0.01171237
239	-0.04234573	-0.02326985	0.01357121
240	-0.04280543	-0.02204921	0.01527783
241	-0.04307339	-0.02075208	0.01680627
242	-0.04316928	-0.01938416	0.01818845
243	-0.04309833	-0.01796702	0.01938745
244	-0.04288469	-0.01648776	0.02045101
245	-0.04251190	-0.01496819	0.02134714
246	-0.04194658	-0.01335978	0.02208120
247	-0.04122537	-0.01171653	0.02264389
248	-0.04013103	-0.00977920	0.02317404
249	-0.03891342	-0.00789103	0.02339685
250	-0.03757997	-0.00603269	0.02333239
251	-0.03612316	-0.00421851	0.02298515
252	-0.03457502	-0.00244218	0.02236291
253	-0.03298520	-0.00059142	0.02156995
254	-0.03104639	0.00094796	0.02023291

255	-0.02905013	0.00254424	0.01870258
256	-0.02719004	0.00426947	0.01701131
257	-0.01420492	-0.00895315	0.00083756
258	-0.01362852	-0.00923638	0.00155711
259	-0.01293438	-0.00949925	0.00213629
260	-0.01213240	-0.00968167	0.00251142
261	-0.01129125	-0.00982879	0.00280732
262	-0.01045304	-0.01007004	0.00295865
263	-0.00950672	-0.01035670	0.00294027
264	-0.00854636	-0.01069007	0.00289594
265	-0.00740862	-0.01091971	0.00315727
266	-0.00644379	-0.01139151	0.00217907
267	-0.00519335	-0.01148261	0.00169384
268	-0.00408083	-0.01161007	0.00118547
269	-0.00317717	-0.01171258	0.00058195
270	-0.00244467	-0.01181043	-0.00011340
271	-0.00186863	-0.01198125	-0.00087273
272	-0.00139618	-0.01214156	-0.00173581
273	-0.00106708	-0.01221241	-0.00271789
274	-0.00097424	-0.01225575	-0.00380308
275	-0.00109211	-0.01226381	-0.00495813
276	-0.00181558	-0.01255486	-0.00585019
277	-0.00265436	-0.01267661	-0.00655561
278	-0.00352794	-0.01272289	-0.00709354
279	-0.00444229	-0.01278634	-0.00742448
280	-0.00539185	-0.01279159	-0.00757620
281	-0.00639964	-0.01275603	-0.00755145
282	-0.00743214	-0.01262899	-0.00732805
283	-0.00859772	-0.01256264	-0.00696748
284	-0.00973407	-0.01239425	-0.00642912
285	-0.01078608	-0.01202259	-0.00585542
286	-0.01173719	-0.01165365	-0.00519610
287	-0.01251652	-0.01124299	-0.00448940
288	-0.01318365	-0.01085834	-0.00376242
289	-0.01370455	-0.01048911	-0.00298549
290	-0.01408853	-0.01012381	-0.00212894
291	-0.01431493	-0.00976411	-0.00119754
292	-0.01435066	-0.00937240	-0.00019292
293	0.02587333	0.01926473	0.01617969
294	0.02816657	0.01805224	0.01850146
295	0.03003528	0.01703775	0.02018269
296	0.03267677	0.01459136	0.02173532
297	0.03362073	0.01306809	0.02168953
298	0.03446001	0.01126160	0.02116429
299	0.03496819	0.00901716	0.01983755
300	0.03707364	0.01699636	-0.01060991
301	0.03923838	0.01638373	-0.01093459
302	0.04097023	0.01570535	-0.01111726
303	0.04219610	0.01478278	-0.01105536
304	0.04350500	0.01254245	-0.01048141
305	0.04375876	0.01115959	-0.00997934
306	0.04362935	0.00951276	-0.00926276
307	0.02070917	0.00418236	-0.01852877
308	0.02123998	0.00252670	-0.01979348
309	0.02187883	0.00107518	-0.02051511
310	0.02256028	-0.00014793	-0.02081252
311	0.02411657	-0.00211887	-0.02029710
312	0.02503308	-0.00299290	-0.01955280
313	0.02608090	-0.00378639	-0.01840111
314	0.03185011	0.00463990	0.01061681

315	0.03345782	0.00346569	0.01181123
316	0.03461084	0.00242664	0.01257250
317	0.03548554	0.00143281	0.01291878
318	0.03657419	-0.00059275	0.01250653
319	0.03685028	-0.00172227	0.01179215
320	0.03695813	-0.00299166	0.01069417
321	0.03846695	0.00676464	-0.00647510
322	0.04109393	0.00486686	-0.00675629
323	0.04276408	0.00408199	-0.00718175
324	0.04411850	0.00221221	-0.00702164
325	0.04422334	0.00123872	-0.00674590
326	0.04409352	0.00014020	-0.00634048
327	0.04365107	-0.00119541	-0.00575169
328	0.02812671	-0.00288162	-0.01147439
329	0.02834146	-0.00458265	-0.01324353
330	0.02884814	-0.00600743	-0.01412604
331	0.02997272	-0.00791211	-0.01445270
332	0.03060502	-0.00858125	-0.01405170
333	0.03135400	-0.00917384	-0.01333860
334	0.03225444	-0.00968688	-0.01218141
335	-0.01172028	-0.00127986	0.01812014
336	-0.00981966	-0.00017310	0.01596653
337	-0.00748292	0.00135292	0.01318677
338	-0.00467901	0.00176626	0.01257339
339	-0.00160492	0.00166516	0.01345472
340	0.00073520	0.00261184	0.01288804
341	0.00309907	0.00379113	0.01238177
342	0.00544885	0.00525394	0.01189193
343	0.00775190	0.00691334	0.01155331
344	0.01000933	0.00859471	0.01167153
345	0.01159026	0.00924354	0.01172904
346	0.01313523	0.00992465	0.01169330
347	0.01462754	0.01061938	0.01153682
348	0.01607257	0.01123832	0.01130270
349	0.01746758	0.01184949	0.01094656
350	0.01883896	0.01248093	0.01049223
351	0.02019423	0.01308261	0.00995225
352	0.02149643	0.01371791	0.00923701
353	0.02272847	0.01433868	0.00841663
354	0.02481547	0.01491058	0.00685853
355	0.02656942	0.01530765	0.00518982
356	0.02808847	0.01552710	0.00350573
357	0.02930815	0.01554265	0.00175550
358	0.03028629	0.01537772	-0.00000438
359	0.03107232	0.01504573	-0.00178924
360	0.03160693	0.01450721	-0.00360661
361	0.03177200	0.01365817	-0.00540187
362	0.03160557	0.01293630	-0.00744600
363	0.03106170	0.01175631	-0.00900205
364	0.03038092	0.01111919	-0.01031657
365	0.02952562	0.01019383	-0.01133570
366	0.02853786	0.00936300	-0.01204035
367	0.02748432	0.00852688	-0.01249330
368	0.02632847	0.00767429	-0.01272849
369	0.02507221	0.00679246	-0.01271952
370	0.02370023	0.00589785	-0.01246631
371	0.02219163	0.00511297	-0.01183295
372	0.02060253	0.00332031	-0.00966574
373	0.01936455	0.00241102	-0.00757266
374	0.01871548	0.00159697	-0.00553696

375	0.01852484	0.00115230	-0.00365728
376	0.01884631	0.00065988	-0.00185211
377	0.01974608	0.00010857	-0.00008481
378	0.02094316	-0.00003790	0.00176350
379	0.02291868	0.00010279	0.00337593
380	0.02528540	0.00115883	0.00421022
381	0.02774686	0.00133632	0.00399910
382	0.02960979	0.00217368	0.00353866
383	0.03088601	0.00273921	0.00281149
384	0.03169197	0.00299553	0.00187416
385	0.03231657	0.00294339	0.00088867
386	0.03279191	0.00259206	-0.00014395
387	0.03321028	0.00185201	-0.00142195
388	0.03280723	0.00113424	-0.00303939
389	0.03138479	0.00071611	-0.00423056
390	0.03074004	-0.00050220	-0.00429013
391	0.02978969	-0.00125837	-0.00387234
392	0.02934388	-0.00169521	-0.00329803
393	0.02954951	-0.00213744	-0.00293272
394	0.03011139	-0.00272617	-0.00249838
395	0.03123281	-0.00373416	-0.00218899
396	0.03270543	-0.00474862	-0.00183261
397	0.03581692	-0.00584704	-0.00153825
398	0.00175150	0.01384106	0.01986431
399	0.00395592	0.01323955	0.01977547
400	0.00636728	0.01320706	0.01958638
401	0.00877127	0.01345148	0.01923816
402	0.01114184	0.01399185	0.01872838
403	0.01349436	0.01464576	0.01796054
404	0.01589219	0.01557216	0.01697272
405	0.01835448	0.01668598	0.01584368
406	0.02091562	0.01787523	0.01456537
407	0.02362854	0.01904545	0.01310844
408	0.02615073	0.01994385	0.01081755
409	0.02836713	0.02052226	0.00846297
410	0.03029835	0.02075309	0.00605305
411	0.03188309	0.02071802	0.00358688
412	0.03312215	0.02044034	0.00105844
413	0.03402449	0.01992269	-0.00155966
414	0.03459416	0.01916495	-0.00424869
415	0.03481121	0.01814049	-0.00701032
416	0.03466972	0.01691722	-0.00986995
417	0.03359050	0.01554510	-0.01201557
418	0.03238080	0.01422233	-0.01382808
419	0.03102669	0.01296553	-0.01530783
420	0.02957444	0.01170741	-0.01642472
421	0.02800219	0.01049636	-0.01719415
422	0.02631005	0.00928329	-0.01761252
423	0.02448131	0.00808574	-0.01767455
424	0.02254429	0.00687127	-0.01737555
425	0.02058327	0.00558651	-0.01669829
426	0.01785690	0.00399632	-0.01437669
427	0.01585703	0.00261997	-0.01195234
428	0.01453051	0.00146287	-0.00946739
429	0.01384650	0.00056285	-0.00691465
430	0.01375780	-0.00001375	-0.00427298
431	0.01440182	-0.00041373	-0.00156825
432	0.01576558	-0.00059723	0.00122092
433	0.01780654	-0.00054486	0.00410058
434	0.02045436	-0.00015461	0.00707657

435	0.02296861	0.00095982	0.00803691
436	0.02522707	0.00206162	0.00864778
437	0.02727785	0.00306918	0.00889625
438	0.02911712	0.00395986	0.00878205
439	0.03075297	0.00470902	0.00828189
440	0.03219306	0.00534578	0.00740889
441	0.03341590	0.00592685	0.00616181
442	0.03451745	0.00625965	0.00452302
443	0.03543575	0.00641256	0.00252476
444	0.03585532	0.00629171	0.00071914
445	0.03609805	0.00604791	-0.00094565
446	0.03618289	0.00573616	-0.00249520
447	0.03608863	0.00534430	-0.00390653
448	0.03584725	0.00484086	-0.00518997
449	0.03540758	0.00424762	-0.00634779
450	0.03479334	0.00358544	-0.00738212
451	0.03401438	0.00281988	-0.00829128
452	0.03307100	0.00195507	-0.00905581
453	0.03197989	0.00104425	-0.00949076
454	0.03092847	0.00026206	-0.00970118
455	0.02998050	-0.00049538	-0.00966793
456	0.02907338	-0.00114972	-0.00940987
457	0.02824415	-0.00176644	-0.00889215
458	0.02748598	-0.00234658	-0.00810671
459	0.02687795	-0.00295596	-0.00702761
460	0.02643400	-0.00358347	-0.00567919
461	0.02622359	-0.00427971	-0.00412512
462	0.02697931	-0.00414388	-0.00228177
463	0.02783022	-0.00416996	-0.00083607
464	0.02872124	-0.00424154	0.00027241
465	0.02970001	-0.00433671	0.00101924
466	0.03066197	-0.00426093	0.00164923
467	0.03189605	-0.00440755	0.00162636
468	0.03374501	-0.00469686	0.00105143
469	0.03562753	-0.00567469	-0.00040085
470	-0.01405999	-0.00448353	0.01199846
471	-0.01327624	-0.00615359	0.01155577
472	-0.01218013	-0.00765257	0.01108889
473	-0.01089008	-0.00894656	0.01074745
474	-0.00952228	-0.01014643	0.01065119
475	-0.00802525	-0.01122904	0.01074947
476	-0.00648290	-0.01228996	0.01106501
477	-0.00526464	-0.01342736	0.01160294
478	-0.00284469	-0.01445149	0.01218824
479	-0.00235071	-0.01505638	0.01362634
480	-0.00057922	-0.01551150	0.01468553
481	0.00112223	-0.01579763	0.01578951
482	0.00277592	-0.01587528	0.01692713
483	0.00435767	-0.01575872	0.01807645
484	0.00588584	-0.01544568	0.01926245
485	0.00736047	-0.01496314	0.02049256
486	0.00878267	-0.01424004	0.02174220
487	0.01016040	-0.01325788	0.02300620
488	0.01150151	-0.01205326	0.02424520
489	0.01272474	-0.01063365	0.02541270
490	0.01394615	-0.00912507	0.02639944
491	0.01517522	-0.00756413	0.02728012
492	0.01643602	-0.00591284	0.02795602
493	0.01767138	-0.00416505	0.02847710
494	0.01893487	-0.00230851	0.02881471

495	0.02021383	-0.00038022	0.02899355
496	0.02150517	0.00165100	0.02897907
497	0.02279615	0.00377396	0.02871005
498	0.02479946	0.00683549	0.02788249
499	0.02678725	0.00955162	0.02661579
500	0.02876705	0.01190745	0.02497364
501	0.03075035	0.01392402	0.02302300
502	0.03276864	0.01564003	0.02077317
503	0.03480122	0.01701378	0.01814617
504	0.03685017	0.01804085	0.01513511
505	0.03885990	0.01867420	0.01169836
506	0.04071137	0.01880678	0.00783131
507	0.04234804	0.01842942	0.00319138
508	0.04319392	0.01758835	-0.00120791
509	0.04329672	0.01643156	-0.00516137
510	0.04283799	0.01510102	-0.00863886
511	0.04191714	0.01363323	-0.01167814
512	0.04058038	0.01206258	-0.01437005
513	0.03881765	0.01036889	-0.01677530
514	0.03653839	0.00849577	-0.01883607
515	0.03369222	0.00641862	-0.02043009
516	0.03121893	0.00480826	-0.02122917
517	0.02881560	0.00326752	-0.02153818
518	0.02653170	0.00180376	-0.02142990
519	0.02438529	0.00042885	-0.02093315
520	0.02235112	-0.00087884	-0.02012587
521	0.02043778	-0.00211728	-0.01893960
522	0.01862180	-0.00329883	-0.01740436
523	0.01691967	-0.00439601	-0.01547014
524	0.01539708	-0.00537614	-0.01311932
525	0.01436649	-0.00635815	-0.01077700
526	0.01369976	-0.00717745	-0.00841675
527	0.01337480	-0.00782977	-0.00607145
528	0.01334378	-0.00834772	-0.00375622
529	0.01362223	-0.00870699	-0.00147973
530	0.01416921	-0.00895092	0.00078057
531	0.01500797	-0.00907364	0.00304707
532	0.01619837	-0.00902670	0.00527835
533	0.01774937	-0.00877777	0.00743715
534	0.01943712	-0.00824749	0.00936803
535	0.02124334	-0.00752661	0.01093038
536	0.02308198	-0.00671600	0.01217647
537	0.02497056	-0.00579067	0.01311554
538	0.02693106	-0.00470307	0.01367410
539	0.02893472	-0.00360428	0.01407307
540	0.03101090	-0.00229582	0.01403004
541	0.03316389	-0.00088888	0.01367675
542	0.03533969	0.00060278	0.01283318
543	0.03767007	0.00181293	0.01145107
544	0.03961497	0.00276158	0.00973062
545	0.04116412	0.00344369	0.00784756
546	0.04240633	0.00390778	0.00583676
547	0.04334771	0.00412907	0.00370463
548	0.04403160	0.00415911	0.00139968
549	0.04438067	0.00396289	-0.00110620
550	0.04430687	0.00347775	-0.00380985
551	0.04372093	0.00269044	-0.00660298
552	0.04285048	0.00178208	-0.00882628
553	0.04174237	0.00079350	-0.01068087
554	0.04049535	-0.00023208	-0.01218732

555	0.03909850	-0.00130016	-0.01334704
556	0.03759087	-0.00240096	-0.01421729
557	0.03592539	-0.00354340	-0.01477669
558	0.03411289	-0.00474780	-0.01505795
559	0.03211970	-0.00599507	-0.01494660
560	0.03003332	-0.00725745	-0.01433042
561	0.02818582	-0.00857227	-0.01319741
562	0.02673746	-0.00964840	-0.01175889
563	0.02568626	-0.01052689	-0.01021597
564	0.02497676	-0.01122878	-0.00860865
565	0.02455074	-0.01182021	-0.00692180
566	0.02440537	-0.01230005	-0.00512194
567	0.02459443	-0.01265729	-0.00317997
568	0.02522170	-0.01269135	-0.00109534
569	0.02616218	-0.01232520	0.00112710
570	0.02799792	-0.01164154	0.00307416
571	0.02985354	-0.01086215	0.00428979
572	0.03149247	-0.01011763	0.00487646
573	0.03286284	-0.00952532	0.00507522
574	0.03415731	-0.00902859	0.00498691
575	0.03571407	-0.00844504	0.00434290
576	0.03719703	-0.00815875	0.00316565
577	0.03796199	-0.00783864	0.00091595
578	-0.00472278	-0.00591580	-0.01389955
579	-0.00282152	-0.00534411	-0.01307654
580	-0.00077831	-0.00526381	-0.01217037
581	0.00097025	-0.00534827	-0.01103326
582	0.00242991	-0.00558909	-0.00963486
583	0.00351485	-0.00586547	-0.00795511
584	0.00433010	-0.00629561	-0.00603422
585	0.00486325	-0.00686536	-0.00381865
586	0.00519077	-0.00760052	-0.00137754
587	0.00556083	-0.00865338	0.00112401
588	0.00646828	-0.01013170	0.00343617
589	0.00783613	-0.01149684	0.00551644
590	0.00930897	-0.01254350	0.00744219
591	0.01084713	-0.01319676	0.00926903
592	0.01241892	-0.01342319	0.01104797
593	0.01408775	-0.01328703	0.01276525
594	0.01583373	-0.01277122	0.01443718
595	0.01763102	-0.01183438	0.01607692
596	0.01947566	-0.01048279	0.01764952
597	0.02140151	-0.00861850	0.01893748
598	0.02317182	-0.00663277	0.01995184
599	0.02480619	-0.00457297	0.02065572
600	0.02639007	-0.00251694	0.02101676
601	0.02795184	-0.00048046	0.02101025
602	0.02950971	0.00154632	0.02063967
603	0.03113609	0.00352040	0.01987287
604	0.03288489	0.00539669	0.01874626
605	0.03474481	0.00711491	0.01730788
606	0.03686200	0.00862248	0.01474500
607	0.03872174	0.00971562	0.01211694
608	0.04029211	0.01043822	0.00941802
609	0.04158210	0.01077047	0.00664789
610	0.04252638	0.01077335	0.00380530
611	0.04314034	0.01041931	0.00085880
612	0.04337970	0.00977831	-0.00218957
613	0.04322285	0.00884295	-0.00533685
614	0.04260484	0.00769363	-0.00858368

615	0.04161695	0.00644682	-0.01099706
616	0.04035395	0.00523362	-0.01302552
617	0.03893777	0.00397600	-0.01467091
618	0.03733841	0.00270040	-0.01593965
619	0.03557586	0.00137918	-0.01681177
620	0.03364114	0.00002475	-0.01730930
621	0.03156233	-0.00139408	-0.01743314
622	0.02931854	-0.00288202	-0.01718814
623	0.02690239	-0.00442226	-0.01654399
624	0.02483326	-0.00585611	-0.01531786
625	0.02310872	-0.00708084	-0.01384842
626	0.02174057	-0.00812270	-0.01217845
627	0.02073371	-0.00900456	-0.01031061
628	0.02008726	-0.00970652	-0.00824582
629	0.01984304	-0.01036388	-0.00598328
630	0.01997215	-0.01087333	-0.00350758
631	0.02047542	-0.01124309	-0.00083346
632	0.02133162	-0.01140695	0.00203385
633	0.02270881	-0.01122814	0.00426263
634	0.02414832	-0.01080825	0.00614223
635	0.02564284	-0.01017136	0.00763857
636	0.02724265	-0.00941665	0.00876399
637	0.02893980	-0.00857386	0.00952246
638	0.03074246	-0.00759938	0.00990687
639	0.03266598	-0.00653779	0.00993561
640	0.03473008	-0.00542265	0.00962073
641	0.03688195	-0.00415521	0.00892264
642	0.03844788	-0.00340503	0.00785551
643	0.03978015	-0.00279195	0.00666462
644	0.04087124	-0.00232931	0.00534496
645	0.04173090	-0.00202117	0.00390837
646	0.04236048	-0.00186649	0.00235601
647	0.04276548	-0.00186371	0.00067766
648	0.04297459	-0.00204918	-0.00111624
649	0.04290986	-0.00235173	-0.00304896
650	0.04257675	-0.00277653	-0.00509931
651	0.04199268	-0.00337785	-0.00662625
652	0.04128121	-0.00403213	-0.00792095
653	0.04042464	-0.00471125	-0.00897988
654	0.03946348	-0.00545763	-0.00981281
655	0.03840840	-0.00624549	-0.01039310
656	0.03724789	-0.00710525	-0.01074771
657	0.03598539	-0.00803769	-0.01087718
658	0.03462609	-0.00903747	-0.01077708
659	0.03312538	-0.01004584	-0.01042879
660	0.03197561	-0.01098321	-0.00948970
661	0.03103462	-0.01173975	-0.00848024
662	0.03033323	-0.01236701	-0.00741586
663	0.02986373	-0.01285649	-0.00629548
664	0.02963806	-0.01321170	-0.00513194
665	0.02963912	-0.01344038	-0.00390085
666	0.02985399	-0.01349280	-0.00262084
667	0.03031734	-0.01340983	-0.00128204
668	0.03100931	-0.01318364	0.00011827
669	0.03213870	-0.01275362	0.00090662
670	0.03314645	-0.01225541	0.00146206
671	0.03410651	-0.01180367	0.00179509
672	0.03498578	-0.01133088	0.00191744
673	0.03582410	-0.01084690	0.00182995
674	0.03664555	-0.01030458	0.00150342

675	0.03744968	-0.00965418	0.00083948
676	0.03793364	-0.00861659	-0.00017299
677	-0.00929247	0.01399275	-0.00004503
678	-0.01283930	0.01696123	-0.00155635
679	-0.01665438	0.01954362	-0.00296851
680	-0.02025991	0.02198157	-0.00416642
681	-0.02350131	0.02441735	-0.00522117
682	-0.02664614	0.02660005	-0.00607178
683	-0.02988727	0.02846567	-0.00678561
684	-0.03277505	0.03017638	-0.00712933
685	-0.03566587	0.03171160	-0.00735360
686	-0.03850607	0.03290508	-0.00743352

Procrustes sums of squares: 0.3657145180346275
Tangent sums of squares: 0.35793716368340484

Data matrices in this dataset:
- Evolution Cervidae final, raw data
- Sans les Indet., centroid size
- Sans les Indet., Procrustes coordinates

New Procrustes fit:
Dataset 'Sans les Indet.'

686 landmarks in 3 dimensions.
The dataset contains 40 observations, of which 36 are included for analyses.

Average shape:

Lmk.	Axis 1 (x)	Axis 2 (y)	Axis 3 (z)
1	-0.04036362	0.02592451	-0.00811150
2	-0.02486511	-0.00981714	-0.02627340
3	-0.02630368	-0.01137480	-0.02731431
4	-0.02818995	-0.01223046	-0.02847610
5	-0.03017741	-0.01259941	-0.02952329
6	-0.03220235	-0.01261672	-0.03047543
7	-0.03431632	-0.01234278	-0.03134544
8	-0.03656948	-0.01172608	-0.03215652
9	-0.03897484	-0.01071316	-0.03289171
10	-0.04144477	-0.00924722	-0.03353796
11	-0.04391420	-0.00725860	-0.03406058
12	-0.04660985	-0.00439912	-0.03449515
13	-0.04870323	-0.00138086	-0.03453497
14	-0.05020484	0.00168057	-0.03420577
15	-0.05127422	0.00468345	-0.03352928
16	-0.05194588	0.00763278	-0.03255234
17	-0.05218638	0.01059840	-0.03122656
18	-0.05198752	0.01357426	-0.02955756
19	-0.05126707	0.01655038	-0.02751849
20	-0.04987035	0.01940290	-0.02515643
21	-0.04786808	0.02164297	-0.02204558
22	-0.04544731	0.02307481	-0.01919084
23	-0.04287669	0.02382276	-0.01656435
24	-0.04021532	0.02392447	-0.01415795
25	-0.03749705	0.02347117	-0.01185990
26	-0.03451674	0.02226598	-0.00987187
27	-0.03131650	0.02036366	-0.00814552

28	-0.02795494	0.01785428	-0.00653419
29	-0.02446861	0.01459152	-0.00530442
30	-0.02274492	0.01293029	-0.00496445
31	-0.02130127	0.01127595	-0.00504291
32	-0.02018054	0.00965077	-0.00552535
33	-0.01937887	0.00805876	-0.00639792
34	-0.01887320	0.00649494	-0.00763445
35	-0.01863959	0.00493692	-0.00923185
36	-0.01861014	0.00336333	-0.01114849
37	-0.01881318	0.00170255	-0.01342681
38	-0.01950246	0.00028609	-0.01612496
39	-0.02051828	-0.00009089	-0.01770052
40	-0.02170450	-0.00033282	-0.01922434
41	-0.02269031	-0.00088248	-0.02062383
42	-0.02347663	-0.00171594	-0.02187741
43	-0.02404150	-0.00284644	-0.02298066
44	-0.02444990	-0.00424358	-0.02398036
45	-0.02463759	-0.00594249	-0.02481416
46	-0.02463999	-0.00790540	-0.02549130
47	-0.01971082	-0.01429760	-0.02654986
48	-0.02207603	-0.01533880	-0.02788349
49	-0.02452514	-0.01601771	-0.02907491
50	-0.02697985	-0.01635938	-0.03012478
51	-0.02938783	-0.01643944	-0.03108082
52	-0.03172828	-0.01627283	-0.03197942
53	-0.03405324	-0.01585177	-0.03278889
54	-0.03635762	-0.01517846	-0.03352073
55	-0.03864152	-0.01423175	-0.03417591
56	-0.04088868	-0.01300501	-0.03472953
57	-0.04298360	-0.01153854	-0.03527308
58	-0.04489822	-0.00994488	-0.03566984
59	-0.04665507	-0.00824501	-0.03596318
60	-0.04822977	-0.00641306	-0.03612856
61	-0.04969089	-0.00450700	-0.03623035
62	-0.05095120	-0.00244136	-0.03614721
63	-0.05204945	-0.00025523	-0.03596464
64	-0.05297543	0.00205276	-0.03560948
65	-0.05371134	0.00447999	-0.03512109
66	-0.05419957	0.00667575	-0.03445464
67	-0.05451852	0.00881152	-0.03371248
68	-0.05466005	0.01088919	-0.03286446
69	-0.05459579	0.01289528	-0.03188451
70	-0.05437111	0.01483793	-0.03081159
71	-0.05400202	0.01676676	-0.02966597
72	-0.05338614	0.01857604	-0.02833334
73	-0.05259955	0.02033943	-0.02692452
74	-0.05160894	0.02199484	-0.02538766
75	-0.05032088	0.02348538	-0.02386987
76	-0.04889424	0.02477045	-0.02222484
77	-0.04734848	0.02583566	-0.02045486
78	-0.04568356	0.02668656	-0.01854892
79	-0.04389958	0.02729224	-0.01651489
80	-0.04196674	0.02768918	-0.01436050
81	-0.03993372	0.02788108	-0.01206327
82	-0.03778278	0.02786411	-0.00962310
83	-0.03550002	0.02805675	-0.00713649
84	-0.02475516	0.00500360	0.02164341
85	-0.02663734	0.00345875	0.02300711
86	-0.02893429	0.00246871	0.02432777
87	-0.03125222	0.00179496	0.02541824

88	-0.03351854	0.00126713	0.02624892
89	-0.03576266	0.00091725	0.02689810
90	-0.03801045	0.00073757	0.02735358
91	-0.04026958	0.00071849	0.02765156
92	-0.04255395	0.00091244	0.02778557
93	-0.04484174	0.00133713	0.02770046
94	-0.04746711	0.00203698	0.02715615
95	-0.04978796	0.00300011	0.02632534
96	-0.05182017	0.00415271	0.02524752
97	-0.05352649	0.00550637	0.02392747
98	-0.05500355	0.00695445	0.02240050
99	-0.05624612	0.00856683	0.02065234
100	-0.05722574	0.01032876	0.01866341
101	-0.05794862	0.01226252	0.01643623
102	-0.05829858	0.01437348	0.01400590
103	-0.05820941	0.01695260	0.01097585
104	-0.05748421	0.01920360	0.00826470
105	-0.05622342	0.02109539	0.00589852
106	-0.05456535	0.02262365	0.00381355
107	-0.05245204	0.02378773	0.00203401
108	-0.04992802	0.02461653	0.00047559
109	-0.04691429	0.02511807	-0.00083014
110	-0.04338655	0.02524868	-0.00180378
111	-0.03939899	0.02500765	-0.00219625
112	-0.03571669	0.02334225	-0.00172767
113	-0.03242682	0.02173363	-0.00074659
114	-0.02951264	0.02020953	0.00028187
115	-0.02694310	0.01876207	0.00131585
116	-0.02471578	0.01734922	0.00240066
117	-0.02278449	0.01593180	0.00362812
118	-0.02116758	0.01449232	0.00516745
119	-0.02005743	0.01321894	0.00717437
120	-0.01982002	0.01265052	0.00928625
121	-0.02048050	0.01276028	0.01082980
122	-0.02140577	0.01295381	0.01235614
123	-0.02221766	0.01279049	0.01383239
124	-0.02290441	0.01229057	0.01525892
125	-0.02344077	0.01132780	0.01664227
126	-0.02388330	0.01016281	0.01795521
127	-0.02417959	0.00871239	0.01919274
128	-0.02428154	0.00676757	0.02037126
129	-0.01889190	0.00302862	0.02394466
130	-0.02110260	0.00214561	0.02522712
131	-0.02349788	0.00139899	0.02617422
132	-0.02586912	0.00076067	0.02701963
133	-0.02819728	0.00019760	0.02778945
134	-0.03049387	-0.00028099	0.02849185
135	-0.03278938	-0.00066872	0.02911548
136	-0.03508840	-0.00096192	0.02966257
137	-0.03739207	-0.00114914	0.03004513
138	-0.03968475	-0.00124508	0.03031424
139	-0.04199260	-0.00107408	0.03043188
140	-0.04419000	-0.00078425	0.03037969
141	-0.04626033	-0.00034011	0.03007118
142	-0.04822308	0.00018904	0.02966283
143	-0.05005434	0.00084689	0.02904747
144	-0.05177324	0.00165080	0.02827374
145	-0.05339725	0.00256569	0.02734367
146	-0.05488949	0.00361482	0.02622050
147	-0.05627996	0.00479674	0.02496187

148	-0.05780268	0.00638624	0.02325633
149	-0.05900770	0.00801934	0.02146877
150	-0.05993875	0.00967549	0.01961293
151	-0.06065928	0.01137019	0.01772820
152	-0.06111308	0.01310885	0.01578557
153	-0.06132017	0.01486941	0.01377255
154	-0.06123036	0.01664026	0.01167593
155	-0.06084204	0.01843626	0.00949995
156	-0.06010631	0.02023352	0.00726995
157	-0.05876156	0.02231800	0.00499395
158	-0.05702064	0.02406532	0.00291125
159	-0.05495415	0.02551484	0.00103142
160	-0.05263172	0.02673985	-0.00076846
161	-0.04991975	0.02759773	-0.00236395
162	-0.04685180	0.02816012	-0.00379531
163	-0.04340435	0.02836941	-0.00505424
164	-0.03957295	0.02841727	-0.00620094
165	-0.03550002	0.02805675	-0.00713649
166	-0.02499274	-0.01797812	-0.01144966
167	-0.02594114	-0.01926394	-0.01127705
168	-0.02715963	-0.02032120	-0.01063659
169	-0.02835612	-0.02123163	-0.00977690
170	-0.02953381	-0.02203539	-0.00869693
171	-0.03072663	-0.02273138	-0.00741166
172	-0.03191706	-0.02340512	-0.00589307
173	-0.03311180	-0.02396404	-0.00412300
174	-0.03431553	-0.02442274	-0.00211313
175	-0.03556056	-0.02468571	0.00012660
176	-0.03649211	-0.02473268	0.00178126
177	-0.03733966	-0.02465161	0.00340695
178	-0.03820179	-0.02302148	0.00481856
179	-0.03880320	-0.02403014	0.00653744
180	-0.03941172	-0.02351355	0.00803519
181	-0.03995039	-0.02286350	0.00951174
182	-0.04039727	-0.02207565	0.01095243
183	-0.04079079	-0.02116340	0.01237129
184	-0.04109684	-0.02011810	0.01374697
185	-0.04130890	-0.01876998	0.01527170
186	-0.04134585	-0.01740235	0.01661532
187	-0.04122223	-0.01601967	0.01784617
188	-0.04086897	-0.01457793	0.01871371
189	-0.04035602	-0.01312194	0.01951769
190	-0.03964819	-0.01161107	0.02003250
191	-0.03873596	-0.01005176	0.02032875
192	-0.03763316	-0.00846149	0.02042424
193	-0.03633865	-0.00682257	0.02029050
194	-0.03442252	-0.00477936	0.01989045
195	-0.03261396	-0.00294886	0.01921052
196	-0.03090104	-0.00137572	0.01823817
197	-0.02927633	-0.00002425	0.01701181
198	-0.02777946	0.00114102	0.01557634
199	-0.02638943	0.00209796	0.01386781
200	-0.02507999	0.00277584	0.01189396
201	-0.02383005	0.00335102	0.00971822
202	-0.02268630	0.00367457	0.00728109
203	-0.02198686	0.00350864	0.00526205
204	-0.02150943	0.00305574	0.00328699
205	-0.02109940	0.00246504	0.00146270
206	-0.02070629	0.00172063	-0.00021211
207	-0.02031526	0.00079952	-0.00175701

208	-0.02001998	-0.00032600	-0.00321626
209	-0.01977998	-0.00165156	-0.00460495
210	-0.01972435	-0.00318700	-0.00591164
211	-0.02012169	-0.00482420	-0.00683380
212	-0.02086359	-0.00632844	-0.00688795
213	-0.02179806	-0.00781622	-0.00684819
214	-0.02269357	-0.00929351	-0.00694264
215	-0.02340306	-0.01076302	-0.00724541
216	-0.02389958	-0.01220369	-0.00778537
217	-0.02423287	-0.01365489	-0.00854983
218	-0.02439367	-0.01512932	-0.00955153
219	-0.02446461	-0.01665275	-0.01071370
220	-0.02301658	-0.01983875	-0.01657561
221	-0.02449962	-0.02103703	-0.01580409
222	-0.02591209	-0.02210145	-0.01482174
223	-0.02725720	-0.02308326	-0.01371343
224	-0.02855787	-0.02398036	-0.01249724
225	-0.02979391	-0.02477324	-0.01114796
226	-0.03097478	-0.02545093	-0.00967183
227	-0.03210955	-0.02607016	-0.00809480
228	-0.03319008	-0.02659988	-0.00638774
229	-0.03426191	-0.02708908	-0.00460141
230	-0.03533030	-0.02742900	-0.00265247
231	-0.03638312	-0.02766692	-0.00077013
232	-0.03734717	-0.02770520	0.00110545
233	-0.03826857	-0.02760746	0.00292934
234	-0.03908166	-0.02730438	0.00475011
235	-0.03985656	-0.02685237	0.00652661
236	-0.04056376	-0.02621526	0.00828375
237	-0.04119816	-0.02541774	0.01001183
238	-0.04177051	-0.02445017	0.01171237
239	-0.04234573	-0.02326985	0.01357121
240	-0.04280543	-0.02204921	0.01527783
241	-0.04307339	-0.02075208	0.01680627
242	-0.04316928	-0.01938416	0.01818845
243	-0.04309833	-0.01796702	0.01938745
244	-0.04288469	-0.01648776	0.02045101
245	-0.04251190	-0.01496819	0.02134714
246	-0.04194658	-0.01335978	0.02208120
247	-0.04122537	-0.01171653	0.02264389
248	-0.04013103	-0.00977920	0.02317404
249	-0.03891342	-0.00789103	0.02339685
250	-0.03757997	-0.00603269	0.02333239
251	-0.03612316	-0.00421851	0.02298515
252	-0.03457502	-0.00244218	0.02236291
253	-0.03298520	-0.00059142	0.02156995
254	-0.03104639	0.00094796	0.02023291
255	-0.02905013	0.00254424	0.01870258
256	-0.02719004	0.00426947	0.01701131
257	-0.01420492	-0.00895315	0.00083756
258	-0.01362852	-0.00923638	0.00155711
259	-0.01293438	-0.00949925	0.00213629
260	-0.01213240	-0.00968167	0.00251142
261	-0.01129125	-0.00982879	0.00280732
262	-0.01045304	-0.01007004	0.00295865
263	-0.00950672	-0.01035670	0.00294027
264	-0.00854636	-0.01069007	0.00289594
265	-0.00740862	-0.01091971	0.00315727
266	-0.00644379	-0.01139151	0.00217907
267	-0.00519335	-0.01148261	0.00169384

268	-0.00408083	-0.01161007	0.00118547
269	-0.00317717	-0.01171258	0.00058195
270	-0.00244467	-0.01181043	-0.00011340
271	-0.00186863	-0.01198125	-0.00087273
272	-0.00139618	-0.01214156	-0.00173581
273	-0.00106708	-0.01221241	-0.00271789
274	-0.00097424	-0.01225575	-0.00380308
275	-0.00109211	-0.01226381	-0.00495813
276	-0.00181558	-0.01255486	-0.00585019
277	-0.00265436	-0.01267661	-0.00655561
278	-0.00352794	-0.01272289	-0.00709354
279	-0.00444229	-0.01278634	-0.00742448
280	-0.00539185	-0.01279159	-0.00757620
281	-0.00639964	-0.01275603	-0.00755145
282	-0.00743214	-0.01262899	-0.00732805
283	-0.00859772	-0.01256264	-0.00696748
284	-0.00973407	-0.01239425	-0.00642912
285	-0.01078608	-0.01202259	-0.00585542
286	-0.01173719	-0.01165365	-0.00519610
287	-0.01251652	-0.01124299	-0.00448940
288	-0.01318365	-0.01085834	-0.00376242
289	-0.01370455	-0.01048911	-0.00298549
290	-0.01408853	-0.01012381	-0.00212894
291	-0.01431493	-0.00976411	-0.00119754
292	-0.01435066	-0.00937240	-0.00019292
293	0.02587333	0.01926473	0.01617969
294	0.02816657	0.01805224	0.01850146
295	0.03003528	0.01703775	0.02018269
296	0.03267677	0.01459136	0.02173532
297	0.03362073	0.01306809	0.02168953
298	0.03446001	0.01126160	0.02116429
299	0.03496819	0.00901716	0.01983755
300	0.03707364	0.01699636	-0.01060991
301	0.03923838	0.01638373	-0.01093459
302	0.04097023	0.01570535	-0.01111726
303	0.04219610	0.01478278	-0.01105536
304	0.04350500	0.01254245	-0.01048141
305	0.04375876	0.01115959	-0.00997934
306	0.04362935	0.00951276	-0.00926276
307	0.02070917	0.00418236	-0.01852877
308	0.02123998	0.00252670	-0.01979348
309	0.02187883	0.00107518	-0.02051511
310	0.02256028	-0.00014793	-0.02081252
311	0.02411657	-0.00211887	-0.02029710
312	0.02503308	-0.00299290	-0.01955280
313	0.02608090	-0.00378639	-0.01840111
314	0.03185011	0.00463990	0.01061681
315	0.03345782	0.00346569	0.01181123
316	0.03461084	0.00242664	0.01257250
317	0.03548554	0.00143281	0.01291878
318	0.03657419	-0.00059275	0.01250653
319	0.03685028	-0.00172227	0.01179215
320	0.03695813	-0.00299166	0.01069417
321	0.03846695	0.00676464	-0.00647510
322	0.04109393	0.00486686	-0.00675629
323	0.04276408	0.00408199	-0.00718175
324	0.04411850	0.00221221	-0.00702164
325	0.04422334	0.00123872	-0.00674590
326	0.04409352	0.00014020	-0.00634048
327	0.04365107	-0.00119541	-0.00575169

328	0.02812671	-0.00288162	-0.01147439
329	0.02834146	-0.00458265	-0.01324353
330	0.02884814	-0.00600743	-0.01412604
331	0.02997272	-0.00791211	-0.01445270
332	0.03060502	-0.00858125	-0.01405170
333	0.03135400	-0.00917384	-0.01333860
334	0.03225444	-0.00968688	-0.01218141
335	-0.01172028	-0.00127986	0.01812014
336	-0.00981966	-0.00017310	0.01596653
337	-0.00748292	0.00135292	0.01318677
338	-0.00467901	0.00176626	0.01257339
339	-0.00160492	0.00166516	0.01345472
340	0.00073520	0.00261184	0.01288804
341	0.00309907	0.00379113	0.01238177
342	0.00544885	0.00525394	0.01189193
343	0.00775190	0.00691334	0.01155331
344	0.01000933	0.00859471	0.01167153
345	0.01159026	0.00924354	0.01172904
346	0.01313523	0.00992465	0.01169330
347	0.01462754	0.01061938	0.01153682
348	0.01607257	0.01123832	0.01130270
349	0.01746758	0.01184949	0.01094656
350	0.01883896	0.01248093	0.01049223
351	0.02019423	0.01308261	0.00995225
352	0.02149643	0.01371791	0.00923701
353	0.02272847	0.01433868	0.00841663
354	0.02481547	0.01491058	0.00685853
355	0.02656942	0.01530765	0.00518982
356	0.02808847	0.01552710	0.00350573
357	0.02930815	0.01554265	0.00175550
358	0.03028629	0.01537772	-0.00000438
359	0.03107232	0.01504573	-0.00178924
360	0.03160693	0.01450721	-0.00360661
361	0.03177200	0.01365817	-0.00540187
362	0.03160557	0.01293630	-0.00744600
363	0.03106170	0.01175631	-0.00900205
364	0.03038092	0.01111919	-0.01031657
365	0.02952562	0.01019383	-0.01133570
366	0.02853786	0.00936300	-0.01204035
367	0.02748432	0.00852688	-0.01249330
368	0.02632847	0.00767429	-0.01272849
369	0.02507221	0.00679246	-0.01271952
370	0.02370023	0.00589785	-0.01246631
371	0.02219163	0.00511297	-0.01183295
372	0.02060253	0.00332031	-0.00966574
373	0.01936455	0.00241102	-0.00757266
374	0.01871548	0.00159697	-0.00553696
375	0.01852484	0.00115230	-0.00365728
376	0.01884631	0.00065988	-0.00185211
377	0.01974608	0.00010857	-0.00008481
378	0.02094316	-0.00003790	0.00176350
379	0.02291868	0.00010279	0.00337593
380	0.02528540	0.00115883	0.00421022
381	0.02774686	0.00133632	0.00399910
382	0.02960979	0.00217368	0.00353866
383	0.03088601	0.00273921	0.00281149
384	0.03169197	0.00299553	0.00187416
385	0.03231657	0.00294339	0.00088867
386	0.03279191	0.00259206	-0.00014395
387	0.03321028	0.00185201	-0.00142195

388	0.03280723	0.00113424	-0.00303939
389	0.03138479	0.00071611	-0.00423056
390	0.03074004	-0.00050220	-0.00429013
391	0.02978969	-0.00125837	-0.00387234
392	0.02934388	-0.00169521	-0.00329803
393	0.02954951	-0.00213744	-0.00293272
394	0.03011139	-0.00272617	-0.00249838
395	0.03123281	-0.00373416	-0.00218899
396	0.03270543	-0.00474862	-0.00183261
397	0.03581692	-0.00584704	-0.00153825
398	0.00175150	0.01384106	0.01986431
399	0.00395592	0.01323955	0.01977547
400	0.00636728	0.01320706	0.01958638
401	0.00877127	0.01345148	0.01923816
402	0.01114184	0.01399185	0.01872838
403	0.01349436	0.01464576	0.01796054
404	0.01589219	0.01557216	0.01697272
405	0.01835448	0.01668598	0.01584368
406	0.02091562	0.01787523	0.01456537
407	0.02362854	0.01904545	0.01310844
408	0.02615073	0.01994385	0.01081755
409	0.02836713	0.02052226	0.00846297
410	0.03029835	0.02075309	0.00605305
411	0.03188309	0.02071802	0.00358688
412	0.03312215	0.02044034	0.00105844
413	0.03402449	0.01992269	-0.00155966
414	0.03459416	0.01916495	-0.00424869
415	0.03481121	0.01814049	-0.00701032
416	0.03466972	0.01691722	-0.00986995
417	0.03359050	0.01554510	-0.01201557
418	0.03238080	0.01422233	-0.01382808
419	0.03102669	0.01296553	-0.01530783
420	0.02957444	0.01170741	-0.01642472
421	0.02800219	0.01049636	-0.01719415
422	0.02631005	0.00928329	-0.01761252
423	0.02448131	0.00808574	-0.01767455
424	0.02254429	0.00687127	-0.01737555
425	0.02058327	0.00558651	-0.01669829
426	0.01785690	0.00399632	-0.01437669
427	0.01585703	0.00261997	-0.01195234
428	0.01453051	0.00146287	-0.00946739
429	0.01384650	0.00056285	-0.00691465
430	0.01375780	-0.00001375	-0.00427298
431	0.01440182	-0.00041373	-0.00156825
432	0.01576558	-0.00059723	0.00122092
433	0.01780654	-0.00054486	0.00410058
434	0.02045436	-0.00015461	0.00707657
435	0.02296861	0.00095982	0.00803691
436	0.02522707	0.00206162	0.00864778
437	0.02727785	0.00306918	0.00889625
438	0.02911712	0.00395986	0.00878205
439	0.03075297	0.00470902	0.00828189
440	0.03219306	0.00534578	0.00740889
441	0.03341590	0.00592685	0.00616181
442	0.03451745	0.00625965	0.00452302
443	0.03543575	0.00641256	0.00252476
444	0.03585532	0.00629171	0.00071914
445	0.03609805	0.00604791	-0.00094565
446	0.03618289	0.00573616	-0.00249520
447	0.03608863	0.00534430	-0.00390653

448	0.03584725	0.00484086	-0.00518997
449	0.03540758	0.00424762	-0.00634779
450	0.03479334	0.00358544	-0.00738212
451	0.03401438	0.00281988	-0.00829128
452	0.03307100	0.00195507	-0.00905581
453	0.03197989	0.00104425	-0.00949076
454	0.03092847	0.00026206	-0.00970118
455	0.02998050	-0.00049538	-0.00966793
456	0.02907338	-0.00114972	-0.00940987
457	0.02824415	-0.00176644	-0.00889215
458	0.02748598	-0.00234658	-0.00810671
459	0.02687795	-0.00295596	-0.00702761
460	0.02643400	-0.00358347	-0.00567919
461	0.02622359	-0.00427971	-0.00412512
462	0.02697931	-0.00414388	-0.00228177
463	0.02783022	-0.00416996	-0.00083607
464	0.02872124	-0.00424154	0.00027241
465	0.02970001	-0.00433671	0.00101924
466	0.03066197	-0.00426093	0.00164923
467	0.03189605	-0.00440755	0.00162636
468	0.03374501	-0.00469686	0.00105143
469	0.03562753	-0.00567469	-0.00040085
470	-0.01405999	-0.00448353	0.01199846
471	-0.01327624	-0.00615359	0.01155577
472	-0.01218013	-0.00765257	0.01108889
473	-0.01089008	-0.00894656	0.01074745
474	-0.00952228	-0.01014643	0.01065119
475	-0.00802525	-0.01122904	0.01074947
476	-0.00648290	-0.01228996	0.01106501
477	-0.00526464	-0.01342736	0.01160294
478	-0.00284469	-0.01445149	0.01218824
479	-0.00235071	-0.01505638	0.01362634
480	-0.00057922	-0.01551150	0.01468553
481	0.00112223	-0.01579763	0.01578951
482	0.00277592	-0.01587528	0.01692713
483	0.00435767	-0.01575872	0.01807645
484	0.00588584	-0.01544568	0.01926245
485	0.00736047	-0.01496314	0.02049256
486	0.00878267	-0.01424004	0.02174220
487	0.01016040	-0.01325788	0.02300620
488	0.01150151	-0.01205326	0.02424520
489	0.01272474	-0.01063365	0.02541270
490	0.01394615	-0.00912507	0.02639944
491	0.01517522	-0.00756413	0.02728012
492	0.01643602	-0.00591284	0.02795602
493	0.01767138	-0.00416505	0.02847710
494	0.01893487	-0.00230851	0.02881471
495	0.02021383	-0.00038022	0.02899355
496	0.02150517	0.00165100	0.02897907
497	0.02279615	0.00377396	0.02871005
498	0.02479946	0.00683549	0.02788249
499	0.02678725	0.00955162	0.02661579
500	0.02876705	0.01190745	0.02497364
501	0.03075035	0.01392402	0.02302300
502	0.03276864	0.01564003	0.02077317
503	0.03480122	0.01701378	0.01814617
504	0.03685017	0.01804085	0.01513511
505	0.03885990	0.01867420	0.01169836
506	0.04071137	0.01880678	0.00783131
507	0.04234804	0.01842942	0.00319138

508	0.04319392	0.01758835	-0.00120791
509	0.04329672	0.01643156	-0.00516137
510	0.04283799	0.01510102	-0.00863886
511	0.04191714	0.01363323	-0.01167814
512	0.04058038	0.01206258	-0.01437005
513	0.03881765	0.01036889	-0.01677530
514	0.03653839	0.00849577	-0.01883607
515	0.03369222	0.00641862	-0.02043009
516	0.03121893	0.00480826	-0.02122917
517	0.02881560	0.00326752	-0.02153818
518	0.02653170	0.00180376	-0.02142990
519	0.02438529	0.00042885	-0.02093315
520	0.02235112	-0.00087884	-0.02012587
521	0.02043778	-0.00211728	-0.01893960
522	0.01862180	-0.00329883	-0.01740436
523	0.01691967	-0.00439601	-0.01547014
524	0.01539708	-0.00537614	-0.01311932
525	0.01436649	-0.00635815	-0.01077700
526	0.01369976	-0.00717745	-0.00841675
527	0.01337480	-0.00782977	-0.00607145
528	0.01334378	-0.00834772	-0.00375622
529	0.01362223	-0.00870699	-0.00147973
530	0.01416921	-0.00895092	0.00078057
531	0.01500797	-0.00907364	0.00304707
532	0.01619837	-0.00902670	0.00527835
533	0.01774937	-0.00877777	0.00743715
534	0.01943712	-0.00824749	0.00936803
535	0.02124334	-0.00752661	0.01093038
536	0.02308198	-0.00671600	0.01217647
537	0.02497056	-0.00579067	0.01311554
538	0.02693106	-0.00470307	0.01367410
539	0.02893472	-0.00360428	0.01407307
540	0.03101090	-0.00229582	0.01403004
541	0.03316389	-0.00088888	0.01367675
542	0.03533969	0.00060278	0.01283318
543	0.03767007	0.00181293	0.01145107
544	0.03961497	0.00276158	0.00973062
545	0.04116412	0.00344369	0.00784756
546	0.04240633	0.00390778	0.00583676
547	0.04334771	0.00412907	0.00370463
548	0.04403160	0.00415911	0.00139968
549	0.04438067	0.00396289	-0.00110620
550	0.04430687	0.00347775	-0.00380985
551	0.04372093	0.00269044	-0.00660298
552	0.04285048	0.00178208	-0.00882628
553	0.04174237	0.00079350	-0.01068087
554	0.04049535	-0.00023208	-0.01218732
555	0.03909850	-0.00130016	-0.01334704
556	0.03759087	-0.00240096	-0.01421729
557	0.03592539	-0.00354340	-0.01477669
558	0.03411289	-0.00474780	-0.01505795
559	0.03211970	-0.00599507	-0.01494660
560	0.03003332	-0.00725745	-0.01433042
561	0.02818582	-0.00857227	-0.01319741
562	0.02673746	-0.00964840	-0.01175889
563	0.02568626	-0.01052689	-0.01021597
564	0.02497676	-0.01122878	-0.00860865
565	0.02455074	-0.01182021	-0.00692180
566	0.02440537	-0.01230005	-0.00512194
567	0.02459443	-0.01265729	-0.00317997

568	0.02522170	-0.01269135	-0.00109534
569	0.02616218	-0.01232520	0.00112710
570	0.02799792	-0.01164154	0.00307416
571	0.02985354	-0.01086215	0.00428979
572	0.03149247	-0.01011763	0.00487646
573	0.03286284	-0.00952532	0.00507522
574	0.03415731	-0.00902859	0.00498691
575	0.03571407	-0.00844504	0.00434290
576	0.03719703	-0.00815875	0.00316565
577	0.03796199	-0.00783864	0.00091595
578	-0.00472278	-0.00591580	-0.01389955
579	-0.00282152	-0.00534411	-0.01307654
580	-0.00077831	-0.00526381	-0.01217037
581	0.00097025	-0.00534827	-0.01103326
582	0.00242991	-0.00558909	-0.00963486
583	0.00351485	-0.00586547	-0.00795511
584	0.00433010	-0.00629561	-0.00603422
585	0.00486325	-0.00686536	-0.00381865
586	0.00519077	-0.00760052	-0.00137754
587	0.00556083	-0.00865338	0.00112401
588	0.00646828	-0.01013170	0.00343617
589	0.00783613	-0.01149684	0.00551644
590	0.00930897	-0.01254350	0.00744219
591	0.01084713	-0.01319676	0.00926903
592	0.01241892	-0.01342319	0.01104797
593	0.01408775	-0.01328703	0.01276525
594	0.01583373	-0.01277122	0.01443718
595	0.01763102	-0.01183438	0.01607692
596	0.01947566	-0.01048279	0.01764952
597	0.02140151	-0.00861850	0.01893748
598	0.02317182	-0.00663277	0.01995184
599	0.02480619	-0.00457297	0.02065572
600	0.02639007	-0.00251694	0.02101676
601	0.02795184	-0.00048046	0.02101025
602	0.02950971	0.00154632	0.02063967
603	0.03113609	0.00352040	0.01987287
604	0.03288489	0.00539669	0.01874626
605	0.03474481	0.00711491	0.01730788
606	0.03686200	0.00862248	0.01474500
607	0.03872174	0.00971562	0.01211694
608	0.04029211	0.01043822	0.00941802
609	0.04158210	0.01077047	0.00664789
610	0.04252638	0.01077335	0.00380530
611	0.04314034	0.01041931	0.00085880
612	0.04337970	0.00977831	-0.00218957
613	0.04322285	0.00884295	-0.00533685
614	0.04260484	0.00769363	-0.00858368
615	0.04161695	0.00644682	-0.01099706
616	0.04035395	0.00523362	-0.01302552
617	0.03893777	0.00397600	-0.01467091
618	0.03733841	0.00270040	-0.01593965
619	0.03557586	0.00137918	-0.01681177
620	0.03364114	0.00002475	-0.01730930
621	0.03156233	-0.00139408	-0.01743314
622	0.02931854	-0.00288202	-0.01718814
623	0.02690239	-0.00442226	-0.01654399
624	0.02483326	-0.00585611	-0.01531786
625	0.02310872	-0.00708084	-0.01384842
626	0.02174057	-0.00812270	-0.01217845
627	0.02073371	-0.00900456	-0.01031061

628	0.02008726	-0.00970652	-0.00824582
629	0.01984304	-0.01036388	-0.00598328
630	0.01997215	-0.01087333	-0.00350758
631	0.02047542	-0.01124309	-0.00083346
632	0.02133162	-0.01140695	0.00203385
633	0.02270881	-0.01122814	0.00426263
634	0.02414832	-0.01080825	0.00614223
635	0.02564284	-0.01017136	0.00763857
636	0.02724265	-0.00941665	0.00876399
637	0.02893980	-0.00857386	0.00952246
638	0.03074246	-0.00759938	0.00990687
639	0.03266598	-0.00653779	0.00993561
640	0.03473008	-0.00542265	0.00962073
641	0.03688195	-0.00415521	0.00892264
642	0.03844788	-0.00340503	0.00785551
643	0.03978015	-0.00279195	0.00666462
644	0.04087124	-0.00232931	0.00534496
645	0.04173090	-0.00202117	0.00390837
646	0.04236048	-0.00186649	0.00235601
647	0.04276548	-0.00186371	0.00067766
648	0.04297459	-0.00204918	-0.00111624
649	0.04290986	-0.00235173	-0.00304896
650	0.04257675	-0.00277653	-0.00509931
651	0.04199268	-0.00337785	-0.00662625
652	0.04128121	-0.00403213	-0.00792095
653	0.04042464	-0.00471125	-0.00897988
654	0.03946348	-0.00545763	-0.00981281
655	0.03840840	-0.00624549	-0.01039310
656	0.03724789	-0.00710525	-0.01074771
657	0.03598539	-0.00803769	-0.01087718
658	0.03462609	-0.00903747	-0.01077708
659	0.03312538	-0.01004584	-0.01042879
660	0.03197561	-0.01098321	-0.00948970
661	0.03103462	-0.01173975	-0.00848024
662	0.03033323	-0.01236701	-0.00741586
663	0.02986373	-0.01285649	-0.00629548
664	0.02963806	-0.01321170	-0.00513194
665	0.02963912	-0.01344038	-0.00390085
666	0.02985399	-0.01349280	-0.00262084
667	0.03031734	-0.01340983	-0.00128204
668	0.03100931	-0.01318364	0.00011827
669	0.03213870	-0.01275362	0.00090662
670	0.03314645	-0.01225541	0.00146206
671	0.03410651	-0.01180367	0.00179509
672	0.03498578	-0.01133088	0.00191744
673	0.03582410	-0.01084690	0.00182995
674	0.03664555	-0.01030458	0.00150342
675	0.03744968	-0.00965418	0.00083948
676	0.03793364	-0.00861659	-0.00017299
677	-0.00929247	0.01399275	-0.00004503
678	-0.01283930	0.01696123	-0.00155635
679	-0.01665438	0.01954362	-0.00296851
680	-0.02025991	0.02198157	-0.00416642
681	-0.02350131	0.02441735	-0.00522117
682	-0.02664614	0.02660005	-0.00607178
683	-0.02988727	0.02846567	-0.00678561
684	-0.03277505	0.03017638	-0.00712933
685	-0.03566587	0.03171160	-0.00735360
686	-0.03850607	0.03290508	-0.00743352

Procrustes sums of squares: 0.3657145180346275
Tangent sums of squares: 0.35793716368340484

Data matrices in this dataset:
- Evolution Cervidae final, raw data
- Sans les Indet., centroid size
- Sans les Indet., Procrustes coordinates

New Procrustes fit:
Dataset 'anterior ampulla'

10 landmarks in 3 dimensions.
The dataset contains 40 observations, of which 36 are included for analyses.

Average shape:

Lmk.	Axis 1 (x)	Axis 2 (y)	Axis 3 (z)
1	0.54108484	-0.03103519	-0.06306921
2	0.35803623	0.02170934	-0.05356290
3	0.24930508	0.01825100	-0.00938039
4	0.14350865	0.00822104	0.04325600
5	0.03771910	0.00131569	0.07264004
6	-0.06583022	-0.00439802	0.07958000
7	-0.16683412	-0.00714560	0.06241527
8	-0.26816771	-0.00614047	0.02535913
9	-0.36669495	-0.00259153	-0.03685678
10	-0.46212691	0.00181373	-0.12038116

Procrustes sums of squares: 0.218432565136065
Tangent sums of squares: 0.21651513127315702

Data matrices in this dataset:
- anterior ampulla, raw data
- anterior ampulla, centroid size
- anterior ampulla, Procrustes coordinates

New Procrustes fit:
Dataset 'anterior ampulla'

10 landmarks in 3 dimensions.
The dataset contains 40 observations, of which 36 are included for analyses.

Average shape:

Lmk.	Axis 1 (x)	Axis 2 (y)	Axis 3 (z)
1	0.54108484	-0.03103519	-0.06306921
2	0.35803623	0.02170934	-0.05356290
3	0.24930508	0.01825100	-0.00938039
4	0.14350865	0.00822104	0.04325600
5	0.03771910	0.00131569	0.07264004
6	-0.06583022	-0.00439802	0.07958000
7	-0.16683412	-0.00714560	0.06241527
8	-0.26816771	-0.00614047	0.02535913
9	-0.36669495	-0.00259153	-0.03685678

10	-0.46212691	0.00181373	-0.12038116

Procrustes sums of squares: 0.218432565136065
Tangent sums of squares: 0.21651513127315702

Data matrices in this dataset:
- anterior ampulla, raw data
- anterior ampulla, centroid size
- anterior ampulla, Procrustes coordinates

Canonical Variate Analysis: CVA ... anterior ampulla

Dataset: anterior ampulla
Classification criterion: subfam

Groups		Observations
1.	Ca	9
2.	Ce	13
3.	Di	3
4.	Pr	11

Variation among groups, scaled by the inverse of the within-group variation

	Eigenvalues	% Variance	Cumulative %
1.	12.92708627	67.973	67.973
2.	4.09625721	21.539	89.512
3.	1.99463338	10.488	100.000

Mahalanobis distances among groups:

	Ca	Ce	Di
Ce	3.9709		
Di	12.8710	12.3465	
Pr	4.5866	5.0694	10.8551

P-values from permutation tests (10000 permutation rounds) for Mahalanobis distances among groups:

	Ca	Ce	Di
Ce	<.0001		
Di	0.0036	0.0013	
Pr	<.0001	<.0001	0.0022

Procrustes distances among groups:

	Ca	Ce	Di
Ce	0.0370		
Di	0.0294	0.0376	
Pr	0.0306	0.0333	0.0349

P-values from permutation tests (10000 permutation rounds) for Procrustes distances among groups:

	Ca	Ce	Di
Ce	0.2563		
Di	0.8450	0.6515	

Pr	0.6789	0.4344	0.8458

Canonical coefficients:

	CV1	CV2	CV3
x1	-43.1538	-30.2064	20.9690
y1	-36.4224	-38.2226	7.9154
z1	28.0755	21.2569	-56.8733
x2	536.6953	250.6293	-332.2696
y2	-128.9534	-181.1467	-82.9631
z2	-129.0805	-72.2866	49.1494
x3	-451.6931	-368.8678	304.9432
y3	250.4041	421.0671	311.2121
z3	178.9209	155.0349	54.7747
x4	-879.8248	-161.2938	786.1338
y4	-373.2746	-334.9767	-302.0285
z4	-271.7962	-240.1304	-10.9052
x5	1106.9058	467.4296	-1746.7173
y5	473.0904	675.3365	-291.7965
z5	110.6001	274.9201	-229.6764
x6	-1504.2400	340.5116	2122.9523
y6	-626.3409	-575.7241	994.8127
z6	96.5607	19.8938	518.5749
x7	2810.7080	-1516.9475	-2129.5691
y7	1809.5772	-453.5376	-1181.4279
z7	997.8969	-601.3754	-655.5300
x8	-1709.4171	2084.4406	1473.1813
y8	-1663.3887	457.3516	461.9541
z8	-1335.3440	732.3846	597.1892
x9	5.8178	-1524.7300	-715.9937
y9	-141.8859	275.6125	350.4381
z9	32.3593	-330.5365	-357.4973
x10	128.2021	459.0345	216.3702
y10	437.1942	-245.7600	-268.1163
z10	291.8073	40.8387	90.7940

New Procrustes fit:
Dataset 'posterior ampulla'

10 landmarks in 3 dimensions.
The dataset contains 40 observations, of which 36 are included for analyses.

Average shape:

Lmk.	Axis 1 (x)	Axis 2 (y)	Axis 3 (z)
1	0.48473697	-0.07057001	-0.04074851
2	0.36125491	-0.06411814	0.02090948
3	0.26445313	-0.01408873	0.02402365
4	0.16512438	0.04146442	0.01336493
5	0.06195595	0.07286149	0.00483863
6	-0.04390473	0.08141201	-0.00159956
7	-0.15633404	0.07601984	-0.01186101
8	-0.26730742	0.03833331	-0.01192473
9	-0.37734960	-0.03493061	-0.00257407
10	-0.49262954	-0.12638359	0.00557119

Procrustes sums of squares: 0.49844639384447625
Tangent sums of squares: 0.4653741476291842

Data matrices in this dataset:
- posterior ampulla, raw data
- posterior ampulla, centroid size
- posterior ampulla, Procrustes coordinates

New Procrustes fit:
Dataset 'posterior ampulla'

10 landmarks in 3 dimensions.
The dataset contains 40 observations, of which 36 are included for analyses.

Average shape:

Lmk.	Axis 1 (x)	Axis 2 (y)	Axis 3 (z)
1	0.48473697	-0.07057001	-0.04074851
2	0.36125491	-0.06411814	0.02090948
3	0.26445313	-0.01408873	0.02402365
4	0.16512438	0.04146442	0.01336493
5	0.06195595	0.07286149	0.00483863
6	-0.04390473	0.08141201	-0.00159956
7	-0.15633404	0.07601984	-0.01186101
8	-0.26730742	0.03833331	-0.01192473
9	-0.37734960	-0.03493061	-0.00257407
10	-0.49262954	-0.12638359	0.00557119

Procrustes sums of squares: 0.49844639384447625
Tangent sums of squares: 0.4653741476291842

Data matrices in this dataset:
- posterior ampulla, raw data
- posterior ampulla, centroid size
- posterior ampulla, Procrustes coordinates

New Procrustes fit:
Dataset 'posterior ampulla'

10 landmarks in 3 dimensions.
The dataset contains 40 observations, of which 36 are included for analyses.

Average shape:

Lmk.	Axis 1 (x)	Axis 2 (y)	Axis 3 (z)
1	0.48473697	-0.07057001	-0.04074851
2	0.36125491	-0.06411814	0.02090948
3	0.26445313	-0.01408873	0.02402365
4	0.16512438	0.04146442	0.01336493
5	0.06195595	0.07286149	0.00483863
6	-0.04390473	0.08141201	-0.00159956
7	-0.15633404	0.07601984	-0.01186101
8	-0.26730742	0.03833331	-0.01192473
9	-0.37734960	-0.03493061	-0.00257407
10	-0.49262954	-0.12638359	0.00557119

Procrustes sums of squares: 0.49844639384447625
Tangent sums of squares: 0.4653741476291842

Data matrices in this dataset:
- posterior ampulla, raw data
- posterior ampulla, centroid size
- posterior ampulla, Procrustes coordinates

New Procrustes fit:
Dataset 'posterior ampulla'

10 landmarks in 3 dimensions.
The dataset contains 40 observations, of which 36 are included for analyses.

Average shape:

Lmk.	Axis 1 (x)	Axis 2 (y)	Axis 3 (z)
1	0.48473697	-0.07057001	-0.04074851
2	0.36125491	-0.06411814	0.02090948
3	0.26445313	-0.01408873	0.02402365
4	0.16512438	0.04146442	0.01336493
5	0.06195595	0.07286149	0.00483863
6	-0.04390473	0.08141201	-0.00159956
7	-0.15633404	0.07601984	-0.01186101
8	-0.26730742	0.03833331	-0.01192473
9	-0.37734960	-0.03493061	-0.00257407
10	-0.49262954	-0.12638359	0.00557119

Procrustes sums of squares: 0.49844639384447625
Tangent sums of squares: 0.4653741476291842

Data matrices in this dataset:
- posterior ampulla, raw data
- posterior ampulla, centroid size
- posterior ampulla, Procrustes coordinates

Canonical Variate Analysis: CVA ... posterior ampulla

Dataset: posterior ampulla
Classification criterion: subfam

Groups		Observations
1.	Ca	9
2.	Ce	13
3.	Di	3
4.	Pr	11

Variation among groups, scaled by the inverse of the within-group variation

	Eigenvalues	% Variance	Cumulative %
1.	3.09122602	50.728	50.728
2.	2.28392590	37.480	88.207
3.	0.71861485	11.793	100.000

4.	0.00000000	0.000	100.000

Mahalanobis distances among groups:

	Ca	Ce	Di
Ce	3.7637		
Di	3.8472	3.8100	
Pr	4.0815	4.0470	3.5060

P-values from permutation tests (10000 permutation rounds) for Mahalanobis distances among groups:

	Ca	Ce	Di
Ce	<.0001		
Di	0.0278	0.0099	
Pr	<.0001	<.0001	0.1157

Procrustes distances among groups:

	Ca	Ce	Di
Ce	0.0741		
Di	0.0274	0.0707	
Pr	0.0475	0.0467	0.0496

P-values from permutation tests (10000 permutation rounds) for Procrustes distances among groups:

	Ca	Ce	Di
Ce	0.1126		
Di	0.9611	0.5523	
Pr	0.2052	0.5274	0.4075

Canonical coefficients:

	CV1	CV2	CV3	CV4
x1	7.7449	13.0583	29.9648	-0.5364
y1	-41.8404	-24.3462	-20.4750	-5.4970
z1	39.2599	15.0849	8.3340	2.7595
x2	-389.8606	-71.1948	-236.9707	5.1011
y2	167.4071	122.4842	92.0395	13.7001
z2	86.3401	-52.8669	62.7943	-21.8497
x3	-251.9075	139.8865	316.5903	-5.3399
y3	-109.2449	-97.0851	-105.2439	-3.9310
z3	-195.8026	218.4930	-90.1206	55.4962
x4	535.6164	-961.1823	-964.7174	-87.2259
y4	28.6123	-143.8191	-13.0474	-31.4172
z4	369.3868	-655.1894	-106.9242	-135.5382
x5	2102.4352	2422.4804	2417.2156	199.3489
y5	-120.4451	372.4058	-40.2387	88.7412
z5	-736.0646	904.3220	-0.8984	199.6291
x6	-2549.2691	-1935.4893	-2025.4030	-214.0541
y6	-267.8932	-546.2055	121.0760	-147.1679
z6	476.8447	-675.5088	443.1159	-219.7177
x7	778.5343	272.9893	503.5484	-16.7620
y7	482.3504	515.6038	-8.1090	113.4817
z7	-148.5112	534.3470	-488.7470	181.4414
x8	-42.6590	-178.0629	-15.1130	-40.4897
y8	108.4910	-202.8263	53.5610	-56.6981
z8	404.5567	-249.9497	232.0557	-87.4273
x9	-922.8614	435.7631	-276.0312	132.9157

y9	-387.9090	-42.4453	-140.4980	14.2390
z9	-426.5841	-221.6006	-83.9730	-32.5173
x10	732.2269	-138.2484	250.9162	-88.1063
y10	140.4717	46.2339	60.9355	1.6424
z10	130.5742	182.8685	24.3634	34.6761

New Procrustes fit:
Dataset 'sans capreolus zurich'

10 landmarks in 3 dimensions.
The dataset contains 40 observations, of which 35 are included for analyses.

Average shape:

Lmk.	Axis 1 (x)	Axis 2 (y)	Axis 3 (z)
1	0.48545205	-0.06888841	-0.04082619
2	0.36214410	-0.06381720	0.02095241
3	0.26477558	-0.01445585	0.02405463
4	0.16489125	0.04112795	0.01326563
5	0.06131934	0.07248016	0.00471829
6	-0.04469999	0.08066910	-0.00153662
7	-0.15710790	0.07516941	-0.01173939
8	-0.26770063	0.03754054	-0.01166449
9	-0.37720429	-0.03482844	-0.00248331
10	-0.49186950	-0.12499725	0.00525904

Procrustes sums of squares: 0.4857899685317421
Tangent sums of squares: 0.4529438966726556

Data matrices in this dataset:
- posterior ampulla, raw data
- sans capreolus zurich, centroid size
- sans capreolus zurich, Procrustes coordinates

New Procrustes fit:
Dataset 'sans capreolus zurich'

10 landmarks in 3 dimensions.
The dataset contains 40 observations, of which 35 are included for analyses.

Average shape:

Lmk.	Axis 1 (x)	Axis 2 (y)	Axis 3 (z)
1	0.48545205	-0.06888841	-0.04082619
2	0.36214410	-0.06381720	0.02095241
3	0.26477558	-0.01445585	0.02405463
4	0.16489125	0.04112795	0.01326563
5	0.06131934	0.07248016	0.00471829
6	-0.04469999	0.08066910	-0.00153662
7	-0.15710790	0.07516941	-0.01173939
8	-0.26770063	0.03754054	-0.01166449
9	-0.37720429	-0.03482844	-0.00248331
10	-0.49186950	-0.12499725	0.00525904

Procrustes sums of squares: 0.4857899685317421

Tangent sums of squares: 0.4529438966726556

Data matrices in this dataset:
- posterior ampulla, raw data
- sans capreolus zurich, centroid size
- sans capreolus zurich, Procrustes coordinates

New Procrustes fit:
Dataset 'sans capreolus zurich'

10 landmarks in 3 dimensions.
The dataset contains 40 observations, of which 35 are included for analyses.

Average shape:

Lmk.	Axis 1 (x)	Axis 2 (y)	Axis 3 (z)
1	0.48545205	-0.06888841	-0.04082619
2	0.36214410	-0.06381720	0.02095241
3	0.26477558	-0.01445585	0.02405463
4	0.16489125	0.04112795	0.01326563
5	0.06131934	0.07248016	0.00471829
6	-0.04469999	0.08066910	-0.00153662
7	-0.15710790	0.07516941	-0.01173939
8	-0.26770063	0.03754054	-0.01166449
9	-0.37720429	-0.03482844	-0.00248331
10	-0.49186950	-0.12499725	0.00525904

Procrustes sums of squares: 0.4857899685317421
Tangent sums of squares: 0.4529438966726556

Data matrices in this dataset:
- posterior ampulla, raw data
- sans capreolus zurich, centroid size
- sans capreolus zurich, Procrustes coordinates

Canonical Variate Analysis: CVA ...postAmpu sans capr

Dataset: sans capreolus zurich
Classification criterion: subfam

Groups		Observations
1.	Ca	8
2.	Ce	13
3.	Di	3
4.	Pr	11

Variation among groups, scaled by the inverse of the within-group variation

	Eigenvalues	% Variance	Cumulative %
1.	9.51477528	71.996	71.996
2.	2.96707680	22.451	94.447
3.	0.73389656	5.553	100.000
4.	0.00000000	0.000	100.000

Mahalanobis distances among groups:

	Ca	Ce	Di
Ce	6.5426		
Di	6.7168	3.7550	
Pr	7.7790	4.1771	3.6286

P-values from permutation tests (10000 permutation rounds) for Mahalanobis distances among groups:

	Ca	Ce	Di
Ce	<.0001		
Di	0.0054	0.0097	
Pr	<.0001	<.0001	0.0692

Procrustes distances among groups:

	Ca	Ce	Di
Ce	0.0841		
Di	0.0364	0.0707	
Pr	0.0622	0.0467	0.0496

P-values from permutation tests (10000 permutation rounds) for Procrustes distances among groups:

	Ca	Ce	Di
Ce	0.0595		
Di	0.8626	0.5634	
Pr	0.0325	0.5162	0.4090

Canonical coefficients:

	CV1	CV2	CV3	CV4
x1	-32.5380	-0.5891	28.8093	-1.4302
y1	58.9738	26.5731	-17.2626	-1.2876
z1	-54.0819	-27.1857	7.0101	-0.4566
x2	210.5018	328.1748	-211.1416	91.2285
y2	-225.7284	-101.9126	74.9881	-7.1798
z2	-3.6993	-96.3540	65.6671	-28.8980
x3	-548.3830	325.4661	337.5989	58.3513
y3	170.7249	58.9621	-91.0748	10.4018
z3	-127.6766	259.8615	-105.8336	83.5135
x4	2129.8225	-970.2819	-954.0563	-253.7280
y4	227.9309	-88.1122	-6.8156	-37.7935
z4	823.5018	-611.7976	-70.4600	-185.6815
x5	-4281.6746	-900.0699	2118.3254	-95.8589
y5	-768.0658	284.8434	-39.8306	150.2235
z5	-1527.3649	1090.9169	-7.8567	362.6786
x6	3310.0065	1547.0279	-1729.1668	217.1321
y6	1511.1116	-32.9790	118.5648	-208.4692
z6	1762.6082	-797.7314	398.8405	-432.8955
x7	-969.3114	-569.7235	467.6556	-75.6115
y7	-1547.8911	-173.0004	-13.9422	171.0970
z7	-1441.8305	417.5220	-459.0110	317.2958
x8	553.9092	-57.0534	-19.3936	-58.3763
y8	384.0627	-192.5136	52.8031	-87.2107
z8	407.0562	-489.6727	217.1851	-172.5908
x9	-362.9640	1025.9649	-264.6786	273.5435
y9	351.1075	322.8788	-131.9553	47.8687

z9	533.8734	291.9966	-56.7656	-10.9065
x10	-9.3690	-728.9159	226.0477	-177.7671
y10	-162.2261	-104.7397	54.5251	-5.3433
z10	-372.3862	-37.5557	11.2240	31.8163

New Procrustes fit:
Dataset 'Sans Ind & Capreolus'

686 landmarks in 3 dimensions.
The dataset contains 40 observations, of which 36 are included for analyses.

Average shape:

Lmk.	Axis 1 (x)	Axis 2 (y)	Axis 3 (z)
1	-0.04036362	0.02592451	-0.00811150
2	-0.02486511	-0.00981714	-0.02627340
3	-0.02630368	-0.01137480	-0.02731431
4	-0.02818995	-0.01223046	-0.02847610
5	-0.03017741	-0.01259941	-0.02952329
6	-0.03220235	-0.01261672	-0.03047543
7	-0.03431632	-0.01234278	-0.03134544
8	-0.03656948	-0.01172608	-0.03215652
9	-0.03897484	-0.01071316	-0.03289171
10	-0.04144477	-0.00924722	-0.03353796
11	-0.04391420	-0.00725860	-0.03406058
12	-0.04660985	-0.00439912	-0.03449515
13	-0.04870323	-0.00138086	-0.03453497
14	-0.05020484	0.00168057	-0.03420577
15	-0.05127422	0.00468345	-0.03352928
16	-0.05194588	0.00763278	-0.03255234
17	-0.05218638	0.01059840	-0.03122656
18	-0.05198752	0.01357426	-0.02955756
19	-0.05126707	0.01655038	-0.02751849
20	-0.04987035	0.01940290	-0.02515643
21	-0.04786808	0.02164297	-0.02204558
22	-0.04544731	0.02307481	-0.01919084
23	-0.04287669	0.02382276	-0.01656435
24	-0.04021532	0.02392447	-0.01415795
25	-0.03749705	0.02347117	-0.01185990
26	-0.03451674	0.02226598	-0.00987187
27	-0.03131650	0.02036366	-0.00814552
28	-0.02795494	0.01785428	-0.00653419
29	-0.02446861	0.01459152	-0.00530442
30	-0.02274492	0.01293029	-0.00496445
31	-0.02130127	0.01127595	-0.00504291
32	-0.02018054	0.00965077	-0.00552535
33	-0.01937887	0.00805876	-0.00639792
34	-0.01887320	0.00649494	-0.00763445
35	-0.01863959	0.00493692	-0.00923185
36	-0.01861014	0.00336333	-0.01114849
37	-0.01881318	0.00170255	-0.01342681
38	-0.01950246	0.00028609	-0.01612496
39	-0.02051828	-0.00009089	-0.01770052
40	-0.02170450	-0.00033282	-0.01922434
41	-0.02269031	-0.00088248	-0.02062383
42	-0.02347663	-0.00171594	-0.02187741
43	-0.02404150	-0.00284644	-0.02298066
44	-0.02444990	-0.00424358	-0.02398036

45	-0.02463759	-0.00594249	-0.02481416
46	-0.02463999	-0.00790540	-0.02549130
47	-0.01971082	-0.01429760	-0.02654986
48	-0.02207603	-0.01533880	-0.02788349
49	-0.02452514	-0.01601771	-0.02907491
50	-0.02697985	-0.01635938	-0.03012478
51	-0.02938783	-0.01643944	-0.03108082
52	-0.03172828	-0.01627283	-0.03197942
53	-0.03405324	-0.01585177	-0.03278889
54	-0.03635762	-0.01517846	-0.03352073
55	-0.03864152	-0.01423175	-0.03417591
56	-0.04088868	-0.01300501	-0.03472953
57	-0.04298360	-0.01153854	-0.03527308
58	-0.04489822	-0.00994488	-0.03566984
59	-0.04665507	-0.00824501	-0.03596318
60	-0.04822977	-0.00641306	-0.03612856
61	-0.04969089	-0.00450700	-0.03623035
62	-0.05095120	-0.00244136	-0.03614721
63	-0.05204945	-0.00025523	-0.03596464
64	-0.05297543	0.00205276	-0.03560948
65	-0.05371134	0.00447999	-0.03512109
66	-0.05419957	0.00667575	-0.03445464
67	-0.05451852	0.00881152	-0.03371248
68	-0.05466005	0.01088919	-0.03286446
69	-0.05459579	0.01289528	-0.03188451
70	-0.05437111	0.01483793	-0.03081159
71	-0.05400202	0.01676676	-0.02966597
72	-0.05338614	0.01857604	-0.02833334
73	-0.05259955	0.02033943	-0.02692452
74	-0.05160894	0.02199484	-0.02538766
75	-0.05032088	0.02348538	-0.02386987
76	-0.04889424	0.02477045	-0.02222484
77	-0.04734848	0.02583566	-0.02045486
78	-0.04568356	0.02668656	-0.01854892
79	-0.04389958	0.02729224	-0.01651489
80	-0.04196674	0.02768918	-0.01436050
81	-0.03993372	0.02788108	-0.01206327
82	-0.03778278	0.02786411	-0.00962310
83	-0.03550002	0.02805675	-0.00713649
84	-0.02475516	0.00500360	0.02164341
85	-0.02663734	0.00345875	0.02300711
86	-0.02893429	0.00246871	0.02432777
87	-0.03125222	0.00179496	0.02541824
88	-0.03351854	0.00126713	0.02624892
89	-0.03576266	0.00091725	0.02689810
90	-0.03801045	0.00073757	0.02735358
91	-0.04026958	0.00071849	0.02765156
92	-0.04255395	0.00091244	0.02778557
93	-0.04484174	0.00133713	0.02770046
94	-0.04746711	0.00203698	0.02715615
95	-0.04978796	0.00300011	0.02632534
96	-0.05182017	0.00415271	0.02524752
97	-0.05352649	0.00550637	0.02392747
98	-0.05500355	0.00695445	0.02240050
99	-0.05624612	0.00856683	0.02065234
100	-0.05722574	0.01032876	0.01866341
101	-0.05794862	0.01226252	0.01643623
102	-0.05829858	0.01437348	0.01400590
103	-0.05820941	0.01695260	0.01097585
104	-0.05748421	0.01920360	0.00826470

105	-0.05622342	0.02109539	0.00589852
106	-0.05456535	0.02262365	0.00381355
107	-0.05245204	0.02378773	0.00203401
108	-0.04992802	0.02461653	0.00047559
109	-0.04691429	0.02511807	-0.00083014
110	-0.04338655	0.02524868	-0.00180378
111	-0.03939899	0.02500765	-0.00219625
112	-0.03571669	0.02334225	-0.00172767
113	-0.03242682	0.02173363	-0.00074659
114	-0.02951264	0.02020953	0.00028187
115	-0.02694310	0.01876207	0.00131585
116	-0.02471578	0.01734922	0.00240066
117	-0.02278449	0.01593180	0.00362812
118	-0.02116758	0.01449232	0.00516745
119	-0.02005743	0.01321894	0.00717437
120	-0.01982002	0.01265052	0.00928625
121	-0.02048050	0.01276028	0.01082980
122	-0.02140577	0.01295381	0.01235614
123	-0.02221766	0.01279049	0.01383239
124	-0.02290441	0.01229057	0.01525892
125	-0.02344077	0.01132780	0.01664227
126	-0.02388330	0.01016281	0.01795521
127	-0.02417959	0.00871239	0.01919274
128	-0.02428154	0.00676757	0.02037126
129	-0.01889190	0.00302862	0.02394466
130	-0.02110260	0.00214561	0.02522712
131	-0.02349788	0.00139899	0.02617422
132	-0.02586912	0.00076067	0.02701963
133	-0.02819728	0.00019760	0.02778945
134	-0.03049387	-0.00028099	0.02849185
135	-0.03278938	-0.00066872	0.02911548
136	-0.03508840	-0.00096192	0.02966257
137	-0.03739207	-0.00114914	0.03004513
138	-0.03968475	-0.00124508	0.03031424
139	-0.04199260	-0.00107408	0.03043188
140	-0.04419000	-0.00078425	0.03037969
141	-0.04626033	-0.00034011	0.03007118
142	-0.04822308	0.00018904	0.02966283
143	-0.05005434	0.00084689	0.02904747
144	-0.05177324	0.00165080	0.02827374
145	-0.05339725	0.00256569	0.02734367
146	-0.05488949	0.00361482	0.02622050
147	-0.05627996	0.00479674	0.02496187
148	-0.05780268	0.00638624	0.02325633
149	-0.05900770	0.00801934	0.02146877
150	-0.05993875	0.00967549	0.01961293
151	-0.06065928	0.01137019	0.01772820
152	-0.06111308	0.01310885	0.01578557
153	-0.06132017	0.01486941	0.01377255
154	-0.06123036	0.01664026	0.01167593
155	-0.06084204	0.01843626	0.00949995
156	-0.06010631	0.02023352	0.00726995
157	-0.05876156	0.02231800	0.00499395
158	-0.05702064	0.02406532	0.00291125
159	-0.05495415	0.02551484	0.00103142
160	-0.05263172	0.02673985	-0.00076846
161	-0.04991975	0.02759773	-0.00236395
162	-0.04685180	0.02816012	-0.00379531
163	-0.04340435	0.02836941	-0.00505424
164	-0.03957295	0.02841727	-0.00620094

165	-0.03550002	0.02805675	-0.00713649
166	-0.02499274	-0.01797812	-0.01144966
167	-0.02594114	-0.01926394	-0.01127705
168	-0.02715963	-0.02032120	-0.01063659
169	-0.02835612	-0.02123163	-0.00977690
170	-0.02953381	-0.02203539	-0.00869693
171	-0.03072663	-0.02273138	-0.00741166
172	-0.03191706	-0.02340512	-0.00589307
173	-0.03311180	-0.02396404	-0.00412300
174	-0.03431553	-0.02442274	-0.00211313
175	-0.03556056	-0.02468571	0.00012660
176	-0.03649211	-0.02473268	0.00178126
177	-0.03733966	-0.02465161	0.00340695
178	-0.03820179	-0.02302148	0.00481856
179	-0.03880320	-0.02403014	0.00653744
180	-0.03941172	-0.02351355	0.00803519
181	-0.03995039	-0.02286350	0.00951174
182	-0.04039727	-0.02207565	0.01095243
183	-0.04079079	-0.02116340	0.01237129
184	-0.04109684	-0.02011810	0.01374697
185	-0.04130890	-0.01876998	0.01527170
186	-0.04134585	-0.01740235	0.01661532
187	-0.04122223	-0.01601967	0.01784617
188	-0.04086897	-0.01457793	0.01871371
189	-0.04035602	-0.01312194	0.01951769
190	-0.03964819	-0.01161107	0.02003250
191	-0.03873596	-0.01005176	0.02032875
192	-0.03763316	-0.00846149	0.02042424
193	-0.03633865	-0.00682257	0.02029050
194	-0.03442252	-0.00477936	0.01989045
195	-0.03261396	-0.00294886	0.01921052
196	-0.03090104	-0.00137572	0.01823817
197	-0.02927633	-0.00002425	0.01701181
198	-0.02777946	0.00114102	0.01557634
199	-0.02638943	0.00209796	0.01386781
200	-0.02507999	0.00277584	0.01189396
201	-0.02383005	0.00335102	0.00971822
202	-0.02268630	0.00367457	0.00728109
203	-0.02198686	0.00350864	0.00526205
204	-0.02150943	0.00305574	0.00328699
205	-0.02109940	0.00246504	0.00146270
206	-0.02070629	0.00172063	-0.00021211
207	-0.02031526	0.00079952	-0.00175701
208	-0.02001998	-0.00032600	-0.00321626
209	-0.01977998	-0.00165156	-0.00460495
210	-0.01972435	-0.00318700	-0.00591164
211	-0.02012169	-0.00482420	-0.00683380
212	-0.02086359	-0.00632844	-0.00688795
213	-0.02179806	-0.00781622	-0.00684819
214	-0.02269357	-0.00929351	-0.00694264
215	-0.02340306	-0.01076302	-0.00724541
216	-0.02389958	-0.01220369	-0.00778537
217	-0.02423287	-0.01365489	-0.00854983
218	-0.02439367	-0.01512932	-0.00955153
219	-0.02446461	-0.01665275	-0.01071370
220	-0.02301658	-0.01983875	-0.01657561
221	-0.02449962	-0.02103703	-0.01580409
222	-0.02591209	-0.02210145	-0.01482174
223	-0.02725720	-0.02308326	-0.01371343
224	-0.02855787	-0.02398036	-0.01249724

225	-0.02979391	-0.02477324	-0.01114796
226	-0.03097478	-0.02545093	-0.00967183
227	-0.03210955	-0.02607016	-0.00809480
228	-0.03319008	-0.02659988	-0.00638774
229	-0.03426191	-0.02708908	-0.00460141
230	-0.03533030	-0.02742900	-0.00265247
231	-0.03638312	-0.02766692	-0.00077013
232	-0.03734717	-0.02770520	0.00110545
233	-0.03826857	-0.02760746	0.00292934
234	-0.03908166	-0.02730438	0.00475011
235	-0.03985656	-0.02685237	0.00652661
236	-0.04056376	-0.02621526	0.00828375
237	-0.04119816	-0.02541774	0.01001183
238	-0.04177051	-0.02445017	0.01171237
239	-0.04234573	-0.02326985	0.01357121
240	-0.04280543	-0.02204921	0.01527783
241	-0.04307339	-0.02075208	0.01680627
242	-0.04316928	-0.01938416	0.01818845
243	-0.04309833	-0.01796702	0.01938745
244	-0.04288469	-0.01648776	0.02045101
245	-0.04251190	-0.01496819	0.02134714
246	-0.04194658	-0.01335978	0.02208120
247	-0.04122537	-0.01171653	0.02264389
248	-0.04013103	-0.00977920	0.02317404
249	-0.03891342	-0.00789103	0.02339685
250	-0.03757997	-0.00603269	0.02333239
251	-0.03612316	-0.00421851	0.02298515
252	-0.03457502	-0.00244218	0.02236291
253	-0.03298520	-0.00059142	0.02156995
254	-0.03104639	0.00094796	0.02023291
255	-0.02905013	0.00254424	0.01870258
256	-0.02719004	0.00426947	0.01701131
257	-0.01420492	-0.00895315	0.00083756
258	-0.01362852	-0.00923638	0.00155711
259	-0.01293438	-0.00949925	0.00213629
260	-0.01213240	-0.00968167	0.00251142
261	-0.01129125	-0.00982879	0.00280732
262	-0.01045304	-0.01007004	0.00295865
263	-0.00950672	-0.01035670	0.00294027
264	-0.00854636	-0.01069007	0.00289594
265	-0.00740862	-0.01091971	0.00315727
266	-0.00644379	-0.01139151	0.00217907
267	-0.00519335	-0.01148261	0.00169384
268	-0.00408083	-0.01161007	0.00118547
269	-0.00317717	-0.01171258	0.00058195
270	-0.00244467	-0.01181043	-0.00011340
271	-0.00186863	-0.01198125	-0.00087273
272	-0.00139618	-0.01214156	-0.00173581
273	-0.00106708	-0.01221241	-0.00271789
274	-0.00097424	-0.01225575	-0.00380308
275	-0.00109211	-0.01226381	-0.00495813
276	-0.00181558	-0.01255486	-0.00585019
277	-0.00265436	-0.01267661	-0.00655561
278	-0.00352794	-0.01272289	-0.00709354
279	-0.00444229	-0.01278634	-0.00742448
280	-0.00539185	-0.01279159	-0.00757620
281	-0.00639964	-0.01275603	-0.00755145
282	-0.00743214	-0.01262899	-0.00732805
283	-0.00859772	-0.01256264	-0.00696748
284	-0.00973407	-0.01239425	-0.00642912

285	-0.01078608	-0.01202259	-0.00585542
286	-0.01173719	-0.01165365	-0.00519610
287	-0.01251652	-0.01124299	-0.00448940
288	-0.01318365	-0.01085834	-0.00376242
289	-0.01370455	-0.01048911	-0.00298549
290	-0.01408853	-0.01012381	-0.00212894
291	-0.01431493	-0.00976411	-0.00119754
292	-0.01435066	-0.00937240	-0.00019292
293	0.02587333	0.01926473	0.01617969
294	0.02816657	0.01805224	0.01850146
295	0.03003528	0.01703775	0.02018269
296	0.03267677	0.01459136	0.02173532
297	0.03362073	0.01306809	0.02168953
298	0.03446001	0.01126160	0.02116429
299	0.03496819	0.00901716	0.01983755
300	0.03707364	0.01699636	-0.01060991
301	0.03923838	0.01638373	-0.01093459
302	0.04097023	0.01570535	-0.01111726
303	0.04219610	0.01478278	-0.01105536
304	0.04350500	0.01254245	-0.01048141
305	0.04375876	0.01115959	-0.00997934
306	0.04362935	0.00951276	-0.00926276
307	0.02070917	0.00418236	-0.01852877
308	0.02123998	0.00252670	-0.01979348
309	0.02187883	0.00107518	-0.02051511
310	0.02256028	-0.00014793	-0.02081252
311	0.02411657	-0.00211887	-0.02029710
312	0.02503308	-0.00299290	-0.01955280
313	0.02608090	-0.00378639	-0.01840111
314	0.03185011	0.00463990	0.01061681
315	0.03345782	0.00346569	0.01181123
316	0.03461084	0.00242664	0.01257250
317	0.03548554	0.00143281	0.01291878
318	0.03657419	-0.00059275	0.01250653
319	0.03685028	-0.00172227	0.01179215
320	0.03695813	-0.00299166	0.01069417
321	0.03846695	0.00676464	-0.00647510
322	0.04109393	0.00486686	-0.00675629
323	0.04276408	0.00408199	-0.00718175
324	0.04411850	0.00221221	-0.00702164
325	0.04422334	0.00123872	-0.00674590
326	0.04409352	0.00014020	-0.00634048
327	0.04365107	-0.00119541	-0.00575169
328	0.02812671	-0.00288162	-0.01147439
329	0.02834146	-0.00458265	-0.01324353
330	0.02884814	-0.00600743	-0.01412604
331	0.02997272	-0.00791211	-0.01445270
332	0.03060502	-0.00858125	-0.01405170
333	0.03135400	-0.00917384	-0.01333860
334	0.03225444	-0.00968688	-0.01218141
335	-0.01172028	-0.00127986	0.01812014
336	-0.00981966	-0.00017310	0.01596653
337	-0.00748292	0.00135292	0.01318677
338	-0.00467901	0.00176626	0.01257339
339	-0.00160492	0.00166516	0.01345472
340	0.00073520	0.00261184	0.01288804
341	0.00309907	0.00379113	0.01238177
342	0.00544885	0.00525394	0.01189193
343	0.00775190	0.00691334	0.01155331
344	0.01000933	0.00859471	0.01167153

345	0.01159026	0.00924354	0.01172904
346	0.01313523	0.00992465	0.01169330
347	0.01462754	0.01061938	0.01153682
348	0.01607257	0.01123832	0.01130270
349	0.01746758	0.01184949	0.01094656
350	0.01883896	0.01248093	0.01049223
351	0.02019423	0.01308261	0.00995225
352	0.02149643	0.01371791	0.00923701
353	0.02272847	0.01433868	0.00841663
354	0.02481547	0.01491058	0.00685853
355	0.02656942	0.01530765	0.00518982
356	0.02808847	0.01552710	0.00350573
357	0.02930815	0.01554265	0.00175550
358	0.03028629	0.01537772	-0.00000438
359	0.03107232	0.01504573	-0.00178924
360	0.03160693	0.01450721	-0.00360661
361	0.03177200	0.01365817	-0.00540187
362	0.03160557	0.01293630	-0.00744600
363	0.03106170	0.01175631	-0.00900205
364	0.03038092	0.01111919	-0.01031657
365	0.02952562	0.01019383	-0.01133570
366	0.02853786	0.00936300	-0.01204035
367	0.02748432	0.00852688	-0.01249330
368	0.02632847	0.00767429	-0.01272849
369	0.02507221	0.00679246	-0.01271952
370	0.02370023	0.00589785	-0.01246631
371	0.02219163	0.00511297	-0.01183295
372	0.02060253	0.00332031	-0.00966574
373	0.01936455	0.00241102	-0.00757266
374	0.01871548	0.00159697	-0.00553696
375	0.01852484	0.00115230	-0.00365728
376	0.01884631	0.00065988	-0.00185211
377	0.01974608	0.00010857	-0.00008481
378	0.02094316	-0.00003790	0.00176350
379	0.02291868	0.00010279	0.00337593
380	0.02528540	0.00115883	0.00421022
381	0.02774686	0.00133632	0.00399910
382	0.02960979	0.00217368	0.00353866
383	0.03088601	0.00273921	0.00281149
384	0.03169197	0.00299553	0.00187416
385	0.03231657	0.00294339	0.00088867
386	0.03279191	0.00259206	-0.00014395
387	0.03321028	0.00185201	-0.00142195
388	0.03280723	0.00113424	-0.00303939
389	0.03138479	0.00071611	-0.00423056
390	0.03074004	-0.00050220	-0.00429013
391	0.02978969	-0.00125837	-0.00387234
392	0.02934388	-0.00169521	-0.00329803
393	0.02954951	-0.00213744	-0.00293272
394	0.03011139	-0.00272617	-0.00249838
395	0.03123281	-0.00373416	-0.00218899
396	0.03270543	-0.00474862	-0.00183261
397	0.03581692	-0.00584704	-0.00153825
398	0.00175150	0.01384106	0.01986431
399	0.00395592	0.01323955	0.01977547
400	0.00636728	0.01320706	0.01958638
401	0.00877127	0.01345148	0.01923816
402	0.01114184	0.01399185	0.01872838
403	0.01349436	0.01464576	0.01796054
404	0.01589219	0.01557216	0.01697272

405	0.01835448	0.01668598	0.01584368
406	0.02091562	0.01787523	0.01456537
407	0.02362854	0.01904545	0.01310844
408	0.02615073	0.01994385	0.01081755
409	0.02836713	0.02052226	0.00846297
410	0.03029835	0.02075309	0.00605305
411	0.03188309	0.02071802	0.00358688
412	0.03312215	0.02044034	0.00105844
413	0.03402449	0.01992269	-0.00155966
414	0.03459416	0.01916495	-0.00424869
415	0.03481121	0.01814049	-0.00701032
416	0.03466972	0.01691722	-0.00986995
417	0.03359050	0.01554510	-0.01201557
418	0.03238080	0.01422233	-0.01382808
419	0.03102669	0.01296553	-0.01530783
420	0.02957444	0.01170741	-0.01642472
421	0.02800219	0.01049636	-0.01719415
422	0.02631005	0.00928329	-0.01761252
423	0.02448131	0.00808574	-0.01767455
424	0.02254429	0.00687127	-0.01737555
425	0.02058327	0.00558651	-0.01669829
426	0.01785690	0.00399632	-0.01437669
427	0.01585703	0.00261997	-0.01195234
428	0.01453051	0.00146287	-0.00946739
429	0.01384650	0.00056285	-0.00691465
430	0.01375780	-0.00001375	-0.00427298
431	0.01440182	-0.00041373	-0.00156825
432	0.01576558	-0.00059723	0.00122092
433	0.01780654	-0.00054486	0.00410058
434	0.02045436	-0.00015461	0.00707657
435	0.02296861	0.00095982	0.00803691
436	0.02522707	0.00206162	0.00864778
437	0.02727785	0.00306918	0.00889625
438	0.02911712	0.00395986	0.00878205
439	0.03075297	0.00470902	0.00828189
440	0.03219306	0.00534578	0.00740889
441	0.03341590	0.00592685	0.00616181
442	0.03451745	0.00625965	0.00452302
443	0.03543575	0.00641256	0.00252476
444	0.03585532	0.00629171	0.00071914
445	0.03609805	0.00604791	-0.00094565
446	0.03618289	0.00573616	-0.00249520
447	0.03608863	0.00534430	-0.00390653
448	0.03584725	0.00484086	-0.00518997
449	0.03540758	0.00424762	-0.00634779
450	0.03479334	0.00358544	-0.00738212
451	0.03401438	0.00281988	-0.00829128
452	0.03307100	0.00195507	-0.00905581
453	0.03197989	0.00104425	-0.00949076
454	0.03092847	0.00026206	-0.00970118
455	0.02998050	-0.00049538	-0.00966793
456	0.02907338	-0.00114972	-0.00940987
457	0.02824415	-0.00176644	-0.00889215
458	0.02748598	-0.00234658	-0.00810671
459	0.02687795	-0.00295596	-0.00702761
460	0.02643400	-0.00358347	-0.00567919
461	0.02622359	-0.00427971	-0.00412512
462	0.02697931	-0.00414388	-0.00228177
463	0.02783022	-0.00416996	-0.00083607
464	0.02872124	-0.00424154	0.00027241

465	0.02970001	-0.00433671	0.00101924
466	0.03066197	-0.00426093	0.00164923
467	0.03189605	-0.00440755	0.00162636
468	0.03374501	-0.00469686	0.00105143
469	0.03562753	-0.00567469	-0.00040085
470	-0.01405999	-0.00448353	0.01199846
471	-0.01327624	-0.00615359	0.01155577
472	-0.01218013	-0.00765257	0.01108889
473	-0.01089008	-0.00894656	0.01074745
474	-0.00952228	-0.01014643	0.01065119
475	-0.00802525	-0.01122904	0.01074947
476	-0.00648290	-0.01228996	0.01106501
477	-0.00526464	-0.01342736	0.01160294
478	-0.00284469	-0.01445149	0.01218824
479	-0.00235071	-0.01505638	0.01362634
480	-0.00057922	-0.01551150	0.01468553
481	0.00112223	-0.01579763	0.01578951
482	0.00277592	-0.01587528	0.01692713
483	0.00435767	-0.01575872	0.01807645
484	0.00588584	-0.01544568	0.01926245
485	0.00736047	-0.01496314	0.02049256
486	0.00878267	-0.01424004	0.02174220
487	0.01016040	-0.01325788	0.02300620
488	0.01150151	-0.01205326	0.02424520
489	0.01272474	-0.01063365	0.02541270
490	0.01394615	-0.00912507	0.02639944
491	0.01517522	-0.00756413	0.02728012
492	0.01643602	-0.00591284	0.02795602
493	0.01767138	-0.00416505	0.02847710
494	0.01893487	-0.00230851	0.02881471
495	0.02021383	-0.00038022	0.02899355
496	0.02150517	0.00165100	0.02897907
497	0.02279615	0.00377396	0.02871005
498	0.02479946	0.00683549	0.02788249
499	0.02678725	0.00955162	0.02661579
500	0.02876705	0.01190745	0.02497364
501	0.03075035	0.01392402	0.02302300
502	0.03276864	0.01564003	0.02077317
503	0.03480122	0.01701378	0.01814617
504	0.03685017	0.01804085	0.01513511
505	0.03885990	0.01867420	0.01169836
506	0.04071137	0.01880678	0.00783131
507	0.04234804	0.01842942	0.00319138
508	0.04319392	0.01758835	-0.00120791
509	0.04329672	0.01643156	-0.00516137
510	0.04283799	0.01510102	-0.00863886
511	0.04191714	0.01363323	-0.01167814
512	0.04058038	0.01206258	-0.01437005
513	0.03881765	0.01036889	-0.01677530
514	0.03653839	0.00849577	-0.01883607
515	0.03369222	0.00641862	-0.02043009
516	0.03121893	0.00480826	-0.02122917
517	0.02881560	0.00326752	-0.02153818
518	0.02653170	0.00180376	-0.02142990
519	0.02438529	0.00042885	-0.02093315
520	0.02235112	-0.00087884	-0.02012587
521	0.02043778	-0.00211728	-0.01893960
522	0.01862180	-0.00329883	-0.01740436
523	0.01691967	-0.00439601	-0.01547014
524	0.01539708	-0.00537614	-0.01311932

525	0.01436649	-0.00635815	-0.01077700
526	0.01369976	-0.00717745	-0.00841675
527	0.01337480	-0.00782977	-0.00607145
528	0.01334378	-0.00834772	-0.00375622
529	0.01362223	-0.00870699	-0.00147973
530	0.01416921	-0.00895092	0.00078057
531	0.01500797	-0.00907364	0.00304707
532	0.01619837	-0.00902670	0.00527835
533	0.01774937	-0.00877777	0.00743715
534	0.01943712	-0.00824749	0.00936803
535	0.02124334	-0.00752661	0.01093038
536	0.02308198	-0.00671600	0.01217647
537	0.02497056	-0.00579067	0.01311554
538	0.02693106	-0.00470307	0.01367410
539	0.02893472	-0.00360428	0.01407307
540	0.03101090	-0.00229582	0.01403004
541	0.03316389	-0.00088888	0.01367675
542	0.03533969	0.00060278	0.01283318
543	0.03767007	0.00181293	0.01145107
544	0.03961497	0.00276158	0.00973062
545	0.04116412	0.00344369	0.00784756
546	0.04240633	0.00390778	0.00583676
547	0.04334771	0.00412907	0.00370463
548	0.04403160	0.00415911	0.00139968
549	0.04438067	0.00396289	-0.00110620
550	0.04430687	0.00347775	-0.00380985
551	0.04372093	0.00269044	-0.00660298
552	0.04285048	0.00178208	-0.00882628
553	0.04174237	0.00079350	-0.01068087
554	0.04049535	-0.00023208	-0.01218732
555	0.03909850	-0.00130016	-0.01334704
556	0.03759087	-0.00240096	-0.01421729
557	0.03592539	-0.00354340	-0.01477669
558	0.03411289	-0.00474780	-0.01505795
559	0.03211970	-0.00599507	-0.01494660
560	0.03003332	-0.00725745	-0.01433042
561	0.02818582	-0.00857227	-0.01319741
562	0.02673746	-0.00964840	-0.01175889
563	0.02568626	-0.01052689	-0.01021597
564	0.02497676	-0.01122878	-0.00860865
565	0.02455074	-0.01182021	-0.00692180
566	0.02440537	-0.01230005	-0.00512194
567	0.02459443	-0.01265729	-0.00317997
568	0.02522170	-0.01269135	-0.00109534
569	0.02616218	-0.01232520	0.00112710
570	0.02799792	-0.01164154	0.00307416
571	0.02985354	-0.01086215	0.00428979
572	0.03149247	-0.01011763	0.00487646
573	0.03286284	-0.00952532	0.00507522
574	0.03415731	-0.00902859	0.00498691
575	0.03571407	-0.00844504	0.00434290
576	0.03719703	-0.00815875	0.00316565
577	0.03796199	-0.00783864	0.00091595
578	-0.00472278	-0.00591580	-0.01389955
579	-0.00282152	-0.00534411	-0.01307654
580	-0.00077831	-0.00526381	-0.01217037
581	0.00097025	-0.00534827	-0.01103326
582	0.00242991	-0.00558909	-0.00963486
583	0.00351485	-0.00586547	-0.00795511
584	0.00433010	-0.00629561	-0.00603422

585	0.00486325	-0.00686536	-0.00381865
586	0.00519077	-0.00760052	-0.00137754
587	0.00556083	-0.00865338	0.00112401
588	0.00646828	-0.01013170	0.00343617
589	0.00783613	-0.01149684	0.00551644
590	0.00930897	-0.01254350	0.00744219
591	0.01084713	-0.01319676	0.00926903
592	0.01241892	-0.01342319	0.01104797
593	0.01408775	-0.01328703	0.01276525
594	0.01583373	-0.01277122	0.01443718
595	0.01763102	-0.01183438	0.01607692
596	0.01947566	-0.01048279	0.01764952
597	0.02140151	-0.00861850	0.01893748
598	0.02317182	-0.00663277	0.01995184
599	0.02480619	-0.00457297	0.02065572
600	0.02639007	-0.00251694	0.02101676
601	0.02795184	-0.00048046	0.02101025
602	0.02950971	0.00154632	0.02063967
603	0.03113609	0.00352040	0.01987287
604	0.03288489	0.00539669	0.01874626
605	0.03474481	0.00711491	0.01730788
606	0.03686200	0.00862248	0.01474500
607	0.03872174	0.00971562	0.01211694
608	0.04029211	0.01043822	0.00941802
609	0.04158210	0.01077047	0.00664789
610	0.04252638	0.01077335	0.00380530
611	0.04314034	0.01041931	0.00085880
612	0.04337970	0.00977831	-0.00218957
613	0.04322285	0.00884295	-0.00533685
614	0.04260484	0.00769363	-0.00858368
615	0.04161695	0.00644682	-0.01099706
616	0.04035395	0.00523362	-0.01302552
617	0.03893777	0.00397600	-0.01467091
618	0.03733841	0.00270040	-0.01593965
619	0.03557586	0.00137918	-0.01681177
620	0.03364114	0.00002475	-0.01730930
621	0.03156233	-0.00139408	-0.01743314
622	0.02931854	-0.00288202	-0.01718814
623	0.02690239	-0.00442226	-0.01654399
624	0.02483326	-0.00585611	-0.01531786
625	0.02310872	-0.00708084	-0.01384842
626	0.02174057	-0.00812270	-0.01217845
627	0.02073371	-0.00900456	-0.01031061
628	0.02008726	-0.00970652	-0.00824582
629	0.01984304	-0.01036388	-0.00598328
630	0.01997215	-0.01087333	-0.00350758
631	0.02047542	-0.01124309	-0.00083346
632	0.02133162	-0.01140695	0.00203385
633	0.02270881	-0.01122814	0.00426263
634	0.02414832	-0.01080825	0.00614223
635	0.02564284	-0.01017136	0.00763857
636	0.02724265	-0.00941665	0.00876399
637	0.02893980	-0.00857386	0.00952246
638	0.03074246	-0.00759938	0.00990687
639	0.03266598	-0.00653779	0.00993561
640	0.03473008	-0.00542265	0.00962073
641	0.03688195	-0.00415521	0.00892264
642	0.03844788	-0.00340503	0.00785551
643	0.03978015	-0.00279195	0.00666462
644	0.04087124	-0.00232931	0.00534496

645	0.04173090	-0.00202117	0.00390837
646	0.04236048	-0.00186649	0.00235601
647	0.04276548	-0.00186371	0.00067766
648	0.04297459	-0.00204918	-0.00111624
649	0.04290986	-0.00235173	-0.00304896
650	0.04257675	-0.00277653	-0.00509931
651	0.04199268	-0.00337785	-0.00662625
652	0.04128121	-0.00403213	-0.00792095
653	0.04042464	-0.00471125	-0.00897988
654	0.03946348	-0.00545763	-0.00981281
655	0.03840840	-0.00624549	-0.01039310
656	0.03724789	-0.00710525	-0.01074771
657	0.03598539	-0.00803769	-0.01087718
658	0.03462609	-0.00903747	-0.01077708
659	0.03312538	-0.01004584	-0.01042879
660	0.03197561	-0.01098321	-0.00948970
661	0.03103462	-0.01173975	-0.00848024
662	0.03033323	-0.01236701	-0.00741586
663	0.02986373	-0.01285649	-0.00629548
664	0.02963806	-0.01321170	-0.00513194
665	0.02963912	-0.01344038	-0.00390085
666	0.02985399	-0.01349280	-0.00262084
667	0.03031734	-0.01340983	-0.00128204
668	0.03100931	-0.01318364	0.00011827
669	0.03213870	-0.01275362	0.00090662
670	0.03314645	-0.01225541	0.00146206
671	0.03410651	-0.01180367	0.00179509
672	0.03498578	-0.01133088	0.00191744
673	0.03582410	-0.01084690	0.00182995
674	0.03664555	-0.01030458	0.00150342
675	0.03744968	-0.00965418	0.00083948
676	0.03793364	-0.00861659	-0.00017299
677	-0.00929247	0.01399275	-0.00004503
678	-0.01283930	0.01696123	-0.00155635
679	-0.01665438	0.01954362	-0.00296851
680	-0.02025991	0.02198157	-0.00416642
681	-0.02350131	0.02441735	-0.00522117
682	-0.02664614	0.02660005	-0.00607178
683	-0.02988727	0.02846567	-0.00678561
684	-0.03277505	0.03017638	-0.00712933
685	-0.03566587	0.03171160	-0.00735360
686	-0.03850607	0.03290508	-0.00743352

Procrustes sums of squares: 0.3657145180346275
Tangent sums of squares: 0.35793716368340484

Data matrices in this dataset:
- Evolution Cervidae final, raw data
- Sans Ind & Capreolus, centroid size
- Sans Ind & Capreolus, Procrustes coordinates

New Procrustes fit:
Dataset 'Sans Ind & Capreolus'

686 landmarks in 3 dimensions.
The dataset contains 40 observations, of which 36 are included for analyses.

Average shape:

Lmk.	Axis 1 (x)	Axis 2 (y)	Axis 3 (z)
1	-0.04036362	0.02592451	-0.00811150
2	-0.02486511	-0.00981714	-0.02627340
3	-0.02630368	-0.01137480	-0.02731431
4	-0.02818995	-0.01223046	-0.02847610
5	-0.03017741	-0.01259941	-0.02952329
6	-0.03220235	-0.01261672	-0.03047543
7	-0.03431632	-0.01234278	-0.03134544
8	-0.03656948	-0.01172608	-0.03215652
9	-0.03897484	-0.01071316	-0.03289171
10	-0.04144477	-0.00924722	-0.03353796
11	-0.04391420	-0.00725860	-0.03406058
12	-0.04660985	-0.00439912	-0.03449515
13	-0.04870323	-0.00138086	-0.03453497
14	-0.05020484	0.00168057	-0.03420577
15	-0.05127422	0.00468345	-0.03352928
16	-0.05194588	0.00763278	-0.03255234
17	-0.05218638	0.01059840	-0.03122656
18	-0.05198752	0.01357426	-0.02955756
19	-0.05126707	0.01655038	-0.02751849
20	-0.04987035	0.01940290	-0.02515643
21	-0.04786808	0.02164297	-0.02204558
22	-0.04544731	0.02307481	-0.01919084
23	-0.04287669	0.02382276	-0.01656435
24	-0.04021532	0.02392447	-0.01415795
25	-0.03749705	0.02347117	-0.01185990
26	-0.03451674	0.02226598	-0.00987187
27	-0.03131650	0.02036366	-0.00814552
28	-0.02795494	0.01785428	-0.00653419
29	-0.02446861	0.01459152	-0.00530442
30	-0.02274492	0.01293029	-0.00496445
31	-0.02130127	0.01127595	-0.00504291
32	-0.02018054	0.00965077	-0.00552535
33	-0.01937887	0.00805876	-0.00639792
34	-0.01887320	0.00649494	-0.00763445
35	-0.01863959	0.00493692	-0.00923185
36	-0.01861014	0.00336333	-0.01114849
37	-0.01881318	0.00170255	-0.01342681
38	-0.01950246	0.00028609	-0.01612496
39	-0.02051828	-0.00009089	-0.01770052
40	-0.02170450	-0.00033282	-0.01922434
41	-0.02269031	-0.00088248	-0.02062383
42	-0.02347663	-0.00171594	-0.02187741
43	-0.02404150	-0.00284644	-0.02298066
44	-0.02444990	-0.00424358	-0.02398036
45	-0.02463759	-0.00594249	-0.02481416
46	-0.02463999	-0.00790540	-0.02549130
47	-0.01971082	-0.01429760	-0.02654986
48	-0.02207603	-0.01533880	-0.02788349
49	-0.02452514	-0.01601771	-0.02907491
50	-0.02697985	-0.01635938	-0.03012478
51	-0.02938783	-0.01643944	-0.03108082
52	-0.03172828	-0.01627283	-0.03197942
53	-0.03405324	-0.01585177	-0.03278889
54	-0.03635762	-0.01517846	-0.03352073
55	-0.03864152	-0.01423175	-0.03417591
56	-0.04088868	-0.01300501	-0.03472953
57	-0.04298360	-0.01153854	-0.03527308

58	-0.04489822	-0.00994488	-0.03566984
59	-0.04665507	-0.00824501	-0.03596318
60	-0.04822977	-0.00641306	-0.03612856
61	-0.04969089	-0.00450700	-0.03623035
62	-0.05095120	-0.00244136	-0.03614721
63	-0.05204945	-0.00025523	-0.03596464
64	-0.05297543	0.00205276	-0.03560948
65	-0.05371134	0.00447999	-0.03512109
66	-0.05419957	0.00667575	-0.03445464
67	-0.05451852	0.00881152	-0.03371248
68	-0.05466005	0.01088919	-0.03286446
69	-0.05459579	0.01289528	-0.03188451
70	-0.05437111	0.01483793	-0.03081159
71	-0.05400202	0.01676676	-0.02966597
72	-0.05338614	0.01857604	-0.02833334
73	-0.05259955	0.02033943	-0.02692452
74	-0.05160894	0.02199484	-0.02538766
75	-0.05032088	0.02348538	-0.02386987
76	-0.04889424	0.02477045	-0.02222484
77	-0.04734848	0.02583566	-0.02045486
78	-0.04568356	0.02668656	-0.01854892
79	-0.04389958	0.02729224	-0.01651489
80	-0.04196674	0.02768918	-0.01436050
81	-0.03993372	0.02788108	-0.01206327
82	-0.03778278	0.02786411	-0.00962310
83	-0.03550002	0.02805675	-0.00713649
84	-0.02475516	0.00500360	0.02164341
85	-0.02663734	0.00345875	0.02300711
86	-0.02893429	0.00246871	0.02432777
87	-0.03125222	0.00179496	0.02541824
88	-0.03351854	0.00126713	0.02624892
89	-0.03576266	0.00091725	0.02689810
90	-0.03801045	0.00073757	0.02735358
91	-0.04026958	0.00071849	0.02765156
92	-0.04255395	0.00091244	0.02778557
93	-0.04484174	0.00133713	0.02770046
94	-0.04746711	0.00203698	0.02715615
95	-0.04978796	0.00300011	0.02632534
96	-0.05182017	0.00415271	0.02524752
97	-0.05352649	0.00550637	0.02392747
98	-0.05500355	0.00695445	0.02240050
99	-0.05624612	0.00856683	0.02065234
100	-0.05722574	0.01032876	0.01866341
101	-0.05794862	0.01226252	0.01643623
102	-0.05829858	0.01437348	0.01400590
103	-0.05820941	0.01695260	0.01097585
104	-0.05748421	0.01920360	0.00826470
105	-0.05622342	0.02109539	0.00589852
106	-0.05456535	0.02262365	0.00381355
107	-0.05245204	0.02378773	0.00203401
108	-0.04992802	0.02461653	0.00047559
109	-0.04691429	0.02511807	-0.00083014
110	-0.04338655	0.02524868	-0.00180378
111	-0.03939899	0.02500765	-0.00219625
112	-0.03571669	0.02334225	-0.00172767
113	-0.03242682	0.02173363	-0.00074659
114	-0.02951264	0.02020953	0.00028187
115	-0.02694310	0.01876207	0.00131585
116	-0.02471578	0.01734922	0.00240066
117	-0.02278449	0.01593180	0.00362812

118	-0.02116758	0.01449232	0.00516745
119	-0.02005743	0.01321894	0.00717437
120	-0.01982002	0.01265052	0.00928625
121	-0.02048050	0.01276028	0.01082980
122	-0.02140577	0.01295381	0.01235614
123	-0.02221766	0.01279049	0.01383239
124	-0.02290441	0.01229057	0.01525892
125	-0.02344077	0.01132780	0.01664227
126	-0.02388330	0.01016281	0.01795521
127	-0.02417959	0.00871239	0.01919274
128	-0.02428154	0.00676757	0.02037126
129	-0.01889190	0.00302862	0.02394466
130	-0.02110260	0.00214561	0.02522712
131	-0.02349788	0.00139899	0.02617422
132	-0.02586912	0.00076067	0.02701963
133	-0.02819728	0.00019760	0.02778945
134	-0.03049387	-0.00028099	0.02849185
135	-0.03278938	-0.00066872	0.02911548
136	-0.03508840	-0.00096192	0.02966257
137	-0.03739207	-0.00114914	0.03004513
138	-0.03968475	-0.00124508	0.03031424
139	-0.04199260	-0.00107408	0.03043188
140	-0.04419000	-0.00078425	0.03037969
141	-0.04626033	-0.00034011	0.03007118
142	-0.04822308	0.00018904	0.02966283
143	-0.05005434	0.00084689	0.02904747
144	-0.05177324	0.00165080	0.02827374
145	-0.05339725	0.00256569	0.02734367
146	-0.05488949	0.00361482	0.02622050
147	-0.05627996	0.00479674	0.02496187
148	-0.05780268	0.00638624	0.02325633
149	-0.05900770	0.00801934	0.02146877
150	-0.05993875	0.00967549	0.01961293
151	-0.06065928	0.01137019	0.01772820
152	-0.06111308	0.01310885	0.01578557
153	-0.06132017	0.01486941	0.01377255
154	-0.06123036	0.01664026	0.01167593
155	-0.06084204	0.01843626	0.00949995
156	-0.06010631	0.02023352	0.00726995
157	-0.05876156	0.02231800	0.00499395
158	-0.05702064	0.02406532	0.00291125
159	-0.05495415	0.02551484	0.00103142
160	-0.05263172	0.02673985	-0.00076846
161	-0.04991975	0.02759773	-0.00236395
162	-0.04685180	0.02816012	-0.00379531
163	-0.04340435	0.02836941	-0.00505424
164	-0.03957295	0.02841727	-0.00620094
165	-0.03550002	0.02805675	-0.00713649
166	-0.02499274	-0.01797812	-0.01144966
167	-0.02594114	-0.01926394	-0.01127705
168	-0.02715963	-0.02032120	-0.01063659
169	-0.02835612	-0.02123163	-0.00977690
170	-0.02953381	-0.02203539	-0.00869693
171	-0.03072663	-0.02273138	-0.00741166
172	-0.03191706	-0.02340512	-0.00589307
173	-0.03311180	-0.02396404	-0.00412300
174	-0.03431553	-0.02442274	-0.00211313
175	-0.03556056	-0.02468571	0.00012660
176	-0.03649211	-0.02473268	0.00178126
177	-0.03733966	-0.02465161	0.00340695

178	-0.03820179	-0.02302148	0.00481856
179	-0.03880320	-0.02403014	0.00653744
180	-0.03941172	-0.02351355	0.00803519
181	-0.03995039	-0.02286350	0.00951174
182	-0.04039727	-0.02207565	0.01095243
183	-0.04079079	-0.02116340	0.01237129
184	-0.04109684	-0.02011810	0.01374697
185	-0.04130890	-0.01876998	0.01527170
186	-0.04134585	-0.01740235	0.01661532
187	-0.04122223	-0.01601967	0.01784617
188	-0.04086897	-0.01457793	0.01871371
189	-0.04035602	-0.01312194	0.01951769
190	-0.03964819	-0.01161107	0.02003250
191	-0.03873596	-0.01005176	0.02032875
192	-0.03763316	-0.00846149	0.02042424
193	-0.03633865	-0.00682257	0.02029050
194	-0.03442252	-0.00477936	0.01989045
195	-0.03261396	-0.00294886	0.01921052
196	-0.03090104	-0.00137572	0.01823817
197	-0.02927633	-0.00002425	0.01701181
198	-0.02777946	0.00114102	0.01557634
199	-0.02638943	0.00209796	0.01386781
200	-0.02507999	0.00277584	0.01189396
201	-0.02383005	0.00335102	0.00971822
202	-0.02268630	0.00367457	0.00728109
203	-0.02198686	0.00350864	0.00526205
204	-0.02150943	0.00305574	0.00328699
205	-0.02109940	0.00246504	0.00146270
206	-0.02070629	0.00172063	-0.00021211
207	-0.02031526	0.00079952	-0.00175701
208	-0.02001998	-0.00032600	-0.00321626
209	-0.01977998	-0.00165156	-0.00460495
210	-0.01972435	-0.00318700	-0.00591164
211	-0.02012169	-0.00482420	-0.00683380
212	-0.02086359	-0.00632844	-0.00688795
213	-0.02179806	-0.00781622	-0.00684819
214	-0.02269357	-0.00929351	-0.00694264
215	-0.02340306	-0.01076302	-0.00724541
216	-0.02389958	-0.01220369	-0.00778537
217	-0.02423287	-0.01365489	-0.00854983
218	-0.02439367	-0.01512932	-0.00955153
219	-0.02446461	-0.01665275	-0.01071370
220	-0.02301658	-0.01983875	-0.01657561
221	-0.02449962	-0.02103703	-0.01580409
222	-0.02591209	-0.02210145	-0.01482174
223	-0.02725720	-0.02308326	-0.01371343
224	-0.02855787	-0.02398036	-0.01249724
225	-0.02979391	-0.02477324	-0.01114796
226	-0.03097478	-0.02545093	-0.00967183
227	-0.03210955	-0.02607016	-0.00809480
228	-0.03319008	-0.02659988	-0.00638774
229	-0.03426191	-0.02708908	-0.00460141
230	-0.03533030	-0.02742900	-0.00265247
231	-0.03638312	-0.02766692	-0.00077013
232	-0.03734717	-0.02770520	0.00110545
233	-0.03826857	-0.02760746	0.00292934
234	-0.03908166	-0.02730438	0.00475011
235	-0.03985656	-0.02685237	0.00652661
236	-0.04056376	-0.02621526	0.00828375
237	-0.04119816	-0.02541774	0.01001183

238	-0.04177051	-0.02445017	0.01171237
239	-0.04234573	-0.02326985	0.01357121
240	-0.04280543	-0.02204921	0.01527783
241	-0.04307339	-0.02075208	0.01680627
242	-0.04316928	-0.01938416	0.01818845
243	-0.04309833	-0.01796702	0.01938745
244	-0.04288469	-0.01648776	0.02045101
245	-0.04251190	-0.01496819	0.02134714
246	-0.04194658	-0.01335978	0.02208120
247	-0.04122537	-0.01171653	0.02264389
248	-0.04013103	-0.00977920	0.02317404
249	-0.03891342	-0.00789103	0.02339685
250	-0.03757997	-0.00603269	0.02333239
251	-0.03612316	-0.00421851	0.02298515
252	-0.03457502	-0.00244218	0.02236291
253	-0.03298520	-0.00059142	0.02156995
254	-0.03104639	0.00094796	0.02023291
255	-0.02905013	0.00254424	0.01870258
256	-0.02719004	0.00426947	0.01701131
257	-0.01420492	-0.00895315	0.00083756
258	-0.01362852	-0.00923638	0.00155711
259	-0.01293438	-0.00949925	0.00213629
260	-0.01213240	-0.00968167	0.00251142
261	-0.01129125	-0.00982879	0.00280732
262	-0.01045304	-0.01007004	0.00295865
263	-0.00950672	-0.01035670	0.00294027
264	-0.00854636	-0.01069007	0.00289594
265	-0.00740862	-0.01091971	0.00315727
266	-0.00644379	-0.01139151	0.00217907
267	-0.00519335	-0.01148261	0.00169384
268	-0.00408083	-0.01161007	0.00118547
269	-0.00317717	-0.01171258	0.00058195
270	-0.00244467	-0.01181043	-0.00011340
271	-0.00186863	-0.01198125	-0.00087273
272	-0.00139618	-0.01214156	-0.00173581
273	-0.00106708	-0.01221241	-0.00271789
274	-0.00097424	-0.01225575	-0.00380308
275	-0.00109211	-0.01226381	-0.00495813
276	-0.00181558	-0.01255486	-0.00585019
277	-0.00265436	-0.01267661	-0.00655561
278	-0.00352794	-0.01272289	-0.00709354
279	-0.00444229	-0.01278634	-0.00742448
280	-0.00539185	-0.01279159	-0.00757620
281	-0.00639964	-0.01275603	-0.00755145
282	-0.00743214	-0.01262899	-0.00732805
283	-0.00859772	-0.01256264	-0.00696748
284	-0.00973407	-0.01239425	-0.00642912
285	-0.01078608	-0.01202259	-0.00585542
286	-0.01173719	-0.01165365	-0.00519610
287	-0.01251652	-0.01124299	-0.00448940
288	-0.01318365	-0.01085834	-0.00376242
289	-0.01370455	-0.01048911	-0.00298549
290	-0.01408853	-0.01012381	-0.00212894
291	-0.01431493	-0.00976411	-0.00119754
292	-0.01435066	-0.00937240	-0.00019292
293	0.02587333	0.01926473	0.01617969
294	0.02816657	0.01805224	0.01850146
295	0.03003528	0.01703775	0.02018269
296	0.03267677	0.01459136	0.02173532
297	0.03362073	0.01306809	0.02168953

298	0.03446001	0.01126160	0.02116429
299	0.03496819	0.00901716	0.01983755
300	0.03707364	0.01699636	-0.01060991
301	0.03923838	0.01638373	-0.01093459
302	0.04097023	0.01570535	-0.01111726
303	0.04219610	0.01478278	-0.01105536
304	0.04350500	0.01254245	-0.01048141
305	0.04375876	0.01115959	-0.00997934
306	0.04362935	0.00951276	-0.00926276
307	0.02070917	0.00418236	-0.01852877
308	0.02123998	0.00252670	-0.01979348
309	0.02187883	0.00107518	-0.02051511
310	0.02256028	-0.00014793	-0.02081252
311	0.02411657	-0.00211887	-0.02029710
312	0.02503308	-0.00299290	-0.01955280
313	0.02608090	-0.00378639	-0.01840111
314	0.03185011	0.00463990	0.01061681
315	0.03345782	0.00346569	0.01181123
316	0.03461084	0.00242664	0.01257250
317	0.03548554	0.00143281	0.01291878
318	0.03657419	-0.00059275	0.01250653
319	0.03685028	-0.00172227	0.01179215
320	0.03695813	-0.00299166	0.01069417
321	0.03846695	0.00676464	-0.00647510
322	0.04109393	0.00486686	-0.00675629
323	0.04276408	0.00408199	-0.00718175
324	0.04411850	0.00221221	-0.00702164
325	0.04422334	0.00123872	-0.00674590
326	0.04409352	0.00014020	-0.00634048
327	0.04365107	-0.00119541	-0.00575169
328	0.02812671	-0.00288162	-0.01147439
329	0.02834146	-0.00458265	-0.01324353
330	0.02884814	-0.00600743	-0.01412604
331	0.02997272	-0.00791211	-0.01445270
332	0.03060502	-0.00858125	-0.01405170
333	0.03135400	-0.00917384	-0.01333860
334	0.03225444	-0.00968688	-0.01218141
335	-0.01172028	-0.00127986	0.01812014
336	-0.00981966	-0.00017310	0.01596653
337	-0.00748292	0.00135292	0.01318677
338	-0.00467901	0.00176626	0.01257339
339	-0.00160492	0.00166516	0.01345472
340	0.00073520	0.00261184	0.01288804
341	0.00309907	0.00379113	0.01238177
342	0.00544885	0.00525394	0.01189193
343	0.00775190	0.00691334	0.01155331
344	0.01000933	0.00859471	0.01167153
345	0.01159026	0.00924354	0.01172904
346	0.01313523	0.00992465	0.01169330
347	0.01462754	0.01061938	0.01153682
348	0.01607257	0.01123832	0.01130270
349	0.01746758	0.01184949	0.01094656
350	0.01883896	0.01248093	0.01049223
351	0.02019423	0.01308261	0.00995225
352	0.02149643	0.01371791	0.00923701
353	0.02272847	0.01433868	0.00841663
354	0.02481547	0.01491058	0.00685853
355	0.02656942	0.01530765	0.00518982
356	0.02808847	0.01552710	0.00350573
357	0.02930815	0.01554265	0.00175550

358	0.03028629	0.01537772	-0.00000438
359	0.03107232	0.01504573	-0.00178924
360	0.03160693	0.01450721	-0.00360661
361	0.03177200	0.01365817	-0.00540187
362	0.03160557	0.01293630	-0.00744600
363	0.03106170	0.01175631	-0.00900205
364	0.03038092	0.01111919	-0.01031657
365	0.02952562	0.01019383	-0.01133570
366	0.02853786	0.00936300	-0.01204035
367	0.02748432	0.00852688	-0.01249330
368	0.02632847	0.00767429	-0.01272849
369	0.02507221	0.00679246	-0.01271952
370	0.02370023	0.00589785	-0.01246631
371	0.02219163	0.00511297	-0.01183295
372	0.02060253	0.00332031	-0.00966574
373	0.01936455	0.00241102	-0.00757266
374	0.01871548	0.00159697	-0.00553696
375	0.01852484	0.00115230	-0.00365728
376	0.01884631	0.00065988	-0.00185211
377	0.01974608	0.00010857	-0.00008481
378	0.02094316	-0.00003790	0.00176350
379	0.02291868	0.00010279	0.00337593
380	0.02528540	0.00115883	0.00421022
381	0.02774686	0.00133632	0.00399910
382	0.02960979	0.00217368	0.00353866
383	0.03088601	0.00273921	0.00281149
384	0.03169197	0.00299553	0.00187416
385	0.03231657	0.00294339	0.00088867
386	0.03279191	0.00259206	-0.00014395
387	0.03321028	0.00185201	-0.00142195
388	0.03280723	0.00113424	-0.00303939
389	0.03138479	0.00071611	-0.00423056
390	0.03074004	-0.00050220	-0.00429013
391	0.02978969	-0.00125837	-0.00387234
392	0.02934388	-0.00169521	-0.00329803
393	0.02954951	-0.00213744	-0.00293272
394	0.03011139	-0.00272617	-0.00249838
395	0.03123281	-0.00373416	-0.00218899
396	0.03270543	-0.00474862	-0.00183261
397	0.03581692	-0.00584704	-0.00153825
398	0.00175150	0.01384106	0.01986431
399	0.00395592	0.01323955	0.01977547
400	0.00636728	0.01320706	0.01958638
401	0.00877127	0.01345148	0.01923816
402	0.01114184	0.01399185	0.01872838
403	0.01349436	0.01464576	0.01796054
404	0.01589219	0.01557216	0.01697272
405	0.01835448	0.01668598	0.01584368
406	0.02091562	0.01787523	0.01456537
407	0.02362854	0.01904545	0.01310844
408	0.02615073	0.01994385	0.01081755
409	0.02836713	0.02052226	0.00846297
410	0.03029835	0.02075309	0.00605305
411	0.03188309	0.02071802	0.00358688
412	0.03312215	0.02044034	0.00105844
413	0.03402449	0.01992269	-0.00155966
414	0.03459416	0.01916495	-0.00424869
415	0.03481121	0.01814049	-0.00701032
416	0.03466972	0.01691722	-0.00986995
417	0.03359050	0.01554510	-0.01201557

418	0.03238080	0.01422233	-0.01382808
419	0.03102669	0.01296553	-0.01530783
420	0.02957444	0.01170741	-0.01642472
421	0.02800219	0.01049636	-0.01719415
422	0.02631005	0.00928329	-0.01761252
423	0.02448131	0.00808574	-0.01767455
424	0.02254429	0.00687127	-0.01737555
425	0.02058327	0.00558651	-0.01669829
426	0.01785690	0.00399632	-0.01437669
427	0.01585703	0.00261997	-0.01195234
428	0.01453051	0.00146287	-0.00946739
429	0.01384650	0.00056285	-0.00691465
430	0.01375780	-0.00001375	-0.00427298
431	0.01440182	-0.00041373	-0.00156825
432	0.01576558	-0.00059723	0.00122092
433	0.01780654	-0.00054486	0.00410058
434	0.02045436	-0.00015461	0.00707657
435	0.02296861	0.00095982	0.00803691
436	0.02522707	0.00206162	0.00864778
437	0.02727785	0.00306918	0.00889625
438	0.02911712	0.00395986	0.00878205
439	0.03075297	0.00470902	0.00828189
440	0.03219306	0.00534578	0.00740889
441	0.03341590	0.00592685	0.00616181
442	0.03451745	0.00625965	0.00452302
443	0.03543575	0.00641256	0.00252476
444	0.03585532	0.00629171	0.00071914
445	0.03609805	0.00604791	-0.00094565
446	0.03618289	0.00573616	-0.00249520
447	0.03608863	0.00534430	-0.00390653
448	0.03584725	0.00484086	-0.00518997
449	0.03540758	0.00424762	-0.00634779
450	0.03479334	0.00358544	-0.00738212
451	0.03401438	0.00281988	-0.00829128
452	0.03307100	0.00195507	-0.00905581
453	0.03197989	0.00104425	-0.00949076
454	0.03092847	0.00026206	-0.00970118
455	0.02998050	-0.00049538	-0.00966793
456	0.02907338	-0.00114972	-0.00940987
457	0.02824415	-0.00176644	-0.00889215
458	0.02748598	-0.00234658	-0.00810671
459	0.02687795	-0.00295596	-0.00702761
460	0.02643400	-0.00358347	-0.00567919
461	0.02622359	-0.00427971	-0.00412512
462	0.02697931	-0.00414388	-0.00228177
463	0.02783022	-0.00416996	-0.00083607
464	0.02872124	-0.00424154	0.00027241
465	0.02970001	-0.00433671	0.00101924
466	0.03066197	-0.00426093	0.00164923
467	0.03189605	-0.00440755	0.00162636
468	0.03374501	-0.00469686	0.00105143
469	0.03562753	-0.00567469	-0.00040085
470	-0.01405999	-0.00448353	0.01199846
471	-0.01327624	-0.00615359	0.01155577
472	-0.01218013	-0.00765257	0.01108889
473	-0.01089008	-0.00894656	0.01074745
474	-0.00952228	-0.01014643	0.01065119
475	-0.00802525	-0.01122904	0.01074947
476	-0.00648290	-0.01228996	0.01106501
477	-0.00526464	-0.01342736	0.01160294

478	-0.00284469	-0.01445149	0.01218824
479	-0.00235071	-0.01505638	0.01362634
480	-0.00057922	-0.01551150	0.01468553
481	0.00112223	-0.01579763	0.01578951
482	0.00277592	-0.01587528	0.01692713
483	0.00435767	-0.01575872	0.01807645
484	0.00588584	-0.01544568	0.01926245
485	0.00736047	-0.01496314	0.02049256
486	0.00878267	-0.01424004	0.02174220
487	0.01016040	-0.01325788	0.02300620
488	0.01150151	-0.01205326	0.02424520
489	0.01272474	-0.01063365	0.02541270
490	0.01394615	-0.00912507	0.02639944
491	0.01517522	-0.00756413	0.02728012
492	0.01643602	-0.00591284	0.02795602
493	0.01767138	-0.00416505	0.02847710
494	0.01893487	-0.00230851	0.02881471
495	0.02021383	-0.00038022	0.02899355
496	0.02150517	0.00165100	0.02897907
497	0.02279615	0.00377396	0.02871005
498	0.02479946	0.00683549	0.02788249
499	0.02678725	0.00955162	0.02661579
500	0.02876705	0.01190745	0.02497364
501	0.03075035	0.01392402	0.02302300
502	0.03276864	0.01564003	0.02077317
503	0.03480122	0.01701378	0.01814617
504	0.03685017	0.01804085	0.01513511
505	0.03885990	0.01867420	0.01169836
506	0.04071137	0.01880678	0.00783131
507	0.04234804	0.01842942	0.00319138
508	0.04319392	0.01758835	-0.00120791
509	0.04329672	0.01643156	-0.00516137
510	0.04283799	0.01510102	-0.00863886
511	0.04191714	0.01363323	-0.01167814
512	0.04058038	0.01206258	-0.01437005
513	0.03881765	0.01036889	-0.01677530
514	0.03653839	0.00849577	-0.01883607
515	0.03369222	0.00641862	-0.02043009
516	0.03121893	0.00480826	-0.02122917
517	0.02881560	0.00326752	-0.02153818
518	0.02653170	0.00180376	-0.02142990
519	0.02438529	0.00042885	-0.02093315
520	0.02235112	-0.00087884	-0.02012587
521	0.02043778	-0.00211728	-0.01893960
522	0.01862180	-0.00329883	-0.01740436
523	0.01691967	-0.00439601	-0.01547014
524	0.01539708	-0.00537614	-0.01311932
525	0.01436649	-0.00635815	-0.01077700
526	0.01369976	-0.00717745	-0.00841675
527	0.01337480	-0.00782977	-0.00607145
528	0.01334378	-0.00834772	-0.00375622
529	0.01362223	-0.00870699	-0.00147973
530	0.01416921	-0.00895092	0.00078057
531	0.01500797	-0.00907364	0.00304707
532	0.01619837	-0.00902670	0.00527835
533	0.01774937	-0.00877777	0.00743715
534	0.01943712	-0.00824749	0.00936803
535	0.02124334	-0.00752661	0.01093038
536	0.02308198	-0.00671600	0.01217647
537	0.02497056	-0.00579067	0.01311554

538	0.02693106	-0.00470307	0.01367410
539	0.02893472	-0.00360428	0.01407307
540	0.03101090	-0.00229582	0.01403004
541	0.03316389	-0.00088888	0.01367675
542	0.03533969	0.00060278	0.01283318
543	0.03767007	0.00181293	0.01145107
544	0.03961497	0.00276158	0.00973062
545	0.04116412	0.00344369	0.00784756
546	0.04240633	0.00390778	0.00583676
547	0.04334771	0.00412907	0.00370463
548	0.04403160	0.00415911	0.00139968
549	0.04438067	0.00396289	-0.00110620
550	0.04430687	0.00347775	-0.00380985
551	0.04372093	0.00269044	-0.00660298
552	0.04285048	0.00178208	-0.00882628
553	0.04174237	0.00079350	-0.01068087
554	0.04049535	-0.00023208	-0.01218732
555	0.03909850	-0.00130016	-0.01334704
556	0.03759087	-0.00240096	-0.01421729
557	0.03592539	-0.00354340	-0.01477669
558	0.03411289	-0.00474780	-0.01505795
559	0.03211970	-0.00599507	-0.01494660
560	0.03003332	-0.00725745	-0.01433042
561	0.02818582	-0.00857227	-0.01319741
562	0.02673746	-0.00964840	-0.01175889
563	0.02568626	-0.01052689	-0.01021597
564	0.02497676	-0.01122878	-0.00860865
565	0.02455074	-0.01182021	-0.00692180
566	0.02440537	-0.01230005	-0.00512194
567	0.02459443	-0.01265729	-0.00317997
568	0.02522170	-0.01269135	-0.00109534
569	0.02616218	-0.01232520	0.00112710
570	0.02799792	-0.01164154	0.00307416
571	0.02985354	-0.01086215	0.00428979
572	0.03149247	-0.01011763	0.00487646
573	0.03286284	-0.00952532	0.00507522
574	0.03415731	-0.00902859	0.00498691
575	0.03571407	-0.00844504	0.00434290
576	0.03719703	-0.00815875	0.00316565
577	0.03796199	-0.00783864	0.00091595
578	-0.00472278	-0.00591580	-0.01389955
579	-0.00282152	-0.00534411	-0.01307654
580	-0.00077831	-0.00526381	-0.01217037
581	0.00097025	-0.00534827	-0.01103326
582	0.00242991	-0.00558909	-0.00963486
583	0.00351485	-0.00586547	-0.00795511
584	0.00433010	-0.00629561	-0.00603422
585	0.00486325	-0.00686536	-0.00381865
586	0.00519077	-0.00760052	-0.00137754
587	0.00556083	-0.00865338	0.00112401
588	0.00646828	-0.01013170	0.00343617
589	0.00783613	-0.01149684	0.00551644
590	0.00930897	-0.01254350	0.00744219
591	0.01084713	-0.01319676	0.00926903
592	0.01241892	-0.01342319	0.01104797
593	0.01408775	-0.01328703	0.01276525
594	0.01583373	-0.01277122	0.01443718
595	0.01763102	-0.01183438	0.01607692
596	0.01947566	-0.01048279	0.01764952
597	0.02140151	-0.00861850	0.01893748

598	0.02317182	-0.00663277	0.01995184
599	0.02480619	-0.00457297	0.02065572
600	0.02639007	-0.00251694	0.02101676
601	0.02795184	-0.00048046	0.02101025
602	0.02950971	0.00154632	0.02063967
603	0.03113609	0.00352040	0.01987287
604	0.03288489	0.00539669	0.01874626
605	0.03474481	0.00711491	0.01730788
606	0.03686200	0.00862248	0.01474500
607	0.03872174	0.00971562	0.01211694
608	0.04029211	0.01043822	0.00941802
609	0.04158210	0.01077047	0.00664789
610	0.04252638	0.01077335	0.00380530
611	0.04314034	0.01041931	0.00085880
612	0.04337970	0.00977831	-0.00218957
613	0.04322285	0.00884295	-0.00533685
614	0.04260484	0.00769363	-0.00858368
615	0.04161695	0.00644682	-0.01099706
616	0.04035395	0.00523362	-0.01302552
617	0.03893777	0.00397600	-0.01467091
618	0.03733841	0.00270040	-0.01593965
619	0.03557586	0.00137918	-0.01681177
620	0.03364114	0.00002475	-0.01730930
621	0.03156233	-0.00139408	-0.01743314
622	0.02931854	-0.00288202	-0.01718814
623	0.02690239	-0.00442226	-0.01654399
624	0.02483326	-0.00585611	-0.01531786
625	0.02310872	-0.00708084	-0.01384842
626	0.02174057	-0.00812270	-0.01217845
627	0.02073371	-0.00900456	-0.01031061
628	0.02008726	-0.00970652	-0.00824582
629	0.01984304	-0.01036388	-0.00598328
630	0.01997215	-0.01087333	-0.00350758
631	0.02047542	-0.01124309	-0.00083346
632	0.02133162	-0.01140695	0.00203385
633	0.02270881	-0.01122814	0.00426263
634	0.02414832	-0.01080825	0.00614223
635	0.02564284	-0.01017136	0.00763857
636	0.02724265	-0.00941665	0.00876399
637	0.02893980	-0.00857386	0.00952246
638	0.03074246	-0.00759938	0.00990687
639	0.03266598	-0.00653779	0.00993561
640	0.03473008	-0.00542265	0.00962073
641	0.03688195	-0.00415521	0.00892264
642	0.03844788	-0.00340503	0.00785551
643	0.03978015	-0.00279195	0.00666462
644	0.04087124	-0.00232931	0.00534496
645	0.04173090	-0.00202117	0.00390837
646	0.04236048	-0.00186649	0.00235601
647	0.04276548	-0.00186371	0.00067766
648	0.04297459	-0.00204918	-0.00111624
649	0.04290986	-0.00235173	-0.00304896
650	0.04257675	-0.00277653	-0.00509931
651	0.04199268	-0.00337785	-0.00662625
652	0.04128121	-0.00403213	-0.00792095
653	0.04042464	-0.00471125	-0.00897988
654	0.03946348	-0.00545763	-0.00981281
655	0.03840840	-0.00624549	-0.01039310
656	0.03724789	-0.00710525	-0.01074771
657	0.03598539	-0.00803769	-0.01087718

658	0.03462609	-0.00903747	-0.01077708
659	0.03312538	-0.01004584	-0.01042879
660	0.03197561	-0.01098321	-0.00948970
661	0.03103462	-0.01173975	-0.00848024
662	0.03033323	-0.01236701	-0.00741586
663	0.02986373	-0.01285649	-0.00629548
664	0.02963806	-0.01321170	-0.00513194
665	0.02963912	-0.01344038	-0.00390085
666	0.02985399	-0.01349280	-0.00262084
667	0.03031734	-0.01340983	-0.00128204
668	0.03100931	-0.01318364	0.00011827
669	0.03213870	-0.01275362	0.00090662
670	0.03314645	-0.01225541	0.00146206
671	0.03410651	-0.01180367	0.00179509
672	0.03498578	-0.01133088	0.00191744
673	0.03582410	-0.01084690	0.00182995
674	0.03664555	-0.01030458	0.00150342
675	0.03744968	-0.00965418	0.00083948
676	0.03793364	-0.00861659	-0.00017299
677	-0.00929247	0.01399275	-0.00004503
678	-0.01283930	0.01696123	-0.00155635
679	-0.01665438	0.01954362	-0.00296851
680	-0.02025991	0.02198157	-0.00416642
681	-0.02350131	0.02441735	-0.00522117
682	-0.02664614	0.02660005	-0.00607178
683	-0.02988727	0.02846567	-0.00678561
684	-0.03277505	0.03017638	-0.00712933
685	-0.03566587	0.03171160	-0.00735360
686	-0.03850607	0.03290508	-0.00743352

Procrustes sums of squares: 0.3657145180346275
Tangent sums of squares: 0.35793716368340484

Data matrices in this dataset:
- Evolution Cervidae final, raw data
- Sans Ind & Capreolus, centroid size
- Sans Ind & Capreolus, Procrustes coordinates

New Procrustes fit:
Dataset 'Sans Ind & Capreolus'

686 landmarks in 3 dimensions.
The dataset contains 40 observations, of which 36 are included for analyses.

Average shape:

Lmk.	Axis 1 (x)	Axis 2 (y)	Axis 3 (z)
1	-0.04036362	0.02592451	-0.00811150
2	-0.02486511	-0.00981714	-0.02627340
3	-0.02630368	-0.01137480	-0.02731431
4	-0.02818995	-0.01223046	-0.02847610
5	-0.03017741	-0.01259941	-0.02952329
6	-0.03220235	-0.01261672	-0.03047543
7	-0.03431632	-0.01234278	-0.03134544
8	-0.03656948	-0.01172608	-0.03215652
9	-0.03897484	-0.01071316	-0.03289171
10	-0.04144477	-0.00924722	-0.03353796

11	-0.04391420	-0.00725860	-0.03406058
12	-0.04660985	-0.00439912	-0.03449515
13	-0.04870323	-0.00138086	-0.03453497
14	-0.05020484	0.00168057	-0.03420577
15	-0.05127422	0.00468345	-0.03352928
16	-0.05194588	0.00763278	-0.03255234
17	-0.05218638	0.01059840	-0.03122656
18	-0.05198752	0.01357426	-0.02955756
19	-0.05126707	0.01655038	-0.02751849
20	-0.04987035	0.01940290	-0.02515643
21	-0.04786808	0.02164297	-0.02204558
22	-0.04544731	0.02307481	-0.01919084
23	-0.04287669	0.02382276	-0.01656435
24	-0.04021532	0.02392447	-0.01415795
25	-0.03749705	0.02347117	-0.01185990
26	-0.03451674	0.02226598	-0.00987187
27	-0.03131650	0.02036366	-0.00814552
28	-0.02795494	0.01785428	-0.00653419
29	-0.02446861	0.01459152	-0.00530442
30	-0.02274492	0.01293029	-0.00496445
31	-0.02130127	0.01127595	-0.00504291
32	-0.02018054	0.00965077	-0.00552535
33	-0.01937887	0.00805876	-0.00639792
34	-0.01887320	0.00649494	-0.00763445
35	-0.01863959	0.00493692	-0.00923185
36	-0.01861014	0.00336333	-0.01114849
37	-0.01881318	0.00170255	-0.01342681
38	-0.01950246	0.00028609	-0.01612496
39	-0.02051828	-0.00009089	-0.01770052
40	-0.02170450	-0.00033282	-0.01922434
41	-0.02269031	-0.00088248	-0.02062383
42	-0.02347663	-0.00171594	-0.02187741
43	-0.02404150	-0.00284644	-0.02298066
44	-0.02444990	-0.00424358	-0.02398036
45	-0.02463759	-0.00594249	-0.02481416
46	-0.02463999	-0.00790540	-0.02549130
47	-0.01971082	-0.01429760	-0.02654986
48	-0.02207603	-0.01533880	-0.02788349
49	-0.02452514	-0.01601771	-0.02907491
50	-0.02697985	-0.01635938	-0.03012478
51	-0.02938783	-0.01643944	-0.03108082
52	-0.03172828	-0.01627283	-0.03197942
53	-0.03405324	-0.01585177	-0.03278889
54	-0.03635762	-0.01517846	-0.03352073
55	-0.03864152	-0.01423175	-0.03417591
56	-0.04088868	-0.01300501	-0.03472953
57	-0.04298360	-0.01153854	-0.03527308
58	-0.04489822	-0.00994488	-0.03566984
59	-0.04665507	-0.00824501	-0.03596318
60	-0.04822977	-0.00641306	-0.03612856
61	-0.04969089	-0.00450700	-0.03623035
62	-0.05095120	-0.00244136	-0.03614721
63	-0.05204945	-0.00025523	-0.03596464
64	-0.05297543	0.00205276	-0.03560948
65	-0.05371134	0.00447999	-0.03512109
66	-0.05419957	0.00667575	-0.03445464
67	-0.05451852	0.00881152	-0.03371248
68	-0.05466005	0.01088919	-0.03286446
69	-0.05459579	0.01289528	-0.03188451
70	-0.05437111	0.01483793	-0.03081159

71	-0.05400202	0.01676676	-0.02966597
72	-0.05338614	0.01857604	-0.02833334
73	-0.05259955	0.02033943	-0.02692452
74	-0.05160894	0.02199484	-0.02538766
75	-0.05032088	0.02348538	-0.02386987
76	-0.04889424	0.02477045	-0.02222484
77	-0.04734848	0.02583566	-0.02045486
78	-0.04568356	0.02668656	-0.01854892
79	-0.04389958	0.02729224	-0.01651489
80	-0.04196674	0.02768918	-0.01436050
81	-0.03993372	0.02788108	-0.01206327
82	-0.03778278	0.02786411	-0.00962310
83	-0.03550002	0.02805675	-0.00713649
84	-0.02475516	0.00500360	0.02164341
85	-0.02663734	0.00345875	0.02300711
86	-0.02893429	0.00246871	0.02432777
87	-0.03125222	0.00179496	0.02541824
88	-0.03351854	0.00126713	0.02624892
89	-0.03576266	0.00091725	0.02689810
90	-0.03801045	0.00073757	0.02735358
91	-0.04026958	0.00071849	0.02765156
92	-0.04255395	0.00091244	0.02778557
93	-0.04484174	0.00133713	0.02770046
94	-0.04746711	0.00203698	0.02715615
95	-0.04978796	0.00300011	0.02632534
96	-0.05182017	0.00415271	0.02524752
97	-0.05352649	0.00550637	0.02392747
98	-0.05500355	0.00695445	0.02240050
99	-0.05624612	0.00856683	0.02065234
100	-0.05722574	0.01032876	0.01866341
101	-0.05794862	0.01226252	0.01643623
102	-0.05829858	0.01437348	0.01400590
103	-0.05820941	0.01695260	0.01097585
104	-0.05748421	0.01920360	0.00826470
105	-0.05622342	0.02109539	0.00589852
106	-0.05456535	0.02262365	0.00381355
107	-0.05245204	0.02378773	0.00203401
108	-0.04992802	0.02461653	0.00047559
109	-0.04691429	0.02511807	-0.00083014
110	-0.04338655	0.02524868	-0.00180378
111	-0.03939899	0.02500765	-0.00219625
112	-0.03571669	0.02334225	-0.00172767
113	-0.03242682	0.02173363	-0.00074659
114	-0.02951264	0.02020953	0.00028187
115	-0.02694310	0.01876207	0.00131585
116	-0.02471578	0.01734922	0.00240066
117	-0.02278449	0.01593180	0.00362812
118	-0.02116758	0.01449232	0.00516745
119	-0.02005743	0.01321894	0.00717437
120	-0.01982002	0.01265052	0.00928625
121	-0.02048050	0.01276028	0.01082980
122	-0.02140577	0.01295381	0.01235614
123	-0.02221766	0.01279049	0.01383239
124	-0.02290441	0.01229057	0.01525892
125	-0.02344077	0.01132780	0.01664227
126	-0.02388330	0.01016281	0.01795521
127	-0.02417959	0.00871239	0.01919274
128	-0.02428154	0.00676757	0.02037126
129	-0.01889190	0.00302862	0.02394466
130	-0.02110260	0.00214561	0.02522712

131	-0.02349788	0.00139899	0.02617422
132	-0.02586912	0.00076067	0.02701963
133	-0.02819728	0.00019760	0.02778945
134	-0.03049387	-0.00028099	0.02849185
135	-0.03278938	-0.00066872	0.02911548
136	-0.03508840	-0.00096192	0.02966257
137	-0.03739207	-0.00114914	0.03004513
138	-0.03968475	-0.00124508	0.03031424
139	-0.04199260	-0.00107408	0.03043188
140	-0.04419000	-0.00078425	0.03037969
141	-0.04626033	-0.00034011	0.03007118
142	-0.04822308	0.00018904	0.02966283
143	-0.05005434	0.00084689	0.02904747
144	-0.05177324	0.00165080	0.02827374
145	-0.05339725	0.00256569	0.02734367
146	-0.05488949	0.00361482	0.02622050
147	-0.05627996	0.00479674	0.02496187
148	-0.05780268	0.00638624	0.02325633
149	-0.05900770	0.00801934	0.02146877
150	-0.05993875	0.00967549	0.01961293
151	-0.06065928	0.01137019	0.01772820
152	-0.06111308	0.01310885	0.01578557
153	-0.06132017	0.01486941	0.01377255
154	-0.06123036	0.01664026	0.01167593
155	-0.06084204	0.01843626	0.00949995
156	-0.06010631	0.02023352	0.00726995
157	-0.05876156	0.02231800	0.00499395
158	-0.05702064	0.02406532	0.00291125
159	-0.05495415	0.02551484	0.00103142
160	-0.05263172	0.02673985	-0.00076846
161	-0.04991975	0.02759773	-0.00236395
162	-0.04685180	0.02816012	-0.00379531
163	-0.04340435	0.02836941	-0.00505424
164	-0.03957295	0.02841727	-0.00620094
165	-0.03550002	0.02805675	-0.00713649
166	-0.02499274	-0.01797812	-0.01144966
167	-0.02594114	-0.01926394	-0.01127705
168	-0.02715963	-0.02032120	-0.01063659
169	-0.02835612	-0.02123163	-0.00977690
170	-0.02953381	-0.02203539	-0.00869693
171	-0.03072663	-0.02273138	-0.00741166
172	-0.03191706	-0.02340512	-0.00589307
173	-0.03311180	-0.02396404	-0.00412300
174	-0.03431553	-0.02442274	-0.00211313
175	-0.03556056	-0.02468571	0.00012660
176	-0.03649211	-0.02473268	0.00178126
177	-0.03733966	-0.02465161	0.00340695
178	-0.03820179	-0.02302148	0.00481856
179	-0.03880320	-0.02403014	0.00653744
180	-0.03941172	-0.02351355	0.00803519
181	-0.03995039	-0.02286350	0.00951174
182	-0.04039727	-0.02207565	0.01095243
183	-0.04079079	-0.02116340	0.01237129
184	-0.04109684	-0.02011810	0.01374697
185	-0.04130890	-0.01876998	0.01527170
186	-0.04134585	-0.01740235	0.01661532
187	-0.04122223	-0.01601967	0.01784617
188	-0.04086897	-0.01457793	0.01871371
189	-0.04035602	-0.01312194	0.01951769
190	-0.03964819	-0.01161107	0.02003250

191	-0.03873596	-0.01005176	0.02032875
192	-0.03763316	-0.00846149	0.02042424
193	-0.03633865	-0.00682257	0.02029050
194	-0.03442252	-0.00477936	0.01989045
195	-0.03261396	-0.00294886	0.01921052
196	-0.03090104	-0.00137572	0.01823817
197	-0.02927633	-0.00002425	0.01701181
198	-0.02777946	0.00114102	0.01557634
199	-0.02638943	0.00209796	0.01386781
200	-0.02507999	0.00277584	0.01189396
201	-0.02383005	0.00335102	0.00971822
202	-0.02268630	0.00367457	0.00728109
203	-0.02198686	0.00350864	0.00526205
204	-0.02150943	0.00305574	0.00328699
205	-0.02109940	0.00246504	0.00146270
206	-0.02070629	0.00172063	-0.00021211
207	-0.02031526	0.00079952	-0.00175701
208	-0.02001998	-0.00032600	-0.00321626
209	-0.01977998	-0.00165156	-0.00460495
210	-0.01972435	-0.00318700	-0.00591164
211	-0.02012169	-0.00482420	-0.00683380
212	-0.02086359	-0.00632844	-0.00688795
213	-0.02179806	-0.00781622	-0.00684819
214	-0.02269357	-0.00929351	-0.00694264
215	-0.02340306	-0.01076302	-0.00724541
216	-0.02389958	-0.01220369	-0.00778537
217	-0.02423287	-0.01365489	-0.00854983
218	-0.02439367	-0.01512932	-0.00955153
219	-0.02446461	-0.01665275	-0.01071370
220	-0.02301658	-0.01983875	-0.01657561
221	-0.02449962	-0.02103703	-0.01580409
222	-0.02591209	-0.02210145	-0.01482174
223	-0.02725720	-0.02308326	-0.01371343
224	-0.02855787	-0.02398036	-0.01249724
225	-0.02979391	-0.02477324	-0.01114796
226	-0.03097478	-0.02545093	-0.00967183
227	-0.03210955	-0.02607016	-0.00809480
228	-0.03319008	-0.02659988	-0.00638774
229	-0.03426191	-0.02708908	-0.00460141
230	-0.03533030	-0.02742900	-0.00265247
231	-0.03638312	-0.02766692	-0.00077013
232	-0.03734717	-0.02770520	0.00110545
233	-0.03826857	-0.02760746	0.00292934
234	-0.03908166	-0.02730438	0.00475011
235	-0.03985656	-0.02685237	0.00652661
236	-0.04056376	-0.02621526	0.00828375
237	-0.04119816	-0.02541774	0.01001183
238	-0.04177051	-0.02445017	0.01171237
239	-0.04234573	-0.02326985	0.01357121
240	-0.04280543	-0.02204921	0.01527783
241	-0.04307339	-0.02075208	0.01680627
242	-0.04316928	-0.01938416	0.01818845
243	-0.04309833	-0.01796702	0.01938745
244	-0.04288469	-0.01648776	0.02045101
245	-0.04251190	-0.01496819	0.02134714
246	-0.04194658	-0.01335978	0.02208120
247	-0.04122537	-0.01171653	0.02264389
248	-0.04013103	-0.00977920	0.02317404
249	-0.03891342	-0.00789103	0.02339685
250	-0.03757997	-0.00603269	0.02333239

251	-0.03612316	-0.00421851	0.02298515
252	-0.03457502	-0.00244218	0.02236291
253	-0.03298520	-0.00059142	0.02156995
254	-0.03104639	0.00094796	0.02023291
255	-0.02905013	0.00254424	0.01870258
256	-0.02719004	0.00426947	0.01701131
257	-0.01420492	-0.00895315	0.00083756
258	-0.01362852	-0.00923638	0.00155711
259	-0.01293438	-0.00949925	0.00213629
260	-0.01213240	-0.00968167	0.00251142
261	-0.01129125	-0.00982879	0.00280732
262	-0.01045304	-0.01007004	0.00295865
263	-0.00950672	-0.01035670	0.00294027
264	-0.00854636	-0.01069007	0.00289594
265	-0.00740862	-0.01091971	0.00315727
266	-0.00644379	-0.01139151	0.00217907
267	-0.00519335	-0.01148261	0.00169384
268	-0.00408083	-0.01161007	0.00118547
269	-0.00317717	-0.01171258	0.00058195
270	-0.00244467	-0.01181043	-0.00011340
271	-0.00186863	-0.01198125	-0.00087273
272	-0.00139618	-0.01214156	-0.00173581
273	-0.00106708	-0.01221241	-0.00271789
274	-0.00097424	-0.01225575	-0.00380308
275	-0.00109211	-0.01226381	-0.00495813
276	-0.00181558	-0.01255486	-0.00585019
277	-0.00265436	-0.01267661	-0.00655561
278	-0.00352794	-0.01272289	-0.00709354
279	-0.00444229	-0.01278634	-0.00742448
280	-0.00539185	-0.01279159	-0.00757620
281	-0.00639964	-0.01275603	-0.00755145
282	-0.00743214	-0.01262899	-0.00732805
283	-0.00859772	-0.01256264	-0.00696748
284	-0.00973407	-0.01239425	-0.00642912
285	-0.01078608	-0.01202259	-0.00585542
286	-0.01173719	-0.01165365	-0.00519610
287	-0.01251652	-0.01124299	-0.00448940
288	-0.01318365	-0.01085834	-0.00376242
289	-0.01370455	-0.01048911	-0.00298549
290	-0.01408853	-0.01012381	-0.00212894
291	-0.01431493	-0.00976411	-0.00119754
292	-0.01435066	-0.00937240	-0.00019292
293	0.02587333	0.01926473	0.01617969
294	0.02816657	0.01805224	0.01850146
295	0.03003528	0.01703775	0.02018269
296	0.03267677	0.01459136	0.02173532
297	0.03362073	0.01306809	0.02168953
298	0.03446001	0.01126160	0.02116429
299	0.03496819	0.00901716	0.01983755
300	0.03707364	0.01699636	-0.01060991
301	0.03923838	0.01638373	-0.01093459
302	0.04097023	0.01570535	-0.01111726
303	0.04219610	0.01478278	-0.01105536
304	0.04350500	0.01254245	-0.01048141
305	0.04375876	0.01115959	-0.00997934
306	0.04362935	0.00951276	-0.00926276
307	0.02070917	0.00418236	-0.01852877
308	0.02123998	0.00252670	-0.01979348
309	0.02187883	0.00107518	-0.02051511
310	0.02256028	-0.00014793	-0.02081252

311	0.02411657	-0.00211887	-0.02029710
312	0.02503308	-0.00299290	-0.01955280
313	0.02608090	-0.00378639	-0.01840111
314	0.03185011	0.00463990	0.01061681
315	0.03345782	0.00346569	0.01181123
316	0.03461084	0.00242664	0.01257250
317	0.03548554	0.00143281	0.01291878
318	0.03657419	-0.00059275	0.01250653
319	0.03685028	-0.00172227	0.01179215
320	0.03695813	-0.00299166	0.01069417
321	0.03846695	0.00676464	-0.00647510
322	0.04109393	0.00486686	-0.00675629
323	0.04276408	0.00408199	-0.00718175
324	0.04411850	0.00221221	-0.00702164
325	0.04422334	0.00123872	-0.00674590
326	0.04409352	0.00014020	-0.00634048
327	0.04365107	-0.00119541	-0.00575169
328	0.02812671	-0.00288162	-0.01147439
329	0.02834146	-0.00458265	-0.01324353
330	0.02884814	-0.00600743	-0.01412604
331	0.02997272	-0.00791211	-0.01445270
332	0.03060502	-0.00858125	-0.01405170
333	0.03135400	-0.00917384	-0.01333860
334	0.03225444	-0.00968688	-0.01218141
335	-0.01172028	-0.00127986	0.01812014
336	-0.00981966	-0.00017310	0.01596653
337	-0.00748292	0.00135292	0.01318677
338	-0.00467901	0.00176626	0.01257339
339	-0.00160492	0.00166516	0.01345472
340	0.00073520	0.00261184	0.01288804
341	0.00309907	0.00379113	0.01238177
342	0.00544885	0.00525394	0.01189193
343	0.00775190	0.00691334	0.01155331
344	0.01000933	0.00859471	0.01167153
345	0.01159026	0.00924354	0.01172904
346	0.01313523	0.00992465	0.01169330
347	0.01462754	0.01061938	0.01153682
348	0.01607257	0.01123832	0.01130270
349	0.01746758	0.01184949	0.01094656
350	0.01883896	0.01248093	0.01049223
351	0.02019423	0.01308261	0.00995225
352	0.02149643	0.01371791	0.00923701
353	0.02272847	0.01433868	0.00841663
354	0.02481547	0.01491058	0.00685853
355	0.02656942	0.01530765	0.00518982
356	0.02808847	0.01552710	0.00350573
357	0.02930815	0.01554265	0.00175550
358	0.03028629	0.01537772	-0.00000438
359	0.03107232	0.01504573	-0.00178924
360	0.03160693	0.01450721	-0.00360661
361	0.03177200	0.01365817	-0.00540187
362	0.03160557	0.01293630	-0.00744600
363	0.03106170	0.01175631	-0.00900205
364	0.03038092	0.01111919	-0.01031657
365	0.02952562	0.01019383	-0.01133570
366	0.02853786	0.00936300	-0.01204035
367	0.02748432	0.00852688	-0.01249330
368	0.02632847	0.00767429	-0.01272849
369	0.02507221	0.00679246	-0.01271952
370	0.02370023	0.00589785	-0.01246631

371	0.02219163	0.00511297	-0.01183295
372	0.02060253	0.00332031	-0.00966574
373	0.01936455	0.00241102	-0.00757266
374	0.01871548	0.00159697	-0.00553696
375	0.01852484	0.00115230	-0.00365728
376	0.01884631	0.00065988	-0.00185211
377	0.01974608	0.00010857	-0.00008481
378	0.02094316	-0.00003790	0.00176350
379	0.02291868	0.00010279	0.00337593
380	0.02528540	0.00115883	0.00421022
381	0.02774686	0.00133632	0.00399910
382	0.02960979	0.00217368	0.00353866
383	0.03088601	0.00273921	0.00281149
384	0.03169197	0.00299553	0.00187416
385	0.03231657	0.00294339	0.00088867
386	0.03279191	0.00259206	-0.00014395
387	0.03321028	0.00185201	-0.00142195
388	0.03280723	0.00113424	-0.00303939
389	0.03138479	0.00071611	-0.00423056
390	0.03074004	-0.00050220	-0.00429013
391	0.02978969	-0.00125837	-0.00387234
392	0.02934388	-0.00169521	-0.00329803
393	0.02954951	-0.00213744	-0.00293272
394	0.03011139	-0.00272617	-0.00249838
395	0.03123281	-0.00373416	-0.00218899
396	0.03270543	-0.00474862	-0.00183261
397	0.03581692	-0.00584704	-0.00153825
398	0.00175150	0.01384106	0.01986431
399	0.00395592	0.01323955	0.01977547
400	0.00636728	0.01320706	0.01958638
401	0.00877127	0.01345148	0.01923816
402	0.01114184	0.01399185	0.01872838
403	0.01349436	0.01464576	0.01796054
404	0.01589219	0.01557216	0.01697272
405	0.01835448	0.01668598	0.01584368
406	0.02091562	0.01787523	0.01456537
407	0.02362854	0.01904545	0.01310844
408	0.02615073	0.01994385	0.01081755
409	0.02836713	0.02052226	0.00846297
410	0.03029835	0.02075309	0.00605305
411	0.03188309	0.02071802	0.00358688
412	0.03312215	0.02044034	0.00105844
413	0.03402449	0.01992269	-0.00155966
414	0.03459416	0.01916495	-0.00424869
415	0.03481121	0.01814049	-0.00701032
416	0.03466972	0.01691722	-0.00986995
417	0.03359050	0.01554510	-0.01201557
418	0.03238080	0.01422233	-0.01382808
419	0.03102669	0.01296553	-0.01530783
420	0.02957444	0.01170741	-0.01642472
421	0.02800219	0.01049636	-0.01719415
422	0.02631005	0.00928329	-0.01761252
423	0.02448131	0.00808574	-0.01767455
424	0.02254429	0.00687127	-0.01737555
425	0.02058327	0.00558651	-0.01669829
426	0.01785690	0.00399632	-0.01437669
427	0.01585703	0.00261997	-0.01195234
428	0.01453051	0.00146287	-0.00946739
429	0.01384650	0.00056285	-0.00691465
430	0.01375780	-0.00001375	-0.00427298

431	0.01440182	-0.00041373	-0.00156825
432	0.01576558	-0.00059723	0.00122092
433	0.01780654	-0.00054486	0.00410058
434	0.02045436	-0.00015461	0.00707657
435	0.02296861	0.00095982	0.00803691
436	0.02522707	0.00206162	0.00864778
437	0.02727785	0.00306918	0.00889625
438	0.02911712	0.00395986	0.00878205
439	0.03075297	0.00470902	0.00828189
440	0.03219306	0.00534578	0.00740889
441	0.03341590	0.00592685	0.00616181
442	0.03451745	0.00625965	0.00452302
443	0.03543575	0.00641256	0.00252476
444	0.03585532	0.00629171	0.00071914
445	0.03609805	0.00604791	-0.00094565
446	0.03618289	0.00573616	-0.00249520
447	0.03608863	0.00534430	-0.00390653
448	0.03584725	0.00484086	-0.00518997
449	0.03540758	0.00424762	-0.00634779
450	0.03479334	0.00358544	-0.00738212
451	0.03401438	0.00281988	-0.00829128
452	0.03307100	0.00195507	-0.00905581
453	0.03197989	0.00104425	-0.00949076
454	0.03092847	0.00026206	-0.00970118
455	0.02998050	-0.00049538	-0.00966793
456	0.02907338	-0.00114972	-0.00940987
457	0.02824415	-0.00176644	-0.00889215
458	0.02748598	-0.00234658	-0.00810671
459	0.02687795	-0.00295596	-0.00702761
460	0.02643400	-0.00358347	-0.00567919
461	0.02622359	-0.00427971	-0.00412512
462	0.02697931	-0.00414388	-0.00228177
463	0.02783022	-0.00416996	-0.00083607
464	0.02872124	-0.00424154	0.00027241
465	0.02970001	-0.00433671	0.00101924
466	0.03066197	-0.00426093	0.00164923
467	0.03189605	-0.00440755	0.00162636
468	0.03374501	-0.00469686	0.00105143
469	0.03562753	-0.00567469	-0.00040085
470	-0.01405999	-0.00448353	0.01199846
471	-0.01327624	-0.00615359	0.01155577
472	-0.01218013	-0.00765257	0.01108889
473	-0.01089008	-0.00894656	0.01074745
474	-0.00952228	-0.01014643	0.01065119
475	-0.00802525	-0.01122904	0.01074947
476	-0.00648290	-0.01228996	0.01106501
477	-0.00526464	-0.01342736	0.01160294
478	-0.00284469	-0.01445149	0.01218824
479	-0.00235071	-0.01505638	0.01362634
480	-0.00057922	-0.01551150	0.01468553
481	0.00112223	-0.01579763	0.01578951
482	0.00277592	-0.01587528	0.01692713
483	0.00435767	-0.01575872	0.01807645
484	0.00588584	-0.01544568	0.01926245
485	0.00736047	-0.01496314	0.02049256
486	0.00878267	-0.01424004	0.02174220
487	0.01016040	-0.01325788	0.02300620
488	0.01150151	-0.01205326	0.02424520
489	0.01272474	-0.01063365	0.02541270
490	0.01394615	-0.00912507	0.02639944

491	0.01517522	-0.00756413	0.02728012
492	0.01643602	-0.00591284	0.02795602
493	0.01767138	-0.00416505	0.02847710
494	0.01893487	-0.00230851	0.02881471
495	0.02021383	-0.00038022	0.02899355
496	0.02150517	0.00165100	0.02897907
497	0.02279615	0.00377396	0.02871005
498	0.02479946	0.00683549	0.02788249
499	0.02678725	0.00955162	0.02661579
500	0.02876705	0.01190745	0.02497364
501	0.03075035	0.01392402	0.02302300
502	0.03276864	0.01564003	0.02077317
503	0.03480122	0.01701378	0.01814617
504	0.03685017	0.01804085	0.01513511
505	0.03885990	0.01867420	0.01169836
506	0.04071137	0.01880678	0.00783131
507	0.04234804	0.01842942	0.00319138
508	0.04319392	0.01758835	-0.00120791
509	0.04329672	0.01643156	-0.00516137
510	0.04283799	0.01510102	-0.00863886
511	0.04191714	0.01363323	-0.01167814
512	0.04058038	0.01206258	-0.01437005
513	0.03881765	0.01036889	-0.01677530
514	0.03653839	0.00849577	-0.01883607
515	0.03369222	0.00641862	-0.02043009
516	0.03121893	0.00480826	-0.02122917
517	0.02881560	0.00326752	-0.02153818
518	0.02653170	0.00180376	-0.02142990
519	0.02438529	0.00042885	-0.02093315
520	0.02235112	-0.00087884	-0.02012587
521	0.02043778	-0.00211728	-0.01893960
522	0.01862180	-0.00329883	-0.01740436
523	0.01691967	-0.00439601	-0.01547014
524	0.01539708	-0.00537614	-0.01311932
525	0.01436649	-0.00635815	-0.01077700
526	0.01369976	-0.00717745	-0.00841675
527	0.01337480	-0.00782977	-0.00607145
528	0.01334378	-0.00834772	-0.00375622
529	0.01362223	-0.00870699	-0.00147973
530	0.01416921	-0.00895092	0.00078057
531	0.01500797	-0.00907364	0.00304707
532	0.01619837	-0.00902670	0.00527835
533	0.01774937	-0.00877777	0.00743715
534	0.01943712	-0.00824749	0.00936803
535	0.02124334	-0.00752661	0.01093038
536	0.02308198	-0.00671600	0.01217647
537	0.02497056	-0.00579067	0.01311554
538	0.02693106	-0.00470307	0.01367410
539	0.02893472	-0.00360428	0.01407307
540	0.03101090	-0.00229582	0.01403004
541	0.03316389	-0.00088888	0.01367675
542	0.03533969	0.00060278	0.01283318
543	0.03767007	0.00181293	0.01145107
544	0.03961497	0.00276158	0.00973062
545	0.04116412	0.00344369	0.00784756
546	0.04240633	0.00390778	0.00583676
547	0.04334771	0.00412907	0.00370463
548	0.04403160	0.00415911	0.00139968
549	0.04438067	0.00396289	-0.00110620
550	0.04430687	0.00347775	-0.00380985

551	0.04372093	0.00269044	-0.00660298
552	0.04285048	0.00178208	-0.00882628
553	0.04174237	0.00079350	-0.01068087
554	0.04049535	-0.00023208	-0.01218732
555	0.03909850	-0.00130016	-0.01334704
556	0.03759087	-0.00240096	-0.01421729
557	0.03592539	-0.00354340	-0.01477669
558	0.03411289	-0.00474780	-0.01505795
559	0.03211970	-0.00599507	-0.01494660
560	0.03003332	-0.00725745	-0.01433042
561	0.02818582	-0.00857227	-0.01319741
562	0.02673746	-0.00964840	-0.01175889
563	0.02568626	-0.01052689	-0.01021597
564	0.02497676	-0.01122878	-0.00860865
565	0.02455074	-0.01182021	-0.00692180
566	0.02440537	-0.01230005	-0.00512194
567	0.02459443	-0.01265729	-0.00317997
568	0.02522170	-0.01269135	-0.00109534
569	0.02616218	-0.01232520	0.00112710
570	0.02799792	-0.01164154	0.00307416
571	0.02985354	-0.01086215	0.00428979
572	0.03149247	-0.01011763	0.00487646
573	0.03286284	-0.00952532	0.00507522
574	0.03415731	-0.00902859	0.00498691
575	0.03571407	-0.00844504	0.00434290
576	0.03719703	-0.00815875	0.00316565
577	0.03796199	-0.00783864	0.00091595
578	-0.00472278	-0.00591580	-0.01389955
579	-0.00282152	-0.00534411	-0.01307654
580	-0.00077831	-0.00526381	-0.01217037
581	0.00097025	-0.00534827	-0.01103326
582	0.00242991	-0.00558909	-0.00963486
583	0.00351485	-0.00586547	-0.00795511
584	0.00433010	-0.00629561	-0.00603422
585	0.00486325	-0.00686536	-0.00381865
586	0.00519077	-0.00760052	-0.00137754
587	0.00556083	-0.00865338	0.00112401
588	0.00646828	-0.01013170	0.00343617
589	0.00783613	-0.01149684	0.00551644
590	0.00930897	-0.01254350	0.00744219
591	0.01084713	-0.01319676	0.00926903
592	0.01241892	-0.01342319	0.01104797
593	0.01408775	-0.01328703	0.01276525
594	0.01583373	-0.01277122	0.01443718
595	0.01763102	-0.01183438	0.01607692
596	0.01947566	-0.01048279	0.01764952
597	0.02140151	-0.00861850	0.01893748
598	0.02317182	-0.00663277	0.01995184
599	0.02480619	-0.00457297	0.02065572
600	0.02639007	-0.00251694	0.02101676
601	0.02795184	-0.00048046	0.02101025
602	0.02950971	0.00154632	0.02063967
603	0.03113609	0.00352040	0.01987287
604	0.03288489	0.00539669	0.01874626
605	0.03474481	0.00711491	0.01730788
606	0.03686200	0.00862248	0.01474500
607	0.03872174	0.00971562	0.01211694
608	0.04029211	0.01043822	0.00941802
609	0.04158210	0.01077047	0.00664789
610	0.04252638	0.01077335	0.00380530

611	0.04314034	0.01041931	0.00085880
612	0.04337970	0.00977831	-0.00218957
613	0.04322285	0.00884295	-0.00533685
614	0.04260484	0.00769363	-0.00858368
615	0.04161695	0.00644682	-0.01099706
616	0.04035395	0.00523362	-0.01302552
617	0.03893777	0.00397600	-0.01467091
618	0.03733841	0.00270040	-0.01593965
619	0.03557586	0.00137918	-0.01681177
620	0.03364114	0.00002475	-0.01730930
621	0.03156233	-0.00139408	-0.01743314
622	0.02931854	-0.00288202	-0.01718814
623	0.02690239	-0.00442226	-0.01654399
624	0.02483326	-0.00585611	-0.01531786
625	0.02310872	-0.00708084	-0.01384842
626	0.02174057	-0.00812270	-0.01217845
627	0.02073371	-0.00900456	-0.01031061
628	0.02008726	-0.00970652	-0.00824582
629	0.01984304	-0.01036388	-0.00598328
630	0.01997215	-0.01087333	-0.00350758
631	0.02047542	-0.01124309	-0.00083346
632	0.02133162	-0.01140695	0.00203385
633	0.02270881	-0.01122814	0.00426263
634	0.02414832	-0.01080825	0.00614223
635	0.02564284	-0.01017136	0.00763857
636	0.02724265	-0.00941665	0.00876399
637	0.02893980	-0.00857386	0.00952246
638	0.03074246	-0.00759938	0.00990687
639	0.03266598	-0.00653779	0.00993561
640	0.03473008	-0.00542265	0.00962073
641	0.03688195	-0.00415521	0.00892264
642	0.03844788	-0.00340503	0.00785551
643	0.03978015	-0.00279195	0.00666462
644	0.04087124	-0.00232931	0.00534496
645	0.04173090	-0.00202117	0.00390837
646	0.04236048	-0.00186649	0.00235601
647	0.04276548	-0.00186371	0.00067766
648	0.04297459	-0.00204918	-0.00111624
649	0.04290986	-0.00235173	-0.00304896
650	0.04257675	-0.00277653	-0.00509931
651	0.04199268	-0.00337785	-0.00662625
652	0.04128121	-0.00403213	-0.00792095
653	0.04042464	-0.00471125	-0.00897988
654	0.03946348	-0.00545763	-0.00981281
655	0.03840840	-0.00624549	-0.01039310
656	0.03724789	-0.00710525	-0.01074771
657	0.03598539	-0.00803769	-0.01087718
658	0.03462609	-0.00903747	-0.01077708
659	0.03312538	-0.01004584	-0.01042879
660	0.03197561	-0.01098321	-0.00948970
661	0.03103462	-0.01173975	-0.00848024
662	0.03033323	-0.01236701	-0.00741586
663	0.02986373	-0.01285649	-0.00629548
664	0.02963806	-0.01321170	-0.00513194
665	0.02963912	-0.01344038	-0.00390085
666	0.02985399	-0.01349280	-0.00262084
667	0.03031734	-0.01340983	-0.00128204
668	0.03100931	-0.01318364	0.00011827
669	0.03213870	-0.01275362	0.00090662
670	0.03314645	-0.01225541	0.00146206

671	0.03410651	-0.01180367	0.00179509
672	0.03498578	-0.01133088	0.00191744
673	0.03582410	-0.01084690	0.00182995
674	0.03664555	-0.01030458	0.00150342
675	0.03744968	-0.00965418	0.00083948
676	0.03793364	-0.00861659	-0.00017299
677	-0.00929247	0.01399275	-0.00004503
678	-0.01283930	0.01696123	-0.00155635
679	-0.01665438	0.01954362	-0.00296851
680	-0.02025991	0.02198157	-0.00416642
681	-0.02350131	0.02441735	-0.00522117
682	-0.02664614	0.02660005	-0.00607178
683	-0.02988727	0.02846567	-0.00678561
684	-0.03277505	0.03017638	-0.00712933
685	-0.03566587	0.03171160	-0.00735360
686	-0.03850607	0.03290508	-0.00743352

Procrustes sums of squares: 0.3657145180346275
Tangent sums of squares: 0.35793716368340484

Data matrices in this dataset:
- Evolution Cervidae final, raw data
- Sans Ind & Capreolus, centroid size
- Sans Ind & Capreolus, Procrustes coordinates

New Procrustes fit:
Dataset 'lateral canal'

54 landmarks in 3 dimensions.
The dataset contains 40 observations, of which 36 are included for analyses.

Average shape:

Lmk.	Axis 1 (x)	Axis 2 (y)	Axis 3 (z)
1	0.12523216	-0.07289714	-0.00344158
2	0.11875481	-0.08457362	-0.00282463
3	0.10807652	-0.09405026	-0.00201661
4	0.09612589	-0.10176273	-0.00133525
5	0.08284101	-0.10805890	-0.00056638
6	0.06821423	-0.11301506	-0.00019943
7	0.05202810	-0.11714825	-0.00019262
8	0.03415503	-0.11977938	-0.00036286
9	0.01472171	-0.12102257	-0.00085061
10	-0.00629998	-0.12031180	-0.00118113
11	-0.02190505	-0.11886587	-0.00106239
12	-0.03686247	-0.11632333	-0.00078393
13	-0.04897345	-0.10478462	0.00560376
14	-0.06453091	-0.10751410	0.00006947
15	-0.07717765	-0.10143758	0.00068005
16	-0.08924490	-0.09425647	0.00136385
17	-0.10054512	-0.08592306	0.00206978
18	-0.11136395	-0.07658311	0.00287333
19	-0.12138368	-0.06617147	0.00381779
20	-0.13163110	-0.05267682	0.00396572
21	-0.13984058	-0.03905284	0.00397796
22	-0.14651389	-0.02507348	0.00378685
23	-0.14965942	-0.01118081	0.00332988

24	-0.15160798	0.00320408	0.00250129
25	-0.15063826	0.01769100	0.00155424
26	-0.14722767	0.03248167	0.00039669
27	-0.14153365	0.04748342	-0.00097283
28	-0.13337846	0.06271266	-0.00261867
29	-0.12032582	0.08177373	-0.00495400
30	-0.10601729	0.09819660	-0.00663828
31	-0.09036216	0.11169144	-0.00767088
32	-0.07362148	0.12263025	-0.00781780
33	-0.05607168	0.13123427	-0.00732766
34	-0.03733087	0.13723935	-0.00592807
35	-0.01745146	0.14024661	-0.00377172
36	0.00346875	0.14177224	-0.00096141
37	0.02540325	0.14040464	0.00252327
38	0.04216580	0.13574670	0.00424557
39	0.05740850	0.12854878	0.00574023
40	0.07119390	0.12052331	0.00674945
41	0.08373224	0.11173113	0.00715818
42	0.09525607	0.10200100	0.00683460
43	0.10562677	0.09073279	0.00635618
44	0.11509981	0.07807053	0.00551322
45	0.12303593	0.06362536	0.00413997
46	0.12632918	0.04818415	0.00230948
47	0.12207989	0.03502944	-0.00017106
48	0.11637506	0.02176139	-0.00151267
49	0.11179149	0.00835670	-0.00243193
50	0.10946608	-0.00507356	-0.00312384
51	0.10975965	-0.01831741	-0.00382384
52	0.11230151	-0.03178830	-0.00418372
53	0.11723132	-0.04556871	-0.00439186
54	0.12362430	-0.05986198	-0.00444316

Procrustes sums of squares: 0.5818490465667662
Tangent sums of squares: 0.5561001658390572

Data matrices in this dataset:
- lateral canal, raw data
- lateral canal, centroid size
- lateral canal, Procrustes coordinates

New Procrustes fit:
Dataset 'lateral canal'

54 landmarks in 3 dimensions.
The dataset contains 40 observations, of which 36 are included for analyses.

Average shape:

Lmk.	Axis 1 (x)	Axis 2 (y)	Axis 3 (z)
1	0.12523216	-0.07289714	-0.00344158
2	0.11875481	-0.08457362	-0.00282463
3	0.10807652	-0.09405026	-0.00201661
4	0.09612589	-0.10176273	-0.00133525
5	0.08284101	-0.10805890	-0.00056638
6	0.06821423	-0.11301506	-0.00019943
7	0.05202810	-0.11714825	-0.00019262
8	0.03415503	-0.11977938	-0.00036286

9	0.01472171	-0.12102257	-0.00085061
10	-0.00629998	-0.12031180	-0.00118113
11	-0.02190505	-0.11886587	-0.00106239
12	-0.03686247	-0.11632333	-0.00078393
13	-0.04897345	-0.10478462	0.00560376
14	-0.06453091	-0.10751410	0.00006947
15	-0.07717765	-0.10143758	0.00068005
16	-0.08924490	-0.09425647	0.00136385
17	-0.10054512	-0.08592306	0.00206978
18	-0.11136395	-0.07658311	0.00287333
19	-0.12138368	-0.06617147	0.00381779
20	-0.13163110	-0.05267682	0.00396572
21	-0.13984058	-0.03905284	0.00397796
22	-0.14651389	-0.02507348	0.00378685
23	-0.14965942	-0.01118081	0.00332988
24	-0.15160798	0.00320408	0.00250129
25	-0.15063826	0.01769100	0.00155424
26	-0.14722767	0.03248167	0.00039669
27	-0.14153365	0.04748342	-0.00097283
28	-0.13337846	0.06271266	-0.00261867
29	-0.12032582	0.08177373	-0.00495400
30	-0.10601729	0.09819660	-0.00663828
31	-0.09036216	0.11169144	-0.00767088
32	-0.07362148	0.12263025	-0.00781780
33	-0.05607168	0.13123427	-0.00732766
34	-0.03733087	0.13723935	-0.00592807
35	-0.01745146	0.14024661	-0.00377172
36	0.00346875	0.14177224	-0.00096141
37	0.02540325	0.14040464	0.00252327
38	0.04216580	0.13574670	0.00424557
39	0.05740850	0.12854878	0.00574023
40	0.07119390	0.12052331	0.00674945
41	0.08373224	0.11173113	0.00715818
42	0.09525607	0.10200100	0.00683460
43	0.10562677	0.09073279	0.00635618
44	0.11509981	0.07807053	0.00551322
45	0.12303593	0.06362536	0.00413997
46	0.12632918	0.04818415	0.00230948
47	0.12207989	0.03502944	-0.00017106
48	0.11637506	0.02176139	-0.00151267
49	0.11179149	0.00835670	-0.00243193
50	0.10946608	-0.00507356	-0.00312384
51	0.10975965	-0.01831741	-0.00382384
52	0.11230151	-0.03178830	-0.00418372
53	0.11723132	-0.04556871	-0.00439186
54	0.12362430	-0.05986198	-0.00444316

Procrustes sums of squares: 0.5818490465667662
Tangent sums of squares: 0.5561001658390572

Data matrices in this dataset:
- lateral canal, raw data
- lateral canal, centroid size
- lateral canal, Procrustes coordinates

New Procrustes fit:
Dataset 'lateral canal'

54 landmarks in 3 dimensions.
The dataset contains 40 observations, of which 36 are included for analyses.

Average shape:

Lmk.	Axis 1 (x)	Axis 2 (y)	Axis 3 (z)
1	0.12523216	-0.07289714	-0.00344158
2	0.11875481	-0.08457362	-0.00282463
3	0.10807652	-0.09405026	-0.00201661
4	0.09612589	-0.10176273	-0.00133525
5	0.08284101	-0.10805890	-0.00056638
6	0.06821423	-0.11301506	-0.00019943
7	0.05202810	-0.11714825	-0.00019262
8	0.03415503	-0.11977938	-0.00036286
9	0.01472171	-0.12102257	-0.00085061
10	-0.00629998	-0.12031180	-0.00118113
11	-0.02190505	-0.11886587	-0.00106239
12	-0.03686247	-0.11632333	-0.00078393
13	-0.04897345	-0.10478462	0.00560376
14	-0.06453091	-0.10751410	0.00006947
15	-0.07717765	-0.10143758	0.00068005
16	-0.08924490	-0.09425647	0.00136385
17	-0.10054512	-0.08592306	0.00206978
18	-0.11136395	-0.07658311	0.00287333
19	-0.12138368	-0.06617147	0.00381779
20	-0.13163110	-0.05267682	0.00396572
21	-0.13984058	-0.03905284	0.00397796
22	-0.14651389	-0.02507348	0.00378685
23	-0.14965942	-0.01118081	0.00332988
24	-0.15160798	0.00320408	0.00250129
25	-0.15063826	0.01769100	0.00155424
26	-0.14722767	0.03248167	0.00039669
27	-0.14153365	0.04748342	-0.00097283
28	-0.13337846	0.06271266	-0.00261867
29	-0.12032582	0.08177373	-0.00495400
30	-0.10601729	0.09819660	-0.00663828
31	-0.09036216	0.11169144	-0.00767088
32	-0.07362148	0.12263025	-0.00781780
33	-0.05607168	0.13123427	-0.00732766
34	-0.03733087	0.13723935	-0.00592807
35	-0.01745146	0.14024661	-0.00377172
36	0.00346875	0.14177224	-0.00096141
37	0.02540325	0.14040464	0.00252327
38	0.04216580	0.13574670	0.00424557
39	0.05740850	0.12854878	0.00574023
40	0.07119390	0.12052331	0.00674945
41	0.08373224	0.11173113	0.00715818
42	0.09525607	0.10200100	0.00683460
43	0.10562677	0.09073279	0.00635618
44	0.11509981	0.07807053	0.00551322
45	0.12303593	0.06362536	0.00413997
46	0.12632918	0.04818415	0.00230948
47	0.12207989	0.03502944	-0.00017106
48	0.11637506	0.02176139	-0.00151267
49	0.11179149	0.00835670	-0.00243193
50	0.10946608	-0.00507356	-0.00312384
51	0.10975965	-0.01831741	-0.00382384
52	0.11230151	-0.03178830	-0.00418372
53	0.11723132	-0.04556871	-0.00439186

54	0.12362430	-0.05986198	-0.00444316

Procrustes sums of squares: 0.5818490465667662
Tangent sums of squares: 0.5561001658390572

Data matrices in this dataset:
- lateral canal, raw data
- lateral canal, centroid size
- lateral canal, Procrustes coordinates

New Procrustes fit:
Dataset 'lateral canal'

54 landmarks in 3 dimensions.
The dataset contains 40 observations, of which 36 are included for analyses.

Average shape:

Lmk.	Axis 1 (x)	Axis 2 (y)	Axis 3 (z)
1	0.12523216	-0.07289714	-0.00344158
2	0.11875481	-0.08457362	-0.00282463
3	0.10807652	-0.09405026	-0.00201661
4	0.09612589	-0.10176273	-0.00133525
5	0.08284101	-0.10805890	-0.00056638
6	0.06821423	-0.11301506	-0.00019943
7	0.05202810	-0.11714825	-0.00019262
8	0.03415503	-0.11977938	-0.00036286
9	0.01472171	-0.12102257	-0.00085061
10	-0.00629998	-0.12031180	-0.00118113
11	-0.02190505	-0.11886587	-0.00106239
12	-0.03686247	-0.11632333	-0.00078393
13	-0.04897345	-0.10478462	0.00560376
14	-0.06453091	-0.10751410	0.00006947
15	-0.07717765	-0.10143758	0.00068005
16	-0.08924490	-0.09425647	0.00136385
17	-0.10054512	-0.08592306	0.00206978
18	-0.11136395	-0.07658311	0.00287333
19	-0.12138368	-0.06617147	0.00381779
20	-0.13163110	-0.05267682	0.00396572
21	-0.13984058	-0.03905284	0.00397796
22	-0.14651389	-0.02507348	0.00378685
23	-0.14965942	-0.01118081	0.00332988
24	-0.15160798	0.00320408	0.00250129
25	-0.15063826	0.01769100	0.00155424
26	-0.14722767	0.03248167	0.00039669
27	-0.14153365	0.04748342	-0.00097283
28	-0.13337846	0.06271266	-0.00261867
29	-0.12032582	0.08177373	-0.00495400
30	-0.10601729	0.09819660	-0.00663828
31	-0.09036216	0.11169144	-0.00767088
32	-0.07362148	0.12263025	-0.00781780
33	-0.05607168	0.13123427	-0.00732766
34	-0.03733087	0.13723935	-0.00592807
35	-0.01745146	0.14024661	-0.00377172
36	0.00346875	0.14177224	-0.00096141
37	0.02540325	0.14040464	0.00252327
38	0.04216580	0.13574670	0.00424557

39	0.05740850	0.12854878	0.00574023
40	0.07119390	0.12052331	0.00674945
41	0.08373224	0.11173113	0.00715818
42	0.09525607	0.10200100	0.00683460
43	0.10562677	0.09073279	0.00635618
44	0.11509981	0.07807053	0.00551322
45	0.12303593	0.06362536	0.00413997
46	0.12632918	0.04818415	0.00230948
47	0.12207989	0.03502944	-0.00017106
48	0.11637506	0.02176139	-0.00151267
49	0.11179149	0.00835670	-0.00243193
50	0.10946608	-0.00507356	-0.00312384
51	0.10975965	-0.01831741	-0.00382384
52	0.11230151	-0.03178830	-0.00418372
53	0.11723132	-0.04556871	-0.00439186
54	0.12362430	-0.05986198	-0.00444316

Procrustes sums of squares: 0.5818490465667662
Tangent sums of squares: 0.5561001658390572

Data matrices in this dataset:
- lateral canal, raw data
- lateral canal, centroid size
- lateral canal, Procrustes coordinates

Canonical Variate Analysis: CVA ...

Dataset: lateral canal
Classification criterion: subfam

Groups		Observations
1.	Ca	9
2.	Ce	13
3.	Di	3
4.	Pr	11

Variation among groups, scaled by the inverse of the within-group variation

	Eigenvalues	% Variance	Cumulative %
1.	7.78804006	58.420	58.420
2.	2.87510233	21.567	79.987
3.	2.66802268	20.013	100.000

Mahalanobis distances among groups:

	Ca	Ce	Di
Ce	4.1866		
Di	6.5054	6.1571	
Pr	5.5563	6.1773	8.1136

P-values from permutation tests (10000 permutation rounds) for Mahalanobis distances among groups:

	Ca	Ce	Di
Ce	<.0001		
Di	0.0028	0.0014	

Pr	<.0001	<.0001	0.0007

Procrustes distances among groups:

	Ca	Ce	Di
Ce	0.0601		
Di	0.0829	0.0690	
Pr	0.0625	0.0809	0.0691

P-values from permutation tests (10000 permutation rounds) for Procrustes distances among groups:

	Ca	Ce	Di
Ce	0.2738		
Di	0.3301	0.4749	
Pr	0.2137	0.0178	0.5035

Canonical coefficients:

	CV1	CV2	CV3
x1	-53.3195	-5.6963	36.5897
y1	54.3800	6.4582	-2.1396
z1	-84.3341	5.9417	-22.3517
x2	10.2629	45.2789	36.7998
y2	-42.7832	-23.4099	-11.8187
z2	29.0031	54.5507	-25.9310
x3	32.5652	-19.1743	52.1469
y3	-44.2823	64.6927	-24.8750
z3	-3.7654	12.5127	-16.7129
x4	24.0169	-13.0371	17.6659
y4	-45.8481	85.1864	-16.3046
z4	1.6990	44.9852	8.0472
x5	0.8675	20.3698	-22.7296
y5	7.2111	18.2664	23.6530
z5	-23.6818	46.2541	1.2041
x6	-8.6849	15.9725	-35.3247
y6	-0.1254	3.7815	56.7756
z6	-22.3343	32.0023	21.2817
x7	-3.0803	9.5775	-29.1716
y7	0.5180	46.2396	33.4086
z7	7.4888	22.9459	15.3239
x8	-4.4137	11.5042	-24.3092
y8	-2.0421	22.6754	31.8071
z8	14.3036	10.7731	1.4931
x9	0.1927	-5.6959	-11.1777
y9	80.9421	-34.9229	-17.4514
z9	57.9396	-55.5752	-0.5595
x10	-0.2439	-38.3173	7.1413
y10	17.9849	3.1107	-17.8174
z10	52.0804	-53.5528	33.6227
x11	-20.3898	-20.4848	6.6564
y11	-39.2961	-16.4289	-15.8711
z11	28.2759	-61.5103	13.9334
x12	-39.6708	-2.1092	15.9257
y12	-66.7333	2.5135	14.1642
z12	-12.5494	-20.6410	15.1959
x13	-47.7912	5.3847	13.7298
y13	-24.8589	4.1440	-4.2861
z13	16.6140	5.6235	9.7864
x14	-38.8689	21.0070	22.0994

y14	-27.1200	-39.6183	17.2566
z14	-32.6317	-24.1151	10.6494
x15	-57.9466	18.2158	2.5011
y15	-28.2371	-30.3487	-3.3306
z15	-5.3458	-3.1153	31.1368
x16	-13.6049	33.4674	12.6629
y16	16.1457	-20.3025	9.9424
z16	-14.0659	-10.4652	13.2618
x17	38.6918	1.9253	-0.2835
y17	26.7457	-46.5008	-14.3324
z17	10.1931	-5.3147	2.7899
x18	59.4852	20.6592	3.1444
y18	-24.4721	-9.0569	-18.7993
z18	11.8540	13.1932	-9.2477
x19	-4.6425	57.5601	-12.5014
y19	-113.7834	43.7641	-31.5751
z19	-16.7459	59.9907	-20.6991
x20	36.6267	2.9524	-10.7432
y20	-20.0568	79.0483	-0.2361
z20	-6.5496	46.2748	-32.7590
x21	-44.0676	-36.6557	-28.0068
y21	-22.8371	82.7068	6.0945
z21	-57.4939	52.9545	-47.9339
x22	63.5407	-28.8870	-5.4204
y22	-9.2510	40.7573	25.4049
z22	-31.1967	4.4596	-29.3995
x23	-9.9985	-97.3232	-6.0888
y23	16.5860	53.3285	22.7353
z23	-43.5817	-9.7430	-19.3715
x24	42.5507	-47.9488	1.9479
y24	10.3110	17.6160	11.9781
z24	-7.2362	-11.9901	3.5022
x25	51.6634	-4.1150	-18.7004
y25	20.1537	15.9440	9.2343
z25	-2.6753	-26.4217	5.5487
x26	16.9230	33.7221	7.0639
y26	21.2752	-18.5360	-11.4050
z26	39.3963	-25.6764	41.4000
x27	6.7674	-35.9515	16.9322
y27	37.0280	2.1554	-23.3738
z27	18.4842	-36.2807	28.2929
x28	27.7776	-52.9623	8.4827
y28	13.1871	21.7254	-37.6347
z28	2.9436	-48.4940	14.6951
x29	41.8264	14.5358	3.7834
y29	-24.5352	-56.7783	-13.8194
z29	26.2953	-0.9962	27.6532
x30	-26.9758	-37.4055	-11.4408
y30	0.0556	-27.8603	14.8347
z30	47.1316	24.5785	9.5628
x31	-71.2411	28.4881	-40.0298
y31	59.6973	-119.7992	65.9261
z31	16.4138	58.4017	-10.1417
x32	3.5071	25.8450	17.7011
y32	-41.5931	-175.9457	-19.3476
z32	-3.8679	69.8796	-9.6709
x33	3.2164	13.2919	-0.3382
y33	5.0856	-95.0121	15.0388
z33	-14.1888	40.9325	-29.4068
x34	17.8457	-3.7225	1.0069

y34	-36.3141	6.9733	22.0660
z34	-14.8933	-28.6085	-13.8617
x35	-9.2249	17.5320	-1.8379
y35	20.5894	121.4410	48.0029
z35	-31.5440	-62.0932	-31.5162
x36	-3.4067	7.4062	7.9517
y36	52.2443	24.5154	44.1207
z36	-31.9325	-41.3098	-15.7329
x37	25.8467	4.3083	-5.7040
y37	-28.3363	44.2497	-72.4243
z37	37.5900	3.4619	38.8997
x38	-29.1599	-12.1421	-11.6883
y38	-40.1438	4.9257	-120.3971
z38	57.7919	-15.8546	6.3493
x39	26.3880	-4.4280	14.3475
y39	68.5546	-6.3950	-25.0950
z39	6.4453	30.2259	-43.6446
x40	7.6422	34.0640	-0.1401
y40	52.3286	7.5770	0.2932
z40	-32.6532	88.2939	-26.8295
x41	-6.2440	47.3789	6.0433
y41	13.9353	5.7105	13.6184
z41	5.9420	-2.3596	6.8319
x42	-3.9045	44.7680	9.3180
y42	-1.9783	5.5715	30.1254
z42	13.3774	-80.1025	16.9586
x43	26.6976	47.1596	-11.6402
y43	1.1708	6.9619	10.7642
z43	-34.0075	-36.5324	39.5629
x44	-23.8823	2.1066	-36.4806
y44	5.8919	14.8669	18.1818
z44	-34.3878	1.7457	59.1116
x45	-108.7522	-0.0850	8.3171
y45	38.2206	34.6245	36.6613
z45	-15.3213	9.7119	38.8546
x46	40.9943	-21.9354	69.7461
y46	-72.6807	35.6886	-44.3683
z46	40.5574	120.2803	34.2208
x47	24.8440	68.2207	-26.2958
y47	-76.7286	33.6494	-36.4223
z47	-10.4483	-25.4433	-38.2538
x48	102.2597	-20.5501	-35.3470
y48	-26.4472	-13.8340	-24.6305
z48	-45.6340	-1.5399	-26.1930
x49	45.5673	-91.6566	-26.6369
y49	-5.5470	-33.1212	-26.5055
z49	13.3085	-1.6605	15.1760
x50	39.9195	-52.7706	10.1329
y50	30.9863	-47.1465	-10.5018
z50	25.9517	-1.3040	7.4158
x51	2.7769	-15.8770	21.9197
y51	45.3224	-56.9594	14.1589
z51	3.0683	-17.9702	-34.2844
x52	-48.6715	-73.2203	10.2805
y52	45.7900	-43.8961	18.0218
z52	13.2357	-57.9547	-25.4699
x53	-88.4728	62.4751	-49.9648
y53	52.6724	-25.2866	22.9351
z53	22.8450	-90.2015	-8.0769
x54	-54.6048	26.9743	19.9633

y54	51.0178	-19.7103	7.5590
z54	12.8366	-3.1472	-33.7146

New Procrustes fit:
Dataset 'lateral ampulla'

10 landmarks in 3 dimensions.
The dataset contains 40 observations, of which 36 are included for analyses.

Average shape:

Lmk.	Axis 1 (x)	Axis 2 (y)	Axis 3 (z)
1	-0.49039026	0.00873083	0.07377795
2	0.48195237	0.00358925	0.08655685
3	0.37691702	-0.00381854	0.04265556
4	0.27154060	-0.00261491	-0.00889215
5	0.16471040	0.00176874	-0.04982723
6	0.05751783	0.00314274	-0.07103385
7	-0.04915225	0.00186744	-0.06999551
8	-0.15799661	-0.00042235	-0.04826330
9	-0.26980618	-0.00401132	-0.00541953
10	-0.38529293	-0.00823188	0.05044121

Procrustes sums of squares: 0.2300763713330483
Tangent sums of squares: 0.2277359474153324

Data matrices in this dataset:
- lateral ampulla, raw data
- lateral ampulla, centroid size
- lateral ampulla, Procrustes coordinates

New Procrustes fit:
Dataset 'lateral ampulla'

10 landmarks in 3 dimensions.
The dataset contains 40 observations, of which 36 are included for analyses.

Average shape:

Lmk.	Axis 1 (x)	Axis 2 (y)	Axis 3 (z)
1	-0.49039026	0.00873083	0.07377795
2	0.48195237	0.00358925	0.08655685
3	0.37691702	-0.00381854	0.04265556
4	0.27154060	-0.00261491	-0.00889215
5	0.16471040	0.00176874	-0.04982723
6	0.05751783	0.00314274	-0.07103385
7	-0.04915225	0.00186744	-0.06999551
8	-0.15799661	-0.00042235	-0.04826330
9	-0.26980618	-0.00401132	-0.00541953
10	-0.38529293	-0.00823188	0.05044121

Procrustes sums of squares: 0.2300763713330483
Tangent sums of squares: 0.2277359474153324

Data matrices in this dataset:

- lateral ampulla, raw data
- lateral ampulla, centroid size
- lateral ampulla, Procrustes coordinates

Canonical Variate Analysis: CVA ...lateral ampulla

Dataset: lateral ampulla
Classification criterion: subfam

Groups		Observations
1.	Ca	9
2.	Ce	13
3.	Di	3
4.	Pr	11

Variation among groups, scaled by the inverse of the within-group variation

	Eigenvalues	% Variance	Cumulative %
1.	3.64193932	61.469	61.469
2.	1.25243347	21.139	82.607
3.	1.03049979	17.393	100.000

Mahalanobis distances among groups:

	Ca	Ce	Di
Ce	3.7551		
Di	6.5688	4.7474	
Pr	2.7831	2.9494	5.8280

P-values from permutation tests (10000 permutation rounds) for Mahalanobis distances among groups:

	Ca	Ce	Di
Ce	<.0001		
Di	0.0036	0.0009	
Pr	0.0040	0.0002	0.0022

Procrustes distances among groups:

	Ca	Ce	Di
Ce	0.0333		
Di	0.0453	0.0273	
Pr	0.0383	0.0148	0.0313

P-values from permutation tests (10000 permutation rounds) for Procrustes distances among groups:

	Ca	Ce	Di
Ce	0.4528		
Di	0.5726	0.8966	
Pr	0.2638	0.9358	0.8355

Canonical coefficients:

	CV1	CV2	CV3
x1	-172.5012	-281.8297	-41.8397

y1	-56.7850	149.7679	86.7400
z1	-30.4483	-90.8332	-15.7825
x2	-12.0205	472.6764	108.3296
y2	-0.8243	54.3640	-88.9888
z2	62.1107	-35.2507	-64.4879
x3	-57.4531	-372.8710	40.4304
y3	-111.3689	-144.5674	229.6690
z3	-245.8241	26.0171	143.9143
x4	560.9743	-462.5037	-427.1807
y4	296.5917	223.4587	-153.2464
z4	334.7098	41.0727	-65.1580
x5	-205.8780	750.9401	533.0204
y5	-221.5034	-264.1084	-29.2949
z5	-62.3750	-71.3574	-94.2405
x6	-1158.9029	-705.2555	-585.2017
y6	302.5267	76.1099	-39.3635
z6	10.4125	28.3720	138.9830
x7	901.6937	-576.8087	319.8735
y7	-1202.4353	185.0871	151.8954
z7	-485.1214	111.8568	42.1318
x8	-346.2830	756.3684	-31.1018
y8	1624.0736	30.4657	103.4894
z8	641.7551	-72.0618	-154.8202
x9	638.7953	615.1163	121.1943
y9	-853.6433	-232.4416	-247.8412
z9	-247.8650	-74.9449	17.9714
x10	-148.4246	-195.8325	-37.5243
y10	223.3681	-78.1358	-13.0590
z10	22.6457	137.1294	51.4887

New Procrustes fit:
Dataset 'lateral canal without ampulla'

46 landmarks in 3 dimensions.
The dataset contains 40 observations, of which 36 are included for analyses.

Average shape:

Lmk.	Axis 1 (x)	Axis 2 (y)	Axis 3 (z)
1	-0.01422325	-0.00486392	0.17502202
2	-0.02849486	-0.00460471	0.17343794
3	-0.04231882	-0.00388989	0.16677824
4	-0.05491600	-0.00318597	0.15812771
5	-0.06666181	-0.00242626	0.14757605
6	-0.07762733	-0.00204783	0.13514006
7	-0.08841510	-0.00202901	0.12081960
8	-0.09841438	-0.00210618	0.10420282
9	-0.10767680	-0.00253689	0.08546599
10	-0.11565837	-0.00276059	0.06435356
11	-0.12069570	-0.00230653	0.04828588
12	-0.12437649	-0.00176142	0.03240773
13	-0.11808934	0.00517224	0.01571079
14	-0.12710737	-0.00042876	0.00132863
15	-0.12632406	0.00045969	-0.01372684
16	-0.12420428	0.00134459	-0.02866362
17	-0.12062505	0.00229722	-0.04332537
18	-0.11584749	0.00339602	-0.05792602
19	-0.10967595	0.00461990	-0.07218078

20	-0.10052035	0.00492492	-0.08793656
21	-0.09039393	0.00502196	-0.10172373
22	-0.07927052	0.00487383	-0.11412862
23	-0.06678694	0.00440415	-0.12301204
24	-0.05331054	0.00350106	-0.13090674
25	-0.03853135	0.00244442	-0.13595588
26	-0.02243868	0.00111448	-0.13871310
27	-0.00519335	-0.00038209	-0.13929013
28	0.01329088	-0.00219973	-0.13752471
29	0.03759241	-0.00492554	-0.13250246
30	0.05979814	-0.00691009	-0.12513507
31	0.07965690	-0.00813938	-0.11520791
32	0.09743373	-0.00847814	-0.10314037
33	0.11323254	-0.00816059	-0.08928651
34	0.12694489	-0.00687189	-0.07315716
35	0.13815403	-0.00481031	-0.05465567
36	0.14831421	-0.00198107	-0.03449907
37	0.15601673	0.00158705	-0.01214472
38	0.15834885	0.00334267	0.00642109
39	0.15752703	0.00498154	0.02452515
40	0.15526957	0.00617165	0.04151531
41	0.15172945	0.00651432	0.05757514
42	0.14683610	0.00615869	0.07302015
43	0.13993658	0.00567980	0.08796764
44	0.13127773	0.00473089	0.10259479
45	0.12020947	0.00320688	0.11642608
46	0.10622888	0.00185881	0.12604074

Procrustes sums of squares: 0.585947954281353
Tangent sums of squares: 0.5552664316745958

Data matrices in this dataset:
- lateral canal without ampulla, raw data
- lateral canal without ampulla, centroid size
- lateral canal without ampulla, Procrustes coordinates

New Procrustes fit:
Dataset 'lateral canal without ampulla'

46 landmarks in 3 dimensions.
The dataset contains 40 observations, of which 36 are included for analyses.

Average shape:

Lmk.	Axis 1 (x)	Axis 2 (y)	Axis 3 (z)
1	-0.01422325	-0.00486392	0.17502202
2	-0.02849486	-0.00460471	0.17343794
3	-0.04231882	-0.00388989	0.16677824
4	-0.05491600	-0.00318597	0.15812771
5	-0.06666181	-0.00242626	0.14757605
6	-0.07762733	-0.00204783	0.13514006
7	-0.08841510	-0.00202901	0.12081960
8	-0.09841438	-0.00210618	0.10420282
9	-0.10767680	-0.00253689	0.08546599
10	-0.11565837	-0.00276059	0.06435356
11	-0.12069570	-0.00230653	0.04828588
12	-0.12437649	-0.00176142	0.03240773

13	-0.11808934	0.00517224	0.01571079
14	-0.12710737	-0.00042876	0.00132863
15	-0.12632406	0.00045969	-0.01372684
16	-0.12420428	0.00134459	-0.02866362
17	-0.12062505	0.00229722	-0.04332537
18	-0.11584749	0.00339602	-0.05792602
19	-0.10967595	0.00461990	-0.07218078
20	-0.10052035	0.00492492	-0.08793656
21	-0.09039393	0.00502196	-0.10172373
22	-0.07927052	0.00487383	-0.11412862
23	-0.06678694	0.00440415	-0.12301204
24	-0.05331054	0.00350106	-0.13090674
25	-0.03853135	0.00244442	-0.13595588
26	-0.02243868	0.00111448	-0.13871310
27	-0.00519335	-0.00038209	-0.13929013
28	0.01329088	-0.00219973	-0.13752471
29	0.03759241	-0.00492554	-0.13250246
30	0.05979814	-0.00691009	-0.12513507
31	0.07965690	-0.00813938	-0.11520791
32	0.09743373	-0.00847814	-0.10314037
33	0.11323254	-0.00816059	-0.08928651
34	0.12694489	-0.00687189	-0.07315716
35	0.13815403	-0.00481031	-0.05465567
36	0.14831421	-0.00198107	-0.03449907
37	0.15601673	0.00158705	-0.01214472
38	0.15834885	0.00334267	0.00642109
39	0.15752703	0.00498154	0.02452515
40	0.15526957	0.00617165	0.04151531
41	0.15172945	0.00651432	0.05757514
42	0.14683610	0.00615869	0.07302015
43	0.13993658	0.00567980	0.08796764
44	0.13127773	0.00473089	0.10259479
45	0.12020947	0.00320688	0.11642608
46	0.10622888	0.00185881	0.12604074

Procrustes sums of squares: 0.585947954281353
Tangent sums of squares: 0.5552664316745958

Data matrices in this dataset:
- lateral canal without ampulla, raw data
- lateral canal without ampulla, centroid size
- lateral canal without ampulla, Procrustes coordinates

Canonical Variate Analysis: CVA ... latarale canal without ampulla

Dataset: lateral canal without ampulla
Classification criterion: subfam

Groups		Observations
1.	Ca	9
2.	Ce	13
3.	Di	3
4.	Pr	11

Variation among groups, scaled by the inverse of the within-group variation

	Eigenvalues	% Variance	Cumulative %
1.	8.84755842	59.638	59.638
2.	4.07573224	27.473	87.111
3.	1.91212452	12.889	100.000

Mahalanobis distances among groups:

	Ca	Ce	Di
Ce	4.9714		
Di	6.6899	6.0031	
Pr	6.2854	6.7661	5.8447

P-values from permutation tests (10000 permutation rounds) for Mahalanobis distances among groups:

	Ca	Ce	Di
Ce	<.0001		
Di	0.0017	0.0011	
Pr	<.0001	<.0001	0.0027

Procrustes distances among groups:

	Ca	Ce	Di
Ce	0.0631		
Di	0.0801	0.0677	
Pr	0.0548	0.0816	0.0711

P-values from permutation tests (10000 permutation rounds) for Procrustes distances among groups:

	Ca	Ce	Di
Ce	0.2132		
Di	0.3285	0.5155	
Pr	0.3529	0.0177	0.4250

Canonical coefficients:

	CV1	CV2	CV3
x1	-25.7663	16.4081	-134.3474
y1	84.8573	92.7956	30.2190
z1	110.3509	-122.9652	32.0154
x2	-8.8982	51.9773	-2.4608
y2	-13.4620	-23.3740	20.8658
z2	61.4303	161.4505	-29.0886
x3	-33.6291	-69.3531	33.9870
y3	-52.0474	-27.5719	25.6000
z3	82.0504	14.3346	-62.1002
x4	42.3344	-37.5161	-32.7154
y4	-53.8530	-46.7199	-38.9045
z4	-167.1738	-113.5559	101.9945
x5	-36.5198	-72.7581	27.1763
y5	28.5369	-5.3326	-86.3637
z5	43.4557	49.2342	-26.3146
x6	-16.1292	-68.5437	77.6807
y6	11.6798	27.5700	12.2361
z6	10.1753	71.6604	-12.6124
x7	-29.2913	70.8271	53.0626
y7	23.9026	90.6757	-29.2612
z7	9.6725	-4.8370	-14.9411
x8	0.9020	76.8113	104.9683

y8	16.1544	95.3179	46.5103
z8	-18.0224	-8.5216	-15.1913
x9	99.1520	-8.8145	-187.5448
y9	61.4234	-23.6611	-86.6392
z9	-48.9526	30.3981	106.7665
x10	13.5998	28.0789	-69.2625
y10	-132.3093	-29.2128	126.6418
z10	40.8778	-13.7065	15.6795
x11	-21.5480	-44.6688	9.8575
y11	78.4001	-28.9182	-27.4136
z11	52.2049	-3.3090	-25.9516
x12	-134.4154	-44.3609	179.5478
y12	-39.2809	-94.7146	65.5309
z12	30.1266	-33.5232	-31.8024
x13	32.3125	16.8568	14.9468
y13	-24.5432	-20.6032	-16.8762
z13	2.2829	-38.0320	5.9103
x14	125.8630	-36.4280	-18.8493
y14	-14.2711	-85.3909	11.5683
z14	-24.0868	-22.8748	7.4435
x15	86.6905	-69.7225	-30.4888
y15	-8.1746	-100.4757	-50.0587
z15	9.8900	-20.9942	-1.2228
x16	151.8623	61.7831	-14.0274
y16	27.2864	10.2017	-62.9457
z16	62.3963	58.0727	-15.5217
x17	-63.8273	-91.8901	-84.1692
y17	13.3496	56.9633	-64.2211
z17	-80.4108	-24.0489	-5.5634
x18	3.1385	62.9253	-46.4075
y18	21.9719	97.2319	-14.8250
z18	-55.9350	38.4697	-17.5421
x19	27.9435	101.9426	-4.0760
y19	-64.9067	58.7174	98.0085
z19	-26.9668	70.7694	-13.1504
x20	-75.0544	10.1828	13.2014
y20	33.1285	52.8351	-25.0168
z20	-15.2084	-0.7886	-50.9083
x21	-119.7120	21.5743	152.3082
y21	36.3676	81.0241	89.0801
z21	7.7290	-15.0748	43.3343
x22	-22.1674	16.1508	70.4156
y22	43.7646	1.8352	-3.0509
z22	5.8307	74.6634	89.2932
x23	-13.2065	29.5533	26.5441
y23	24.8534	-4.6871	54.6947
z23	94.9052	10.7346	-54.2199
x24	27.8695	-24.5087	-44.3949
y24	-1.3296	-95.2883	12.0544
z24	-4.0292	-38.1156	-5.7091
x25	-10.6027	-36.3632	-14.8896
y25	-4.5212	-95.8137	-3.6054
z25	-20.8527	-37.6504	-4.6339
x26	2.7316	-35.9312	-18.2210
y26	-48.4466	-79.2547	-81.8210
z26	-102.6503	-79.3820	2.7989
x27	21.0102	21.8570	-34.0386
y27	11.8210	-0.5727	-75.4024
z27	64.9250	50.2165	-36.0059
x28	-9.3560	45.1064	-56.2202

y28	-20.3428	29.5914	-67.4589
z28	29.8536	27.4219	-8.0764
x29	-11.8043	10.7647	22.3108
y29	-64.0480	10.7869	76.7271
z29	7.0491	-20.9398	-117.8860
x30	-30.5500	12.5358	33.2205
y30	5.8264	77.9181	67.4357
z30	27.4386	-61.4693	-25.0685
x31	29.2046	-43.4224	-27.7896
y31	16.3666	18.1510	65.2718
z31	-38.0985	59.0688	94.4750
x32	130.4722	-23.6831	-8.8808
y32	-9.0969	-24.2263	-7.6699
z32	-103.6217	-30.3396	8.5550
x33	117.6477	-73.3425	-136.7602
y33	40.4160	106.6772	-48.3699
z33	-78.1129	33.6445	110.2909
x34	-5.6535	78.6181	-83.8275
y34	18.0814	-56.3991	-46.1151
z34	47.2327	-97.7717	34.7694
x35	-64.8615	-23.6116	80.3628
y35	7.9065	33.1082	-13.2652
z35	54.8762	92.6294	-24.0119
x36	-97.6321	-155.4759	87.0085
y36	5.1302	19.5809	40.8876
z36	50.0331	118.8392	-28.5154
x37	-28.0815	46.6730	95.8965
y37	-49.3347	-36.0573	7.5008
z37	-20.6668	-24.1931	-107.4956
x38	-1.2123	64.4475	2.0887
y38	35.8454	52.9535	-84.7054
z38	-30.5160	-91.2857	17.1777
x39	28.1427	103.6860	-60.4169
y39	-26.0566	-40.1985	34.1074
z39	11.8021	-16.9673	-10.1220
x40	71.7295	181.4320	-98.7121
y40	-91.1016	-139.2304	77.0537
z40	-2.0145	-10.3634	36.1987
x41	-41.0644	-31.4589	29.0573
y41	-17.7017	51.2399	12.0108
z41	-26.0152	-1.3624	93.3233
x42	-141.1876	-63.1727	80.8336
y42	57.0886	17.6320	-85.5877
z42	-9.4897	34.6652	77.8494
x43	-76.7029	-80.3688	-121.0636
y43	139.3696	52.8231	-55.5745
z43	4.5682	85.5471	-44.8420
x44	-45.7451	-136.4850	63.3395
y44	54.0770	-11.8368	88.5966
z44	32.2617	-4.6046	-60.3396
x45	49.0782	59.6375	102.6609
y45	-77.6329	-135.8599	33.9043
z45	35.2994	-105.9912	-11.4455
x46	102.9346	82.0501	-30.9115
y46	-85.1445	69.7701	-21.3536
z46	-115.8941	-39.1525	-17.5929

New Procrustes fit:
Dataset 'anterior canal'

45 landmarks in 3 dimensions.
The dataset contains 40 observations, of which 36 are included for analyses.

Average shape:

Lmk.	Axis 1 (x)	Axis 2 (y)	Axis 3 (z)
1	-0.03993505	0.12281877	0.00853177
2	-0.05705920	0.12484950	0.00277316
3	-0.07431261	0.12060500	-0.00155152
4	-0.08971966	0.11299453	-0.00505493
5	-0.10351453	0.10311918	-0.00788451
6	-0.11625976	0.09109379	-0.01045237
7	-0.12809965	0.07635960	-0.01262566
8	-0.13879926	0.05853652	-0.01435004
9	-0.14774752	0.03773457	-0.01513335
10	-0.15419989	0.01390003	-0.01462843
11	-0.15810425	-0.01610136	-0.01237334
12	-0.15674753	-0.04409017	-0.00929943
13	-0.15068919	-0.06944744	-0.00575053
14	-0.14117108	-0.09237155	-0.00263553
15	-0.12850458	-0.11307778	0.00020938
16	-0.11205449	-0.13178405	0.00288852
17	-0.09184179	-0.14843693	0.00544448
18	-0.06743322	-0.16255337	0.00803593
19	-0.03882778	-0.17260224	0.01105980
20	-0.00589144	-0.17605887	0.01026846
21	0.02458168	-0.17278966	0.00951813
22	0.05213088	-0.16479847	0.00801711
23	0.07671945	-0.15252109	0.00573985
24	0.09901799	-0.13667844	0.00216344
25	0.11825075	-0.11516376	-0.00154199
26	0.13463928	-0.08852136	-0.00557218
27	0.14898252	-0.05754486	-0.01056072
28	0.15920771	-0.02156265	-0.01582120
29	0.16233541	-0.00326920	-0.01687694
30	0.16214382	0.01368543	-0.01672341
31	0.15854200	0.02900264	-0.01553389
32	0.15161778	0.04257480	-0.01333676
33	0.14164325	0.05451993	-0.01024221
34	0.12863805	0.06505091	-0.00623711
35	0.11304329	0.07471651	-0.00137110
36	0.09423923	0.08376532	0.00425860
37	0.07201712	0.08900676	0.01117890
38	0.05804353	0.08653051	0.01441282
39	0.04407076	0.08243270	0.01720033
40	0.03051111	0.08104515	0.01896798
41	0.01757128	0.08222628	0.01966491
42	0.00535760	0.08618170	0.01931869
43	-0.00655094	0.09240610	0.01806055
44	-0.01769466	0.10140850	0.01566551
45	-0.02814639	0.11280851	0.01217882

Procrustes sums of squares: 0.3165685946407284
Tangent sums of squares: 0.31274914641129736

Data matrices in this dataset:
- anterior canal, raw data
- anterior canal, centroid size

- anterior canal, Procrustes coordinates

New Procrustes fit:
Dataset 'anterior canal'

45 landmarks in 3 dimensions.
The dataset contains 40 observations, of which 36 are included for analyses.

Average shape:

Lmk.	Axis 1 (x)	Axis 2 (y)	Axis 3 (z)
1	-0.03993505	0.12281877	0.00853177
2	-0.05705920	0.12484950	0.00277316
3	-0.07431261	0.12060500	-0.00155152
4	-0.08971966	0.11299453	-0.00505493
5	-0.10351453	0.10311918	-0.00788451
6	-0.11625976	0.09109379	-0.01045237
7	-0.12809965	0.07635960	-0.01262566
8	-0.13879926	0.05853652	-0.01435004
9	-0.14774752	0.03773457	-0.01513335
10	-0.15419989	0.01390003	-0.01462843
11	-0.15810425	-0.01610136	-0.01237334
12	-0.15674753	-0.04409017	-0.00929943
13	-0.15068919	-0.06944744	-0.00575053
14	-0.14117108	-0.09237155	-0.00263553
15	-0.12850458	-0.11307778	0.00020938
16	-0.11205449	-0.13178405	0.00288852
17	-0.09184179	-0.14843693	0.00544448
18	-0.06743322	-0.16255337	0.00803593
19	-0.03882778	-0.17260224	0.01105980
20	-0.00589144	-0.17605887	0.01026846
21	0.02458168	-0.17278966	0.00951813
22	0.05213088	-0.16479847	0.00801711
23	0.07671945	-0.15252109	0.00573985
24	0.09901799	-0.13667844	0.00216344
25	0.11825075	-0.11516376	-0.00154199
26	0.13463928	-0.08852136	-0.00557218
27	0.14898252	-0.05754486	-0.01056072
28	0.15920771	-0.02156265	-0.01582120
29	0.16233541	-0.00326920	-0.01687694
30	0.16214382	0.01368543	-0.01672341
31	0.15854200	0.02900264	-0.01553389
32	0.15161778	0.04257480	-0.01333676
33	0.14164325	0.05451993	-0.01024221
34	0.12863805	0.06505091	-0.00623711
35	0.11304329	0.07471651	-0.00137110
36	0.09423923	0.08376532	0.00425860
37	0.07201712	0.08900676	0.01117890
38	0.05804353	0.08653051	0.01441282
39	0.04407076	0.08243270	0.01720033
40	0.03051111	0.08104515	0.01896798
41	0.01757128	0.08222628	0.01966491
42	0.00535760	0.08618170	0.01931869
43	-0.00655094	0.09240610	0.01806055
44	-0.01769466	0.10140850	0.01566551
45	-0.02814639	0.11280851	0.01217882

Procrustes sums of squares: 0.3165685946407284
Tangent sums of squares: 0.31274914641129736

Data matrices in this dataset:
- anterior canal, raw data
- anterior canal, centroid size
- anterior canal, Procrustes coordinates

New Procrustes fit:
Dataset 'anterior canal'

45 landmarks in 3 dimensions.
The dataset contains 40 observations, of which 36 are included for analyses.

Average shape:

Lmk.	Axis 1 (x)	Axis 2 (y)	Axis 3 (z)
1	-0.03993505	0.12281877	0.00853177
2	-0.05705920	0.12484950	0.00277316
3	-0.07431261	0.12060500	-0.00155152
4	-0.08971966	0.11299453	-0.00505493
5	-0.10351453	0.10311918	-0.00788451
6	-0.11625976	0.09109379	-0.01045237
7	-0.12809965	0.07635960	-0.01262566
8	-0.13879926	0.05853652	-0.01435004
9	-0.14774752	0.03773457	-0.01513335
10	-0.15419989	0.01390003	-0.01462843
11	-0.15810425	-0.01610136	-0.01237334
12	-0.15674753	-0.04409017	-0.00929943
13	-0.15068919	-0.06944744	-0.00575053
14	-0.14117108	-0.09237155	-0.00263553
15	-0.12850458	-0.11307778	0.00020938
16	-0.11205449	-0.13178405	0.00288852
17	-0.09184179	-0.14843693	0.00544448
18	-0.06743322	-0.16255337	0.00803593
19	-0.03882778	-0.17260224	0.01105980
20	-0.00589144	-0.17605887	0.01026846
21	0.02458168	-0.17278966	0.00951813
22	0.05213088	-0.16479847	0.00801711
23	0.07671945	-0.15252109	0.00573985
24	0.09901799	-0.13667844	0.00216344
25	0.11825075	-0.11516376	-0.00154199
26	0.13463928	-0.08852136	-0.00557218
27	0.14898252	-0.05754486	-0.01056072
28	0.15920771	-0.02156265	-0.01582120
29	0.16233541	-0.00326920	-0.01687694
30	0.16214382	0.01368543	-0.01672341
31	0.15854200	0.02900264	-0.01553389
32	0.15161778	0.04257480	-0.01333676
33	0.14164325	0.05451993	-0.01024221
34	0.12863805	0.06505091	-0.00623711
35	0.11304329	0.07471651	-0.00137110
36	0.09423923	0.08376532	0.00425860
37	0.07201712	0.08900676	0.01117890
38	0.05804353	0.08653051	0.01441282
39	0.04407076	0.08243270	0.01720033
40	0.03051111	0.08104515	0.01896798

41	0.01757128	0.08222628	0.01966491
42	0.00535760	0.08618170	0.01931869
43	-0.00655094	0.09240610	0.01806055
44	-0.01769466	0.10140850	0.01566551
45	-0.02814639	0.11280851	0.01217882

Procrustes sums of squares: 0.3165685946407284
Tangent sums of squares: 0.31274914641129736

Data matrices in this dataset:
- anterior canal, raw data
- anterior canal, centroid size
- anterior canal, Procrustes coordinates

New Procrustes fit:
Dataset 'anterior canal'

45 landmarks in 3 dimensions.
The dataset contains 40 observations, of which 36 are included for analyses.

Average shape:

Lmk.	Axis 1 (x)	Axis 2 (y)	Axis 3 (z)
1	-0.03993505	0.12281877	0.00853177
2	-0.05705920	0.12484950	0.00277316
3	-0.07431261	0.12060500	-0.00155152
4	-0.08971966	0.11299453	-0.00505493
5	-0.10351453	0.10311918	-0.00788451
6	-0.11625976	0.09109379	-0.01045237
7	-0.12809965	0.07635960	-0.01262566
8	-0.13879926	0.05853652	-0.01435004
9	-0.14774752	0.03773457	-0.01513335
10	-0.15419989	0.01390003	-0.01462843
11	-0.15810425	-0.01610136	-0.01237334
12	-0.15674753	-0.04409017	-0.00929943
13	-0.15068919	-0.06944744	-0.00575053
14	-0.14117108	-0.09237155	-0.00263553
15	-0.12850458	-0.11307778	0.00020938
16	-0.11205449	-0.13178405	0.00288852
17	-0.09184179	-0.14843693	0.00544448
18	-0.06743322	-0.16255337	0.00803593
19	-0.03882778	-0.17260224	0.01105980
20	-0.00589144	-0.17605887	0.01026846
21	0.02458168	-0.17278966	0.00951813
22	0.05213088	-0.16479847	0.00801711
23	0.07671945	-0.15252109	0.00573985
24	0.09901799	-0.13667844	0.00216344
25	0.11825075	-0.11516376	-0.00154199
26	0.13463928	-0.08852136	-0.00557218
27	0.14898252	-0.05754486	-0.01056072
28	0.15920771	-0.02156265	-0.01582120
29	0.16233541	-0.00326920	-0.01687694
30	0.16214382	0.01368543	-0.01672341
31	0.15854200	0.02900264	-0.01553389
32	0.15161778	0.04257480	-0.01333676
33	0.14164325	0.05451993	-0.01024221
34	0.12863805	0.06505091	-0.00623711

35	0.11304329	0.07471651	-0.00137110
36	0.09423923	0.08376532	0.00425860
37	0.07201712	0.08900676	0.01117890
38	0.05804353	0.08653051	0.01441282
39	0.04407076	0.08243270	0.01720033
40	0.03051111	0.08104515	0.01896798
41	0.01757128	0.08222628	0.01966491
42	0.00535760	0.08618170	0.01931869
43	-0.00655094	0.09240610	0.01806055
44	-0.01769466	0.10140850	0.01566551
45	-0.02814639	0.11280851	0.01217882

Procrustes sums of squares: 0.3165685946407284
Tangent sums of squares: 0.31274914641129736

Data matrices in this dataset:
- anterior canal, raw data
- anterior canal, centroid size
- anterior canal, Procrustes coordinates

Canonical Variate Analysis: CVA ... anterior canal

Dataset: anterior canal
Classification criterion: subfam

Groups		Observations
1.	Ca	9
2.	Ce	13
3.	Di	3
4.	Pr	11

Variation among groups, scaled by the inverse of the within-group variation

	Eigenvalues	% Variance	Cumulative %
1.	9.44559667	64.978	64.978
2.	3.83766863	26.400	91.378
3.	1.25338817	8.622	100.000

Mahalanobis distances among groups:

	Ca	Ce	Di
Ce	6.3213		
Di	4.5646	5.6767	
Pr	4.9191	6.8464	5.9116

P-values from permutation tests (10000 permutation rounds) for Mahalanobis distances among groups:

	Ca	Ce	Di
Ce	<.0001		
Di	0.0152	0.0010	
Pr	<.0001	<.0001	0.0020

Procrustes distances among groups:

	Ca	Ce	Di

Ce	0.0599		
Di	0.0474	0.0799	
Pr	0.0346	0.0457	0.0575

P-values from permutation tests (10000 permutation rounds) for Procrustes distances among groups:

	Ca	Ce	Di
Ce	0.0384		
Di	0.7674	0.1240	
Pr	0.7051	0.2035	0.5371

Canonical coefficients:

	CV1	CV2	CV3
x1	36.7984	91.1076	-12.9625
y1	-1.1667	91.4539	-13.8652
z1	68.7593	60.2121	99.5373
x2	-3.1630	25.7302	7.7034
y2	41.3154	41.8173	82.7413
z2	-66.5710	-91.5158	-30.2997
x3	51.8824	24.1210	-9.6589
y3	-169.7448	16.1392	35.7052
z3	-141.0394	-18.7444	-41.5302
x4	-18.6147	78.1282	-12.1963
y4	103.2433	-206.3855	12.1767
z4	-231.8868	83.5534	-85.0992
x5	-25.0250	-19.2422	-6.7654
y5	59.7108	-2.2326	23.0874
z5	-19.1870	11.6657	43.9499
x6	2.5346	-46.3365	34.5315
y6	-12.8674	29.1188	-10.1964
z6	59.9398	12.7279	78.1750
x7	-16.0599	-21.7594	-17.4692
y7	-14.4078	-28.7478	-1.2913
z7	37.8643	-15.0097	12.3892
x8	12.8417	33.8394	-9.4268
y8	1.5419	24.4698	17.5487
z8	77.2231	10.5057	-28.2580
x9	19.8220	-21.6171	-31.3148
y9	59.2664	10.2269	3.8705
z9	116.0767	-83.0432	-68.3718
x10	-128.0627	-0.6280	-0.4455
y10	67.8100	61.0105	-8.5445
z10	113.9333	63.1445	-23.1943
x11	-48.4112	143.7317	122.0588
y11	30.8882	-25.9454	-37.4730
z11	84.1346	-17.2177	32.2440
x12	117.2885	-34.8162	27.3719
y12	20.4963	-37.7662	6.7997
z12	73.1175	-53.7464	45.9893
x13	40.8886	-37.8349	26.2742
y13	-18.6807	-32.3970	17.7664
z13	-98.1899	54.1398	21.9403
x14	76.0628	-55.1034	-33.5235
y14	-32.0558	-30.7884	7.6239
z14	-100.3670	69.6080	-5.3037
x15	1.7597	-29.5014	-68.1088
y15	-88.7385	-28.1083	-14.6676
z15	-137.8603	93.3255	-4.5714

x16	109.5085	-1.6795	-39.9716
y16	10.4349	3.0768	-17.0350
z16	-30.3570	37.0184	-6.6831
x17	57.5750	27.6465	29.7229
y17	25.4202	23.7961	38.9460
z17	18.5183	-48.9068	6.1548
x18	12.4024	54.7723	15.2756
y18	17.1661	106.9268	-57.4645
z18	58.2365	-19.2999	-7.1286
x19	-89.6658	-57.1081	12.3445
y19	-37.3360	-70.3906	-47.9650
z19	34.0966	-126.5350	27.9171
x20	-60.2651	-69.0731	-32.3990
y20	-136.7262	63.2377	62.6311
z20	-22.4756	-18.5369	-12.6251
x21	-68.1696	5.7075	-12.0615
y21	82.6899	15.9530	75.8057
z21	31.1307	-56.2893	-29.9178
x22	-19.4327	53.5608	8.5399
y22	30.8165	-81.1972	-14.9510
z22	-15.5202	0.4876	-5.6225
x23	23.6361	83.9629	75.9918
y23	-94.4517	-47.1667	-97.7891
z23	-37.5098	118.1067	23.9523
x24	23.9444	-47.8904	-20.4642
y24	15.1977	15.2482	-19.0182
z24	-43.4036	-49.5781	-101.0810
x25	-29.5581	-141.0001	-84.8693
y25	59.3005	78.4537	55.6047
z25	52.4085	39.8230	-21.3041
x26	-46.3498	112.4445	-2.1644
y26	-8.8447	-48.5095	18.7603
z26	44.9374	9.7284	27.7299
x27	40.9549	154.6327	-26.3042
y27	-50.8877	-49.3945	-1.3889
z27	8.3456	69.0309	112.1432
x28	22.8097	39.1091	92.3115
y28	-37.9736	-12.4977	-20.4036
z28	15.0793	-81.2843	61.7434
x29	61.1210	-73.0658	15.0413
y29	26.5903	26.5372	-2.7924
z29	37.1398	-36.9999	42.6656
x30	94.9741	-198.3709	24.6664
y30	51.3485	-3.1530	2.0730
z30	-9.7448	-6.9791	-25.2615
x31	-100.5737	-43.9362	8.8066
y31	5.1100	23.6690	28.8622
z31	29.7398	32.7862	-23.8641
x32	-42.4413	-0.7757	-72.7938
y32	15.2430	-17.1306	-38.7288
z32	-14.8985	18.3576	-23.9008
x33	50.2332	83.8415	-7.8982
y33	111.7090	32.6708	-22.0827
z33	-66.1813	-19.5898	-64.8983
x34	-11.2422	48.9754	5.8953
y34	58.1214	79.8889	28.7815
z34	-47.1711	8.0497	-34.9074
x35	-81.3905	122.6741	41.7232
y35	-108.8518	142.4040	91.9478
z35	-5.3452	12.0963	-0.4083

x36	-32.6902	-61.9857	-48.5531
y36	-177.3081	-158.9805	-0.1595
z36	4.7798	26.4519	13.7635
x37	49.3126	-37.6205	-26.7736
y37	79.1906	-64.2904	-52.5388
z37	37.1274	16.6250	56.7387
x38	-47.2647	-11.8804	-25.7087
y38	-62.6558	-173.3704	-108.4566
z38	31.3860	22.8574	-31.1929
x39	-4.4394	16.4652	39.4406
y39	-3.5950	9.2797	-19.8622
z39	1.7445	5.1907	-15.0830
x40	46.4807	1.2062	59.7368
y40	119.4121	43.6971	79.1707
z40	-24.2206	-30.0924	-33.5065
x41	18.1601	-59.8163	14.0074
y41	3.6839	1.7354	30.4984
z41	-4.0095	-19.5911	-8.2047
x42	-50.1401	-77.2935	-13.4295
y42	-126.4263	37.5164	34.9024
z42	21.5377	6.8028	20.4292
x43	-22.3778	-22.0911	3.2989
y43	-71.0665	78.4656	29.4107
z43	-0.1554	-10.8859	20.4345
x44	26.5326	5.4612	8.9317
y44	84.3820	76.4139	-6.3769
z44	-12.4993	-31.4858	-8.2515
x45	-52.1867	-36.6915	-58.4116
y45	73.6963	-14.7545	-171.6631
z45	71.3369	-46.9636	-7.4279

New Procrustes fit:
Dataset 'anterior ampula'

10 landmarks in 3 dimensions.
The dataset contains 40 observations, of which 36 are included for analyses.

Average shape:

Lmk.	Axis 1 (x)	Axis 2 (y)	Axis 3 (z)
1	-0.49411640	0.11107828	-0.00205324
2	0.47180122	0.11844285	-0.00361960
3	0.36826646	0.05547351	0.00365462
4	0.26889888	-0.01610676	0.00192914
5	0.16552153	-0.06367307	0.00061711
6	0.06074913	-0.08810482	-0.00142793
7	-0.04525086	-0.08720801	-0.00239204
8	-0.15464262	-0.06534853	-0.00090695
9	-0.26514652	-0.01733822	0.00129633
10	-0.37608082	0.05278477	0.00290257

Procrustes sums of squares: 0.2172701254813195
Tangent sums of squares: 0.2149152560157059

Data matrices in this dataset:
- anterior ampula, raw data
- anterior ampula, centroid size
- anterior ampula, Procrustes coordinates

New Procrustes fit:
Dataset 'anterior ampula'

10 landmarks in 3 dimensions.
The dataset contains 40 observations, of which 36 are included for analyses.

Average shape:

Lmk.	Axis 1 (x)	Axis 2 (y)	Axis 3 (z)
1	-0.49411640	0.11107828	-0.00205324
2	0.47180122	0.11844285	-0.00361960
3	0.36826646	0.05547351	0.00365462
4	0.26889888	-0.01610676	0.00192914
5	0.16552153	-0.06367307	0.00061711
6	0.06074913	-0.08810482	-0.00142793
7	-0.04525086	-0.08720801	-0.00239204
8	-0.15464262	-0.06534853	-0.00090695
9	-0.26514652	-0.01733822	0.00129633
10	-0.37608082	0.05278477	0.00290257

Procrustes sums of squares: 0.2172701254813195
Tangent sums of squares: 0.2149152560157059

Data matrices in this dataset:
- anterior ampula, raw data
- anterior ampula, centroid size
- anterior ampula, Procrustes coordinates

Canonical Variate Analysis: CVA ...

Dataset: anterior ampula
Classification criterion: subfam

Groups		Observations
1.	Ca	9
2.	Ce	13
3.	Di	3
4.	Pr	11

Variation among groups, scaled by the inverse of the within-group variation

	Eigenvalues	% Variance	Cumulative %
1.	10.48287923	70.452	70.452
2.	3.35203135	22.528	92.980
3.	1.04447235	7.020	100.000

Mahalanobis distances among groups:

	Ca	Ce	Di
Ce	2.8171		
Di	10.5719	11.6555	
Pr	3.8926	4.4280	10.4342

P-values from permutation tests (10000 permutation rounds) for Mahalanobis distances among groups:

	Ca	Ce	Di
Ce	0.0012		
Di	0.0039	0.0017	
Pr	<.0001	<.0001	0.0022

Procrustes distances among groups:

	Ca	Ce	Di
Ce	0.0464		
Di	0.0325	0.0380	
Pr	0.0341	0.0221	0.0236

P-values from permutation tests (10000 permutation rounds) for Procrustes distances among groups:

	Ca	Ce	Di
Ce	0.1500		
Di	0.6793	0.5727	
Pr	0.4446	0.7170	0.8816

Canonical coefficients:

	CV1	CV2	CV3
x1	136.4378	66.7096	-46.7897
y1	-60.7923	-112.2248	-17.7606
z1	72.0924	190.7760	-146.8441
x2	-127.4067	-119.0206	-53.8869
y2	-26.5740	66.6783	18.0706
z2	2.8866	-138.8731	-3.3346
x3	-160.1468	210.5495	23.0332
y3	20.5086	144.3325	38.7524
z3	-248.2845	-282.5169	-151.6293
x4	1186.2224	685.1715	188.8673
y4	-40.0404	-294.5795	-100.9111
z4	1089.6471	948.6376	441.0478
x5	-1790.7959	-3046.9602	-664.9564
y5	6.4687	-61.2991	-31.7553
z5	-1067.9007	-634.3717	-134.0690
x6	1532.0911	3801.3464	1259.9726
y6	66.7427	-48.4445	-33.4228
z6	-208.7904	54.2688	-251.3620
x7	-1014.7129	-1383.7552	-1180.9890
y7	413.7180	72.6358	295.7491
z7	48.5301	583.7163	-407.2640
x8	964.1618	212.2976	756.0984
y8	-661.9548	3.7708	-315.6168
z8	642.3103	-302.5592	664.7967
x9	-919.3598	-793.7039	-590.8966
y9	51.5926	318.0464	187.6817
z9	-10.0801	-624.7395	-138.5273
x10	193.5089	367.3654	309.5471
y10	230.3310	-88.9159	-40.7871
z10	-320.4108	205.6618	127.1857

New Procrustes fit:

Dataset 'anterior canal without ampula'

36 landmarks in 3 dimensions.
The dataset contains 40 observations, of which 36 are included for analyses.

Average shape:

Lmk.	Axis 1 (x)	Axis 2 (y)	Axis 3 (z)
1	-0.05517093	0.14780051	0.01993525
2	-0.07343957	0.14808895	0.01379575
3	-0.09105834	0.14168733	0.00886818
4	-0.10631211	0.13193762	0.00466681
5	-0.11958731	0.11997455	0.00108455
6	-0.13148801	0.10586952	-0.00231425
7	-0.14209724	0.08900847	-0.00541739
8	-0.15112015	0.06901763	-0.00817983
9	-0.15792778	0.04606612	-0.01007626
10	-0.16172958	0.02017128	-0.01072910
11	-0.16206838	-0.01196936	-0.00978933
12	-0.15712623	-0.04137664	-0.00784408
13	-0.14757182	-0.06745048	-0.00523455
14	-0.13468299	-0.09050773	-0.00294338
15	-0.11875952	-0.11082624	-0.00081038
16	-0.09910918	-0.12855678	0.00127517
17	-0.07576013	-0.14365075	0.00335718
18	-0.04831773	-0.15555050	0.00562599
19	-0.01697151	-0.16266916	0.00857995
20	0.01811838	-0.16213005	0.00784057
21	0.04976927	-0.15483200	0.00745017
22	0.07776102	-0.14290593	0.00647124
23	0.10210840	-0.12680092	0.00486187
24	0.12360061	-0.10717549	0.00204343
25	0.14116181	-0.08197694	-0.00067316
26	0.15509322	-0.05173614	-0.00350976
27	0.16633421	-0.01715115	-0.00715884
28	0.17261895	0.02219465	-0.01088128
29	0.17360959	0.04191068	-0.01112916
30	0.17127194	0.05974139	-0.01018372
31	0.16554931	0.07536398	-0.00824729
32	0.15654153	0.08867651	-0.00534703
33	0.14452631	0.09983443	-0.00159997
34	0.12949532	0.10906348	0.00301824
35	0.11184532	0.11700783	0.00848635
36	0.09089335	0.12385132	0.01470804

Procrustes sums of squares: 0.2772720697566326
Tangent sums of squares: 0.2742148073435071

Data matrices in this dataset:
- anterior canal without ampula, raw data
- anterior canal without ampula, centroid size
- anterior canal without ampula, Procrustes coordinates

New Procrustes fit:
Dataset 'anterior canal without ampula'

36 landmarks in 3 dimensions.

The dataset contains 40 observations, of which 36 are included for analyses.

Average shape:

Lmk.	Axis 1 (x)	Axis 2 (y)	Axis 3 (z)
1	-0.05517093	0.14780051	0.01993525
2	-0.07343957	0.14808895	0.01379575
3	-0.09105834	0.14168733	0.00886818
4	-0.10631211	0.13193762	0.00466681
5	-0.11958731	0.11997455	0.00108455
6	-0.13148801	0.10586952	-0.00231425
7	-0.14209724	0.08900847	-0.00541739
8	-0.15112015	0.06901763	-0.00817983
9	-0.15792778	0.04606612	-0.01007626
10	-0.16172958	0.02017128	-0.01072910
11	-0.16206838	-0.01196936	-0.00978933
12	-0.15712623	-0.04137664	-0.00784408
13	-0.14757182	-0.06745048	-0.00523455
14	-0.13468299	-0.09050773	-0.00294338
15	-0.11875952	-0.11082624	-0.00081038
16	-0.09910918	-0.12855678	0.00127517
17	-0.07576013	-0.14365075	0.00335718
18	-0.04831773	-0.15555050	0.00562599
19	-0.01697151	-0.16266916	0.00857995
20	0.01811838	-0.16213005	0.00784057
21	0.04976927	-0.15483200	0.00745017
22	0.07776102	-0.14290593	0.00647124
23	0.10210840	-0.12680092	0.00486187
24	0.12360061	-0.10717549	0.00204343
25	0.14116181	-0.08197694	-0.00067316
26	0.15509322	-0.05173614	-0.00350976
27	0.16633421	-0.01715115	-0.00715884
28	0.17261895	0.02219465	-0.01088128
29	0.17360959	0.04191068	-0.01112916
30	0.17127194	0.05974139	-0.01018372
31	0.16554931	0.07536398	-0.00824729
32	0.15654153	0.08867651	-0.00534703
33	0.14452631	0.09983443	-0.00159997
34	0.12949532	0.10906348	0.00301824
35	0.11184532	0.11700783	0.00848635
36	0.09089335	0.12385132	0.01470804

Procrustes sums of squares: 0.2772720697566326
Tangent sums of squares: 0.2742148073435071

Data matrices in this dataset:
- anterior canal without ampula, raw data
- anterior canal without ampula, centroid size
- anterior canal without ampula, Procrustes coordinates

Canonical Variate Analysis: CVA ...anterior canal without ampula

Dataset: anterior canal without ampula
Classification criterion: subfam

Groups   Observations

1.	Ca	9
2.	Ce	13
3.	Di	3
4.	Pr	11

Variation among groups, scaled by the inverse of the within-group variation

	Eigenvalues	% Variance	Cumulative %
1.	12.89297513	71.673	71.673
2.	3.52526733	19.597	91.270
3.	1.57044504	8.730	100.000

Mahalanobis distances among groups:

	Ca	Ce	Di
Ce	6.7863		
Di	5.3458	5.8346	
Pr	5.0881	8.0790	6.3691

P-values from permutation tests (10000 permutation rounds) for Mahalanobis distances among groups:

	Ca	Ce	Di
Ce	<.0001		
Di	0.0029	0.0015	
Pr	<.0001	<.0001	0.0011

Procrustes distances among groups:

	Ca	Ce	Di
Ce	0.0521		
Di	0.0484	0.0769	
Pr	0.0300	0.0415	0.0570

P-values from permutation tests (10000 permutation rounds) for Procrustes distances among groups:

	Ca	Ce	Di
Ce	0.0804		
Di	0.5867	0.1273	
Pr	0.7612	0.2508	0.4347

Canonical coefficients:

	CV1	CV2	CV3
x1	-76.8449	74.9186	-64.6924
y1	73.1612	202.3216	41.8543
z1	57.5555	-69.0286	-101.5315
x2	45.6855	-91.9972	-34.2319
y2	35.1352	-31.7173	55.8862
z2	-55.2085	-52.9095	-36.1535
x3	130.0726	44.7528	-87.1714
y3	-219.8584	-9.1845	140.4773
z3	-119.3652	116.4090	140.1627
x4	7.9765	56.0671	100.1664
y4	62.8772	-66.4047	-159.6285
z4	-116.3689	-84.2839	240.1280
x5	-118.2941	-64.2443	119.7271
y5	192.2592	21.6674	-195.6677

z5	-85.4058	48.7243	-38.3737
x6	-30.7328	-112.2823	9.9987
y6	6.2232	65.7392	21.5381
z6	62.1786	-6.7299	-142.6555
x7	-15.1160	89.0565	-31.4674
y7	-74.4546	-106.2214	106.7808
z7	21.9150	7.2529	-142.7120
x8	54.9850	177.4959	-15.1044
y8	-56.1210	-53.7082	-21.1510
z8	104.9107	81.2571	-76.2692
x9	-73.9921	-111.7469	10.7481
y9	52.8607	26.0552	-3.2623
z9	11.7699	-133.5837	44.3400
x10	-151.1271	-32.3023	67.3546
y10	21.2898	109.7140	3.6004
z10	253.6177	169.1384	71.1799
x11	-65.9696	17.3780	14.8553
y11	31.7401	14.3131	66.2342
z11	19.6700	-126.2769	53.2225
x12	266.2445	3.3547	-228.5610
y12	90.2628	-57.6664	5.7112
z12	62.9703	-115.3543	7.3078
x13	32.6565	94.9479	-11.8272
y13	43.8225	9.9163	-41.0530
z13	-138.4169	135.6819	32.1021
x14	88.6440	-23.1312	-4.4631
y14	51.5107	-20.1006	-64.1573
z14	-22.4321	45.1558	-70.4607
x15	-7.7689	-110.4421	89.5857
y15	-103.9541	-113.4048	10.8221
z15	-148.2825	-26.9973	47.9227
x16	48.0765	4.3703	40.3910
y16	-52.9836	-33.1494	18.6063
z16	-55.2266	-28.9348	-34.4680
x17	2.9680	68.0256	54.9649
y17	-56.5036	43.5929	19.9262
z17	-26.9176	-39.0251	-33.6228
x18	35.4744	56.5014	21.9544
y18	63.7204	99.3258	-12.5436
z18	108.0601	116.9416	-53.9232
x19	-173.9990	-88.3958	17.1060
y19	8.6527	21.7312	56.5669
z19	59.3950	-106.2676	57.1903
x20	10.2482	-53.5186	-67.0699
y20	-184.8566	80.3969	-48.0430
z20	51.9699	115.1275	-69.1365
x21	26.7042	71.8958	-0.5131
y21	-65.8620	-144.7637	-29.3544
z21	-39.3795	-132.1531	11.3042
x22	8.4405	-12.2442	-84.0843
y22	175.9976	-42.8736	122.9380
z22	-47.2997	-53.5115	173.0456
x23	149.7270	-21.3429	0.2269
y23	-25.9345	26.8100	-25.4351
z23	-145.5240	115.5313	127.6507
x24	-48.1781	-24.5229	24.4379
y24	14.2071	-18.7919	-68.4279
z24	50.7335	-100.9804	-121.8347
x25	-242.5181	-99.8854	72.4209
y25	-2.8642	71.4715	10.9621

z25	104.1502	141.9012	-49.6805
x26	-0.5360	386.4589	7.3846
y26	-95.0646	-103.1247	27.7364
z26	-35.3464	-30.9271	11.5122
x27	192.4940	-217.4988	-123.4195
y27	-12.8600	71.6360	-14.6834
z27	63.6399	144.1661	-36.9367
x28	-103.4744	-14.4450	79.3744
y28	-9.5174	-13.7927	39.3653
z28	104.5567	-147.3858	-175.3336
x29	107.2533	-1.2113	82.4286
y29	106.2764	41.6437	-10.2116
z29	-51.9422	-96.0719	-70.8663
x30	-3.7404	-95.2198	-97.1585
y30	67.3347	-23.2463	-22.1241
z30	-54.5831	-98.7546	72.3615
x31	-226.6071	-51.0897	51.7140
y31	-124.4435	-70.5280	-32.7671
z31	72.5412	66.6523	20.4615
x32	8.2643	-15.5233	22.9899
y32	-47.4782	-161.1124	-50.1755
z32	8.9306	46.7397	12.1606
x33	190.9394	96.5532	-77.4832
y33	279.0233	93.0157	-121.1565
z33	-74.0327	8.1700	79.0422
x34	-29.3401	34.2074	43.9728
y34	101.8294	266.8229	134.2439
z34	-68.7845	35.7096	70.4523
x35	-46.4073	115.9535	50.9788
y35	-217.8965	258.6499	164.7197
z35	21.1685	57.3035	50.3333
x36	7.7917	-150.8937	-55.5334
y36	-127.5314	-455.0329	-128.1274
z36	44.7831	-2.6861	-67.9222

New Procrustes fit:
Dataset 'posterior canal'

45 landmarks in 3 dimensions.
The dataset contains 40 observations, of which 36 are included for analyses.

Average shape:

Lmk.	Axis 1 (x)	Axis 2 (y)	Axis 3 (z)
1	0.09669688	0.00334310	0.09036122
2	0.08090621	-0.00096968	0.10490077
3	0.06178644	-0.00237322	0.11620806
4	0.04267607	-0.00292573	0.12454322
5	0.02415159	-0.00380549	0.13061524
6	0.00594489	-0.00440340	0.13475518
7	-0.01216558	-0.00487461	0.13689266
8	-0.03027599	-0.00514255	0.13730229
9	-0.04848184	-0.00487348	0.13566745
10	-0.06658550	-0.00427744	0.13154613
11	-0.08698162	-0.00443940	0.12282711
12	-0.10473797	-0.00410488	0.11123321
13	-0.11994792	-0.00355156	0.09735448
14	-0.13234074	-0.00269673	0.08121843

15	-0.14269886	-0.00208017	0.06345659
16	-0.15096020	-0.00135146	0.04369999
17	-0.15688037	-0.00067018	0.02189952
18	-0.16052627	0.00012839	-0.00205225
19	-0.16101904	0.00131246	-0.02790541
20	-0.15714782	0.00272073	-0.05935628
21	-0.14857862	0.00403790	-0.08670587
22	-0.13616879	0.00515997	-0.10968461
23	-0.12093406	0.00568528	-0.12884255
24	-0.10243238	0.00575593	-0.14397260
25	-0.08092526	0.00509331	-0.15573259
26	-0.05586693	0.00394917	-0.16394234
27	-0.02710273	0.00244560	-0.16783940
28	0.00465165	0.00172449	-0.16582754
29	0.03321942	-0.00381678	-0.15210598
30	0.05811428	-0.00679677	-0.13575752
31	0.07999141	-0.00927555	-0.11980977
32	0.09915481	-0.01149898	-0.10442611
33	0.11556422	-0.01349485	-0.08911392
34	0.12948653	-0.01504160	-0.07310415
35	0.14060731	-0.01548022	-0.05522341
36	0.14716856	-0.01300460	-0.03562271
37	0.14688830	-0.00613312	-0.01960372
38	0.14020824	0.00169039	-0.01119344
39	0.13150347	0.00972962	-0.00338898
40	0.12373480	0.01537815	0.00595297
41	0.11701264	0.01877586	0.01669843
42	0.11150012	0.01904877	0.02946449
43	0.10683532	0.01790583	0.04291412
44	0.10342029	0.01480518	0.05747378
45	0.10153504	0.00839234	0.07422582

Procrustes sums of squares: 0.35301572754402816
Tangent sums of squares: 0.34855973632036596

Data matrices in this dataset:
- posterior canal, raw data
- posterior canal, centroid size
- posterior canal, Procrustes coordinates

New Procrustes fit:
Dataset 'posterior canal'

45 landmarks in 3 dimensions.
The dataset contains 40 observations, of which 36 are included for analyses.

Average shape:

Lmk.	Axis 1 (x)	Axis 2 (y)	Axis 3 (z)
1	0.09669688	0.00334310	0.09036122
2	0.08090621	-0.00096968	0.10490077
3	0.06178644	-0.00237322	0.11620806
4	0.04267607	-0.00292573	0.12454322
5	0.02415159	-0.00380549	0.13061524
6	0.00594489	-0.00440340	0.13475518
7	-0.01216558	-0.00487461	0.13689266
8	-0.03027599	-0.00514255	0.13730229

9	-0.04848184	-0.00487348	0.13566745
10	-0.06658550	-0.00427744	0.13154613
11	-0.08698162	-0.00443940	0.12282711
12	-0.10473797	-0.00410488	0.11123321
13	-0.11994792	-0.00355156	0.09735448
14	-0.13234074	-0.00269673	0.08121843
15	-0.14269886	-0.00208017	0.06345659
16	-0.15096020	-0.00135146	0.04369999
17	-0.15688037	-0.00067018	0.02189952
18	-0.16052627	0.00012839	-0.00205225
19	-0.16101904	0.00131246	-0.02790541
20	-0.15714782	0.00272073	-0.05935628
21	-0.14857862	0.00403790	-0.08670587
22	-0.13616879	0.00515997	-0.10968461
23	-0.12093406	0.00568528	-0.12884255
24	-0.10243238	0.00575593	-0.14397260
25	-0.08092526	0.00509331	-0.15573259
26	-0.05586693	0.00394917	-0.16394234
27	-0.02710273	0.00244560	-0.16783940
28	0.00465165	0.00172449	-0.16582754
29	0.03321942	-0.00381678	-0.15210598
30	0.05811428	-0.00679677	-0.13575752
31	0.07999141	-0.00927555	-0.11980977
32	0.09915481	-0.01149898	-0.10442611
33	0.11556422	-0.01349485	-0.08911392
34	0.12948653	-0.01504160	-0.07310415
35	0.14060731	-0.01548022	-0.05522341
36	0.14716856	-0.01300460	-0.03562271
37	0.14688830	-0.00613312	-0.01960372
38	0.14020824	0.00169039	-0.01119344
39	0.13150347	0.00972962	-0.00338898
40	0.12373480	0.01537815	0.00595297
41	0.11701264	0.01877586	0.01669843
42	0.11150012	0.01904877	0.02946449
43	0.10683532	0.01790583	0.04291412
44	0.10342029	0.01480518	0.05747378
45	0.10153504	0.00839234	0.07422582

Procrustes sums of squares: 0.35301572754402816
Tangent sums of squares: 0.34855973632036596

Data matrices in this dataset:
- posterior canal, raw data
- posterior canal, centroid size
- posterior canal, Procrustes coordinates

Canonical Variate Analysis: CVA ...posterior canal

Dataset: posterior canal
Classification criterion: subfam

Groups		Observations
1.	Ca	9
2.	Ce	13
3.	Di	3
4.	Pr	11

Variation among groups, scaled by the inverse of the within-group variation

	Eigenvalues	% Variance	Cumulative %
1.	9.75788687	66.345	66.345
2.	3.23379478	21.987	88.332
3.	1.71613687	11.668	100.000

Mahalanobis distances among groups:

	Ca	Ce	Di
Ce	7.5257		
Di	7.8513	4.8897	
Pr	5.8480	4.8350	5.4815

P-values from permutation tests (10000 permutation rounds) for Mahalanobis distances among groups:

	Ca	Ce	Di
Ce	<.0001		
Di	0.0027	0.0022	
Pr	<.0001	<.0001	0.0008

Procrustes distances among groups:

	Ca	Ce	Di
Ce	0.0957		
Di	0.0940	0.0745	
Pr	0.0703	0.0560	0.0755

P-values from permutation tests (10000 permutation rounds) for Procrustes distances among groups:

	Ca	Ce	Di
Ce	<.0001		
Di	0.0096	0.1553	
Pr	0.0118	0.0542	0.1649

Canonical coefficients:

	CV1	CV2	CV3
x1	89.5996	49.4040	-86.5881
y1	41.3306	-32.4773	-10.2501
z1	-39.2124	-37.4368	-117.3876
x2	-6.4772	44.9694	75.6356
y2	-77.0313	14.4122	-27.0721
z2	-100.2057	-31.8742	9.6151
x3	-34.9428	48.3190	18.0408
y3	-60.2692	21.9843	74.7992
z3	2.6189	28.2010	49.4471
x4	-50.5821	23.0958	24.6489
y4	-31.6041	-2.9070	-18.9366
z4	114.3124	7.8589	39.3647
x5	-50.8997	-36.7138	-6.6984
y5	29.9585	47.4266	6.8998
z5	-49.0006	-28.6867	39.9204
x6	-15.5215	-22.0651	-0.8851
y6	10.9341	-2.6240	-33.7563
z6	-21.2034	19.9790	57.2412
x7	21.2002	-29.7291	-42.0548

y7	-24.8518	-42.9895	-23.0175
z7	56.0380	88.5477	56.8884
x8	23.8572	-25.9022	-26.5868
y8	-46.6299	-26.5546	15.8041
z8	113.8064	8.3706	-17.7024
x9	88.2689	-27.2758	-8.9742
y9	14.5939	-18.8380	33.6465
z9	-87.8149	6.4148	-93.9935
x10	88.4305	-10.3096	-16.0939
y10	67.5308	-15.2560	11.3440
z10	-75.4395	-14.5044	9.6898
x11	11.5082	18.4943	43.1223
y11	-11.9815	-5.7068	1.5620
z11	-74.0023	-49.8303	-28.3791
x12	-91.0195	34.7225	50.2400
y12	18.8900	18.9604	-25.0149
z12	-38.6687	-28.6321	-5.7038
x13	-77.1450	12.3498	14.9280
y13	44.9162	25.0378	-25.6574
z13	-50.3997	7.8671	5.3578
x14	-84.7351	53.8279	22.2329
y14	-11.6266	72.5358	-34.0030
z14	34.3234	-4.4094	16.9586
x15	-34.0105	45.7606	52.7083
y15	22.8370	56.2292	4.4840
z15	12.2406	19.5829	-25.1465
x16	-110.5044	3.7673	90.9638
y16	-52.4441	24.4168	21.4047
z16	70.6797	26.0841	-36.3693
x17	12.6593	-75.8993	8.3251
y17	-17.2519	-21.9192	-4.5352
z17	22.6156	29.1447	-5.2644
x18	37.3939	-26.3589	-31.6416
y18	-2.9720	-6.1797	38.6919
z18	9.7685	9.1660	1.5155
x19	60.3137	16.4591	-155.3214
y19	31.6625	-8.6773	12.3079
z19	5.8491	-29.8104	-22.4349
x20	72.1326	56.3569	-57.1219
y20	10.6513	-38.9829	27.1283
z20	14.3856	-15.3987	13.7382
x21	-0.1980	-9.3644	-18.9420
y21	33.8543	-37.3197	3.1778
z21	27.3250	-31.6634	30.9071
x22	-31.5209	5.0387	-45.6815
y22	4.2223	0.1009	-10.9191
z22	13.2018	-27.8961	-1.6808
x23	14.0788	-22.7051	-0.0711
y23	13.9968	-15.8046	-23.3930
z23	42.7780	-48.8402	-10.1218
x24	16.4404	-27.5736	52.1304
y24	-48.3882	-11.6654	14.8208
z24	25.5391	-14.0029	51.2458
x25	11.0542	-51.8515	22.7339
y25	-43.5905	-23.4347	-26.0422
z25	3.5993	-10.9105	4.8463
x26	-15.8776	-36.7651	18.9271
y26	-140.8321	23.2656	-23.9266
z26	-211.4031	50.8263	-4.6912
x27	46.8263	-7.4882	17.1378

y27	103.5518	24.8507	-28.2725
z27	99.9982	87.4555	-45.5051
x28	-35.8690	12.7113	-3.1643
y28	73.9681	60.3066	34.4283
z28	155.7973	86.2015	12.3356
x29	37.0233	58.3335	-1.1881
y29	-120.9651	-77.8288	34.7548
z29	-230.0100	-147.6199	33.5076
x30	-30.2750	59.7832	26.7218
y30	-28.4768	-19.8516	-18.8637
z30	-66.7274	-61.1019	-29.4430
x31	-25.6659	12.0843	0.6409
y31	93.3970	76.6155	-37.8668
z31	69.8557	99.6592	18.5150
x32	-66.4560	9.2949	-40.2908
y32	90.4338	17.6782	55.3790
z32	90.0566	60.1201	94.0135
x33	-3.0776	-36.9239	-42.8492
y33	7.1851	21.2972	47.7567
z33	23.9881	46.1251	46.5414
x34	-0.1261	34.6733	18.4714
y34	75.1294	-67.0612	-38.4442
z34	8.7892	-68.3948	-63.7500
x35	90.6682	-39.8068	71.1844
y35	-94.1629	-8.5040	-17.5583
z35	17.3014	-41.1733	-70.8074
x36	-22.8626	-81.7107	59.8731
y36	-72.8231	32.3340	-2.3537
z36	46.4302	-75.2684	2.7938
x37	-63.9192	99.9196	-15.7824
y37	127.9780	100.8928	75.8669
z37	-40.7674	-0.4458	-134.4045
x38	8.4118	-35.5669	-39.0337
y38	-46.4996	31.9711	-29.4043
z38	8.5250	16.4536	-25.4062
x39	15.2115	61.2593	43.4416
y39	-11.1072	-34.4282	-54.1435
z39	6.9289	49.7228	41.1658
x40	-29.6853	5.3535	63.2331
y40	-13.0552	-11.9691	-37.1529
z40	-26.0531	13.2956	58.5657
x41	-23.3786	-8.9785	44.7090
y41	-23.3431	-61.4117	-31.0705
z41	-33.0839	29.6077	63.5314
x42	14.2746	-35.9262	30.2341
y42	-7.5062	-14.4678	-5.6125
z42	-46.3903	-28.1678	16.7023
x43	58.4453	-47.5073	-42.4516
y43	53.2301	-41.9994	20.4603
z43	5.0897	-44.8372	2.6746
x44	74.2403	-49.5094	-65.2873
y44	15.6338	-57.4815	-42.1794
z44	47.4029	12.1967	16.6918
x45	12.7109	-20.0466	-123.5766
y45	1.5272	36.0243	94.7292
z45	41.1375	38.0242	-55.5832

New Procrustes fit:
Dataset 'posterior ampula'

10 landmarks in 3 dimensions.
The dataset contains 40 observations, of which 36 are included for analyses.

Average shape:

Lmk.	Axis 1 (x)	Axis 2 (y)	Axis 3 (z)
1	-0.51466351	-0.10591980	-0.00545286
2	0.44589319	-0.12077180	-0.00540653
3	0.36041976	-0.05855315	0.00525548
4	0.27304380	0.01044503	0.00265921
5	0.17826881	0.05676174	0.00054167
6	0.07758205	0.08121823	-0.00097313
7	-0.03218949	0.09473114	-0.00442837
8	-0.14424490	0.07497755	-0.00262408
9	-0.25986173	0.01927122	0.00361083
10	-0.38424796	-0.05216015	0.00681779

Procrustes sums of squares: 0.49118702542282655
Tangent sums of squares: 0.4568706376358257

Data matrices in this dataset:
- posterior ampula, raw data
- posterior ampula, centroid size
- posterior ampula, Procrustes coordinates

New Procrustes fit:
Dataset 'posterior ampula'

10 landmarks in 3 dimensions.
The dataset contains 40 observations, of which 36 are included for analyses.

Average shape:

Lmk.	Axis 1 (x)	Axis 2 (y)	Axis 3 (z)
1	-0.51466351	-0.10591980	-0.00545286
2	0.44589319	-0.12077180	-0.00540653
3	0.36041976	-0.05855315	0.00525548
4	0.27304380	0.01044503	0.00265921
5	0.17826881	0.05676174	0.00054167
6	0.07758205	0.08121823	-0.00097313
7	-0.03218949	0.09473114	-0.00442837
8	-0.14424490	0.07497755	-0.00262408
9	-0.25986173	0.01927122	0.00361083
10	-0.38424796	-0.05216015	0.00681779

Procrustes sums of squares: 0.49118702542282655
Tangent sums of squares: 0.4568706376358257

Data matrices in this dataset:
- posterior ampula, raw data
- posterior ampula, centroid size
- posterior ampula, Procrustes coordinates

Canonical Variate Analysis: CVA ...posterior ampulla

Dataset: posterior ampula
Classification criterion: subfam

Groups		Observations
1.	Ca	9
2.	Ce	13
3.	Di	3
4.	Pr	11

Variation among groups, scaled by the inverse of the within-group variation

	Eigenvalues	% Variance	Cumulative %
1.	4.53050777	74.022	74.022
2.	1.01691835	16.615	90.637
3.	0.57305677	9.363	100.000
4.	0.00000000	0.000	100.000

Mahalanobis distances among groups:

	Ca	Ce	Di
Ce	4.2391		
Di	5.1789	3.0215	
Pr	5.1854	2.5595	2.8570

P-values from permutation tests (10000 permutation rounds) for Mahalanobis distances among groups:

	Ca	Ce	Di
Ce	0.0001		
Di	0.0043	0.6589	
Pr	<.0001	0.0022	0.6887

Procrustes distances among groups:

	Ca	Ce	Di
Ce	0.0817		
Di	0.0141	0.0740	
Pr	0.0511	0.0532	0.0414

P-values from permutation tests (10000 permutation rounds) for Procrustes distances among groups:

	Ca	Ce	Di
Ce	0.0804		
Di	0.9999	0.4587	
Pr	0.1782	0.2712	0.4799

Canonical coefficients:

	CV1	CV2	CV3	CV4
x1	134.9799	-38.6000	-138.7847	13.1468
y1	-113.0174	-7.9753	-27.8958	24.2213
z1	-131.6587	-56.8744	14.7019	24.7979
x2	-107.4533	166.2981	69.2473	-20.0435
y2	154.3917	8.3081	-10.9269	-22.9621
z2	242.9219	-26.1554	-27.7510	-27.4179

x3	588.6564	140.8781	-360.7704	-39.0871
y3	-230.3917	-38.5837	167.2152	6.0820
z3	-222.1253	143.7996	87.3612	-5.0970
x4	-2335.7538	-534.7121	1059.4926	220.4138
y4	245.7843	-58.0748	-242.7797	21.3555
z4	84.1812	-541.9054	91.7530	49.3221
x5	3994.9772	-93.1882	-1123.1639	-334.1335
y5	-501.3876	165.4996	117.4030	25.9390
z5	-668.0730	681.6838	-298.3169	67.3998
x6	-3216.2143	380.8448	246.7852	389.3056
y6	26.8618	28.3177	-119.7783	17.1086
z6	626.9272	-150.3634	-39.1397	-67.0370
x7	1960.9418	-196.3021	-163.4755	-236.0772
y7	739.4752	-113.1363	63.4320	-107.4667
z7	-88.8402	-66.8200	373.5918	-56.7731
x8	-1288.8472	-166.1578	196.4053	200.7747
y8	-438.6612	-98.0908	-19.8553	84.2613
z8	50.8627	-215.3523	-260.4532	76.4930
x9	421.5257	718.6734	232.9812	-218.5314
y9	-62.1775	116.3826	94.2543	-26.3714
z9	-49.9589	193.9897	143.3925	-49.7737
x10	-152.8124	-377.7343	-18.7169	81.6840
y10	179.1226	-2.6470	-21.0686	-22.9552
z10	155.7630	37.9978	-85.1395	-12.4578

New Procrustes fit:
Dataset 'posterior canal without ampulla'

37 landmarks in 3 dimensions.
The dataset contains 40 observations, of which 36 are included for analyses.

Average shape:

Lmk.	Axis 1 (x)	Axis 2 (y)	Axis 3 (z)
1	-0.05323346	0.01271376	0.15922296
2	-0.07386227	0.00825342	0.14867390
3	-0.09242437	0.00620618	0.13341080
4	-0.10794954	0.00498584	0.11703373
5	-0.12095560	0.00335902	0.10042747
6	-0.13186471	0.00197422	0.08342333
7	-0.14069146	0.00062155	0.06577090
8	-0.14775694	-0.00056495	0.04747416
9	-0.15277436	-0.00129250	0.02830124
10	-0.15521942	-0.00181816	0.00830485
11	-0.15381891	-0.00295174	-0.01568158
12	-0.14851723	-0.00349531	-0.03805842
13	-0.13995110	-0.00388606	-0.05867989
14	-0.12804826	-0.00402477	-0.07727167
15	-0.11373529	-0.00395448	-0.09437698
16	-0.09662386	-0.00383525	-0.11007812
17	-0.07656860	-0.00357439	-0.12413962
18	-0.05348164	-0.00289377	-0.13668156
19	-0.02729662	-0.00180185	-0.14672356
20	0.00622468	-0.00012166	-0.15435992
21	0.03727837	0.00152362	-0.15569765
22	0.06528621	0.00322051	-0.15150847
23	0.09043825	0.00436991	-0.14301294
24	0.11268539	0.00522065	-0.12968545

25	0.13260423	0.00535702	-0.11202103
26	0.15020053	0.00498347	-0.08941925
27	0.16475745	0.00446520	-0.06144624
28	0.17437189	0.00429787	-0.02828418
29	0.17089996	-0.00030768	0.00610220
30	0.16338127	-0.00238492	0.03759767
31	0.15514856	-0.00402873	0.06584376
32	0.14648410	-0.00555761	0.09110223
33	0.13687376	-0.00680219	0.11351648
34	0.12562919	-0.00768300	0.13362682
35	0.11143570	-0.00761403	0.15151793
36	0.09378768	-0.00485762	0.16525502
37	0.07728643	0.00189844	0.17052109

Procrustes sums of squares: 0.30653190701893734
Tangent sums of squares: 0.30325025861540666

Data matrices in this dataset:
- posterior canal without ampulla, raw data
- posterior canal without ampulla, centroid size
- posterior canal without ampulla, Procrustes coordinates

New Procrustes fit:
Dataset 'posterior canal without ampulla'

37 landmarks in 3 dimensions.
The dataset contains 40 observations, of which 36 are included for analyses.

Average shape:

Lmk.	Axis 1 (x)	Axis 2 (y)	Axis 3 (z)
1	-0.05323346	0.01271376	0.15922296
2	-0.07386227	0.00825342	0.14867390
3	-0.09242437	0.00620618	0.13341080
4	-0.10794954	0.00498584	0.11703373
5	-0.12095560	0.00335902	0.10042747
6	-0.13186471	0.00197422	0.08342333
7	-0.14069146	0.00062155	0.06577090
8	-0.14775694	-0.00056495	0.04747416
9	-0.15277436	-0.00129250	0.02830124
10	-0.15521942	-0.00181816	0.00830485
11	-0.15381891	-0.00295174	-0.01568158
12	-0.14851723	-0.00349531	-0.03805842
13	-0.13995110	-0.00388606	-0.05867989
14	-0.12804826	-0.00402477	-0.07727167
15	-0.11373529	-0.00395448	-0.09437698
16	-0.09662386	-0.00383525	-0.11007812
17	-0.07656860	-0.00357439	-0.12413962
18	-0.05348164	-0.00289377	-0.13668156
19	-0.02729662	-0.00180185	-0.14672356
20	0.00622468	-0.00012166	-0.15435992
21	0.03727837	0.00152362	-0.15569765
22	0.06528621	0.00322051	-0.15150847
23	0.09043825	0.00436991	-0.14301294
24	0.11268539	0.00522065	-0.12968545
25	0.13260423	0.00535702	-0.11202103
26	0.15020053	0.00498347	-0.08941925

27	0.16475745	0.00446520	-0.06144624
28	0.17437189	0.00429787	-0.02828418
29	0.17089996	-0.00030768	0.00610220
30	0.16338127	-0.00238492	0.03759767
31	0.15514856	-0.00402873	0.06584376
32	0.14648410	-0.00555761	0.09110223
33	0.13687376	-0.00680219	0.11351648
34	0.12562919	-0.00768300	0.13362682
35	0.11143570	-0.00761403	0.15151793
36	0.09378768	-0.00485762	0.16525502
37	0.07728643	0.00189844	0.17052109

Procrustes sums of squares: 0.30653190701893734
Tangent sums of squares: 0.30325025861540666

Data matrices in this dataset:
- posterior canal without ampulla, raw data
- posterior canal without ampulla, centroid size
- posterior canal without ampulla, Procrustes coordinates

Canonical Variate Analysis: CVA ...posterior canal without ampulla

Dataset: posterior canal without ampulla
Classification criterion: subfam

Groups		Observations
1.	Ca	9
2.	Ce	13
3.	Di	3
4.	Pr	11

Variation among groups, scaled by the inverse of the within-group variation

	Eigenvalues	% Variance	Cumulative %
1.	13.20189656	68.722	68.722
2.	4.26937628	22.224	90.946
3.	1.73929088	9.054	100.000

Mahalanobis distances among groups:

	Ca	Ce	Di
Ce	8.7334		
Di	8.7210	4.9254	
Pr	7.2714	5.2068	5.7621

P-values from permutation tests (10000 permutation rounds) for Mahalanobis distances among groups:

	Ca	Ce	Di
Ce	<.0001		
Di	0.0045	0.0014	
Pr	<.0001	<.0001	0.0023

Procrustes distances among groups:

	Ca	Ce	Di

Ce	0.0793		
Di	0.0857	0.0765	
Pr	0.0611	0.0454	0.0760

P-values from permutation tests (10000 permutation rounds) for Procrustes distances among groups:

	Ca	Ce	Di
Ce	0.0009		
Di	0.0188	0.1056	
Pr	0.0423	0.1661	0.1626

Canonical coefficients:

	CV1	CV2	CV3
x1	-9.2666	38.9056	52.8944
y1	20.0712	77.5629	-118.5334
z1	-25.0879	52.3862	-112.9663
x2	-54.1955	51.1800	74.0410
y2	53.5845	58.0374	62.7348
z2	-112.1352	-134.5574	-12.3821
x3	-98.8727	9.2889	-37.9802
y3	-111.1534	-100.5684	142.8974
z3	45.3199	50.7722	-13.6731
x4	66.7838	-142.8199	-138.2231
y4	108.9362	35.2645	25.0355
z4	-27.1185	19.5522	19.2857
x5	33.0536	-2.7240	-10.7441
y5	93.1866	-15.3101	28.1426
z5	28.9095	-43.8328	-14.1636
x6	13.5033	7.2611	-11.4326
y6	104.5134	66.5128	-32.7634
z6	45.0341	-23.4113	23.0449
x7	10.8482	110.8576	-85.2407
y7	54.8879	55.9268	-79.9821
z7	34.6030	3.7458	60.5787
x8	15.4539	-82.2311	-27.4401
y8	19.7972	-117.2878	-17.9780
z8	19.5741	90.0526	25.2811
x9	-219.9313	170.5681	91.0788
y9	-104.4415	26.1887	-27.5290
z9	-0.1223	19.9572	17.3250
x10	105.2719	9.6982	55.3585
y10	-184.3789	57.3465	-18.7008
z10	-71.9844	5.6078	-6.2845
x11	61.6995	-211.8068	78.2446
y11	-80.4663	-23.8317	61.3008
z11	-1.7076	-87.4580	7.8697
x12	2.4504	-100.5550	-25.9552
y12	-84.2722	8.7204	7.3243
z12	52.9866	-148.6374	-38.0684
x13	-29.1000	115.9475	-33.4292
y13	-48.7224	22.5571	-34.3416
z13	77.4851	11.1506	-59.0844
x14	141.3334	12.0153	45.7560
y14	71.0911	-80.4323	-16.2859
z14	101.1404	23.5332	27.9965
x15	-24.0845	88.2462	6.8249
y15	106.6910	-30.4368	-69.9009
z15	34.9074	102.3978	2.4874

x16	-50.6639	-5.3792	33.0796
y16	102.0330	-77.4210	-20.9240
z16	-20.1821	78.2376	64.4336
x17	-61.8751	33.1025	12.8577
y17	70.6477	-64.8539	31.3249
z17	-68.0055	69.1962	60.5535
x18	12.0916	-10.9176	11.4266
y18	-13.3823	46.6952	131.8616
z18	8.9250	-9.8069	37.5476
x19	65.6356	-90.5270	-30.0208
y19	-121.7872	108.0702	56.8250
z19	-56.7603	-85.4559	-79.8261
x20	17.7609	20.4142	-42.6662
y20	-17.3344	-22.4769	11.8637
z20	127.4120	-175.0685	-127.3646
x21	0.0425	45.9904	-33.7074
y21	-71.8328	36.7476	-29.3487
z21	-107.2934	55.2689	-106.3188
x22	57.4686	-16.0624	-30.8238
y22	33.0295	-14.9164	3.5496
z22	-139.9662	80.7716	9.9351
x23	-14.9879	9.0989	-35.4072
y23	80.5973	-58.6066	-53.9354
z23	3.0657	31.9093	25.5623
x24	53.1950	-80.9047	-24.8569
y24	-23.4460	80.2711	-36.0719
z24	-47.6091	59.3238	56.9913
x25	39.4741	-111.0864	40.1197
y25	24.1664	80.8502	-91.8014
z25	26.3258	31.2904	31.4710
x26	-151.0431	258.0194	117.6743
y26	67.0961	-35.0657	-6.4525
z26	122.3524	-201.9228	37.2139
x27	-5.5032	82.9194	-29.6451
y27	-19.1915	7.6494	94.8285
z27	-28.6946	24.8475	67.3016
x28	177.2390	-260.4428	39.9049
y28	-105.6602	41.4631	-44.8035
z28	-72.8873	1.2597	0.9582
x29	-203.5536	-4.3036	-28.8722
y29	114.9333	-31.3550	36.6246
z29	-8.0724	19.4223	-36.0863
x30	-96.4508	117.1120	-102.3320
y30	39.3865	-144.9950	46.8072
z30	40.3917	-4.4112	-52.9097
x31	-15.7219	24.9223	-64.2349
y31	-3.1903	25.6938	30.5378
z31	32.4987	45.6801	-41.0043
x32	160.4763	-62.5675	-82.4297
y32	-134.4927	-9.2497	36.3791
z32	116.3409	-55.1991	-59.6888
x33	81.3672	-80.0815	94.3162
y33	-4.0955	-49.3607	-33.7657
z33	9.2803	-63.4029	49.5840
x34	-20.2286	124.2172	73.4285
y34	-20.1896	-8.1672	83.1287
z34	-46.8990	97.1695	56.6086
x35	16.4131	-214.4441	107.0472
y35	-77.3452	296.9168	-153.5615
z35	-101.2225	-91.1962	126.6350

x36	24.6766	49.0381	-56.6792
y36	129.3063	-197.3771	-61.6164
z36	-114.0289	94.0623	12.5173
x37	-100.7597	98.0508	-1.9321
y37	-68.5727	-50.7623	57.1299
z37	123.2247	56.7654	-61.3610

New Procrustes fit:
Dataset 'clean (sans Hetero-Capreo)'

686 landmarks in 3 dimensions.
The dataset contains 40 observations, of which 38 are included for analyses.

Average shape:

Lmk.	Axis 1 (x)	Axis 2 (y)	Axis 3 (z)
1	-0.04015670	0.02593798	-0.00845672
2	-0.02476392	-0.01007103	-0.02618987
3	-0.02626692	-0.01162506	-0.02724689
4	-0.02819962	-0.01246964	-0.02841702
5	-0.03022306	-0.01282559	-0.02946582
6	-0.03227961	-0.01281649	-0.03042172
7	-0.03442059	-0.01253426	-0.03129073
8	-0.03669808	-0.01183152	-0.03212023
9	-0.03912105	-0.01077862	-0.03286784
10	-0.04159632	-0.00921124	-0.03352705
11	-0.04406628	-0.00712670	-0.03405689
12	-0.04668711	-0.00425341	-0.03449166
13	-0.04867697	-0.00117586	-0.03451283
14	-0.05015568	0.00188291	-0.03419151
15	-0.05114250	0.00488845	-0.03349081
16	-0.05176587	0.00784733	-0.03251355
17	-0.05196950	0.01080020	-0.03118272
18	-0.05173871	0.01375225	-0.02950635
19	-0.05099392	0.01670781	-0.02747092
20	-0.04957142	0.01952307	-0.02511990
21	-0.04750105	0.02171347	-0.02203332
22	-0.04504397	0.02311805	-0.01919880
23	-0.04243218	0.02382089	-0.01659867
24	-0.03974989	0.02389346	-0.01420899
25	-0.03702774	0.02339656	-0.01193409
26	-0.03405414	0.02222149	-0.00992008
27	-0.03088337	0.02027946	-0.00821327
28	-0.02757249	0.01772410	-0.00664596
29	-0.02415190	0.01447721	-0.00539788
30	-0.02250142	0.01281686	-0.00507966
31	-0.02111189	0.01116378	-0.00515655
32	-0.02005899	0.00953286	-0.00565176
33	-0.01929888	0.00793579	-0.00651254
34	-0.01881416	0.00635803	-0.00772961
35	-0.01859105	0.00478942	-0.00929247
36	-0.01858450	0.00321670	-0.01118692
37	-0.01879041	0.00155497	-0.01342025
38	-0.01946415	0.00013769	-0.01606233
39	-0.02049211	-0.00025962	-0.01764203
40	-0.02167348	-0.00052893	-0.01916901
41	-0.02266505	-0.00108738	-0.02057403
42	-0.02345237	-0.00193061	-0.02182930

43	-0.02402068	-0.00306450	-0.02293494
44	-0.02441903	-0.00447180	-0.02392827
45	-0.02456592	-0.00619131	-0.02475623
46	-0.02454863	-0.00816005	-0.02541649
47	-0.01960736	-0.01438050	-0.02641857
48	-0.02204888	-0.01543629	-0.02774931
49	-0.02453272	-0.01614826	-0.02896659
50	-0.02701245	-0.01651534	-0.03004764
51	-0.02945024	-0.01659118	-0.03101951
52	-0.03181768	-0.01640470	-0.03192399
53	-0.03416528	-0.01594144	-0.03271965
54	-0.03647314	-0.01522324	-0.03345538
55	-0.03875581	-0.01425710	-0.03414087
56	-0.04099450	-0.01295831	-0.03469073
57	-0.04241781	-0.01069179	-0.03453319
58	-0.04497796	-0.00979933	-0.03563165
59	-0.04668740	-0.00806039	-0.03589321
60	-0.04825654	-0.00619646	-0.03606147
61	-0.04969084	-0.00424916	-0.03615350
62	-0.05092167	-0.00215657	-0.03606679
63	-0.05196678	0.00004962	-0.03586059
64	-0.05282127	0.00238977	-0.03546921
65	-0.05354483	0.00481975	-0.03499596
66	-0.05402972	0.00701650	-0.03432714
67	-0.05431107	0.00914369	-0.03356116
68	-0.05441684	0.01121313	-0.03269626
69	-0.05432902	0.01321736	-0.03170872
70	-0.05406073	0.01515314	-0.03062875
71	-0.05367277	0.01708328	-0.02948503
72	-0.05302038	0.01889092	-0.02815886
73	-0.05218182	0.02064297	-0.02674539
74	-0.05115843	0.02231282	-0.02522553
75	-0.04981330	0.02371867	-0.02372914
76	-0.04835928	0.02494860	-0.02212344
77	-0.04680382	0.02596777	-0.02039769
78	-0.04514100	0.02677747	-0.01853765
79	-0.04337791	0.02735728	-0.01656341
80	-0.04149717	0.02771639	-0.01445633
81	-0.03946969	0.02784753	-0.01213435
82	-0.03741468	0.02789106	-0.00982218
83	-0.03523559	0.02805948	-0.00743223
84	-0.02477594	0.00515966	0.02167881
85	-0.02667946	0.00365020	0.02302310
86	-0.02898491	0.00268501	0.02431767
87	-0.03130719	0.00203712	0.02538368
88	-0.03356387	0.00152194	0.02620037
89	-0.03579564	0.00118314	0.02683760
90	-0.03802668	0.00101244	0.02728254
91	-0.04026570	0.00098488	0.02757703
92	-0.04252216	0.00118280	0.02770383
93	-0.04477499	0.00157391	0.02762830
94	-0.04741032	0.00224302	0.02710979
95	-0.04974834	0.00317128	0.02630469
96	-0.05178934	0.00428551	0.02523210
97	-0.05348142	0.00562838	0.02390567
98	-0.05495583	0.00705598	0.02237315
99	-0.05619463	0.00865512	0.02061346
100	-0.05717890	0.01039623	0.01860222
101	-0.05787606	0.01234189	0.01635439
102	-0.05821280	0.01446245	0.01389575

103	-0.05808321	0.01702058	0.01083199
104	-0.05735072	0.01923913	0.00806779
105	-0.05606466	0.02113759	0.00568255
106	-0.05437557	0.02266562	0.00359529
107	-0.05226534	0.02383416	0.00179258
108	-0.04977547	0.02465709	0.00014734
109	-0.04677307	0.02516301	-0.00116685
110	-0.04327802	0.02528764	-0.00216054
111	-0.03931958	0.02507520	-0.00251827
112	-0.03564777	0.02340592	-0.00202380
113	-0.03236840	0.02180983	-0.00103188
114	-0.02945539	0.02031951	0.00008542
115	-0.02690895	0.01887903	0.00116209
116	-0.02470900	0.01746644	0.00228332
117	-0.02279467	0.01605921	0.00355245
118	-0.02119791	0.01463613	0.00513867
119	-0.02010403	0.01340482	0.00718804
120	-0.01989024	0.01286376	0.00933641
121	-0.02058184	0.01294226	0.01082177
122	-0.02153250	0.01310446	0.01228820
123	-0.02238107	0.01292859	0.01373176
124	-0.02309489	0.01242012	0.01515166
125	-0.02360777	0.01143711	0.01652243
126	-0.02400803	0.01025056	0.01783850
127	-0.02426265	0.00880344	0.01910673
128	-0.02431528	0.00688125	0.02033653
129	-0.01889503	0.00327235	0.02390352
130	-0.02108746	0.00240664	0.02516064
131	-0.02346161	0.00167412	0.02609997
132	-0.02580734	0.00103405	0.02696211
133	-0.02813037	0.00047845	0.02772358
134	-0.03042699	0.00001696	0.02840591
135	-0.03272051	-0.00035765	0.02901302
136	-0.03501764	-0.00063926	0.02954263
137	-0.03732397	-0.00082874	0.02993511
138	-0.03961758	-0.00091649	0.03021253
139	-0.04196965	-0.00074942	0.03031954
140	-0.04420176	-0.00046849	0.03032667
141	-0.04630257	-0.00002757	0.03005716
142	-0.04828369	0.00050053	0.02963036
143	-0.05013024	0.00115204	0.02900365
144	-0.05184762	0.00195760	0.02818573
145	-0.05347120	0.00288054	0.02721714
146	-0.05496428	0.00393371	0.02604724
147	-0.05633605	0.00511946	0.02471032
148	-0.05777461	0.00666591	0.02300903
149	-0.05892263	0.00826219	0.02122839
150	-0.05981535	0.00989932	0.01938351
151	-0.06048348	0.01157775	0.01750122
152	-0.06091432	0.01330114	0.01556527
153	-0.06108035	0.01503302	0.01353685
154	-0.06097237	0.01678589	0.01143240
155	-0.06057152	0.01856374	0.00924079
156	-0.05982914	0.02033459	0.00698968
157	-0.05848371	0.02242092	0.00467906
158	-0.05672703	0.02414115	0.00257226
159	-0.05465168	0.02557969	0.00066721
160	-0.05232626	0.02679122	-0.00114569
161	-0.04960567	0.02764264	-0.00274143
162	-0.04655537	0.02821339	-0.00417305

163	-0.04312622	0.02843883	-0.00542518
164	-0.03930845	0.02848531	-0.00654985
165	-0.03523559	0.02805948	-0.00743223
166	-0.02496489	-0.01805460	-0.01132397
167	-0.02592438	-0.01933476	-0.01117929
168	-0.02716619	-0.02038167	-0.01054512
169	-0.02837424	-0.02127793	-0.00970066
170	-0.02954245	-0.02209376	-0.00865870
171	-0.03074758	-0.02277134	-0.00738141
172	-0.03195638	-0.02342018	-0.00586088
173	-0.03317998	-0.02396389	-0.00408804
174	-0.03438779	-0.02441348	-0.00208412
175	-0.03566173	-0.02464625	0.00016393
176	-0.03656015	-0.02469563	0.00178518
177	-0.03739701	-0.02460601	0.00338818
178	-0.03824865	-0.02304083	0.00477600
179	-0.03886265	-0.02398645	0.00647525
180	-0.03947632	-0.02348427	0.00796298
181	-0.04002373	-0.02284705	0.00942900
182	-0.04048355	-0.02208153	0.01086567
183	-0.04088162	-0.02118264	0.01227667
184	-0.04120603	-0.02013026	0.01364965
185	-0.04143593	-0.01879463	0.01520326
186	-0.04148637	-0.01741076	0.01656042
187	-0.04137595	-0.01604560	0.01781202
188	-0.04103663	-0.01460940	0.01869504
189	-0.04054675	-0.01316877	0.01951295
190	-0.03985906	-0.01168370	0.02003926
191	-0.03896869	-0.01015188	0.02034262
192	-0.03789383	-0.00860223	0.02046197
193	-0.03662898	-0.00700869	0.02034377
194	-0.03465355	-0.00491619	0.01997027
195	-0.03279608	-0.00303630	0.01929913
196	-0.03103622	-0.00144767	0.01830015
197	-0.02938643	-0.00007050	0.01707897
198	-0.02786649	0.00111032	0.01564516
199	-0.02645018	0.00211953	0.01397142
200	-0.02513867	0.00281140	0.01201112
201	-0.02386069	0.00340751	0.00984303
202	-0.02278734	0.00371111	0.00742135
203	-0.02209738	0.00349666	0.00546869
204	-0.02160847	0.00297949	0.00353285
205	-0.02122081	0.00233096	0.00173799
206	-0.02085223	0.00154031	0.00007832
207	-0.02046935	0.00058380	-0.00146692
208	-0.02012127	-0.00057164	-0.00296380
209	-0.01983958	-0.00192065	-0.00439651
210	-0.01978064	-0.00346584	-0.00574300
211	-0.02020233	-0.00508736	-0.00669146
212	-0.02098381	-0.00656011	-0.00676037
213	-0.02192738	-0.00802093	-0.00674191
214	-0.02282612	-0.00947637	-0.00684990
215	-0.02351324	-0.01092722	-0.00717728
216	-0.02398950	-0.01235038	-0.00772773
217	-0.02427858	-0.01377807	-0.00850117
218	-0.02441595	-0.01523343	-0.00948889
219	-0.02448716	-0.01673363	-0.01060638
220	-0.02290777	-0.01980254	-0.01636919
221	-0.02433361	-0.02096990	-0.01564363
222	-0.02570996	-0.02202564	-0.01475375

223	-0.02703029	-0.02299575	-0.01370344
224	-0.02831085	-0.02387562	-0.01253458
225	-0.02952183	-0.02464749	-0.01121050
226	-0.03072539	-0.02533434	-0.00979763
227	-0.03187897	-0.02595757	-0.00826391
228	-0.03298503	-0.02649765	-0.00659415
229	-0.03407608	-0.02698707	-0.00481654
230	-0.03514054	-0.02733035	-0.00290871
231	-0.03619466	-0.02757422	-0.00106559
232	-0.03718010	-0.02763348	0.00076927
233	-0.03812302	-0.02755430	0.00256218
234	-0.03896421	-0.02728407	0.00435825
235	-0.03977037	-0.02685999	0.00611933
236	-0.04049843	-0.02623688	0.00787598
237	-0.04118291	-0.02548904	0.00959970
238	-0.04179464	-0.02454624	0.01130750
239	-0.04241243	-0.02335866	0.01317271
240	-0.04292097	-0.02213777	0.01487997
241	-0.04324652	-0.02085758	0.01642142
242	-0.04338079	-0.01950165	0.01782416
243	-0.04334974	-0.01810895	0.01905223
244	-0.04316683	-0.01666018	0.02014764
245	-0.04283926	-0.01518146	0.02109055
246	-0.04230123	-0.01362706	0.02185729
247	-0.04163057	-0.01204044	0.02248830
248	-0.04059736	-0.01012738	0.02305072
249	-0.03940631	-0.00825323	0.02330043
250	-0.03808881	-0.00640483	0.02326961
251	-0.03663352	-0.00459561	0.02295796
252	-0.03507293	-0.00281705	0.02238046
253	-0.03345665	-0.00095390	0.02164064
254	-0.03149632	0.00062089	0.02038198
255	-0.02944065	0.00224194	0.01892444
256	-0.02753055	0.00402539	0.01732380
257	-0.01429827	-0.00893286	0.00093271
258	-0.01372726	-0.00920041	0.00158532
259	-0.01306674	-0.00948082	0.00212470
260	-0.01228071	-0.00966956	0.00249277
261	-0.01144299	-0.00981563	0.00276614
262	-0.01063236	-0.01006361	0.00288620
263	-0.00970797	-0.01035150	0.00285430
264	-0.00877275	-0.01067514	0.00278465
265	-0.00762206	-0.01089249	0.00303598
266	-0.00666221	-0.01136580	0.00209851
267	-0.00542303	-0.01144259	0.00163357
268	-0.00433062	-0.01159748	0.00114821
269	-0.00343145	-0.01169562	0.00057288
270	-0.00267372	-0.01180173	-0.00007893
271	-0.00207731	-0.01196340	-0.00079620
272	-0.00160879	-0.01211669	-0.00162883
273	-0.00127133	-0.01216923	-0.00257451
274	-0.00113834	-0.01221378	-0.00359921
275	-0.00120949	-0.01220237	-0.00467333
276	-0.00190997	-0.01247593	-0.00549738
277	-0.00272644	-0.01259365	-0.00615543
278	-0.00357449	-0.01264569	-0.00665181
279	-0.00447755	-0.01268007	-0.00694757
280	-0.00541294	-0.01266919	-0.00708778
281	-0.00641999	-0.01261078	-0.00707616
282	-0.00743276	-0.01251231	-0.00685773

283	-0.00856865	-0.01246775	-0.00650998
284	-0.00968573	-0.01234308	-0.00600454
285	-0.01070997	-0.01199640	-0.00547079
286	-0.01165588	-0.01163327	-0.00486695
287	-0.01242273	-0.01127001	-0.00420058
288	-0.01307487	-0.01089773	-0.00349859
289	-0.01360022	-0.01054941	-0.00274242
290	-0.01399908	-0.01017799	-0.00191236
291	-0.01425641	-0.00982630	-0.00100698
292	-0.01433866	-0.00948085	-0.00004302
293	0.02612859	0.01952022	0.01586025
294	0.02841071	0.01837611	0.01819758
295	0.03024286	0.01735905	0.01987186
296	0.03281106	0.01493751	0.02140506
297	0.03373374	0.01342331	0.02137791
298	0.03454561	0.01161398	0.02087741
299	0.03502466	0.00935832	0.01959987
300	0.03722675	0.01680324	-0.01084942
301	0.03934007	0.01614843	-0.01114570
302	0.04104727	0.01545122	-0.01131865
303	0.04223039	0.01451872	-0.01124403
304	0.04350103	0.01229513	-0.01066762
305	0.04372785	0.01092627	-0.01016598
306	0.04356026	0.00930782	-0.00944652
307	0.02057860	0.00394138	-0.01865185
308	0.02106960	0.00227311	-0.01988715
309	0.02167329	0.00081117	-0.02057945
310	0.02233530	-0.00041606	-0.02083801
311	0.02385516	-0.00237836	-0.02028370
312	0.02476193	-0.00324737	-0.01953550
313	0.02581516	-0.00404375	-0.01837347
314	0.03202536	0.00472688	0.01063477
315	0.03360772	0.00359534	0.01183002
316	0.03474712	0.00254808	0.01255802
317	0.03559168	0.00156644	0.01287586
318	0.03662872	-0.00042987	0.01247346
319	0.03688597	-0.00155823	0.01178537
320	0.03698314	-0.00283832	0.01072520
321	0.03836408	0.00566707	-0.00657096
322	0.04074353	0.00419949	-0.00702099
323	0.04240412	0.00363147	-0.00752129
324	0.04360539	0.00186429	-0.00736772
325	0.04368981	0.00095287	-0.00707683
326	0.04355486	-0.00008514	-0.00663895
327	0.04312100	-0.00135064	-0.00600136
328	0.02817480	-0.00288300	-0.01140616
329	0.02847650	-0.00456644	-0.01308824
330	0.02902266	-0.00598838	-0.01388909
331	0.03015701	-0.00784493	-0.01413801
332	0.03078710	-0.00851458	-0.01373502
333	0.03152637	-0.00913198	-0.01302492
334	0.03238835	-0.00966902	-0.01186516
335	-0.01204222	-0.00138318	0.01812431
336	-0.01013602	-0.00053717	0.01671165
337	-0.00793049	0.00102484	0.01382765
338	-0.00494188	0.00184967	0.01194854
339	-0.00182984	0.00150990	0.01344497
340	0.00058809	0.00245929	0.01285527
341	0.00304194	0.00364379	0.01233844
342	0.00542217	0.00511767	0.01177113

343	0.00776033	0.00680019	0.01139271
344	0.01010220	0.00848507	0.01149999
345	0.01171495	0.00917419	0.01155883
346	0.01328549	0.00990244	0.01151485
347	0.01479777	0.01062673	0.01137746
348	0.01626285	0.01128427	0.01113095
349	0.01767662	0.01192227	0.01076543
350	0.01905292	0.01257581	0.01031888
351	0.02041380	0.01319092	0.00977541
352	0.02170767	0.01383473	0.00904941
353	0.02294315	0.01446153	0.00823836
354	0.02502805	0.01503925	0.00667057
355	0.02680012	0.01542772	0.00499560
356	0.02830235	0.01564095	0.00327723
357	0.02950963	0.01564970	0.00150438
358	0.03046069	0.01547131	-0.00026805
359	0.03122589	0.01512515	-0.00205260
360	0.03175044	0.01455979	-0.00386348
361	0.03192175	0.01368358	-0.00565149
362	0.03179610	0.01290674	-0.00769413
363	0.03122775	0.01176183	-0.00928274
364	0.03051003	0.01113469	-0.01061945
365	0.02964736	0.01020795	-0.01165645
366	0.02864615	0.00936071	-0.01237762
367	0.02755815	0.00851015	-0.01282509
368	0.02636883	0.00764243	-0.01304274
369	0.02507882	0.00672659	-0.01301427
370	0.02366905	0.00579840	-0.01272472
371	0.02211640	0.00498938	-0.01204396
372	0.02045279	0.00316003	-0.00979857
373	0.01916153	0.00227365	-0.00758111
374	0.01848256	0.00147443	-0.00545802
375	0.01828815	0.00105916	-0.00351131
376	0.01864194	0.00057418	-0.00166477
377	0.01959972	0.00003330	0.00014107
378	0.02095640	-0.00027937	0.00195302
379	0.02326370	-0.00029175	0.00366031
380	0.02547285	0.00124219	0.00452259
381	0.02830197	0.00127667	0.00421628
382	0.02992727	0.00223010	0.00355677
383	0.03125153	0.00274714	0.00272099
384	0.03205955	0.00297544	0.00171836
385	0.03261070	0.00288489	0.00067322
386	0.03300508	0.00253051	-0.00039612
387	0.03332681	0.00174970	-0.00169203
388	0.03279990	0.00100424	-0.00330682
389	0.03124374	0.00055376	-0.00446302
390	0.03057061	-0.00076613	-0.00441997
391	0.02968591	-0.00174621	-0.00384154
392	0.02917576	-0.00204734	-0.00321609
393	0.02937683	-0.00243368	-0.00280057
394	0.02997589	-0.00296562	-0.00232441
395	0.03119022	-0.00395080	-0.00196819
396	0.03274871	-0.00501069	-0.00160286
397	0.03580146	-0.00581820	-0.00124378
398	0.00191093	0.01404236	0.01980735
399	0.00414923	0.01341556	0.01967640
400	0.00663408	0.01342038	0.01948632
401	0.00908345	0.01368781	0.01911090
402	0.01145892	0.01424264	0.01857034

403	0.01383231	0.01492714	0.01781561
404	0.01625215	0.01584057	0.01682444
405	0.01870603	0.01692177	0.01566670
406	0.02124517	0.01809575	0.01434379
407	0.02391951	0.01925385	0.01284687
408	0.02639989	0.02011493	0.01055552
409	0.02858519	0.02064683	0.00820387
410	0.03048575	0.02084107	0.00580502
411	0.03205268	0.02076702	0.00332024
412	0.03327754	0.02043264	0.00080762
413	0.03417734	0.01986551	-0.00180717
414	0.03474575	0.01905138	-0.00447936
415	0.03492539	0.01793785	-0.00717998
416	0.03484733	0.01672459	-0.01009585
417	0.03376044	0.01532637	-0.01225283
418	0.03253450	0.01397813	-0.01406555
419	0.03115566	0.01270924	-0.01554718
420	0.02967627	0.01144329	-0.01665938
421	0.02806743	0.01024014	-0.01742624
422	0.02633771	0.00903040	-0.01783111
423	0.02446508	0.00784950	-0.01788558
424	0.02248403	0.00665213	-0.01756752
425	0.02047371	0.00538614	-0.01686068
426	0.01767455	0.00380217	-0.01442357
427	0.01563926	0.00243247	-0.01190028
428	0.01430968	0.00128566	-0.00933315
429	0.01363712	0.00041466	-0.00671793
430	0.01356579	-0.00010382	-0.00403425
431	0.01429771	-0.00048762	-0.00128455
432	0.01574346	-0.00061462	0.00152832
433	0.01792642	-0.00052748	0.00440592
434	0.02070408	-0.00004645	0.00738472
435	0.02328346	0.00110372	0.00827859
436	0.02558047	0.00223850	0.00882758
437	0.02764850	0.00327243	0.00901322
438	0.02951526	0.00413886	0.00883036
439	0.03115171	0.00485656	0.00826345
440	0.03257372	0.00546798	0.00733106
441	0.03377638	0.00596222	0.00602917
442	0.03479851	0.00629137	0.00433796
443	0.03563914	0.00638651	0.00229050
444	0.03602448	0.00624751	0.00044993
445	0.03623213	0.00596586	-0.00124513
446	0.03627958	0.00562795	-0.00281674
447	0.03615337	0.00519496	-0.00424634
448	0.03587823	0.00465630	-0.00553546
449	0.03541166	0.00403900	-0.00668652
450	0.03476122	0.00335616	-0.00771121
451	0.03394886	0.00256416	-0.00859973
452	0.03296539	0.00168732	-0.00933991
453	0.03183031	0.00076257	-0.00973128
454	0.03074419	-0.00003179	-0.00989289
455	0.02976083	-0.00079145	-0.00980645
456	0.02882578	-0.00144526	-0.00949107
457	0.02796388	-0.00204959	-0.00891451
458	0.02718461	-0.00262241	-0.00805416
459	0.02657088	-0.00322995	-0.00688602
460	0.02614744	-0.00387516	-0.00544188
461	0.02597738	-0.00459583	-0.00379273
462	0.02682968	-0.00438352	-0.00194019

463	0.02779161	-0.00440018	-0.00050645
464	0.02876966	-0.00445949	0.00060126
465	0.02982501	-0.00454070	0.00134101
466	0.03085964	-0.00444823	0.00193876
467	0.03215940	-0.00455773	0.00190179
468	0.03406644	-0.00483323	0.00129291
469	0.03598541	-0.00585950	-0.00021268
470	-0.01538039	-0.00451389	0.01269231
471	-0.01459833	-0.00617146	0.01225358
472	-0.01353577	-0.00768469	0.01175815
473	-0.01223218	-0.00896902	0.01139384
474	-0.01085332	-0.01018924	0.01129036
475	-0.00933297	-0.01128480	0.01137456
476	-0.00773229	-0.01234305	0.01166616
477	-0.00605038	-0.01338345	0.01219126
478	-0.00422335	-0.01430850	0.01288085
479	-0.00226586	-0.01511142	0.01374858
480	-0.00044018	-0.01554254	0.01482205
481	0.00128377	-0.01582018	0.01595649
482	0.00296793	-0.01586489	0.01711054
483	0.00458482	-0.01570316	0.01829154
484	0.00613238	-0.01534556	0.01951983
485	0.00762813	-0.01480297	0.02078846
486	0.00907666	-0.01399237	0.02207404
487	0.01046713	-0.01293126	0.02338175
488	0.01181994	-0.01162753	0.02464800
489	0.01306009	-0.01007448	0.02582690
490	0.01430729	-0.00843133	0.02680420
491	0.01556004	-0.00675381	0.02766140
492	0.01683969	-0.00500907	0.02827418
493	0.01809574	-0.00318275	0.02872873
494	0.01937774	-0.00128116	0.02898774
495	0.02067886	0.00070570	0.02906514
496	0.02198802	0.00276618	0.02893177
497	0.02329922	0.00489979	0.02854589
498	0.02532670	0.00790325	0.02757539
499	0.02731458	0.01056007	0.02617940
500	0.02928970	0.01284096	0.02442354
501	0.03127635	0.01477559	0.02237799
502	0.03328830	0.01639545	0.02002901
503	0.03531924	0.01767660	0.01733047
504	0.03736695	0.01861641	0.01426478
505	0.03930494	0.01911488	0.01075890
506	0.04114241	0.01914413	0.00687072
507	0.04272756	0.01856876	0.00220118
508	0.04352676	0.01755187	-0.00219930
509	0.04357300	0.01623593	-0.00613966
510	0.04310310	0.01478781	-0.00959775
511	0.04211778	0.01321118	-0.01260412
512	0.04072187	0.01154995	-0.01525784
513	0.03889001	0.00978655	-0.01760440
514	0.03653700	0.00786271	-0.01959823
515	0.03360799	0.00576495	-0.02110648
516	0.03096580	0.00411612	-0.02186920
517	0.02840588	0.00254223	-0.02210954
518	0.02599237	0.00106731	-0.02188436
519	0.02374732	-0.00029996	-0.02124762
520	0.02165888	-0.00159279	-0.02031671
521	0.01970726	-0.00280548	-0.01900679
522	0.01787906	-0.00393857	-0.01732249

523	0.01618635	-0.00498840	-0.01526225
524	0.01467104	-0.00592675	-0.01280378
525	0.01372144	-0.00682088	-0.01034554
526	0.01313186	-0.00755027	-0.00788546
527	0.01289574	-0.00815067	-0.00552384
528	0.01290191	-0.00856003	-0.00311623
529	0.01325708	-0.00886734	-0.00083889
530	0.01385627	-0.00903955	0.00143847
531	0.01475573	-0.00912087	0.00369296
532	0.01597762	-0.00899668	0.00591192
533	0.01757104	-0.00868936	0.00802332
534	0.01937350	-0.00811555	0.01001476
535	0.02128417	-0.00735466	0.01158936
536	0.02323370	-0.00649075	0.01281715
537	0.02522140	-0.00551558	0.01371664
538	0.02726845	-0.00438476	0.01421390
539	0.02935402	-0.00324699	0.01453383
540	0.03150039	-0.00189537	0.01437119
541	0.03371888	-0.00045510	0.01390557
542	0.03594945	0.00108447	0.01292195
543	0.03826770	0.00224548	0.01138874
544	0.04018564	0.00313758	0.00954945
545	0.04169720	0.00376246	0.00755882
546	0.04290075	0.00416286	0.00546059
547	0.04379390	0.00431077	0.00325203
548	0.04440876	0.00425936	0.00087697
549	0.04468476	0.00397485	-0.00169039
550	0.04450681	0.00340383	-0.00444315
551	0.04381987	0.00252313	-0.00726177
552	0.04288183	0.00154583	-0.00948848
553	0.04168470	0.00051007	-0.01132042
554	0.04035316	-0.00055371	-0.01279008
555	0.03889325	-0.00166315	-0.01390553
556	0.03732232	-0.00279969	-0.01471605
557	0.03558868	-0.00397093	-0.01519348
558	0.03371355	-0.00519929	-0.01538223
559	0.03165956	-0.00645471	-0.01514692
560	0.02951697	-0.00771655	-0.01441895
561	0.02759348	-0.00906305	-0.01309244
562	0.02610527	-0.01014696	-0.01148372
563	0.02504858	-0.01100770	-0.00977568
564	0.02436049	-0.01168748	-0.00803040
565	0.02398572	-0.01224178	-0.00622255
566	0.02391920	-0.01267559	-0.00432000
567	0.02422272	-0.01296599	-0.00228325
568	0.02499559	-0.01292992	-0.00014304
569	0.02624888	-0.01263372	0.00188462
570	0.02821003	-0.01197163	0.00379870
571	0.03015612	-0.01115410	0.00491303
572	0.03181389	-0.01042326	0.00544534
573	0.03319330	-0.00979441	0.00557482
574	0.03448587	-0.00924831	0.00542052
575	0.03581192	-0.00876447	0.00487858
576	0.03722825	-0.00831370	0.00363272
577	0.03800848	-0.00789032	0.00117317
578	-0.00466341	-0.00601524	-0.01373668
579	-0.00281193	-0.00543530	-0.01287591
580	-0.00081863	-0.00533001	-0.01194638
581	0.00088182	-0.00538699	-0.01078723
582	0.00231245	-0.00560779	-0.00938235

583	0.00336665	-0.00586155	-0.00770281
584	0.00404524	-0.00623730	-0.00568428
585	0.00457001	-0.00678864	-0.00350447
586	0.00487402	-0.00748338	-0.00109532
587	0.00522529	-0.00849633	0.00137344
588	0.00625713	-0.01002519	0.00367791
589	0.00766463	-0.01138470	0.00579478
590	0.00917035	-0.01241423	0.00775846
591	0.01073294	-0.01303014	0.00962587
592	0.01233758	-0.01321318	0.01142407
593	0.01402707	-0.01301644	0.01315660
594	0.01580147	-0.01242966	0.01482758
595	0.01763929	-0.01142867	0.01645123
596	0.01953272	-0.01000862	0.01800049
597	0.02143878	-0.00817126	0.01923040
598	0.02319084	-0.00620835	0.02018528
599	0.02482227	-0.00417807	0.02082659
600	0.02639480	-0.00213831	0.02112650
601	0.02795322	-0.00011766	0.02105865
602	0.02950600	0.00189937	0.02062220
603	0.03113520	0.00386847	0.01979802
604	0.03289795	0.00572996	0.01861744
605	0.03477509	0.00743418	0.01712983
606	0.03687864	0.00888717	0.01455126
607	0.03872876	0.00992600	0.01191552
608	0.04029483	0.01058980	0.00922817
609	0.04155667	0.01088086	0.00644548
610	0.04248907	0.01083234	0.00360265
611	0.04309332	0.01042303	0.00066609
612	0.04334440	0.00971012	-0.00236560
613	0.04317824	0.00872257	-0.00551724
614	0.04253464	0.00752632	-0.00875518
615	0.04155173	0.00624920	-0.01117963
616	0.04028065	0.00501574	-0.01321174
617	0.03885610	0.00373865	-0.01485204
618	0.03721970	0.00246869	-0.01610004
619	0.03543946	0.00114008	-0.01695046
620	0.03348335	-0.00021747	-0.01741643
621	0.03136428	-0.00161994	-0.01750300
622	0.02907735	-0.00307941	-0.01721312
623	0.02663645	-0.00460326	-0.01653429
624	0.02453943	-0.00605667	-0.01521649
625	0.02278522	-0.00725907	-0.01366369
626	0.02141041	-0.00828587	-0.01191458
627	0.02040339	-0.00913031	-0.00997486
628	0.01979396	-0.00982490	-0.00784149
629	0.01958360	-0.01042312	-0.00551736
630	0.01976898	-0.01088840	-0.00299823
631	0.02033996	-0.01125321	-0.00026092
632	0.02128979	-0.01133813	0.00264113
633	0.02268939	-0.01112877	0.00480382
634	0.02414266	-0.01066616	0.00661177
635	0.02565798	-0.01001881	0.00804288
636	0.02726550	-0.00924377	0.00909903
637	0.02896595	-0.00838948	0.00979511
638	0.03076924	-0.00742090	0.01012578
639	0.03268348	-0.00635486	0.01009077
640	0.03473599	-0.00524463	0.00972435
641	0.03687386	-0.00400310	0.00898242
642	0.03844028	-0.00325551	0.00789275

643	0.03976952	-0.00266260	0.00668514
644	0.04085248	-0.00222408	0.00534996
645	0.04170355	-0.00194648	0.00389866
646	0.04232742	-0.00183861	0.00233793
647	0.04271579	-0.00187360	0.00064201
648	0.04290284	-0.00210394	-0.00116932
649	0.04280417	-0.00244185	-0.00312280
650	0.04243198	-0.00290415	-0.00518637
651	0.04182768	-0.00351687	-0.00669382
652	0.04110292	-0.00418152	-0.00796906
653	0.04024128	-0.00486510	-0.00900013
654	0.03928936	-0.00561765	-0.00979403
655	0.03822561	-0.00639788	-0.01035224
656	0.03706120	-0.00724410	-0.01067928
657	0.03580478	-0.00816108	-0.01076942
658	0.03445184	-0.00913647	-0.01062386
659	0.03297994	-0.01013779	-0.01023144
660	0.03178822	-0.01106810	-0.00925687
661	0.03082394	-0.01182683	-0.00821804
662	0.03009635	-0.01243989	-0.00712598
663	0.02962127	-0.01293061	-0.00598812
664	0.02939838	-0.01328171	-0.00480434
665	0.02939509	-0.01348718	-0.00356527
666	0.02964751	-0.01355619	-0.00226979
667	0.03014549	-0.01346644	-0.00092488
668	0.03086139	-0.01320598	0.00047939
669	0.03200241	-0.01278588	0.00122238
670	0.03302717	-0.01230007	0.00173144
671	0.03400655	-0.01185759	0.00203943
672	0.03490510	-0.01139296	0.00213246
673	0.03574897	-0.01090453	0.00200898
674	0.03656349	-0.01035245	0.00163944
675	0.03734748	-0.00967271	0.00096725
676	0.03785427	-0.00863453	-0.00000781
677	-0.00915533	0.01388269	0.00005312
678	-0.01265815	0.01688629	-0.00153402
679	-0.01644268	0.01949412	-0.00300594
680	-0.02000836	0.02196285	-0.00425983
681	-0.02324326	0.02438896	-0.00534940
682	-0.02637405	0.02656325	-0.00623533
683	-0.02958320	0.02842424	-0.00698364
684	-0.03244644	0.03012953	-0.00736925
685	-0.03530202	0.03166864	-0.00762570
686	-0.03808761	0.03288723	-0.00774589

Procrustes sums of squares: 0.31411259057623375
Tangent sums of squares: 0.31133812070541633

Data matrices in this dataset:
- Evolution Cervidae final, raw data
- clean (sans Hetero-Capreo), centroid size
- clean (sans Hetero-Capreo), Procrustes coordinates

Principal Component Analysis: PCA: CovMatrix, clean (sans Hetero-Capreo), Procrustes coordinates

Eigenvalues% Variance Cumulative %

1.	0.00132206	15.712	15.712
2.	0.00086081	10.230	25.942
3.	0.00062934	7.479	33.421
4.	0.00057485	6.832	40.252
5.	0.00051749	6.150	46.402
6.	0.00038169	4.536	50.939
7.	0.00037101	4.409	55.348
8.	0.00034337	4.081	59.428
9.	0.00031511	3.745	63.173
10.	0.00028771	3.419	66.592
11.	0.00026328	3.129	69.721
12.	0.00020955	2.490	72.211
13.	0.00019361	2.301	74.512
14.	0.00018855	2.241	76.753
15.	0.00016675	1.982	78.735
16.	0.00015167	1.803	80.537
17.	0.00014630	1.739	82.276
18.	0.00013883	1.650	83.926
19.	0.00012717	1.511	85.437
20.	0.00011974	1.423	86.860
21.	0.00011560	1.374	88.234
22.	0.00010137	1.205	89.439
23.	0.00009211	1.095	90.533
24.	0.00008950	1.064	91.597
25.	0.00008322	0.989	92.586
26.	0.00007481	0.889	93.475
27.	0.00007272	0.864	94.339
28.	0.00006743	0.801	95.140
29.	0.00005929	0.705	95.845
30.	0.00005859	0.696	96.541
31.	0.00005560	0.661	97.202
32.	0.00004785	0.569	97.771
33.	0.00004518	0.537	98.308
34.	0.00004175	0.496	98.804
35.	0.00003709	0.441	99.245
36.	0.00003378	0.401	99.646
37.	0.00002979	0.354	100.000

Total variance: 0.00841454

Variance of the eigenvalues: 0.0000000021561
Eigenvalue variance scaled by total variance: 0.00003
Eigenvalue variance scaled by total variance and number of variables: 0.06249
Note: throughout all calculations of eigenvalue variances, the dimensionality used was 2051.
Please double-check because this dimensionality may not be appropriate for all situations.
Warning: There appear to be fewer PCs than dimensions in shape space.
This can have the following reasons, among others:
-- the degrees of freedom are less than the shape dimensionality (sample size, etc.).
-- there is complete integration in the data.
Interpret the results cautiously and check for reasons why this is the case.

Principal Component Coefficients

PC1 PC2 PC3 PC4 PC5 PC6 PC7 PC8 PC9 PC10 PC11 PC12

PC13 PC14 PC15 PC16 PC17 PC18
PC19 PC20 PC21 PC22 PC23 PC24
PC25 PC26 PC27 PC28 PC29 PC30
PC31 PC32 PC33 PC34 PC35 PC36
PC37
x1 -0.014537 0.011401 0.027553 0.022412 0.002147 0.027710
0.017175 0.027914 0.015881 0.010561 -0.008846 -0.026326
0.015525 -0.007508 -0.000415 -0.012847 0.031898 0.011614
0.017070 -0.004192 0.059708 0.001226 -0.035917 -0.002787 -
0.002839 -0.067361 0.068500 -0.002213 0.000382 -0.004263 -
0.032734 0.001034 -0.026154 -0.032917 0.026183 -0.004233
0.015425
y1 -0.031414 -0.005050 0.012627 0.032834 -0.002139 0.020919
0.003996 -0.035107 -0.029759 -0.028889 -0.026089 0.021009 -
0.002347 -0.002648 0.029917 -0.002927 -0.006784 0.000874
0.017682 -0.027718 -0.000818 0.020526 -0.018491 0.024223
0.011935 0.017004 -0.011593 0.015238 0.018664 -0.009937
0.012513 0.022910 0.027096 -0.025183 0.003716 -0.037945 -
0.018014
z1 0.002494 0.016218 -0.001922 0.013526 0.017229 -0.036375
0.067598 -0.001337 0.028326 -0.005021 0.005387 -0.040356 -
0.009882 -0.010834 0.017590 -0.035280 0.009988 0.030677 -
0.002745 0.051796 0.026709 0.007039 -0.008577 -0.007568
0.020427 -0.002497 0.022395 -0.030934 -0.013735 0.008093
0.024627 -0.005814 -0.007038 -0.021087 0.003089 -0.022835 -
0.009215
x2 -0.030273 0.006279 -0.003759 0.026211 -0.001424 0.028632
0.015311 0.010240 -0.005018 -0.023179 0.021753 0.005742 -
0.021455 0.001831 -0.030440 -0.016397 0.028294 -0.009646 -
0.011572 0.001853 -0.013665 0.019354 -0.003677 0.005203
0.009959 0.013530 -0.030426 0.002721 -0.012829 0.028082
0.002878 -0.039370 0.001723 0.027086 0.031837 -0.009991
0.011593
y2 0.016752 0.004494 -0.005718 0.019126 -0.033267 -0.021531
0.022451 0.005558 0.037320 -0.000502 -0.008082 0.010832
0.011482 -0.000649 0.013745 0.014427 0.008123 0.020260 -
0.014396 -0.035417 -0.026191 0.029065 0.008464 -0.015449 -
0.006172 0.024460 -0.026464 -0.017620 0.006498 0.034301 -
0.010340 -0.031164 0.036851 -0.017011 -0.055167 -0.001381 -
0.022626
z2 0.032988 -0.006272 0.010194 -0.016342 -0.005850 0.030240
0.003057 0.017551 0.009048 -0.001543 -0.000788 0.020770 -
0.030078 0.008612 -0.003536 -0.007842 0.001864 0.010159 -
0.021853 -0.016014 -0.006139 0.024029 -0.000049 0.001748
0.013388 -0.004538 0.024108 -0.003669 0.017568 -0.012103 -
0.028398 -0.005579 -0.006179 0.004992 0.010893 0.017303
0.002821
x3 -0.030728 0.008182 -0.007536 0.027347 -0.008948 0.018018
0.014920 0.014179 -0.007422 -0.031721 0.023964 0.000735 -
0.027506 0.012185 -0.019397 -0.014660 0.024404 -0.006886 -
0.007476 -0.001878 -0.017468 0.020723 -0.010742 0.008046
0.010164 0.005461 -0.033627 -0.002701 -0.010397 0.007473
0.006549 -0.031138 -0.008757 0.033907 0.013556 0.000443
0.013411
y3 0.017104 0.004002 0.000642 0.019983 -0.034454 -0.021526
0.018031 0.008225 0.041044 0.002046 -0.008792 0.012305
0.019901 0.003059 0.011241 0.007860 0.003039 0.007533 -
0.019337 -0.029576 -0.033141 0.023041 0.010371 -0.016431 -
0.003315 0.007699 -0.023837 -0.003873 -0.002078 0.022721 -

0.025575 -0.013489 0.048321 -0.028752 -0.052493 -0.005775 -
0.024896
z3 0.037925 -0.010749 0.008847 -0.016267 -0.005193 0.027516
0.004838 0.020155 0.004153 -0.005861 -0.004215 0.024172 -
0.022789 0.012844 -0.000992 -0.007783 -0.001227 0.004680 -
0.021995 -0.026791 -0.003658 0.023143 -0.002349 -0.000375
0.012645 -0.005645 0.017063 -0.005809 0.011648 -0.013669 -
0.013091 -0.005173 -0.003315 0.012095 0.001449 0.018304 -
0.007685
x4 -0.028589 0.009326 -0.008542 0.028191 -0.013421 0.012331
0.015436 0.017445 -0.007510 -0.036866 0.029278 -0.005857 -
0.029467 0.020984 -0.011626 -0.018130 0.018332 -0.010241 -
0.005662 -0.002168 -0.028553 0.020559 -0.012892 0.004084
0.013126 -0.006253 -0.034511 -0.002108 -0.017457 -0.019126
0.000381 -0.013426 -0.004799 0.029198 0.001342 0.009425 -
0.000606
y4 0.013933 0.002486 0.005828 0.019947 -0.036033 -0.021821
0.009662 0.010982 0.044286 0.006472 -0.013275 0.017038
0.023343 0.004326 0.003855 0.006218 -0.000158 0.002025 -
0.021111 -0.030018 -0.029753 0.016915 0.014787 -0.013412 -
0.002128 -0.003340 -0.018487 0.004606 0.001517 0.025074 -
0.029790 -0.015938 0.048570 -0.027085 -0.047945 -0.013977 -
0.011139
z4 0.044776 -0.014662 0.008706 -0.016591 -0.004192 0.025097
0.007206 0.022013 0.000985 -0.007001 -0.007446 0.026288 -
0.016120 0.017859 0.000937 -0.008048 -0.003124 -0.003915 -
0.023401 -0.032283 -0.005736 0.021042 -0.002272 -0.001662
0.015326 -0.010989 0.009278 -0.001058 -0.000248 -0.018070 -
0.003508 0.005917 0.003078 0.015586 -0.008348 0.022560 -
0.022267
x5 -0.028304 0.009153 -0.007648 0.028924 -0.018609 0.008586
0.013436 0.021728 -0.006181 -0.039525 0.031906 -0.010390 -
0.030918 0.028886 -0.005465 -0.021446 0.011584 -0.010911 -
0.003295 -0.004094 -0.035059 0.020512 -0.012309 0.002213
0.017640 -0.014254 -0.035892 0.000407 -0.019244 -0.036821 -
0.001381 -0.004380 -0.003958 0.032082 -0.008216 0.014798 -
0.007716
y5 0.011354 0.003235 0.009079 0.018824 -0.034992 -0.021821
0.002825 0.010758 0.044587 0.007921 -0.012060 0.019567
0.027380 0.003998 -0.003264 0.002740 -0.000658 -0.004565 -
0.022466 -0.027808 -0.029274 0.009999 0.014979 -0.007763 -
0.007962 -0.011026 -0.013675 0.006997 0.009959 0.026086 -
0.038270 -0.017495 0.046719 -0.035514 -0.042457 -0.012716 -
0.006062
z5 0.050021 -0.017200 0.008349 -0.017156 -0.003440 0.021591
0.009424 0.022241 -0.001969 -0.007350 -0.011744 0.028662 -
0.011452 0.021246 0.001774 -0.006041 -0.004334 -0.009143 -
0.021989 -0.036428 -0.005861 0.020360 -0.003315 -0.003061
0.016642 -0.014217 0.002621 -0.000577 -0.006737 -0.020064
0.005170 0.011284 0.007801 0.020409 -0.015231 0.023140 -
0.031275
x6 -0.028443 0.008883 -0.005417 0.028495 -0.022909 0.004852
0.011454 0.027088 -0.004599 -0.040968 0.033909 -0.012509 -
0.030619 0.034806 -0.000335 -0.022218 0.007427 -0.011070 -
0.001701 -0.002335 -0.036692 0.020611 -0.012832 0.005704
0.020807 -0.019009 -0.036852 0.001351 -0.026618 -0.049104 -
0.001609 0.003811 -0.007018 0.031318 -0.015512 0.018297 -
0.012814
y6 0.011141 0.002978 0.010061 0.018230 -0.033006 -0.022396
-0.003092 0.010085 0.043345 0.009986 -0.009845 0.020357

0.031271 -0.001394 -0.000621 0.002043 -0.002555 -0.010069 -
0.023814 -0.027621 -0.026645 0.004990 0.011705 -0.009364 -
0.005983 -0.020906 -0.009594 0.008164 0.004933 0.025275 -
0.037477 -0.008189 0.049012 -0.049418 -0.044322 -0.016970
0.006106
z6 0.053922 -0.017715 0.008576 -0.017501 -0.002630 0.018205
0.011142 0.020410 -0.004227 -0.007950 -0.014484 0.028899 -
0.006922 0.024602 0.001962 -0.006425 -0.006494 -0.012113 -
0.019047 -0.041193 -0.007128 0.017350 -0.003574 -0.006477
0.014124 -0.016208 -0.004451 0.001310 0.001250 -0.020737
0.009053 0.008823 0.014307 0.025788 -0.018082 0.021817 -
0.042491
x7 -0.028842 0.008433 -0.002690 0.029115 -0.026365 0.002575
0.009456 0.032197 -0.002407 -0.041249 0.036049 -0.015046 -
0.028767 0.038134 0.006958 -0.023182 0.001549 -0.009671 -
0.001479 -0.001829 -0.038963 0.018526 -0.015206 0.004609
0.023693 -0.023399 -0.039085 0.004552 -0.029359 -0.058745 -
0.002747 0.008384 -0.008871 0.025518 -0.019942 0.017260 -
0.019140
y7 0.008836 0.004162 0.011514 0.017497 -0.032654 -0.023319
-0.011111 0.006964 0.040028 0.011055 -0.013190 0.021128
0.032354 -0.005993 -0.002522 0.006161 -0.004795 -0.015146 -
0.027336 -0.024873 -0.022800 0.005723 0.005121 -0.006669 -
0.003335 -0.027439 -0.002655 0.012583 0.008231 0.024651 -
0.038921 -0.012166 0.042654 -0.053473 -0.042506 -0.015738
0.017918
z7 0.057816 -0.017337 0.010052 -0.018924 -0.002627 0.013751
0.012708 0.018599 -0.006210 -0.008040 -0.016898 0.029264 -
0.002490 0.026391 0.003190 -0.005144 -0.006860 -0.014821 -
0.014629 -0.042600 -0.005444 0.014141 -0.005380 -0.007578
0.013117 -0.016472 -0.011844 0.003608 0.001151 -0.020545
0.012667 0.005575 0.019207 0.026835 -0.020461 0.021958 -
0.046934
x8 -0.029774 0.007840 0.001576 0.030600 -0.029303 0.001621
0.007297 0.038066 0.000504 -0.039367 0.036856 -0.016369 -
0.025209 0.039713 0.015195 -0.024326 -0.005048 -0.007276 -
0.001863 -0.000250 -0.038063 0.015682 -0.016655 0.001440
0.024698 -0.025103 -0.042606 0.008493 -0.030338 -0.062862 -
0.007919 0.009307 -0.009787 0.015116 -0.020374 0.012253 -
0.030326
y8 0.008086 0.005813 0.014230 0.011071 -0.027295 -0.017077
-0.014337 0.004120 0.038309 0.022085 -0.004368 0.031469
0.039849 -0.009889 -0.013684 -0.003181 0.004484 -0.020707 -
0.021741 -0.015272 -0.018038 0.002647 0.009738 -0.007184 -
0.009023 -0.022880 -0.000664 0.013667 0.013474 0.026824 -
0.041411 -0.015749 0.040145 -0.050008 -0.038707 -0.012607
0.017036
z8 0.060885 -0.017521 0.011555 -0.019491 -0.003064 0.006751
0.013995 0.016577 -0.008601 -0.008366 -0.020539 0.029300
0.001068 0.025802 0.004588 -0.003510 -0.009976 -0.016023 -
0.009181 -0.042006 -0.001772 0.012020 -0.009104 -0.007873
0.013132 -0.015380 -0.015509 0.003555 0.001419 -0.018284
0.014210 0.000751 0.022527 0.025906 -0.020812 0.023904 -
0.048620
x9 -0.031247 0.007353 0.008298 0.032251 -0.031632 0.001854
0.004297 0.044292 0.005349 -0.037092 0.036211 -0.017408 -
0.020018 0.041084 0.022000 -0.023905 -0.012321 -0.003191 -
0.003591 0.002138 -0.034086 0.011323 -0.015853 -0.000795
0.026445 -0.026883 -0.044522 0.013987 -0.030158 -0.061178 -

0.009441 0.005241 -0.009919 0.004697 -0.017528 0.000099 -
0.040794
y9 0.007273 0.006013 0.012350 0.009897 -0.022726 -0.012369
-0.023710 -0.001412 0.036032 0.023761 -0.003132 0.031306
0.039742 -0.015852 -0.014751 -0.000753 0.005323 -0.023055 -
0.019873 -0.013399 -0.019330 0.001571 0.009755 -0.011032 -
0.005999 -0.029918 0.006885 0.009897 0.024540 0.028504 -
0.038309 -0.013301 0.037037 -0.056290 -0.036121 -0.015362
0.022828
z9 0.063975 -0.016368 0.014550 -0.021374 -0.003754 0.000327
0.014117 0.013631 -0.008779 -0.008092 -0.022993 0.026700
0.004450 0.024643 0.008191 -0.001713 -0.011811 -0.017173 -
0.002813 -0.040584 -0.001708 0.008965 -0.012397 -0.009194
0.013279 -0.016185 -0.021323 0.004885 0.002333 -0.015964
0.013374 -0.001990 0.026568 0.020398 -0.018048 0.020745 -
0.046089
x10 -0.032392 0.006379 0.015027 0.033596 -0.032888
0.004557 0.000710 0.049680 0.010117 -0.032053 0.036162 -
0.017207 -0.012939 0.039321 0.030575 -0.023208 -0.020427
0.000667 -0.008251 0.004557 -0.029508 0.005604 -0.016186 -
0.004949 0.029341 -0.027007 -0.047393 0.023929 -0.033713 -
0.058398 -0.013200 0.001299 -0.008351 -0.008642 -0.006681 -
0.008844 -0.049051
y10 0.006587 0.008533 0.010415 0.001962 -0.015280 -
0.004634 -0.030932 -0.008751 0.032489 0.032104 0.005030
0.038065 0.041188 -0.020398 -0.026022 -0.000666 0.018334 -
0.026423 -0.016625 -0.004632 -0.015807 0.001416 0.016459 -
0.002795 -0.007634 -0.026887 0.013844 0.010407 0.034357
0.028217 -0.030949 -0.013401 0.027617 -0.043737 -0.028466 -
0.004183 0.032325
z10 0.066046 -0.013617 0.017500 -0.023681 -0.004550 -
0.007447 0.013834 0.009419 -0.009101 -0.007226 -0.025973
0.023465 0.005825 0.023564 0.009286 0.001522 -0.012499 -
0.017160 0.003737 -0.036768 0.000435 0.005957 -0.015731 -
0.007363 0.012580 -0.014184 -0.024307 0.006073 0.006077 -
0.013343 0.010753 -0.008143 0.030198 0.018209 -0.011892
0.022469 -0.041082
x11 -0.033513 0.005174 0.021554 0.034349 -0.034168
0.007611 -0.003688 0.056839 0.014861 -0.027366 0.034894 -
0.017330 -0.006647 0.036637 0.033911 -0.019249 -0.025321
0.004060 -0.010970 0.005916 -0.025102 0.001573 -0.019116 -
0.005144 0.032349 -0.027114 -0.049479 0.033585 -0.039310 -
0.050564 -0.014177 0.002891 -0.007258 -0.022193 0.005531 -
0.018488 -0.053926
y11 0.005788 0.010708 0.005237 -0.005407 -0.008245
0.002560 -0.037615 -0.017078 0.026427 0.036672 0.013409
0.043266 0.038676 -0.026092 -0.038846 0.001294 0.033117 -
0.030456 -0.010315 0.000601 -0.015932 0.005117 0.018911
0.002918 -0.009484 -0.022838 0.021464 0.005734 0.045350
0.027969 -0.019601 -0.005292 0.018050 -0.033743 -0.028525
0.008657 0.042600
z11 0.067022 -0.009439 0.020080 -0.026188 -0.005818 -
0.015924 0.013146 0.003329 -0.008820 -0.006829 -0.027592
0.019492 0.003989 0.022201 0.010974 0.006749 -0.012806 -
0.016658 0.011981 -0.031521 0.003218 0.003553 -0.019601 -
0.007065 0.010843 -0.011033 -0.025546 0.005934 0.012425 -
0.011321 0.006329 -0.014870 0.034508 0.014996 -0.004725
0.023289 -0.035502
x12 -0.030371 0.005665 0.022778 0.027917 -0.035645
0.012556 -0.008357 0.050512 0.016831 -0.021718 0.036881 -

0.015450	0.003614	0.033999	0.026469	-0.011023	-0.009829	
0.013447	-0.004872	0.009946	-0.015819	-0.004611	-0.009001	
0.005517	0.031293	-0.024241	-0.035160	0.020122	-0.011322	-
0.030274	0.000708	0.009647	0.000821	-0.027904	0.009975	-
0.015945	-0.038501					
y12	-0.000926	0.011865	0.002437	-0.008426	0.005802	
0.004843	-0.048540	-0.019626	0.022410	0.037719	0.020188	
0.037930	0.026166	-0.025638	-0.047467	0.012346	0.026979	-
0.036831	-0.017118	-0.011685	-0.009585	-0.000669	0.017020	
0.000702	-0.007104	-0.033885	0.031734	0.002769	0.049379	
0.015478	-0.009398	0.000624	0.004993	-0.023506	-0.021985	
0.005807	0.036965					
z12	0.063677	-0.007868	0.021644	-0.034264	-0.007823	-
0.020011	0.009567	0.003059	-0.009323	-0.005059	-0.026346	
0.016355	0.008113	0.013318	0.001780	0.014315	-0.008972	-
0.014072	0.017223	-0.022517	0.002560	0.003739	-0.013817	-
0.004901	0.007107	-0.006783	-0.023422	0.002897	0.019931	-
0.003533	-0.008482	-0.018603	0.026805	0.014242	0.000089	
0.019292	-0.024730					
x13	-0.027794	0.007590	0.025978	0.021011	-0.035795	
0.018585	-0.014113	0.046678	0.018955	-0.015095	0.036397	-
0.015539	0.010182	0.030390	0.016986	-0.002904	0.007450	
0.021464	-0.000211	0.010467	-0.005987	-0.013039	-0.001506	
0.015220	0.027403	-0.017245	-0.027131	0.015835	0.008638	-
0.010625	0.017889	0.019125	0.006252	-0.023713	0.016896	-
0.010280	-0.025414					
y13	-0.006392	0.014853	0.002537	-0.010457	0.017972	
0.010485	-0.055422	-0.021394	0.017954	0.037953	0.024068	
0.031840	0.012266	-0.028020	-0.050477	0.015740	0.025489	-
0.041760	-0.019427	-0.021656	-0.006420	-0.008856	0.013190	-
0.006341	-0.008556	-0.035890	0.033114	0.008650	0.047614	
0.003590	-0.000903	0.006754	-0.004436	-0.012195	-0.012837	
0.003426	0.026764					
z13	0.059919	-0.005061	0.022878	-0.041986	-0.007702	-
0.022278	0.005067	0.002761	-0.007559	-0.001968	-0.023931	
0.012801	0.010668	0.007127	-0.005611	0.020835	-0.003312	-
0.014693	0.022170	-0.015853	0.002997	0.003369	-0.011250	-
0.005645	0.005919	-0.001930	-0.022466	0.006242	0.024965	-
0.000188	-0.015105	-0.018497	0.018267	0.012673	0.007106	
0.016388	-0.021859					
x14	-0.024481	0.006689	0.025604	0.017855	-0.033484	
0.019330	-0.021691	0.047347	0.017722	-0.010987	0.030414	-
0.017123	0.011760	0.026808	0.013756	0.009152	0.006880	
0.029165	-0.003204	0.007421	-0.000735	-0.018388	0.005070	
0.025831	0.023484	-0.012544	-0.028833	0.003032	0.024288	
0.003951	0.016047	0.026886	0.004050	-0.017130	0.018644	-
0.005677	-0.017072					
y14	-0.010384	0.015316	-0.000182	-0.009447	0.029927	
0.012081	-0.061405	-0.021119	0.011867	0.036302	0.025944	
0.026642	-0.000701	-0.027098	-0.047707	0.019968	0.013246	-
0.045810	-0.024867	-0.030625	-0.007514	-0.013231	0.011293	-
0.010607	-0.009037	-0.037159	0.026210	0.005743	0.044174	-
0.006644	-0.003539	0.012339	-0.015086	-0.000020	-0.010456	
0.001892	0.015575					
z14	0.055143	-0.003737	0.022369	-0.046052	-0.005588	-
0.027961	0.001183	0.002272	-0.009022	-0.003588	-0.021248	
0.010228	0.011883	0.002401	-0.009966	0.027531	0.000056	-
0.012720	0.022719	-0.012254	0.002255	0.002165	-0.010529	-
0.005197	0.013143	-0.008503	-0.024288	0.004599	0.027535	-

0.003399 -0.023772 -0.017626 0.014557 0.012104 0.009514
0.009419 -0.015918
x15 -0.021614 0.008046 0.026310 0.013443 -0.025977
0.022966 -0.026773 0.046642 0.012798 -0.003590 0.035675 -
0.017321 0.015686 0.024351 0.003693 0.009950 0.012803
0.027853 -0.005749 0.004002 0.004911 -0.026227 0.010900
0.028409 0.020267 -0.005775 -0.027667 0.002100 0.038478
0.007032 0.018419 0.030417 0.005141 -0.009763 0.021907 -
0.003693 -0.010021
y15 -0.014441 0.015724 -0.002759 -0.008725 0.041517
0.012967 -0.063631 -0.021665 0.006354 0.035800 0.031513
0.022755 -0.009136 -0.023292 -0.047598 0.018559 0.004717 -
0.049353 -0.028003 -0.037479 -0.006275 -0.016669 0.009200 -
0.013633 -0.007776 -0.038072 0.021871 0.007394 0.039642 -
0.017117 -0.002122 0.014830 -0.022032 0.009228 -0.006483 -
0.001723 0.006900
z15 0.052033 -0.001711 0.023420 -0.049515 -0.002001 -
0.030542 -0.003318 0.003319 -0.010845 -0.004231 -0.015757
0.008133 0.012005 -0.002577 -0.015295 0.029692 0.003600 -
0.013006 0.023816 -0.006918 0.000998 -0.000563 -0.008725 -
0.006495 0.016777 -0.008944 -0.027991 0.004977 0.026358 -
0.008669 -0.028630 -0.015968 0.010292 0.014594 0.011975
0.007004 -0.015011
x16 -0.018607 0.009113 0.026643 0.009950 -0.019307
0.022442 -0.032892 0.047001 0.008296 -0.000112 0.035371 -
0.016325 0.016939 0.017748 -0.000020 0.011936 0.014250
0.032398 -0.012361 -0.002344 0.011102 -0.035209 0.010257
0.037684 0.015702 -0.001084 -0.021029 -0.002624 0.045945
0.013487 0.014178 0.031737 -0.000082 -0.004864 0.025121
0.002319 0.001810
y16 -0.017715 0.016332 -0.003497 -0.007434 0.049551
0.013687 -0.063192 -0.022416 -0.000333 0.031110 0.032647
0.018683 -0.018758 -0.023148 -0.041043 0.017659 0.001580 -
0.045388 -0.028915 -0.040334 -0.005304 -0.020852 0.005911 -
0.010392 -0.006363 -0.038007 0.019495 0.005986 0.031315 -
0.021944 -0.004454 0.017133 -0.029900 0.019027 -0.004524 -
0.004886 0.003351
z16 0.049487 -0.000282 0.023314 -0.052518 0.002145 -
0.033934 -0.008850 0.004673 -0.010951 -0.005269 -0.011367
0.006727 0.011110 -0.007268 -0.018650 0.031310 0.002746 -
0.010891 0.022855 -0.004825 0.001343 -0.002851 -0.012871 -
0.005018 0.019764 -0.009196 -0.028538 0.004023 0.024311 -
0.011430 -0.029522 -0.012582 -0.000751 0.012776 0.015026
0.006362 -0.013338
x17 -0.016005 0.008909 0.027910 0.005796 -0.010340
0.020933 -0.039107 0.052298 0.000039 0.002884 0.032916 -
0.014010 0.019255 0.016346 -0.004404 0.013672 0.016065
0.030611 -0.019588 -0.005980 0.015772 -0.040010 0.009309
0.040518 0.024664 0.004276 -0.020528 -0.002573 0.040084
0.020064 0.003430 0.036910 -0.005538 0.005126 0.021763
0.014600 0.011358
y17 -0.020796 0.015447 -0.004902 -0.005298 0.056640
0.012503 -0.060359 -0.021770 -0.006156 0.026220 0.034215
0.014383 -0.025736 -0.020252 -0.033718 0.015365 -0.004176 -
0.039965 -0.028464 -0.040618 -0.003175 -0.022048 0.002540 -
0.005491 -0.002615 -0.036957 0.014458 0.003400 0.015762 -
0.024078 -0.009822 0.020534 -0.035116 0.026186 -0.004534 -
0.009892 -0.000114
z17 0.047126 0.000551 0.024027 -0.054008 0.008046 -
0.036685 -0.014456 0.007452 -0.013696 -0.007756 -0.007678

0.004757	0.009295	-0.009586	-0.020260	0.031897	0.002685	-
0.008675	0.020748	-0.001344	0.000070	-0.005903	-0.016901	-
0.003863	0.026774	-0.012148	-0.033727	0.002935	0.018455	-
0.017027	-0.030498	-0.008887	-0.008623	0.014618	0.017009	
0.008043	-0.011489					
x18	-0.013725	0.007479	0.027543	-0.000475	0.001028	
0.020419	-0.045581	0.057117	-0.008463	0.006413	0.034214	-
0.012507	0.021272	0.014660	-0.007458	0.016298	0.016010	
0.030330	-0.024549	-0.007867	0.019961	-0.043037	0.003301	
0.038011	0.027026	0.009675	-0.017210	-0.012035	0.028607	
0.027934	-0.007583	0.043396	-0.006350	0.012607	0.014729	
0.023314	0.023308					
y18	-0.023399	0.013885	-0.006540	-0.001905	0.062023	
0.010242	-0.055231	-0.020893	-0.013048	0.019396	0.034895	
0.010149	-0.032240	-0.016914	-0.022413	0.011037	-0.010413	-
0.031134	-0.026603	-0.039793	-0.002261	-0.021816	-0.001275	
0.002441	0.002057	-0.034868	0.009804	0.000373	-0.001046	-
0.023500	-0.017355	0.024207	-0.040706	0.032008	-0.006441	-
0.016596	-0.005663					
z18	0.045199	0.001422	0.024304	-0.054942	0.015258	-
0.038569	-0.020641	0.010196	-0.016300	-0.010896	-0.003281	
0.001468	0.004710	-0.011725	-0.018728	0.031660	0.000828	-
0.004746	0.017862	0.001634	-0.001499	-0.009369	-0.025878	-
0.002620	0.033318	-0.015538	-0.038567	0.001020	0.010313	-
0.024176	-0.026291	-0.005595	-0.020391	0.014787	0.020230	
0.006525	-0.009705					
x19	-0.012209	0.006427	0.027540	-0.003792	0.013144	
0.019595	-0.050136	0.062811	-0.021110	0.005478	0.032945	-
0.016304	0.018516	0.012585	-0.010069	0.016661	0.011901	
0.025317	-0.033999	-0.014265	0.022359	-0.049773	-0.004164	
0.034725	0.027947	0.012775	-0.014587	-0.013490	0.019499	
0.024875	-0.029128	0.047384	-0.018223	0.015865	0.010966	
0.028974	0.022279					
y19	-0.025011	0.012116	-0.006847	0.002189	0.064725	
0.007911	-0.046901	-0.020044	-0.021154	0.010672	0.033122	
0.006884	-0.038059	-0.015420	-0.006322	0.005144	-0.013401	-
0.017552	-0.022084	-0.034769	-0.002785	-0.020407	-0.005799	
0.013411	0.009834	-0.031509	0.003138	-0.003566	-0.022457	-
0.021177	-0.025760	0.026030	-0.043853	0.038173	-0.009913	-
0.023892	-0.004797					
z19	0.043463	0.001224	0.023429	-0.054317	0.023622	-
0.041239	-0.027712	0.013619	-0.017771	-0.014695	0.003290	-
0.001304	-0.000943	-0.011372	-0.016522	0.030481	-0.003917	-
0.001020	0.012036	0.002161	-0.003549	-0.011662	-0.036043	-
0.002045	0.041879	-0.019703	-0.043584	0.000883	-0.001483	-
0.033832	-0.019260	-0.000568	-0.033140	0.015041	0.020914	
0.004777	-0.011832					
x20	-0.009803	0.002006	0.026556	-0.005839	0.027203	
0.017441	-0.055312	0.068773	-0.030644	0.006417	0.037984	-
0.018961	0.011713	0.004781	-0.008410	0.020668	0.003475	
0.023970	-0.043451	-0.022482	0.023100	-0.047807	-0.016429	
0.030320	0.035457	0.012379	-0.014112	-0.010404	-0.003443	
0.012023	-0.050720	0.052277	-0.031015	0.023927	0.002030	
0.032622	0.035369					
y20	-0.026291	0.011409	-0.006821	0.007449	0.065960	
0.004076	-0.036139	-0.018315	-0.030508	0.000400	0.028287	
0.001620	-0.041376	-0.010971	0.011091	-0.004294	-0.015847	-
0.002538	-0.017694	-0.028013	-0.001929	-0.021548	-0.008704	
0.028417	0.015063	-0.025484	-0.004903	-0.010139	-0.042037	-

0.012425 -0.043628 0.023273 -0.042869 0.043353 -0.012055 -
0.027393 -0.002607
z20 0.041660 0.001384 0.022417 -0.051838 0.032082 -
0.043669 -0.034374 0.018855 -0.020112 -0.020559 0.008599 -
0.003791 -0.007530 -0.008968 -0.013081 0.026366 -0.010317
0.002546 0.004924 0.001388 -0.005145 -0.015779 -0.046206 -
0.000125 0.051014 -0.023226 -0.049914 -0.001418 -0.016670 -
0.042654 -0.008659 0.010831 -0.043332 0.012493 0.019789
0.005000 -0.024382
x21 -0.012720 -0.003174 0.031128 -0.011200 0.031817
0.018146 -0.058287 0.069290 -0.033071 0.010995 0.032818 -
0.020909 0.009663 0.002490 -0.010618 0.014435 0.001655
0.008846 -0.042123 -0.014658 0.013342 -0.031824 -0.025321
0.010844 0.023019 -0.001456 -0.005994 0.001917 -0.016275
0.010702 -0.031879 0.034207 -0.030906 0.024597 0.002888
0.010313 0.007063
y21 -0.030402 0.008913 -0.006308 0.016442 0.051367
0.004338 -0.017282 -0.027027 -0.028449 -0.008338 0.009071
0.002333 -0.037753 -0.006508 0.017981 -0.015659 -0.005357 -
0.001329 -0.006477 -0.016377 -0.004985 -0.010840 -0.000224
0.025849 0.005286 -0.025487 -0.009270 -0.003367 -0.037157 -
0.003687 -0.021741 0.012754 -0.017365 0.022508 -0.002620 -
0.022249 -0.008828
z21 0.032949 0.002488 0.015594 -0.047726 0.038147 -
0.046879 -0.027335 0.009767 -0.023338 -0.023044 0.004695 -
0.007219 -0.013561 -0.011932 -0.015976 0.018213 -0.018811 -
0.000137 -0.005967 0.013604 -0.009165 -0.002952 -0.047007 -
0.007611 0.034759 -0.019492 -0.036131 0.000385 -0.011617 -
0.027513 0.011901 0.002395 -0.034872 0.013760 0.008237 -
0.004080 -0.015469
x22 -0.018787 -0.007889 0.033785 -0.013762 0.036001
0.017424 -0.058446 0.066274 -0.029413 0.013047 0.025074 -
0.024936 0.010130 0.005587 -0.010687 0.010833 -0.000021 -
0.009589 -0.042279 -0.001902 0.007181 -0.022096 -0.022761 -
0.002384 0.011351 -0.023689 0.006630 0.019322 -0.030817
0.018626 -0.018993 0.009369 -0.012534 0.032511 -0.004570 -
0.006084 -0.006021
y22 -0.030530 0.007574 -0.004171 0.022704 0.039700
0.006031 -0.000466 -0.032656 -0.027113 -0.016345 -0.004386
0.002953 -0.035978 -0.005655 0.025431 -0.021950 0.004343
0.004095 0.003624 -0.010947 -0.005660 -0.001952 0.005161
0.026628 -0.001985 -0.018325 -0.018319 -0.004835 -0.030131 -
0.004062 -0.013118 0.006182 -0.009634 0.002232 0.010560 -
0.009085 -0.005147
z22 0.026265 0.003490 0.010776 -0.043223 0.041654 -
0.047848 -0.020612 0.002701 -0.025416 -0.024032 0.002247 -
0.009452 -0.019451 -0.015529 -0.018443 0.010272 -0.025690
0.001393 -0.013886 0.021765 -0.013710 0.009650 -0.051904 -
0.011561 0.020302 -0.010192 -0.024335 -0.002296 -0.007076 -
0.015924 0.036525 -0.001754 -0.036019 0.011481 0.004864 -
0.011509 -0.017895
x23 -0.023356 -0.011289 0.035522 -0.014233 0.040852
0.018160 -0.055906 0.058264 -0.023796 0.013160 0.015831 -
0.028010 0.007642 0.006088 -0.012066 0.009481 0.000722 -
0.017613 -0.039810 0.011755 0.003591 -0.006636 -0.024036 -
0.011821 -0.000897 -0.030842 0.016038 0.029022 -0.044935
0.026039 -0.006260 -0.011586 -0.003130 0.040660 -0.008047 -
0.016090 -0.018999
y23 -0.030269 0.005406 -0.001696 0.026524 0.028167
0.006610 0.011322 -0.033816 -0.026531 -0.020070 -0.013818

0.002704	-0.030463	-0.002559	0.029148	-0.027733	0.011038	
0.004289	0.008832	-0.011580	-0.006542	-0.000814	0.009584	
0.021997	-0.007816	-0.018165	-0.023618	-0.008860	-0.017185	-
0.008472	-0.006160	-0.002484	-0.002255	-0.020915	0.021830	-
0.003909	-0.003143					
z23	0.020874	0.004335	0.007363	-0.039575	0.045058	-
0.048275	-0.014597	-0.000930	-0.025939	-0.025528	0.001496	-
0.011046	-0.024370	-0.016937	-0.021164	0.000687	-0.033114	-
0.000538	-0.021413	0.023837	-0.015243	0.012542	-0.053929	-
0.012446	0.012106	-0.003934	-0.016179	-0.002343	0.002417	-
0.007531	0.053032	-0.002147	-0.035301	0.004939	0.003346	-
0.017394	-0.015884					
x24	-0.024718	-0.014354	0.035869	-0.015027	0.046670	
0.018248	-0.053265	0.049230	-0.019090	0.011505	0.007169	-
0.028454	0.001560	0.007250	-0.017786	0.006721	0.000159	-
0.024065	-0.033128	0.018001	0.005016	-0.000552	-0.028902	-
0.015652	-0.012753	-0.034827	0.021197	0.030700	-0.051944	
0.022430	0.008695	-0.029506	0.002265	0.036584	-0.003006	-
0.022909	-0.024966					
y24	-0.032083	0.005028	0.003135	0.030654	0.013029	
0.007757	0.022657	-0.033655	-0.025755	-0.020939	-0.022740	
0.002239	-0.019301	-0.004718	0.037993	-0.032419	0.020535	
0.005285	0.010403	-0.004488	-0.015044	0.007958	0.013019	
0.014028	-0.013065	-0.014967	-0.022564	-0.007475	-0.011151	-
0.003844	-0.009408	-0.011423	0.002925	-0.029687	0.018018	-
0.003776	-0.001980					
z24	0.014754	0.006816	0.006723	-0.034017	0.044846	-
0.049084	-0.006615	-0.005459	-0.024164	-0.028069	0.001755	-
0.011814	-0.026361	-0.018709	-0.018069	-0.011214	-0.036774	-
0.001727	-0.027966	0.031116	-0.021429	0.022543	-0.053126	-
0.013724	0.005962	0.007467	-0.009706	0.004234	0.004424	
0.010108	0.057608	-0.004347	-0.032796	0.005221	-0.009402	-
0.021010	-0.010156					
x25	-0.023861	-0.013668	0.034995	-0.014790	0.050121	
0.015736	-0.048976	0.038204	-0.016530	0.004690	0.000909	-
0.026182	-0.003639	0.004365	-0.022814	0.000117	-0.000966	-
0.029060	-0.029747	0.025076	0.004944	-0.000620	-0.035249	-
0.015620	-0.022837	-0.028053	0.019865	0.036839	-0.056044	
0.023275	0.021221	-0.042265	0.002983	0.025609	-0.016438	-
0.019714	-0.031520					
y25	-0.035219	0.001303	0.004871	0.032558	0.002654	
0.011319	0.032995	-0.028257	-0.020330	-0.013363	-0.028735	
0.003397	-0.006354	-0.001278	0.044465	-0.030422	0.015852	
0.002932	0.016105	0.000750	-0.011264	0.018321	0.026304	
0.007384	-0.015547	-0.022824	-0.020184	-0.005999	-0.001198	-
0.005906	-0.010415	-0.002545	0.002216	-0.027004	0.036914	-
0.005810	-0.008422					
z25	0.007566	0.006371	0.004578	-0.028169	0.049084	-
0.048870	0.001099	-0.008860	-0.015989	-0.024365	0.000359	-
0.014283	-0.032437	-0.011259	-0.013025	-0.020232	-0.037579	-
0.002387	-0.026687	0.038350	-0.021178	0.032926	-0.045451	-
0.014965	-0.002696	0.012770	-0.005210	0.000652	0.001875	
0.023259	0.063245	-0.008591	-0.025655	0.009971	-0.008378	-
0.026981	-0.004986					
x26	-0.023852	-0.013119	0.034451	-0.014893	0.058092	
0.009170	-0.041461	0.023683	-0.014201	-0.004125	-0.005843	-
0.020681	-0.010281	0.003553	-0.023984	-0.009971	-0.002039	-
0.025784	-0.021827	0.033554	0.005851	0.005015	-0.037280	-
0.018604	-0.032063	-0.016436	0.018381	0.028104	-0.056383	

0.022179 0.028669 -0.054120 -0.000096 0.020078 -0.024394 -
0.023104 -0.028066
y26 -0.036239 0.001757 0.011233 0.035449 -0.014189
0.021482 0.037972 -0.018249 -0.019887 -0.006403 -0.035993 -
0.000450 0.003421 -0.012651 0.054619 -0.020438 0.031021
0.003291 0.020309 0.012686 -0.010382 0.015378 0.034370
0.001709 -0.015895 -0.026068 -0.010147 -0.001762 0.008002 -
0.009675 -0.021042 -0.010517 0.005060 -0.020194 0.045965 -
0.012411 -0.008778
z26 0.003217 0.008562 0.008786 -0.020709 0.046346 -
0.040993 0.007088 -0.008522 -0.003807 -0.023245 -0.004131 -
0.020600 -0.040608 -0.012732 -0.005167 -0.023662 -0.023780 -
0.006983 -0.024870 0.050086 -0.023591 0.031420 -0.038972 -
0.008934 -0.005290 0.018544 -0.004787 0.014163 -0.001281
0.038203 0.056571 -0.007882 -0.017216 0.012216 -0.009143 -
0.033039 0.003075
x27 -0.022431 -0.011456 0.035639 -0.015394 0.068056
0.002170 -0.032493 0.008860 -0.008527 -0.015081 -0.014513 -
0.013916 -0.016743 0.006273 -0.025578 -0.019888 -0.002955 -
0.024057 -0.015097 0.042309 0.004550 0.010609 -0.038830 -
0.020687 -0.036061 0.002583 0.009770 0.018151 -0.053678
0.023321 0.027741 -0.058411 -0.007603 0.014269 -0.027909 -
0.029400 -0.021387
y27 -0.036895 -0.000033 0.010400 0.037337 -0.026763
0.028212 0.037238 -0.004876 -0.016989 0.008048 -0.032238 -
0.001694 0.021297 -0.012602 0.053049 -0.005580 0.024928
0.001769 0.015627 0.010077 -0.001996 0.014783 0.037856
0.004109 -0.014587 -0.032412 0.000368 -0.002992 0.012557 -
0.016800 -0.025388 -0.013588 -0.002566 -0.015611 0.053252 -
0.000598 -0.013610
z27 0.001177 0.008418 0.010858 -0.010533 0.045138 -
0.037118 0.010703 -0.005594 0.012486 -0.020023 -0.002288 -
0.022310 -0.043303 -0.011189 -0.006643 -0.026113 -0.021607 -
0.007051 -0.027194 0.050241 -0.017102 0.029655 -0.037321
0.005963 -0.002986 0.025952 -0.000163 0.022485 -0.009822
0.049073 0.053628 -0.007167 -0.014491 0.011974 -0.007010 -
0.022024 0.016007
x28 -0.019267 -0.007790 0.035794 -0.014068 0.078623 -
0.005178 -0.021050 -0.008721 -0.001876 -0.028399 -0.022401 -
0.006924 -0.023773 0.010015 -0.031692 -0.033007 -0.004949 -
0.019105 -0.009552 0.048423 0.004848 0.010499 -0.041278 -
0.017524 -0.036633 0.031773 -0.004837 0.004446 -0.047456
0.025231 0.022883 -0.059092 -0.015387 0.004516 -0.032941 -
0.025342 -0.009595
y28 -0.038218 -0.001512 0.009737 0.035010 -0.040554
0.035708 0.034218 0.007867 -0.015445 0.022095 -0.024659 -
0.002997 0.042910 -0.014186 0.049951 0.008448 0.015176 -
0.002629 0.010981 0.001988 0.003475 0.012906 0.044494
0.001375 -0.013437 -0.040720 0.011066 -0.005341 0.025117 -
0.021939 -0.034245 -0.008417 -0.008465 -0.006854 0.067168
0.008735 -0.024682
z28 -0.001355 0.008327 0.011712 -0.002867 0.042048 -
0.036082 0.014790 -0.007317 0.029870 -0.020998 0.004422 -
0.024165 -0.043378 -0.005611 -0.011446 -0.035025 -0.020918 -
0.009808 -0.025374 0.044501 -0.012168 0.025228 -0.030627
0.014873 -0.001018 0.040277 0.002603 0.025824 -0.006156
0.062069 0.045818 -0.007710 -0.003408 0.004356 0.002858 -
0.011772 0.016289
x29 -0.015627 -0.002750 0.033987 -0.010710 0.088417 -
0.015583 -0.007288 -0.029363 0.002343 -0.046394 -0.031291

0.002527	-0.032379	0.011853	-0.035878	-0.045534	-0.009298	-
0.009004	-0.005308	0.052074	0.006995	0.009147	-0.043568	-
0.011189	-0.036815	0.064786	-0.023560	-0.016462	-0.037115	
0.024870	0.017692	-0.055813	-0.027220	-0.007501	-0.043704	-
0.017717	0.007012					
y29	-0.039818	-0.002309	0.011765	0.030898	-0.056175	
0.045779	0.029291	0.024021	-0.018886	0.036854	-0.019181	-
0.003020	0.067408	-0.019785	0.051378	0.029810	0.005720	-
0.004249	0.007467	-0.006229	0.010781	0.013862	0.052807	-
0.003129	-0.013699	-0.058841	0.024530	-0.003945	0.037454	-
0.032973	-0.039397	0.000504	-0.018157	0.011818	0.073853	
0.011365	-0.039654					
z29	-0.003781	0.008450	0.015478	0.002613	0.035518	-
0.032970	0.018137	-0.010793	0.040985	-0.024182	0.007642	-
0.022382	-0.042926	-0.000122	-0.004892	-0.039746	-0.015203	-
0.009169	-0.015673	0.033879	-0.008962	0.026053	-0.022859	
0.025802	-0.008898	0.040365	0.007867	0.027996	-0.002235	
0.070438	0.056004	-0.001745	0.005377	0.000849	0.001731	-
0.008575	0.013465					
x30	-0.020355	-0.005075	0.029672	-0.004530	0.071357	-
0.011711	-0.005715	-0.027260	-0.001674	-0.032957	-0.031983	
0.008941	-0.017420	0.006967	-0.025481	-0.040195	-0.008213	-
0.004891	-0.008712	0.044158	0.007781	0.016795	-0.028763	-
0.011513	-0.039688	0.053066	-0.003529	-0.029117	-0.027749	
0.016149	0.010147	-0.055864	-0.024332	-0.001127	-0.016873	-
0.011481	0.001610					
y30	-0.036758	-0.001450	0.013986	0.028253	-0.046261	
0.039401	0.028541	0.022639	-0.012345	0.030329	-0.020002	-
0.008601	0.060718	-0.022960	0.050264	0.029382	0.005449	-
0.000221	0.002463	-0.012543	0.008264	0.012424	0.039746	
0.000488	-0.016376	-0.059666	0.010872	-0.000380	0.029510	-
0.032559	-0.033956	0.009194	-0.025081	0.006348	0.049210	
0.022005	-0.032537					
z30	-0.008550	0.005529	0.018898	0.006842	0.020536	-
0.025261	0.011693	-0.004237	0.039481	-0.019033	0.004519	-
0.019504	-0.032647	0.001897	0.006423	-0.035560	-0.014884	-
0.014645	-0.016354	0.018827	-0.010780	0.033860	-0.013917	
0.024148	-0.011192	0.020051	0.024038	0.034410	0.005370	
0.068707	0.045355	0.000759	0.006755	0.004203	0.011079	-
0.005721	0.016166					
x31	-0.023991	-0.006447	0.026712	0.000739	0.056662	-
0.007068	-0.005327	-0.024788	-0.004140	-0.020166	-0.030323	
0.014164	-0.003779	0.001590	-0.017992	-0.037231	-0.006646	-
0.001663	-0.012632	0.035324	0.006550	0.023314	-0.015442	-
0.012089	-0.044333	0.042894	0.010605	-0.037873	-0.019045	
0.009332	0.002632	-0.054309	-0.019005	0.004586	0.002781	-
0.007302	-0.000911					
y31	-0.032963	-0.000263	0.016321	0.026060	-0.037583	
0.033888	0.029096	0.022150	-0.007142	0.024540	-0.019403	-
0.012880	0.053369	-0.023668	0.047419	0.027474	0.005621	
0.002606	-0.001832	-0.017347	0.005542	0.010514	0.029314	
0.004394	-0.017103	-0.059237	-0.001022	0.002340	0.024514	-
0.030337	-0.026241	0.018546	-0.030620	0.001934	0.023694	
0.029915	-0.025356					
z31	-0.010598	0.005036	0.024873	0.011335	0.008772	-
0.017267	0.007514	0.004362	0.039724	-0.013208	0.004440	-
0.020769	-0.023057	0.003464	0.017070	-0.036212	-0.015011	-
0.020565	-0.017011	0.003547	-0.015801	0.035415	-0.001206	
0.025132	-0.010962	-0.000576	0.028271	0.042351	0.013946	

0.065983 0.036945 0.005696 0.012031 0.007756 0.001811 -
0.008536 0.024363
x32 -0.026845 -0.007979 0.024747 0.007424 0.043309 -
0.003695 -0.006028 -0.019779 -0.007414 -0.011160 -0.029971
0.016937 0.006327 -0.000760 -0.011354 -0.032564 -0.005992 -
0.000832 -0.015707 0.024906 0.004126 0.028458 -0.004352 -
0.008294 -0.043572 0.028779 0.021074 -0.042657 -0.012207
0.005767 -0.001710 -0.046844 -0.017375 0.011285 0.020223 -
0.006495 -0.006265
y32 -0.029356 0.000130 0.016787 0.024615 -0.030154
0.028396 0.030440 0.021270 -0.003664 0.019109 -0.018830 -
0.015198 0.044759 -0.022525 0.043798 0.027150 0.006264
0.006897 -0.006254 -0.019735 0.003489 0.010127 0.020170
0.007348 -0.016269 -0.055685 -0.008747 0.000799 0.021313 -
0.027148 -0.017982 0.025112 -0.035379 -0.000469 0.005793
0.037426 -0.021612
z32 -0.012625 0.004203 0.028631 0.016199 -0.004680 -
0.011046 0.001487 0.015144 0.035953 -0.008644 0.003154 -
0.017455 -0.014775 0.008151 0.025622 -0.031674 -0.009854 -
0.029125 -0.018608 -0.009509 -0.018938 0.034925 0.010530
0.030397 -0.005067 -0.023272 0.033216 0.048326 0.020551
0.059980 0.029274 0.014279 0.008061 0.019557 0.007326 -
0.001268 0.017753
x33 -0.029068 -0.008643 0.022276 0.014455 0.032175
0.000838 -0.005409 -0.014385 -0.011662 -0.004270 -0.027612
0.017748 0.014048 -0.000686 -0.006232 -0.030460 -0.001472 -
0.001045 -0.015926 0.018136 0.003039 0.028925 0.003681 -
0.004227 -0.039072 0.019215 0.025684 -0.039760 -0.004642
0.005324 -0.000581 -0.041638 -0.011457 0.017194 0.025757
0.000572 -0.011174
y33 -0.025002 -0.000122 0.016713 0.022421 -0.023680
0.022950 0.032200 0.019056 0.000280 0.014442 -0.016634 -
0.016146 0.035137 -0.021214 0.039345 0.027162 0.006099
0.011516 -0.010168 -0.019962 0.001709 0.009983 0.012804
0.009489 -0.016162 -0.049433 -0.013373 -0.003550 0.017994 -
0.023885 -0.011477 0.028458 -0.037176 -0.003944 -0.009576
0.039792 -0.017542
z33 -0.012862 0.004890 0.029875 0.020172 -0.014456 -
0.004669 -0.000392 0.023147 0.032561 -0.007327 0.002964 -
0.015106 -0.010274 0.013051 0.031403 -0.030484 -0.002736 -
0.033513 -0.016839 -0.014662 -0.018773 0.029274 0.017843
0.033133 0.003474 -0.036539 0.033806 0.050597 0.026921
0.054025 0.028856 0.014489 0.008281 0.027352 0.002241
0.008478 0.013490
x34 -0.029792 -0.009536 0.019832 0.020351 0.023756
0.005248 -0.003410 -0.011091 -0.012152 -0.000025 -0.025068
0.016985 0.018706 0.000473 -0.003021 -0.026038 0.002346 -
0.002907 -0.015843 0.011957 0.001964 0.028919 0.008794 -
0.000365 -0.034178 0.013828 0.027718 -0.038595 0.003379
0.005648 0.003375 -0.039249 -0.012029 0.018297 0.026547 -
0.000374 -0.011893
y34 -0.020473 -0.000329 0.016142 0.020338 -0.018555
0.017027 0.033309 0.017364 0.003692 0.010818 -0.013594 -
0.015590 0.025952 -0.019629 0.034089 0.026951 0.005855
0.015586 -0.014427 -0.019520 -0.001432 0.009034 0.008707
0.011142 -0.014609 -0.042296 -0.018635 -0.006448 0.014267 -
0.019900 -0.008394 0.031610 -0.034343 -0.005518 -0.020646
0.037846 -0.015989
z34 -0.010610 0.005559 0.029252 0.021516 -0.020664 -
0.000733 0.000408 0.026976 0.033770 -0.008390 0.002666 -

0.012128	-0.010190	0.016514	0.035004	-0.026902	0.002587	-
0.035183	-0.015773	-0.016535	-0.016617	0.024181	0.021470	
0.034704	0.010369	-0.041658	0.032286	0.042170	0.032337	
0.043812	0.029733	0.009279	0.001553	0.029390	-0.000059	
0.005646	0.016127					
x35	-0.029970	-0.009902	0.017218	0.024138	0.018932	
0.008477	-0.002344	-0.008047	-0.010383	0.001450	-0.019776	
0.015458	0.020837	0.000564	-0.003242	-0.023419	0.004264	-
0.006627	-0.013186	0.007830	-0.002078	0.027430	0.012670	
0.002540	-0.028854	0.012244	0.024753	-0.034916	0.005906	
0.009050	0.005714	-0.036872	-0.007639	0.017833	0.025078	-
0.008536	-0.011015					
y35	-0.015601	-0.000514	0.015674	0.018017	-0.014507	
0.010936	0.033687	0.014741	0.006021	0.008241	-0.010888	-
0.013885	0.016629	-0.016729	0.027166	0.027021	0.006741	
0.019813	-0.019727	-0.017673	-0.004738	0.007794	0.005459	
0.012183	-0.012827	-0.034480	-0.023739	-0.007838	0.012238	-
0.014901	-0.005077	0.035282	-0.030205	-0.005073	-0.028866	
0.034240	-0.018874					
z35	-0.007235	0.006790	0.026961	0.018988	-0.022236	
0.000695	-0.000189	0.028264	0.036295	-0.011038	0.005109	-
0.005847	-0.013021	0.015972	0.034624	-0.025712	0.007320	-
0.034489	-0.012421	-0.012110	-0.014619	0.016934	0.022089	
0.031983	0.013761	-0.042016	0.025605	0.031349	0.030639	
0.029358	0.026198	0.002524	0.000843	0.030866	0.003107	-
0.001197	0.014561					
x36	-0.029098	-0.009864	0.014249	0.027065	0.014811	
0.010221	-0.002955	-0.004298	-0.008104	-0.000213	-0.015036	
0.011541	0.021786	0.001897	-0.009005	-0.019280	0.006299	-
0.012276	-0.010956	0.001694	-0.007321	0.024456	0.011442	
0.011144	-0.020197	0.007063	0.017503	-0.025616	0.008915	
0.009873	0.010107	-0.033798	-0.004971	0.014980	0.018240	-
0.018379	-0.007512					
y36	-0.010468	-0.001105	0.014389	0.015879	-0.010709	
0.005865	0.035921	0.010347	0.007303	0.006949	-0.006834	-
0.009626	0.006018	-0.012109	0.021780	0.025561	0.008096	
0.027824	-0.023389	-0.013685	-0.007362	0.007848	0.005208	
0.007212	-0.011954	-0.020462	-0.025047	-0.016217	0.010005	-
0.004038	-0.002510	0.035934	-0.022586	0.000403	-0.032132	
0.027688	-0.024735					
z36	-0.002731	0.007949	0.022821	0.015767	-0.023484	-
0.000092	-0.001584	0.028773	0.038245	-0.015742	0.006055	
0.001192	-0.017244	0.014604	0.030631	-0.021519	0.012061	-
0.031402	-0.010785	-0.005200	-0.010333	0.008426	0.018686	
0.032124	0.017999	-0.042025	0.018000	0.015891	0.028187	
0.008917	0.022605	-0.007835	-0.004181	0.031352	0.007388	-
0.006283	0.011734					
x37	-0.027367	-0.010210	0.013401	0.030095	0.011385	
0.014583	-0.002485	-0.002579	-0.007196	-0.003097	-0.008808	
0.008352	0.020304	0.003206	-0.012737	-0.016311	0.009728	-
0.014952	-0.012414	-0.005364	-0.008437	0.022699	0.011002	
0.012614	-0.011832	0.007400	0.012032	-0.015985	0.009945	
0.016614	0.008785	-0.028477	0.002990	0.014512	0.017036	-
0.029017	-0.008006					
y37	-0.004092	-0.000326	0.011305	0.013690	-0.008236	-
0.000124	0.038008	0.008320	0.009867	0.006499	-0.003051	-
0.006461	-0.005053	-0.004724	0.012474	0.023231	0.010615	
0.030241	-0.025357	-0.006638	-0.013619	0.000358	0.003823	
0.011054	-0.008817	-0.007847	-0.031903	-0.019064	0.006642	

0.004412 0.005142 0.034751 -0.014435 0.002807 -0.043135
0.023146 -0.035021
z37 0.003330 0.009333 0.018218 0.011051 -0.022993 -
0.000051 -0.001376 0.025815 0.039496 -0.021185 0.009582
0.009963 -0.022951 0.011166 0.026684 -0.018999 0.018904 -
0.023279 -0.010049 0.004793 -0.003625 -0.002664 0.014080
0.025407 0.020904 -0.034744 0.008693 -0.007798 0.022415 -
0.013000 0.015835 -0.019669 -0.007569 0.030648 0.018272 -
0.011782 0.003798
x38 -0.023576 -0.009423 0.013938 0.033166 0.009737
0.019797 -0.004749 -0.001170 -0.008952 -0.004516 -0.002608
0.001851 0.020054 0.008885 -0.021649 -0.017837 0.015915 -
0.014997 -0.015227 -0.010364 -0.010749 0.015424 0.007966
0.020399 -0.006915 0.008628 -0.000465 -0.014986 0.005792
0.027864 0.013920 -0.022272 0.004548 0.014602 -0.000867 -
0.036682 -0.012826
y38 0.001209 -0.001752 0.007511 0.010013 -0.005087 -
0.007385 0.040533 0.005712 0.015648 0.002381 0.003518
0.000962 -0.020895 -0.000343 0.003936 0.026630 0.009692
0.027119 -0.023535 0.001390 -0.021932 0.001764 0.007428
0.004849 0.000188 0.007080 -0.031105 -0.012389 0.010980
0.011641 0.006355 0.030642 -0.002180 0.002187 -0.039575
0.000874 -0.029194
z38 0.010713 0.012235 0.012672 0.003512 -0.019610 -
0.003345 0.000773 0.021714 0.042506 -0.027527 0.012255
0.019880 -0.031905 0.008109 0.016074 -0.016357 0.026798 -
0.013738 -0.008433 0.022467 0.005669 -0.016432 0.007436
0.022996 0.022611 -0.021584 -0.004958 -0.037065 0.013231 -
0.033689 0.015840 -0.037906 -0.012690 0.031538 0.026284 -
0.020175 -0.008580
x39 -0.022216 -0.008318 0.011243 0.033319 0.005768
0.023290 -0.004413 0.002622 -0.006179 -0.004342 -0.007486
0.003917 0.018582 0.009695 -0.033572 -0.016884 0.016408 -
0.015945 -0.017780 -0.006837 -0.003632 0.006832 0.001258
0.024160 -0.009036 0.009909 -0.002251 -0.013448 -0.001649
0.030470 0.010127 -0.033738 0.015116 0.003744 0.008544 -
0.022771 -0.014070
y39 0.001760 -0.002649 0.007677 0.012866 -0.006430 -
0.012800 0.037193 0.007258 0.023248 -0.001271 0.005994 -
0.000435 -0.018125 0.002328 0.005772 0.021383 0.007732
0.020916 -0.022050 -0.004074 -0.020446 0.013724 0.016378 -
0.003367 0.009917 0.004618 -0.024926 -0.008643 0.019541
0.013119 0.012070 0.006764 0.004175 0.004705 -0.021697 -
0.003145 -0.021028
z39 0.012662 0.009363 0.012675 0.003649 -0.017664
0.001168 -0.001328 0.019456 0.037981 -0.020458 0.002370
0.015851 -0.028671 0.004707 0.011691 -0.012178 0.018222 -
0.017557 -0.016371 0.006023 -0.003293 -0.011898 0.000855
0.017361 0.024498 -0.022960 0.006365 -0.033054 0.011387 -
0.025968 0.001564 -0.028430 -0.012592 0.018357 0.028037 -
0.026713 -0.027388
x40 -0.020556 -0.008400 0.010007 0.033230 0.003815
0.024470 -0.004487 0.008149 -0.004923 0.000542 -0.007738
0.003678 0.012881 0.008208 -0.041948 -0.015957 0.019942 -
0.013954 -0.016633 -0.010761 -0.004195 0.007949 -0.000356
0.017136 -0.005340 0.015340 0.003042 -0.001755 -0.012924
0.037776 0.003071 -0.051406 0.031804 0.001049 0.012020 -
0.014794 -0.013672
y40 0.001089 -0.002638 0.007580 0.015904 -0.008674 -
0.016081 0.032988 0.006330 0.029060 -0.008210 0.005784 -

0.000086	-0.010832	0.005901	0.003586	0.017171	0.004852	
0.013985	-0.025803	-0.001641	-0.014592	0.016598	0.017430	-
0.000979	0.013249	0.001736	-0.027172	-0.015571	0.031277	
0.009374	0.016709	-0.007011	0.001496	-0.001970	-0.015866	-
0.006704	-0.006999					
z40	0.015119	0.006832	0.011730	0.003735	-0.016798	
0.004536	-0.003247	0.018071	0.035821	-0.012837	-0.005818	
0.013495	-0.026495	0.001901	0.010063	-0.009884	0.011984	-
0.019256	-0.019687	-0.010943	-0.011574	-0.004866	-0.002104	
0.009817	0.026645	-0.023128	0.017637	-0.023222	0.008573	-
0.016845	-0.008966	-0.025423	-0.011457	0.011602	0.028096	-
0.029939	-0.040058					
x41	-0.020086	-0.008764	0.008182	0.031752	0.002970	
0.026619	-0.002854	0.011459	-0.003618	0.003685	-0.004672	
0.004605	0.006267	0.009659	-0.047720	-0.015803	0.019520	-
0.012665	-0.014403	-0.013332	-0.003822	0.011262	0.001831	
0.013926	-0.001144	0.018878	0.001876	0.007692	-0.017219	
0.042167	0.000560	-0.061065	0.040414	-0.002674	0.011245	-
0.006573	-0.011995					
y41	0.001533	-0.001134	0.007854	0.019600	-0.012780	-
0.019332	0.029264	0.006718	0.033688	-0.013653	0.002714	-
0.001691	-0.003009	0.005405	0.002418	0.013924	0.005092	
0.008468	-0.030172	-0.000116	-0.010632	0.015640	0.014118	
0.000050	0.014022	-0.000610	-0.027070	-0.019590	0.033175	
0.008328	0.019564	-0.019771	0.004382	-0.006122	-0.005407	-
0.011588	-0.006338					
z41	0.017605	0.004179	0.010795	0.002764	-0.015070	
0.008306	-0.004421	0.017039	0.032632	-0.006203	-0.011516	
0.012693	-0.025680	0.000837	0.008083	-0.007605	0.006344	-
0.019362	-0.022253	-0.024411	-0.017466	0.002450	-0.002797	
0.004504	0.028887	-0.022157	0.026900	-0.014234	0.007956	-
0.009898	-0.018691	-0.021489	-0.010988	0.006662	0.026045	-
0.028843	-0.046987					
x42	-0.020580	-0.008793	0.005255	0.031998	0.001376	
0.029052	-0.000480	0.011862	-0.001528	0.001894	-0.001592	
0.005249	0.001405	0.009904	-0.049629	-0.015224	0.019848	-
0.012421	-0.011979	-0.012294	-0.003722	0.012406	-0.000095	
0.008646	0.001168	0.018794	-0.004869	0.011134	-0.018882	
0.042959	-0.001169	-0.066195	0.044485	-0.004529	0.016238	-
0.001192	-0.009983					
y42	0.002747	0.000045	0.008718	0.021273	-0.016662	-
0.021408	0.025449	0.008213	0.035664	-0.013826	0.000344	-
0.002124	0.002359	0.005494	0.000498	0.011175	0.004137	
0.005508	-0.032401	-0.002678	-0.009807	0.017144	0.013582	
0.001449	0.015524	0.000368	-0.023583	-0.018114	0.031851	
0.010090	0.022209	-0.029364	0.009829	-0.011867	-0.005013	-
0.016558	-0.011023					
z42	0.020319	0.001732	0.009106	0.001655	-0.012996	
0.012659	-0.004425	0.015493	0.029649	-0.002563	-0.014479	
0.012278	-0.025000	-0.000395	0.007955	-0.007042	0.001734	-
0.018351	-0.023670	-0.033547	-0.021932	0.007825	-0.004545	-
0.002630	0.029494	-0.021461	0.030934	-0.008917	0.008844	-
0.006357	-0.027924	-0.016708	-0.009288	0.000991	0.025716	-
0.024487	-0.047827					
x43	-0.021386	-0.007233	0.002730	0.030587	-0.000094	
0.030251	0.002252	0.012684	-0.000481	-0.002207	0.001176	
0.005622	-0.001957	0.008769	-0.047028	-0.015487	0.018419	-
0.012549	-0.009951	-0.010857	-0.005505	0.013378	-0.003331	
0.004637	0.002535	0.017244	-0.009629	0.010899	-0.020734	

0.041625 -0.003670 -0.065363 0.044829 -0.006530 0.023607 -
0.000659 -0.005650
y43 0.004278 0.000461 0.007876 0.023257 -0.020017 -
0.022449 0.022833 0.008645 0.036824 -0.011794 -0.000945 -
0.001407 0.005052 0.005595 -0.001218 0.009744 0.004995
0.005874 -0.032463 -0.007012 -0.010226 0.020379 0.014586
0.000220 0.015843 0.003078 -0.022866 -0.015717 0.029905
0.013877 0.023194 -0.037710 0.015118 -0.013205 -0.009330 -
0.018344 -0.016696
z43 0.023086 -0.000418 0.008090 -0.000927 -0.011148
0.016902 -0.003872 0.014272 0.026690 -0.001072 -0.015414
0.012508 -0.023803 -0.001117 0.008824 -0.007468 -0.002369 -
0.016530 -0.024038 -0.038455 -0.024523 0.012118 -0.006896 -
0.008941 0.028552 -0.021010 0.033201 -0.006895 0.010593 -
0.005434 -0.035496 -0.010811 -0.008340 -0.005135 0.027277 -
0.020017 -0.043600
x44 -0.022913 -0.004538 0.000285 0.030102 -0.001970
0.029567 0.004799 0.013247 -0.000878 -0.006728 0.002239
0.007444 -0.006743 0.007860 -0.046169 -0.014826 0.021448 -
0.011475 -0.009272 -0.008944 -0.004607 0.011065 -0.006124
0.006269 0.001640 0.018062 -0.012420 0.009735 -0.021079
0.041582 -0.002539 -0.065132 0.039591 -0.000153 0.023665 -
0.000089 0.000258
y44 0.006657 0.001303 0.006257 0.023446 -0.022599 -
0.022789 0.021549 0.008459 0.038547 -0.009642 -0.000519 -
0.000934 0.007958 0.004889 0.000564 0.009531 0.004211
0.006582 -0.030760 -0.012085 -0.013664 0.025763 0.015441 -
0.003509 0.015297 0.005884 -0.024353 -0.014662 0.026777
0.016010 0.017652 -0.039908 0.020474 -0.016428 -0.012862 -
0.017111 -0.019011
z44 0.025195 -0.001954 0.007939 -0.004189 -0.010168
0.019855 -0.002796 0.014408 0.022764 -0.000185 -0.015739
0.014542 -0.023990 0.000299 0.005478 -0.006995 -0.002248 -
0.012091 -0.024104 -0.037784 -0.021545 0.014434 -0.007984 -
0.007361 0.024499 -0.016602 0.034402 -0.006381 0.011709 -
0.003248 -0.035473 -0.010025 -0.008913 -0.003757 0.022541 -
0.012700 -0.036555
x45 -0.024100 -0.001058 -0.001633 0.026932 -0.000998
0.031738 0.009328 0.013077 -0.001134 -0.007334 0.009590
0.009534 -0.010486 0.006201 -0.044961 -0.018686 0.029677 -
0.008079 -0.009529 -0.004262 -0.008164 0.011372 -0.002825
0.007532 0.003451 0.019191 -0.018401 0.010499 -0.022779
0.037271 -0.000932 -0.058380 0.030137 0.007829 0.024411 -
0.000810 0.002984
y45 0.009479 0.002605 0.003364 0.023386 -0.026454 -
0.024495 0.020667 0.007635 0.039342 -0.010977 -0.003406
0.001164 0.009628 0.003817 0.005819 0.012415 0.001927
0.005954 -0.025764 -0.019937 -0.015476 0.028319 0.011964 -
0.007895 0.010694 0.010294 -0.023656 -0.015728 0.026009
0.021139 0.008108 -0.039465 0.023880 -0.018784 -0.021346 -
0.010510 -0.014692
z45 0.027282 -0.004286 0.007850 -0.007392 -0.008802
0.023416 -0.000697 0.015480 0.017365 0.000735 -0.012990
0.015831 -0.026420 0.001565 0.002336 -0.006192 -0.001983 -
0.003397 -0.025181 -0.033819 -0.018839 0.018871 -0.006566 -
0.005533 0.021713 -0.012702 0.033102 -0.006166 0.009695 -
0.004515 -0.034720 -0.008224 -0.006954 -0.002567 0.017160 -
0.005820 -0.028805
x46 -0.027062 0.002405 -0.002684 0.027862 -0.000632
0.030962 0.013605 0.011441 -0.002081 -0.014506 0.013446

0.006028 -0.016852 0.003405 -0.038281 -0.020478 0.030384 -
0.008206 -0.012891 -0.000551 -0.011540 0.011733 -0.004046
0.006753 0.007347 0.014071 -0.023024 0.006766 -0.014498
0.033360 -0.002170 -0.046539 0.017143 0.017034 0.026236 -
0.004410 0.001820
y46 0.013297 0.003975 -0.001877 0.020294 -0.030439 -
0.023842 0.020560 0.006227 0.037195 -0.006848 -0.003979
0.007436 0.011235 0.002451 0.010913 0.015441 0.005381
0.011775 -0.019060 -0.027261 -0.020681 0.032065 0.010213 -
0.011144 0.001654 0.020814 -0.026109 -0.017163 0.018418
0.026494 0.003313 -0.035241 0.027290 -0.019911 -0.036104 -
0.005813 -0.014126
z46 0.029288 -0.005947 0.010059 -0.011036 -0.006960
0.027335 0.002566 0.016815 0.013218 -0.001116 -0.007549
0.016320 -0.028191 0.003656 -0.001126 -0.007534 -0.001284
0.002627 -0.025183 -0.026365 -0.013930 0.020415 -0.004433 -
0.004302 0.018292 -0.011800 0.029692 -0.006584 0.013006 -
0.008492 -0.036942 -0.006960 -0.003408 0.000912 0.014747
0.002324 -0.017142
x47 -0.034851 -0.001304 -0.011273 0.019748 -0.015378
0.017317 0.001194 -0.009906 -0.021637 -0.017193 0.019803 -
0.020512 -0.019908 -0.008541 -0.035656 0.008409 0.035590 -
0.015429 0.020174 -0.007380 -0.032483 0.020620 -0.035146 -
0.017520 0.017679 -0.030598 -0.027707 0.021147 0.009578
0.013343 0.032546 -0.024993 -0.047553 0.013202 0.024259
0.019241 -0.000948
y47 0.025348 0.003702 -0.008167 0.025046 -0.014613 -
0.011509 0.033228 0.005450 0.048006 -0.002920 -0.001583
0.010900 -0.016335 -0.010787 0.016148 -0.000943 0.021182
0.022378 0.001581 -0.022151 -0.032284 0.029960 0.003270 -
0.013170 0.011513 0.013746 -0.029207 0.003769 0.029887
0.024307 0.024700 -0.031669 0.003964 -0.015020 0.018715 -
0.016033 -0.012042
z47 0.029170 -0.009949 0.010059 -0.011357 0.000377
0.045294 -0.009252 0.020122 0.003317 -0.009108 0.012461
0.017322 -0.033702 -0.002772 -0.008048 -0.007597 -0.009751
0.012801 -0.029292 -0.007844 -0.015041 0.050683 -0.006979 -
0.001849 0.020525 -0.016047 0.032439 -0.005796 -0.001638
0.008453 -0.017037 -0.025582 -0.013864 0.018225 0.021805 -
0.013186 0.018147
x48 -0.031121 0.000994 -0.013665 0.022946 -0.016717
0.010897 0.005472 -0.005739 -0.018839 -0.019594 0.020468 -
0.022388 -0.027209 -0.004512 -0.023822 -0.000170 0.019730 -
0.007950 0.020325 -0.015392 -0.043740 0.033219 -0.036651 -
0.019092 0.017725 -0.034937 -0.032043 0.013371 0.005271 -
0.003228 0.030787 -0.020611 -0.047909 0.013052 0.018135
0.028213 0.008740
y48 0.022678 0.006949 -0.001535 0.025436 -0.016947 -
0.013778 0.028693 0.011036 0.053430 -0.003445 -0.006089
0.013636 -0.004376 -0.006985 0.012188 -0.002517 0.019855
0.017972 -0.010090 -0.020989 -0.025568 0.021338 0.001946 -
0.011596 0.003964 0.014815 -0.024858 0.004099 0.018127
0.025406 -0.001343 -0.027714 0.018392 -0.018615 0.017648 -
0.019517 -0.015664
z48 0.035479 -0.011408 0.012086 -0.013538 0.000960
0.042921 -0.004158 0.021258 0.002640 -0.003990 0.005514
0.025688 -0.025380 0.001586 -0.006998 -0.009046 -0.010406
0.009551 -0.031725 -0.017556 -0.007693 0.044377 -0.000198
0.003693 0.016688 -0.011628 0.025253 -0.002749 -0.006259

0.001803 -0.012162 -0.026274 -0.009548 0.023194 0.012060 -
0.007154 0.020860
x49 -0.029492 0.005291 -0.011580 0.024573 -0.019224
0.005799 0.008191 0.000205 -0.014102 -0.018699 0.021428 -
0.020767 -0.030798 0.000485 -0.015104 -0.007132 0.014573 -
0.003489 0.015983 -0.020049 -0.050081 0.041250 -0.035571 -
0.014407 0.013824 -0.036970 -0.034172 0.012725 -0.002926 -
0.018373 0.026576 -0.020751 -0.047867 0.021899 0.012285
0.035014 0.007956
y49 0.021335 0.007737 0.003091 0.024477 -0.017989 -
0.014664 0.024954 0.015283 0.055701 -0.006653 -0.009489
0.014263 0.004806 -0.003098 0.005656 -0.003458 0.011887
0.015216 -0.016970 -0.017997 -0.018514 0.014032 0.002964 -
0.008345 -0.000933 0.014257 -0.024682 -0.001231 0.012194
0.029432 -0.018483 -0.017804 0.026454 -0.024792 0.014745 -
0.024376 -0.008933
z49 0.042083 -0.014085 0.012359 -0.014783 0.000723
0.038989 0.000533 0.022958 0.002170 -0.002330 -0.002635
0.030994 -0.018106 0.005575 -0.006571 -0.008997 -0.017095
0.006833 -0.031789 -0.026313 -0.001014 0.036585 0.005389
0.009833 0.015993 -0.010494 0.015854 -0.001336 -0.006393 -
0.003001 -0.006822 -0.020249 -0.007336 0.021538 0.005753 -
0.002916 0.024867
x50 -0.027932 0.009064 -0.009350 0.025140 -0.019301
0.002742 0.012192 0.006420 -0.008180 -0.019386 0.023604 -
0.017712 -0.032154 0.004473 -0.009973 -0.014997 0.009229
0.002293 0.012921 -0.022300 -0.051682 0.046714 -0.031654 -
0.009483 0.009653 -0.034691 -0.037097 0.009204 -0.008581 -
0.031083 0.021170 -0.017455 -0.049093 0.027203 0.006532
0.034853 0.011818
y50 0.018723 0.007659 0.007911 0.025170 -0.022925 -
0.014804 0.015423 0.018886 0.055931 -0.005746 -0.010837
0.013434 0.012090 0.001552 0.000927 -0.004218 0.007242
0.009130 -0.020431 -0.017505 -0.018011 0.006160 0.000490 -
0.012703 -0.003235 0.004864 -0.022393 -0.000105 0.005877
0.032433 -0.033258 -0.012478 0.030827 -0.033802 0.008290 -
0.015548 -0.008222
z50 0.047481 -0.017040 0.013052 -0.014273 -0.002463
0.033729 0.002468 0.024181 0.000774 -0.000807 -0.010846
0.032827 -0.012642 0.011133 -0.003610 -0.007773 -0.022154
0.004120 -0.030209 -0.034764 0.001860 0.028380 0.006300
0.011447 0.015691 -0.014544 0.008240 0.002720 -0.007194 -
0.006521 -0.001770 -0.017723 -0.000887 0.018602 -0.000663
0.005364 0.022051
x51 -0.027664 0.011535 -0.005809 0.027541 -0.021858
0.001955 0.012718 0.013023 -0.003408 -0.019289 0.023938 -
0.015621 -0.031767 0.010030 -0.003871 -0.020207 0.001461
0.005418 0.012505 -0.023513 -0.052371 0.045624 -0.029444 -
0.006013 0.006377 -0.036159 -0.038814 0.006475 -0.014558 -
0.044356 0.017097 -0.011845 -0.041418 0.027877 0.005868
0.035261 0.011159
y51 0.017578 0.007454 0.007278 0.022945 -0.021244 -
0.017706 0.013335 0.019197 0.055983 -0.008281 -0.007880
0.012865 0.017808 -0.001045 -0.002938 -0.005832 0.007313
0.005796 -0.029574 -0.015192 -0.017405 0.005955 0.000185 -
0.016842 -0.004850 0.002122 -0.014159 -0.001342 0.001982
0.039127 -0.043657 -0.013373 0.031839 -0.043176 0.003720 -
0.021473 -0.008034
z51 0.053215 -0.019197 0.011525 -0.015338 -0.002007
0.026856 0.008339 0.024033 0.000967 -0.002295 -0.015359

0.033037	-0.007660	0.012780	-0.002951	-0.009243	-0.022181	
0.003070	-0.032134	-0.041383	0.005276	0.024748	0.007103	
0.011444	0.015275	-0.015560	0.002755	0.005416	-0.006055	-
0.006458	-0.000196	-0.016510	-0.000299	0.016071	-0.009908	
0.006277	0.016382					
x52	-0.027993	0.012581	-0.004172	0.030087	-0.024246	
0.000995	0.014020	0.020150	0.002863	-0.022757	0.025655	-
0.013993	-0.030151	0.013304	0.000095	-0.025494	-0.003426	
0.010112	0.006394	-0.022347	-0.051083	0.044056	-0.027174	-
0.005143	0.003831	-0.037588	-0.037085	0.006143	-0.018769	-
0.049978	0.008841	-0.007814	-0.037304	0.023660	0.000800	
0.028408	0.004799					
y52	0.016129	0.008705	0.010203	0.020688	-0.019753	-
0.016496	0.004370	0.016386	0.050155	-0.000663	-0.010070	
0.016751	0.022465	-0.000710	-0.003731	-0.005311	0.004262	-
0.003363	-0.028559	-0.010222	-0.014936	0.001499	0.000425	-
0.016525	-0.008136	0.002221	-0.006163	-0.009373	0.001875	
0.035413	-0.046506	-0.011508	0.038154	-0.045829	0.004821	-
0.015832	0.003820					
z52	0.057697	-0.019579	0.013600	-0.016378	-0.002360	
0.021975	0.010684	0.022328	-0.002424	0.001265	-0.022374	
0.034912	-0.003716	0.017685	-0.000144	-0.009470	-0.023871	
0.000579	-0.026001	-0.044881	0.009790	0.016085	0.006666	
0.013224	0.012060	-0.014409	-0.002279	0.000143	-0.004067	-
0.013360	0.005257	-0.012899	0.008684	0.017708	-0.009695	
0.013569	0.017373					
x53	-0.029517	0.012917	-0.001407	0.033820	-0.027131	
0.000852	0.013828	0.027638	0.008216	-0.025602	0.025436	-
0.012964	-0.028065	0.017140	0.005149	-0.028378	-0.008897	
0.014409	0.002515	-0.020287	-0.047031	0.039377	-0.026118	-
0.004852	0.002326	-0.039299	-0.034418	0.002644	-0.021369	-
0.056122	0.001823	-0.002834	-0.029687	0.018155	-0.000484	
0.021268	0.000059					
y53	0.015551	0.009763	0.012615	0.015781	-0.017734	-
0.013419	-0.002046	0.014318	0.045350	0.003082	-0.005008	
0.020862	0.027598	-0.002767	-0.008203	-0.005961	0.008317	-
0.009402	-0.025742	-0.004494	-0.014215	-0.000495	0.001939	-
0.018314	-0.011667	0.001848	-0.001753	-0.007632	-0.001092	
0.035760	-0.046417	-0.008974	0.035392	-0.048033	-0.000393	-
0.012036	0.004250					
z53	0.062281	-0.019152	0.016008	-0.019137	-0.002740	
0.017990	0.014498	0.021006	-0.003658	0.001565	-0.024272	
0.036946	0.001964	0.020620	-0.000971	-0.012835	-0.018911	
0.001317	-0.019016	-0.045936	0.012124	0.006694	0.007550	
0.012833	0.007057	-0.012390	-0.007071	-0.000235	-0.005652	-
0.014303	0.009114	-0.009527	0.014850	0.018371	-0.013963	
0.016017	0.009540					
x54	-0.032034	0.012717	0.002039	0.037086	-0.029990	
0.003134	0.012970	0.035113	0.013927	-0.027645	0.027379	-
0.010810	-0.022302	0.021041	0.008369	-0.032275	-0.010926	
0.018707	-0.000024	-0.016175	-0.041426	0.029743	-0.023436	-
0.005207	-0.000934	-0.040652	-0.030835	0.000672	-0.022742	-
0.059557	-0.004937	0.003102	-0.018032	0.011116	-0.000773	
0.011033	-0.007986					
y54	0.014196	0.010223	0.013037	0.012614	-0.015135	-
0.010223	-0.011082	0.011451	0.040915	0.006381	-0.000688	
0.021800	0.030791	-0.005112	-0.013441	-0.004463	0.010201	-
0.019685	-0.023143	0.000370	-0.013340	-0.000156	0.002486	-
0.019265	-0.009413	-0.004502	0.005106	0.003757	0.000937	

0.031001 -0.045048 -0.012593 0.032489 -0.047349 -0.002402 -
0.004555 0.007589
z54 0.065493 -0.018503 0.017254 -0.019450 -0.003857
0.012623 0.015508 0.020137 -0.003073 -0.000836 -0.028040
0.034154 0.005304 0.024432 -0.002483 -0.013464 -0.017398 -
0.000454 -0.012761 -0.047658 0.015353 -0.000542 0.005453
0.011801 0.007349 -0.016557 -0.008791 0.005092 -0.003206 -
0.018026 0.009462 -0.010215 0.022006 0.021100 -0.014122
0.021130 0.000622
x55 -0.035454 0.011705 0.006425 0.040821 -0.032934
0.006468 0.011504 0.043152 0.020351 -0.030548 0.029102 -
0.008832 -0.016174 0.025314 0.011063 -0.035009 -0.014097
0.022389 -0.002892 -0.010247 -0.032269 0.017572 -0.020430 -
0.006792 -0.003073 -0.041565 -0.026572 -0.001741 -0.023872 -
0.058964 -0.011786 0.011107 -0.005176 0.000206 0.000094 -
0.004548 -0.017068
y55 0.013407 0.009184 0.012720 0.009350 -0.011976 -
0.008392 -0.020326 0.007999 0.034506 0.010668 0.001058
0.022803 0.029645 -0.008823 -0.013299 0.001029 0.005935 -
0.031027 -0.022615 0.003151 -0.012717 0.005343 0.002548 -
0.018867 -0.005228 -0.007537 0.008285 0.008686 0.003585
0.033703 -0.041714 -0.011072 0.019589 -0.049824 -0.005584 -
0.001937 0.012631
z55 0.068492 -0.018278 0.018390 -0.019344 -0.004675
0.004742 0.016826 0.019389 -0.002157 -0.003367 -0.033666
0.029581 0.006768 0.028475 -0.000821 -0.012586 -0.019407 -
0.001544 -0.008088 -0.050356 0.018296 -0.006061 0.000106
0.011529 0.008413 -0.018179 -0.013274 0.004144 -0.001997 -
0.015017 0.007558 -0.005739 0.026211 0.020699 -0.017185
0.018912 -0.008542
x56 -0.039596 0.009933 0.010921 0.044975 -0.036107
0.010768 0.008644 0.051759 0.026598 -0.033301 0.029970 -
0.006790 -0.008094 0.028869 0.012563 -0.036821 -0.015223
0.025389 -0.006275 -0.002185 -0.021508 0.001223 -0.017124 -
0.008807 -0.005067 -0.042207 -0.020105 -0.004203 -0.023424 -
0.057086 -0.019759 0.017960 0.009991 -0.012475 0.003808 -
0.022578 -0.028377
y56 0.014420 0.009384 0.011935 0.002638 -0.006830 -
0.003338 -0.029836 0.002634 0.026086 0.020363 0.008886
0.027124 0.030573 -0.015266 -0.017287 0.003329 0.011126 -
0.043639 -0.018217 0.010601 -0.016132 0.008310 0.006591 -
0.019166 -0.002407 -0.006417 0.012182 0.014600 0.008959
0.030261 -0.032305 -0.017500 0.004819 -0.046612 -0.006567
0.004153 0.016800
z56 0.072002 -0.016184 0.020039 -0.021917 -0.004944 -
0.000789 0.018015 0.017177 -0.002227 -0.003766 -0.034476
0.026939 0.009406 0.029948 -0.002933 -0.014119 -0.015152 -
0.001683 -0.001361 -0.048175 0.017282 -0.014258 -0.002164
0.010307 0.007838 -0.016258 -0.018165 0.002023 0.001621 -
0.016937 0.006550 -0.005688 0.028763 0.024528 -0.015807
0.020209 -0.019971
x57 -0.025189 0.027365 0.039099 -0.015074 -0.012218
0.076772 0.037420 0.043360 0.054597 0.036898 0.137490
0.045114 0.051677 0.039596 -0.046421 -0.127723 0.097644
0.008014 0.052925 0.049888 -0.035423 -0.044371 0.036492 -
0.032784 -0.056759 0.005820 -0.061766 0.049658 -0.038744 -
0.060932 0.011693 0.007474 0.054309 -0.002877 0.028299 -
0.028251 -0.051647
y57 0.026476 0.031705 0.040457 -0.071493 0.026484
0.064267 -0.003286 -0.018030 0.048134 0.101191 0.133386

0.088463	0.087230	0.002765	-0.078231	-0.095459	0.133026	-
0.061111	0.042212	0.064558	-0.024288	-0.033642	0.076467	-
0.043002	-0.055486	0.055400	-0.039206	0.070129	-0.002457	-
0.005439	0.015716	-0.033973	0.035513	-0.022959	0.008062	
0.002410	-0.005880					
z57	0.085608	0.006936	0.048633	-0.089009	0.021010	
0.057284	0.053037	0.002411	0.021066	0.070076	0.077295	
0.079685	0.070006	0.042707	-0.063485	-0.111444	0.101962	-
0.019306	0.063516	0.008324	0.005278	-0.060920	0.051406	-
0.019143	-0.048560	0.046490	-0.069322	0.055862	-0.014638	-
0.031481	0.035353	-0.025580	0.062718	0.043549	-0.003162	
0.013098	-0.038242					
x58	-0.036216	0.006994	0.016360	0.043320	-0.038155	
0.024384	0.000557	0.059300	0.031896	-0.028460	0.030997	-
0.005510	0.000125	0.023546	0.003290	-0.033541	-0.004400	
0.017432	0.000074	0.009342	-0.024766	-0.003646	-0.020287	-
0.003458	-0.002702	-0.044137	-0.013836	0.005374	-0.018412	-
0.027918	-0.020793	0.023821	0.019568	-0.025840	0.019315	-
0.025984	-0.031438					
y58	0.009973	0.011195	0.010574	-0.009049	0.003667	-
0.000980	-0.049113	-0.010349	0.014776	0.027449	0.017572	
0.030874	0.017870	-0.010504	-0.013885	0.013557	0.011331	-
0.047852	-0.022198	0.012067	-0.008971	0.019517	0.019509	-
0.010547	0.009092	0.001980	0.011213	0.014012	0.024860	
0.002855	-0.011168	-0.018991	-0.014705	-0.040230	-0.006806	
0.010841	0.014224					
z58	0.072369	-0.010644	0.022208	-0.028401	-0.005606	-
0.008009	0.017475	0.014201	-0.007961	-0.001017	-0.035306	
0.021043	0.011413	0.025902	-0.003688	-0.013008	-0.011040	-
0.007506	0.012129	-0.034814	0.012782	-0.014873	-0.012623	
0.002559	0.006515	0.000738	-0.025739	0.003851	0.007317	-
0.006847	-0.005909	-0.015540	0.020550	0.021807	-0.016002	
0.017807	-0.019172					
x59	-0.034318	0.007131	0.019606	0.039319	-0.037501	
0.030986	-0.001746	0.060300	0.033001	-0.023292	0.036221	-
0.003464	0.005884	0.020910	-0.001537	-0.034959	0.004322	
0.014255	0.003631	0.016004	-0.023785	-0.008868	-0.018429	-
0.000605	-0.002710	-0.040826	-0.011651	0.009339	-0.016287	-
0.017092	-0.016603	0.024711	0.024966	-0.031605	0.025908	-
0.027147	-0.032242					
y59	0.006981	0.012387	0.009186	-0.012857	0.009009	-
0.001620	-0.056399	-0.016128	0.008705	0.029766	0.022618	
0.030203	0.011318	-0.009589	-0.011885	0.019301	0.011801	-
0.050099	-0.025453	0.010480	-0.004528	0.021754	0.024731	-
0.006696	0.013623	0.005110	0.014026	0.011031	0.029055	-
0.009098	-0.001382	-0.018522	-0.020618	-0.038016	-0.005981	
0.012362	0.014809					
z59	0.071420	-0.006926	0.023714	-0.033355	-0.003939	-
0.010787	0.017934	0.011298	-0.011843	0.002149	-0.029268	
0.019071	0.013709	0.022885	-0.005558	-0.013902	-0.003920	-
0.011092	0.018191	-0.027279	0.012862	-0.017238	-0.013739	-
0.000135	0.005265	0.006712	-0.025387	0.002385	0.007941	-
0.006956	-0.005714	-0.020025	0.019530	0.017673	-0.012613	
0.014208	-0.018492					
x60	-0.032910	0.005682	0.021309	0.037803	-0.037518	
0.033533	-0.007200	0.061677	0.032658	-0.022159	0.034642	-
0.004617	0.009354	0.018492	-0.001369	-0.029529	0.002799	
0.014670	0.004216	0.017140	-0.021977	-0.010811	-0.017097	
0.003707	-0.000276	-0.040148	-0.009150	0.009244	-0.010970	-

0.003826 -0.013723 0.025661 0.022212 -0.032043 0.028057 -
0.023758 -0.031545
y60 0.003907 0.013056 0.008003 -0.017213 0.015192 -
0.000519 -0.062793 -0.020705 0.004182 0.031018 0.027666
0.029166 0.006087 -0.007996 -0.010305 0.024322 0.010190 -
0.050061 -0.027243 0.006474 -0.002238 0.022088 0.030699 -
0.003913 0.015806 0.005441 0.014867 0.010090 0.034613 -
0.015856 0.003574 -0.015544 -0.028699 -0.029922 -0.006821
0.016268 0.015159
z60 0.069091 -0.004661 0.024410 -0.036167 -0.003614 -
0.014014 0.013517 0.010272 -0.015268 0.001207 -0.028879
0.015730 0.013064 0.020016 -0.004333 -0.006848 -0.006805 -
0.010097 0.022423 -0.023431 0.013202 -0.013775 -0.010846
0.002642 0.005232 0.010885 -0.027392 0.002235 0.012101
0.000482 -0.008050 -0.021742 0.009686 0.023989 -0.012320
0.016893 -0.014830
x61 -0.030700 0.004732 0.022624 0.035179 -0.036834
0.035815 -0.010921 0.061727 0.030980 -0.020629 0.033018 -
0.006633 0.012195 0.016723 -0.003273 -0.023415 0.006589
0.015791 0.003084 0.018688 -0.017665 -0.013200 -0.017371
0.007647 0.002928 -0.035546 -0.008939 0.009006 -0.006023
0.000211 -0.007408 0.028485 0.019799 -0.031186 0.031733 -
0.024537 -0.030273
y61 0.001727 0.014014 0.005827 -0.020689 0.021901
0.000536 -0.066949 -0.023497 0.000091 0.032534 0.032855
0.026442 -0.000186 -0.006934 -0.010913 0.028960 0.013910 -
0.051076 -0.030121 0.001708 -0.000571 0.020797 0.032655 -
0.004865 0.018550 0.010012 0.015255 0.010436 0.036799 -
0.028263 0.007554 -0.009407 -0.031176 -0.023348 -0.003452
0.013987 0.015933
z61 0.067034 -0.002350 0.024988 -0.039726 -0.002482 -
0.017481 0.011494 0.008791 -0.018195 0.001012 -0.028438
0.011591 0.012264 0.017484 -0.006333 -0.000227 -0.002553 -
0.010758 0.023608 -0.019069 0.014748 -0.011647 -0.012897
0.003393 0.009397 0.013296 -0.025694 0.002029 0.013237 -
0.002647 -0.009079 -0.021883 0.005913 0.024766 -0.006484
0.012972 -0.010744
x62 -0.029462 0.004030 0.023254 0.031670 -0.034554
0.037159 -0.015183 0.061591 0.027973 -0.017979 0.033752 -
0.006949 0.015121 0.016316 -0.003984 -0.019190 0.008146
0.018918 0.003621 0.020390 -0.013107 -0.017031 -0.011707
0.014265 0.004728 -0.027018 -0.011275 0.004274 0.000149
0.006852 -0.000740 0.025821 0.020398 -0.024799 0.028176 -
0.019120 -0.030262
y62 -0.001233 0.014660 0.003006 -0.022032 0.029543
0.001142 -0.070556 -0.024452 -0.004012 0.031989 0.038936
0.023430 -0.007240 -0.005352 -0.011559 0.031736 0.015574 -
0.051844 -0.032520 -0.003719 -0.001703 0.015933 0.036929 -
0.006047 0.019643 0.012318 0.013836 0.008528 0.038739 -
0.036171 0.007335 -0.004185 -0.026267 -0.013746 -0.005086
0.015194 0.016172
z62 0.062881 -0.000795 0.025401 -0.043435 -0.000473 -
0.020007 0.008214 0.008800 -0.021965 0.000596 -0.025457
0.010674 0.010431 0.016940 -0.007932 0.004650 -0.000900 -
0.008964 0.025420 -0.013990 0.014027 -0.010041 -0.007093
0.007943 0.010098 0.017289 -0.029461 -0.002146 0.016437 -
0.001672 -0.013350 -0.025347 0.007454 0.034216 -0.007109
0.014807 -0.009335
x63 -0.026550 0.004764 0.026285 0.025859 -0.032441
0.038036 -0.019100 0.059246 0.025937 -0.013499 0.034600 -

0.005430 0.018031 0.015508 -0.006816 -0.015605 0.012030
0.020323 0.002371 0.021198 -0.007473 -0.021707 -0.006329
0.021678 0.006850 -0.020161 -0.013279 -0.000027 0.005838
0.014049 0.011103 0.028100 0.014746 -0.020939 0.029091 -
0.017471 -0.027500
y63 -0.003283 0.015392 0.000379 -0.022530 0.036624
0.000386 -0.073503 -0.024337 -0.006590 0.030761 0.043165
0.019087 -0.015245 -0.005597 -0.011592 0.035603 0.017118 -
0.053170 -0.035971 -0.011759 -0.003599 0.010726 0.036997 -
0.009523 0.020596 0.012321 0.016516 0.008419 0.038194 -
0.041020 0.007447 0.006884 -0.024715 -0.009127 -0.001650
0.012526 0.018573
z63 0.059992 0.001844 0.027970 -0.049214 0.000890 -
0.022618 0.005186 0.007814 -0.023226 0.001985 -0.021301
0.010747 0.009420 0.015096 -0.011792 0.009164 0.004566 -
0.009439 0.025009 -0.009177 0.014820 -0.009474 -0.004081
0.012869 0.012685 0.017301 -0.028220 -0.001827 0.015750 -
0.000136 -0.010938 -0.020811 0.005120 0.034835 -0.001733
0.010612 -0.004399
x64 -0.023662 0.006396 0.030051 0.022640 -0.030617
0.039157 -0.022744 0.058297 0.018999 -0.014739 0.029656 -
0.007816 0.016214 0.009352 -0.004814 -0.010654 0.017070
0.024969 0.000112 0.021927 -0.000781 -0.028282 -0.001191
0.028171 0.009482 -0.013150 -0.017469 -0.005122 0.014965
0.016974 0.019876 0.024291 0.009356 -0.013696 0.029253 -
0.015056 -0.025806
y64 -0.005216 0.016418 -0.001403 -0.022920 0.044428
0.001294 -0.073430 -0.022689 -0.009153 0.028680 0.047800
0.014432 -0.023129 -0.006933 -0.012835 0.036077 0.023183 -
0.053193 -0.038261 -0.019904 -0.006791 0.001334 0.035968 -
0.014930 0.018514 0.013776 0.016892 0.010115 0.036328 -
0.044983 0.002672 0.016004 -0.019030 -0.001441 0.001138
0.009190 0.019224
z64 0.057492 0.004121 0.029629 -0.050074 0.003482 -
0.021923 0.000610 0.009848 -0.027570 -0.002982 -0.020474
0.005709 0.003829 0.007583 -0.010157 0.013415 0.007801 -
0.010359 0.024726 -0.005067 0.011222 -0.009325 -0.001191
0.009649 0.015642 0.014896 -0.030691 0.000042 0.017191 -
0.002174 -0.018445 -0.019916 0.009165 0.034203 0.007287
0.007507 -0.003702
x65 -0.019679 0.005686 0.028806 0.015717 -0.024580
0.034602 -0.028213 0.057698 0.016863 -0.007142 0.032677 -
0.007343 0.021638 0.013017 -0.005855 -0.005884 0.010466
0.027503 -0.003417 0.017341 0.007684 -0.029001 0.001093
0.032892 0.011036 -0.002307 -0.018328 -0.010311 0.022498
0.019656 0.035094 0.026331 -0.005671 -0.011602 0.028767 -
0.009889 -0.024124
y65 -0.007085 0.016541 -0.005580 -0.022076 0.053580
0.000342 -0.073617 -0.019471 -0.009613 0.026999 0.054504
0.008587 -0.030362 -0.004978 -0.015318 0.038617 0.023817 -
0.054671 -0.042327 -0.030377 -0.010406 -0.007659 0.034438 -
0.022799 0.016672 0.014318 0.018359 0.011522 0.032420 -
0.048049 -0.001905 0.030093 -0.012603 0.006793 0.004040
0.006281 0.021519
z65 0.055898 0.003948 0.027352 -0.054558 0.007963 -
0.024561 -0.005284 0.012033 -0.025957 -0.000185 -0.014267
0.003790 0.003627 0.005380 -0.011169 0.017743 0.005536 -
0.010154 0.024215 -0.005233 0.007532 -0.005491 -0.004418
0.006754 0.015029 0.015330 -0.024925 0.004368 0.017459 -

0.002200	-0.018746	-0.009884	0.003268	0.021938	0.018512	
0.008370	-0.001710					
x66	-0.016704	0.005442	0.026284	0.013903	-0.017168	
0.030911	-0.032043	0.058721	0.011037	-0.005642	0.031534	-
0.008720	0.019360	0.013040	-0.001718	-0.003048	0.006832	
0.030600	-0.011409	0.013041	0.009428	-0.033332	-0.000355	
0.034561	0.015815	0.008358	-0.016188	-0.017265	0.026230	
0.021385	0.027789	0.031503	-0.009048	-0.006572	0.027605	
0.004392	-0.015277					
y66	-0.007352	0.017819	-0.006256	-0.019096	0.056711	
0.000303	-0.077118	-0.016789	-0.011389	0.025793	0.053380	
0.004222	-0.036305	-0.009416	-0.016092	0.032590	0.018754	-
0.050692	-0.042981	-0.029951	-0.010519	-0.005032	0.027399	-
0.018831	0.015760	0.006872	0.019664	0.004651	0.034871	-
0.039962	0.001657	0.027812	0.000456	0.008362	-0.007625	
0.005572	0.013928					
z66	0.055622	0.003094	0.026035	-0.052859	0.010156	-
0.026292	-0.012444	0.012166	-0.024627	-0.002905	-0.012056	
0.000014	-0.001221	0.000614	-0.009528	0.021942	0.003804	-
0.008050	0.022570	-0.004148	0.005628	-0.003254	-0.009295	-
0.000488	0.018122	0.013463	-0.024919	0.003990	0.022399	-
0.002725	-0.022320	-0.011097	0.007782	0.013456	0.023608	
0.007380	0.001346					
x67	-0.013664	0.006737	0.025838	0.009213	-0.008708	
0.030822	-0.034694	0.059835	0.005693	-0.002407	0.034043	-
0.009990	0.020323	0.013748	-0.003612	-0.003323	0.008225	
0.028606	-0.018550	0.011047	0.011537	-0.040070	0.001992	
0.037312	0.016524	0.016860	-0.016171	-0.021189	0.027734	
0.022324	0.021310	0.040606	-0.010791	-0.004492	0.029635	
0.013114	-0.010745					
y67	-0.008248	0.018149	-0.006984	-0.015287	0.058734	-
0.000467	-0.079228	-0.014938	-0.013145	0.023514	0.050928	
0.000671	-0.041805	-0.013612	-0.014533	0.028165	0.012878	-
0.045185	-0.042678	-0.030412	-0.010029	-0.002158	0.020507	-
0.015179	0.015490	-0.000446	0.020535	-0.001608	0.034942	-
0.032953	0.002940	0.025217	0.010667	0.010714	-0.017786	
0.004965	0.008400					
z67	0.054893	0.004049	0.025974	-0.053682	0.013574	-
0.026221	-0.017816	0.012075	-0.023408	-0.004574	-0.007340	-
0.003582	-0.002066	-0.002803	-0.013159	0.023082	0.006530	-
0.008701	0.021526	-0.001683	0.005769	-0.003759	-0.010720	-
0.001957	0.020664	0.007439	-0.026057	0.006874	0.025714	-
0.004727	-0.021228	-0.008604	0.012045	0.002766	0.029942	
0.002413	0.002216					
x68	-0.010755	0.007158	0.025895	0.005744	-0.001569	
0.029220	-0.038150	0.061778	0.000829	0.000540	0.035904	-
0.010480	0.018507	0.012083	-0.004970	-0.000861	0.009657	
0.028686	-0.024348	0.009030	0.012267	-0.043493	0.004099	
0.041580	0.019719	0.024488	-0.016034	-0.024447	0.026766	
0.022800	0.017344	0.043822	-0.010869	-0.001459	0.025843	
0.024079	-0.001457					
y68	-0.009479	0.018156	-0.007513	-0.011672	0.060159	-
0.000177	-0.079048	-0.014344	-0.015080	0.021217	0.048386	-
0.002305	-0.045189	-0.016807	-0.011868	0.023248	0.007345	-
0.039933	-0.042325	-0.030855	-0.010034	-0.001737	0.014357	-
0.013579	0.013237	-0.008111	0.019248	-0.005972	0.033920	-
0.027649	0.001017	0.023150	0.018847	0.010789	-0.024764	
0.004357	0.001143					
z68	0.054594	0.004846	0.026413	-0.054103	0.016512	-
0.028497	-0.023551	0.012785	-0.021523	-0.006647	-0.003633	-

0.006545	-0.005037	-0.007303	-0.015558	0.026012	0.008712	-
0.007263	0.020259	0.000850	0.005349	-0.002891	-0.012210	-
0.000895	0.025289	0.002721	-0.025124	0.006208	0.027205	-
0.008485	-0.020015	-0.010709	0.013570	-0.003314	0.031531	-
0.000051	0.010312					
x69	-0.008213	0.006400	0.025388	0.004387	0.004624	
0.027528	-0.043150	0.064886	-0.004246	0.002308	0.035067	-
0.013186	0.015335	0.010556	-0.006516	0.000642	0.007397	
0.028254	-0.030321	0.006520	0.016777	-0.042153	0.001254	
0.040165	0.020260	0.031239	-0.016138	-0.027674	0.028476	
0.023992	0.008244	0.048277	-0.009755	0.001923	0.023576	
0.033711	0.004499					
y69	-0.011061	0.018072	-0.007160	-0.009226	0.061252	
0.000455	-0.076485	-0.014380	-0.016874	0.018500	0.046185	-
0.003507	-0.046553	-0.018708	-0.008895	0.019924	0.003354	-
0.034974	-0.040716	-0.030207	-0.010059	-0.003219	0.010940	-
0.008820	0.010627	-0.015649	0.016524	-0.007484	0.027389	-
0.025406	-0.000445	0.018559	0.022243	0.012234	-0.030437	
0.002119	-0.004599					
z69	0.054442	0.005557	0.026533	-0.053562	0.019175	-
0.030292	-0.030326	0.014239	-0.019483	-0.007696	-0.001310	-
0.010790	-0.008399	-0.010619	-0.017538	0.026178	0.007422	-
0.005985	0.018590	0.003304	0.006415	-0.001083	-0.016668	-
0.004850	0.025293	0.000658	-0.026640	0.002951	0.030221	-
0.009203	-0.023244	-0.010601	0.014274	-0.008294	0.033071	-
0.000255	0.011943					
x70	-0.005926	0.005433	0.025376	0.001759	0.012812	
0.027588	-0.046568	0.065345	-0.011020	0.005238	0.037657	-
0.012917	0.014615	0.008486	-0.005600	0.001771	0.008447	
0.028051	-0.035459	0.004310	0.015608	-0.042873	0.001939	
0.039491	0.017756	0.032082	-0.015788	-0.030162	0.025301	
0.021280	0.003405	0.051464	-0.010505	0.006968	0.019248	
0.040076	0.006119					
y70	-0.013098	0.017816	-0.006554	-0.006785	0.060704	
0.000866	-0.073271	-0.014236	-0.018591	0.014789	0.043065	-
0.005356	-0.047823	-0.019984	-0.006207	0.018604	-0.001559	-
0.029816	-0.038960	-0.030321	-0.008172	-0.005060	0.008600	-
0.002612	0.007250	-0.021574	0.012352	-0.007466	0.020528	-
0.022761	-0.004510	0.014313	0.023167	0.014102	-0.034595	-
0.000254	-0.008276					
z70	0.053339	0.006243	0.026393	-0.053067	0.023421	-
0.032006	-0.034575	0.013198	-0.019330	-0.009199	0.001341	-
0.012869	-0.009745	-0.012981	-0.017060	0.025892	0.006821	-
0.003912	0.016388	0.006343	0.004857	-0.002378	-0.020722	-
0.006048	0.026800	-0.007723	-0.027555	0.001426	0.028009	-
0.014954	-0.021372	-0.012331	0.012224	-0.012629	0.032249	-
0.000361	0.010603					
x71	-0.005080	0.004738	0.024776	0.000409	0.018238	
0.026761	-0.051177	0.068127	-0.016860	0.004017	0.036793	-
0.015299	0.011535	0.007126	-0.007370	0.007260	0.006731	
0.026568	-0.042906	0.001813	0.014187	-0.046586	0.002045	
0.039864	0.017258	0.031541	-0.016797	-0.030889	0.022213	
0.016776	-0.000221	0.050212	-0.010710	0.006788	0.016163	
0.048069	0.007215					
y71	-0.014592	0.016604	-0.005504	-0.004991	0.060585	
0.002653	-0.067263	-0.015449	-0.020490	0.013268	0.040854	-
0.005013	-0.045623	-0.022137	-0.000408	0.015344	-0.005796	-
0.024161	-0.034689	-0.029043	-0.006316	-0.005389	0.005899	
0.002624	0.003143	-0.027473	0.005820	-0.005240	0.007569	-

0.021440 -0.012533 0.011070 0.021120 0.015981 -0.038259 -
0.003104 -0.010887
z71 0.051452 0.007812 0.026676 -0.053362 0.026324 -
0.034350 -0.038813 0.013359 -0.018112 -0.012858 0.003450 -
0.015781 -0.011874 -0.012839 -0.019090 0.026877 0.006002 -
0.002610 0.012753 0.008314 0.003360 -0.007475 -0.022427 -
0.006219 0.027323 -0.015817 -0.028531 -0.001836 0.027392 -
0.021777 -0.018395 -0.016870 0.006328 -0.016547 0.034040 -
0.000860 0.009819
x72 -0.003116 0.003206 0.024415 -0.001117 0.024033
0.029460 -0.054217 0.071038 -0.022038 0.006744 0.038612 -
0.017162 0.007882 0.003609 -0.008370 0.009709 0.004212
0.024590 -0.048018 0.000779 0.011872 -0.047340 -0.000631
0.036457 0.014047 0.033942 -0.020800 -0.026506 0.011481
0.012727 -0.008173 0.043517 -0.007437 0.006323 0.008624
0.057771 0.009528
y72 -0.017762 0.015434 -0.003327 -0.003423 0.059717
0.002602 -0.059994 -0.017916 -0.023551 0.007793 0.035678 -
0.003914 -0.041905 -0.020547 0.002391 0.016269 -0.007607 -
0.020273 -0.031806 -0.026530 -0.000581 -0.008362 0.008460
0.011880 -0.001905 -0.036817 0.002725 -0.005775 0.000230 -
0.022390 -0.018942 0.011149 0.012839 0.020283 -0.034321 -
0.011164 -0.017364
z72 0.050644 0.008723 0.026392 -0.053501 0.029080 -
0.034084 -0.041749 0.013835 -0.016819 -0.013954 0.005967 -
0.018749 -0.014338 -0.014657 -0.016164 0.025404 -0.001091 -
0.000523 0.009782 0.009594 -0.001065 -0.010214 -0.026964 -
0.007573 0.026661 -0.021083 -0.034817 -0.001374 0.019025 -
0.027425 -0.020305 -0.023586 -0.000900 -0.020170 0.029916
0.003050 0.010579
x73 -0.002291 0.000939 0.025940 -0.003221 0.029785
0.030637 -0.057665 0.073250 -0.029081 0.006369 0.037974 -
0.018011 0.006060 0.000812 -0.009183 0.013535 0.003621
0.017254 -0.053978 -0.000708 0.012251 -0.044174 -0.001462
0.032979 0.009203 0.028422 -0.021148 -0.021968 0.000753
0.007660 -0.016343 0.041082 -0.010677 0.009587 0.008737
0.057559 0.007955
y73 -0.020695 0.013870 -0.002350 -0.000389 0.057956
0.004378 -0.051149 -0.020002 -0.023363 0.004148 0.032686 -
0.004245 -0.039056 -0.020410 0.007641 0.016291 -0.012635 -
0.011323 -0.025707 -0.025881 0.000139 -0.015285 0.009907
0.018881 -0.006434 -0.040795 -0.006789 -0.001392 -0.011825 -
0.026121 -0.027278 0.004564 0.007626 0.024532 -0.037344 -
0.013561 -0.019589
z73 0.048842 0.010493 0.026313 -0.054452 0.032532 -
0.034785 -0.043478 0.012836 -0.018804 -0.017075 0.006767 -
0.019990 -0.015110 -0.014180 -0.015081 0.023901 -0.005371 -
0.001512 0.005555 0.012811 0.000780 -0.014064 -0.030324 -
0.004132 0.024511 -0.029081 -0.039008 -0.001777 0.009328 -
0.033471 -0.018818 -0.027649 -0.012979 -0.023815 0.027563
0.008870 0.005700
x74 -0.002327 -0.001871 0.027001 -0.005846 0.034330
0.031607 -0.060621 0.076121 -0.035402 0.005209 0.037537 -
0.019517 0.004397 -0.001197 -0.010647 0.021029 -0.000796
0.012854 -0.059265 -0.001068 0.013297 -0.041544 -0.000388
0.032155 0.003940 0.022745 -0.023622 -0.019185 -0.009969
0.001307 -0.025211 0.035062 -0.015046 0.015508 0.004840
0.054908 0.006135
y74 -0.023093 0.012959 -0.001623 0.003979 0.056029
0.007652 -0.041931 -0.023833 -0.024961 0.001362 0.029692 -

0.003080	-0.034664	-0.020712	0.012546	0.014493	-0.010988	-
0.005644	-0.018339	-0.026411	-0.001306	-0.021380	0.008602	
0.022420	-0.012041	-0.044189	-0.016582	0.008214	-0.028624	-
0.029699	-0.033560	-0.003510	0.002136	0.023581	-0.036386	-
0.012411	-0.019592					
z74	0.046195	0.011994	0.026880	-0.056583	0.035427	-
0.036736	-0.043878	0.013108	-0.019439	-0.020701	0.006899	-
0.021107	-0.015377	-0.010859	-0.015061	0.024796	-0.013521	-
0.000547	-0.000005	0.018042	0.004524	-0.020439	-0.029829	
0.005251	0.021856	-0.037415	-0.045066	-0.006414	-0.001105	-
0.041398	-0.018113	-0.033656	-0.029735	-0.018702	0.021366	
0.011854	-0.000127					
x75	-0.002576	-0.001890	0.033413	-0.008848	0.036764	
0.035312	-0.056919	0.075760	-0.033685	-0.004072	0.032911	-
0.016874	0.015396	-0.003078	-0.012702	0.025617	0.004843	
0.009229	-0.053313	0.010151	0.011441	-0.039161	0.004171	
0.024640	-0.002286	0.006760	-0.013683	-0.020179	-0.026230	-
0.002036	-0.033382	0.027759	-0.003697	0.016577	-0.002377	
0.039393	0.003043					
y75	-0.024030	0.014845	-0.000642	0.010343	0.046404	
0.008623	-0.024424	-0.028797	-0.021728	-0.005734	0.019263	
0.002793	-0.021988	-0.016677	0.017299	0.021795	-0.000062	-
0.001719	-0.011129	-0.024405	0.005450	-0.022129	0.013261	
0.030887	-0.012664	-0.036698	-0.010072	-0.004215	-0.030843	-
0.033232	-0.031678	-0.001025	0.007042	0.009257	-0.023964	-
0.023351	-0.008567					
z75	0.043083	0.013235	0.025683	-0.053390	0.031605	-
0.036120	-0.036900	0.006635	-0.017380	-0.029726	0.005967	-
0.018976	-0.005062	-0.010217	-0.012311	0.026567	-0.009552	
0.000462	0.000158	0.027683	0.011071	-0.022045	-0.025434	
0.006320	0.010469	-0.030712	-0.040207	-0.029976	-0.000582	-
0.044284	-0.017874	-0.032165	-0.027831	-0.021823	0.019872	
0.006327	0.010630					
x76	-0.003289	-0.001613	0.037496	-0.011429	0.037607	
0.037940	-0.052022	0.073145	-0.028771	-0.009983	0.027338	-
0.013883	0.026969	-0.003374	-0.014257	0.029767	0.006904	
0.002017	-0.048604	0.019493	0.012521	-0.036633	0.007360	
0.019264	-0.009112	-0.007136	-0.002408	-0.021858	-0.038923	-
0.004498	-0.038592	0.018073	0.004547	0.018403	-0.005192	
0.026332	0.006699					
y76	-0.025136	0.014231	0.001571	0.017091	0.036922	
0.010518	-0.012012	-0.030187	-0.020704	-0.011037	0.012246	
0.007124	-0.014396	-0.014449	0.022287	0.026279	0.011801	
0.005960	-0.001918	-0.024430	0.008503	-0.019742	0.014032	
0.033807	-0.009122	-0.026880	-0.012290	-0.005946	-0.033828	-
0.034541	-0.027282	0.004330	0.007221	-0.000576	-0.025515	-
0.031495	0.001868					
z76	0.039473	0.014621	0.023387	-0.050657	0.028467	-
0.036256	-0.027763	0.000922	-0.013227	-0.035843	0.005079	-
0.017399	0.005566	-0.009013	-0.011130	0.028988	-0.007722	-
0.001453	-0.002444	0.037086	0.018475	-0.022485	-0.020444	
0.010457	-0.000343	-0.024371	-0.031356	-0.050717	-0.001067	-
0.044534	-0.015452	-0.032372	-0.023916	-0.024058	0.023496	
0.003009	0.017621					
x77	-0.004569	-0.001991	0.040341	-0.012932	0.036458	
0.039943	-0.045837	0.069535	-0.022399	-0.013466	0.022964	-
0.012586	0.037358	-0.002879	-0.014132	0.035109	0.007745	-
0.006727	-0.045144	0.026402	0.014865	-0.034681	0.005857	
0.014410	-0.015095	-0.019567	0.007681	-0.020384	-0.049769	-

0.006846	-0.040083	0.008249	0.012134	0.018520	-0.008120		
0.014256	0.008858						
y77	-0.025021	0.014192	0.003380	0.023896	0.028868		
0.012403	-0.002086	-0.030314	-0.022459	-0.016238	0.005047		
0.010826	-0.011567	-0.015158	0.022796	0.023579	0.018258		
0.014864	0.006005	-0.023276	0.005892	-0.018157	0.015980		
0.032308	-0.007135	-0.015195	-0.017238	-0.008651	-0.023384	-	
0.033578	-0.022692	0.009144	0.005672	-0.007048	-0.019096	-	
0.029089	0.005222						
z77	0.035103	0.015062	0.021145	-0.047056	0.026017	-	
0.036367	-0.018093	-0.003559	-0.008080	-0.038564	0.006219	-	
0.015668	0.015385	-0.007422	-0.009303	0.029396	-0.006116	-	
0.002764	-0.005702	0.044418	0.026001	-0.021197	-0.016090		
0.012745	-0.007032	-0.019677	-0.022041	-0.064572	-0.004002	-	
0.042412	-0.011185	-0.031687	-0.021691	-0.023770	0.023737	-	
0.000863	0.023894						
x78	-0.006561	-0.002839	0.042011	-0.013486	0.033584		
0.041833	-0.037824	0.065708	-0.016462	-0.015676	0.019928	-	
0.012266	0.045567	-0.004136	-0.014327	0.039622	0.007588	-	
0.013746	-0.041610	0.031769	0.016522	-0.032481	0.000577		
0.007823	-0.022224	-0.028050	0.016699	-0.018270	-0.055701	-	
0.006837	-0.037901	-0.002741	0.017659	0.019632	-0.009034		
0.006953	0.008342						
y78	-0.023824	0.012929	0.005442	0.029391	0.022334		
0.013520	0.004386	-0.031198	-0.022487	-0.017180	-0.000881		
0.014866	-0.009886	-0.011939	0.023488	0.019669	0.021493		
0.018670	0.015065	-0.023267	0.005671	-0.016196	0.017224		
0.031699	-0.004022	-0.007455	-0.023859	-0.009831	-0.016555	-	
0.033006	-0.014860	0.016448	0.003449	-0.016109	-0.015868	-	
0.020800	0.012705						
z78	0.030623	0.015318	0.019019	-0.041006	0.023634	-	
0.035383	-0.008189	-0.007692	-0.002661	-0.039323	0.006860	-	
0.014290	0.021696	-0.007715	-0.006148	0.025332	-0.004771	-	
0.002072	-0.010336	0.050273	0.030128	-0.019544	-0.012782		
0.012409	-0.009979	-0.016925	-0.014066	-0.071839	-0.003902	-	
0.038872	-0.006630	-0.031628	-0.020330	-0.022192	0.025274	-	
0.003943	0.027082						
x79	-0.008380	-0.003885	0.042968	-0.012210	0.029544		
0.042881	-0.030171	0.059724	-0.009668	-0.013557	0.017269	-	
0.011274	0.051314	-0.004057	-0.014457	0.038434	0.006798	-	
0.020853	-0.038996	0.035878	0.020499	-0.030429	-0.006641		
0.003206	-0.027606	-0.034724	0.023707	-0.016285	-0.056348	-	
0.005486	-0.030919	-0.014058	0.018839	0.020383	-0.008363		
0.007380	0.011567						
y79	-0.021614	0.012167	0.006579	0.031218	0.016044		
0.014548	0.011992	-0.029375	-0.023612	-0.018820	-0.007019		
0.022624	-0.006265	-0.010458	0.022113	0.020077	0.022880		
0.020008	0.022469	-0.021293	0.003755	-0.014152	0.016873		
0.035636	-0.003265	0.004604	-0.031511	-0.005013	-0.011813	-	
0.028622	-0.000700	0.017913	-0.000195	-0.014066	-0.014268	-	
0.016375	0.006876						
z79	0.025553	0.014551	0.015942	-0.033269	0.022024	-	
0.034085	0.002508	-0.010284	0.003688	-0.038711	0.008869	-	
0.014639	0.026293	-0.008397	-0.001174	0.019468	-0.005879	-	
0.001643	-0.014044	0.051835	0.031295	-0.017541	-0.010493		
0.005452	-0.009677	-0.016027	-0.005411	-0.073924	-0.001137	-	
0.033292	-0.003201	-0.027355	-0.016788	-0.023518	0.027235	-	
0.004416	0.029455						
x80	-0.010152	-0.005243	0.042966	-0.008078	0.023373		
0.042855	-0.021863	0.052845	-0.002645	-0.010404	0.012415	-	

0.010774	0.055623	-0.003940	-0.011401	0.036696	0.000183	-
0.027356	-0.034910	0.036235	0.023841	-0.027086	-0.017254	-
0.002808	-0.031353	-0.044655	0.030184	-0.015932	-0.051437	-
0.002355	-0.022370	-0.022139	0.015771	0.019512	-0.005206	
0.005836	0.011992					
y80	-0.018716	0.007170	0.005919	0.030649	0.011348	
0.015837	0.015993	-0.029425	-0.023894	-0.022849	-0.009734	
0.026668	-0.001646	-0.013282	0.030202	0.018633	0.017500	
0.017250	0.024025	-0.025623	-0.001882	-0.010744	0.008361	
0.022222	-0.002031	0.011019	-0.029746	0.001290	-0.005390	-
0.016922	0.007136	0.030142	-0.005065	-0.020375	-0.010199	-
0.004047	0.002045					
z80	0.020169	0.014463	0.012898	-0.025131	0.020765	-
0.031407	0.015175	-0.010875	0.010555	-0.032974	0.011785	-
0.014078	0.028493	-0.009013	-0.001137	0.010356	-0.003430	
0.000028	-0.016488	0.055169	0.031412	-0.015769	-0.008935	
0.003542	-0.008688	-0.010551	-0.000724	-0.066572	0.001013	-
0.027414	0.009349	-0.029564	-0.013856	-0.020846	0.026139	-
0.001302	0.025140					
x81	-0.011402	-0.003677	0.045809	-0.003044	0.013087	
0.046561	-0.009428	0.042962	0.002568	-0.007970	0.005260	-
0.009901	0.053971	-0.013287	-0.004714	0.032467	0.004570	-
0.027295	-0.028361	0.038393	0.028602	-0.028799	-0.030171	-
0.005182	-0.036849	-0.048412	0.033596	-0.011772	-0.039337	
0.000572	-0.010020	-0.032848	0.010025	0.021722	-0.000028	
0.007227	0.013886					
y81	-0.017162	0.000334	0.005554	0.029387	0.008467	
0.014161	0.014811	-0.024468	-0.024068	-0.014644	-0.006582	
0.026308	0.003559	-0.007722	0.021161	0.014901	0.011050	
0.005467	0.017317	-0.025824	-0.008016	-0.001573	-0.001368	
0.012439	0.001907	0.024773	-0.031875	0.015476	-0.004557	-
0.009612	0.019481	0.037547	-0.007255	-0.025842	-0.001162	
0.009195	-0.009751					
z81	0.014894	0.017006	0.016642	-0.013886	0.014271	-
0.022718	0.030632	-0.014568	0.011862	-0.036002	0.005505	-
0.019356	0.019140	-0.028376	0.014450	-0.000102	0.014163	
0.013678	-0.017464	0.058905	0.024701	-0.021706	-0.007431	
0.001312	-0.006504	-0.011021	0.006150	-0.056031	0.008975	-
0.021289	0.012314	-0.034301	-0.008341	-0.014915	0.022356	
0.000078	0.029184					
x82	-0.013038	-0.004974	0.042216	0.002946	0.005320	
0.044612	0.000510	0.035213	0.014055	0.005566	0.006041	-
0.007300	0.057422	-0.008905	-0.004941	0.028097	-0.007763	-
0.036171	-0.025006	0.034392	0.036387	-0.025157	-0.046721	-
0.010344	-0.041863	-0.050593	0.036820	-0.007368	-0.025392	
0.003777	0.008805	-0.041791	-0.001284	0.019097	0.005039	
0.012489	0.010006					
y82	-0.016588	-0.006362	0.009590	0.021223	0.003312	
0.019514	0.010914	-0.021459	-0.030817	-0.012650	-0.007627	
0.025671	-0.005552	-0.006936	0.018271	0.013668	0.009611	-
0.005481	0.012178	-0.016379	-0.017339	0.004324	-0.003301	
0.016375	0.001170	0.038780	-0.046828	0.026457	-0.018715	-
0.012060	0.024769	0.033154	-0.000017	-0.007837	-0.000209	
0.027235	0.002653					
z82	0.008524	0.013241	0.007994	-0.001305	0.018379	-
0.024283	0.042344	-0.011929	0.024174	-0.023094	0.013858	-
0.020828	0.021974	-0.023152	0.017057	-0.014561	0.000929	
0.012577	-0.024250	0.052351	0.023587	-0.014681	-0.005638	-
0.008197	0.002245	-0.014243	0.012710	-0.042298	0.012625	-

0.009187 0.021314 -0.028777 -0.003312 -0.018676 0.020253
0.007165 0.023412
x83 -0.016609 -0.008798 0.038547 0.010308 -0.004316
0.042604 0.013158 0.025097 0.027291 0.018621 0.006741 -
0.009100 0.056819 -0.006610 -0.003388 0.023951 -0.019571 -
0.044597 -0.021743 0.030898 0.044489 -0.020857 -0.066584 -
0.016398 -0.048918 -0.051353 0.037371 -0.005290 -0.006985
0.007620 0.030861 -0.052769 -0.012021 0.015164 0.014724
0.023268 0.015214
y83 -0.017196 -0.009906 0.003388 0.013477 0.005141
0.011974 0.010011 -0.025514 -0.026406 -0.003899 -0.009857
0.023210 -0.016673 0.002426 0.015581 0.005690 -0.006351 -
0.012445 0.005704 -0.017345 -0.022914 0.016542 -0.002779
0.018821 0.004947 0.045014 -0.052369 0.052027 -0.026826 -
0.013406 0.029508 0.042261 0.012822 -0.008492 0.006260
0.045327 0.012731
z83 0.002727 0.009908 0.002828 0.013677 0.019919 -
0.019892 0.054996 -0.008041 0.032733 -0.009649 0.022235 -
0.020689 0.022156 -0.025214 0.019970 -0.029057 -0.006421
0.015092 -0.029883 0.047065 0.017211 -0.010357 -0.005924 -
0.020843 0.009923 -0.018194 0.013840 -0.024059 0.019589
0.005514 0.034914 -0.023915 -0.002754 -0.017933 0.015172
0.017727 0.007747
x84 -0.022482 -0.016440 0.001962 0.039811 0.042240 -
0.003380 0.030251 -0.023781 -0.025138 -0.006015 -0.003606 -
0.015008 0.009127 0.023659 -0.015677 -0.033746 0.029068
0.004456 -0.011715 -0.040397 -0.006049 0.014984 -0.017311
0.023973 0.014193 -0.007689 -0.027184 -0.007885 -0.036740
0.002955 0.014094 0.010775 -0.012072 -0.031207 0.039824 -
0.029468 0.071067
y84 0.014585 -0.016848 -0.014744 -0.012295 -0.015619 -
0.000341 -0.011827 -0.006336 -0.032845 0.010087 -0.014267 -
0.032450 0.019569 0.005250 0.004097 0.026391 0.006920 -
0.001809 0.000413 -0.034943 -0.034095 -0.009177 0.010015
0.006052 0.037105 -0.011589 0.006455 0.014687 0.036311
0.021554 0.025298 0.003783 -0.005634 -0.031277 -0.022533 -
0.052934 0.025275
z84 -0.012988 0.011180 0.017169 -0.003420 -0.016622 -
0.005196 0.018366 0.000268 0.001966 0.008845 0.008624 -
0.033269 0.017567 -0.002341 0.027949 -0.011282 0.012781 -
0.008987 0.009257 -0.008155 -0.004262 -0.043660 -0.023535 -
0.003041 -0.022522 -0.016172 0.026526 0.003435 -0.015306
0.002971 0.008280 0.038900 -0.021672 0.012759 -0.014242 -
0.038037 -0.043763
x85 -0.024913 -0.011498 -0.004022 0.030370 0.044661 -
0.018181 0.029907 -0.024674 -0.020885 -0.016084 0.006104 -
0.019502 0.004969 0.021545 -0.008951 -0.024036 0.025701
0.003097 -0.009408 -0.039667 -0.015677 0.019369 -0.011203
0.020179 0.030403 -0.001726 -0.029550 0.009695 -0.020778
0.016593 0.016228 0.001190 -0.028834 -0.014178 0.011174 -
0.020440 0.080400
y85 0.018881 -0.013253 -0.016197 -0.019956 -0.010478
0.000572 -0.012018 -0.000229 -0.025487 0.014243 -0.018728 -
0.028476 0.022775 0.004162 0.004124 0.022950 0.013509 -
0.003857 0.000925 -0.026197 -0.034761 -0.016428 0.001994
0.003959 0.038076 -0.008983 0.016871 0.016487 0.021770
0.032330 0.010011 0.013157 -0.001746 -0.037347 -0.033552 -
0.044037 0.002625
z85 -0.013039 0.006981 0.015714 -0.003201 -0.013524
0.002130 0.020329 0.001834 -0.002009 0.019195 0.008141 -

0.022407 0.014426 -0.003550 0.021888 -0.012847 0.003890 -
0.007884 0.015206 -0.008971 0.003862 -0.031349 -0.025077 -
0.008543 -0.014484 -0.022343 0.023559 0.002814 -0.019891
0.003504 0.005782 0.044449 -0.016057 0.003286 -0.001772 -
0.034060 -0.058069
x86 -0.026023 -0.007891 -0.011651 0.020786 0.044266 -
0.026782 0.029992 -0.022106 -0.016709 -0.021291 0.007150 -
0.026976 0.005583 0.018871 -0.003049 -0.017916 0.025167
0.002028 -0.008550 -0.040700 -0.021035 0.020785 -0.012772
0.014055 0.040214 0.007607 -0.023883 0.023366 -0.007988
0.028339 0.009467 -0.003293 -0.038224 -0.008849 -0.015189 -
0.017362 0.066190
y86 0.022433 -0.010596 -0.013964 -0.025940 -0.004347 -
0.001112 -0.012713 0.001706 -0.021817 0.015894 -0.019662 -
0.019183 0.022842 0.008642 0.001041 0.019456 0.018537 -
0.003530 -0.001094 -0.015086 -0.032779 -0.024449 -0.000500
0.012073 0.035269 -0.011385 0.020764 0.014621 0.007204
0.036622 0.002067 0.018090 -0.004001 -0.032570 -0.040620 -
0.029972 -0.000915
z86 -0.014673 0.002672 0.014320 -0.001250 -0.009049
0.007372 0.022415 0.001758 -0.007272 0.025540 0.011353 -
0.013128 0.011015 -0.000917 0.015468 -0.013413 -0.006615 -
0.004360 0.018840 -0.006702 0.010576 -0.019805 -0.023289 -
0.007747 -0.012703 -0.026531 0.014097 0.003921 -0.022130
0.002204 0.007452 0.042091 -0.017435 0.005459 0.010905 -
0.029485 -0.054580
x87 -0.026087 -0.004678 -0.017560 0.012743 0.043101 -
0.033860 0.027115 -0.019729 -0.016303 -0.025553 0.005830 -
0.031529 0.004633 0.016425 -0.000133 -0.011272 0.026657 -
0.000033 -0.008252 -0.038383 -0.024900 0.019792 -0.012933
0.012300 0.045611 0.015228 -0.016093 0.031415 -0.001161
0.033430 0.006094 -0.009508 -0.046822 -0.004721 -0.038902 -
0.013084 0.051413
y87 0.026023 -0.009811 -0.014900 -0.028827 0.001234
0.001943 -0.011502 0.001476 -0.015020 0.016029 -0.015161 -
0.014958 0.024501 0.009901 0.008019 0.010898 0.015905 -
0.002099 0.001940 -0.011419 -0.036965 -0.029242 -0.003955
0.002662 0.034973 -0.013588 0.016889 0.021491 -0.002098
0.041615 -0.012059 0.022649 0.002850 -0.032455 -0.038197 -
0.018499 0.003503
z87 -0.015693 -0.001751 0.011134 0.001733 -0.004912
0.014026 0.024246 0.001260 -0.013083 0.030785 0.014188 -
0.007171 0.007902 -0.002181 0.012213 -0.016281 -0.014994 -
0.002042 0.021999 -0.006703 0.013753 -0.011265 -0.026529 -
0.013232 -0.008134 -0.029808 0.005811 0.001324 -0.024112 -
0.003122 0.005922 0.043464 -0.015384 -0.006225 0.020110 -
0.025270 -0.055531
x88 -0.025400 -0.001813 -0.023225 0.004520 0.041398 -
0.036211 0.023440 -0.018711 -0.016225 -0.030267 0.005846 -
0.034681 0.005853 0.014380 0.004474 -0.006901 0.027193 -
0.001468 -0.006546 -0.035902 -0.028538 0.017482 -0.009163
0.009071 0.047028 0.020164 -0.010138 0.039790 0.004314
0.036286 -0.000263 -0.014470 -0.054375 0.000850 -0.054407 -
0.007751 0.039557
y88 0.028280 -0.008379 -0.013700 -0.030276 0.005443
0.000471 -0.010408 0.002305 -0.013260 0.016055 -0.013822 -
0.008989 0.019750 0.011765 0.003324 0.006417 0.013540 -
0.003058 -0.003924 -0.001364 -0.030544 -0.027149 -0.009409
0.008686 0.033231 -0.017985 0.018328 0.018257 -0.005860

0.039659	-0.012584	0.021383	0.002188	-0.027787	-0.035408	-
0.016463	0.008587					
z88	-0.017402	-0.005514	0.010106	0.002789	-0.002201	
0.020338	0.024127	0.003025	-0.015599	0.035982	0.014612	-
0.001489	0.007535	-0.002625	0.008692	-0.016476	-0.018582	
0.000645	0.026271	-0.006810	0.015971	-0.003567	-0.024149	-
0.014132	-0.007473	-0.026721	0.000880	0.000332	-0.026461	-
0.000342	0.002826	0.042459	-0.015593	-0.006090	0.020672	-
0.019975	-0.057579					
x89	-0.024206	0.001199	-0.029181	-0.002570	0.039155	-
0.035278	0.018307	-0.017554	-0.018348	-0.034934	0.005019	-
0.035084	0.005934	0.011142	0.007148	-0.004604	0.027021	-
0.002611	-0.006856	-0.033221	-0.028552	0.016400	-0.005845	
0.006374	0.044848	0.021720	-0.005054	0.042690	0.009479	
0.037377	-0.007064	-0.018375	-0.059164	0.003638	-0.064663	-
0.006420	0.033115					
y89	0.031178	-0.009368	-0.012920	-0.029997	0.008004	
0.001865	-0.009486	0.002313	-0.011805	0.014932	-0.012191	-
0.006883	0.016688	0.014063	0.005841	0.001550	0.008783	-
0.004031	-0.002386	0.004995	-0.030331	-0.026692	-0.011328	
0.007132	0.027756	-0.015635	0.019742	0.018864	-0.010898	
0.038498	-0.015052	0.021215	0.006307	-0.028066	-0.031625	-
0.007150	0.001969					
z89	-0.019090	-0.008709	0.008316	0.004009	0.000898	
0.025194	0.024314	0.003269	-0.017921	0.040033	0.016578	
0.003387	0.006155	-0.005144	0.006326	-0.018278	-0.018945	
0.002728	0.026289	-0.008375	0.014381	0.002265	-0.025597	-
0.018605	-0.000917	-0.025987	-0.004193	-0.001037	-0.025636	-
0.000762	0.000715	0.042431	-0.014119	-0.010370	0.023482	-
0.019987	-0.054700					
x90	-0.022247	0.003937	-0.035518	-0.008778	0.036167	-
0.030471	0.011898	-0.016788	-0.023151	-0.039815	0.003206	-
0.033780	0.007058	0.008040	0.009393	-0.004399	0.026251	-
0.002862	-0.006941	-0.029316	-0.026332	0.016545	-0.002018	
0.005956	0.037259	0.022183	0.001347	0.042366	0.012419	
0.035336	-0.015655	-0.021614	-0.061690	0.004054	-0.072068	-
0.004508	0.027078					
y90	0.033589	-0.010984	-0.010125	-0.028156	0.008794	
0.003383	-0.007892	0.002951	-0.010010	0.014727	-0.011335	-
0.005811	0.009532	0.016159	0.005895	-0.001976	0.003053	-
0.004766	-0.002958	0.009552	-0.027622	-0.027716	-0.011843	
0.008178	0.018791	-0.015351	0.018228	0.016517	-0.011346	
0.036866	-0.012407	0.019174	0.010636	-0.025291	-0.021512	
0.001194	-0.009406					
z90	-0.021223	-0.011400	0.006377	0.004519	0.003816	
0.028619	0.023637	0.003490	-0.019526	0.042464	0.018576	
0.007636	0.007608	-0.007973	0.005090	-0.019452	-0.015806	
0.005329	0.025697	-0.007830	0.010005	0.010451	-0.026504	-
0.020265	0.006856	-0.022330	-0.005738	-0.000951	-0.026537	-
0.000773	-0.001287	0.040126	-0.013759	-0.010638	0.022652	-
0.022457	-0.046347					
x91	-0.019277	0.005928	-0.042136	-0.014340	0.032680	-
0.021359	0.004504	-0.015558	-0.029823	-0.044712	-0.001043	-
0.030472	0.007646	0.004865	0.010217	-0.005835	0.025961	-
0.001646	-0.007367	-0.026672	-0.019808	0.013609	0.003441	
0.006724	0.024440	0.019031	0.007357	0.037308	0.013518	
0.031595	-0.023813	-0.024494	-0.061184	0.001899	-0.073031	-
0.002637	0.017428					
y91	0.033695	-0.014018	-0.006385	-0.026200	0.006645	
0.004128	-0.005595	0.003292	-0.009884	0.014609	-0.012458	-

0.005194	0.004753	0.018755	0.006006	-0.006730	-0.005328	-
0.006370	-0.002416	0.017788	-0.024392	-0.021820	-0.012840	
0.011789	0.014315	-0.011226	0.017795	0.016390	-0.019632	
0.038317	-0.012902	0.020676	0.011901	-0.024794	-0.004857	
0.001430	-0.013221					
z91	-0.022587	-0.013386	0.004471	0.004982	0.007356	
0.031893	0.021041	0.004273	-0.019613	0.043768	0.020435	
0.010353	0.006552	-0.010565	0.002056	-0.018691	-0.009479	
0.008390	0.023615	-0.009156	0.003857	0.011778	-0.025823	-
0.021846	0.010764	-0.018216	-0.006434	-0.003880	-0.018253	-
0.001659	0.000194	0.030964	-0.016238	-0.004258	0.013604	-
0.021681	-0.040594					
x92	-0.015286	0.007692	-0.048934	-0.019441	0.028314	-
0.008312	-0.004001	-0.014740	-0.038867	-0.049881	-0.005959	-
0.025632	0.008494	0.001373	0.011130	-0.008558	0.024611	-
0.000070	-0.008494	-0.023033	-0.010454	0.011817	0.009772	
0.009652	0.007766	0.013145	0.013570	0.029587	0.012872	
0.025029	-0.033281	-0.025848	-0.058475	-0.001995	-0.067601	-
0.000322	0.009144					
y92	0.034003	-0.017542	-0.001763	-0.023958	0.003489	
0.004228	-0.004393	0.002202	-0.008242	0.015931	-0.008063	-
0.007050	0.000877	0.020662	0.005144	-0.010662	-0.008307	-
0.008685	0.000111	0.023536	-0.021783	-0.017074	-0.013661	
0.007281	0.012116	-0.009099	0.016822	0.017472	-0.016720	
0.032384	-0.009319	0.016027	0.016406	-0.020815	0.012431	-
0.002096	-0.010022					
z92	-0.024062	-0.015390	0.002183	0.006030	0.010313	
0.036250	0.018075	0.005403	-0.021315	0.043111	0.018868	
0.012163	0.004340	-0.013612	0.000808	-0.018586	-0.006365	
0.010286	0.019644	-0.008690	-0.003939	0.013851	-0.025038	-
0.019873	0.011425	-0.007275	-0.008150	-0.008583	-0.014466	
0.002582	-0.000613	0.024543	-0.020409	-0.000713	0.001246	-
0.020354	-0.038821					
x93	-0.010698	0.008861	-0.054993	-0.024168	0.022811	
0.007414	-0.013426	-0.014118	-0.048589	-0.054959	-0.012654	-
0.018540	0.009078	-0.003679	0.012022	-0.013071	0.024708	
0.002198	-0.009554	-0.020445	-0.000356	0.007536	0.017348	
0.012469	-0.013489	0.004776	0.021360	0.016869	0.010440	
0.013743	-0.045775	-0.025892	-0.054597	-0.004989	-0.057204	-
0.001812	0.001135					
y93	0.031308	-0.022827	0.004040	-0.019691	0.000142	-
0.000968	-0.002239	0.000253	-0.003839	0.019671	-0.005351	-
0.009060	-0.001444	0.026387	0.000030	-0.012759	-0.018535	-
0.014123	0.004359	0.028475	-0.024094	-0.005395	-0.015589	
0.007909	0.008373	-0.000935	0.015769	0.017247	-0.012117	
0.026429	-0.005158	0.011543	0.015068	-0.011724	0.021647	-
0.012600	-0.003301					
z93	-0.024991	-0.016387	-0.000166	0.005745	0.012063	
0.042341	0.013967	0.006777	-0.025692	0.039866	0.017635	
0.012634	0.001388	-0.017748	-0.001093	-0.018812	0.002624	
0.013947	0.011565	-0.006224	-0.007824	0.011936	-0.022680	-
0.016892	0.012348	0.003083	-0.009730	-0.012864	-0.008119	
0.006014	0.001187	0.015846	-0.024672	0.001931	-0.010611	-
0.015009	-0.034368					
x94	-0.007812	0.007779	-0.059423	-0.023531	0.018252	
0.012245	-0.016385	-0.021166	-0.040499	-0.058102	-0.008278	-
0.019378	0.014175	0.003156	0.013417	-0.002017	0.013058	-
0.002498	-0.006639	-0.019695	0.001284	0.009461	0.015282	
0.008280	-0.017954	0.007126	0.018904	0.014101	0.013341	

0.011966 -0.033665 -0.016436 -0.024707 -0.009500 -0.030330
0.001970 -0.001866
y94 0.027857 -0.026292 0.012709 -0.015305 -0.001754 -
0.005728 0.002399 -0.002379 0.003802 0.025221 -0.000791 -
0.008465 -0.003082 0.031200 -0.007663 -0.010073 -0.028989 -
0.018237 0.008544 0.031261 -0.022254 -0.000765 -0.010502
0.010350 0.004958 0.001477 0.012824 0.014132 -0.008800
0.021000 -0.008031 0.011875 0.016913 -0.005420 0.033295 -
0.007997 -0.002998
z94 -0.024930 -0.014584 -0.005314 0.008896 0.013320
0.054882 0.009173 0.008908 -0.027572 0.028176 0.013664
0.015617 -0.002386 -0.024326 0.003802 -0.013075 0.013915
0.019134 0.009206 -0.005939 -0.015407 0.007140 -0.025483 -
0.018872 0.011264 0.005729 -0.002646 -0.021486 0.000737
0.012336 -0.008364 0.011204 -0.023391 0.011666 -0.011610 -
0.002304 -0.033690
x95 -0.005438 0.005825 -0.060837 -0.022812 0.013866
0.014656 -0.017378 -0.026958 -0.032595 -0.059765 -0.004719 -
0.019856 0.017462 0.008046 0.015361 0.007294 0.002323 -
0.006393 -0.003120 -0.019187 0.003421 0.013977 0.012265
0.003567 -0.020593 0.006193 0.018566 0.010368 0.014490
0.008769 -0.027396 -0.005911 -0.000225 -0.009118 -0.005599 -
0.000102 -0.004189
y95 0.023921 -0.029983 0.021659 -0.012278 -0.001823 -
0.010722 0.007540 -0.003873 0.010602 0.032538 0.004814 -
0.005951 -0.005331 0.034247 -0.015480 -0.010863 -0.034948 -
0.020128 0.013721 0.032461 -0.019959 0.003762 -0.009758
0.008648 0.005468 0.001198 0.009651 0.010313 -0.008342
0.015034 -0.015975 0.011937 0.011405 0.002450 0.037359 -
0.007086 0.000490
z95 -0.024271 -0.012215 -0.012425 0.010877 0.014218
0.066883 0.004847 0.009376 -0.028422 0.018344 0.010250
0.017609 -0.003858 -0.029033 0.007312 -0.007124 0.023796
0.023593 0.005565 -0.003771 -0.022177 -0.000421 -0.025715 -
0.015258 0.006470 0.012433 0.001946 -0.025674 0.008653
0.017226 -0.012375 0.007789 -0.013550 0.016528 -0.012487
0.010265 -0.029421
x96 -0.002211 0.004200 -0.059878 -0.023480 0.010932
0.014875 -0.015997 -0.030960 -0.025189 -0.057482 -0.000974 -
0.018421 0.022733 0.014897 0.015404 0.015087 -0.004827 -
0.006984 0.002628 -0.017203 0.005523 0.015822 0.010345
0.000653 -0.025013 0.004694 0.015614 0.009751 0.012817
0.004579 -0.024537 0.003165 0.014674 -0.004739 0.007525 -
0.000808 -0.002583
y96 0.020692 -0.032890 0.029338 -0.010084 0.000008 -
0.017515 0.012675 -0.004161 0.016556 0.041160 0.009565 -
0.002403 -0.006029 0.039827 -0.022422 -0.010083 -0.040972 -
0.020457 0.019092 0.032695 -0.017273 0.007078 -0.008308
0.008553 0.005325 0.002581 0.005472 0.007196 -0.011964
0.010991 -0.023599 0.008570 0.000107 0.013338 0.032100 -
0.006103 0.003564
z96 -0.024053 -0.010263 -0.020350 0.013713 0.014302
0.076826 0.000583 0.008637 -0.028850 0.008500 0.006597
0.017399 -0.006439 -0.032831 0.010991 -0.001634 0.028443
0.025776 0.000332 -0.003803 -0.026610 -0.005498 -0.026581 -
0.013793 0.002623 0.018505 0.005462 -0.028998 0.016153
0.020487 -0.015311 0.007083 -0.001767 0.018114 -0.007886
0.018288 -0.026302
x97 0.000411 0.003322 -0.055105 -0.024070 0.008072
0.015121 -0.013427 -0.033005 -0.018636 -0.052246 0.001737 -

0.015431	0.024145	0.015170	0.015353	0.019357	-0.010368	-
0.007932	0.009010	-0.012909	0.004690	0.016036	0.013681	-
0.001710	-0.028773	0.001276	0.008734	0.008221	0.015862	
0.007217	-0.024203	0.007711	0.026729	-0.003579	0.016853	
0.002401	0.001755					
y97	0.016851	-0.033519	0.037560	-0.006057	0.000918	-
0.021043	0.016707	-0.004289	0.019633	0.045936	0.011250	-
0.000444	-0.012082	0.037426	-0.023030	-0.011084	-0.046241	-
0.019389	0.023805	0.033108	-0.015957	0.008632	-0.003866	
0.007167	0.004225	0.005133	0.000861	0.001151	-0.010390	
0.011786	-0.032993	0.000743	-0.007381	0.020983	0.028582	-
0.002873	0.004921					
z97	-0.023127	-0.008356	-0.028156	0.015832	0.014222	
0.083990	-0.002645	0.007631	-0.027924	-0.000097	0.002175	
0.015922	-0.007778	-0.033384	0.013787	0.004668	0.032307	
0.028623	-0.003742	-0.004345	-0.028539	-0.010357	-0.028602	-
0.010142	-0.003332	0.024468	0.007932	-0.029797	0.017329	
0.021503	-0.018565	0.007884	0.006956	0.020685	-0.004231	
0.023320	-0.020954					
x98	0.002745	0.001578	-0.048840	-0.024496	0.005877	
0.011734	-0.008270	-0.031668	-0.012619	-0.044664	0.003618	-
0.011961	0.023093	0.015220	0.014186	0.025040	-0.011952	-
0.008353	0.015226	-0.009873	0.005561	0.015211	0.013921	-
0.001153	-0.029002	-0.006815	0.004150	0.007183	0.015724	
0.007251	-0.023014	0.017438	0.035000	-0.004024	0.020016	
0.001019	0.007001					
y98	0.012187	-0.034034	0.043780	-0.002306	0.003755	-
0.026665	0.022227	-0.003871	0.021379	0.050097	0.015623	
0.001282	-0.017322	0.036621	-0.023008	-0.009910	-0.047434	-
0.018025	0.027746	0.029341	-0.011560	0.010534	-0.003510	
0.005483	0.006879	0.002866	0.004839	-0.004386	-0.013732	
0.008698	-0.034726	-0.003514	-0.010047	0.022434	0.024403	-
0.004718	0.005142					
z98	-0.021622	-0.005996	-0.035872	0.016607	0.014875	
0.090663	-0.004919	0.005893	-0.025099	-0.006225	-0.001009	
0.014633	-0.006858	-0.031653	0.015038	0.007486	0.032323	
0.030105	-0.005962	-0.004248	-0.030434	-0.015666	-0.027141	-
0.007394	-0.011849	0.031254	0.006773	-0.027247	0.019050	
0.024051	-0.022448	0.008868	0.015229	0.023167	0.000671	
0.030030	-0.015440					
x99	0.005258	0.000815	-0.040807	-0.025438	0.004799	
0.007979	-0.002650	-0.029798	-0.008275	-0.038012	0.002571	-
0.010671	0.020880	0.014529	0.012226	0.028050	-0.014915	-
0.005231	0.021572	-0.003220	0.009537	0.014868	0.017132	
0.002457	-0.031851	-0.009389	-0.003200	0.005960	0.011352	
0.007091	-0.020865	0.021723	0.035118	-0.001353	0.017057	
0.000006	0.015290					
y99	0.007396	-0.032597	0.049009	0.001140	0.007293	-
0.031009	0.026534	-0.004461	0.022087	0.051123	0.019244	
0.001328	-0.023071	0.034330	-0.020103	-0.010099	-0.049953	-
0.015126	0.032166	0.026316	-0.005803	0.013759	-0.001988	
0.003986	0.005901	0.006250	0.009615	-0.011900	-0.017886	
0.006760	-0.033810	-0.011063	-0.013309	0.025163	0.020673	-
0.008011	0.002955					
z99	-0.019255	-0.003914	-0.043453	0.016318	0.015794	
0.095495	-0.005865	0.004963	-0.020721	-0.009537	-0.003544	
0.012895	-0.004418	-0.027945	0.014415	0.008534	0.032069	
0.030275	-0.006165	-0.004475	-0.031960	-0.021623	-0.025613	-
0.004179	-0.021053	0.035562	0.002436	-0.022550	0.018194	

0.025625	-0.026857	0.012520	0.021625	0.022256	0.006724	
0.036223	-0.008798					
x100	0.007614	-0.000653	-0.032558	-0.025516	0.003108	
0.001792	0.004133	-0.027193	-0.002964	-0.026141	0.004058	-
0.007231	0.019134	0.015252	0.009840	0.030385	-0.018347	-
0.001997	0.028950	0.001331	0.010579	0.014342	0.020829	
0.004530	-0.039325	-0.015632	-0.008481	0.006296	0.008656	
0.005631	-0.019659	0.027061	0.037406	0.002359	0.010884	
0.000941	0.016786					
y100	0.002126	-0.030826	0.051456	0.005414	0.011884	-
0.035894	0.030309	-0.005514	0.022785	0.053187	0.025900	
0.001375	-0.027030	0.033216	-0.015511	-0.009770	-0.052539	-
0.012304	0.035935	0.018707	-0.000892	0.017723	-0.001316	-
0.000421	0.003689	0.008130	0.019675	-0.019617	-0.021267	
0.004629	-0.031873	-0.019547	-0.011864	0.025367	0.017663	-
0.011139	-0.003394					
z100	-0.016424	-0.001964	-0.051270	0.014667	0.017687	
0.098011	-0.006018	0.004204	-0.014098	-0.010679	-0.005760	
0.010637	-0.000701	-0.021140	0.010658	0.008706	0.028804	
0.029569	-0.005192	-0.004639	-0.030976	-0.027454	-0.022416	
0.002377	-0.031977	0.039536	-0.005318	-0.016148	0.014438	
0.027073	-0.030881	0.018858	0.023944	0.020702	0.012468	
0.042867	0.000806					
x101	0.009477	-0.001282	-0.022217	-0.022882	0.000286	-
0.003922	0.013568	-0.023809	-0.002076	-0.016022	0.004572	-
0.006887	0.014540	0.011022	0.008696	0.033076	-0.015882	
0.006490	0.035374	0.008686	0.014992	0.016088	0.025465	
0.010373	-0.044764	-0.018664	-0.014967	0.004762	0.003858	
0.001941	-0.019359	0.035238	0.043361	0.006253	0.001360	-
0.000408	0.022439					
y101	-0.003691	-0.027076	0.054115	0.010451	0.016362	-
0.038762	0.034761	-0.007251	0.020016	0.050917	0.032704	-
0.000433	-0.033046	0.026851	-0.007217	-0.008719	-0.048610	-
0.006262	0.039993	0.011254	0.005473	0.021778	0.000057	-
0.004692	0.002539	0.011399	0.033485	-0.029201	-0.025557	
0.002212	-0.028284	-0.027884	-0.005616	0.023621	0.015367	-
0.018163	-0.008098					
z101	-0.013023	0.000383	-0.058653	0.011842	0.019581	
0.099578	-0.005160	0.003124	-0.005795	-0.010147	-0.007718	
0.006938	0.003812	-0.013097	0.004569	0.006910	0.026011	
0.029243	-0.001600	-0.004115	-0.028394	-0.034934	-0.017423	
0.010112	-0.046938	0.042713	-0.015200	-0.006900	0.008809	
0.027624	-0.035421	0.025797	0.027028	0.017852	0.019316	
0.048742	0.011737					
x102	0.010820	-0.002117	-0.006425	-0.019859	-0.003752	-
0.014365	0.021971	-0.018985	-0.001499	-0.004028	0.005229	-
0.006735	0.009885	0.002044	0.011691	0.035226	-0.011655	
0.011030	0.036745	0.012430	0.015135	0.019875	0.025399	
0.014626	-0.040534	-0.022438	-0.020011	0.002975	-0.004037	-
0.001962	-0.024226	0.043568	0.038714	0.011090	-0.006227	-
0.012827	0.021181					
y102	-0.009560	-0.021816	0.055906	0.016385	0.020904	-
0.043040	0.037749	-0.008792	0.015458	0.046412	0.038786	-
0.003665	-0.040452	0.017718	0.005832	-0.007594	-0.043564	-
0.000182	0.041027	0.001407	0.012022	0.024704	-0.002031	-
0.010575	0.003908	0.014927	0.049302	-0.039072	-0.032379	-
0.000318	-0.022758	-0.039715	-0.000881	0.020165	0.009419	-
0.027431	-0.014878					
z102	-0.008913	0.002410	-0.066596	0.008474	0.021901	
0.099683	-0.003127	0.001580	0.003728	-0.008180	-0.009048	

0.002324	0.009064	-0.004195	-0.000453	0.003859	0.019596	
0.027239	0.002715	-0.003850	-0.025302	-0.041785	-0.012312	
0.017630	-0.061710	0.044566	-0.028272	0.006098	0.000808	
0.026372	-0.039608	0.036896	0.028040	0.012475	0.027964	
0.053995	0.023422					
x103	0.012339	-0.004506	0.009656	-0.013502	-0.002670	-
0.029043	0.034469	-0.010410	-0.000647	0.010932	0.017469	-
0.003049	0.008009	-0.002287	0.013358	0.033509	-0.001645	
0.015991	0.040067	0.012597	0.018196	0.027724	0.022062	
0.006385	-0.035234	-0.015587	-0.019100	-0.001002	-0.011556	-
0.009226	-0.021218	0.039537	0.043794	0.005739	-0.016945	-
0.022213	0.016186					
y103	-0.020452	-0.020832	0.056024	0.017915	0.018911	-
0.033103	0.033092	-0.010446	0.003774	0.036924	0.034761	-
0.001821	-0.027539	0.022458	0.004392	-0.006532	-0.044944	
0.010738	0.037629	0.000348	0.027677	0.024324	0.004814	
0.006384	0.006572	0.028465	0.044062	-0.022891	-0.019202	-
0.002465	-0.019944	-0.028395	-0.005107	0.015658	0.011849	-
0.024452	-0.019075					
z103	-0.005429	0.006482	-0.063264	0.011355	0.022821	
0.086489	0.002786	0.001310	0.011528	-0.000713	-0.002827	-
0.008637	0.000022	-0.007893	-0.008950	0.003103	0.013535	
0.019908	0.001678	0.001270	-0.026353	-0.032035	-0.000114	
0.008998	-0.047330	0.036586	-0.018453	0.001300	-0.012787	
0.024499	-0.042177	0.034665	0.036705	0.011059	0.031770	
0.030114	0.023443					
x104	0.012460	-0.007335	0.024913	-0.006721	-0.005000	-
0.043020	0.043748	0.000345	-0.003148	0.017112	0.020451	-
0.003365	0.010663	-0.006554	0.019028	0.035721	0.004978	
0.021572	0.035138	0.009038	0.020790	0.032558	0.013595	-
0.001213	-0.030815	-0.005570	-0.014478	-0.002329	-0.015324	-
0.013821	-0.021099	0.043204	0.044956	-0.001066	-0.023747	-
0.013978	0.007949					
y104	-0.030114	-0.019536	0.054076	0.020783	0.015525	-
0.024866	0.027996	-0.011613	-0.007635	0.024164	0.025662	-
0.003071	-0.019300	0.023068	0.006916	-0.002324	-0.048487	
0.019598	0.031708	0.000021	0.039282	0.023588	0.007722	
0.022622	0.009812	0.037586	0.040159	-0.010227	-0.007746	-
0.002356	-0.019899	-0.018301	-0.009849	0.011041	0.012430	-
0.023202	-0.022261					
z104	-0.003155	0.009107	-0.059158	0.015779	0.021451	
0.069954	0.007382	0.002748	0.016709	0.002227	-0.002217	-
0.020299	-0.008295	-0.012695	-0.010682	0.005725	0.006463	
0.015061	-0.002604	0.005205	-0.026951	-0.022121	0.004504	
0.001494	-0.033354	0.028493	-0.009433	-0.004207	-0.024293	
0.022574	-0.045732	0.034049	0.042351	0.005930	0.030513	
0.014699	0.022072					
x105	0.012624	-0.009668	0.036127	-0.004691	-0.002855	-
0.049124	0.052958	0.007472	-0.006202	0.025886	0.026564	-
0.000207	0.019090	-0.009026	0.015644	0.030491	0.012524	
0.025363	0.032464	0.007113	0.027766	0.031326	0.008532	-
0.007441	-0.025216	0.002374	-0.006603	-0.006249	-0.018286	-
0.018240	-0.016754	0.050103	0.044200	-0.003870	-0.032748	-
0.013675	-0.001173					
y105	-0.037386	-0.017298	0.051253	0.020292	0.013804	-
0.014432	0.024019	-0.013765	-0.014505	0.016550	0.022349	-
0.000726	-0.009963	0.023812	0.006107	-0.003731	-0.045600	
0.024895	0.030823	0.001353	0.047264	0.021886	0.013996	
0.032996	0.009423	0.046156	0.034301	0.004938	0.000917	-

0.002907 -0.016572 -0.011941 -0.011151 0.008390 0.015402 -
0.022626 -0.024905
z105 -0.000992 0.011493 -0.053844 0.015707 0.022511
0.056975 0.013302 0.003195 0.022027 0.008987 0.004349 -
0.026892 -0.008476 -0.014791 -0.015457 0.001983 0.006072
0.011267 -0.003555 0.010444 -0.023404 -0.016909 0.010591 -
0.006122 -0.023059 0.022867 -0.001924 -0.009990 -0.035261
0.019936 -0.045802 0.036889 0.044681 0.002919 0.024801
0.001866 0.015327
x106 0.011365 -0.011376 0.045400 0.000638 -0.001221 -
0.052036 0.060366 0.015219 -0.008478 0.030450 0.030115
0.001845 0.020831 -0.012373 0.015931 0.027254 0.019735
0.027713 0.028806 0.006704 0.032202 0.031395 0.004093 -
0.013066 -0.017904 0.011326 -0.004273 -0.000273 -0.016956 -
0.020923 -0.008369 0.052549 0.043464 -0.008855 -0.035784 -
0.019221 -0.001757
y106 -0.043084 -0.014712 0.046648 0.020304 0.011077 -
0.004872 0.018807 -0.016902 -0.020910 0.008751 0.016613
0.000342 -0.005695 0.022582 0.005822 -0.003490 -0.042498
0.027120 0.027857 0.001397 0.050219 0.022838 0.017130
0.041089 0.009665 0.049580 0.029873 0.015768 0.007831 -
0.005551 -0.014140 -0.006819 -0.012025 0.006250 0.019221 -
0.022390 -0.027712
z106 -0.000250 0.013637 -0.047274 0.018311 0.022086
0.043978 0.019797 0.004829 0.025969 0.012640 0.009196 -
0.032106 -0.010137 -0.018893 -0.015261 0.000262 0.008923
0.009341 -0.006947 0.015654 -0.020557 -0.011130 0.015342 -
0.011201 -0.012946 0.019610 0.001329 -0.008728 -0.039452
0.017375 -0.043233 0.039071 0.046504 -0.000348 0.019934 -
0.008686 0.010611
x107 0.009372 -0.013920 0.052093 0.003662 0.001393 -
0.051735 0.065148 0.022281 -0.011069 0.034926 0.028745
0.005734 0.020985 -0.012210 0.009684 0.021184 0.022246
0.028706 0.028546 0.006919 0.038991 0.031253 -0.003351 -
0.015948 -0.011937 0.014816 -0.000947 0.000821 -0.016444 -
0.020038 -0.000575 0.053581 0.037390 -0.009642 -0.034739 -
0.022380 -0.005026
y107 -0.047023 -0.012089 0.040424 0.019975 0.008477
0.003793 0.012725 -0.020263 -0.024988 0.002574 0.010442
0.001163 -0.003724 0.020617 0.005987 -0.003178 -0.038880
0.026446 0.025139 0.000180 0.048649 0.024995 0.020220
0.045118 0.009051 0.050018 0.025586 0.023433 0.013333 -
0.007985 -0.012072 -0.002120 -0.011943 0.004613 0.022990 -
0.020859 -0.028741
z107 -0.000512 0.013307 -0.039674 0.018573 0.022450
0.031102 0.026825 0.008077 0.029904 0.017144 0.012700 -
0.032603 -0.009095 -0.019051 -0.016801 -0.003753 0.011216
0.009277 -0.006442 0.022236 -0.012966 -0.004980 0.019591 -
0.014326 -0.006176 0.012152 0.005450 -0.008292 -0.042110
0.018380 -0.042787 0.045013 0.045448 -0.001366 0.018679 -
0.018092 0.000627
x108 0.006707 -0.016620 0.055444 0.007187 0.002621 -
0.048969 0.067148 0.026891 -0.014362 0.037363 0.021997
0.009024 0.019300 -0.013747 0.002979 0.015579 0.019704
0.028024 0.026128 0.003465 0.045110 0.029623 -0.018668 -
0.022131 -0.002482 0.016746 0.002354 -0.000209 -0.015773 -
0.019871 0.007180 0.051650 0.028297 -0.011120 -0.037357 -
0.022498 -0.007134
y108 -0.049625 -0.009606 0.032203 0.020941 0.005662
0.010294 0.006011 -0.023251 -0.028241 -0.003931 0.001996

0.001286	-0.005143	0.018358	0.006817	-0.002458	-0.036696	
0.023322	0.020792	-0.000484	0.042747	0.027601	0.020472	
0.047253	0.008904	0.046039	0.021389	0.029056	0.017152	-
0.010928	-0.010491	0.000354	-0.010932	0.003546	0.029332	-
0.018957	-0.026238					
z108	-0.002113	0.010523	-0.034010	0.023298	0.019565	
0.014026	0.033020	0.011751	0.030902	0.017355	0.006756	-
0.032895	-0.010301	-0.021418	-0.013510	-0.001728	0.005974	
0.013475	-0.010786	0.025833	-0.003513	0.001359	0.013409	-
0.017394	0.004750	0.002220	0.010821	-0.008795	-0.043826	
0.016995	-0.042884	0.051501	0.040409	-0.004587	0.006482	-
0.016090	-0.012460					
x109	0.003220	-0.017841	0.057067	0.008351	0.004091	-
0.040019	0.063735	0.028883	-0.017313	0.039632	0.014989	
0.012985	0.016206	-0.013617	-0.005482	0.008015	0.019794	
0.024424	0.025242	0.000855	0.051062	0.027290	-0.033345	-
0.025571	0.005362	0.021942	0.003713	-0.002746	-0.013735	-
0.018662	0.019134	0.041634	0.017381	-0.007976	-0.034168	-
0.020835	-0.004539					
y109	-0.050252	-0.006235	0.023164	0.020729	0.003135	
0.017128	0.000278	-0.026540	-0.029026	-0.009341	-0.006580	
0.002044	-0.008655	0.015382	0.007482	-0.002995	-0.031617	
0.017219	0.017541	0.001953	0.032600	0.029416	0.019023	
0.045225	0.005268	0.041123	0.014952	0.032458	0.018503	-
0.014779	-0.008870	0.000346	-0.009587	0.002315	0.036728	-
0.017993	-0.022286					
z109	-0.004512	0.009148	-0.024580	0.023209	0.016711	
0.003655	0.037865	0.013342	0.032643	0.022751	0.007550	-
0.027687	-0.008277	-0.023502	-0.008942	-0.006544	0.013528	
0.019471	-0.006668	0.032186	0.008137	0.006058	0.011961	-
0.016476	0.009149	0.002037	0.011251	-0.009491	-0.042004	
0.017358	-0.038320	0.046935	0.037799	-0.001322	-0.002119	-
0.008615	-0.028253					
x110	-0.000710	-0.018910	0.054593	0.008962	0.006665	-
0.028360	0.057624	0.028712	-0.020402	0.038385	0.006227	
0.015710	0.013108	-0.010646	-0.016753	-0.000236	0.015957	
0.016079	0.021048	-0.002937	0.055459	0.021226	-0.053336	-
0.028271	0.014413	0.022835	0.002674	-0.004804	-0.010960	-
0.016789	0.033053	0.033516	0.005062	-0.009765	-0.031279	-
0.022073	0.004145					
y110	-0.049786	-0.002802	0.011499	0.019946	0.000388	
0.022472	-0.006299	-0.030172	-0.025897	-0.012566	-0.015058	
0.003474	-0.013103	0.011592	0.009137	-0.003183	-0.027057	
0.007610	0.015622	0.001921	0.019644	0.034570	0.015708	
0.039911	0.003675	0.030824	0.007950	0.035769	0.017283	-
0.020543	-0.005596	-0.004825	-0.009332	0.002359	0.044079	-
0.016828	-0.020533					
z110	-0.008972	0.006938	-0.017561	0.022097	0.013992	-
0.009259	0.042711	0.012567	0.033252	0.028273	0.010182	-
0.021723	-0.001298	-0.020283	-0.004138	-0.011916	0.021109	
0.018924	-0.008015	0.040388	0.020328	0.005751	0.002094	-
0.013136	0.009874	-0.007851	0.002510	-0.008299	-0.034426	
0.016659	-0.027392	0.046874	0.029718	-0.004526	-0.018311	-
0.002632	-0.029254					
x111	-0.004791	-0.019165	0.048689	0.010558	0.009658	-
0.011353	0.047131	0.028686	-0.024247	0.034314	-0.006797	
0.016578	0.006940	-0.009224	-0.029723	-0.008302	0.009388	
0.006580	0.020928	-0.007003	0.059496	0.015175	-0.072143	-
0.033706	0.024778	0.023020	0.003213	-0.007041	-0.007723	-

0.013277 0.047433 0.026925 -0.004728 -0.009155 -0.023036 -
0.021537 0.014759
y111 -0.047388 0.001463 -0.000342 0.019776 -0.002890
0.029360 -0.014766 -0.034286 -0.022394 -0.016325 -0.025185
0.002288 -0.023220 0.004412 0.012697 -0.003917 -0.020046 -
0.005671 0.014882 0.004781 0.001796 0.039308 0.014915
0.029187 0.000142 0.020157 0.001233 0.036207 0.017906 -
0.025795 -0.002842 -0.011231 -0.006342 0.004783 0.054439 -
0.016874 -0.017204
z111 -0.008006 0.003695 -0.008271 0.020943 0.013390 -
0.020436 0.044945 0.012725 0.034203 0.024265 0.016442 -
0.014461 0.007341 -0.023872 0.003578 -0.012904 0.029752
0.016850 -0.011874 0.043397 0.026750 0.001214 -0.017314 -
0.013193 0.013849 -0.006508 -0.012464 -0.006848 -0.030646
0.005315 -0.017050 0.038627 0.011830 -0.007318 -0.031145
0.011339 -0.039599
x112 -0.009709 -0.019959 0.047223 0.013046 0.013869 -
0.011470 0.040481 0.027757 -0.025902 0.024733 -0.007540
0.021751 0.002188 -0.008243 -0.028405 -0.015974 0.011211 -
0.001910 0.033835 0.012737 0.053292 0.017159 -0.057122 -
0.024034 0.027265 0.004886 0.012379 -0.014088 0.019066 -
0.031974 0.040636 0.014898 -0.000644 -0.015132 -0.010192 -
0.020713 0.024397
y112 -0.044087 0.003644 -0.002176 0.013582 -0.006034
0.028304 -0.011907 -0.034332 -0.014611 -0.007972 -0.028744 -
0.005688 -0.015454 0.008184 0.015191 0.006186 -0.019805 -
0.011289 -0.000574 -0.008340 -0.010956 0.024326 0.009122
0.027905 -0.012752 0.017308 -0.002221 0.032739 -0.007740 -
0.013668 -0.000075 -0.010678 -0.009465 0.018849 0.058241 -
0.026554 -0.017891
z112 -0.007449 0.000148 -0.000376 0.014913 0.016225 -
0.027083 0.054539 0.009282 0.027529 0.013883 0.024055 -
0.011859 0.005119 -0.024523 0.006316 -0.018162 0.015789
0.016256 -0.004730 0.043187 0.023286 0.007893 -0.024200 -
0.013854 0.025863 -0.003830 -0.003011 -0.011373 -0.015994
0.004113 -0.005038 0.035263 -0.000151 -0.006676 -0.038388
0.008304 -0.033293
x113 -0.013881 -0.020255 0.044763 0.016274 0.017696 -
0.010421 0.032902 0.026109 -0.027423 0.017298 -0.010311
0.024772 -0.001441 -0.006264 -0.027148 -0.020818 0.012336 -
0.009711 0.042679 0.027690 0.046545 0.019000 -0.042881 -
0.015223 0.028592 -0.007826 0.021084 -0.019831 0.036743 -
0.047983 0.036001 0.002883 0.006825 -0.020178 0.002332 -
0.019615 0.030002
y113 -0.040499 0.005672 -0.002521 0.008980 -0.009678
0.027010 -0.009941 -0.033152 -0.010420 -0.003946 -0.032909 -
0.014356 -0.012768 0.008877 0.017683 0.015641 -0.017521 -
0.016892 -0.011810 -0.016826 -0.022125 0.011313 0.004138
0.026061 -0.020645 0.015215 -0.004426 0.029334 -0.025773 -
0.004875 0.003064 -0.010407 -0.013150 0.029946 0.058119 -
0.034854 -0.017369
z113 -0.008188 -0.002763 0.001382 0.012086 0.014098 -
0.031728 0.057085 0.005995 0.022680 0.005629 0.023090 -
0.015671 0.002031 -0.032636 0.012137 -0.018840 0.001695
0.010423 -0.000478 0.039180 0.015666 0.011677 -0.033451 -
0.011172 0.034209 -0.010035 0.011827 -0.003229 0.002028
0.011015 0.002465 0.036911 -0.000935 -0.010807 -0.037809
0.004229 -0.028261
x114 -0.016907 -0.020235 0.042840 0.018413 0.020751 -
0.008019 0.027000 0.023133 -0.028717 0.011613 -0.011163

0.028068 -0.002813 -0.004310 -0.026757 -0.026591 0.014716 -
0.014189 0.047181 0.037707 0.038612 0.020515 -0.030336 -
0.008393 0.028303 -0.015229 0.026113 -0.022539 0.049755 -
0.059116 0.031047 -0.008117 0.009009 -0.023908 0.012623 -
0.018671 0.032605
y114 -0.036439 0.007584 -0.002967 0.003502 -0.011381
0.027503 -0.007667 -0.030971 -0.007164 0.001651 -0.030168 -
0.020154 -0.009477 0.010287 0.015396 0.020041 -0.013570 -
0.021472 -0.017594 -0.020225 -0.029117 -0.000131 0.002144
0.022418 -0.028382 0.015787 -0.007248 0.027856 -0.038356
0.001626 0.006880 -0.009152 -0.015099 0.036961 0.054749 -
0.038289 -0.016969
z114 -0.008692 -0.001124 0.003060 0.006199 0.014835 -
0.029402 0.055357 0.004888 0.022878 0.007302 0.027807 -
0.017302 -0.000221 -0.031255 0.010423 -0.020563 0.001919 -
0.000076 0.005894 0.042597 0.016738 0.009610 -0.033552 -
0.009208 0.031602 -0.010175 0.022370 0.006521 0.010096
0.012399 0.009059 0.033746 0.008859 -0.006899 -0.042002
0.010577 -0.015074
x115 -0.019673 -0.020445 0.039558 0.021339 0.022339 -
0.005896 0.021030 0.019625 -0.030271 0.005519 -0.015373
0.029819 -0.003372 -0.001810 -0.024566 -0.030028 0.012925 -
0.016025 0.048043 0.041202 0.032111 0.021958 -0.021629 -
0.001328 0.028047 -0.019374 0.030042 -0.024659 0.056645 -
0.061878 0.025380 -0.014908 0.008892 -0.026328 0.016945 -
0.016747 0.036022
y115 -0.032956 0.007632 -0.004747 0.001369 -0.013792
0.025946 -0.006351 -0.028028 -0.006477 0.002905 -0.031533 -
0.025654 -0.008756 0.008841 0.015874 0.026774 -0.013043 -
0.022811 -0.021676 -0.023034 -0.032759 -0.006679 0.001038
0.019884 -0.032820 0.015734 -0.008689 0.027517 -0.044253
0.008319 0.009385 -0.008960 -0.013417 0.042282 0.047898 -
0.037399 -0.016052
z115 -0.008812 -0.000405 0.004301 0.007504 0.011785 -
0.030335 0.050935 0.006046 0.023371 0.005775 0.024653 -
0.022174 -0.004898 -0.031264 0.013979 -0.016672 0.001013 -
0.005080 0.008860 0.039499 0.014580 0.014952 -0.032673 -
0.011621 0.030105 -0.013819 0.028249 0.015706 0.015976
0.011631 0.013672 0.022877 0.015427 -0.002569 -0.040624
0.025065 -0.017521
x116 -0.020658 -0.020207 0.034434 0.024031 0.023836 -
0.003887 0.015148 0.015037 -0.031740 0.001228 -0.020194
0.030461 -0.004135 0.000944 -0.022142 -0.030888 0.010883 -
0.016105 0.043811 0.037750 0.023924 0.022962 -0.015050
0.004785 0.026855 -0.017732 0.028518 -0.024166 0.061554 -
0.059321 0.020786 -0.022983 0.004619 -0.026510 0.019421 -
0.010566 0.040992
y116 -0.029952 0.006659 -0.006319 0.000419 -0.015436
0.024841 -0.004458 -0.024438 -0.007217 0.001380 -0.032903 -
0.029614 -0.010330 0.005991 0.017417 0.032337 -0.012422 -
0.022917 -0.022744 -0.021444 -0.031251 -0.012681 0.000080
0.015957 -0.033228 0.015257 -0.006216 0.025159 -0.045382
0.016146 0.007696 -0.007343 -0.010869 0.044634 0.037606 -
0.033237 -0.015795
z116 -0.008662 0.001578 0.007129 0.008465 0.010061 -
0.028009 0.048666 0.007098 0.026198 0.001906 0.020310 -
0.025671 -0.007903 -0.029820 0.018678 -0.016527 0.000149 -
0.009309 0.010987 0.039420 0.019021 0.015005 -0.032403 -
0.010623 0.028936 -0.015708 0.038447 0.019143 0.017089

0.012847 0.007589 0.017920 0.017934 0.002186 -0.043653
0.034408 -0.012885
x117 -0.020869 -0.019865 0.030062 0.025857 0.024825 -
0.001827 0.009410 0.008278 -0.031626 -0.000183 -0.025500
0.031709 -0.004769 0.005074 -0.017272 -0.030485 0.011317 -
0.015227 0.034899 0.028362 0.015100 0.022343 -0.010051
0.008121 0.025177 -0.009736 0.027179 -0.022002 0.055526 -
0.050482 0.013923 -0.029501 0.002071 -0.024041 0.019456 -
0.003747 0.040333
y117 -0.026220 0.005653 -0.008543 -0.001136 -0.014848
0.025622 -0.001493 -0.019668 -0.009322 -0.001314 -0.031913 -
0.033106 -0.012226 0.001580 0.014336 0.034400 -0.010366 -
0.024038 -0.019804 -0.013290 -0.027276 -0.017764 0.001659
0.012437 -0.033181 0.014602 -0.002797 0.022397 -0.034686
0.021413 0.006160 -0.006603 -0.006024 0.042211 0.021469 -
0.028273 -0.008105
z117 -0.006802 0.005936 0.007635 0.008828 0.007720 -
0.020734 0.046552 0.009181 0.026136 0.000223 0.018278 -
0.030974 -0.008253 -0.027267 0.012822 -0.020249 -0.001212 -
0.012171 0.015133 0.039852 0.022881 0.012483 -0.030056 -
0.006011 0.022086 -0.015350 0.040426 0.020734 0.027856
0.011803 0.007260 0.009998 0.017379 -0.000372 -0.050181
0.042144 0.001648
x118 -0.021010 -0.018095 0.023431 0.028406 0.023454
0.004152 0.005736 -0.000083 -0.030320 0.002886 -0.033953
0.027925 -0.002385 0.013555 -0.012670 -0.028459 0.014404 -
0.014206 0.023570 0.013973 0.007790 0.017382 -0.003933
0.013146 0.019610 0.004186 0.026092 -0.017975 0.039257 -
0.031597 0.009162 -0.036973 0.007522 -0.021334 0.010329
0.005005 0.032097
y118 -0.022035 0.003562 -0.009494 -0.003127 -0.012559
0.023845 0.000005 -0.013258 -0.014138 -0.007828 -0.028502 -
0.031809 -0.016859 -0.007320 0.010099 0.033215 -0.008511 -
0.024251 -0.014546 -0.001016 -0.021524 -0.016491 0.002724
0.005946 -0.029301 0.014839 -0.001852 0.022263 -0.015935
0.020179 0.006572 -0.002677 -0.001133 0.038062 0.002706 -
0.021395 0.004702
z118 -0.005231 0.009411 0.009801 0.008157 0.004846 -
0.016385 0.037608 0.009401 0.022539 -0.000760 0.017796 -
0.028821 -0.009989 -0.025433 0.006757 -0.025016 -0.002931 -
0.010628 0.019670 0.035957 0.022271 0.013112 -0.029130 -
0.007168 0.013645 -0.013173 0.038001 0.023430 0.032769
0.008610 0.005453 0.000890 0.017327 -0.002852 -0.045551
0.048202 0.003513
x119 -0.020326 -0.013625 0.016293 0.030195 0.020703
0.013058 0.000872 -0.007354 -0.028677 0.005230 -0.041138
0.021123 0.002625 0.024477 -0.015858 -0.031201 0.015276 -
0.009837 0.007479 -0.009873 -0.003766 0.016118 -0.004789
0.009845 0.006926 0.013974 0.019721 -0.016182 0.011747 -
0.006697 0.007632 -0.032061 0.015468 -0.015507 0.002843
0.004744 0.026045
y119 -0.018441 -0.001289 -0.008122 -0.003574 -0.011758
0.020108 0.001646 -0.006006 -0.025146 -0.010545 -0.021464 -
0.027779 -0.022739 -0.018265 0.005804 0.030487 -0.002418 -
0.019845 0.000612 0.021163 -0.007946 -0.012856 0.007511
0.001825 -0.020774 0.022242 -0.001455 0.022118 0.009895
0.011098 0.007073 -0.005966 -0.002197 0.028330 -0.019020 -
0.007492 0.000132
z119 -0.004346 0.013538 0.009464 0.010338 -0.002061 -
0.008397 0.028636 0.007316 0.017422 0.006555 0.014960 -

0.028118	-0.008484	-0.016585	0.001541	-0.025856	-0.000135	-
0.007121	0.023179	0.028240	0.023389	0.007549	-0.022596	-
0.000863	0.003416	-0.001082	0.037054	0.023437	0.028113	
0.009404	0.004001	-0.019010	0.022307	-0.008982	-0.041315	
0.060891	-0.008724					
x120	-0.019807	-0.014514	0.011421	0.035992	0.017206	
0.017438	-0.004103	-0.006499	-0.031006	0.004289	-0.048544	
0.016636	0.006396	0.020147	-0.015660	-0.032058	0.010429	
0.001319	-0.001327	-0.016644	-0.000856	0.023732	-0.002838	
0.002718	-0.000978	0.024458	0.011605	-0.014007	-0.002874	
0.011677	0.000375	-0.019203	0.003007	-0.019593	-0.009498	
0.003041	0.012650					
y120	-0.013877	-0.002511	-0.011687	-0.003167	-0.014585	
0.021254	0.002702	-0.004972	-0.037920	-0.011571	-0.014724	-
0.028923	-0.024346	-0.015594	-0.001153	0.027639	0.001426	-
0.012180	0.006035	0.022539	-0.002094	-0.015960	0.009517	-
0.003523	-0.021256	0.034231	0.007418	0.021102	0.021052	
0.018580	0.004721	-0.016040	0.016412	0.020365	-0.043478	
0.009701	-0.012326					
z120	-0.003181	0.019951	0.007622	0.014587	-0.007510	
0.002425	0.016610	0.006675	0.015470	0.013371	0.009882	-
0.029898	-0.006551	-0.007580	-0.004965	-0.028661	0.002916	-
0.000700	0.020815	0.019522	0.024237	0.001488	-0.017196	
0.002226	-0.016069	0.009460	0.028847	0.025214	0.017620	
0.011266	-0.003067	-0.033491	0.029462	-0.019536	-0.038667	
0.073702	-0.016778					
x121	-0.015978	-0.022028	0.017381	0.033782	0.022669	
0.020591	-0.003158	-0.007431	-0.025189	0.011485	-0.054155	
0.021891	0.009798	0.020189	-0.012703	-0.033611	0.016208	-
0.001849	-0.005247	-0.023623	-0.004775	0.026556	-0.012481	-
0.003667	-0.005011	0.015022	0.003241	-0.020708	-0.007019	
0.015386	-0.002613	-0.008172	0.004134	-0.022440	0.010953	-
0.007767	0.009829					
y121	-0.013315	-0.006751	-0.009717	-0.001439	-0.018535	
0.022921	0.000907	-0.008084	-0.039804	-0.013971	-0.014814	-
0.027688	-0.021063	-0.016037	0.002662	0.019678	-0.000100	-
0.014923	0.009491	0.019513	-0.001763	-0.021746	-0.001517	-
0.009614	-0.015711	0.028291	0.004739	0.011922	0.024905	
0.011959	0.003553	-0.017938	0.020654	0.020943	-0.046681	
0.012322	-0.005316					
z121	-0.008089	0.017917	0.009761	0.017662	-0.011782	-
0.005865	0.017666	0.002657	0.015845	0.005720	0.008843	-
0.030025	-0.003402	-0.001397	-0.004070	-0.028547	-0.000486	
0.000192	0.020148	0.021377	0.022920	0.004762	-0.022116	
0.008444	-0.022786	0.009602	0.016059	0.023868	0.013113	
0.010514	-0.001416	-0.029472	0.010930	-0.006511	-0.037168	
0.081399	-0.006591					
x122	-0.012118	-0.025375	0.016089	0.036130	0.026880	
0.023314	-0.002370	-0.007372	-0.024091	0.017679	-0.055824	
0.023510	0.010081	0.020940	-0.017766	-0.036101	0.020825	-
0.001527	-0.001025	-0.027451	0.001448	0.017919	-0.024184	-
0.006583	-0.011348	0.018722	-0.004790	-0.025845	-0.014771	
0.021939	0.000511	-0.005537	0.019255	-0.036117	0.011302	
0.004652	0.004565					
y122	-0.013579	-0.012376	-0.006705	-0.001696	-0.022472	
0.024330	-0.000935	-0.010151	-0.040648	-0.017414	-0.016540	-
0.026817	-0.015042	-0.016666	0.008952	0.013186	-0.000578	-
0.015993	0.004549	0.014660	-0.006228	-0.016252	-0.006957	-
0.012736	-0.008594	0.017491	0.004107	0.008554	0.027931	

0.008146 -0.000430 -0.015568 0.013153 0.022449 -0.041854 -
0.001278 -0.003258
z122 -0.012511 0.014190 0.013716 0.017950 -0.014496 -
0.013834 0.017949 0.000152 0.017151 -0.000849 0.007110 -
0.030030 0.002994 0.003357 -0.000232 -0.025561 -0.004938
0.000830 0.018393 0.021286 0.019553 0.013603 -0.022338
0.009439 -0.027063 0.000888 0.007527 0.020281 0.010946
0.011536 -0.004800 -0.014199 -0.010905 0.004836 -0.026337
0.070093 0.004651
x123 -0.009761 -0.028711 0.012797 0.040213 0.030357
0.022738 -0.000541 -0.008346 -0.023883 0.017019 -0.056324
0.021342 0.007985 0.021562 -0.018200 -0.035437 0.020905 -
0.001045 0.000038 -0.031525 0.005198 0.011343 -0.033208 -
0.008303 -0.011563 0.018531 -0.013761 -0.029687 -0.017757
0.021411 -0.000024 -0.000911 0.025489 -0.047876 0.009242
0.021653 0.009733
y123 -0.012399 -0.016113 -0.002806 -0.003409 -0.024861
0.024297 -0.003560 -0.010958 -0.039972 -0.015483 -0.019020 -
0.025015 -0.008262 -0.016196 0.010917 0.008516 0.001557 -
0.016948 0.001296 0.009703 -0.009729 -0.011080 -0.010771 -
0.012067 -0.004171 0.009050 0.006374 0.004030 0.026994
0.009129 0.000356 -0.016318 0.008566 0.026792 -0.032989 -
0.021009 -0.006592
z123 -0.015469 0.011527 0.016681 0.017288 -0.016412 -
0.018898 0.018520 -0.001724 0.018072 -0.004656 0.006557 -
0.030402 0.008373 0.006640 0.003113 -0.023634 -0.006561
0.000527 0.017528 0.020566 0.017017 0.016811 -0.022816
0.008768 -0.030518 -0.003219 0.001772 0.017625 0.007319
0.011343 -0.006725 -0.003220 -0.027233 0.013586 -0.018658
0.060412 0.010631
x124 -0.009037 -0.030849 0.010297 0.042947 0.032413
0.019473 0.001074 -0.009267 -0.023850 0.015254 -0.056712
0.017425 0.006978 0.021872 -0.016175 -0.032293 0.019699
0.001365 -0.000809 -0.035345 0.007859 0.009992 -0.038558 -
0.006635 -0.008343 0.016741 -0.019020 -0.031596 -0.018512
0.018297 -0.001204 0.000025 0.023449 -0.054833 0.011009
0.025568 0.017277
y124 -0.009706 -0.018505 -0.000861 -0.004758 -0.024975
0.023793 -0.004482 -0.011909 -0.038925 -0.013582 -0.018459 -
0.024124 -0.002765 -0.014692 0.011983 0.004355 0.003800 -
0.018829 -0.000389 0.004087 -0.014340 -0.010932 -0.012922 -
0.012633 -0.000905 0.000814 0.005991 0.002228 0.024935
0.011293 0.002950 -0.011310 0.008364 0.026101 -0.024793 -
0.030931 -0.008494
z124 -0.017275 0.010104 0.018943 0.015397 -0.017275 -
0.021165 0.019615 -0.003137 0.017714 -0.005905 0.007801 -
0.030239 0.012947 0.008800 0.005904 -0.023062 -0.005079 -
0.000836 0.017029 0.019363 0.014717 0.014354 -0.022839
0.008483 -0.033862 -0.005589 -0.002374 0.016019 0.002710
0.010203 -0.006233 0.005963 -0.037879 0.020709 -0.014255
0.050353 0.010954
x125 -0.007434 -0.032153 0.008178 0.042246 0.038528
0.016844 0.004693 -0.007578 -0.022349 0.015222 -0.052453
0.011717 0.011916 0.021274 -0.021318 -0.033401 0.027884
0.000584 0.006707 -0.037229 0.001449 0.011588 -0.038815
0.003482 -0.008736 0.007618 -0.020886 -0.030513 -0.018500
0.012637 -0.000472 0.002999 0.015435 -0.055396 0.017451
0.017736 0.022013
y125 -0.012060 -0.015591 0.007323 -0.006373 -0.034779
0.022177 -0.002807 -0.024902 -0.041319 -0.011246 -0.007848 -

0.014028	-0.007229	-0.004255	0.030046	0.006913	-0.012847	-
0.014183	-0.032245	-0.005194	0.006279	-0.020112	-0.009308	-
0.047901	0.012306	0.014133	-0.001324	0.002320	0.013392	
0.025014	0.006187	-0.006182	0.021734	0.030324	-0.028912	-
0.037260	0.003375					
z125	-0.018364	0.008832	0.019336	0.013849	-0.018460	-
0.022351	0.019498	-0.003328	0.016274	-0.007089	0.006033	-
0.031223	0.014954	0.009269	0.009361	-0.020290	-0.005556	-
0.001064	0.016265	0.016660	0.011026	0.010654	-0.024196	
0.009807	-0.034890	-0.008169	-0.002221	0.013552	-0.000166	
0.009124	-0.005897	0.014447	-0.044748	0.023526	-0.010806	
0.039310	0.008098					
x126	-0.007581	-0.030831	0.008445	0.041009	0.041405	
0.015582	0.010543	-0.009838	-0.020361	0.014501	-0.044909	
0.007481	0.013392	0.021334	-0.022593	-0.033250	0.032447	
0.000766	0.005693	-0.038122	-0.003077	0.009996	-0.036446	
0.004875	-0.008098	0.005457	-0.023266	-0.026928	-0.022043	
0.012233	0.001139	0.008465	0.009190	-0.052827	0.024117	
0.011277	0.028248					
y126	-0.008964	-0.016527	0.003731	-0.006517	-0.033501	
0.016826	-0.005065	-0.024839	-0.041625	-0.009540	-0.007769	-
0.015853	-0.002593	-0.001803	0.028497	0.008220	-0.011719	-
0.011533	-0.029737	-0.012798	0.002636	-0.016147	-0.008740	-
0.039295	0.021574	0.005324	-0.000070	0.003502	0.016881	
0.022863	0.010232	-0.006126	0.020172	0.019837	-0.027637	-
0.044055	0.011123					
z126	-0.019509	0.008171	0.018828	0.011932	-0.018886	-
0.022591	0.019658	-0.004469	0.012872	-0.007644	0.005988	-
0.031944	0.016533	0.007911	0.013884	-0.017438	-0.002996	-
0.001358	0.014677	0.010886	0.008118	0.003547	-0.025984	
0.010103	-0.030895	-0.015436	0.001818	0.011368	-0.003214	
0.005804	-0.002973	0.021236	-0.043932	0.023574	-0.009264	
0.022739	0.004429					
x127	-0.011375	-0.025630	0.009344	0.039186	0.040871	
0.010473	0.017158	-0.014929	-0.022102	0.010647	-0.032959	
0.004143	0.013115	0.021487	-0.016381	-0.031458	0.033537	
0.003392	-0.008816	-0.039333	0.002201	0.007894	-0.032376	
0.001614	-0.000744	0.006840	-0.025327	-0.022698	-0.032806	
0.009818	0.001164	0.013908	0.006101	-0.041396	0.031998	
0.004554	0.038180					
y127	-0.000308	-0.020593	-0.007749	-0.005753	-0.023866	
0.011561	-0.009100	-0.013664	-0.037600	-0.007164	-0.015132	-
0.027820	0.007184	-0.004129	0.011506	0.007680	-0.000053	-
0.010642	0.003557	-0.019694	-0.025791	-0.005320	-0.007842	-
0.001486	0.023766	-0.015673	0.005120	0.009367	0.035189	
0.015068	0.016440	-0.009263	0.005713	-0.006137	-0.024997	-
0.053125	0.016999					
z127	-0.018888	0.008572	0.018570	0.008092	-0.019381	-
0.019257	0.019122	-0.004567	0.009885	-0.005050	0.006691	-
0.032003	0.017124	0.005570	0.019788	-0.015354	0.000358	-
0.002235	0.013943	0.003921	0.004308	-0.008512	-0.025375	
0.007229	-0.028130	-0.018202	0.007360	0.011064	-0.006056	
0.005866	0.001129	0.026490	-0.039154	0.022631	-0.011187	
0.002743	-0.004287					
x128	-0.016516	-0.019928	0.007376	0.037857	0.040722	
0.007993	0.025039	-0.020068	-0.022653	0.001908	-0.019305	-
0.003059	0.012878	0.018410	-0.016429	-0.037530	0.036213	
0.005757	-0.009786	-0.036840	-0.000962	0.007819	-0.028976	
0.011296	0.006064	-0.000103	-0.026034	-0.013517	-0.040055	

0.006189 0.009392 0.011540 0.004530 -0.033811 0.035949 -
0.011833 0.044834
y128 0.005768 -0.020053 -0.011317 -0.006736 -0.021111
0.001342 -0.011536 -0.009251 -0.035721 -0.000543 -0.013328 -
0.031122 0.011108 0.002555 0.009969 0.019500 0.001431 -
0.006826 0.004113 -0.031098 -0.031526 -0.002316 0.002074
0.003355 0.032271 -0.015927 0.004287 0.012377 0.038660
0.019491 0.020877 -0.005815 -0.006050 -0.017780 -0.024823 -
0.057297 0.030333
z128 -0.015963 0.008997 0.017756 0.003558 -0.019284 -
0.014011 0.017324 -0.001819 0.005290 0.000924 0.004837 -
0.032708 0.017017 0.001249 0.026209 -0.010693 0.005740 -
0.004756 0.012243 -0.002916 -0.001414 -0.024051 -0.024417
0.002834 -0.024692 -0.017323 0.016336 0.009019 -0.010837
0.007635 0.005555 0.032404 -0.029853 0.018297 -0.013443 -
0.021153 -0.022386
x129 -0.032689 -0.011786 0.010259 0.031624 0.030735
0.009037 0.015793 -0.031823 -0.035718 -0.009612 -0.008146 -
0.013378 0.017545 -0.000241 0.000048 0.005420 0.027074 -
0.021358 -0.011429 -0.028786 -0.019081 -0.007394 -0.009570
0.023329 0.024092 -0.018814 -0.027619 0.000635 -0.014250 -
0.023405 0.012703 -0.025396 -0.004394 0.000043 0.011496 -
0.043616 0.073666
y129 0.020756 -0.010705 -0.018482 -0.022858 -0.016392
0.019946 -0.028787 -0.003203 -0.019718 0.016409 -0.016755 -
0.040886 0.015320 -0.008028 0.000830 0.039226 0.019029 -
0.012655 0.024775 -0.015283 -0.038124 -0.041215 0.030465 -
0.000883 0.073246 -0.010249 0.007974 0.003374 0.012992
0.030319 0.010167 -0.002627 -0.002280 -0.043661 -0.000638 -
0.050528 0.006562
z129 -0.007544 0.005966 0.017043 -0.008271 -0.029350 -
0.023080 -0.001496 -0.009300 0.013493 0.023410 0.002797 -
0.018009 0.021614 0.004868 0.017410 -0.001623 -0.003745 -
0.018471 0.011628 -0.023717 -0.019822 -0.013340 -0.037232 -
0.053235 0.008415 -0.051993 0.007486 0.012256 -0.018461
0.015239 0.029358 0.056811 0.002176 -0.007971 -0.023250
0.005211 -0.058620
x130 -0.033173 -0.008991 0.001237 0.027993 0.039318
0.005073 0.017446 -0.033990 -0.042305 -0.022132 -0.001781 -
0.024587 0.023086 -0.002135 0.003362 0.008674 0.034796 -
0.018006 -0.002880 -0.020334 -0.014538 0.004173 -0.021868
0.017478 0.044940 -0.009856 -0.010962 0.012421 -0.012620 -
0.021842 0.019159 -0.012768 0.010329 -0.006563 0.005346 -
0.029530 0.054091
y130 0.023071 -0.010573 -0.017659 -0.027583 -0.009033
0.018161 -0.029348 -0.001162 -0.016301 0.018134 -0.017180 -
0.030919 0.016315 -0.003402 -0.004658 0.041452 0.020892 -
0.008534 0.016681 -0.014266 -0.034194 -0.038136 0.017379
0.002080 0.067819 -0.010554 0.015890 0.007477 0.006279
0.038180 0.009768 0.007026 -0.002238 -0.041228 -0.007966 -
0.042608 -0.003167
z130 -0.007739 0.004029 0.013818 -0.006770 -0.027145 -
0.020306 0.000957 -0.007576 0.005667 0.025161 0.003153 -
0.018868 0.023549 0.000341 0.014441 -0.001962 -0.002898 -
0.014553 0.015141 -0.025550 -0.022376 -0.018672 -0.034731 -
0.035582 0.011641 -0.056961 -0.002070 0.018311 -0.019738 -
0.000913 0.034741 0.048903 -0.011795 -0.002780 -0.019471 -
0.006393 -0.034116
x131 -0.033870 -0.006222 -0.005429 0.021961 0.046584
0.001256 0.018188 -0.036512 -0.043404 -0.031492 0.005541 -

0.030638	0.024969	-0.001658	0.009553	0.013351	0.037762	-
0.016469	0.001581	-0.014153	-0.007806	0.016897	-0.028235	
0.002912	0.059468	-0.001553	0.010645	0.023014	-0.007819	-
0.015442	0.021099	0.002556	0.018787	-0.011039	-0.002248	-
0.012496	0.035619					
y131	0.025212	-0.009795	-0.016273	-0.030074	-0.002564	
0.018038	-0.028296	0.000520	-0.014768	0.017155	-0.016502	-
0.022047	0.017434	-0.000095	-0.006801	0.039755	0.021879	-
0.005183	0.009562	-0.011592	-0.031329	-0.036079	0.005134	
0.003516	0.059326	-0.009835	0.020191	0.010786	-0.000872	
0.044161	0.005663	0.015283	0.002363	-0.040741	-0.012535	-
0.034870	-0.009860					
z131	-0.009836	0.003950	0.015167	-0.008103	-0.025526	-
0.015321	0.003435	-0.008932	0.001946	0.027274	0.007602	-
0.016188	0.020798	-0.000886	0.022669	0.001143	-0.010542	-
0.014037	0.010134	-0.026441	-0.018729	-0.019551	-0.025384	-
0.042045	0.012917	-0.055834	-0.001195	0.020360	-0.020483	-
0.002680	0.027957	0.051063	-0.020116	0.004447	-0.020412	-
0.009398	-0.014225					
x132	-0.033629	-0.004895	-0.012096	0.016552	0.053106	-
0.000923	0.019057	-0.035766	-0.044014	-0.038356	0.010243	-
0.034550	0.025305	-0.002654	0.009958	0.014745	0.043295	-
0.014369	0.011300	-0.009079	-0.004725	0.028422	-0.035383	-
0.003732	0.066580	0.003979	0.024462	0.031077	-0.002578	-
0.010830	0.024759	0.012151	0.022069	-0.018197	-0.005595	-
0.004570	0.008840					
y132	0.027397	-0.009517	-0.015367	-0.031357	0.002364	
0.017101	-0.028018	0.002468	-0.013263	0.016022	-0.016801	-
0.014017	0.017787	0.003916	-0.007221	0.036473	0.020640	-
0.002489	0.001568	-0.009109	-0.028029	-0.035123	-0.003060	
0.005821	0.052446	-0.010800	0.024687	0.012250	-0.007573	
0.049005	0.002070	0.021426	0.006601	-0.037343	-0.016463	-
0.027992	-0.012492					
z132	-0.010728	0.001690	0.013657	-0.008700	-0.019201	-
0.008227	0.007018	-0.003185	-0.000649	0.030848	0.008329	-
0.015636	0.017725	-0.006556	0.019442	0.001901	-0.007785	-
0.013513	0.020442	-0.025649	-0.021119	-0.013599	-0.021935	-
0.037205	0.008585	-0.059542	-0.003658	0.021263	-0.016601	-
0.006564	0.026646	0.054428	-0.024952	-0.001407	-0.008369	-
0.021829	-0.017739					
x133	-0.033504	-0.004474	-0.017492	0.011080	0.056775	-
0.002852	0.019563	-0.035367	-0.043556	-0.043845	0.014511	-
0.034055	0.025134	-0.001288	0.010801	0.014120	0.044007	-
0.011910	0.014867	-0.007370	-0.001274	0.034601	-0.039913	-
0.008120	0.069111	0.010344	0.032877	0.035851	-0.000252	-
0.005501	0.022924	0.015469	0.022210	-0.017844	-0.013487	
0.003344	-0.009018					
y133	0.029799	-0.009284	-0.014648	-0.032148	0.005773	
0.016141	-0.027252	0.004399	-0.012267	0.015133	-0.016929	-
0.008715	0.016837	0.006590	-0.007593	0.032751	0.019771	-
0.000619	-0.002867	-0.005451	-0.025796	-0.033451	-0.010177	
0.008964	0.045536	-0.012005	0.029365	0.014190	-0.012727	
0.051640	-0.000217	0.025111	0.006231	-0.035999	-0.017423	-
0.022329	-0.013676					
z133	-0.012777	0.000119	0.013197	-0.008490	-0.014313	-
0.001368	0.011616	-0.002247	-0.002367	0.034225	0.010648	-
0.011639	0.013788	-0.007088	0.019035	0.000066	-0.012171	-
0.012703	0.018851	-0.024098	-0.017441	-0.011199	-0.022013	-
0.036355	0.005115	-0.055368	-0.008757	0.022237	-0.021033	-

0.008383 0.025780 0.049275 -0.022655 -0.001262 -0.008182 -
0.018623 -0.018663
x134 -0.033103 -0.004589 -0.022043 0.006378 0.056884 -
0.004606 0.017490 -0.032773 -0.043741 -0.048540 0.016980 -
0.032310 0.022942 -0.000351 0.010737 0.013487 0.042614 -
0.007359 0.017096 -0.007873 0.000653 0.038166 -0.041955 -
0.007707 0.066302 0.015933 0.038804 0.037750 0.002573 -
0.000528 0.019780 0.014039 0.015461 -0.015286 -0.019806
0.004701 -0.022393
y134 0.032268 -0.009343 -0.013497 -0.033082 0.009023
0.015881 -0.025146 0.005041 -0.010254 0.015687 -0.015058 -
0.003754 0.016583 0.009560 -0.008948 0.026424 0.019464 -
0.001218 -0.004870 -0.000153 -0.023638 -0.032193 -0.013624
0.010872 0.038479 -0.013211 0.031443 0.016418 -0.017574
0.051906 -0.001783 0.027107 0.006168 -0.032585 -0.018315 -
0.015961 -0.012833
z134 -0.015894 -0.001857 0.012854 -0.006642 -0.012349
0.003845 0.012522 0.000123 -0.005445 0.034860 0.010120 -
0.010822 0.009224 -0.006970 0.016503 0.000415 -0.017626 -
0.008555 0.019081 -0.020763 -0.012373 -0.004653 -0.022857 -
0.030342 0.000725 -0.050291 -0.009881 0.018731 -0.024866 -
0.005864 0.023304 0.046409 -0.021446 -0.003040 -0.000303 -
0.024116 -0.023303
x135 -0.032416 -0.005180 -0.026242 0.001104 0.055232 -
0.005446 0.015242 -0.029871 -0.043628 -0.051471 0.019812 -
0.026523 0.020552 0.000721 0.009155 0.011586 0.042200 -
0.003965 0.017043 -0.010563 0.002373 0.037646 -0.040457 -
0.004583 0.059564 0.017693 0.041856 0.039451 0.007190
0.001858 0.014491 0.009578 0.002326 -0.008520 -0.026886
0.005514 -0.025826
y135 0.034933 -0.010057 -0.012450 -0.032822 0.010712
0.014888 -0.023450 0.006164 -0.008249 0.015423 -0.014660 -
0.001099 0.014177 0.011396 -0.008538 0.019084 0.016219 -
0.002195 -0.005104 0.005362 -0.022685 -0.030800 -0.015944
0.011697 0.031761 -0.013227 0.032283 0.017303 -0.022104
0.051153 -0.002886 0.027243 0.004289 -0.029817 -0.018102 -
0.010784 -0.011727
z135 -0.019123 -0.004092 0.010991 -0.006105 -0.009502
0.009927 0.015202 -0.000075 -0.009737 0.036987 0.012420 -
0.007166 0.006506 -0.007268 0.011838 0.000463 -0.019885 -
0.006579 0.016517 -0.015811 -0.004512 -0.000058 -0.024209 -
0.026485 -0.001383 -0.049647 -0.009728 0.018634 -0.026813 -
0.007756 0.021257 0.043532 -0.023661 -0.001557 0.007567 -
0.021070 -0.018686
x136 -0.031138 -0.006566 -0.029661 -0.003397 0.051074 -
0.005564 0.011729 -0.025412 -0.042571 -0.053217 0.020995 -
0.018313 0.016904 0.001815 0.007193 0.008277 0.041308 -
0.000423 0.016602 -0.015478 0.001336 0.034108 -0.036834
0.000949 0.049044 0.017606 0.041312 0.039705 0.011305
0.004210 0.009141 -0.000501 -0.015746 0.002313 -0.035967
0.000333 -0.025575
y136 0.037560 -0.010538 -0.011283 -0.033403 0.012060
0.014205 -0.021119 0.007458 -0.006448 0.016169 -0.012258
0.000904 0.011238 0.013211 -0.007905 0.010130 0.011576 -
0.004474 -0.004056 0.011843 -0.021161 -0.031390 -0.016100
0.011511 0.024413 -0.011798 0.031879 0.017825 -0.025569
0.048937 -0.004538 0.026295 0.000663 -0.027087 -0.014965 -
0.004918 -0.008812
z136 -0.021789 -0.008184 0.008153 -0.002793 -0.007800
0.014680 0.017148 -0.000356 -0.014528 0.036147 0.009732 -

0.006019	0.002684	-0.008819	0.008303	0.000131	-0.023198	-
0.005001	0.014440	-0.010464	0.001458	0.007246	-0.028189	-
0.026033	0.000500	-0.052444	-0.008084	0.015889	-0.030254	-
0.010850	0.023859	0.038157	-0.024365	-0.002369	0.015124	-
0.023763	-0.018873					
x137	-0.029289	-0.008596	-0.031623	-0.008397	0.044744	-
0.005395	0.006770	-0.019127	-0.039802	-0.052884	0.021419	-
0.006579	0.013457	0.003143	0.005337	0.002959	0.040284	
0.003744	0.015656	-0.025201	-0.002371	0.026484	-0.030860	
0.011068	0.034277	0.018307	0.036278	0.039671	0.016429	
0.007349	0.000393	-0.015700	-0.039729	0.016599	-0.047298	-
0.007366	-0.020402					
y137	0.039975	-0.012161	-0.010647	-0.031772	0.011860	
0.012720	-0.018252	0.007945	-0.005605	0.015002	-0.011341	
0.001352	0.005973	0.015535	-0.007214	0.000597	0.003324	-
0.008181	-0.003677	0.020192	-0.018619	-0.030705	-0.014636	
0.010135	0.015453	-0.012904	0.030927	0.015650	-0.028853	
0.044567	-0.006287	0.024018	-0.004191	-0.025291	-0.010340	
0.000808	-0.003966					
z137	-0.023669	-0.011378	0.006804	-0.002688	-0.004774	
0.021431	0.017945	-0.001293	-0.017279	0.039094	0.012111	-
0.002882	0.003973	-0.010240	0.004310	-0.003890	-0.017947	-
0.002815	0.017679	-0.007696	0.002227	0.009897	-0.030721	-
0.021452	0.004564	-0.047611	-0.007292	0.015553	-0.029721	-
0.009149	0.024789	0.031538	-0.022858	-0.000032	0.017026	-
0.024341	-0.023380					
x138	-0.026796	-0.011002	-0.032925	-0.012863	0.036435	-
0.004328	-0.000362	-0.011412	-0.036319	-0.051552	0.021969	
0.006947	0.007387	0.004493	0.002614	-0.002013	0.036487	
0.008183	0.012844	-0.036321	-0.006625	0.016493	-0.020237	
0.024068	0.014362	0.018353	0.027321	0.037310	0.021879	
0.009190	-0.008816	-0.034659	-0.069553	0.034962	-0.058400	-
0.018070	-0.009386					
y138	0.042342	-0.013843	-0.010116	-0.029741	0.010341	
0.012525	-0.014318	0.007058	-0.004672	0.015118	-0.009761	
0.000815	0.000741	0.017071	-0.007688	-0.010043	-0.004767	-
0.012363	-0.000223	0.029482	-0.015352	-0.029780	-0.011202	
0.008369	0.007031	-0.015422	0.029672	0.013368	-0.032422	
0.037913	-0.005506	0.017927	-0.009036	-0.023118	-0.003516	
0.004472	0.000623					
z138	-0.024814	-0.014982	0.005512	-0.001131	-0.001155	
0.027166	0.014944	-0.000812	-0.018189	0.038860	0.015157	-
0.001688	0.002073	-0.010728	0.004605	-0.005511	-0.016003	-
0.001053	0.019155	-0.005062	0.000604	0.013708	-0.029402	-
0.018661	0.006516	-0.041620	-0.009448	0.014717	-0.025309	-
0.005205	0.028368	0.027517	-0.024098	0.006675	0.018910	-
0.022540	-0.024409					
x139	-0.024737	-0.011128	-0.044096	-0.014309	0.034517	-
0.001321	-0.001379	-0.015204	-0.035361	-0.050405	0.019675	
0.010364	0.010232	0.011336	-0.001597	-0.001956	0.021514	
0.008416	0.012343	-0.033938	0.000612	0.024619	-0.015821	
0.017248	0.008406	0.023705	0.023282	0.025983	0.021428	
0.015303	-0.008080	-0.034618	-0.048628	0.027094	-0.047108	-
0.015406	-0.003729					
y139	0.041522	-0.015995	-0.004933	-0.027031	0.006198	
0.012553	-0.012133	0.004643	-0.005958	0.014633	-0.014355	-
0.002838	-0.000735	0.019893	-0.007001	-0.012406	-0.009634	-
0.014838	0.000766	0.037442	-0.018481	-0.024263	-0.010700	
0.008693	0.004028	-0.012479	0.030610	0.011923	-0.027642	

0.030873	-0.005759	0.011202	-0.007641	-0.021029	0.007827	
0.002462	-0.001014					
z139	-0.027999	-0.019382	0.003872	0.001897	0.001450	
0.030114	0.012345	-0.000494	-0.020333	0.037255	0.015948	-
0.001038	0.003216	-0.011644	0.002570	-0.005103	-0.011968	-
0.000834	0.012791	-0.012820	-0.003532	0.013470	-0.031645	-
0.016484	0.013006	-0.035078	-0.010559	0.014759	-0.016376	-
0.005858	0.029605	0.021165	-0.026632	0.012934	0.012078	-
0.020552	-0.031213					
x140	-0.022568	-0.010858	-0.053652	-0.015302	0.032404	
0.001443	-0.002545	-0.018459	-0.034369	-0.049749	0.017058	
0.012204	0.012139	0.016262	-0.004259	-0.000600	0.007624	
0.008558	0.011710	-0.031348	0.007074	0.032479	-0.010831	
0.011213	0.003359	0.026597	0.020487	0.015564	0.021164	
0.019705	-0.008070	-0.033262	-0.030094	0.020446	-0.036094	-
0.014605	0.002210					
y140	0.039885	-0.018712	0.000117	-0.024406	0.003673	
0.011420	-0.009655	0.002454	-0.005461	0.016007	-0.015446	-
0.007247	-0.000739	0.024202	-0.008163	-0.014695	-0.014948	-
0.018860	0.001674	0.041400	-0.019415	-0.016900	-0.010341	
0.008815	0.002435	-0.010523	0.031787	0.011106	-0.024196	
0.023429	-0.003862	0.008071	-0.008406	-0.016861	0.017211	-
0.000081	-0.005635					
z140	-0.029096	-0.019302	0.005136	0.003458	0.000954	
0.039424	0.009988	-0.000694	-0.025033	0.032585	0.014827	
0.002182	0.000635	-0.019847	0.003610	-0.007193	0.003674	
0.003854	0.012294	-0.010380	-0.008376	0.004326	-0.028859	-
0.018015	0.013312	-0.022338	-0.012889	0.014441	-0.006973	-
0.003149	0.024219	0.015685	-0.017813	0.016263	0.006778	-
0.015060	-0.029341					
x141	-0.020259	-0.010523	-0.061643	-0.016318	0.030046	
0.003502	-0.003257	-0.020738	-0.032667	-0.048506	0.014307	
0.012837	0.014064	0.021295	-0.005965	0.001180	-0.005030	
0.008530	0.011116	-0.027478	0.011894	0.037718	-0.005857	
0.006043	-0.003539	0.030198	0.016550	0.007103	0.019884	
0.023686	-0.009292	-0.030128	-0.014888	0.014132	-0.025919	-
0.013303	0.006915					
y141	0.038221	-0.020538	0.007353	-0.020485	0.000911	
0.010340	-0.007378	-0.000177	-0.004188	0.017292	-0.018266	-
0.011790	-0.002835	0.026223	-0.007009	-0.016674	-0.017063	-
0.020293	0.002631	0.047373	-0.022707	-0.012579	-0.009397	
0.009576	0.000157	-0.006834	0.030105	0.010261	-0.020876	
0.018737	-0.004780	0.007044	-0.009309	-0.011997	0.024445	
0.000350	-0.009309					
z141	-0.030114	-0.019062	0.003125	0.003773	0.002644	
0.046863	0.010134	-0.002694	-0.028693	0.028201	0.019882	
0.007309	0.000386	-0.023751	0.003738	-0.009641	0.012664	
0.006109	0.011204	-0.012110	-0.010221	0.002771	-0.026444	-
0.020682	0.016812	-0.016781	-0.009297	0.011723	-0.000030	-
0.000604	0.022651	0.010212	-0.006240	0.018163	0.002672	-
0.015852	-0.024897					
x142	-0.017555	-0.009890	-0.067831	-0.017645	0.027532	
0.005102	-0.003638	-0.022236	-0.030195	-0.046793	0.012376	
0.013124	0.017150	0.025017	-0.008325	0.001863	-0.014216	
0.007183	0.011675	-0.024465	0.016691	0.041019	-0.001557	
0.003724	-0.010001	0.030016	0.013473	0.003136	0.018115	
0.025029	-0.011182	-0.027089	-0.002663	0.008225	-0.017977	-
0.012904	0.012796					
y142	0.036594	-0.022585	0.014706	-0.017281	-0.002231	
0.008551	-0.004265	-0.002736	-0.002950	0.017992	-0.016106	-

0.013931	-0.005558	0.026919	-0.007386	-0.017455	-0.018989	-
0.024454	0.006187	0.047817	-0.024552	-0.010553	-0.010078	
0.010002	-0.002341	-0.005959	0.030017	0.011428	-0.019838	
0.012393	-0.006041	0.004133	-0.007408	-0.007902	0.030188	-
0.002451	-0.010093					
z142	-0.031269	-0.020628	-0.004136	0.005354	0.007851	
0.051252	0.008155	-0.001195	-0.030321	0.026271	0.017655	
0.009817	0.000402	-0.021579	0.002823	-0.006840	0.013049	
0.010872	0.009723	-0.009835	-0.014433	0.008101	-0.021995	-
0.019080	0.015445	-0.007406	-0.007361	-0.002932	0.008816	
0.009074	0.020814	0.010547	-0.003360	0.020785	0.001613	-
0.010280	-0.029081					
x143	-0.014724	-0.009283	-0.072629	-0.019664	0.025326	
0.006628	-0.003247	-0.024004	-0.028524	-0.044811	0.009714	
0.011997	0.019942	0.028040	-0.009622	0.003801	-0.021920	
0.005873	0.011445	-0.020150	0.020691	0.043270	0.002936	
0.001583	-0.016478	0.028468	0.012750	-0.003892	0.017141	
0.025426	-0.016378	-0.024162	0.004151	0.002109	-0.011867	-
0.013065	0.017020					
y143	0.033784	-0.025152	0.022813	-0.014715	-0.003892	
0.006530	0.000695	-0.003944	-0.001377	0.018960	-0.013017	-
0.017148	-0.008554	0.026355	-0.008251	-0.012729	-0.020004	-
0.027112	0.010315	0.046970	-0.027721	-0.007469	-0.011688	
0.007503	-0.004274	-0.002393	0.027498	0.009757	-0.017788	
0.008550	-0.007874	0.001130	-0.005053	-0.002178	0.033758	-
0.002712	-0.011577					
z143	-0.031315	-0.020925	-0.011352	0.006664	0.011139	
0.055609	0.005140	0.003036	-0.027775	0.024145	0.017983	
0.013990	0.002967	-0.017436	-0.000503	-0.007126	0.013083	
0.009565	0.010519	-0.010363	-0.016925	0.008997	-0.019219	-
0.015276	0.014271	-0.000607	-0.009672	-0.001491	0.013579	
0.016167	0.020986	0.013400	0.004195	0.020360	0.001807	-
0.007366	-0.028058					
x144	-0.011139	-0.007924	-0.074854	-0.022006	0.022364	
0.008159	-0.003424	-0.023880	-0.025071	-0.042888	0.007799	
0.008998	0.022282	0.029910	-0.009996	0.006272	-0.028501	
0.001425	0.011468	-0.015713	0.023664	0.043355	0.008946	
0.001153	-0.024515	0.025551	0.010453	-0.004724	0.017012	
0.024333	-0.022188	-0.015900	0.010432	-0.003623	-0.005029	-
0.014558	0.017179					
y144	0.031890	-0.026110	0.032340	-0.010717	-0.005616	
0.004164	0.003198	-0.007027	0.001879	0.021276	-0.010586	-
0.017787	-0.013968	0.024451	-0.007087	-0.013377	-0.019795	-
0.029181	0.013461	0.045649	-0.029362	-0.006411	-0.010519	
0.009346	-0.004027	-0.002230	0.024536	0.010840	-0.016474	
0.006907	-0.010154	0.001743	-0.004736	0.003478	0.037259	-
0.002654	-0.014141					
z144	-0.032447	-0.022672	-0.022191	0.007826	0.015320	
0.058838	0.005476	0.004943	-0.028284	0.022286	0.016581	
0.020180	0.005829	-0.012004	-0.001744	-0.006103	0.011240	
0.013909	0.010264	-0.010709	-0.019550	0.011987	-0.022680	-
0.018197	0.013109	0.005846	-0.006998	-0.012034	0.016102	
0.024279	0.016293	0.007384	0.007812	0.018564	-0.002712	-
0.003370	-0.016847					
x145	-0.008453	-0.006811	-0.076836	-0.023867	0.020837	
0.009474	-0.002499	-0.022768	-0.021882	-0.040412	0.006017	
0.006837	0.026592	0.031882	-0.010957	0.007773	-0.032233	
0.000250	0.012603	-0.010741	0.025742	0.041874	0.012379	-
0.000303	-0.030833	0.020869	0.010061	-0.004208	0.015422	

0.022879 -0.027718 -0.010205 0.014693 -0.010293 0.001014 -
0.016215 0.022934
y145 0.028977 -0.027181 0.043375 -0.007042 -0.007028
0.001508 0.007485 -0.009456 0.003858 0.022266 -0.007091 -
0.018136 -0.019691 0.019658 -0.006782 -0.010612 -0.015508 -
0.027946 0.018149 0.043570 -0.032371 -0.007101 -0.010775
0.009516 -0.002971 -0.001679 0.020337 0.009644 -0.014882
0.005945 -0.012535 0.000111 -0.001793 0.011054 0.038347 -
0.001680 -0.012670
z145 -0.031263 -0.022796 -0.032230 0.007642 0.017098
0.062017 0.003771 0.008518 -0.025998 0.019440 0.014520
0.023631 0.006642 -0.005595 -0.002343 -0.001143 0.005207
0.010030 0.007670 -0.010206 -0.020959 0.014066 -0.020694 -
0.014809 0.007885 0.012342 -0.006741 -0.018063 0.019476
0.031863 0.010958 0.009971 0.009514 0.017161 -0.007149
0.001224 -0.014935
x146 -0.004856 -0.005831 -0.077586 -0.026284 0.018086
0.010067 -0.001373 -0.021247 -0.017813 -0.038179 0.003726
0.003596 0.030121 0.032691 -0.009667 0.010414 -0.035688 -
0.002420 0.013057 -0.006342 0.024898 0.039949 0.015921
0.000028 -0.037276 0.016186 0.009760 -0.004608 0.014754
0.018203 -0.034270 -0.000844 0.013818 -0.013050 0.003261 -
0.017893 0.023703
y146 0.026059 -0.028106 0.054977 -0.003274 -0.008048 -
0.001533 0.011822 -0.012007 0.007701 0.024100 -0.002150 -
0.016773 -0.026955 0.014285 -0.006535 -0.007905 -0.011154 -
0.027102 0.023571 0.039740 -0.035216 -0.008674 -0.010740
0.010047 -0.001085 -0.000709 0.016002 0.008011 -0.014049
0.006280 -0.015508 0.001153 0.000675 0.020678 0.037736
0.000580 -0.013250
z146 -0.030441 -0.022765 -0.044851 0.007583 0.021243
0.065294 0.002432 0.013232 -0.023363 0.017778 0.013030
0.027520 0.010325 0.004018 -0.004823 0.002871 -0.002078
0.006004 0.007422 -0.008514 -0.019913 0.015980 -0.020326 -
0.015844 0.002732 0.015136 -0.005422 -0.021165 0.020928
0.042284 0.003770 0.010581 0.015013 0.007057 -0.007306
0.005038 -0.004918
x147 -0.001188 -0.004213 -0.075363 -0.027838 0.015198
0.010568 0.000116 -0.018602 -0.014565 -0.034672 0.000999 -
0.000446 0.032327 0.031017 -0.008732 0.013272 -0.036278 -
0.003284 0.014233 0.000367 0.025188 0.037509 0.019222
0.005080 -0.045953 0.009925 0.006335 -0.004608 0.012196
0.018915 -0.042951 0.005906 0.014839 -0.016233 0.005410 -
0.022035 0.026341
y147 0.023317 -0.029173 0.066906 0.000967 -0.007700 -
0.004923 0.016089 -0.015204 0.011894 0.027234 0.005101 -
0.014793 -0.034108 0.008045 -0.005899 -0.006073 -0.007287 -
0.025469 0.029449 0.033569 -0.037921 -0.010818 -0.010420
0.008212 0.001428 -0.001836 0.008719 0.005954 -0.014430
0.008781 -0.019873 0.001489 0.004448 0.026529 0.036939
0.003097 -0.012248
z147 -0.029097 -0.023542 -0.061621 0.007182 0.025360
0.067653 0.001242 0.017949 -0.018016 0.015303 0.011275
0.030795 0.014910 0.015997 -0.005332 0.006893 -0.014109 -
0.000204 0.005965 -0.009276 -0.021236 0.017183 -0.021728 -
0.020788 -0.001868 0.016548 -0.000234 -0.023766 0.022618
0.045117 -0.003737 0.016578 0.016581 -0.004485 -0.008342
0.010486 0.003837
x148 0.000826 -0.001585 -0.062469 -0.029534 0.010082
0.015026 0.006189 -0.020760 -0.015033 -0.031361 0.002157 -

0.006823	0.023206	0.020789	-0.002159	0.018130	-0.029043		
0.002531	0.020151	0.011427	0.025639	0.031676	0.022782		
0.006331	-0.046174	0.004223	0.001587	-0.000855	0.008363		
0.005790	-0.045967	0.006346	0.013100	-0.007486	0.009451	-
0.021740	0.019970					
y148	0.020177	-0.028672	0.071561	0.006072	-0.002426	-
0.011134	0.017383	-0.012859	0.018892	0.027041	0.006130	-
0.006657	-0.040830	0.008941	-0.003601	-0.006646	-0.004238	-
0.025589	0.031161	0.020083	-0.036377	-0.013257	-0.010295	
0.004289	-0.000665	0.000846	0.009593	-0.006410	-0.014693	
0.022353	-0.010379	0.004917	0.007028	0.028349	0.035789	
0.014259	-0.001149					
z148	-0.028293	-0.019924	-0.068886	0.007723	0.028414	
0.075173	0.003444	0.019100	-0.016067	0.015285	0.013427	
0.031209	0.014044	0.017798	-0.007866	0.012893	-0.022690	
0.004989	0.002622	-0.005899	-0.016817	0.018703	-0.020524	-
0.013124	-0.015550	0.015995	-0.001539	-0.019785	0.016619	
0.037208	-0.008697	0.000802	0.014433	-0.002982	-0.004563	
0.013640	0.009846					
x149	0.003937	0.000691	-0.049078	-0.029525	0.007384	
0.014994	0.012072	-0.019391	-0.013970	-0.026050	0.002702	-
0.006611	0.015516	0.012666	0.002365	0.022667	-0.024945	
0.007056	0.024960	0.019084	0.024898	0.029192	0.026453	
0.005494	-0.050842	0.000533	-0.001093	0.000427	0.007110	
0.001057	-0.044666	0.005105	0.015121	-0.006814	0.009950	-
0.019741	0.014144					
y149	0.016943	-0.027727	0.074691	0.010788	0.001500	-
0.017463	0.018614	-0.013578	0.022326	0.026398	0.006524	-
0.001221	-0.045843	0.009680	-0.001184	-0.008113	-0.001153	-
0.022454	0.034559	0.009004	-0.034002	-0.016693	-0.008876	
0.000464	0.000636	0.004117	0.010796	-0.015663	-0.015000	
0.031721	-0.005475	0.007605	0.008314	0.030903	0.034521	
0.019829	0.007858					
z149	-0.028075	-0.016620	-0.076453	0.006822	0.029092	
0.080359	0.005717	0.015935	-0.015624	0.014404	0.015882	
0.024757	0.014112	0.020299	-0.009017	0.017128	-0.027632	
0.009651	0.002267	-0.001598	-0.010697	0.017587	-0.018476	-
0.006199	-0.020111	0.014877	-0.005231	-0.013497	0.007860	
0.025628	-0.017175	-0.009555	0.009376	0.001356	0.002753	
0.013911	0.014509					
x150	0.007203	0.002133	-0.037614	-0.027820	0.005339	
0.012641	0.016489	-0.019080	-0.012588	-0.020920	0.003134	-
0.005403	0.010465	0.008496	0.006692	0.025882	-0.019191	
0.011522	0.029896	0.024980	0.023703	0.027640	0.029793	
0.006250	-0.053581	-0.002620	-0.004683	0.001345	0.005856	-
0.002313	-0.042389	0.008209	0.015319	-0.004399	0.008234	-
0.019049	0.012681					
y150	0.013392	-0.025974	0.078629	0.015743	0.004139	-
0.020407	0.019545	-0.015003	0.023216	0.024052	0.005405	
0.003973	-0.050794	0.006850	0.001714	-0.009174	0.004194	-
0.016912	0.036812	-0.000138	-0.031154	-0.019721	-0.008126	-
0.001882	0.001359	0.007090	0.012016	-0.022859	-0.014004	
0.038011	-0.003019	0.008924	0.010391	0.032610	0.032706	
0.023164	0.015369					
z150	-0.027419	-0.012970	-0.081312	0.006028	0.029851	
0.084533	0.008494	0.014788	-0.014334	0.013758	0.017253	
0.018966	0.012945	0.019781	-0.008956	0.020769	-0.031039	
0.013357	0.000927	0.002210	-0.005525	0.016351	-0.016836	-
0.001425	-0.026153	0.014328	-0.006758	-0.009819	-0.000203	

0.016749 -0.023604 -0.018721 0.007911 0.002695 0.009823
0.013221 0.016539
x151 0.009252 0.002654 -0.024947 -0.025901 0.003320
0.008763 0.019943 -0.018259 -0.011243 -0.016066 0.004688 -
0.005756 0.004764 0.003093 0.011633 0.029676 -0.011037
0.016075 0.031186 0.028125 0.019452 0.025032 0.032571
0.008740 -0.053897 -0.002266 -0.004231 -0.002753 0.003213 -
0.004605 -0.037739 0.014720 0.020704 0.004844 0.004240 -
0.021806 0.010914
y151 0.009534 -0.024336 0.082254 0.020038 0.005858 -
0.021655 0.020250 -0.016827 0.021937 0.021638 0.004194
0.008089 -0.054283 0.002849 0.003675 -0.009725 0.007682 -
0.010771 0.037966 -0.008152 -0.027302 -0.021628 -0.007133 -
0.003371 0.001930 0.011460 0.012380 -0.026265 -0.012684
0.041298 -0.003234 0.009505 0.012043 0.032736 0.030422
0.025970 0.020730
z151 -0.026295 -0.009713 -0.085953 0.005357 0.030320
0.086461 0.011684 0.013809 -0.011649 0.013474 0.017980
0.013218 0.011912 0.019120 -0.007801 0.021943 -0.034217
0.015724 0.001278 0.006019 -0.001965 0.015272 -0.014162
0.002924 -0.032838 0.014270 -0.009347 -0.006368 -0.006523
0.009286 -0.028449 -0.023645 0.005278 0.002171 0.014996
0.012613 0.017359
x152 0.011043 0.002271 -0.013956 -0.021756 0.000613
0.005158 0.024551 -0.018626 -0.010826 -0.012282 0.001516 -
0.004347 0.000259 -0.004049 0.013099 0.032306 -0.003075
0.021516 0.035176 0.029658 0.021818 0.022939 0.032034
0.013575 -0.049880 -0.002072 -0.006301 -0.003685 0.001582 -
0.004455 -0.036943 0.023126 0.024087 0.007939 0.000386 -
0.019067 0.009451
y152 0.006010 -0.022808 0.085305 0.023507 0.006482 -
0.023365 0.019989 -0.020635 0.018766 0.020334 0.004298
0.009768 -0.055191 0.000102 0.002355 -0.009134 0.009860 -
0.005092 0.039040 -0.013755 -0.023037 -0.023616 -0.004817 -
0.005320 0.003326 0.018403 0.009606 -0.026519 -0.012027
0.041457 -0.006206 0.005546 0.011368 0.032939 0.027351
0.028451 0.023222
z152 -0.024554 -0.006326 -0.089353 0.004010 0.030571
0.083896 0.014590 0.012795 -0.007189 0.014738 0.020001
0.004902 0.010827 0.020343 -0.006046 0.022834 -0.034984
0.015745 0.001132 0.011060 -0.002139 0.013506 -0.009129
0.004440 -0.038578 0.017000 -0.009386 -0.004778 -0.014832
0.002640 -0.030005 -0.030503 0.005139 0.006248 0.019531
0.006593 0.017313
x153 0.014607 0.002591 0.000460 -0.017854 -0.000362
0.000839 0.025477 -0.016733 -0.008789 -0.007550 0.003220 -
0.002832 -0.002952 -0.012276 0.015671 0.031719 0.006895
0.025781 0.038458 0.027850 0.019400 0.020785 0.033196
0.011632 -0.047102 0.003377 -0.008563 -0.007475 -0.004171 -
0.007154 -0.036124 0.028029 0.026846 0.008422 -0.006611 -
0.016239 0.016358
y153 0.000971 -0.021383 0.087279 0.027569 0.006646 -
0.024233 0.019731 -0.023889 0.012842 0.017893 0.003769
0.010936 -0.055029 -0.002696 -0.000088 -0.007780 0.010132
0.002826 0.037916 -0.020467 -0.015056 -0.022732 -0.004767 -
0.004187 0.005729 0.021060 0.007346 -0.023909 -0.008199
0.040234 -0.011190 0.003744 0.010118 0.029240 0.026604
0.029738 0.023642
z153 -0.024178 -0.003808 -0.093492 0.003132 0.030382
0.078728 0.019223 0.011868 -0.002514 0.015088 0.019237 -

0.002799 0.009978 0.021579 -0.003251 0.023302 -0.034884
0.016047 0.000686 0.014698 -0.000041 0.013004 -0.004152
0.010737 -0.042793 0.016846 -0.006892 -0.004564 -0.022409
0.000023 -0.029261 -0.028539 0.006992 0.009669 0.024219 -
0.000928 0.015094
x154 0.016803 0.001095 0.015163 -0.012291 -0.002570 -
0.007195 0.025801 -0.012086 -0.007391 -0.003129 0.002633 -
0.001744 -0.005439 -0.021253 0.014840 0.032758 0.019603
0.028766 0.037864 0.022090 0.017591 0.020576 0.027622
0.013973 -0.045946 0.006506 -0.011689 -0.010812 -0.007687 -
0.004833 -0.035595 0.035410 0.034019 0.006068 -0.014097 -
0.012768 0.020549
y154 -0.003952 -0.018944 0.089334 0.031457 0.007669 -
0.020803 0.018732 -0.026746 0.005595 0.015408 0.004455
0.013188 -0.052504 -0.005657 -0.003430 -0.007456 0.009063
0.012616 0.034501 -0.025246 -0.005411 -0.019727 -0.004794 -
0.002761 0.006010 0.024668 0.005848 -0.019209 -0.005516
0.034162 -0.016789 0.000292 0.006858 0.024540 0.025495
0.028225 0.022930
z154 -0.023160 -0.000754 -0.096357 0.002382 0.031000
0.072611 0.023849 0.011578 0.003997 0.016515 0.019319 -
0.010632 0.008824 0.023103 0.001521 0.021408 -0.033965
0.015503 0.001828 0.019538 0.000846 0.013376 0.002586
0.015042 -0.046544 0.018434 -0.002249 -0.006628 -0.030271 -
0.002513 -0.024045 -0.024042 0.011342 0.015518 0.028161 -
0.011510 0.014376
x155 0.018543 -0.000859 0.030136 -0.006135 -0.003067 -
0.015522 0.027912 -0.008756 -0.008380 -0.001698 0.002965 -
0.000625 -0.006993 -0.028731 0.012913 0.034551 0.032202
0.031538 0.033585 0.013589 0.016777 0.018439 0.019215
0.013626 -0.042000 0.012528 -0.014606 -0.009194 -0.010985 -
0.000317 -0.034146 0.041395 0.041037 -0.005711 -0.018900 -
0.007991 0.025644
y155 -0.008755 -0.016451 0.090315 0.034653 0.007420 -
0.016650 0.016254 -0.030541 -0.003179 0.014156 0.005015
0.013486 -0.047745 -0.008897 -0.008556 -0.007568 0.007299
0.023198 0.031813 -0.028191 0.006641 -0.014645 -0.002977 -
0.000507 0.007038 0.028625 0.003639 -0.013757 -0.003196
0.024796 -0.027427 -0.003253 0.004104 0.020003 0.022805
0.024173 0.018855
z155 -0.022230 0.002138 -0.098571 0.001660 0.030918
0.062853 0.028144 0.011712 0.011702 0.019020 0.018336 -
0.020122 0.008583 0.025074 0.005856 0.018597 -0.030941
0.013521 0.004810 0.024186 0.001812 0.014275 0.010864
0.018805 -0.048390 0.020920 0.003990 -0.010861 -0.038300 -
0.003001 -0.017892 -0.017930 0.019019 0.021969 0.031869 -
0.025226 0.012761
x156 0.023621 -0.002710 0.045472 0.001234 -0.001499 -
0.022299 0.028295 -0.008144 -0.008047 0.001631 0.002139
0.001741 -0.005499 -0.034952 0.010354 0.030729 0.041550
0.034554 0.029155 0.004231 0.019834 0.017345 0.011534
0.011398 -0.038415 0.019914 -0.018101 -0.011988 -0.013366
0.002981 -0.039013 0.048683 0.044549 -0.017965 -0.018037
0.000628 0.021037
y156 -0.015557 -0.014262 0.090876 0.038676 0.004829 -
0.013048 0.014001 -0.034488 -0.015334 0.010019 0.004243
0.011817 -0.043100 -0.015046 -0.013974 -0.003390 0.004927
0.035034 0.028351 -0.030622 0.017129 -0.008428 -0.002836
0.003026 0.010500 0.032010 0.001136 -0.004758 0.000038

0.014406 -0.040364 -0.007406 0.003269 0.009773 0.018875
0.017665 0.018932
z156 -0.021357 0.004849 -0.099511 0.001009 0.030097
0.050421 0.032746 0.012361 0.019867 0.020755 0.015917 -
0.031019 0.007872 0.026824 0.011674 0.015422 -0.025896
0.011080 0.007750 0.028364 0.001288 0.014667 0.019131
0.021830 -0.048804 0.024976 0.011573 -0.015602 -0.045305 -
0.000946 -0.011712 -0.009742 0.028764 0.026091 0.034824 -
0.043396 0.010169
x157 0.025324 -0.006785 0.060174 0.008508 0.000560 -
0.026664 0.028353 -0.002078 -0.007382 0.009175 0.004070
0.005170 -0.002245 -0.044675 0.010305 0.032867 0.035219
0.033934 0.016323 0.001003 0.023105 0.018230 -0.000401 -
0.006699 -0.028842 0.027803 -0.019099 -0.004388 -0.008199
0.002730 -0.029900 0.041137 0.026698 -0.022249 -0.021832 -
0.000740 0.018853
y157 -0.028511 -0.011993 0.079559 0.036171 0.000746 -
0.004038 0.010376 -0.034722 -0.019864 0.008024 0.004919
0.014847 -0.027956 -0.013184 -0.006401 0.000039 -0.004651
0.032253 0.022775 -0.019222 0.029140 -0.001608 0.000761
0.002175 0.007288 0.031529 0.008353 0.013265 0.007306
0.004181 -0.031502 -0.008627 0.000203 0.003998 0.007596 -
0.000093 0.022320
z157 -0.020817 0.006667 -0.085831 0.007397 0.028534
0.038278 0.038274 0.015995 0.013784 0.028616 0.010339 -
0.032123 -0.001916 0.020300 -0.012338 0.003524 -0.015706
0.019554 0.005339 0.036147 0.003631 0.015901 0.025959
0.027468 -0.038085 0.013770 0.018817 -0.013738 -0.052234
0.002444 -0.022542 0.020528 0.029453 0.033334 0.027259 -
0.034872 -0.007008
x158 0.027658 -0.009837 0.069453 0.013454 0.003979 -
0.026298 0.033555 0.000246 -0.001023 0.012858 0.007911
0.010694 0.003738 -0.051865 0.016813 0.029043 0.033248
0.029489 0.006541 -0.003539 0.026743 0.016269 -0.012271 -
0.027060 -0.016310 0.033652 -0.022284 0.001582 -0.005868
0.000943 -0.021156 0.035664 0.006936 -0.030293 -0.020763 -
0.002944 0.029393
y158 -0.039215 -0.010689 0.067733 0.035344 -0.003004
0.001481 0.003666 -0.032846 -0.025227 0.004674 0.004583
0.013363 -0.018180 -0.011181 -0.001331 0.007771 -0.017790
0.028557 0.016470 -0.009553 0.035404 0.007015 0.003125
0.001748 0.003830 0.029403 0.015648 0.027452 0.014653 -
0.005161 -0.025465 -0.007406 0.002400 -0.003013 -0.000806 -
0.010442 0.022927
z158 -0.020348 0.008152 -0.073459 0.012741 0.026644
0.027845 0.042376 0.018308 0.009390 0.034229 0.006560 -
0.033136 -0.008249 0.013485 -0.028891 -0.005774 -0.006805
0.025549 0.002973 0.042514 0.006777 0.016580 0.029642
0.028023 -0.025813 0.001638 0.025793 -0.011388 -0.058485
0.002505 -0.029256 0.046768 0.030483 0.035807 0.024521 -
0.025394 -0.019039
x159 0.030613 -0.012138 0.077834 0.016627 0.008049 -
0.026538 0.035338 0.007015 0.006185 0.013639 0.014005
0.013878 0.007731 -0.056212 0.021516 0.026683 0.027721
0.020790 -0.001478 -0.002935 0.025455 0.015027 -0.023940 -
0.047210 -0.012319 0.043691 -0.024192 -0.000086 -0.004116
0.004335 -0.019522 0.023430 -0.012297 -0.038258 -0.019063 -
0.006523 0.038736
y159 -0.047924 -0.009239 0.055368 0.034365 -0.007722
0.009795 -0.001338 -0.033488 -0.030155 0.004300 0.000738

0.011217	-0.009037	-0.010311	0.001693	0.011832	-0.025410	
0.027756	0.010212	-0.005935	0.041251	0.010810	0.005890	
0.005567	0.002671	0.024970	0.018228	0.040607	0.019921	-
0.014086	-0.018932	-0.001997	0.007183	-0.005040	-0.009022	-
0.010075	0.015095					
z159	-0.017689	0.008805	-0.059972	0.015739	0.024723	
0.018849	0.044161	0.020666	0.007752	0.036381	0.003213	-
0.032168	-0.011730	0.005978	-0.038092	-0.013491	-0.002379	
0.027763	0.000760	0.047162	0.008612	0.017135	0.030917	
0.025890	-0.018204	-0.000553	0.028528	-0.013808	-0.062983	
0.006684	-0.041193	0.063407	0.028898	0.035031	0.022013	-
0.016701	-0.032168					
x160	0.029170	-0.014004	0.080166	0.019379	0.009291	-
0.024263	0.037076	0.010444	0.012160	0.012810	0.017522	
0.014040	0.012053	-0.058693	0.021784	0.028039	0.023712	
0.013242	-0.011092	-0.005050	0.028796	0.012690	-0.028772	-
0.055473	-0.006983	0.034999	-0.019747	0.000099	0.004730	
0.005189	-0.005735	0.013727	-0.024440	-0.038841	-0.017645	-
0.008651	0.049472					
y160	-0.053013	-0.008097	0.045562	0.032080	-0.009521	
0.015301	-0.003697	-0.032731	-0.035015	0.006038	-0.002492	
0.012583	-0.000186	-0.006336	0.003567	0.010870	-0.029450	
0.025718	0.007970	0.001564	0.042481	0.011724	-0.000595	
0.007815	-0.001049	0.026998	0.016999	0.052061	0.010447	-
0.019023	-0.017479	0.003346	0.009516	-0.008024	-0.013496	-
0.003310	0.007575					
z160	-0.015903	0.009193	-0.048863	0.018291	0.022651	
0.008627	0.048930	0.018807	0.006735	0.035048	0.003417	-
0.030192	-0.012118	-0.001344	-0.043122	-0.021093	0.004125	
0.029972	-0.003341	0.048344	0.010716	0.014976	0.031742	
0.023440	-0.010151	-0.013567	0.033019	-0.013610	-0.054934	
0.007601	-0.038546	0.071760	0.027201	0.033332	0.021351	-
0.013225	-0.031012					
x161	0.024353	-0.014246	0.081928	0.018789	0.009354	-
0.016652	0.036538	0.014225	0.017653	0.015849	0.020404	
0.014415	0.019918	-0.051866	0.014938	0.026428	0.021248	
0.005416	-0.016287	-0.001237	0.032814	0.008496	-0.036292	-
0.054890	-0.007004	0.029620	-0.014535	0.000250	0.004593	
0.004614	0.004961	0.001763	-0.033949	-0.039960	-0.017226	-
0.001796	0.054056					
y161	-0.053276	-0.007764	0.033037	0.031058	-0.009455	
0.017131	-0.006733	-0.032638	-0.039707	0.002766	-0.006161	
0.014287	-0.000147	-0.008358	0.012775	0.014598	-0.032317	
0.018103	0.004642	0.001603	0.037525	0.017342	0.000731	
0.008689	-0.003080	0.028235	0.010336	0.058966	0.010738	-
0.023168	-0.012106	0.008435	0.011621	0.000193	-0.004890	-
0.014278	-0.002381					
z161	-0.013802	0.009688	-0.036134	0.019322	0.022537	
0.000637	0.051670	0.015781	0.009663	0.031723	0.005381	-
0.029319	-0.008912	-0.004282	-0.044465	-0.024643	0.008993	
0.031810	-0.008280	0.049605	0.014442	0.012501	0.028348	
0.021919	0.000692	-0.016917	0.035477	-0.014242	-0.049510	
0.006427	-0.029879	0.068834	0.023684	0.022965	0.015928	-
0.000943	-0.028884					
x162	0.016776	-0.013982	0.076480	0.018919	0.007320	-
0.008224	0.032668	0.017455	0.019602	0.017856	0.016001	
0.010284	0.027181	-0.043850	0.012047	0.027249	0.012485	-
0.006612	-0.021449	0.003768	0.035497	0.006017	-0.042759	-
0.048109	-0.013346	0.015558	-0.004475	-0.001705	0.001818	

0.006888 0.013427 -0.009767 -0.036241 -0.029756 -0.011139
0.002699 0.053105
y162 -0.047724 -0.007619 0.023759 0.026620 -0.007339
0.020754 -0.004874 -0.030718 -0.037772 -0.001792 -0.002067
0.020769 -0.002584 -0.008825 0.015603 0.014750 -0.032265
0.012529 0.005163 0.002931 0.027952 0.017282 -0.001882
0.005722 0.000451 0.034247 -0.002578 0.067070 0.009503 -
0.031750 -0.003605 0.005160 0.008850 0.000983 -0.002907 -
0.014765 -0.008034
z162 -0.011772 0.010372 -0.026419 0.019171 0.022369 -
0.006911 0.054261 0.011716 0.012958 0.028771 0.005361 -
0.030485 -0.005574 -0.006964 -0.036655 -0.026335 0.010150
0.029406 -0.015469 0.051392 0.013977 0.011141 0.024341
0.016252 0.004330 -0.025247 0.032856 -0.015794 -0.040426
0.005438 -0.016093 0.058536 0.016348 0.016792 0.011868
0.008870 -0.020224
x163 0.008038 -0.012559 0.066394 0.017953 0.004874
0.004312 0.027747 0.019709 0.022121 0.017872 0.012258
0.005332 0.036029 -0.035136 0.008746 0.026713 0.001174 -
0.019725 -0.023640 0.010290 0.036631 0.001368 -0.051782 -
0.038086 -0.021551 -0.001021 0.008097 -0.000708 -0.000088
0.006437 0.018155 -0.022183 -0.030286 -0.015587 -0.002486
0.004553 0.044889
y163 -0.039210 -0.009724 0.017479 0.021969 -0.007634
0.024495 -0.005763 -0.024855 -0.034569 -0.001556 0.004189
0.025396 -0.005818 -0.010932 0.014560 0.013849 -0.025946
0.004123 0.002145 -0.001421 0.016488 0.016971 -0.000500
0.001598 0.007562 0.044997 -0.026339 0.064847 0.002575 -
0.034058 0.006551 0.010036 0.001978 -0.009889 -0.000022 -
0.007062 -0.013754
z163 -0.008723 0.011037 -0.018187 0.017822 0.023380 -
0.013867 0.056565 0.004999 0.018546 0.019172 0.006675 -
0.030741 0.000069 -0.010727 -0.021742 -0.028281 0.008584
0.025549 -0.019846 0.050763 0.014517 0.006455 0.016565
0.009006 0.007530 -0.031683 0.032200 -0.015503 -0.025262
0.003848 -0.001745 0.039374 0.015735 0.008493 0.010548
0.014162 -0.008356
x164 -0.003002 -0.010630 0.054185 0.015205 0.000119
0.022306 0.019759 0.024050 0.023820 0.018163 0.010319 -
0.000537 0.047584 -0.023591 0.003006 0.026016 -0.012183 -
0.031135 -0.020448 0.019083 0.037875 -0.006593 -0.061191 -
0.030672 -0.033569 -0.025108 0.023578 -0.002933 -0.001437
0.006177 0.020920 -0.038014 -0.026697 -0.002369 0.003596
0.010039 0.025207
y164 -0.030428 -0.014096 0.015147 0.013502 -0.008470
0.027197 -0.004913 -0.024345 -0.032988 0.001491 0.003068
0.032174 -0.005426 -0.006149 0.012600 0.005475 -0.021037 -
0.006753 -0.002091 -0.003106 0.005389 0.012326 0.000523 -
0.005733 0.005885 0.045624 -0.037335 0.049268 -0.020108 -
0.020575 0.029790 0.019205 0.005894 -0.000429 0.003058
0.028909 -0.007110
z164 -0.002503 0.011513 -0.009499 0.016458 0.025430 -
0.020483 0.058058 -0.000784 0.026626 0.003204 0.012364 -
0.030278 0.006327 -0.017575 -0.000999 -0.028706 0.003875
0.022186 -0.024637 0.045764 0.015688 -0.002311 0.007282
0.001616 0.012143 -0.023992 0.025086 -0.017106 -0.002215
0.002557 0.010387 0.011083 0.007930 -0.009501 0.009942
0.016269 0.006444
x165 -0.016609 -0.008798 0.038547 0.010308 -0.004316
0.042604 0.013158 0.025097 0.027291 0.018621 0.006741 -

0.009100	0.056819	-0.006610	-0.003388	0.023951	-0.019571	-
0.044597	-0.021743	0.030898	0.044489	-0.020857	-0.066584	-
0.016398	-0.048918	-0.051353	0.037371	-0.005290	-0.006985	
0.007620	0.030861	-0.052769	-0.012021	0.015164	0.014724	
0.023268	0.015214					
y165	-0.017196	-0.009906	0.003388	0.013477	0.005141	
0.011974	0.010011	-0.025514	-0.026406	-0.003899	-0.009857	
0.023210	-0.016673	0.002426	0.015581	0.005690	-0.006351	-
0.012445	0.005704	-0.017345	-0.022914	0.016542	-0.002779	
0.018821	0.004947	0.045014	-0.052369	0.052027	-0.026826	-
0.013406	0.029508	0.042261	0.012822	-0.008492	0.006260	
0.045327	0.012731					
z165	0.002727	0.009908	0.002828	0.013677	0.019919	-
0.019892	0.054996	-0.008041	0.032733	-0.009649	0.022235	-
0.020689	0.022156	-0.025214	0.019970	-0.029057	-0.006421	
0.015092	-0.029883	0.047065	0.017211	-0.010357	-0.005924	-
0.020843	0.009923	-0.018194	0.013840	-0.024059	0.019589	
0.005514	0.034914	-0.023915	-0.002754	-0.017933	0.015172	
0.017727	0.007747					
x166	-0.006254	-0.006929	-0.016674	0.005036	0.019720	
0.012254	0.015867	-0.020598	-0.011183	0.009162	0.004321	
0.003580	-0.017269	-0.004412	-0.012011	-0.010734	0.015956	-
0.010674	0.005148	-0.022990	0.008147	-0.017253	-0.004067	
0.012294	0.009550	0.012729	-0.005135	0.005540	0.011729	
0.018922	0.014335	-0.012505	0.017463	-0.002265	0.004949	
0.021152	-0.005174					
y166	0.016138	0.000892	-0.013266	0.001533	-0.005213	
0.015489	0.023644	0.010044	0.024944	-0.002608	-0.038955	-
0.019852	-0.006535	-0.011803	-0.015960	0.008301	0.009255	
0.029335	-0.003915	-0.033722	-0.003838	0.033817	0.021689	-
0.020928	0.038569	0.006756	-0.006629	-0.008775	-0.023122	-
0.009135	0.006037	-0.020080	0.002689	-0.005717	0.009281	
0.001746	-0.025904					
z166	-0.005212	-0.007478	0.013606	-0.002059	-0.039305	
0.012505	-0.012027	-0.006265	-0.002048	-0.006843	-0.009759	-
0.047496	-0.019943	0.014860	-0.012358	-0.005198	0.007597	-
0.000452	-0.017009	-0.020847	0.011977	0.002702	0.011279	-
0.016823	-0.006422	-0.000118	-0.016164	0.015520	-0.002771	-
0.013005	0.009034	-0.003487	0.019867	-0.005373	0.009757	-
0.005400	0.014970					
x167	-0.002968	-0.010916	-0.013840	0.002754	0.018927	
0.012423	0.017506	-0.022449	-0.012706	0.007380	0.004530	
0.001005	-0.019491	-0.006029	-0.012152	-0.013450	0.012033	-
0.011739	0.003022	-0.018844	0.009847	-0.015161	0.006885	
0.009602	0.010197	0.012138	-0.002203	0.006729	0.001898	
0.013531	0.011448	-0.028130	0.008180	-0.017254	0.002535	
0.029268	-0.002146					
y167	0.018942	0.001163	-0.010219	0.000254	-0.006271	
0.015311	0.019490	0.012657	0.021348	-0.005245	-0.039786	-
0.019658	-0.006290	-0.013279	-0.015886	0.009021	0.006426	
0.034004	-0.010646	-0.027929	-0.002502	0.038586	0.031107	-
0.018156	0.031716	0.003938	-0.013459	-0.013430	-0.024670	-
0.011574	0.015383	-0.020074	0.007620	-0.014217	0.004797	
0.012474	-0.027256					
z167	-0.012871	-0.009135	0.011540	-0.003707	-0.050913	
0.009594	-0.020111	-0.004992	-0.004525	-0.000753	-0.012217	-
0.042744	-0.016363	0.015477	-0.016051	-0.009696	0.004269	-
0.008953	-0.015345	-0.017560	0.004170	-0.003624	-0.000207	-
0.017759	-0.001044	0.000053	-0.009168	0.020738	0.000745	-

0.011513 0.010848 0.005198 0.028768 0.019225 0.004158 -
0.006500 0.013997
x168 0.001064 -0.013270 -0.012637 0.003651 0.016790
0.012668 0.015050 -0.024490 -0.014420 0.002493 0.002522 -
0.002181 -0.021478 -0.010794 -0.007946 -0.010733 0.007031 -
0.005004 0.000952 -0.015894 0.006149 -0.013595 0.018993
0.013149 0.005959 0.007911 0.000166 -0.006065 -0.005456
0.009611 0.015230 -0.027948 0.010476 -0.030803 -0.001493
0.043522 -0.009804
y168 0.019502 0.000862 -0.007827 -0.002824 -0.008129
0.014959 0.014407 0.015227 0.016038 -0.005326 -0.039827 -
0.018024 -0.003468 -0.014613 -0.018716 0.006796 0.003263
0.035008 -0.016936 -0.022957 -0.001424 0.043079 0.036480 -
0.016895 0.026563 0.000670 -0.016954 -0.013821 -0.021231 -
0.012253 0.019925 -0.016343 0.008728 -0.019238 -0.005090
0.010971 -0.016681
z168 -0.020179 -0.010797 0.010935 -0.007361 -0.057151
0.007939 -0.024419 -0.002776 -0.005863 0.007600 -0.009691 -
0.038197 -0.011843 0.019663 -0.019540 -0.017663 0.005159 -
0.020503 -0.016531 -0.015283 -0.000562 -0.007404 -0.012067 -
0.019285 -0.001520 0.000607 -0.003767 0.026791 0.005707 -
0.006941 0.008568 0.010049 0.033007 0.035955 0.002407 -
0.015779 0.019896
x169 0.003969 -0.015424 -0.010290 0.002729 0.015603
0.013607 0.013897 -0.025356 -0.015250 0.000181 0.001697 -
0.002823 -0.021588 -0.013394 -0.008044 -0.011186 0.002447 -
0.000364 -0.001808 -0.012910 0.003071 -0.009714 0.025945
0.013939 0.004463 0.004655 0.002844 -0.013639 -0.009729
0.003898 0.019438 -0.029886 0.009989 -0.039496 -0.002994
0.047291 -0.009304
y169 0.020775 0.001482 -0.005919 -0.005149 -0.011897
0.014722 0.008938 0.017353 0.012625 -0.006942 -0.040111 -
0.020000 0.000535 -0.016214 -0.016818 0.004103 0.000014
0.032946 -0.022913 -0.018603 0.000129 0.041427 0.040875 -
0.015344 0.019178 -0.000683 -0.022741 -0.008546 -0.020949 -
0.012135 0.021232 -0.010738 0.009835 -0.023200 -0.011145
0.016830 -0.018662
z169 -0.025578 -0.012414 0.010138 -0.008091 -0.062927
0.005790 -0.029953 -0.000887 -0.006002 0.011183 -0.008476 -
0.035790 -0.007946 0.021094 -0.018901 -0.021029 0.004678 -
0.029866 -0.019085 -0.014872 -0.004597 -0.013940 -0.021747 -
0.018380 -0.003098 0.000816 -0.000529 0.032765 0.009175 -
0.002933 0.006927 0.015994 0.035295 0.041885 -0.001870 -
0.018619 0.018715
x170 0.006317 -0.016449 -0.007247 -0.000182 0.013149
0.017165 0.012010 -0.025143 -0.015237 0.000915 0.002577 -
0.002672 -0.019294 -0.015844 -0.009135 -0.014839 0.000754
0.001319 -0.003736 -0.009157 0.000294 -0.008402 0.033039
0.011536 0.000243 0.004229 0.002777 -0.014604 -0.012389 -
0.000989 0.020688 -0.027104 0.008872 -0.044695 -0.005412
0.049693 -0.010226
y170 0.021297 0.000512 -0.006582 -0.006952 -0.013575
0.010248 0.007031 0.015762 0.007497 -0.009514 -0.039226 -
0.023475 0.003062 -0.014710 -0.014391 0.003870 -0.004155
0.034641 -0.025833 -0.014917 -0.000427 0.041833 0.039569 -
0.015539 0.015016 -0.005653 -0.019405 -0.013057 -0.023071 -
0.014840 0.029140 -0.011356 0.015636 -0.021044 -0.011895
0.018721 -0.015068
z170 -0.030603 -0.015864 0.006870 -0.007131 -0.065611
0.000812 -0.033463 -0.000008 -0.007770 0.011847 -0.008583 -

0.032800	-0.007203	0.021479	-0.019107	-0.019380	0.002369	-
0.030806	-0.019856	-0.013868	-0.008468	-0.013055	-0.029823	-
0.019371	-0.005226	0.000439	0.009129	0.025899	0.011047	
0.001462	0.007202	0.015543	0.036642	0.045409	-0.004377	-
0.020281	0.023053					
x171	0.007669	-0.017503	-0.004936	-0.002103	0.009202	
0.017625	0.009620	-0.025851	-0.016098	-0.000009	0.001264	-
0.004385	-0.017591	-0.018011	-0.007686	-0.014910	-0.004334	
0.002511	-0.009155	-0.008432	-0.002878	-0.007097	0.036035	
0.008161	0.000073	0.000933	0.004913	-0.018139	-0.015730	-
0.006493	0.022994	-0.021418	0.009707	-0.044428	-0.005123	
0.049840	-0.012415					
y171	0.022134	0.001325	-0.005362	-0.009921	-0.017379	
0.010136	0.003514	0.014142	0.003197	-0.009381	-0.039454	-
0.029299	0.002767	-0.013521	-0.008549	0.002160	-0.006731	
0.032961	-0.028181	-0.009068	-0.001953	0.041073	0.037799	-
0.015185	0.008419	-0.001363	-0.023378	-0.009515	-0.022338	-
0.015367	0.027077	-0.012623	0.017278	-0.016237	-0.016490	
0.015182	-0.016850					
z171	-0.033935	-0.017999	0.005513	-0.007177	-0.066311	-
0.001692	-0.035962	-0.000147	-0.008008	0.013372	-0.004979	-
0.028721	-0.004834	0.019722	-0.020685	-0.019337	0.003969	-
0.029909	-0.020376	-0.011118	-0.011119	-0.012445	-0.032408	-
0.018672	-0.008567	0.002167	0.013790	0.023601	0.013226	
0.004969	0.005646	0.015235	0.035106	0.041414	-0.007906	-
0.022393	0.023104					
x172	0.008421	-0.018304	-0.002988	-0.004071	0.003719	
0.016953	0.006049	-0.027051	-0.017321	-0.000340	-0.001715	-
0.007504	-0.016932	-0.020175	-0.004709	-0.012715	-0.012636	
0.001419	-0.016607	-0.008108	-0.007957	-0.006277	0.036653	
0.002797	0.002390	-0.000883	0.005756	-0.020444	-0.017033	-
0.012818	0.024458	-0.015426	0.008791	-0.038013	-0.004223	
0.047627	-0.015242					
y172	0.022778	0.001975	-0.003835	-0.012551	-0.020380	
0.009358	0.001920	0.010162	-0.002535	-0.008557	-0.036796	-
0.033635	0.011044	-0.013776	-0.008680	-0.004578	-0.010605	
0.033929	-0.028066	-0.005948	0.001902	0.038298	0.033841	-
0.017129	0.005878	-0.003741	-0.018958	-0.005913	-0.023837	-
0.013707	0.029873	-0.006781	0.018473	-0.008967	-0.020466	
0.009933	-0.019085					
z172	-0.035545	-0.020321	0.004273	-0.007570	-0.064286	-
0.004072	-0.037215	-0.001650	-0.007731	0.014414	0.000815	-
0.024015	-0.000928	0.016988	-0.024060	-0.019096	0.006130	-
0.024926	-0.020447	-0.007852	-0.012161	-0.010198	-0.031542	-
0.018433	-0.013214	0.003529	0.017818	0.019675	0.013649	
0.007566	0.006401	0.011568	0.028295	0.031541	-0.011487	-
0.021805	0.022732					
x173	0.008798	-0.018974	-0.002064	-0.006235	-0.002701	
0.015283	0.001345	-0.028178	-0.018897	-0.000298	-0.006672	-
0.012608	-0.015138	-0.022639	-0.001251	-0.009498	-0.022209	-
0.001161	-0.024104	-0.008817	-0.014889	-0.005851	0.034685	-
0.002655	0.005855	-0.003819	0.006662	-0.022343	-0.016638	-
0.019447	0.025134	-0.005354	0.008107	-0.028052	-0.002962	
0.041048	-0.017768					
y173	0.021821	0.003575	-0.003764	-0.015933	-0.024137	
0.008217	0.000844	0.008881	-0.004953	-0.007888	-0.032892	-
0.035995	0.019657	-0.013987	-0.008617	-0.013098	-0.010849	
0.034927	-0.024965	-0.004065	0.002660	0.035885	0.028060	-
0.015485	0.004347	-0.003796	-0.020974	0.000673	-0.023892	-

0.017360	0.032037	-0.002201	0.021010	0.003449	-0.019052	
0.004151	-0.021470					
z173	-0.035861	-0.022789	0.002166	-0.007807	-0.060012	-
0.006698	-0.037469	-0.003051	-0.006062	0.014132	0.008129	-
0.018783	0.002815	0.012376	-0.028467	-0.017932	0.010018	-
0.016232	-0.018776	-0.003393	-0.014413	-0.006780	-0.026384	-
0.016574	-0.018458	0.004963	0.018816	0.013780	0.014437	
0.008057	0.006688	0.006406	0.020257	0.014661	-0.014407	-
0.019778	0.022362					
x174	0.008757	-0.018324	-0.000159	-0.010897	-0.008898	
0.015514	-0.001907	-0.029019	-0.018890	0.002456	-0.008615	-
0.015579	-0.011293	-0.023074	0.000062	-0.009518	-0.027827	-
0.005009	-0.030580	-0.008062	-0.022404	-0.007174	0.032061	-
0.010390	0.007720	-0.005249	0.005473	-0.019962	-0.016064	-
0.026790	0.026868	0.006121	0.008863	-0.013104	0.003268	
0.031482	-0.021965					
y174	0.022485	0.003634	-0.006135	-0.016328	-0.027831	
0.002783	-0.001624	0.005252	-0.010465	-0.008755	-0.030447	-
0.040206	0.027863	-0.014989	-0.007445	-0.019686	-0.013841	
0.035471	-0.026105	-0.003536	0.001765	0.031387	0.021210	-
0.017554	0.000542	-0.003518	-0.016813	0.007441	-0.023000	-
0.018021	0.027955	0.007746	0.020998	0.015157	-0.024851	-
0.000384	-0.018269					
z174	-0.034998	-0.025830	-0.000853	-0.005892	-0.053470	-
0.011164	-0.037254	-0.004680	-0.004471	0.010553	0.013685	-
0.014437	0.004841	0.006040	-0.032445	-0.012768	0.012254	-
0.004028	-0.018556	0.000417	-0.014315	0.000093	-0.019817	-
0.014563	-0.024196	0.005092	0.021238	0.004610	0.014278	
0.008981	0.005555	-0.001911	0.007967	-0.009709	-0.017668	-
0.016026	0.023045					
x175	0.008049	-0.017992	0.001085	-0.013845	-0.017225	
0.013554	-0.006977	-0.029309	-0.020548	0.003754	-0.014247	-
0.020113	-0.007026	-0.024945	0.002480	-0.007044	-0.037129	-
0.008191	-0.039851	-0.009372	-0.030804	-0.007557	0.024814	-
0.018003	0.011839	-0.007921	0.006016	-0.020494	-0.013169	-
0.034390	0.027487	0.022817	0.007769	0.010407	0.007486	
0.019532	-0.027140					
y175	0.021703	0.003954	-0.009608	-0.019508	-0.029954	
0.000946	-0.003418	-0.001836	-0.011357	-0.007442	-0.025803	-
0.046845	0.034994	-0.013482	-0.003962	-0.026082	-0.013241	
0.031693	-0.020506	-0.003821	0.003903	0.028932	0.017751	-
0.022274	-0.003272	-0.004008	-0.018763	0.016679	-0.023672	-
0.021860	0.028317	0.009246	0.022142	0.021055	-0.025429	-
0.009697	-0.012119					
z175	-0.032724	-0.029053	-0.003199	-0.004886	-0.043972	-
0.014264	-0.034943	-0.006915	-0.002096	0.007235	0.022335	-
0.008491	0.008269	-0.001471	-0.038228	-0.007922	0.017675	
0.012138	-0.016709	0.005224	-0.013289	0.008477	-0.008618	-
0.010925	-0.032900	0.005786	0.022061	-0.006876	0.013696	
0.010409	0.005979	-0.012385	-0.008179	-0.038995	-0.021034	-
0.010596	0.022405					
x176	0.009620	-0.017227	0.003551	-0.017496	-0.016611	
0.016757	-0.008563	-0.034109	-0.018888	0.006930	-0.014417	-
0.016842	-0.006725	-0.025431	0.004668	-0.006591	-0.035780	-
0.010961	-0.032020	-0.001676	-0.031620	-0.010022	0.031027	-
0.010632	0.010123	-0.007200	0.004688	-0.023712	-0.012662	-
0.027268	0.032785	0.019924	0.004663	0.001709	0.014602	
0.001299	-0.016933					
y176	0.020068	0.004802	-0.013334	-0.020415	-0.031205	-
0.004575	-0.007641	-0.003631	-0.012832	-0.007286	-0.020174	-

0.048620	0.037574	-0.014770	-0.003045	-0.027670	-0.016557	
0.031111	-0.022112	-0.003184	0.002772	0.030117	0.015584	-
0.019336	-0.004788	-0.009172	-0.018890	0.018157	-0.017430	-
0.015609	0.033178	0.013337	0.019287	0.024112	-0.021962	-
0.012722	-0.004456					
z176	-0.036420	-0.030187	-0.003833	-0.002743	-0.042929	-
0.019057	-0.032921	-0.005996	0.007379	0.003729	0.023883	-
0.003123	0.005024	-0.004960	-0.031476	-0.008866	0.020826	
0.015163	-0.018881	-0.003038	-0.018083	0.012354	-0.009652	-
0.011971	-0.037717	0.010221	0.015924	-0.009779	0.015353	-
0.000897	0.001968	-0.000418	-0.004863	-0.041377	-0.031602	-
0.007307	0.011843					
x177	0.010799	-0.016850	0.004897	-0.019566	-0.016372	
0.017735	-0.010268	-0.037924	-0.017845	0.008814	-0.015666	-
0.014246	-0.007824	-0.027102	0.007818	-0.003978	-0.038140	-
0.013487	-0.027903	0.003916	-0.034170	-0.011601	0.033854	-
0.004552	0.009183	-0.008988	0.003484	-0.027088	-0.010967	-
0.021744	0.037321	0.016952	-0.002111	-0.005277	0.019187	-
0.012562	-0.008966					
y177	0.016197	0.006492	-0.016178	-0.022494	-0.031887	-
0.009888	-0.007266	-0.005788	-0.011919	-0.007763	-0.016836	-
0.050655	0.040328	-0.015341	-0.003875	-0.032055	-0.013308	
0.026114	-0.020869	-0.004283	0.005485	0.028774	0.013946	-
0.016158	-0.012884	-0.009746	-0.019120	0.020161	-0.017356	-
0.011344	0.032571	0.017487	0.018260	0.026557	-0.023722	-
0.018904	0.000435					
z177	-0.039242	-0.031101	-0.004251	-0.000106	-0.040878	-
0.022387	-0.031185	-0.005328	0.015061	0.001683	0.026179	
0.002804	0.001191	-0.008029	-0.025408	-0.010566	0.024038	
0.017561	-0.021062	-0.009463	-0.021925	0.014272	-0.008919	-
0.014250	-0.041531	0.012876	0.011270	-0.010902	0.015773	-
0.011098	-0.001469	0.009318	-0.003367	-0.042299	-0.039517	-
0.002286	0.002149					
x178	0.007816	-0.013303	0.011766	-0.021974	-0.022282	
0.017106	-0.009620	-0.048447	-0.018394	0.010081	-0.010294	-
0.005362	-0.014484	-0.022913	0.021301	0.001619	-0.050204	-
0.014404	-0.044934	0.006044	-0.020219	-0.018898	0.038552	-
0.021821	0.011594	0.000319	-0.000096	-0.029313	-0.018213	-
0.009117	0.041064	0.014579	-0.000515	-0.001859	0.015885	-
0.027002	0.002784					
y178	0.057540	-0.032442	-0.108690	-0.007780	0.049720	-
0.013936	-0.042060	0.098175	0.013748	-0.014775	-0.115604	-
0.141562	0.108313	-0.096997	-0.151904	-0.069148	0.121471	-
0.001862	0.261062	0.024496	-0.212696	0.120523	-0.034693	
0.317684	-0.080974	-0.160239	0.032789	0.021889	0.105581	-
0.121223	0.011348	0.013649	-0.105425	-0.097789	0.044566	
0.025265	-0.063643					
z178	-0.047522	-0.026932	0.007858	0.000691	-0.049255	-
0.025169	-0.025320	-0.017744	0.017759	0.000589	0.041149	
0.019958	-0.012064	0.001096	-0.000718	-0.005231	0.008483	
0.022409	-0.059852	-0.018585	0.005189	0.003009	-0.000675	-
0.059636	-0.037252	0.036296	0.001155	-0.012499	-0.001666	-
0.003163	-0.002392	0.015967	0.013961	-0.023730	-0.053918	-
0.003090	0.001460					
x179	0.010647	-0.015654	0.005719	-0.022871	-0.017287	
0.016664	-0.013599	-0.042589	-0.014348	0.010387	-0.019194	-
0.009277	-0.010886	-0.029509	0.013411	-0.000097	-0.041885	-
0.017840	-0.022284	0.011521	-0.038014	-0.014181	0.037312	
0.006922	0.003635	-0.010600	0.003803	-0.030504	-0.008798	-

0.011010	0.038706	0.016598	-0.011180	-0.019051	0.021358	-
0.031777	0.002467					
y179	0.007765	0.008785	-0.022893	-0.023620	-0.033462	-
0.021076	-0.010436	-0.010495	-0.007798	-0.008061	-0.004043	-
0.047859	0.042211	-0.015452	-0.003498	-0.040393	-0.008134	
0.019483	-0.023274	-0.008076	0.008884	0.019390	0.008668	-
0.011605	-0.028418	-0.003055	-0.017915	0.025952	-0.015312	-
0.006002	0.027817	0.028819	0.011549	0.035508	-0.033180	-
0.011296	0.001446					
z179	-0.043376	-0.034210	-0.003550	0.005574	-0.035503	-
0.026794	-0.028312	-0.002452	0.024983	-0.002144	0.027839	
0.012990	-0.006997	-0.010982	-0.015181	-0.008380	0.027102	
0.021184	-0.022897	-0.018331	-0.024745	0.015315	-0.005441	-
0.017485	-0.046711	0.016739	0.004513	-0.015328	0.012376	-
0.023492	-0.007572	0.013830	-0.000245	-0.040376	-0.045021	
0.007254	-0.013612					
x180	0.009935	-0.015024	0.005622	-0.025182	-0.017791	
0.015681	-0.014936	-0.044314	-0.011338	0.011571	-0.019806	-
0.005700	-0.012333	-0.030205	0.015644	-0.000566	-0.043081	-
0.018462	-0.020884	0.015598	-0.040982	-0.013885	0.039407	
0.010371	-0.000466	-0.011042	0.004501	-0.030890	-0.007806	-
0.007205	0.037750	0.017362	-0.015200	-0.021760	0.020308	-
0.035901	0.007130					
y180	0.003079	0.008413	-0.027509	-0.022610	-0.034046	-
0.027656	-0.012324	-0.013010	-0.004394	-0.010615	0.001167	-
0.045528	0.039763	-0.013732	-0.002159	-0.044555	-0.003448	
0.016975	-0.021845	-0.011884	0.011925	0.017706	0.006330	-
0.012344	-0.032210	-0.003456	-0.011215	0.028683	-0.015149	-
0.007226	0.022775	0.030182	0.014138	0.038710	-0.027934	-
0.012334	0.003428					
z180	-0.045115	-0.036030	-0.002301	0.008794	-0.032240	-
0.027460	-0.027224	-0.000439	0.027239	-0.003628	0.028926	
0.017919	-0.010503	-0.011581	-0.012091	-0.005241	0.027880	
0.022183	-0.022750	-0.019808	-0.023056	0.012572	-0.002903	-
0.018368	-0.046905	0.016972	0.001869	-0.017136	0.008702	-
0.026751	-0.007630	0.009951	0.001597	-0.037381	-0.044244	
0.014436	-0.018638					
x181	0.008441	-0.014295	0.005090	-0.027997	-0.018986	
0.014502	-0.015654	-0.044263	-0.008276	0.012469	-0.019636	-
0.002243	-0.013762	-0.031147	0.016983	-0.001121	-0.043392	-
0.019733	-0.020839	0.019091	-0.044980	-0.014868	0.040028	
0.013291	-0.006278	-0.010329	0.004074	-0.029198	-0.006606	-
0.004470	0.033907	0.021319	-0.016528	-0.025665	0.016724	-
0.036751	0.010644					
y181	-0.002028	0.007648	-0.030184	-0.021212	-0.035407	-
0.033451	-0.013462	-0.013404	-0.001898	-0.014426	0.005862	-
0.043549	0.035103	-0.011902	-0.000074	-0.044985	0.000665	
0.014622	-0.019237	-0.014826	0.015471	0.012942	0.000634	-
0.010722	-0.036930	-0.003217	-0.006272	0.028614	-0.016953	-
0.006799	0.017471	0.031167	0.014855	0.040680	-0.028761	-
0.006713	0.003819					
z181	-0.046206	-0.038384	-0.001374	0.012592	-0.028209	-
0.027666	-0.027133	0.000847	0.028282	-0.004201	0.029776	
0.024000	-0.013858	-0.011165	-0.009594	-0.002702	0.028100	
0.023018	-0.023203	-0.020174	-0.019980	0.008925	0.001598	-
0.020075	-0.045999	0.017682	0.000991	-0.020070	0.003770	-
0.028605	-0.008377	0.000880	0.003122	-0.031748	-0.037466	
0.021976	-0.024967					
x182	0.006475	-0.013911	0.003169	-0.030412	-0.019370	
0.012889	-0.015810	-0.045266	-0.004585	0.012547	-0.018970	

0.002776	-0.016249	-0.031253	0.017224	-0.002064	-0.043998	-
0.018294	-0.020921	0.023303	-0.048173	-0.013479	0.040720	
0.013451	-0.014688	-0.007205	0.006040	-0.032845	-0.005559	-
0.002129	0.029697	0.024315	-0.016029	-0.026885	0.013432	-
0.032951	0.010883					
y182	-0.007348	0.005438	-0.034874	-0.017458	-0.035228	-
0.039681	-0.014840	-0.016293	0.001244	-0.018343	0.011356	-
0.038482	0.029764	-0.008741	0.000970	-0.044525	0.001648	
0.014014	-0.017627	-0.018529	0.021214	0.009635	-0.005081	-
0.014126	-0.042859	0.001430	0.000305	0.022831	-0.017132	-
0.007904	0.014255	0.024822	0.019165	0.044716	-0.022254	
0.002330	-0.003019					
z182	-0.046925	-0.040517	0.001481	0.016317	-0.024405	-
0.026678	-0.027947	0.003754	0.028081	-0.004894	0.030299	
0.027950	-0.018254	-0.009762	-0.007677	0.001679	0.029110	
0.020406	-0.023762	-0.019559	-0.016177	0.001148	0.006505	-
0.018573	-0.041737	0.015238	0.000658	-0.018072	-0.002893	-
0.029787	-0.009708	-0.008581	0.003439	-0.026317	-0.032341	
0.028064	-0.028276					
x183	0.003969	-0.012759	0.001510	-0.032584	-0.020606	
0.010937	-0.017577	-0.043638	0.000573	0.012355	-0.016279	
0.006211	-0.018629	-0.032166	0.017571	-0.003747	-0.043374	-
0.018852	-0.021678	0.025941	-0.052950	-0.013924	0.043016	
0.014950	-0.020412	-0.006447	0.005797	-0.028688	-0.004729	-
0.000944	0.026338	0.031333	-0.013388	-0.028292	0.007846	-
0.026262	0.011161					
y183	-0.013961	0.002903	-0.038633	-0.011916	-0.035427	-
0.046828	-0.018694	-0.016602	0.004243	-0.023986	0.017421	-
0.034702	0.021717	-0.005882	0.003581	-0.040316	0.005220	
0.012243	-0.015309	-0.024438	0.027007	0.002917	-0.011076	-
0.011241	-0.041008	-0.002226	0.005749	0.022786	-0.017851	-
0.012678	0.015274	0.016963	0.021327	0.049046	-0.015489	
0.013482	-0.009413					
z183	-0.047043	-0.043498	0.003690	0.021017	-0.019308	-
0.025390	-0.028801	0.005381	0.025980	-0.006526	0.030510	
0.032554	-0.023369	-0.007798	-0.006518	0.007191	0.027583	
0.020248	-0.023077	-0.017329	-0.009503	-0.004221	0.012612	-
0.021091	-0.037439	0.017086	0.004843	-0.020652	-0.010716	-
0.028561	-0.008894	-0.023905	0.004625	-0.016681	-0.023165	
0.033652	-0.028360					
x184	0.000896	-0.012443	0.000071	-0.033193	-0.021594	
0.007293	-0.018955	-0.042140	0.005316	0.010743	-0.016398	
0.008820	-0.021118	-0.033273	0.018671	-0.005579	-0.044292	-
0.021773	-0.025101	0.024418	-0.055355	-0.012685	0.041152	
0.016179	-0.028605	-0.004238	0.006372	-0.025212	-0.004495	
0.001383	0.017743	0.037995	-0.014650	-0.027225	-0.000738	-
0.017618	0.005877					
y184	-0.020275	0.000500	-0.039632	-0.007051	-0.034259	-
0.052007	-0.021033	-0.017556	0.009370	-0.027449	0.025810	-
0.026869	0.015702	-0.001700	0.002305	-0.040595	0.015118	
0.008749	-0.011277	-0.029668	0.037532	-0.010232	-0.017301	-
0.008612	-0.041340	0.003999	0.011113	0.020778	-0.022400	-
0.017430	0.010769	0.002369	0.028740	0.058756	-0.011455	
0.027933	-0.024475					
z184	-0.046840	-0.046473	0.004509	0.023888	-0.013847	-
0.022314	-0.028111	0.006829	0.022064	-0.005637	0.031583	
0.037661	-0.029234	-0.003902	-0.005286	0.011850	0.027343	
0.021148	-0.021214	-0.011889	-0.003914	-0.009287	0.020952	-
0.025481	-0.035586	0.013422	0.008521	-0.022516	-0.020327	-

0.024138 -0.005340 -0.042635 0.005173 -0.010437 -0.010875
0.039667 -0.023694
x185 -0.006419 -0.013926 -0.000041 -0.028454 -0.023705 -
0.001001 -0.021233 -0.038338 0.009091 0.007203 -0.008310
0.012022 -0.025359 -0.034098 0.019400 -0.009815 -0.041127 -
0.015107 -0.019906 0.022585 -0.046317 -0.011465 0.036881
0.013957 -0.026224 0.000634 0.009544 -0.022738 -0.005345 -
0.001146 0.013325 0.035910 -0.015928 -0.020897 0.003256 -
0.005244 0.005698
y185 -0.022406 -0.003459 -0.045058 -0.006397 -0.032397 -
0.051758 -0.022531 -0.018637 0.011779 -0.028025 0.027847 -
0.017258 0.012196 -0.000571 0.004006 -0.033227 0.012759
0.002202 -0.004278 -0.024946 0.036151 -0.014233 -0.014618 -
0.009109 -0.039297 -0.004421 0.010516 0.011138 -0.021985 -
0.017004 0.008065 0.007801 0.027644 0.054916 -0.006793
0.026382 -0.007190
z185 -0.042677 -0.047360 0.007321 0.023057 -0.007648 -
0.013717 -0.021913 0.005014 0.014714 -0.003553 0.026867
0.039131 -0.019889 -0.001431 -0.003606 0.019902 0.023723
0.018994 -0.016966 -0.005321 -0.001188 -0.012861 0.027739 -
0.010515 -0.022533 0.000431 -0.001005 -0.008670 -0.019809 -
0.021111 0.002768 -0.023974 0.001171 -0.020598 -0.007011
0.029585 -0.008726
x186 -0.013104 -0.015429 -0.000163 -0.024383 -0.024422 -
0.009358 -0.022315 -0.035420 0.012347 0.003643 -0.001442
0.014533 -0.026785 -0.034640 0.019447 -0.012955 -0.036214 -
0.010846 -0.014034 0.019248 -0.038478 -0.010717 0.031410
0.014258 -0.022070 -0.001985 0.011150 -0.016492 -0.005350 -
0.004128 0.009883 0.035301 -0.017539 -0.017659 0.009445
0.002843 0.002429
y186 -0.024081 -0.006203 -0.047683 -0.007860 -0.029173 -
0.050123 -0.022833 -0.019200 0.014280 -0.026843 0.030822 -
0.008256 0.011590 0.000431 0.001039 -0.030192 0.017580 -
0.001709 0.003786 -0.018686 0.033719 -0.021098 -0.013617 -
0.004481 -0.036580 -0.014123 0.005624 0.005441 -0.019520 -
0.018707 0.009903 0.013404 0.026337 0.053825 -0.001742
0.027540 0.001614
z186 -0.037630 -0.047975 0.008171 0.022706 -0.002363 -
0.005191 -0.015560 0.002789 0.006464 -0.001234 0.021228
0.039792 -0.013498 0.001680 -0.001120 0.027106 0.016183
0.017808 -0.014043 0.001108 0.002078 -0.012697 0.034736 -
0.004173 -0.014307 -0.004831 -0.006711 -0.000728 -0.020690 -
0.015404 0.007490 -0.013006 -0.003450 -0.031418 -0.006773
0.020711 0.008912
x187 -0.020033 -0.017295 -0.001420 -0.019998 -0.024943 -
0.017555 -0.021962 -0.034225 0.014959 0.000124 0.006494
0.017986 -0.027328 -0.034159 0.020622 -0.012419 -0.031093 -
0.007896 -0.008105 0.013221 -0.029646 -0.009717 0.026407
0.014210 -0.014601 -0.008092 0.012445 -0.007072 -0.005098 -
0.006245 0.011060 0.033118 -0.017581 -0.014248 0.012448
0.009059 0.002761
y187 -0.025874 -0.010278 -0.051688 -0.006459 -0.027770 -
0.049898 -0.024050 -0.018833 0.015160 -0.027864 0.030186 -
0.001766 0.009783 0.000495 0.002349 -0.021844 0.014927 -
0.004935 0.009057 -0.013221 0.032385 -0.024701 -0.012269 -
0.001266 -0.032267 -0.020262 0.002912 -0.002191 -0.017137 -
0.018622 0.010419 0.018779 0.023363 0.047852 -0.002018
0.030852 0.009952
z187 -0.027560 -0.047067 0.012257 0.019972 0.001973
0.006810 -0.012565 0.006741 0.001816 0.004825 0.011008

0.038638	-0.011000	0.006799	-0.000153	0.031117	0.003752	
0.014859	-0.011645	0.010978	0.001668	-0.012325	0.044832	-
0.000801	-0.023074	0.010566	-0.021352	-0.001472	-0.022407	-
0.000302	-0.000388	-0.012154	-0.017990	-0.026635	-0.005810	
0.019512	0.006202					
x188	-0.024767	-0.019741	-0.002655	-0.017128	-0.024896	-
0.023259	-0.020981	-0.031528	0.017628	-0.002191	0.010297	
0.021322	-0.028288	-0.033401	0.021648	-0.012163	-0.027096	-
0.007719	-0.000183	0.009622	-0.025178	-0.007437	0.024119	
0.014741	-0.013571	-0.008648	0.011574	-0.003951	-0.003062	-
0.002001	0.006247	0.027983	-0.021121	-0.009365	0.016361	
0.014374	-0.004153					
y188	-0.026143	-0.014200	-0.053072	-0.007660	-0.026071	-
0.047429	-0.023311	-0.017518	0.015531	-0.027500	0.029000	
0.006165	0.009620	-0.000142	0.001962	-0.017279	0.017874	-
0.004442	0.012424	-0.008721	0.028246	-0.029050	-0.012437	
0.002386	-0.029744	-0.025269	0.001624	-0.010256	-0.012143	-
0.020296	0.013706	0.016458	0.019888	0.043788	0.001523	
0.029958	0.014447					
z188	-0.024845	-0.044464	0.013028	0.020080	0.006501	
0.008585	-0.004071	0.000895	-0.007125	0.004467	0.011166	
0.036483	-0.003411	0.011391	-0.003433	0.034843	0.005222	
0.014930	-0.011510	0.013185	0.012802	-0.012893	0.047068	
0.008375	-0.003295	-0.009153	-0.018611	0.011331	-0.024955	-
0.000781	0.012985	-0.002775	-0.014029	-0.040092	0.000101	
0.010404	0.024257					
x189	-0.029109	-0.022217	-0.005855	-0.015047	-0.024309	-
0.028981	-0.018408	-0.030466	0.021412	-0.004054	0.016004	
0.026835	-0.028652	-0.031247	0.022863	-0.010072	-0.023046	-
0.006631	0.007280	0.005226	-0.020960	-0.006150	0.023020	
0.015730	-0.007036	-0.012585	0.010115	0.002429	-0.001562	
0.001143	0.004494	0.020959	-0.021038	-0.007698	0.020119	
0.022619	-0.006141					
y189	-0.025790	-0.018053	-0.054498	-0.009240	-0.024204	-
0.044940	-0.022169	-0.014551	0.016726	-0.025557	0.027481	
0.014781	0.009340	0.000066	0.001910	-0.011426	0.018398	-
0.004720	0.013937	-0.002808	0.027368	-0.030636	-0.008217	
0.006416	-0.028865	-0.027082	0.000933	-0.018832	-0.010123	-
0.017048	0.016166	0.014623	0.013667	0.039204	0.004836	
0.031008	0.016980					
z189	-0.016623	-0.041632	0.017049	0.020847	0.008476	
0.014455	-0.000740	0.001067	-0.014557	0.005587	0.003441	
0.028835	-0.000336	0.016475	-0.003374	0.036709	-0.006333	
0.010491	-0.012345	0.016201	0.015140	-0.011519	0.047517	
0.007101	-0.002502	-0.003297	-0.027681	0.013548	-0.026173	
0.005780	0.009059	-0.002827	-0.022930	-0.040276	-0.001049	
0.005848	0.017878					
x190	-0.032849	-0.024949	-0.008642	-0.013326	-0.023312	-
0.034199	-0.014727	-0.029329	0.024951	-0.005958	0.020925	
0.032666	-0.028673	-0.028508	0.025119	-0.005057	-0.020310	-
0.009134	0.015037	-0.001025	-0.016167	-0.002424	0.025088	
0.015303	-0.001262	-0.017703	0.009142	0.008401	-0.000464	
0.007660	0.001686	0.014866	-0.022567	-0.003816	0.026361	
0.024367	-0.009064					
y190	-0.025021	-0.022238	-0.054844	-0.011755	-0.023309	-
0.043214	-0.019913	-0.011551	0.017020	-0.024077	0.025616	
0.022095	0.009620	0.000713	0.001370	-0.004518	0.018930	-
0.003562	0.013779	0.001946	0.025571	-0.029944	-0.003982	
0.009619	-0.024097	-0.028214	0.001647	-0.026537	-0.009107	-

0.014913 0.021281 0.010488 0.007599 0.031756 0.010966
0.029329 0.015784
z190 -0.010888 -0.037501 0.019730 0.022097 0.010817
0.017761 0.005149 -0.000562 -0.022750 0.005173 -0.002704
0.021337 0.006439 0.023578 -0.008897 0.031205 -0.007713
0.010997 -0.011382 0.018189 0.024006 -0.014397 0.043095
0.010811 0.003013 -0.007633 -0.029754 0.014737 -0.026259
0.007923 0.009634 -0.000287 -0.019006 -0.041258 -0.003756 -
0.002591 0.010563
x191 -0.035887 -0.027619 -0.011461 -0.012541 -0.022216 -
0.038660 -0.008594 -0.028682 0.028288 -0.006527 0.023882
0.040283 -0.028040 -0.024950 0.026738 0.000948 -0.017254 -
0.012536 0.023722 -0.006347 -0.012515 0.001825 0.026498
0.015311 0.005731 -0.025041 0.006885 0.016025 0.000456
0.015311 -0.000835 0.006162 -0.023555 -0.001222 0.034781
0.020266 -0.017709
y191 -0.023987 -0.026647 -0.053349 -0.014230 -0.023453 -
0.040341 -0.016698 -0.007054 0.016262 -0.022465 0.020708
0.029931 0.009771 0.000708 0.001393 0.000101 0.021496 -
0.000419 0.011986 0.009170 0.025874 -0.027714 -0.000136
0.012985 -0.018758 -0.028158 0.003438 -0.033851 -0.009294 -
0.011877 0.024960 0.002368 0.003160 0.022892 0.018747
0.025314 0.006793
z191 -0.004734 -0.032053 0.021584 0.023285 0.011321
0.019084 0.007712 -0.002919 -0.031597 0.004727 -0.007433
0.010162 0.011806 0.031771 -0.016769 0.028971 -0.012722
0.008794 -0.014073 0.017816 0.030702 -0.015813 0.035916
0.012479 0.003788 -0.005124 -0.027823 0.013201 -0.022330
0.005075 0.013230 -0.000070 -0.027512 -0.035407 -0.010264 -
0.000148 0.008726
x192 -0.038331 -0.030987 -0.014417 -0.012713 -0.020824 -
0.042304 -0.001069 -0.028711 0.030752 -0.006765 0.026007
0.048608 -0.026267 -0.019775 0.028098 0.008283 -0.015984 -
0.019451 0.031180 -0.011535 -0.009572 0.005779 0.029190
0.013958 0.013175 -0.033915 0.005706 0.020883 0.002000
0.025681 -0.002646 -0.001060 -0.023958 0.000452 0.044448
0.012687 -0.029349
y192 -0.022450 -0.030953 -0.051860 -0.017487 -0.023741 -
0.037640 -0.012116 -0.001302 0.015573 -0.019921 0.015721
0.036742 0.009982 0.001863 -0.001222 0.006029 0.022203
0.003185 0.009050 0.015474 0.025859 -0.023703 0.004059
0.016347 -0.014086 -0.025482 0.005718 -0.043013 -0.010528 -
0.006806 0.030948 -0.006780 -0.001246 0.012391 0.025680
0.021839 -0.004467
z192 0.002083 -0.024209 0.023680 0.026499 0.013008
0.019165 0.009530 -0.005550 -0.039244 0.002185 -0.012445 -
0.002689 0.014499 0.036237 -0.026200 0.022333 -0.016417
0.008958 -0.011585 0.017937 0.044460 -0.020442 0.031870
0.016223 0.006326 0.001263 -0.023887 0.010478 -0.014375
0.002565 0.014296 0.002949 -0.027792 -0.030502 -0.016765
0.005079 0.012130
x193 -0.039676 -0.034014 -0.018382 -0.013689 -0.018695 -
0.045048 0.008146 -0.029236 0.032936 -0.006842 0.027650
0.057532 -0.025293 -0.013428 0.028737 0.016755 -0.015230 -
0.027480 0.038367 -0.015988 -0.006376 0.009297 0.032377
0.012887 0.020548 -0.042655 0.004120 0.025775 0.004660
0.034612 -0.004480 -0.009900 -0.023204 -0.000176 0.054420
0.005332 -0.041551
y193 -0.020298 -0.035174 -0.048733 -0.021240 -0.024543 -
0.035305 -0.006451 0.005373 0.015170 -0.016983 0.008275

0.043431	0.010011	0.001524	-0.003435	0.011918	0.022936	
0.006413	0.004725	0.021801	0.026199	-0.018285	0.010409	
0.019952	-0.008491	-0.021694	0.009490	-0.051379	-0.011828	
0.002010	0.036528	-0.019004	-0.006499	-0.000078	0.035850	
0.016976	-0.018385					
z193	0.009038	-0.016672	0.026134	0.030392	0.013727	
0.018485	0.010599	-0.008658	-0.044674	0.001144	-0.017174	-
0.017475	0.018651	0.040363	-0.032173	0.015182	-0.020593	
0.007519	-0.011304	0.011420	0.051334	-0.028051	0.022995	
0.018027	0.003781	0.006125	-0.019734	0.002413	-0.005452	
0.004111	0.014643	0.004429	-0.031099	-0.022362	-0.027200	
0.006875	0.007547					
x194	-0.039451	-0.033913	-0.015313	-0.002757	-0.007292	-
0.041932	0.016187	-0.024231	0.031437	-0.009915	0.023835	
0.056158	-0.024844	-0.004734	0.034613	0.023540	-0.019404	-
0.032947	0.039907	-0.021921	-0.003365	0.009441	0.027649	
0.009932	0.009865	-0.036828	-0.012126	0.035467	-0.001937	
0.014866	-0.002376	-0.013531	-0.011271	-0.005121	0.038475	
0.000483	-0.030171					
y194	-0.018301	-0.029814	-0.043900	-0.013398	-0.016654	-
0.022484	-0.005190	0.012544	0.014705	-0.011407	-0.001964	
0.037735	0.006633	-0.001032	0.004542	0.019079	0.013856	
0.015876	0.007245	0.016698	0.020313	-0.015385	0.005229	
0.014886	-0.004095	-0.010438	0.011120	-0.041931	-0.010487	-
0.004289	0.038026	-0.018029	-0.013775	0.003019	0.020052	
0.019259	-0.007266					
z194	0.015007	-0.008365	0.033757	0.030839	0.009716	
0.016914	0.010055	-0.009919	-0.053483	-0.001370	-0.021436	-
0.034768	0.020239	0.039150	-0.040097	0.005178	-0.016596	
0.002607	-0.020126	0.004114	0.049004	-0.027096	0.016446	
0.011572	-0.000914	0.011401	-0.020450	-0.014429	0.005716	
0.003227	0.021496	0.007214	-0.038527	-0.024176	-0.016411	
0.018444	-0.011460					
x195	-0.039336	-0.033035	-0.011883	0.006356	0.002618	-
0.038475	0.021770	-0.019471	0.029268	-0.011766	0.018990	
0.053494	-0.023231	0.002303	0.037799	0.028590	-0.023251	-
0.036617	0.040122	-0.027423	-0.000818	0.009225	0.023813	
0.005045	0.000426	-0.030920	-0.025016	0.041805	-0.006888	
0.000620	0.000166	-0.016084	-0.001586	-0.007587	0.023989	-
0.003251	-0.020966					
y195	-0.015964	-0.024836	-0.039344	-0.008147	-0.009744	-
0.010323	-0.002746	0.018387	0.013323	-0.004620	-0.010055	
0.032456	0.002291	-0.003418	0.009907	0.022975	0.007472	
0.022686	0.009823	0.014309	0.015738	-0.014066	0.000490	
0.009767	-0.001592	-0.002133	0.012597	-0.032955	-0.006475	-
0.010110	0.035347	-0.015293	-0.018986	0.003585	0.007087	
0.019683	0.001522					
z195	0.019620	-0.001893	0.037476	0.028299	0.004986	
0.015197	0.011891	-0.010902	-0.059224	-0.000034	-0.023180	-
0.050428	0.019959	0.036903	-0.049274	-0.006592	-0.012437	-
0.004910	-0.022787	-0.000543	0.047298	-0.025896	0.011160	
0.003653	-0.005921	0.011836	-0.018542	-0.025082	0.020221	
0.003954	0.017281	0.013038	-0.035355	-0.029662	-0.006984	
0.025386	-0.023795					
x196	-0.038418	-0.030747	-0.007650	0.013441	0.011294	-
0.034426	0.024818	-0.015549	0.026747	-0.012225	0.015409	
0.050578	-0.020899	0.008351	0.037648	0.029964	-0.024990	-
0.038742	0.039872	-0.029458	0.001625	0.007539	0.021391	
0.000270	-0.007974	-0.025199	-0.035052	0.043246	-0.010882	-

0.008058 0.003259 -0.017465 0.007176 -0.009850 0.013014 -
0.005018 -0.014098
y196 -0.013886 -0.020606 -0.036889 -0.001070 -0.004538 -
0.003250 0.000249 0.023029 0.011251 -0.001208 -0.018945
0.023998 -0.002287 -0.005841 0.016204 0.026023 0.000368
0.028597 0.012804 0.008190 0.012312 -0.011557 -0.007939
0.005634 0.000515 0.006174 0.011044 -0.026946 -0.002609 -
0.014907 0.029010 -0.014548 -0.023611 0.002009 -0.005537
0.023182 0.005298
z196 0.023517 0.002734 0.037881 0.029834 -0.000184
0.008568 0.012817 -0.014682 -0.060603 -0.001436 -0.024673 -
0.065243 0.020767 0.034337 -0.051939 -0.017819 -0.009255 -
0.010158 -0.021506 -0.008950 0.043329 -0.024435 0.004678
0.001873 -0.007150 0.012030 -0.021667 -0.035689 0.029048
0.007974 0.008496 0.013282 -0.031439 -0.034246 0.000894
0.035331 -0.036080
x197 -0.036546 -0.028310 -0.003243 0.019735 0.017329 -
0.030511 0.025026 -0.011608 0.023281 -0.011054 0.008370
0.047061 -0.018870 0.011543 0.035449 0.029439 -0.025206 -
0.039166 0.038683 -0.031065 0.001131 0.008600 0.018628 -
0.002313 -0.015201 -0.018236 -0.043186 0.041043 -0.012248 -
0.011244 0.002759 -0.018509 0.011819 -0.010323 0.005397 -
0.007992 -0.012080
y197 -0.012588 -0.016390 -0.031969 0.001786 -0.001303
0.003602 0.003633 0.024586 0.008599 0.002676 -0.021531
0.016735 -0.003495 -0.005664 0.018384 0.026237 -0.003123
0.032874 0.015207 0.004017 0.008400 -0.005878 -0.010232
0.000222 0.005174 0.009512 0.013653 -0.016484 0.002877 -
0.018261 0.025283 -0.014248 -0.024072 0.009029 -0.011183
0.018494 0.011438
z197 0.025475 0.007263 0.041221 0.027247 -0.008087
0.004850 0.013252 -0.022230 -0.059614 -0.000916 -0.021558 -
0.073207 0.022449 0.031797 -0.055195 -0.027603 -0.000708 -
0.013185 -0.020974 -0.012275 0.037399 -0.021241 0.005574
0.004124 -0.001463 0.009094 -0.019368 -0.035808 0.035867
0.014316 0.003807 0.009586 -0.025794 -0.023730 0.012107
0.033261 -0.035646
x198 -0.034850 -0.025534 0.001263 0.024318 0.021484 -
0.027210 0.023646 -0.009304 0.016728 -0.008421 0.002313
0.042741 -0.017276 0.014085 0.030458 0.026886 -0.022191 -
0.036580 0.034532 -0.032778 0.001791 0.009772 0.016160 -
0.005135 -0.016996 -0.013894 -0.045805 0.034065 -0.012727 -
0.012420 0.003810 -0.015429 0.018169 -0.011521 0.001219 -
0.009185 -0.012279
y198 -0.010522 -0.013419 -0.029386 0.004052 0.002477
0.010170 0.006468 0.025255 0.001587 0.007003 -0.026045
0.004818 -0.009946 -0.004862 0.017044 0.027558 -0.010173
0.031891 0.014411 0.003314 0.003676 -0.003629 -0.013425 -
0.001676 0.007241 0.013015 0.019403 -0.017450 0.013223 -
0.018962 0.013076 -0.009347 -0.025808 0.006443 -0.012997
0.020985 0.002980
z198 0.026974 0.008921 0.040228 0.024317 -0.013152
0.002935 0.012785 -0.028607 -0.058407 0.001642 -0.019276 -
0.080529 0.020301 0.028124 -0.056176 -0.033705 0.004735 -
0.018540 -0.019090 -0.010163 0.027408 -0.021446 0.006141
0.012766 0.002474 0.006878 -0.016394 -0.041744 0.042354
0.019514 -0.009788 0.009800 -0.021804 -0.022803 0.021392
0.038629 -0.042473
x199 -0.031995 -0.021726 0.007281 0.025605 0.022823 -
0.023477 0.019349 -0.008153 0.010352 -0.003712 -0.004083

0.036708	-0.014207	0.012742	0.021931	0.021197	-0.017060	-
0.033916	0.030240	-0.031550	0.001305	0.010592	0.010715	-
0.007171	-0.018999	-0.008305	-0.044668	0.023710	-0.009602	-
0.008489	0.010424	-0.007849	0.016598	-0.013272	0.006023	-
0.010332	-0.017343					
y199	-0.008137	-0.009975	-0.024062	0.000337	0.006515	
0.018464	0.011633	0.024189	-0.003996	0.014652	-0.023841	-
0.003816	-0.010162	-0.004316	0.009199	0.020337	-0.009986	
0.025817	0.018211	0.007120	-0.003136	-0.004343	-0.014718	-
0.001473	0.006411	0.016111	0.017661	-0.019567	0.024933	-
0.018605	0.000522	-0.004372	-0.028881	0.009768	-0.004494	
0.016247	-0.011079					
z199	0.027431	0.010240	0.040726	0.016572	-0.018163	
0.003338	0.013358	-0.036441	-0.051088	0.006960	-0.013237	-
0.080474	0.022633	0.019593	-0.056589	-0.042096	0.015973	-
0.024450	-0.011341	-0.003308	0.014975	-0.024116	0.004787	
0.025756	0.003594	0.004548	-0.015215	-0.038184	0.046280	
0.023446	-0.016920	0.010425	-0.025618	-0.019495	0.037421	
0.036330	-0.044369					
x200	-0.029373	-0.017529	0.013188	0.026630	0.022700	-
0.020808	0.011534	-0.004300	0.000569	0.001729	-0.013205	
0.030805	-0.013135	0.009661	0.012561	0.014416	-0.010662	-
0.028648	0.021394	-0.027652	-0.000457	0.012376	0.007183	-
0.004468	-0.018368	-0.004438	-0.043256	0.006542	-0.009974	
0.002928	0.012538	0.000385	0.016695	-0.009206	0.009507	-
0.009130	-0.023928					
y200	-0.008632	-0.005847	-0.020981	-0.001843	0.006341	
0.020485	0.016806	0.019487	-0.013342	0.020206	-0.023591	-
0.013262	-0.015756	-0.002194	0.005942	0.020168	-0.009899	
0.024140	0.014610	0.006262	-0.002374	-0.002644	-0.017362	-
0.002299	0.010994	0.012643	0.022249	-0.014936	0.032003	-
0.018013	-0.008160	0.002834	-0.028927	0.011512	0.000631	
0.009103	-0.024664					
z200	0.023736	0.011128	0.039123	0.011267	-0.023166	
0.002761	0.013323	-0.044179	-0.043556	0.010302	-0.005849	-
0.071862	0.020651	0.012698	-0.047349	-0.044288	0.026006	-
0.025806	-0.010162	0.004172	0.005883	-0.027832	0.006300	
0.039308	0.010295	-0.003421	-0.016524	-0.027981	0.038233	
0.026119	-0.030421	0.006533	-0.019527	-0.011403	0.040570	
0.031217	-0.032945					
x201	-0.025606	-0.011588	0.020498	0.022980	0.023153	-
0.014155	0.003330	-0.000278	-0.006406	0.010192	-0.015739	
0.031303	-0.007629	0.005834	0.000155	-0.001435	0.001912	-
0.020958	0.014927	-0.019003	-0.003035	0.011803	0.004062	-
0.007446	-0.022733	0.003593	-0.049476	-0.007576	-0.013814	
0.018317	0.016539	0.008590	0.019249	-0.005141	0.014865	-
0.006623	-0.022639					
y201	-0.006574	-0.001889	-0.017669	-0.007799	0.005446	
0.023225	0.021831	0.016320	-0.016158	0.024294	-0.019516	-
0.019003	-0.015918	0.001194	0.004188	0.012948	-0.011823	
0.018465	0.017843	0.010776	-0.001061	-0.001045	-0.014408	-
0.014408	-0.000404	0.018806	0.013088	-0.006362	0.036922	-
0.017947	-0.022691	0.003237	-0.030867	0.009018	-0.004231	
0.010996	-0.038181					
z201	0.018851	0.009971	0.035038	0.005337	-0.026501	
0.004185	0.012326	-0.050808	-0.028528	0.011845	0.003779	-
0.055787	0.021010	0.005172	-0.034349	-0.046438	0.033967	-
0.027074	-0.002813	0.014563	-0.005644	-0.031334	0.007466	
0.052405	0.013928	-0.010763	-0.024183	-0.008912	0.025393	

0.027671 -0.048015 -0.005113 -0.014698 -0.004621 0.035387
0.025469 -0.008544
x202 -0.023368 -0.008468 0.023695 0.026387 0.020013 -
0.013327 -0.009495 0.003800 -0.014752 0.008713 -0.031904
0.026993 -0.006823 0.002348 -0.010345 -0.004983 0.005602 -
0.009912 0.003081 -0.021963 -0.004496 0.016686 -0.007121 -
0.009201 -0.015998 0.005780 -0.040469 -0.027395 -0.014354
0.034359 0.018775 0.008622 0.013351 -0.003549 0.019100 -
0.004216 -0.013976
y202 -0.007623 -0.000637 -0.019098 -0.012161 0.005585
0.020834 0.029048 0.008488 -0.020651 0.019690 -0.013838 -
0.025453 -0.013289 0.008733 -0.004058 0.009238 -0.010351
0.011897 0.020999 0.004595 0.002523 -0.001673 -0.016054 -
0.021139 0.002053 0.009510 0.016357 0.005410 0.045351 -
0.027593 -0.033095 -0.005397 -0.031456 0.004172 -0.005948
0.007436 -0.039929
z202 0.012335 0.007020 0.028661 -0.002124 -0.029333
0.007726 0.012477 -0.059053 -0.008715 0.014450 0.016522 -
0.034886 0.023206 -0.001652 -0.022136 -0.046210 0.046963 -
0.028607 0.010292 0.024393 -0.020962 -0.035828 0.009710
0.071439 0.021580 -0.018518 -0.028721 0.018214 0.007833
0.028017 -0.063795 -0.025639 -0.011263 0.005879 0.028394
0.016967 0.022955
x203 -0.025148 -0.005201 0.022244 0.026793 0.014851 -
0.012162 -0.011851 0.004516 -0.014788 0.001375 -0.035602
0.021119 0.001010 0.000250 -0.009937 -0.004554 -0.002706 -
0.007711 -0.008390 -0.022282 -0.000864 0.023230 -0.007437 -
0.013962 -0.008593 0.000352 -0.027623 -0.029072 -0.000470
0.030425 0.004280 -0.007398 0.001959 0.006519 0.021771 -
0.004172 -0.020158
y203 -0.006679 -0.002083 -0.020720 -0.012455 0.004126
0.019337 0.030905 0.004940 -0.019250 0.018396 -0.016414 -
0.030247 -0.008010 0.017124 -0.005210 0.006586 -0.010932
0.002759 0.017374 0.010039 0.009109 0.000116 -0.015061 -
0.015925 -0.007429 0.005042 0.015953 0.004644 0.041187 -
0.029795 -0.036835 -0.010841 -0.028510 0.002201 -0.011364
0.001199 -0.033162
z203 0.013326 0.006211 0.033128 -0.002117 -0.031283
0.011970 0.007473 -0.057708 -0.013259 0.005725 0.009182 -
0.024024 0.012205 -0.005353 -0.011195 -0.040232 0.047907 -
0.016759 0.011493 0.032313 -0.004324 -0.034368 0.016660
0.066494 0.009136 -0.017750 -0.032884 0.029783 0.013484
0.038058 -0.038286 -0.021224 -0.016682 0.002133 0.009329 -
0.000650 0.029962
x204 -0.026466 -0.001688 0.020312 0.024051 0.008005 -
0.008845 -0.007867 0.000795 -0.013292 -0.001401 -0.031873
0.019616 0.005939 -0.000175 -0.002304 -0.015189 -0.008557 -
0.012201 -0.011403 -0.023831 0.003060 0.024024 -0.001080 -
0.022333 -0.002930 -0.000646 -0.017289 -0.026948 0.004537
0.026731 -0.001164 -0.010784 -0.000066 0.015204 0.014355 -
0.002572 -0.017658
y204 -0.005865 -0.003964 -0.023585 -0.012258 0.000818
0.015817 0.035905 0.000896 -0.016130 0.018755 -0.017737 -
0.030720 -0.005352 0.024052 -0.000074 -0.000353 -0.015252 -
0.008737 0.018179 0.009319 0.015085 0.001257 -0.012516 -
0.014390 -0.012065 0.001132 0.016392 0.001820 0.032947 -
0.034472 -0.033611 -0.012683 -0.019103 -0.000199 -0.019652 -
0.004124 -0.022372
z204 0.013505 0.005463 0.036269 -0.000921 -0.033755
0.014659 0.004436 -0.055886 -0.016537 -0.001722 0.003133 -

0.015401	0.000810	-0.006056	0.001564	-0.033728	0.044410	-
0.007822	0.012811	0.034463	0.011686	-0.032745	0.024297	
0.059916	0.000822	-0.016241	-0.036472	0.037743	0.015701	
0.046840	-0.014313	-0.016638	-0.016771	0.003374	-0.010123	-
0.013067	0.039837					
x205	-0.028558	0.000293	0.016351	0.023844	0.006302	-
0.008488	-0.003888	0.002625	-0.010993	-0.003253	-0.032312	
0.020313	0.008275	0.000811	-0.003559	-0.018058	-0.019372	-
0.018995	-0.015862	-0.024531	0.007974	0.029538	-0.001203	-
0.020468	-0.001177	-0.006460	-0.007808	-0.029502	0.014372	
0.024756	-0.000198	-0.014038	-0.004267	0.026157	0.003514	-
0.006823	-0.015805					
y205	-0.004982	-0.005442	-0.026467	-0.011846	-0.000223	
0.011351	0.039901	0.000385	-0.011350	0.018880	-0.019208	-
0.029584	-0.002659	0.029438	-0.002301	-0.002597	-0.019679	-
0.017485	0.018643	0.008189	0.018975	0.003148	-0.010063	-
0.009599	-0.014284	-0.002949	0.013190	-0.003901	0.030006	-
0.036588	-0.027474	-0.017345	-0.012550	0.004690	-0.023656	-
0.012963	-0.016547					
z205	0.013630	0.004734	0.037547	-0.000319	-0.033039	
0.017889	0.003609	-0.051555	-0.017252	-0.006746	-0.000172	-
0.008335	-0.008055	-0.007398	0.008702	-0.027776	0.041607	-
0.001828	0.014433	0.035118	0.022871	-0.029692	0.027984	
0.053073	-0.007415	-0.018184	-0.038548	0.041811	0.018922	
0.051060	0.005232	-0.012050	-0.015996	0.003917	-0.024597	-
0.022570	0.045988					
x206	-0.030042	0.000126	0.012748	0.024043	0.007493	-
0.006159	-0.002722	0.005772	-0.009230	-0.005335	-0.032049	
0.022316	0.012608	-0.000753	-0.007179	-0.011904	-0.025399	-
0.023709	-0.018241	-0.026094	0.010127	0.031650	-0.004599	-
0.011795	-0.003237	-0.012818	-0.000358	-0.035296	0.019357	
0.029030	-0.003770	-0.017589	0.000638	0.030848	0.006048	-
0.003314	-0.019227					
y206	-0.003505	-0.006627	-0.028391	-0.011086	-0.002035	
0.008021	0.042280	0.000796	-0.006228	0.018027	-0.021001	-
0.027138	-0.000378	0.030381	-0.004007	-0.000066	-0.021977	-
0.022072	0.018199	0.005866	0.020798	0.002944	-0.008114	-
0.006687	-0.013910	-0.006036	0.008630	-0.010155	0.025289	-
0.035774	-0.022770	-0.022176	-0.006383	0.011934	-0.020537	-
0.017869	-0.012563					
z206	0.013324	0.003727	0.037976	0.000093	-0.030822	
0.020568	0.002874	-0.045797	-0.015734	-0.010181	-0.001952	-
0.003571	-0.014363	-0.009073	0.012644	-0.020581	0.039553	
0.003274	0.015563	0.031603	0.029775	-0.025975	0.029569	
0.048250	-0.014724	-0.021537	-0.037373	0.043052	0.022582	
0.053051	0.018835	-0.009802	-0.014056	0.003281	-0.030808	-
0.025870	0.047301					
x207	-0.029414	-0.000170	0.009497	0.023062	0.007678	-
0.005266	-0.002379	0.004605	-0.006353	-0.004261	-0.033010	
0.021250	0.016313	-0.003530	-0.008132	-0.008315	-0.027265	-
0.028314	-0.018998	-0.025931	0.007613	0.029304	-0.010164	-
0.002628	-0.006863	-0.012030	0.001996	-0.037152	0.025033	
0.031868	-0.004951	-0.022155	-0.005645	0.025027	0.013184	
0.000623	-0.021693					
y207	-0.001194	-0.006479	-0.029933	-0.009441	-0.005421	
0.004431	0.043462	0.001015	0.000195	0.016845	-0.024134	-
0.024410	0.000603	0.028318	-0.002820	0.000424	-0.023890	-
0.022982	0.016797	0.002015	0.021710	0.002170	-0.005304	-
0.009058	-0.011089	-0.007140	0.003072	-0.016526	0.021525	-

0.035215 -0.016254 -0.025698 -0.004492 0.016399 -0.015276 -
0.022336 -0.009402
z207 0.012496 0.002717 0.037464 0.000870 -0.028101
0.021728 0.002819 -0.039269 -0.012333 -0.012681 -0.002425 -
0.002190 -0.018960 -0.008535 0.014410 -0.013620 0.037113
0.006514 0.015865 0.025287 0.034142 -0.022001 0.030785
0.043058 -0.018584 -0.026352 -0.035255 0.040405 0.025266
0.052127 0.025730 -0.008489 -0.012804 0.003334 -0.034605 -
0.026424 0.047762
x208 -0.026624 0.001086 0.011406 0.019904 0.007653 -
0.000604 -0.000931 0.001305 -0.006906 0.002229 -0.030744
0.021467 0.020659 -0.006238 -0.013394 -0.009762 -0.023695 -
0.032564 -0.012014 -0.016502 0.005324 0.017260 -0.009434
0.002691 -0.016015 -0.005090 0.005940 -0.030185 0.029381
0.036917 -0.005191 -0.022759 -0.007073 0.016722 0.011565
0.002904 -0.013603
y208 0.001857 -0.005781 -0.030822 -0.006766 -0.009207 -
0.000025 0.044193 0.002039 0.006354 0.015167 -0.028147 -
0.020838 0.000036 0.024262 -0.001599 0.001781 -0.026651 -
0.020246 0.014629 -0.003336 0.021570 0.001194 -0.000341 -
0.013954 -0.004237 -0.008828 -0.000463 -0.022473 0.018605 -
0.035393 -0.008922 -0.029132 -0.003681 0.023146 -0.009769 -
0.027399 -0.006836
z208 0.010745 0.000988 0.035046 0.003407 -0.025216
0.020035 0.003769 -0.031539 -0.008346 -0.015236 -0.003623 -
0.004630 -0.024014 -0.005634 0.016108 -0.004340 0.030732
0.008326 0.012293 0.013941 0.036728 -0.015940 0.029592
0.036138 -0.017639 -0.034806 -0.030121 0.034215 0.025455
0.048322 0.025671 -0.006883 -0.011057 0.005494 -0.037169 -
0.023780 0.046786
x209 -0.023763 0.001162 0.012627 0.019569 0.006038
0.001199 -0.003959 -0.004746 -0.009300 0.004830 -0.034076
0.019891 0.018262 -0.005277 -0.008072 -0.008097 -0.011830 -
0.032216 -0.010127 -0.007230 0.004895 0.008738 -0.007292
0.015292 -0.021062 -0.004967 0.008044 -0.023475 0.019610
0.043022 -0.007053 -0.021677 -0.010727 0.003740 0.003350
0.002514 -0.006148
y209 0.005135 -0.004862 -0.031991 -0.004415 -0.013007 -
0.005210 0.044645 0.004152 0.014116 0.013866 -0.030712 -
0.015672 -0.000698 0.019438 -0.000264 0.003379 -0.026635 -
0.014465 0.012670 -0.011016 0.020617 0.000936 0.004949 -
0.020557 0.006914 -0.011747 -0.005094 -0.027614 0.012907 -
0.036858 0.001224 -0.032601 -0.004662 0.031693 -0.003097 -
0.032284 -0.003699
z209 0.008423 -0.000436 0.031019 0.003325 -0.020552
0.018818 0.008626 -0.022413 -0.001470 -0.014483 0.001236 -
0.008918 -0.025225 -0.000927 0.012891 0.003836 0.024817
0.006227 0.008635 -0.001129 0.037221 -0.009593 0.025846
0.024435 -0.015513 -0.043872 -0.023723 0.025202 0.023928
0.038475 0.021988 -0.005722 -0.007819 0.009302 -0.036073 -
0.018111 0.041705
x210 -0.023475 -0.000056 0.009520 0.019457 0.006169
0.000120 -0.003851 -0.011585 -0.010425 0.006600 -0.034601
0.017133 0.018350 -0.000944 -0.005340 -0.010082 -0.004222 -
0.036599 -0.008677 -0.006868 0.008187 0.002539 -0.012223
0.027245 -0.022722 -0.012375 0.003651 -0.018833 0.015891
0.043089 -0.002736 -0.017548 -0.010084 -0.021600 -0.005917 -
0.010350 -0.009474
y210 0.008461 -0.003311 -0.032755 -0.001793 -0.017339 -
0.010198 0.045771 0.007979 0.022425 0.010592 -0.032408 -

0.011013	-0.002075	0.010994	0.000099	0.006653	-0.025573	-
0.005479	0.012296	-0.020825	0.016244	-0.002233	0.008489	-
0.032156	0.018997	-0.013254	-0.011042	-0.033132	0.012496	-
0.039582	0.012352	-0.038131	-0.007172	0.042853	0.008660	-
0.037503	0.000465					
z210	0.006230	-0.001126	0.027046	0.002145	-0.015130	
0.018168	0.015823	-0.010805	0.007137	-0.010954	0.009128	-
0.015727	-0.024692	0.003189	0.005204	0.014664	0.019132	
0.002115	0.005618	-0.015601	0.033774	-0.005399	0.022140	
0.009291	-0.013734	-0.053667	-0.012178	0.013570	0.020107	
0.026731	0.013552	-0.007629	-0.004092	0.022699	-0.031543	-
0.003817	0.040100					
x211	-0.023118	-0.002019	0.002278	0.016675	0.010987	-
0.005682	0.005390	-0.012248	-0.007353	0.011196	-0.028094	
0.012324	0.019448	0.005696	-0.009337	0.000918	-0.004102	-
0.049327	-0.010768	-0.008389	0.010648	-0.010682	-0.021722	
0.024397	-0.031151	-0.020750	0.011089	-0.017239	0.012609	
0.040850	-0.007280	-0.021529	-0.006290	-0.022705	-0.016118	-
0.002645	0.011640					
y211	0.013560	-0.000773	-0.029713	0.003912	-0.022704	-
0.014067	0.041192	0.010759	0.030236	0.006102	-0.039149	-
0.003766	-0.004039	-0.000651	0.002466	0.005717	-0.021742	
0.012321	0.011855	-0.031227	0.008709	-0.005473	0.015937	-
0.038111	0.036520	-0.011256	-0.016262	-0.038767	0.012876	-
0.040447	0.026264	-0.043676	-0.013702	0.051287	0.026511	-
0.047880	-0.006782					
z211	0.005255	-0.000493	0.025750	0.004755	-0.009125	
0.018814	0.016095	-0.004857	0.015991	-0.005920	0.013587	-
0.022110	-0.025307	0.007827	-0.000206	0.013887	0.018808	
0.002519	0.001009	-0.029834	0.027943	-0.000371	0.022571	
0.004167	-0.009936	-0.060633	-0.004131	0.004071	0.023609	
0.016761	0.000861	-0.002776	-0.001696	0.016032	-0.018189	
0.008645	0.033386					
x212	-0.022562	-0.004428	-0.005271	0.016694	0.014176	-
0.004660	0.008559	-0.014606	-0.003222	0.012840	-0.022905	
0.008295	0.011529	0.014779	-0.010594	0.007034	-0.006966	-
0.038855	-0.009758	-0.020726	0.002974	-0.012664	-0.019105	
0.020773	-0.024956	-0.010221	0.016560	-0.011371	0.014963	
0.029483	-0.022878	-0.013049	0.012179	-0.005093	-0.013125	-
0.025794	-0.003685					
y212	0.013239	0.000488	-0.030376	0.004441	-0.021035	-
0.008315	0.033128	0.008076	0.023147	0.003708	-0.042855	
0.001050	-0.003008	-0.008129	-0.002536	-0.001911	-0.021554	
0.007644	0.008893	-0.025800	0.003608	-0.006211	0.016370	-
0.030003	0.031415	-0.005012	-0.015444	-0.040283	0.004158	-
0.026444	0.024720	-0.044593	-0.008592	0.045468	0.023826	-
0.035535	-0.005097					
z212	0.002244	-0.002278	0.024130	0.006855	-0.010541	
0.014538	0.014854	-0.005301	0.023591	-0.013736	0.012175	-
0.025310	-0.019960	0.011660	0.005013	0.009612	0.014540	-
0.001273	-0.009137	-0.030488	0.024869	0.003236	0.022068	
0.006257	-0.000436	-0.051897	-0.002087	-0.009823	0.013540	-
0.002759	0.001296	0.004450	0.000416	0.024963	-0.007509	-
0.003338	0.048073					
x213	-0.022546	-0.006569	-0.009043	0.017167	0.016045	-
0.002113	0.009885	-0.017749	-0.001827	0.011642	-0.017072	
0.005160	0.005771	0.019853	-0.008729	0.011732	-0.005405	-
0.029462	-0.008243	-0.025248	-0.005433	-0.003149	-0.016814	
0.018633	-0.018276	0.004588	0.022820	-0.010749	0.003819	

0.022865 -0.034838 0.002961 0.025928 0.001439 -0.010838 -
0.033075 -0.010239
y213 0.012891 0.000860 -0.030211 0.005021 -0.019113 -
0.002728 0.025821 0.007050 0.017194 0.001050 -0.046195
0.003626 -0.002235 -0.013477 -0.007131 -0.010826 -0.021238
0.004569 0.005955 -0.021274 -0.002295 -0.004417 0.019578 -
0.022134 0.028380 0.000229 -0.014233 -0.040922 -0.005970 -
0.014968 0.022862 -0.044954 -0.007414 0.037737 0.024674 -
0.023973 -0.002608
z213 0.000702 -0.003554 0.021443 0.008765 -0.011736
0.009920 0.014223 -0.006184 0.030231 -0.018866 0.011788 -
0.028602 -0.016278 0.015586 0.008567 0.004622 0.007526 -
0.007442 -0.019171 -0.029726 0.024275 0.000124 0.016260
0.009026 0.003887 -0.049698 -0.001799 -0.016896 0.010910 -
0.020349 -0.002438 0.008763 0.007488 0.031516 -0.000156 -
0.015564 0.059319
x214 -0.022832 -0.008215 -0.010332 0.016364 0.017545 -
0.001814 0.010638 -0.018597 0.001253 0.009798 -0.012939
0.003281 0.000710 0.023106 -0.009535 0.017266 -0.001111 -
0.023906 -0.009706 -0.027566 -0.009979 0.002853 -0.014906
0.023783 -0.012231 0.015578 0.026279 -0.010568 -0.001680
0.016721 -0.038140 0.012102 0.036942 0.013272 -0.013760 -
0.032248 -0.011191
y214 0.012764 0.000671 -0.029560 0.005257 -0.017546
0.002434 0.019792 0.006579 0.012924 -0.001179 -0.048878
0.004296 -0.002297 -0.017702 -0.010866 -0.017336 -0.020416
0.002884 0.003649 -0.017305 -0.007371 -0.001552 0.021779 -
0.015709 0.026113 0.005484 -0.013294 -0.040785 -0.014205 -
0.004779 0.021155 -0.044423 -0.005899 0.030422 0.025082 -
0.013583 -0.001934
z214 -0.000471 -0.004326 0.018526 0.010174 -0.014019
0.006966 0.012858 -0.007890 0.032464 -0.021174 0.010260 -
0.031173 -0.013482 0.019796 0.009364 0.000627 0.001754 -
0.008973 -0.024854 -0.028245 0.024343 -0.000261 0.014180
0.010132 0.004711 -0.045519 -0.000119 -0.021262 0.006803 -
0.031353 -0.006452 0.011054 0.008508 0.035292 0.010274 -
0.030581 0.064805
x215 -0.021540 -0.008692 -0.011318 0.015526 0.018621 -
0.000923 0.011060 -0.018561 0.003601 0.010236 -0.008552
0.001129 -0.002533 0.023467 -0.009552 0.018537 0.003435 -
0.020421 -0.009122 -0.029033 -0.010772 0.004176 -0.012201
0.021106 -0.007038 0.024538 0.025869 -0.010578 -0.005162
0.012400 -0.042574 0.016708 0.045555 0.015111 -0.019137 -
0.028054 -0.010290
y215 0.012746 0.000782 -0.028663 0.005250 -0.015648
0.006585 0.015953 0.006196 0.010432 -0.002731 -0.049928
0.003583 -0.001859 -0.020542 -0.014242 -0.020233 -0.017362
0.003225 0.002607 -0.015504 -0.010330 0.001918 0.022602 -
0.010883 0.025105 0.008312 -0.012178 -0.038927 -0.020062
0.002058 0.019986 -0.042576 -0.002985 0.022564 0.024738 -
0.006210 -0.003454
z215 -0.001877 -0.005757 0.015496 0.010183 -0.016457
0.005321 0.011129 -0.009306 0.031826 -0.023132 0.007904 -
0.033069 -0.012767 0.022517 0.009147 -0.001929 -0.002148 -
0.008626 -0.027902 -0.027369 0.020898 0.000628 0.011703
0.011811 0.005649 -0.040511 0.002651 -0.022657 0.003163 -
0.038446 -0.005046 0.012423 0.009068 0.040037 0.022179 -
0.042607 0.064284
x216 -0.020436 -0.007982 -0.013631 0.015231 0.020061 -
0.000639 0.012416 -0.019304 0.003743 0.010249 -0.004834 -

0.000266 -0.006841 0.020839 -0.008121 0.015836 0.005417 -
0.018890 -0.009380 -0.034419 -0.008749 0.001976 -0.014974
0.015857 0.001484 0.025291 0.022792 -0.008796 -0.002786
0.009100 -0.040254 0.019540 0.046975 0.015020 -0.016257 -
0.024398 -0.009699
y216 0.013250 0.001019 -0.027024 0.005236 -0.013572
0.009702 0.013930 0.006532 0.010333 -0.003634 -0.050510
0.001344 -0.002134 -0.021957 -0.015890 -0.019852 -0.014392
0.004553 0.001586 -0.015773 -0.011881 0.006588 0.022201 -
0.008219 0.024261 0.010908 -0.011599 -0.036217 -0.023506
0.005681 0.017755 -0.039836 -0.001992 0.017914 0.022606 -
0.001413 -0.007544
z216 -0.002018 -0.008064 0.013531 0.008819 -0.019343
0.006401 0.007949 -0.009348 0.028171 -0.023342 0.005119 -
0.035937 -0.012266 0.023283 0.007453 -0.003435 -0.004096 -
0.006405 -0.027546 -0.024869 0.015819 0.001645 0.014245
0.010045 0.003425 -0.031799 0.003103 -0.020012 -0.002402 -
0.040351 -0.004461 0.008935 0.013616 0.035726 0.026069 -
0.044397 0.060552
x217 -0.017980 -0.007275 -0.013911 0.012946 0.021148
0.002970 0.014133 -0.019987 0.002716 0.011999 0.000413 -
0.000173 -0.009171 0.016580 -0.008895 0.013419 0.010549 -
0.016575 -0.005932 -0.035294 -0.005238 -0.000414 -0.012830
0.011590 0.005686 0.023032 0.015907 -0.004605 0.000173
0.012583 -0.028600 0.021426 0.045073 0.014315 -0.012586 -
0.014039 -0.014323
y217 0.014028 0.001245 -0.024389 0.004560 -0.010871
0.012386 0.014161 0.006663 0.011927 -0.003737 -0.048626 -
0.002471 -0.002459 -0.021485 -0.016735 -0.016890 -0.009018
0.008271 -0.000089 -0.017618 -0.011030 0.012500 0.022497 -
0.008209 0.025844 0.012007 -0.010819 -0.030886 -0.024492
0.006035 0.015124 -0.035802 0.000080 0.013249 0.020218
0.001264 -0.009904
z217 -0.002781 -0.009612 0.011292 0.007322 -0.023303
0.006722 0.003831 -0.009256 0.022686 -0.023702 0.000601 -
0.037521 -0.014550 0.022335 0.004558 -0.006605 -0.006030 -
0.005955 -0.027161 -0.023768 0.012538 -0.000527 0.015097
0.007492 0.002282 -0.021538 0.002806 -0.015641 -0.007412 -
0.042460 -0.005907 0.002442 0.015355 0.028673 0.026239 -
0.043439 0.057684
x218 -0.015491 -0.007403 -0.015829 0.010982 0.020501
0.005181 0.015324 -0.020399 -0.000512 0.010907 0.003737
0.001632 -0.011483 0.011172 -0.010438 0.009868 0.010266 -
0.015120 -0.001263 -0.038265 -0.001115 -0.002595 -0.010373
0.013791 0.010053 0.017100 0.008940 0.001946 0.004757
0.016848 -0.014410 0.019666 0.037734 0.013056 -0.008309 -
0.003470 -0.012921
y218 0.015266 0.001149 -0.020821 0.003969 -0.008135
0.014157 0.016288 0.007162 0.015330 -0.003957 -0.045601 -
0.008487 -0.004275 -0.019731 -0.015961 -0.011203 -0.003666
0.014435 -0.002469 -0.021083 -0.008340 0.019386 0.022998 -
0.009988 0.028802 0.012619 -0.009452 -0.024770 -0.024651
0.002533 0.011644 -0.032364 -0.000359 0.007234 0.018334
0.003845 -0.013741
z218 -0.003126 -0.009972 0.011088 0.004196 -0.027236
0.008878 -0.000979 -0.008738 0.015123 -0.020243 -0.004303 -
0.040183 -0.017529 0.019964 0.001040 -0.008989 -0.002625 -
0.005558 -0.025296 -0.018082 0.011363 -0.003499 0.016488 -
0.001577 -0.000455 -0.010251 0.000026 -0.008833 -0.010723 -

0.040120 -0.005426 -0.006144 0.013532 0.015985 0.022173 -
0.033770 0.044076
x219 -0.012451 -0.007532 -0.018081 0.009425 0.019213
0.006907 0.015019 -0.021010 -0.003973 0.007780 0.003538
0.005399 -0.012923 0.003829 -0.009725 -0.000120 0.008590 -
0.016015 0.000585 -0.031110 0.002588 -0.012414 -0.008015
0.016317 0.011745 0.012738 0.004470 -0.001477 0.008147
0.016578 -0.001489 0.005811 0.029069 0.009569 -0.005558
0.006434 -0.004559
y219 0.017036 0.000665 -0.016448 0.003161 -0.005427
0.015439 0.020271 0.008252 0.019138 -0.003022 -0.041765 -
0.016682 -0.007487 -0.016253 -0.015381 -0.002755 0.003804
0.024288 -0.003633 -0.026928 -0.005476 0.028357 0.024196 -
0.014346 0.033739 0.010381 -0.007956 -0.015294 -0.025182 -
0.003272 0.008397 -0.030256 -0.003636 -0.003293 0.017021
0.007975 -0.020636
z219 -0.003193 -0.010058 0.012981 -0.000377 -0.032697
0.011885 -0.005459 -0.007115 0.006012 -0.011782 -0.006629 -
0.045237 -0.020668 0.016091 -0.005738 -0.005504 0.006030 -
0.000803 -0.016788 -0.020215 0.010454 0.002017 0.016351 -
0.013470 -0.001718 -0.002279 -0.004697 0.011639 -0.009615 -
0.029472 0.000630 -0.009536 0.010136 0.004390 0.015747 -
0.017720 0.021210
x220 -0.000121 -0.004815 -0.011169 0.002951 0.020998
0.010846 -0.006685 -0.052064 -0.039177 0.008294 0.016843
0.001240 -0.008895 -0.030529 -0.006052 -0.001565 -0.002983 -
0.006189 0.011606 -0.022560 -0.019835 -0.025982 -0.003572
0.028323 0.009726 0.010211 0.000757 -0.024381 0.056971
0.018071 0.052303 0.012864 -0.029941 0.040185 0.000166
0.016345 -0.011251
y220 0.029988 0.007386 -0.008540 0.000826 0.006534
0.018986 0.023514 -0.004435 0.025912 0.002197 -0.016385 -
0.016574 -0.006050 -0.031158 -0.024414 0.006471 0.021591
0.065018 -0.035691 -0.043955 -0.034638 0.017500 0.033498
0.008883 0.001599 0.038231 -0.005606 -0.060934 0.023554 -
0.009298 0.048168 0.018380 -0.001357 0.027653 0.021799
0.001287 -0.009574
z220 0.002619 -0.003685 0.011540 -0.003255 -0.021093
0.024703 0.007989 0.016400 0.021340 -0.014298 -0.017715 -
0.025471 -0.033541 0.019191 0.000221 -0.024614 0.008711 -
0.017265 0.009813 -0.006847 0.035028 0.003403 0.006811 -
0.014711 0.006404 -0.002926 0.004323 0.025934 -0.015896 -
0.002433 0.010797 -0.026028 0.017209 0.014698 0.055695 -
0.012786 0.041734
x221 0.002742 -0.005636 -0.005314 0.003563 0.022024
0.008211 0.002903 -0.049726 -0.031751 0.007654 0.019226
0.002886 -0.015167 -0.031512 -0.004893 -0.013916 0.004184 -
0.001827 0.014050 -0.023874 -0.018129 -0.028106 -0.003510
0.017466 0.003225 0.016928 0.007687 -0.008218 0.041614
0.014149 0.055649 0.002449 -0.025884 0.038564 -0.002056
0.030699 -0.001416
y221 0.030503 0.007199 -0.003530 0.001843 0.002959
0.021290 0.024649 0.003454 0.027845 0.000082 -0.021552 -
0.014586 -0.006024 -0.024918 -0.025510 0.005644 0.022791
0.059374 -0.037357 -0.042009 -0.028588 0.023526 0.028520
0.000588 -0.000511 0.033523 -0.002149 -0.053516 0.011360 -
0.011099 0.054953 0.018881 -0.000769 0.019179 0.019102
0.009369 -0.010803
z221 -0.007316 -0.006110 0.006769 -0.004915 -0.027324
0.025148 -0.003195 0.020254 0.014997 -0.005939 -0.023368 -

0.030773	-0.024087	0.022280	-0.002097	-0.023237	0.008612	-
0.029673	0.002383	0.002129	0.026198	0.000546	0.000445	-
0.024336	0.010142	-0.007656	-0.005961	0.022922	-0.011873	-
0.000387	-0.004367	-0.018206	0.029551	0.013196	0.038044	-
0.012894	0.026745					
x222	0.004249	-0.007686	-0.000962	0.004122	0.020375	
0.006401	0.009497	-0.046449	-0.028890	0.007923	0.020547	
0.005021	-0.019414	-0.031474	-0.006548	-0.021162	0.008496	
0.000632	0.013837	-0.024469	-0.016404	-0.027412	-0.004246	
0.007223	-0.002550	0.020134	0.015679	0.003620	0.027240	
0.010221	0.057815	-0.006878	-0.022446	0.035028	-0.001975	
0.043048	0.000937					
y222	0.029881	0.005911	0.000223	0.002088	-0.001527	
0.022042	0.024205	0.009517	0.025530	-0.000696	-0.026837	-
0.012085	-0.006659	-0.019747	-0.027621	0.003431	0.020910	
0.054716	-0.036905	-0.040635	-0.021500	0.030024	0.023231	-
0.005972	0.000802	0.028054	0.001400	-0.044962	0.003350	-
0.010604	0.056394	0.018758	-0.003035	0.008698	0.009648	
0.014212	-0.007466					
z222	-0.017138	-0.011072	0.001650	-0.007188	-0.035954	
0.021804	-0.014321	0.022160	0.004081	0.000891	-0.027982	-
0.030913	-0.016468	0.027094	-0.007765	-0.022133	0.004442	-
0.037438	-0.004860	0.006572	0.023158	0.003052	-0.005318	-
0.024475	0.017035	-0.010994	-0.008803	0.020220	-0.006383	
0.006344	-0.020847	-0.013560	0.030588	0.007739	0.016011	-
0.014854	0.014219					
x223	0.005992	-0.009509	0.002131	0.003212	0.017630	
0.005623	0.015913	-0.044358	-0.026237	0.006287	0.020753	
0.006204	-0.024017	-0.030448	-0.007960	-0.025976	0.009922	-
0.000331	0.011869	-0.024682	-0.014553	-0.027069	-0.002384	
0.001951	-0.007914	0.022463	0.024105	0.009438	0.016504	
0.005221	0.057274	-0.014819	-0.019113	0.028831	-0.000769	
0.046418	0.005752					
y223	0.030471	0.005442	0.002140	-0.000349	-0.006169	
0.022411	0.023768	0.012790	0.024007	-0.003754	-0.029981	-
0.009227	-0.006285	-0.015639	-0.027307	0.000775	0.018020	
0.047716	-0.034865	-0.039374	-0.015236	0.033525	0.022792	-
0.007261	-0.001669	0.023508	0.004291	-0.037323	-0.006309	-
0.011715	0.054832	0.015752	-0.001752	0.001460	0.007478	
0.009286	-0.008275					
z223	-0.023013	-0.013628	-0.003332	-0.012626	-0.044095	
0.019915	-0.022753	0.020733	-0.002770	0.003386	-0.026475	-
0.028892	-0.009427	0.031137	-0.012618	-0.023433	0.001971	-
0.049047	-0.009850	0.010790	0.019488	0.000537	-0.004021	-
0.018398	0.014360	-0.013308	-0.007296	0.015034	-0.004263	
0.006557	-0.034300	-0.011753	0.033459	0.000709	0.007476	-
0.029490	0.003130					
x224	0.007271	-0.011221	0.004267	0.002226	0.014932	
0.005586	0.019950	-0.042000	-0.023653	0.006245	0.020643	
0.006220	-0.026766	-0.030120	-0.008315	-0.028481	0.010330	-
0.000828	0.009823	-0.024878	-0.014043	-0.026187	0.001094	-
0.004195	-0.008650	0.023016	0.030265	0.014785	0.006729	
0.002076	0.056117	-0.019455	-0.016124	0.023969	-0.000173	
0.052716	0.010729					
y224	0.031333	0.006012	0.003532	-0.002113	-0.009021	
0.021752	0.022170	0.015634	0.021820	-0.004967	-0.031384	-
0.009875	-0.005817	-0.012945	-0.025060	-0.001538	0.014715	
0.041843	-0.034324	-0.036022	-0.010368	0.035197	0.024648	-
0.008929	0.000864	0.017351	0.008093	-0.027115	-0.013218	-

0.011191	0.055472	0.014703	-0.002784	-0.004576	0.007658		
0.010430	-0.010116						
z224	-0.027200	-0.015770	-0.007454	-0.015026	-0.048280		
0.016628	-0.031277	0.018943	-0.006331	0.007816	-0.022583	-	
0.029881	-0.003491	0.032032	-0.013700	-0.026272	0.000752	-	
0.055146	-0.015075	0.014791	0.013224	-0.002129	-0.004088	-	
0.019320	0.016713	-0.016435	-0.005610	0.015798	-0.004123		
0.007777	-0.038956	-0.008060	0.033352	-0.005874	0.002028	-	
0.029972	-0.003396						
x225	0.008090	-0.012867	0.005600	0.000289	0.012123		
0.008030	0.023463	-0.041049	-0.022098	0.007881	0.020136		
0.006692	-0.027342	-0.030204	-0.009428	-0.030834	0.010188	-	
0.002049	0.008708	-0.023627	-0.014736	-0.023687	0.007115	-	
0.009947	-0.010074	0.023805	0.031754	0.018249	-0.000709	-	
0.000073	0.056813	-0.022084	-0.009368	0.021117	0.000410		
0.054414	0.012511						
y225	0.031697	0.006016	0.003506	-0.004039	-0.010973		
0.021736	0.022926	0.016501	0.019073	-0.005922	-0.032580	-	
0.011236	-0.003984	-0.012081	-0.022781	-0.003882	0.011981		
0.036968	-0.031099	-0.031254	-0.006304	0.037632	0.026823	-	
0.012074	-0.000946	0.015943	0.007218	-0.020496	-0.018279	-	
0.010708	0.056450	0.014415	0.000581	-0.004111	0.008597		
0.007788	-0.013829						
z225	-0.030654	-0.017741	-0.010724	-0.017195	-0.050488		
0.014064	-0.035519	0.015856	-0.007447	0.011727	-0.017183	-	
0.030007	0.002074	0.029686	-0.014924	-0.031695	0.001896	-	
0.056491	-0.015583	0.018788	0.006338	-0.003156	-0.004605	-	
0.022956	0.011637	-0.013657	-0.006842	0.015309	-0.004808		
0.008379	-0.038266	-0.004486	0.036297	-0.006813	-0.001233	-	
0.029424	-0.008226						
x226	0.007459	-0.015418	0.006000	0.000197	0.007194		
0.007854	0.022946	-0.039255	-0.021934	0.006719	0.012824		
0.005047	-0.029936	-0.028477	-0.009249	-0.023729	0.003989	-	
0.002871	0.002287	-0.023417	-0.013836	-0.017725	0.011767	-	
0.009606	-0.007514	0.022204	0.036242	0.013432	-0.004668	-	
0.000204	0.054147	-0.025893	-0.007594	0.019598	-0.003518		
0.056213	0.018665						
y226	0.031073	0.005089	0.003383	-0.005212	-0.012818		
0.019672	0.021133	0.016652	0.015157	-0.008044	-0.036502	-	
0.012988	-0.003925	-0.010356	-0.020366	-0.001879	0.005680		
0.034849	-0.029792	-0.026372	-0.001661	0.040991	0.027483	-	
0.011329	-0.001104	0.015689	0.008095	-0.019353	-0.019272	-	
0.008347	0.051375	0.008659	-0.000356	-0.001932	0.002500		
0.006896	-0.010792						
z226	-0.034027	-0.020374	-0.011655	-0.016488	-0.052546		
0.005837	-0.042398	0.013214	-0.007278	0.011273	-0.016552	-	
0.030526	0.003329	0.028992	-0.016158	-0.029108	-0.000705	-	
0.052295	-0.020886	0.019978	0.003520	-0.000553	-0.008147	-	
0.016672	0.007823	-0.007780	-0.001884	0.008596	-0.004721		
0.010809	-0.036702	-0.009197	0.028937	-0.005320	-0.007872	-	
0.025336	-0.004545						
x227	0.006805	-0.017924	0.006462	-0.001287	0.001763		
0.010177	0.020169	-0.036635	-0.021863	0.007065	0.007216		
0.002904	-0.028829	-0.028352	-0.009759	-0.017233	-0.004044	-	
0.004214	-0.003154	-0.022339	-0.016034	-0.012429	0.017711	-	
0.011149	-0.001717	0.015182	0.038023	0.008622	-0.006319	-	
0.001109	0.049823	-0.022266	-0.004058	0.016165	-0.003602		
0.054749	0.023479						
y227	0.031003	0.005126	0.003650	-0.007226	-0.015379		
0.018682	0.017608	0.018149	0.011383	-0.009225	-0.037696	-	

0.019059	0.001007	-0.011065	-0.017261	-0.001981	-0.001729	
0.032064	-0.028811	-0.021509	0.001667	0.040247	0.029026	-
0.009849	0.001937	0.008943	0.005737	-0.011621	-0.018374	-
0.008477	0.043700	0.011260	0.000025	-0.001962	0.002682	
0.003240	-0.008726					
z227	-0.034882	-0.022561	-0.011484	-0.015374	-0.052896	-
0.000984	-0.048640	0.010409	-0.004147	0.010814	-0.010645	-
0.032649	0.008044	0.023872	-0.016520	-0.032065	-0.002548	-
0.045269	-0.023026	0.018986	-0.001592	-0.000189	-0.012378	-
0.015994	0.004283	-0.005982	0.001380	0.008506	-0.006497	
0.010593	-0.032616	-0.007232	0.023144	-0.009185	-0.005542	-
0.019759	0.001705					
x228	0.005805	-0.020284	0.004981	-0.003153	-0.002873	
0.014324	0.016550	-0.034974	-0.022031	0.008175	0.000827	
0.000978	-0.026635	-0.027642	-0.011418	-0.010300	-0.012018	-
0.006710	-0.008720	-0.020031	-0.019117	-0.006408	0.023054	-
0.013177	0.004831	0.009163	0.037269	-0.000366	-0.008271	-
0.002416	0.045469	-0.018604	0.003317	0.008001	-0.004041	
0.049664	0.023831					
y228	0.030922	0.005259	0.001495	-0.008979	-0.015779	
0.017733	0.015832	0.016672	0.008053	-0.010536	-0.038995	-
0.024396	0.005996	-0.012370	-0.014005	-0.003523	-0.007998	
0.030375	-0.025903	-0.015751	0.004929	0.039828	0.029463	-
0.009512	0.004726	0.006758	0.001579	-0.009946	-0.017580	-
0.006606	0.036684	0.009694	0.003348	-0.005519	0.004525	-
0.003192	-0.011537					
z228	-0.034178	-0.024055	-0.011686	-0.013627	-0.049928	-
0.008344	-0.052204	0.005635	0.001643	0.009651	-0.002892	-
0.033430	0.010894	0.018202	-0.017090	-0.037898	-0.002830	-
0.035490	-0.023237	0.017738	-0.006250	0.000595	-0.018339	-
0.017607	-0.001844	0.001132	0.006057	0.005990	-0.011755	
0.009309	-0.023546	-0.010006	0.017991	-0.018169	0.000125	-
0.012311	0.006998					
x229	0.004760	-0.021659	0.003743	-0.005962	-0.008004	
0.019543	0.011064	-0.032432	-0.023337	0.011759	-0.004481	
0.001486	-0.024042	-0.026526	-0.016052	-0.002647	-0.018454	-
0.009694	-0.013997	-0.015807	-0.022688	-0.001023	0.029350	-
0.010249	0.009511	0.002608	0.030993	-0.011427	-0.007251	-
0.004915	0.043197	-0.016057	0.007244	0.002991	-0.008487	
0.048341	0.022708					
y229	0.031874	0.006208	-0.000692	-0.011542	-0.016385	
0.017172	0.013452	0.015563	0.003254	-0.009677	-0.037994	-
0.028786	0.012254	-0.013764	-0.010963	-0.007139	-0.012350	
0.029248	-0.023262	-0.007938	0.008154	0.036450	0.030582	-
0.005044	0.002635	0.005753	-0.007138	-0.004798	-0.014706	-
0.010757	0.029921	0.006687	0.003371	-0.000519	0.001220	-
0.007516	-0.014436					
z229	-0.031371	-0.024382	-0.009359	-0.012787	-0.045604	-
0.015895	-0.054750	0.000390	0.009445	0.009403	0.009338	-
0.031335	0.013819	0.011642	-0.018829	-0.046399	0.001396	-
0.021596	-0.021915	0.017075	-0.010228	-0.000032	-0.022892	-
0.015585	-0.014921	0.011599	0.009544	0.005892	-0.017985	
0.004765	-0.008588	-0.014833	0.007973	-0.018858	0.004681	-
0.001623	0.015417					
x230	0.006844	-0.020233	0.005877	-0.009908	-0.010662	
0.022892	0.006775	-0.036045	-0.023899	0.011343	-0.007844	-
0.004978	-0.018722	-0.028229	-0.008438	-0.003477	-0.021223	-
0.013953	-0.016337	-0.013585	-0.029595	-0.003930	0.031459	-
0.014188	0.005913	-0.004399	0.024442	-0.010172	-0.010882	-

0.005492	0.040117	-0.010417	-0.002993	0.001639	0.002633	
0.042315	0.010287					
y230	0.031840	0.006768	-0.002521	-0.014719	-0.018436	
0.016411	0.010855	0.011986	0.000976	-0.008965	-0.034122	-
0.035051	0.020248	-0.014749	-0.008629	-0.014623	-0.014470	
0.029144	-0.024649	-0.002563	0.008628	0.036397	0.027877	-
0.006325	-0.004604	0.000934	-0.007359	-0.002472	-0.015886	-
0.007147	0.026488	0.007982	0.004573	0.001306	-0.002871	-
0.007294	-0.013044					
z230	-0.032239	-0.028626	-0.011953	-0.012001	-0.044128	-
0.021784	-0.052384	0.003533	0.015815	0.008217	0.015091	-
0.023468	0.011159	0.010721	-0.025612	-0.042667	0.003736	-
0.008446	-0.024509	0.014682	-0.010295	0.003910	-0.024067	-
0.015359	-0.015759	0.011953	0.018457	0.002301	-0.013843	-
0.002987	-0.011535	-0.009032	0.006517	-0.021453	-0.003311	-
0.012333	0.020901					
x231	0.008235	-0.018453	0.007663	-0.012877	-0.013613	
0.024392	0.002304	-0.040576	-0.024485	0.012510	-0.010608	-
0.008462	-0.016575	-0.030390	-0.002264	-0.003461	-0.024288	-
0.017683	-0.021668	-0.011461	-0.033825	-0.006978	0.033257	-
0.014828	0.001260	-0.008526	0.014943	-0.010767	-0.013235	-
0.009020	0.040688	-0.006773	-0.010100	0.000981	0.006867	
0.036522	0.002146					
y231	0.031309	0.007369	-0.005355	-0.015919	-0.021643	
0.013442	0.007519	0.007173	-0.001659	-0.007720	-0.029884	-
0.037993	0.027029	-0.016889	-0.008531	-0.022509	-0.016090	
0.029307	-0.028311	0.000733	0.011976	0.034363	0.024091	-
0.005906	-0.012849	-0.001050	-0.010965	-0.000353	-0.014712	-
0.011437	0.027188	0.011373	0.005611	0.006566	-0.011237	-
0.009808	-0.008998					
z231	-0.033498	-0.032116	-0.014185	-0.010827	-0.042964	-
0.027793	-0.051216	0.005304	0.020778	0.007408	0.020347	-
0.014949	0.008074	0.009288	-0.031401	-0.038389	0.005736	
0.002983	-0.028110	0.011067	-0.010082	0.005414	-0.023689	-
0.012737	-0.017751	0.012911	0.023348	-0.001994	-0.008698	-
0.012604	-0.011999	-0.003187	0.005217	-0.022217	-0.014056	-
0.022191	0.024516					
x232	0.009161	-0.017917	0.007622	-0.015032	-0.014917	
0.025556	-0.002033	-0.043493	-0.022924	0.013415	-0.012429	-
0.012972	-0.014938	-0.030336	0.005034	-0.004083	-0.030470	-
0.020552	-0.023529	-0.009519	-0.040131	-0.008806	0.033692	-
0.019923	0.000760	-0.013502	0.008793	-0.008857	-0.017521	-
0.010551	0.039120	0.000321	-0.013642	-0.005752	0.008444	
0.032618	-0.004492					
y232	0.028831	0.007766	-0.009315	-0.016794	-0.023632	
0.010062	0.004258	0.003992	-0.001935	-0.008086	-0.026019	-
0.042257	0.029986	-0.014563	-0.005682	-0.029133	-0.018117	
0.029055	-0.027891	0.002162	0.013893	0.031401	0.019380	-
0.009275	-0.015981	-0.002297	-0.010315	0.005211	-0.016682	-
0.013934	0.024851	0.014656	0.008757	0.004463	-0.020021	-
0.010597	-0.005133					
z232	-0.035457	-0.035154	-0.016577	-0.008944	-0.041091	-
0.032386	-0.050253	0.006899	0.026376	0.005766	0.024681	-
0.007344	0.004495	0.008620	-0.033350	-0.035139	0.006469	
0.012178	-0.028748	0.006361	-0.011408	0.005485	-0.023279	-
0.015061	-0.015272	0.013782	0.026312	-0.002344	-0.006053	-
0.020266	-0.013843	0.001287	0.007278	-0.028636	-0.020224	-
0.027640	0.026517					
x233	0.009741	-0.017205	0.008759	-0.019588	-0.016050	
0.024657	-0.004881	-0.043730	-0.019955	0.016302	-0.015290	-

0.013162	-0.013265	-0.028450	0.008227	-0.004053	-0.033377	-
0.023595	-0.025487	-0.003725	-0.046463	-0.006912	0.037493	-
0.015582	-0.005462	-0.013638	0.002890	-0.010726	-0.018060	-
0.007663	0.039723	0.004307	-0.024778	0.000647	0.010321	
0.021821	-0.009887					
y233	0.025400	0.008868	-0.013538	-0.020161	-0.025445	
0.004340	0.002953	0.002152	-0.001830	-0.006491	-0.021270	-
0.041693	0.032732	-0.012100	-0.004081	-0.036364	-0.015684	
0.027633	-0.026455	0.003417	0.013517	0.028583	0.016536	-
0.007042	-0.025821	0.000634	-0.011659	0.005875	-0.014701	-
0.013012	0.024738	0.016959	0.004687	0.011817	-0.024794	-
0.017931	-0.003573					
z233	-0.037956	-0.037072	-0.016583	-0.008543	-0.039822	-
0.037083	-0.049263	0.008412	0.030705	0.005281	0.027176	
0.002857	0.001672	0.007964	-0.035878	-0.031342	0.010283	
0.019705	-0.029366	0.002457	-0.013013	0.004073	-0.018852	-
0.009280	-0.016518	0.017465	0.026401	-0.005726	-0.001116	-
0.022487	-0.014368	0.002078	0.004053	-0.026919	-0.025298	-
0.032477	0.024893					
x234	0.010301	-0.017123	0.009242	-0.021280	-0.016844	
0.025168	-0.007388	-0.045923	-0.018126	0.015548	-0.018897	-
0.016468	-0.012549	-0.030787	0.016397	-0.005003	-0.038185	-
0.025415	-0.026744	0.000989	-0.054660	-0.006254	0.036453	-
0.017517	-0.005733	-0.019032	0.001010	-0.011905	-0.018305	-
0.007686	0.035555	0.011795	-0.029558	-0.004754	0.011095	
0.011386	-0.011589					
y234	0.021312	0.009299	-0.017689	-0.019965	-0.027191	-
0.001938	0.000142	-0.001125	-0.002368	-0.009406	-0.017901	-
0.042621	0.035521	-0.012244	-0.000679	-0.042734	-0.016815	
0.025921	-0.024259	0.001492	0.014302	0.023508	0.009677	-
0.009025	-0.028540	-0.000122	-0.008327	0.007619	-0.012390	-
0.012924	0.019454	0.021335	0.002385	0.009981	-0.026842	-
0.023404	-0.001258					
z234	-0.040667	-0.038610	-0.016606	-0.006118	-0.038292	-
0.039782	-0.048124	0.008077	0.033415	0.002483	0.028884	
0.010959	-0.001694	0.004065	-0.034020	-0.028569	0.012677	
0.024617	-0.028957	-0.003140	-0.016089	-0.000461	-0.016347	-
0.009509	-0.013013	0.017998	0.025819	-0.007285	0.003407	-
0.026430	-0.017615	0.003678	0.005791	-0.033950	-0.024506	-
0.033187	0.021937					
x235	0.010321	-0.017205	0.009401	-0.022939	-0.016801	
0.022483	-0.010074	-0.046303	-0.014844	0.017472	-0.020170	-
0.016591	-0.013675	-0.029030	0.018861	-0.005033	-0.042061	-
0.026511	-0.030385	0.008453	-0.057753	-0.004759	0.037194	-
0.011850	-0.009706	-0.019137	-0.001210	-0.016468	-0.016498	-
0.004016	0.033890	0.016707	-0.038545	-0.002161	0.011585	
0.002792	-0.009555					
y235	0.016207	0.009900	-0.022391	-0.019013	-0.028476	-
0.011183	-0.003361	-0.004121	-0.002237	-0.010280	-0.011437	-
0.038793	0.036126	-0.011691	-0.001258	-0.049130	-0.015566	
0.024144	-0.022988	-0.001161	0.017959	0.016269	0.004540	-
0.006823	-0.032723	0.003545	-0.006792	0.005714	-0.007544	-
0.008766	0.016069	0.024053	-0.002063	0.013605	-0.023781	-
0.026548	0.000655					
z235	-0.043961	-0.039110	-0.015292	-0.003992	-0.036965	-
0.042192	-0.047602	0.007530	0.035373	0.000191	0.029904	
0.019964	-0.006004	0.000853	-0.032660	-0.024516	0.016647	
0.027813	-0.028801	-0.008454	-0.017034	-0.007081	-0.010840	-
0.005350	-0.010714	0.021130	0.022684	-0.010202	0.008647	-

0.027323 -0.019189 0.002153 0.006549 -0.036654 -0.021669 -
0.030595 0.017505
x236 0.010451 -0.017271 0.009329 -0.024371 -0.016470
0.022146 -0.013850 -0.045151 -0.009918 0.019070 -0.020201 -
0.015888 -0.015640 -0.029739 0.020978 -0.003974 -0.045690 -
0.025984 -0.029083 0.016793 -0.059522 0.000653 0.041437 -
0.006423 -0.014545 -0.014005 -0.000422 -0.020516 -0.018161
0.002767 0.030463 0.021173 -0.040491 0.000665 0.007776 -
0.001905 -0.004451
y236 0.010375 0.010887 -0.027221 -0.018048 -0.029163 -
0.018599 -0.008463 -0.005696 -0.000353 -0.012341 -0.002821 -
0.033785 0.035143 -0.011336 -0.003473 -0.052670 -0.013353
0.021664 -0.016394 -0.004251 0.023517 0.010566 0.002137 -
0.006765 -0.037184 0.013860 -0.001811 0.005384 -0.005748 -
0.003521 0.012177 0.024508 -0.001604 0.015060 -0.018306 -
0.024630 0.003156
z236 -0.047164 -0.038585 -0.013276 -0.001134 -0.035699 -
0.042563 -0.048196 0.006815 0.037443 -0.002852 0.030189
0.028626 -0.011301 -0.003857 -0.028906 -0.020521 0.020201
0.029079 -0.025710 -0.013670 -0.018461 -0.014685 -0.002558 -
0.003376 -0.006796 0.026628 0.017013 -0.010797 0.011794 -
0.027036 -0.020366 -0.001608 0.009620 -0.039102 -0.015063 -
0.022963 0.011965
x237 0.010460 -0.018236 0.007546 -0.024978 -0.016251
0.020038 -0.015886 -0.044383 -0.004916 0.019964 -0.022818 -
0.015411 -0.020252 -0.031984 0.023472 -0.002915 -0.050851 -
0.025059 -0.031411 0.023429 -0.062630 0.002869 0.039996
0.000408 -0.014108 -0.013563 0.001434 -0.024716 -0.017144
0.002520 0.027583 0.025075 -0.039649 -0.002564 0.002258 -
0.003494 0.000682
y237 0.002991 0.011161 -0.034226 -0.015460 -0.030438 -
0.029556 -0.012157 -0.008181 0.001976 -0.016246 0.002672 -
0.028305 0.031653 -0.010830 -0.004860 -0.055731 -0.010903
0.018660 -0.012466 -0.013317 0.028881 -0.000157 -0.007118 -
0.004367 -0.035725 0.018863 0.004233 0.006411 -0.002965 -
0.004128 0.009106 0.024224 0.000278 0.010236 -0.010604 -
0.019374 0.002977
z237 -0.051003 -0.037679 -0.010757 0.002281 -0.034699 -
0.042740 -0.048245 0.005102 0.037948 -0.007046 0.028308
0.037449 -0.016368 -0.009869 -0.023707 -0.017346 0.023947
0.028596 -0.023050 -0.020604 -0.019933 -0.026069 0.004707 -
0.001338 -0.000621 0.030192 0.009208 -0.011542 0.016050 -
0.025908 -0.022709 -0.006124 0.014620 -0.045754 -0.005494 -
0.012626 0.004534
x238 0.009936 -0.018428 0.007707 -0.027687 -0.016029
0.020044 -0.017901 -0.041934 0.002713 0.020898 -0.024844 -
0.011283 -0.025008 -0.031768 0.025798 -0.001913 -0.053342 -
0.023177 -0.032307 0.032758 -0.064492 0.003874 0.042745
0.005472 -0.017900 -0.008487 0.001523 -0.031651 -0.016515
0.004128 0.023144 0.026159 -0.034463 -0.000470 -0.000815 -
0.003362 0.003582
y238 -0.004906 0.012457 -0.038358 -0.014661 -0.032195 -
0.037787 -0.015491 -0.009548 0.006308 -0.020833 0.010047 -
0.018946 0.027637 -0.009467 -0.006227 -0.058442 -0.004742
0.015059 -0.006018 -0.021683 0.034594 -0.014437 -0.012234 -
0.005570 -0.040162 0.031257 0.007261 0.004570 -0.000680 -
0.002472 0.001741 0.020986 0.004050 0.011575 0.003022 -
0.012907 -0.002959
z238 -0.054833 -0.035922 -0.006857 0.005746 -0.033616 -
0.041685 -0.048571 0.002943 0.037664 -0.010448 0.026619

0.047092	-0.021461	-0.017176	-0.017719	-0.015523	0.026760	
0.026143	-0.019159	-0.027404	-0.021878	-0.039867	0.014619	-
0.001079	0.005556	0.036654	-0.001157	-0.010588	0.020340	-
0.021123	-0.025374	-0.012015	0.019663	-0.051340	0.005792	
0.001392	-0.005021					
x239	0.005575	-0.017647	0.005024	-0.027214	-0.015159	
0.014001	-0.019919	-0.040321	0.005496	0.016422	-0.018588	-
0.004452	-0.029007	-0.029259	0.024911	-0.007367	-0.053160	-
0.021343	-0.032327	0.031235	-0.060921	0.001103	0.040075	
0.005909	-0.018701	-0.003560	0.005294	-0.032640	-0.015327	
0.003027	0.020971	0.029247	-0.017989	-0.012450	0.001966	-
0.005309	0.011431					
y239	-0.008273	0.010397	-0.043287	-0.013591	-0.028350	-
0.041177	-0.017129	-0.015042	0.008327	-0.023298	0.015546	-
0.013891	0.021386	-0.012898	-0.005379	-0.054269	-0.001101	
0.011765	0.002344	-0.024771	0.038869	-0.015036	-0.011061	-
0.011470	-0.033962	0.028468	0.007073	0.001216	0.004947	-
0.010068	0.001243	0.018695	0.005847	0.012607	0.007844	-
0.015220	-0.002997					
z239	-0.053287	-0.040862	-0.003909	0.004006	-0.026947	-
0.036766	-0.044567	0.002161	0.035362	-0.007425	0.019888	
0.048870	-0.022186	-0.023865	-0.008303	-0.006980	0.026633	
0.031717	-0.020011	-0.023279	-0.021933	-0.031186	0.024556	-
0.003692	0.017769	0.033577	-0.006405	-0.011831	0.011341	-
0.025763	-0.023671	-0.013920	0.017449	-0.045443	0.010437	
0.009522	0.000658					
x240	0.001031	-0.016880	0.005083	-0.029218	-0.016295	
0.007397	-0.020520	-0.036301	0.011178	0.014276	-0.014966	
0.003760	-0.028514	-0.028028	0.021640	-0.014974	-0.046756	-
0.019277	-0.031346	0.028673	-0.057184	-0.006573	0.039993	
0.015940	-0.019774	0.000624	0.003072	-0.033585	-0.020137	
0.005367	0.015417	0.030118	-0.010094	-0.019688	0.005287	-
0.001683	0.007566					
y240	-0.010962	0.008649	-0.044513	-0.014534	-0.026960	-
0.043985	-0.018395	-0.016888	0.011630	-0.023784	0.016482	-
0.008657	0.017352	-0.017743	-0.004560	-0.052485	0.006241	
0.008440	0.007790	-0.025544	0.042174	-0.020664	-0.008519	-
0.007282	-0.030732	0.028422	0.001164	-0.004382	0.004848	-
0.012210	-0.003780	0.014491	0.002470	0.016588	0.010137	-
0.010932	-0.014021					
z240	-0.051280	-0.045567	-0.001526	0.002648	-0.020902	-
0.031308	-0.039500	0.000807	0.032408	-0.003837	0.013188	
0.049725	-0.021361	-0.026946	0.000151	-0.000787	0.025489	
0.036212	-0.019847	-0.020400	-0.021455	-0.024104	0.030505	-
0.007849	0.027690	0.029691	-0.011690	-0.010916	0.003143	-
0.027761	-0.025034	-0.013690	0.017029	-0.043753	0.010182	
0.014458	0.007118					
x241	-0.003732	-0.017555	0.002287	-0.028597	-0.016263	
0.001225	-0.022143	-0.032901	0.017202	0.010081	-0.012238	
0.008777	-0.031073	-0.027031	0.024052	-0.018899	-0.045005	-
0.016963	-0.029179	0.023245	-0.053324	-0.009171	0.035091	
0.017930	-0.010841	0.002565	0.002323	-0.026787	-0.022881	
0.003886	0.012562	0.026703	0.001685	-0.025348	0.008129	
0.007464	0.014023					
y241	-0.014586	0.005560	-0.048639	-0.014077	-0.024264	-
0.046715	-0.020321	-0.018950	0.014085	-0.025628	0.017657	-
0.006435	0.012998	-0.021305	-0.000834	-0.048022	0.010765	
0.006462	0.011524	-0.026939	0.045945	-0.022806	-0.010351	-
0.007398	-0.020557	0.024819	-0.000858	-0.007206	0.004851	-

0.019073   -0.004090   0.010085   0.007071   0.017471   0.010673   -
0.003841   -0.014434
z241   -0.048706   -0.048598   0.001652   0.002283   -0.016128   -
0.026848   -0.035288   -0.000626   0.028747   -0.000861   0.008572
0.050116   -0.019431   -0.028742   0.005397   0.006614   0.024317
0.040072   -0.018472   -0.016337   -0.019491   -0.017209   0.038869   -
0.009343   0.035581   0.024914   -0.016603   -0.008624   -0.003355   -
0.025233   -0.022891   -0.012543   0.013477   -0.039580   0.011756
0.015899   0.012159
x242   -0.007875   -0.018223   0.001448   -0.027787   -0.017012   -
0.003770   -0.022268   -0.032012   0.019877   0.006261   -0.007972
0.014244   -0.035805   -0.028404   0.027011   -0.021422   -0.043858   -
0.012210   -0.026434   0.019825   -0.049672   -0.009835   0.032978
0.016208   -0.005577   0.007369   0.001767   -0.027951   -0.022422
0.003127   0.009885   0.024151   0.005027   -0.026616   0.013262
0.010978   0.013704
y242   -0.017126   0.001972   -0.050958   -0.013833   -0.022908   -
0.047753   -0.022831   -0.020406   0.015238   -0.026594   0.018193   -
0.003813   0.007916   -0.025984   0.003451   -0.043156   0.014377
0.006820   0.016322   -0.025585   0.047991   -0.022573   -0.009821   -
0.008719   -0.016067   0.025106   -0.004375   -0.016945   0.005418   -
0.022441   -0.006762   0.006631   0.006113   0.021922   0.011741
0.001181   -0.019646
z242   -0.045951   -0.049904   0.003912   0.002382   -0.011750   -
0.022081   -0.031492   -0.001803   0.024085   0.002585   0.004661
0.048980   -0.016586   -0.027090   0.008012   0.013881   0.023474
0.039981   -0.018088   -0.010762   -0.015889   -0.013512   0.045094   -
0.008056   0.041527   0.019864   -0.021533   -0.003048   -0.008761   -
0.021736   -0.019493   -0.012594   0.012040   -0.038377   0.010290
0.017951   0.016627
x243   -0.012271   -0.018801   0.000195   -0.027737   -0.016808   -
0.011327   -0.022237   -0.031190   0.024844   0.004180   -0.003225
0.020162   -0.035396   -0.027933   0.026085   -0.022114   -0.037551   -
0.009877   -0.021670   0.013615   -0.044642   -0.010639   0.031330
0.016471   -0.000509   0.008801   0.003925   -0.023269   -0.017907
0.002909   0.004796   0.022251   0.002340   -0.024475   0.018386
0.012455   0.016230
y243   -0.020435   -0.001875   -0.053901   -0.014098   -0.020785   -
0.050748   -0.025616   -0.021827   0.017344   -0.025721   0.018983   -
0.001048   0.007038   -0.028168   0.004784   -0.036926   0.020923
0.007047   0.019622   -0.024491   0.051465   -0.021436   -0.009535   -
0.006679   -0.009863   0.021156   -0.004107   -0.024471   0.007270   -
0.025359   -0.007310   0.004168   0.005827   0.026525   0.010942
0.004808   -0.020137
z243   -0.042439   -0.050156   0.005539   0.003801   -0.007772   -
0.016583   -0.026640   -0.003339   0.017451   0.005614   0.002036
0.046054   -0.014457   -0.023690   0.010529   0.019051   0.020960
0.039207   -0.016728   -0.004539   -0.011577   -0.011690   0.049352   -
0.005734   0.045985   0.014994   -0.028154   0.002001   -0.015135   -
0.016937   -0.013959   -0.013209   0.013269   -0.038849   0.007570
0.020881   0.018136
x244   -0.015969   -0.019240   -0.001587   -0.026865   -0.017358   -
0.015235   -0.021415   -0.030152   0.028612   0.003749   0.002432
0.025864   -0.035193   -0.028615   0.026013   -0.025219   -0.031744   -
0.008069   -0.013799   0.011601   -0.042993   -0.011114   0.031200
0.013616   0.006024   0.012597   0.000552   -0.017785   -0.013513
0.003558   -0.000883   0.017236   0.003467   -0.020029   0.020365
0.017077   0.013306
y244   -0.023167   -0.006375   -0.057752   -0.013654   -0.019522   -
0.051517   -0.029135   -0.021781   0.018609   -0.023562   0.018769

0.001171	0.005764	-0.030554	0.006989	-0.031520	0.025339		
0.007729	0.023338	-0.019686	0.052814	-0.019798	-0.008660	-	
0.005239	-0.005840	0.020386	-0.006821	-0.034645	0.007156	-	
0.025550	-0.008916	-0.000550	0.008992	0.031505	0.006868		
0.012554	-0.023838						
z244	-0.039019	-0.049803	0.007840	0.005047	-0.004769	-	
0.012861	-0.023078	-0.005271	0.010896	0.007426	0.000136		
0.041903	-0.010971	-0.016923	0.011352	0.026160	0.018679		
0.038846	-0.016247	-0.000251	-0.005044	-0.010680	0.051877	-	
0.000019	0.048928	0.009572	-0.030326	0.008329	-0.018905	-	
0.012065	-0.006941	-0.010021	0.010377	-0.039880	0.006841		
0.017546	0.017509						
x245	-0.021371	-0.021599	-0.005322	-0.024305	-0.018147	-	
0.022657	-0.021080	-0.032062	0.030147	0.001511	0.006694		
0.029563	-0.035434	-0.030715	0.027334	-0.023004	-0.024401	-	
0.003447	-0.005408	0.005926	-0.037672	-0.009177	0.028981		
0.012316	0.015040	0.013884	0.006166	-0.017146	-0.007635		
0.003625	-0.006046	0.015717	-0.000159	-0.017906	0.028544		
0.019318	0.008842						
y245	-0.026939	-0.011834	-0.062337	-0.013364	-0.018087	-	
0.053692	-0.032568	-0.022678	0.019010	-0.021530	0.019215		
0.002533	0.006469	-0.032238	0.009151	-0.024512	0.032442		
0.010503	0.026090	-0.015388	0.055710	-0.016964	-0.009621	-	
0.000186	0.001063	0.014936	-0.005276	-0.045164	0.006846	-	
0.027107	-0.005585	-0.003684	0.014288	0.032791	0.003084		
0.017386	-0.024710						
z245	-0.034120	-0.046868	0.010473	0.006326	-0.001783	-	
0.007018	-0.018092	-0.005916	0.003083	0.010419	-0.000571		
0.036589	-0.006206	-0.006744	0.008487	0.030057	0.015392		
0.034553	-0.014723	0.007851	0.002764	-0.011987	0.055287		
0.004897	0.047417	0.002698	-0.038281	0.017626	-0.021543	-	
0.002739	0.000374	-0.008338	0.009609	-0.041978	0.000269		
0.012269	0.018564						
x246	-0.024884	-0.023151	-0.009122	-0.024460	-0.017369	-	
0.028101	-0.018974	-0.033527	0.033548	0.005057	0.014819		
0.035114	-0.032936	-0.030158	0.027141	-0.023702	-0.015498	-	
0.002001	0.005561	0.005025	-0.030553	-0.008400	0.026940		
0.011728	0.020519	0.017348	0.006274	-0.011595	-0.006562		
0.004784	-0.009763	0.014924	-0.005481	-0.017674	0.030019		
0.025684	-0.000792						
y246	-0.030286	-0.017716	-0.067516	-0.013127	-0.016717	-	
0.055041	-0.037039	-0.022329	0.019615	-0.017553	0.018071		
0.004263	0.007528	-0.032116	0.011475	-0.017606	0.037339		
0.013957	0.027864	-0.008056	0.058368	-0.012870	-0.009332		
0.005645	0.004993	0.011983	-0.004395	-0.058661	0.005459	-	
0.026050	-0.003631	-0.007729	0.018613	0.035607	-0.003990		
0.025549	-0.028070						
z246	-0.030450	-0.044214	0.013310	0.010251	-0.000505	-	
0.003529	-0.014494	-0.007403	-0.006046	0.010006	-0.002101		
0.029723	-0.001925	0.004067	0.003974	0.036451	0.009736		
0.029642	-0.014085	0.012160	0.009191	-0.013989	0.057095		
0.010968	0.047821	-0.006154	-0.040460	0.024627	-0.015964		
0.007181	0.006820	-0.004184	0.008184	-0.041869	-0.000120		
0.001310	0.017602						
x247	-0.030870	-0.026048	-0.015338	-0.020270	-0.017531	-	
0.035593	-0.018713	-0.034467	0.034183	0.002317	0.018307		
0.035804	-0.034389	-0.028586	0.025538	-0.018313	-0.009777		
0.004137	0.017895	0.001315	-0.024268	-0.005850	0.027563		
0.010978	0.026404	0.018242	0.008472	-0.010098	0.009851		

0.009816	-0.014270	0.012997	-0.007504	-0.012890	0.037833	
0.023365	-0.014011					
y247	-0.034041	-0.023957	-0.072454	-0.013118	-0.015826	-
0.055922	-0.041520	-0.021228	0.018993	-0.013400	0.017224	
0.004679	0.010477	-0.031745	0.012849	-0.008553	0.043768	
0.017293	0.028946	-0.000295	0.060808	-0.008512	-0.008989	
0.012056	0.008368	0.008348	-0.002885	-0.072204	0.005210	-
0.024853	-0.001122	-0.009229	0.025408	0.038256	-0.009835	
0.033557	-0.029306					
z247	-0.023815	-0.039155	0.017181	0.011569	0.000525	
0.002729	-0.010178	-0.009181	-0.015286	0.014425	0.001024	
0.023538	0.007396	0.016495	-0.001368	0.038456	0.007737	
0.021428	-0.011549	0.020469	0.018735	-0.016483	0.057224	
0.016180	0.046056	-0.014299	-0.044191	0.036281	-0.021843	
0.014336	0.015455	0.001380	0.003808	-0.042685	-0.005627	-
0.005804	0.018494					
x248	-0.033631	-0.027784	-0.019139	-0.018060	-0.011747	-
0.039916	-0.014031	-0.026214	0.038110	0.004153	0.016275	
0.035592	-0.028321	-0.028828	0.028998	-0.009641	-0.008750	
0.007799	0.021479	-0.002175	-0.010610	-0.003098	0.028958	
0.009945	0.034061	0.017756	0.005428	-0.004379	0.016739	
0.028278	-0.008100	0.009047	-0.024629	-0.010438	0.034603	
0.021081	-0.022352					
y248	-0.032789	-0.027000	-0.071005	-0.016399	-0.011522	-
0.051975	-0.033109	-0.007960	0.022236	-0.010060	0.008188	-
0.000476	0.021586	-0.032435	0.012002	0.000145	0.044288	
0.020058	0.020848	0.011159	0.059964	-0.013831	0.001723	
0.014906	0.018356	0.008021	-0.004291	-0.067794	0.010879	-
0.004455	0.021859	-0.009602	0.020119	0.026031	-0.009573	
0.040377	-0.016345					
z248	-0.017241	-0.033653	0.020802	0.016352	0.002432	
0.008323	-0.002570	-0.007431	-0.025488	0.008341	-0.005376	
0.014374	0.012894	0.028769	-0.006815	0.036134	-0.001589	
0.018393	-0.013553	0.023142	0.028719	-0.018071	0.044037	
0.014249	0.038004	-0.017797	-0.038014	0.033415	-0.014680	
0.009354	0.021902	0.012622	-0.008952	-0.044072	-0.001313	
0.000772	0.014736					
x249	-0.036354	-0.029062	-0.021860	-0.017922	-0.005053	-
0.042014	-0.006966	-0.016891	0.040811	0.003840	0.015933	
0.033818	-0.019036	-0.027937	0.030373	0.001187	-0.003441	
0.007627	0.026331	-0.001826	0.000943	-0.002973	0.033198	
0.010290	0.040346	0.014712	0.004447	0.005864	0.021739	
0.042939	0.003512	0.013776	-0.029382	-0.009919	0.035545	
0.012792	-0.028889					
y249	-0.032216	-0.029331	-0.069856	-0.018085	-0.007510	-
0.047113	-0.024862	0.002297	0.023084	-0.007087	0.000677	-
0.005790	0.030412	-0.032813	0.010601	0.008856	0.041423	
0.024350	0.014293	0.021558	0.060229	-0.015715	0.009394	
0.014323	0.024685	0.005402	-0.003967	-0.065054	0.015541	
0.011080	0.038455	-0.011879	0.013601	0.016494	-0.006432	
0.047435	-0.002920					
z249	-0.009412	-0.027747	0.023338	0.023297	0.002001	
0.013033	0.000204	-0.007764	-0.035884	0.004719	-0.013032	
0.006249	0.013423	0.038154	-0.009752	0.030889	-0.014377	
0.016805	-0.015737	0.021453	0.034932	-0.016805	0.028877	
0.010068	0.028359	-0.016561	-0.034176	0.026835	-0.008379	
0.003277	0.021099	0.012484	-0.025613	-0.038401	-0.005866	
0.010389	0.009712					
x250	-0.038707	-0.030187	-0.024841	-0.014934	0.003095	-
0.043078	0.000075	-0.011293	0.042588	0.004910	0.013747	

0.031308	-0.009804	-0.026390	0.032355	0.010590	-0.001843	
0.007370	0.029632	-0.000282	0.011514	-0.002380	0.036159	
0.011848	0.046282	0.008500	0.002392	0.014400	0.024286	
0.053110	0.008506	0.013226	-0.038727	-0.003654	0.035613	
0.009355	-0.027129					
y250	-0.031162	-0.030685	-0.068409	-0.019970	-0.004944	-
0.041011	-0.018210	0.013154	0.025042	-0.003404	-0.004583	-
0.009662	0.036694	-0.032910	0.009505	0.015234	0.037945	
0.028134	0.008465	0.029851	0.058819	-0.018049	0.014427	
0.010326	0.028825	0.001155	-0.004306	-0.059494	0.018822	
0.023217	0.051314	-0.012389	0.006330	0.008130	-0.005269	
0.048346	0.004636					
z250	-0.002024	-0.022287	0.027227	0.027253	-0.000376	
0.015937	0.001605	-0.007324	-0.044679	0.000882	-0.015355	-
0.001021	0.010989	0.045444	-0.012880	0.023714	-0.021045	
0.012813	-0.014796	0.015272	0.037109	-0.017730	0.015613	
0.005836	0.019039	-0.010695	-0.029985	0.020634	-0.002355	-
0.003989	0.021514	0.016176	-0.031239	-0.035208	-0.007837	
0.013278	0.003323					
x251	-0.040178	-0.031440	-0.027525	-0.012428	0.010816	-
0.040587	0.006382	-0.004733	0.044215	0.007328	0.012165	
0.028563	-0.002101	-0.025378	0.034955	0.018559	-0.003497	
0.006722	0.034038	0.001748	0.018217	-0.001557	0.039438	
0.008872	0.047046	0.004351	-0.003462	0.022771	0.023417	
0.060132	0.008483	0.011344	-0.044903	0.005522	0.037028	
0.005942	-0.028312					
y251	-0.030607	-0.030844	-0.066350	-0.019895	-0.002676	-
0.035404	-0.012471	0.022727	0.026794	0.000922	-0.009044	-
0.015082	0.043509	-0.031972	0.008562	0.021491	0.034165	
0.031519	0.003527	0.037136	0.055441	-0.018171	0.013740	
0.004327	0.032107	-0.005917	-0.005894	-0.053449	0.020663	
0.035470	0.059548	-0.012531	-0.005146	0.000762	-0.009366	
0.046301	0.015015					
z251	0.004449	-0.015913	0.032397	0.031826	-0.003040	
0.014716	0.002024	-0.009639	-0.053552	-0.002442	-0.017002	-
0.008260	0.008218	0.052979	-0.016568	0.017330	-0.023377	
0.008383	-0.014875	0.007621	0.037690	-0.020364	0.000528	
0.007129	0.012083	-0.005888	-0.025089	0.013708	0.004827	-
0.009203	0.024082	0.020537	-0.035187	-0.033856	-0.011940	
0.016801	0.001475					
x252	-0.042183	-0.032320	-0.029833	-0.008474	0.018325	-
0.037846	0.012045	-0.001008	0.045511	0.010049	0.012487	
0.024794	0.006214	-0.023606	0.034966	0.025290	-0.003519	
0.006496	0.034698	0.004239	0.024858	-0.003135	0.036312	
0.002725	0.051189	-0.007101	-0.010094	0.032650	0.019631	
0.064385	0.010309	0.010170	-0.050109	0.016519	0.032457	-
0.001475	-0.019630					
y252	-0.029891	-0.030604	-0.064838	-0.019506	-0.000045	-
0.028129	-0.005521	0.032112	0.027700	0.006018	-0.013388	-
0.019952	0.049125	-0.029493	0.010493	0.026101	0.028414	
0.034211	0.003525	0.042151	0.046455	-0.013647	0.013592	
0.000863	0.029020	-0.012851	-0.006277	-0.046058	0.019415	
0.044098	0.059334	-0.014429	-0.015010	-0.004818	-0.003485	
0.044709	0.018553					
z252	0.010948	-0.010615	0.038029	0.033932	-0.006417	
0.013356	0.002448	-0.012269	-0.061721	-0.005608	-0.018432	-
0.014582	0.000628	0.060262	-0.017364	0.008493	-0.024102	
0.000156	-0.011846	-0.002908	0.033574	-0.020835	-0.008220	
0.011996	0.000421	0.005868	-0.018985	0.007105	0.010152	-

0.015332	0.020699	0.020332	-0.031615	-0.035512	-0.006512		
0.019433	-0.004144						
x253	-0.044944	-0.031662	-0.036235	-0.005210	0.028951	-	
0.019310	0.012709	0.006204	0.048276	0.021139	0.016309		
0.013019	0.010087	-0.025500	0.037175	0.034768	-0.011223		
0.005706	0.042246	0.015602	0.029172	-0.013482	0.018216		
0.006523	0.069498	-0.018283	-0.017035	0.031479	0.001022		
0.089210	-0.017535	0.003279	-0.076325	0.049306	0.040470	-	
0.032043	-0.028650						
y253	-0.027840	-0.031991	-0.058280	-0.014975	-0.000970	-	
0.033597	0.004878	0.038889	0.025319	0.005088	-0.019839	-	
0.016802	0.057961	-0.018309	0.013028	0.027276	0.024059		
0.039537	-0.000801	0.040595	0.033462	-0.006531	0.023274	-	
0.018273	-0.000106	-0.017088	-0.008940	-0.021485	0.027600		
0.035282	0.083205	-0.010675	-0.007049	-0.024018	-0.004329		
0.058958	0.043708						
z253	0.018964	-0.006628	0.047718	0.039769	-0.012137	-	
0.000091	0.004848	-0.017347	-0.073783	-0.013440	-0.019519	-	
0.012972	-0.006061	0.074076	-0.019439	-0.002467	-0.022230	-	
0.005588	-0.011673	-0.013961	0.023528	-0.028521	-0.010978		
0.011886	-0.031187	0.023791	-0.012398	0.014183	0.023419	-	
0.028065	0.035858	0.027182	-0.014296	-0.046491	0.003549		
0.030338	0.012301						
x254	-0.046357	-0.033483	-0.036705	0.001613	0.034494	-	
0.020345	0.019934	0.015581	0.047546	0.022117	0.015025		
0.008594	0.024418	-0.019001	0.037746	0.035246	-0.015519		
0.004390	0.043830	0.017751	0.022127	-0.007828	0.019225	-	
0.006149	0.055137	-0.033651	-0.035094	0.042589	-0.007156		
0.078742	-0.006773	0.001960	-0.069181	0.045237	0.031254	-	
0.032968	-0.013792						
y254	-0.027367	-0.028780	-0.057168	-0.015642	0.001420	-	
0.017264	0.008316	0.039674	0.026115	0.015237	-0.019781	-	
0.022228	0.056568	-0.020905	0.015104	0.032788	0.014065		
0.039225	0.000766	0.039490	0.031235	-0.003401	0.013416	-	
0.023924	0.008051	-0.025659	-0.001335	-0.011739	0.023491		
0.045292	0.064425	-0.017525	-0.023455	-0.004232	-0.003409		
0.044012	0.034732						
z254	0.025834	0.001450	0.053506	0.037701	-0.016560		
0.004111	-0.000296	-0.027594	-0.080269	-0.011899	-0.015779	-	
0.017698	-0.023018	0.067115	-0.025713	-0.011750	-0.021309	-	
0.020995	-0.009219	-0.030928	0.025530	-0.029528	-0.021746		
0.027201	-0.023500	0.040838	0.001725	-0.002515	0.028690	-	
0.032395	0.020243	0.018718	-0.004284	-0.028487	0.002019		
0.024644	-0.012022						
x255	-0.049727	-0.030579	-0.038435	0.003778	0.043413	-	
0.020757	0.027536	0.030323	0.041733	0.028878	0.020368	-	
0.000105	0.038879	-0.014383	0.044730	0.028315	-0.015827		
0.006269	0.045226	0.015430	0.015838	-0.005873	0.015939	-	
0.011681	0.050909	-0.052147	-0.042530	0.062569	-0.018349		
0.066892	-0.002501	-0.004955	-0.065905	0.050013	0.027281	-	
0.048935	0.007647						
y255	-0.026546	-0.025445	-0.056018	-0.013868	0.002271	-	
0.001387	0.010650	0.040015	0.026958	0.022299	-0.019634	-	
0.025725	0.054025	-0.023360	0.016922	0.037975	-0.006245		
0.041530	0.006867	0.039867	0.026278	0.001163	0.003215	-	
0.036035	0.011082	-0.034586	-0.001007	-0.008176	0.015494		
0.051257	0.040691	-0.022314	-0.041045	0.011378	-0.003764		
0.037756	0.024446						
z255	0.032938	0.009030	0.060801	0.037286	-0.022954		
0.000606	-0.004571	-0.039544	-0.087320	-0.014592	-0.011754	-	

0.017364	-0.039688	0.061113	-0.032536	-0.022399	-0.020553	-
0.034697	-0.003709	-0.048832	0.022674	-0.031647	-0.027829	
0.037834	-0.022981	0.058647	0.015884	-0.018101	0.036456	-
0.045460	0.014373	0.014311	0.017357	-0.025569	0.000838	
0.029109	-0.027622					
x256	-0.049297	-0.036695	-0.039011	0.017340	0.049719	-
0.007739	0.033918	0.027623	0.045912	0.034275	0.016469	-
0.009558	0.048408	-0.006254	0.038330	0.043589	-0.017173	
0.000918	0.046587	0.019764	0.004927	-0.005801	-0.002060	-
0.031296	0.039547	-0.068425	-0.057165	0.073493	-0.033053	
0.058694	-0.004676	-0.006759	-0.060950	0.072428	0.019677	-
0.057362	0.026738					
y256	-0.026682	-0.021139	-0.054263	-0.012248	0.003856	
0.012100	0.012541	0.042639	0.026978	0.028623	-0.015125	-
0.027660	0.054601	-0.020799	0.018315	0.038591	-0.018275	
0.044649	0.011590	0.045342	0.016551	0.011132	-0.001020	-
0.047356	0.008229	-0.055735	-0.006623	0.003358	0.003251	
0.053509	0.014925	-0.023729	-0.058464	0.019623	-0.001483	
0.023844	0.025213					
z256	0.040200	0.016053	0.071858	0.034927	-0.029464	-
0.007247	-0.012407	-0.051248	-0.094577	-0.017974	-0.004677	-
0.014504	-0.056653	0.060077	-0.036663	-0.036388	-0.012870	-
0.048179	0.000218	-0.068808	0.014493	-0.031297	-0.025041	
0.053538	-0.026146	0.079845	0.029113	-0.026661	0.043606	-
0.057089	0.006691	0.008230	0.045016	-0.030547	0.004207	
0.027915	-0.036653					
x257	-0.018106	0.000422	0.005218	0.004837	-0.008695	-
0.005613	-0.004465	-0.025005	0.011741	0.022958	-0.018213	-
0.018997	-0.015696	0.014210	0.002166	0.006001	-0.003161	-
0.026952	0.008065	-0.011024	-0.008654	-0.002582	0.002611	-
0.005308	0.009277	0.023501	-0.010965	-0.025421	0.023451	
0.023507	-0.007668	0.022937	0.016427	0.021358	-0.005082	
0.015175	-0.024185					
y257	0.012280	0.002538	-0.025294	0.006708	0.001900	
0.005878	0.019938	-0.001805	0.001406	-0.012877	-0.010604	
0.010197	0.003207	0.001595	-0.008662	-0.019447	-0.007010	-
0.012028	-0.011472	0.010727	0.005625	0.002362	-0.015915	
0.004642	0.023625	0.004421	0.023596	0.028740	-0.008719	-
0.012574	0.035531	-0.010221	0.041959	-0.033474	0.025675	
0.000940	0.027847					
z257	-0.009755	0.012228	0.007254	-0.010391	-0.017971	
0.020813	0.007055	0.006549	0.008583	-0.002844	0.028001	
0.008986	-0.014346	-0.006300	0.039112	0.010842	0.020033	-
0.009653	0.014376	-0.050661	-0.001986	-0.010211	0.019801	
0.029818	-0.000921	0.000949	-0.003481	-0.015719	-0.003740	-
0.003151	0.035621	-0.005679	0.014349	-0.027021	-0.023006	-
0.008930	0.011267					
x258	-0.019192	0.000224	0.006087	0.003884	-0.010332	-
0.008625	-0.006991	-0.025806	0.010403	0.017909	-0.020161	-
0.020470	-0.020995	0.011339	0.005808	0.004873	-0.000930	-
0.025614	0.013050	-0.011764	-0.009729	-0.005080	0.003918	
0.005854	0.008008	0.025858	-0.005708	-0.032450	0.017566	
0.023310	-0.007953	0.031041	0.021747	0.014711	-0.006004	
0.008481	-0.023450					
y258	0.012702	0.003508	-0.022843	0.008270	0.000954	
0.004740	0.022069	0.002769	0.000868	-0.012826	-0.012583	
0.007084	0.011382	-0.002877	-0.020343	-0.014221	-0.004780	-
0.011298	-0.010850	0.016552	0.017022	0.001009	-0.010001	
0.009267	0.029295	0.000578	0.032764	0.023280	-0.009981	

0.003742 0.023190 0.007873 0.036256 -0.017051 0.030809 -
0.008348 0.017063
z258 -0.012498 0.010528 0.002971 -0.005763 -0.018564
0.016846 0.002684 0.009139 0.007889 -0.003874 0.021350
0.003545 -0.018428 -0.004796 0.038306 0.017193 0.006344 -
0.009083 0.010355 -0.049423 0.001342 -0.004743 0.009932
0.028085 0.001952 -0.003115 -0.003947 -0.009774 0.001591 -
0.004772 0.033541 -0.006947 0.008199 -0.028158 -0.018712 -
0.008205 0.012570
x259 -0.020923 -0.001562 0.004525 0.001895 -0.010475 -
0.013058 -0.011431 -0.025211 0.012288 0.016396 -0.021185 -
0.021500 -0.028594 0.017688 0.009397 0.005578 -0.004570 -
0.027422 0.012775 -0.015557 -0.008589 -0.007519 0.004511
0.017285 0.005358 0.026562 -0.006156 -0.033456 0.018266
0.021461 -0.002996 0.030067 0.019975 0.008606 -0.010015
0.001639 -0.020566
y259 0.010676 0.004162 -0.023725 0.006069 0.002925
0.001573 0.020623 0.007296 0.006950 -0.007272 -0.011224
0.004047 0.013805 0.013449 -0.028877 -0.007818 -0.006060 -
0.016713 -0.014403 0.018886 0.028688 0.001446 -0.000536
0.018757 0.024658 0.001218 0.035380 0.022298 -0.008287
0.018047 0.017768 0.013436 0.025934 -0.007456 0.034475 -
0.018959 0.006012
z259 -0.014416 0.009766 0.001205 -0.001746 -0.019464
0.015177 -0.000113 0.011031 0.005775 -0.006610 0.013669 -
0.002231 -0.021888 -0.008336 0.037363 0.024323 -0.000883 -
0.007080 0.008536 -0.046801 0.003300 0.000560 0.000599
0.029105 0.006206 -0.007550 -0.003514 -0.006652 0.002052 -
0.009253 0.030525 -0.010861 0.003260 -0.026946 -0.017333 -
0.005532 0.012704
x260 -0.020052 -0.003370 0.002619 0.000545 -0.009431 -
0.013743 -0.012286 -0.026222 0.012454 0.015197 -0.018766 -
0.023030 -0.034071 0.015147 0.017180 0.002244 -0.007985 -
0.028755 0.014093 -0.017945 -0.013307 -0.012343 -0.001872
0.018402 0.006008 0.026247 -0.010618 -0.032720 0.015120
0.013742 -0.000171 0.032632 0.022237 -0.003261 -0.014590 -
0.000951 -0.017731
y260 0.015021 0.002201 -0.026418 0.007192 0.007274
0.002452 0.022204 0.010945 0.007585 -0.003743 -0.003675
0.003704 0.020539 0.009142 -0.030636 -0.011515 -0.016751 -
0.019792 -0.013819 0.018924 0.025822 -0.006007 -0.004436
0.007254 0.030235 0.001283 0.028003 0.023209 -0.009439
0.021273 0.009452 0.026697 0.021530 -0.006719 0.035524 -
0.017993 -0.004262
z260 -0.016110 0.010268 0.002765 0.003067 -0.023300
0.013565 -0.000649 0.011783 0.000720 -0.010722 0.005316 -
0.005808 -0.023402 -0.017583 0.033550 0.028852 -0.007272
0.000061 0.007481 -0.041137 0.005710 0.004748 -0.003643
0.027926 0.009687 -0.013102 0.002138 -0.003167 0.009329 -
0.008911 0.028724 -0.014363 -0.003255 -0.015273 -0.013410 -
0.004420 0.017144
x261 -0.019868 -0.004714 0.002251 -0.000698 -0.009336 -
0.014104 -0.014196 -0.026501 0.012302 0.016968 -0.015250 -
0.022055 -0.035421 0.012486 0.020559 0.000396 -0.006490 -
0.029714 0.013362 -0.018575 -0.017203 -0.016772 -0.007740
0.020758 0.005400 0.027664 -0.009385 -0.032298 0.012647
0.005101 0.000617 0.031974 0.023365 -0.008639 -0.019637
0.001753 -0.014331
y261 0.018225 0.001142 -0.027943 0.005978 0.009772
0.004787 0.022348 0.012385 0.009653 0.003143 -0.000399

0.004671	0.019028	0.009712	-0.024865	-0.008364	-0.020271	-	
0.024094	-0.022690	0.019276	0.015253	-0.010229	-0.018717		
0.004667	0.028708	-0.000609	0.019616	0.028871	-0.010850		
0.009155	0.013769	0.015248	0.000821	-0.002622	0.030271	-	
0.006556	-0.004684						
z261	-0.019440	0.010498	0.003129	0.005951	-0.024646		
0.011441	-0.003118	0.011616	-0.000057	-0.016174	-0.000335	-	
0.011530	-0.025118	-0.016538	0.033928	0.035504	-0.011800		
0.002381	0.006157	-0.040043	0.008694	0.011855	-0.005347		
0.027903	0.013078	-0.018910	0.002706	-0.001987	0.011514	-	
0.008742	0.022286	-0.019433	-0.002310	-0.017762	-0.005982	-	
0.009140	0.014701						
x262	-0.020320	-0.006027	0.000617	0.000304	-0.010560	-	
0.016045	-0.015677	-0.026223	0.010285	0.017160	-0.016411	-	
0.024769	-0.035740	0.010648	0.023155	0.001676	-0.011069	-	
0.029863	0.013758	-0.018175	-0.018550	-0.018018	-0.012764		
0.023929	0.007560	0.024501	-0.008811	-0.033486	0.009075		
0.007186	0.001861	0.031077	0.024043	-0.011888	-0.022169	-	
0.006212	-0.014342						
y262	0.019460	0.000197	-0.029386	0.005961	0.010593		
0.004732	0.020745	0.012823	0.009870	0.003487	-0.001103		
0.001712	0.016777	0.012608	-0.019916	-0.005302	-0.021231	-	
0.025722	-0.023286	0.014681	0.008896	-0.015411	-0.023157		
0.005314	0.030319	-0.001092	0.011904	0.028372	-0.010995		
0.009329	0.013652	0.009844	-0.004197	-0.004043	0.024440	-	
0.007808	-0.011160						
z262	-0.021721	0.011060	0.001936	0.008799	-0.024749		
0.008261	-0.003435	0.012750	0.001803	-0.018670	-0.004382	-	
0.016676	-0.028622	-0.014486	0.032777	0.040467	-0.018291		
0.000625	0.001972	-0.037294	0.011871	0.018325	-0.005015		
0.025272	0.011801	-0.025818	0.007117	0.001610	0.013322	-	
0.015467	0.015485	-0.018793	-0.003799	-0.020109	-0.006967	-	
0.000234	0.020301						
x263	-0.020614	-0.006820	0.001351	0.000782	-0.012432	-	
0.021609	-0.014478	-0.024770	0.010413	0.015015	-0.020497	-	
0.024604	-0.035112	0.009616	0.021909	0.001510	-0.012193	-	
0.032703	0.012515	-0.018082	-0.017462	-0.013203	-0.010078		
0.022692	0.004917	0.025256	-0.006557	-0.031824	0.010008	-	
0.002284	0.006989	0.030261	0.031854	-0.019072	-0.030782	-	
0.001664	-0.011564						
y263	0.020073	0.000535	-0.028796	0.005661	0.010797		
0.001759	0.018429	0.013919	0.009598	0.000924	-0.005355		
0.002646	0.014112	0.013424	-0.019045	-0.005330	-0.020560	-	
0.026935	-0.021483	0.010390	0.003697	-0.018022	-0.023112		
0.004099	0.024268	0.003748	0.007836	0.032100	-0.004059		
0.003387	0.020150	0.006449	-0.007343	-0.008831	0.016236	-	
0.002469	-0.016022						
z263	-0.023404	0.011452	0.000351	0.012730	-0.025358		
0.008576	-0.004121	0.013215	0.000876	-0.019728	-0.004792	-	
0.022206	-0.028407	-0.017812	0.031427	0.046784	-0.025973		
0.003601	-0.001062	-0.034211	0.015830	0.018983	-0.006874		
0.025905	0.016732	-0.035575	0.008317	-0.001247	0.011319	-	
0.010252	0.007206	-0.021209	-0.007741	-0.014069	0.000112	-	
0.001275	0.021296						
x264	-0.020946	-0.007536	0.000553	0.002074	-0.013485	-	
0.024520	-0.015058	-0.023444	0.008311	0.013153	-0.024375	-	
0.025767	-0.035221	0.008199	0.023683	0.006876	-0.013963	-	
0.034945	0.010324	-0.015362	-0.015200	-0.013479	-0.012811		
0.025147	0.010180	0.024747	-0.001293	-0.033865	0.010371	-	

0.002804	0.009585	0.032017	0.031902	-0.018527	-0.035102	-
0.003966	-0.012474					
y264	0.021118	0.002066	-0.029970	0.005505	0.009174	-
0.000005	0.015955	0.013660	0.005599	-0.003415	-0.009278	
0.006341	0.010069	0.016529	-0.009675	-0.000684	-0.015845	-
0.031191	-0.018889	0.008683	-0.000835	-0.019077	-0.023605	
0.005543	0.026246	0.010702	0.006056	0.029221	-0.000014	
0.003578	0.031640	0.010934	-0.006887	-0.012640	0.003154	
0.001717	-0.022078					
z264	-0.025696	0.009245	0.003206	0.019576	-0.027050	
0.004426	-0.004622	0.012767	0.006534	-0.017013	-0.002118	-
0.037122	-0.029202	-0.032209	0.020995	0.051458	-0.039744	
0.019475	-0.008123	-0.041307	0.025726	0.021798	-0.002927	
0.033041	0.000780	-0.051822	0.003521	-0.001483	0.001083	-
0.021054	-0.011141	-0.030623	-0.004715	-0.017740	0.009558	
0.004756	0.036909					
x265	-0.023036	-0.008937	0.005681	0.003569	-0.015420	-
0.025018	-0.011961	-0.026185	0.017851	0.026786	-0.012199	-
0.039701	-0.032985	-0.006214	0.010868	0.006796	-0.018272	-
0.018637	0.003629	-0.016113	-0.002973	-0.015385	-0.009642	
0.040227	-0.014034	0.015872	-0.004517	-0.034030	-0.008596	-
0.018996	-0.006402	0.020124	0.042185	-0.024004	-0.026397	-
0.008979	0.005697					
y265	0.019866	0.000254	-0.026530	0.007896	0.005477	-
0.003444	0.016012	0.009562	0.010900	0.003430	-0.002985	-
0.002766	0.006141	0.000400	-0.013220	0.001446	-0.020874	-
0.013037	-0.019722	-0.000854	0.003924	-0.021190	-0.017881	
0.022165	-0.001266	0.006450	-0.003844	0.021333	-0.016360	-
0.008981	0.024217	0.005963	0.006014	-0.020048	0.004087	
0.004790	-0.010252					
z265	-0.035655	0.000289	0.019066	0.044189	-0.038431	-
0.003251	-0.002970	0.000060	0.044616	0.028540	0.041517	-
0.108183	-0.038381	-0.105224	-0.033104	0.054966	-0.092446	
0.107898	-0.047415	-0.073442	0.077858	0.026656	0.013458	
0.095362	-0.112947	-0.101760	-0.031204	-0.003575	-0.080079	-
0.090584	-0.091510	-0.077279	0.041103	-0.055558	0.066000	-
0.004084	0.132052					
x266	-0.021955	-0.006967	0.002740	0.001450	-0.013860	-
0.025192	-0.011125	-0.024242	0.009615	0.020227	-0.019653	-
0.030314	-0.026783	0.002369	0.017907	0.011176	-0.009695	-
0.032268	0.006870	-0.005964	-0.009455	-0.017516	-0.012396	
0.033007	0.004619	0.023633	0.006691	-0.037299	0.007771	-
0.007603	0.007794	0.024492	0.035920	-0.011792	-0.046479	-
0.004483	-0.007730					
y266	0.021641	0.003217	-0.029688	0.006873	0.007124	-
0.005131	0.011365	0.011419	0.003853	-0.006445	-0.008751	
0.006003	0.000388	0.009851	0.001747	0.004560	-0.009069	-
0.023948	-0.010495	-0.002582	-0.007106	-0.035554	-0.022296	
0.023638	0.012507	0.015633	-0.010744	0.017778	-0.009741	-
0.001813	0.042621	0.016150	0.004199	-0.022690	-0.027072	
0.021067	-0.018919					
z266	-0.025371	0.011634	0.004864	0.021853	-0.025943	
0.005566	-0.003632	0.017194	-0.003156	-0.032019	-0.012083	-
0.028380	-0.021441	-0.028721	0.018677	0.053242	-0.038704	
0.009004	-0.004422	-0.033004	0.023721	0.033381	0.000396	
0.008404	0.023516	-0.053301	0.007925	0.002679	0.018992	-
0.015843	-0.003655	-0.035872	-0.015521	-0.008601	0.007940	
0.006580	0.023232					
x267	-0.023409	-0.006552	0.003424	0.001941	-0.013838	-
0.025173	-0.006854	-0.026410	0.009169	0.020822	-0.020880	-

0.031941	-0.024924	0.002348	0.013631	0.011201	-0.007767	-
0.034095	0.007493	-0.004283	-0.009286	-0.014563	-0.009930	
0.027572	0.010229	0.017805	0.001575	-0.025656	0.000799	-
0.013149	-0.002206	0.020247	0.035360	-0.008565	-0.034204	-
0.001311	-0.016973					
y267	0.021749	0.003584	-0.026551	0.002415	0.003673	-
0.005247	0.010163	0.011348	0.008069	-0.004910	-0.008064	
0.006708	0.001034	0.007837	0.002665	0.005416	-0.010092	-
0.028718	-0.005031	-0.004040	-0.005467	-0.025197	-0.019221	
0.021041	0.015673	0.010400	-0.006044	0.015443	-0.010003	-
0.006875	0.037208	0.015871	-0.002239	-0.013125	-0.008230	-
0.004928	-0.008440					
z267	-0.024388	0.012255	0.003242	0.022659	-0.022845	
0.008237	-0.005066	0.018508	-0.005668	-0.031960	-0.009971	-
0.028378	-0.017284	-0.030514	0.015684	0.052204	-0.037765	
0.011092	-0.005810	-0.032946	0.026133	0.031742	0.001979	
0.003589	0.029174	-0.060791	0.004837	0.006110	0.023982	-
0.014366	-0.003142	-0.033709	-0.022648	-0.007273	-0.002505	
0.011037	0.011846					
x268	-0.024745	-0.005407	0.002515	0.004787	-0.014860	-
0.025176	-0.002360	-0.027477	0.008328	0.018711	-0.025719	-
0.034726	-0.024837	-0.000118	0.010962	0.012305	-0.005980	-
0.035427	0.006744	-0.000518	-0.006170	-0.014495	-0.011398	
0.023554	0.018211	0.015102	-0.003698	-0.015560	-0.006612	-
0.018248	-0.012210	0.011007	0.038437	-0.005380	-0.027507	
0.007043	-0.021236					
y268	0.021266	0.003221	-0.027644	0.002569	0.002678	-
0.007571	0.009607	0.011637	0.011396	-0.002472	-0.009692	
0.004405	0.001944	0.012615	-0.001966	0.004673	-0.018755	-
0.028895	-0.001516	-0.007864	0.000109	-0.021170	-0.021196	
0.018281	0.016008	0.013022	-0.001027	0.017982	-0.014889	-
0.007168	0.038606	0.009761	-0.003714	-0.021002	-0.001298	-
0.004227	-0.005407					
z268	-0.023124	0.013198	0.002020	0.023159	-0.021634	
0.011271	-0.005067	0.020163	-0.006411	-0.032849	-0.009392	-
0.028022	-0.014827	-0.034632	0.013909	0.051062	-0.035014	
0.010768	-0.007348	-0.030041	0.029576	0.031532	0.003985	-
0.001502	0.035749	-0.067418	0.001701	0.009539	0.028376	-
0.015600	-0.006791	-0.034843	-0.027512	0.000640	-0.006468	
0.008660	0.008977					
x269	-0.024999	-0.005003	0.001018	0.005443	-0.014794	-
0.024607	-0.000560	-0.029880	0.006926	0.021995	-0.022389	-
0.036846	-0.020814	-0.003539	0.007969	0.012580	-0.004567	-
0.034213	0.005531	0.000858	-0.006726	-0.016487	-0.008352	
0.022003	0.018027	0.010337	-0.009297	-0.010478	-0.010614	-
0.023024	-0.015982	0.010252	0.038724	-0.002219	-0.027966	
0.010487	-0.026874					
y269	0.021216	0.004513	-0.026791	0.001917	0.001333	-
0.005495	0.009879	0.008765	0.011771	-0.002530	-0.008764	
0.003713	-0.001199	0.011943	-0.000135	0.003214	-0.017660	-
0.025478	0.001083	-0.011084	0.001557	-0.022824	-0.017022	
0.019711	0.015172	0.012344	-0.001131	0.014996	-0.015219	-
0.007666	0.037348	0.007239	-0.002666	-0.019492	-0.000862	-
0.004254	0.000988					
z269	-0.021520	0.013449	0.001576	0.021810	-0.019940	
0.014995	-0.004439	0.022792	-0.005018	-0.028848	-0.006883	-
0.027357	-0.010400	-0.036337	0.006053	0.046423	-0.033967	
0.012376	-0.007204	-0.025089	0.033555	0.033307	0.004384	-
0.009393	0.038561	-0.069173	0.000847	0.018446	0.027967	-

0.017655 -0.008299 -0.035076 -0.029497 0.002461 -0.004279
0.004237 0.002864
x270 -0.024752 -0.004317 0.000723 0.005294 -0.015129 -
0.022142 0.002114 -0.032646 0.005002 0.025994 -0.017639 -
0.039186 -0.016819 -0.009615 0.005009 0.011736 0.000260 -
0.030612 0.004959 0.002260 -0.007262 -0.020092 -0.003441
0.021545 0.017528 0.005563 -0.012637 -0.005354 -0.011410 -
0.027338 -0.017055 0.010018 0.038326 -0.000065 -0.027524
0.010892 -0.029990
y270 0.021315 0.005617 -0.026994 -0.000221 0.000994 -
0.004443 0.010190 0.005780 0.013855 -0.001778 -0.004856
0.006642 -0.000266 0.011942 0.001864 -0.000941 -0.015607 -
0.023959 0.003245 -0.014033 0.002523 -0.025321 -0.013342
0.017980 0.011047 0.009553 -0.004327 0.010748 -0.012735 -
0.010385 0.038332 0.006330 -0.002640 -0.019598 -0.002620 -
0.005107 0.004943
z270 -0.019476 0.014046 0.001689 0.020422 -0.018738
0.019775 -0.003567 0.024156 -0.003939 -0.023969 -0.003589 -
0.027644 -0.006358 -0.038322 0.000805 0.042245 -0.029436
0.014741 -0.007407 -0.021798 0.034408 0.033517 0.005993 -
0.013999 0.038945 -0.067380 0.001582 0.023267 0.029053 -
0.016708 -0.007298 -0.032650 -0.031453 0.002845 -0.003783
0.000336 -0.001710
x271 -0.024956 -0.004142 -0.000045 0.004948 -0.014174 -
0.019938 0.002888 -0.035600 0.002899 0.030216 -0.008909 -
0.040828 -0.011354 -0.013492 0.003711 0.011495 0.005227 -
0.028023 0.003422 0.001626 -0.009545 -0.024171 0.003147
0.023757 0.016793 -0.001693 -0.016976 -0.006154 -0.007847 -
0.031661 -0.016697 0.011094 0.037705 0.000394 -0.032985
0.009121 -0.030883
y271 0.021690 0.007962 -0.028503 -0.002804 -0.000175 -
0.002572 0.012087 0.000115 0.013330 -0.000012 0.001482
0.009823 0.001224 0.008484 0.005682 -0.004719 -0.009151 -
0.017804 0.005215 -0.014743 -0.003662 -0.030956 -0.008129
0.023009 0.004121 0.003843 -0.008460 0.007484 -0.009994 -
0.009600 0.043051 0.011520 -0.003110 -0.016157 -0.012991 -
0.003450 0.009675
z271 -0.017708 0.013861 0.002355 0.019375 -0.015703
0.023852 -0.004217 0.025148 -0.003155 -0.019470 0.001277 -
0.026869 -0.002595 -0.037176 -0.003311 0.037887 -0.023099
0.014136 -0.008613 -0.022852 0.035760 0.032771 0.007598 -
0.016990 0.037603 -0.063877 0.000851 0.023026 0.031919 -
0.014928 -0.006436 -0.029477 -0.034625 0.002753 -0.006253 -
0.004325 -0.007275
x272 -0.024946 -0.004474 -0.001471 0.005214 -0.012888 -
0.017519 0.005915 -0.035412 0.004272 0.034123 -0.005554 -
0.042347 -0.005745 -0.017537 -0.003708 0.009550 0.003181 -
0.027192 0.001595 0.004120 -0.003238 -0.023747 0.006992
0.019671 0.020364 -0.003071 -0.016076 0.002721 -0.005237 -
0.035304 -0.017450 0.005507 0.037714 -0.001766 -0.025138
0.005519 -0.027064
y272 0.022844 0.009988 -0.026738 -0.003620 -0.001689 -
0.001730 0.015376 -0.000748 0.013218 0.000076 0.002599
0.010302 -0.002597 0.007255 0.007471 -0.010752 -0.000356 -
0.012311 0.008451 -0.014556 -0.006243 -0.036078 -0.010103
0.021542 0.005404 -0.000501 -0.009776 0.001966 -0.008744 -
0.007769 0.032573 0.011214 -0.008632 -0.016480 -0.014483 -
0.003045 0.007234
z272 -0.015860 0.013755 0.000929 0.016549 -0.012243
0.027496 -0.004232 0.024734 -0.000683 -0.012435 0.005838 -

0.024411	0.002721	-0.035503	-0.007494	0.032625	-0.017388	
0.013355	-0.011424	-0.022476	0.034656	0.031719	0.010284	-
0.020365	0.032046	-0.059644	0.002541	0.026524	0.033906	-
0.011965	-0.000396	-0.024452	-0.033076	0.002860	-0.009223	-
0.009178	-0.008022					
x273	-0.024321	-0.004308	-0.002281	0.005359	-0.011056	-
0.014526	0.009521	-0.032584	0.007004	0.036200	-0.002981	-
0.043847	-0.002501	-0.019190	-0.012224	0.005946	0.002837	-
0.027635	0.002503	0.008975	0.006590	-0.022636	0.009553	
0.011457	0.028717	-0.003225	-0.017485	0.011504	-0.002758	-
0.040085	-0.021320	-0.001848	0.033083	-0.002447	-0.013491	
0.002927	-0.021744					
y273	0.024858	0.012444	-0.023643	-0.002084	-0.001843	
0.002288	0.020227	0.003451	0.015251	-0.002534	-0.004455	
0.009749	-0.008023	0.005399	0.001217	-0.016123	0.002338	-
0.009291	0.009745	-0.004578	-0.004284	-0.030816	-0.004243	
0.013628	0.005802	0.010140	-0.000251	0.013538	-0.008441	-
0.011336	0.022162	-0.003062	-0.001852	-0.018493	-0.001935	-
0.002103	0.006756					
z273	-0.013614	0.013887	0.000164	0.013397	-0.007747	
0.030243	-0.004171	0.025278	0.002152	-0.004200	0.012905	-
0.021297	0.004267	-0.029727	-0.012168	0.025171	-0.008702	
0.012426	-0.012753	-0.022269	0.034241	0.030779	0.012135	-
0.026800	0.026630	-0.056704	0.000100	0.026933	0.033209	-
0.008198	0.004310	-0.016181	-0.037406	0.003588	-0.011112	-
0.010856	-0.009653					
x274	-0.022925	-0.002737	-0.002589	0.004902	-0.010377	-
0.008952	0.012091	-0.031179	0.004917	0.036517	-0.001293	-
0.045354	-0.002716	-0.026424	-0.016156	0.002134	0.009920	-
0.022983	0.002589	0.015487	0.011042	-0.026027	0.014874	
0.005185	0.033018	-0.006888	-0.018293	0.018003	0.006642	-
0.045497	-0.022598	-0.008545	0.030395	-0.000622	-0.011477	-
0.002436	-0.013577					
y274	0.026190	0.013349	-0.022876	-0.002720	-0.002281	
0.003697	0.022416	0.000854	0.017462	-0.003840	-0.000513	
0.011591	-0.003334	0.002214	0.004902	-0.020781	0.004829	-
0.004527	0.011398	-0.005317	-0.006269	-0.035527	-0.002383	
0.013316	-0.001366	0.015379	-0.003849	0.007246	-0.005814	-
0.010836	0.020512	0.000595	0.000332	-0.022692	-0.003980	
0.001017	0.002543					
z274	-0.011569	0.015663	0.000426	0.009849	-0.004956	
0.034482	-0.003855	0.024471	0.004402	0.001231	0.016931	-
0.018668	0.003622	-0.027781	-0.014767	0.015683	0.005319	
0.013908	-0.012741	-0.019265	0.030412	0.027514	0.013050	-
0.031118	0.016590	-0.053450	-0.002690	0.025852	0.036375	-
0.005118	0.008698	-0.009871	-0.039861	0.004841	-0.011428	-
0.015599	-0.007794					
x275	-0.021184	-0.000352	-0.002920	0.005158	-0.009189	-
0.004421	0.013328	-0.029426	0.002125	0.034960	-0.000308	-
0.048061	-0.003398	-0.032660	-0.018018	-0.001220	0.016479	-
0.017591	0.001267	0.022006	0.015491	-0.029097	0.018902	-
0.001237	0.035437	-0.008300	-0.017393	0.023031	0.020100	-
0.049173	-0.022868	-0.011740	0.024145	-0.000248	-0.009832	-
0.007904	-0.008144					
y275	0.027496	0.014526	-0.022135	-0.003366	-0.002314	
0.007094	0.021654	-0.000203	0.019056	-0.003397	0.005038	
0.013526	-0.001749	-0.001930	0.003831	-0.018127	0.005821	-
0.001180	0.012390	0.003147	-0.001745	-0.035542	0.006932	
0.014232	-0.000516	0.022384	-0.003030	0.007209	-0.006466	-

0.015693 0.026718 -0.002152 0.007687 -0.013756 -0.006662
0.005917 0.008817
z275 -0.009329 0.017806 0.002003 0.006666 -0.002189
0.039237 -0.003540 0.022158 0.006180 0.005899 0.021177 -
0.016964 0.001949 -0.027840 -0.015800 0.006766 0.023183
0.016329 -0.012496 -0.017455 0.025034 0.023587 0.014392 -
0.033014 0.004324 -0.048026 -0.003261 0.023362 0.040815 -
0.001461 0.015918 -0.002776 -0.041189 0.006298 -0.009865 -
0.021706 -0.004340
x276 -0.020465 0.000008 -0.004458 0.008177 -0.006677 -
0.003681 0.016166 -0.029463 0.005170 0.037597 -0.002059 -
0.052831 -0.004299 -0.034139 -0.025449 -0.004531 0.018337 -
0.017670 0.002158 0.023633 0.025095 -0.028160 0.026176 -
0.011690 0.037968 -0.009330 -0.014000 0.027290 0.021542 -
0.045581 -0.028839 -0.013107 0.013467 -0.000755 -0.000803 -
0.006109 -0.006735
y276 0.025833 0.016881 -0.017944 -0.006392 -0.006306
0.002317 0.021632 0.001123 0.022375 -0.000077 0.005215
0.015393 0.000300 0.003259 -0.009028 -0.016797 0.011974 -
0.000631 0.014312 0.009792 0.007412 -0.027588 0.017732
0.027349 -0.001640 0.030396 -0.003761 0.013372 -0.014278 -
0.007955 0.032591 -0.014605 -0.001490 0.009997 -0.006641
0.009052 0.013199
z276 -0.007316 0.019945 0.005626 0.002349 -0.000538
0.043366 -0.000399 0.021856 0.006866 0.005145 0.027863 -
0.009464 0.005610 -0.025067 -0.018842 -0.001553 0.029776
0.014788 -0.005562 -0.016554 0.022700 0.019766 0.018193 -
0.034345 0.000186 -0.037817 -0.001733 0.021455 0.037074 -
0.000155 0.018548 -0.000822 -0.038107 0.004321 -0.007276 -
0.024998 -0.005154
x277 -0.019410 0.000637 -0.006064 0.010576 -0.005760 -
0.002820 0.016341 -0.032228 0.005813 0.040896 0.002569 -
0.056959 -0.001040 -0.036733 -0.024022 -0.006653 0.020700 -
0.015337 0.002595 0.022320 0.019750 -0.026554 0.035061 -
0.015746 0.033550 -0.012928 -0.013178 0.020944 0.026002 -
0.040436 -0.034328 -0.004026 0.002444 -0.001828 -0.004576 -
0.005791 -0.004100
y277 0.026506 0.015932 -0.019727 -0.006647 -0.006494
0.004243 0.022771 -0.000506 0.023994 0.002986 0.005568
0.013730 0.000465 0.002655 -0.011151 -0.022357 0.011799 -
0.001242 0.017442 0.007913 0.012342 -0.029481 0.014653
0.020089 0.003593 0.033420 -0.004707 0.022367 -0.018663 -
0.004960 0.028090 -0.019779 0.004166 0.011198 0.001070
0.015463 0.009959
z277 -0.005942 0.021499 0.008745 -0.002182 0.000827
0.045670 0.003303 0.021784 0.008024 0.004905 0.032289 -
0.001538 0.008085 -0.020844 -0.022972 -0.008583 0.035627
0.012868 0.000920 -0.015482 0.024015 0.016389 0.020193 -
0.032818 -0.002642 -0.026289 -0.000474 0.023662 0.032127
0.002550 0.020974 -0.002793 -0.032607 0.005214 -0.006249 -
0.024643 -0.007431
x278 -0.018344 0.001183 -0.005604 0.010899 -0.005146 -
0.002615 0.017579 -0.032186 0.008019 0.043421 0.003908 -
0.057472 0.000816 -0.036614 -0.028453 -0.008433 0.023652 -
0.013369 0.004738 0.022394 0.022023 -0.024648 0.043232 -
0.019097 0.032379 -0.012747 -0.011343 0.020483 0.028919 -
0.034671 -0.038181 -0.000757 -0.006442 0.002170 -0.001420 -
0.007660 -0.002490
y278 0.027407 0.014866 -0.023874 -0.002690 -0.005396
0.008183 0.022718 -0.003542 0.024339 0.001697 0.008475

0.008189	-0.000981	-0.000751	-0.005888	-0.030495	0.007850	-
0.002372	0.017998	0.003457	0.009080	-0.031161	0.005158	
0.003766	0.010466	0.036100	-0.008998	0.029421	-0.023769	-
0.010446	0.024710	-0.023328	0.013557	-0.006619	0.005956	
0.028861	0.008930					
z278	-0.004760	0.023749	0.009859	-0.005812	0.002754	
0.048444	0.005722	0.020049	0.008172	0.005735	0.039315	
0.005115	0.009681	-0.016372	-0.024332	-0.014300	0.042364	
0.010950	0.005654	-0.014991	0.022517	0.009494	0.021429	-
0.027334	-0.008143	-0.015186	-0.002373	0.023386	0.026779	
0.002242	0.027869	-0.005499	-0.025526	0.002754	-0.013693	-
0.017722	-0.010492					
x279	-0.017558	0.001340	-0.006516	0.011763	-0.004723	-
0.002194	0.018266	-0.032893	0.009454	0.044200	0.004297	-
0.059218	0.000520	-0.036734	-0.029980	-0.009952	0.025004	-
0.011621	0.005089	0.020833	0.022886	-0.024639	0.048082	-
0.023124	0.030913	-0.012363	-0.011119	0.019177	0.030383	-
0.030588	-0.038562	0.002736	-0.010208	0.000038	-0.001178	-
0.005901	-0.000710					
y279	0.026919	0.015747	-0.024584	-0.001738	-0.005403	
0.010810	0.021738	-0.002711	0.024871	0.003636	0.009008	
0.007749	0.000091	-0.000741	-0.007316	-0.033563	0.007317	-
0.003036	0.020639	0.007977	0.010831	-0.027752	0.003040	
0.001163	0.011150	0.033098	-0.008936	0.030441	-0.026656	-
0.006876	0.025027	-0.025200	0.013292	-0.000609	0.014936	
0.028166	0.010896					
z279	-0.003894	0.024878	0.012225	-0.008643	0.003841	
0.049890	0.007161	0.020156	0.008733	0.004904	0.042738	
0.012039	0.010698	-0.012453	-0.024435	-0.018062	0.044233	
0.007657	0.010246	-0.012478	0.021769	0.007977	0.021749	-
0.024580	-0.009789	-0.009810	-0.000408	0.022840	0.022055	
0.004845	0.029321	-0.008315	-0.019750	0.003873	-0.009123	-
0.021616	-0.011974					
x280	-0.016760	0.001554	-0.006553	0.011999	-0.003747	-
0.001509	0.018569	-0.033444	0.011191	0.044233	0.003704	-
0.059841	0.000770	-0.036643	-0.030399	-0.009977	0.025754	-
0.009423	0.004357	0.019231	0.022776	-0.024655	0.050774	-
0.026802	0.030100	-0.010508	-0.010753	0.017970	0.032624	-
0.025471	-0.039181	0.007022	-0.010213	-0.002783	0.000373	-
0.003645	0.000109					
y280	0.026881	0.015417	-0.022351	-0.002654	-0.004698	
0.012224	0.021700	-0.002643	0.026093	0.002305	0.010874	
0.008233	0.002883	-0.000397	-0.008178	-0.035192	0.006101	-
0.003494	0.018771	0.009182	0.011737	-0.022734	0.003496	-
0.000923	0.011783	0.032420	-0.005757	0.031188	-0.026507	-
0.006843	0.027582	-0.026625	0.015201	0.001532	0.020065	
0.026019	0.020108					
z280	-0.002581	0.025146	0.014490	-0.012071	0.004330	
0.049642	0.008316	0.019544	0.009018	0.002681	0.046785	
0.018853	0.011328	-0.008664	-0.022160	-0.019366	0.044649	
0.003186	0.013223	-0.012362	0.019460	0.007003	0.023575	-
0.019077	-0.013517	-0.008039	0.003956	0.020743	0.019618	
0.003335	0.029382	-0.010484	-0.015269	0.004402	-0.006180	-
0.026880	-0.007273					
x281	-0.015964	0.001120	-0.006341	0.011622	-0.003855	-
0.001445	0.019475	-0.033787	0.012468	0.042135	0.001236	-
0.058474	-0.000593	-0.036190	-0.029771	-0.010247	0.024528	-
0.007917	0.002267	0.016048	0.022165	-0.025026	0.050168	-
0.028458	0.027323	-0.009215	-0.010544	0.014325	0.033923	-

0.021030 -0.040824 0.010904 -0.007456 -0.009772 0.002703 -
0.004207 0.001616
y281 0.026318 0.015623 -0.020208 -0.006581 -0.003637
0.015040 0.024894 -0.001178 0.027885 0.005489 0.014153
0.012623 0.008031 0.000972 -0.011422 -0.042808 0.013174 -
0.003548 0.019627 0.010580 0.009844 -0.018016 0.001893 -
0.002415 0.006176 0.034252 -0.012139 0.032407 -0.025328 -
0.008193 0.028758 -0.026399 0.020664 0.002320 0.024451
0.024793 0.023456
z281 -0.002350 0.024829 0.015639 -0.014546 0.004139
0.047279 0.009349 0.019392 0.009534 0.000679 0.047636
0.024630 0.011061 -0.004231 -0.018151 -0.020004 0.045378
0.000200 0.015820 -0.013373 0.016682 0.007255 0.021305 -
0.012197 -0.016871 -0.004742 0.003354 0.021372 0.015326
0.001880 0.028482 -0.014092 -0.006814 0.005659 -0.008509 -
0.027293 -0.006977
x282 -0.014647 0.000717 -0.005044 0.009861 -0.003652
0.001804 0.019529 -0.032834 0.013002 0.041285 0.001181 -
0.055743 -0.000358 -0.035342 -0.032300 -0.012725 0.024383 -
0.006064 0.004829 0.016166 0.017812 -0.025104 0.048838 -
0.026861 0.022583 -0.008348 -0.010293 0.012362 0.032671 -
0.017193 -0.033601 0.013799 0.002158 -0.018938 0.006449 -
0.010433 -0.005332
y282 0.024975 0.014036 -0.021377 -0.002411 -0.003753
0.016152 0.021908 0.004575 0.023708 0.002893 0.007761
0.009220 0.010648 0.000827 -0.013731 -0.039903 0.006968 -
0.000339 0.022361 0.012162 0.005011 -0.009820 -0.007134
0.002638 0.011740 0.026402 -0.006471 0.029095 -0.023395 -
0.007120 0.036435 -0.024140 0.022445 -0.000193 0.029590
0.018424 0.015224
z282 -0.002143 0.025141 0.016536 -0.016795 0.003100
0.048528 0.010563 0.019209 0.008125 0.003037 0.052877
0.030223 0.013369 0.001880 -0.016914 -0.022738 0.045457 -
0.003064 0.021080 -0.009366 0.014011 0.004503 0.018934 -
0.006335 -0.016541 -0.000946 0.003880 0.024087 0.006796
0.002364 0.035887 -0.019726 0.003333 0.008679 -0.009291 -
0.032203 -0.016928
x283 -0.013731 0.002120 -0.000863 0.006754 -0.004914
0.003797 0.020856 -0.035322 0.013879 0.038154 0.005832 -
0.048418 -0.003628 -0.033864 -0.028906 -0.013091 0.021142 -
0.003350 -0.003019 0.013472 0.021347 -0.029183 0.049744 -
0.036398 0.020250 -0.000378 -0.012374 0.008127 0.031565 -
0.012011 -0.030358 0.019014 0.013985 -0.023315 0.008217 -
0.008787 0.001226
y283 0.022075 0.013896 -0.019750 0.001074 -0.005532
0.012478 0.019401 0.005897 0.019045 -0.002556 0.006526
0.007975 0.007498 0.000206 -0.007352 -0.030868 -0.000711
0.004062 0.010164 0.009317 0.011605 -0.003062 -0.010349 -
0.008073 0.022064 0.026830 -0.006444 0.018645 -0.018380 -
0.006745 0.039340 -0.017824 0.019463 0.008466 0.030733
0.027819 0.027987
z283 -0.001119 0.024041 0.014847 -0.018917 0.003614
0.045026 0.010239 0.018196 0.007110 0.003593 0.057912
0.034813 0.015111 0.004317 -0.010995 -0.021046 0.048353 -
0.005299 0.025730 -0.011193 0.007856 -0.000149 0.019187
0.005925 -0.018134 0.000530 0.001372 0.020746 0.004458
0.001217 0.036234 -0.022475 0.008676 0.017488 -0.019707 -
0.023890 -0.014084
x284 -0.012435 0.000811 0.000359 0.003553 -0.004186
0.006001 0.020739 -0.033697 0.015262 0.035611 0.003539 -

0.044918	-0.006114	-0.030687	-0.029317	-0.015390	0.023810	-
0.002483	0.000735	0.009044	0.013607	-0.030246	0.043642	-
0.030400	0.013318	0.000434	-0.013642	0.004789	0.031716	-
0.010358	-0.028890	0.021284	0.021549	-0.038060	0.012141	-
0.007088	-0.004854					
y284	0.019430	0.011934	-0.021244	0.005449	-0.006407	
0.010053	0.017593	0.010246	0.012783	-0.003629	-0.000280	
0.005783	0.003702	0.006669	-0.005654	-0.031326	-0.000985	
0.003234	0.010898	0.002312	0.007607	0.001811	-0.019708	-
0.002972	0.020619	0.019072	-0.006717	0.011353	-0.010351	-
0.007370	0.035704	-0.017026	0.006338	0.006599	0.023886	
0.038769	0.020584					
z284	-0.001683	0.022780	0.013027	-0.020277	0.001842	
0.041515	0.007638	0.016081	0.006163	0.004820	0.060671	
0.039016	0.012552	0.013746	-0.000487	-0.018058	0.046186	-
0.010305	0.026690	-0.014767	0.002317	-0.003640	0.017380	
0.017883	-0.019648	0.000166	0.000891	0.020727	0.000815	
0.000870	0.036574	-0.028738	0.015077	0.022834	-0.031610	-
0.022297	-0.016393					
x285	-0.011109	0.002154	-0.000458	0.003696	-0.000281	
0.005793	0.017151	-0.029703	0.014093	0.034251	0.000996	-
0.042912	-0.002770	-0.026957	-0.030162	-0.018022	0.025746	-
0.011896	0.004571	0.015684	0.001581	-0.023031	0.035154	-
0.028065	0.008876	0.003332	-0.013596	0.008767	0.037287	-
0.006330	-0.022816	0.021043	0.024708	-0.032997	0.006085	-
0.007411	-0.013680					
y285	0.019113	0.010480	-0.024828	0.003491	-0.005200	
0.010902	0.017015	0.011729	0.012009	-0.001234	-0.003399	
0.006760	0.005841	0.008387	-0.002192	-0.029976	-0.000117	-
0.001103	0.011603	-0.000221	0.002497	-0.001517	-0.017249	
0.003408	0.017666	0.010253	-0.001623	0.012093	-0.010693	-
0.006011	0.045690	-0.015813	0.017359	0.006537	0.020350	
0.030757	0.025296					
z285	-0.002067	0.021460	0.015011	-0.020741	-0.001329	
0.041078	0.008013	0.015161	0.008530	0.004871	0.061952	
0.042505	0.012836	0.018527	0.003399	-0.014276	0.043560	-
0.012768	0.024818	-0.019050	0.005622	-0.005035	0.020792	
0.022062	-0.015447	0.003071	0.003632	0.016824	-0.001935	
0.002672	0.039978	-0.030986	0.019610	0.017870	-0.037685	-
0.020326	-0.016343					
x286	-0.011732	0.003939	0.000219	0.004455	-0.000240	
0.004753	0.013911	-0.029058	0.010713	0.031877	0.000052	-
0.038454	-0.002890	-0.022727	-0.026454	-0.016790	0.022641	-
0.018172	0.000704	0.018989	-0.002304	-0.018223	0.028700	-
0.033829	0.007387	0.007475	-0.015347	0.006376	0.038756	-
0.001334	-0.018862	0.023469	0.025850	-0.023285	-0.003557	-
0.003442	-0.017565					
y286	0.018030	0.009911	-0.025037	0.002494	-0.005439	
0.013360	0.015574	0.010246	0.008598	-0.002355	-0.003355	
0.009482	0.006492	0.007602	-0.001059	-0.026922	0.003861	
0.000364	0.011991	-0.003956	0.002463	-0.006605	-0.017362	
0.008956	0.020630	0.011295	0.000893	0.008395	-0.010607	-
0.009432	0.050907	-0.017306	0.023794	0.002203	0.013104	
0.033166	0.024060					
z286	-0.002427	0.019045	0.015628	-0.019295	-0.004499	
0.039132	0.007929	0.016173	0.009181	0.002010	0.057066	
0.042457	0.011424	0.019454	0.007100	-0.008330	0.039822	-
0.013395	0.024627	-0.023228	0.007723	-0.004631	0.021605	
0.028020	-0.010619	0.001906	0.007190	0.010646	-0.003768	

0.002042 0.040268 -0.030383 0.019249 0.013543 -0.040133 -
0.019889 -0.011478
x287 -0.011145 0.005006 0.001451 0.003020 0.000803
0.005669 0.011603 -0.026835 0.010206 0.031863 0.000665 -
0.033088 -0.001756 -0.018432 -0.024068 -0.016637 0.022449 -
0.023496 0.001637 0.023246 -0.003555 -0.015455 0.019945 -
0.036369 0.009812 0.011502 -0.016954 0.005392 0.031776
0.003059 -0.016910 0.027361 0.022137 -0.014359 -0.005829
0.000118 -0.019248
y287 0.017334 0.007760 -0.025789 0.002321 -0.006426
0.012348 0.014660 0.011528 0.008287 -0.004135 -0.008111
0.010980 0.005585 0.008591 0.001173 -0.021502 -0.001086 -
0.000283 0.008954 -0.005533 0.005750 -0.005705 -0.020480
0.009503 0.028898 0.006073 0.005817 0.006834 -0.016090 -
0.010652 0.049235 -0.014721 0.023614 -0.001290 0.017267
0.030984 0.028743
z287 -0.002258 0.016939 0.015533 -0.018705 -0.006481
0.038311 0.007813 0.017110 0.010361 0.001806 0.054744
0.042036 0.010038 0.019567 0.011124 -0.003428 0.037775 -
0.013317 0.025431 -0.026063 0.011679 -0.007321 0.020195
0.032858 -0.004594 0.002760 0.008537 0.005445 -0.013065
0.001907 0.041531 -0.026358 0.017176 0.008314 -0.041268 -
0.016961 -0.006201
x288 -0.011588 0.006058 0.002852 0.002459 0.001667
0.005869 0.009254 -0.025592 0.009848 0.031085 0.000723 -
0.030482 -0.002928 -0.011210 -0.023329 -0.018652 0.021985 -
0.026339 0.003064 0.021685 -0.007310 -0.013243 0.018664 -
0.037675 0.004911 0.015675 -0.017732 0.004197 0.039397
0.005681 -0.015178 0.026530 0.021215 -0.005049 -0.005547
0.004170 -0.024384
y288 0.015811 0.006588 -0.025540 0.004196 -0.006529
0.011576 0.014170 0.010799 0.006539 -0.006570 -0.010780
0.008558 0.003215 0.009539 0.001909 -0.018985 -0.003523
0.000041 0.005296 -0.009979 0.006865 -0.006635 -0.017095
0.010524 0.026836 0.004468 0.010616 0.006361 -0.010149 -
0.010838 0.047281 -0.015040 0.025435 -0.003153 0.022000
0.027951 0.030703
z288 -0.003116 0.016071 0.015742 -0.018381 -0.008376
0.037609 0.008249 0.016219 0.011849 0.002110 0.052859
0.039423 0.007994 0.019377 0.012106 -0.003121 0.035657 -
0.012316 0.025361 -0.030970 0.012451 -0.009833 0.023177
0.035330 -0.005091 0.003106 0.007540 0.002597 -0.007588 -
0.000557 0.042047 -0.028978 0.018551 0.006377 -0.040073 -
0.015610 -0.006457
x289 -0.012088 0.006480 0.004366 0.001617 0.001119
0.005514 0.007008 -0.023698 0.010878 0.030869 -0.000966 -
0.026714 -0.002315 -0.005594 -0.020790 -0.017623 0.020161 -
0.028137 0.005464 0.019791 -0.009637 -0.011120 0.014947 -
0.034670 0.005192 0.019118 -0.018204 0.003980 0.038705
0.009786 -0.013947 0.025833 0.018187 0.008153 -0.001514
0.004235 -0.025399
y289 0.014710 0.005320 -0.026129 0.004449 -0.006914
0.009889 0.014573 0.011163 0.006627 -0.007370 -0.013739
0.009773 0.003779 0.010615 0.002155 -0.016188 -0.007298 -
0.000911 0.001531 -0.009943 0.006183 -0.005289 -0.015787
0.010623 0.029299 0.001480 0.014224 0.009203 -0.009587 -
0.009522 0.045614 -0.013856 0.025600 -0.002547 0.028375
0.021322 0.035066
z289 -0.004041 0.015585 0.015642 -0.018229 -0.009975
0.036446 0.008039 0.015066 0.012504 0.002689 0.050786

0.036738	0.005673	0.017701	0.014880	-0.000883	0.035471	-
0.011377	0.026016	-0.034057	0.012966	-0.011997	0.024574	
0.039677	-0.003746	0.005372	0.005940	-0.000373	-0.008034	-
0.000538	0.044037	-0.028556	0.018370	0.005451	-0.040241	-
0.013477	-0.004667					
x290	-0.012932	0.005730	0.006075	0.001893	0.000174	
0.004941	0.004143	-0.022301	0.010037	0.031237	-0.002180	-
0.023555	-0.004158	0.001107	-0.018567	-0.015382	0.018242	-
0.026399	0.008704	0.015520	-0.011672	-0.007134	0.012605	-
0.031292	0.006533	0.022484	-0.018692	0.000011	0.036796	
0.012035	-0.013555	0.024199	0.015887	0.018442	-0.002923	
0.007867	-0.027636					
y290	0.013661	0.004014	-0.025392	0.006280	-0.006620	
0.008477	0.014007	0.009148	0.003949	-0.009602	-0.015720	
0.008947	0.002125	0.008932	0.000935	-0.013288	-0.008002	
0.000198	-0.000835	-0.008323	0.006078	-0.003731	-0.015397	
0.011261	0.033417	0.003004	0.016437	0.011441	-0.008440	-
0.009733	0.041539	-0.015600	0.027606	-0.005383	0.027506	
0.018380	0.033040					
z290	-0.005151	0.014990	0.014847	-0.017735	-0.011529	
0.034413	0.008401	0.013878	0.012982	0.002989	0.048095	
0.032947	0.002706	0.015106	0.018841	0.000346	0.034066	-
0.011479	0.025441	-0.037278	0.011380	-0.012809	0.025035	
0.039915	-0.004361	0.004828	0.003253	-0.003461	-0.008298	-
0.001035	0.044402	-0.024887	0.017488	0.000914	-0.037823	-
0.012751	0.000288					
x291	-0.013977	0.005720	0.008564	0.000776	-0.002835	
0.003513	0.001121	-0.022034	0.011594	0.030075	-0.003930	-
0.020630	-0.004378	0.007028	-0.015665	-0.011854	0.014156	-
0.030291	0.011829	0.010583	-0.011016	-0.005963	0.009721	-
0.022370	0.006123	0.023409	-0.017963	-0.004253	0.034007	
0.012614	-0.016124	0.023755	0.014249	0.026147	-0.001622	
0.009444	-0.021780					
y291	0.012490	0.002827	-0.024973	0.008443	-0.006829	
0.005063	0.013363	0.007261	0.002287	-0.014084	-0.018912	
0.007433	0.000046	0.007354	-0.001115	-0.009600	-0.014245	-
0.004608	-0.005183	-0.005418	0.008205	-0.000418	-0.017022	
0.013863	0.033651	-0.001427	0.021691	0.013718	-0.008245	-
0.010154	0.034184	-0.015076	0.027799	-0.009066	0.028588	
0.011270	0.036473					
z291	-0.006530	0.014675	0.013149	-0.016628	-0.012732	
0.031762	0.009046	0.011486	0.012315	0.003432	0.044333	
0.027534	-0.001284	0.010165	0.023335	0.002056	0.032968	-
0.010826	0.023322	-0.039705	0.008372	-0.013495	0.024729	
0.039323	-0.004830	0.006466	0.000450	-0.006814	-0.008518	-
0.000963	0.045438	-0.020674	0.018162	-0.007257	-0.035834	-
0.009652	0.002880					
x292	-0.014848	0.003766	0.008681	-0.000323	-0.005096	
0.001712	0.000125	-0.022974	0.012639	0.028819	-0.007897	-
0.016439	-0.007548	0.012112	-0.011182	-0.006588	0.009271	-
0.027961	0.012950	0.002718	-0.009109	-0.005517	0.008762	-
0.014744	0.006411	0.026211	-0.015842	-0.012334	0.027642	
0.014413	-0.009426	0.018728	0.016576	0.030340	0.001754	
0.010505	-0.023566					
y292	0.011061	0.000264	-0.027257	0.014509	-0.006193	-
0.001113	0.012927	0.005533	-0.001292	-0.022144	-0.029379	
0.004101	-0.005531	0.003558	-0.000499	-0.001751	-0.024451	-
0.005669	-0.015214	-0.003413	0.010057	0.006764	-0.023275	
0.015351	0.036817	-0.004721	0.028524	0.013936	-0.006603	-

0.009782 0.032027 -0.015637 0.028409 -0.018049 0.028058
0.005438 0.036281
z292 -0.008129 0.013632 0.010002 -0.013984 -0.014874
0.026696 0.008512 0.008805 0.011056 0.001060 0.037521
0.018939 -0.007588 0.002559 0.031245 0.005669 0.028309 -
0.011426 0.018983 -0.044810 0.003595 -0.012873 0.022859
0.034435 -0.003704 0.004257 -0.001832 -0.010010 -0.007422 -
0.000530 0.041881 -0.013283 0.017005 -0.018717 -0.031529 -
0.008091 0.005891
x293 0.029955 0.055785 -0.003073 0.020799 -0.040766
0.008608 -0.010848 -0.040863 -0.005756 -0.047497 0.025880
0.010280 -0.001040 0.028957 0.006589 0.002561 -0.013745
0.030373 -0.019682 0.037236 0.014380 0.022919 -0.003206 -
0.021621 0.002646 -0.008285 -0.031019 -0.009140 0.012557
0.031175 0.006294 0.008433 -0.001438 -0.008061 0.020123
0.033093 0.005539
y293 0.002211 0.006756 -0.000519 -0.013490 0.034227 -
0.002117 0.003808 -0.006277 -0.010507 -0.004322 0.019326
0.001339 0.035494 -0.012844 -0.013423 -0.020473 0.016043 -
0.004481 -0.002059 0.018156 -0.007627 0.040420 0.020592
0.016057 0.018768 -0.018782 -0.009987 0.003828 -0.015170 -
0.020175 -0.001120 -0.008111 0.015441 0.015726 0.001778 -
0.004962 -0.008750
z293 0.003091 -0.051048 -0.002542 -0.009083 0.002525
0.000803 -0.022927 0.037752 -0.003933 -0.016275 -0.049196
0.027647 0.010204 -0.024309 0.045968 -0.044775 -0.000261 -
0.007026 0.019986 0.005274 -0.021834 -0.008495 0.001780
0.017728 0.006026 0.005919 -0.024378 -0.018852 0.002031
0.016141 -0.007851 -0.003766 -0.020367 0.012769 -0.023342
0.014911 -0.016628
x294 0.022985 0.051547 0.001966 0.019410 -0.040978
0.008609 -0.021109 -0.041263 -0.004071 -0.037586 0.024592
0.012158 0.004588 0.025487 -0.003831 -0.001909 -0.015667
0.029474 -0.017741 0.044602 0.001519 0.021705 -0.004384 -
0.032360 0.001978 -0.007423 -0.034083 -0.007488 0.009957
0.022401 0.000208 -0.000220 -0.007044 -0.000519 0.008346
0.030031 -0.000426
y294 0.004266 0.016316 0.008708 -0.014143 0.032418 -
0.001810 0.015303 -0.010166 -0.002575 0.011942 0.024746
0.003950 0.035993 -0.013577 -0.000799 -0.020506 0.023564 -
0.001880 -0.004263 0.011724 -0.009902 0.039535 0.015602
0.017217 0.014923 -0.003705 -0.004536 0.004007 -0.013970 -
0.024825 0.012332 0.006694 0.006507 -0.000625 0.008457
0.008487 -0.007826
z294 0.000120 -0.055755 -0.001384 -0.018974 0.006411
0.007840 -0.022356 0.032234 0.000773 -0.011583 -0.043915
0.023866 0.018016 -0.031096 0.050089 -0.060194 -0.000307 -
0.002739 0.020830 0.007442 -0.029508 -0.009575 0.007329
0.015187 0.007257 0.008824 -0.020976 -0.007849 0.006991
0.007305 -0.028758 0.001421 -0.012253 0.011596 -0.032071
0.019088 -0.025712
x295 0.017272 0.047761 0.004951 0.020019 -0.040717
0.005857 -0.027951 -0.039809 -0.002728 -0.031717 0.020654
0.010840 0.007594 0.022905 -0.010864 -0.000770 -0.019437
0.029623 -0.016748 0.050698 -0.006980 0.022534 -0.007945 -
0.040953 0.001209 -0.005391 -0.033125 -0.007336 0.004548
0.017529 0.000405 -0.008311 -0.007387 0.007655 0.006522
0.032443 -0.001799
y295 0.002071 0.021598 0.011398 -0.009912 0.034156 -
0.008731 0.019256 -0.012782 -0.002973 0.011695 0.023231 -

0.001729 0.034392 -0.004695 -0.006172 -0.006813 0.023037 -
0.011536 -0.014856 0.019596 -0.002945 0.039415 0.008835
0.011687 0.007802 -0.001827 0.000362 -0.004263 -0.012985 -
0.027163 0.014791 -0.000479 0.006854 -0.005468 0.024679
0.025102 0.021801
z295 -0.003189 -0.058526 -0.002738 -0.024720 0.011071
0.008672 -0.020505 0.028244 0.000848 -0.013976 -0.041923
0.017478 0.022802 -0.028913 0.045454 -0.064098 -0.002713 -
0.002500 0.018689 0.015866 -0.026500 -0.007132 0.007455
0.008984 0.002406 0.005694 -0.017679 -0.005976 0.012368
0.004185 -0.041741 -0.007353 -0.004945 0.008345 -0.025433
0.029165 -0.017197
x296 0.008002 0.044022 0.009269 0.023415 -0.039649 -
0.004023 -0.038568 -0.029726 -0.002246 -0.016920 0.007804
0.009724 0.011767 0.018692 -0.019075 0.006419 -0.020835
0.023378 -0.020558 0.046628 -0.018682 0.025635 -0.014210 -
0.033101 -0.002334 -0.007904 -0.029703 -0.005110 -0.005350
0.014566 0.010579 -0.017220 -0.009111 0.023027 -0.005206
0.033480 0.000321
y296 -0.001525 0.026777 0.017691 0.000464 0.030719 -
0.015978 0.021991 -0.010036 -0.001986 0.025189 0.021892
0.002618 0.037613 0.001155 -0.004381 -0.002277 0.021619 -
0.023951 -0.023547 0.010573 -0.005036 0.037482 0.000057
0.024424 0.007003 0.012869 0.011999 -0.008151 -0.017923 -
0.030012 0.036749 0.014920 -0.002453 -0.001993 0.016161
0.010067 0.012942
z296 -0.008945 -0.058054 -0.002010 -0.025925 0.010975
0.004009 -0.019767 0.034076 0.000554 -0.005723 -0.042134
0.015871 0.031712 -0.026432 0.037990 -0.062888 -0.001560 -
0.010011 0.011863 0.008617 -0.024000 -0.004416 0.007437
0.029980 0.001491 0.006844 -0.003981 -0.003392 0.010131 -
0.000965 -0.024825 -0.004970 -0.004581 0.013913 -0.037342
0.014526 -0.015673
x297 0.005667 0.042522 0.010669 0.023683 -0.037905 -
0.007234 -0.038682 -0.023349 -0.000524 -0.009167 0.004625
0.010529 0.012651 0.016498 -0.020686 0.011553 -0.017432
0.019546 -0.016950 0.039960 -0.022677 0.027370 -0.014858 -
0.022623 -0.004999 -0.008441 -0.023230 -0.006453 -0.011951
0.010903 0.019361 -0.018069 -0.016464 0.031261 -0.012087
0.032624 0.000899
y297 -0.000544 0.026344 0.019622 0.002438 0.031364 -
0.016518 0.022796 -0.012220 -0.000202 0.023561 0.022361
0.004478 0.038835 0.002753 -0.000997 -0.000750 0.018640 -
0.026160 -0.026975 0.010743 -0.002664 0.037678 -0.000243
0.026234 0.005884 0.016512 0.015375 -0.008458 -0.015582 -
0.029677 0.030853 0.019621 -0.008216 -0.002584 0.014839
0.008282 0.011614
z297 -0.007799 -0.055163 -0.001830 -0.026446 0.011847
0.002565 -0.015069 0.034807 0.002334 -0.004286 -0.036676
0.016493 0.036137 -0.024282 0.037216 -0.055893 0.001010 -
0.013148 0.012270 0.004141 -0.021022 -0.001715 0.009432
0.038043 0.000716 0.008057 0.007533 -0.006655 0.005870 -
0.006138 -0.017521 -0.005008 -0.012184 0.013014 -0.037100
0.011066 -0.006391
x298 0.004148 0.042206 0.012012 0.022653 -0.036193 -
0.011522 -0.037329 -0.017128 0.000914 -0.001305 0.002178
0.011189 0.014241 0.015272 -0.020567 0.013761 -0.012778
0.012407 -0.014003 0.028353 -0.027440 0.027153 -0.017203 -
0.012024 -0.003715 -0.008854 -0.020395 -0.004991 -0.020400

0.007418	0.026504	-0.016217	-0.022344	0.035613	-0.018036	
0.032425	-0.002431					
y298	-0.000601	0.023379	0.021754	0.002459	0.033656	-
0.017151	0.020420	-0.016130	-0.001036	0.018451	0.019304	
0.005345	0.040256	0.004154	0.001436	-0.003265	0.012314	-
0.025753	-0.033522	0.015250	0.000194	0.039205	-0.000727	
0.024671	0.003484	0.018092	0.011751	-0.005365	-0.010615	-
0.021352	0.021715	0.023251	-0.002606	-0.004482	0.012687	
0.002814	0.002419					
z298	-0.006956	-0.049087	-0.000537	-0.027056	0.012014	-
0.000445	-0.011039	0.032040	0.001521	-0.003827	-0.029644	
0.017305	0.042110	-0.019816	0.037071	-0.050176	0.000357	-
0.018225	0.008362	-0.001243	-0.017978	-0.000300	0.008863	
0.037559	0.003383	0.010051	0.012561	-0.004460	0.000790	-
0.007286	-0.012042	-0.006053	-0.007399	0.004911	-0.036180	
0.002625	-0.001022					
x299	0.001455	0.039321	0.013003	0.020021	-0.033828	-
0.014108	-0.033452	-0.012541	0.002914	0.007777	0.001321	
0.009690	0.013714	0.014070	-0.019335	0.009594	-0.007735	
0.006042	-0.010466	0.018086	-0.030885	0.026794	-0.013576	-
0.007537	-0.002320	-0.010975	-0.012328	-0.005615	-0.026125	
0.007677	0.027113	-0.009458	-0.017307	0.031748	-0.022426	
0.034069	-0.004689					
y299	0.000951	0.016656	0.022647	0.000333	0.037186	-
0.017066	0.016045	-0.019753	-0.002926	0.007829	0.013623	
0.006432	0.039894	0.003868	0.004037	-0.006813	0.004404	-
0.025158	-0.038580	0.023399	0.005835	0.040940	0.000144	
0.027273	-0.001663	0.017148	0.002940	-0.001305	-0.002833	-
0.008884	0.009793	0.023487	0.006769	-0.006881	0.010725	-
0.008305	-0.006967					
z299	-0.006869	-0.042233	0.001401	-0.027223	0.012530	-
0.002978	-0.004863	0.024820	-0.000246	-0.003626	-0.020990	
0.016345	0.047809	-0.013573	0.036332	-0.046381	-0.002084	-
0.021643	0.002239	-0.000564	-0.013780	0.003405	0.012140	
0.029752	0.002994	0.010781	0.018771	-0.003197	-0.000299	-
0.002202	-0.011707	-0.006436	0.007787	-0.009094	-0.031728	-
0.007238	0.009785					
x300	0.028876	-0.000818	-0.001066	-0.037730	-0.012775	
0.030776	0.004103	-0.006160	0.006111	-0.016770	0.014514	-
0.010510	0.007405	0.000094	0.020338	-0.017019	0.017828	
0.017017	0.012540	-0.006381	-0.021296	-0.011802	0.002358	-
0.027854	0.028512	-0.007578	0.009904	0.002394	0.008975	-
0.004013	-0.009286	0.001809	0.006740	0.000061	0.030154	
0.019368	-0.002996					
y300	-0.014092	-0.019191	0.005222	-0.014371	0.003254	-
0.017296	-0.023637	0.021063	0.002821	0.000876	0.001256	
0.001814	-0.019916	0.013127	-0.000252	-0.004018	0.017584	
0.009967	0.002221	-0.006828	0.015946	-0.001318	0.011335	-
0.003187	-0.019988	-0.008268	-0.006935	-0.015289	0.018340	
0.009389	0.011987	-0.008580	0.000273	0.005225	0.004766	-
0.024608	0.013831					
z300	0.004011	-0.002251	-0.010334	-0.008809	-0.009410	
0.006498	0.003865	-0.008132	-0.007776	0.007070	0.013003	-
0.002160	-0.030397	0.024544	-0.019792	-0.014421	-0.008634	
0.003282	0.004868	-0.033857	0.029005	-0.016660	0.006310	
0.000947	-0.004885	-0.021143	0.017122	0.004193	0.001487	
0.006372	0.017513	0.019826	-0.007827	0.023837	-0.002880	
0.012487	-0.008816					
x301	0.021320	-0.007047	-0.000019	-0.030135	-0.009223	
0.022315	0.001425	-0.006163	0.008138	-0.021991	0.014622	-

0.019442	0.003869	-0.003525	0.017509	-0.015892	0.015597	
0.020606	0.010589	-0.005421	-0.022705	-0.012417	0.001559	-
0.025865	0.034151	-0.006813	-0.000193	-0.002771	0.006205	-
0.003075	-0.020558	-0.006426	0.000989	-0.000434	0.014623	
0.015450	-0.001192					
y301	-0.012292	-0.022941	0.007631	-0.014200	0.004552	-
0.022826	-0.020589	0.022466	0.003610	0.006610	-0.002986	-
0.000389	-0.017674	0.014152	0.000041	-0.001592	0.011340	
0.015093	0.005187	-0.018062	0.014971	-0.001514	0.010858	-
0.001708	-0.023933	-0.010002	-0.008128	-0.011729	0.019303	
0.000025	0.024864	-0.012401	0.003495	0.006464	0.008660	-
0.021278	0.005747					
z301	0.005031	-0.001783	-0.008030	-0.015071	-0.007225	
0.011303	0.002515	-0.008053	-0.007733	0.006914	0.013077	
0.004776	-0.034389	0.027916	-0.021053	-0.016069	-0.003145	
0.007019	0.004553	-0.035200	0.026898	-0.015286	0.011014	-
0.000385	-0.013562	-0.022231	0.022594	0.009697	0.004436	
0.006583	0.016198	0.013983	-0.006138	0.016811	-0.005417	
0.011178	0.001404					
x302	0.015869	-0.009118	0.001788	-0.026919	-0.004303	
0.016456	0.001253	-0.005527	0.008237	-0.022453	0.019129	-
0.023993	0.002518	-0.004887	0.012574	-0.018469	0.015424	
0.021597	0.007763	0.000868	-0.023336	-0.012329	0.000626	-
0.026363	0.031615	-0.001835	-0.004663	-0.007906	0.004163	-
0.006287	-0.025489	-0.008681	-0.000287	-0.004064	0.014298	
0.007157	0.000937					
y302	-0.011792	-0.022142	0.009863	-0.015036	0.007086	-
0.024445	-0.020746	0.025271	0.000707	0.013919	-0.003843	
0.002050	-0.013995	0.015621	-0.003501	-0.002993	0.006385	
0.016754	0.001049	-0.016320	0.016579	0.002500	0.009052	-
0.005278	-0.029820	-0.006355	-0.001382	-0.014359	0.023308	-
0.011586	0.031829	-0.003954	0.008381	0.003319	0.015978	-
0.028233	0.019799					
z302	0.006285	-0.003868	-0.006443	-0.018947	-0.006700	
0.013851	0.003236	-0.009853	-0.006233	0.004686	0.011264	
0.007225	-0.038338	0.028691	-0.020460	-0.015993	-0.000219	
0.010502	0.008586	-0.041617	0.022919	-0.016277	0.014111	
0.003335	-0.017204	-0.026408	0.022477	0.015967	0.007419	
0.009164	0.013827	0.003248	-0.007836	0.012258	-0.013736	
0.016598	-0.003657					
x303	0.010889	-0.012468	0.002586	-0.024143	-0.002224	
0.012304	-0.001430	-0.002184	0.009007	-0.022540	0.018468	-
0.024687	0.001274	-0.005368	0.010598	-0.015391	0.010192	
0.020979	0.004367	0.000446	-0.021526	-0.007845	-0.003443	-
0.021509	0.032221	-0.001109	-0.006011	-0.008584	0.005614	-
0.007082	-0.022508	-0.010052	-0.005227	-0.001344	0.010473	
0.003840	-0.000466					
y303	-0.010577	-0.023875	0.011007	-0.015488	0.006859	-
0.023569	-0.021555	0.026841	-0.000695	0.019174	-0.006682	
0.005488	-0.010066	0.015877	-0.003255	-0.002551	0.002701	
0.017045	0.000879	-0.020796	0.016526	0.004982	0.006475	-
0.004411	-0.028504	-0.005305	0.000574	-0.011051	0.028335	-
0.017650	0.038327	-0.001174	0.008804	0.006246	0.015956	-
0.028325	0.022894					
z303	0.007162	-0.003907	-0.005328	-0.021001	-0.004777	
0.014396	0.005301	-0.011518	-0.005564	0.002944	0.011056	
0.007378	-0.042164	0.027510	-0.019684	-0.017372	0.001769	
0.014547	0.012082	-0.042287	0.020526	-0.016256	0.018549	
0.002032	-0.020109	-0.027863	0.021421	0.018187	0.006627	

0.011210 0.009626 0.000613 -0.006698 0.005327 -0.015304
0.017247 -0.003793
x304 0.005811 -0.015543 0.002399 -0.022449 -0.000658
0.009809 -0.005748 0.003135 0.011406 -0.015791 0.019981 -
0.021009 0.001763 -0.005845 0.009193 -0.008065 0.002678
0.013929 0.004623 -0.003105 -0.021827 0.005683 -0.013687 -
0.013571 0.033460 0.002063 -0.003251 -0.007373 0.007042 -
0.012333 -0.009789 -0.011656 -0.008961 -0.000540 0.009317 -
0.000792 -0.002941
y304 -0.006145 -0.024643 0.010868 -0.013960 0.006871 -
0.022585 -0.018659 0.022854 -0.002001 0.022402 -0.007749
0.005182 -0.004393 0.013620 -0.001064 -0.003247 0.000887
0.018349 0.004133 -0.025695 0.017120 0.005519 0.006395 -
0.004055 -0.024042 -0.002192 -0.002466 -0.009680 0.025306 -
0.018007 0.039338 0.004598 0.006280 0.010705 0.015665 -
0.026765 0.023119
z304 0.006407 -0.004068 -0.004024 -0.022721 -0.002653
0.014765 0.009361 -0.012236 -0.005219 0.002129 0.008701
0.007525 -0.047269 0.022147 -0.016769 -0.020140 -0.000886
0.019666 0.016150 -0.039036 0.017736 -0.013194 0.024926
0.003837 -0.020208 -0.029709 0.017554 0.022239 0.013138
0.014635 0.002206 0.002906 -0.004748 0.000688 -0.010756
0.018014 -0.010309
x305 0.003597 -0.015802 0.004244 -0.021540 -0.000557
0.007127 -0.006983 0.006504 0.012922 -0.014929 0.017751 -
0.019745 -0.002307 -0.005915 0.009775 -0.004193 -0.006104
0.007648 0.008162 -0.006689 -0.020361 0.008842 -0.014831 -
0.008294 0.037122 0.000911 -0.000617 -0.001613 0.008889 -
0.016081 -0.003302 -0.011091 -0.008106 -0.003013 0.009421
0.002915 -0.007203
y305 -0.003495 -0.024112 0.009718 -0.012478 0.007544 -
0.022581 -0.015773 0.018004 -0.002555 0.020926 -0.006323
0.002757 -0.003182 0.011709 0.000981 -0.004151 0.001509
0.019416 0.005278 -0.027733 0.019022 0.001618 0.009004 -
0.005164 -0.021559 -0.000071 -0.009067 -0.010940 0.020140 -
0.012693 0.035252 0.010064 0.002676 0.016450 0.013969 -
0.024639 0.019737
z305 0.005786 -0.004430 -0.005365 -0.022897 -0.003014
0.015896 0.011628 -0.011793 -0.005366 0.004788 0.007082
0.007593 -0.047253 0.016335 -0.013827 -0.019727 -0.003135
0.020932 0.017721 -0.035583 0.016408 -0.007583 0.025400
0.007484 -0.017435 -0.030442 0.015080 0.021455 0.016611
0.016253 0.000329 0.008235 -0.004601 0.000116 -0.003934
0.017038 -0.014903
x306 0.001490 -0.015032 0.003899 -0.020576 -0.001857
0.003030 -0.007148 0.008266 0.011635 -0.011799 0.016896 -
0.019695 -0.005988 -0.005848 0.010948 0.000078 -0.013395
0.005497 0.007419 -0.011747 -0.017700 0.007258 -0.015752 -
0.010041 0.042612 0.004888 -0.001217 -0.005130 0.009994 -
0.016275 0.001571 -0.009892 -0.006418 0.003011 0.004939
0.006281 -0.009624
y306 0.000080 -0.022871 0.008152 -0.012035 0.007999 -
0.021470 -0.012290 0.011240 -0.003463 0.018471 -0.004731
0.001102 -0.001438 0.010465 0.003266 -0.003116 0.004642
0.020737 0.008073 -0.028632 0.022907 -0.002105 0.013398 -
0.003473 -0.018702 0.002090 -0.016878 -0.013102 0.012403 -
0.002881 0.031936 0.016613 -0.005139 0.027678 0.013743 -
0.019925 0.016270
z306 0.004815 -0.004922 -0.006181 -0.021702 -0.003553
0.016525 0.013738 -0.009864 -0.004274 0.007875 0.004511

0.007225	-0.046620	0.008110	-0.011224	-0.019389	-0.007755	
0.019902	0.020659	-0.030249	0.015584	0.000683	0.025536	
0.013494	-0.012018	-0.031657	0.014090	0.023109	0.020706	
0.016392	-0.000573	0.014824	-0.003587	-0.001176	0.007286	
0.014324	-0.023071					
x307	0.020546	-0.017545	-0.012358	-0.052117	-0.020960	
0.010935	0.004058	0.000745	0.001866	0.021330	0.000894	
0.005082	-0.005376	-0.015683	-0.007887	-0.013750	-0.012095	
0.017198	-0.006227	-0.003837	0.018208	0.030543	0.003828	-
0.005957	0.006670	0.017547	-0.003933	0.020369	-0.024490	-
0.004335	0.003757	-0.007266	-0.035331	-0.002923	-0.003422	-
0.015396	0.007221					
y307	-0.023563	0.004445	0.000617	-0.010382	-0.032116	-
0.019512	-0.000833	0.019084	-0.005560	-0.040902	0.005265	-
0.004482	-0.012512	0.030648	-0.022058	-0.004324	0.000131	-
0.009368	0.027444	0.012952	0.020695	-0.015923	0.009731	-
0.017532	-0.017197	0.015096	-0.016465	0.007359	-0.005559	
0.027603	-0.005996	-0.000547	-0.021967	-0.034077	0.016255	-
0.009350	0.015015					
z307	0.000324	0.029533	-0.025747	0.038893	-0.020181	-
0.021233	-0.006163	-0.001145	-0.017836	0.018410	-0.024911	
0.017774	0.011465	0.015029	-0.024425	0.028759	-0.005286	-
0.014625	0.020778	0.020555	-0.000061	-0.003216	-0.001414	-
0.014299	0.011722	-0.006440	-0.009860	0.003039	-0.001377	-
0.019762	-0.009440	0.010399	0.004106	-0.002457	0.004405	-
0.005569	0.004320					
x308	0.017959	-0.019064	-0.010041	-0.055697	-0.019694	
0.012024	0.005088	0.003499	0.002897	0.025692	0.004717	
0.009105	-0.008921	-0.015375	-0.011992	-0.017192	-0.016291	
0.019438	-0.005920	-0.002577	0.020830	0.031034	0.008075	-
0.005461	-0.000279	0.022886	-0.002656	0.016887	-0.030033	-
0.001364	-0.004408	-0.014880	-0.038694	0.001028	-0.007880	-
0.015318	0.015907					
y308	-0.019240	0.003448	-0.000434	-0.013019	-0.035968	-
0.015931	-0.001895	0.016965	-0.013067	-0.036774	0.002397	
0.001422	-0.014797	0.030999	-0.018428	-0.009046	-0.001343	-
0.001631	0.017955	0.008238	0.021479	-0.020213	0.010520	-
0.015805	-0.010588	0.009057	-0.009609	0.001309	-0.007045	
0.026430	0.000975	0.006905	-0.020839	-0.022886	-0.000719	-
0.000418	0.008886					
z308	0.002522	0.030386	-0.026369	0.035956	-0.023832	-
0.015160	-0.011569	-0.003342	-0.023130	0.017260	-0.022248	
0.025475	0.012572	0.017361	-0.021085	0.026333	-0.005959	-
0.015109	0.019045	0.022033	-0.006496	-0.005180	-0.002873	-
0.006528	0.012067	-0.011411	-0.002593	0.000642	-0.001999	-
0.016323	-0.004147	0.011006	0.004044	0.017450	-0.005730	-
0.007330	0.004725					
x309	0.016723	-0.020296	-0.007770	-0.057352	-0.017128	
0.012401	0.008131	0.004575	0.007645	0.031044	0.004610	
0.009971	-0.011987	-0.019874	-0.013950	-0.018140	-0.019094	
0.021706	-0.004578	-0.006317	0.020185	0.036236	0.008750	-
0.007877	-0.004606	0.025650	-0.004644	0.012896	-0.033293	-
0.003689	-0.013399	-0.018918	-0.039118	-0.003118	-0.007835	-
0.011607	0.019071					
y309	-0.016023	0.002644	-0.001889	-0.015047	-0.039418	-
0.012899	-0.003596	0.015998	-0.019784	-0.034105	0.000302	
0.005967	-0.015574	0.032543	-0.015131	-0.013651	-0.002323	
0.003147	0.010579	0.005922	0.022089	-0.025499	0.009662	-
0.014151	-0.005571	0.003893	-0.002989	-0.000491	-0.008160	

0.027584	0.004489	0.012243	-0.021145	-0.013678	-0.015007	
0.002568	0.007426					
z309	0.005746	0.030100	-0.023669	0.032504	-0.024465	-
0.008972	-0.012329	-0.005364	-0.018617	0.020310	-0.021858	
0.029201	0.013839	0.012514	-0.018009	0.024131	-0.006323	-
0.015196	0.019479	0.017395	-0.015140	0.001154	-0.005864	-
0.005105	0.008750	-0.018069	-0.001041	-0.000985	-0.003365	-
0.017767	-0.006074	0.011350	0.003148	0.023060	-0.002571	-
0.009811	0.006597					
x310	0.015639	-0.020432	-0.005825	-0.057430	-0.013862	
0.010445	0.010482	0.005460	0.009514	0.032754	0.004687	
0.009924	-0.014705	-0.021681	-0.016546	-0.016163	-0.020617	
0.023315	-0.004838	-0.006806	0.021218	0.039732	0.011716	-
0.009370	-0.006220	0.028485	-0.004677	0.009420	-0.031999	-
0.007940	-0.019387	-0.020739	-0.038030	-0.003792	-0.007979	-
0.008770	0.020946					
y310	-0.012488	0.001845	-0.001760	-0.015858	-0.041439	-
0.009540	-0.004594	0.015996	-0.021879	-0.030216	-0.002419	
0.008150	-0.014997	0.030880	-0.012912	-0.016514	-0.004015	
0.006853	0.005931	0.003105	0.022767	-0.025805	0.009229	-
0.014575	-0.004134	0.000795	0.000859	-0.000925	-0.010184	
0.025701	0.002740	0.014674	-0.021161	-0.009705	-0.020758	
0.003262	0.007172					
z310	0.009651	0.029636	-0.019588	0.030683	-0.021884	-
0.006867	-0.011030	-0.006660	-0.013494	0.021957	-0.020537	
0.028109	0.014384	0.008141	-0.019987	0.024219	-0.008024	-
0.011840	0.018490	0.016132	-0.017490	0.008277	-0.001736	-
0.006531	0.001896	-0.016615	0.001118	-0.002861	-0.001165	-
0.024567	-0.013400	0.008240	0.001720	0.023734	0.004687	-
0.009676	0.009752					
x311	0.010750	-0.019646	-0.004617	-0.055857	-0.009680	
0.005619	0.012128	0.007040	0.008558	0.033341	0.003788	
0.010006	-0.018243	-0.021361	-0.016982	-0.012596	-0.019878	
0.022510	-0.004807	-0.004819	0.019787	0.040583	0.009683	-
0.009443	-0.002528	0.026066	-0.005272	0.007179	-0.029178	-
0.009067	-0.022847	-0.020385	-0.027379	0.003564	-0.012308	-
0.006433	0.021906					
y311	-0.004788	0.000554	-0.000555	-0.014123	-0.041469	-
0.004782	-0.004330	0.015888	-0.021091	-0.022228	-0.007264	
0.007968	-0.011522	0.023447	-0.010015	-0.019856	-0.006526	
0.009578	0.003665	-0.000466	0.019298	-0.017881	0.010586	-
0.017851	-0.002072	-0.000660	0.004765	-0.002437	-0.014490	
0.018303	-0.006151	0.016838	-0.018495	-0.004315	-0.018127	
0.001498	0.013643					
z311	0.012130	0.028691	-0.015625	0.029106	-0.016148	-
0.006504	-0.007355	-0.008014	-0.007941	0.026823	-0.016619	
0.024151	0.012231	0.003290	-0.019527	0.019737	-0.013566	-
0.005336	0.023265	0.017013	-0.029298	0.019594	-0.003265	-
0.009232	-0.005128	-0.012827	0.002713	-0.007417	-0.004390	-
0.021580	-0.018660	0.001163	0.006439	0.026703	0.009500	-
0.010303	0.019886					
x312	0.007644	-0.018567	-0.004557	-0.054816	-0.007379	
0.002565	0.012094	0.007374	0.007457	0.031952	0.002846	
0.009207	-0.018888	-0.020315	-0.016568	-0.009588	-0.016808	
0.021171	-0.006724	-0.002890	0.020557	0.037920	0.005851	-
0.008653	-0.001102	0.022364	-0.004150	0.007969	-0.025136	-
0.009433	-0.023093	-0.018695	-0.019686	0.008805	-0.014847	-
0.003626	0.019025					
y312	-0.000727	0.000459	-0.000447	-0.012562	-0.041438	-
0.004166	-0.004256	0.015136	-0.022399	-0.018968	-0.009585	

0.008049	-0.007434	0.019226	-0.009082	-0.019895	-0.007220	
0.007325	0.003361	0.000965	0.017545	-0.015268	0.011177	-
0.016562	-0.002218	-0.000021	0.006551	-0.004754	-0.016027	
0.011602	-0.008878	0.021373	-0.018664	-0.003297	-0.009777	-
0.003862	0.020703					
z312	0.012368	0.028000	-0.015961	0.026510	-0.012598	-
0.009121	-0.005713	-0.008908	-0.008813	0.027114	-0.014093	
0.022928	0.010716	0.002373	-0.018141	0.018429	-0.014600	-
0.002387	0.020611	0.018850	-0.029564	0.015787	-0.006171	-
0.009827	-0.012319	-0.010178	0.005970	-0.011874	-0.002676	-
0.020363	-0.019514	0.002674	0.004720	0.023178	0.013401	-
0.010980	0.025717					
x313	0.004025	-0.016542	-0.004102	-0.053488	-0.003911	-
0.000825	0.012288	0.007719	0.006991	0.030436	0.003161	
0.007650	-0.018391	-0.018372	-0.016239	-0.006666	-0.009952	
0.019432	-0.007384	0.000361	0.020726	0.034966	0.001020	-
0.007622	0.000446	0.017256	-0.003415	0.012342	-0.020752	-
0.007854	-0.021730	-0.016891	-0.009100	0.015774	-0.018783	-
0.000190	0.015743					
y313	0.003015	-0.000518	-0.001798	-0.008994	-0.040797	-
0.003215	-0.006261	0.012913	-0.023174	-0.019580	-0.014978	
0.006792	-0.003746	0.012050	-0.003156	-0.017129	-0.010714	
0.004826	0.002817	-0.002509	0.015326	-0.009775	0.008723	-
0.017194	-0.000909	0.000464	0.007790	-0.007999	-0.015235	
0.001506	-0.013533	0.023764	-0.017498	-0.003876	-0.005595	-
0.011338	0.022509					
z313	0.011210	0.026431	-0.016333	0.022941	-0.006229	-
0.011136	-0.004484	-0.010351	-0.007112	0.024835	-0.011001	
0.021052	0.007890	0.000783	-0.012837	0.016826	-0.014516	
0.003449	0.017860	0.015759	-0.028598	0.013646	-0.010352	-
0.013989	-0.020458	-0.008137	0.008871	-0.013800	0.001954	-
0.017012	-0.019849	-0.000415	0.004299	0.020380	0.006744	-
0.013619	0.025723					
x314	0.017255	0.029084	0.012997	0.011502	-0.006238	-
0.021222	0.016442	0.004358	-0.001880	-0.033157	-0.000880	
0.006822	0.038748	-0.036247	-0.036160	0.004992	-0.009333	
0.002524	0.016297	-0.001576	-0.010428	-0.014157	0.007642	
0.010480	-0.005671	-0.003606	0.018045	0.011519	0.007425	
0.024598	0.010272	0.032214	-0.024710	0.012977	-0.004947	
0.066605	0.005959					
y314	-0.021888	0.007990	0.012392	-0.010907	0.025971	
0.011335	-0.001763	-0.023067	0.029104	0.003303	0.005162	-
0.019524	-0.009740	0.067905	0.033448	0.020766	0.031325	-
0.022561	-0.019624	-0.007860	-0.027557	0.047170	-0.011722	
0.013510	-0.020776	-0.009445	-0.023909	0.004986	0.001446	-
0.026822	-0.026901	-0.050398	0.007310	-0.070566	-0.016292	
0.008678	-0.018239					
z314	-0.019835	-0.004908	-0.009561	-0.014656	0.023108	
0.003376	-0.005910	-0.003781	0.014336	-0.023016	-0.004005	-
0.016734	-0.032136	0.066095	0.055680	0.020688	0.025103	-
0.007244	0.000866	-0.005606	-0.003123	-0.007852	-0.009191	
0.041391	-0.002158	-0.006623	0.016860	0.004620	-0.028164	-
0.034231	0.000263	-0.058100	0.023648	-0.045428	0.001308	
0.004465	0.001498					
x315	0.004535	0.028478	0.011135	0.006999	-0.007010	-
0.017442	0.003861	0.001408	0.006375	-0.023700	-0.005999	-
0.006178	0.023125	-0.006291	-0.024929	0.021934	0.007763	-
0.008449	0.016673	-0.006166	-0.013203	0.003044	-0.008728	
0.019305	-0.014181	-0.010171	0.018388	0.003340	-0.004663	

0.015652	0.016818	0.006352	-0.008477	-0.010315	-0.013190	
0.038580	0.007721					
y315	-0.005906	0.007349	0.022028	0.003281	0.020505	-
0.004608	0.011046	-0.019294	0.013731	0.004481	0.022101	
0.000142	0.029158	0.004108	-0.006610	-0.005222	-0.003822	-
0.003129	-0.011648	-0.000831	-0.014653	0.024686	-0.009091	-
0.007810	0.003707	0.000452	-0.015570	0.004898	0.025102	
0.005947	-0.013635	-0.000373	0.001638	0.002908	0.000424	
0.012478	-0.023667					
z315	-0.011969	-0.008630	-0.008346	-0.006212	0.023951	-
0.005973	-0.001626	0.000897	-0.002809	-0.026432	0.007542	-
0.006338	-0.004716	0.016691	0.018287	0.011367	-0.004502	-
0.003342	0.003136	0.007679	0.006248	-0.020553	-0.003498	
0.017929	0.015761	0.009779	0.031787	0.007138	-0.000683	-
0.013945	0.010862	-0.018729	0.017055	-0.001657	0.008546	
0.010954	-0.000014					
x316	-0.000258	0.026838	0.013167	0.008155	-0.010062	-
0.018409	-0.001614	0.001275	0.006439	-0.015698	-0.003514	-
0.006987	0.024758	-0.004639	-0.027482	0.025782	0.008003	-
0.009614	0.016605	-0.006710	-0.008265	0.007336	-0.017343	
0.012754	-0.010857	-0.008836	0.018555	-0.001422	-0.005735	
0.019637	0.023464	0.004917	0.002564	-0.000900	-0.014027	
0.023279	0.009704					
y316	-0.004871	0.008074	0.025058	0.004570	0.019920	-
0.010216	0.015162	-0.022334	0.017864	0.005247	0.019938	-
0.001192	0.026299	0.004588	0.000598	-0.007459	-0.005119	
0.006302	-0.016797	-0.006701	-0.014637	0.023342	-0.009145	-
0.006663	0.002621	-0.002031	-0.024741	0.003555	0.018023	
0.012850	-0.017919	-0.002582	0.004567	0.008672	0.002610	
0.011235	-0.025421					
z316	-0.013776	-0.009338	-0.008738	-0.007810	0.027595	-
0.010745	0.002349	-0.001636	-0.002569	-0.028808	0.006080	-
0.008852	-0.006404	0.015661	0.019401	0.013400	-0.008113	-
0.002499	-0.005617	0.007680	0.007170	-0.020893	0.000952	
0.012117	0.013346	0.016402	0.024935	0.009962	-0.001033	-
0.008945	0.002868	-0.021054	0.019433	-0.002871	0.009927	
0.012845	-0.003821					
x317	-0.003916	0.024732	0.015139	0.008913	-0.012999	-
0.020392	-0.004729	0.001557	0.006216	-0.009747	-0.003362	-
0.006230	0.024579	-0.002383	-0.028014	0.026690	0.007826	-
0.008500	0.014493	-0.007057	-0.006031	0.007911	-0.024602	
0.008590	-0.010514	-0.011503	0.015604	-0.008102	-0.007286	
0.024511	0.023760	0.005045	0.008349	0.005101	-0.017097	
0.015436	0.009331					
y317	-0.002890	0.008346	0.026746	0.004854	0.019773	-
0.011516	0.015431	-0.023517	0.020754	0.006789	0.021702	-
0.000657	0.025148	0.004693	0.003306	-0.010050	-0.002318	
0.011421	-0.017212	-0.007674	-0.016885	0.020824	-0.009278	-
0.004462	-0.000370	-0.004121	-0.027364	0.003349	0.015856	
0.017578	-0.016331	-0.003101	0.006025	0.011579	0.002584	
0.010160	-0.023723					
z317	-0.014923	-0.009722	-0.007481	-0.010486	0.028981	-
0.015008	0.005893	-0.002384	-0.002723	-0.029720	0.005663	-
0.006367	-0.007428	0.016607	0.017565	0.010216	-0.006891	-
0.000861	-0.010917	0.010763	0.003991	-0.023710	0.001336	
0.011806	0.007770	0.013290	0.019038	0.010034	-0.000593	-
0.003852	-0.003965	-0.022080	0.016973	-0.005590	0.005777	
0.016358	-0.009799					
x318	-0.004974	0.019891	0.015436	0.010125	-0.012234	-
0.022114	-0.008651	0.005194	0.006556	-0.003794	-0.004601	-

0.004285	0.021953	0.001688	-0.027990	0.025123	0.011159	-
0.003659	0.016386	-0.004501	-0.007798	0.011286	-0.027248	
0.011896	-0.015652	-0.018584	0.006721	-0.013377	-0.008512	
0.027039	0.020360	0.007803	0.007652	0.011565	-0.016449	
0.013856	0.002995					
y318	0.002549	0.007060	0.025964	0.006696	0.020033	-
0.007804	0.010394	-0.024108	0.023364	0.010559	0.025779	-
0.003393	0.026013	0.002916	0.005477	-0.008674	-0.000068	
0.013352	-0.014097	-0.008911	-0.016098	0.016877	-0.010654	-
0.003145	-0.004572	0.005265	-0.024626	0.002228	0.018215	
0.021897	-0.011098	0.000004	0.006547	0.013724	0.001837	
0.002771	-0.018053					
z318	-0.010404	-0.008792	-0.007607	-0.013342	0.030790	-
0.018169	0.012706	-0.004225	-0.000231	-0.029943	0.010862	-
0.003860	-0.006085	0.016778	0.017121	0.004851	-0.000831	
0.003441	-0.008224	0.012834	-0.001348	-0.021807	0.005889	
0.016791	-0.002691	0.011103	0.009598	0.018623	0.004017	-
0.002856	-0.010332	-0.024026	0.011837	-0.013089	0.008289	
0.014539	-0.015625					
x319	-0.004391	0.017015	0.015429	0.010541	-0.009324	-
0.021064	-0.010681	0.005924	0.007278	-0.003222	-0.005650	-
0.002608	0.020169	0.004608	-0.027321	0.023220	0.010747	-
0.001755	0.015299	-0.002918	-0.007298	0.014165	-0.023104	
0.009819	-0.018021	-0.019052	0.004095	-0.013760	-0.010739	
0.028023	0.019488	0.009926	0.005122	0.016174	-0.016934	
0.018445	-0.000799					
y319	0.005182	0.005468	0.024072	0.009466	0.019376	-
0.005151	0.004938	-0.022594	0.022419	0.012400	0.025965	-
0.007203	0.026141	0.000816	0.007090	-0.004380	-0.002416	
0.011495	-0.012109	-0.008872	-0.015425	0.013679	-0.014036	-
0.002408	-0.004727	0.012859	-0.022327	-0.003089	0.021440	
0.023555	-0.010095	0.004507	0.003123	0.011739	0.001381	-
0.002520	-0.016665					
z319	-0.007885	-0.008614	-0.007781	-0.012711	0.030402	-
0.018166	0.014105	-0.007312	0.001610	-0.029574	0.011824	-
0.004065	-0.003367	0.016666	0.017221	0.003548	-0.001892	
0.003366	-0.007478	0.010340	-0.000160	-0.019629	0.007700	
0.014346	-0.003904	0.014086	0.010893	0.019219	0.006457	-
0.003462	-0.010037	-0.023493	0.011416	-0.014248	0.009136	
0.006249	-0.012822					
x320	-0.003406	0.013976	0.016025	0.011399	-0.005726	-
0.019637	-0.014259	0.007141	0.008285	-0.003232	-0.006377	-
0.001684	0.017036	0.007389	-0.025148	0.023757	0.010497	-
0.001092	0.014060	-0.000827	-0.009000	0.014426	-0.016726	
0.007957	-0.018643	-0.018022	-0.002577	-0.013406	-0.012323	
0.030318	0.018230	0.016130	0.002255	0.017971	-0.017549	
0.022204	-0.007432					
y320	0.008620	0.003192	0.020452	0.012665	0.018936	-
0.001254	-0.000827	-0.020053	0.019966	0.014805	0.026633	-
0.010307	0.026522	-0.001784	0.009985	-0.000510	-0.006654	
0.010571	-0.007924	-0.007285	-0.013923	0.010024	-0.017943	-
0.001061	-0.006685	0.022438	-0.019304	-0.007937	0.025198	
0.024980	-0.009571	0.007501	-0.003947	0.009907	0.001999	-
0.007092	-0.014519					
z320	-0.004962	-0.007774	-0.007128	-0.010451	0.028161	-
0.018383	0.014050	-0.010216	0.003229	-0.027654	0.013649	-
0.007272	-0.001491	0.016235	0.018647	0.005934	-0.003660	
0.000868	-0.005237	0.008060	0.000540	-0.022001	0.005845	
0.013646	-0.001228	0.018715	0.008816	0.016470	0.011356	-

0.003742 -0.011322 -0.017396 0.013403 -0.017914 0.010789 -
0.012086 -0.009292
x321 0.009104 -0.014120 -0.012142 -0.015541 0.008059 -
0.009362 0.017264 0.017225 0.023751 -0.005657 0.029572 -
0.001294 0.010340 0.045224 0.025003 -0.002024 -0.046207 -
0.036403 -0.015516 -0.018152 0.025911 0.008414 0.022572
0.002756 0.015297 0.007197 0.015346 0.004535 0.008433 -
0.015507 -0.002672 0.013481 0.016394 -0.037121 0.004282 -
0.004099 -0.012615
y321 0.002309 -0.014069 -0.049947 -0.029683 0.050786 -
0.006915 -0.039520 0.022071 0.053230 0.032317 -0.005272 -
0.056224 -0.007143 0.159385 -0.053380 0.007774 0.016666 -
0.050940 0.037659 -0.112867 -0.091159 0.068357 -0.030914
0.091239 -0.053200 -0.053706 0.005825 -0.025651 -0.057253 -
0.010179 0.016157 -0.015212 0.036053 -0.076011 0.048853
0.025053 0.047756
z321 -0.007815 -0.008671 -0.015673 -0.020474 -0.001840 -
0.007223 -0.006539 -0.009825 -0.003839 0.048980 0.020156
0.013589 -0.009640 0.055365 0.048725 0.002536 -0.035126 -
0.002331 -0.009036 0.016631 0.061384 -0.007745 0.026614 -
0.022870 -0.003469 0.016648 0.013310 0.014304 -0.007394 -
0.006695 0.015587 -0.048614 -0.006613 -0.039756 0.002220 -
0.054128 -0.019958
x322 0.013712 -0.012353 -0.024006 -0.024982 0.022154 -
0.005366 0.021846 0.018704 0.037570 -0.001940 0.044354 -
0.017493 0.025891 0.064631 -0.007964 -0.007919 -0.064279 -
0.047099 0.009294 -0.017989 0.019768 0.011790 0.023802
0.013227 0.007926 0.014468 -0.000378 0.000952 0.006644 -
0.010797 0.032506 0.018012 0.003828 -0.032015 -0.016997 -
0.017549 0.015439
y322 0.013343 -0.021214 -0.003240 -0.000958 0.049749 -
0.029434 0.004236 0.013311 0.044235 0.029857 0.031786 -
0.028883 0.014603 0.077961 -0.024454 -0.014815 -0.086806 -
0.012584 0.026007 -0.028974 0.017485 0.002815 0.016549
0.003910 0.004098 0.000413 -0.007346 -0.025147 -0.013531
0.027596 0.063037 0.002658 0.016379 -0.056677 -0.083291 -
0.118913 0.054105
z322 -0.002574 -0.011870 -0.028942 -0.022463 -0.004901 -
0.001759 -0.025231 0.003228 -0.016628 0.055667 0.022592
0.039656 0.013745 0.027472 -0.003179 -0.006803 -0.024485
0.002070 -0.003441 -0.015755 0.030025 0.006473 0.006653 -
0.014854 -0.014498 -0.012935 0.038956 0.023603 0.002093 -
0.000282 -0.010921 0.000936 -0.009952 0.006990 0.089794
0.057592 -0.035754
x323 0.008401 -0.016254 -0.034229 -0.026420 0.024396 -
0.012697 0.012437 0.019643 0.035052 0.007899 0.048975 -
0.018012 0.037358 0.075564 -0.022100 0.005185 -0.074700 -
0.052440 0.010428 -0.020409 0.023247 0.011739 0.020522
0.001252 0.002730 0.007571 0.004749 0.000978 -0.000712 -
0.020159 0.030886 0.013757 -0.008445 -0.012983 -0.005265
0.022807 0.006428
y323 0.001003 -0.027038 -0.008929 0.005224 0.032545 -
0.035272 -0.021718 0.017318 0.018383 0.046210 0.023755
0.000690 0.023788 0.053678 -0.024555 0.006807 -0.052831 -
0.005310 0.005960 -0.039252 0.015855 -0.001498 0.007394 -
0.016368 -0.006157 -0.027942 0.022912 -0.011495 -0.018642
0.002073 0.007271 0.003822 0.013462 -0.014086 0.004098
0.004680 -0.004400
z323 0.007284 -0.010744 -0.023030 -0.023737 0.010760
0.001511 -0.008811 0.000713 0.004045 0.041013 0.024386

0.025153	-0.001677	0.028834	-0.001780	-0.020185	-0.055095	
0.009184	0.008518	-0.013903	0.034125	0.013210	0.002654	-
0.001823	-0.010702	-0.003744	0.020158	0.010794	0.012701	
0.022888	0.018827	0.010017	-0.012781	-0.029296	0.028605	-
0.022581	0.009025					
x324	0.005238	-0.021021	-0.037846	-0.021608	0.036805	-
0.031490	0.006938	0.025391	0.035124	0.019642	0.045558	-
0.017250	0.039465	0.079855	-0.034812	0.010630	-0.088712	-
0.045986	0.004153	-0.037140	0.023165	0.016035	0.012916	-
0.006740	0.000304	-0.002326	0.004332	-0.010569	-0.001448	-
0.013650	0.018835	0.020239	-0.004473	-0.016667	-0.006036	
0.018852	-0.001627					
y324	0.004153	-0.022085	0.001227	0.007769	0.029499	-
0.030062	-0.022806	0.014687	0.017295	0.043178	0.018450	
0.000953	0.016449	0.029230	-0.016655	0.002943	-0.032811	
0.008620	0.008302	-0.034963	0.012026	-0.012274	0.005891	-
0.012533	-0.003544	-0.034900	0.022057	-0.010515	-0.015888	
0.007265	0.000218	0.006172	0.015317	-0.017928	0.002640	
0.002047	-0.002564					
z324	0.007377	-0.011615	-0.024897	-0.025326	0.013343	
0.005408	-0.010298	-0.002862	0.012219	0.032356	0.024078	
0.020442	-0.004447	0.027513	0.000261	-0.015285	-0.063580	
0.006137	0.007644	-0.012880	0.038570	0.018390	-0.007881	-
0.001963	-0.019725	-0.007597	0.016616	0.010663	0.017671	
0.020377	0.014979	0.013324	-0.023452	-0.034229	0.022490	-
0.010656	0.010111					
x325	0.004745	-0.021583	-0.038237	-0.020256	0.039456	-
0.036123	0.004395	0.025861	0.034956	0.023535	0.045342	-
0.015605	0.041356	0.080141	-0.036425	0.012056	-0.091426	-
0.045587	0.001507	-0.042917	0.023884	0.016429	0.014372	-
0.004764	0.001039	-0.008080	0.003483	-0.011357	0.002314	-
0.011415	0.017967	0.023059	-0.000497	-0.020076	-0.005423	
0.016429	-0.005932					
y325	0.005736	-0.019008	0.004021	0.007901	0.024732	-
0.023911	-0.022837	0.012223	0.015221	0.039360	0.014813	-
0.002602	0.014638	0.019960	-0.012038	0.002788	-0.022513	
0.012190	0.010030	-0.028995	0.011114	-0.018746	0.003694	-
0.010766	-0.002915	-0.029466	0.020231	-0.008673	-0.015600	
0.007683	-0.000854	0.002699	0.010446	-0.016292	-0.000207	
0.002150	-0.002421					
z325	0.007356	-0.011497	-0.024377	-0.025763	0.015685	
0.004695	-0.009660	-0.004699	0.014105	0.029341	0.023360	
0.017540	-0.004982	0.025515	0.001323	-0.014463	-0.062155	
0.005862	0.005539	-0.012258	0.038432	0.019461	-0.011299	-
0.002637	-0.021853	-0.007981	0.013399	0.009565	0.019265	
0.017163	0.011160	0.013567	-0.024024	-0.034252	0.021213	-
0.008853	0.007324					
x326	0.005169	-0.022371	-0.037162	-0.019076	0.041104	-
0.039193	0.002514	0.025568	0.033746	0.025628	0.045800	-
0.014414	0.042148	0.080792	-0.035207	0.011142	-0.091834	-
0.043516	0.000771	-0.046462	0.024485	0.016189	0.015477	-
0.006411	0.003810	-0.008213	0.001157	-0.014316	0.004285	-
0.013015	0.018979	0.025613	0.003389	-0.018454	-0.005292	
0.016997	-0.005747					
y326	0.007066	-0.015896	0.005647	0.008386	0.019226	-
0.016543	-0.022299	0.009749	0.011594	0.034057	0.009413	-
0.008081	0.012060	0.010549	-0.006561	0.002999	-0.011534	
0.017696	0.010823	-0.021637	0.010355	-0.025333	-0.000935	-
0.011112	-0.002618	-0.018009	0.018212	-0.008554	-0.015891	

0.008158	-0.004017	-0.003470	0.002596	-0.014043	-0.002471	
0.002290	-0.002557					
z326	0.007398	-0.010731	-0.023376	-0.026168	0.017108	
0.002894	-0.008102	-0.007584	0.013703	0.026070	0.021344	
0.015188	-0.005050	0.019959	0.003903	-0.013256	-0.056257	
0.006509	0.002860	-0.011132	0.037433	0.019048	-0.012805	-
0.001045	-0.022701	-0.009035	0.010369	0.007909	0.022473	
0.012728	0.007668	0.010646	-0.021961	-0.033367	0.021233	-
0.010792	0.002680					
x327	0.006224	-0.021338	-0.035263	-0.016690	0.041257	-
0.042461	0.000862	0.025569	0.033516	0.024710	0.043804	-
0.014090	0.039326	0.080919	-0.034466	0.011026	-0.087845	-
0.040550	-0.001258	-0.046646	0.022411	0.011170	0.015894	-
0.008738	0.007345	-0.004816	-0.003003	-0.019251	0.002321	-
0.013657	0.016687	0.022217	0.001733	-0.016137	-0.004528	
0.019868	-0.007456					
y327	0.008263	-0.012267	0.006540	0.008656	0.012657	-
0.006676	-0.021240	0.006809	0.006539	0.029033	0.003167	-
0.014010	0.009436	-0.000438	0.000862	0.003281	-0.000710	
0.023217	0.011344	-0.012778	0.010796	-0.031835	-0.006057	-
0.010564	-0.002014	-0.002649	0.016296	-0.007166	-0.014398	
0.010102	-0.008115	-0.008371	-0.006971	-0.014515	-0.002368	
0.002598	-0.003770					
z327	0.007319	-0.010014	-0.021262	-0.026039	0.017366	-
0.000257	-0.005998	-0.011390	0.010253	0.021761	0.017360	
0.011641	-0.003647	0.011699	0.008803	-0.012093	-0.046116	
0.007855	-0.000310	-0.009980	0.035426	0.017701	-0.012957	
0.001367	-0.021983	-0.010282	0.007108	0.005182	0.026370	
0.004793	0.005063	0.006044	-0.016152	-0.029293	0.020614	-
0.016613	-0.002328					
x328	0.026810	-0.015107	0.017164	-0.015410	-0.006108	
0.024105	0.016702	-0.022915	0.001477	-0.030646	-0.054160	-
0.026496	-0.036599	-0.060099	-0.033479	0.010829	0.074223	
0.030147	0.059461	0.036614	-0.016193	0.016613	-0.024133	
0.062738	-0.011582	-0.036383	-0.038146	0.002706	0.020429	
0.009307	0.009640	-0.014631	-0.007533	-0.054937	-0.025628	-
0.091904	0.044585					
y328	-0.023024	0.022322	0.016803	-0.020164	-0.050142	-
0.011677	-0.035387	0.031515	-0.076601	0.002212	0.004235	-
0.002668	-0.005257	-0.035771	0.048739	-0.014542	0.027139	
0.036410	-0.021464	-0.009484	0.023864	-0.011329	0.023455	-
0.022436	-0.001808	0.026587	0.037510	0.046983	-0.028102	-
0.007377	-0.070006	-0.045170	-0.007697	0.039033	0.095290	
0.100439	-0.028912					
z328	0.019377	0.007961	-0.000788	0.036767	-0.000476	
0.013497	-0.000486	-0.041064	0.018958	0.003384	-0.044267	-
0.007249	-0.008551	-0.023500	-0.045181	0.029002	0.039067	
0.028603	0.058653	0.055226	-0.065448	0.000151	-0.034439	
0.035529	0.004092	-0.026179	-0.037720	-0.005549	0.014680	
0.013229	0.034373	0.020534	0.000217	-0.011778	-0.062441	-
0.080961	0.019498					
x329	0.009455	0.007625	0.030806	-0.038084	-0.019916	
0.013643	0.016743	-0.004351	-0.030612	-0.020601	-0.031148	-
0.017243	-0.046625	-0.072482	0.023026	-0.010842	0.054993	
0.045793	0.009531	0.014167	0.031146	0.002272	-0.003941	
0.021455	0.001010	0.006992	-0.021489	0.014744	-0.000178	
0.007382	-0.008849	-0.028094	0.005239	-0.025163	-0.021822	-
0.069039	0.032911					
y329	-0.005791	0.004285	0.022767	-0.002767	-0.043364	
0.006590	-0.024468	0.005885	-0.045765	-0.017840	-0.020999	-

0.003504	-0.013504	-0.038669	0.015327	-0.003312	0.048612	
0.030758	0.018460	0.009468	0.002398	-0.007049	0.008961	-
0.011682	-0.010418	-0.001855	0.017244	0.033021	-0.015952	
0.000752	-0.048542	-0.022313	-0.007125	0.010109	0.045839	
0.034544	-0.010507					
z329	0.011478	0.030017	0.012496	0.020990	-0.003573	
0.004078	0.011109	-0.023243	0.000714	0.003175	-0.026216	-
0.004671	-0.021996	-0.032438	0.012250	0.005641	-0.000639	
0.042245	0.022886	0.029812	-0.028618	-0.025979	-0.029474	
0.018573	0.017030	0.013994	-0.025364	0.004504	0.005388	
0.034703	0.020343	0.014742	0.020404	-0.018332	-0.086040	-
0.116963	0.023273					
x330	0.005077	0.006854	0.028511	-0.041731	-0.026443	
0.019375	0.006510	-0.003254	-0.036268	-0.015883	-0.031025	-
0.005609	-0.040895	-0.081961	0.024913	-0.006109	0.077718	
0.044506	-0.001095	0.011026	0.034533	0.012849	-0.001460	
0.018256	-0.009636	0.004422	-0.007830	0.009878	0.002763	-
0.010678	-0.017503	-0.032257	-0.004184	-0.005679	0.010300	-
0.017070	0.012605					
y330	-0.000398	0.003866	0.028980	-0.002036	-0.040204	
0.007449	-0.014247	0.004605	-0.040337	-0.024728	-0.026327	-
0.004614	-0.021616	-0.041219	0.020301	-0.013694	0.039552	
0.034240	0.028407	0.008506	0.004889	-0.014708	0.004860	-
0.013080	-0.004394	-0.000821	0.011745	0.031895	-0.014020	
0.010719	-0.041151	-0.014842	-0.003887	-0.008829	0.004513	-
0.016868	0.000321					
z330	0.006304	0.028437	0.006731	0.018002	-0.013986	
0.014652	-0.003534	-0.023938	-0.006819	0.009340	-0.022442	
0.024221	-0.010588	-0.043789	0.021191	0.017691	0.027244	
0.035002	0.000176	0.020973	-0.027727	-0.023630	-0.038589	
0.017687	-0.004454	0.004093	-0.000489	0.012820	0.016613	
0.010690	-0.005942	0.010590	0.011099	0.000099	-0.018545	-
0.033207	-0.018752					
x331	0.004392	0.007419	0.030851	-0.042534	-0.025235	
0.020506	0.008442	-0.002569	-0.034614	-0.017350	-0.035950	-
0.008794	-0.039720	-0.085364	0.025153	-0.005904	0.087744	
0.047642	-0.001807	0.008932	0.037073	0.019338	-0.000286	
0.020513	-0.010887	0.008879	-0.009760	-0.005937	-0.001625	-
0.012583	-0.019349	-0.033986	-0.004984	0.007374	0.004050	-
0.016610	0.014778					
y331	0.003890	0.003362	0.029709	-0.000258	-0.041750	
0.015083	-0.015003	0.002453	-0.040101	-0.028294	-0.027253	
0.002715	-0.022832	-0.044617	0.023279	-0.014125	0.042831	
0.033563	0.026100	0.008002	0.008909	-0.015473	-0.000531	-
0.015387	0.001541	-0.001589	0.026327	0.030164	-0.010318	
0.004896	-0.034745	-0.014466	-0.006418	-0.012689	-0.002930	-
0.024855	0.003031					
z331	0.010733	0.027715	0.005313	0.015229	-0.010875	
0.024808	-0.007770	-0.027885	-0.002547	0.001820	-0.016928	
0.025551	-0.011415	-0.045356	0.025444	0.027063	0.029426	
0.034702	-0.014204	0.021266	-0.024337	-0.010342	-0.040639	
0.027607	-0.007920	0.011290	0.009807	0.004413	0.018007	-
0.000419	0.006079	-0.005135	0.008918	0.004787	-0.004070	-
0.019973	-0.009954					
x332	0.003763	0.007611	0.031949	-0.041185	-0.022818	
0.018096	0.009968	-0.001459	-0.032277	-0.017727	-0.036522	-
0.009910	-0.038390	-0.083664	0.025476	-0.005476	0.087071	
0.046228	-0.000047	0.008420	0.035507	0.016563	-0.000715	
0.020952	-0.012260	0.008834	-0.011693	-0.008827	-0.001207	-

0.010360 -0.022091 -0.030900 -0.004481 0.012342 -0.001287 -
0.018452 0.013864
y332 0.004649 0.002540 0.028126 0.000045 -0.040806
0.017472 -0.016738 0.001572 -0.038817 -0.026865 -0.025863
0.002156 -0.021034 -0.043305 0.020873 -0.013499 0.041384
0.031068 0.022674 0.008872 0.010826 -0.012935 -0.004122 -
0.013433 0.005309 -0.000663 0.030518 0.025133 -0.010643
0.004965 -0.030738 -0.015131 -0.008746 -0.013434 -0.003364 -
0.023360 0.005454
z332 0.010568 0.025549 0.006021 0.013174 -0.008111
0.025166 -0.008626 -0.028565 0.000471 -0.000079 -0.015606
0.023031 -0.011585 -0.046045 0.025322 0.027251 0.028932
0.033479 -0.017321 0.021373 -0.022016 -0.003912 -0.040559
0.031117 -0.007087 0.012006 0.007329 0.001110 0.017694 -
0.001517 0.011406 -0.004136 0.006330 0.004109 -0.002415 -
0.015617 -0.004891
x333 0.003636 0.008202 0.031681 -0.038675 -0.018750
0.013512 0.011728 -0.000087 -0.029196 -0.017552 -0.034772 -
0.009235 -0.034956 -0.076392 0.024862 -0.002478 0.081042
0.043671 0.003963 0.007557 0.032852 0.011134 -0.001719
0.020337 -0.015457 0.006881 -0.012146 -0.006608 0.002165 -
0.008767 -0.025376 -0.026193 -0.004182 0.017047 -0.009167 -
0.021716 0.011480
y333 0.004401 0.002098 0.025415 -0.000028 -0.038902
0.018005 -0.018655 0.001570 -0.035492 -0.022064 -0.023413
0.001797 -0.017595 -0.039687 0.016814 -0.010184 0.035997
0.028683 0.018792 0.009426 0.012524 -0.011766 -0.009248 -
0.010255 0.007477 0.002364 0.032500 0.020433 -0.013284
0.007652 -0.030764 -0.014156 -0.012498 -0.015351 -0.001251 -
0.021395 0.004633
z333 0.010090 0.023993 0.007366 0.011141 -0.005055
0.022236 -0.008915 -0.027348 0.004379 -0.000491 -0.014418
0.022660 -0.011062 -0.045467 0.025892 0.029172 0.028217
0.034318 -0.018191 0.021157 -0.020093 -0.000582 -0.039915
0.032374 -0.009340 0.014254 0.003647 0.001836 0.015655 -
0.003329 0.011919 0.000010 0.002697 0.004212 -0.000851 -
0.013303 -0.001955
x334 0.003725 0.007779 0.029470 -0.034457 -0.012773
0.004808 0.012615 0.002353 -0.024781 -0.015591 -0.030676 -
0.009248 -0.027782 -0.062489 0.021680 0.001246 0.069108
0.037200 0.008099 0.006095 0.028546 0.004180 -0.003992
0.018064 -0.018356 0.003521 -0.012026 -0.003790 0.003629 -
0.004738 -0.029184 -0.021014 -0.004341 0.022673 -0.016985 -
0.025260 0.008065
y334 0.003457 0.001552 0.020734 -0.001397 -0.035057
0.019585 -0.020396 0.000368 -0.031368 -0.016129 -0.019060
0.001240 -0.013329 -0.034767 0.013598 -0.005909 0.027339
0.026658 0.015080 0.009054 0.015118 -0.008601 -0.015722 -
0.005903 0.007081 0.007264 0.034466 0.019960 -0.007731
0.008174 -0.028463 -0.012168 -0.013445 -0.019681 0.000325 -
0.019518 0.000849
z334 0.009012 0.020979 0.008293 0.007748 -0.000523
0.019657 -0.008139 -0.026487 0.006713 -0.002235 -0.012075
0.022900 -0.009539 -0.043887 0.028315 0.028184 0.026270
0.035775 -0.015775 0.019629 -0.016991 0.006852 -0.036894
0.031390 -0.013719 0.015334 -0.003942 0.010297 0.021432 -
0.009818 0.016184 0.010036 0.002509 0.003442 -0.005564 -
0.009838 0.000853
x335 -0.026993 -0.006693 -0.002438 0.008991 0.027892 -
0.061784 0.017773 0.018996 0.028764 -0.028307 -0.043006

0.033245	0.017857	-0.013122	0.024060	-0.014466	-0.014325	
0.040938	-0.021712	0.057384	-0.044479	0.006993	0.014524	-
0.060224	-0.021875	0.028662	-0.025547	0.069984	0.040079	
0.007922	-0.015690	0.036634	0.056252	-0.048066	0.012053	
0.043875	-0.004579					
y335	-0.026410	-0.005703	-0.009662	-0.004499	0.037246	
0.002169	0.036187	0.058620	-0.045535	0.000916	-0.034514	
0.047914	-0.035663	-0.058464	-0.001854	0.029238	-0.095045	
0.082615	0.003276	0.030850	-0.031351	0.026749	0.005743	-
0.031953	-0.059718	0.007628	0.017663	0.005275	0.085145	
0.039561	0.017935	0.045733	0.041126	-0.042056	0.001794	
0.089337	0.041152					
z335	-0.027751	0.002499	0.002082	-0.023903	-0.040983	
0.056049	0.056079	0.018118	-0.077327	0.070431	0.072919	
0.032054	-0.000281	-0.014489	0.037104	-0.021817	-0.058038	-
0.043617	0.042029	-0.032447	0.004344	-0.000233	-0.016896	
0.061843	-0.058230	0.019148	0.011175	-0.066572	0.032584	-
0.032868	0.130852	-0.086230	-0.004950	-0.151726	-0.027685	-
0.011176	-0.011493					
x336	-0.018796	-0.021118	-0.012658	0.007224	0.007114	-
0.070611	0.023829	0.039453	0.021675	-0.044473	-0.040608	
0.070529	-0.014511	-0.007747	-0.007993	-0.024982	-0.021878	
0.015430	-0.000921	0.029461	-0.043364	-0.041824	0.018320	-
0.027262	-0.047160	0.008477	-0.000093	0.088938	0.042177	-
0.033764	-0.031021	-0.002907	0.034487	-0.004158	0.010097	-
0.025405	0.008187					
y336	-0.027878	-0.009543	-0.009806	-0.009426	0.030049	
0.000714	0.038014	0.061521	-0.053761	0.009481	-0.023034	
0.021520	-0.043972	-0.040200	-0.003441	0.021869	-0.079990	
0.070336	0.004785	0.037261	-0.034324	0.001223	0.010244	-
0.032079	-0.058211	0.017093	0.003138	-0.006563	0.061378	
0.027598	0.032762	0.000528	0.018795	-0.053921	-0.014675	
0.045293	-0.000191					
z336	-0.026064	0.003746	0.011437	0.000163	-0.021486	
0.042587	0.045924	0.011785	-0.050618	0.043206	0.058493	
0.000177	0.019018	-0.027522	0.034691	-0.021609	-0.051746	-
0.027647	0.023567	-0.023881	-0.023223	0.011977	-0.012981	-
0.006202	-0.021098	0.011966	-0.021491	-0.040173	0.037109	-
0.018906	0.075556	-0.022982	0.006404	-0.117207	-0.028793	
0.011107	-0.056910					
x337	-0.022471	-0.028550	-0.010580	0.027134	0.025844	-
0.078821	0.047800	0.058360	0.013854	-0.034661	-0.080825	
0.065818	0.056428	-0.077859	-0.082786	-0.008448	-0.041828	
0.040551	-0.036231	0.037022	-0.089793	-0.112309	0.049893	-
0.081253	-0.023572	0.048731	0.005495	0.084574	0.026263	-
0.044869	0.005324	0.068928	-0.081655	0.059217	0.018310	-
0.015328	0.010420					
y337	-0.004940	-0.002467	-0.005447	0.017206	0.021453	-
0.033441	-0.010293	0.029734	-0.046954	0.057287	-0.011166	
0.041587	-0.092203	0.024802	0.043661	-0.111861	-0.035311	
0.078689	-0.020873	0.086767	0.065727	0.038968	-0.005709	
0.038392	0.018775	-0.084196	-0.023985	-0.028019	0.135285	
0.105881	0.006963	0.003351	0.077851	0.002437	-0.055551	
0.015013	-0.056784					
z337	-0.040467	-0.002316	0.035364	0.014300	-0.015135	
0.025576	0.081642	0.057231	-0.078536	-0.036119	0.038532	
0.024602	0.143262	-0.161823	-0.146401	0.057822	-0.136807	
0.006203	0.050266	-0.028393	-0.118282	-0.105137	0.101193	-
0.109289	-0.013322	0.114232	0.011230	-0.019216	-0.057448	-

0.085213	0.097565	0.028869	-0.145529	-0.013789	0.055850	
0.002070	-0.026809					
x338	-0.014012	-0.039461	0.007715	0.009827	0.036327	-
0.077235	0.049501	0.059002	0.006687	0.041687	-0.111246	
0.024545	-0.070340	0.048423	-0.038105	0.039303	0.061379	
0.034170	-0.102502	0.034713	-0.049368	-0.031877	0.064349	
0.055732	-0.040512	0.004904	0.062854	0.143706	0.045555	-
0.007918	-0.012136	0.013277	-0.066261	0.094964	-0.058673	-
0.005142	-0.029445					
y338	-0.038897	0.006934	-0.024389	0.036804	0.050622	-
0.018877	-0.002401	0.012989	-0.050225	-0.008271	0.006148	
0.025902	-0.020773	-0.016434	0.029529	-0.146206	-0.057368	
0.003745	0.026924	0.021764	0.011901	-0.057145	0.019371	-
0.076014	-0.008283	-0.051267	-0.093320	-0.073641	0.010712	
0.000423	0.020716	-0.034433	-0.058570	-0.061992	-0.025375	-
0.074273	-0.062031					
z338	-0.018361	-0.022306	0.081320	-0.086121	-0.068119	-
0.008708	0.043100	0.098437	-0.033093	0.072260	-0.020204	
0.032600	-0.093471	0.090464	-0.159544	0.211342	-0.021055	
0.176533	-0.073852	0.138071	-0.118092	0.047801	0.196493	
0.224603	0.051024	0.023732	0.142116	0.084361	0.046335	-
0.017123	0.026277	-0.049108	0.126684	0.034466	-0.021256	
0.090169	-0.035298					
x339	-0.009571	-0.027681	-0.022065	-0.003049	0.017848	-
0.043358	0.050425	0.056019	0.041172	0.012373	-0.083453	
0.018745	-0.014411	0.022816	0.003149	0.000333	0.057591	
0.009716	-0.068145	0.030075	-0.060330	-0.078977	-0.015423	-
0.029096	-0.045896	0.013259	-0.026107	0.077324	0.078447	-
0.058110	-0.002491	0.032315	-0.024934	-0.001363	-0.018136	-
0.005824	0.010197					
y339	-0.055675	-0.014211	-0.003496	-0.009281	0.034754	-
0.030784	0.029794	0.075479	-0.072478	-0.008082	0.009221	
0.009340	-0.061201	0.022658	-0.056184	-0.006109	-0.055672	
0.059416	0.010026	0.072567	-0.055294	-0.060062	0.070035	-
0.020857	0.004652	-0.023204	-0.057903	-0.042928	0.023022	-
0.022653	0.017062	-0.065691	-0.012633	0.011942	-0.051897	-
0.063059	-0.067113					
z339	-0.005037	0.007999	0.030725	-0.003632	-0.033619	
0.011365	0.003362	-0.012072	-0.003027	-0.000905	0.017762	-
0.008365	0.013233	-0.014611	0.006732	0.006629	-0.035712	-
0.029494	0.018048	-0.004271	-0.003797	0.025957	0.006891	
0.021875	-0.026577	0.002248	-0.012512	0.004986	0.027798	-
0.020548	0.024413	0.007699	0.055070	-0.046614	0.014391	
0.027879	-0.055672					
x340	-0.005697	-0.025883	-0.027164	-0.003573	0.019553	-
0.031463	0.045565	0.063676	0.038024	0.014668	-0.078372	
0.016435	-0.020123	0.032681	0.006354	0.004288	0.061248	
0.007134	-0.063115	0.026236	-0.056765	-0.076552	-0.015437	-
0.038969	-0.041419	0.015148	-0.015656	0.074040	0.078946	-
0.066019	0.008294	0.035593	-0.024635	0.008401	-0.012987	-
0.001866	0.022998					
y340	-0.056552	-0.016410	-0.002448	-0.012277	0.036266	-
0.028883	0.030114	0.075459	-0.073921	-0.011748	0.014159	
0.005365	-0.061312	0.031858	-0.057967	-0.005866	-0.048752	
0.051355	0.011819	0.069811	-0.063882	-0.067606	0.068680	-
0.023872	0.006845	-0.021523	-0.065723	-0.041477	0.036070	-
0.029377	0.010376	-0.082669	-0.006287	0.021195	-0.049430	-
0.075845	-0.071426					
z340	0.000334	0.007729	0.027541	0.000795	-0.031508	
0.004650	-0.009205	-0.024262	0.002184	-0.007161	0.010092	-

0.011512	0.015978	-0.010003	0.020872	0.000080	-0.035153	-
0.041895	0.027303	-0.002888	0.015628	0.033177	0.007725	
0.019938	-0.011223	0.002898	-0.023834	0.006931	-0.010454	-
0.003582	0.017482	0.033830	0.058613	-0.027856	-0.006678	
0.038898	-0.056698					
x341	-0.002417	-0.023064	-0.028488	-0.003216	0.025222	-
0.022697	0.034621	0.065906	0.031210	0.013998	-0.072683	
0.017589	-0.028648	0.039533	0.010315	0.006516	0.059526	
0.011491	-0.061707	0.023144	-0.054146	-0.073398	-0.021283	-
0.044991	-0.031861	0.025448	0.007995	0.066970	0.075192	-
0.081164	0.014232	0.030533	-0.026053	0.019342	-0.000236	-
0.001305	0.031581					
y341	-0.050530	-0.017940	-0.005067	-0.015507	0.037644	-
0.024093	0.030914	0.072062	-0.072668	-0.009188	0.017126	
0.008797	-0.059115	0.035058	-0.046039	-0.009215	-0.041271	
0.035673	0.014954	0.072774	-0.059178	-0.063481	0.061983	-
0.029552	0.009885	-0.030606	-0.073866	-0.040481	0.029177	-
0.026470	0.004302	-0.068696	-0.004223	0.020702	-0.050312	-
0.064352	-0.052720					
z341	-0.000576	0.008576	0.028874	0.006200	-0.027550	-
0.006783	-0.023819	-0.030661	-0.000692	-0.014716	-0.000002	-
0.013332	0.010648	-0.004836	0.015854	-0.003825	-0.032748	-
0.044002	0.024935	-0.004905	0.027038	0.025145	0.006661	
0.021043	-0.004529	0.015213	-0.014554	-0.000148	-0.027837	
0.002556	0.020753	0.027154	0.044679	0.006328	-0.017659	
0.018542	-0.068319					
x342	0.000600	-0.019793	-0.030763	-0.004916	0.031825	-
0.015276	0.029539	0.063151	0.020267	0.021760	-0.070179	
0.024751	-0.027314	0.048793	0.017162	-0.009164	0.054523	
0.013603	-0.060433	0.018312	-0.051650	-0.067639	-0.029177	-
0.053591	-0.030025	0.025665	0.024080	0.060461	0.060596	-
0.085222	0.005163	0.023169	-0.033426	0.018693	0.014271	-
0.001038	0.034093					
y342	-0.038115	-0.016774	-0.006643	-0.014069	0.039665	-
0.019234	0.017187	0.058055	-0.068750	-0.008632	0.017196	
0.012827	-0.056095	0.037861	-0.026761	-0.015060	-0.038407	
0.022282	0.017731	0.074673	-0.046036	-0.048309	0.055173	-
0.029061	0.026189	-0.039317	-0.068327	-0.037492	0.033169	-
0.015368	0.000501	-0.049816	0.001650	0.017357	-0.042994	-
0.039569	-0.030904					
z342	-0.003686	0.008496	0.022367	0.001745	-0.021238	-
0.008503	-0.028788	-0.019866	-0.001356	-0.014815	-0.002060	-
0.000713	0.011804	0.001443	0.010671	-0.000741	-0.038609	-
0.033307	0.010298	0.008454	0.018248	0.015402	0.009973	
0.019903	-0.002197	0.021153	-0.016465	-0.011330	-0.042068	-
0.009972	0.020561	0.030179	0.029443	-0.014083	-0.020745	
0.004680	-0.053252					
x343	0.002850	-0.013899	-0.030314	-0.006045	0.038007	-
0.006663	0.021879	0.060586	0.008228	0.027395	-0.065864	
0.033701	-0.026883	0.054940	0.026704	-0.026802	0.046847	
0.017272	-0.055389	0.013454	-0.051400	-0.052270	-0.039055	-
0.066674	-0.025570	0.027016	0.042385	0.054759	0.041399	-
0.090267	-0.003856	0.020667	-0.032390	0.021734	0.032399	
0.002494	0.034140					
y343	-0.021578	-0.016014	-0.008475	-0.012475	0.044071	-
0.009552	-0.000393	0.041928	-0.062325	-0.008346	0.016855	
0.023611	-0.047274	0.036103	0.000298	-0.014862	-0.036578	
0.009934	0.021718	0.073333	-0.029448	-0.023611	0.041083	-
0.031279	0.044623	-0.047229	-0.052393	-0.034557	0.028354	-

0.002213 0.003194 -0.015901 0.012529 0.005375 -0.032578
0.002707 0.004585
z343 -0.008969 0.011536 0.013878 -0.003751 -0.020728 -
0.014405 -0.026665 -0.006854 -0.004153 -0.008171 -0.012622
0.007650 0.005325 0.005140 0.008358 -0.002154 -0.038794 -
0.033875 -0.000780 0.024825 0.011345 0.012037 0.014006
0.021251 -0.003705 0.028438 -0.022398 -0.010683 -0.040501 -
0.020838 0.006122 0.028760 0.007706 -0.009406 -0.023142 -
0.007180 -0.041119
x344 0.006155 -0.006293 -0.028459 -0.004811 0.047019
0.004947 0.004264 0.055239 -0.005216 0.028456 -0.051694
0.039961 -0.030274 0.058599 0.041115 -0.039293 0.030560
0.025651 -0.044326 0.011014 -0.043644 -0.022253 -0.047133 -
0.088118 -0.012682 0.028559 0.068354 0.045105 0.026340 -
0.098395 -0.010450 0.015408 -0.020688 0.024660 0.056585
0.015349 0.036720
y344 -0.006043 -0.016419 -0.011070 -0.011497 0.046725
0.001080 -0.011008 0.027460 -0.057154 -0.005428 0.009664
0.036412 -0.032117 0.029731 0.029692 -0.011405 -0.034172 -
0.005604 0.025709 0.070444 -0.018895 0.000027 0.022294 -
0.037711 0.047596 -0.052410 -0.039452 -0.028428 0.026709
0.013571 0.003225 0.016529 0.025998 -0.011198 -0.014958
0.039661 0.044242
z344 -0.006180 0.009203 0.003968 -0.009435 -0.015469 -
0.007673 -0.030000 -0.005218 -0.002174 0.003651 -0.014280
0.022107 0.003562 0.002063 0.013906 -0.010869 -0.037324 -
0.031918 -0.007103 0.032844 0.012769 0.011275 0.008543
0.013868 -0.003859 0.035049 -0.022615 -0.009581 -0.038308 -
0.032817 -0.009663 0.037315 0.014118 -0.011046 -0.022222
0.010165 -0.005189
x345 0.012366 0.004315 -0.024956 0.002465 0.042897
0.006073 -0.001413 0.044239 -0.003340 0.018474 -0.047002
0.045976 -0.034805 0.064175 0.036535 -0.050358 0.031062
0.026555 -0.048746 0.019585 -0.028927 -0.015878 -0.039625 -
0.071199 -0.020776 0.018187 0.067207 0.043899 0.022201 -
0.090559 -0.012392 0.015661 -0.034548 0.006308 0.036327 -
0.003628 0.025218
y345 0.000077 -0.011586 -0.013723 -0.012013 0.042070
0.005209 -0.005106 0.021205 -0.055350 -0.002006 0.009934
0.040087 -0.023531 0.023832 0.029028 -0.019836 -0.025672 -
0.001376 0.020625 0.063522 -0.004725 0.009891 0.019804 -
0.034015 0.042640 -0.055685 -0.032615 -0.025505 0.016984
0.010614 -0.000115 0.019554 0.014683 -0.015064 -0.014606
0.047583 0.040432
z345 -0.004903 0.008724 0.001459 -0.008672 -0.012421 -
0.006941 -0.030642 -0.000359 -0.000321 0.000092 -0.011288
0.030713 0.005152 0.003122 0.014046 -0.004112 -0.031951 -
0.032512 -0.006924 0.036800 0.005957 0.010376 0.003713
0.013848 0.005156 0.037576 -0.028381 -0.015232 -0.054488 -
0.023736 -0.003938 0.029062 0.015798 -0.004583 -0.028634
0.007919 0.002086
x346 0.017337 0.014086 -0.021636 0.006985 0.037618
0.009182 -0.005337 0.033308 -0.000201 0.009159 -0.042028
0.048433 -0.037183 0.066460 0.031296 -0.058700 0.028665
0.025740 -0.049556 0.023938 -0.014836 -0.007083 -0.031542 -
0.054062 -0.025014 0.006826 0.064738 0.039785 0.021049 -
0.082206 -0.015250 0.017503 -0.045447 -0.008794 0.017209 -
0.016853 0.009531
y346 0.006451 -0.007766 -0.015693 -0.009704 0.039755
0.005497 -0.003260 0.017791 -0.054162 0.001709 0.010939

0.043600	-0.015790	0.019514	0.028562	-0.026275	-0.012535	
0.001374	0.014158	0.060639	0.004330	0.015155	0.019211	-
0.032207	0.039993	-0.055575	-0.025240	-0.028737	0.000056	
0.005828	-0.005710	0.018436	0.007518	-0.015267	-0.015538	
0.046560	0.053120					
z346	-0.007012	0.005731	0.003800	-0.009721	-0.014255	-
0.001283	-0.024641	0.000722	0.000090	-0.006462	-0.008374	
0.034382	0.008166	0.002532	0.018426	0.001319	-0.035161	-
0.033196	-0.008037	0.030045	0.005810	0.011882	-0.002731	
0.018851	0.011407	0.041899	-0.033881	-0.019438	-0.055183	-
0.012070	0.005466	0.030319	0.008334	-0.001586	-0.028758	
0.012159	-0.007071					
x347	0.021178	0.022623	-0.018002	0.011185	0.031887	
0.010652	-0.008354	0.022037	0.003567	0.000916	-0.036441	
0.048518	-0.037154	0.068017	0.026319	-0.065791	0.025918	
0.025598	-0.048854	0.026188	-0.003030	-0.002694	-0.024534	-
0.038980	-0.027994	-0.003210	0.064539	0.034014	0.022045	-
0.072893	-0.018852	0.019342	-0.051472	-0.021105	0.003839	-
0.025343	-0.004656					
y347	0.011401	-0.004945	-0.016747	-0.009126	0.036513	
0.006664	-0.000201	0.014009	-0.051847	0.004146	0.010985	
0.046470	-0.008255	0.014042	0.027711	-0.030838	-0.003820	
0.001863	0.007857	0.056587	0.012322	0.019169	0.016586	-
0.026839	0.038644	-0.054155	-0.022721	-0.029582	-0.016595	
0.004020	-0.010193	0.023811	-0.001705	-0.017062	-0.018847	
0.045347	0.058045					
z347	-0.008379	0.005239	0.003113	-0.006768	-0.014195	
0.001802	-0.018025	0.003604	0.000833	-0.010885	-0.002559	
0.033573	0.009986	-0.000917	0.017833	0.010148	-0.034363	-
0.031503	-0.008137	0.030213	0.006590	0.012655	-0.001163	
0.011402	0.009548	0.036637	-0.026336	-0.021778	-0.052395	-
0.006751	0.013101	0.031428	0.003350	0.012184	-0.029366	
0.008721	-0.014495					
x348	0.023955	0.030136	-0.014562	0.015212	0.026248	
0.011150	-0.010649	0.011013	0.006976	-0.007501	-0.028475	
0.046969	-0.034781	0.068170	0.021271	-0.069456	0.023448	
0.025134	-0.046430	0.027617	0.007130	0.001028	-0.015759	-
0.025171	-0.024958	-0.014635	0.059833	0.027545	0.021711	-
0.064888	-0.020897	0.020991	-0.051907	-0.029030	-0.004984	-
0.030802	-0.013103					
y348	0.015340	-0.001971	-0.016457	-0.010433	0.033440	
0.007434	-0.000235	0.007922	-0.047247	0.006231	0.010518	
0.046111	-0.003571	0.007259	0.027655	-0.032691	0.001026	
0.003089	0.004451	0.053441	0.019883	0.021978	0.012359	-
0.025351	0.032782	-0.049289	-0.016656	-0.023148	-0.025980	
0.004687	-0.007230	0.030433	-0.010879	-0.018332	-0.017369	
0.044054	0.055469					
z348	-0.010775	0.001068	0.003219	-0.002986	-0.014394	
0.004742	-0.007102	0.010134	0.000517	-0.014951	0.000721	
0.034024	0.017371	-0.001514	0.016293	0.014075	-0.027728	-
0.029134	-0.005539	0.024471	0.002166	0.012375	0.002356	
0.013645	0.014835	0.027781	-0.030045	-0.034153	-0.055708	-
0.003933	0.008687	0.027893	0.008464	0.023767	-0.030355	
0.006042	-0.012346					
x349	0.026754	0.036796	-0.011561	0.017856	0.019353	
0.010634	-0.012904	-0.000815	0.009831	-0.013496	-0.020541	
0.042899	-0.031095	0.066529	0.016429	-0.069868	0.020651	
0.024330	-0.042887	0.026917	0.015057	0.005999	-0.009526	-
0.012703	-0.018295	-0.023415	0.052495	0.020562	0.022379	-

0.055884 -0.021169 0.021207 -0.053146 -0.036727 -0.008101 -
0.034127 -0.017291
y349 0.017289 0.000263 -0.015074 -0.010008 0.031989
0.010031 0.000236 0.004455 -0.042396 0.004693 0.011044
0.045453 0.002460 0.003147 0.027431 -0.032925 0.004405
0.002603 -0.000243 0.050492 0.024244 0.021455 0.013073 -
0.021469 0.028824 -0.047342 -0.016551 -0.020572 -0.033400
0.004699 -0.008316 0.033756 -0.012121 -0.015478 -0.017667
0.041295 0.052088
z349 -0.011428 -0.003176 0.004031 -0.003560 -0.015685
0.004692 0.000325 0.012809 -0.001019 -0.015990 0.001357
0.032891 0.021162 -0.004194 0.014601 0.019813 -0.019921 -
0.024203 -0.003509 0.016587 -0.005008 0.015732 -0.004503
0.015423 0.018010 0.025346 -0.030842 -0.040390 -0.055727 -
0.002066 0.006266 0.023283 0.006341 0.025830 -0.026894 -
0.002125 -0.013810
x350 0.028927 0.041596 -0.007804 0.018982 0.012770
0.009856 -0.013116 -0.012231 0.012857 -0.017284 -0.013056
0.037868 -0.025355 0.063798 0.012984 -0.070049 0.016954
0.024393 -0.038534 0.023857 0.019621 0.007060 -0.003628 -
0.001676 -0.009203 -0.031758 0.046804 0.013408 0.024689 -
0.045365 -0.023183 0.021371 -0.050510 -0.041889 -0.010868 -
0.030100 -0.022084
y350 0.018111 0.001577 -0.013768 -0.009957 0.029197
0.011706 0.001111 0.001114 -0.036831 0.002187 0.011557
0.041259 0.009801 0.000540 0.026830 -0.031388 0.005870
0.000806 -0.002805 0.046779 0.026360 0.018708 0.014536 -
0.015034 0.028700 -0.042272 -0.017729 -0.021261 -0.037842
0.005593 -0.008099 0.037269 -0.012152 -0.010792 -0.015047
0.034780 0.046258
z350 -0.011806 -0.007553 0.007728 -0.004685 -0.011852
0.005692 0.003120 0.016719 -0.003605 -0.017281 0.004879
0.031055 0.021140 -0.007425 0.014299 0.028084 -0.015899 -
0.018448 -0.004239 0.014212 -0.010832 0.016390 -0.004489
0.009218 0.024617 0.027265 -0.022878 -0.036676 -0.051033 -
0.007687 0.010070 0.018949 0.012557 0.026662 -0.023327 -
0.004209 -0.016768
x351 0.029750 0.046696 -0.004784 0.018590 0.003974
0.007623 -0.014446 -0.023749 0.018294 -0.023083 -0.003743
0.028529 -0.014805 0.058407 0.010707 -0.067255 0.010007
0.022058 -0.027852 0.021848 0.023340 0.004500 -0.000677
0.006534 0.003532 -0.037859 0.034186 0.001986 0.028582 -
0.033665 -0.021355 0.021077 -0.049579 -0.041591 -0.008261 -
0.021750 -0.020067
y351 0.017791 0.001611 -0.011175 -0.009110 0.028742
0.014837 0.002260 0.000606 -0.031522 -0.000217 0.012664
0.038121 0.014353 -0.001131 0.026907 -0.028581 0.005558
0.001278 -0.007159 0.041981 0.025251 0.018505 0.017645 -
0.008465 0.027059 -0.037872 -0.015058 -0.021055 -0.038788
0.008541 -0.006183 0.041066 -0.005931 -0.003361 -0.012857
0.027331 0.038223
z351 -0.013764 -0.010746 0.007818 -0.008348 -0.011594
0.002760 0.005654 0.019950 -0.002729 -0.021015 0.007482
0.025856 0.027227 -0.012286 0.014674 0.032487 -0.015070 -
0.017561 0.002171 0.015289 -0.015558 0.011709 -0.010582 -
0.000297 0.028651 0.029479 -0.027141 -0.037766 -0.046327 -
0.011911 0.014114 0.014093 0.007997 0.029389 -0.017692 -
0.008761 -0.013444
x352 0.029992 0.050665 -0.001757 0.017831 -0.004243
0.005865 -0.013796 -0.035042 0.022617 -0.025394 0.002971

0.020000	-0.002690	0.053163	0.009083	-0.064350	0.002553	
0.017756	-0.020982	0.017129	0.023609	-0.000694	0.002771	
0.016134	0.015993	-0.042701	0.024623	-0.010921	0.030826	-
0.021178	-0.023926	0.021675	-0.044950	-0.043865	-0.005567	-
0.008705	-0.016635					
y352	0.016109	0.001004	-0.009176	-0.010307	0.027174	
0.015163	0.002835	-0.002676	-0.026115	-0.004512	0.014374	
0.029820	0.018115	-0.001416	0.026304	-0.023209	0.002721	-
0.001499	-0.007838	0.041577	0.023511	0.015491	0.022544	-
0.003178	0.026805	-0.033069	-0.016694	-0.023353	-0.035112	
0.012213	-0.004197	0.045551	0.001476	0.008727	-0.006944	
0.013620	0.027323					
z352	-0.015866	-0.014352	0.007600	-0.011276	-0.008699	
0.001607	0.012414	0.025245	-0.005758	-0.019680	0.004604	
0.024982	0.030552	-0.014699	0.014225	0.033427	-0.011977	-
0.019699	-0.002072	0.014345	-0.022371	0.005109	-0.015899	-
0.008739	0.022348	0.032069	-0.020908	-0.037849	-0.045882	-
0.017803	0.009447	0.010970	0.008142	0.020563	-0.020293	-
0.013668	-0.012124					
x353	0.030425	0.053540	0.000305	0.017602	-0.012760	
0.003690	-0.014191	-0.045616	0.025408	-0.027097	0.010933	
0.010900	0.006541	0.046555	0.006539	-0.056789	-0.004388	
0.013564	-0.014450	0.010476	0.022421	-0.003638	0.008821	
0.026783	0.031729	-0.041287	0.011568	-0.017883	0.035896	-
0.010129	-0.025959	0.023351	-0.037347	-0.036834	0.004922	-
0.000067	-0.010013					
y353	0.013281	-0.000517	-0.005752	-0.011099	0.025596	
0.016369	0.003504	-0.005152	-0.019217	-0.010490	0.016328	
0.021213	0.022890	-0.000885	0.025874	-0.015735	-0.001376	-
0.005075	-0.007489	0.039482	0.019129	0.009919	0.027953	
0.002874	0.028403	-0.027863	-0.018394	-0.026022	-0.032871	
0.017292	-0.001166	0.050229	0.009847	0.020317	-0.002586	-
0.000498	0.014315					
z353	-0.017026	-0.019137	0.008714	-0.014210	-0.005431	
0.000151	0.016507	0.031189	-0.010867	-0.021378	0.002002	
0.024230	0.028922	-0.016812	0.011002	0.038628	-0.008065	-
0.018238	-0.007066	0.013205	-0.027705	0.001979	-0.019836	-
0.012924	0.016316	0.039744	-0.012929	-0.024991	-0.040322	-
0.025639	0.003705	0.004728	0.005839	0.022477	-0.021815	-
0.024917	-0.013811					
x354	0.030096	0.046295	0.001277	0.013006	-0.014921	
0.003773	-0.008607	-0.041819	0.018826	-0.027419	0.008933	
0.007134	0.018514	0.048569	0.014305	-0.048585	-0.002827	
0.012767	-0.009217	0.010969	0.003916	-0.000815	0.013192	
0.021765	0.027467	-0.031161	0.021439	-0.022289	0.020456	-
0.012108	-0.029517	0.017694	-0.027696	-0.033612	-0.012975	-
0.002161	-0.005381					
y354	0.008406	-0.005042	-0.000601	-0.012674	0.021869	
0.016608	0.003003	-0.002037	-0.016190	-0.011935	0.014660	
0.025530	0.031409	-0.000479	0.024274	-0.008155	0.004845	-
0.010166	-0.007779	0.034111	0.010288	0.005829	0.031199	-
0.003865	0.021360	-0.015267	-0.022019	-0.008542	-0.019531	
0.018443	-0.004449	0.033903	0.015654	0.023518	0.005362	-
0.004048	0.010873					
z354	-0.016247	-0.019920	0.008684	-0.018027	-0.004438	-
0.000054	0.020246	0.032709	-0.012622	-0.025373	0.006521	
0.017796	0.023252	-0.020071	0.006926	0.039435	-0.011246	-
0.014310	-0.006314	0.001855	-0.025260	-0.000268	-0.020667	-
0.014417	0.013322	0.038980	-0.017394	-0.019661	-0.028620	-

0.022996 0.006685 -0.001016 0.012220 0.011821 -0.009451 -
0.018504 -0.014044
x355 0.030458 0.041186 0.000999 0.006868 -0.014739
0.005148 -0.001023 -0.039228 0.016791 -0.026237 0.010714
0.003545 0.028467 0.044784 0.019501 -0.044511 0.003377
0.008953 -0.004721 0.011546 -0.013267 -0.001638 0.020505
0.018472 0.026661 -0.014373 0.023662 -0.022278 0.010362 -
0.013704 -0.034196 0.017374 -0.020236 -0.023655 -0.020116 -
0.008852 0.005303
y355 0.003933 -0.008942 0.003523 -0.013556 0.017817
0.015033 0.002712 0.001891 -0.013969 -0.014142 0.012438
0.027499 0.037233 -0.001101 0.023607 -0.001522 0.008355 -
0.013093 -0.007079 0.028921 0.002618 0.003064 0.032519 -
0.009680 0.015872 -0.005796 -0.022609 0.004683 -0.010596
0.020705 -0.006294 0.023081 0.018974 0.027933 0.011986 -
0.006199 0.008408
z355 -0.015109 -0.018445 0.007323 -0.022015 -0.002516 -
0.000159 0.025791 0.031748 -0.011096 -0.027631 0.012312
0.009579 0.018396 -0.025867 0.002468 0.036668 -0.012824 -
0.013161 -0.005575 -0.005958 -0.022235 -0.007049 -0.019114 -
0.012924 0.013639 0.041067 -0.021190 -0.015031 -0.017798 -
0.018245 0.007042 -0.002828 0.015087 0.012202 0.004270 -
0.013029 -0.009130
x356 0.029046 0.034540 0.000509 0.001085 -0.014740
0.006303 0.004575 -0.036639 0.012330 -0.026426 0.011734 -
0.002787 0.036405 0.041333 0.019241 -0.036146 0.005846
0.006164 -0.004533 0.012783 -0.022882 -0.004351 0.021404
0.015938 0.030713 -0.003440 0.027100 -0.027283 0.003914 -
0.017607 -0.034248 0.015436 -0.011121 -0.020848 -0.026665 -
0.016670 0.010802
y356 -0.000057 -0.011812 0.007318 -0.015347 0.014385
0.013202 0.002978 0.004696 -0.013252 -0.016062 0.011664
0.027463 0.041069 -0.001030 0.020428 0.004179 0.010883 -
0.015285 -0.007845 0.025485 -0.001763 -0.001889 0.033445 -
0.013767 0.010495 0.002581 -0.021363 0.013570 -0.001092
0.020788 -0.008493 0.016104 0.021539 0.028091 0.018536 -
0.010555 0.006148
z356 -0.015219 -0.018370 0.005177 -0.023546 -0.001827 -
0.001504 0.027634 0.030063 -0.012076 -0.028965 0.015614
0.001000 0.013133 -0.028146 -0.004556 0.036965 -0.016799 -
0.010707 -0.007941 -0.012826 -0.013923 -0.014220 -0.022298 -
0.010255 0.017834 0.041130 -0.022749 -0.016624 -0.007618 -
0.017645 0.009697 -0.005769 0.019140 0.007905 0.011791 -
0.009777 -0.008317
x357 0.028091 0.028500 0.000139 -0.001613 -0.016699
0.008580 0.006973 -0.029313 0.009292 -0.025767 0.013658 -
0.006816 0.039596 0.037223 0.023331 -0.024579 0.002580
0.003437 -0.003977 0.010038 -0.032040 -0.003770 0.018214
0.012627 0.031383 0.009503 0.028223 -0.030202 -0.003944 -
0.019091 -0.029525 0.011764 -0.005012 -0.009060 -0.030287 -
0.019340 0.012081
y357 -0.003737 -0.013373 0.010823 -0.016681 0.010638
0.011552 0.003668 0.008080 -0.012068 -0.017078 0.012414
0.026939 0.042859 -0.000896 0.018665 0.008036 0.011900 -
0.014871 -0.006758 0.020076 -0.005857 -0.005549 0.033430 -
0.016690 0.005575 0.009452 -0.019983 0.022723 0.004966
0.022098 -0.006771 0.011103 0.023487 0.031317 0.022429 -
0.016095 0.003553
z357 -0.014537 -0.017336 0.003094 -0.022190 -0.002589 -
0.001674 0.028070 0.030340 -0.012180 -0.027721 0.018426 -

0.003833	0.006745	-0.027767	-0.007880	0.036772	-0.022065	-
0.009072	-0.009786	-0.019869	-0.007248	-0.017436	-0.028844	-
0.007602	0.017025	0.042085	-0.023698	-0.019215	-0.001547	-
0.017675	0.014227	-0.009501	0.021160	0.009967	0.017076	-
0.002635	-0.011607					
x358	0.026449	0.020817	0.000122	-0.007163	-0.018341	
0.010571	0.011206	-0.024962	0.007224	-0.024061	0.015854	-
0.006274	0.042948	0.031730	0.025828	-0.017298	0.002335	-
0.000025	-0.003452	0.001871	-0.043442	-0.006038	0.013585	
0.004283	0.031585	0.014562	0.025810	-0.034227	-0.004666	-
0.016339	-0.036955	0.012098	-0.000554	-0.006186	-0.028430	-
0.023862	0.010674					
y358	-0.006734	-0.014036	0.012967	-0.017471	0.007080	
0.008939	0.004206	0.011004	-0.012111	-0.018978	0.013205	
0.023607	0.041795	-0.000694	0.016107	0.011957	0.011638	-
0.013285	-0.004993	0.014905	-0.008819	-0.009256	0.032980	-
0.018348	0.002277	0.013740	-0.016709	0.027843	0.009770	
0.022662	-0.004484	0.010799	0.024720	0.031734	0.023662	-
0.022353	0.002935					
z358	-0.013842	-0.016642	0.001149	-0.021761	-0.003193	-
0.001884	0.027420	0.027987	-0.011770	-0.026037	0.021406	-
0.007145	0.000518	-0.026236	-0.011694	0.035886	-0.024206	-
0.009216	-0.008945	-0.026294	-0.002943	-0.018583	-0.031688	-
0.010031	0.016371	0.038328	-0.023308	-0.022745	0.006685	-
0.018041	0.012257	-0.008122	0.022114	0.009344	0.024631	-
0.000445	-0.012419					
x359	0.025687	0.013678	-0.002154	-0.010456	-0.018394	
0.012861	0.009833	-0.018029	0.003512	-0.026764	0.014957	-
0.008142	0.036497	0.022979	0.025599	0.000924	0.002163	-
0.006350	-0.007590	-0.005251	-0.047725	-0.004557	0.005727	-
0.001136	0.038461	0.021304	0.018411	-0.033067	-0.004318	-
0.019599	-0.037650	0.012173	-0.000024	-0.009201	-0.020537	-
0.020454	0.006840					
y359	-0.008751	-0.013704	0.014425	-0.019203	0.004556	
0.005907	0.005655	0.014377	-0.012698	-0.020493	0.013696	
0.018085	0.038209	0.000549	0.011056	0.014395	0.012211	-
0.011724	-0.003111	0.010483	-0.010552	-0.014286	0.030972	-
0.016775	-0.001713	0.015123	-0.012642	0.030158	0.014351	
0.021590	-0.001042	0.011536	0.023881	0.029597	0.024334	-
0.030567	0.001408					
z359	-0.012424	-0.015463	-0.001754	-0.021085	-0.003450	-
0.000846	0.026198	0.024969	-0.011256	-0.023061	0.023211	-
0.009237	-0.007293	-0.023090	-0.016466	0.034426	-0.021660	-
0.009285	-0.007789	-0.031257	0.003644	-0.020694	-0.036442	-
0.011030	0.015071	0.036195	-0.025339	-0.024965	0.010805	-
0.020786	0.011882	-0.008369	0.022858	0.008739	0.026130	
0.007157	-0.016767					
x360	0.024605	0.007013	-0.001275	-0.016988	-0.017897	
0.019202	0.013515	-0.015046	0.004510	-0.028803	0.018047	-
0.009788	0.034378	0.016151	0.026992	0.008877	0.001360	-
0.010663	-0.005994	-0.012107	-0.054818	-0.005307	-0.002992	-
0.018657	0.040439	0.020367	0.014601	-0.028935	0.005815	-
0.023763	-0.042833	0.008880	0.007963	-0.011171	-0.022337	-
0.007144	0.008407					
y360	-0.011338	-0.012134	0.015914	-0.020453	0.000942	-
0.000159	0.006723	0.017961	-0.013945	-0.023062	0.013103	
0.011376	0.031814	0.003533	0.005153	0.019389	0.011105	-
0.007498	-0.001440	0.007446	-0.008225	-0.015841	0.029790	-
0.008373	-0.004716	0.014701	-0.003802	0.028524	0.015889	

0.022476 0.004602 0.013830 0.019039 0.034231 0.022110 -
0.036002 0.002864
z360 -0.010333 -0.013943 -0.005720 -0.018739 -0.003852
0.002448 0.024998 0.019016 -0.010046 -0.019279 0.026437 -
0.012560 -0.013564 -0.018396 -0.016671 0.028382 -0.019818 -
0.009658 -0.003206 -0.034904 0.006271 -0.020952 -0.042382 -
0.020936 0.012324 0.031387 -0.023297 -0.028222 0.014833 -
0.027324 0.005286 -0.005309 0.028869 0.005539 0.027528
0.016568 -0.020505
x361 0.024057 0.000952 -0.000939 -0.025583 -0.016389
0.025736 0.018841 -0.010918 0.005257 -0.028613 0.024610 -
0.012422 0.029664 0.010985 0.020494 0.016762 0.004367 -
0.014330 -0.006035 -0.015664 -0.053711 -0.008820 -0.009546 -
0.024340 0.037565 0.023025 0.009640 -0.024993 0.006616 -
0.022159 -0.038853 0.007665 0.015419 -0.009003 -0.011263 -
0.009181 0.005534
y361 -0.016881 -0.006946 0.022856 -0.025578 -0.005778 -
0.014340 0.006241 0.025686 -0.016321 -0.023821 0.005866
0.007988 0.022687 0.014518 -0.019122 0.035188 0.017023
0.000490 -0.004242 0.013035 0.012325 -0.010128 0.040561
0.038465 -0.014439 0.016553 0.012483 0.018967 0.015159
0.033659 0.026947 0.011180 -0.006551 0.071657 0.014126 -
0.049268 0.004536
z361 -0.006113 -0.013474 -0.014674 -0.012317 -0.003442
0.010007 0.022794 0.010082 -0.009038 -0.014914 0.032150 -
0.018059 -0.020202 -0.016258 -0.007526 0.015791 -0.021189 -
0.011118 0.004431 -0.043534 0.000821 -0.023226 -0.055526 -
0.050938 0.012482 0.023960 -0.023710 -0.031120 0.014157 -
0.040812 -0.011014 0.004465 0.045394 -0.016805 0.032340
0.028172 -0.030330
x362 0.023251 -0.002739 0.001037 -0.036631 -0.015517
0.033524 0.022867 -0.002302 0.009276 -0.023252 0.035197 -
0.011164 0.022363 0.011149 0.012175 0.020412 0.016204 -
0.013202 0.001145 -0.020609 -0.050660 -0.004532 -0.007077 -
0.028240 0.031304 0.030728 -0.000756 -0.022046 0.001008 -
0.015787 -0.030273 -0.002121 0.017140 0.009979 -0.002030 -
0.015061 -0.002473
y362 -0.014107 -0.007960 0.011587 -0.018296 -0.005624 -
0.009682 0.008748 0.022277 -0.019543 -0.030309 0.013453 -
0.013016 0.011556 0.005393 0.001236 0.022541 -0.000070
0.007808 0.007741 -0.007137 -0.007993 -0.017489 0.017680 -
0.005151 -0.005513 0.005249 0.016939 0.018235 0.011837
0.018410 0.010735 0.035516 0.015925 0.019086 0.017260 -
0.049219 0.003339
z362 -0.008901 -0.008585 -0.011281 -0.012405 -0.009465
0.001655 0.015792 0.009018 -0.008498 -0.008029 0.022573 -
0.011004 -0.028522 0.002573 -0.031049 0.028455 -0.007645 -
0.010683 0.001737 -0.033887 0.026197 -0.011456 -0.047973 -
0.011325 0.000543 0.024288 -0.012051 -0.044394 0.015329 -
0.034774 0.000051 -0.004090 0.021654 0.028739 0.020925
0.030650 -0.033294
x363 0.020983 -0.004562 0.003014 -0.041579 -0.016242
0.031355 0.024484 0.006143 0.011883 -0.022760 0.033012 -
0.011922 0.013881 0.011248 0.005258 0.030791 0.018990 -
0.007316 -0.001250 -0.026456 -0.035874 -0.002979 -0.007746 -
0.016782 0.026296 0.026788 -0.001350 -0.020995 -0.000440 -
0.006356 -0.013097 -0.008802 0.012259 0.027506 -0.001910 -
0.023212 -0.005091
y363 -0.023197 0.000655 0.026651 -0.031516 -0.014596 -
0.026872 0.008522 0.035540 -0.018122 -0.027686 0.006535

0.001464	0.006222	0.026643	-0.042127	0.045797	0.030867	
0.010102	0.000428	0.020694	0.027682	-0.002062	0.041608	
0.078226	-0.031749	0.023418	0.029132	0.005408	0.011742	
0.032259	0.049793	0.004698	-0.024970	0.094806	0.001482	-
0.056052	0.005158					
z363	-0.009741	-0.004455	-0.010956	-0.009000	-0.011849	-
0.005477	0.009506	0.007629	-0.006580	0.002721	0.013994	-
0.004535	-0.020045	-0.000921	-0.027381	0.023216	-0.010766	-
0.009550	0.005786	-0.021066	0.021259	-0.002119	-0.048409	-
0.010323	-0.002366	0.022338	-0.014003	-0.032439	0.006611	-
0.033800	-0.007352	-0.008610	0.009375	0.017889	0.018570	
0.027324	-0.019188					
x364	0.021122	-0.007694	0.002408	-0.043919	-0.017432	
0.033475	0.027176	0.008372	0.014970	-0.023743	0.031930	-
0.013821	0.009204	0.004699	0.010055	0.033745	0.016302	-
0.006670	-0.000418	-0.033092	-0.033891	0.003176	-0.018452	-
0.026282	0.026880	0.022814	-0.007377	-0.010757	0.002878	-
0.007560	-0.018023	-0.004558	0.017245	0.014385	0.006507	-
0.025409	-0.000953					
y364	-0.017059	-0.001649	0.011958	-0.023998	-0.011368	-
0.010181	0.013289	0.027370	-0.018272	-0.032764	0.027450	-
0.012086	0.003393	0.009805	-0.007023	0.016896	0.020166	
0.004145	0.014904	0.006949	-0.010776	-0.012994	0.006438	-
0.001147	-0.024784	0.017051	0.021173	0.014300	0.012078	-
0.000643	0.017162	0.020959	0.018688	0.006237	0.016972	-
0.038174	-0.007425					
z364	-0.011100	0.000705	-0.010068	-0.004330	-0.013489	-
0.013617	0.002951	0.006700	-0.005972	0.011843	0.005018	
0.002032	-0.012935	-0.000796	-0.027814	0.019224	-0.012382	-
0.007325	0.007407	-0.005801	0.020067	0.003590	-0.044985	-
0.000866	-0.006263	0.020938	-0.012774	-0.025934	-0.003776	-
0.030822	-0.009958	-0.017283	-0.004398	0.015623	0.011447	
0.027355	-0.008255					
x365	0.020255	-0.008408	0.002000	-0.047719	-0.016314	
0.035337	0.028885	0.010059	0.015854	-0.021649	0.033705	-
0.011334	0.007619	0.000743	0.005678	0.032981	0.017115	-
0.002687	0.002359	-0.030243	-0.030979	0.007513	-0.021884	-
0.026080	0.020280	0.026709	-0.009672	-0.006682	0.001032	-
0.009494	-0.018626	-0.012625	0.016068	0.005356	0.010691	-
0.023868	0.000813					
y365	-0.019644	0.001640	0.013337	-0.025092	-0.015585	-
0.013601	0.013528	0.028666	-0.018183	-0.034777	0.027384	-
0.011510	-0.000787	0.012941	-0.012970	0.018703	0.025670	
0.001815	0.014982	0.014281	-0.009574	-0.008369	0.004116	
0.009856	-0.031720	0.021225	0.025583	0.012180	0.010030	-
0.004886	0.020983	0.015767	0.011703	0.006761	0.014303	-
0.035562	-0.010916					
z365	-0.011866	0.004903	-0.007951	0.000831	-0.016054	-
0.022361	-0.003338	0.006378	-0.004606	0.019775	-0.004893	
0.007187	-0.007170	0.000298	-0.025640	0.015720	-0.012508	-
0.006669	0.006643	0.004724	0.021809	0.007870	-0.040902	
0.007240	-0.006493	0.014764	-0.013351	-0.018535	-0.009200	-
0.025258	-0.011995	-0.019329	-0.016460	0.019478	0.005904	
0.020760	0.002942					
x366	0.019124	-0.008445	0.004662	-0.050795	-0.018572	
0.033841	0.028636	0.013900	0.016024	-0.019299	0.032884	-
0.010068	0.006407	-0.000778	0.002719	0.031205	0.019513	-
0.003873	0.001354	-0.026098	-0.026852	0.008854	-0.027208	-
0.020834	0.014134	0.025218	-0.010689	-0.001170	-0.001581	-

0.012235 -0.024170 -0.019117 0.012738 -0.002864 0.011629 -
0.028304 -0.000243
y366 -0.020392 0.003030 0.010331 -0.023479 -0.016975 -
0.013162 0.013206 0.026809 -0.017546 -0.037435 0.030019 -
0.014749 -0.004766 0.011964 -0.010937 0.017788 0.022668 -
0.001556 0.018081 0.015958 -0.015588 -0.003479 -0.002828
0.001457 -0.032352 0.021855 0.026928 0.011798 0.010618 -
0.011476 0.019328 0.019201 0.014239 -0.006539 0.016482 -
0.031235 -0.012495
z366 -0.011816 0.008303 -0.011579 0.008512 -0.013937 -
0.027942 -0.008626 0.004045 -0.003202 0.027347 -0.009666
0.010736 -0.001729 0.000426 -0.023071 0.010836 -0.017269 -
0.003865 0.009114 0.010517 0.021707 0.009180 -0.035684
0.003100 -0.002559 0.013819 -0.018959 -0.012538 -0.014074 -
0.019501 -0.008578 -0.020136 -0.019753 0.018208 -0.000351
0.024185 0.014676
x367 0.016927 -0.007977 0.004604 -0.050924 -0.018965
0.029970 0.026776 0.017719 0.016134 -0.015467 0.029973 -
0.011731 0.006453 -0.000935 0.000138 0.029237 0.015668 -
0.003044 -0.000459 -0.023643 -0.021056 0.010949 -0.029915 -
0.013524 0.010650 0.025229 -0.013525 0.006776 -0.004279 -
0.010534 -0.024836 -0.025292 0.009912 -0.006546 0.005600 -
0.027137 0.003465
y367 -0.020891 0.004402 0.008963 -0.022106 -0.019212 -
0.012142 0.012601 0.023802 -0.017058 -0.039792 0.031435 -
0.014561 -0.008881 0.009712 -0.010361 0.015892 0.021962 -
0.003828 0.022605 0.018765 -0.019629 0.001381 -0.006968 -
0.002992 -0.032356 0.021573 0.026609 0.009328 0.009307 -
0.015112 0.015660 0.020328 0.015051 -0.013685 0.018483 -
0.028644 -0.012766
z367 -0.010655 0.011671 -0.012890 0.014726 -0.013629 -
0.031891 -0.013303 -0.000268 -0.003984 0.031048 -0.015763
0.015929 0.001344 -0.001989 -0.019556 0.004227 -0.016463 -
0.000780 0.010465 0.018194 0.021490 0.008399 -0.029723 -
0.001835 0.001037 0.011732 -0.020847 -0.013581 -0.015651 -
0.015644 -0.008042 -0.018027 -0.024682 0.016302 0.002064
0.019784 0.025139
x368 0.015238 -0.007735 0.003106 -0.050145 -0.019233
0.025362 0.024205 0.019671 0.015116 -0.012634 0.027527 -
0.011342 0.006155 -0.001597 -0.003174 0.026932 0.011588 -
0.001630 -0.000223 -0.019363 -0.013465 0.014441 -0.031047 -
0.006514 0.008708 0.028727 -0.016182 0.009550 -0.009155 -
0.006649 -0.025715 -0.030625 0.004736 -0.006636 0.001595 -
0.027360 0.012986
y368 -0.021915 0.005213 0.008685 -0.019908 -0.021441 -
0.011623 0.011785 0.020580 -0.016765 -0.040864 0.029496 -
0.014622 -0.010759 0.007366 -0.010303 0.013972 0.018242 -
0.007335 0.025886 0.020915 -0.025060 0.006352 -0.010872 -
0.006919 -0.031159 0.018180 0.024427 0.008548 0.010682 -
0.016144 0.009981 0.024296 0.016000 -0.019450 0.017783 -
0.025894 -0.015229
z368 -0.009891 0.015467 -0.013604 0.021374 -0.012598 -
0.035398 -0.017360 -0.004548 -0.004759 0.035603 -0.022759
0.018605 0.005560 -0.002786 -0.018406 -0.003521 -0.015694
0.001426 0.008614 0.024975 0.020109 0.003224 -0.024153 -
0.006775 0.002984 0.009158 -0.023036 -0.010599 -0.013903 -
0.011731 -0.004471 -0.013739 -0.026825 0.012373 -0.000654
0.021441 0.027956
x369 0.013727 -0.007155 0.001203 -0.047373 -0.018887
0.021096 0.021000 0.019519 0.012590 -0.009493 0.023283 -

0.012370	0.009198	-0.002795	-0.007676	0.021921	0.005144	-
0.002681	0.000192	-0.011677	-0.009652	0.015227	-0.032222	-
0.005323	0.007558	0.030855	-0.019724	0.010989	-0.012741	-
0.002865	-0.025345	-0.031805	0.001668	-0.008657	-0.004141	-
0.024918	0.019139					
y369	-0.023542	0.005623	0.008501	-0.017318	-0.024317	-
0.013088	0.010192	0.017557	-0.016121	-0.041642	0.026249	-
0.011952	-0.013324	0.006171	-0.012184	0.015223	0.012480	-
0.009390	0.029409	0.021452	-0.026083	0.014944	-0.012117	-
0.002675	-0.029327	0.016597	0.022400	0.008549	0.010612	-
0.012787	0.000956	0.026708	0.011675	-0.017603	0.013464	-
0.024279	-0.011844					
z369	-0.009045	0.018558	-0.015504	0.029019	-0.011214	-
0.039281	-0.020935	-0.007602	-0.004619	0.040184	-0.025710	
0.022743	0.007701	-0.000928	-0.017878	-0.009932	-0.014051	
0.007595	0.007708	0.026802	0.023679	-0.000729	-0.016054	-
0.006041	0.005126	0.009924	-0.027822	-0.007227	-0.013828	-
0.007900	-0.005393	-0.013121	-0.025551	0.014906	-0.006463	
0.023449	0.036942					
x370	0.011392	-0.006600	-0.000736	-0.042690	-0.018988	
0.015165	0.017372	0.018166	0.009026	-0.005485	0.018608	-
0.012793	0.014310	-0.003074	-0.013381	0.015041	-0.003189	-
0.001741	0.002531	-0.002964	-0.005360	0.016076	-0.032032	-
0.001964	0.003175	0.036737	-0.024965	0.014404	-0.016264	
0.004490	-0.027872	-0.031559	-0.002625	-0.008139	-0.013059	-
0.018283	0.034064					
y370	-0.024028	0.006165	0.006511	-0.013345	-0.026496	-
0.013902	0.006662	0.014741	-0.014893	-0.042945	0.023712	-
0.011480	-0.017986	0.002233	-0.012464	0.019254	0.003613	-
0.014715	0.031480	0.022342	-0.028069	0.024452	-0.013238	-
0.001264	-0.020672	0.012420	0.019739	0.008604	0.009377	-
0.012301	-0.004348	0.029694	0.009670	-0.017711	0.011754	-
0.024787	-0.014878					
z370	-0.006588	0.022212	-0.018387	0.035419	-0.008598	-
0.041673	-0.025062	-0.009565	-0.004666	0.042649	-0.027484	
0.025304	0.006841	0.000601	-0.016691	-0.014147	-0.011338	
0.008837	0.002180	0.030228	0.027108	-0.008529	-0.007559	-
0.009282	0.013485	0.008163	-0.028902	-0.007814	-0.010582	-
0.009024	0.002926	-0.010812	-0.023772	0.013389	-0.006217	
0.021237	0.035277					
x371	0.009431	-0.005182	-0.004601	-0.036440	-0.019278	
0.008600	0.012381	0.015885	0.005345	-0.000389	0.013972	-
0.013831	0.019061	-0.004938	-0.018642	0.006409	-0.013080	-
0.003561	0.004114	0.005367	-0.001124	0.020159	-0.031620	
0.001287	0.003813	0.044057	-0.032613	0.015193	-0.020497	
0.008859	-0.033340	-0.032427	-0.006474	-0.008070	-0.024246	-
0.012018	0.046300					
y371	-0.025622	0.005389	0.007697	-0.011430	-0.027247	-
0.013481	0.002846	0.011164	-0.013591	-0.045374	0.017232	-
0.009900	-0.019283	0.002454	-0.011216	0.025347	-0.006464	-
0.017434	0.032798	0.024327	-0.030360	0.031679	-0.011150	-
0.000150	-0.015292	0.000077	0.021419	0.007629	0.009003	-
0.006137	-0.009843	0.032219	0.003955	-0.015781	0.011021	-
0.032592	-0.007950					
z371	-0.004751	0.024886	-0.019962	0.041507	-0.004207	-
0.041102	-0.027712	-0.012007	-0.005497	0.042130	-0.030632	
0.027340	0.007633	0.005326	-0.013295	-0.019049	-0.007977	
0.014670	-0.004953	0.032556	0.029989	-0.022640	0.000897	-
0.017135	0.018843	0.002839	-0.025725	-0.011033	-0.005395	-

0.007910	0.013874	-0.009350	-0.020413	0.007899	-0.004789	
0.017974	0.040045					
x372	0.012991	-0.004876	-0.016769	-0.020929	-0.014144	
0.007471	0.005878	0.009835	-0.000840	0.000309	0.008935	-
0.011404	0.019509	-0.010478	-0.005035	-0.010097	-0.024562	
0.001353	0.006879	0.001009	-0.004721	0.000403	-0.041653	-
0.028555	0.007684	0.029224	-0.033228	0.014976	-0.003741	
0.002912	-0.035592	-0.008498	0.001398	-0.048640	-0.016337	-
0.001978	0.027351					
y372	-0.034183	0.013707	0.016605	-0.020583	-0.030895	-
0.017258	0.004553	0.012451	-0.009189	-0.032273	0.003634	
0.011854	-0.025107	0.018747	-0.038987	0.041773	0.012802	-
0.014375	0.027704	0.042897	-0.004545	0.052259	0.012431	
0.070582	-0.013197	0.006062	0.026809	-0.023605	0.006796	-
0.004601	0.011771	0.007894	-0.027678	0.055730	-0.008111	-
0.025394	0.004715					
z372	0.007691	0.020905	-0.032531	0.052950	0.005665	-
0.022548	-0.029175	-0.019429	-0.009516	0.029254	-0.030874	
0.006687	0.011569	-0.018812	0.016277	-0.026886	-0.021003	
0.011996	-0.002909	0.011994	0.007837	-0.035563	-0.015644	-
0.080777	0.038227	-0.015344	-0.025871	0.000410	-0.002729	-
0.016520	-0.002688	0.004174	0.021972	-0.055919	0.022955	
0.011152	0.003244					
x373	0.012725	-0.000845	-0.018723	-0.013794	-0.012854	-
0.000969	-0.000612	0.009150	-0.004008	0.003772	-0.003436	-
0.002775	0.019268	-0.005848	-0.016227	-0.009559	-0.023989	
0.005852	0.002360	0.005229	0.012499	-0.007894	-0.033329	-
0.008070	0.005724	0.021586	-0.026306	0.006660	0.006404	
0.009813	-0.019095	-0.009567	-0.016345	-0.033052	-0.015446	-
0.002993	0.011928					
y373	-0.031695	0.013086	0.012586	-0.016613	-0.026634	-
0.006184	0.010328	0.004907	-0.007415	-0.028001	0.003151	
0.011215	-0.030420	0.013280	-0.015781	0.030652	0.006647	-
0.017126	0.032354	0.034578	-0.016272	0.047664	0.000404	
0.042257	-0.000303	-0.005029	0.021094	-0.031306	0.010555	-
0.019673	-0.000667	0.009203	-0.000028	0.041586	-0.004681	
0.000266	0.012905					
z373	0.011804	0.022978	-0.024886	0.051772	0.007342	-
0.024214	-0.029915	-0.015855	-0.013422	0.020416	-0.044247	-
0.001977	0.013526	-0.021349	0.009222	-0.011556	-0.012629	
0.010293	-0.010417	0.011776	0.023945	-0.036499	-0.004160	-
0.058504	0.036044	-0.015271	-0.016084	0.010737	-0.001435	
0.003761	0.019393	-0.008366	0.021231	-0.028260	0.034328	
0.000356	-0.015890					
x374	0.014299	0.000982	-0.022538	-0.004777	-0.010161	-
0.006190	-0.005470	0.008151	-0.006697	0.005299	-0.012581	
0.000285	0.019879	-0.006193	-0.017735	-0.012426	-0.025243	
0.008182	-0.000671	0.005680	0.018622	-0.021627	-0.032351	-
0.001957	0.005144	0.011077	-0.022522	0.004896	0.015843	
0.010053	-0.010276	-0.009062	-0.021732	-0.033344	-0.010091	-
0.004370	0.000019					
y374	-0.030999	0.013108	0.012874	-0.015446	-0.025814	-
0.001047	0.012642	0.000132	-0.006546	-0.019360	0.001363	
0.016470	-0.028847	0.015255	-0.010188	0.026060	0.007954	-
0.019683	0.026221	0.033050	-0.012665	0.038155	-0.003262	
0.041863	0.005078	-0.010073	0.013393	-0.030367	0.011775	-
0.028828	-0.005424	0.004377	0.010351	0.047171	-0.008602	
0.012271	0.028757					
z374	0.015990	0.022132	-0.022437	0.051588	0.009117	-
0.020495	-0.030181	-0.015397	-0.015940	0.012826	-0.048164	-

0.010130	0.016719	-0.025899	0.011209	-0.005037	-0.010868	
0.007428	-0.015743	0.007296	0.027462	-0.041891	-0.002867	-
0.056453	0.038268	-0.017788	-0.011985	0.022075	-0.001852	
0.011878	0.023078	-0.012120	0.030212	-0.028631	0.042731	-
0.007955	-0.027392					
x375	0.014724	0.003593	-0.021810	0.002062	-0.010400	-
0.011219	-0.010618	0.007543	-0.008493	0.006393	-0.020819	
0.004299	0.017911	-0.004428	-0.019029	-0.009654	-0.027639	
0.009819	-0.007915	0.009653	0.030764	-0.031638	-0.026719	
0.005740	-0.001493	0.009218	-0.018224	0.001585	0.020850	
0.017567	0.000133	-0.013103	-0.030372	-0.023119	-0.005746	-
0.009621	-0.012245					
y375	-0.026127	0.010553	0.007164	-0.009228	-0.020197	
0.011290	0.010376	-0.006651	-0.003007	-0.011122	0.005089	
0.013020	-0.026841	0.010574	0.004108	0.012004	0.001649	-
0.022852	0.029140	0.027718	-0.023959	0.034250	-0.011171	
0.014144	0.009079	-0.016229	0.001719	-0.028821	0.016316	-
0.045310	-0.017243	0.011821	0.023105	0.019403	-0.001497	
0.023232	0.029334					
z375	0.016323	0.021495	-0.017260	0.047294	0.010706	-
0.021504	-0.031745	-0.010735	-0.015072	0.006587	-0.051539	-
0.013737	0.017452	-0.021428	0.001561	0.009933	-0.005961	
0.005113	-0.018990	0.009317	0.036171	-0.034173	0.007566	-
0.030697	0.034166	-0.017636	-0.003963	0.024567	-0.000426	
0.022383	0.035860	-0.018316	0.021056	-0.008849	0.047086	-
0.017399	-0.034971					
x376	0.017184	0.004348	-0.024197	0.008399	-0.006803	-
0.013396	-0.014870	0.008199	-0.008502	0.007070	-0.027561	
0.002429	0.017613	-0.009847	-0.017901	-0.010617	-0.024541	
0.009646	-0.003502	0.012190	0.028658	-0.039740	-0.028662	
0.007478	-0.009341	0.001165	-0.009070	0.002608	0.026385	
0.017902	0.005132	-0.017657	-0.032752	-0.028042	0.001852	-
0.011875	-0.027298					
y376	-0.024235	0.010308	0.007291	-0.005568	-0.017497	
0.013615	0.008605	-0.013525	-0.002303	-0.003838	0.005952	
0.016273	-0.022949	0.011859	0.009018	0.005438	-0.003425	-
0.018595	0.020548	0.026521	-0.014763	0.028715	-0.015431	-
0.001585	0.009956	-0.013306	-0.001959	-0.028758	0.014547	-
0.046431	-0.016096	0.011901	0.020998	0.020481	-0.008222	
0.030502	0.028389					
z376	0.016994	0.019326	-0.015557	0.043420	0.013225	-
0.019443	-0.029393	-0.009310	-0.014031	0.001339	-0.045861	-
0.015403	0.018027	-0.019435	0.001981	0.014821	-0.007632	
0.002981	-0.020415	0.004553	0.035966	-0.032424	0.007998	-
0.027735	0.034562	-0.016833	0.001284	0.026086	-0.001854	
0.027509	0.037761	-0.020433	0.022588	-0.009017	0.049696	-
0.017193	-0.040710					
x377	0.023223	0.001643	-0.031479	0.015325	0.002862	-
0.012173	-0.019499	0.015764	-0.005829	0.007932	-0.038624	-
0.008445	0.021338	-0.023433	-0.023820	-0.012815	-0.008869	
0.004668	0.020389	0.016111	0.007154	-0.039999	-0.032753	
0.028079	-0.020123	-0.015616	0.002131	0.004237	0.037866	
0.007819	0.005539	-0.022211	-0.033642	-0.042094	0.017553	-
0.012341	-0.046037					
y377	-0.024835	0.013111	0.016543	-0.002121	-0.021821	
0.014735	0.011034	-0.029621	-0.004177	0.001347	0.011329	
0.024925	-0.022834	0.017991	0.024137	0.004465	-0.019032	-
0.015295	-0.014584	0.020227	0.013292	0.013156	-0.011372	-
0.035576	0.013549	0.004259	-0.017372	-0.026790	0.000496	-

0.034695	-0.014731	0.013925	0.031139	0.024477	-0.022453	
0.020529	0.030461					
z377	0.016798	0.016565	-0.013577	0.038106	0.014639	-
0.016223	-0.023699	-0.008589	-0.012952	-0.003304	-0.037441	-
0.013828	0.017280	-0.016992	0.005077	0.016246	-0.006926	
0.001749	-0.020778	-0.002230	0.031844	-0.031840	0.007123	-
0.024118	0.033907	-0.015644	0.002851	0.025475	-0.002923	
0.029402	0.034788	-0.025665	0.025095	-0.010696	0.053231	-
0.013986	-0.041450					
x378	0.023100	0.007379	-0.022736	0.022004	-0.000928	-
0.004786	-0.021782	0.003234	-0.007862	0.005860	-0.020873	-
0.010502	0.006626	-0.026753	0.005055	-0.002193	-0.020666	
0.000824	-0.006307	0.016804	0.023259	-0.055438	-0.021859	-
0.031707	-0.013568	-0.003813	0.006289	0.010002	0.026158	
0.011836	0.020196	-0.026563	-0.001867	-0.031154	0.029710	-
0.018853	-0.039458					
y378	-0.016805	-0.000731	0.005722	-0.005494	-0.013215	
0.008299	0.022207	-0.021056	-0.001838	0.014569	-0.026224	
0.032825	0.007473	0.011659	0.005508	-0.021139	0.005601	-
0.013192	0.000324	0.003575	-0.006355	0.009464	-0.043453	
0.020556	-0.008057	-0.013346	-0.035598	-0.037450	0.019337	-
0.024860	-0.057854	0.006537	0.001202	-0.000546	-0.027241	
0.028702	-0.011327					
z378	0.015283	0.009196	-0.013667	0.030876	0.015900	-
0.016650	-0.007352	-0.006721	-0.011026	-0.004522	-0.033266	-
0.009445	0.022187	-0.016303	0.009948	0.003943	-0.005584	
0.004018	-0.016364	-0.020133	0.016240	-0.034029	-0.009507	-
0.013492	0.028194	-0.016813	-0.001578	0.020039	-0.004164	
0.033488	0.010668	-0.033833	0.027110	-0.027138	0.053115	-
0.014229	-0.049991					
x379	0.029005	0.015033	-0.007252	0.012938	0.004498	
0.013668	-0.019077	0.001678	-0.001997	0.008477	0.008348	-
0.015382	0.013936	-0.026428	0.004269	-0.005710	0.000962	-
0.002585	0.009777	0.037407	0.026107	-0.079228	-0.000143	-
0.048726	-0.032715	-0.013923	0.032905	0.024780	0.014569	
0.015806	0.050033	-0.061103	0.018469	0.002780	0.032482	-
0.001759	-0.044524					
y379	-0.011717	-0.011077	-0.015325	0.028014	-0.007475	-
0.018432	0.012491	-0.018264	-0.014580	0.007284	-0.054866	
0.014305	-0.003129	-0.008589	0.021880	0.002955	-0.028025	-
0.015693	-0.037846	-0.026398	-0.001987	0.027274	-0.055537	
0.011690	0.003767	0.006387	-0.072371	-0.034614	0.039780	-
0.029902	-0.057396	0.029098	-0.001289	-0.030005	0.031883	-
0.036217	-0.005806					
z379	0.012418	0.009344	-0.008412	0.021374	0.012823	-
0.015974	-0.000719	-0.002665	-0.013539	-0.007430	-0.015671	-
0.000177	0.014693	-0.016142	0.007737	0.007258	0.011132	
0.005333	-0.014382	-0.019879	0.012957	-0.018712	0.001170	
0.012456	0.028023	0.003597	-0.006570	0.020976	0.000583	
0.026129	0.011823	-0.037361	0.029563	-0.002008	0.065899	-
0.030401	-0.033754					
x380	0.028447	0.006348	-0.017618	0.034979	-0.019792	
0.008277	0.011642	0.016355	-0.001457	0.013678	-0.019112	-
0.024545	0.025959	-0.018249	0.029132	0.008274	-0.001420	-
0.014370	-0.030112	-0.006425	0.017443	-0.074218	-0.004516	-
0.018616	-0.014699	0.010668	0.036373	-0.018081	0.021528	
0.021910	0.024291	-0.077950	-0.005976	-0.016892	0.055741	-
0.047185	-0.031416					
y380	0.000505	-0.000848	0.002439	0.024601	0.010553	
0.006405	-0.001912	-0.026154	-0.013172	0.001591	-0.002370	-

0.007001	0.013059	-0.013361	0.014201	-0.002485	-0.016327	-
0.010093	-0.005143	0.001956	0.007776	-0.001881	-0.016057	-
0.034715	-0.015621	-0.020317	-0.036690	0.013523	0.030407	-
0.025702	0.007043	-0.006539	0.014236	0.004195	0.026592	
0.010512	-0.007883					
z380	0.000226	0.011917	0.007389	-0.017868	0.058191	-
0.039580	-0.048064	-0.022237	-0.009174	-0.037665	-0.005226	
0.017509	-0.028291	-0.024840	-0.011933	0.006569	-0.014108	
0.016522	0.019518	0.026416	0.000187	-0.005234	-0.030292	-
0.005066	-0.030291	-0.033388	-0.023586	0.080686	-0.049338	
0.012289	-0.014695	-0.008513	0.059331	0.034038	0.031305	
0.003693	-0.070141					
x381	0.030678	0.009654	-0.004786	0.044623	0.015315	
0.010081	-0.021588	0.004797	-0.003428	-0.021116	-0.001411	-
0.068832	-0.003641	0.001586	0.046159	0.034894	-0.002405	-
0.044240	-0.007014	-0.011202	0.033100	-0.040916	0.024924	-
0.030603	-0.054754	-0.025877	0.061070	-0.003624	-0.008637	
0.021642	0.064788	-0.097739	-0.010713	0.038544	0.026873	-
0.010710	-0.037299					
y381	0.003705	-0.006496	0.008395	0.006183	0.022327	-
0.028761	-0.005157	-0.030525	-0.019418	-0.005540	-0.003649	
0.037123	0.027555	-0.062579	-0.024856	-0.026574	-0.028643	
0.033852	-0.006534	0.010205	0.002117	-0.025486	-0.055707	-
0.049913	0.004911	-0.033626	-0.054863	0.051703	0.031845	-
0.015268	-0.010563	0.029079	0.060110	0.016145	0.038726	
0.005674	-0.037181					
z381	-0.004366	0.011069	0.008257	-0.023918	0.047490	-
0.041225	-0.027949	-0.019942	-0.015903	-0.032962	-0.004289	
0.037750	-0.018883	-0.035861	-0.023000	-0.008196	-0.008395	
0.011268	0.018741	0.022756	-0.001246	0.012765	-0.032545	
0.005449	-0.028880	-0.010226	-0.035653	0.068653	-0.038531	
0.016916	-0.029583	0.022319	0.043251	0.031924	0.029708	-
0.009438	-0.054979					
x382	0.032538	-0.002345	0.003565	0.026988	0.024820	-
0.005792	-0.022850	0.001325	0.005342	-0.038309	-0.026807	-
0.055233	0.006619	0.004087	0.038804	0.013854	-0.026615	-
0.012169	-0.009903	-0.015288	0.006067	-0.054642	-0.007702	-
0.013754	-0.062236	-0.067685	0.045505	-0.013959	-0.019674	
0.035865	0.001648	-0.092596	0.014853	0.025129	-0.010566	
0.038103	-0.084758					
y382	-0.001705	-0.002608	0.006570	0.005950	0.029421	-
0.027089	-0.007460	-0.033244	-0.012462	-0.011579	0.010843	
0.016139	0.007668	-0.029168	-0.003650	0.001095	0.000882	
0.015525	-0.011223	-0.009252	0.016171	0.002075	-0.035089	-
0.037758	0.005101	-0.044023	-0.037277	0.054888	0.021345	-
0.026105	0.022908	0.006570	0.050263	-0.001495	0.048063	-
0.011996	-0.003141					
z382	-0.010759	0.011146	-0.002345	-0.034059	0.027410	-
0.028364	-0.018258	-0.013495	-0.000564	-0.023626	0.001775	
0.047256	-0.017343	-0.008667	-0.018107	-0.004935	0.000918	
0.018317	0.012233	0.028829	-0.018592	0.025780	-0.024815	
0.026213	0.006199	0.006408	-0.037732	0.057753	-0.031299	-
0.009205	-0.032175	0.027184	0.053197	-0.004663	0.030617	-
0.012618	-0.013581					
x383	0.035663	-0.004502	0.003447	0.015901	0.033040	-
0.005639	-0.024439	0.001586	0.004917	-0.035492	-0.022281	-
0.037990	-0.000105	-0.004625	0.025662	0.006972	-0.009409	-
0.005523	-0.005218	-0.009940	-0.017161	-0.033559	-0.002406	-
0.001327	-0.074829	-0.036247	0.000524	-0.010672	-0.016387	

0.016778 -0.005150 -0.073973 0.040679 0.022189 0.028179 -
0.007026 -0.099637
y383 -0.003984 -0.003525 0.008429 0.000297 0.032325 -
0.033239 -0.013804 -0.031395 -0.006929 -0.024830 0.007193
0.010350 -0.001196 -0.015148 0.003238 0.011223 -0.003759
0.019889 -0.009144 -0.013296 0.016547 0.006387 -0.036795 -
0.028953 0.004607 -0.063156 -0.022265 0.054107 0.003628 -
0.019228 0.014194 -0.006248 0.049220 -0.003656 0.026374
0.010055 -0.002982
z383 -0.017544 0.010896 -0.002095 -0.040459 0.010885 -
0.017896 -0.006742 -0.013675 0.001978 -0.022885 0.005339
0.049426 -0.007286 0.002161 -0.015933 -0.006447 0.001733
0.024947 0.021131 0.032011 -0.020284 0.027519 -0.027551
0.031270 0.018918 -0.000395 -0.007118 0.049495 -0.026251 -
0.012898 -0.023254 0.024125 0.043500 -0.015720 0.010573
0.000120 0.004862
x384 0.036189 -0.002891 0.008483 0.007477 0.030130 -
0.006578 -0.020396 0.000803 0.004909 -0.035980 -0.016511 -
0.025573 0.000195 -0.008480 0.020622 0.004648 -0.005851 -
0.002529 -0.011879 -0.004879 -0.019811 -0.027499 0.002507 -
0.006770 -0.072295 -0.019147 -0.010805 -0.010604 -0.013034
0.014319 -0.007081 -0.053506 0.046672 0.030195 0.035673 -
0.015944 -0.099039
y384 -0.007228 -0.002929 0.008278 -0.006419 0.032900 -
0.035462 -0.016617 -0.030925 -0.002925 -0.032556 0.007552
0.007572 -0.008495 -0.002393 0.009048 0.016105 0.001155
0.024742 -0.007678 -0.014793 0.014673 0.009615 -0.038121 -
0.021967 0.008309 -0.070530 -0.017568 0.056528 -0.006512 -
0.024366 0.008134 -0.012390 0.055675 -0.010361 0.016381
0.019965 -0.000981
z384 -0.020185 0.011878 -0.001274 -0.042230 -0.001848 -
0.012532 0.000842 -0.011588 0.002621 -0.017135 0.007908
0.050644 0.002552 0.003231 -0.018545 -0.008823 0.003996
0.023991 0.024565 0.034351 -0.017436 0.024636 -0.028818
0.036424 0.027270 0.008021 0.010022 0.044424 -0.017362 -
0.012996 -0.018344 0.031638 0.023692 -0.015537 -0.003304
0.005746 0.013532
x385 0.032477 -0.003329 0.009824 0.002998 0.024638 -
0.006005 -0.018326 0.000263 0.009845 -0.039904 -0.016399 -
0.023666 -0.000130 -0.004394 0.020694 0.011508 -0.014575
0.001316 -0.015284 -0.002053 -0.019168 -0.020261 0.003140 -
0.009252 -0.057026 -0.022952 0.000133 -0.014384 -0.013846
0.015463 -0.010887 -0.046264 0.042700 0.031853 0.024619 -
0.002371 -0.079030
y385 -0.009181 -0.001605 0.007358 -0.012635 0.031829 -
0.038700 -0.018613 -0.029467 -0.000260 -0.034285 0.007952
0.006360 -0.009229 0.001526 0.008013 0.016287 0.004352
0.031716 -0.006055 -0.012088 0.015425 0.010674 -0.039664 -
0.017176 0.008193 -0.064274 -0.013063 0.062886 -0.012321 -
0.031983 0.006200 -0.010508 0.062272 -0.003501 0.011956
0.025447 -0.005582
z385 -0.022991 0.011516 -0.003884 -0.041112 -0.009335 -
0.008144 0.005867 -0.010479 0.002814 -0.007683 0.008966
0.051520 0.007819 0.005659 -0.021472 -0.010132 0.005536
0.017294 0.025539 0.032449 -0.012852 0.024569 -0.030521
0.042149 0.033426 0.022403 0.011488 0.042172 -0.011669 -
0.014441 -0.019000 0.039429 0.007868 -0.016328 -0.010377 -
0.001880 0.023915
x386 0.026806 -0.005194 0.016787 -0.005291 0.016626 -
0.015354 -0.013904 0.001475 0.011632 -0.042894 -0.018824 -

0.007844	-0.002805	-0.009864	0.022387	0.022239	-0.015406	-
0.006841	-0.015769	-0.007913	-0.022699	-0.000882	0.005286	-
0.008622	-0.037150	-0.033224	0.005206	-0.015122	-0.003867	
0.016918	-0.022306	-0.022527	0.030942	0.019576	0.046543	-
0.040397	-0.045607					
y386	-0.011343	0.000336	0.006274	-0.018786	0.030025	-
0.037943	-0.022666	-0.030833	-0.000175	-0.037335	0.006440	-
0.000059	-0.011773	0.001461	0.005334	0.009721	0.008259	
0.039375	0.002833	-0.005022	0.016683	0.004373	-0.048210	-
0.015850	0.000962	-0.058162	-0.002398	0.075541	-0.016233	-
0.046687	0.003429	-0.010125	0.069465	0.011005	0.003484	
0.033574	-0.019825					
z386	-0.024736	0.011427	-0.002768	-0.040101	-0.019772	-
0.002981	0.011076	-0.007420	0.002363	-0.000264	0.007400	
0.050010	0.013027	0.007820	-0.020656	-0.009055	0.006306	
0.011760	0.026361	0.033035	-0.007238	0.024058	-0.027024	
0.046517	0.039903	0.025053	0.017002	0.032775	-0.008813	-
0.016217	-0.020844	0.052358	-0.011956	-0.009657	-0.016897	-
0.011479	0.038986					
x387	0.013563	-0.006138	0.017470	-0.003428	0.017648	-
0.025011	0.000352	0.005029	0.006974	-0.043089	-0.007629	
0.023057	-0.010293	-0.011404	0.004786	0.032666	-0.012234	-
0.023817	-0.012384	-0.003070	-0.036482	-0.004998	0.025977	
0.006146	0.016136	-0.065912	-0.011884	-0.038008	-0.000592	
0.037528	-0.046779	-0.041190	0.061249	-0.000722	0.032107	-
0.046796	0.010089					
y387	-0.013103	0.004409	-0.000449	-0.030561	0.016419	-
0.037083	-0.023515	-0.043491	0.004520	-0.027801	0.002286	-
0.017315	-0.007944	-0.008740	0.003904	0.000624	0.020216	
0.055775	0.018566	-0.009429	0.028612	0.004599	-0.080579	-
0.018895	-0.013008	-0.036827	0.057788	0.106397	-0.000084	-
0.068612	0.055397	0.011416	0.039859	-0.013989	0.024255	
0.038930	-0.037105					
z387	-0.027413	0.010719	-0.007051	-0.032350	-0.027505	
0.005411	0.018584	-0.004570	-0.006299	0.004678	0.010744	
0.043353	0.022771	0.020153	-0.019896	-0.011530	0.010126	
0.011427	0.021916	0.031672	0.005121	0.022679	-0.020302	
0.054324	0.052858	0.024983	0.009162	0.016761	-0.008576	-
0.036676	-0.029688	0.061954	-0.017171	0.016129	-0.035654	-
0.024054	0.056942					
x388	0.010472	0.001935	0.012332	0.003799	-0.002249	
0.003190	0.023211	0.031151	0.012016	-0.035412	-0.006186	
0.043180	0.007349	-0.001374	0.008418	0.039133	-0.005265	-
0.014163	-0.026813	-0.025594	-0.031445	0.021012	0.003326	
0.019401	0.017069	-0.054387	0.040770	-0.067715	0.032927	
0.019019	-0.022084	-0.019800	0.030599	-0.033436	0.031302	-
0.051133	0.036102					
y388	-0.011244	0.004946	-0.002652	-0.035313	-0.001830	-
0.022611	-0.025854	-0.035320	0.007689	-0.019073	-0.004139	-
0.031830	-0.009092	-0.008415	0.005116	-0.010227	0.015936	
0.053604	0.026620	0.006529	0.028082	0.005612	-0.078350	-
0.010445	-0.031663	0.011896	0.075441	0.091373	-0.000244	-
0.070525	0.059285	0.013544	0.016656	-0.003483	0.019356	
0.059205	-0.047050					
z388	-0.028000	0.015562	-0.005940	-0.029139	-0.025714	
0.010598	0.009584	-0.005431	-0.005441	0.016650	0.006618	
0.046176	0.011482	0.006658	-0.016307	-0.009995	-0.000998	
0.008183	0.020415	0.031830	0.012976	0.018120	-0.029968	
0.045062	0.026689	0.030624	0.010773	0.020899	0.001138	-

0.024019 -0.020359 0.067375 -0.025638 0.013040 -0.028015 -
0.023731 0.035962
x389 -0.003897 0.008283 0.013442 -0.035001 0.003331 -
0.025752 0.000897 0.003433 0.008046 -0.033143 -0.019929
0.055086 -0.020571 -0.032517 -0.010830 0.033738 -0.019892
0.000468 0.012205 0.007830 -0.027724 0.018797 -0.046729 -
0.012339 -0.005728 -0.053636 0.053983 -0.007409 0.018599
0.015467 -0.024081 0.013247 0.036070 -0.035496 0.046421 -
0.053971 -0.018930
y389 -0.009884 0.005049 -0.006763 -0.012806 -0.026197 -
0.004491 -0.004304 -0.004692 0.000168 -0.012080 0.002759 -
0.043164 0.020495 0.026091 0.004223 -0.003676 0.034186
0.043228 0.018617 0.018499 0.029183 -0.002407 -0.029366
0.023262 -0.000647 0.037511 0.064694 0.016417 -0.008797 -
0.099972 0.015747 -0.016658 0.015773 0.067850 -0.027897
0.050106 -0.014169
z389 -0.020628 0.015865 -0.010922 0.003290 -0.051156
0.024280 0.029600 0.008938 -0.005679 0.044201 0.012484
0.020512 0.031958 0.023315 -0.004932 0.002081 0.013009 -
0.016614 -0.002050 0.001143 0.019646 -0.002619 0.004212
0.048670 0.047938 0.061691 0.033158 -0.040002 0.039520 -
0.002817 0.004833 0.030845 -0.072991 0.010549 -0.014879 -
0.040940 0.070589
x390 -0.001521 0.008433 0.007940 -0.018912 -0.008578 -
0.016963 0.009902 0.012900 0.008044 -0.014667 -0.013810
0.044533 -0.013547 -0.022240 0.002632 0.024009 -0.019181 -
0.006555 0.020103 -0.000800 -0.007313 0.027188 -0.050658
0.004311 0.006652 -0.038388 0.048263 -0.015815 0.040975
0.028947 -0.026546 0.016941 0.014929 -0.016790 0.039606 -
0.061445 -0.009411
y390 -0.020748 0.012754 -0.012005 -0.007327 -0.031320
0.006715 0.006125 -0.013561 -0.001490 -0.012235 -0.009632 -
0.046951 0.027160 0.018421 -0.000731 0.002690 0.024643
0.026954 0.023793 0.004562 -0.005072 -0.040597 0.004795 -
0.004325 0.024170 0.066005 0.067921 -0.016689 -0.045754 -
0.118429 0.010555 -0.017810 -0.041875 0.002500 -0.036504
0.050194 0.013630
z390 -0.008532 0.009252 -0.014875 0.019722 -0.037408
0.027379 0.018025 0.004690 -0.009894 0.039798 0.015886
0.001500 0.039178 0.019165 0.002312 -0.008234 0.006011 -
0.010282 -0.000619 -0.008216 0.014070 -0.010932 0.018690
0.041665 0.039962 0.040063 -0.009639 -0.022480 0.016766 -
0.009575 0.002405 0.025468 -0.060334 0.010010 -0.017691 -
0.033325 0.050089
x391 -0.004733 0.011920 0.006499 -0.007035 -0.010426
0.005304 0.018347 0.023889 0.001151 -0.007181 -0.006140
0.029491 0.008988 -0.011197 0.012624 0.009115 -0.016061 -
0.018639 0.016333 -0.022931 -0.002502 0.005828 -0.038026
0.008617 -0.009812 -0.011847 0.034673 -0.022011 0.033185
0.012795 -0.019522 0.026819 -0.031326 -0.002479 0.016556 -
0.048135 0.004210
y391 -0.014410 0.001791 -0.014800 0.007175 -0.055352
0.006060 0.008131 -0.027124 0.017582 -0.003582 -0.016723 -
0.045845 0.010752 0.017611 -0.004716 0.050171 0.023047
0.024035 0.018790 0.024423 -0.003572 -0.031812 -0.033976
0.003574 0.048072 0.040805 0.056507 -0.047546 -0.012424 -
0.082134 -0.001874 0.000712 0.011015 -0.017038 -0.054940
0.059150 0.029733
z391 0.004436 0.006905 -0.016177 0.031994 -0.020812
0.023645 0.006054 -0.000181 -0.012182 0.043562 0.023093 -

0.003784	0.032921	0.010246	0.009275	-0.022298	0.002227	-	
0.012120	0.000330	-0.011089	0.016778	-0.010804	0.023157		
0.044925	0.029398	0.023170	-0.044825	-0.002690	0.013020		
0.022733	0.009407	0.003763	-0.029593	0.009685	-0.015907	-	
0.026921	0.021264						
x392	-0.002569	0.010799	-0.002380	0.005916	-0.017492		
0.014628	0.024023	0.031562	-0.007919	-0.004007	-0.000854		
0.006907	0.017919	-0.003954	0.012463	0.017714	-0.019736	-	
0.019376	0.012247	-0.030095	0.006480	-0.019743	-0.044029		
0.019317	-0.015583	0.013315	0.003506	-0.019954	0.029205		
0.017554	-0.010503	0.019544	-0.034351	-0.006450	-0.013704	-	
0.012734	0.014008						
y392	-0.014497	0.000350	-0.007348	0.005753	-0.047143		
0.002514	0.015051	-0.017535	0.012562	0.004021	-0.007926	-	
0.051022	0.010669	0.001663	0.008012	0.060562	0.019984	-	
0.009766	0.023525	0.006822	-0.009948	-0.011833	-0.021756	-	
0.013306	0.050986	0.042696	0.069298	-0.067810	0.002160	-	
0.054913	-0.021324	-0.010827	-0.013828	0.004506	-0.038489		
0.019100	0.037771						
z392	0.013097	0.004802	-0.016013	0.038850	-0.006430		
0.018790	-0.006763	-0.010717	-0.007320	0.041261	0.016165	-	
0.000950	0.023800	0.005731	0.011191	-0.030937	-0.006454	-	
0.004281	-0.008291	-0.006387	0.009671	-0.003419	0.031492		
0.040077	0.028900	0.001310	-0.057158	0.003977	0.014095		
0.034821	0.012737	-0.008858	-0.021629	0.008681	0.000244	-	
0.024582	0.006421						
x393	0.003923	0.011914	-0.006554	0.019253	-0.013828		
0.019619	0.019273	0.033714	-0.009501	0.003187	-0.002742		
0.007928	0.020362	-0.000462	0.016002	-0.001011	-0.025542	-	
0.010497	-0.003652	-0.037554	0.024202	-0.020835	-0.037694		
0.033718	-0.011060	0.006098	-0.003816	-0.023611	0.029507		
0.023459	-0.005366	0.015010	-0.040383	-0.008374	-0.026004	-	
0.017814	0.008543						
y393	-0.010296	-0.001799	-0.005387	0.012339	-0.043297		
0.006735	0.011060	-0.017718	0.011001	0.010570	-0.009072	-	
0.039987	0.009061	0.002266	0.010914	0.044725	0.021487	-	
0.013656	0.018206	0.006691	-0.001866	-0.004332	-0.003213	-	
0.007906	0.057320	0.039939	0.075104	-0.068952	0.004289	-	
0.038797	-0.017723	-0.009353	-0.021840	0.001964	-0.028232		
0.009286	0.031278						
z393	0.011267	0.001024	-0.015409	0.032347	0.000692		
0.014738	-0.004286	-0.009463	-0.011521	0.029404	0.017605	-	
0.010484	0.020637	0.003815	0.005314	-0.011558	-0.011338		
0.004907	-0.005528	-0.002422	-0.005952	-0.002986	0.020240		
0.024543	0.011449	-0.000132	-0.063927	0.015732	0.010584		
0.032288	0.017489	-0.010944	0.001031	0.004659	0.006908	-	
0.012731	-0.007756						
x394	0.008145	0.007041	-0.010942	0.022124	-0.009742		
0.017476	0.022606	0.034868	-0.015778	0.004632	0.000337		
0.000663	0.022262	-0.002119	0.008984	-0.006646	-0.031594	-	
0.005704	-0.010678	-0.042081	0.031297	-0.015606	-0.028120		
0.036323	-0.007530	0.011736	-0.016526	-0.020242	0.029352		
0.020995	-0.003143	0.005652	-0.030990	-0.001268	-0.021379	-	
0.028854	-0.001927						
y394	-0.009124	-0.004540	0.000140	0.016443	-0.041554		
0.012363	0.014318	-0.014099	0.009333	0.012434	-0.005403	-	
0.039295	0.013788	-0.002956	0.013770	0.044700	0.023379	-	
0.013438	0.022266	0.007680	-0.007902	-0.005181	0.003819	-	
0.015871	0.051989	0.041537	0.079585	-0.061364	-0.002722	-	

0.034946	-0.014320	-0.005129	-0.019761	-0.000294	-0.018662	
0.019792	0.040891					
z394	0.005199	0.005041	-0.013160	0.022250	0.004936	
0.009328	-0.005828	-0.015970	-0.011580	0.022010	0.012962	-
0.008965	0.021023	0.006131	0.006438	-0.007082	-0.012311	
0.018631	-0.004044	0.005301	-0.015247	-0.004623	0.010832	
0.015266	0.002701	-0.015561	-0.055582	0.019550	0.000596	
0.030572	0.019588	-0.005813	0.008245	-0.002503	0.002427	
0.010704	-0.008814					
x395	0.000785	-0.000311	-0.017397	0.027467	-0.010498	
0.011908	0.029484	0.033794	-0.002877	-0.002535	0.006427	-
0.000367	0.004766	-0.013431	-0.007113	-0.045270	-0.033213	-
0.002164	-0.015896	-0.033643	0.019822	-0.025660	-0.014843	
0.029297	0.005869	0.021466	0.014032	-0.009323	0.007385	
0.018808	-0.020382	0.040311	-0.031890	0.032224	-0.050977	-
0.055681	-0.017525					
y395	-0.013445	-0.002843	-0.010414	0.022965	-0.038124	
0.029422	0.023822	-0.015830	-0.003396	0.014683	0.008788	-
0.038430	0.032298	-0.009841	0.012260	0.039138	0.046950	
0.006371	0.013408	0.017722	0.000913	-0.023205	-0.013488	-
0.021321	0.057206	0.011533	0.067008	-0.032109	-0.057344	
0.035299	-0.057954	-0.023280	-0.038026	-0.038450	0.029704	
0.012257	0.053407					
z395	-0.002605	0.017151	-0.015697	-0.001803	0.008060	
0.013390	-0.008067	-0.016533	-0.018651	0.015502	0.010286	-
0.009807	0.031968	0.007662	0.009742	0.026829	-0.007122	
0.032828	-0.000236	0.011033	-0.010088	-0.007510	-0.011673	
0.004949	-0.015459	-0.017698	-0.070229	0.013617	-0.014099	
0.062434	0.005451	-0.030596	0.001854	-0.016675	0.032118	
0.037852	-0.028556					
x396	0.005573	-0.009175	-0.019653	0.027751	-0.007211	
0.009859	0.034499	0.017804	-0.028148	0.006021	0.018006	-
0.008832	0.022505	-0.003249	-0.014876	-0.031929	-0.045168	
0.004471	-0.023502	-0.023129	0.002783	-0.028307	-0.035495	
0.020631	-0.000795	0.013419	-0.017429	0.017045	-0.016262	
0.026617	-0.028520	-0.007502	-0.007318	0.044581	-0.021859	-
0.011480	0.031787					
y396	-0.009354	-0.009084	-0.009917	0.023600	-0.021553	
0.016573	0.028774	-0.016984	0.017743	0.040873	0.032666	-
0.023008	0.040344	0.018392	-0.005318	0.043881	-0.008200	-
0.019675	0.001333	0.017607	-0.014156	-0.007549	-0.027535	-
0.006092	0.063326	0.032629	0.051204	-0.034358	-0.103304	
0.013060	-0.006527	0.002137	-0.039637	0.004823	-0.022316	
0.062955	0.053144					
z396	-0.017044	0.030212	-0.021680	-0.029892	0.023896	-
0.009537	0.001371	-0.030411	0.009082	0.012042	0.015819	
0.000662	0.035579	0.034362	0.008100	0.013179	-0.006762	
0.048522	-0.004336	-0.010071	-0.011399	-0.027703	-0.012534	
0.018147	-0.012366	-0.049270	-0.034307	-0.005240	-0.017823	
0.030540	0.066855	-0.017054	0.010950	-0.028939	0.052934	
0.073647	-0.098721					
x397	0.011980	-0.045191	-0.017661	0.016096	-0.007495	-
0.007472	0.033335	-0.002037	-0.032021	-0.039951	0.022591	
0.004835	0.024065	-0.016530	-0.032700	-0.009384	-0.020241	
0.025669	-0.080922	-0.031855	-0.016507	-0.011148	0.018166	-
0.020968	-0.032824	-0.044071	0.004437	0.035613	-0.012781	
0.014981	-0.035964	0.000179	-0.003616	-0.014196	-0.022126	-
0.047577	-0.009857					
y397	-0.010187	-0.024584	0.015385	0.013420	-0.019897	
0.004956	0.027345	-0.027521	0.009532	-0.003812	0.018604	-

0.006262	0.038998	-0.003365	-0.046085	0.045860	0.016617	
0.016582	-0.032666	-0.006866	-0.019400	-0.001332	0.010068	
0.008150	0.022010	-0.009808	0.063800	-0.011375	-0.009527	
0.016632	0.013713	-0.021748	-0.057351	0.002934	-0.018406	-
0.007878	-0.006559					
z397	-0.049316	0.052224	-0.008582	-0.074823	0.012605	-
0.005187	0.007593	-0.046303	-0.009972	0.018639	0.016810	
0.015842	0.027718	0.035862	-0.006414	0.021155	0.002982	
0.046353	0.013720	0.032619	-0.035592	-0.004427	-0.045246	-
0.007642	-0.036891	-0.057227	0.024550	-0.009127	0.007522	
0.011726	0.023845	0.027285	-0.003422	-0.051643	0.005462	
0.069171	0.002226					
x398	0.043892	-0.015594	-0.005816	0.006398	0.000467	
0.007716	-0.015588	0.014114	-0.029606	-0.034710	-0.034924	
0.067648	-0.010368	0.036417	0.048991	0.123511	0.056560	
0.027161	0.041498	0.051447	0.055244	0.002954	-0.033821	
0.059295	-0.039953	0.042342	-0.002337	-0.015842	-0.081897	-
0.005472	-0.001422	0.032453	-0.006995	-0.019663	0.028341	
0.017755	-0.062417					
y398	-0.017818	-0.012678	-0.002688	0.002771	0.048280	-
0.003182	0.047373	0.049150	-0.053624	0.007136	0.014790	
0.030044	-0.048675	-0.038315	0.002174	0.002838	0.007110	-
0.018354	0.051615	-0.000137	-0.034375	0.027268	-0.018458	-
0.048570	0.039571	-0.022843	-0.007601	-0.010531	0.002335	-
0.040228	-0.038355	-0.020607	0.020609	-0.012871	-0.044607	-
0.022990	0.029954					
z398	-0.017611	0.002463	0.010707	0.023519	0.005939	-
0.008452	-0.023047	0.017831	0.047546	0.015481	0.007134	-
0.023001	-0.046230	-0.010505	0.003288	-0.065976	-0.018438	
0.013689	0.003166	-0.012252	-0.009169	-0.023953	-0.024675	
0.020205	0.031141	0.025921	-0.008106	0.004103	-0.024992	-
0.022267	0.032379	-0.016852	0.045195	-0.017704	0.020152	
0.012351	0.046466					
x399	0.046738	-0.008692	-0.001306	0.007962	-0.001517	
0.006698	-0.030484	0.005447	-0.023168	-0.032802	-0.023287	
0.068265	-0.026400	0.035193	0.055092	0.087518	0.069003	
0.031495	0.028575	0.041533	0.040153	0.019507	-0.020398	
0.039893	-0.020113	0.026192	0.012431	-0.027579	-0.088767	
0.000655	0.020004	0.035754	-0.016717	-0.002542	0.021812	-
0.004611	-0.063016					
y399	-0.013198	-0.015096	-0.002443	0.002380	0.051536	
0.000335	0.042062	0.044707	-0.048275	0.011383	0.012292	
0.021789	-0.049710	-0.046798	0.014372	0.002325	0.015987	-
0.020882	0.034840	-0.016966	-0.017930	0.034701	-0.016594	-
0.043939	0.054614	-0.045898	0.007808	-0.022789	-0.009221	-
0.033369	-0.036727	-0.004296	0.011994	-0.022400	-0.046074	-
0.010101	0.007399					
z399	-0.019747	-0.001349	0.010399	0.016791	0.001091	-
0.003698	-0.012337	0.028366	0.034106	0.000999	0.000136	-
0.019813	-0.030687	-0.015701	-0.002102	-0.033839	-0.022101	
0.014537	0.008656	-0.008267	-0.009825	-0.020497	-0.032679	
0.009129	0.020485	0.035394	-0.021967	0.006233	-0.007462	-
0.018814	0.021609	-0.019129	0.049357	-0.021221	0.002035	
0.016141	0.038061					
x400	0.049714	0.000721	0.000355	0.012231	-0.005198	
0.002755	-0.042971	-0.005213	-0.021253	-0.039340	-0.013197	
0.063578	-0.041723	0.036952	0.054523	0.059131	0.069296	
0.033468	0.013251	0.045205	0.027051	0.028626	-0.011084	
0.026712	-0.013375	0.016623	0.031176	-0.036631	-0.091432	

0.000917 0.044885 0.044505 -0.021856 0.009138 0.034541 -
0.029860 -0.051874
y400 -0.005677 -0.007357 -0.002786 0.002511 0.059154
0.001165 0.034719 0.034407 -0.042131 0.017858 0.018111
0.012933 -0.048810 -0.047951 0.018225 -0.012334 0.028098 -
0.018825 0.022379 -0.019130 -0.014865 0.046091 -0.012852 -
0.040205 0.062215 -0.047683 0.025648 -0.030095 -0.018003 -
0.041681 -0.020122 0.021250 0.005047 -0.023857 -0.025068
0.002553 0.007364
z400 -0.016683 -0.001918 0.008274 0.016870 -0.002215 -
0.000972 -0.018019 0.027390 0.026297 -0.005453 -0.011016 -
0.009367 -0.019333 -0.016100 -0.001354 -0.029495 -0.027105
0.016313 0.007019 -0.003002 -0.001923 -0.011326 -0.025418
0.010892 0.024558 0.028061 -0.027540 0.007894 -0.001668 -
0.009623 0.015754 -0.020533 0.043802 -0.025632 -0.005004
0.012519 0.034886
x401 0.050496 0.009126 0.002872 0.014790 -0.009279
0.001341 -0.050689 -0.013757 -0.018977 -0.042436 -0.003203
0.058148 -0.050012 0.037859 0.053228 0.038403 0.066919
0.035135 0.003335 0.047095 0.018043 0.036376 -0.002880
0.013374 -0.006656 0.008102 0.036945 -0.042137 -0.089611
0.003261 0.063970 0.049911 -0.024155 0.016782 0.041867 -
0.044019 -0.043118
y401 0.001622 -0.000724 -0.002620 0.006133 0.056586
0.000736 0.027944 0.019011 -0.037491 0.017006 0.016525
0.010430 -0.047573 -0.048163 0.012804 -0.023134 0.033401 -
0.009702 0.002259 -0.024036 -0.014171 0.047138 -0.008853 -
0.025393 0.051804 -0.050539 0.039922 -0.027144 -0.036343 -
0.030587 -0.002994 0.030931 0.008361 -0.019856 -0.002619 -
0.001893 0.024366
z401 -0.014784 -0.004054 0.006450 0.017085 -0.004943
0.000035 -0.021123 0.027480 0.019590 -0.011263 -0.020045 -
0.002771 -0.008868 -0.017234 0.000849 -0.025073 -0.031371
0.018846 0.005264 0.000118 0.001019 -0.003736 -0.020464
0.009818 0.023208 0.022592 -0.033788 0.009535 0.003331 -
0.002054 0.008498 -0.018691 0.036694 -0.027592 -0.008009
0.011014 0.031923
x402 0.050801 0.016732 0.005309 0.015060 -0.012053
0.000206 -0.052629 -0.020088 -0.015613 -0.041728 0.005694
0.052060 -0.053517 0.038150 0.050691 0.019747 0.065205
0.035228 -0.002728 0.043264 0.011217 0.040540 0.001774
0.001416 0.002581 0.000258 0.036136 -0.051146 -0.080601
0.001788 0.068867 0.050935 -0.029778 0.019420 0.041307 -
0.045631 -0.035103
y402 0.006057 0.003840 -0.007060 0.011483 0.051727
0.002565 0.016039 0.008481 -0.037599 0.005971 0.011117
0.005735 -0.047863 -0.046168 0.009035 -0.022560 0.027813
0.000909 -0.011646 -0.021098 -0.001232 0.047217 0.000548 -
0.012612 0.040587 -0.054371 0.041772 -0.012009 -0.047300 -
0.017872 0.025150 0.039788 0.026212 -0.014459 0.010808
0.000352 0.025050
z402 -0.012000 -0.007002 0.006051 0.014786 -0.005949
0.001140 -0.021584 0.029968 0.014801 -0.016783 -0.024357
0.003320 -0.001032 -0.017704 0.003254 -0.020918 -0.029055
0.019175 0.008456 0.003788 0.002889 -0.001406 -0.013068
0.007411 0.018533 0.020739 -0.042300 0.010362 0.012302
0.003361 0.008112 -0.015973 0.028732 -0.024666 -0.009954
0.010894 0.032539
x403 0.050371 0.023676 0.006790 0.015070 -0.015482 -
0.000333 -0.049127 -0.027035 -0.012710 -0.041517 0.013534

0.046195	-0.053362	0.038712	0.048276	0.005425	0.057349	
0.034438	-0.009913	0.036507	0.006939	0.041745	0.006152	-
0.005186	0.009897	-0.005970	0.034841	-0.054310	-0.068111	
0.003170	0.065876	0.051780	-0.029579	0.018775	0.038044	-
0.042326	-0.029299					
y403	0.009194	0.008504	-0.010660	0.011703	0.048938	
0.004285	0.007736	0.004689	-0.034599	-0.001847	0.009330	
0.001536	-0.040531	-0.043999	0.005723	-0.021402	0.030035	
0.009537	-0.015404	-0.009809	0.004284	0.045269	0.006737	-
0.004712	0.034292	-0.055498	0.032668	-0.006268	-0.056330	-
0.009502	0.040888	0.034995	0.032372	-0.005097	0.027959	
0.004983	0.019784					
z403	-0.009115	-0.011431	0.005434	0.012344	-0.007079	
0.001307	-0.021197	0.032213	0.009476	-0.021959	-0.030248	
0.009372	0.004113	-0.018706	0.008482	-0.016392	-0.025995	
0.016284	0.010848	0.006964	0.004359	-0.003083	-0.009731	
0.007895	0.014898	0.020761	-0.045953	0.005921	0.014536	
0.009118	0.007899	-0.012929	0.018817	-0.019180	-0.014211	
0.007478	0.032759					
x404	0.048399	0.031199	0.007385	0.015765	-0.019852	
0.001990	-0.043536	-0.033373	-0.011199	-0.042345	0.019644	
0.038967	-0.049209	0.038458	0.043781	-0.002787	0.045272	
0.033009	-0.014012	0.031643	0.004664	0.041546	0.007848	-
0.011668	0.016090	-0.010582	0.027205	-0.051735	-0.052253	
0.005468	0.061640	0.050678	-0.026059	0.017884	0.031121	-
0.039421	-0.017272					
y404	0.009105	0.009814	-0.011328	0.006923	0.043001	
0.001606	0.002016	0.004556	-0.031575	-0.003969	0.009342	
0.001611	-0.028474	-0.039747	-0.002888	-0.022649	0.030743	
0.011615	-0.016025	-0.000824	0.010416	0.045817	0.009301	
0.008416	0.025479	-0.048383	0.028218	0.001944	-0.045493	
0.002040	0.036714	0.029977	0.029312	-0.010448	0.022109	
0.007672	0.025645					
z404	-0.005708	-0.017474	0.005613	0.010054	-0.007843	-
0.001246	-0.020013	0.034077	0.005248	-0.024737	-0.037023	
0.015482	0.007716	-0.017031	0.015459	-0.013294	-0.021289	
0.013021	0.012990	0.009262	0.003861	-0.001901	-0.006498	
0.013976	0.010544	0.020027	-0.047025	0.001018	0.012408	
0.017760	0.010952	-0.010960	0.010677	-0.006604	-0.017393	
0.005973	0.026882					
x405	0.045832	0.039333	0.006115	0.015873	-0.025021	
0.003329	-0.032789	-0.037868	-0.009180	-0.043728	0.025412	
0.031692	-0.040306	0.036601	0.037822	-0.006252	0.031217	
0.031970	-0.018631	0.026882	0.005412	0.037747	0.008789	-
0.014495	0.018312	-0.012616	0.017023	-0.040390	-0.035345	
0.013106	0.049425	0.044658	-0.020117	0.014775	0.029148	-
0.024759	-0.013489					
y405	0.008359	0.007237	-0.011566	0.003826	0.038302	
0.004284	-0.002105	0.004232	-0.027938	-0.012119	0.007991	-
0.000209	-0.011727	-0.030341	-0.008577	-0.022182	0.027650	
0.011327	-0.010457	0.008281	0.012950	0.046699	0.009532	
0.012759	0.016239	-0.043427	0.013248	0.001432	-0.029603	
0.000511	0.029796	0.027755	0.025752	-0.010871	0.015884	-
0.002700	0.033869					
z405	-0.001752	-0.024270	0.004231	0.006935	-0.006921	-
0.002696	-0.019016	0.035995	0.001363	-0.024706	-0.041518	
0.022370	0.010636	-0.017294	0.026323	-0.015685	-0.014130	
0.007792	0.014953	0.008592	-0.000899	-0.004909	-0.003317	
0.016478	0.006275	0.018268	-0.043955	-0.003545	0.007937	

0.023810	0.013350	-0.008685	0.004084	0.004666	-0.013040	
0.008428	0.016178					
x406	0.042177	0.047485	0.002686	0.015672	-0.031523	
0.006620	-0.018580	-0.040252	-0.006173	-0.048198	0.030033	
0.022950	-0.026549	0.034484	0.027908	-0.003998	0.014101	
0.028959	-0.019070	0.025285	0.009844	0.030246	0.007406	-
0.011607	0.016584	-0.013630	0.000057	-0.026635	-0.010493	
0.021732	0.028755	0.035921	-0.016987	0.001126	0.026550	-
0.006330	-0.003978					
y406	0.008159	0.005071	-0.011956	0.000659	0.036856	
0.002574	-0.000719	0.003413	-0.024515	-0.019090	0.008488	-
0.002426	0.007782	-0.021657	-0.004660	-0.023856	0.021276	
0.011152	-0.003939	0.012790	0.009684	0.046136	0.007019	
0.008491	0.005806	-0.033997	-0.005280	0.003141	-0.021834	-
0.000045	0.025378	0.018269	0.038328	-0.000235	0.024883	-
0.001273	0.011680					
z406	0.003057	-0.033185	0.001486	0.004116	-0.004885	-
0.002491	-0.018266	0.036903	-0.003346	-0.021822	-0.043746	
0.028091	0.009915	-0.016868	0.037864	-0.023119	-0.006232	-
0.000635	0.017783	0.006246	-0.009964	-0.008649	-0.003574	
0.015197	0.003740	0.014890	-0.036716	-0.009126	0.004419	
0.025409	0.011240	-0.003119	-0.003852	0.004999	-0.013739	
0.009182	0.009325					
x407	0.036790	0.055393	-0.002124	0.013856	-0.038257	
0.010188	-0.002308	-0.041230	-0.002659	-0.055447	0.033969	
0.013185	-0.004364	0.033938	0.014645	0.003057	-0.006825	
0.026091	-0.016241	0.026606	0.019665	0.021378	0.001795	-
0.007779	0.009305	-0.009210	-0.024461	-0.014266	0.018944	
0.028464	-0.000559	0.027989	-0.008269	-0.018122	0.020956	
0.013785	0.007256					
y407	0.006497	0.002103	-0.009448	-0.003804	0.031861	
0.004617	0.003000	0.003233	-0.022978	-0.018471	0.006479	-
0.001148	0.023161	-0.016080	-0.003880	-0.023002	0.013822	
0.003816	0.002945	0.011386	-0.000823	0.041775	0.008015	
0.007045	0.005567	-0.026800	-0.022664	0.012991	-0.006681	
0.002060	0.015776	-0.001715	0.037532	0.006532	0.016738	-
0.000422	-0.007337					
z407	0.007719	-0.042280	-0.000454	-0.000233	-0.000888	-
0.001954	-0.017192	0.036530	-0.006766	-0.016751	-0.045449	
0.032850	0.008319	-0.016804	0.049169	-0.031782	0.004937	-
0.010647	0.018253	0.003174	-0.018912	-0.012766	0.000488	
0.017723	0.004408	0.011668	-0.025269	-0.016190	-0.004358	
0.023970	0.008477	0.000419	-0.009657	0.012562	-0.015945	
0.007240	-0.004020					
x408	0.033786	0.047303	-0.006020	0.008928	-0.034574	
0.005247	0.001654	-0.032746	-0.005333	-0.050751	0.030157	
0.011056	0.003413	0.031649	0.016933	-0.004739	-0.007383	
0.029788	-0.014477	0.024788	0.013697	0.008553	-0.006903	-
0.008752	0.010039	-0.008093	-0.017947	-0.013603	0.022417	
0.019913	0.004260	0.016696	0.007103	-0.021196	0.016817	
0.009985	0.004752					
y408	0.007414	-0.007679	-0.007859	-0.009199	0.030350	
0.000749	-0.004508	0.008540	-0.018021	-0.017296	0.003122	
0.003802	0.028481	-0.016200	0.011611	-0.018856	0.016234	-
0.000238	0.006964	0.000949	-0.011266	0.038490	0.012448	
0.002069	0.000615	-0.021996	-0.020520	0.009542	-0.000205	
0.001698	0.002634	-0.011857	0.026486	0.018981	0.024420	-
0.003140	0.002099					
z408	0.009272	-0.038588	-0.000384	0.001426	-0.002260	-
0.002064	-0.014253	0.031519	-0.006323	-0.021200	-0.036276	

0.028942	0.007971	-0.012395	0.047284	-0.032663	0.005794	-
0.006400	0.021085	-0.012523	-0.006309	-0.009701	0.002782	
0.017252	0.013090	0.012184	-0.020591	-0.018229	0.001968	
0.030646	0.001990	0.002249	-0.015321	0.005981	-0.013330	
0.007421	0.013774					
x409	0.032139	0.039109	-0.007549	0.001553	-0.029731	
0.000271	0.004738	-0.024985	-0.003987	-0.047154	0.027154	
0.009195	0.010871	0.028758	0.023025	-0.008565	-0.004008	
0.031648	-0.012359	0.020217	0.005452	0.002894	-0.006853	-
0.009110	0.008888	-0.013018	-0.010024	-0.014789	0.027857	
0.010910	-0.001995	0.009881	0.010807	-0.013925	0.027557	
0.008986	0.008203					
y409	0.007030	-0.014611	-0.007332	-0.010568	0.026560	-
0.000499	-0.008963	0.012092	-0.016221	-0.016314	0.000060	
0.007110	0.029000	-0.013732	0.018661	-0.019191	0.012167	-
0.002855	0.008224	-0.004826	-0.014400	0.026489	0.005667	-
0.007416	-0.000943	-0.010104	-0.021112	0.010457	0.001511	
0.004114	0.002796	-0.018873	0.027388	0.011108	0.017605	-
0.009266	0.008965					
z409	0.010247	-0.034768	-0.000479	0.001896	-0.002918	-
0.002723	-0.011221	0.026590	-0.004989	-0.023472	-0.026533	
0.025215	0.008226	-0.007094	0.045500	-0.031803	0.007519	-
0.003209	0.022306	-0.025365	0.003716	-0.007401	0.008155	
0.017504	0.018641	0.009347	-0.016123	-0.019913	0.005462	
0.034918	-0.003281	0.004815	-0.019136	0.006056	-0.004697	
0.008820	0.023520					
x410	0.030223	0.031498	-0.009766	-0.004106	-0.026698	-
0.000885	0.006674	-0.018855	-0.005068	-0.041949	0.023265	
0.006210	0.018113	0.026218	0.027577	-0.013063	-0.002973	
0.033022	-0.009548	0.017756	-0.002470	-0.002002	-0.011569	-
0.013781	0.010888	-0.016228	-0.006652	-0.014846	0.024801	
0.004097	-0.000366	0.005074	0.021382	-0.015061	0.031129	
0.005559	0.000464					
y410	0.006492	-0.019935	-0.003309	-0.013642	0.023120	-
0.003400	-0.013663	0.017301	-0.012777	-0.016145	-0.004012	
0.010219	0.023029	-0.011911	0.020938	-0.013102	0.014071	-
0.003422	0.007016	-0.012262	-0.012060	0.015174	0.010034	-
0.002746	-0.005806	-0.005431	-0.017763	0.008285	0.012713	
0.008436	0.002542	-0.026565	0.012520	0.021061	0.014373	-
0.015350	0.019470					
z410	0.010656	-0.030376	-0.001459	0.001666	-0.003373	-
0.001934	-0.007929	0.020962	-0.004041	-0.022950	-0.017229	
0.021219	0.009423	-0.002393	0.041526	-0.032070	0.008657	-
0.001627	0.023109	-0.033572	0.011571	-0.005198	0.011313	
0.015900	0.022967	0.008098	-0.012064	-0.019380	0.005667	
0.037331	-0.007296	0.007953	-0.016875	0.004918	0.000127	
0.009558	0.028773					
x411	0.029341	0.024826	-0.009818	-0.011308	-0.023832	
0.000481	0.007598	-0.014121	-0.004753	-0.038188	0.019995	
0.006350	0.020895	0.024062	0.030389	-0.016309	-0.000458	
0.030683	-0.006959	0.013506	-0.009261	-0.006938	-0.012349	-
0.016006	0.012276	-0.017049	0.001327	-0.012312	0.028945	-
0.001847	-0.002070	-0.000507	0.022826	-0.010656	0.032118	
0.002546	0.000676					
y411	0.005758	-0.023164	-0.003668	-0.016030	0.020392	-
0.005655	-0.016377	0.020397	-0.007799	-0.010841	-0.000865	
0.009178	0.022742	-0.008952	0.021510	-0.012758	0.014746	-
0.003702	0.010450	-0.015604	-0.010153	0.008510	0.010716	-
0.005224	-0.006515	-0.000314	-0.021437	0.004350	0.009611	

0.010225 0.002566 -0.025226 0.015799 0.017749 0.012789 -
0.013529 0.018432
z411 0.009966 -0.026766 -0.003032 0.003167 -0.005119 -
0.002791 -0.006778 0.016040 -0.004668 -0.023860 -0.013276
0.016684 0.005145 0.001469 0.037161 -0.027891 0.004604 -
0.000706 0.020052 -0.041346 0.018612 -0.002874 0.010461
0.016112 0.027221 0.004678 -0.005047 -0.018819 0.009068
0.037114 -0.009898 0.009664 -0.020345 0.006044 -0.000123
0.008790 0.034399
x412 0.029223 0.018864 -0.010792 -0.017078 -0.022047
0.003927 0.007179 -0.010668 -0.004838 -0.034747 0.016930
0.005544 0.022163 0.020987 0.031843 -0.018373 0.001260
0.027761 -0.004262 0.007798 -0.013421 -0.011635 -0.013675 -
0.018628 0.016066 -0.014690 0.005857 -0.008718 0.030511 -
0.005929 -0.005266 -0.005257 0.023918 -0.009178 0.027664
0.005339 0.005610
y412 0.003795 -0.025358 -0.001576 -0.017258 0.017718 -
0.009954 -0.017949 0.022890 -0.004587 -0.009405 -0.002323
0.007167 0.017048 -0.005799 0.022883 -0.007863 0.014503 -
0.001948 0.010842 -0.015886 -0.009085 0.007010 0.013186 -
0.005208 -0.009939 -0.004005 -0.015621 -0.002657 0.008344
0.013084 0.003232 -0.020657 0.013229 0.016958 0.020712 -
0.020409 0.011677
z412 0.009361 -0.022231 -0.004288 0.001998 -0.006177 -
0.000163 -0.004837 0.010495 -0.004808 -0.019786 -0.004833
0.013880 0.002512 0.005274 0.028471 -0.028234 0.004137 -
0.000666 0.018758 -0.044904 0.023742 -0.004668 0.010143
0.012837 0.027965 0.004128 -0.002729 -0.014899 0.011104
0.034121 -0.010370 0.011092 -0.020895 0.007437 -0.003334
0.009060 0.039015
x413 0.030166 0.013510 -0.009770 -0.022488 -0.020257
0.008704 0.007284 -0.007608 -0.003745 -0.031038 0.015977
0.004287 0.022132 0.017357 0.031577 -0.018981 0.004268
0.025563 -0.000973 0.002768 -0.016923 -0.013892 -0.010327 -
0.019386 0.021258 -0.012972 0.008911 -0.007366 0.028073 -
0.006596 -0.006189 -0.006667 0.021947 -0.005998 0.028409
0.007572 0.000598
y413 0.000679 -0.025346 -0.000367 -0.019149 0.014324 -
0.011512 -0.020202 0.023805 -0.002582 -0.005889 -0.004555
0.005716 0.009844 -0.000242 0.021815 -0.005653 0.015075 -
0.002520 0.011841 -0.015178 -0.004027 -0.000068 0.009472 -
0.008741 -0.015497 -0.007495 -0.011933 -0.003348 0.010133
0.010829 0.006436 -0.015729 0.016689 0.014537 0.025076 -
0.021312 0.017332
z413 0.008347 -0.017509 -0.005564 0.002343 -0.007494
0.000347 -0.002501 0.005643 -0.005105 -0.016116 0.001832
0.009234 -0.002300 0.008443 0.019489 -0.025557 0.001289
0.000453 0.015707 -0.044850 0.027249 -0.004776 0.010535
0.012876 0.027707 0.001846 0.000199 -0.014571 0.009979
0.031241 -0.008627 0.013336 -0.022581 0.011645 -0.002876
0.010643 0.030734
x414 0.030816 0.009083 -0.008830 -0.027664 -0.018948
0.015700 0.006410 -0.006792 -0.001938 -0.025975 0.013230
0.000493 0.019059 0.014282 0.031533 -0.016343 0.007496
0.021634 0.001868 -0.003022 -0.018806 -0.014258 -0.007623 -
0.020724 0.026118 -0.010844 0.012238 -0.003398 0.023490 -
0.007689 -0.004927 -0.006439 0.020330 -0.004328 0.027324
0.012822 -0.000498
y414 -0.001962 -0.024092 0.000481 -0.020016 0.009687 -
0.014278 -0.021186 0.025790 -0.001742 -0.007496 -0.004445

0.005578	0.000547	0.001846	0.018796	-0.004602	0.014377		
0.001879	0.009889	-0.012339	0.001161	-0.001567	0.009664	-	
0.010097	-0.019469	-0.013375	-0.007138	-0.009162	0.013913		
0.016225	0.005124	-0.006592	0.010863	0.013678	0.028671	-	
0.028089	0.012121						
z414	0.006388	-0.012257	-0.007158	0.001144	-0.008233		
0.002433	-0.000022	-0.000086	-0.004800	-0.008269	0.008434		
0.004039	-0.007792	0.012734	0.008179	-0.022885	-0.000514	-	
0.000430	0.012360	-0.041187	0.029465	-0.007353	0.009447		
0.010771	0.023929	-0.000696	0.002772	-0.010693	0.006382		
0.024286	-0.002434	0.014575	-0.019871	0.016487	-0.003843		
0.012945	0.021005						
x415	0.032001	0.004299	-0.008870	-0.029194	-0.019891		
0.020481	0.002584	-0.005039	-0.001228	-0.026968	0.005347	-	
0.005660	0.010275	0.008141	0.032559	-0.007856	0.003617		
0.017324	0.002984	-0.010856	-0.018774	-0.009874	-0.006802	-	
0.022027	0.034303	-0.012688	0.017642	-0.002402	0.016981	-	
0.006914	-0.007309	0.001457	0.013032	-0.000679	0.025833		
0.017121	0.001208						
y415	-0.007450	-0.022487	-0.000225	-0.015052	0.003381	-	
0.019497	-0.021971	0.023757	-0.003483	-0.010616	-0.012612		
0.000653	-0.014330	0.007007	0.019837	0.004606	0.007051		
0.007061	0.003740	-0.011748	0.007924	0.000908	0.006501	-	
0.004864	-0.018582	-0.020579	0.001570	-0.015796	0.019928		
0.022160	0.002924	-0.002491	0.003275	0.007026	0.030067	-	
0.021250	0.008442						
z415	0.005477	-0.005316	-0.007352	-0.005181	-0.007117		
0.008791	0.004121	-0.004999	-0.002823	0.005492	0.022856		
0.002587	-0.010609	0.016422	-0.010441	-0.027680	0.005140	-	
0.002358	0.013158	-0.031069	0.030252	-0.015357	0.011600		
0.004289	0.013522	-0.001610	0.000910	-0.003824	-0.001342		
0.014211	0.008796	0.018559	-0.012413	0.025565	-0.003663		
0.011893	0.004118						
x416	0.035519	0.002902	-0.004043	-0.039822	-0.017323		
0.035376	0.005132	-0.006214	0.003718	-0.016168	0.012127	-	
0.004853	0.008669	0.003217	0.025370	-0.012867	0.016488		
0.012591	0.011883	-0.013001	-0.020127	-0.010218	0.004288	-	
0.028112	0.033942	-0.009821	0.014792	0.004380	0.008234	-	
0.003301	-0.005876	0.010553	0.009038	0.004503	0.028410		
0.023333	-0.003911						
y416	-0.011446	-0.015394	0.001873	-0.018224	-0.000188	-	
0.013280	-0.021308	0.018899	-0.001447	-0.004712	-0.002271		
0.002466	-0.022555	0.017087	0.006710	0.000206	0.014435		
0.006145	0.006763	0.000611	0.016299	-0.004072	0.008572	-	
0.003295	-0.024243	-0.016129	0.003659	-0.010825	0.016331		
0.019942	0.013466	-0.004199	0.004886	0.003656	0.029953	-	
0.015323	-0.001555						
z416	0.001467	-0.001831	-0.012082	-0.000726	-0.010343		
0.003682	0.003897	-0.007580	-0.006043	0.008783	0.016543	-	
0.008572	-0.024847	0.015977	-0.019489	-0.015528	-0.008315	-	
0.000329	0.002444	-0.026931	0.031313	-0.015431	0.003599		
0.002199	0.010554	-0.014404	0.006675	-0.007137	-0.003964		
0.007348	0.013386	0.028366	-0.012169	0.031993	-0.005530		
0.011313	-0.012207						
x417	0.032808	-0.000138	-0.003056	-0.045848	-0.014791		
0.035970	0.008036	-0.005399	0.006045	-0.013562	0.015811	-	
0.005181	0.006193	0.001302	0.021963	-0.017811	0.018269		
0.008749	0.009468	-0.018560	-0.021249	-0.012484	0.009035	-	
0.032831	0.029751	-0.007502	0.017603	0.013774	0.001572	-	

0.001125	-0.004957	0.010070	0.010781	0.003776	0.023654	
0.014117	-0.008247					
y417	-0.016001	-0.012027	0.002375	-0.019613	-0.004754	-
0.016488	-0.020067	0.017577	-0.004507	-0.007999	-0.000541	
0.003523	-0.024484	0.023713	-0.000400	-0.001894	0.014069	
0.003623	0.000729	0.006120	0.018549	-0.009126	0.010489	-
0.002650	-0.027170	-0.012189	0.007183	-0.005880	0.015308	
0.018020	0.008061	-0.005794	0.007156	0.008304	0.026656	-
0.025160	-0.007063					
z417	-0.001061	0.000109	-0.011779	0.003080	-0.014205	-
0.003209	0.002072	-0.008961	-0.009253	0.010805	0.009800	-
0.007733	-0.015990	0.014882	-0.022491	-0.015574	-0.013663	-
0.003554	0.000000	-0.016543	0.024395	-0.015824	-0.002825	
0.000878	0.007330	-0.009035	0.009566	-0.004650	-0.009507	
0.006084	0.009514	0.014958	-0.015489	0.030988	-0.015913	
0.010632	-0.009245					
x418	0.030626	-0.003134	-0.002987	-0.050491	-0.013189	
0.035919	0.009264	-0.004302	0.007455	-0.011724	0.017949	-
0.005394	0.003442	-0.000807	0.017832	-0.020333	0.017604	
0.008344	0.007209	-0.022966	-0.018976	-0.012643	0.013393	-
0.037566	0.026216	-0.005747	0.021386	0.021771	-0.003094	
0.002865	-0.002885	0.010675	0.011200	0.003494	0.020511	
0.006225	-0.013702					
y418	-0.020870	-0.008606	0.003902	-0.019736	-0.010019	-
0.020543	-0.017099	0.015438	-0.007149	-0.011996	-0.000965	
0.003114	-0.024218	0.028966	-0.004433	-0.004691	0.012747	-
0.001320	-0.001979	0.012790	0.015687	-0.013526	0.007659	
0.001901	-0.030756	-0.009860	0.010182	-0.004114	0.010779	
0.015479	0.002936	-0.012486	0.005650	0.009504	0.018796	-
0.030066	-0.008005					
z418	-0.002659	0.002328	-0.012725	0.006669	-0.016685	-
0.008757	-0.000203	-0.009121	-0.011742	0.013349	0.004945	-
0.005519	-0.009362	0.014183	-0.026336	-0.013757	-0.016698	-
0.005680	-0.001589	-0.008292	0.021183	-0.016015	-0.005072	-
0.003181	0.004948	-0.003089	0.009293	-0.001598	-0.011751	
0.004072	0.007393	0.006933	-0.016205	0.030291	-0.019622	
0.006138	-0.006915					
x419	0.028872	-0.005517	-0.003086	-0.054484	-0.012905	
0.034579	0.010098	-0.003531	0.008644	-0.008994	0.018230	-
0.005260	0.001190	-0.003012	0.013391	-0.022063	0.016234	
0.007512	0.005331	-0.025216	-0.016181	-0.011874	0.014229	-
0.039612	0.021850	-0.003441	0.024675	0.028605	-0.007461	
0.004932	0.001075	0.007938	0.008057	0.002663	0.019500	-
0.001983	-0.019690					
y419	-0.024572	-0.006001	0.004260	-0.018432	-0.013642	-
0.023064	-0.015152	0.014113	-0.008432	-0.016570	-0.000078	
0.000916	-0.023619	0.032195	-0.006296	-0.005556	0.010614	-
0.003605	-0.002041	0.015395	0.014111	-0.016055	0.008091	
0.001150	-0.031287	-0.008423	0.011900	-0.001924	0.008237	
0.016482	-0.001770	-0.010369	0.005886	0.007271	0.010882	-
0.028963	-0.004421					
z419	-0.004045	0.005155	-0.013578	0.010219	-0.018951	-
0.013755	-0.001348	-0.009071	-0.013740	0.015632	-0.000006	-
0.002870	-0.003496	0.014164	-0.029900	-0.011575	-0.017823	-
0.008244	-0.002098	-0.000101	0.017993	-0.016252	-0.007772	-
0.004987	0.002252	0.002355	0.007646	0.001599	-0.013660	
0.001162	0.004957	-0.001540	-0.017230	0.028242	-0.021090	
0.001628	-0.005763					
x420	0.026617	-0.007865	-0.004592	-0.057388	-0.012653	
0.033191	0.011276	-0.003622	0.009061	-0.005994	0.017591	-

0.004119	-0.001769	-0.003958	0.010233	-0.022867	0.013572		
0.007405	0.004504	-0.023917	-0.012057	-0.007400	0.014688	-
0.038101	0.015769	-0.001243	0.024464	0.035505	-0.009230		
0.004151	0.001668	0.007694	0.002635	0.000219	0.018184	-
0.005242	-0.016098						
y420	-0.026776	-0.003189	0.005532	-0.016458	-0.017233	-
0.025456	-0.014164	0.013960	-0.009128	-0.021422	0.000635	-
0.002167	-0.021162	0.032907	-0.009886	-0.005238	0.010042	-
0.005120	-0.001048	0.014463	0.012699	-0.019240	0.007875	-
0.001138	-0.027434	-0.004635	0.012746	-0.003283	0.002889		
0.021369	-0.001205	-0.012254	0.006297	0.005964	0.004095	-
0.030346	-0.009980						
z420	-0.005299	0.008059	-0.014781	0.013884	-0.020236	-
0.017913	-0.002117	-0.008910	-0.014990	0.017359	-0.004254	-
0.000173	0.001722	0.014973	-0.031574	-0.008096	-0.018196	-
0.010608	-0.000755	0.007628	0.014740	-0.014172	-0.009384	-
0.006600	-0.000428	0.005888	0.005999	0.004663	-0.013290	-
0.003066	0.000212	-0.006291	-0.017738	0.023619	-0.019783	-
0.002157	-0.002277						
x421	0.024126	-0.009733	-0.005214	-0.057560	-0.013790		
0.030141	0.009680	-0.000203	0.008408	-0.002421	0.016045	-
0.003746	-0.002250	-0.001840	0.006298	-0.021992	0.013854		
0.004518	0.001656	-0.020264	-0.010931	-0.004946	0.015194	-
0.035765	0.014634	-0.003157	0.017403	0.035880	-0.016849		
0.002184	-0.001180	0.000322	0.006083	0.000750	0.010818	-
0.016439	-0.015819						
y421	-0.027418	-0.001382	0.004926	-0.013987	-0.018060	-
0.026055	-0.010714	0.011573	-0.009562	-0.027262	0.003294	-
0.004583	-0.019900	0.030207	-0.011714	-0.006053	0.004700	-
0.006783	0.002735	0.013756	0.013794	-0.019913	0.007047	-
0.004323	-0.025861	0.000387	0.011994	0.000426	0.003673		
0.022112	-0.002832	-0.006164	0.000167	-0.000159	0.004822	-
0.022755	-0.008302						
z421	-0.006423	0.011405	-0.015720	0.017956	-0.021426	-
0.021531	-0.002841	-0.007149	-0.015739	0.018481	-0.008930		
0.002053	0.006410	0.016558	-0.032428	-0.003122	-0.016039	-
0.013183	0.001156	0.012926	0.011764	-0.013866	-0.009599	-
0.008230	-0.001246	0.006707	0.001691	0.006153	-0.013838	-
0.005607	-0.005161	-0.009454	-0.015036	0.017563	-0.018840	-
0.006451	-0.000674						
x422	0.022292	-0.011659	-0.007055	-0.057345	-0.013680		
0.027161	0.010336	0.000145	0.008140	-0.000429	0.013384	-
0.002560	-0.003379	-0.002768	0.004390	-0.019906	0.010218		
0.006706	0.002572	-0.016220	-0.003902	0.003471	0.015842	-
0.030184	0.013631	0.000301	0.010753	0.037383	-0.019340		
0.001633	-0.000575	-0.001565	-0.000835	-0.001025	0.007661	-
0.015959	-0.009470						
y422	-0.027810	0.000486	0.004326	-0.011997	-0.021422	-
0.027456	-0.009693	0.012085	-0.009799	-0.030187	0.005015	-
0.006027	-0.017297	0.029541	-0.016079	-0.005852	0.002946	-
0.010672	0.004781	0.012770	0.011275	-0.022067	0.004384	-
0.007315	-0.026483	0.003086	0.010134	-0.000389	0.003016		
0.022794	-0.004854	-0.003673	-0.002301	-0.006676	0.006681	-
0.025289	-0.008537						
z422	-0.006793	0.015322	-0.017607	0.022998	-0.020877	-
0.023884	-0.002709	-0.005500	-0.015867	0.018224	-0.012557		
0.003635	0.008981	0.016975	-0.031845	0.003515	-0.013774	-
0.013791	0.006510	0.018386	0.010898	-0.012177	-0.007580	-
0.010731	-0.000231	0.008256	-0.002915	0.007962	-0.013041	-

0.008166 -0.007179 -0.010193 -0.011911 0.008948 -0.016205 -
0.006260 0.002862
x423 0.021119 -0.013583 -0.009683 -0.055509 -0.015404
0.022203 0.008802 -0.000497 0.007201 0.004092 0.008175 -
0.001324 -0.003410 -0.005369 0.000156 -0.015831 0.006444
0.008981 0.000056 -0.011618 0.004628 0.010833 0.011880 -
0.023920 0.010450 0.005387 0.008875 0.033428 -0.018338 -
0.000059 0.001115 -0.003906 -0.011968 -0.007912 0.008402 -
0.018476 -0.005374
y423 -0.027400 0.002974 0.003935 -0.010226 -0.024005 -
0.026886 -0.007662 0.014182 -0.008323 -0.034923 0.008127 -
0.008711 -0.014773 0.028072 -0.016543 -0.005701 0.001982 -
0.013362 0.014451 0.012166 0.009131 -0.020711 0.004897 -
0.011264 -0.023735 0.006348 0.000744 0.007341 -0.000949
0.024625 -0.003350 -0.000560 -0.004340 -0.011093 0.007083 -
0.020371 -0.003074
z423 -0.006846 0.018781 -0.020025 0.028319 -0.019914 -
0.026106 -0.002272 -0.003878 -0.015208 0.018257 -0.017512
0.006102 0.011190 0.017389 -0.031060 0.011789 -0.010905 -
0.014913 0.011123 0.021291 0.010496 -0.010951 -0.005716 -
0.013963 0.000232 0.006363 -0.006419 0.006669 -0.009203 -
0.010335 -0.011992 -0.006706 -0.009686 -0.003485 -0.008379 -
0.008401 0.004327
x424 0.020992 -0.014447 -0.011634 -0.051964 -0.019012
0.015610 0.006193 0.000637 0.006679 0.009899 0.001649 -
0.002271 -0.001596 -0.008380 -0.004310 -0.010931 0.000856
0.009063 -0.001054 -0.006999 0.013240 0.018251 0.006162 -
0.014942 0.007650 0.009765 0.002945 0.036033 -0.018325
0.000096 0.008266 -0.002728 -0.024980 -0.015653 0.008938 -
0.020558 -0.001779
y424 -0.027200 0.004378 0.002316 -0.008702 -0.025080 -
0.024323 -0.005174 0.015275 -0.007424 -0.040520 0.010810 -
0.008125 -0.013083 0.026075 -0.016777 -0.004367 0.002222 -
0.012926 0.022430 0.012865 0.008627 -0.016951 0.004894 -
0.014451 -0.021255 0.009896 -0.007809 0.004878 -0.001863
0.023298 -0.006917 0.000293 -0.006963 -0.018817 0.010358 -
0.016196 0.004215
z424 -0.005949 0.023410 -0.022753 0.033910 -0.018439 -
0.028017 -0.002040 -0.000878 -0.013470 0.018189 -0.021238
0.007012 0.012309 0.017599 -0.029787 0.021619 -0.007517 -
0.016792 0.018603 0.022573 0.010296 -0.009392 -0.001552 -
0.017405 0.002783 0.003547 -0.011694 0.008857 -0.004900 -
0.012363 -0.013243 0.000451 -0.005587 -0.017638 0.001178 -
0.009336 0.006069
x425 0.021150 -0.015201 -0.013757 -0.045495 -0.021567
0.008760 0.003345 0.003135 0.005562 0.013690 -0.003951 -
0.002254 0.000990 -0.011881 -0.007875 -0.006356 -0.004876
0.009904 -0.000791 -0.001878 0.019563 0.026173 -0.002255 -
0.005978 0.009613 0.010971 -0.004602 0.034097 -0.020533 -
0.001362 0.009633 -0.006664 -0.032983 -0.018208 0.003833 -
0.016678 0.005575
y425 -0.026981 0.005165 -0.000103 -0.009204 -0.026867 -
0.023793 -0.003284 0.015238 -0.006939 -0.043757 0.011706 -
0.007681 -0.011021 0.026702 -0.019734 0.000711 0.001395 -
0.011360 0.031488 0.016117 0.013026 -0.009217 0.010657 -
0.011998 -0.022422 0.017209 -0.019349 0.000943 -0.000386
0.023828 -0.004355 0.004356 -0.018561 -0.024621 0.019247 -
0.014688 0.012826
z425 -0.004645 0.029007 -0.024833 0.040022 -0.016197 -
0.028934 -0.001272 0.003511 -0.011126 0.017008 -0.024260

0.009038	0.011709	0.017351	-0.028099	0.032379	-0.000719	-
0.017283	0.027053	0.022588	0.009113	-0.008226	0.002158	-
0.019038	0.006611	-0.001319	-0.016800	0.009397	-0.000770	-
0.014320	-0.014240	0.007261	0.001539	-0.027428	0.009792	-
0.007302	0.009652					
x426	0.019954	-0.013232	-0.016828	-0.035991	-0.020356	
0.010029	0.003070	0.004152	0.001727	0.022407	-0.012662	-
0.001267	0.004191	-0.008709	-0.005799	-0.002835	-0.001654	
0.004330	-0.009058	0.009687	0.020529	0.014887	-0.004836	-
0.006647	0.010863	0.007606	-0.001990	0.028163	-0.007716	-
0.004432	-0.003390	-0.014469	-0.027801	-0.018313	-0.011432	-
0.006506	-0.002012					
y426	-0.027232	0.007173	-0.000008	-0.007773	-0.027874	-
0.012569	-0.001405	0.011219	-0.007642	-0.044091	0.006438	-
0.005723	-0.009520	0.026677	-0.011806	0.008665	-0.004256	-
0.009619	0.024451	0.022988	0.009157	-0.000157	0.002489	-
0.006024	-0.021589	0.013939	-0.016125	-0.003920	0.001365	
0.022706	-0.010739	0.006211	-0.011060	-0.018333	0.028099	-
0.008678	0.023601					
z426	-0.001949	0.035724	-0.028248	0.041677	-0.016126	-
0.023874	-0.004701	0.012381	-0.012654	0.007646	-0.014311	
0.012030	0.011087	0.009495	-0.034853	0.034307	-0.005056	-
0.019856	0.029870	0.010364	0.005336	0.000442	0.015138	-
0.028565	0.015808	0.009445	-0.012901	0.013398	-0.009363	-
0.008385	-0.003287	0.003111	0.018236	-0.030414	0.008766	-
0.011334	0.001193					
x427	0.019794	-0.010188	-0.018782	-0.026392	-0.020874	
0.007407	0.000946	0.006156	-0.000922	0.028334	-0.017031	-
0.001350	0.004753	-0.011579	-0.005383	0.005144	-0.000281	
0.004252	-0.016129	0.020897	0.021872	0.015913	-0.005933	-
0.000562	0.011354	0.003556	0.002808	0.022258	-0.007491	-
0.011871	-0.007778	-0.016255	-0.022428	-0.019913	-0.020247	-
0.006981	-0.002442					
y427	-0.026696	0.008099	-0.000570	-0.006995	-0.027451	-
0.000728	0.001669	0.006333	-0.009791	-0.043201	0.002066	-
0.003384	-0.006107	0.029455	-0.004718	0.010230	-0.008624	-
0.012364	0.021530	0.025353	0.002351	-0.005645	-0.005217	-
0.008428	-0.020082	0.013458	-0.018235	-0.005264	0.011157	
0.023088	-0.017863	0.005636	-0.003797	-0.015659	0.033347	
0.004786	0.028378					
z427	0.000574	0.040494	-0.030097	0.042959	-0.015919	-
0.020938	-0.008335	0.020128	-0.013926	-0.002130	-0.005227	
0.013283	0.009647	0.000918	-0.038177	0.036293	-0.011263	-
0.020640	0.031124	-0.002330	0.001782	0.010943	0.024741	-
0.034997	0.023892	0.015118	-0.007043	0.015570	-0.018790	-
0.003822	0.005713	0.002853	0.031334	-0.032329	0.011323	-
0.020764	-0.003900					
x428	0.019188	-0.005275	-0.020013	-0.017463	-0.020511	
0.005937	-0.000599	0.008481	-0.004132	0.027896	-0.018215	-
0.001333	0.006884	-0.009449	-0.005707	0.007125	-0.000904	-
0.000222	-0.015968	0.022974	0.018582	0.015000	-0.004346	-
0.003524	0.009518	0.001884	0.008816	0.017379	0.004683	-
0.012067	-0.014480	-0.021302	-0.014134	-0.015110	-0.022509	-
0.012050	-0.012212					
y428	-0.025476	0.007891	-0.000196	-0.006293	-0.027641	
0.006593	0.003735	0.001804	-0.011543	-0.039675	-0.002090	-
0.000538	-0.005037	0.027647	-0.001928	0.016661	-0.011303	-
0.010966	0.016427	0.030634	-0.000221	-0.007114	-0.010245	-
0.001953	-0.019777	0.012251	-0.020168	-0.006945	0.012386	

0.017598 -0.017867 0.008685 0.000158 -0.016006 0.034788
0.015557 0.039244
z428 0.002503 0.043367 -0.030053 0.042152 -0.015019 -
0.018565 -0.010906 0.026135 -0.014840 -0.011536 0.002701
0.014615 0.009662 -0.004676 -0.041229 0.036028 -0.016081 -
0.020923 0.033720 -0.013409 -0.001639 0.016589 0.033565 -
0.038614 0.029434 0.019099 -0.000902 0.016563 -0.024298
0.002637 0.011619 0.003300 0.041108 -0.031447 0.013940 -
0.029472 -0.009181
x429 0.019640 -0.000021 -0.021822 -0.010979 -0.021056
0.004365 -0.001732 0.012059 -0.005154 0.026189 -0.016833 -
0.002061 0.007410 -0.012528 -0.007298 0.011403 -0.002242 -
0.005623 -0.015641 0.018625 0.015212 0.017967 -0.002666 -
0.005850 0.011195 0.000482 0.011193 0.012604 0.002114 -
0.008414 -0.020360 -0.021651 -0.009219 -0.016862 -0.025183 -
0.021152 -0.022836
y429 -0.024193 0.007997 0.002635 -0.004702 -0.026622
0.011418 0.004702 -0.003457 -0.015122 -0.035828 -0.005416
0.003118 -0.002320 0.029181 -0.001200 0.021544 -0.015031 -
0.011010 0.017357 0.036111 -0.001788 -0.010891 -0.012952
0.003798 -0.023491 0.012800 -0.019620 -0.010892 0.023034
0.004075 -0.012365 0.010383 0.007750 -0.012822 0.040345
0.028210 0.053236
z429 0.004131 0.043811 -0.027688 0.041065 -0.013605 -
0.017895 -0.012230 0.029885 -0.016389 -0.019710 0.007795
0.015631 0.010335 -0.008441 -0.042012 0.035296 -0.020821 -
0.019914 0.038243 -0.022159 -0.004136 0.015634 0.038378 -
0.037897 0.031550 0.022099 0.004313 0.017125 -0.028448
0.006029 0.017171 0.005689 0.047934 -0.031125 0.018133 -
0.033956 -0.007654
x430 0.021326 0.005332 -0.024903 -0.001832 -0.022243 -
0.001369 -0.002291 0.015634 -0.008824 0.018545 -0.017580 -
0.001770 0.006222 -0.013110 -0.010678 0.017445 -0.005274 -
0.009903 -0.010487 0.010417 0.012732 0.012541 -0.000919 -
0.003072 0.009307 0.004031 0.015236 0.010938 -0.000032 -
0.011723 -0.017981 -0.016550 -0.000102 -0.023351 -0.026795 -
0.015532 -0.018944
y430 -0.022486 0.008643 0.005070 -0.006291 -0.025187
0.016992 0.004810 -0.007259 -0.014533 -0.032351 0.000663
0.006261 0.001639 0.025815 -0.003700 0.022036 -0.015234 -
0.010929 0.015039 0.040667 -0.005795 -0.006886 -0.011936
0.002658 -0.026368 0.011224 -0.020276 -0.010876 0.029816 -
0.000805 -0.010226 0.006600 0.007322 -0.010046 0.040426
0.022967 0.047906
z430 0.005142 0.042488 -0.025392 0.039714 -0.011831 -
0.018468 -0.012465 0.031616 -0.017555 -0.029850 0.012414
0.014718 0.010366 -0.013667 -0.042228 0.033385 -0.024115 -
0.016111 0.038919 -0.027964 -0.005283 0.010785 0.038976 -
0.034274 0.033864 0.023086 0.010266 0.018866 -0.031146
0.012949 0.019882 0.006255 0.047574 -0.031310 0.015549 -
0.036137 -0.010832
x431 0.024098 0.011660 -0.024866 0.003235 -0.021274 -
0.006833 -0.001292 0.017976 -0.012906 0.009690 -0.005722
0.002863 0.009872 -0.012732 -0.017833 0.018527 -0.002696 -
0.009446 0.000849 0.003707 0.002344 -0.000813 0.007224 -
0.003127 -0.002048 0.010350 0.019372 0.016572 0.000050 -
0.005309 -0.005621 -0.007641 0.007859 -0.033390 -0.013138 -
0.018648 -0.005117
y431 -0.021278 0.008114 0.007054 -0.003925 -0.026195
0.017897 0.002451 -0.009570 -0.014199 -0.029560 -0.003834

0.005696	0.000692	0.021134	-0.005405	0.028602	-0.023615	-
0.014845	0.012423	0.038589	-0.003653	0.004903	-0.014802	
0.005680	-0.022677	0.007040	-0.021552	-0.018763	0.034924	-
0.016587	-0.009269	-0.000182	0.005505	-0.002200	0.032574	
0.021741	0.037788					
z431	0.006121	0.039446	-0.019502	0.035225	-0.009232	-
0.017604	-0.011219	0.030344	-0.017373	-0.036913	0.016940	
0.014206	0.012100	-0.018919	-0.041785	0.028598	-0.025495	-
0.012992	0.042684	-0.031377	-0.004757	0.003969	0.037940	-
0.028502	0.035563	0.020775	0.015710	0.017222	-0.031550	
0.013149	0.021099	0.006036	0.044145	-0.026074	0.012956	-
0.038000	-0.014098					
x432	0.026899	0.017459	-0.022192	0.010253	-0.023504	-
0.014878	-0.003875	0.020769	-0.016813	-0.006967	0.003394	
0.005332	0.010977	-0.012565	-0.025506	0.022906	-0.008279	-
0.007816	0.013962	-0.004381	-0.007834	-0.012841	0.012324	-
0.002102	-0.007690	0.015209	0.025556	0.022240	-0.001985	-
0.000345	0.010211	-0.002033	0.011147	-0.041818	0.004135	-
0.028064	0.009042					
y432	-0.018767	0.008112	0.007814	-0.005081	-0.024447	
0.018666	0.004142	-0.012014	-0.013456	-0.021042	-0.000869	
0.008463	0.001357	0.014260	-0.009625	0.028868	-0.024231	-
0.019350	0.010230	0.032778	-0.003853	0.016871	-0.012175	
0.006280	-0.025654	0.005019	-0.026578	-0.023208	0.039496	-
0.033679	-0.007035	-0.004356	0.004901	0.004269	0.023722	
0.021418	0.022918					
z432	0.006825	0.034069	-0.012540	0.030020	-0.005906	-
0.018752	-0.008305	0.027560	-0.017372	-0.043991	0.019611	
0.012642	0.013649	-0.023978	-0.038589	0.023465	-0.026886	-
0.008108	0.044347	-0.034012	-0.004891	-0.005658	0.033385	-
0.019592	0.034551	0.014725	0.021853	0.015330	-0.029901	
0.013704	0.020229	0.007286	0.034879	-0.019532	0.009991	-
0.038520	-0.016925					
x433	0.030260	0.022278	-0.016923	0.013913	-0.025874	-
0.021728	-0.005142	0.021173	-0.019263	-0.024188	0.015603	
0.009285	0.010136	-0.019615	-0.035324	0.026101	-0.013605	-
0.007885	0.030980	-0.016587	-0.017726	-0.021912	0.017026	-
0.000778	-0.015796	0.018348	0.029990	0.024560	-0.004600	-
0.001396	0.031363	0.001835	0.009591	-0.039856	0.022174	-
0.038705	0.023576					
y433	-0.015158	0.009480	0.006199	-0.003880	-0.022214	
0.014920	0.004858	-0.010679	-0.013226	-0.015422	0.004546	
0.008286	0.002133	0.011006	-0.013674	0.028529	-0.025399	-
0.021297	0.006651	0.024411	-0.007039	0.028848	-0.007016	
0.003083	-0.025315	0.005879	-0.029444	-0.023690	0.042225	-
0.046051	-0.004516	-0.007304	0.006956	-0.001991	0.011922	
0.015746	0.003240					
z433	0.007628	0.027177	-0.004702	0.023775	-0.001551	-
0.021999	-0.003512	0.023956	-0.017697	-0.051175	0.022293	
0.010950	0.016712	-0.024907	-0.033638	0.017143	-0.027719	
0.000248	0.044525	-0.034398	-0.006853	-0.020616	0.025851	-
0.008254	0.030807	0.007190	0.028401	0.016269	-0.026732	
0.016927	0.017584	0.008991	0.020339	-0.015652	0.008090	-
0.039086	-0.017374					
x434	0.033981	0.027510	-0.011527	0.014694	-0.029238	-
0.027489	-0.005041	0.023149	-0.018906	-0.040354	0.028116	
0.012307	0.006716	-0.029512	-0.044507	0.030913	-0.020630	-
0.006339	0.051445	-0.030594	-0.027940	-0.023258	0.019698	-
0.005741	-0.019774	0.014427	0.033887	0.018992	-0.009097	-

0.007002 0.053330 0.005189 0.013177 -0.037305 0.030261 -
0.045178 0.030219
y434 -0.010053 0.012069 0.002183 -0.000035 -0.017878
0.009643 0.002734 -0.011092 -0.015695 -0.017009 0.017397
0.007222 0.005239 0.005983 -0.019971 0.025419 -0.027438 -
0.027008 0.004844 0.009359 -0.011960 0.033429 0.005024 -
0.000531 -0.027036 0.017827 -0.032687 -0.011389 0.046803 -
0.055979 0.001670 -0.001978 0.009749 -0.019250 0.015838
0.005170 -0.006513
z434 0.008532 0.019081 0.004307 0.017604 0.003586 -
0.026849 0.001616 0.017417 -0.019827 -0.060588 0.025604
0.009128 0.021178 -0.024143 -0.029054 0.007940 -0.027127
0.008850 0.046064 -0.034083 -0.009319 -0.044637 0.017123
0.007405 0.022454 0.002256 0.034284 0.021006 -0.021856
0.022004 0.016033 0.013249 0.000510 -0.012019 0.014401 -
0.039745 -0.007918
x435 0.033324 0.027652 -0.005947 0.011697 -0.026169 -
0.022407 0.000698 0.016922 -0.018775 -0.043834 0.025000
0.020761 0.010583 -0.033625 -0.042982 0.020259 -0.022632 -
0.006219 0.051086 -0.023659 -0.022879 -0.024872 0.022937
0.002062 -0.023391 0.008732 0.036400 0.019561 -0.013611 -
0.003565 0.041307 -0.001217 0.000056 -0.022804 0.020700 -
0.016149 0.019919
y435 -0.003474 0.011030 0.005951 0.003963 -0.013916
0.006376 0.005795 -0.010488 -0.014589 -0.020916 0.020099
0.013501 0.011537 -0.003154 -0.023877 0.008727 -0.032575 -
0.028898 0.009442 0.002256 -0.008376 0.021322 0.004189
0.002076 -0.022722 0.006999 -0.021706 -0.003048 0.036235 -
0.049760 -0.012525 0.000663 0.001752 -0.021970 0.017920
0.000341 -0.004581
z435 0.003942 0.013551 0.003692 0.015197 0.008082 -
0.020848 0.002893 0.012935 -0.012962 -0.050901 0.018464
0.007845 0.018459 -0.013887 -0.012550 0.004541 -0.020488
0.013150 0.034570 -0.024505 -0.003955 -0.038777 0.007582
0.013943 0.020938 -0.002027 0.030719 0.020166 -0.017463
0.010029 0.011610 0.008227 0.001403 -0.006368 0.013400 -
0.020210 -0.003815
x436 0.033195 0.027884 -0.002153 0.010376 -0.024478 -
0.019393 0.005212 0.012559 -0.017622 -0.048140 0.019517
0.025293 0.011213 -0.036411 -0.040152 0.012771 -0.024904 -
0.008770 0.048003 -0.018888 -0.017899 -0.029235 0.025579
0.013281 -0.026799 0.001116 0.033995 0.022340 -0.017588 -
0.002433 0.029816 -0.001144 -0.010654 -0.013869 0.013076
0.007562 0.011668
y436 -0.000713 0.007799 0.010559 0.006335 -0.009101
0.004687 0.007840 -0.010075 -0.015099 -0.020496 0.025083
0.023850 0.021958 -0.009778 -0.027380 -0.006501 -0.034986 -
0.024162 0.016425 -0.002755 -0.005965 0.019520 0.002674 -
0.003547 -0.015440 0.002171 -0.002651 0.003242 0.028402 -
0.039103 -0.021961 -0.008828 -0.004618 -0.013286 0.021555 -
0.001225 -0.003070
z436 -0.000722 0.008293 0.003523 0.012246 0.012332 -
0.014977 0.004068 0.008937 -0.007224 -0.040593 0.013205
0.006131 0.017409 -0.003493 0.001916 0.001017 -0.013890
0.017401 0.025146 -0.015767 0.000353 -0.031696 0.000089
0.015850 0.022420 -0.004323 0.028982 0.017888 -0.013321
0.002016 0.010141 0.001843 0.003649 0.000556 0.012161 -
0.003780 -0.001259
x437 0.032285 0.026948 0.001627 0.010118 -0.023660 -
0.016965 0.009350 0.008824 -0.016401 -0.051705 0.015402

0.028618	0.012792	-0.038893	-0.036942	0.004813	-0.029656	-
0.010014	0.045201	-0.016720	-0.013467	-0.028627	0.026603	
0.021408	-0.029092	-0.006474	0.033957	0.024053	-0.019285	-
0.002163	0.015644	-0.000722	-0.018391	-0.006855	0.008138	
0.020975	0.008971					
y437	0.001858	0.004865	0.014089	0.006607	-0.002853	
0.002526	0.009605	-0.008925	-0.016400	-0.020182	0.028139	
0.032114	0.027389	-0.012680	-0.031052	-0.015374	-0.033547	-
0.022713	0.017543	-0.001397	-0.002927	0.010969	0.002049	-
0.002330	-0.009856	0.001737	0.010893	0.007178	0.023592	-
0.028074	-0.023505	-0.015037	-0.008936	-0.007674	0.024880	
0.006106	-0.010700					
z437	-0.004636	0.003938	0.002812	0.008326	0.016144	-
0.010477	0.004342	0.005845	-0.003167	-0.031131	0.006835	
0.002881	0.014343	0.007756	0.014672	0.000431	-0.006084	
0.018668	0.014974	-0.006257	0.003509	-0.027953	-0.005808	
0.018723	0.024706	-0.003704	0.025205	0.012916	-0.008948	-
0.003755	0.012414	-0.002038	0.005720	0.005005	0.010670	
0.010184	-0.001935					
x438	0.031735	0.025482	0.005218	0.007377	-0.020585	-
0.014307	0.014900	0.005172	-0.014456	-0.054470	0.016316	
0.035494	0.015164	-0.039296	-0.035203	-0.004915	-0.029704	-
0.010962	0.044933	-0.014104	-0.008038	-0.028507	0.029330	
0.025777	-0.029631	-0.007816	0.036824	0.026067	-0.017557	-
0.000241	0.002471	-0.002276	-0.024121	-0.000272	0.008036	
0.034740	0.011187					
y438	0.003495	0.001498	0.015525	0.009589	0.000494	-
0.000349	0.008095	-0.005341	-0.015924	-0.018719	0.026447	
0.032230	0.030706	-0.015633	-0.027703	-0.018983	-0.036436	-
0.021918	0.013819	-0.003801	-0.004605	0.005773	0.000145	
0.000839	-0.006718	-0.001418	0.013264	0.008748	0.021059	-
0.021317	-0.027185	-0.014570	-0.008382	-0.002837	0.020289	
0.009434	-0.014797					
z438	-0.007795	0.000481	0.001090	0.004729	0.018561	-
0.006565	0.003741	0.003805	0.000690	-0.021128	0.000421	-
0.003643	0.010693	0.017505	0.027019	0.001026	0.000595	
0.019454	0.004760	0.000431	0.004236	-0.024055	-0.009692	
0.020524	0.026376	-0.001673	0.017676	0.005440	-0.005184	-
0.009276	0.014227	-0.004218	0.009178	0.007478	0.008687	
0.018521	-0.002571					
x439	0.030613	0.023105	0.008257	0.006384	-0.018026	-
0.013179	0.019862	0.003626	-0.012366	-0.056545	0.016922	
0.040316	0.018019	-0.038070	-0.032706	-0.013446	-0.031342	-
0.011958	0.041685	-0.013309	-0.003051	-0.029730	0.029336	
0.030831	-0.027791	-0.008543	0.037789	0.029977	-0.013096	
0.004152	-0.008427	-0.003376	-0.023660	0.004540	0.006888	
0.046814	0.010279					
y439	0.003840	-0.002544	0.014875	0.010184	0.004341	-
0.001598	0.005722	-0.003369	-0.013004	-0.015153	0.025355	
0.031723	0.032243	-0.016894	-0.023885	-0.018408	-0.036962	-
0.019925	0.014260	-0.005813	-0.007328	0.008707	0.001186	-
0.000326	-0.002182	-0.002855	0.013600	0.006937	0.018134	-
0.018513	-0.029529	-0.010449	-0.011157	0.003532	0.017981	
0.007097	-0.012464					
z439	-0.010081	-0.002292	-0.001250	0.000295	0.020078	-
0.002940	0.002792	0.001251	0.004390	-0.011448	-0.005593	-
0.010996	0.006753	0.026406	0.037078	0.003392	0.008580	
0.018275	-0.003196	0.005377	0.003559	-0.019542	-0.011263	
0.019847	0.029855	0.001186	0.009536	-0.003559	-0.003516	-

0.014630	0.017167	-0.004377	0.012079	0.007911	0.008637	
0.019690	-0.003292					
x440	0.029149	0.019819	0.010178	0.005406	-0.016304	-
0.011704	0.023622	0.003211	-0.010409	-0.058127	0.018500	
0.044039	0.019974	-0.036186	-0.028455	-0.020272	-0.034095	-
0.014881	0.037324	-0.013531	-0.000708	-0.028707	0.029548	
0.032646	-0.022963	-0.007723	0.036820	0.034724	-0.006756	
0.006745	-0.019911	-0.001345	-0.019076	0.004750	0.009545	
0.049337	0.009354					
y440	0.003131	-0.006295	0.016028	0.012215	0.009269	-
0.004462	0.004370	-0.002052	-0.011101	-0.011920	0.022334	
0.028076	0.032607	-0.016178	-0.019505	-0.016623	-0.031323	-
0.015124	0.014072	-0.007199	-0.008009	0.008582	-0.002767	
0.000610	-0.002243	-0.005512	0.016741	0.001434	0.018614	-
0.015176	-0.025034	-0.007672	-0.010387	0.012586	0.011028	
0.010684	-0.007631					
z440	-0.011691	-0.003890	-0.003127	-0.004038	0.020799	-
0.000516	0.001721	-0.000993	0.006761	-0.002647	-0.011696	-
0.019980	0.002782	0.035269	0.044562	0.006332	0.017973	
0.016268	-0.010571	0.009443	0.003194	-0.017967	-0.011921	
0.019270	0.033176	0.004129	0.002206	-0.013065	-0.002067	-
0.017102	0.023166	-0.003384	0.015369	0.006990	0.008115	
0.017704	-0.004117					
x441	0.026475	0.015601	0.011901	0.003786	-0.013978	-
0.011667	0.027191	0.003740	-0.007990	-0.058087	0.021239	
0.046910	0.021391	-0.032019	-0.024729	-0.025259	-0.035159	-
0.016249	0.031813	-0.014775	0.002662	-0.023847	0.029218	
0.034541	-0.016594	-0.005279	0.036148	0.036490	0.006577	
0.010304	-0.028522	0.002365	-0.010113	0.006953	0.014066	
0.044161	0.009920					
y441	0.002126	-0.009768	0.014671	0.015642	0.014696	-
0.007447	0.001975	0.000889	-0.009080	-0.007592	0.017713	
0.021934	0.032710	-0.012374	-0.014024	-0.012391	-0.029946	-
0.013047	0.010116	-0.006037	-0.010951	0.005276	-0.006614	-
0.001117	-0.002264	-0.005496	0.017024	-0.001306	0.014187	-
0.012850	-0.022810	-0.004546	-0.013128	0.014687	0.006717	
0.017216	-0.003285					
z441	-0.012222	-0.004313	-0.005977	-0.008591	0.020519	
0.001308	0.000288	-0.002662	0.007520	0.005555	-0.017347	-
0.030099	-0.001540	0.043683	0.051343	0.009386	0.026520	
0.013126	-0.016600	0.012454	0.001530	-0.019088	-0.010997	
0.015768	0.038119	0.009839	-0.005545	-0.023672	-0.003891	-
0.018113	0.030047	-0.000985	0.019433	0.000119	0.008327	
0.012302	-0.004368					
x442	0.022866	0.010021	0.011171	0.004953	-0.010568	-
0.012397	0.029514	0.005631	-0.006098	-0.057360	0.022904	
0.045684	0.021702	-0.028814	-0.018468	-0.027220	-0.037505	-
0.016167	0.024890	-0.017110	0.002567	-0.018063	0.024951	
0.033059	-0.007607	-0.000942	0.034204	0.037799	0.019046	
0.011735	-0.036004	0.008141	0.001191	0.002533	0.017499	
0.040862	0.014394					
y442	0.001589	-0.011282	0.016808	0.014122	0.016097	-
0.008233	0.001582	0.003711	-0.006623	-0.005121	0.012615	
0.016794	0.030978	-0.008325	-0.006238	-0.010376	-0.019864	-
0.006128	0.006511	-0.003333	-0.010621	0.004149	-0.007177	-
0.002235	-0.000617	-0.003535	0.015450	-0.003960	0.016223	-
0.009632	-0.021147	-0.003780	-0.004845	0.018207	0.009351	
0.011813	-0.004010					
z442	-0.012207	-0.003828	-0.008709	-0.014233	0.018566	
0.002266	-0.001760	-0.004087	0.007018	0.012952	-0.022707	-

0.040926	-0.006656	0.051856	0.056539	0.013261	0.035016	
0.008310	-0.021667	0.012893	-0.000414	-0.019955	-0.006978	
0.010423	0.043817	0.016657	-0.013079	-0.036519	-0.002921	-
0.016848	0.037303	0.002023	0.024356	-0.006216	0.010768	-
0.002391	-0.004803					
x443	0.018288	0.003081	0.011132	0.004873	-0.007012	-
0.014477	0.031186	0.008933	-0.003928	-0.054998	0.024456	
0.046068	0.022314	-0.021645	-0.013507	-0.026643	-0.040320	-
0.013183	0.017862	-0.017426	0.003735	-0.007219	0.021167	
0.034386	0.000654	0.004379	0.035193	0.034125	0.030841	
0.016176	-0.038870	0.008992	0.010435	0.004919	0.021752	
0.033996	0.018188					
y443	0.000961	-0.013606	0.015767	0.013260	0.017511	-
0.009032	-0.000056	0.006812	-0.002901	-0.001617	0.009744	
0.006387	0.027456	-0.002212	0.001477	-0.005850	-0.009214	-
0.004155	0.003630	-0.002198	-0.011114	0.000514	-0.008642	-
0.007361	0.000138	0.003206	0.012103	-0.007086	0.019190	-
0.004535	-0.017832	0.005053	0.010508	0.009933	0.011237	
0.000367	-0.000374					
z443	-0.011426	-0.002983	-0.013177	-0.019179	0.016267	
0.003186	-0.004015	-0.005961	0.004882	0.020526	-0.027987	-
0.052261	-0.013327	0.059068	0.059465	0.017985	0.043370	
0.003665	-0.024885	0.012243	-0.004782	-0.020939	-0.001589	
0.002348	0.047953	0.028938	-0.023605	-0.052874	-0.001555	-
0.014688	0.046169	0.003252	0.029848	-0.012283	0.014887	-
0.019781	-0.007142					
x444	0.018645	-0.000472	0.009607	0.002275	-0.006403	-
0.010020	0.027532	0.012464	0.000596	-0.046230	0.020982	
0.035325	0.016951	-0.015530	-0.000542	-0.024524	-0.036144	-
0.015757	0.011253	-0.018314	0.003122	-0.005288	0.021761	
0.027760	0.010413	0.001099	0.039069	0.022330	0.030254	
0.008932	-0.033022	0.014009	0.016063	0.000400	0.019580	
0.021998	0.008761					
y444	0.000395	-0.012508	0.013956	0.009992	0.017780	-
0.008204	-0.001914	0.007971	0.001780	0.001759	0.004539	-
0.002958	0.022626	0.005518	0.008152	-0.001260	0.000371	-
0.000914	0.001752	-0.003839	-0.005966	-0.005412	-0.002343	-
0.008646	0.000686	0.003935	0.006457	-0.008516	0.014648	-
0.006445	-0.006230	0.000127	0.017603	0.004632	0.005836	
0.000088	-0.002262					
z444	-0.013258	-0.000486	-0.014995	-0.020141	0.015361	
0.001993	-0.003412	-0.008325	0.001618	0.029717	-0.030841	-
0.053115	-0.012148	0.063452	0.060000	0.017811	0.045125	
0.007867	-0.024635	0.009327	-0.000380	-0.013076	-0.001194	
0.002053	0.037623	0.028395	-0.021320	-0.038428	-0.002160	-
0.010215	0.045240	-0.004211	0.023279	-0.016732	0.012305	-
0.021999	-0.008143					
x445	0.020597	-0.003683	0.008530	0.000635	-0.006349	-
0.006486	0.024740	0.016807	0.002841	-0.037790	0.018692	
0.027177	0.015620	-0.015852	0.006568	-0.025027	-0.034109	-
0.016324	0.005807	-0.017129	0.004108	-0.007615	0.021417	
0.019884	0.018834	0.002078	0.039137	0.013266	0.029400	
0.005629	-0.028204	0.023037	0.020327	-0.001493	0.017759	
0.015975	0.001099					
y445	-0.002102	-0.012740	0.011416	0.007700	0.018583	-
0.008440	-0.003330	0.007683	0.004372	0.005765	-0.000956	-
0.011881	0.017778	0.016171	0.016748	0.005103	0.004963	-
0.000058	-0.000642	-0.008207	-0.002848	-0.002982	0.002167	-
0.008800	0.002671	-0.000272	0.004061	-0.009882	0.010943	-

0.005837 0.002803 -0.005160 0.017569 0.001288 0.002517 -
0.004063 -0.004525
z445 -0.014432 0.002139 -0.015666 -0.021185 0.013218
0.001151 -0.003945 -0.009917 -0.000327 0.036875 -0.033240 -
0.053228 -0.011165 0.066099 0.058933 0.017630 0.045712
0.011588 -0.025027 0.007771 0.000988 -0.005961 0.000359
0.001398 0.027721 0.026719 -0.019900 -0.026078 -0.001207 -
0.006190 0.040597 -0.010681 0.017457 -0.020522 0.011939 -
0.026037 -0.009241
x446 0.020236 -0.005708 0.006345 -0.002186 -0.005699 -
0.002622 0.021607 0.018559 0.005771 -0.029739 0.015552
0.016179 0.010478 -0.008719 0.017368 -0.023051 -0.028488 -
0.019913 -0.001313 -0.017552 0.002276 -0.006372 0.019091
0.015276 0.023971 0.000417 0.034726 0.010014 0.025085 -
0.000238 -0.025995 0.026265 0.026316 -0.012629 0.015396
0.006622 -0.005136
y446 -0.003653 -0.012802 0.010735 0.004930 0.017904 -
0.008769 -0.003978 0.008083 0.006612 0.007920 -0.005862 -
0.017967 0.014029 0.020696 0.021679 0.008430 0.012358
0.005797 -0.001300 -0.008752 0.002467 -0.002410 0.005976 -
0.010374 0.001716 0.002135 0.005879 -0.015695 0.010483 -
0.005189 0.010461 -0.009300 0.013246 -0.001027 0.003726 -
0.002101 -0.002461
z446 -0.014990 0.004695 -0.016322 -0.021279 0.010836 -
0.000004 -0.004584 -0.011453 -0.002773 0.042578 -0.033562 -
0.051742 -0.009034 0.066399 0.055250 0.017173 0.044173
0.014150 -0.025243 0.006873 0.000983 -0.000323 0.001360
0.000789 0.020807 0.022543 -0.019192 -0.013691 -0.001681 -
0.001865 0.035852 -0.015366 0.012475 -0.022515 0.010136 -
0.027902 -0.009595
x447 0.019605 -0.007574 0.003137 -0.003977 -0.004180 -
0.000913 0.018084 0.019710 0.005727 -0.023502 0.013235
0.007722 0.005634 -0.005534 0.023381 -0.019624 -0.022215 -
0.018973 -0.005897 -0.015979 0.004700 -0.002285 0.018974
0.009814 0.028781 0.003806 0.030966 0.006074 0.023543 -
0.004475 -0.017489 0.029200 0.029877 -0.017907 0.017837 -
0.002620 -0.003886
y447 -0.004969 -0.012626 0.011765 0.001538 0.016248 -
0.008148 -0.003147 0.008750 0.007251 0.011678 -0.008638 -
0.022987 0.012590 0.027200 0.025279 0.009313 0.014677
0.008046 -0.006519 -0.008427 -0.000394 -0.002819 0.007960 -
0.009334 -0.000552 -0.003212 0.002432 -0.018517 0.007925 -
0.002914 0.005685 -0.017391 0.007488 -0.003697 0.000531 -
0.001257 -0.005388
z447 -0.015459 0.006986 -0.017005 -0.021088 0.007908 -
0.001288 -0.005729 -0.013102 -0.004738 0.046016 -0.033438 -
0.048816 -0.007037 0.064934 0.049696 0.016158 0.042972
0.017822 -0.022858 0.006717 0.001789 0.004834 0.002098 -
0.000639 0.014365 0.019567 -0.016554 -0.005316 -0.003267
0.003616 0.031498 -0.018486 0.008996 -0.023753 0.008189 -
0.027575 -0.009787
x448 0.017545 -0.009099 0.001917 -0.008987 -0.002474
0.001259 0.016649 0.018928 0.006998 -0.017038 0.011075 -
0.001023 0.002158 0.002612 0.029846 -0.017653 -0.014145 -
0.018551 -0.009805 -0.014440 0.005263 0.001436 0.016250
0.007007 0.027234 0.002239 0.026792 0.004626 0.019760 -
0.006576 -0.014207 0.024989 0.027588 -0.025146 0.015887 -
0.006978 -0.002730
y448 -0.006588 -0.011346 0.009558 0.001974 0.013913 -
0.008152 -0.005887 0.010300 0.005438 0.012299 -0.012526 -

0.028410	0.007612	0.031699	0.026281	0.012787	0.017892	
0.012160	-0.009720	-0.005996	0.000361	-0.000207	0.012770	-
0.009452	-0.001152	0.000336	-0.000445	-0.021600	0.002518	
0.000928	0.002988	-0.019608	0.012707	-0.011561	-0.000106	-
0.002759	-0.007676					
z448	-0.014985	0.009195	-0.017307	-0.020432	0.004532	-
0.002704	-0.006998	-0.014841	-0.006970	0.049139	-0.031015	-
0.043801	-0.005663	0.060627	0.042182	0.015468	0.040154	
0.020066	-0.022357	0.008240	-0.000731	0.009635	0.000992	-
0.000791	0.009992	0.017209	-0.018310	0.003406	-0.004481	
0.005799	0.028889	-0.023613	0.001916	-0.020926	0.007995	-
0.026838	-0.008200					
x449	0.015741	-0.011157	-0.000870	-0.012706	-0.001098	
0.002050	0.015179	0.018899	0.005307	-0.011649	0.006769	-
0.006607	-0.001797	0.006069	0.032193	-0.015367	-0.008427	-
0.012648	-0.013936	-0.012227	0.007623	0.004228	0.013822	
0.001486	0.025859	0.006686	0.018573	0.000712	0.018539	-
0.005675	-0.009803	0.021243	0.026005	-0.029905	0.017263	-
0.012316	0.000818					
y449	-0.008020	-0.009184	0.010170	0.001087	0.011905	-
0.009132	-0.007091	0.009252	0.003410	0.013674	-0.010051	-
0.034177	0.007976	0.036013	0.026090	0.013438	0.020412	
0.009455	-0.012347	-0.003806	-0.001643	0.005581	0.015397	-
0.006675	-0.004978	-0.001657	0.000050	-0.014868	-0.003565	
0.003486	-0.002801	-0.019993	0.005174	-0.016347	0.000520	-
0.001774	-0.006144					
z449	-0.014018	0.011112	-0.017705	-0.019844	0.000083	-
0.003423	-0.009436	-0.015401	-0.008209	0.049770	-0.028942	-
0.037011	-0.005328	0.054483	0.032502	0.015118	0.036790	
0.025244	-0.020644	0.011699	-0.003892	0.011264	0.001561	-
0.002854	0.008293	0.014037	-0.019607	0.006819	-0.005646	
0.009240	0.024922	-0.027594	0.000108	-0.018666	0.007291	-
0.023690	-0.005815					
x450	0.012873	-0.011558	-0.002895	-0.016087	0.000512	
0.001779	0.014341	0.016631	0.002768	-0.007604	0.003344	-
0.013571	-0.004289	0.011972	0.034527	-0.012204	-0.002012	-
0.010014	-0.017986	-0.009207	0.008216	0.010878	0.009822	
0.000646	0.021370	0.008584	0.010073	0.002385	0.014685	-
0.003197	-0.008475	0.014169	0.020877	-0.038374	0.020560	-
0.013222	0.005643					
y450	-0.009280	-0.008101	0.009929	-0.000841	0.008919	-
0.008971	-0.009030	0.009495	0.000978	0.014564	-0.008758	-
0.037368	0.005775	0.038260	0.023423	0.012614	0.023482	
0.014524	-0.013750	0.001767	-0.002998	0.008883	0.018543	-
0.008544	-0.009492	0.001519	-0.001417	-0.013979	-0.008765	
0.004420	-0.008113	-0.019550	0.002923	-0.015994	-0.000620	-
0.004730	-0.004208					
z450	-0.012491	0.013473	-0.017500	-0.017789	-0.004619	-
0.004723	-0.011773	-0.016600	-0.010319	0.049200	-0.024362	-
0.028630	-0.003711	0.045822	0.020722	0.014749	0.031586	
0.026808	-0.018924	0.015880	-0.009594	0.014542	0.001095	-
0.003103	0.007895	0.010616	-0.021982	0.010934	-0.008076	
0.014138	0.019848	-0.031900	-0.003998	-0.016226	0.008809	-
0.018286	-0.003607					
x451	0.009851	-0.011840	-0.004449	-0.019549	0.002885	
0.000549	0.013967	0.013755	-0.001091	-0.004652	0.000401	-
0.020596	-0.006318	0.017008	0.033897	-0.009589	0.003855	-
0.005414	-0.021684	-0.005551	0.010902	0.017600	0.007878	-
0.001205	0.013754	0.010884	0.001062	0.004097	0.011827	-

0.000928	-0.007477	0.009101	0.013258	-0.044781	0.024160	-
0.017639	0.013012					
y451	-0.010551	-0.006217	0.009416	-0.001924	0.004174	-
0.008702	-0.010683	0.008720	-0.003020	0.015510	-0.006532	-
0.036161	0.004334	0.037897	0.018727	0.012322	0.025355	
0.017929	-0.013931	0.009840	-0.008306	0.013181	0.016149	-
0.007770	-0.011535	0.006480	-0.005271	-0.008716	-0.014381	
0.011424	-0.012890	-0.026795	-0.000105	-0.018105	-0.000099	
0.003910	0.000287					
z451	-0.010422	0.015576	-0.017216	-0.015049	-0.009444	-
0.006073	-0.014660	-0.017145	-0.012466	0.046663	-0.018401	-
0.020506	-0.002120	0.035388	0.006215	0.013193	0.025796	
0.029257	-0.017150	0.020348	-0.014347	0.016243	0.002469	-
0.004268	0.007380	0.007429	-0.023826	0.011580	-0.011481	
0.016408	0.012825	-0.030338	-0.006331	-0.011441	0.009557	-
0.015621	-0.002558					
x452	0.006318	-0.011424	-0.006119	-0.022612	0.004625	-
0.000887	0.014718	0.009853	-0.007669	-0.000779	-0.002782	-
0.024308	-0.005466	0.020220	0.029468	-0.009005	0.008900	
0.000445	-0.025114	0.000100	0.013580	0.024977	0.003042	-
0.001746	0.002549	0.016220	-0.008162	0.010280	0.008703	
0.005387	-0.010487	0.003300	0.001695	-0.049964	0.024422	-
0.015302	0.020540					
y452	-0.011722	-0.004930	0.009746	-0.004123	-0.000493	-
0.010393	-0.013815	0.007901	-0.005982	0.014079	-0.001953	-
0.037134	0.001729	0.038625	0.013883	0.013307	0.029080	
0.021577	-0.013202	0.017241	-0.013560	0.018586	0.015969	-
0.009370	-0.012662	0.007475	-0.009238	-0.007944	-0.017633	
0.015452	-0.018906	-0.033888	-0.000559	-0.019158	0.007065	
0.002068	0.010978					
z452	-0.008264	0.018130	-0.016972	-0.011661	-0.014868	-
0.006865	-0.017854	-0.017976	-0.014907	0.043195	-0.011919	-
0.010568	-0.001468	0.023703	-0.009224	0.012431	0.018900	
0.030030	-0.016313	0.026734	-0.022173	0.018970	0.002255	-
0.004025	0.007297	0.005130	-0.026092	0.012803	-0.016658	
0.018172	0.003538	-0.028993	-0.010433	-0.007566	0.009036	-
0.009741	-0.001066					
x453	0.003507	-0.007830	-0.006065	-0.028459	0.003831	-
0.003600	0.008016	0.007531	-0.007220	0.005742	-0.005122	-
0.026220	-0.007182	0.020495	0.023924	-0.001913	0.010959	
0.001709	-0.026887	0.000460	0.016634	0.027005	-0.001065	
0.001664	0.003379	0.008365	-0.011152	0.016456	0.003659	
0.009013	-0.011093	-0.005760	0.001084	-0.056029	0.024879	-
0.007660	0.013518					
y453	-0.013854	-0.002380	0.010067	-0.005312	-0.002847	-
0.012023	-0.015185	0.007277	-0.009139	0.012757	-0.003493	-
0.037347	0.002187	0.035181	0.007708	0.012499	0.023258	
0.022891	-0.009829	0.013630	-0.010799	0.020145	0.016152	-
0.004801	-0.009653	0.011412	-0.013833	0.001278	-0.020318	
0.019096	-0.015562	-0.029326	-0.009302	-0.015364	0.017549	
0.009178	0.009316					
z453	-0.007899	0.019665	-0.015783	-0.006030	-0.016685	-
0.010532	-0.017932	-0.016555	-0.016096	0.041616	-0.010762	-
0.002713	-0.000087	0.016069	-0.015991	0.010707	0.013424	
0.027018	-0.006090	0.024864	-0.022107	0.013261	-0.002013	-
0.002087	0.008320	0.003124	-0.025031	0.019560	-0.016562	
0.011400	0.005723	-0.017656	-0.006177	0.002425	0.003044	-
0.010622	-0.004729					
x454	0.001439	-0.004547	-0.006058	-0.031824	0.003093	-
0.006225	0.003298	0.005575	-0.007894	0.011472	-0.006165	-

0.026325	-0.007978	0.017866	0.017532	0.001866	0.011851	
0.004610	-0.027409	-0.000125	0.019086	0.029123	-0.003517	
0.004719	0.004928	0.004215	-0.017180	0.021664	-0.001646	
0.011978	-0.010789	-0.009809	0.002468	-0.057949	0.028157	-
0.002303	0.006149					
y454	-0.015166	-0.000589	0.009727	-0.007963	-0.005238	-
0.012764	-0.017869	0.006853	-0.010258	0.011688	-0.005570	-
0.039587	-0.000129	0.036577	0.007567	0.014769	0.021366	
0.020488	-0.007231	0.013246	-0.008675	0.020841	0.011123	-
0.001419	-0.007904	0.011039	-0.013784	0.007367	-0.017948	
0.021938	-0.014547	-0.030195	-0.014343	-0.015364	0.019992	
0.015717	0.008800					
z454	-0.007304	0.020746	-0.014865	0.000196	-0.017363	-
0.013153	-0.018044	-0.015816	-0.016269	0.039697	-0.008736	
0.002722	0.000914	0.009685	-0.021017	0.008830	0.008393	
0.025193	0.003343	0.022484	-0.022293	0.009574	-0.002745	
0.000593	0.008268	0.001685	-0.023615	0.023963	-0.021092	
0.004199	0.006968	-0.009816	-0.001943	0.008747	-0.000389	-
0.010878	-0.006461					
x455	-0.000203	-0.002499	-0.006063	-0.034477	0.001397	-
0.008077	-0.000013	0.003817	-0.008486	0.016312	-0.008418	-
0.024784	-0.009256	0.017480	0.013455	0.004833	0.013333	
0.008224	-0.026929	-0.000598	0.019836	0.028795	-0.007795	
0.007233	0.005032	-0.000818	-0.020564	0.024116	-0.003009	
0.015337	-0.010626	-0.012778	0.001871	-0.055767	0.027134	
0.001648	-0.002248					
y455	-0.015962	0.001489	0.008526	-0.008795	-0.005859	-
0.012841	-0.020907	0.006415	-0.010973	0.010803	-0.005082	-
0.042439	-0.000613	0.034195	0.005023	0.016431	0.016718	
0.017333	-0.005293	0.012736	-0.006548	0.022227	0.009230	
0.000786	-0.004978	0.012024	-0.015213	0.013441	-0.020519	
0.020416	-0.013199	-0.028412	-0.016177	-0.018710	0.023570	
0.022603	0.011168					
z455	-0.005018	0.020700	-0.013987	0.006201	-0.018018	-
0.015151	-0.017748	-0.014891	-0.017420	0.036705	-0.007253	
0.008368	0.001870	0.003893	-0.023594	0.006681	0.004734	
0.023759	0.011818	0.022243	-0.023088	0.004954	-0.004702	
0.001726	0.009355	-0.000082	-0.019839	0.025175	-0.020163	-
0.000286	0.008393	-0.004699	0.001147	0.017649	-0.004382	-
0.011079	-0.007890					
x456	-0.001360	-0.000828	-0.006736	-0.035091	0.000387	-
0.009765	-0.002290	0.001919	-0.008258	0.019168	-0.010985	-
0.021476	-0.009085	0.017065	0.007907	0.007416	0.015473	
0.009529	-0.027315	-0.000220	0.019232	0.028022	-0.010355	
0.008101	0.007702	-0.003153	-0.024579	0.027095	-0.005019	
0.014104	-0.007232	-0.012985	-0.000691	-0.051545	0.028691	
0.000695	-0.007256					
y456	-0.016196	0.002640	0.007646	-0.009521	-0.007205	-
0.010872	-0.024067	0.006284	-0.011788	0.011374	-0.004470	-
0.046077	-0.002899	0.031044	0.004849	0.017378	0.011128	
0.016611	-0.002474	0.012303	-0.005119	0.022937	0.005770	
0.002577	-0.006617	0.012150	-0.015879	0.016072	-0.021643	
0.020355	-0.016866	-0.028020	-0.015130	-0.022163	0.022118	
0.031717	0.012122					
z456	-0.001364	0.020111	-0.013614	0.012595	-0.017287	-
0.016551	-0.017649	-0.013750	-0.018738	0.032093	-0.005944	
0.013396	0.005024	-0.001190	-0.025239	0.004672	0.000460	
0.020366	0.018968	0.024279	-0.025282	0.000492	-0.005035	-
0.000234	0.013287	-0.001040	-0.013448	0.023854	-0.019421	-

0.004229	0.011053	-0.001715	0.001549	0.025919	-0.007453	-	
0.010266	-0.008424						
x457	-0.002795	0.000948	-0.008077	-0.035447	-0.001026	-	
0.008790	-0.004236	0.001018	-0.006934	0.021238	-0.012613	-	
0.020236	-0.009371	0.018391	0.003888	0.009076	0.014956		
0.010430	-0.024347	-0.001087	0.018988	0.027739	-0.011020		
0.009994	0.006889	-0.006151	-0.027499	0.028713	-0.004686		
0.013917	-0.006445	-0.008215	-0.001371	-0.049875	0.024395		
0.003441	-0.013891						
y457	-0.015273	0.002565	0.006615	-0.008459	-0.007562	-	
0.010047	-0.026374	0.005149	-0.013680	0.010797	-0.004193	-	
0.045474	-0.002629	0.026034	0.003507	0.018060	0.008039		
0.015847	-0.001858	0.014034	-0.005959	0.021660	0.001923		
0.001320	-0.003404	0.012238	-0.015490	0.017141	-0.023536		
0.017298	-0.016630	-0.027657	-0.014812	-0.020121	0.023584		
0.036010	0.015410						
z457	0.002956	0.019122	-0.014176	0.019147	-0.016177	-	
0.016286	-0.018180	-0.012458	-0.019738	0.027406	-0.003271		
0.014530	0.006735	-0.005826	-0.024256	0.002288	-0.004355		
0.017688	0.026738	0.026634	-0.026365	-0.001941	-0.003272	-	
0.001815	0.014728	-0.002272	-0.006257	0.019080	-0.017308	-	
0.006401	0.009015	0.000729	0.006035	0.027752	-0.009890	-	
0.007926	-0.007842						
x458	-0.003507	0.001599	-0.010269	-0.032008	-0.003200	-	
0.008287	-0.007559	0.000622	-0.006481	0.022092	-0.015455	-	
0.018019	-0.011447	0.017828	0.001090	0.011777	0.013215		
0.011407	-0.020629	-0.001887	0.016381	0.025495	-0.010517		
0.010076	0.006671	-0.006513	-0.030272	0.028191	-0.003990		
0.010071	-0.005706	-0.000274	-0.002443	-0.047776	0.020612		
0.003613	-0.016524						
y458	-0.014353	0.002451	0.005348	-0.009014	-0.006467	-	
0.007442	-0.026017	0.003745	-0.013580	0.009828	-0.002805	-	
0.044369	-0.000204	0.023631	0.001925	0.017162	0.005986		
0.014413	-0.001676	0.015479	-0.007220	0.021256	-0.002202		
0.000628	-0.000360	0.009612	-0.014231	0.017001	-0.024344		
0.014852	-0.015478	-0.028173	-0.012627	-0.016542	0.021907		
0.039611	0.014655						
z458	0.009186	0.017086	-0.014976	0.027070	-0.014554	-	
0.015703	-0.019724	-0.010995	-0.021593	0.021957	0.000344		
0.014016	0.007943	-0.012424	-0.020947	0.000179	-0.009548		
0.015877	0.034392	0.031074	-0.027884	-0.005178	-0.001175	-	
0.005212	0.016426	-0.004116	0.004267	0.010231	-0.016596	-	
0.008232	0.006007	-0.000209	0.010975	0.025882	-0.011322	-	
0.004050	-0.002333						
x459	-0.003040	0.001556	-0.012347	-0.026294	-0.002784	-	
0.005982	-0.009747	0.000591	-0.008062	0.020772	-0.015593	-	
0.014797	-0.012097	0.020895	-0.001644	0.010909	0.012555		
0.010359	-0.014665	0.001044	0.011367	0.019913	-0.010501		
0.009303	0.004310	-0.008398	-0.031946	0.023907	-0.000401		
0.004700	-0.007641	0.004781	0.000122	-0.044226	0.012845		
0.006396	-0.017866						
y459	-0.013405	0.002098	0.003422	-0.010073	-0.007523	-	
0.005146	-0.025467	0.001845	-0.010825	0.010129	-0.003632	-	
0.043460	0.001750	0.017727	0.000665	0.018761	0.002845		
0.013869	-0.002615	0.013832	-0.007425	0.023116	-0.006927	-	
0.001109	0.003508	0.008436	-0.010341	0.018940	-0.024890		
0.012229	-0.009331	-0.024298	-0.014467	-0.012563	0.021527		
0.039576	0.012241						
z459	0.017288	0.014465	-0.016272	0.035417	-0.011095	-	
0.014180	-0.021372	-0.009692	-0.024525	0.016208	0.005917		

0.011961	0.008752	-0.018538	-0.014909	-0.002430	-0.012933	
0.012804	0.040884	0.038791	-0.030058	-0.007799	0.000918	-
0.009199	0.018639	-0.005446	0.015666	-0.002841	-0.015562	-
0.010444	0.001494	-0.005216	0.018103	0.020671	-0.011456	
0.000088	0.005933					
x460	-0.000457	0.002752	-0.015794	-0.021144	-0.003429	-
0.003377	-0.010820	0.001777	-0.009690	0.021592	-0.012636	-
0.010752	-0.013084	0.020054	-0.002965	0.006289	0.013191	
0.012474	-0.006861	0.008211	0.004373	0.014723	-0.008599	
0.006688	0.002799	-0.009053	-0.031383	0.019319	0.006691	-
0.003185	-0.003601	0.007407	0.002262	-0.041932	0.006564	
0.006997	-0.011334					
y460	-0.013369	-0.000191	0.002520	-0.009642	-0.007011	-
0.002028	-0.024116	-0.000140	-0.007236	0.008027	-0.006611	-
0.041021	0.005199	0.016101	-0.000899	0.021791	-0.001006	
0.010359	-0.005856	0.009747	-0.008489	0.022035	-0.014361	-
0.001545	0.007245	0.005048	-0.009023	0.018391	-0.025975	
0.008941	-0.010085	-0.019038	-0.012186	-0.004934	0.016923	
0.039702	0.002369					
z460	0.027344	0.010976	-0.017699	0.043791	-0.007263	-
0.012244	-0.022948	-0.007900	-0.027509	0.010843	0.012167	
0.008103	0.009918	-0.027292	-0.006241	-0.004726	-0.017193	
0.010735	0.045326	0.048767	-0.032343	-0.009657	0.001699	-
0.014106	0.021498	-0.008000	0.029097	-0.018609	-0.016060	-
0.012745	-0.002993	-0.012837	0.024160	0.014007	-0.010755	
0.005991	0.018089					
x461	0.004933	0.001873	-0.018241	-0.012903	-0.002682	
0.000080	-0.011246	0.002702	-0.011442	0.019721	-0.010077	-
0.008331	-0.011070	0.016457	-0.000797	0.002272	0.013311	
0.012732	0.004401	0.016381	0.003851	0.012749	-0.008531	
0.004385	0.003145	-0.015482	-0.025261	0.011264	0.001210	-
0.007947	-0.001466	0.015306	0.005634	-0.036612	-0.001448	
0.010982	-0.006569					
y461	-0.015091	-0.001951	0.001032	-0.010365	-0.007350	
0.001547	-0.021766	-0.002825	-0.003216	0.009082	-0.009627	-
0.036309	0.007212	0.015718	-0.004722	0.023119	-0.003607	
0.006713	-0.012202	0.004372	-0.012313	0.017665	-0.022897	-
0.002867	0.007125	0.005530	-0.010312	0.020304	-0.016581	
0.002337	-0.008543	-0.013918	-0.011661	0.001053	0.007339	
0.033355	-0.002153					
z461	0.037950	0.006445	-0.019104	0.052748	-0.002585	-
0.009974	-0.023728	-0.005607	-0.031325	0.004344	0.018326	
0.002914	0.011553	-0.036400	0.003851	-0.006554	-0.021689	
0.008179	0.046748	0.058085	-0.034555	-0.011918	0.002661	-
0.019915	0.024137	-0.007830	0.040670	-0.037182	-0.013883	-
0.014522	-0.011381	-0.021246	0.031461	0.007086	-0.007834	
0.009890	0.030501					
x462	0.020398	-0.005293	-0.016682	0.002432	-0.003628	-
0.003422	-0.017213	0.004718	-0.016218	0.013313	-0.003693	-
0.012865	-0.009660	0.005481	0.000989	-0.005433	0.003655	
0.006937	0.022857	0.022322	-0.003220	0.006410	-0.002802	-
0.003157	0.009000	-0.008119	-0.016699	-0.002902	0.007029	-
0.004914	-0.003846	-0.000236	0.012870	-0.023761	-0.007600	
0.014661	0.003926					
y462	-0.006829	-0.004712	-0.002392	0.000112	-0.002102	
0.004246	-0.025969	-0.004325	-0.002876	0.004832	-0.002355	-
0.037675	-0.000461	0.005363	0.006995	0.018613	-0.015886	-
0.008442	-0.005370	0.006565	-0.002424	0.028697	-0.023820	-
0.000474	0.012583	0.015879	-0.006831	0.012340	-0.020788	-

0.012674 -0.008104 -0.017014 -0.005161 0.006099 0.006206
0.030249 0.003875
z462 0.035248 -0.008490 -0.009155 0.050306 -0.000454 -
0.010809 -0.010803 -0.011146 -0.025104 -0.007668 0.005884
0.002245 0.011604 -0.035730 -0.000892 -0.005654 -0.016921
0.009457 0.031923 0.023946 -0.018672 -0.023058 -0.001046 -
0.016970 0.029623 -0.010450 0.034973 -0.038938 -0.013123
0.003342 -0.023176 -0.022384 0.025996 0.014543 -0.022226
0.000947 0.018488
x463 0.031079 -0.010711 -0.015499 0.020443 -0.002480 -
0.002667 -0.023135 0.005648 -0.020514 0.004949 -0.000847 -
0.023081 -0.014346 -0.005740 -0.001419 -0.008318 -0.005903
0.000394 0.028514 0.022223 -0.003489 -0.003256 0.003871 -
0.008393 0.013657 -0.003551 -0.015544 -0.007643 0.028731 -
0.004103 -0.001344 -0.013712 0.025549 -0.011614 -0.015300
0.018308 0.011306
y463 -0.001197 -0.009363 -0.003677 0.003400 0.000665
0.003245 -0.021269 -0.005047 -0.000945 0.003919 0.001008 -
0.029137 0.002231 -0.001396 0.016315 0.011091 -0.023113 -
0.014095 0.000094 0.005341 0.002692 0.039088 -0.033064
0.004169 0.009324 0.021671 0.004441 -0.001674 -0.040400 -
0.017701 -0.013203 -0.016288 -0.009763 0.003114 0.004669
0.031297 0.001997
z463 0.030591 -0.018305 -0.000286 0.041950 0.001100 -
0.011275 0.002850 -0.016059 -0.018398 -0.015749 -0.004464
0.004208 0.016045 -0.030927 -0.003729 -0.005213 -0.011906
0.012534 0.019410 -0.001366 -0.010026 -0.029723 -0.006361 -
0.014213 0.028252 -0.015210 0.032325 -0.042074 -0.015433
0.019364 -0.035650 -0.018656 0.015736 0.016239 -0.031795 -
0.001862 0.007140
x464 0.038356 -0.016712 -0.016136 0.034386 0.001115 -
0.000164 -0.022972 0.005691 -0.022792 -0.005113 0.000045 -
0.026220 -0.019545 -0.012530 -0.003177 -0.010278 -0.013456
0.004899 0.030030 0.022704 -0.000973 -0.002819 0.004010 -
0.015977 0.010068 0.001076 -0.003338 -0.022540 0.040084 -
0.005857 0.005907 -0.025849 0.036059 -0.001247 -0.019693
0.024614 0.019876
y464 0.002509 -0.013492 -0.000461 0.007681 0.001975
0.002472 -0.018300 -0.002301 -0.000429 0.003417 0.004669 -
0.019205 0.008492 -0.006943 0.018563 0.006616 -0.025914 -
0.023650 -0.001471 0.001244 0.002390 0.039173 -0.035640
0.016613 0.010169 0.021314 0.003490 -0.005787 -0.054254 -
0.017270 -0.023017 -0.015977 -0.014011 -0.000015 -0.005243
0.026171 0.001964
z464 0.023614 -0.022370 0.006569 0.033019 0.002477 -
0.011109 0.011647 -0.020351 -0.014670 -0.020474 -0.009715
0.007246 0.019352 -0.025279 -0.008018 -0.001805 -0.008068
0.014166 0.009363 -0.019292 -0.006494 -0.034567 -0.007781 -
0.009573 0.024798 -0.019274 0.028981 -0.042561 -0.012768
0.034809 -0.040600 -0.016208 0.007850 0.012518 -0.039372 -
0.004421 0.001732
x465 0.041390 -0.023281 -0.014881 0.045025 0.005993
0.004122 -0.019766 0.006895 -0.024652 -0.015483 0.000958 -
0.018683 -0.023957 -0.017052 -0.005712 -0.010588 -0.017667
0.011677 0.025511 0.019592 0.001106 0.003182 0.001589 -
0.021613 0.001142 0.001294 0.010597 -0.039474 0.045937 -
0.005161 0.014563 -0.036019 0.042485 0.003876 -0.027660
0.029169 0.032734
y465 0.006938 -0.017284 0.002603 0.012183 0.001922
0.002198 -0.014897 0.001549 0.004053 0.003332 0.007841 -

0.014099	0.013321	-0.012731	0.018610	0.003233	-0.026340	-
0.030002	-0.005466	-0.001653	-0.002604	0.036225	-0.038743	
0.027771	0.011154	0.017709	0.009020	-0.002788	-0.059622	-
0.019586	-0.027503	-0.006465	-0.017042	0.000067	-0.001893	
0.012651	-0.003481					
z465	0.015162	-0.020543	0.007311	0.024491	0.003554	-
0.010293	0.020395	-0.024296	-0.012114	-0.021236	-0.012519	
0.009026	0.020410	-0.018322	-0.013596	-0.001081	-0.004733	
0.020942	0.000823	-0.028894	-0.004502	-0.043143	-0.016004	-
0.002233	0.018664	-0.015449	0.027196	-0.039948	-0.012681	
0.042889	-0.035884	-0.017007	0.011233	0.006255	-0.047348	
0.006289	-0.010834					
x466	0.043412	-0.027312	-0.010778	0.054860	0.009529	
0.009820	-0.012061	0.013341	-0.023271	-0.025371	0.002278	-
0.008028	-0.025353	-0.023866	-0.011300	-0.012936	-0.020896	
0.012363	0.009786	0.013927	0.001107	0.000039	-0.008463	-
0.017133	-0.004407	-0.004110	0.017875	-0.038717	0.044249	-
0.003343	0.026597	-0.029447	0.050903	0.001623	-0.040793	
0.021427	0.039561					
y466	0.009845	-0.021777	0.006491	0.018090	0.004638	
0.006305	-0.011877	0.002261	0.007970	-0.003607	0.010819	-
0.014828	0.020193	-0.006870	0.013360	-0.000962	-0.032597	-
0.021877	0.000240	0.000593	0.001983	0.023645	-0.040600	
0.028837	0.002127	-0.003832	0.044421	-0.012720	-0.065995	-
0.009800	-0.023617	-0.025902	-0.013967	0.002352	-0.037071	
0.060876	-0.013631					
z466	0.003185	-0.018205	0.018212	0.005357	0.004467	-
0.008405	0.027162	-0.028757	-0.005348	-0.025780	-0.014253	
0.008353	0.035089	-0.003198	-0.011499	-0.000538	-0.014203	
0.028130	-0.001411	-0.037265	-0.007545	-0.045625	-0.018943	-
0.011098	0.003728	-0.058492	0.058036	-0.043199	-0.005897	
0.055509	-0.042890	-0.014655	-0.001414	0.007281	-0.049375	
0.015570	-0.015631					
x467	0.040407	-0.032922	-0.006670	0.062115	0.012549	
0.016694	0.000213	0.019287	-0.022673	-0.035490	0.003951	
0.008197	-0.025005	-0.028790	-0.017190	-0.013952	-0.019014	
0.019401	-0.014876	0.005858	0.001548	-0.002909	-0.024092	-
0.017227	-0.018530	-0.019253	0.034487	-0.036694	0.032917	-
0.005424	0.034655	-0.023855	0.064079	-0.003446	-0.047915	
0.025563	0.043369					
y467	0.014710	-0.027783	0.003647	0.028149	0.008593	
0.006795	-0.007008	0.004486	0.006828	-0.000631	0.006537	-
0.004600	0.015674	-0.017500	0.005625	-0.002558	-0.011966	-
0.019463	-0.015629	-0.014025	0.000052	0.028813	-0.039344	
0.039052	0.001026	0.020583	0.019830	-0.012965	-0.050989	-
0.016947	-0.024001	-0.023267	-0.000250	-0.003584	-0.018958	
0.017859	-0.016069					
z467	-0.012469	-0.007097	0.013129	-0.010658	0.008008	-
0.008613	0.035406	-0.035027	-0.009132	-0.018121	-0.014348	
0.019094	0.035018	-0.000462	-0.011750	0.001392	-0.000733	
0.033036	-0.020798	-0.047703	-0.010495	-0.037811	-0.029454	-
0.012011	-0.003998	-0.048036	0.039158	-0.033957	0.006761	
0.042114	-0.035851	-0.003177	0.011721	-0.011016	-0.020739	-
0.020697	-0.006280					
x468	0.033988	-0.047957	0.007531	0.058800	0.015808	
0.023518	0.018542	0.025270	-0.013541	-0.050558	-0.000680	
0.027690	-0.014425	-0.033604	-0.012493	-0.013026	-0.021606	
0.021681	-0.052132	-0.019985	-0.015028	0.013178	-0.025500	-
0.043702	-0.042439	-0.065614	0.062812	-0.034022	0.036020	-

0.013793	0.013520	0.001551	0.077539	-0.001007	-0.000640	-
0.044512	0.062251					
y468	0.010145	-0.034592	0.012994	0.030223	0.011043	
0.008212	0.004370	-0.000567	0.010019	-0.009176	0.004624	-
0.000838	0.024136	-0.014020	-0.012830	0.000225	-0.005913	-
0.007921	-0.025680	-0.036762	-0.002624	0.011463	-0.030775	
0.028124	-0.002622	0.002000	0.038373	-0.006238	-0.009914	-
0.010462	-0.011862	-0.033950	0.008532	0.007746	0.000341	-
0.005978	-0.022173					
z468	-0.031755	0.020589	-0.001943	-0.031567	0.010538	-
0.004751	0.043904	-0.040256	-0.019647	-0.009649	-0.006168	
0.028968	0.033867	0.000780	-0.013924	0.012252	0.002508	
0.046915	-0.025977	-0.021685	-0.002725	-0.054692	-0.063686	-
0.009098	-0.007109	-0.023656	0.041388	-0.022327	-0.061728	
0.036258	0.005595	0.000298	0.034298	-0.057383	-0.069034	
0.080550	-0.000700					
x469	0.015648	-0.053518	0.020930	0.070205	0.028770	
0.052451	0.041930	0.070588	-0.000325	-0.062779	-0.000665	
0.096003	-0.008846	-0.037220	-0.020084	-0.008806	-0.013506	
0.046276	-0.155364	-0.056201	0.001761	0.032212	-0.064694	-
0.028772	-0.114945	-0.091237	0.097722	-0.028958	0.003623	-
0.026682	0.047227	0.009491	0.126441	-0.020929	-0.005904	-
0.034160	0.089601					
y469	0.000689	-0.038096	0.010588	0.016693	0.005136	-
0.011006	0.016715	-0.020288	0.007530	-0.007711	0.002940	-
0.009434	0.029772	-0.011381	-0.060225	0.027834	0.016376	
0.014797	-0.005804	-0.013079	-0.023452	-0.006792	-0.018160	
0.046109	0.008886	-0.012131	0.034589	0.009998	-0.032197	-
0.009388	0.013700	-0.020910	-0.047209	0.005445	-0.005615	-
0.004102	-0.022757					
z469	-0.068213	0.058674	0.009550	-0.086237	-0.017757	-
0.018352	0.049145	-0.095976	-0.035977	0.005306	0.030757	
0.026677	0.027800	0.039656	0.014005	0.031318	-0.031753	
0.060514	-0.053928	0.007261	-0.006564	-0.052679	-0.034818	-
0.094162	0.002196	0.008372	0.033553	-0.015468	-0.035154	
0.055206	-0.016458	0.014468	0.013615	-0.037683	-0.038157	
0.063070	0.000225					
x470	-0.002782	0.019319	-0.012560	0.012569	0.009049	-
0.015957	0.015586	-0.013070	-0.003746	0.001042	-0.021029	
0.008798	-0.003399	0.028313	0.031053	-0.023852	0.004249	-
0.001304	0.020530	-0.039701	-0.001387	-0.039512	0.010324	
0.032869	-0.019646	0.012005	0.003055	-0.044865	-0.017154	
0.006252	0.005078	0.036300	-0.038290	-0.021677	-0.022200	
0.009486	0.026589					
y470	0.004607	-0.012738	-0.005421	0.006989	0.011186	
0.006960	0.008270	0.014733	0.012933	-0.006136	0.000005	-
0.015901	-0.024218	0.005055	-0.007447	0.028409	0.000409	-
0.029037	0.007335	0.034817	-0.024621	0.023014	-0.028837	-
0.004220	-0.010315	-0.002542	0.000582	-0.021865	0.031019	
0.024977	0.003012	-0.024003	-0.009053	-0.043220	0.038718	-
0.025293	0.007763					
z470	0.008957	0.006409	0.014787	-0.002374	-0.021443	-
0.006746	-0.003469	-0.002377	0.003477	0.005483	0.023056	-
0.035608	-0.000858	-0.007491	0.045732	0.011952	0.022263	-
0.027040	0.018663	-0.015251	-0.028530	-0.013620	-0.008318	
0.019242	-0.032120	0.029692	-0.028793	-0.007192	0.006748	-
0.031580	0.007718	-0.017656	-0.008795	-0.001741	-0.045561	-
0.022637	0.000816					
x471	-0.006853	0.020608	-0.011983	0.009526	0.007152	-
0.007065	0.014874	-0.011650	0.002167	-0.004683	-0.034651	

0.012931	-0.006282	0.029314	0.016229	-0.029600	0.004991	
0.013989	0.021234	-0.047096	0.012087	-0.041002	0.001660	
0.025229	-0.004805	0.024888	0.008192	-0.028160	-0.021709	-
0.000451	0.003592	0.039364	-0.026487	-0.010678	-0.004866	
0.009924	0.012043					
y471	0.009098	-0.012588	-0.004012	0.006798	0.005001	
0.005641	0.007912	0.013398	0.003181	-0.007183	0.008307	-
0.009129	-0.022702	-0.004048	0.008358	0.024532	0.005904	-
0.035260	0.011139	0.035146	-0.029618	0.012204	-0.029800	-
0.000378	-0.019393	-0.008656	0.007547	-0.027078	0.025701	
0.026430	0.008439	-0.029707	-0.009967	-0.027544	0.024974	-
0.027882	0.011638					
z471	0.007209	0.009243	0.013886	-0.004501	-0.019054	-
0.006743	-0.000081	0.002269	0.006137	0.003010	0.024090	-
0.031695	-0.006632	-0.004030	0.044664	0.014802	0.027185	-
0.021901	0.022759	-0.017871	-0.027133	-0.007838	-0.009121	
0.020936	-0.033337	0.031140	-0.028750	-0.001339	0.002454	-
0.017062	0.011477	-0.008647	-0.015311	0.005049	-0.036069	-
0.015634	0.008008					
x472	-0.011600	0.022959	-0.011171	0.007671	0.007476	-
0.001634	0.014187	-0.011515	0.010817	-0.006509	-0.044728	
0.017622	-0.006994	0.028253	0.001182	-0.030075	0.001996	
0.022912	0.020408	-0.051063	0.025594	-0.043861	-0.006552	
0.020882	0.009691	0.032659	0.010458	-0.015722	-0.021282	-
0.003361	0.002675	0.038017	-0.019106	-0.007195	0.008332	
0.013237	0.000765					
y472	0.011690	-0.013629	-0.005879	0.006422	0.002710	
0.001303	0.006754	0.012578	-0.000217	-0.005257	0.018922	-
0.002051	-0.017435	-0.003799	0.015496	0.021930	0.000686	-
0.044592	0.013226	0.031731	-0.028499	0.004439	-0.032227	
0.002956	-0.024465	-0.011506	0.012439	-0.031309	0.020862	
0.025663	0.015133	-0.030677	-0.016108	-0.019107	0.012041	-
0.024214	0.009900					
z472	0.006172	0.010419	0.012927	-0.007102	-0.015687	-
0.006248	0.002425	0.008584	0.009368	0.004101	0.023013	-
0.024506	-0.009957	-0.001076	0.041034	0.015779	0.023942	-
0.017277	0.023338	-0.022757	-0.027519	0.000815	-0.009680	
0.014125	-0.032548	0.032574	-0.023324	0.005557	-0.001148	
0.000145	0.014249	0.003110	-0.017567	0.013442	-0.024089	-
0.007313	0.011835					
x473	-0.014892	0.025633	-0.009354	0.004303	0.008295	
0.005420	0.012983	-0.011303	0.018569	-0.004145	-0.050658	
0.021088	-0.003819	0.027069	-0.011835	-0.030870	0.002162	
0.029211	0.022297	-0.049095	0.034064	-0.044554	-0.010785	
0.018552	0.018277	0.036817	0.008875	-0.003970	-0.019960	-
0.008246	0.002040	0.039817	-0.009327	-0.005273	0.017695	
0.009946	-0.010341					
y473	0.014234	-0.012185	-0.005740	0.004585	0.000549	
0.001261	0.007196	0.011040	-0.003289	-0.002654	0.029609	
0.003649	-0.013120	-0.004454	0.019976	0.018537	0.004072	-
0.050331	0.017596	0.032409	-0.027357	-0.002632	-0.031238	
0.006169	-0.032261	-0.013519	0.012194	-0.032180	0.017818	
0.024366	0.019622	-0.028442	-0.019069	-0.014238	-0.000112	-
0.021692	0.008374					
z473	0.004026	0.012010	0.012688	-0.007333	-0.013208	-
0.006242	0.003424	0.013444	0.012500	0.001238	0.015177	-
0.020416	-0.015693	-0.002000	0.039425	0.018771	0.018018	-
0.011842	0.021002	-0.029123	-0.026794	0.006068	-0.012605	
0.006202	-0.029936	0.033881	-0.015548	0.009640	-0.001698	

0.012849	0.013429	0.010775	-0.021983	0.021881	-0.013507	
0.000654	0.014619					
x474	-0.016999	0.027155	-0.009441	0.003629	0.009605	
0.010325	0.009956	-0.010388	0.026466	-0.004083	-0.058555	
0.020840	-0.002880	0.024181	-0.018779	-0.026689	-0.001895	
0.033161	0.022431	-0.046688	0.039196	-0.044268	-0.012926	
0.020445	0.028752	0.037349	0.006021	0.002836	-0.015854	-
0.014887	-0.006237	0.038683	-0.004177	-0.000412	0.023545	
0.008110	-0.012201					
y474	0.016491	-0.010971	-0.007329	0.005933	-0.001395	
0.000590	0.006928	0.011097	-0.004611	-0.003660	0.035328	
0.005292	-0.014066	-0.004301	0.024219	0.018602	0.003770	-
0.053048	0.019483	0.030693	-0.026964	-0.007140	-0.028349	
0.010332	-0.036698	-0.016564	0.008273	-0.035545	0.017984	
0.022823	0.017717	-0.027885	-0.024667	-0.009275	-0.012160	-
0.017416	0.006903					
z474	0.002014	0.014625	0.013931	-0.009536	-0.009721	-
0.004637	0.003937	0.017703	0.016828	0.000485	0.009629	-
0.016430	-0.018289	-0.002782	0.036666	0.019727	0.015489	-
0.007978	0.020914	-0.031659	-0.025199	0.008744	-0.014537	-
0.002504	-0.029899	0.035960	-0.009696	0.014915	-0.004190	
0.021730	0.013471	0.019641	-0.025878	0.026561	-0.003264	
0.005745	0.018510					
x475	-0.020062	0.028505	-0.008387	0.002943	0.010211	
0.014818	0.006506	-0.010966	0.032455	-0.001399	-0.065511	
0.020578	-0.000467	0.020648	-0.022561	-0.020525	-0.006573	
0.034295	0.023872	-0.040258	0.043081	-0.044097	-0.016963	
0.021782	0.038273	0.036012	0.005518	0.008329	-0.014892	-
0.019739	-0.008254	0.039530	0.000978	0.003776	0.030695	-
0.000642	-0.015218					
y475	0.016651	-0.009051	-0.008204	0.007314	-0.002789	
0.000528	0.008221	0.010911	-0.004751	-0.004972	0.038140	
0.006853	-0.016485	-0.003591	0.024785	0.019016	0.005471	-
0.054714	0.022454	0.029627	-0.024040	-0.011000	-0.027943	
0.011713	-0.039674	-0.016856	0.005755	-0.037283	0.016634	
0.022382	0.017014	-0.022480	-0.030304	-0.007676	-0.018078	-
0.012502	0.003352					
z475	0.000500	0.017682	0.014758	-0.011299	-0.005886	-
0.002246	0.002565	0.021806	0.021577	-0.000934	0.002102	-
0.016078	-0.020227	-0.003715	0.033251	0.021234	0.011591	-
0.004775	0.019901	-0.033409	-0.022879	0.011706	-0.014319	-
0.010636	-0.029855	0.036132	-0.005647	0.019779	-0.001975	
0.026656	0.009825	0.024156	-0.030349	0.029139	0.006038	
0.009189	0.021712					
x476	-0.021871	0.030237	-0.006240	0.001845	0.011402	
0.018086	0.002632	-0.012297	0.038889	0.002327	-0.068999	
0.022224	0.003779	0.016108	-0.021973	-0.014322	-0.009880	
0.033226	0.024102	-0.030947	0.045458	-0.045793	-0.019433	
0.022433	0.047124	0.033743	0.004643	0.012643	-0.015463	-
0.026947	-0.012784	0.036325	0.005349	0.010184	0.033579	-
0.008000	-0.008127					
y476	0.017002	-0.006214	-0.007797	0.008596	-0.003504	
0.000701	0.009969	0.011664	-0.002898	-0.006829	0.041652	
0.011222	-0.019289	-0.001641	0.022334	0.015610	0.011286	-
0.053508	0.025342	0.028503	-0.021184	-0.015783	-0.028261	
0.009742	-0.041028	-0.015653	0.001456	-0.038158	0.011403	
0.019167	0.013897	-0.022877	-0.036083	-0.007237	-0.023900	-
0.005778	0.002056					
z476	-0.001507	0.020794	0.015467	-0.012374	-0.002006	-
0.000034	-0.000523	0.025704	0.027652	-0.003010	-0.008045	-

0.018816   -0.022758 -0.005286   0.030425   0.024334   0.005694 -
0.003143   0.019061 -0.034545 -0.020644   0.014351 -0.013338 -
0.018670   -0.029259   0.035462 -0.001789   0.023612   0.003654
0.029118   0.004397   0.028835 -0.035988   0.030602   0.015414
0.010208   0.024596
x477   -0.024104   0.032228 -0.003851   0.001081   0.012192
0.021328   -0.002502 -0.013356   0.044189   0.007776 -0.074468
0.020986   0.009426   0.011606 -0.018457 -0.005357 -0.017228
0.031392   0.022614 -0.019915   0.045809 -0.045014 -0.021379
0.025161   0.053650   0.029780   0.005757   0.015770 -0.011740 -
0.031775   -0.013531   0.031802   0.011607   0.018113   0.033997 -
0.022483   -0.007815
y477   0.015672 -0.002508 -0.007558   0.010082 -0.003233
0.002561   0.012298   0.013908 -0.000261 -0.009722   0.038120
0.011776   -0.022586   0.003226   0.016286   0.013754   0.014575 -
0.047396   0.024515   0.025029 -0.016693 -0.016343 -0.027845
0.007695   -0.043070 -0.010962 -0.000823 -0.035765   0.013639
0.019576   0.011525 -0.022680 -0.037572 -0.006384 -0.028040 -
0.001819   -0.010128
z477   -0.003216   0.023948   0.016973 -0.013706   0.001852
0.002283   -0.004804   0.029133   0.035482 -0.004598 -0.018153 -
0.021216   -0.024622 -0.008779   0.028432   0.027987   0.000746 -
0.003959   0.020049 -0.032446 -0.018384   0.014872 -0.013145 -
0.027450   -0.028096   0.034999   0.001381   0.025930   0.003848
0.028635   -0.000565   0.036629 -0.043710   0.027647   0.024602
0.010044   0.031001
x478   -0.025104   0.033631 -0.001224   0.000508   0.013963
0.023896   -0.009292 -0.015047   0.049983   0.014620 -0.078818
0.019036   0.015379   0.004033 -0.007934   0.007116 -0.025997
0.025685   0.021797 -0.006741   0.042068 -0.042027 -0.023024
0.026524   0.061043   0.021061   0.007915   0.016519 -0.011383 -
0.037691   -0.018331   0.031018   0.014175   0.025868   0.033036 -
0.038655   0.002534
y478   0.014492   0.001175 -0.007439   0.012246 -0.001809
0.005248   0.013209   0.016241   0.004214 -0.012344   0.032660
0.012088   -0.028648   0.006640   0.013062   0.014259   0.017707 -
0.040005   0.024513   0.019935 -0.015579 -0.013135 -0.027385
0.001295   -0.042387 -0.007977 -0.004116 -0.033048   0.009938
0.019345   0.006159 -0.014440 -0.041348 -0.007205 -0.032546
0.002890   -0.018306
z478   -0.005370   0.028003   0.018307 -0.014511   0.005996
0.005409   -0.010224   0.032017   0.043900 -0.006533 -0.030069 -
0.026077   -0.025492 -0.011816   0.024505   0.030102 -0.005051 -
0.004591   0.020878 -0.029941 -0.014959   0.012219 -0.012131 -
0.036334   -0.029184   0.035584   0.002969   0.029878   0.010853
0.026019   -0.006579   0.037643 -0.049740   0.023346   0.033670
0.010139   0.034995
x479   -0.025384   0.035415   0.001178 -0.000454   0.015940
0.025874   -0.017660 -0.017867   0.056945   0.022694 -0.082895
0.014970   0.021910 -0.005121   0.006538   0.021529 -0.036153
0.016342   0.021986   0.008607   0.037633 -0.040102 -0.025663
0.030433   0.065930   0.012644   0.012561   0.014202 -0.009401 -
0.044113   -0.026537   0.028379   0.015288   0.031717   0.029831 -
0.052037   0.015650
y479   0.012997   0.005732 -0.007663   0.013598 -0.000369
0.009090   0.013507   0.018405   0.013444 -0.016802   0.022941
0.010198   -0.037026   0.010825   0.007184   0.013452   0.019193 -
0.033278   0.026487   0.013953 -0.010303 -0.011687 -0.030372 -
0.004986   -0.044000   0.001979 -0.002409 -0.029917   0.006819

0.018032	-0.006046	-0.008860	-0.042790	-0.013446	-0.032894	
0.014447	-0.028912					
z479	-0.007597	0.032059	0.020491	-0.015037	0.011049	
0.007916	-0.016416	0.034513	0.052018	-0.008431	-0.043213	-
0.032175	-0.024860	-0.016016	0.019926	0.033387	-0.010563	-
0.004995	0.020048	-0.025862	-0.010816	0.008696	-0.009564	-
0.046844	-0.028886	0.033750	0.004032	0.034300	0.020085	
0.020356	-0.012196	0.040582	-0.057124	0.017691	0.044409	
0.006725	0.042969					
x480	-0.026076	0.037185	0.007535	-0.001225	0.022646	
0.028672	-0.020633	-0.019475	0.062465	0.022783	-0.078807	
0.015448	0.024570	-0.000685	0.004934	0.014606	-0.040248	
0.013451	0.020864	0.007848	0.036529	-0.044971	-0.014838	
0.034092	0.062961	0.007837	0.014339	0.012025	-0.005079	-
0.043178	-0.010265	0.018189	0.013783	0.031868	0.015827	-
0.043518	0.016086					
y480	0.011701	0.008976	-0.007359	0.009951	0.002871	
0.013482	0.012384	0.016340	0.024930	-0.014201	0.012450	
0.013667	-0.045722	0.011583	0.007629	0.015724	0.020420	-
0.029325	0.022208	0.012738	-0.004759	-0.020238	-0.033737	-
0.006765	-0.039380	0.007713	-0.008063	-0.028483	0.005986	
0.012278	-0.015400	-0.010666	-0.046460	-0.011978	-0.030175	
0.016922	-0.028057					
z480	-0.011814	0.036609	0.023139	-0.015081	0.017979	
0.010308	-0.022154	0.032683	0.059179	-0.014345	-0.054402	-
0.034793	-0.019970	-0.015069	0.018536	0.029859	-0.021158	-
0.005852	0.022390	-0.029393	-0.010067	0.006988	-0.006667	-
0.046683	-0.026167	0.033215	0.008079	0.028141	0.019017	
0.021603	-0.013608	0.043722	-0.048815	0.005809	0.035654	
0.012907	0.046431					
x481	-0.027160	0.038687	0.013197	-0.000364	0.027667	
0.029779	-0.024731	-0.020086	0.066212	0.020949	-0.077431	
0.014269	0.027861	0.002691	0.004486	0.010447	-0.046160	
0.011780	0.020436	0.007487	0.034332	-0.046167	-0.005071	
0.039751	0.060877	0.000479	0.018882	0.009239	-0.002947	-
0.040201	0.001064	0.008841	0.008769	0.031722	0.004604	-
0.036224	0.018462					
y481	0.008883	0.012971	-0.006156	0.007993	0.006973	
0.016923	0.008960	0.015635	0.034677	-0.014600	-0.002641	
0.013046	-0.048169	0.012685	0.007066	0.019722	0.018202	-
0.023735	0.021304	0.010845	0.000969	-0.024157	-0.030980	-
0.006563	-0.033255	0.006322	-0.008774	-0.026298	0.001146	
0.012811	-0.023331	-0.011566	-0.053304	-0.012888	-0.028601	
0.022043	-0.023151					
z481	-0.015051	0.039364	0.025346	-0.016604	0.024105	
0.012926	-0.026411	0.032102	0.065221	-0.018162	-0.061560	-
0.034595	-0.018052	-0.014369	0.016312	0.025666	-0.027603	-
0.006290	0.024446	-0.034603	-0.007638	0.001095	-0.002686	-
0.048036	-0.025521	0.035344	0.007367	0.022530	0.016824	
0.020091	-0.009259	0.043909	-0.037983	-0.002825	0.026328	
0.016746	0.045539					
x482	-0.027419	0.040074	0.019280	-0.000955	0.031694	
0.032072	-0.026838	-0.019745	0.069871	0.020107	-0.071380	
0.013609	0.030921	0.004920	0.004363	0.006197	-0.049119	
0.009328	0.020812	0.008127	0.031298	-0.047480	-0.000496	
0.042074	0.055954	-0.005425	0.021496	0.008565	-0.000090	-
0.037481	0.011103	-0.000138	0.009760	0.031112	-0.007097	-
0.026480	0.017997					
y482	0.006392	0.018271	-0.003163	0.005064	0.010878	
0.021569	0.007314	0.015584	0.044870	-0.015765	-0.010622	

0.011827	-0.049616	0.012825	0.005913	0.021851	0.016359	-
0.024055	0.023084	0.009212	0.004633	-0.028568	-0.033892	-
0.009632	-0.031124	0.005503	-0.008698	-0.022169	-0.001888	
0.013061	-0.031614	-0.009546	-0.047267	-0.017008	-0.030726	
0.028061	-0.022211					
z482	-0.017766	0.040480	0.026820	-0.016881	0.029582	
0.013290	-0.031250	0.031893	0.068056	-0.021400	-0.069257	-
0.034486	-0.016006	-0.013913	0.015256	0.021491	-0.033667	-
0.004314	0.024599	-0.038415	-0.005099	0.000048	0.003045	-
0.047361	-0.023414	0.034800	0.006111	0.016641	0.014420	
0.018724	-0.005544	0.044926	-0.030890	-0.009868	0.020294	
0.015879	0.044572					
x483	-0.027679	0.040400	0.024365	-0.000587	0.034647	
0.032427	-0.029318	-0.018450	0.071417	0.018696	-0.066944	
0.013050	0.031947	0.006750	0.005567	0.003056	-0.052322	
0.009638	0.021405	0.009023	0.028031	-0.045645	0.004249	
0.044515	0.052088	-0.010775	0.022031	0.004011	0.001368	-
0.035583	0.017838	-0.009216	0.011293	0.031940	-0.014954	-
0.022554	0.016027					
y483	0.003638	0.023230	0.000516	0.003073	0.016511	
0.024178	0.004082	0.016287	0.053113	-0.016590	-0.020320	
0.010018	-0.051870	0.013833	0.004394	0.022747	0.011835	-
0.021383	0.025202	0.007804	0.005066	-0.030719	-0.034004	-
0.013369	-0.028223	0.004864	-0.010375	-0.024061	-0.003754	
0.009232	-0.036004	-0.014298	-0.044766	-0.015444	-0.030689	
0.025879	-0.023414					
z483	-0.020058	0.041413	0.028591	-0.017794	0.033844	
0.013863	-0.033899	0.031010	0.068992	-0.023841	-0.071652	-
0.033421	-0.012063	-0.013868	0.012866	0.016511	-0.036986	-
0.003595	0.025948	-0.039689	-0.002848	0.000447	0.007604	-
0.044801	-0.023023	0.033874	0.005811	0.016100	0.009336	
0.018377	-0.001014	0.045458	-0.022351	-0.017586	0.011128	
0.021922	0.045252					
x484	-0.027244	0.040192	0.028493	-0.000282	0.036212	
0.031554	-0.031292	-0.017026	0.072538	0.017707	-0.061921	
0.013156	0.032137	0.007797	0.008011	0.000686	-0.053872	
0.011915	0.021754	0.009964	0.025134	-0.042302	0.006923	
0.045965	0.046566	-0.013438	0.022611	0.001198	0.002061	-
0.033073	0.023377	-0.016175	0.011124	0.029588	-0.020130	-
0.017242	0.016681					
y484	0.001052	0.028051	0.004630	0.000864	0.022684	
0.026073	0.000863	0.017060	0.061559	-0.017265	-0.029287	
0.008787	-0.052275	0.014544	0.004049	0.022880	0.007674	-
0.016983	0.028789	0.005053	0.005882	-0.030801	-0.032067	-
0.017647	-0.024997	0.006055	-0.011836	-0.025240	-0.010478	
0.007908	-0.037561	-0.014645	-0.043631	-0.019051	-0.032691	
0.028098	-0.018813					
z484	-0.022136	0.041712	0.031005	-0.018963	0.036834	
0.014923	-0.035101	0.030703	0.067237	-0.026567	-0.070774	-
0.032516	-0.007870	-0.015108	0.009920	0.010898	-0.038416	-
0.003172	0.028344	-0.039287	-0.001911	0.003612	0.013499	-
0.040995	-0.022859	0.029833	0.004871	0.016715	0.004620	
0.016550	0.002617	0.042833	-0.011997	-0.020013	0.002091	
0.024659	0.041570					
x485	-0.026442	0.039732	0.032612	-0.000716	0.036776	
0.030920	-0.032243	-0.015113	0.073054	0.016606	-0.054999	
0.013816	0.032263	0.008184	0.010071	-0.001808	-0.053584	
0.015210	0.021609	0.012256	0.021596	-0.037733	0.008990	
0.046822	0.040407	-0.017205	0.021817	-0.001043	0.003093	-

0.031574   0.025876   -0.026028   0.012169   0.030517   -0.026526   -
0.010495   0.014940
y485   -0.002505   0.033778   0.009791   -0.002031   0.028622
0.029428   -0.001957   0.017572   0.069852   -0.020467   -0.036426
0.007754   -0.051320   0.016369   0.000445   0.020831   0.003027   -
0.012897   0.034143   0.001888   0.008515   -0.030191   -0.023440   -
0.019794   -0.024790   0.004257   -0.012536   -0.024623   -0.013250
0.007767   -0.039701   -0.015181   -0.036691   -0.019722   -0.037590
0.029255   -0.018332
z485   -0.023557   0.040530   0.032710   -0.019201   0.039364
0.013331   -0.035940   0.031725   0.062535   -0.027629   -0.067940   -
0.031641   -0.003748   -0.016968   0.007887   0.006264   -0.038275   -
0.002468   0.029771   -0.037605   -0.002086   0.010832   0.017947   -
0.036216   -0.022716   0.027722   0.002035   0.018381   -0.003365
0.016029   0.007600   0.043417   -0.003447   -0.024502   -0.003363
0.024165   0.039167
x486   -0.025098   0.038738   0.035625   -0.001324   0.036503
0.029204   -0.032896   -0.012042   0.072055   0.016023   -0.046235
0.014623   0.031714   0.007415   0.013495   -0.004382   -0.050794
0.019517   0.022430   0.014566   0.017828   -0.030351   0.010374
0.046306   0.032809   -0.018966   0.019108   -0.002911   0.003389   -
0.029685   0.025174   -0.032130   0.015205   0.028565   -0.028882   -
0.007808   0.013244
y486   -0.005096   0.039526   0.015057   -0.005141   0.036930
0.031216   -0.005080   0.019739   0.075008   -0.022745   -0.039977
0.004860   -0.050012   0.015677   -0.001413   0.017845   -0.001839   -
0.010048   0.040009   -0.001674   0.009178   -0.025188   -0.014287   -
0.023008   -0.024513   0.001413   -0.012291   -0.023109   -0.019999
0.003968   -0.037056   -0.013434   -0.030605   -0.025562   -0.042795
0.028376   -0.014645
z486   -0.024865   0.038540   0.034795   -0.018707   0.039460
0.012533   -0.034791   0.031643   0.056823   -0.030286   -0.064192   -
0.030145   0.000693   -0.018782   0.004991   0.001017   -0.037557   -
0.000577   0.031587   -0.033614   -0.002277   0.017141   0.020140   -
0.031010   -0.022641   0.023635   -0.001605   0.021310   -0.011679
0.016437   0.011106   0.039097   0.006564   -0.024897   -0.013140
0.029742   0.032323
x487   -0.023775   0.037123   0.037626   -0.001108   0.034547
0.026410   -0.033005   -0.007952   0.069800   0.014933   -0.036694
0.015618   0.030809   0.007658   0.016261   -0.007379   -0.044908
0.025294   0.024973   0.018461   0.014942   -0.023905   0.011465
0.044662   0.023062   -0.018487   0.014748   -0.003525   0.001954   -
0.025149   0.019072   -0.036029   0.023585   0.024213   -0.028686   -
0.003524   0.010809
y487   -0.007447   0.044680   0.020724   -0.007368   0.044697
0.031757   -0.006604   0.022136   0.079666   -0.025586   -0.042009
0.002728   -0.046812   0.015912   -0.003764   0.012192   -0.008589   -
0.007753   0.044953   -0.005151   0.004553   -0.021537   -0.007613   -
0.025084   -0.023430   -0.003420   -0.018199   -0.011614   -0.028847   -
0.003113   -0.033885   -0.016910   -0.027020   -0.032587   -0.048868
0.034539   -0.009874
z487   -0.025506   0.035595   0.037936   -0.020135   0.039182
0.011623   -0.033953   0.031116   0.048646   -0.032154   -0.057118   -
0.028488   0.005268   -0.023657   0.004532   -0.001622   -0.036043
0.002009   0.032581   -0.029321   -0.003137   0.031718   0.024895   -
0.025895   -0.022692   0.015861   -0.001966   0.018079   -0.018086
0.015787   0.017535   0.035237   0.011650   -0.017387   -0.022602
0.026240   0.024197
x488   -0.021650   0.034638   0.040013   -0.002076   0.031542
0.022812   -0.032547   -0.003149   0.066249   0.014701   -0.025591

0.017719	0.029476	0.005922	0.020938	-0.008059	-0.037558	
0.031933	0.027370	0.023525	0.011945	-0.012855	0.009586	
0.042694	0.014028	-0.018669	0.009436	-0.006160	-0.004686	-
0.022687	0.012793	-0.039278	0.030439	0.021674	-0.029670	-
0.000151	0.008913					
y488	-0.009977	0.048926	0.027287	-0.010158	0.053414	
0.032484	-0.007750	0.023833	0.083310	-0.029611	-0.040328	
0.000222	-0.043077	0.015168	-0.008278	0.007397	-0.012793	-
0.003652	0.054291	-0.006753	0.003579	-0.008675	0.002443	-
0.029170	-0.026239	-0.010368	-0.023312	-0.007281	-0.041982	-
0.006125	-0.028986	-0.013169	-0.016777	-0.038634	-0.061544	
0.038929	-0.009103					
z488	-0.026191	0.033245	0.039578	-0.019689	0.037449	
0.008758	-0.033032	0.032694	0.038501	-0.033827	-0.050840	-
0.027054	0.009501	-0.026795	0.003213	-0.006465	-0.034934	
0.003786	0.031305	-0.024884	-0.004147	0.040203	0.028209	-
0.016405	-0.021120	0.011605	-0.003156	0.022445	-0.020259	
0.016639	0.023229	0.026529	0.017373	-0.011224	-0.026852	
0.024798	0.016263					
x489	-0.021649	0.036689	0.034704	0.000045	0.027552	
0.024833	-0.031921	-0.011581	0.060485	0.019850	-0.021493	
0.022500	0.032705	0.004281	0.017848	-0.007776	-0.034286	
0.031164	0.024964	0.020627	0.009723	-0.009243	0.008185	
0.035230	0.010075	-0.012638	0.010624	-0.005423	0.008596	-
0.021752	0.009013	-0.043895	0.027182	0.031005	-0.021987	-
0.004016	0.009814					
y489	-0.011537	0.061614	0.028076	-0.008813	0.061131	
0.033901	-0.013794	0.012834	0.080803	-0.034101	-0.040262	
0.000331	-0.038261	0.010954	-0.017910	-0.001465	-0.012916	
0.004130	0.054906	-0.009749	0.008393	-0.005814	0.012844	-
0.029547	-0.015158	-0.006303	-0.024500	-0.008063	-0.039880	
0.000099	-0.028637	-0.006318	-0.009395	-0.037910	-0.057873	
0.034848	-0.010634					
z489	-0.026303	0.031532	0.033551	-0.016064	0.033446	
0.008810	-0.033343	0.021524	0.025290	-0.036014	-0.046806	-
0.026447	0.012874	-0.026872	-0.004574	-0.009797	-0.029096	-
0.000176	0.018229	-0.015391	-0.001129	0.033825	0.032978	-
0.008561	-0.021627	0.015672	0.002989	0.023420	-0.011346	
0.018905	0.021505	0.026361	0.014613	-0.007973	-0.020275	
0.023929	0.011888					
x490	-0.021413	0.038920	0.029621	0.003155	0.023461	
0.026123	-0.032153	-0.018926	0.054611	0.022750	-0.016892	
0.025487	0.034720	0.004134	0.014321	-0.008088	-0.031287	
0.031397	0.022707	0.018374	0.006454	-0.009599	0.007506	
0.026672	0.004423	-0.006195	0.011627	-0.002575	0.021538	-
0.020779	0.007731	-0.049291	0.025637	0.038725	-0.013006	-
0.007341	0.012210					
y490	-0.012690	0.071511	0.027420	-0.007019	0.066749	
0.035569	-0.017373	0.000836	0.077319	-0.039910	-0.038407	
0.000994	-0.034180	0.008574	-0.029274	-0.006229	-0.012242	
0.010336	0.052109	-0.009181	0.011994	-0.004610	0.022581	-
0.028255	-0.008837	-0.003661	-0.026388	-0.005703	-0.035674	
0.005091	-0.024915	0.001635	-0.007815	-0.034478	-0.051400	
0.032603	-0.011128					
z490	-0.025664	0.028753	0.030472	-0.014555	0.029957	
0.007372	-0.034020	0.014185	0.013315	-0.033821	-0.044267	-
0.025906	0.018532	-0.030052	-0.005788	-0.016237	-0.023471	-
0.004199	0.006800	-0.011368	-0.001885	0.030854	0.033653	-
0.005800	-0.017214	0.017130	0.011110	0.018334	-0.005911	

0.018482	0.013225	0.021801	0.015338	-0.010242	-0.017703	
0.016287	0.005503					
x491	-0.020955	0.041189	0.025297	0.005788	0.019202	
0.027795	-0.032299	-0.025745	0.049003	0.023580	-0.012348	
0.027167	0.035805	0.003306	0.010239	-0.007862	-0.029114	
0.032351	0.020061	0.016510	0.002915	-0.009100	0.007576	
0.018662	-0.000334	-0.001741	0.011976	0.000072	0.032214	-
0.019237	0.008283	-0.053763	0.022743	0.045460	-0.006813	-
0.011090	0.014173					
y491	-0.013227	0.080832	0.027651	-0.005358	0.071623	
0.036371	-0.021236	-0.008935	0.072628	-0.044189	-0.035260	-
0.000280	-0.029590	0.005557	-0.039365	-0.010110	-0.010277	
0.014595	0.047291	-0.009177	0.013995	-0.005767	0.029132	-
0.027365	-0.003696	-0.000705	-0.025269	-0.003354	-0.030679	
0.007922	-0.020059	0.005302	-0.003270	-0.033773	-0.047792	
0.027436	-0.013151					
z491	-0.025114	0.023248	0.025673	-0.012923	0.026434	
0.005861	-0.033872	0.007808	0.003641	-0.031372	-0.041698	-
0.023492	0.021213	-0.030873	-0.004786	-0.021235	-0.020438	-
0.006937	-0.001992	-0.004797	-0.000696	0.032804	0.041256	-
0.003791	-0.012565	0.015764	0.014242	0.016299	-0.001675	
0.014601	0.011263	0.025651	0.011786	-0.008194	-0.010400	
0.010925	0.004323					
x492	-0.020110	0.042848	0.021932	0.009161	0.014788	
0.027347	-0.032161	-0.031232	0.043047	0.022488	-0.008132	
0.028807	0.036418	0.005067	0.006642	-0.008271	-0.026391	
0.035495	0.016726	0.016509	-0.001388	-0.012359	0.007014	
0.012095	-0.007470	0.002449	0.010893	0.004470	0.039249	-
0.015024	0.011392	-0.054829	0.018507	0.048998	0.000653	-
0.014375	0.020140					
y492	-0.013648	0.087920	0.027558	-0.004439	0.075478	
0.036161	-0.023189	-0.018556	0.067215	-0.047308	-0.032533	-
0.001406	-0.023972	0.002398	-0.047545	-0.014625	-0.008048	
0.018323	0.040167	-0.010030	0.013486	-0.008012	0.037888	-
0.027390	0.000261	0.000268	-0.024309	-0.001203	-0.026171	
0.007695	-0.016973	0.008008	-0.001418	-0.032935	-0.044319	
0.019066	-0.014906					
z492	-0.025114	0.015570	0.020985	-0.012826	0.020766	
0.005922	-0.029848	0.003602	-0.004863	-0.026264	-0.041817	-
0.019764	0.024637	-0.032605	-0.003894	-0.026475	-0.015780	-
0.009826	-0.009000	-0.001859	0.002542	0.036639	0.037658	
0.002148	-0.007784	0.013948	0.014299	0.010813	0.000897	
0.013279	0.006023	0.024926	0.007504	-0.007779	-0.015556	
0.010545	-0.007563					
x493	-0.018922	0.045152	0.018706	0.010335	0.009806	
0.028839	-0.033067	-0.036979	0.038490	0.021248	-0.004943	
0.028812	0.035251	0.001982	0.000807	-0.007915	-0.023614	
0.036744	0.011854	0.013715	-0.003234	-0.008679	0.008375	
0.005387	-0.006747	0.004411	0.009291	0.002825	0.042480	-
0.016221	0.014158	-0.059035	0.014724	0.053804	-0.001418	-
0.020449	0.016487					
y493	-0.013348	0.092457	0.026561	-0.003173	0.076961	
0.035726	-0.023662	-0.027159	0.060528	-0.048933	-0.028305	-
0.003253	-0.018761	-0.000572	-0.053984	-0.017382	-0.004105	
0.021311	0.031663	-0.010387	0.012827	-0.009236	0.044334	-
0.027156	0.003004	-0.000182	-0.023455	0.002357	-0.020225	
0.006300	-0.010748	0.012503	-0.000675	-0.033423	-0.041460	
0.010114	-0.017953					
z493	-0.024922	0.007560	0.018790	-0.011782	0.016242	
0.002436	-0.026545	0.003973	-0.013231	-0.022348	-0.038931	-

0.014518	0.029663	-0.027257	-0.000543	-0.030481	-0.014103	-
0.011127	-0.012540	0.005470	0.001680	0.032321	0.035884	
0.008698	-0.010130	0.012113	0.019050	0.008702	0.001142	
0.015783	0.006347	0.020982	0.002630	-0.003898	-0.014013	
0.012820	-0.004024					
x494	-0.017375	0.046886	0.016479	0.012133	0.004636	
0.028796	-0.033944	-0.041340	0.033353	0.017789	-0.002090	
0.027487	0.033831	0.001271	-0.005194	-0.007931	-0.021132	
0.039378	0.006265	0.012873	-0.006324	-0.009932	0.008831	-
0.000930	-0.009096	0.004769	0.007916	0.002454	0.043533	-
0.015295	0.019327	-0.060425	0.010291	0.054858	-0.003699	-
0.027208	0.015711					
y494	-0.012970	0.094580	0.025465	-0.002443	0.076912	
0.034725	-0.022392	-0.034736	0.052464	-0.049353	-0.023026	-
0.003916	-0.012955	-0.003314	-0.058112	-0.018594	0.000993	
0.024724	0.021667	-0.010574	0.011891	-0.009416	0.049782	-
0.027552	0.004996	-0.001542	-0.021116	0.004235	-0.017920	
0.003505	-0.002623	0.015891	-0.002298	-0.033279	-0.040848	
0.001328	-0.019598					
z494	-0.023759	-0.000832	0.016638	-0.011444	0.011462	-
0.001090	-0.021792	0.005805	-0.018996	-0.015717	-0.037195	-
0.012280	0.034525	-0.023411	0.001277	-0.035506	-0.011360	-
0.017074	-0.017193	0.012989	-0.001632	0.030492	0.030913	
0.017788	-0.011307	0.011179	0.018872	0.004319	0.005273	
0.012899	0.003891	0.012707	-0.000449	-0.002707	-0.010284	
0.010495	-0.008209					
x495	-0.015379	0.048842	0.014679	0.014550	-0.000528	
0.028660	-0.035765	-0.044780	0.028166	0.012530	0.001775	
0.024978	0.031246	0.000682	-0.011043	-0.007243	-0.018718	
0.042994	0.000621	0.013450	-0.010052	-0.012193	0.010035	-
0.008440	-0.010923	0.005040	0.005363	0.004079	0.040248	-
0.014957	0.028697	-0.060757	0.006417	0.054111	-0.007554	-
0.032391	0.016929					
y495	-0.011928	0.095041	0.024380	-0.001673	0.075179	
0.033438	-0.020202	-0.041059	0.043292	-0.048814	-0.016168	-
0.006632	-0.007056	-0.005291	-0.062132	-0.017654	0.007105	
0.026714	0.010159	-0.009638	0.009667	-0.008438	0.056856	-
0.026594	0.006274	-0.005047	-0.016989	0.005489	-0.011265	-
0.002013	0.009128	0.017900	-0.005159	-0.031416	-0.039537	-
0.013486	-0.021583					
z495	-0.022249	-0.009664	0.013194	-0.008858	0.007298	-
0.003473	-0.020159	0.009723	-0.023436	-0.009814	-0.034807	-
0.010268	0.036138	-0.022649	0.009796	-0.037224	-0.012392	-
0.021188	-0.019906	0.016655	-0.005231	0.028936	0.023059	
0.018122	-0.007598	0.011562	0.016341	0.002427	0.004559	
0.006317	0.001378	0.007935	0.001410	-0.004705	-0.008135	
0.017698	-0.007220					
x496	-0.013328	0.050460	0.013496	0.016529	-0.006133	
0.028231	-0.037059	-0.047677	0.022690	0.005578	0.004761	
0.022077	0.027905	0.000700	-0.017671	-0.007475	-0.016786	
0.047690	-0.006175	0.014057	-0.014052	-0.015758	0.010935	-
0.014160	-0.013341	0.004151	0.001159	0.006031	0.035207	-
0.014320	0.039208	-0.060793	0.001638	0.051974	-0.013893	-
0.038422	0.016249					
y496	-0.010170	0.092867	0.022945	-0.001283	0.071438	
0.031388	-0.015970	-0.046141	0.032819	-0.046826	-0.008706	-
0.008599	-0.000498	-0.007861	-0.063817	-0.015546	0.014729	
0.027446	-0.003012	-0.009109	0.007047	-0.007415	0.061323	-
0.025229	0.007456	-0.010303	-0.012446	0.005123	-0.009601	-

0.009304	0.020601	0.021096	-0.009502	-0.032343	-0.041230	-	
0.028509	-0.023552						
z496	-0.021157	-0.019754	0.010028	-0.008264	0.001984	-	
0.005034	-0.016209	0.014217	-0.026004	-0.003646	-0.033988	-	
0.005964	0.037521	-0.020825	0.016658	-0.042060	-0.015796	-	
0.025567	-0.020044	0.018866	-0.007628	0.027204	0.015297		
0.021862	-0.007781	0.010017	0.014520	0.002247	0.007749		
0.004479	-0.001671	0.000313	0.003060	-0.005522	-0.008073		
0.025614	-0.015359						
x497	-0.010960	0.052078	0.012960	0.018314	-0.011653		
0.027459	-0.039185	-0.049846	0.018426	-0.003245	0.007412		
0.018109	0.023501	0.000003	-0.024084	-0.007016	-0.014895		
0.053543	-0.013078	0.013862	-0.018118	-0.017939	0.013895	-	
0.020041	-0.013375	0.002168	-0.003992	0.008551	0.027207	-	
0.013853	0.051516	-0.060587	-0.005270	0.050434	-0.023033	-	
0.043366	0.017899						
y497	-0.007943	0.088725	0.020984	-0.000856	0.066167		
0.029095	-0.010890	-0.050148	0.021380	-0.043656	-0.000059	-	
0.011228	0.006578	-0.010217	-0.063945	-0.012052	0.023999		
0.027449	-0.017689	-0.007308	0.004210	-0.006117	0.064922	-	
0.024200	0.008415	-0.016433	-0.007314	0.005389	-0.007632	-	
0.017091	0.034641	0.025670	-0.013993	-0.033111	-0.043487	-	
0.044105	-0.025748						
z497	-0.020799	-0.029961	0.007660	-0.008256	-0.001454	-	
0.007401	-0.013831	0.021110	-0.023836	0.002584	-0.033858	-	
0.000828	0.040146	-0.017652	0.025122	-0.046554	-0.016501	-	
0.027521	-0.014367	0.021077	-0.008439	0.027503	0.011525		
0.023952	-0.006099	0.011552	0.010578	0.004164	0.009811		
0.008693	-0.007588	-0.003579	0.003607	-0.000185	-0.003063		
0.036085	-0.013357						
x498	-0.004998	0.052226	0.012431	0.020641	-0.020238		
0.018123	-0.038651	-0.047498	0.010801	-0.007658	0.009469		
0.016742	0.017792	0.007323	-0.026270	-0.000253	-0.016258		
0.047027	-0.016544	0.027272	-0.015808	-0.005324	0.005537	-	
0.026491	-0.013193	0.001289	-0.010743	0.002924	0.017934	-	
0.005109	0.039573	-0.048814	-0.006047	0.045513	-0.013900	-	
0.013486	0.009716						
y498	-0.006308	0.074147	0.019722	0.001403	0.054188		
0.014855	0.001091	-0.040782	0.012488	-0.024102	0.009291	-	
0.007383	0.015575	-0.005462	-0.048380	-0.008107	0.021183		
0.011184	-0.020967	-0.001920	0.002379	0.009411	0.044549	-	
0.008762	0.007377	-0.009060	-0.000504	0.001101	-0.010285	-	
0.020377	0.036774	0.018576	-0.012377	-0.024786	-0.023319	-	
0.025815	-0.015053						
z498	-0.017527	-0.040532	0.005250	-0.009138	-0.003494	-	
0.010534	-0.015116	0.028156	-0.022818	0.004033	-0.038601		
0.006683	0.037839	-0.019274	0.035033	-0.044307	-0.014092	-	
0.024977	-0.012232	0.024996	-0.009940	0.029638	0.003216		
0.032179	-0.002341	0.012004	0.008661	-0.002094	0.003238		
0.008575	-0.013449	-0.004475	-0.004334	0.011250	-0.011274		
0.047671	-0.004970						
x499	0.000183	0.050970	0.011220	0.023508	-0.028082		
0.009297	-0.038908	-0.043638	0.005224	-0.012948	0.009526		
0.013623	0.013099	0.012277	-0.024751	0.005849	-0.019991		
0.039454	-0.020431	0.035492	-0.015117	0.005673	-0.002851	-	
0.031857	-0.009718	-0.002254	-0.017754	-0.000130	0.011199		
0.002445	0.028298	-0.036452	-0.006805	0.037257	-0.007069		
0.006969	0.005297						
y499	-0.004416	0.059664	0.018245	0.001665	0.044009		
0.003227	0.010394	-0.031169	0.005687	-0.006295	0.017954	-	

0.003157	0.024018	-0.002328	-0.033114	-0.004636	0.021766	-
0.002724	-0.022570	0.004107	0.002125	0.023279	0.026322	
0.002947	0.008086	-0.001758	0.003364	-0.004072	-0.016642	-
0.024486	0.038760	0.016509	-0.010241	-0.018528	-0.005798	-
0.009900	-0.004160					
z499	-0.014132	-0.047970	0.001761	-0.012382	-0.002185	-
0.007259	-0.018248	0.033012	-0.016078	0.002859	-0.039469	
0.009442	0.036575	-0.022190	0.042223	-0.051421	-0.010830	-
0.023544	-0.006525	0.022247	-0.016329	0.024260	-0.000411	
0.028364	0.000237	0.008528	-0.001478	0.000267	0.010651	
0.004711	-0.022238	-0.002426	-0.002999	0.011276	-0.014263	
0.040746	-0.006773					
x500	0.004099	0.048863	0.009925	0.024577	-0.033981	
0.002454	-0.037983	-0.039658	0.000480	-0.017513	0.008628	
0.011565	0.010304	0.016075	-0.022777	0.008786	-0.022567	
0.034479	-0.021680	0.042613	-0.014039	0.015771	-0.009204	-
0.034875	-0.006287	-0.004316	-0.022447	-0.005409	0.005463	
0.009266	0.019029	-0.028431	-0.008253	0.031287	-0.004270	
0.022463	0.002560					
y500	-0.003396	0.045898	0.017202	0.000984	0.036872	-
0.005913	0.017247	-0.022245	0.000435	0.008367	0.021681	-
0.000680	0.030829	-0.000866	-0.020313	-0.003198	0.021379	-
0.012697	-0.023041	0.009168	0.000962	0.032167	0.012027	
0.011477	0.008396	0.003498	0.007002	-0.007575	-0.018196	-
0.026709	0.038722	0.013712	-0.005734	-0.010071	0.005243	
0.002421	0.001961					
z500	-0.012723	-0.053454	-0.000482	-0.017576	0.002486	-
0.003289	-0.019603	0.034623	-0.010412	-0.000705	-0.043588	
0.012833	0.033945	-0.024272	0.042180	-0.057660	-0.009090	-
0.016062	0.001462	0.020303	-0.018946	0.014729	0.001633	
0.026785	-0.000065	0.007513	-0.003971	-0.002740	0.015704	
0.004343	-0.025773	-0.007706	-0.002976	0.018966	-0.024373	
0.032608	-0.011878					
x501	0.008191	0.046365	0.008809	0.023827	-0.038326	-
0.000902	-0.037294	-0.034979	-0.002184	-0.019993	0.009767	
0.009942	0.009937	0.018508	-0.019232	0.006839	-0.022202	
0.028144	-0.021966	0.047068	-0.015768	0.022541	-0.012578	-
0.037378	-0.001942	-0.006617	-0.028279	-0.004919	-0.000209	
0.012802	0.009555	-0.019515	-0.008134	0.022455	-0.001498	
0.031517	0.000041					
y501	-0.002077	0.032779	0.016701	-0.000618	0.032435	-
0.012778	0.020550	-0.013485	-0.002323	0.019842	0.022672	
0.001264	0.034664	-0.000051	-0.009805	-0.002840	0.022138	-
0.019263	-0.023268	0.011629	-0.003670	0.037376	0.003167	
0.019153	0.009380	0.008415	0.009203	-0.007420	-0.018805	-
0.030194	0.036266	0.012843	-0.002102	-0.004531	0.014707	
0.010050	0.009133					
z501	-0.009614	-0.057366	-0.001774	-0.023099	0.008165	
0.002283	-0.020747	0.034481	-0.003449	-0.004632	-0.043262	
0.015148	0.030569	-0.026792	0.041500	-0.063197	-0.004457	-
0.011022	0.008489	0.014710	-0.025154	0.002418	0.004208	
0.024800	0.003019	0.006841	-0.007399	-0.000971	0.012067	-
0.000558	-0.030520	-0.004942	-0.003733	0.013986	-0.030865	
0.023042	-0.016712					
x502	0.010913	0.043213	0.006798	0.021960	-0.041083	-
0.003121	-0.037127	-0.030369	-0.003979	-0.021483	0.009672	
0.008810	0.011611	0.019505	-0.014777	0.002296	-0.021058	
0.023203	-0.020594	0.050804	-0.016328	0.028055	-0.013942	-
0.037343	0.002551	-0.008653	-0.032496	-0.007456	-0.002595	

0.015870 0.002383 -0.013094 -0.007948 0.014340 -0.000148
0.036511 0.001210
y502 -0.002042 0.020618 0.015700 -0.001861 0.030876 -
0.017702 0.020779 -0.005427 -0.003860 0.028958 0.018525
0.000616 0.038439 -0.001591 -0.001819 -0.003208 0.022276 -
0.023572 -0.023168 0.013322 -0.008193 0.038362 -0.001034
0.024683 0.009699 0.011969 0.010837 -0.007810 -0.013591 -
0.030790 0.034135 0.013087 0.003119 -0.000509 0.018969
0.014110 0.018327
z502 -0.008388 -0.059447 -0.003749 -0.027694 0.014196
0.006837 -0.021067 0.030597 0.001535 -0.009792 -0.043509
0.015684 0.026896 -0.029418 0.036930 -0.065295 -0.001987 -
0.005311 0.015342 0.008793 -0.025906 -0.010624 0.007411
0.023959 0.005155 0.006701 -0.005229 -0.006709 0.012717 -
0.001681 -0.030774 -0.004944 -0.001888 0.009430 -0.038896
0.012626 -0.017583
x503 0.012561 0.039014 0.005107 0.017667 -0.043537 -
0.003530 -0.036668 -0.024848 -0.003637 -0.022442 0.012186
0.009741 0.016628 0.019507 -0.008160 -0.005786 -0.017275
0.018786 -0.016078 0.051544 -0.016335 0.029554 -0.013340 -
0.035840 0.005869 -0.009589 -0.037702 -0.008016 -0.005190
0.016664 -0.001949 -0.013402 -0.006266 0.009672 0.002116
0.033450 0.005334
y503 -0.002630 0.008142 0.016064 -0.003658 0.029366 -
0.020664 0.019016 0.002477 -0.004285 0.033932 0.014803
0.001268 0.040606 -0.003192 0.007004 -0.004986 0.021297 -
0.025781 -0.021076 0.009939 -0.011885 0.033383 -0.001247
0.027478 0.008079 0.015757 0.010781 -0.005040 -0.010242 -
0.032349 0.033050 0.007723 0.009224 0.006828 0.023615
0.009268 0.025884
z503 -0.008012 -0.060167 -0.003299 -0.032780 0.018821
0.010724 -0.018901 0.023706 0.006581 -0.017097 -0.037912
0.016749 0.022755 -0.030235 0.032913 -0.064399 0.000004
0.001640 0.020686 -0.001607 -0.021838 -0.027043 0.010506
0.019590 0.008155 0.008725 -0.002407 -0.014038 0.007373 -
0.003210 -0.025870 -0.009550 0.002149 0.009849 -0.041917 -
0.001840 -0.022989
x504 0.014314 0.034079 0.002794 0.011895 -0.043690 -
0.001505 -0.036483 -0.018807 -0.001900 -0.019939 0.015332
0.010463 0.023548 0.018502 -0.001508 -0.018066 -0.010792
0.013814 -0.011208 0.050579 -0.016610 0.029902 -0.009598 -
0.031917 0.010903 -0.013345 -0.041542 -0.004998 -0.005094
0.014138 -0.004799 -0.010495 -0.002833 0.003684 0.004021
0.027412 0.008919
y504 -0.002526 -0.003312 0.016182 -0.006005 0.031757 -
0.021251 0.015058 0.010567 -0.002806 0.038515 0.008474 -
0.000968 0.041601 -0.006764 0.011576 -0.007614 0.023841 -
0.026230 -0.018198 0.007193 -0.017213 0.027758 0.003017
0.029592 0.007596 0.016128 0.010198 0.000277 -0.004239 -
0.035597 0.029893 0.010854 0.014307 0.012673 0.025958
0.008221 0.032016
z504 -0.006889 -0.058587 -0.003147 -0.036751 0.025057
0.014594 -0.014833 0.013843 0.010809 -0.024306 -0.029700
0.013996 0.017718 -0.031078 0.024681 -0.061786 0.002566
0.006732 0.026338 -0.010654 -0.019016 -0.039933 0.011941
0.017043 0.012320 0.008464 0.005134 -0.021893 0.001324 -
0.007128 -0.022344 -0.006420 0.007061 0.004658 -0.042392 -
0.010994 -0.031667
x505 0.014051 0.025709 -0.002158 0.010081 -0.044620 -
0.004131 -0.037341 -0.010418 -0.002237 -0.022364 0.009202

0.007205	0.027886	0.014878	0.008971	-0.021469	-0.012691	
0.010789	-0.010179	0.042272	-0.018535	0.032846	-0.011583	-
0.024057	0.019493	-0.019321	-0.038978	-0.009485	-0.002100	
0.011178	-0.008574	-0.011205	-0.003778	-0.002037	0.001671	
0.018570	0.016084					
y505	-0.003167	-0.015855	0.015070	-0.004931	0.034816	-
0.023478	0.008991	0.018683	-0.002287	0.036386	-0.006143	-
0.006770	0.038162	-0.012459	0.015372	-0.004207	0.020592	-
0.023051	-0.017898	-0.000973	-0.024981	0.020776	0.004654	
0.030267	0.008929	0.014303	0.010593	0.000339	0.004801	-
0.038004	0.023521	0.012069	0.014335	0.016553	0.023479	
0.008792	0.037240					
z505	-0.007200	-0.055875	-0.002913	-0.037825	0.030674	
0.015116	-0.009034	0.001038	0.012452	-0.035750	-0.023237	
0.008129	0.009841	-0.031351	0.014165	-0.051319	-0.000130	
0.013500	0.028678	-0.022823	-0.015119	-0.051422	0.009346	
0.014347	0.017524	0.010100	0.018542	-0.038170	-0.006125	-
0.009184	-0.018521	-0.005890	0.009718	-0.001522	-0.043422	-
0.019043	-0.042310					
x506	0.014453	0.017859	-0.004226	0.003170	-0.043334	-
0.000320	-0.032789	-0.003183	-0.001209	-0.017601	0.014092	
0.007815	0.035584	0.012294	0.014429	-0.029339	-0.006168	
0.006289	-0.003310	0.037297	-0.020701	0.030434	-0.011635	-
0.022078	0.021760	-0.016255	-0.034401	-0.012013	-0.000593	
0.006947	-0.011996	-0.013310	0.001425	-0.004404	0.000858	
0.003861	0.018853					
y506	-0.003394	-0.026032	0.016916	-0.006192	0.038983	-
0.019995	0.003609	0.022259	0.000260	0.034311	-0.015113	-
0.009708	0.031849	-0.016511	0.013831	-0.004443	0.021056	-
0.018377	-0.014892	-0.006657	-0.028449	0.005328	0.009964	
0.018566	0.004071	0.017092	0.009328	-0.000116	0.012978	-
0.037413	0.014953	0.014135	0.019019	0.022165	0.021138	
0.002599	0.034802					
z506	-0.007768	-0.049915	-0.000147	-0.040804	0.036862	
0.017486	0.000192	-0.015908	0.013592	-0.045426	-0.009719	
0.002889	0.001642	-0.029555	-0.001076	-0.040906	-0.000309	
0.019874	0.031196	-0.033123	-0.007737	-0.065770	0.007792	
0.007158	0.020557	0.016481	0.034724	-0.056320	-0.017404	-
0.010581	-0.011695	-0.005603	0.015227	-0.006994	-0.042746	-
0.028583	-0.056359					
x507	0.011900	0.005089	-0.003240	-0.001372	-0.028768	
0.000543	-0.027870	-0.003323	0.001115	-0.028412	0.008312	-
0.001679	0.033425	0.003695	0.016314	-0.025714	-0.004257	
0.011269	-0.000131	0.027830	-0.023288	0.010065	-0.013824	-
0.019749	0.031501	-0.006840	-0.021415	-0.020817	0.001972	
0.001767	-0.018263	-0.011357	0.002371	-0.001640	0.002926	-
0.004555	0.011233					
y507	-0.005611	-0.030494	0.015160	-0.007910	0.035448	-
0.024065	-0.004761	0.024817	0.000104	0.029321	-0.017338	-
0.003055	0.026841	-0.010940	0.010427	0.001107	0.016633	-
0.009438	-0.010773	-0.013353	-0.015354	0.004908	0.005596	
0.018934	-0.001421	0.017787	0.014234	-0.008693	0.012313	-
0.033779	0.026031	0.012538	0.016587	0.023846	0.017941	-
0.013105	0.030400					
z507	-0.003374	-0.038504	-0.001341	-0.036628	0.026845	
0.018606	0.002344	-0.015779	0.008595	-0.035355	-0.002143	
0.002134	-0.015915	-0.012657	-0.005947	-0.036654	-0.000159	
0.022580	0.026171	-0.041056	0.000935	-0.055384	0.013582	
0.002262	0.006523	0.001391	0.032268	-0.034453	-0.001444	-

0.003629 -0.006177 -0.004403 0.014471 -0.006841 -0.031860 -
0.017068 -0.046112
x508 0.011517 -0.004558 -0.002488 -0.006908 -0.016830
0.003600 -0.021083 -0.003188 0.004021 -0.029827 0.012105 -
0.010700 0.030174 -0.002424 0.018565 -0.024626 -0.003069
0.013612 0.003301 0.017566 -0.028204 -0.000873 -0.015875 -
0.026965 0.037765 -0.000132 -0.012033 -0.022089 0.001961 -
0.002784 -0.027429 -0.005022 0.009128 -0.005054 0.005175 -
0.006736 0.005019
y508 -0.006793 -0.030847 0.014038 -0.011035 0.027637 -
0.024925 -0.011730 0.026301 0.001342 0.027532 -0.013848
0.002145 0.019452 -0.004178 0.006282 0.001222 0.012612 -
0.000242 -0.006937 -0.017274 -0.003743 0.006547 0.004347
0.011412 -0.009316 0.013343 0.012127 -0.013516 0.015050 -
0.027611 0.031333 0.010608 0.017192 0.022085 0.015687 -
0.024140 0.025899
z508 0.000327 -0.026450 -0.001816 -0.032834 0.016160
0.018757 0.004820 -0.014310 0.004226 -0.023063 0.004663
0.002575 -0.030873 0.002822 -0.011936 -0.032248 0.000999
0.023266 0.021665 -0.044211 0.007869 -0.042511 0.017707 -
0.000268 -0.006713 -0.014026 0.027352 -0.013965 0.009176
0.002471 0.000534 -0.004306 0.008356 -0.004683 -0.023473 -
0.003047 -0.033493
x509 0.009722 -0.010506 -0.001289 -0.013619 -0.009909
0.005510 -0.014345 -0.002222 0.005804 -0.029575 0.013502 -
0.017986 0.018594 -0.005417 0.015402 -0.018994 -0.001762
0.015086 0.003712 0.007663 -0.027117 -0.003119 -0.013076 -
0.021356 0.041506 0.001910 -0.007724 -0.017724 0.003270 -
0.004935 -0.026953 -0.003191 0.002287 -0.004520 0.006280 -
0.003871 -0.001825
y509 -0.008321 -0.029099 0.012460 -0.013720 0.018236 -
0.024597 -0.017246 0.026864 0.001048 0.024078 -0.011174
0.005030 0.006865 0.003187 0.001590 0.000059 0.007272
0.007668 -0.003492 -0.021032 0.005087 0.007684 0.004537
0.005714 -0.016217 0.005534 0.007410 -0.012597 0.021359 -
0.022928 0.036337 0.006164 0.012943 0.015768 0.015175 -
0.028579 0.020414
z509 0.003326 -0.015750 -0.001963 -0.028568 0.006969
0.017358 0.006447 -0.012510 0.000251 -0.011662 0.007861
0.004454 -0.040108 0.015675 -0.016728 -0.025700 0.002863
0.022331 0.017604 -0.043715 0.015109 -0.030282 0.020470
0.001556 -0.016773 -0.024447 0.023919 0.001838 0.012309
0.007972 0.006696 -0.003240 0.000656 0.000584 -0.018335
0.008153 -0.019157
x510 0.009583 -0.012327 0.002668 -0.021686 -0.003219
0.010092 -0.006662 -0.000498 0.009013 -0.022734 0.019744 -
0.021855 0.009626 -0.004159 0.008825 -0.016641 0.008592
0.017350 0.004734 0.005213 -0.023235 -0.004095 -0.007968 -
0.019271 0.033133 0.002152 -0.004197 -0.012595 0.003518 -
0.008422 -0.020976 -0.009031 -0.004262 0.002815 0.007965 -
0.000646 -0.001688
y510 -0.009005 -0.025914 0.011916 -0.015213 0.010666 -
0.023356 -0.020513 0.026983 -0.000248 0.022586 -0.007772
0.005887 -0.002804 0.011739 -0.002048 -0.001904 0.004848
0.014015 0.000460 -0.021372 0.012779 0.007398 0.004685 -
0.001685 -0.025213 -0.001349 0.003515 -0.011766 0.025948 -
0.021189 0.039623 0.001611 0.009964 0.011342 0.016256 -
0.029125 0.022027
z510 0.005905 -0.007419 -0.003770 -0.023912 -0.000745
0.015686 0.007050 -0.012357 -0.003434 -0.002216 0.009807

0.006679	-0.044248	0.023539	-0.018243	-0.021086	0.002019	
0.019199	0.014426	-0.043579	0.018226	-0.021069	0.020953	
0.001425	-0.020247	-0.028499	0.020846	0.014887	0.011392	
0.011709	0.007365	-0.001385	-0.003125	0.000951	-0.015515	
0.015015	-0.009063					
x511	0.009194	-0.013415	0.001431	-0.024882	0.000114	
0.011402	0.000890	-0.001134	0.009523	-0.017929	0.020260	-
0.026651	-0.004696	-0.004453	0.009014	-0.014277	0.009795	
0.018777	0.001567	-0.002787	-0.019720	-0.003691	-0.006726	-
0.019993	0.029088	0.001424	-0.006389	-0.005387	0.004460	-
0.010481	-0.020507	-0.013076	-0.006931	-0.005381	0.007387	
0.006578	-0.000878					
y511	-0.010052	-0.021982	0.009902	-0.015031	0.002818	-
0.023270	-0.022021	0.025936	-0.002889	0.019179	-0.006167	
0.005827	-0.012238	0.019540	-0.004217	-0.004497	0.000699	
0.017976	0.002071	-0.021874	0.019458	0.005459	0.005046	-
0.005895	-0.030679	-0.005202	-0.000827	-0.008963	0.028486	-
0.018116	0.038360	-0.000707	0.007240	0.002613	0.012540	-
0.026348	0.026006					
z511	0.008108	-0.001076	-0.004744	-0.019889	-0.007037	
0.013692	0.005620	-0.012058	-0.006760	0.006355	0.010868	
0.008294	-0.042859	0.028848	-0.019294	-0.014178	-0.000456	
0.014370	0.013005	-0.040447	0.019519	-0.011408	0.022200	
0.002267	-0.020002	-0.029310	0.020456	0.024136	0.007186	
0.013437	0.007301	0.000667	-0.009873	0.007370	-0.011944	
0.017416	-0.000930					
x512	0.010075	-0.012553	0.002568	-0.029761	0.002299	
0.013331	0.007526	-0.002517	0.010772	-0.009954	0.025764	-
0.029936	-0.014026	-0.000725	0.006356	-0.011560	0.009555	
0.016566	0.000697	-0.008399	-0.014394	0.000154	0.001936	-
0.017039	0.024168	-0.000189	-0.009698	0.009763	0.003412	-
0.010802	-0.018423	-0.014104	-0.011301	-0.006466	0.011064	
0.015284	0.001340					
y512	-0.010399	-0.017395	0.008790	-0.013780	-0.004663	-
0.022689	-0.022673	0.024306	-0.006417	0.015196	-0.003216	
0.003781	-0.017934	0.026804	-0.005420	-0.006686	-0.003772	
0.017871	0.003389	-0.021434	0.024276	0.004030	0.007219	-
0.007282	-0.033869	-0.007368	-0.004501	-0.002697	0.027829	-
0.015624	0.033377	0.001459	0.003079	-0.003350	0.010935	-
0.021355	0.032105					
z512	0.009286	0.003975	-0.008243	-0.015852	-0.012736	
0.011562	0.004881	-0.012817	-0.010719	0.015231	0.009304	
0.010205	-0.040626	0.029763	-0.018940	-0.010835	-0.004067	
0.010842	0.011808	-0.035467	0.018183	-0.004780	0.020456	-
0.000976	-0.017979	-0.025435	0.018129	0.027101	0.002787	
0.013690	0.005152	-0.001877	-0.016107	0.010478	-0.014368	
0.017474	0.003053					
x513	0.011639	-0.011335	0.001724	-0.035920	0.003524	
0.014392	0.013879	-0.005207	0.010207	0.000810	0.031467	-
0.032489	-0.024186	0.004701	0.005311	-0.008815	0.006264	
0.015919	-0.000740	-0.013507	-0.007027	0.007396	0.013007	-
0.018166	0.017150	-0.000899	-0.012406	0.020681	0.002239	-
0.010233	-0.014719	-0.018069	-0.017213	-0.008319	0.012880	
0.023151	-0.000167					
y513	-0.010688	-0.012727	0.006571	-0.012068	-0.012163	-
0.021828	-0.022045	0.022104	-0.011873	0.010710	-0.000294	
0.000762	-0.022895	0.033535	-0.005933	-0.009556	-0.008299	
0.017530	0.002971	-0.019031	0.028126	0.002517	0.010108	-
0.009015	-0.036620	-0.006859	-0.007383	0.002729	0.024827	-

0.011926	0.028150	0.003084	-0.002982	-0.009359	0.006488	-
0.017649	0.034832					
z513	0.009915	0.009127	-0.011415	-0.010609	-0.018231	
0.009342	0.003798	-0.013597	-0.013853	0.024130	0.006146	
0.011824	-0.036394	0.027123	-0.018763	-0.008719	-0.007765	
0.004091	0.011988	-0.029547	0.015456	-0.002024	0.017940	-
0.002662	-0.011124	-0.019103	0.012395	0.031830	-0.003288	
0.010674	-0.000648	-0.005133	-0.022776	0.014267	-0.017436	
0.020074	0.012756					
x514	0.014319	-0.008331	-0.000750	-0.041054	0.001946	
0.015116	0.020846	-0.009217	0.007255	0.014857	0.038330	-
0.034678	-0.035564	0.006783	0.004922	-0.008130	0.000944	
0.013417	-0.002464	-0.018792	0.000681	0.015287	0.025515	-
0.020582	0.013678	0.000975	-0.019895	0.036055	-0.001232	-
0.009332	-0.014670	-0.023950	-0.024954	-0.013111	0.012476	
0.034249	-0.000462					
y514	-0.010885	-0.007182	0.004040	-0.009415	-0.020024	-
0.020365	-0.020235	0.019408	-0.019124	0.007019	0.003108	-
0.003479	-0.026737	0.039088	-0.006782	-0.013763	-0.013516	
0.014337	0.001400	-0.016042	0.031269	-0.000164	0.013742	-
0.009372	-0.036609	-0.003589	-0.010143	0.011463	0.020315	-
0.008895	0.018547	0.005475	-0.012114	-0.015845	-0.002752	-
0.010711	0.042104					
z514	0.009585	0.013718	-0.013595	-0.005662	-0.022328	
0.005879	0.001770	-0.014555	-0.014518	0.031594	0.001199	
0.013553	-0.028407	0.027109	-0.017768	-0.005539	-0.012719	-
0.002341	0.013054	-0.020410	0.011640	0.001866	0.015835	-
0.004275	-0.007386	-0.013289	0.007457	0.032847	-0.009942	
0.007071	-0.002651	-0.008246	-0.028857	0.023169	-0.016746	
0.019774	0.021603					
x515	0.017741	-0.003946	-0.003979	-0.045823	-0.002467	
0.014220	0.026709	-0.014242	0.004359	0.032518	0.045734	-
0.036992	-0.048078	0.011818	0.005284	-0.006687	-0.008033	
0.010837	-0.005082	-0.022117	0.009944	0.026567	0.040703	-
0.024178	0.008453	0.004253	-0.030173	0.054610	-0.008759	-
0.007811	-0.012772	-0.033374	-0.036922	-0.013602	0.013278	
0.047557	0.003632					
y515	-0.010786	-0.000272	0.001595	-0.006205	-0.027762	-
0.018177	-0.016729	0.015545	-0.027033	0.003617	0.006731	-
0.009273	-0.030130	0.044540	-0.006254	-0.018582	-0.018375	
0.010188	-0.000260	-0.011334	0.032574	-0.003823	0.018823	-
0.009232	-0.034708	0.000016	-0.013081	0.022416	0.014025	-
0.007815	0.006479	0.008086	-0.020676	-0.022483	-0.012149	-
0.000480	0.052755					
z515	0.008462	0.018900	-0.015850	0.000025	-0.024701	
0.002413	0.000315	-0.015017	-0.015529	0.035379	-0.004861	
0.016556	-0.015184	0.024894	-0.018077	-0.003969	-0.013712	-
0.012412	0.014966	-0.010719	0.005374	0.004856	0.010087	-
0.003194	-0.004459	-0.009517	0.001273	0.029585	-0.012346	
0.001171	-0.007413	-0.007777	-0.030821	0.021584	-0.018561	
0.018198	0.023619					
x516	0.016949	-0.009892	-0.003889	-0.052173	-0.005136	
0.013597	0.022799	-0.008941	0.007470	0.033713	0.036680	-
0.023935	-0.040272	0.002464	-0.002376	-0.010080	-0.011381	
0.017597	-0.006466	-0.019359	0.014640	0.033451	0.035068	-
0.019231	0.003603	0.013212	-0.024363	0.044418	-0.015856	-
0.010028	-0.015136	-0.031545	-0.038975	-0.010969	0.006559	
0.033782	0.013880					
y516	-0.010840	-0.000978	-0.000385	-0.008849	-0.031985	-
0.016554	-0.015209	0.016507	-0.026970	-0.005366	0.004620	-

0.004020	-0.025384	0.041064	-0.009813	-0.019898	-0.014358		
0.009614	0.002022	-0.007557	0.031160	-0.009753	0.017914	-
0.011697	-0.028001	0.002740	-0.009732	0.013916	0.005498	
0.003159	0.005245	0.011573	-0.021933	-0.017919	-0.014844	
0.001901	0.038023					
z516	0.008391	0.017769	-0.016199	0.005864	-0.024661	-
0.000571	-0.002361	-0.009288	-0.014166	0.031135	-0.014497	
0.025012	-0.001554	0.014319	-0.017145	-0.001366	-0.015325	-
0.009822	0.016023	-0.002032	-0.002875	0.002127	0.002233	-
0.008498	-0.003238	-0.006771	0.004721	0.015092	-0.016409	-
0.001386	-0.013759	0.001042	-0.021316	0.021361	-0.012309	
0.010793	0.017719					
x517	0.016297	-0.015513	-0.003973	-0.056709	-0.007647	
0.012757	0.019249	-0.003051	0.009647	0.035072	0.026665	-
0.010707	-0.030871	-0.006387	-0.008419	-0.015283	-0.013984	
0.022467	-0.007140	-0.016893	0.017533	0.037127	0.027709	-
0.015831	-0.000839	0.021616	-0.018465	0.031905	-0.023423	-
0.009875	-0.018624	-0.028755	-0.039648	-0.009250	-0.000483	
0.020837	0.019454					
y517	-0.010793	-0.001623	-0.001961	-0.011376	-0.035410	-
0.014708	-0.012509	0.016992	-0.026109	-0.013302	0.001254	
0.001087	-0.020573	0.037400	-0.011384	-0.021530	-0.010615	
0.009781	0.003566	-0.004907	0.028468	-0.016046	0.015405	-
0.014783	-0.020329	0.003805	-0.006478	0.006471	-0.001034	
0.011958	0.001942	0.014219	-0.022557	-0.016016	-0.017762	
0.004172	0.024799					
z517	0.008492	0.019499	-0.017724	0.012787	-0.024012	-
0.002376	-0.004742	-0.006011	-0.012279	0.026679	-0.020298	
0.028313	0.007815	0.007562	-0.018128	0.003046	-0.017167	-
0.008109	0.015499	0.004892	-0.009936	0.004392	-0.002629	-
0.012417	0.000334	-0.008409	0.005822	0.005678	-0.013423	-
0.009303	-0.016652	0.006142	-0.013772	0.020263	-0.006145	
0.003219	0.013031					
x518	0.015677	-0.019330	-0.004655	-0.058282	-0.009918	
0.011649	0.015492	0.001898	0.010301	0.034832	0.016391	-
0.000451	-0.023505	-0.013888	-0.012684	-0.017648	-0.017819	
0.024871	-0.007432	-0.013786	0.019400	0.040143	0.020526	-
0.013689	-0.002856	0.026736	-0.012244	0.019994	-0.028693	-
0.009806	-0.020503	-0.025175	-0.039344	-0.007884	-0.006024	
0.007832	0.020749					
y518	-0.010734	-0.001029	-0.002520	-0.013873	-0.038250	-
0.012051	-0.009451	0.016813	-0.024538	-0.019856	-0.001155	
0.005327	-0.017110	0.034014	-0.012466	-0.021610	-0.007034	
0.009772	0.004267	-0.002392	0.026466	-0.020730	0.013326	-
0.015803	-0.012658	0.003247	-0.003194	0.001639	-0.005884	
0.017646	-0.000084	0.015620	-0.022557	-0.013580	-0.020384	
0.005431	0.015061					
z518	0.008957	0.023582	-0.017881	0.019272	-0.022815	-
0.001924	-0.006979	-0.006619	-0.012011	0.025044	-0.021089	
0.028540	0.010946	0.005673	-0.020629	0.010606	-0.016513	-
0.008579	0.016330	0.011407	-0.013212	0.008708	-0.002328	-
0.010273	0.003092	-0.012620	0.003944	0.000514	-0.008616	-
0.019156	-0.013769	0.009530	-0.005686	0.021186	-0.000488	-
0.005589	0.005399					
x519	0.014932	-0.020729	-0.005138	-0.057607	-0.012077	
0.009835	0.012203	0.005085	0.009850	0.033436	0.007339	
0.006699	-0.018475	-0.019807	-0.015291	-0.016255	-0.020740	
0.024665	-0.005871	-0.009395	0.020759	0.041641	0.014007	-
0.010757	-0.004143	0.028117	-0.007008	0.011083	-0.031174	-

0.008754 -0.020569 -0.022487 -0.037129 -0.004384 -0.009205 -
0.003569 0.021225
y519 -0.010468 0.000546 -0.001656 -0.015844 -0.040764 -
0.009214 -0.006274 0.016486 -0.022645 -0.025144 -0.002483
0.007995 -0.015011 0.030813 -0.012924 -0.019538 -0.004266
0.008874 0.004829 0.000899 0.024471 -0.023668 0.011065 -
0.015348 -0.007172 0.001455 0.000169 -0.000934 -0.009394
0.022588 0.000170 0.014867 -0.021009 -0.009123 -0.022060
0.004827 0.008972
z519 0.010425 0.027860 -0.016819 0.025627 -0.021900 -
0.004502 -0.009073 -0.006854 -0.011215 0.024892 -0.019004
0.027604 0.013667 0.005542 -0.020966 0.018718 -0.010298 -
0.009940 0.020320 0.016929 -0.017598 0.011138 -0.002770 -
0.008279 0.000030 -0.015695 0.001614 -0.003114 -0.004037 -
0.022119 -0.012711 0.004658 0.003969 0.026249 0.002718 -
0.010051 0.006811
x520 0.014185 -0.019517 -0.005188 -0.055454 -0.013190
0.008790 0.010198 0.006207 0.008680 0.032795 0.001485
0.011400 -0.014136 -0.023448 -0.018558 -0.013879 -0.022688
0.021290 -0.002801 -0.003127 0.020498 0.040423 0.009805 -
0.008388 -0.007181 0.028310 -0.002512 0.005264 -0.030964 -
0.008990 -0.019199 -0.020146 -0.033460 -0.000570 -0.006788 -
0.014434 0.019499
y520 -0.010564 0.002641 -0.000936 -0.015666 -0.042506 -
0.006590 -0.003074 0.015455 -0.021275 -0.031003 -0.004277
0.008267 -0.013960 0.028054 -0.011633 -0.015916 -0.005118
0.006903 0.004121 0.003352 0.021453 -0.025372 0.008425 -
0.015677 -0.001078 -0.001220 0.004794 -0.000178 -0.011882
0.024239 0.001906 0.015622 -0.019832 -0.007535 -0.019432
0.001415 0.006780
z520 0.011318 0.031175 -0.018278 0.035254 -0.018899 -
0.006581 -0.011104 -0.007741 -0.012478 0.023214 -0.019298
0.025229 0.014895 0.009261 -0.021155 0.025058 -0.008482 -
0.011504 0.020690 0.016548 -0.022520 0.014175 0.001140 -
0.006428 -0.002059 -0.015200 0.001750 -0.004347 0.002443 -
0.030078 -0.014666 0.006562 0.005968 0.023555 0.012550 -
0.013962 0.010022
x521 0.013390 -0.016809 -0.004993 -0.051578 -0.014544
0.006609 0.008891 0.005706 0.006642 0.031212 -0.004554
0.013144 -0.010668 -0.026576 -0.019549 -0.009574 -0.025242
0.016045 0.000567 0.003337 0.019586 0.036804 0.004083 -
0.005401 -0.008504 0.024199 0.000590 0.000315 -0.031297 -
0.007676 -0.018116 -0.016506 -0.027112 0.002909 -0.002203 -
0.025116 0.013350
y521 -0.010258 0.005407 0.001041 -0.015557 -0.044064 -
0.003554 0.000392 0.013712 -0.019661 -0.036215 -0.005737
0.007358 -0.012350 0.025451 -0.007673 -0.012318 -0.007229
0.002888 0.002317 0.005681 0.017178 -0.026538 0.004400 -
0.015849 0.006003 -0.005771 0.008952 0.003069 -0.013293
0.025320 0.003491 0.018584 -0.016833 -0.006904 -0.015980 -
0.004262 0.004847
z521 0.014109 0.033078 -0.019382 0.042340 -0.015801 -
0.008449 -0.013861 -0.008167 -0.013107 0.023281 -0.018806
0.023649 0.017488 0.013881 -0.017952 0.028048 -0.003041 -
0.013302 0.020854 0.012088 -0.024929 0.015640 0.004750 -
0.003804 -0.000069 -0.016832 -0.001215 -0.006727 0.003340 -
0.034315 -0.017665 0.013347 0.007636 0.019726 0.015235 -
0.016024 0.008931
x522 0.012413 -0.012987 -0.004930 -0.045935 -0.015516
0.004597 0.007532 0.005096 0.004329 0.028778 -0.009632

0.012052	-0.008514	-0.028421	-0.019472	-0.004436	-0.027225	
0.009385	0.005829	0.009058	0.017323	0.032434	-0.002787	-
0.003215	-0.008291	0.016841	0.003006	-0.001650	-0.029265	-
0.005527	-0.015036	-0.014391	-0.020919	0.007565	0.001032	-
0.031450	0.005911					
y522	-0.009475	0.008888	0.003311	-0.015813	-0.044343	
0.001162	0.004524	0.011937	-0.017748	-0.040063	-0.005547	
0.005842	-0.011028	0.023691	-0.003521	-0.010458	-0.008486	-
0.001774	0.002424	0.008914	0.011767	-0.026346	-0.000524	-
0.016248	0.013436	-0.008576	0.012594	0.008657	-0.012929	
0.024090	0.008688	0.018826	-0.013594	-0.005999	-0.014611	-
0.006942	0.003177					
z522	0.018329	0.034019	-0.021481	0.047774	-0.010147	-
0.006810	-0.016638	-0.006790	-0.012407	0.026729	-0.013959	
0.024083	0.019243	0.021649	-0.016626	0.024504	0.009200	-
0.012944	0.024342	0.003467	-0.026062	0.018103	0.011053	-
0.002477	0.002017	-0.014472	-0.005817	-0.006634	0.005620	-
0.039532	-0.017650	0.014611	0.004479	0.017308	0.007787	-
0.006700	0.009908					
x523	0.011428	-0.008666	-0.005830	-0.038303	-0.016247	
0.002589	0.006798	0.004017	0.001235	0.027205	-0.014485	
0.011517	-0.007477	-0.030789	-0.020438	0.002053	-0.026350	
0.000496	0.009623	0.016202	0.014331	0.025119	-0.008836	-
0.001338	-0.007760	0.007833	0.003744	-0.005607	-0.026869	-
0.002865	-0.012505	-0.013501	-0.013108	0.012535	0.008418	-
0.038322	-0.001550					
y523	-0.008822	0.011693	0.005328	-0.014752	-0.044303	
0.005499	0.008859	0.010170	-0.015417	-0.042570	-0.007049	
0.004008	-0.011560	0.020222	0.001133	-0.007108	-0.012240	-
0.004950	0.000652	0.012428	0.006241	-0.023968	-0.005650	-
0.015142	0.019354	-0.011397	0.016812	0.011190	-0.012747	
0.021479	0.012451	0.015912	-0.009752	-0.005818	-0.010910	-
0.011682	0.006682					
z523	0.023161	0.032457	-0.025893	0.055130	-0.004024	-
0.005985	-0.020377	-0.004737	-0.011935	0.031886	-0.011348	
0.026250	0.017845	0.028826	-0.015917	0.021643	0.022596	-
0.010754	0.023500	-0.009126	-0.024303	0.021135	0.020097	
0.000027	0.003796	-0.011212	-0.009573	-0.012792	0.006265	-
0.044189	-0.021639	0.013739	-0.001727	0.012866	0.000010	
0.003974	0.016914					
x524	0.010775	-0.004242	-0.007092	-0.027567	-0.017372	-
0.000509	0.005345	0.002952	-0.001335	0.025543	-0.020343	
0.009681	-0.004883	-0.030981	-0.018346	0.006373	-0.027589	-
0.007794	0.012908	0.020382	0.012938	0.018686	-0.012264	
0.002293	-0.003645	-0.004423	0.006944	-0.011886	-0.022895	
0.002412	-0.012503	-0.012296	-0.009735	0.017794	0.009645	-
0.035860	-0.008573					
y524	-0.007769	0.013572	0.007836	-0.012998	-0.043682	
0.009939	0.012591	0.008803	-0.011963	-0.043406	-0.010483	
0.002249	-0.011244	0.017260	0.008540	-0.005810	-0.018999	-
0.006368	-0.002137	0.013976	0.000895	-0.017834	-0.011034	-
0.012631	0.024768	-0.013786	0.021762	0.011334	-0.011831	
0.018049	0.012437	0.012972	-0.009226	-0.005862	-0.011225	-
0.013014	0.011821					
z524	0.028716	0.029386	-0.030732	0.062475	0.002926	-
0.005812	-0.024930	-0.001945	-0.010997	0.037903	-0.008487	
0.028068	0.017095	0.039029	-0.012678	0.015676	0.035113	-
0.006500	0.022260	-0.028706	-0.020137	0.027859	0.032115	
0.003854	0.006775	-0.007002	-0.012215	-0.019102	0.008361	-

0.047977 -0.029956 0.015098 -0.014413 0.008105 -0.017104
0.023608 0.026649
x525 0.013997 -0.002271 -0.009053 -0.016680 -0.016049
0.001182 0.001723 0.001570 -0.001667 0.031944 -0.019353
0.010529 -0.005101 -0.025185 -0.016736 0.002704 -0.020233 -
0.010678 0.014991 0.013657 0.010778 0.019295 -0.006492
0.005473 -0.006752 0.003564 -0.002298 -0.019180 -0.026896 -
0.004826 -0.018367 -0.003283 -0.008207 0.010153 0.007331 -
0.034453 -0.010167
y525 -0.005938 0.014126 0.008934 -0.010846 -0.038352
0.020626 0.015049 0.008752 -0.009498 -0.036785 -0.009423
0.004385 -0.008531 0.015614 0.007535 -0.009210 -0.020401 -
0.010635 -0.001804 0.016870 -0.003708 -0.012933 -0.011679 -
0.002686 0.025213 -0.011448 0.014082 0.001222 -0.002808
0.015147 0.007713 0.011963 -0.004142 0.003999 -0.015273 -
0.007696 0.017347
z525 0.031741 0.031729 -0.032297 0.061858 0.003247 -
0.012239 -0.023040 -0.002298 -0.013095 0.038893 -0.006642
0.031060 0.016087 0.040188 -0.007963 0.016346 0.041459 -
0.009091 0.019910 -0.035449 -0.013682 0.026957 0.035422 -
0.002669 0.007012 0.001825 -0.012221 -0.013877 0.006857 -
0.041038 -0.036994 0.004750 -0.017431 0.007394 -0.013505
0.021937 0.017693
x526 0.017272 -0.000351 -0.010546 -0.006922 -0.015896
0.000186 -0.002728 0.001382 -0.001518 0.035858 -0.017679
0.012614 -0.003213 -0.020276 -0.014580 0.000963 -0.016025 -
0.007845 0.019294 0.007465 0.009847 0.016826 -0.002704
0.003528 -0.006497 -0.000299 -0.002209 -0.021690 -0.029867 -
0.006851 -0.016986 -0.000178 -0.012349 0.009051 0.000408 -
0.028906 -0.009805
y526 -0.004209 0.014421 0.009746 -0.008408 -0.033692
0.028188 0.016631 0.007838 -0.007419 -0.031551 -0.006820
0.007248 -0.004730 0.013443 0.006972 -0.011680 -0.021704 -
0.010957 0.000232 0.018918 -0.005631 -0.009409 -0.011500
0.002322 0.028475 -0.015825 0.012072 -0.004248 0.006453
0.013192 0.008082 0.010450 -0.001648 0.015276 -0.017637 -
0.002730 0.026253
z526 0.033751 0.033168 -0.033143 0.059892 0.003439 -
0.019360 -0.021365 -0.001646 -0.014256 0.038377 -0.004495
0.033747 0.015999 0.040770 -0.003630 0.018085 0.046692 -
0.008798 0.018120 -0.040989 -0.007536 0.024371 0.037846 -
0.010986 0.008477 0.004742 -0.009374 -0.007564 0.007058 -
0.032888 -0.038782 -0.005563 -0.022560 0.009267 -0.008892
0.020333 0.010289
x527 0.020915 0.003524 -0.012415 -0.001508 -0.015387 -
0.001304 -0.004298 0.000775 0.000353 0.043685 -0.014983
0.016260 -0.002563 -0.009874 -0.012634 0.000385 -0.002639 -
0.004455 0.021290 0.001950 0.009412 0.014133 0.002704
0.002396 -0.012925 0.002299 -0.004084 -0.022933 -0.030022 -
0.010061 -0.014828 0.004758 -0.013693 0.001070 0.000655 -
0.026861 -0.015938
y527 -0.002720 0.014371 0.008765 -0.004286 -0.030956
0.030339 0.016998 0.006726 -0.006216 -0.028529 -0.011538
0.007125 -0.006051 0.014586 0.009524 -0.007597 -0.025279 -
0.010331 -0.003409 0.017888 -0.005153 -0.004215 -0.012237
0.008335 0.028476 -0.017992 0.011561 -0.011721 0.016708
0.012016 0.004971 0.011426 -0.002619 0.021228 -0.017608
0.001476 0.028419
z527 0.033605 0.032345 -0.035946 0.061738 0.002387 -
0.031297 -0.021925 0.000105 -0.017094 0.031426 -0.012436

0.030929	0.010278	0.040653	0.004626	0.027384	0.043285	-
0.007857	0.009765	-0.048806	-0.002268	0.025281	0.035077	-
0.014495	0.013180	0.004653	-0.004448	-0.006673	0.008618	-
0.024418	-0.042391	-0.012708	-0.029137	0.007485	-0.003425	
0.020587	0.003386					
x528	0.023684	0.005605	-0.017514	0.009701	-0.016340	-
0.007931	-0.007524	0.001361	-0.001156	0.046168	-0.019029	
0.018211	-0.004312	-0.002474	-0.008176	0.006352	0.001803	-
0.002390	0.017261	-0.007104	0.008972	0.012516	0.003143	
0.001905	-0.011766	-0.001876	-0.004151	-0.026115	-0.031046	-
0.010669	-0.016486	0.004143	-0.019655	-0.002825	-0.008431	-
0.014344	-0.021200					
y528	0.000086	0.015516	0.008723	-0.003582	-0.025970	
0.036180	0.019959	0.003660	-0.004398	-0.019787	-0.006006	
0.012174	-0.001952	0.015926	0.007159	-0.012445	-0.019254	-
0.013282	-0.003017	0.019392	-0.004717	-0.004822	-0.006939	
0.009229	0.024734	-0.016952	0.005361	-0.012575	0.022889	
0.010053	0.001983	0.008567	-0.001142	0.029695	-0.021475	
0.011456	0.024764					
z528	0.034599	0.033774	-0.034380	0.053514	0.006097	-
0.033001	-0.017101	0.000001	-0.015870	0.034278	-0.003101	
0.036351	0.014251	0.040423	0.002345	0.020785	0.054790	-
0.009048	0.010980	-0.047042	-0.001197	0.018302	0.039961	-
0.020707	0.010018	0.012107	-0.007296	0.002045	0.007260	-
0.018683	-0.038279	-0.021807	-0.028255	0.008445	0.003077	
0.023096	-0.005152					
x529	0.028478	0.008467	-0.021006	0.014129	-0.015921	-
0.011596	-0.008532	0.002714	-0.001471	0.052149	-0.017225	
0.024607	-0.001476	0.003388	-0.008403	0.005931	0.014493	-
0.002065	0.019634	-0.010790	0.004791	0.012806	0.007434	
0.000608	-0.014925	-0.005329	-0.004640	-0.027135	-0.029229	-
0.016565	-0.021629	0.005130	-0.023210	-0.006479	-0.008243	-
0.014533	-0.023669					
y529	0.003439	0.014331	0.005756	0.000820	-0.022908	
0.035996	0.019739	0.002400	-0.004875	-0.017291	-0.010288	
0.012527	-0.001226	0.014974	0.007810	-0.010907	-0.020252	-
0.016063	-0.003669	0.018975	-0.005802	0.001724	-0.004529	
0.013000	0.022765	-0.021548	0.004594	-0.017050	0.032178	
0.005099	-0.009388	0.009839	-0.005401	0.033341	-0.020821	
0.013768	0.023435					
z529	0.032737	0.032047	-0.034619	0.051487	0.007003	-
0.041422	-0.015926	0.001314	-0.017487	0.027495	-0.006677	
0.036081	0.012108	0.037310	0.006507	0.024475	0.051180	-
0.008008	0.004366	-0.049266	-0.000504	0.016848	0.037626	-
0.023436	0.013813	0.013398	-0.005359	0.004720	0.007314	-
0.010580	-0.035827	-0.027270	-0.031512	0.007714	0.010860	
0.023305	-0.007141					
x530	0.032609	0.010662	-0.026396	0.020390	-0.016524	-
0.018471	-0.010692	0.003761	-0.003831	0.055446	-0.018718	
0.031409	-0.002258	0.007595	-0.008340	0.009047	0.020931	-
0.000161	0.019843	-0.016581	0.000420	0.011615	0.007883	
0.001877	-0.016624	-0.007006	-0.003655	-0.025922	-0.031169	-
0.016906	-0.020953	-0.004338	-0.027570	-0.010391	-0.014343	-
0.009184	-0.025006					
y530	0.007231	0.013625	0.002910	0.004067	-0.019654	
0.035953	0.020167	-0.000860	-0.005404	-0.012687	-0.010499	
0.015393	-0.000525	0.014270	0.006121	-0.012287	-0.019191	-
0.018493	-0.003290	0.018495	-0.005200	0.004581	0.001226	
0.015834	0.017243	-0.020774	0.001675	-0.016976	0.037770	

0.004250   -0.017230   0.004081 -0.008355   0.036950 -0.023013
0.019381   0.021429
z530   0.030175   0.030727 -0.033088   0.046324   0.009524 -
0.046552   -0.012666   0.002360 -0.017730   0.022876 -0.005416
0.038340   0.013627   0.033908   0.007274   0.023711   0.050842 -
0.006464   -0.000064 -0.047790 -0.002202   0.012558   0.036898 -
0.025703   0.016053   0.015828 -0.006311   0.009201   0.007287 -
0.003249   -0.029548 -0.030738 -0.030748   0.006084   0.020983
0.024592   -0.009321
x531   0.035549   0.013366 -0.029602   0.024769 -0.018375 -
0.024600   -0.011477   0.004527 -0.007009   0.058997 -0.017784
0.042054   -0.000327   0.011882 -0.007398   0.010436   0.027671
0.003673   0.018861 -0.021180 -0.004029   0.010139   0.008579 -
0.001061   -0.015985 -0.008321 -0.004667 -0.021478 -0.033759 -
0.015632   -0.019352 -0.008068 -0.027258 -0.014922 -0.014935 -
0.007012   -0.026015
y531   0.009462   0.012092   0.000372   0.010127 -0.019335
0.032344   0.019678 -0.005417 -0.008348 -0.011697 -0.013298
0.018540   -0.001453   0.013725   0.009205 -0.010844 -0.023925 -
0.019230   -0.008761   0.015304 -0.001457   0.008735   0.005023
0.013595   0.016193 -0.021843 -0.000058 -0.015775   0.041322
0.004706   -0.027117   0.004685 -0.009776   0.037444 -0.022710
0.022322   0.019468
z531   0.026796   0.028171 -0.032115   0.041343   0.013302 -
0.052509   -0.009676   0.004470 -0.017710   0.015877 -0.007302
0.039084   0.014815   0.028675   0.006887   0.024100   0.047658 -
0.004315   -0.003924 -0.045337 -0.007039   0.009620   0.034479 -
0.023161   0.018681   0.015722 -0.007356   0.012155   0.008985
0.003706   -0.021089 -0.033054 -0.032650   0.003733   0.032226
0.025569   -0.011213
x532   0.037497   0.015333 -0.033086   0.029620 -0.018222 -
0.035228   -0.011192   0.006402 -0.010231   0.059665 -0.020890
0.052385   0.001918   0.015454 -0.004313   0.014626   0.030098
0.009212   0.012547 -0.027248 -0.009691   0.009254   0.008341 -
0.004938   -0.013194 -0.011678 -0.006133 -0.018676 -0.035020 -
0.012693   -0.017818 -0.010166 -0.032714 -0.017421 -0.011005 -
0.006906   -0.024677
y532   0.012422   0.011167 -0.000436   0.012285 -0.016050
0.029397   0.022387 -0.011164 -0.009050 -0.006898 -0.010193
0.024918   0.001650   0.013744   0.009798 -0.015967 -0.021992 -
0.019800   -0.013418   0.013422   0.002575   0.009146   0.014175
0.007613   0.010651 -0.018843 -0.005509 -0.011526   0.043756
0.004732   -0.036786   0.006523 -0.012276   0.039033 -0.018342
0.024188   0.015410
z532   0.023068   0.025408 -0.031003   0.034427   0.016839 -
0.056414   -0.006560   0.006727 -0.017021   0.008984 -0.007326
0.040527   0.016821   0.021818   0.003708   0.023253   0.045476 -
0.002453   -0.004618 -0.040216 -0.014423   0.006729   0.031691 -
0.017469   0.020946   0.015136 -0.008346   0.016114   0.011440
0.009602   -0.010477 -0.035760 -0.031390   0.000312   0.044084
0.028529   -0.012868
x533   0.039683   0.018094 -0.036660   0.032493 -0.017667 -
0.045957   -0.010592   0.009197 -0.013162   0.059218 -0.023153
0.064731   0.005162   0.016655 -0.003582   0.018956   0.035148
0.015058   0.010029 -0.031729 -0.019538   0.008281   0.007773 -
0.005247   -0.008912 -0.016323 -0.008859 -0.013779 -0.034423 -
0.008772   -0.012087 -0.015374 -0.038733 -0.020028 -0.003950 -
0.004489   -0.023032
y533   0.015662   0.009863 -0.003017   0.016536 -0.013161
0.022762   0.022345 -0.016030 -0.010853 -0.005331 -0.010703

0.029219 0.003234 0.011910 0.009587 -0.016988 -0.021810 -
0.020594 -0.016207 0.010224 0.003421 0.012583 0.022600
0.004992 0.007683 -0.018242 -0.009461 -0.007228 0.046331
0.005394 -0.043496 0.008728 -0.017242 0.036838 -0.011186
0.027043 0.009364
z533 0.018101 0.021407 -0.029750 0.027437 0.019605 -
0.060192 -0.003201 0.008157 -0.016874 0.000939 -0.008419
0.041636 0.018066 0.015000 0.001581 0.021884 0.039186 -
0.000138 -0.008603 -0.033943 -0.020185 0.003419 0.027524 -
0.012940 0.022782 0.014188 -0.007907 0.019085 0.012348
0.015494 -0.001049 -0.037594 -0.028447 -0.002677 0.053632
0.030129 -0.013356
x534 0.034371 0.021409 -0.032368 0.033633 -0.016970 -
0.045881 -0.013000 0.010226 -0.011070 0.055586 -0.023110
0.059648 0.004274 0.015242 -0.003933 0.018237 0.034929
0.013708 0.008024 -0.032098 -0.023484 0.010657 0.006015 -
0.006031 -0.015443 -0.015882 -0.014252 -0.012700 -0.030274
0.003734 -0.002119 -0.018831 -0.032853 -0.010955 -0.012569 -
0.009064 -0.019113
y534 0.017053 0.009379 -0.003563 0.019145 -0.009072
0.014741 0.023730 -0.016977 -0.008792 -0.004809 -0.009440
0.031815 0.010103 0.009755 0.010515 -0.018371 -0.018429 -
0.012271 -0.023161 -0.000092 0.002360 0.016395 0.021873
0.007762 0.009255 -0.009563 -0.021330 -0.007404 0.050440
0.005221 -0.042528 0.006655 -0.016308 0.041750 0.003282
0.026853 0.001819
z534 0.006442 0.021590 -0.018291 0.017743 0.022282 -
0.050830 -0.002382 0.006239 -0.006776 -0.006635 -0.001326
0.028562 0.018724 0.010225 -0.002495 0.016566 0.035025
0.000177 -0.005861 -0.030823 -0.018249 0.003720 0.022401 -
0.003742 0.015184 0.017493 -0.009131 0.017908 0.013476
0.018686 0.003953 -0.034747 -0.020535 0.007503 0.045934
0.024263 -0.013943
x535 0.028096 0.024899 -0.026688 0.032660 -0.016642 -
0.043896 -0.015118 0.011216 -0.007326 0.051049 -0.022180
0.053437 0.004212 0.012874 -0.006387 0.016684 0.034549
0.011242 0.006388 -0.032391 -0.027062 0.012466 0.005214 -
0.005618 -0.021407 -0.014935 -0.017023 -0.011483 -0.027126
0.012877 0.004478 -0.021070 -0.029521 -0.004520 -0.021044 -
0.012857 -0.014406
y535 0.016737 0.009830 -0.002037 0.019657 -0.005490
0.009156 0.023825 -0.017612 -0.005141 -0.002487 -0.006064
0.032621 0.015882 0.007928 0.008578 -0.020854 -0.015131 -
0.006115 -0.028237 -0.008752 -0.000506 0.019425 0.021581
0.009879 0.008470 -0.001976 -0.031095 -0.006565 0.051672
0.004658 -0.040211 0.004362 -0.013886 0.043219 0.013510
0.024113 -0.005801
z535 -0.002351 0.019898 -0.010005 0.010627 0.025092 -
0.043614 -0.001115 0.004087 0.000287 -0.013759 0.003758
0.017584 0.017938 0.008283 -0.002863 0.013204 0.031119
0.002140 -0.002721 -0.026222 -0.015290 0.002655 0.015455
0.004031 0.008723 0.019875 -0.010391 0.016762 0.015750
0.022162 0.008943 -0.031113 -0.010824 0.017062 0.039935
0.020486 -0.013294
x536 0.022195 0.027690 -0.020395 0.031177 -0.015822 -
0.041394 -0.016523 0.011440 -0.003419 0.045391 -0.020851
0.046972 0.005304 0.011019 -0.009121 0.015677 0.035345
0.009356 0.006207 -0.031402 -0.029221 0.013899 0.003003 -
0.004361 -0.026746 -0.012471 -0.018219 -0.010874 -0.023064

0.021308 0.011076 -0.021609 -0.024178 0.002051 -0.026775 -
0.015551 -0.010872
y536 0.015833 0.010642 0.000351 0.019525 -0.001126
0.004453 0.024009 -0.018821 -0.000507 -0.001241 -0.002822
0.031677 0.020633 0.007132 0.006960 -0.022903 -0.010496 -
0.000516 -0.032019 -0.015340 -0.003685 0.021876 0.020670
0.010823 0.005989 0.005218 -0.038400 -0.006395 0.054175
0.005553 -0.037796 0.002665 -0.009957 0.045288 0.021714
0.023068 -0.011478
z536 -0.009267 0.016927 -0.004987 0.004519 0.025858 -
0.037361 -0.000307 0.003072 0.003960 -0.018530 0.007771
0.008807 0.015234 0.006406 -0.001849 0.011303 0.024167
0.002949 -0.000721 -0.021689 -0.012239 0.000510 0.010160
0.010995 0.006925 0.017890 -0.010802 0.016652 0.014344
0.022178 0.010997 -0.029389 -0.005403 0.021090 0.032787
0.017082 -0.013030
x537 0.016726 0.029451 -0.014748 0.029287 -0.015649 -
0.038239 -0.017301 0.011491 -0.000299 0.039199 -0.018735
0.040279 0.006137 0.008761 -0.010735 0.015727 0.034767
0.008170 0.007373 -0.030519 -0.030081 0.014984 0.000366 -
0.001763 -0.029360 -0.011540 -0.020135 -0.008971 -0.019164
0.027733 0.016288 -0.020038 -0.017966 0.007249 -0.032039 -
0.014984 -0.007326
y537 0.013917 0.010949 0.003289 0.018830 0.001756
0.001205 0.022827 -0.019552 0.003562 0.000739 0.000501
0.029447 0.022808 0.006831 0.007005 -0.023891 -0.006272
0.004832 -0.033287 -0.020813 -0.007873 0.023957 0.017903
0.011446 0.003975 0.009296 -0.045428 -0.004553 0.052403
0.007697 -0.033793 -0.000020 -0.005070 0.044746 0.025260
0.019539 -0.017880
z537 -0.013936 0.013709 -0.001296 -0.000489 0.028778 -
0.033821 0.002311 0.001014 0.006200 -0.023303 0.010113
0.001480 0.015041 0.005803 -0.002727 0.010496 0.017098
0.001460 -0.000830 -0.014592 -0.007374 -0.003042 0.006248
0.014153 0.004367 0.017609 -0.004104 0.014448 0.013665
0.019255 0.010841 -0.027793 -0.004554 0.021779 0.029220
0.014332 -0.011369
x538 0.012306 0.029718 -0.010353 0.026773 -0.014078 -
0.035288 -0.016929 0.011884 0.002200 0.032239 -0.017080
0.031693 0.009411 0.005803 -0.014787 0.015157 0.033646
0.005283 0.011046 -0.027802 -0.031897 0.015245 -0.004199
0.002900 -0.031010 -0.012546 -0.017641 -0.008984 -0.015313
0.030235 0.020153 -0.017857 -0.013405 0.007956 -0.033557 -
0.011770 -0.005868
y538 0.013350 0.009696 0.003065 0.018337 0.008383 -
0.002092 0.019620 -0.016706 0.007954 0.002183 0.001761
0.022965 0.027253 0.003593 0.000922 -0.024309 -0.000614
0.006943 -0.023711 -0.022132 -0.018343 0.028390 0.013416
0.020522 0.000799 0.006907 -0.045681 -0.002551 0.055708
0.005265 -0.030651 -0.001226 -0.006437 0.037812 0.027420
0.018230 -0.021569
z538 -0.019234 0.010775 0.006779 -0.005457 0.025491 -
0.030670 0.007073 -0.005129 0.006805 -0.027746 0.013354 -
0.000168 0.008978 0.010274 0.005836 0.012405 0.007313
0.004570 -0.013670 -0.009226 0.007320 -0.009884 0.004369
0.005539 0.006723 0.021257 -0.002852 0.013767 0.003695
0.022641 0.010447 -0.026142 0.005059 0.027489 0.027964
0.012125 -0.009832
x539 0.007250 0.029949 -0.003444 0.023313 -0.014303 -
0.032289 -0.015321 0.010114 0.003771 0.023909 -0.013804

0.024147 0.011688 0.004710 -0.017778 0.017958 0.029195
0.002983 0.010410 -0.023304 -0.026191 0.014508 -0.007575
0.003652 -0.028989 -0.011281 -0.013451 -0.008105 -0.013834
0.032854 0.024242 -0.014201 -0.007737 0.012032 -0.033522 -
0.007950 -0.001174
y539 0.008973 0.010548 0.010527 0.015806 0.008758 -
0.005890 0.020041 -0.021185 0.011373 0.004138 0.008909
0.021913 0.026795 0.006372 0.005763 -0.022031 -0.003389
0.011189 -0.031828 -0.024134 -0.013031 0.025213 0.010305
0.008466 0.001241 0.011864 -0.047643 -0.000315 0.046157
0.012786 -0.027270 -0.003073 -0.000111 0.039268 0.023750
0.014736 -0.023142
z539 -0.018129 0.004246 0.002626 -0.008048 0.030444 -
0.026598 0.006564 -0.002113 0.006479 -0.031848 0.008191 -
0.009819 0.008576 0.006218 0.001391 0.012497 0.008499
0.002572 -0.001024 -0.000656 -0.000127 -0.008806 -0.001649
0.016451 0.004432 0.013512 0.008013 0.009723 0.006861
0.010309 0.006976 -0.022321 0.003677 0.009944 0.028466
0.012529 -0.011109
x540 0.002362 0.028922 0.003889 0.018918 -0.014803 -
0.029243 -0.012181 0.007075 0.004941 0.014884 -0.010542
0.016279 0.015352 0.003820 -0.020542 0.020617 0.022605
0.000034 0.009468 -0.018681 -0.019143 0.012174 -0.013635
0.002529 -0.024965 -0.010878 -0.006555 -0.008710 -0.013849
0.034747 0.025309 -0.009444 -0.001450 0.011617 -0.031487 -
0.002786 0.001116
y540 0.006966 0.009297 0.013681 0.013731 0.013896 -
0.008918 0.017692 -0.019939 0.015052 0.004386 0.010989
0.013566 0.027770 0.004078 0.002267 -0.018464 0.000452
0.012698 -0.023526 -0.020029 -0.018138 0.026553 0.004671
0.012075 -0.000542 0.006287 -0.042525 0.001070 0.041646
0.011908 -0.023282 -0.003222 -0.000425 0.030939 0.022153
0.013562 -0.024044
z540 -0.020388 0.000049 0.004071 -0.010216 0.027402 -
0.024177 0.008511 -0.007545 0.004253 -0.032737 0.011666 -
0.008183 0.003358 0.011996 0.012217 0.013053 -0.004726
0.002987 -0.012452 0.001700 0.007667 -0.016516 -0.004174
0.002556 0.006667 0.013850 0.008040 0.010149 -0.004263
0.013450 0.001217 -0.021520 0.015264 0.004941 0.013585
0.015756 -0.013998
x541 -0.002045 0.027050 0.010590 0.014502 -0.014603 -
0.025628 -0.009035 0.004120 0.005771 0.003920 -0.006626
0.006230 0.018747 0.001804 -0.023737 0.024013 0.014488 -
0.003873 0.010655 -0.011962 -0.011429 0.010557 -0.019894
0.001462 -0.017950 -0.011701 0.003425 -0.009497 -0.012759
0.033384 0.026247 -0.003634 0.005140 0.009552 -0.024829
0.005991 0.005695
y541 0.002872 0.008506 0.020105 0.009987 0.016430 -
0.011444 0.016816 -0.021954 0.018477 0.005935 0.016139
0.007067 0.026743 0.004471 0.003707 -0.014378 -0.000534
0.013735 -0.021319 -0.015352 -0.017279 0.023853 -0.002767
0.004676 -0.001219 0.001685 -0.036886 0.002474 0.029966
0.016817 -0.019457 -0.003251 0.003511 0.022511 0.013719
0.012100 -0.023850
z541 -0.018857 -0.004561 -0.000437 -0.011073 0.028414 -
0.021554 0.009097 -0.006318 0.001234 -0.032737 0.010060 -
0.008222 -0.003531 0.014104 0.015121 0.011779 -0.007976
0.000858 -0.011767 0.010443 0.007116 -0.016921 -0.004727
0.004065 0.006730 0.010783 0.014380 0.008881 -0.002730

0.007095	-0.001792	-0.022741	0.018447	-0.002029	0.011554		
0.020685	-0.011423						
x542	-0.004247	0.022930	0.014093	0.009470	-0.012510	-
0.021318	-0.006235	0.003992	0.007070	-0.007405	-0.005555	-
0.007158	0.025326	-0.002406	-0.030913	0.026170	0.010206	-
0.008064	0.019064	-0.005916	-0.009176	0.010324	-0.027447	
0.013503	-0.012030	-0.016782	0.014707	-0.010070	-0.006122	
0.024545	0.023293	0.005493	0.007144	0.004927	-0.015609	
0.015971	0.007143					
y542	-0.001545	0.008162	0.026822	0.005232	0.019812	-
0.011453	0.014746	-0.023944	0.021972	0.007652	0.022670	-
0.000998	0.024986	0.004387	0.004172	-0.010246	-0.001353	
0.012901	-0.016480	-0.008106	-0.017045	0.020412	-0.009400	-
0.003353	-0.001584	-0.003684	-0.027460	0.003953	0.016263	
0.019183	-0.014951	-0.002603	0.006888	0.012088	0.002940	
0.009739	-0.022480					
z542	-0.014004	-0.010201	-0.008515	-0.011211	0.029810	-
0.017045	0.007450	-0.001460	-0.001332	-0.029572	0.004604	-
0.006613	-0.006672	0.016184	0.015009	0.008779	-0.004181	-
0.000112	-0.007807	0.012724	0.000685	-0.021331	0.001507	
0.017393	0.004471	0.008473	0.017650	0.012371	0.001567	-
0.003298	-0.005872	-0.021903	0.013478	-0.009232	0.008129	
0.021686	-0.013173					
x543	-0.005798	0.018832	0.012099	0.010657	-0.008399	-
0.021032	-0.000834	0.005674	0.008338	-0.016858	-0.003332	-
0.004338	0.025256	0.003963	-0.029813	0.026110	0.010329	-
0.008816	0.008241	-0.002770	-0.009972	0.002523	-0.024587	
0.010967	-0.012716	-0.007464	0.013292	-0.005401	-0.000798	
0.016293	0.013297	0.000273	0.012767	0.002368	-0.016768	
0.014454	-0.004143					
y543	-0.001311	0.004366	0.022297	0.006158	0.023480	-
0.010982	0.008788	-0.020527	0.022598	0.007138	0.018155	-
0.002448	0.023216	0.004582	0.009779	-0.002251	-0.001862	
0.011619	-0.014444	-0.010359	-0.017355	0.015698	-0.011828	
0.004359	-0.001605	-0.002136	-0.026948	0.001629	0.011379	
0.014777	-0.013728	-0.000535	0.011421	0.014109	0.004291	
0.016691	-0.018087					
z543	-0.009451	-0.014824	-0.012013	-0.014744	0.028082	-
0.009596	0.008920	-0.001850	-0.003609	-0.024743	0.007112	-
0.006682	-0.011946	0.013255	0.022963	0.000588	-0.006828	-
0.000064	-0.009674	0.011864	0.003672	-0.023025	0.004903	
0.005551	0.007714	0.006053	0.017874	0.013194	-0.003795	-
0.010660	-0.008053	-0.023254	0.020823	-0.007874	0.004889	
0.011621	-0.006013					
x544	-0.006400	0.014651	0.009507	0.010820	-0.004799	-
0.020509	0.002477	0.007743	0.008811	-0.024030	-0.001821	-
0.004732	0.025194	0.008561	-0.029225	0.025576	0.010682	-
0.009942	0.002940	0.000977	-0.012669	-0.001524	-0.021695	
0.013409	-0.013743	-0.002168	0.013885	-0.004521	0.004046	
0.007932	0.009368	-0.004472	0.014561	-0.000315	-0.017761	
0.014603	-0.014781					
y544	-0.000916	0.000526	0.018513	0.006585	0.026483	-
0.009849	0.002975	-0.017432	0.022151	0.007733	0.014153	-
0.004360	0.021653	0.003731	0.014071	0.002739	-0.001801	
0.010225	-0.010679	-0.012967	-0.017853	0.010332	-0.013648	
0.009227	-0.000202	-0.001367	-0.024605	-0.002155	0.006760	
0.009225	-0.011490	-0.000283	0.016736	0.013782	0.006484	
0.021658	-0.014603					
z544	-0.006034	-0.017411	-0.012694	-0.018823	0.024661	-
0.005121	0.008223	-0.002110	-0.007114	-0.017906	0.007412	-

0.007726	-0.015430	0.011839	0.024478	-0.004602	-0.006503	-
0.001056	-0.007383	0.015424	0.007060	-0.018010	0.010366	
0.009979	0.008622	0.004924	0.022385	0.010391	-0.008450	-
0.013479	-0.001578	-0.025817	0.016904	-0.000365	0.003662	
0.004259	0.002080					
x545	-0.005988	0.010665	0.007105	0.009442	-0.002207	-
0.019576	0.006367	0.009530	0.008635	-0.027433	0.000551	-
0.006330	0.024414	0.010926	-0.026911	0.023858	0.011638	-
0.010394	-0.000938	0.002000	-0.014484	-0.006360	-0.017413	
0.014060	-0.012977	0.001787	0.012648	-0.001400	0.005994	
0.001066	0.003126	-0.006122	0.016480	-0.003225	-0.013847	
0.012548	-0.019101					
y545	-0.000354	-0.002846	0.016136	0.006657	0.028172	-
0.009358	-0.001801	-0.013928	0.020999	0.009494	0.011444	-
0.006445	0.019996	0.002416	0.016136	0.006551	-0.000060	
0.009882	-0.006547	-0.015039	-0.017374	0.004036	-0.013252	
0.012986	0.001516	-0.003083	-0.020391	-0.003255	0.001190	
0.004974	-0.010110	0.000048	0.020880	0.013625	0.009600	
0.023772	-0.009982					
z545	-0.002085	-0.019254	-0.013652	-0.022320	0.021305	
0.000489	0.008511	-0.003213	-0.009643	-0.010048	0.009840	-
0.009053	-0.017493	0.007846	0.028274	-0.011898	-0.006292	-
0.001355	-0.003758	0.014792	0.008485	-0.017003	0.013358	
0.006546	0.010326	0.001870	0.023052	0.011902	-0.012987	-
0.015432	-0.001208	-0.023496	0.015779	-0.000988	0.007229	-
0.002391	0.010887					
x546	-0.004958	0.006919	0.004986	0.006669	0.001091	-
0.017730	0.010284	0.010291	0.007686	-0.028699	0.002234	-
0.008598	0.021058	0.013329	-0.022710	0.020963	0.010343	-
0.013667	-0.002572	0.002458	-0.012713	-0.010030	-0.011773	
0.013997	-0.012098	0.005993	0.010367	-0.000753	0.007067	-
0.005245	0.000440	-0.007244	0.014907	-0.000939	-0.014220	
0.011778	-0.015790					
y546	0.000613	-0.006011	0.014422	0.006651	0.029053	-
0.009250	-0.005500	-0.010552	0.019065	0.011486	0.008185	-
0.007884	0.016453	0.001248	0.018133	0.008422	0.000547	
0.009430	-0.002621	-0.016135	-0.015578	-0.001149	-0.011791	
0.014515	0.002262	-0.004900	-0.016543	-0.005281	-0.004476	
0.002640	-0.008926	0.000768	0.022235	0.013207	0.009006	
0.024659	-0.004136					
z546	0.001177	-0.019805	-0.013654	-0.025716	0.018238	
0.005216	0.008169	-0.004110	-0.011360	-0.002824	0.010327	-
0.007701	-0.020634	0.005681	0.029910	-0.016862	-0.007179	-
0.001369	-0.000901	0.015137	0.013130	-0.015195	0.016393	
0.006011	0.009557	0.000767	0.022524	0.010885	-0.014630	-
0.014756	0.001731	-0.021793	0.010809	0.002382	0.006533	-
0.007636	0.020420					
x547	-0.003463	0.003740	0.003173	0.002495	0.003620	-
0.014402	0.013766	0.010645	0.006861	-0.028933	0.005597	-
0.012799	0.017658	0.016691	-0.019466	0.016454	0.009086	-
0.012504	-0.001108	0.002125	-0.012176	-0.014579	-0.003437	
0.010204	-0.009426	0.004898	0.011679	0.000966	0.015145	-
0.013289	-0.002082	-0.011253	0.016939	-0.001114	-0.012293	
0.009975	-0.011364					
y547	0.000813	-0.008495	0.013544	0.007143	0.028444	-
0.010117	-0.008602	-0.007608	0.016024	0.012976	0.004922	-
0.009458	0.012093	0.000909	0.017189	0.008560	0.002106	
0.012469	0.000678	-0.018261	-0.013983	-0.006898	-0.008196	
0.014385	0.001909	-0.010037	-0.009934	-0.005929	-0.000788	

0.000116 -0.008901 -0.002688 0.024956 0.013112 0.010075
0.020043 -0.000175
z547 0.004212 -0.019269 -0.014769 -0.026989 0.015558
0.011447 0.007528 -0.006165 -0.011906 0.002746 0.012878 -
0.006643 -0.022351 0.002321 0.032398 -0.023877 -0.009183
0.001526 0.003451 0.011662 0.013545 -0.015315 0.015565 -
0.003323 0.008046 -0.002223 0.020989 0.012187 -0.007733 -
0.015646 0.000665 -0.019826 0.011760 -0.004467 0.010739 -
0.013039 0.024546
x548 -0.001556 0.000505 -0.000099 -0.002255 0.005077 -
0.011380 0.016246 0.011102 0.007079 -0.028163 0.008758 -
0.017322 0.015226 0.017691 -0.013559 0.014244 0.004316 -
0.012623 0.001098 0.001142 -0.009572 -0.018711 -0.001020
0.008787 -0.006728 0.002796 0.009380 0.002062 0.015063 -
0.016140 -0.002143 -0.012252 0.016595 0.000239 -0.017175
0.004634 -0.006931
y548 0.001642 -0.010818 0.013216 0.007511 0.026603 -
0.011403 -0.011380 -0.003680 0.012906 0.015520 0.002468 -
0.009636 0.008188 -0.000770 0.016181 0.009318 0.002816
0.015237 0.004342 -0.019540 -0.010569 -0.012578 -0.007203
0.012814 0.001704 -0.015126 -0.004847 -0.006907 -0.004904 -
0.000325 -0.008730 -0.002484 0.026492 0.010043 0.007360
0.013249 0.004196
z548 0.006547 -0.017667 -0.014882 -0.027371 0.012137
0.015092 0.005751 -0.006906 -0.011393 0.007494 0.012853 -
0.002903 -0.023498 -0.001102 0.032799 -0.025315 -0.011733
0.005607 0.006208 0.008799 0.016599 -0.013022 0.011922 -
0.005380 0.004608 -0.003575 0.019272 0.012883 -0.004649 -
0.011675 0.000788 -0.015213 0.008376 -0.009431 0.012441 -
0.016858 0.026991
x549 0.001692 -0.002572 -0.003266 -0.007478 0.008041 -
0.008608 0.018279 0.011613 0.006337 -0.023609 0.010212 -
0.021243 0.013134 0.016765 -0.006861 0.012904 0.004395 -
0.014661 0.004883 -0.002212 -0.003712 -0.023292 0.001324
0.006365 -0.002207 0.002621 0.008080 -0.001090 0.005229 -
0.022229 0.000197 -0.010235 0.012617 0.001423 -0.022681
0.004930 0.002539
y549 0.002907 -0.012941 0.013446 0.008419 0.024484 -
0.013474 -0.014075 0.000600 0.008506 0.018658 -0.000467 -
0.008261 0.004330 -0.003620 0.014527 0.009411 0.006162
0.018524 0.007423 -0.021254 -0.005376 -0.019031 -0.005670
0.008048 0.002223 -0.021238 0.002866 -0.009209 -0.013702 -
0.001468 -0.008724 -0.000903 0.025426 0.004552 0.002920
0.008774 0.008308
z549 0.008088 -0.014405 -0.013392 -0.027545 0.008222
0.017302 0.003206 -0.006829 -0.009414 0.011272 0.011327
0.003498 -0.023855 -0.003496 0.029402 -0.024125 -0.013375
0.012229 0.009295 0.006552 0.021556 -0.009625 0.008470 -
0.002515 -0.001664 -0.004312 0.018266 0.012237 0.000195 -
0.005116 0.003464 -0.011226 0.001692 -0.012810 0.015976 -
0.019215 0.027652
x550 0.004079 -0.005447 -0.006322 -0.012285 0.008858 -
0.006495 0.019544 0.012518 0.006793 -0.022701 0.008723 -
0.026662 0.006759 0.015293 -0.001894 0.011681 -0.002543 -
0.012081 0.012724 -0.004413 0.000187 -0.017968 0.005279
0.009855 0.001319 0.002928 0.003160 -0.001423 0.003334 -
0.024825 0.000671 -0.006245 0.008738 0.002475 -0.016791
0.003903 0.010702
y550 0.003611 -0.014293 0.015452 0.009126 0.021136 -
0.014762 -0.015056 0.004830 0.004864 0.020929 -0.001509 -

0.007317	0.000127	-0.005557	0.009156	0.005640	0.008448	
0.024464	0.012661	-0.021847	-0.002453	-0.023947	-0.000886	
0.002611	-0.000932	-0.027689	0.010972	-0.009224	-0.013204	-
0.000485	-0.009390	-0.004047	0.025978	-0.000045	0.002280	
0.001051	0.008862					
z550	0.008323	-0.009243	-0.011032	-0.025586	0.003828	
0.017890	-0.000234	-0.007325	-0.007079	0.013059	0.008172	
0.009907	-0.023504	-0.005158	0.024634	-0.020309	-0.017267	
0.020502	0.011929	0.003195	0.026782	-0.003662	0.002695	
0.002855	-0.011287	-0.006399	0.019290	0.011774	0.011272	
0.005261	0.005109	-0.007332	-0.007619	-0.014727	0.019790	-
0.019673	0.025144					
x551	0.007435	-0.005301	-0.006876	-0.018706	0.010144	-
0.003221	0.020171	0.012823	0.007962	-0.018699	0.010139	-
0.029259	0.004202	0.013616	0.001597	0.008768	-0.006844	-
0.005977	0.020309	-0.010065	0.007828	-0.015929	0.012341	
0.009412	-0.003121	-0.000522	0.004388	-0.004915	0.006710	-
0.024638	0.005892	-0.006781	0.005731	0.006345	-0.008052	
0.002844	0.018491					
y551	0.004399	-0.014294	0.018449	0.010129	0.016234	-
0.017178	-0.015249	0.008757	0.001023	0.022590	-0.002455	-
0.005071	-0.004006	-0.006481	0.002641	0.002648	0.011298	
0.031847	0.016232	-0.022907	0.003017	-0.028857	0.005489	-
0.004234	-0.006170	-0.035809	0.022359	-0.009135	-0.010520	
0.001014	-0.009163	-0.008933	0.024175	-0.004289	0.002537	-
0.008842	0.008121					
z551	0.008101	-0.003572	-0.008896	-0.022792	-0.000440	
0.018835	-0.004083	-0.007979	-0.004033	0.015583	0.005407	
0.016198	-0.022430	-0.007284	0.019046	-0.017013	-0.020989	
0.028375	0.015770	-0.000647	0.029999	0.000983	-0.006090	
0.005441	-0.019696	-0.007712	0.017179	0.012117	0.022487	
0.016508	0.005638	-0.000901	-0.015559	-0.019695	0.022311	-
0.021442	0.021197					
x552	0.008123	-0.006359	-0.003980	-0.026450	0.012178	
0.004001	0.020502	0.010887	0.007937	-0.010420	0.015049	-
0.029071	0.001096	0.011053	0.000181	-0.001553	-0.005369	-
0.009371	0.021294	-0.011595	0.015467	-0.018167	0.019939	
0.008829	-0.006987	-0.002751	-0.003990	-0.000426	0.011719	-
0.020258	0.008236	-0.014880	0.009320	0.002601	-0.002238	
0.005474	0.021535					
y552	0.003957	-0.014218	0.017219	0.007489	0.009431	-
0.016608	-0.016708	0.010241	-0.001517	0.021264	-0.004911	-
0.004090	-0.004382	-0.005273	0.003977	-0.000331	0.012537	
0.027512	0.017078	-0.025574	0.003594	-0.024401	0.010585	-
0.007317	-0.005564	-0.034724	0.021772	-0.007278	-0.014951	
0.000534	-0.010236	-0.004215	0.023236	-0.010377	-0.006300	-
0.001145	0.007580					
z552	0.007318	-0.002385	-0.006981	-0.022498	-0.003632	
0.017402	-0.011025	-0.009043	-0.005992	0.017195	0.000855	
0.017686	-0.021963	-0.011226	0.014214	-0.015005	-0.021465	
0.022756	0.003871	0.001958	0.028917	0.014211	-0.010871	
0.005355	-0.016919	0.003456	0.012572	0.016683	0.015591	
0.019210	0.002037	-0.008144	-0.014494	-0.021462	0.013649	-
0.013161	0.014869					
x553	0.007957	-0.008422	-0.002051	-0.030846	0.012697	
0.005741	0.016695	0.011163	0.007552	-0.006543	0.013617	-
0.030658	-0.004302	0.010232	-0.002514	-0.006176	-0.007458	-
0.009380	0.019144	-0.012395	0.024550	-0.016241	0.024696	
0.008581	-0.010826	-0.002411	-0.007504	-0.002284	0.011581	-

0.012410 0.007820 -0.026732 0.007436 0.000455 0.001756
0.009974 0.024442
y553 0.003583 -0.014338 0.016356 0.004267 0.003403 -
0.014828 -0.017681 0.011255 -0.003832 0.019551 -0.005600 -
0.002538 -0.004427 -0.003011 0.003144 -0.004414 0.013882
0.024174 0.018192 -0.025866 0.005328 -0.020178 0.015715 -
0.008830 -0.006135 -0.030134 0.020507 -0.004727 -0.019073
0.000721 -0.009877 -0.001936 0.020767 -0.014152 -0.012572
0.006319 0.008016
z553 0.006746 -0.000518 -0.005472 -0.021019 -0.006048
0.016846 -0.015337 -0.011182 -0.008336 0.019755 -0.002588
0.019672 -0.020735 -0.016301 0.012013 -0.011886 -0.020657
0.017199 -0.006438 0.002852 0.025276 0.024841 -0.016508
0.005352 -0.012359 0.010867 0.008232 0.021706 0.011099
0.019332 -0.000732 -0.009147 -0.011569 -0.024220 0.005814 -
0.008479 0.007938
x554 0.007337 -0.010435 0.000315 -0.034966 0.012051
0.008169 0.014888 0.010097 0.003490 -0.003035 0.010048 -
0.029553 -0.009802 0.006086 -0.004120 -0.009406 -0.008371 -
0.007436 0.015207 -0.012812 0.031895 -0.010196 0.025814
0.010854 -0.010441 0.000297 -0.012875 -0.000157 0.008495 -
0.008729 0.007150 -0.031478 0.002981 -0.004704 0.004549
0.012769 0.028159
y554 0.003300 -0.013856 0.015923 0.001381 -0.002580 -
0.012712 -0.018921 0.011944 -0.006421 0.017188 -0.005701 -
0.001443 -0.003861 -0.002208 0.002934 -0.008083 0.015276
0.020536 0.019228 -0.024280 0.006761 -0.015985 0.017969 -
0.010811 -0.005951 -0.025162 0.019376 -0.002029 -0.023536
0.001036 -0.009279 -0.000084 0.018162 -0.018021 -0.018353
0.010462 0.009022
z554 0.006763 0.002509 -0.005630 -0.018597 -0.006948
0.015401 -0.019594 -0.012428 -0.006079 0.022326 -0.002011
0.021595 -0.017275 -0.017343 0.009360 -0.008464 -0.020761
0.010914 -0.013402 0.003226 0.021490 0.030093 -0.019800
0.004195 -0.011484 0.014229 0.005948 0.023810 0.011936
0.020164 -0.002556 -0.011157 -0.007545 -0.022525 0.000680 -
0.004402 -0.001344
x555 0.007267 -0.010963 0.001916 -0.039641 0.011621
0.010418 0.011417 0.009225 0.001782 0.002763 0.011400 -
0.026817 -0.012372 0.003598 -0.006038 -0.012927 -0.005839 -
0.007096 0.011979 -0.012765 0.037425 -0.004130 0.027271
0.011987 -0.012597 0.003665 -0.018017 0.001335 0.008042 -
0.004255 0.006499 -0.034210 0.000450 -0.006395 0.005019
0.014024 0.027338
y555 0.002912 -0.013319 0.014940 -0.000390 -0.008030 -
0.010958 -0.020014 0.012393 -0.009115 0.014195 -0.006574 -
0.000536 -0.003834 -0.001596 0.002830 -0.010652 0.013932
0.017027 0.019242 -0.022950 0.008658 -0.012340 0.018107 -
0.012378 -0.004811 -0.021386 0.019316 0.001550 -0.025458
0.002349 -0.011200 0.001339 0.014287 -0.020906 -0.020778
0.012531 0.008331
z555 0.006712 0.005243 -0.005428 -0.014785 -0.007660
0.013280 -0.020206 -0.013641 -0.004710 0.023153 -0.004510
0.023869 -0.013564 -0.019184 0.007691 -0.003485 -0.021105
0.008168 -0.018449 0.003386 0.016896 0.032497 -0.024326
0.003220 -0.009327 0.015310 0.005649 0.024195 0.009610
0.017832 -0.002878 -0.010440 -0.004135 -0.023813 -0.002566 -
0.002653 -0.008193
x556 0.007225 -0.010969 0.003362 -0.043373 0.010733
0.010615 0.009447 0.009024 0.000191 0.008197 0.011539 -

0.023438	-0.013489	0.000502	-0.008327	-0.015636	-0.004493	-
0.007582	0.008228	-0.012903	0.042162	0.002683	0.026062	
0.011501	-0.013493	0.005520	-0.020751	0.003739	0.005963	-
0.002528	0.005357	-0.036005	-0.002252	-0.010524	0.006611	
0.016196	0.022890					
y556	0.002453	-0.012218	0.013839	-0.001610	-0.013155	-
0.010032	-0.020991	0.012681	-0.011825	0.010325	-0.007315	
0.000849	-0.003405	-0.001876	0.003279	-0.012377	0.011557	
0.014228	0.019508	-0.021570	0.010398	-0.008487	0.017838	-
0.014526	-0.004124	-0.017405	0.018844	0.005150	-0.025815	
0.003968	-0.012070	0.002506	0.010834	-0.023862	-0.021811	
0.011872	0.007019					
z556	0.006325	0.008317	-0.006639	-0.010373	-0.008416	
0.012392	-0.022077	-0.015664	-0.002314	0.021824	-0.006057	
0.027054	-0.009354	-0.020664	0.007602	0.003595	-0.018876	
0.009540	-0.022604	0.004928	0.012732	0.032944	-0.029324	
0.004624	-0.007384	0.016420	0.005756	0.019648	0.008624	
0.015976	-0.002000	-0.006889	-0.003379	-0.018250	-0.004497	-
0.007462	-0.010271					
x557	0.006843	-0.010584	0.003446	-0.045631	0.008428	
0.008893	0.006233	0.008901	-0.001440	0.012440	0.010315	-
0.018687	-0.013351	-0.003999	-0.008982	-0.015808	-0.001916	-
0.005541	0.003286	-0.012848	0.045006	0.012317	0.022347	
0.009120	-0.014009	0.007258	-0.020595	0.002056	0.002701	-
0.001150	0.002870	-0.034592	-0.004828	-0.011424	0.008023	
0.013231	0.019857					
y557	0.002049	-0.011004	0.013171	-0.002699	-0.017398	-
0.008340	-0.021591	0.012497	-0.014736	0.006209	-0.007512	
0.002654	-0.002731	-0.001875	0.003192	-0.013244	0.008368	
0.012203	0.019528	-0.019215	0.012538	-0.005428	0.016079	-
0.016025	-0.002539	-0.013435	0.019599	0.010220	-0.024756	
0.005303	-0.014555	0.004164	0.005401	-0.026478	-0.019514	
0.008730	0.003543					
z557	0.006301	0.010958	-0.007236	-0.005996	-0.007258	
0.012014	-0.021517	-0.017395	0.000341	0.020628	-0.006591	
0.029700	-0.005149	-0.019997	0.005638	0.010919	-0.017818	
0.009044	-0.024350	0.005177	0.011170	0.029803	-0.032080	
0.011746	-0.004322	0.015791	0.006319	0.017677	0.007325	
0.012500	0.004848	-0.002702	-0.004340	-0.019085	-0.008811	-
0.006348	-0.017175					
x558	0.006049	-0.009988	0.002956	-0.047398	0.005795	
0.006271	0.002750	0.008572	-0.003247	0.015146	0.008142	-
0.012698	-0.012136	-0.008544	-0.009724	-0.013140	0.000443	-
0.001924	-0.002458	-0.013374	0.049259	0.022846	0.017483	
0.008489	-0.012962	0.007282	-0.017821	-0.000211	-0.001020	-
0.001592	0.003284	-0.030715	-0.009440	-0.012645	0.007340	
0.011394	0.013554					
y558	0.001773	-0.009545	0.012537	-0.003360	-0.021228	-
0.006859	-0.021555	0.012391	-0.017750	0.002131	-0.007903	
0.004924	-0.001864	-0.002645	0.002802	-0.014302	0.004831	
0.010268	0.019659	-0.016210	0.014185	-0.002637	0.012384	-
0.018537	-0.000934	-0.009079	0.019865	0.016609	-0.023404	
0.006945	-0.018977	0.005817	-0.000096	-0.029771	-0.015411	
0.002377	-0.001737					
z558	0.006492	0.014127	-0.008412	-0.000002	-0.005435	
0.011867	-0.019351	-0.019188	0.002989	0.019952	-0.005985	
0.031829	0.000992	-0.018719	0.005412	0.015353	-0.016426	
0.008415	-0.024383	0.006412	0.001672	0.026431	-0.037447	
0.014214	-0.003217	0.015243	0.006210	0.017648	0.009892	

0.007734 0.001467 0.004958 -0.003477 -0.021459 -0.005384 -
0.011379 -0.022750
x559 0.004700 -0.009087 0.001731 -0.047850 0.002317
0.002132 -0.000692 0.008388 -0.005466 0.016671 0.004605 -
0.005380 -0.009168 -0.013738 -0.010046 -0.009422 0.003691
0.001959 -0.009104 -0.013627 0.051891 0.036323 0.010117
0.007547 -0.010440 0.008201 -0.013199 -0.002498 -0.005440 -
0.003349 0.001358 -0.020871 -0.013913 -0.016409 0.007716
0.006906 0.004926
y559 0.001908 -0.007694 0.012273 -0.003475 -0.024386 -
0.004590 -0.021970 0.011622 -0.020600 -0.002055 -0.006817
0.007065 -0.001617 -0.003654 0.002476 -0.014097 -0.000205
0.008891 0.019238 -0.011483 0.015622 -0.000315 0.008150 -
0.021920 0.000884 -0.005425 0.021065 0.024258 -0.020633
0.008293 -0.021869 0.005750 -0.007498 -0.029871 -0.009444 -
0.005524 -0.006535
z559 0.007964 0.017495 -0.008557 0.006035 -0.002397
0.011327 -0.017165 -0.019456 0.006606 0.020121 -0.002790
0.032740 0.004575 -0.015806 0.006858 0.020606 -0.015452
0.010113 -0.023374 0.007623 -0.007413 0.015799 -0.037338
0.014954 -0.002761 0.012599 0.008291 0.014246 0.011445
0.003971 0.009092 0.010806 -0.004205 -0.017579 -0.005418 -
0.015441 -0.024901
x560 0.002417 -0.006429 -0.000929 -0.047227 -0.002812 -
0.004527 -0.005203 0.008324 -0.005214 0.015506 0.000794
0.003484 -0.001963 -0.020536 -0.009576 -0.005232 0.006695
0.004368 -0.015113 -0.013009 0.054918 0.049041 -0.003223
0.005894 -0.008691 0.009389 -0.012835 -0.010859 -0.009713 -
0.004642 0.002203 -0.010269 -0.019565 -0.017585 0.009871 -
0.000082 -0.001252
y560 0.002684 -0.006805 0.012215 -0.002624 -0.025878 -
0.001948 -0.020550 0.010629 -0.025501 -0.003926 -0.005819
0.008241 -0.004891 -0.003057 0.001236 -0.012602 -0.005275
0.010049 0.017159 -0.007997 0.015292 0.004727 0.005141 -
0.024373 0.003149 -0.001175 0.027459 0.034314 -0.017797
0.012119 -0.028392 0.004566 -0.011710 -0.027586 -0.002294 -
0.013592 -0.012125
z560 0.008942 0.020929 -0.011170 0.014096 0.000628
0.009515 -0.013612 -0.020691 0.012586 0.020618 0.000773
0.032608 0.008199 -0.011181 0.007232 0.026833 -0.012674
0.011649 -0.019124 0.008459 -0.016012 0.004239 -0.041528
0.018720 -0.007250 0.011668 0.006000 0.002086 0.013891
0.005638 0.014101 0.012204 0.001890 -0.006984 -0.002184 -
0.017752 -0.020448
x561 -0.004895 0.004832 0.003440 -0.049761 -0.005392 -
0.004295 -0.002857 0.002274 -0.002995 0.025311 -0.002201
0.012234 0.008952 -0.016724 -0.008877 -0.004089 0.007567
0.008106 -0.018568 -0.019051 0.052242 0.040232 -0.011651
0.018702 -0.014748 0.011715 -0.011949 -0.019280 0.002514 -
0.005926 0.007351 -0.005120 -0.021955 -0.013313 0.009872 -
0.008491 -0.012232
y561 -0.002254 0.002030 0.014421 -0.007530 -0.028201
0.002171 -0.015702 0.006112 -0.023499 0.001202 -0.009057
0.015044 0.002197 -0.002456 0.004797 -0.008568 -0.005345
0.009674 0.010783 -0.010795 0.006629 -0.006461 0.001773 -
0.016903 0.005743 -0.012997 0.027853 0.031238 -0.024107
0.009457 -0.024128 0.000758 -0.013890 -0.025738 -0.011537 -
0.011766 -0.019907
z561 0.016468 0.016222 -0.014944 0.026971 0.007854
0.012872 -0.017224 -0.017593 0.014303 0.012493 0.015634

0.020148	0.002130	-0.011167	0.004525	0.028563	-0.012260	-
0.000190	-0.011381	0.017014	-0.018975	-0.002651	-0.035341
0.012989	0.002511	0.012226	0.007255	0.008383	0.023771
0.004500	0.021313	0.016398	0.014398	-0.007021	0.004462	-
0.014870	-0.009398
x562	-0.008843	0.011986	0.005724	-0.048621	-0.007099	-
0.004836	-0.001687	-0.000182	-0.000957	0.032602	-0.003808
0.017803	0.016642	-0.013743	-0.008287	-0.002241	0.009843
0.008758	-0.019271	-0.021953	0.049988	0.033658	-0.019373
0.027795	-0.020747	0.014895	-0.012222	-0.023890	0.010228	-
0.004201	0.010603	-0.000483	-0.024518	-0.011423	0.010481	-
0.016744	-0.018703
y562	-0.004259	0.007874	0.014860	-0.009446	-0.029548
0.005254	-0.013523	0.005058	-0.020677	0.004473	-0.011178
0.018133	0.005408	-0.002533	0.008880	-0.001634	-0.005038
0.008841	0.007889	-0.012157	0.002054	-0.009122	-0.001579	-
0.010627	0.009719	-0.020265	0.028383	0.029845	-0.030375
0.008356	-0.019540	-0.003235	-0.016591	-0.021574	-0.017222	-
0.009147	-0.021992
z562	0.026321	0.007195	-0.020354	0.041861	0.013688
0.013837	-0.023096	-0.011340	0.015828	0.003259	0.026237
0.005197	-0.006360	-0.012302	0.004824	0.032829	-0.012527	-
0.012189	-0.000344	0.024176	-0.021246	-0.002158	-0.031958	-
0.000161	0.010120	0.016033	0.006448	0.014989	0.028397
0.006404	0.021819	0.018606	0.022901	-0.009501	0.009466	-
0.012979	0.004351
x563	-0.009290	0.015809	0.005418	-0.043359	-0.007136	-
0.003135	-0.003181	-0.000992	0.001109	0.036133	-0.001235
0.018733	0.020254	-0.013113	-0.005477	-0.000305	0.010126
0.006223	-0.016783	-0.022685	0.044525	0.028255	-0.023347
0.029669	-0.025020	0.015583	-0.014529	-0.024372	0.017696	-
0.003429	0.013300	0.005862	-0.022262	-0.012010	0.013003	-
0.023471	-0.023298
y563	-0.003732	0.011737	0.014654	-0.009735	-0.029811
0.009486	-0.013685	0.004285	-0.016941	0.006415	-0.009491
0.018040	0.006381	-0.003088	0.013889	0.004973	-0.006012
0.006948	0.007095	-0.012723	-0.002124	-0.008895	-0.002885	-
0.006461	0.012370	-0.027026	0.026699	0.031150	-0.030259
0.007192	-0.015305	-0.003752	-0.015737	-0.015528	-0.019623	-
0.006620	-0.022762
z563	0.035325	-0.002308	-0.024307	0.054200	0.018704
0.015630	-0.026661	-0.007663	0.017822	-0.006314	0.035689	-
0.007169	-0.012878	-0.012986	0.005459	0.034498	-0.014210	-
0.021005	0.008865	0.029236	-0.023877	-0.002848	-0.025373	-
0.009908	0.014748	0.016155	0.003434	0.021674	0.031751
0.007266	0.020696	0.022436	0.028814	-0.010532	0.013520	-
0.010093	0.014192
x564	-0.006745	0.016751	0.001942	-0.034404	-0.006237
0.000300	-0.007082	0.000304	0.002173	0.035583	0.002883
0.015862	0.020648	-0.013746	-0.002389	0.002095	0.008459
0.003059	-0.012359	-0.019534	0.037549	0.023369	-0.028504
0.024055	-0.025997	0.018352	-0.014590	-0.023518	0.021216	-
0.004376	0.015959	0.011847	-0.016898	-0.015825	0.010931	-
0.023027	-0.025281
y564	-0.001405	0.013617	0.011942	-0.007823	-0.028577
0.013780	-0.014328	0.003440	-0.015150	0.006186	-0.007480
0.016342	0.003812	-0.003790	0.017417	0.011390	-0.008871
0.006667	0.006600	-0.010046	-0.003541	-0.007521	-0.009290	-
0.005649	0.015043	-0.027285	0.027444	0.027712	-0.027957

0.005854 -0.012113 -0.004744 -0.011046 -0.013056 -0.024307
0.003302 -0.024068
z564 0.042686 -0.011501 -0.027912 0.064620 0.022704
0.015627 -0.027850 -0.004919 0.018235 -0.015817 0.040920 -
0.016341 -0.018388 -0.012435 0.002668 0.034958 -0.015446 -
0.024871 0.013700 0.034875 -0.022518 -0.004975 -0.021839 -
0.015506 0.017133 0.019650 0.004296 0.023665 0.030426
0.007545 0.018979 0.021704 0.030629 -0.009772 0.008863 -
0.001987 0.022780
x565 -0.003110 0.015319 -0.001941 -0.025721 -0.004072
0.003506 -0.011000 0.004691 0.003401 0.034308 0.008519
0.012360 0.020124 -0.014819 -0.000140 0.003521 0.007403 -
0.004040 -0.005968 -0.016496 0.031113 0.018917 -0.029465
0.015715 -0.026987 0.022955 -0.014280 -0.021321 0.023811 -
0.002496 0.017340 0.016233 -0.012990 -0.018668 0.011009 -
0.025535 -0.028163
y565 0.000943 0.014337 0.009689 -0.007787 -0.026351
0.017566 -0.014358 0.004357 -0.012531 0.006944 -0.004144
0.014923 0.002507 -0.004537 0.021636 0.013585 -0.010410
0.004985 0.008658 -0.010605 -0.003718 -0.004919 -0.014296 -
0.007213 0.015206 -0.025340 0.024767 0.026408 -0.022741
0.006976 -0.013053 -0.004102 -0.006314 -0.007954 -0.023749
0.006055 -0.025426
z565 0.048425 -0.020118 -0.030009 0.071368 0.025852
0.013358 -0.027390 -0.001072 0.019540 -0.023650 0.045153 -
0.022690 -0.022182 -0.012134 0.000784 0.033541 -0.016170 -
0.026536 0.015772 0.037806 -0.019457 -0.004958 -0.014097 -
0.017837 0.017328 0.022518 0.003444 0.027388 0.027992
0.007309 0.015204 0.020994 0.028350 -0.005670 0.008018 -
0.001431 0.032616
x566 0.002636 0.012640 -0.007403 -0.014504 -0.001756
0.006436 -0.017429 0.010426 0.005362 0.030626 0.015145
0.006222 0.013928 -0.017658 0.001449 0.007585 0.006144 -
0.012929 -0.000614 -0.008675 0.022902 0.019538 -0.028243
0.005649 -0.021437 0.028972 -0.012576 -0.019620 0.027362 -
0.005082 0.019327 0.014841 -0.006580 -0.017824 0.009490 -
0.028356 -0.028352
y566 0.004287 0.014057 0.006324 -0.006214 -0.023543
0.020228 -0.015958 0.004596 -0.009783 0.007550 -0.000378
0.011689 -0.004407 -0.006100 0.025314 0.016825 -0.013182
0.003873 0.008963 -0.009523 -0.002386 0.003099 -0.021063 -
0.009288 0.019372 -0.021639 0.022023 0.023823 -0.011450
0.003207 -0.012810 -0.006372 0.002100 0.001851 -0.021919
0.008274 -0.024447
z566 0.053649 -0.029132 -0.031508 0.077760 0.028066
0.009156 -0.024878 0.001404 0.021522 -0.031013 0.046273 -
0.027051 -0.026389 -0.012108 -0.001189 0.030763 -0.015921 -
0.024305 0.012929 0.039459 -0.015234 -0.004300 -0.006004 -
0.016214 0.014076 0.025441 0.000885 0.031468 0.021596
0.005459 0.008638 0.018366 0.021440 -0.000970 0.004900 -
0.002627 0.043731
x567 0.010840 0.007095 -0.013979 0.000943 0.001291
0.009709 -0.025034 0.018648 0.008286 0.023552 0.023876 -
0.003142 0.006496 -0.020707 0.001304 0.011823 0.007660 -
0.021297 0.005422 0.001921 0.016171 0.015923 -0.021467 -
0.006520 -0.020237 0.034466 -0.010152 -0.013105 0.025962 -
0.004369 0.020852 0.015550 0.004055 -0.021417 0.011481 -
0.032296 -0.024118
y567 0.008393 0.012323 0.003250 -0.002379 -0.020021
0.023911 -0.017461 0.004720 -0.005931 0.007248 0.004559

0.006477	-0.010041	-0.007341	0.026103	0.017864	-0.013428	
0.003827	0.009142	-0.009708	0.002569	0.007341	-0.023225	-
0.010303	0.019260	-0.016373	0.018345	0.025160	-0.002448	
0.000059	-0.013682	-0.003249	0.015127	0.004433	-0.014383	
0.008819	-0.020619					
z567	0.058866	-0.038565	-0.032334	0.083443	0.029243	
0.004605	-0.021247	0.002951	0.024705	-0.038852	0.045393	-
0.029475	-0.030180	-0.012537	-0.004144	0.027531	-0.016428	-
0.017981	0.006464	0.041175	-0.008456	-0.002211	0.005849	-
0.010511	0.007006	0.027303	-0.003169	0.037462	0.010274	
0.003307	0.000509	0.016409	0.008967	0.004773	-0.001533	-
0.001868	0.055072					
x568	0.022822	-0.002218	-0.023190	0.019925	0.005147	
0.012725	-0.034954	0.028679	0.011176	0.011052	0.035884	-
0.014430	-0.004684	-0.024773	0.001340	0.017508	0.004923	-
0.030407	0.010248	0.019135	0.008783	0.015426	-0.009586	-
0.016427	-0.017928	0.036943	-0.007857	0.001273	0.022410	-
0.001110	0.023128	0.020190	0.005677	-0.022223	0.010808	-
0.029102	-0.010129					
y568	0.017852	0.005868	-0.001459	0.002598	-0.018762	
0.023066	-0.020753	0.007255	-0.001102	0.006050	0.015009	-
0.001951	-0.023092	-0.006840	0.031181	0.020962	-0.015062	
0.004586	0.007535	-0.001734	0.017495	0.023669	-0.037427	-
0.002236	0.018412	-0.016900	0.012886	0.031130	0.026166	
0.008796	-0.015514	0.007164	0.035407	0.021877	-0.008947	
0.016269	-0.010729					
z568	0.063085	-0.048561	-0.032005	0.086336	0.028927	-
0.000807	-0.014298	0.002758	0.028519	-0.045448	0.041607	-
0.028580	-0.033096	-0.013368	-0.006586	0.021815	-0.018982	-
0.007644	-0.002702	0.038888	0.001796	0.001894	0.016482	-
0.004329	-0.001436	0.026903	-0.009146	0.042631	-0.002118	
0.000112	-0.013661	0.013759	-0.005829	0.011997	-0.012321	-
0.000533	0.063492					
x569	0.037066	-0.015381	-0.033564	0.043309	0.009825	
0.017282	-0.043825	0.038701	0.016981	-0.002375	0.048305	-
0.026291	-0.019821	-0.031677	-0.000895	0.023834	-0.001205	-
0.035807	0.015608	0.036557	0.005580	0.017461	0.007500	-
0.028936	-0.015835	0.036770	-0.013311	0.020757	0.010033	-
0.002164	0.024668	0.024618	0.007574	-0.026755	0.003399	-
0.019139	0.006370					
y569	0.026404	-0.000595	-0.008502	0.013431	-0.012992	
0.026596	-0.023820	0.009182	0.006453	-0.001151	0.021750	-
0.008624	-0.031976	-0.010588	0.029076	0.023259	-0.021334	
0.009018	0.004129	0.003221	0.019124	0.035720	-0.027298	-
0.005724	0.013094	-0.002773	0.004518	0.039793	0.025955	
0.003216	-0.016549	0.008428	0.025939	0.024630	-0.008608	
0.022056	0.001527					
z569	0.063380	-0.058860	-0.029253	0.084234	0.027449	-
0.007771	-0.003700	-0.000350	0.030624	-0.050835	0.031611	-
0.024787	-0.031708	-0.012222	-0.009773	0.012107	-0.017022	
0.002462	-0.013307	0.032081	0.010976	0.003622	0.023444	
0.004949	-0.009689	0.028269	-0.012585	0.042247	-0.013179	-
0.002193	-0.027382	0.007737	-0.020758	0.020627	-0.023216	
0.000670	0.070493					
x570	0.057413	-0.031164	-0.034181	0.067338	0.008913	
0.015889	-0.052247	0.041095	0.017906	-0.007216	0.037998	-
0.030384	-0.026175	-0.029619	0.000838	0.013006	-0.001480	-
0.037898	0.021237	0.042748	0.008856	0.016531	0.020417	-
0.021331	-0.012525	0.045918	-0.019760	0.033186	-0.000523	-

0.008493 0.020389 0.023194 0.000243 -0.014068 -0.013853 -
0.008779 0.006484
y570 0.039392 -0.010847 -0.009241 0.023331 -0.003119
0.026599 -0.026250 0.009394 0.013850 0.003361 0.036007 -
0.006496 -0.027434 -0.009560 0.027139 0.003826 -0.013133
0.000730 0.009962 0.008967 0.024936 0.038213 -0.022240
0.000485 0.013364 0.010104 -0.003849 0.037898 0.020008
0.011243 -0.030777 0.001601 0.025065 0.028331 -0.005435
0.013257 0.000683
z570 0.052057 -0.060281 -0.010369 0.072757 0.021662 -
0.021797 0.013796 -0.019513 0.016245 -0.046908 0.010707 -
0.012254 -0.015475 0.000272 -0.011220 -0.000590 -0.019083
0.014509 -0.039763 0.007809 0.015006 -0.008314 0.033269
0.012325 0.000153 0.020660 -0.009374 0.023145 -0.037396
0.004523 -0.033889 0.004717 -0.031367 0.027643 -0.019807 -
0.014998 0.044336
x571 0.072884 -0.045083 -0.033316 0.084383 0.006685
0.010807 -0.057986 0.038642 0.016159 -0.009950 0.023514 -
0.031778 -0.031229 -0.025659 0.002949 -0.001021 0.001091 -
0.036282 0.024227 0.046362 0.012916 0.018832 0.033938 -
0.007788 -0.006260 0.041629 -0.024324 0.042910 -0.011826 -
0.015437 0.020786 0.023282 -0.006526 -0.006187 -0.027273 -
0.003017 0.003984
y571 0.047549 -0.020236 -0.006109 0.035654 0.001909
0.024133 -0.029123 0.004455 0.015080 0.002894 0.037619 -
0.005768 -0.021358 -0.008111 0.031959 -0.009841 -0.014881 -
0.006527 0.006549 0.007495 0.023565 0.038226 -0.007791 -
0.000828 0.017377 0.023992 -0.008824 0.029830 -0.002033
0.012852 -0.039713 -0.007007 0.010550 0.026846 -0.000581
0.000757 -0.011075
z571 0.036435 -0.057316 0.002382 0.052747 0.018474 -
0.032443 0.033854 -0.032470 0.004083 -0.045348 -0.001237 -
0.005506 0.003312 0.007169 -0.020821 -0.001950 -0.012111
0.025816 -0.051883 -0.012495 0.007824 -0.017853 0.033018
0.020262 0.002266 0.008784 0.000784 0.010957 -0.043406
0.004780 -0.039626 0.004305 -0.037385 0.030460 -0.013127 -
0.026543 0.030353
x572 0.082363 -0.058346 -0.031207 0.092958 0.000481
0.000548 -0.055085 0.036057 0.011550 -0.013996 0.013979 -
0.033569 -0.036719 -0.021719 0.005188 -0.005495 0.003375 -
0.030639 0.022397 0.046375 0.022785 0.024620 0.026385
0.006357 0.001604 0.027832 -0.024787 0.049349 -0.003414 -
0.011601 0.014168 0.029994 0.004767 0.010073 -0.039712 -
0.000565 0.004813
y572 0.051319 -0.028735 -0.003994 0.044590 0.004283
0.018442 -0.029106 0.001023 0.012555 -0.002548 0.033434 -
0.009319 -0.016446 -0.010918 0.031453 -0.011845 -0.015927 -
0.010077 0.003556 0.001949 0.019863 0.037070 0.000992
0.002005 0.022874 0.030580 -0.008555 0.024859 -0.013435
0.007404 -0.043369 -0.010832 -0.002820 0.025711 -0.001028 -
0.010371 -0.010726
z572 0.019584 -0.050340 0.011729 0.030909 0.014480 -
0.037998 0.052414 -0.040889 -0.004420 -0.039830 -0.000790
0.000800 0.017184 0.016525 -0.029222 -0.000372 -0.003954
0.038225 -0.058993 -0.021970 0.004549 -0.022492 0.017356
0.023328 -0.000246 -0.003152 0.011049 0.002930 -0.036151
0.013219 -0.041385 0.001569 -0.022775 0.033084 -0.008841 -
0.028135 0.015736
x573 0.083765 -0.067046 -0.028727 0.096120 -0.003131 -
0.005489 -0.047302 0.031914 0.006488 -0.020144 0.005240 -

0.032961	-0.035977	-0.017832	0.000665	-0.006378	0.003695	-
0.022832	0.016391	0.039783	0.023169	0.020096	0.029961	
0.012518	0.004050	0.022176	-0.020810	0.050789	-0.010191	-
0.014073	0.008769	0.028724	0.001542	0.018106	-0.047452	-
0.002471	0.006498					
y573	0.051388	-0.034171	-0.001306	0.047909	0.005915	
0.014184	-0.026081	-0.001915	0.010351	-0.005740	0.031987	-
0.011897	-0.010602	-0.011938	0.026599	-0.011321	-0.012614	-
0.008729	0.003297	-0.001880	0.016123	0.035464	0.004918	
0.005934	0.024526	0.033151	-0.006225	0.024065	-0.020347	
0.004201	-0.043492	-0.015837	-0.010941	0.026353	-0.004454	-
0.014874	-0.007265					
z573	0.002966	-0.040713	0.017804	0.011710	0.012101	-
0.038604	0.064712	-0.046691	-0.010281	-0.034096	-0.001926	
0.007462	0.028757	0.023338	-0.037098	0.002472	-0.002079	
0.046107	-0.065674	-0.028888	-0.001641	-0.029606	0.010988	
0.021676	-0.004774	-0.010308	0.019570	-0.007356	-0.033699	
0.017495	-0.038938	-0.000789	-0.018334	0.028326	-0.002404	-
0.030742	0.001275					
x574	0.082200	-0.073572	-0.025357	0.095281	-0.005587	-
0.009381	-0.036592	0.027797	0.002221	-0.024633	0.000964	-
0.030708	-0.034116	-0.013997	-0.005868	-0.008022	0.003911	-
0.015268	0.007355	0.032240	0.023836	0.012136	0.033603	
0.014400	0.002779	0.020199	-0.016267	0.047952	-0.018349	-
0.012977	0.000894	0.025177	0.000470	0.026603	-0.051035	-
0.004541	0.004271					
y574	0.048283	-0.037227	0.002322	0.047309	0.006393	
0.011360	-0.022173	-0.005229	0.008123	-0.004941	0.031505	-
0.011230	-0.002881	-0.011648	0.019262	-0.010874	-0.008598	-
0.006058	0.002876	-0.003419	0.011729	0.031978	0.008330	
0.008067	0.021200	0.034501	-0.003179	0.024225	-0.027519	
0.002916	-0.041595	-0.020572	-0.019009	0.025821	-0.008950	-
0.019180	-0.006846					
z574	-0.011688	-0.029059	0.021982	-0.005043	0.011401	-
0.037172	0.072442	-0.050667	-0.012358	-0.029122	-0.003959	
0.012785	0.035680	0.028381	-0.042102	0.007402	-0.005663	
0.051572	-0.070107	-0.031639	-0.004597	-0.030841	0.003794	
0.019418	-0.008395	-0.016486	0.026668	-0.018752	-0.032803	
0.024070	-0.042699	0.000462	-0.018398	0.019355	0.007373	-
0.025741	-0.005689					
x575	0.077415	-0.078887	-0.020471	0.092352	-0.006406	-
0.013213	-0.021806	0.022334	-0.002119	-0.028988	0.000107	-
0.027580	-0.030911	-0.010597	-0.015257	-0.008871	0.003232	-
0.008327	-0.007434	0.021495	0.024799	0.002334	0.037216	
0.016172	0.001860	0.017170	-0.009727	0.040167	-0.026557	-
0.011187	-0.007087	0.018314	-0.001691	0.035594	-0.046645	-
0.007140	0.004329					
y575	0.041664	-0.038016	0.007221	0.043220	0.006316	
0.008150	-0.015979	-0.011114	0.006516	-0.003393	0.029847	-
0.007898	0.005139	-0.010044	0.008769	-0.009472	-0.005960	-
0.001364	0.000617	-0.003635	0.009325	0.026209	0.007764	
0.009540	0.015100	0.031691	0.001593	0.023370	-0.034011	
0.006233	-0.038683	-0.020414	-0.027855	0.022589	-0.014336	-
0.023823	-0.008319					
z575	-0.025862	-0.014559	0.023782	-0.020677	0.011429	-
0.035697	0.074907	-0.054747	-0.014147	-0.022187	-0.004427	
0.016892	0.041666	0.032127	-0.041645	0.015757	-0.009618	
0.055776	-0.071760	-0.031046	-0.011567	-0.027590	-0.006639	
0.016292	-0.010692	-0.023182	0.033784	-0.032990	-0.027998	

0.032210 -0.043963 -0.000375 -0.020413 0.007099 0.014789 -
0.012892 -0.008488
x576 0.067558 -0.084331 -0.010792 0.086059 -0.005953 -
0.022230 -0.002958 0.013769 -0.007400 -0.034711 -0.002824 -
0.021744 -0.023341 -0.005712 -0.031376 -0.008864 0.001112 -
0.001200 -0.029835 0.002212 0.025643 -0.008305 0.035914
0.020199 0.002304 0.015313 0.001024 0.027962 -0.033335 -
0.002108 -0.019279 0.013010 -0.001836 0.032365 -0.030929 -
0.013445 -0.000994
y576 0.029210 -0.036728 0.013800 0.035596 0.005757
0.002407 -0.006660 -0.020282 0.005431 -0.002229 0.025885 -
0.002615 0.014103 -0.008253 -0.005237 -0.001609 -0.002780
0.009120 -0.007096 -0.004645 0.005772 0.015141 0.000576
0.011583 0.011235 0.022999 0.011728 0.020747 -0.037788
0.013365 -0.030820 -0.017902 -0.038932 0.016760 -0.017613 -
0.024701 -0.013889
z576 -0.041705 0.006967 0.021674 -0.043191 0.009754 -
0.032407 0.072473 -0.060930 -0.017590 -0.014049 -0.003230
0.020980 0.047605 0.035071 -0.038893 0.026458 -0.014242
0.058955 -0.066409 -0.020152 -0.027592 -0.021013 -0.019915
0.002847 -0.014368 -0.035339 0.041938 -0.041525 -0.014163
0.040871 -0.035277 0.012085 -0.017516 -0.017412 0.021521
0.004800 -0.010605
x577 0.043580 -0.082735 0.002201 0.063560 -0.006827 -
0.030070 0.029454 -0.004090 -0.021705 -0.033764 -0.013735 -
0.010457 -0.011136 -0.001614 -0.051058 -0.001900 -0.004738
0.009819 -0.043471 -0.024078 0.022291 -0.028730 0.025348
0.024828 0.000359 -0.001654 0.011481 0.004840 -0.042636 -
0.010368 -0.021472 0.020269 0.011633 0.032014 -0.000086 -
0.012588 0.005321
y577 0.007850 -0.034716 0.019993 0.021947 0.003410 -
0.004513 0.011847 -0.028120 0.003426 -0.004949 0.020499
0.001507 0.029272 -0.008749 -0.034984 0.020342 0.004112
0.026161 -0.019538 -0.013894 -0.007505 -0.006318 -0.015895
0.015282 0.016815 0.008124 0.030492 0.016631 -0.033070
0.012137 -0.007811 -0.015123 -0.040523 -0.002157 -0.019396 -
0.018724 -0.021677
z577 -0.060075 0.039544 0.001325 -0.077963 -0.000355 -
0.018008 0.071323 -0.062846 -0.022262 -0.001408 0.002039
0.023419 0.044731 0.034503 -0.030113 0.039075 -0.016907
0.068945 -0.044096 0.018289 -0.061193 -0.015516 -0.030790 -
0.029128 -0.039189 -0.063054 0.042481 -0.037362 0.002204
0.030894 -0.013222 0.032960 -0.005973 -0.040308 0.007886
0.041783 0.013691
x578 -0.026801 0.012621 -0.009806 0.020879 0.006307 -
0.006831 0.042046 0.023283 0.005247 -0.007248 -0.013336 -
0.008156 -0.062625 0.038319 -0.021774 0.025728 0.006216 -
0.050397 0.001863 0.056010 0.018402 0.052861 0.028688 -
0.057560 0.034570 0.010644 0.068257 0.026545 0.005189
0.057143 0.010687 -0.022289 0.003748 0.033748 -0.017694
0.055720 -0.015741
y578 0.011180 0.041589 -0.027601 0.025216 0.009468 -
0.017747 0.085225 0.039916 0.034129 0.019888 0.003123 -
0.014133 -0.060455 0.026303 0.011681 -0.007526 -0.017631 -
0.003900 -0.047564 -0.034819 -0.048608 0.043260 -0.011914 -
0.014874 0.029601 -0.004720 0.038672 0.006559 0.014500
0.041879 0.038991 0.020681 -0.007235 0.014638 -0.044711
0.052694 0.001086
z578 -0.000088 0.014438 0.001983 0.010021 0.004872
0.015374 0.007503 0.042002 0.053783 -0.025056 0.008008

0.002183	-0.031263	0.013298	0.003503	0.010608	0.015243	-
0.016749	-0.002332	0.031798	0.035029	0.029624	0.046370	-
0.006108	0.045009	0.025570	0.037674	-0.049474	-0.025652	
0.017110	-0.024092	-0.052025	0.002199	-0.014015	0.009066	-
0.040148	-0.042544					
x579	-0.028414	0.011427	-0.012037	0.018754	0.002049	-
0.003214	0.037410	0.020772	0.000940	-0.007517	-0.014346	-
0.018773	-0.049476	0.024373	-0.015110	0.030346	0.008571	-
0.054637	-0.000797	0.050066	0.019334	0.038361	0.036163	-
0.050734	0.040314	0.008641	0.053193	0.017406	0.022566	
0.061765	0.001569	-0.006253	-0.012921	0.029288	-0.032094	
0.044930	-0.028543					
y579	0.014016	0.036896	-0.023982	0.023064	-0.001913	-
0.015955	0.067031	0.028852	0.021264	0.020609	0.003231	
0.000950	-0.050944	0.017965	0.002049	-0.003091	-0.010189	
0.006571	-0.039755	-0.028729	-0.036380	0.030009	-0.024846	-
0.000115	0.020519	0.005447	0.016619	0.011478	-0.014708	
0.043025	0.035812	0.024940	-0.002313	-0.026210	-0.025255	
0.041276	0.011226					
z579	-0.002928	0.013615	0.006073	0.009135	0.003820	
0.017107	0.005625	0.034864	0.050886	-0.026745	0.007225	
0.002807	-0.027177	0.017707	0.004883	0.001975	0.011838	-
0.020803	-0.006692	0.034577	0.034686	0.032832	0.056526	-
0.013585	0.029848	0.024061	0.032718	-0.044658	-0.017181	-
0.009020	-0.032191	-0.045574	-0.004798	-0.015468	-0.003221	-
0.022165	-0.038732					
x580	-0.027351	0.012005	-0.014829	0.016075	-0.004519	-
0.002383	0.030327	0.013690	-0.004275	-0.006173	-0.015623	-
0.018034	-0.034649	0.015011	-0.011867	0.018875	0.010470	-
0.057279	-0.008251	0.036375	0.016041	0.035430	0.032169	-
0.056326	0.039393	0.004896	0.027608	0.012637	0.041384	
0.047368	0.000036	0.002581	-0.006352	0.009027	-0.041153	
0.047911	-0.040420					
y580	0.016560	0.034010	-0.022487	0.022254	-0.005622	-
0.011336	0.059595	0.020126	0.012588	0.017595	0.006338	
0.000597	-0.050715	0.011552	-0.003164	0.006006	-0.008345	
0.016485	-0.029700	-0.021605	-0.033104	0.010936	-0.026744	
0.010894	0.010114	0.014544	0.006915	0.021908	-0.015763	
0.053294	0.022750	0.021174	0.001377	-0.043560	-0.005146	
0.028552	0.020869					
z580	-0.008310	0.013895	0.008745	0.011425	0.000671	
0.019385	0.003055	0.030667	0.047597	-0.025854	0.005252	
0.000821	-0.022750	0.019401	0.003619	0.002800	0.009305	-
0.023145	-0.005546	0.040617	0.039075	0.028619	0.067167	-
0.012070	0.020245	0.025771	0.039609	-0.042919	-0.010004	-
0.011520	-0.034802	-0.036796	-0.021661	-0.007014	-0.004362	-
0.012296	-0.027684					
x581	-0.029441	0.010853	-0.013813	0.013783	-0.012108	-
0.003993	0.020392	0.008921	-0.013916	-0.004856	-0.017679	-
0.016341	-0.020214	0.010878	-0.017888	0.014745	0.012043	-
0.053950	-0.014508	0.030494	0.017707	0.029735	0.029500	-
0.047084	0.032679	0.009540	0.012915	0.009988	0.053613	
0.034880	0.002012	0.014804	-0.006372	-0.001506	-0.039752	
0.040846	-0.048281					
y581	0.019336	0.032595	-0.023222	0.019832	-0.008171	-
0.006115	0.053736	0.012138	0.008107	0.015604	0.006151	
0.000848	-0.051812	0.004877	-0.007347	0.009730	-0.006732	
0.022152	-0.021775	-0.017355	-0.028173	-0.004242	-0.024711	
0.017718	0.002740	0.018501	-0.004420	0.027407	-0.011597	

0.058545	0.015960	0.009554	0.004402	-0.049567	0.003702	
0.017401	0.024519					
z581	-0.010113	0.016012	0.007054	0.011783	-0.000027	
0.020917	0.006376	0.026893	0.046628	-0.024713	0.003130	
0.000809	-0.021040	0.016213	0.008385	0.003363	0.009071	-
0.029476	-0.005996	0.041888	0.039729	0.027727	0.072342	-
0.018939	0.015331	0.020653	0.040164	-0.040366	-0.002121	-
0.013355	-0.037522	-0.033203	-0.032285	-0.003338	-0.009091	
0.000686	-0.016860					
x582	-0.029643	0.009701	-0.014594	0.011402	-0.017261	-
0.005156	0.015712	0.004122	-0.019948	-0.001725	-0.019268	-
0.011124	-0.010758	0.005529	-0.021576	0.012674	0.011091	-
0.049106	-0.013616	0.028596	0.022162	0.024181	0.021675	-
0.036276	0.027526	0.010786	0.002222	0.010721	0.048992	
0.025371	-0.001854	0.024153	-0.005270	-0.008768	-0.031017	
0.035463	-0.045353					
y582	0.019430	0.031721	-0.023750	0.018053	-0.010953	
0.001374	0.045447	0.003820	0.004143	0.012621	0.005440	-
0.002202	-0.052854	0.001909	-0.015387	0.006837	-0.008954	
0.026759	-0.016489	-0.018508	-0.028063	-0.017303	-0.017095	
0.019544	-0.007817	0.022526	-0.012660	0.033139	0.021703	
0.056304	0.010855	-0.015874	0.011157	-0.044699	0.011759	-
0.002584	0.009913					
z582	-0.010694	0.018207	0.006071	0.010646	-0.001075	
0.020045	0.008175	0.023927	0.042289	-0.025474	0.001470	
0.001962	-0.017855	0.011605	0.013226	0.006504	0.009886	-
0.037049	-0.008609	0.039543	0.038664	0.030411	0.071921	-
0.026023	0.016804	0.020248	0.039296	-0.041264	0.003404	-
0.016965	-0.033819	-0.023903	-0.039348	-0.006925	-0.014052	
0.006601	-0.013310					
x583	-0.028781	0.008619	-0.015848	0.009697	-0.021728	-
0.006756	0.012484	-0.000280	-0.023836	0.000232	-0.018574	-
0.008653	-0.004344	-0.000081	-0.025804	0.011192	0.009448	-
0.041929	-0.010461	0.023517	0.021165	0.018814	0.010840	-
0.025342	0.023444	0.011150	0.000528	0.017505	0.049587	
0.019196	-0.001796	0.025155	0.006150	-0.009401	-0.025736	
0.023539	-0.044747					
y583	0.021243	0.029265	-0.024006	0.014278	-0.013240	
0.006340	0.037106	-0.001274	0.001355	0.011812	0.004473	
0.000770	-0.051604	-0.003757	-0.015769	0.008531	-0.008181	
0.026814	-0.011873	-0.013258	-0.020740	-0.023868	-0.014573	
0.023037	-0.009568	0.023911	-0.021198	0.028290	0.014752	
0.046168	0.010235	-0.022225	0.006338	-0.046527	0.010141	-
0.016219	0.005506					
z583	-0.011568	0.021540	0.004389	0.009712	-0.002049	
0.017832	0.010863	0.020220	0.036121	-0.025469	-0.000767	
0.004906	-0.014813	0.006882	0.015443	0.008947	0.008721	-
0.043544	-0.010956	0.035094	0.037167	0.031760	0.068584	-
0.032567	0.015977	0.019121	0.034444	-0.041590	0.012011	-
0.022222	-0.031793	-0.017312	-0.044417	-0.013523	-0.007037	
0.007505	-0.008853					
x584	-0.028699	0.005594	-0.020881	0.017414	-0.027177	-
0.016566	0.006617	-0.003641	-0.030031	-0.006530	-0.031800	-
0.013135	-0.010081	-0.005691	-0.021256	0.024818	-0.008313	-
0.029460	-0.017924	0.009484	0.017607	0.021185	-0.006342	-
0.013223	0.028731	0.000907	0.005092	0.016823	0.055103	
0.017099	-0.002870	0.022629	0.013116	-0.009892	-0.019698	
0.009544	-0.034139					
y584	0.023136	0.027312	-0.024069	0.008560	-0.014481	
0.012499	0.030830	-0.005101	0.000705	0.012902	0.006408	

0.004637	-0.047115	-0.009709	-0.014609	0.007782	-0.005417	
0.023126	-0.005278	-0.003437	-0.013079	-0.026307	-0.013442	
0.026030	-0.010503	0.028131	-0.026726	0.021863	-0.001304	
0.031533	0.011264	-0.025020	0.001435	-0.044439	0.001873	-
0.024827	-0.001212					
z584	-0.010632	0.027715	0.005957	-0.000099	-0.000756	
0.021195	0.017922	0.016364	0.032511	-0.016122	0.010535	
0.016228	-0.002694	0.002204	0.006218	0.000371	0.022574	-
0.051187	-0.003177	0.036591	0.037298	0.026515	0.066953	-
0.036337	0.007471	0.028175	0.025783	-0.033464	0.016955	-
0.026192	-0.022734	-0.013616	-0.038505	-0.017907	0.007534	
0.002765	-0.012771					
x585	-0.025468	0.006158	-0.024133	0.013571	-0.025906	-
0.016952	0.007184	-0.008997	-0.032425	0.002214	-0.029792	-
0.007895	-0.009247	-0.009284	-0.024168	0.027271	-0.008805	-
0.014617	-0.019751	0.003735	0.009976	0.017058	-0.020749	-
0.002959	0.031665	-0.001821	0.001695	0.023871	0.046214	
0.008698	0.002437	0.024213	0.031171	-0.005717	-0.012426	
0.001314	-0.025062					
y585	0.023292	0.025463	-0.023828	0.005568	-0.015326	
0.017009	0.024874	-0.007760	0.001925	0.010391	0.005864	
0.004262	-0.043770	-0.012519	-0.012253	0.003950	-0.008457	
0.016825	0.000479	0.005545	-0.008721	-0.025787	-0.009749	
0.024983	-0.015078	0.030105	-0.026757	0.016253	-0.001406	
0.016417	0.011716	-0.034319	-0.005380	-0.032884	-0.005031	-
0.036852	-0.011695					
z585	-0.011144	0.031103	0.005907	-0.000347	-0.003881	
0.012351	0.021496	0.016387	0.025147	-0.018576	0.007329	
0.022109	0.002098	-0.004168	0.002292	0.007621	0.022015	-
0.056220	-0.001534	0.031797	0.041749	0.026720	0.054451	-
0.032224	0.004073	0.033772	0.024249	-0.031428	0.024441	-
0.019930	-0.013468	-0.010227	-0.038219	-0.028281	0.029787	-
0.008977	-0.009941					
x586	-0.023273	0.008925	-0.026081	0.010407	-0.026110	-
0.016304	0.009920	-0.012568	-0.029826	0.009976	-0.032965	-
0.000741	-0.011020	-0.012419	-0.031717	0.028203	-0.008993	-
0.002576	-0.013881	0.005514	0.001794	0.018403	-0.035961	
0.002895	0.034215	-0.001869	-0.004122	0.029296	0.036528	
0.001621	0.006777	0.016967	0.047778	0.004045	-0.000695	-
0.008347	-0.012211					
y586	0.024347	0.021653	-0.023437	0.002317	-0.013880	
0.020896	0.019083	-0.008766	0.002108	0.009550	0.006276	
0.003740	-0.042458	-0.014728	-0.005698	0.002068	-0.011339	
0.010390	0.003468	0.013253	-0.003966	-0.025453	-0.005979	
0.024120	-0.015569	0.032689	-0.020377	0.010565	-0.009046	-
0.000718	0.016336	-0.038385	-0.014612	-0.018744	-0.019587	-
0.046291	-0.025280					
z586	-0.009837	0.033614	0.003867	0.001012	-0.005617	
0.000566	0.025601	0.016337	0.014248	-0.020263	0.006800	
0.027281	0.006237	-0.011342	0.002208	0.018660	0.017571	-
0.059228	-0.004109	0.019618	0.042663	0.023534	0.036007	-
0.025321	0.000994	0.040550	0.024849	-0.028048	0.033286	-
0.007752	-0.002413	-0.003574	-0.031649	-0.045383	0.059327	-
0.023619	-0.007178					
x587	-0.021996	0.015333	-0.031177	0.009850	-0.024425	-
0.015273	0.016569	-0.013000	-0.025800	0.023099	-0.034916	
0.006681	-0.010484	-0.017520	-0.033972	0.033766	-0.019001	-
0.005814	-0.008001	0.001838	0.000151	0.023418	-0.049304	
0.004865	0.033590	-0.000613	0.002144	0.028408	0.031279	

0.002301 0.010185 0.006540 0.060907 0.015623 0.019795 -
0.031296 0.008776
y587 0.026213 0.017611 -0.022620 0.001208 -0.012192
0.026710 0.013802 -0.005063 0.002742 0.007376 0.008434
0.004648 -0.043798 -0.012221 0.000840 -0.001975 -0.012988
0.012663 0.001925 0.020312 -0.001352 -0.020714 -0.007625
0.026620 -0.012142 0.034506 -0.011082 -0.002374 -0.018819 -
0.030026 0.025942 -0.040939 -0.026754 -0.010435 -0.046443 -
0.048789 -0.043002
z587 -0.007543 0.033988 0.000374 -0.001672 -0.008929 -
0.016944 0.027853 0.011057 -0.000959 -0.025589 -0.000994
0.029410 0.009578 -0.017762 -0.002742 0.032869 0.016549 -
0.052399 -0.006227 0.013427 0.042285 0.016411 0.014798 -
0.009733 -0.000756 0.049370 0.022584 -0.014107 0.034031
0.001951 0.011436 0.004398 -0.014806 -0.047909 0.083816 -
0.042337 -0.007860
x588 -0.017441 0.024365 -0.032922 0.004823 -0.022883 -
0.012777 0.025310 -0.006876 -0.026951 0.029939 -0.025673
0.017714 -0.015127 -0.021555 -0.022068 0.023820 0.004857 -
0.020714 -0.010515 0.009952 0.012373 0.006992 -0.038120
0.006304 0.020665 0.002267 0.002841 0.014424 0.024764 -
0.002735 0.011102 -0.003618 0.064369 0.012980 0.014610 -
0.022858 0.010147
y588 0.022004 0.011701 -0.019565 0.005709 -0.008692
0.028437 0.008401 -0.006866 0.011290 0.002203 0.005946 -
0.001457 -0.035250 -0.006744 -0.001212 0.010157 -0.022315
0.021796 0.004700 0.022224 -0.000589 -0.022142 -0.020573
0.034304 -0.008363 0.028957 -0.008562 0.006289 -0.008093 -
0.024989 0.013606 -0.036106 -0.021129 -0.003248 -0.049878 -
0.016073 -0.051403
z588 -0.011011 0.035349 0.000057 -0.005952 -0.006857 -
0.020804 0.022100 0.017427 -0.003066 -0.031142 -0.016488
0.020594 0.000397 -0.018679 0.004994 0.029914 0.026172 -
0.064099 -0.014664 0.011157 0.039769 0.013356 0.017138 -
0.003455 -0.006705 0.029516 0.031306 -0.016999 0.027170
0.009376 0.014294 0.020848 -0.022464 -0.026508 0.066686 -
0.021400 -0.008932
x589 -0.015877 0.031638 -0.033962 -0.001028 -0.020393 -
0.012898 0.029237 -0.002874 -0.026056 0.031557 -0.020762
0.021088 -0.017415 -0.018467 -0.008834 0.008638 0.020358 -
0.031560 -0.014548 0.013587 0.021999 -0.010239 -0.025626
0.012711 0.019800 -0.008498 0.005350 0.005201 0.019924 -
0.000006 0.013757 -0.014314 0.064603 0.023062 0.003489 -
0.007515 0.007312
y589 0.020813 0.009640 -0.019256 0.010033 -0.007274
0.029795 0.006358 -0.006424 0.015728 0.004047 0.006103 -
0.001202 -0.032665 -0.009892 -0.002347 0.022303 -0.024899
0.025458 0.003803 0.025130 0.000294 -0.018942 -0.033859
0.037622 -0.011362 0.035370 -0.009873 0.014465 -0.006652 -
0.023347 0.008738 -0.028396 -0.014137 -0.007084 -0.045348 -
0.000796 -0.049782
z589 -0.012537 0.036604 0.001486 -0.008984 -0.005001 -
0.020063 0.018700 0.022593 -0.004861 -0.033152 -0.025608
0.014790 -0.005190 -0.022730 0.008005 0.030985 0.034578 -
0.072876 -0.018813 0.010926 0.036629 0.011148 0.017884 -
0.000203 -0.017125 0.018504 0.038729 -0.018371 0.016276
0.012335 0.015458 0.032894 -0.026571 -0.017710 0.048440
0.000289 -0.013688
x590 -0.013182 0.036854 -0.033593 -0.005418 -0.018671 -
0.011989 0.030175 0.000974 -0.022414 0.034322 -0.016471

0.023262	-0.018562	-0.017196	0.001775	0.002604	0.030107	-
0.036973	-0.013150	0.012538	0.028861	-0.021999	-0.016103	
0.020804	0.018312	-0.020142	0.008756	0.001190	0.003049	
0.005927	0.025492	-0.012732	0.060353	0.032483	-0.009592	
0.014850	0.011946					
y590	0.018605	0.011025	-0.019874	0.013239	-0.006600	
0.029361	0.009555	-0.006422	0.017003	0.005757	0.006112	
0.000676	-0.032642	-0.013134	-0.003191	0.025294	-0.025459	
0.022819	-0.002421	0.026790	0.000546	-0.021639	-0.042232	
0.036166	-0.016931	0.040747	-0.014435	0.019640	0.005325	-
0.023406	-0.007823	-0.035753	-0.001801	-0.009883	-0.035535	
0.000586	-0.050533					
z590	-0.014704	0.038616	0.001618	-0.010956	-0.003886	-
0.020107	0.018797	0.026067	-0.008479	-0.032583	-0.032568	
0.011305	-0.009663	-0.027568	0.012296	0.025750	0.043593	-
0.083588	-0.027434	0.010862	0.032071	0.008635	0.019163	-
0.002748	-0.026474	0.015601	0.038314	-0.019262	0.013254	
0.010813	0.007335	0.032103	-0.024791	-0.012781	0.038277	
0.005328	-0.019846					
x591	-0.010760	0.041718	-0.033901	-0.007361	-0.017793	-
0.012117	0.030634	0.004512	-0.018625	0.037094	-0.013352	
0.024118	-0.020573	-0.017558	0.013209	-0.002876	0.035978	-
0.041438	-0.015639	0.012004	0.029418	-0.033043	-0.009338	
0.026238	0.013853	-0.027307	0.013197	0.000464	-0.011367	
0.009739	0.027324	-0.014866	0.056504	0.033860	-0.017140	
0.028213	0.016100					
y591	0.017337	0.014499	-0.019300	0.013628	-0.005411	
0.028478	0.014949	-0.005765	0.015915	0.007241	0.006935	
0.003241	-0.032858	-0.018220	-0.004351	0.028111	-0.025395	
0.016778	-0.006786	0.026559	0.005815	-0.026093	-0.044916	
0.032836	-0.019596	0.040643	-0.019313	0.020878	0.012915	-
0.022490	-0.014128	-0.039176	0.007857	-0.009941	-0.029479	
0.001696	-0.045863					
z591	-0.016362	0.040032	0.002481	-0.013591	-0.002903	-
0.019542	0.019885	0.028680	-0.012728	-0.030238	-0.037352	
0.009388	-0.011918	-0.032147	0.013618	0.022612	0.051512	-
0.093457	-0.033436	0.009187	0.031807	0.007475	0.022994	-
0.007845	-0.031837	0.012490	0.032706	-0.020348	0.008383	
0.007554	0.008152	0.030744	-0.022247	-0.003024	0.028725	
0.007210	-0.021215					
x592	-0.007969	0.045118	-0.032651	-0.007618	-0.017972	-
0.012031	0.030697	0.006816	-0.015186	0.040702	-0.010530	
0.024289	-0.022146	-0.020158	0.022882	-0.004012	0.036724	-
0.045138	-0.017168	0.008891	0.026353	-0.044628	-0.003725	
0.028578	0.006195	-0.034568	0.015627	0.000950	-0.020847	
0.011826	0.030175	-0.019752	0.052419	0.035748	-0.023339	
0.035481	0.022944					
y592	0.015771	0.020804	-0.019725	0.011758	-0.003361	
0.025969	0.022573	-0.003482	0.013662	0.008637	0.007610	
0.006165	-0.033708	-0.023168	-0.003825	0.028638	-0.021358	
0.007504	-0.013574	0.027756	0.013014	-0.029824	-0.045938	
0.028522	-0.017704	0.040865	-0.022658	0.020528	0.011651	-
0.021076	-0.020137	-0.037901	0.015401	-0.013589	-0.022313	
0.004428	-0.039239					
z592	-0.018185	0.041583	0.001980	-0.015511	-0.001610	-
0.019482	0.021008	0.030255	-0.016957	-0.027332	-0.040700	
0.007925	-0.012310	-0.035392	0.015699	0.017654	0.059357	-
0.101270	-0.039810	0.008192	0.031126	0.009226	0.026894	-
0.013002	-0.033217	0.013093	0.025003	-0.019351	-0.001080	

0.004118	0.007844	0.030816	-0.018482	0.002194	0.021494	
0.006473	-0.021456					
x593	-0.006044	0.047966	-0.030551	-0.007070	-0.017918	-
0.013420	0.031505	0.009806	-0.012223	0.044564	-0.006673	
0.023576	-0.021674	-0.022153	0.029559	-0.003188	0.034752	-
0.048058	-0.018738	0.007589	0.023493	-0.051602	0.001048	
0.027625	0.002652	-0.038755	0.015678	0.003787	-0.031795	
0.013184	0.032792	-0.028027	0.049047	0.033926	-0.024441	
0.035250	0.036097					
y593	0.015189	0.029172	-0.020811	0.008021	-0.002269	
0.023494	0.031151	-0.001395	0.009871	0.010217	0.007300	
0.010478	-0.037731	-0.030767	0.000027	0.026803	-0.015326	-
0.004953	-0.021125	0.024000	0.017022	-0.039617	-0.044028	
0.024210	-0.020189	0.037721	-0.022748	0.018109	0.011447	-
0.019977	-0.024945	-0.032090	0.019838	-0.015503	-0.016921	
0.008040	-0.037051					
z593	-0.018907	0.041948	0.001293	-0.016046	-0.000589	-
0.018184	0.021576	0.030560	-0.019507	-0.023663	-0.044802	
0.006275	-0.011959	-0.038914	0.017420	0.012047	0.061963	-
0.106808	-0.044528	0.004361	0.028085	0.008221	0.030030	-
0.019061	-0.037014	0.012554	0.017055	-0.015891	-0.011234	-
0.000845	0.004715	0.029003	-0.014414	0.006797	0.013788	
0.007572	-0.024958					
x594	-0.004165	0.050230	-0.027969	-0.006249	-0.018292	-
0.013894	0.032658	0.011693	-0.009209	0.050007	-0.002632	
0.022998	-0.020655	-0.027179	0.035647	-0.001256	0.030190	-
0.050904	-0.020596	0.003724	0.016978	-0.058452	0.001846	
0.019052	-0.000393	-0.040054	0.014274	0.007751	-0.042060	
0.012827	0.031411	-0.032733	0.044827	0.027034	-0.020998	
0.031758	0.046003					
y594	0.015136	0.040057	-0.021897	0.003384	-0.000465	
0.019137	0.040212	0.002327	0.004295	0.010560	0.007618	
0.013716	-0.043295	-0.036517	0.003752	0.024828	-0.008765	-
0.020075	-0.028930	0.021609	0.022008	-0.048517	-0.035256	
0.023218	-0.021377	0.032129	-0.022696	0.015327	0.008824	-
0.017581	-0.021316	-0.028601	0.023721	-0.015839	-0.014611	
0.005067	-0.026747					
z594	-0.019402	0.041513	0.001407	-0.014575	0.000396	-
0.017641	0.021109	0.030105	-0.021594	-0.020131	-0.047205	
0.002405	-0.008906	-0.040462	0.016115	0.007848	0.060289	-
0.108307	-0.047812	0.002339	0.025992	0.008855	0.035416	-
0.019838	-0.040966	0.011211	0.009806	-0.008992	-0.022887	-
0.006459	0.005829	0.019866	-0.009464	0.010853	0.006940	
0.006205	-0.022337					
x595	-0.002718	0.052682	-0.023738	-0.004945	-0.019712	-
0.014343	0.034158	0.013343	-0.007162	0.055012	0.002842	
0.022969	-0.020896	-0.033695	0.041602	0.001896	0.024950	-
0.054149	-0.023461	-0.001299	0.008231	-0.064066	0.002284	
0.009905	-0.003544	-0.039686	0.011764	0.013226	-0.051344	
0.009923	0.028485	-0.039799	0.039414	0.022782	-0.016450	
0.021862	0.058899					
y595	0.015511	0.052076	-0.024146	-0.002685	0.001994	
0.013335	0.050130	0.007052	-0.002217	0.013039	0.006557	
0.016615	-0.047296	-0.042487	0.006900	0.021559	-0.003758	-
0.036908	-0.037327	0.017745	0.026881	-0.058076	-0.025249	
0.018275	-0.021042	0.025324	-0.022668	0.011470	0.002703	-
0.012251	-0.014353	-0.021655	0.026306	-0.022739	-0.011033	
0.000268	-0.017776					
z595	-0.019391	0.039614	0.001633	-0.011323	0.001679	-
0.016503	0.020114	0.028101	-0.023248	-0.015366	-0.048930	-

0.002878	-0.001965	-0.042628	0.013632	0.003807	0.054263	-
0.105390	-0.049254	-0.000925	0.022635	0.009101	0.039302	-
0.022555	-0.044284	0.011660	0.000413	-0.000305	-0.036830	-
0.012421	0.005489	0.008937	-0.001886	0.008212	0.002285	
0.003086	-0.021277					
x596	-0.000580	0.055916	-0.019112	-0.004615	-0.022044	-
0.013427	0.035193	0.013000	-0.005236	0.060362	0.006039	
0.023467	-0.024143	-0.039953	0.043963	0.008370	0.017248	-
0.056857	-0.026763	-0.007885	-0.000947	-0.068190	0.005900	
0.001671	-0.008380	-0.038904	0.006816	0.014847	-0.058488	
0.007335	0.027536	-0.044416	0.032597	0.019410	-0.013170	
0.009617	0.073073					
y596	0.015426	0.064982	-0.025982	-0.008575	0.004443	
0.005461	0.061976	0.013222	-0.010489	0.015411	0.007620	
0.019701	-0.049566	-0.051057	0.012300	0.014353	0.001700	-
0.056953	-0.046610	0.012079	0.030586	-0.068840	-0.016953	
0.009401	-0.017341	0.017525	-0.019935	0.011469	-0.007694	-
0.008967	-0.009400	-0.016362	0.029212	-0.033419	-0.002316	-
0.010219	-0.007210					
z596	-0.019401	0.036359	0.002175	-0.006288	0.002967	-
0.015768	0.019519	0.024669	-0.025281	-0.009943	-0.047002	-
0.008066	0.008344	-0.045763	0.012996	-0.000922	0.046409	-
0.099765	-0.051148	-0.005254	0.016590	0.008595	0.039951	-
0.027377	-0.045305	0.014921	-0.010935	0.012073	-0.051941	-
0.022272	0.003856	-0.005559	0.008243	-0.000230	0.000408	-
0.005015	-0.019184					
x597	-0.000872	0.052644	-0.017735	-0.003087	-0.023469	-
0.015429	0.026217	0.011390	-0.003883	0.053449	0.010040	
0.021511	-0.013718	-0.033752	0.045834	0.007202	0.008251	-
0.046456	-0.023313	-0.008731	-0.007061	-0.063485	0.002403	
0.008940	-0.004253	-0.038705	0.002785	0.017967	-0.055546	
0.003413	0.011833	-0.044665	0.016050	0.017489	-0.011925	
0.009404	0.068876					
y597	0.013456	0.070810	-0.031993	-0.010677	0.004846	-
0.004393	0.063737	0.016473	-0.017337	0.005037	0.010566	
0.016994	-0.041853	-0.049800	0.016273	0.007728	0.000431	-
0.056177	-0.050583	0.011702	0.032486	-0.072840	-0.005253	
0.030516	-0.016404	0.004719	-0.026729	0.016779	-0.015335	-
0.009449	-0.026320	-0.013396	0.017922	-0.037281	0.004068	-
0.005324	-0.003943					
z597	-0.019574	0.025080	0.005355	-0.001681	0.007498	-
0.015666	0.012824	0.019401	-0.025683	-0.012219	-0.047919	-
0.009968	0.019587	-0.034738	0.009453	-0.006120	0.043544	-
0.090167	-0.049506	0.005494	0.015121	0.026974	0.044228	-
0.018601	-0.040650	0.009304	-0.015818	0.017230	-0.047223	-
0.026641	0.001481	-0.008856	0.014378	0.006497	-0.007207	
0.005703	-0.019617					
x598	-0.000747	0.051773	-0.016353	-0.003751	-0.024985	-
0.018632	0.018498	0.012722	-0.002940	0.046320	0.012968	
0.017499	-0.007697	-0.030425	0.044626	0.005831	0.003136	-
0.035621	-0.021441	-0.009938	-0.011500	-0.059449	-0.000219	
0.021346	-0.001160	-0.033877	0.000128	0.019979	-0.048719	
0.001110	0.000802	-0.049635	0.004719	0.014514	-0.009041	
0.005878	0.059611					
y598	0.010772	0.072017	-0.034285	-0.010954	0.006282	-
0.011471	0.062172	0.017051	-0.023379	-0.002841	0.013170	
0.016561	-0.031403	-0.045102	0.020441	0.001308	-0.004119	-
0.055542	-0.051680	0.010767	0.032117	-0.070786	0.003101	
0.041440	-0.014045	-0.006190	-0.030720	0.020283	-0.022092	-

0.011460 -0.041531 -0.007111 0.007463 -0.038787 0.006011 -
0.002077 0.001060
z598 -0.018849 0.013292 0.008054 0.002300 0.011073 -
0.013858 0.006901 0.013199 -0.024764 -0.013939 -0.047731 -
0.009851 0.030130 -0.024661 0.007862 -0.010318 0.038814 -
0.080766 -0.046365 0.015317 0.011394 0.040780 0.045596 -
0.013819 -0.035785 0.002275 -0.018777 0.020514 -0.042933 -
0.029867 -0.000213 -0.009315 0.018177 0.010988 -0.015199
0.015492 -0.016389
x599 -0.000804 0.052029 -0.015129 -0.004727 -0.026588 -
0.021229 0.012363 0.014480 -0.005074 0.040157 0.014425
0.015463 -0.003927 -0.026140 0.043243 0.003952 -0.002372 -
0.027478 -0.017616 -0.009807 -0.015254 -0.053691 -0.003674
0.026911 -0.001602 -0.028555 0.000115 0.018220 -0.050492
0.000803 -0.003412 -0.046611 -0.002612 0.011142 -0.009109 -
0.001806 0.050673
y599 0.008384 0.070033 -0.033644 -0.010782 0.008706 -
0.017729 0.058185 0.016391 -0.025770 -0.009005 0.015667
0.014209 -0.020523 -0.041132 0.022563 -0.003903 -0.007516 -
0.052314 -0.053154 0.009789 0.031548 -0.065125 0.010480
0.052791 -0.010423 -0.013125 -0.034451 0.025161 -0.021790 -
0.014008 -0.054989 -0.005970 -0.001802 -0.038518 0.009345
0.003996 0.003434
z599 -0.017491 0.001575 0.011072 0.005494 0.013875 -
0.011144 0.000938 0.006413 -0.021440 -0.015635 -0.045506 -
0.008596 0.040041 -0.015907 0.006393 -0.012946 0.033949 -
0.070958 -0.043983 0.021515 0.008391 0.050016 0.044590 -
0.006627 -0.029658 -0.001383 -0.019453 0.021744 -0.034237 -
0.030930 -0.002673 -0.010583 0.020804 0.012166 -0.020946
0.023560 -0.014309
x600 -0.000126 0.052314 -0.013539 -0.006472 -0.027142 -
0.025750 0.008447 0.016976 -0.005374 0.034526 0.015141
0.012156 -0.000609 -0.022803 0.042515 0.003976 -0.005692 -
0.020315 -0.015043 -0.009934 -0.018361 -0.047901 -0.005945
0.032865 -0.002143 -0.023054 -0.003512 0.017684 -0.049333
0.002398 -0.008968 -0.044080 -0.010406 0.010556 -0.004005
0.000450 0.044570
y600 0.005692 0.066256 -0.030633 -0.009558 0.011589 -
0.020947 0.051864 0.014619 -0.028310 -0.013041 0.017358
0.012275 -0.009993 -0.035868 0.022670 -0.009754 -0.011172 -
0.047619 -0.053735 0.010216 0.029973 -0.054073 0.015437
0.058505 -0.007394 -0.017291 -0.033222 0.027252 -0.021895 -
0.015742 -0.058052 -0.002755 -0.006037 -0.037697 0.009006
0.002802 0.000115
z600 -0.015774 -0.009564 0.013314 0.007674 0.015200 -
0.007190 -0.004680 -0.000594 -0.017292 -0.016573 -0.042989 -
0.005518 0.048094 -0.008025 0.004624 -0.015242 0.029059 -
0.062054 -0.039728 0.026083 0.004909 0.055093 0.041243 -
0.001053 -0.023011 -0.004079 -0.017613 0.020629 -0.027497 -
0.029823 -0.003064 -0.010145 0.023269 0.010346 -0.026773
0.025434 -0.012845
x601 0.000039 0.053048 -0.012342 -0.007621 -0.025845 -
0.028852 0.004257 0.019765 -0.006605 0.029849 0.015478
0.007156 0.002704 -0.020845 0.039078 0.002475 -0.009675 -
0.011581 -0.014380 -0.010938 -0.020245 -0.038745 -0.004920
0.035319 -0.003102 -0.015893 -0.009035 0.018427 -0.047305
0.005249 -0.012246 -0.037616 -0.015345 0.008112 0.001367
0.009145 0.037112
y601 0.003708 0.060419 -0.024574 -0.008089 0.014545 -
0.023839 0.044284 0.011634 -0.029128 -0.014979 0.017691

0.011273 0.000184 -0.028813 0.023515 -0.014152 -0.012705 -
0.043726 -0.052216 0.012923 0.027309 -0.040933 0.016372
0.060752 -0.003532 -0.019183 -0.027676 0.026110 -0.020082 -
0.016879 -0.054218 -0.001631 -0.007907 -0.033573 0.006924 -
0.003079 -0.004532
z601 -0.013379 -0.019957 0.014848 0.008316 0.015218 -
0.003269 -0.008330 -0.006827 -0.011531 -0.016870 -0.040112 -
0.000410 0.054268 -0.001108 0.003239 -0.016418 0.025531 -
0.055063 -0.034174 0.029087 0.001223 0.055192 0.035624
0.003957 -0.017690 -0.005929 -0.013820 0.016857 -0.021316 -
0.026163 -0.002740 -0.009707 0.024715 0.007838 -0.031021
0.023149 -0.010138
x602 -0.000835 0.053701 -0.010771 -0.006787 -0.026205 -
0.033439 -0.001302 0.023892 -0.009526 0.023005 0.011472
0.003037 0.002583 -0.019371 0.034365 0.001380 -0.014742 -
0.007271 -0.013831 -0.011373 -0.018968 -0.028869 -0.005709
0.037991 -0.000756 -0.011003 -0.011779 0.019057 -0.049448
0.005002 -0.012582 -0.031297 -0.016232 0.006467 0.002674
0.010860 0.027112
y602 0.002595 0.052540 -0.015677 -0.007147 0.020088 -
0.023816 0.035728 0.006874 -0.027311 -0.012382 0.019486
0.009184 0.011814 -0.020598 0.023674 -0.019139 -0.013159 -
0.036092 -0.050673 0.016240 0.021950 -0.023387 0.017483
0.058027 -0.001891 -0.015383 -0.020809 0.022849 -0.012916 -
0.013992 -0.044631 0.002172 -0.008841 -0.026831 0.004538 -
0.003276 -0.008953
z602 -0.010214 -0.029879 0.014960 0.007950 0.014498
0.000730 -0.009472 -0.012426 -0.004534 -0.017312 -0.037919
0.005188 0.057901 0.003759 0.002711 -0.015011 0.021990 -
0.049486 -0.028724 0.029763 -0.002316 0.050989 0.028106
0.007509 -0.013429 -0.006739 -0.010107 0.010816 -0.016375 -
0.019990 -0.003563 -0.007511 0.024995 0.003640 -0.032660
0.020558 -0.004821
x603 -0.001542 0.054007 -0.005978 -0.005937 -0.025968 -
0.035491 -0.008912 0.025208 -0.013568 0.019898 0.009001
0.000262 0.007523 -0.014156 0.027103 -0.005882 -0.019166 -
0.005175 -0.013244 -0.009752 -0.023353 -0.015019 -0.004947
0.036475 0.001006 -0.010197 -0.017766 0.019898 -0.043206
0.008319 -0.003393 -0.028564 -0.018128 0.005814 -0.006388
0.009158 0.014851
y603 0.001260 0.042828 -0.005439 -0.005380 0.026629 -
0.023654 0.026699 0.003071 -0.023550 -0.009498 0.019166
0.005802 0.019862 -0.014194 0.024092 -0.019551 -0.013130 -
0.025559 -0.048818 0.018770 0.020968 -0.002994 0.016411
0.052617 0.001404 -0.005498 -0.006596 0.017470 -0.007997 -
0.013011 -0.035070 0.010258 -0.005934 -0.019394 0.006847 -
0.001574 -0.011266
z603 -0.006348 -0.038942 0.013854 0.005543 0.012879
0.005284 -0.007191 -0.018227 0.004369 -0.016993 -0.034139
0.012980 0.059516 0.006604 0.000854 -0.012292 0.021178 -
0.047105 -0.021463 0.029396 -0.007474 0.042526 0.019660
0.009437 -0.013658 -0.006312 -0.008501 0.004242 -0.011719 -
0.013205 -0.004349 -0.004747 0.026064 0.000960 -0.031829
0.016057 0.001753
x604 -0.003004 0.052656 0.001818 -0.004399 -0.024006 -
0.037712 -0.018252 0.025655 -0.016079 0.016673 0.007165 -
0.001378 0.009681 -0.007427 0.020467 -0.011410 -0.021354 -
0.000166 -0.011930 -0.005279 -0.025876 0.003557 -0.002551
0.032554 0.005452 -0.006793 -0.010926 0.013162 -0.039371

0.006609 0.007087 -0.022052 -0.015009 0.010306 -0.015850
0.011474 0.004679
y604 0.000314 0.031646 0.005784 -0.004193 0.033441 -
0.021857 0.018134 -0.001213 -0.019192 -0.004292 0.016422
0.002265 0.030143 -0.007413 0.023036 -0.021650 -0.013982 -
0.016371 -0.047017 0.018806 0.017550 0.019440 0.011454
0.043914 0.003877 0.007376 0.001613 0.014335 0.002074 -
0.005684 -0.019134 0.018438 -0.003569 -0.013847 0.006338 -
0.005102 -0.018302
z604 -0.001557 -0.047478 0.011712 0.001421 0.010274
0.010303 -0.002502 -0.023792 0.014397 -0.016015 -0.030055
0.022749 0.058896 0.007653 -0.000806 -0.007373 0.021669 -
0.047486 -0.012989 0.029065 -0.012960 0.030677 0.009171
0.009408 -0.015763 -0.006151 -0.006774 -0.005186 -0.010110 -
0.004424 -0.004217 -0.000547 0.027175 -0.002851 -0.029304
0.006470 0.012022
x605 -0.004908 0.048980 0.009511 -0.002534 -0.020853 -
0.036496 -0.030042 0.023021 -0.019849 0.016559 0.004207 -
0.005282 0.011288 0.000716 0.011908 -0.020206 -0.025529
0.010337 -0.010894 -0.002815 -0.024488 0.025397 -0.003545
0.024418 0.009866 -0.000410 -0.001548 0.007962 -0.026639
0.007827 0.024447 -0.019156 -0.009806 0.014668 -0.028407
0.011783 -0.009401
y605 0.000589 0.019703 0.019461 -0.003793 0.039596 -
0.021609 0.010928 -0.003666 -0.013350 0.000067 0.013489
0.002288 0.039305 -0.001022 0.022085 -0.020436 -0.011751 -
0.013991 -0.042390 0.018685 0.011181 0.041974 0.006551
0.033756 0.003627 0.022379 0.011591 0.007994 0.006526 -
0.000652 -0.003634 0.025956 0.003729 -0.006511 0.007416 -
0.012298 -0.021828
z605 0.003694 -0.056450 0.008911 -0.004277 0.007275
0.016702 0.002654 -0.029667 0.026285 -0.014331 -0.025610
0.033137 0.056104 0.007691 -0.002831 -0.000417 0.022636 -
0.047199 -0.003661 0.025305 -0.016556 0.012863 -0.002297
0.007724 -0.018309 -0.005276 -0.002154 -0.016636 -0.005387
0.007481 -0.000515 0.004332 0.028829 -0.006917 -0.024808 -
0.009185 0.021927
x606 -0.004190 0.042937 0.008157 -0.002956 -0.019635 -
0.032683 -0.031643 0.019447 -0.020027 0.008820 0.008672
0.003349 0.016299 0.000054 0.014510 -0.015935 -0.024479
0.011931 -0.014458 -0.000062 -0.021575 0.023070 -0.014870
0.018848 0.015480 0.007180 -0.000378 0.000687 -0.017085
0.005283 0.019339 -0.010621 -0.002499 0.013424 -0.030137
0.005827 -0.005771
y606 0.002473 0.005605 0.021205 -0.004465 0.038306 -
0.014960 0.008042 -0.004188 -0.002915 0.005472 0.007619
0.007577 0.041772 -0.000595 0.019279 -0.014179 -0.001333 -
0.011487 -0.036527 0.015434 0.005005 0.025822 0.004836
0.020658 0.000710 0.029858 0.005158 0.001793 0.011646
0.003059 0.002109 0.019499 0.005533 0.013306 0.008638 -
0.022963 -0.013559
z606 0.003721 -0.060372 0.002719 -0.004432 0.002943
0.026622 0.004247 -0.036500 0.030172 -0.016157 -0.025838
0.023353 0.039157 0.011151 -0.013206 0.003016 0.025563 -
0.044167 0.014609 0.032021 -0.020509 0.008302 -0.004060 -
0.006464 -0.019676 -0.025139 0.000483 -0.016213 -0.013207 -
0.005096 0.008299 0.005751 0.017791 -0.005542 -0.027729 -
0.000220 0.036269
x607 -0.003603 0.035043 0.009143 -0.004057 -0.019766 -
0.029016 -0.031801 0.015124 -0.015870 0.001215 0.009026

0.011258 0.021543 0.001551 0.012203 -0.008278 -0.019059
0.012047 -0.017526 0.001209 -0.018783 0.018198 -0.017061
0.017237 0.020247 0.012603 0.000494 -0.006232 -0.003407
0.004186 0.015314 -0.009752 -0.000125 0.025269 -0.021660 -
0.000241 -0.006483
y607 0.004882 -0.005645 0.020276 -0.004441 0.038090 -
0.008795 0.005086 -0.005865 0.003482 0.010174 0.005966
0.010002 0.040692 -0.001464 0.019805 -0.010679 0.002587 -
0.009322 -0.028613 0.012259 -0.000145 0.014158 -0.000731
0.004367 -0.001721 0.034017 -0.001756 -0.002071 0.011162
0.004540 0.007755 0.020188 0.011331 0.017519 0.002730 -
0.033334 -0.001836
z607 0.003952 -0.063274 -0.002154 -0.004877 -0.000597
0.034813 0.005784 -0.042329 0.034096 -0.017637 -0.026869
0.015560 0.024076 0.013477 -0.022013 0.006323 0.028432 -
0.039904 0.028512 0.033479 -0.022398 0.001656 -0.002887 -
0.019163 -0.020220 -0.043464 0.001817 -0.015397 -0.015115 -
0.013714 0.015857 0.007133 0.010011 -0.002241 -0.025669
0.004058 0.044046
x608 -0.002628 0.026710 0.010197 -0.006301 -0.019126 -
0.024307 -0.030452 0.009763 -0.012494 -0.003251 0.011345
0.017694 0.025124 0.004373 0.008235 -0.004371 -0.012970
0.011166 -0.019615 0.002152 -0.017026 0.012708 -0.023304
0.014258 0.023992 0.020891 -0.001064 -0.008391 0.005709
0.005806 0.010731 -0.010626 0.004960 0.031796 -0.024211 -
0.002431 -0.000210
y608 0.006708 -0.015015 0.018167 -0.002734 0.036488 -
0.004212 0.001496 -0.007190 0.009373 0.012018 0.002165
0.009937 0.037624 -0.003581 0.021355 -0.003854 0.004693 -
0.006939 -0.020354 0.008248 -0.003302 0.006055 -0.001986 -
0.008953 -0.003353 0.031669 -0.006620 -0.008142 0.012117
0.003106 0.013654 0.024379 0.013534 0.022411 0.007365 -
0.039866 0.002170
z608 0.004576 -0.063553 -0.005368 -0.006461 -0.002944
0.042525 0.007967 -0.047321 0.036592 -0.016102 -0.023889
0.011171 0.012115 0.015786 -0.030953 0.006051 0.032882 -
0.035755 0.040024 0.034600 -0.022883 -0.003923 -0.001564 -
0.028501 -0.021796 -0.054240 0.001972 -0.011877 -0.016261 -
0.018199 0.020468 0.008848 0.007627 -0.000563 -0.025349
0.008611 0.048467
x609 -0.001524 0.017624 0.010358 -0.005572 -0.017502 -
0.018245 -0.030108 0.004739 -0.008203 -0.008196 0.012482
0.018631 0.024022 0.006551 0.006630 0.000654 -0.014366
0.009326 -0.019762 0.000944 -0.018513 0.007394 -0.028638
0.006302 0.027534 0.025993 -0.002853 -0.014488 0.012101
0.005369 0.006782 -0.008526 0.007325 0.033351 -0.020568 -
0.005730 -0.000642
y609 0.007896 -0.021935 0.017059 -0.004138 0.033221 -
0.001851 0.000011 -0.008808 0.013691 0.014670 -0.000858
0.012610 0.035806 -0.003807 0.017390 0.001924 0.013713 -
0.004168 -0.012958 0.006041 -0.001815 0.001275 -0.001266 -
0.014064 -0.007049 0.028753 -0.011796 -0.009867 0.012105
0.003864 0.020264 0.025637 0.012946 0.030601 0.007819 -
0.040750 0.008071
z609 0.004573 -0.062380 -0.007616 -0.005544 -0.005391
0.046255 0.008701 -0.050193 0.037242 -0.015805 -0.023585
0.007056 -0.001008 0.017577 -0.037396 0.008768 0.031183 -
0.030441 0.046099 0.032518 -0.021383 -0.006301 -0.000364 -
0.032333 -0.021961 -0.062367 0.003098 -0.011194 -0.014211 -

0.019328   0.022804   0.011661   0.003581   0.002500   -0.022249
0.010799   0.046213
x610   -0.000535   0.006857   0.010475   -0.005407   -0.016314   -
0.011695   -0.026473   -0.002320   -0.002196   -0.011807   0.012138
0.019063   0.021608   0.007235   0.004230   0.008204   -0.009721
0.005752   -0.016326   0.003285   -0.017602   0.004870   -0.031444   -
0.000634   0.030220   0.025670   -0.005783   -0.019090   0.011423
0.001818   0.006810   -0.004888   0.009152   0.035752   -0.016727   -
0.009693   0.000065
y610   0.009081   -0.026024   0.015162   -0.004504   0.029802   -
0.000622   -0.003581   -0.010078   0.015699   0.016259   -0.002535
0.012318   0.031874   -0.002533   0.014333   0.004534   0.015919   -
0.000045   -0.007271   0.000184   -0.001549   -0.003635   -0.000732   -
0.018895   -0.010613   0.025178   -0.015720   -0.012123   0.015592
0.006604   0.023980   0.026119   0.010957   0.034181   0.007646   -
0.036629   0.010167
z610   0.004943   -0.059187   -0.008721   -0.005209   -0.007121
0.048357   0.010582   -0.051714   0.036751   -0.013775   -0.021913
0.006057   -0.012397   0.017606   -0.041264   0.010431   0.029852   -
0.024893   0.049337   0.029892   -0.017705   -0.006000   0.001534   -
0.033145   -0.021458   -0.066600   0.003319   -0.009021   -0.013724   -
0.018452   0.023494   0.016417   0.001837   0.005400   -0.017280
0.011652   0.040762
x611   0.001987   -0.002471   0.009741   -0.006142   -0.014611   -
0.004080   -0.022328   -0.008620   0.005374   -0.011726   0.012852
0.015610   0.017582   0.008060   0.002556   0.014177   -0.005690
0.001864   -0.011319   0.003300   -0.019287   0.002576   -0.030265   -
0.009360   0.031336   0.025730   -0.008846   -0.020804   0.011040
0.000009   0.009394   -0.002029   0.007947   0.034958   -0.011023   -
0.005808   -0.001590
y611   0.008115   -0.029357   0.014659   -0.004414   0.025054   -
0.002372   -0.006345   -0.011066   0.015797   0.014350   -0.006508
0.012974   0.025513   0.000729   0.005706   0.010466   0.018174
0.003932   -0.002463   -0.002853   0.004976   -0.004822   0.000437   -
0.015210   -0.013742   0.018004   -0.020313   -0.015083   0.015723
0.008650   0.026691   0.025840   0.006884   0.044379   0.006573   -
0.034480   0.012082
z611   0.005874   -0.053084   -0.008340   -0.005605   -0.008238
0.048981   0.012507   -0.051905   0.035590   -0.009241   -0.018208
0.007248   -0.021511   0.017264   -0.044443   0.010845   0.028603   -
0.018837   0.049076   0.025438   -0.012782   -0.004486   0.005140   -
0.029780   -0.022301   -0.065613   0.002425   -0.005300   -0.009488   -
0.013090   0.023403   0.021125   0.000603   0.008447   -0.010917
0.013690   0.029410
x612   0.003482   -0.010284   0.008786   -0.008393   -0.011915
0.004702   -0.017146   -0.015313   0.012394   -0.011342   0.015099
0.011011   0.013613   0.008802   -0.004207   0.017368   -0.000126   -
0.002064   -0.001050   0.003433   -0.020448   0.001739   -0.028058   -
0.014920   0.031549   0.021473   -0.015088   -0.023143   0.009252   -
0.003636   0.013375   0.000832   0.005625   0.038923   -0.007674   -
0.001773   -0.002927
y612   0.008347   -0.032401   0.013061   -0.001950   0.018641   -
0.004969   -0.008411   -0.012853   0.015876   0.012184   -0.011089
0.011319   0.016305   0.003921   0.000841   0.015978   0.017667
0.007427   -0.000414   -0.008162   0.011356   -0.004542   0.001229   -
0.017210   -0.015127   0.010147   -0.022043   -0.015660   0.013820
0.009994   0.027280   0.026561   0.002153   0.042869   0.010461   -
0.027495   0.011781
z612   0.006870   -0.044819   -0.007227   -0.005281   -0.008486
0.048057   0.014314   -0.051377   0.032405   -0.004423   -0.013303

0.010290	-0.029311	0.015852	-0.045785	0.009830	0.025187	-
0.011946	0.045562	0.018575	-0.007256	-0.001291	0.008688	-
0.023634	-0.022421	-0.062376	-0.000613	-0.000616	-0.001300	-
0.005861	0.020008	0.026716	0.001181	0.011057	-0.003611	
0.015958	0.012968					
x613	0.005764	-0.018885	0.007711	-0.008642	-0.009392	
0.010579	-0.012435	-0.019825	0.020696	-0.011567	0.013960	
0.001930	0.005518	0.006181	-0.010473	0.025205	0.004117	-
0.006683	0.006805	0.002670	-0.020876	0.001178	-0.025767	-
0.019720	0.035488	0.011700	-0.018070	-0.021431	0.006113	-
0.007845	0.014142	0.002808	-0.001641	0.034171	-0.003591	
0.008414	-0.004639					
y613	0.008024	-0.033219	0.011302	-0.000813	0.011926	-
0.004858	-0.008683	-0.016975	0.014427	0.010801	-0.011768	
0.010506	0.008111	0.010251	-0.005158	0.016029	0.017105	
0.010198	0.005286	-0.013553	0.017673	-0.003324	0.005445	-
0.017670	-0.019741	0.001023	-0.028511	-0.016905	0.011792	
0.010011	0.029740	0.030616	-0.000109	0.043915	0.014153	-
0.019107	0.009274					
z613	0.008001	-0.035015	-0.005754	-0.002425	-0.009316	
0.042481	0.014557	-0.048677	0.027540	-0.000885	-0.011547	
0.013201	-0.037626	0.012470	-0.043943	0.011998	0.017393	-
0.004374	0.035742	0.008204	0.000226	0.004764	0.009896	-
0.013953	-0.019569	-0.057520	-0.002438	0.003876	0.008656	
0.005479	0.012844	0.033037	0.001823	0.010336	0.005602	
0.017999	-0.007447					
x614	0.007399	-0.027110	0.005268	-0.008113	-0.006846	
0.017462	-0.006793	-0.025266	0.027516	-0.012622	0.009673	-
0.008278	-0.006402	0.004047	-0.013550	0.030702	0.003922	-
0.008054	0.013100	-0.002948	-0.019373	0.005676	-0.024780	-
0.028278	0.036820	-0.004023	-0.023385	-0.018256	0.004853	-
0.013162	0.014634	0.008458	-0.005579	0.026951	0.001710	
0.021590	-0.011031					
y614	0.008501	-0.033438	0.009215	0.001446	0.004921	-
0.005136	-0.008177	-0.020340	0.013969	0.008348	-0.009867	
0.006883	0.000318	0.015297	-0.012247	0.016028	0.017149	
0.010170	0.013504	-0.016870	0.021690	-0.003382	0.008026	-
0.019443	-0.021324	-0.009907	-0.031336	-0.018406	0.002571	
0.009253	0.028503	0.033845	-0.003295	0.035304	0.017905	-
0.006647	0.008290					
z614	0.009182	-0.022529	-0.003044	-0.000126	-0.009521	
0.036479	0.014890	-0.045798	0.021208	0.004236	-0.007351	
0.018842	-0.044036	0.009836	-0.041638	0.012010	0.009143	
0.004236	0.023288	-0.003342	0.009349	0.012086	0.014281	-
0.001432	-0.018727	-0.047045	-0.006730	0.010406	0.024921	
0.019706	0.005239	0.040403	0.005501	0.012619	0.017892	
0.018321	-0.033889					
x615	0.008110	-0.030538	0.004792	-0.011011	-0.004373	
0.022488	-0.002526	-0.025379	0.032815	-0.013007	0.007771	-
0.011488	-0.014450	0.002885	-0.016882	0.033878	0.010785	-
0.010244	0.018111	-0.005669	-0.011836	0.010358	-0.017365	-
0.028755	0.034509	-0.004867	-0.023528	-0.012007	0.001433	-
0.009150	0.015007	0.003086	-0.001785	0.031530	-0.000211	
0.032551	-0.016146					
y615	0.009214	-0.029577	0.007716	0.004020	0.000794	-
0.002711	-0.006409	-0.017455	0.010475	0.002367	-0.013363	
0.006041	-0.000237	0.018442	-0.015647	0.015348	0.019356	
0.006670	0.014376	-0.019556	0.021827	-0.010691	0.008823	-
0.021553	-0.021028	-0.014381	-0.024769	-0.015763	0.008372	

0.008972	0.022553	0.034042	0.001906	0.033755	0.020882	-
0.006662	0.001840					
z615	0.008485	-0.016546	-0.004339	-0.004097	-0.008650	
0.025767	0.013408	-0.037582	0.017504	0.009006	-0.010144	
0.019854	-0.033431	0.005068	-0.034451	0.005478	0.003778	-
0.000565	0.019817	-0.000046	0.006066	0.012355	0.009746	-
0.004918	-0.012337	-0.039130	0.002314	0.011571	0.018025	
0.024397	-0.000690	0.024915	0.008600	0.016331	0.011507	
0.016872	-0.027742					
x616	0.009273	-0.032666	0.004905	-0.014730	-0.001440	
0.024943	0.003468	-0.025011	0.036438	-0.013877	0.005306	-
0.013567	-0.019103	0.000506	-0.023139	0.034903	0.015308	-
0.011229	0.019543	-0.006309	-0.003970	0.008406	-0.010964	-
0.029894	0.032358	-0.006681	-0.023513	-0.007107	0.001695	-
0.008149	0.015585	0.003213	0.006808	0.031763	0.005016	
0.036668	-0.020864					
y616	0.009092	-0.026348	0.006778	0.006162	-0.004899	-
0.000116	-0.007276	-0.014613	0.008559	-0.001945	-0.016344	
0.006768	-0.001234	0.022459	-0.017387	0.016317	0.022556	
0.004049	0.015532	-0.020711	0.022186	-0.011235	0.010690	-
0.018818	-0.021281	-0.018179	-0.018499	-0.012855	0.007362	
0.013027	0.017714	0.030386	-0.000831	0.037480	0.013598	-
0.003777	-0.001919					
z616	0.007574	-0.010398	-0.006119	-0.006617	-0.007777	
0.016401	0.011040	-0.029734	0.013428	0.013390	-0.012186	
0.020479	-0.024273	0.001582	-0.027402	-0.000207	-0.001572	-
0.003499	0.017504	0.002071	0.002161	0.013212	0.006142	-
0.009234	-0.007461	-0.031311	0.009909	0.012694	0.013857	
0.026828	-0.006599	0.010499	0.011546	0.019640	0.006661	
0.014180	-0.021480					
x617	0.009635	-0.034440	0.003926	-0.018468	0.000755	
0.027747	0.008828	-0.025736	0.038721	-0.013613	0.004180	-
0.012247	-0.023159	-0.000826	-0.027246	0.035280	0.018389	-
0.010187	0.021603	-0.005667	0.001683	0.010374	-0.005334	-
0.030546	0.026820	-0.006276	-0.024842	-0.002304	0.000792	-
0.002359	0.014548	-0.000801	0.012262	0.032807	0.004493	
0.041165	-0.023862					
y617	0.009336	-0.023346	0.006356	0.008040	-0.010176	
0.001252	-0.008059	-0.012163	0.006345	-0.005462	-0.019759	
0.006338	-0.001781	0.024468	-0.019328	0.016192	0.024216	
0.000875	0.015038	-0.020833	0.022661	-0.012541	0.009897	-
0.018245	-0.019014	-0.021097	-0.011896	-0.012262	0.006468	
0.009828	0.012805	0.031508	-0.001327	0.037522	0.012395	-
0.004015	-0.002474					
z617	0.006862	-0.003947	-0.007759	-0.008188	-0.006668	
0.007461	0.008569	-0.022199	0.009028	0.017236	-0.013133	
0.020373	-0.016344	-0.001497	-0.020939	-0.004349	-0.006437	-
0.005655	0.014873	0.003716	-0.001725	0.013277	0.003127	-
0.011782	-0.004030	-0.025146	0.016860	0.012575	0.010784	
0.027114	-0.011268	-0.000065	0.013104	0.021337	0.003003	
0.010896	-0.015241					
x618	0.010068	-0.035372	0.003885	-0.022827	0.001605	
0.027115	0.011609	-0.025500	0.039411	-0.011490	0.001346	-
0.010695	-0.024621	-0.000613	-0.032029	0.035755	0.021009	-
0.011552	0.018090	-0.003861	0.006122	0.010634	-0.001809	-
0.030233	0.019573	-0.009810	-0.022558	-0.001760	0.000536	-
0.002936	0.011330	-0.000690	0.015608	0.031636	0.005695	
0.036184	-0.027573					
y618	0.009499	-0.020885	0.005194	0.010424	-0.015139	
0.004576	-0.007683	-0.010993	0.005299	-0.009506	-0.020724	

0.006601	-0.002876	0.023579	-0.019316	0.014705	0.024391	-
0.000201	0.017165	-0.020039	0.021538	-0.012048	0.008201	-
0.020539	-0.015034	-0.020328	-0.009781	-0.007945	0.003050	
0.011163	0.007502	0.029376	-0.000692	0.036980	0.007916	
0.001192	0.000753					
z618	0.006100	0.002635	-0.009653	-0.008060	-0.005458	-
0.000253	0.006103	-0.015004	0.004353	0.020305	-0.012944	
0.019823	-0.009988	-0.004068	-0.014458	-0.007009	-0.010319	-
0.004625	0.014246	0.004690	-0.005181	0.014034	0.000957	-
0.012213	-0.001822	-0.017651	0.021022	0.012154	0.007510	
0.026563	-0.014234	-0.009068	0.013221	0.024997	-0.002606	
0.009049	-0.006025					
x619	0.010526	-0.035371	0.003087	-0.025473	0.002347	
0.027132	0.013005	-0.025366	0.039741	-0.007711	0.000118	-
0.007889	-0.025142	-0.002568	-0.036417	0.035152	0.022786	-
0.010609	0.016323	-0.001740	0.008771	0.014511	-0.000735	-
0.030612	0.016859	-0.009249	-0.022262	0.002639	0.000385	-
0.003231	0.008626	-0.000624	0.018523	0.031414	0.007100	
0.035432	-0.024123					
y619	0.008898	-0.018837	0.004937	0.010599	-0.020887	
0.005610	-0.007215	-0.010418	0.003241	-0.013662	-0.022744	
0.007350	-0.003290	0.024681	-0.020001	0.014412	0.023188	-
0.001841	0.015490	-0.015549	0.021506	-0.012267	0.007904	-
0.019515	-0.016815	-0.022067	-0.006153	-0.009716	-0.001659	
0.012295	0.003100	0.028121	-0.003030	0.037410	-0.000001	-
0.000354	0.000739					
z619	0.005293	0.008959	-0.011334	-0.007273	-0.004006	-
0.007481	0.004683	-0.008311	-0.000678	0.022966	-0.012349	
0.018909	-0.004892	-0.005434	-0.007902	-0.009367	-0.014433	-
0.002867	0.013552	0.005822	-0.008604	0.013192	-0.002038	-
0.012539	-0.002353	-0.012050	0.023504	0.009727	0.004461	
0.023379	-0.018184	-0.015821	0.013172	0.023936	-0.006539	
0.004061	-0.000541					
x620	0.009810	-0.035336	0.002046	-0.027805	0.001391	
0.026049	0.014753	-0.026308	0.038679	-0.003061	-0.002873	-
0.002489	-0.025995	-0.003207	-0.037940	0.034337	0.022264	-
0.007918	0.012168	0.001680	0.010191	0.017605	-0.001719	-
0.030522	0.012364	-0.010096	-0.023274	0.004144	-0.002557	-
0.002266	0.003126	0.002061	0.018061	0.029513	0.007427	
0.030473	-0.017775					
y620	0.009115	-0.016481	0.004671	0.009633	-0.025648	
0.006079	-0.007871	-0.009476	0.001325	-0.016950	-0.022137	
0.007299	-0.001596	0.023491	-0.022556	0.012849	0.021741	-
0.003777	0.013907	-0.010245	0.022005	-0.009576	0.007326	-
0.018826	-0.017443	-0.019737	-0.002196	-0.009617	-0.004582	
0.010939	0.000352	0.024911	-0.004285	0.037783	-0.008373	-
0.000233	0.001714					
z620	0.004885	0.015736	-0.013227	-0.005243	-0.002183	-
0.014548	0.002370	-0.001539	-0.005858	0.025038	-0.010190	
0.016651	-0.000823	-0.007106	-0.002494	-0.010041	-0.018510	-
0.000042	0.013459	0.006182	-0.011810	0.012989	-0.004056	-
0.012188	-0.004117	-0.006714	0.026043	0.006991	0.003574	
0.017828	-0.020471	-0.020407	0.012124	0.022827	-0.009757	
0.000073	0.006136					
x621	0.008923	-0.034324	0.000594	-0.029335	-0.000838	
0.022947	0.014083	-0.026319	0.036731	0.003720	-0.004937	
0.002918	-0.025157	-0.004746	-0.041014	0.033905	0.021933	-
0.004016	0.008009	0.006928	0.013013	0.023960	-0.003550	-
0.027559	0.005666	-0.008682	-0.022107	0.006019	-0.006020	-

0.002469 -0.000784 0.001054 0.016631 0.033318 0.000831
0.026212 -0.008859
y621 0.009372 -0.015008 0.004593 0.007788 -0.029486
0.007282 -0.007080 -0.010839 -0.001049 -0.020828 -0.021868
0.009047 -0.000186 0.022089 -0.022740 0.009824 0.018128 -
0.006618 0.009910 -0.003864 0.018279 -0.008828 0.004417 -
0.022922 -0.015652 -0.018128 -0.002205 -0.012470 -0.008055
0.008300 -0.005623 0.027328 -0.005259 0.028155 -0.009259 -
0.005583 0.005212
z621 0.004557 0.022560 -0.014757 -0.002676 0.000242 -
0.020887 0.001169 0.004319 -0.011208 0.025968 -0.007832
0.014376 0.001729 -0.007190 0.002535 -0.010036 -0.022544
0.004675 0.012978 0.006386 -0.015260 0.011238 -0.006247 -
0.010077 -0.007567 -0.002754 0.026529 0.002429 0.003112
0.011028 -0.022657 -0.021182 0.009945 0.019300 -0.010546 -
0.006319 0.012763
x622 0.008048 -0.032939 -0.000718 -0.030382 -0.003935
0.017892 0.012463 -0.026226 0.033076 0.010567 -0.008293
0.008433 -0.021985 -0.006351 -0.044197 0.033151 0.018958 -
0.001047 -0.000814 0.014023 0.013929 0.028587 -0.009554 -
0.026139 0.001158 -0.007131 -0.022646 0.005177 -0.011117 -
0.003988 -0.009289 0.003431 0.013817 0.030451 -0.007703
0.017452 -0.000525
y622 0.009105 -0.013193 0.005045 0.004475 -0.033346
0.009326 -0.006392 -0.013116 -0.001961 -0.021558 -0.019505
0.012982 0.000507 0.019920 -0.021626 0.005980 0.016414 -
0.007061 0.009820 0.002604 0.015156 -0.004257 0.004278 -
0.023906 -0.017780 -0.015018 -0.000425 -0.011427 -0.010535
0.005991 -0.005164 0.027570 -0.007383 0.026493 -0.008898 -
0.007729 0.015654
z622 0.004082 0.029565 -0.016698 0.001619 0.002673 -
0.026256 -0.000452 0.009799 -0.016252 0.026307 -0.004805
0.011786 0.002077 -0.007271 0.008707 -0.008165 -0.026202
0.012286 0.014014 0.004362 -0.018456 0.010829 -0.007666 -
0.006742 -0.012912 0.000618 0.027519 -0.000428 0.005371
0.002686 -0.023101 -0.020080 0.006033 0.016022 -0.008537 -
0.009832 0.021061
x623 0.006626 -0.031028 -0.001507 -0.031586 -0.007673
0.012649 0.010464 -0.027110 0.028546 0.019542 -0.010236
0.016551 -0.018005 -0.007467 -0.047044 0.029853 0.015729
0.001295 -0.007859 0.022466 0.014081 0.034452 -0.015612 -
0.023436 -0.007503 -0.006064 -0.023597 0.006049 -0.017734 -
0.005920 -0.016388 0.004344 0.009609 0.029809 -0.018386
0.008888 0.013566
y623 0.009103 -0.011434 0.004081 0.001471 -0.037792
0.009251 -0.006619 -0.014152 -0.003799 -0.023850 -0.018680
0.015325 0.001995 0.016156 -0.019120 0.004893 0.011585 -
0.004523 0.003779 0.008908 0.011556 0.001575 0.001213 -
0.026238 -0.014243 -0.009507 0.003069 -0.014785 -0.012019
0.003203 -0.008595 0.030785 -0.011777 0.019767 -0.007174 -
0.015166 0.023344
z623 0.004119 0.037286 -0.018813 0.006526 0.005651 -
0.030494 -0.001276 0.015488 -0.021486 0.027107 0.000436
0.008882 0.002207 -0.006399 0.013899 -0.005610 -0.027836
0.021315 0.014700 0.001860 -0.021379 0.008912 -0.008514 -
0.002880 -0.019338 0.004692 0.027652 -0.005390 0.008537 -
0.008320 -0.023672 -0.016759 0.001099 0.011356 -0.005043 -
0.015129 0.028560
x624 0.006436 -0.030454 -0.003980 -0.025611 -0.006190
0.007924 0.012359 -0.020816 0.024985 0.024342 -0.007370

0.018082	-0.010153	-0.005261	-0.036193	0.028344	0.007791	-
0.002223	-0.014372	0.015962	0.016305	0.031299	-0.015982	-
0.012415	-0.013658	-0.004348	-0.018086	0.015111	-0.009213	-
0.004942	-0.010992	-0.001643	0.002907	0.015552	-0.025043	
0.015703	0.003531					
y624	0.008290	-0.011963	0.002213	0.004831	-0.039848	
0.008007	-0.005453	-0.010722	0.000630	-0.025470	-0.017498	
0.019849	0.008858	0.017066	-0.011363	0.010257	0.006737	-
0.006768	0.002279	0.008792	0.010913	0.005326	0.000120	-
0.019627	-0.014172	-0.004395	0.007689	-0.015406	-0.007652	
0.004808	-0.000348	0.022868	-0.021904	0.015898	0.005142	-
0.018295	0.026358					
z624	0.005921	0.050035	-0.018306	0.002010	0.011034	-
0.034693	-0.004445	0.019605	-0.032961	0.027902	0.003254	
0.000075	-0.004298	-0.010587	0.015493	-0.015040	-0.028839	
0.027324	0.019073	-0.005676	-0.023485	0.011496	-0.007543	-
0.003973	-0.015267	0.011990	0.029839	-0.011796	0.003991	-
0.009118	-0.025191	-0.014918	0.007561	0.014985	-0.010436	-
0.022276	0.028860					
x625	0.005210	-0.028191	-0.006639	-0.020816	-0.005021	
0.001540	0.013330	-0.014583	0.021456	0.028809	-0.005829	
0.018837	-0.005386	-0.003198	-0.027649	0.027030	0.000173	-
0.002049	-0.020061	0.011496	0.017033	0.033021	-0.016266	-
0.003212	-0.018318	-0.001116	-0.009811	0.015553	-0.000059	-
0.002362	-0.005455	-0.006532	-0.003785	0.006271	-0.027555	
0.019128	-0.003342					
y625	0.009661	-0.011220	0.001070	0.008170	-0.040311	
0.011709	-0.003774	-0.006728	0.001705	-0.026359	-0.013244	
0.022170	0.014853	0.014379	-0.001984	0.009480	0.004085	-
0.009752	0.005640	0.004067	0.008489	0.000451	-0.000241	-
0.018667	-0.011100	-0.002415	0.002134	-0.004751	-0.009749	
0.004763	0.002530	0.017201	-0.021475	0.010236	0.012244	-
0.015335	0.025159					
z625	0.007322	0.060129	-0.017034	-0.001650	0.014985	-
0.037204	-0.007239	0.023072	-0.042901	0.027633	0.004649	-
0.008944	-0.011449	-0.014883	0.017497	-0.022480	-0.028935	
0.032118	0.023992	-0.012512	-0.026218	0.012316	-0.005815	-
0.007888	-0.010383	0.014757	0.029872	-0.017079	-0.000601	-
0.008352	-0.028347	-0.010956	0.014038	0.018353	-0.011893	-
0.028683	0.028859					
x626	0.004368	-0.024100	-0.008591	-0.017165	-0.004104	-
0.003941	0.013721	-0.007420	0.016829	0.032498	-0.003129	
0.018694	-0.002196	-0.002178	-0.021374	0.023163	-0.004657	-
0.000995	-0.021057	0.006618	0.016964	0.034575	-0.015397	
0.002628	-0.021992	0.001663	-0.003325	0.014674	0.006701	
0.001450	-0.000084	-0.009082	-0.007447	0.001115	-0.026857	
0.021380	-0.009367					
y626	0.010370	-0.008882	0.000758	0.010525	-0.039304	
0.014249	-0.002748	-0.004993	0.002341	-0.025809	-0.012643	
0.022359	0.016622	0.012442	0.005528	0.012232	-0.000018	-
0.013901	0.001367	0.001930	0.006286	-0.005810	-0.001979	-
0.014392	-0.009262	-0.002215	-0.000334	0.000664	-0.010993	
0.002486	0.000466	0.013927	-0.020227	0.004903	0.012974	-
0.016239	0.024051					
z626	0.008211	0.068705	-0.014967	-0.004790	0.018010	-
0.038738	-0.009449	0.025218	-0.049982	0.028119	0.005990	-
0.017180	-0.018746	-0.018597	0.016843	-0.028262	-0.026771	
0.034540	0.027487	-0.016574	-0.027951	0.010890	-0.003697	-
0.011597	-0.006957	0.016889	0.029811	-0.023094	-0.003822	-

0.006389 -0.031407 -0.006369 0.018875 0.021786 -0.013264 -
0.033656 0.030201
x627 0.003431 -0.017971 -0.009944 -0.013277 -0.004243 -
0.009574 0.012257 0.000531 0.011233 0.034738 -0.002816
0.016105 -0.001901 -0.002126 -0.015777 0.020033 -0.009876 -
0.000252 -0.022486 0.000922 0.015915 0.034805 -0.014498
0.007300 -0.022303 0.001371 0.001491 0.013124 0.010501
0.005170 0.003079 -0.007808 -0.008471 -0.001786 -0.024991
0.020280 -0.013946
y627 0.011140 -0.006137 0.000411 0.008830 -0.034741
0.016607 -0.000877 -0.004040 0.004315 -0.021535 -0.007369
0.022295 0.017932 0.011878 0.008258 0.009416 -0.000654 -
0.017005 0.001849 0.002228 0.003813 -0.008207 -0.003421 -
0.012245 -0.011753 0.003652 0.000034 0.000386 -0.010315
0.002638 -0.002217 0.007738 -0.020165 0.003252 0.011806 -
0.011945 0.026041
z627 0.008441 0.074789 -0.013116 -0.007758 0.020738 -
0.040020 -0.010892 0.026777 -0.053687 0.029020 0.008332 -
0.025111 -0.025287 -0.020649 0.013664 -0.033244 -0.023217
0.034344 0.031245 -0.018465 -0.028803 0.009798 -0.001092 -
0.015999 -0.004733 0.019827 0.029552 -0.030940 -0.005040 -
0.002196 -0.034039 -0.002411 0.021845 0.026343 -0.012663 -
0.034964 0.034780
x628 0.002420 -0.009356 -0.010754 -0.010719 -0.003897 -
0.013527 0.009992 0.008345 0.005395 0.038621 -0.000719
0.012259 -0.003885 -0.002655 -0.012823 0.013643 -0.010972
0.000562 -0.020105 -0.004161 0.014057 0.034485 -0.012838
0.008805 -0.023149 0.002266 0.003528 0.009910 0.010673
0.009120 0.005034 -0.005019 -0.005562 -0.001044 -0.021120
0.016232 -0.016460
y628 0.012117 -0.004038 0.000813 0.006274 -0.029257
0.017027 0.002570 -0.002403 0.002993 -0.021222 -0.006627
0.020556 0.016497 0.009642 0.011084 0.010107 -0.007172 -
0.018512 -0.003665 0.002799 0.003193 -0.010696 -0.004375 -
0.005832 -0.010150 0.003609 0.008080 -0.004945 -0.003972
0.002344 -0.006488 0.007149 -0.023527 0.000416 0.007171 -
0.003015 0.028391
z628 0.008173 0.078591 -0.010537 -0.010530 0.022561 -
0.040070 -0.010795 0.027921 -0.054880 0.029580 0.010626 -
0.032416 -0.031710 -0.021673 0.009342 -0.036381 -0.017330
0.032592 0.034069 -0.018412 -0.028886 0.007653 0.003195 -
0.020016 -0.002693 0.021153 0.029444 -0.039337 -0.004589
0.003345 -0.035237 0.002416 0.023146 0.030959 -0.009779 -
0.033682 0.040122
x629 0.001258 0.000765 -0.010827 -0.008786 -0.003487 -
0.017847 0.006453 0.016646 -0.001168 0.040190 -0.000035
0.006300 -0.008623 -0.004162 -0.011276 0.007868 -0.012807
0.002452 -0.016360 -0.008887 0.010790 0.034155 -0.011857
0.008123 -0.020798 -0.000042 0.007836 0.002064 0.009519
0.014495 0.004770 -0.000601 -0.001308 0.001564 -0.017738
0.013849 -0.016285
y629 0.013036 -0.000132 0.002253 0.002124 -0.024692
0.020055 0.005097 -0.001192 0.004101 -0.013193 -0.001317
0.019730 0.016197 0.007263 0.015878 0.004093 -0.005509 -
0.016282 -0.002776 -0.000921 -0.000045 -0.011627 -0.001314 -
0.002816 -0.012086 0.011437 0.006391 0.001275 -0.002517
0.004633 -0.006232 0.002980 -0.022359 0.009627 0.000658 -
0.003496 0.028455
z629 0.006938 0.080399 -0.007483 -0.012092 0.022744 -
0.039211 -0.010441 0.028431 -0.053857 0.030854 0.012967 -

0.039500 -0.037540 -0.021353 0.003865 -0.038443 -0.009795
0.029134 0.036705 -0.016761 -0.029329 0.004685 0.008469 -
0.024599 -0.001364 0.021475 0.026759 -0.047962 -0.003676
0.011048 -0.034762 0.006506 0.025000 0.037773 -0.004621 -
0.033734 0.043456
x630 0.000056 0.012950 -0.009967 -0.008640 -0.002670 -
0.021334 0.002696 0.024850 -0.007577 0.042262 0.002017 -
0.000561 -0.015614 -0.005664 -0.012082 -0.000505 -0.010831
0.004217 -0.010988 -0.014064 0.007412 0.032918 -0.010476
0.006880 -0.019466 -0.001923 0.011006 -0.006477 0.005432
0.020370 0.002550 0.004283 0.003816 0.010105 -0.015070
0.008508 -0.013893
y630 0.014292 0.004798 0.003079 -0.002392 -0.018865
0.021021 0.006697 0.001629 0.003620 -0.003995 0.004469
0.017094 0.017200 0.004755 0.016448 -0.001817 -0.010008 -
0.013481 0.000366 -0.002112 -0.004002 -0.008629 0.001624 -
0.004750 -0.008604 0.016391 0.005380 0.002423 -0.002268
0.008811 -0.002765 0.003844 -0.017942 0.013310 0.003723
0.000961 0.021727
z630 0.005006 0.080023 -0.004626 -0.012796 0.021829 -
0.038080 -0.009714 0.028597 -0.050206 0.031920 0.015103 -
0.046660 -0.042667 -0.019074 -0.003353 -0.038314 -0.002615
0.023291 0.039075 -0.013034 -0.029407 0.002078 0.013831 -
0.030141 0.000380 0.019730 0.023572 -0.058446 -0.003078
0.020053 -0.032666 0.010956 0.026943 0.044008 0.006019 -
0.031306 0.045513
x631 -0.001198 0.027041 -0.008025 -0.008816 -0.001644 -
0.024548 -0.002786 0.033440 -0.014204 0.042680 0.002935 -
0.010347 -0.025086 -0.007741 -0.013523 -0.007927 -0.008643
0.006460 -0.002858 -0.019406 0.002935 0.030875 -0.009187
0.002554 -0.015213 -0.006057 0.015443 -0.017530 -0.000296
0.027490 -0.000582 0.011925 0.010452 0.021716 -0.011261
0.005482 -0.007185
y631 0.014051 0.009823 0.000964 -0.003340 -0.015246
0.015859 0.006716 0.004905 -0.003834 0.001198 0.001273
0.010958 0.010472 -0.001060 0.019025 -0.005774 -0.020514 -
0.007375 -0.006037 -0.006471 -0.008258 -0.000775 -0.001476 -
0.002116 -0.003942 0.018865 0.006315 -0.007040 0.000871
0.011210 -0.008143 0.004942 -0.011861 0.018362 0.003888 -
0.005183 0.013160
z631 0.002819 0.078420 -0.000594 -0.014845 0.020328 -
0.033976 -0.007103 0.026947 -0.043249 0.035606 0.020434 -
0.051703 -0.046011 -0.016141 -0.014211 -0.040233 0.011422
0.015534 0.042106 -0.005977 -0.029711 -0.002691 0.021530 -
0.036758 -0.001060 0.019535 0.017521 -0.068350 -0.000583
0.030266 -0.029186 0.015185 0.029003 0.051829 0.019538 -
0.028351 0.047270
x632 -0.002822 0.042718 -0.004437 -0.011593 -0.001498 -
0.026240 -0.008783 0.042248 -0.021675 0.044084 0.004239 -
0.018705 -0.035548 -0.009249 -0.018280 -0.018199 -0.003553
0.009170 0.008384 -0.022930 -0.003646 0.028817 -0.006706 -
0.002611 -0.009650 -0.010494 0.018446 -0.030291 -0.010530
0.035879 -0.002540 0.018631 0.019656 0.037193 -0.006685 -
0.004944 -0.005663
y632 0.016269 0.020042 0.000067 -0.009194 -0.007631
0.014803 0.009881 0.006266 -0.009522 0.010213 0.007560
0.001367 0.004457 -0.008684 0.014714 -0.017468 -0.017406 -
0.001119 -0.007087 -0.008692 -0.010989 -0.000181 0.003030 -
0.002132 -0.003683 0.027189 0.003282 -0.018058 0.009039

0.011459	-0.016853	0.013467	-0.002876	0.032064	0.005930	
0.002716	0.020119					
z632	-0.000342	0.073789	0.002409	-0.013656	0.017079	-
0.031088	-0.005091	0.025363	-0.034121	0.036634	0.022393	-
0.057977	-0.051104	-0.012334	-0.024200	-0.036966	0.022867	
0.005326	0.043388	0.001876	-0.028345	-0.007364	0.028206	-
0.043017	-0.001716	0.016560	0.012246	-0.080647	0.004617	
0.041929	-0.025621	0.019937	0.027891	0.058663	0.034502	-
0.022218	0.051501					
x633	-0.003160	0.042933	-0.002852	-0.009538	-0.003735	-
0.025061	-0.011164	0.045608	-0.021659	0.043339	0.004709	-
0.022841	-0.036352	-0.010020	-0.013962	-0.022101	0.002802	
0.011870	0.015357	-0.025232	-0.011616	0.024060	-0.002623	-
0.004024	-0.011758	-0.013405	0.014623	-0.031680	-0.002428	
0.040016	-0.010513	0.016983	0.028122	0.020236	0.006831	-
0.001245	-0.006238					
y633	0.016787	0.024386	0.002371	-0.007637	-0.004458	
0.012782	0.010514	0.006088	-0.011358	0.013360	0.010661	-
0.004321	0.002194	-0.013554	0.011469	-0.019866	-0.016814	
0.004353	-0.011147	-0.007999	-0.014949	0.007330	0.003344	-
0.003493	-0.001690	0.028010	0.000511	-0.024562	0.010922	
0.012456	-0.017360	0.010735	0.001530	0.035001	0.012919	
0.002724	0.015373					
z633	-0.000326	0.060160	0.003411	-0.007753	0.018173	-
0.028377	-0.004105	0.016909	-0.024589	0.021828	0.021696	-
0.048024	-0.037856	-0.009398	-0.021615	-0.025482	0.021986	
0.008178	0.033338	0.002885	-0.026972	-0.004743	0.021681	-
0.031352	-0.002448	0.015673	0.010923	-0.069613	0.013349	
0.030637	-0.016395	0.017784	0.016453	0.046523	0.034914	-
0.015975	0.042424					
x634	-0.003695	0.042747	-0.000018	-0.008590	-0.005678	-
0.024885	-0.013236	0.048194	-0.021080	0.041827	0.004627	-
0.024192	-0.034672	-0.009320	-0.013106	-0.023319	0.008476	
0.015082	0.020038	-0.025498	-0.015568	0.020258	0.001688	
0.000449	-0.012667	-0.013139	0.012576	-0.032614	0.003596	
0.044464	-0.016324	0.014050	0.030953	0.011396	0.015964	
0.002958	-0.005740					
y634	0.016396	0.027258	0.003219	-0.005012	-0.002098	
0.011501	0.009873	0.005605	-0.012224	0.016644	0.012686	-
0.010189	-0.001244	-0.017706	0.010353	-0.022572	-0.015588	
0.004374	-0.012314	-0.006743	-0.020567	0.011397	0.005376	-
0.006463	-0.002663	0.026442	-0.004897	-0.028203	0.017876	
0.011390	-0.015245	0.009948	0.004961	0.034009	0.017440	
0.000060	0.010529					
z634	-0.000261	0.047097	0.003460	-0.002489	0.018787	-
0.025521	-0.002899	0.008934	-0.015781	0.008679	0.020911	-
0.039018	-0.026343	-0.005859	-0.018639	-0.015213	0.020488	
0.009867	0.024526	0.003547	-0.025707	-0.002910	0.015528	-
0.022178	-0.003504	0.014207	0.009761	-0.058752	0.021118	
0.020778	-0.009407	0.016266	0.007849	0.034313	0.035634	-
0.011854	0.035433					
x635	-0.004577	0.042317	0.002896	-0.007721	-0.006753	-
0.025257	-0.014906	0.049442	-0.020581	0.039730	0.003782	-
0.024216	-0.031915	-0.007939	-0.013184	-0.022430	0.012586	
0.016846	0.022803	-0.024185	-0.016985	0.017030	0.007089	
0.006518	-0.014389	-0.012443	0.009529	-0.033010	0.006309	
0.048452	-0.016407	0.011589	0.029074	0.009002	0.019113	
0.005838	-0.004960					
y635	0.016231	0.027762	0.004345	-0.002819	-0.000431	
0.010274	0.009174	0.005397	-0.011137	0.018858	0.015678	-

0.013756	-0.002391	-0.020198	0.009982	-0.024952	-0.013081	
0.006269	-0.012070	-0.006557	-0.024982	0.015418	0.003024	-
0.008033	-0.003833	0.026940	-0.004057	-0.032650	0.026809	
0.009882	-0.019484	0.010356	0.010054	0.028448	0.024722	-
0.001512	0.005241					
z635	-0.000222	0.034810	0.003138	0.002070	0.019613	-
0.023038	-0.002133	0.001275	-0.008071	-0.002950	0.019416	-
0.031209	-0.016391	-0.002156	-0.015522	-0.005012	0.018372	
0.010531	0.016875	0.004354	-0.024167	-0.001890	0.010379	-
0.014219	-0.003869	0.012731	0.007978	-0.047334	0.026322	
0.013008	-0.003575	0.013884	0.000492	0.023841	0.035232	-
0.008620	0.028897					
x636	-0.004936	0.040919	0.005273	-0.006439	-0.007000	-
0.025657	-0.015871	0.049160	-0.019622	0.036608	0.002890	-
0.023771	-0.028185	-0.007038	-0.011885	-0.020936	0.014249	
0.017723	0.024749	-0.022728	-0.017640	0.013915	0.008525	
0.008982	-0.015144	-0.011018	0.007081	-0.032599	0.006869	
0.049326	-0.015952	0.009885	0.027649	0.006336	0.021210	
0.009370	-0.006283					
y636	0.015705	0.027198	0.005644	-0.000199	0.001579	
0.009631	0.006527	0.005079	-0.008882	0.019639	0.019705	-
0.016815	-0.001042	-0.020238	0.009546	-0.024997	-0.012827	
0.006775	-0.013179	-0.006661	-0.028025	0.019040	0.004752	-
0.005786	-0.003333	0.025003	-0.006578	-0.029553	0.035047	
0.010112	-0.019855	0.010976	0.009314	0.023950	0.025266	-
0.004888	0.006463					
z636	-0.000075	0.023432	0.002628	0.005038	0.020489	-
0.020510	-0.000240	-0.006324	-0.001460	-0.012548	0.017366	-
0.024077	-0.008669	0.001322	-0.012611	0.003657	0.016527	
0.010396	0.011271	0.005666	-0.021741	-0.002750	0.004803	-
0.007836	-0.005209	0.012278	0.006233	-0.037391	0.029122	
0.007107	-0.000419	0.011828	-0.003248	0.014146	0.033933	-
0.006701	0.021025					
x637	-0.005397	0.038825	0.006969	-0.005697	-0.006570	-
0.025965	-0.016952	0.047889	-0.018033	0.032726	0.002271	-
0.022434	-0.023451	-0.006189	-0.010776	-0.017611	0.013532	
0.018582	0.025026	-0.020004	-0.017600	0.012498	0.009616	
0.012937	-0.015505	-0.009827	0.004192	-0.032574	0.005835	
0.048122	-0.012914	0.009989	0.023182	0.007347	0.017512	
0.011338	-0.007841					
y637	0.015621	0.025363	0.008248	0.003156	0.003392	
0.007733	0.004796	0.004048	-0.006367	0.019006	0.021977	-
0.019227	0.003320	-0.018455	0.010707	-0.024768	-0.010329	
0.006019	-0.013378	-0.009087	-0.027608	0.018267	0.004226	-
0.004796	-0.004223	0.023979	-0.007408	-0.022746	0.039939	
0.014080	-0.022004	0.009769	0.010271	0.014702	0.028063	-
0.005732	0.004518					
z637	0.000103	0.013204	0.001701	0.006576	0.021605	-
0.017855	0.001624	-0.013495	0.004631	-0.019586	0.016196	-
0.017702	-0.003301	0.004724	-0.010214	0.011560	0.013937	
0.009394	0.006645	0.008149	-0.019265	-0.004505	0.000467	-
0.002733	-0.006115	0.012792	0.004235	-0.028849	0.029883	
0.001283	0.002187	0.009741	-0.005230	0.008184	0.030719	-
0.006430	0.014314					
x638	-0.005575	0.036426	0.008849	-0.004290	-0.006142	-
0.026952	-0.017939	0.045522	-0.016450	0.027441	0.002179	-
0.019964	-0.017051	-0.005087	-0.009431	-0.012563	0.010435	
0.017459	0.023044	-0.016161	-0.015805	0.011323	0.010294	
0.015879	-0.014356	-0.007705	0.001437	-0.028163	0.002424	

0.045913 -0.008177 0.010688 0.017734 0.009284 0.012377
0.013437 -0.007764
y638 0.014542 0.020567 0.009405 0.005040 0.007964
0.006371 0.004897 0.001685 -0.002487 0.018435 0.022870 -
0.018786 0.007588 -0.015369 0.012217 -0.025126 -0.008476
0.010036 -0.011047 -0.011836 -0.026696 0.018594 -0.001694 -
0.003956 -0.008369 0.025788 -0.005232 -0.025583 0.043792
0.016951 -0.023108 0.006517 0.006768 0.008136 0.025888 -
0.006145 -0.001486
z638 0.000815 0.004637 0.000779 0.007657 0.021944 -
0.014964 0.002797 -0.019812 0.009668 -0.024957 0.015431 -
0.012905 0.000294 0.008206 -0.007809 0.019129 0.011379
0.006330 0.003584 0.011115 -0.015489 -0.007021 -0.000731
0.001461 -0.005594 0.012608 0.001498 -0.017982 0.028174 -
0.002661 0.004450 0.007133 -0.004031 0.003235 0.027343 -
0.008507 0.010526
x639 -0.005353 0.032335 0.009256 -0.002006 -0.004438 -
0.027956 -0.017799 0.041899 -0.014212 0.020332 0.000978 -
0.016998 -0.009197 -0.003896 -0.006207 -0.007863 0.005179
0.016334 0.020954 -0.012364 -0.015208 0.010837 0.007150
0.016526 -0.013452 -0.006927 -0.001490 -0.022795 -0.000745
0.041078 -0.002669 0.013519 0.011641 0.007951 0.005753
0.014545 -0.008229
y639 0.013849 0.016453 0.012627 0.006426 0.011566
0.005734 0.001797 -0.000624 0.002630 0.018385 0.027253 -
0.018666 0.013638 -0.011738 0.012979 -0.020813 -0.006918
0.010027 -0.009243 -0.013783 -0.022148 0.015892 -0.003485 -
0.002048 -0.008205 0.029618 -0.004810 -0.019566 0.040416
0.019859 -0.020297 0.002467 0.000951 0.007820 0.019691 -
0.004773 -0.005263
z639 0.001397 -0.003796 -0.001074 0.008034 0.021967 -
0.012689 0.005788 -0.026337 0.013429 -0.028730 0.012729 -
0.008629 0.002136 0.012092 -0.004259 0.024021 0.007915
0.002964 0.000911 0.013607 -0.011506 -0.011306 -0.003926
0.003093 -0.005845 0.011908 -0.000550 -0.010847 0.026248 -
0.004639 0.001876 0.005499 0.000202 -0.004036 0.023112 -
0.012612 0.005588
x640 -0.004721 0.026728 0.010419 0.000016 -0.001665 -
0.028711 -0.016509 0.036754 -0.010805 0.012337 0.001104 -
0.013893 0.000348 -0.001438 -0.002649 -0.002796 -0.001735
0.013312 0.018483 -0.009742 -0.012137 0.010484 0.001002
0.017594 -0.011541 -0.004855 -0.004014 -0.013632 -0.004855
0.035939 0.002262 0.014622 0.002763 0.007072 -0.003947
0.015077 -0.011743
y640 0.012459 0.011960 0.014677 0.008488 0.015178
0.003531 -0.003389 -0.003015 0.007339 0.016824 0.028397 -
0.016123 0.020610 -0.008477 0.014477 -0.013876 -0.005796
0.011874 -0.007591 -0.013977 -0.017526 0.012719 -0.005056 -
0.000755 -0.008982 0.032785 -0.004360 -0.015975 0.033949
0.022358 -0.013608 0.002319 -0.003386 0.008722 0.011171 -
0.003693 -0.006211
z640 0.002550 -0.011568 -0.002823 0.006702 0.021891 -
0.009358 0.010435 -0.032719 0.016903 -0.029046 0.012226 -
0.005387 0.002758 0.016639 -0.000983 0.025906 0.005401 -
0.002552 0.000349 0.015983 -0.006803 -0.017414 -0.006730
0.002924 -0.006527 0.012445 -0.003826 -0.002220 0.022786 -
0.005004 -0.003696 0.002361 0.006915 -0.010434 0.018814 -
0.019032 0.000122
x641 -0.004682 0.020445 0.010979 0.001550 0.002205 -
0.029853 -0.016106 0.031409 -0.006648 0.003123 0.001081 -

0.008285	0.010988	0.001954	-0.000562	0.003536	-0.008257	
0.010334	0.013738	-0.006626	-0.007253	0.009946	-0.003119	
0.019831	-0.012091	-0.000911	-0.003423	-0.007059	-0.010182	
0.031490	0.009621	0.014715	-0.010258	0.010024	-0.016959	
0.017865	-0.011186					
y641	0.011782	0.005730	0.017797	0.010646	0.018719	
0.000785	-0.006838	-0.007942	0.011296	0.014167	0.027890	-
0.014616	0.028593	-0.004852	0.018119	-0.005148	-0.009867	
0.011128	-0.006565	-0.012967	-0.011491	0.007581	-0.013894	
0.001692	-0.007288	0.032978	-0.007863	-0.005967	0.026260	
0.020556	-0.007975	0.005161	-0.008722	0.005153	0.000725	-
0.005584	-0.015644					
z641	0.003302	-0.018201	-0.004934	0.004244	0.022088	-
0.005929	0.014346	-0.037873	0.020293	-0.027449	0.012020	-
0.001960	0.001464	0.022029	0.001370	0.026556	0.004514	-
0.007658	0.002187	0.018406	-0.001342	-0.023858	-0.005575	
0.002336	-0.008384	0.015248	-0.006165	0.004627	0.016398	-
0.001322	-0.008712	-0.002245	0.015616	-0.013065	0.013349	-
0.027286	-0.001639					
x642	-0.003477	0.016732	0.008420	0.002463	0.003315	-
0.029835	-0.012216	0.030841	-0.007003	-0.001710	0.004935	-
0.005220	0.012012	0.002412	-0.002176	0.003820	-0.005475	
0.010706	0.009505	-0.003313	-0.006651	0.006796	0.000021	
0.023170	-0.002762	0.002301	-0.003849	-0.003755	-0.010612	
0.024088	0.007363	0.013731	-0.003771	0.005902	-0.022593	
0.008594	-0.009033					
y642	0.012287	0.002339	0.015861	0.011039	0.020130	
0.001860	-0.009214	-0.006505	0.011301	0.016673	0.030476	-
0.014060	0.032251	-0.004786	0.019175	-0.003171	-0.002575	
0.012511	-0.005686	-0.010476	-0.014331	0.003091	-0.013231	-
0.002899	-0.003083	0.028172	-0.006126	-0.004795	0.018694	
0.019755	-0.004973	0.000873	-0.011423	0.003851	-0.002471	-
0.008616	-0.010284					
z642	0.006274	-0.021039	-0.009528	0.005094	0.019921	-
0.001212	0.018240	-0.041132	0.017567	-0.028263	0.009928	-
0.008006	0.000802	0.022040	-0.001334	0.028368	0.012020	-
0.009347	-0.001944	0.020118	-0.001588	-0.026316	-0.005923	-
0.005691	-0.015830	0.005306	-0.004035	-0.007606	0.018626	
0.000958	0.000546	-0.004394	0.011167	-0.007940	0.008850	-
0.026433	0.003868					
x643	-0.002193	0.013076	0.005737	0.002481	0.004282	-
0.028170	-0.009452	0.029365	-0.006578	-0.004840	0.009006	-
0.003138	0.013364	0.002888	-0.002398	0.003359	-0.004015	
0.009002	0.006035	-0.001652	-0.007388	0.003219	0.002418	
0.020518	0.004936	0.005635	-0.005230	-0.001291	-0.010220	
0.016721	0.000426	0.012877	0.000837	0.002482	-0.023675	
0.002530	-0.010960					
y643	0.012693	-0.000930	0.014195	0.012542	0.020662	
0.000797	-0.010482	-0.005229	0.010475	0.016508	0.028422	-
0.014421	0.032853	-0.004218	0.017794	0.001567	0.002560	
0.015130	-0.006325	-0.007679	-0.013588	-0.000412	-0.012478	-
0.000657	-0.000015	0.022196	-0.002484	-0.006684	0.012558	
0.019227	0.002514	-0.002101	-0.013116	0.001850	-0.009329	-
0.011533	-0.001794					
z643	0.009064	-0.023180	-0.013183	0.004881	0.018139	
0.004077	0.020865	-0.044217	0.016064	-0.027292	0.009478	-
0.012300	0.000780	0.020905	-0.002216	0.027286	0.017772	-
0.010804	-0.003654	0.020315	-0.003044	-0.027924	-0.006868	-
0.015997	-0.021523	-0.002600	-0.004114	-0.015866	0.020286	

0.002115 0.005824 -0.005698 0.008282 -0.005752 0.006153 -
0.025715 0.006288
x644 -0.001079 0.009166 0.003983 0.001441 0.005226 -
0.026544 -0.006079 0.027514 -0.005612 -0.007620 0.012428 -
0.003087 0.014224 0.003982 -0.003494 0.004296 -0.002127
0.008719 0.005472 -0.001267 -0.008203 -0.000006 0.003222
0.020923 0.010799 0.006901 -0.004376 -0.000131 -0.008247
0.008479 -0.001031 0.012068 0.003391 -0.002528 -0.023648 -
0.001931 -0.007727
y644 0.013156 -0.003800 0.011681 0.014700 0.021082
0.000185 -0.012690 -0.004639 0.010078 0.016882 0.025085 -
0.013826 0.032453 -0.004443 0.016764 0.004834 0.006026
0.015341 -0.008129 -0.005067 -0.011614 -0.004489 -0.011236 -
0.003739 0.001976 0.017975 -0.001886 -0.007300 0.005427
0.019957 0.003153 -0.004620 -0.011345 0.002694 -0.013714 -
0.013753 0.000622
z644 0.011081 -0.024691 -0.015232 0.003660 0.016143
0.008337 0.022319 -0.046495 0.014869 -0.025426 0.008881 -
0.014437 0.000455 0.019465 -0.003633 0.026757 0.022445 -
0.010507 -0.004418 0.020402 -0.003144 -0.027608 -0.008179 -
0.021085 -0.026740 -0.009158 -0.003596 -0.021988 0.022456
0.002214 0.011686 -0.006326 0.004575 -0.003890 0.005340 -
0.024700 0.009011
x645 0.000759 0.005730 0.002705 0.001116 0.006071 -
0.024336 -0.002836 0.024308 -0.003278 -0.010572 0.012949 -
0.003262 0.012584 0.006790 -0.004387 0.005002 -0.001028
0.008375 0.005361 0.000359 -0.007445 -0.004472 0.006127
0.019321 0.013500 0.009533 -0.005697 -0.000846 -0.005662
0.002936 -0.002718 0.010355 0.008147 -0.002543 -0.019045 -
0.003068 -0.006869
y645 0.013118 -0.006822 0.009443 0.015628 0.020942 -
0.000202 -0.014936 -0.003881 0.009138 0.018710 0.023402 -
0.013968 0.033180 -0.005445 0.015546 0.008150 0.008960
0.014983 -0.008576 -0.005127 -0.010445 -0.008078 -0.012286 -
0.006066 0.003640 0.012453 0.000162 -0.008226 -0.000475
0.018731 0.003823 -0.005969 -0.013969 -0.000290 -0.020169 -
0.015581 0.003808
z645 0.012529 -0.025167 -0.016302 0.002274 0.014204
0.011586 0.022095 -0.047952 0.014082 -0.023138 0.007101 -
0.014507 -0.000955 0.017855 -0.004768 0.025347 0.024517 -
0.009055 -0.004654 0.020910 -0.001043 -0.025717 -0.008617 -
0.023894 -0.030601 -0.013641 -0.003358 -0.025355 0.024710
0.002666 0.015143 -0.006718 0.002639 -0.001351 0.006551 -
0.023705 0.010234
x646 0.002365 0.001692 0.001196 0.000678 0.008314 -
0.021566 0.000674 0.019046 -0.000546 -0.012170 0.014032 -
0.003999 0.011387 0.009688 -0.005697 0.007285 0.002597
0.007867 0.005121 0.000726 -0.006471 -0.009387 0.009113
0.014790 0.016600 0.009295 -0.007205 -0.003430 -0.004447 -
0.002026 -0.000379 0.010792 0.010728 -0.003633 -0.015119 -
0.002837 -0.003221
y646 0.013108 -0.009072 0.007812 0.016451 0.018710 -
0.001029 -0.017419 -0.002192 0.008653 0.019152 0.018416 -
0.014729 0.031375 -0.005859 0.013867 0.010261 0.007613
0.014627 -0.007607 -0.005286 -0.007673 -0.010991 -0.014404 -
0.006900 0.003122 0.008854 0.002670 -0.008301 -0.003810
0.017491 0.000205 -0.008587 -0.014440 -0.001725 -0.022650 -
0.015178 0.003629
z646 0.013420 -0.024847 -0.016522 0.000110 0.013028
0.014254 0.020595 -0.048881 0.013371 -0.019392 0.006381 -

0.012664 -0.002016 0.014960 -0.004896 0.023210 0.025389 -
0.006575 -0.004335 0.020600 0.001691 -0.022330 -0.009013 -
0.025429 -0.032739 -0.017074 -0.003631 -0.026517 0.025957
0.002211 0.017896 -0.005795 0.000889 -0.000568 0.007845 -
0.022924 0.010637
x647 0.003857 -0.001491 -0.001087 0.000809 0.009373 -
0.018755 0.003799 0.013211 0.001977 -0.013014 0.013827 -
0.007926 0.009832 0.011304 -0.006175 0.010352 0.002927
0.005038 0.005721 0.000010 -0.006154 -0.011512 0.007284
0.009937 0.014980 0.009483 -0.011118 -0.007310 -0.001513 -
0.007457 -0.001754 0.010232 0.010270 -0.002374 -0.011622 -
0.004244 -0.002833
y647 0.013499 -0.011558 0.007823 0.015211 0.017248 -
0.000233 -0.019085 -0.001131 0.010112 0.020129 0.014264 -
0.013040 0.029324 -0.004226 0.009707 0.010804 0.008779
0.016380 -0.004780 -0.004922 -0.003363 -0.017387 -0.011063 -
0.009342 0.003464 0.005268 0.007036 -0.008354 -0.007499
0.016695 -0.001094 -0.009877 -0.011111 -0.005313 -0.022743 -
0.008530 0.004664
z647 0.013733 -0.023908 -0.016165 -0.001673 0.011400
0.015438 0.017515 -0.049278 0.012941 -0.015593 0.004081 -
0.009987 -0.003852 0.010676 -0.003684 0.021450 0.022469 -
0.003945 -0.004199 0.018813 0.005168 -0.016468 -0.011885 -
0.024324 -0.033934 -0.019907 -0.004636 -0.025356 0.027882
0.000862 0.016631 -0.004477 -0.001318 -0.000623 0.009404 -
0.022566 0.009357
x648 0.006599 -0.004810 -0.002252 -0.000607 0.012084 -
0.015050 0.007217 0.007727 0.006053 -0.013119 0.012732 -
0.012107 0.008268 0.012951 -0.006980 0.012122 0.003529
0.004005 0.007360 -0.001810 -0.005385 -0.017094 0.006141
0.002200 0.013840 0.007399 -0.013863 -0.010593 -0.000224 -
0.013324 -0.004237 0.007722 0.010312 -0.003479 -0.007585
0.000674 -0.000151
y648 0.012688 -0.013853 0.006367 0.016017 0.013631
0.000053 -0.021161 -0.001479 0.010342 0.021285 0.008515 -
0.012532 0.024642 -0.002676 0.006254 0.012789 0.007867
0.015358 -0.001986 -0.005833 0.001584 -0.019795 -0.009673 -
0.010750 0.000606 0.003894 0.008367 -0.010729 -0.008034
0.016296 -0.002135 -0.006961 -0.009269 -0.007154 -0.019048 -
0.006991 0.002061
z648 0.013695 -0.022487 -0.014411 -0.004297 0.010074
0.016232 0.013001 -0.048860 0.013365 -0.010634 0.001512 -
0.004723 -0.005497 0.005840 -0.002564 0.018721 0.017906 -
0.000126 -0.003173 0.016763 0.009768 -0.009754 -0.015140 -
0.021278 -0.033388 -0.022053 -0.005921 -0.021248 0.028223 -
0.001054 0.013467 -0.002734 -0.001962 -0.002412 0.011810 -
0.021724 0.006308
x649 0.008786 -0.007481 -0.003009 -0.002852 0.013877 -
0.011512 0.009643 0.002585 0.010640 -0.011965 0.008530 -
0.015388 0.004643 0.015544 -0.009210 0.014705 0.003060
0.002541 0.009755 -0.005112 -0.001829 -0.020943 0.007231 -
0.001332 0.008937 0.004647 -0.017416 -0.018013 0.003283 -
0.015345 -0.005602 0.006320 0.009461 -0.001473 -0.004724
0.005041 0.003952
y649 0.012451 -0.016203 0.005932 0.016541 0.010596
0.001180 -0.022169 -0.003118 0.011376 0.022434 0.004175 -
0.013188 0.020846 -0.000724 0.002372 0.013907 0.007190
0.013575 0.001702 -0.006191 0.006295 -0.024113 -0.010346 -
0.015599 -0.001957 0.003296 0.011252 -0.010520 -0.009903

0.013195 -0.003449 -0.007277 -0.008497 -0.008129 -0.012293 -
0.001121 -0.003682
z649 0.012740 -0.020312 -0.011564 -0.007196 0.008448
0.015688 0.006741 -0.047633 0.014087 -0.005168 -0.002284
0.002599 -0.007870 0.000543 -0.001373 0.016349 0.011255
0.004485 -0.001788 0.013790 0.016636 -0.000715 -0.017937 -
0.013702 -0.032440 -0.023236 -0.007421 -0.015536 0.028968 -
0.002284 0.008776 0.000430 -0.002570 -0.003510 0.014817 -
0.021501 0.001659
x650 0.010894 -0.010911 -0.004194 -0.003667 0.015197 -
0.009110 0.011107 -0.002859 0.015343 -0.011068 0.003471 -
0.020606 0.000661 0.017683 -0.010636 0.018842 0.001424 -
0.001786 0.011964 -0.011854 0.004689 -0.025087 0.008151 -
0.005475 0.003156 -0.000681 -0.018466 -0.024584 0.005674 -
0.018571 -0.004219 0.006892 0.007734 0.002460 0.003473
0.014684 0.008676
y650 0.012115 -0.017535 0.006720 0.014878 0.006915
0.004144 -0.022554 -0.005355 0.013051 0.024017 -0.001510 -
0.012869 0.015361 0.002118 -0.003473 0.013645 0.006790
0.014655 0.006979 -0.003961 0.009360 -0.028787 -0.011152 -
0.020048 -0.005800 0.004036 0.011298 -0.011877 -0.009402
0.011622 -0.008058 -0.008949 -0.006979 -0.009485 -0.007795
0.002561 -0.009447
z650 0.011511 -0.017176 -0.007472 -0.010856 0.007006
0.014964 -0.000635 -0.045968 0.015176 0.001620 -0.005343
0.011871 -0.009968 -0.006146 0.000335 0.012676 0.003364
0.009897 0.000710 0.010403 0.024081 0.009167 -0.021178 -
0.004970 -0.030400 -0.022696 -0.009861 -0.006502 0.029406 -
0.004653 0.002762 0.004537 -0.002277 -0.006137 0.018476 -
0.021919 -0.004345
x651 0.012682 -0.014387 -0.004770 -0.006310 0.017110 -
0.008880 0.011726 -0.006083 0.015883 -0.010362 0.002109 -
0.021593 -0.001813 0.015104 -0.010440 0.021368 0.001333 -
0.004430 0.017344 -0.010231 0.008327 -0.024532 0.011703 -
0.010396 -0.000078 -0.006197 -0.027850 -0.022849 0.007381 -
0.019929 -0.006491 0.012567 0.006907 0.001512 -0.000473
0.005777 0.013539
y651 0.011440 -0.018973 0.007037 0.013062 0.001787
0.003722 -0.025805 -0.007098 0.009373 0.019189 -0.003866 -
0.011737 0.012438 0.002103 -0.006322 0.017126 0.006868
0.014199 0.012763 -0.000415 0.008983 -0.026429 -0.010690 -
0.021072 -0.005387 0.001825 0.011970 -0.006537 -0.005308
0.006556 -0.011351 -0.005868 -0.008199 -0.011079 -0.009110 -
0.006495 -0.006928
z651 0.010206 -0.013877 -0.004189 -0.014590 0.004269
0.008664 -0.001713 -0.042318 0.012938 0.003949 -0.007655
0.014096 -0.008626 -0.009527 0.001340 0.013776 0.001516
0.006493 -0.003568 0.010272 0.015903 0.016837 -0.022102 -
0.001537 -0.024322 -0.019712 -0.012414 -0.001266 0.024785 -
0.000790 -0.003147 0.006829 -0.004469 -0.004276 0.013890 -
0.024512 -0.002684
x652 0.013320 -0.016936 -0.003772 -0.009659 0.016725 -
0.008889 0.011437 -0.008978 0.015926 -0.009969 0.001124 -
0.019837 -0.003538 0.012787 -0.013639 0.023983 0.003354 -
0.005459 0.020325 -0.006670 0.010580 -0.022229 0.014487 -
0.010563 -0.003538 -0.009785 -0.034008 -0.020145 0.009385 -
0.018164 -0.008183 0.014289 0.006809 0.002658 -0.000510 -
0.001467 0.014877
y652 0.010899 -0.019945 0.006598 0.012177 -0.001377
0.003219 -0.027573 -0.007956 0.006077 0.015806 -0.006317 -

0.011682	0.010047	0.002685	-0.006902	0.019799	0.005487	
0.011547	0.017806	0.001014	0.011631	-0.023657	-0.010581	-
0.022165	-0.005619	-0.000254	0.012487	-0.003125	-0.003479	
0.002260	-0.013475	-0.001554	-0.012063	-0.010901	-0.014199	-
0.013532	-0.002447					
z652	0.009134	-0.010452	-0.001609	-0.016998	0.002645	
0.003338	-0.002713	-0.038637	0.011132	0.006027	-0.009120	
0.015785	-0.007033	-0.012350	0.003065	0.014475	-0.000599	
0.004105	-0.007105	0.009681	0.009010	0.023219	-0.023127	
0.001519	-0.019588	-0.016068	-0.014277	0.003416	0.021426	
0.001605	-0.007266	0.009294	-0.006694	-0.002395	0.009188	-
0.025996	-0.001147					
x653	0.013486	-0.018751	-0.002693	-0.012565	0.016396	-
0.009595	0.011666	-0.010596	0.015534	-0.009133	-0.001127	-
0.017791	-0.003278	0.011276	-0.017127	0.024952	0.004161	-
0.006132	0.022291	-0.005020	0.013354	-0.018952	0.017501	-
0.011220	-0.005518	-0.011127	-0.037980	-0.018542	0.009587	-
0.015964	-0.007607	0.016294	0.003521	0.005920	-0.000819	-
0.006219	0.014901					
y653	0.010225	-0.020101	0.006827	0.010996	-0.005074	
0.004388	-0.029715	-0.009318	0.003048	0.012238	-0.007578	-
0.010956	0.006361	0.001945	-0.006429	0.022732	0.006642	
0.009772	0.020847	0.004233	0.013224	-0.021304	-0.012921	-
0.021227	-0.006894	-0.002790	0.013051	0.001211	-0.001418	-
0.000374	-0.017750	0.000724	-0.012766	-0.011998	-0.018938	-
0.020669	0.001518					
z653	0.008352	-0.006865	0.000533	-0.018074	0.001642	-
0.000435	-0.003807	-0.035087	0.009731	0.007501	-0.009596	
0.017383	-0.005534	-0.015469	0.005310	0.015193	-0.001843	
0.003254	-0.009884	0.009454	0.002784	0.027632	-0.024130	
0.004063	-0.015927	-0.012237	-0.015776	0.007711	0.018923	
0.002707	-0.009448	0.011843	-0.007930	-0.001560	0.004783	-
0.026188	-0.000104					
x654	0.012825	-0.019544	-0.001258	-0.015508	0.014875	-
0.009799	0.010513	-0.012122	0.014442	-0.007142	-0.003691	-
0.015835	-0.004540	0.010698	-0.018523	0.027022	0.006431	-
0.005654	0.022106	-0.002397	0.016271	-0.013734	0.016896	-
0.009794	-0.010136	-0.011964	-0.041238	-0.017703	0.008958	-
0.011796	-0.009805	0.018428	0.000822	0.008865	-0.006450	-
0.010174	0.016900					
y654	0.010203	-0.019869	0.007243	0.010356	-0.007739	
0.005261	-0.029792	-0.009650	0.001267	0.008273	-0.007481	-
0.009057	0.005893	0.000266	-0.007911	0.022860	0.005766	
0.007089	0.022848	0.005675	0.014725	-0.019568	-0.013538	-
0.021321	-0.005099	-0.003931	0.015169	0.005801	-0.000283	-
0.002985	-0.018434	0.001437	-0.013286	-0.012524	-0.015573	-
0.024285	-0.000745					
z654	0.007737	-0.003312	0.001798	-0.017844	0.001689	-
0.003005	-0.004961	-0.031499	0.009140	0.008507	-0.009000	
0.018439	-0.003996	-0.018028	0.008306	0.015794	-0.003089	
0.003226	-0.012059	0.008836	-0.002476	0.030449	-0.025396	
0.006219	-0.012509	-0.008405	-0.016454	0.011700	0.017008	
0.002259	-0.009483	0.014303	-0.007911	-0.002321	0.001193	-
0.025146	0.000440					
x655	0.012377	-0.019540	-0.000046	-0.017618	0.013523	-
0.009808	0.009710	-0.012281	0.013805	-0.005378	-0.004943	-
0.014459	-0.005330	0.007811	-0.019689	0.027269	0.007666	-
0.006787	0.021479	-0.000461	0.018994	-0.010316	0.014432	-
0.009768	-0.012113	-0.012782	-0.041713	-0.016049	0.008326	-

0.009686 -0.012005 0.021063 -0.000726 0.009883 -0.009104 -
0.013331 0.016062
y655 0.009238 -0.019445 0.007796 0.009203 -0.010657
0.006825 -0.029521 -0.011243 -0.001431 0.005042 -0.008356 -
0.004784 0.005509 0.000648 -0.008637 0.022706 0.006207
0.008201 0.023126 0.006451 0.015518 -0.013943 -0.015305 -
0.020616 -0.006120 -0.003050 0.017206 0.007638 -0.000444 -
0.001413 -0.019216 -0.000787 -0.014156 -0.012394 -0.014858 -
0.024949 -0.001394
z655 0.007047 0.000283 0.002698 -0.016581 0.002366 -
0.004663 -0.006104 -0.028023 0.008647 0.009344 -0.008286
0.020245 -0.002174 -0.019572 0.010744 0.016786 -0.003334
0.005973 -0.013669 0.008672 -0.006036 0.032575 -0.026401
0.009931 -0.011197 -0.003573 -0.016124 0.013912 0.015699
0.002278 -0.006945 0.015394 -0.008492 -0.001022 -0.002536 -
0.023001 0.000684
x656 0.011167 -0.018138 0.001421 -0.019112 0.011873 -
0.009645 0.009321 -0.012792 0.012259 -0.003886 -0.007205 -
0.011305 -0.005677 0.005516 -0.020593 0.027107 0.009012 -
0.006408 0.019481 0.001051 0.020185 -0.005576 0.010716 -
0.009335 -0.014461 -0.011134 -0.039348 -0.014178 0.006905 -
0.006299 -0.014197 0.021195 -0.003505 0.010128 -0.010646 -
0.015217 0.012710
y656 0.008586 -0.018854 0.008828 0.007871 -0.013800
0.008705 -0.029733 -0.011066 -0.002776 0.003030 -0.007550 -
0.001676 0.006242 0.000348 -0.009420 0.021483 0.006945
0.007066 0.021336 0.007021 0.017766 -0.009325 -0.017764 -
0.018677 -0.006027 -0.004089 0.018179 0.009921 -0.001573
0.000970 -0.019853 -0.001405 -0.013761 -0.011356 -0.015061 -
0.023871 -0.001676
z656 0.006579 0.003886 0.003151 -0.014180 0.003728 -
0.005100 -0.007682 -0.024140 0.009039 0.009871 -0.006609
0.021199 -0.000050 -0.020831 0.013567 0.017789 -0.003186
0.009286 -0.015145 0.008514 -0.008147 0.032842 -0.027115
0.014325 -0.010696 0.001080 -0.015541 0.016259 0.015016
0.000534 -0.001937 0.016654 -0.008379 0.000276 -0.006557 -
0.019438 0.000998
x657 0.009281 -0.016156 0.003254 -0.020405 0.009716 -
0.009859 0.008331 -0.012214 0.010500 -0.002555 -0.008836 -
0.007903 -0.005523 0.003350 -0.021233 0.027066 0.010563 -
0.003770 0.014758 0.001406 0.023807 0.000897 0.006397 -
0.006445 -0.017078 -0.009884 -0.036930 -0.013179 0.004681 -
0.001342 -0.016202 0.018915 -0.007660 0.012806 -0.013483 -
0.013934 0.008913
y657 0.008137 -0.017412 0.010150 0.006818 -0.016822
0.011474 -0.028865 -0.011146 -0.003622 0.002385 -0.007326
0.002189 0.007434 -0.000458 -0.010377 0.018559 0.008004
0.003888 0.019647 0.008210 0.017371 -0.005766 -0.022631 -
0.018289 -0.005427 -0.003295 0.022016 0.012971 -0.003616
0.003655 -0.020407 -0.000927 -0.010909 -0.012963 -0.012556 -
0.023391 -0.003777
z657 0.006475 0.007499 0.002975 -0.010228 0.005975 -
0.004079 -0.009159 -0.020483 0.010033 0.010285 -0.004248
0.021869 0.002040 -0.021913 0.017225 0.018389 -0.002761
0.013237 -0.015423 0.008360 -0.010537 0.030967 -0.028960
0.017054 -0.010844 0.006043 -0.013945 0.018224 0.015104 -
0.003209 0.004729 0.018167 -0.006356 -0.000536 -0.009828 -
0.015194 0.000530
x658 0.007256 -0.013634 0.005104 -0.021264 0.007382 -
0.009814 0.007426 -0.010916 0.009397 0.000254 -0.011294 -

0.003661	-0.004250	0.001065	-0.020837	0.026594	0.012386	-
0.001256	0.009675	0.001080	0.027888	0.007534	-0.001418	-
0.003889	-0.018206	-0.010241	-0.031508	-0.011253	0.002109	
0.003456	-0.018632	0.013968	-0.007305	0.016104	-0.016031	-
0.013466	0.002199					
y658	0.007161	-0.015101	0.011716	0.005678	-0.019963	
0.014608	-0.027851	-0.011205	-0.005598	0.000162	-0.005227	
0.006167	0.007822	-0.001540	-0.012172	0.014646	0.008751	
0.001954	0.015081	0.009604	0.016022	-0.000360	-0.025527	-
0.015886	-0.006768	0.001891	0.025266	0.013794	-0.006336	
0.009124	-0.019193	0.002129	-0.013949	-0.015368	-0.009345	-
0.017342	-0.004345					
z658	0.006428	0.011346	0.002405	-0.005553	0.009044	-
0.001999	-0.010813	-0.016685	0.011205	0.009952	-0.000516	
0.022324	0.004019	-0.022409	0.020517	0.019172	-0.001456	
0.018626	-0.015870	0.009109	-0.011666	0.028095	-0.029590	
0.021525	-0.013143	0.012812	-0.011462	0.018938	0.016197	-
0.007151	0.014284	0.019892	-0.005156	0.000007	-0.012858	-
0.009187	0.001041					
x659	0.004823	-0.009752	0.008276	-0.022167	0.004876	-
0.009197	0.006131	-0.008588	0.009033	0.004252	-0.011362	
0.001322	-0.001327	-0.001397	-0.021919	0.023439	0.017002	
0.000916	0.003372	0.001543	0.029856	0.015249	-0.011098	
0.000184	-0.021530	-0.008170	-0.023009	-0.010303	-0.003144	
0.007936	-0.020441	0.008365	-0.008490	0.017157	-0.017921	-
0.010910	-0.006258					
y659	0.006098	-0.012560	0.012127	0.005019	-0.023015	
0.017947	-0.025738	-0.011358	-0.008587	-0.002597	-0.005374	
0.010885	0.007405	-0.002441	-0.011736	0.012435	0.005962	
0.001247	0.008989	0.009265	0.017107	0.004855	-0.028304	-
0.014691	-0.005095	0.006083	0.027182	0.015777	-0.007399	
0.016754	-0.018951	0.003883	-0.014570	-0.014883	-0.005848	-
0.010488	-0.005394					
z659	0.006572	0.015330	0.000840	0.000296	0.012918	
0.001451	-0.012143	-0.013183	0.012471	0.009054	0.003546	
0.022632	0.005758	-0.022345	0.024721	0.020329	-0.000904	
0.025802	-0.015115	0.009207	-0.010982	0.023308	-0.029846	
0.025202	-0.015441	0.019602	-0.009359	0.020074	0.018965	-
0.012399	0.025302	0.022136	-0.003424	-0.000198	-0.015397	-
0.001800	0.000414					
x660	-0.000600	-0.005189	0.007695	-0.023799	0.004021	-
0.008813	0.009473	-0.008514	0.009339	0.006922	-0.013108	
0.007576	0.000347	-0.000164	-0.017142	0.024782	0.014084	
0.003223	0.005142	0.000271	0.028673	0.009861	-0.020612	
0.000714	-0.015496	-0.010322	-0.022228	-0.011862	0.002857	
0.006385	-0.019816	0.012093	-0.007295	0.014398	-0.018438	-
0.006334	-0.008815					
y660	0.002978	-0.007290	0.013299	0.000418	-0.023270	
0.019614	-0.022311	-0.008243	-0.007517	-0.002343	-0.005284	
0.016122	0.010431	0.000348	-0.008756	0.016408	0.006507	-
0.002256	0.010249	0.009932	0.015201	0.001590	-0.032860	-
0.016316	0.000809	0.012829	0.029303	0.018578	-0.006230	
0.018002	-0.015580	0.003002	-0.012312	-0.010103	-0.004397	-
0.008991	-0.004135					
z660	0.015130	0.010766	-0.004263	0.011433	0.014065	
0.001840	-0.015686	-0.013136	0.016641	0.013469	0.012561	
0.010960	0.003217	-0.025444	0.026271	0.013793	-0.005455	
0.023885	-0.014370	0.009315	-0.007231	0.023957	-0.024390	
0.031003	-0.020856	0.016682	-0.009153	0.009289	0.021427	-

0.017081	0.022203	0.020789	-0.004694	-0.006560	-0.006802	-
0.003914	-0.002503					
x661	-0.004016	-0.001859	0.007232	-0.023845	0.004052	-
0.008704	0.010587	-0.007602	0.011351	0.011865	-0.012378	
0.012401	0.002261	0.000318	-0.014425	0.025112	0.011570	
0.005493	0.006994	-0.001107	0.030473	0.005946	-0.027220	
0.003581	-0.012897	-0.009958	-0.019757	-0.013098	0.005476	
0.005548	-0.018592	0.013824	-0.005584	0.012332	-0.017001	-
0.003984	-0.009404					
y661	0.000960	-0.003217	0.012652	-0.002574	-0.024359	
0.022024	-0.019333	-0.006577	-0.007934	-0.004377	-0.005401	
0.018835	0.011142	0.001166	-0.004179	0.018572	0.006550	-
0.004134	0.009564	0.010878	0.010364	-0.001407	-0.036717	-
0.019699	0.007136	0.015115	0.029806	0.020405	-0.001663	
0.018726	-0.013008	0.003444	-0.010621	-0.006997	-0.003946	-
0.004267	-0.005229					
z661	0.022996	0.006331	-0.009170	0.020976	0.014881	
0.002195	-0.018065	-0.013915	0.019077	0.015942	0.020373	
0.000179	0.000756	-0.028317	0.027929	0.007935	-0.009385	
0.022527	-0.014356	0.008687	-0.005715	0.023052	-0.019840	
0.033648	-0.024898	0.012862	-0.009142	0.000844	0.023909	-
0.020280	0.018756	0.020569	-0.006470	-0.012880	0.000380	-
0.004441	-0.005909					
x662	-0.005134	0.000394	0.005754	-0.022297	0.004009	-
0.008341	0.009807	-0.006056	0.013338	0.015379	-0.011965	
0.015517	0.003153	0.000225	-0.010862	0.024656	0.010045	
0.007608	0.008421	-0.001004	0.030791	0.002518	-0.031385	
0.006081	-0.009874	-0.009893	-0.015841	-0.014188	0.009696	
0.006436	-0.016191	0.015733	-0.004391	0.010804	-0.012409	-
0.003045	-0.012993					
y662	-0.000330	-0.000366	0.012109	-0.004060	-0.024795	
0.023891	-0.017637	-0.005916	-0.008401	-0.003501	-0.003678	
0.020297	0.010983	0.000311	-0.000727	0.018963	0.005195	-
0.005594	0.008850	0.010396	0.009722	-0.002468	-0.039587	-
0.020449	0.009688	0.017617	0.029468	0.021169	-0.000277	
0.017694	-0.011191	0.003044	-0.008877	-0.004569	-0.006781	
0.003529	-0.001742					
z662	0.029882	0.002096	-0.012737	0.028968	0.015741	
0.001466	-0.019514	-0.014898	0.020589	0.018322	0.027451	-
0.009288	-0.001179	-0.030839	0.028284	0.003435	-0.012289	
0.021578	-0.014900	0.007150	-0.003332	0.021664	-0.015525	
0.035887	-0.029577	0.009281	-0.009110	-0.005402	0.023413	-
0.022285	0.015325	0.019516	-0.009359	-0.016723	0.005267	-
0.003957	-0.006790					
x663	-0.003794	0.001949	0.003439	-0.019703	0.004554	-
0.006867	0.007687	-0.004658	0.013849	0.018685	-0.009462	
0.017706	0.002447	0.000045	-0.006897	0.022663	0.010128	
0.006846	0.009697	0.000986	0.032359	-0.000144	-0.033405	
0.007142	-0.007948	-0.009393	-0.010605	-0.016369	0.014475	
0.007635	-0.011449	0.019312	-0.004533	0.008668	-0.008000	-
0.000965	-0.017209					
y663	-0.001083	0.000759	0.011064	-0.003599	-0.025155	
0.024292	-0.017728	-0.005380	-0.006752	-0.002180	-0.004490	
0.020150	0.010975	-0.002253	0.000784	0.017245	0.001625	-
0.002926	0.006449	0.009235	0.008228	-0.003668	-0.040962	-
0.019229	0.010582	0.020212	0.029211	0.021921	0.000341	
0.016425	-0.013257	-0.000589	-0.007275	-0.003926	-0.007390	
0.009629	-0.000106					
z663	0.035713	-0.001980	-0.015491	0.035320	0.016297	-
0.000254	-0.020186	-0.015812	0.021820	0.019918	0.032773	-

0.017464	-0.002616	-0.032737	0.026610	0.000253	-0.014777	
0.022602	-0.016376	0.005783	-0.002003	0.018917	-0.011003	
0.037358	-0.034375	0.005667	-0.008847	-0.008939	0.021454	-
0.023401	0.011317	0.016759	-0.012332	-0.019262	0.009813	-
0.003550	-0.006368					
x664	-0.000706	0.001448	0.000215	-0.013835	0.004116	-
0.005796	0.003940	-0.003179	0.015866	0.022829	-0.006401	
0.015405	0.000561	-0.004128	-0.002802	0.018957	0.007600	
0.010106	0.008767	0.001990	0.033824	-0.002682	-0.033755	
0.008422	-0.009872	-0.010782	-0.006227	-0.017346	0.015959	
0.007863	-0.009851	0.020822	-0.001077	0.003136	-0.002124	
0.002814	-0.016477					
y664	-0.000439	0.001700	0.009724	-0.002964	-0.023542	
0.025531	-0.020110	-0.005504	-0.005743	-0.000072	-0.002558	
0.020068	0.009794	-0.003823	0.001129	0.015830	-0.000327	-
0.003802	0.006214	0.008506	0.009972	-0.002112	-0.039808	-
0.016761	0.010855	0.024637	0.030965	0.021275	0.002808	
0.015434	-0.011527	-0.002873	-0.010016	0.000234	-0.006320	
0.012976	-0.003298					
z664	0.040733	-0.005579	-0.017457	0.039764	0.017438	-
0.002567	-0.019863	-0.016853	0.021409	0.020607	0.037225	-
0.023615	-0.003432	-0.033113	0.023963	-0.001233	-0.015306	
0.021944	-0.017441	0.003777	-0.000468	0.015777	-0.007241	
0.037109	-0.038231	0.002810	-0.007777	-0.010234	0.018657	-
0.022075	0.008893	0.014370	-0.018257	-0.018246	0.012201	-
0.003309	-0.007228					
x665	0.004136	-0.000284	-0.003369	-0.004774	0.003669	-
0.006299	-0.003187	-0.000564	0.016530	0.025467	-0.004402	
0.011281	-0.004070	-0.007616	0.001069	0.016767	0.004732	
0.012144	0.006858	0.003436	0.037321	-0.002909	-0.031690	
0.011841	-0.011600	-0.011117	0.001137	-0.017332	0.018288	
0.011196	-0.004228	0.020896	-0.002178	0.002714	0.003786	
0.006675	-0.016621					
y665	0.001199	0.001313	0.007105	-0.003823	-0.022019	
0.028858	-0.020856	-0.006748	-0.002558	0.004127	0.001440	
0.019373	0.011174	-0.008478	0.001006	0.010420	-0.001561	-
0.002074	0.006456	0.007832	0.009728	-0.001268	-0.037388	-
0.015190	0.008374	0.027975	0.029482	0.016888	0.002955	
0.011113	-0.015203	-0.003285	-0.008015	-0.004444	-0.004972	
0.017252	-0.005965					
z665	0.045069	-0.008901	-0.018773	0.041849	0.018081	-
0.004997	-0.018389	-0.018060	0.020902	0.020915	0.041189	-
0.028085	-0.003211	-0.033572	0.020256	-0.001617	-0.014926	
0.023386	-0.019105	0.002243	-0.000652	0.011302	-0.003639	
0.035170	-0.041069	-0.000328	-0.007246	-0.009625	0.013785	-
0.020885	0.005680	0.011556	-0.022655	-0.017869	0.013475	-
0.001996	-0.005802					
x666	0.011812	-0.003042	-0.008775	0.004400	0.004377	-
0.004418	-0.011288	0.001669	0.017720	0.028875	0.000985	
0.006654	-0.007607	-0.011596	0.005829	0.009310	0.004311	
0.012388	0.007056	0.005930	0.038765	-0.004798	-0.026966	
0.010249	-0.014590	-0.009360	0.007604	-0.017409	0.021022	
0.015287	0.001086	0.023707	-0.000457	-0.003519	0.009655	
0.012257	-0.017741					
y666	0.003412	-0.000009	0.005907	-0.001243	-0.021048	
0.029636	-0.023740	-0.007803	-0.000728	0.007540	0.002929	
0.016487	0.008020	-0.014887	-0.000190	0.008635	-0.005036	
0.001434	0.002448	0.008064	0.013599	0.004529	-0.036455	-
0.007235	0.006417	0.027849	0.031642	0.013388	0.004878	

0.005039 -0.016113 -0.009548 -0.009846 0.000176 -0.001043
0.019235 -0.007848
z666 0.048417 -0.012031 -0.019031 0.043142 0.018478 -
0.008422 -0.015962 -0.019473 0.018839 0.019932 0.043379 -
0.032015 -0.003371 -0.033996 0.015984 -0.000167 -0.013609
0.025473 -0.021023 -0.000295 -0.000520 0.006379 -0.001197
0.031869 -0.043728 -0.004026 -0.005910 -0.006401 0.007260 -
0.017678 0.001652 0.008421 -0.027388 -0.015145 0.013799 -
0.000920 -0.004012
x667 0.021009 -0.007000 -0.013024 0.014845 0.004279 -
0.003470 -0.021054 0.004419 0.020169 0.034177 0.007182 -
0.000976 -0.012707 -0.018570 0.005254 0.002179 0.003156
0.015351 0.002818 0.010135 0.045399 -0.005887 -0.017676
0.016171 -0.023196 -0.007981 0.015007 -0.018532 0.022261
0.017708 0.005825 0.023626 -0.000287 -0.006469 0.017659
0.017317 -0.014152
y667 0.006974 -0.002560 0.001677 0.004044 -0.018436
0.031105 -0.028086 -0.010510 0.000826 0.010902 0.007848
0.011127 0.004454 -0.020868 0.003260 0.003626 -0.008415
0.004072 0.001964 0.006428 0.013995 0.011488 -0.037082 -
0.005270 0.003192 0.026633 0.033789 0.009744 0.004532
0.000529 -0.018852 -0.013889 -0.008225 -0.002873 0.003407
0.020804 -0.013967
z667 0.050842 -0.014829 -0.018584 0.042809 0.019098 -
0.012915 -0.012525 -0.020936 0.015249 0.017796 0.044260 -
0.034173 -0.002967 -0.032659 0.010791 0.003059 -0.010892
0.026972 -0.023111 -0.002972 -0.000746 0.000640 0.000633
0.027717 -0.046227 -0.007405 -0.003934 -0.001306 -0.000797 -
0.011841 -0.001414 0.005706 -0.034118 -0.010184 0.012334
0.000260 -0.002967
x668 0.032002 -0.012929 -0.019952 0.028184 0.004203 -
0.003074 -0.033109 0.007181 0.020571 0.036524 0.011133 -
0.010533 -0.020676 -0.025836 0.008069 -0.004631 -0.000509
0.017345 -0.002834 0.013973 0.050520 -0.004380 -0.009642
0.018611 -0.030648 -0.006170 0.025524 -0.022014 0.025154
0.020726 0.010722 0.022195 -0.002154 -0.011328 0.025239
0.023750 -0.010299
y668 0.012339 -0.004907 -0.000598 0.010006 -0.015834
0.032003 -0.033549 -0.011950 0.004911 0.017839 0.015537
0.005810 0.002718 -0.028452 0.003331 -0.002617 -0.007870
0.008262 0.004634 0.005637 0.018311 0.016609 -0.031954
0.001498 -0.001313 0.024458 0.033482 0.008886 0.002205 -
0.000308 -0.021472 -0.013238 -0.003676 -0.002963 0.008280
0.021198 -0.021950
z668 0.052596 -0.017292 -0.016978 0.040186 0.019992 -
0.017346 -0.007316 -0.022784 0.010828 0.015420 0.045187 -
0.034705 -0.001469 -0.031358 0.003797 0.006818 -0.007045
0.029076 -0.025969 -0.005695 -0.002429 -0.007245 0.002162
0.021520 -0.049049 -0.010529 -0.002455 0.006519 -0.010787 -
0.006513 -0.003923 0.001640 -0.041875 -0.004182 0.010482
0.002093 -0.001281
x669 0.041292 -0.020315 -0.022439 0.036553 0.007904 -
0.003785 -0.033357 0.011150 0.019688 0.034911 0.010841 -
0.014313 -0.025049 -0.023061 0.008189 -0.005449 0.000439
0.012219 -0.001763 0.015818 0.049948 -0.014082 -0.002824
0.021035 -0.030201 -0.006677 0.024442 -0.019651 0.023371
0.014439 0.006099 0.028166 -0.005928 -0.012453 0.025252
0.026207 -0.019371
y669 0.016345 -0.008582 -0.002603 0.014826 -0.012546
0.031873 -0.036165 -0.010529 0.004656 0.016563 0.017809

0.004258 0.000700 -0.028598 0.002574 -0.002341 -0.006670
0.006974 0.004732 0.007246 0.016160 0.011806 -0.025944
0.000145 0.004182 0.026937 0.034820 0.013498 0.002117 -
0.003241 -0.013117 -0.015031 -0.007395 -0.001626 0.009148
0.019726 -0.031067
z669 0.043807 -0.019138 -0.009250 0.031260 0.022387 -
0.021230 0.008157 -0.020750 0.007394 0.001882 0.037625 -
0.026753 0.004642 -0.020189 0.003043 0.008781 0.001562
0.028865 -0.038493 -0.012099 -0.012590 -0.011707 0.002788
0.014898 -0.036214 -0.011797 -0.013519 0.005480 -0.016688 -
0.008329 -0.020574 -0.004613 -0.032319 0.004135 0.007957 -
0.004044 0.000431
x670 0.047976 -0.026579 -0.024321 0.042150 0.010140 -
0.004387 -0.032014 0.013884 0.016499 0.029967 0.010108 -
0.015279 -0.027277 -0.019693 0.006118 -0.004391 0.001825
0.006638 -0.000593 0.017684 0.046412 -0.022841 0.005503
0.020289 -0.027452 -0.004861 0.025503 -0.016162 0.021825
0.010412 0.005680 0.033525 -0.010286 -0.011084 0.023520
0.027467 -0.027734
y670 0.018506 -0.013500 -0.002799 0.019192 -0.008975
0.030350 -0.035752 -0.009873 0.005732 0.017220 0.019428
0.000722 -0.000307 -0.026824 0.002060 -0.003469 -0.004258
0.007244 0.001261 0.007021 0.015585 0.008755 -0.025096
0.000743 0.007630 0.024122 0.034655 0.013230 -0.000095 -
0.009176 -0.012059 -0.019426 -0.006234 -0.002004 0.011433
0.014568 -0.034205
z670 0.034041 -0.018070 -0.003247 0.020929 0.023796 -
0.023608 0.020953 -0.020108 0.003812 -0.008758 0.030386 -
0.019027 0.010352 -0.009058 0.001922 0.011633 0.007913
0.028977 -0.046894 -0.015879 -0.022086 -0.014944 0.003224
0.008950 -0.026268 -0.011925 -0.020104 0.003666 -0.020413 -
0.008600 -0.031635 -0.008780 -0.026205 0.009151 0.005231 -
0.006765 0.000870
x671 0.051307 -0.033237 -0.024394 0.046618 0.010624 -
0.006620 -0.028198 0.013924 0.014451 0.025677 0.010628 -
0.018510 -0.028929 -0.018330 0.000652 -0.002778 0.001242
0.006636 -0.003827 0.017618 0.043615 -0.028395 0.012555
0.021586 -0.028808 -0.005710 0.025761 -0.013047 0.015451
0.004669 0.001586 0.034376 -0.010953 -0.010675 0.023008
0.025555 -0.030385
y671 0.020056 -0.016652 -0.003009 0.021965 -0.005742
0.030509 -0.033482 -0.008809 0.003743 0.015255 0.019330
0.001678 0.002051 -0.025179 0.000338 -0.003747 -0.002090
0.006079 0.000875 0.005108 0.014448 0.003722 -0.022624
0.000560 0.014447 0.022583 0.035201 0.013527 0.001669 -
0.009604 -0.006963 -0.017372 -0.009234 0.000159 0.010688
0.012812 -0.037285
z671 0.023685 -0.014783 0.001356 0.009742 0.023950 -
0.025503 0.031453 -0.021355 0.000841 -0.015249 0.025195 -
0.012708 0.015287 0.000152 0.000048 0.014385 0.011039
0.030748 -0.052878 -0.016949 -0.030054 -0.017197 0.001772
0.005070 -0.020440 -0.013254 -0.023730 0.000917 -0.022759 -
0.008762 -0.040040 -0.011592 -0.021639 0.011225 0.003208 -
0.006113 0.002967
x672 0.052638 -0.039302 -0.023415 0.048863 0.010939 -
0.009284 -0.021832 0.013129 0.010266 0.018562 0.009486 -
0.018033 -0.028114 -0.015846 -0.006221 -0.000635 0.002377
0.006903 -0.008504 0.017574 0.038801 -0.032169 0.018575
0.019683 -0.026168 -0.007116 0.028008 -0.011378 0.011667

0.002559 0.001224 0.034016 -0.013878 -0.006408 0.021278
0.021184 -0.032332
y672 0.019229 -0.019505 -0.002553 0.023278 -0.004861
0.027405 -0.030890 -0.010120 0.003987 0.015773 0.020643 -
0.001766 0.004247 -0.023340 -0.002863 -0.002658 0.000248
0.006589 -0.001080 0.001047 0.012180 -0.002361 -0.019731
0.002632 0.015999 0.021089 0.035004 0.013550 -0.003477 -
0.011835 -0.006885 -0.019018 -0.007739 -0.001268 0.005258
0.010244 -0.036574
z672 0.012690 -0.008835 0.004273 -0.002854 0.023817 -
0.025508 0.039347 -0.023331 -0.002390 -0.019049 0.020495 -
0.005026 0.019743 0.009115 -0.001450 0.017519 0.012418
0.032451 -0.055881 -0.015332 -0.037462 -0.018443 -0.001794
0.001589 -0.017346 -0.015729 -0.023684 -0.003028 -0.021270 -
0.006998 -0.043093 -0.011252 -0.019891 0.009949 0.000785 -
0.002403 0.004537
x673 0.051240 -0.045403 -0.021128 0.049556 0.009128 -
0.013367 -0.013094 0.010859 0.004965 0.010733 0.006782 -
0.016856 -0.026158 -0.013098 -0.014818 0.002795 0.002319
0.008799 -0.014494 0.015155 0.032522 -0.035353 0.022924
0.019015 -0.024576 -0.010934 0.029025 -0.010079 0.004147
0.002847 -0.000269 0.031668 -0.014450 -0.000231 0.017632
0.015829 -0.033273
y673 0.016931 -0.021572 -0.000709 0.022863 -0.003384
0.023968 -0.025601 -0.012447 0.004393 0.014595 0.022466 -
0.003107 0.008297 -0.022355 -0.008690 -0.000670 0.003700
0.009039 -0.006055 -0.001498 0.009691 -0.006323 -0.016959
0.001570 0.018510 0.019331 0.038550 0.011708 -0.004625 -
0.013249 -0.004767 -0.020383 -0.010229 -0.003621 0.000079
0.005078 -0.032306
z673 0.000906 -0.000156 0.005356 -0.016818 0.022697 -
0.024627 0.044764 -0.026943 -0.005005 -0.019501 0.016677
0.001624 0.023381 0.016989 -0.003064 0.021505 0.011061
0.035589 -0.054600 -0.011518 -0.043559 -0.019104 -0.006126 -
0.001349 -0.017571 -0.018238 -0.020892 -0.008926 -0.018004 -
0.002762 -0.044320 -0.008186 -0.019752 0.004630 -0.002171
0.005214 0.007434
x674 0.047532 -0.051381 -0.017519 0.048785 0.006696 -
0.019098 -0.001650 0.007246 -0.000907 -0.000480 0.003461 -
0.014883 -0.021997 -0.010613 -0.025417 0.007624 0.002762
0.012792 -0.023608 0.008633 0.024667 -0.037945 0.025433
0.014545 -0.021502 -0.015903 0.030463 -0.008195 -0.003455
0.005348 -0.004094 0.032275 -0.016258 0.005268 0.009364
0.009291 -0.029276
y674 0.011621 -0.023347 0.002076 0.019874 -0.003602
0.020527 -0.015792 -0.017407 0.004021 0.014193 0.022248 -
0.002146 0.013056 -0.020342 -0.016636 0.002923 0.004904
0.014537 -0.011449 -0.002450 0.006111 -0.009821 -0.014240
0.004288 0.019622 0.013553 0.040137 0.010990 -0.007906 -
0.011840 -0.000832 -0.022642 -0.012599 -0.004747 -0.003323
0.001593 -0.030082
z674 -0.012468 0.012133 0.004142 -0.032408 0.020055 -
0.022073 0.047529 -0.032064 -0.007514 -0.016320 0.013024
0.007827 0.027442 0.023655 -0.004704 0.026722 0.006638
0.040945 -0.049661 -0.004599 -0.049556 -0.018544 -0.012407 -
0.005991 -0.021104 -0.024093 -0.013173 -0.017294 -0.012418
0.003458 -0.041763 -0.001857 -0.019576 -0.005259 -0.004172
0.015670 0.007811
x675 0.041014 -0.058252 -0.011859 0.044038 0.001388 -
0.025048 0.014643 0.000080 -0.008012 -0.013552 -0.000237 -

0.009183 -0.015484 -0.007153 -0.040476 0.011316 -0.000187
0.022615 -0.037762 -0.001645 0.012865 -0.038977 0.026220
0.012652 -0.019658 -0.023077 0.028976 -0.002197 -0.011665
0.006819 -0.008599 0.026896 -0.019352 0.011127 0.000771
0.001651 -0.023878
y675 0.003643 -0.023687 0.006365 0.016965 -0.003397
0.012713 -0.002056 -0.022536 0.003046 0.011423 0.020623 -
0.000612 0.020356 -0.017373 -0.026828 0.012134 0.009673
0.019787 -0.016705 -0.005380 0.001367 -0.016025 -0.015560
0.002029 0.021569 0.003070 0.046438 0.008799 -0.014809 -
0.006332 0.004331 -0.019861 -0.016630 -0.004060 -0.011043 -
0.004784 -0.025737
z675 -0.028531 0.028989 0.000128 -0.050916 0.015945 -
0.016725 0.049601 -0.039947 -0.010266 -0.008810 0.010757
0.013059 0.032152 0.028813 -0.007380 0.032018 0.000606
0.047286 -0.038949 0.006359 -0.055597 -0.015665 -0.019753 -
0.013878 -0.029651 -0.035883 0.001331 -0.026639 -0.003853
0.011179 -0.036154 0.008540 -0.019723 -0.018459 -0.006403
0.030808 0.010824
x676 0.030166 -0.065389 -0.006736 0.035191 -0.006691 -
0.030403 0.036863 -0.009454 -0.019658 -0.029615 -0.003656 -
0.005376 -0.007103 -0.004252 -0.059343 0.009721 0.000537
0.031996 -0.051163 -0.014040 0.001878 -0.037354 0.026146
0.008086 -0.015471 -0.029955 0.028849 -0.004399 -0.028688
0.005533 -0.016802 0.023300 -0.010249 0.014069 -0.007132 -
0.014277 -0.011274
y676 -0.004864 -0.024797 0.014299 0.013458 -0.003510
0.003671 0.012547 -0.028934 0.002965 0.004692 0.017647
0.003128 0.028287 -0.012219 -0.041695 0.027325 0.007824
0.029755 -0.025088 -0.011003 -0.005796 -0.020861 -0.016358
0.005857 0.019211 -0.005112 0.049848 0.013640 -0.019574
0.002350 0.013516 -0.015301 -0.029104 -0.000510 -0.016479 -
0.004958 -0.024129
z676 -0.046778 0.048227 -0.006034 -0.071870 0.010216 -
0.009069 0.048255 -0.050150 -0.014093 0.001216 0.010243
0.017356 0.038055 0.033242 -0.011302 0.033455 -0.006084
0.055105 -0.024106 0.022111 -0.063191 -0.010661 -0.030277 -
0.021129 -0.042815 -0.046926 0.025723 -0.034749 0.009913
0.019109 -0.022933 0.021213 -0.017595 -0.038151 -0.005019
0.050996 0.016037
x677 -0.041270 -0.011665 0.023410 0.028042 0.002336 -
0.003073 -0.020737 -0.025874 -0.073171 0.021037 -0.027693 -
0.029391 0.016472 0.020754 -0.007469 -0.027208 0.020157
0.018560 0.049709 -0.008810 -0.014139 0.009497 0.007182 -
0.036442 -0.000838 0.024930 0.031875 0.024519 -0.027802 -
0.012136 -0.006085 -0.026975 0.014200 -0.005208 0.060602
0.024340 0.051044
y677 -0.000715 0.020774 -0.006606 -0.014907 -0.001308
0.000305 0.057202 0.009805 0.060754 -0.021107 0.005800
0.004833 -0.015149 -0.007158 0.000809 0.043044 -0.031769 -
0.017517 -0.054356 -0.025830 -0.012588 -0.004976 -0.005887
0.047683 0.025146 -0.014691 -0.030359 -0.000059 0.026061 -
0.011189 0.012405 0.047477 0.006633 0.023059 -0.006624
0.019560 0.027682
z677 -0.015961 0.003791 0.024872 0.008952 -0.006555 -
0.002525 -0.004710 -0.005365 0.019691 -0.019902 0.010691 -
0.025980 0.005924 0.003473 0.019281 -0.025958 0.016494
0.007713 0.033488 0.005697 -0.026378 0.032308 0.007444 -
0.024408 0.010043 0.019513 0.075795 -0.018471 -0.033128

0.001289	-0.003905	-0.028994	-0.000186	-0.017209	-0.011773	
0.025906	-0.033771					
x678	-0.031072	-0.013933	0.024324	0.030758	-0.001183	
0.006695	-0.021084	-0.022971	-0.075146	0.027627	-0.031773	-
0.024667	0.018223	0.032200	0.013935	-0.013524	0.020010	
0.022490	0.056971	-0.014126	-0.041751	-0.003254	0.006433	-
0.053458	-0.002326	0.017091	0.040037	0.027755	-0.047825	
0.001805	-0.003986	-0.041497	0.039299	-0.006597	0.055371	
0.015759	0.024901					
y678	-0.008999	0.015031	-0.004423	-0.009002	-0.009813	
0.000948	0.044750	0.005298	0.049121	-0.019678	-0.004442	
0.002971	-0.031503	-0.001914	-0.004048	0.023040	-0.015997	-
0.023017	-0.053022	-0.019219	-0.028393	-0.012191	-0.023193	
0.037961	0.027150	-0.015285	-0.026150	0.010827	0.017755	
0.002037	0.002971	0.033620	0.004066	0.034094	-0.015772	
0.011741	0.025016					
z678	-0.012209	0.002146	0.024054	0.012706	-0.003633	-
0.020384	0.002384	-0.004778	0.026861	-0.013981	0.015707	-
0.027244	0.006277	0.004791	0.027351	-0.020494	0.019848	
0.006216	0.036843	0.009034	-0.028695	0.028512	0.016442	-
0.023851	0.004062	0.015788	0.068132	-0.022232	-0.034769	
0.006291	0.002886	-0.025615	-0.021258	-0.024288	-0.009253	
0.014549	-0.031888					
x679	-0.018437	-0.017538	0.026871	0.031807	0.005902	
0.005641	-0.011361	-0.016614	-0.060650	0.031351	-0.026810	-
0.020816	0.015518	0.040546	0.043934	0.003090	0.014284	
0.014467	0.070954	-0.002197	-0.042373	-0.018938	0.019217	-
0.060152	-0.026520	0.018996	0.033020	0.039865	-0.043325	
0.001697	-0.004868	-0.037338	0.029833	-0.032700	0.060633	
0.010963	0.007910					
y679	-0.015269	0.007212	-0.002492	-0.006030	-0.011818	
0.000290	0.040828	0.004072	0.046270	-0.016482	-0.007037	
0.004995	-0.044931	0.000449	-0.003650	0.004227	-0.008593	-
0.037635	-0.037850	0.002300	-0.029992	-0.021853	-0.034054	
0.029431	0.005854	-0.013973	-0.037932	0.022088	0.012514	
0.003543	0.005631	0.032228	-0.011825	0.020132	-0.001504	-
0.000415	0.009218					
z679	-0.006184	0.001016	0.023901	0.014445	0.001028	-
0.036604	0.010335	-0.005100	0.037762	-0.006254	0.023373	-
0.029844	0.009627	0.006388	0.033847	-0.015940	0.022011	
0.001122	0.035315	0.013349	-0.029087	0.022969	0.027415	-
0.018691	-0.000288	0.014459	0.060687	-0.019242	-0.032924	
0.006363	0.008690	-0.028579	-0.038907	-0.026619	-0.008642	
0.007097	-0.027398					
x680	-0.003798	-0.013471	0.029921	0.033476	0.016094	
0.000910	-0.002702	-0.007955	-0.053296	0.027721	-0.019929	-
0.019234	0.029471	0.033927	0.056297	0.014495	0.003994	
0.013504	0.076643	-0.004147	-0.032369	-0.033244	0.030935	-
0.061620	-0.035802	0.018282	0.035564	0.037147	-0.030861	
0.009303	-0.009371	-0.018178	0.019120	-0.031983	0.049405	
0.037295	0.016496					
y680	-0.017563	0.004493	0.001377	0.000884	-0.009060	-
0.002500	0.036312	0.007522	0.036828	-0.019119	-0.007919	
0.006815	-0.039721	-0.012295	-0.019795	-0.012932	-0.009097	-
0.041526	-0.029696	0.010242	-0.020580	-0.030181	-0.043380	
0.025238	-0.000626	-0.009538	-0.040957	0.018428	0.009944	
0.016554	0.009850	0.046787	-0.028267	0.016481	0.003977	
0.005581	0.003487					
z680	-0.001458	-0.000277	0.023755	0.015499	0.005036	-
0.047644	0.016307	-0.007628	0.044669	0.001516	0.027486	-

0.032407	0.011848	0.009995	0.042071	-0.011543	0.022523	-
0.000529	0.030173	0.014858	-0.033085	0.017743	0.031159	-
0.012724	-0.001695	0.011697	0.057231	-0.010173	-0.028596	
0.004277	0.007981	-0.031645	-0.049024	-0.024458	-0.005802	
0.003037	-0.019653					
x681	0.004210	-0.006758	0.029394	0.028328	0.018401	
0.002927	-0.001470	-0.002509	-0.043007	0.028413	-0.016340	-
0.024857	0.031380	0.047835	0.082525	0.030211	-0.000739	
0.005514	0.073138	-0.009474	-0.030079	-0.035541	0.036259	-
0.055438	-0.055673	0.015890	0.021405	0.032540	-0.031418	
0.001953	-0.016775	-0.023336	0.018978	-0.038345	0.031558	
0.050502	0.015432					
y681	-0.023718	0.002567	0.001881	0.000559	-0.010907	-
0.000136	0.026540	0.007972	0.034158	-0.015415	-0.011309	-
0.000414	-0.044303	-0.002558	-0.022122	-0.018200	0.001977	-
0.050835	-0.028614	0.009134	-0.019108	-0.029553	-0.051963	
0.031160	-0.013723	-0.008552	-0.056663	0.015231	-0.008496	
0.012521	-0.000424	0.029261	-0.036093	0.000713	0.009997	
0.013150	0.005273					
z681	0.004603	-0.001739	0.024758	0.017511	0.008643	-
0.053881	0.020328	-0.009587	0.047000	0.008689	0.030246	-
0.034330	0.013838	0.010438	0.048171	-0.010174	0.018263	
0.000066	0.023862	0.019351	-0.038327	0.008903	0.031928	-
0.009871	-0.002183	0.013018	0.054831	0.002413	-0.021750	
0.005308	0.006220	-0.028217	-0.054563	-0.018607	-0.003774	-
0.002803	-0.017253					
x682	0.013568	-0.000441	0.028571	0.025898	0.020055	
0.004478	0.000874	0.001659	-0.039082	0.027893	-0.012510	-
0.024761	0.037452	0.048330	0.099474	0.039160	-0.010681	
0.006600	0.069991	-0.009570	-0.030522	-0.037609	0.039059	-
0.049990	-0.070503	0.011623	0.014118	0.030555	-0.023416	-
0.003378	-0.013647	-0.015096	0.014898	-0.032599	0.004788	
0.052318	0.022690					
y682	-0.026560	0.000539	0.004445	0.003525	-0.010710	
0.003893	0.017721	0.008731	0.027233	-0.008452	-0.016959	
0.001847	-0.041204	-0.006668	-0.032378	-0.026019	0.004288	-
0.050552	-0.024202	0.008420	-0.021290	-0.031493	-0.060179	
0.034933	-0.020322	-0.007870	-0.064103	0.009031	-0.022359	
0.009072	-0.000984	0.022202	-0.045256	-0.006631	0.016955	
0.021208	0.028501					
z682	0.009817	-0.003047	0.024742	0.019109	0.011081	-
0.056571	0.022863	-0.013116	0.048356	0.013144	0.033807	-
0.036386	0.015334	0.012154	0.053549	-0.011115	0.014766	
0.004653	0.014647	0.023789	-0.041911	0.003723	0.027061	-
0.008540	-0.003002	0.014466	0.049715	0.014078	-0.013013	
0.003712	0.003794	-0.028613	-0.058137	-0.013377	-0.000777	-
0.007123	-0.018947					
x683	0.013843	0.004737	0.021308	0.024523	0.023642	-
0.003635	0.012832	-0.007323	-0.047863	0.022391	0.004946	-
0.023594	0.053345	0.049512	0.114187	0.040809	0.001268	
0.022804	0.071600	-0.020024	-0.021521	-0.037583	0.033943	-
0.038542	-0.051212	-0.043117	0.027268	0.046336	-0.000950	-
0.040336	0.031646	-0.005710	0.034885	-0.030684	-0.007665	
0.027940	0.046706					
y683	-0.031678	-0.004127	-0.003178	0.005790	-0.010531	
0.001363	0.016169	0.001374	0.011352	-0.007770	-0.010650	
0.003742	-0.027061	-0.010696	-0.041707	-0.035940	0.023866	-
0.033864	-0.013766	-0.001087	-0.020437	-0.029807	-0.064445	
0.030290	-0.006874	-0.041243	-0.058431	0.012882	-0.032743	-

0.005653 0.021091 0.008083 -0.046340 -0.010122 0.035143
0.012891 0.056747
z683 0.010134 -0.003783 0.024216 0.020088 0.015437 -
0.060733 0.028747 -0.024946 0.045536 0.015222 0.039741 -
0.036328 0.021684 0.012321 0.053611 -0.015445 0.017228
0.013222 0.005116 0.025586 -0.038955 -0.002078 0.008480
0.000474 0.012873 -0.000277 0.057017 0.029782 -0.001284 -
0.016928 0.015475 -0.029833 -0.041400 -0.015435 0.012723 -
0.018130 0.003977
x684 0.034765 0.011700 0.020833 0.021822 0.022153
0.006364 0.011918 0.006177 -0.044241 0.029061 0.000411 -
0.026108 0.046632 0.053027 0.143348 0.048019 -0.029661
0.023373 0.064803 -0.003812 -0.028545 -0.030809 0.057701 -
0.035896 -0.101090 0.002815 -0.010293 0.026400 0.008825 -
0.024764 0.012884 0.002842 0.023605 0.015362 -0.027122
0.039172 -0.009505
y684 -0.018880 -0.006275 -0.008363 0.007873 -0.011167
0.010543 0.012718 0.008922 0.005918 0.002421 -0.019613
0.004667 -0.025242 -0.011273 -0.036313 -0.043295 0.018180 -
0.026599 -0.004035 0.007344 -0.027991 -0.026769 -0.049267
0.026344 -0.032260 -0.014717 -0.091690 -0.009007 -0.059247
0.010303 0.008830 -0.003947 -0.046760 0.013359 0.075924
0.041899 0.025069
z684 0.017932 -0.002949 0.026634 0.019945 0.014872 -
0.050975 0.023022 -0.027101 0.046918 0.020718 0.035215 -
0.038805 0.021190 0.011928 0.054578 -0.018042 -0.003984
0.014628 -0.007666 0.036110 -0.043688 -0.007216 -0.002719
0.002266 -0.006625 0.027634 0.039204 0.029529 0.008746 -
0.014319 -0.002239 -0.036014 -0.043216 0.001557 0.011060 -
0.007101 -0.005120
x685 0.042697 0.015925 0.021026 0.019687 0.023740
0.007557 0.012686 0.005231 -0.053097 0.031227 0.010992 -
0.029091 0.046051 0.056591 0.160707 0.052032 -0.049120
0.029562 0.056942 0.002585 -0.020185 -0.025045 0.066067 -
0.031679 -0.111406 0.012828 -0.022479 0.037651 0.045730 -
0.041577 0.025319 0.022112 0.015444 0.030764 -0.091324 -
0.005952 0.002967
y685 -0.012712 -0.016051 -0.001238 0.009755 -0.003050
0.013562 -0.000830 0.005284 -0.005775 0.005032 -0.013731 -
0.000107 -0.013930 -0.008224 -0.048870 -0.042603 0.009720 -
0.014659 0.013566 -0.003500 -0.027563 -0.029978 -0.049679
0.029031 -0.012367 0.025023 -0.100582 -0.007182 -0.052121
0.001568 0.009480 -0.014901 -0.059505 -0.020763 0.042879 -
0.015071 0.064455
z685 0.020371 -0.002435 0.026498 0.020433 0.010693 -
0.040941 0.017315 -0.034688 0.042539 0.024321 0.032637 -
0.036427 0.030091 0.006063 0.058734 -0.021886 -0.008549
0.022429 -0.025024 0.040423 -0.045571 -0.012346 -0.024620
0.005165 -0.011397 0.030426 0.034382 0.041868 0.020643 -
0.027901 -0.014061 -0.045929 -0.026168 0.021857 0.029065
0.010389 -0.004263
x686 0.047790 0.023452 0.018679 0.016284 0.021074
0.009052 0.012958 0.004112 -0.064214 0.033130 0.019642 -
0.026887 0.047104 0.054579 0.179915 0.052985 -0.058754
0.034766 0.041592 0.007021 -0.009968 -0.015977 0.075122 -
0.028177 -0.130865 0.009461 -0.033634 0.051552 0.080210 -
0.062730 0.032637 0.046556 0.017277 0.069004 -0.122104 -
0.019365 -0.012649
y686 -0.012230 -0.020687 -0.004881 0.010602 -0.007015
0.020200 -0.011467 0.009470 -0.019741 0.019863 -0.017630

0.012506   -0.002764 -0.016393 -0.055417 -0.040249   0.024611 -
0.015215   0.017463 -0.009519 -0.026341 -0.027390 -0.039200
0.013453   -0.025412   0.017906 -0.101023   0.006100 -0.065122 -
0.012268   -0.007232 -0.011590 -0.064090 -0.000116   0.085762
0.015912   0.064803
z686   0.025961 -0.001857   0.027937   0.020019   0.011579 -
0.028601   0.016731 -0.047146   0.039891   0.025697   0.032174 -
0.040132   0.037168   0.006287   0.060512 -0.028796 -0.028100
0.034253   -0.041042   0.047025 -0.046001 -0.016359 -0.049057
0.011067   -0.010063   0.039565   0.025322   0.047030   0.036898 -
0.037291   -0.011677 -0.053969 -0.003845   0.031134   0.025651 -
0.007011   0.013276

New Procrustes fit:
Dataset 'stapedial fenestra'

36 landmarks in 3 dimensions.
The dataset contains 40 observations, of which 36 are included for analyses.

Average shape:

Lmk.	Axis 1 (x)	Axis 2 (y)	Axis 3 (z)
1	-0.20121508	0.02134770	-0.00514863
2	-0.19474438	0.01262524	0.01919547
3	-0.18376063	0.00558055	0.04180220
4	-0.16797005	0.00224095	0.06106441
5	-0.15061371	0.00038378	0.07872723
6	-0.13093829	-0.00305589	0.09249915
7	-0.10630638	-0.00640517	0.10353949
8	-0.08017015	-0.01022061	0.11350935
9	-0.05124226	-0.01160738	0.12845383
10	-0.01749876	-0.01675349	0.12228231
11	0.01847174	-0.01162679	0.12616656
12	0.05179732	-0.00786087	0.12746823
13	0.08139226	-0.00392742	0.12410011
14	0.10792430	-0.00015844	0.11636267
15	0.13180393	0.00124157	0.10514996
16	0.15435116	0.00334859	0.08973629
17	0.17428469	0.00781829	0.06975485
18	0.18998523	0.01255868	0.04468609
19	0.20134220	0.01742689	0.01537468
20	0.19664690	0.01113605	-0.01643391
21	0.18596712	0.00779482	-0.04453743
22	0.17197504	0.00550107	-0.06914757
23	0.15445038	0.00167379	-0.08897246
24	0.13352105	-0.00176939	-0.10488309
25	0.10882563	-0.00519757	-0.11716732
26	0.08052596	-0.00723890	-0.12464580
27	0.04777581	-0.01221515	-0.13071848
28	0.01288354	-0.01520985	-0.13180231
29	-0.02102760	-0.01249875	-0.13047191
30	-0.05356523	-0.00989333	-0.12583203
31	-0.08294675	-0.00574331	-0.11785592
32	-0.10958291	-0.00183399	-0.10793296
33	-0.13353024	0.00199159	-0.09500990
34	-0.15516899	0.00587437	-0.07838758
35	-0.17408318	0.00975050	-0.05787500
36	-0.18955969	0.01492189	-0.03305060

Procrustes sums of squares: 0.5467144236321867
Tangent sums of squares: 0.506872782648445

Data matrices in this dataset:
- stapedial fenestra, raw data
- stapedial fenestra, centroid size
- stapedial fenestra, Procrustes coordinates

New Procrustes fit:
Dataset 'stapedial fenestra'

36 landmarks in 3 dimensions.
The dataset contains 40 observations, of which 36 are included for analyses.

Average shape:

Lmk.	Axis 1 (x)	Axis 2 (y)	Axis 3 (z)
1	-0.20121508	0.02134770	-0.00514863
2	-0.19474438	0.01262524	0.01919547
3	-0.18376063	0.00558055	0.04180220
4	-0.16797005	0.00224095	0.06106441
5	-0.15061371	0.00038378	0.07872723
6	-0.13093829	-0.00305589	0.09249915
7	-0.10630638	-0.00640517	0.10353949
8	-0.08017015	-0.01022061	0.11350935
9	-0.05124226	-0.01160738	0.12845383
10	-0.01749876	-0.01675349	0.12228231
11	0.01847174	-0.01162679	0.12616656
12	0.05179732	-0.00786087	0.12746823
13	0.08139226	-0.00392742	0.12410011
14	0.10792430	-0.00015844	0.11636267
15	0.13180393	0.00124157	0.10514996
16	0.15435116	0.00334859	0.08973629
17	0.17428469	0.00781829	0.06975485
18	0.18998523	0.01255868	0.04468609
19	0.20134220	0.01742689	0.01537468
20	0.19664690	0.01113605	-0.01643391
21	0.18596712	0.00779482	-0.04453743
22	0.17197504	0.00550107	-0.06914757
23	0.15445038	0.00167379	-0.08897246
24	0.13352105	-0.00176939	-0.10488309
25	0.10882563	-0.00519757	-0.11716732
26	0.08052596	-0.00723890	-0.12464580
27	0.04777581	-0.01221515	-0.13071848
28	0.01288354	-0.01520985	-0.13180231
29	-0.02102760	-0.01249875	-0.13047191
30	-0.05356523	-0.00989333	-0.12583203
31	-0.08294675	-0.00574331	-0.11785592
32	-0.10958291	-0.00183399	-0.10793296
33	-0.13353024	0.00199159	-0.09500990
34	-0.15516899	0.00587437	-0.07838758
35	-0.17408318	0.00975050	-0.05787500
36	-0.18955969	0.01492189	-0.03305060

Procrustes sums of squares: 0.5467144236321867
Tangent sums of squares: 0.506872782648445

Data matrices in this dataset:
- stapedial fenestra, raw data
- stapedial fenestra, centroid size
- stapedial fenestra, Procrustes coordinates

Canonical Variate Analysis: CVA ...stapedial fenestra

Dataset: stapedial fenestra
Classification criterion: subfam

Groups		Observations
1.	Ca	9
2.	Ce	13
3.	Di	3
4.	Pr	11

Variation among groups, scaled by the inverse of the within-group variation

	Eigenvalues	% Variance	Cumulative %
1.	5.33380507	51.823	51.823
2.	3.65155692	35.478	87.301
3.	1.30704353	12.699	100.000

Mahalanobis distances among groups:

	Ca	Ce	Di
Ce	4.2294		
Di	6.2816	7.5863	
Pr	3.8828	4.3396	8.2913

P-values from permutation tests (10000 permutation rounds) for Mahalanobis distances among groups:

	Ca	Ce	Di
Ce	<.0001		
Di	0.0029	<.0001	
Pr	0.0004	0.0001	0.0015

Procrustes distances among groups:

	Ca	Ce	Di
Ce	0.0524		
Di	0.1388	0.1508	
Pr	0.0460	0.0558	0.1404

P-values from permutation tests (10000 permutation rounds) for Procrustes distances among groups:

	Ca	Ce	Di
Ce	0.1663		
Di	0.1526	0.0493	
Pr	0.2868	0.0443	0.1286

Canonical coefficients:

CV1 CV2 CV3

x1	18.3100	-2.1943	-67.1253
y1	-59.2289	28.9058	-42.1707
z1	75.0736	54.1524	44.6455
x2	16.4226	43.8472	-11.3195
y2	-62.5298	40.4364	-11.2959
z2	62.4474	20.4111	-0.6061
x3	-5.9174	29.3378	49.9117
y3	106.4493	43.4155	-11.1801
z3	63.4477	-4.0838	-67.3415
x4	12.4528	55.5382	38.5459
y4	-13.4939	-21.5345	5.9183
z4	-170.9498	-10.0575	-29.0336
x5	60.3643	12.1419	52.3384
y5	38.1085	51.3273	75.0975
z5	-14.5185	4.7477	-29.2000
x6	8.8112	-24.6362	-69.9528
y6	22.5887	-28.1589	-28.3337
z6	-94.4528	34.3171	-8.9138
x7	11.1438	-10.4014	13.7312
y7	-55.0749	-34.7451	26.5009
z7	-19.0147	21.2094	39.0802
x8	-16.9914	1.9571	23.3085
y8	-22.6916	34.0397	30.1787
z8	11.5829	18.6637	55.6784
x9	-47.9171	22.7262	40.4477
y9	21.5251	-47.1225	-2.2958
z9	12.4505	6.0873	-14.5835
x10	31.6359	-8.2029	-0.6272
y10	52.4894	20.0672	-34.7922
z10	-12.2575	-50.8834	10.0730
x11	-28.4087	-5.6224	3.1835
y11	32.1787	-59.4305	12.4170
z11	21.8168	8.9077	0.9327
x12	27.2735	-19.8355	-32.1782
y12	-51.3776	14.4421	3.4241
z12	83.7689	-17.0228	-16.0237
x13	36.1319	-34.8963	-46.1385
y13	-65.6412	26.3202	-21.2589
z13	20.0234	-30.0479	-31.9602
x14	-14.4394	-23.2071	-25.7068
y14	43.2271	-4.5786	9.3452
z14	-43.4139	-1.4133	1.4459
x15	-39.3352	-17.4315	12.0699
y15	34.5984	-59.7906	19.9916
z15	2.9096	29.4734	28.1411
x16	-25.1530	-5.6021	32.2679
y16	-28.4779	4.4437	2.5382
z16	-10.4810	32.1721	43.2809
x17	9.1284	-16.4434	63.4581
y17	8.7719	20.4740	-86.3897
z17	-34.6201	33.0827	-0.2868
x18	22.0701	-41.7056	-28.3103
y18	42.7010	16.1817	8.2129
z18	21.6281	-3.1414	-33.5856
x19	46.7826	12.8855	-31.7239
y19	-15.7368	-9.4689	-0.8934
z19	36.9344	13.5084	0.6756
x20	19.8137	60.9500	12.9747
y20	-39.4129	54.2567	2.1859
z20	8.9556	-18.4501	-8.5406

x21	2.7139	65.2546	24.6153
y21	-39.6025	33.1804	7.2849
z21	0.2887	-45.9758	-23.0465
x22	-53.9961	14.1754	-7.3460
y22	66.2137	21.3668	48.4804
z22	32.9833	-8.2847	-2.8875
x23	47.4899	-9.8884	14.1420
y23	-76.3902	3.6688	0.2832
z23	-23.1028	1.2734	-9.2961
x24	42.7216	-19.3375	-10.5993
y24	-33.6035	3.8258	28.9280
z24	-27.7309	13.8753	14.9790
x25	17.7906	-3.2906	14.9591
y25	17.5194	-61.6666	-35.4236
z25	2.0335	-23.9398	-30.6465
x26	-1.8215	-38.1128	-33.8376
y26	75.4002	-41.8076	-1.1967
z26	22.4007	41.6808	102.6894
x27	-108.3431	0.2342	9.7831
y27	71.5086	-28.1387	-10.3933
z27	35.8102	-9.1725	39.0471
x28	-85.0021	-21.4557	-11.5491
y28	67.5852	47.1995	3.1078
z28	-47.2907	-11.7606	5.7818
x29	9.0315	-9.4094	-6.5236
y29	-33.3474	-17.2709	26.4295
z29	11.5822	6.4110	23.9270
x30	5.9951	-4.1746	2.0441
y30	-50.4862	-1.7010	-23.9138
z30	-6.2694	-9.5173	-22.3430
x31	20.0722	11.6769	-1.1900
y31	-137.1727	41.9695	-32.1208
z31	-70.4936	23.9796	-132.4927
x32	31.9572	10.2757	-12.7860
y32	-42.9239	19.5065	18.5119
z32	10.0523	-13.4358	8.7563
x33	54.0069	3.3944	14.9271
y33	56.4136	-31.6272	47.2332
z33	38.3134	-41.3706	30.7369
x34	6.2615	-29.1544	-59.3615
y34	95.3338	-48.5001	-29.2724
z34	3.6788	-75.4526	-28.3963
x35	-93.5689	20.9142	13.5277
y35	42.3823	-21.0929	16.4215
z35	-57.5000	2.1619	12.8790
x36	-37.4875	-20.3072	20.0398
y36	-67.8033	-8.3930	-21.5598
z36	53.9136	7.8950	26.4339

New Procrustes fit:
Dataset 'cochlea thickness'

40 landmarks in 3 dimensions.
The dataset contains 40 observations, of which 36 are included for analyses.

Average shape:

Lmk. Axis 1 (x) Axis 2 (y) Axis 3 (z)

1	0.17424318	-0.10516996	-0.00557481
2	0.19182873	-0.08211598	-0.00170438
3	0.20441025	-0.06343124	0.00151336
4	0.21282231	-0.03178483	0.00508676
5	0.20922943	-0.01710533	0.00546793
6	0.20093338	-0.00183379	0.00542765
7	0.18456836	0.01344144	0.00384300
8	-0.14695906	-0.08929841	-0.00012965
9	-0.16094879	-0.07796146	0.00064828
10	-0.17000796	-0.06647291	0.00131274
11	-0.17503356	-0.05554470	0.00189223
12	-0.17477974	-0.03401492	0.00282057
13	-0.17036731	-0.02250650	0.00320802
14	-0.16241262	-0.01017867	0.00356104
15	0.09534730	0.01221898	-0.00886344
16	0.10342647	0.02968847	-0.00636775
17	0.10781824	0.04353162	-0.00478273
18	0.10874503	0.05496920	-0.00369788
19	0.10100360	0.07300374	-0.00315549
20	0.09246577	0.08062849	-0.00361984
21	0.08029750	0.08772294	-0.00472316
22	-0.10362939	0.00595952	0.00000681
23	-0.12195777	0.01522891	0.00053167
24	-0.13227081	0.02556834	0.00065699
25	-0.13894194	0.04316628	0.00148323
26	-0.13696240	0.05137789	0.00192858
27	-0.13213066	0.06016269	0.00253722
28	-0.12340585	0.06991068	0.00320146
29	0.14664102	-0.11976082	-0.01243244
30	-0.12876608	-0.09604644	-0.00092153
31	0.07607912	0.00169738	-0.00617607
32	-0.08059182	0.00241009	0.00608116
33	0.20894642	-0.04048387	0.02246372
34	-0.17266417	-0.04948984	-0.01229940
35	0.10582972	0.05891455	0.00114060
36	-0.13619544	0.03991046	-0.00165564
37	0.15884102	0.02087341	0.00202751
38	-0.14879368	0.00126839	0.00135636
39	0.06295013	0.09206122	-0.00476130
40	-0.10960795	0.07948500	0.00266866

Procrustes sums of squares: 0.40502941752868593
Tangent sums of squares: 0.3829459052070069

Data matrices in this dataset:
- cochlea thickness, raw data
- cochlea thickness, centroid size
- cochlea thickness, Procrustes coordinates

New Procrustes fit:
Dataset 'cochlea thickness'

40 landmarks in 3 dimensions.
The dataset contains 40 observations, of which 36 are included for analyses.

Average shape:

Lmk.	Axis 1 (x)	Axis 2 (y)	Axis 3 (z)
1	0.17424318	-0.10516996	-0.00557481
2	0.19182873	-0.08211598	-0.00170438
3	0.20441025	-0.06343124	0.00151336
4	0.21282231	-0.03178483	0.00508676
5	0.20922943	-0.01710533	0.00546793
6	0.20093338	-0.00183379	0.00542765
7	0.18456836	0.01344144	0.00384300
8	-0.14695906	-0.08929841	-0.00012965
9	-0.16094879	-0.07796146	0.00064828
10	-0.17000796	-0.06647291	0.00131274
11	-0.17503356	-0.05554470	0.00189223
12	-0.17477974	-0.03401492	0.00282057
13	-0.17036731	-0.02250650	0.00320802
14	-0.16241262	-0.01017867	0.00356104
15	0.09534730	0.01221898	-0.00886344
16	0.10342647	0.02968847	-0.00636775
17	0.10781824	0.04353162	-0.00478273
18	0.10874503	0.05496920	-0.00369788
19	0.10100360	0.07300374	-0.00315549
20	0.09246577	0.08062849	-0.00361984
21	0.08029750	0.08772294	-0.00472316
22	-0.10362939	0.00595952	0.00000681
23	-0.12195777	0.01522891	0.00053167
24	-0.13227081	0.02556834	0.00065699
25	-0.13894194	0.04316628	0.00148323
26	-0.13696240	0.05137789	0.00192858
27	-0.13213066	0.06016269	0.00253722
28	-0.12340585	0.06991068	0.00320146
29	0.14664102	-0.11976082	-0.01243244
30	-0.12876608	-0.09604644	-0.00092153
31	0.07607912	0.00169738	-0.00617607
32	-0.08059182	0.00241009	0.00608116
33	0.20894642	-0.04048387	0.02246372
34	-0.17266417	-0.04948984	-0.01229940
35	0.10582972	0.05891455	0.00114060
36	-0.13619544	0.03991046	-0.00165564
37	0.15884102	0.02087341	0.00202751
38	-0.14879368	0.00126839	0.00135636
39	0.06295013	0.09206122	-0.00476130
40	-0.10960795	0.07948500	0.00266866

Procrustes sums of squares: 0.40502941752868593
Tangent sums of squares: 0.3829459052070069

Data matrices in this dataset:
- cochlea thickness, raw data
- cochlea thickness, centroid size
- cochlea thickness, Procrustes coordinates

New Procrustes fit:
Dataset 'cochlea thickness'

37 landmarks in 3 dimensions.
The dataset contains 40 observations, of which 36 are included for analyses.

Average shape:

Lmk.	Axis 1 (x)	Axis 2 (y)	Axis 3 (z)
1	0.18501104	-0.11186764	0.00251102
2	0.20335087	-0.08725447	0.00503036
3	0.21650190	-0.06728461	0.00756247
4	0.22511486	-0.03374877	0.00879391
5	0.22124143	-0.01834213	0.00790994
6	0.21242034	-0.00238314	0.00640333
7	0.19512409	0.01339943	0.00302332
8	-0.15323016	-0.09691690	0.00133884
9	-0.16801047	-0.08503287	0.00092439
10	-0.17760855	-0.07297203	0.00053985
11	-0.18296248	-0.06148085	0.00022446
12	-0.18281209	-0.03878037	-0.00031825
13	-0.17823103	-0.02662860	-0.00061274
14	-0.16992580	-0.01359104	-0.00090973
15	0.10137949	0.01056250	-0.01104476
16	0.10980464	0.02915194	-0.00958734
17	0.11434565	0.04383283	-0.00918429
18	0.11524986	0.05592675	-0.00912413
19	0.10699077	0.07486024	-0.01018032
20	0.09796522	0.08277429	-0.01148922
21	0.08513114	0.09006417	-0.01348452
22	-0.03290924	0.08725304	0.09171014
23	-0.10806170	0.00350730	-0.00415721
24	-0.12739466	0.01309421	-0.00512767
25	-0.13830393	0.02389412	-0.00583226
26	-0.14542524	0.04240728	-0.00632335
27	-0.14338945	0.05107835	-0.00646910
28	-0.13836149	0.06037849	-0.00645398
29	-0.12924812	0.07072320	-0.00639069
30	0.15611352	-0.12793450	-0.00433463
31	-0.13404135	-0.10394567	0.00149926
32	0.08092586	-0.00068598	-0.01077946
33	-0.08374979	0.00047329	0.00428844
34	0.16802958	0.02082930	-0.00026721
35	-0.15564463	-0.00161801	-0.00399466
36	0.06685445	0.09449463	-0.01427257
37	-0.11224457	0.08176223	0.00857837

Procrustes sums of squares: 0.3025260832657731
Tangent sums of squares: 0.29768764307091977

Data matrices in this dataset:
- cochlea thickness, raw data
- cochlea thickness, centroid size
- cochlea thickness, Procrustes coordinates

New Procrustes fit:
Dataset 'cochlea thickness'

37 landmarks in 3 dimensions.
The dataset contains 40 observations, of which 36 are included for analyses.

Average shape:

Lmk. Axis 1 (x) Axis 2 (y) Axis 3 (z)

1	0.18501104	-0.11186764	0.00251102
2	0.20335087	-0.08725447	0.00503036
3	0.21650190	-0.06728461	0.00756247
4	0.22511486	-0.03374877	0.00879391
5	0.22124143	-0.01834213	0.00790994
6	0.21242034	-0.00238314	0.00640333
7	0.19512409	0.01339943	0.00302332
8	-0.15323016	-0.09691690	0.00133884
9	-0.16801047	-0.08503287	0.00092439
10	-0.17760855	-0.07297203	0.00053985
11	-0.18296248	-0.06148085	0.00022446
12	-0.18281209	-0.03878037	-0.00031825
13	-0.17823103	-0.02662860	-0.00061274
14	-0.16992580	-0.01359104	-0.00090973
15	0.10137949	0.01056250	-0.01104476
16	0.10980464	0.02915194	-0.00958734
17	0.11434565	0.04383283	-0.00918429
18	0.11524986	0.05592675	-0.00912413
19	0.10699077	0.07486024	-0.01018032
20	0.09796522	0.08277429	-0.01148922
21	0.08513114	0.09006417	-0.01348452
22	-0.03290924	0.08725304	0.09171014
23	-0.10806170	0.00350730	-0.00415721
24	-0.12739466	0.01309421	-0.00512767
25	-0.13830393	0.02389412	-0.00583226
26	-0.14542524	0.04240728	-0.00632335
27	-0.14338945	0.05107835	-0.00646910
28	-0.13836149	0.06037849	-0.00645398
29	-0.12924812	0.07072320	-0.00639069
30	0.15611352	-0.12793450	-0.00433463
31	-0.13404135	-0.10394567	0.00149926
32	0.08092586	-0.00068598	-0.01077946
33	-0.08374979	0.00047329	0.00428844
34	0.16802958	0.02082930	-0.00026721
35	-0.15564463	-0.00161801	-0.00399466
36	0.06685445	0.09449463	-0.01427257
37	-0.11224457	0.08176223	0.00857837

Procrustes sums of squares: 0.3025260832657731
Tangent sums of squares: 0.29768764307091977

Data matrices in this dataset:
- cochlea thickness, raw data
- cochlea thickness, centroid size
- cochlea thickness, Procrustes coordinates

New Procrustes fit:
Dataset 'coclhea thickness'

37 landmarks in 3 dimensions.
The dataset contains 40 observations, of which 36 are included for analyses.

Average shape:

Lmk.	Axis 1 (x)	Axis 2 (y)	Axis 3 (z)
1	0.18498098	-0.11188103	0.00043740
2	0.20332532	-0.08732060	0.00335289

3	0.21648110	-0.06740248	0.00621585
4	0.22509419	-0.03389846	0.00805581
5	0.22121724	-0.01848100	0.00748177
6	0.21239073	-0.00250072	0.00631576
7	0.19508338	0.01333554	0.00330712
8	-0.15319564	-0.09696105	0.00099181
9	-0.16797452	-0.08507463	0.00086313
10	-0.17757260	-0.07301183	0.00074811
11	-0.18292725	-0.06151887	0.00067169
12	-0.18278011	-0.03881575	0.00055295
13	-0.17820185	-0.02666182	0.00046665
14	-0.16990017	-0.01362212	0.00037791
15	0.10130167	0.01074866	-0.01040626
16	0.10973077	0.02930528	-0.00864205
17	0.11427166	0.04397367	-0.00798004
18	0.11517549	0.05606101	-0.00769524
19	0.10691409	0.07500261	-0.00835887
20	0.09788550	0.08293499	-0.00948066
21	0.08504696	0.09025520	-0.01128551
22	-0.03252570	0.08540873	0.09431050
23	-0.10806659	0.00353977	-0.00275002
24	-0.12739905	0.01314109	-0.00350178
25	-0.13830895	0.02394847	-0.00396077
26	-0.14543102	0.04246392	-0.00407100
27	-0.14339655	0.05113451	-0.00406033
28	-0.13837005	0.06043116	-0.00388853
29	-0.12925874	0.07077121	-0.00366489
30	0.15606480	-0.12781972	-0.00659614
31	-0.13401010	-0.10398879	0.00093999
32	0.08085879	-0.00052383	-0.01027904
33	-0.08372638	0.00035323	0.00549046
34	0.16797603	0.02083938	0.00026703
35	-0.15563044	-0.00161353	-0.00253988
36	0.06676865	0.09470577	-0.01191616
37	-0.11189162	0.08274203	-0.01976967

Procrustes sums of squares: 0.3022140321133854
Tangent sums of squares: 0.2974564543391678

Data matrices in this dataset:
- coclhea thickness, raw data
- coclhea thickness, centroid size
- coclhea thickness, Procrustes coordinates

New Procrustes fit:
Dataset 'coclhea thickness'

37 landmarks in 3 dimensions.
The dataset contains 40 observations, of which 36 are included for analyses.

Average shape:

Lmk.	Axis 1 (x)	Axis 2 (y)	Axis 3 (z)
1	0.18498098	-0.11188103	0.00043740
2	0.20332532	-0.08732060	0.00335289
3	0.21648110	-0.06740248	0.00621585
4	0.22509419	-0.03389846	0.00805581

5	0.22121724	-0.01848100	0.00748177
6	0.21239073	-0.00250072	0.00631576
7	0.19508338	0.01333554	0.00330712
8	-0.15319564	-0.09696105	0.00099181
9	-0.16797452	-0.08507463	0.00086313
10	-0.17757260	-0.07301183	0.00074811
11	-0.18292725	-0.06151887	0.00067169
12	-0.18278011	-0.03881575	0.00055295
13	-0.17820185	-0.02666182	0.00046665
14	-0.16990017	-0.01362212	0.00037791
15	0.10130167	0.01074866	-0.01040626
16	0.10973077	0.02930528	-0.00864205
17	0.11427166	0.04397367	-0.00798004
18	0.11517549	0.05606101	-0.00769524
19	0.10691409	0.07500261	-0.00835887
20	0.09788550	0.08293499	-0.00948066
21	0.08504696	0.09025520	-0.01128551
22	-0.03252570	0.08540873	0.09431050
23	-0.10806659	0.00353977	-0.00275002
24	-0.12739905	0.01314109	-0.00350178
25	-0.13830895	0.02394847	-0.00396077
26	-0.14543102	0.04246392	-0.00407100
27	-0.14339655	0.05113451	-0.00406033
28	-0.13837005	0.06043116	-0.00388853
29	-0.12925874	0.07077121	-0.00366489
30	0.15606480	-0.12781972	-0.00659614
31	-0.13401010	-0.10398879	0.00093999
32	0.08085879	-0.00052383	-0.01027904
33	-0.08372638	0.00035323	0.00549046
34	0.16797603	0.02083938	0.00026703
35	-0.15563044	-0.00161353	-0.00253988
36	0.06676865	0.09470577	-0.01191616
37	-0.11189162	0.08274203	-0.01976967

Procrustes sums of squares: 0.3022140321133854
Tangent sums of squares: 0.2974564543391678

Data matrices in this dataset:
- coclhea thickness, raw data
- coclhea thickness, centroid size
- coclhea thickness, Procrustes coordinates

New Procrustes fit:
Dataset 'cochlea thickness'

36 landmarks in 3 dimensions.
The dataset contains 40 observations, of which 36 are included for analyses.

Average shape:

Lmk.	Axis 1 (x)	Axis 2 (y)	Axis 3 (z)
1	0.18529530	-0.11118768	-0.00416073
2	0.20391004	-0.08669776	0.00037006
3	0.21727279	-0.06685790	0.00451012
4	0.22610274	-0.03325406	0.00838463
5	0.22225153	-0.01767620	0.00869686
6	0.21340667	-0.00147609	0.00844817

7	0.19599743	0.01472258	0.00636285
8	-0.15584483	-0.09489493	-0.00183817
9	-0.17070879	-0.08286031	-0.00089078
10	-0.18034456	-0.07067071	-0.00004078
11	-0.18570082	-0.05907163	0.00072665
12	-0.18546073	-0.03620004	0.00205215
13	-0.18079250	-0.02397126	0.00264352
14	-0.17236421	-0.01086471	0.00320690
15	0.10132228	0.01331252	-0.00808430
16	0.10990732	0.03186488	-0.00512532
17	0.11454977	0.04658063	-0.00328765
18	0.11551120	0.05873116	-0.00207583
19	0.10725129	0.07786994	-0.00160358
20	0.09817042	0.08595488	-0.00223823
21	0.08524070	0.09347782	-0.00360610
22	-0.10991804	0.00639345	0.00001342
23	-0.12938865	0.01618117	0.00062124
24	-0.14035907	0.02713363	0.00091558
25	-0.14747187	0.04581382	0.00200938
26	-0.14538407	0.05453520	0.00253135
27	-0.14027334	0.06386455	0.00322204
28	-0.13103650	0.07422367	0.00397965
29	0.15603344	-0.12670693	-0.01216975
30	-0.13651497	-0.10204184	-0.00276915
31	0.08065441	0.00199922	-0.00909365
32	-0.08534404	0.00257665	0.00809501
33	0.16866725	0.02260607	0.00376099
34	-0.15793062	0.00131838	0.00076433
35	0.06681698	0.09807971	-0.00394854
36	-0.11352394	0.08719213	-0.01038234

Procrustes sums of squares: 0.29625981136587026
Tangent sums of squares: 0.2915561189425964

Data matrices in this dataset:
- cochlea thickness, raw data
- cochlea thickness, centroid size
- cochlea thickness, Procrustes coordinates

New Procrustes fit:
Dataset 'cochlea thickness'

36 landmarks in 3 dimensions.
The dataset contains 40 observations, of which 36 are included for analyses.

Average shape:

Lmk.	Axis 1 (x)	Axis 2 (y)	Axis 3 (z)
1	0.18529530	-0.11118768	-0.00416073
2	0.20391004	-0.08669776	0.00037006
3	0.21727279	-0.06685790	0.00451012
4	0.22610274	-0.03325406	0.00838463
5	0.22225153	-0.01767620	0.00869686
6	0.21340667	-0.00147609	0.00844817
7	0.19599743	0.01472258	0.00636285
8	-0.15584483	-0.09489493	-0.00183817
9	-0.17070879	-0.08286031	-0.00089078

10	-0.18034456	-0.07067071	-0.00004078
11	-0.18570082	-0.05907163	0.00072665
12	-0.18546073	-0.03620004	0.00205215
13	-0.18079250	-0.02397126	0.00264352
14	-0.17236421	-0.01086471	0.00320690
15	0.10132228	0.01331252	-0.00808430
16	0.10990732	0.03186488	-0.00512532
17	0.11454977	0.04658063	-0.00328765
18	0.11551120	0.05873116	-0.00207583
19	0.10725129	0.07786994	-0.00160358
20	0.09817042	0.08595488	-0.00223823
21	0.08524070	0.09347782	-0.00360610
22	-0.10991804	0.00639345	0.00001342
23	-0.12938865	0.01618117	0.00062124
24	-0.14035907	0.02713363	0.00091558
25	-0.14747187	0.04581382	0.00200938
26	-0.14538407	0.05453520	0.00253135
27	-0.14027334	0.06386455	0.00322204
28	-0.13103650	0.07422367	0.00397965
29	0.15603344	-0.12670693	-0.01216975
30	-0.13651497	-0.10204184	-0.00276915
31	0.08065441	0.00199922	-0.00909365
32	-0.08534404	0.00257665	0.00809501
33	0.16866725	0.02260607	0.00376099
34	-0.15793062	0.00131838	0.00076433
35	0.06681698	0.09807971	-0.00394854
36	-0.11352394	0.08719213	-0.01038234

Procrustes sums of squares: 0.29625981136587026
Tangent sums of squares: 0.2915561189425964

Data matrices in this dataset:
- cochlea thickness, raw data
- cochlea thickness, centroid size
- cochlea thickness, Procrustes coordinates

New Procrustes fit:
Dataset 'cochlea thickness'

36 landmarks in 3 dimensions.
The dataset contains 40 observations, of which 36 are included for analyses.

Average shape:

Lmk.	Axis 1 (x)	Axis 2 (y)	Axis 3 (z)
1	0.18529530	-0.11118768	-0.00416073
2	0.20391004	-0.08669776	0.00037006
3	0.21727279	-0.06685790	0.00451012
4	0.22610274	-0.03325406	0.00838463
5	0.22225153	-0.01767620	0.00869686
6	0.21340667	-0.00147609	0.00844817
7	0.19599743	0.01472258	0.00636285
8	-0.15584483	-0.09489493	-0.00183817
9	-0.17070879	-0.08286031	-0.00089078
10	-0.18034456	-0.07067071	-0.00004078
11	-0.18570082	-0.05907163	0.00072665
12	-0.18546073	-0.03620004	0.00205215

13	-0.18079250	-0.02397126	0.00264352
14	-0.17236421	-0.01086471	0.00320690
15	0.10132228	0.01331252	-0.00808430
16	0.10990732	0.03186488	-0.00512532
17	0.11454977	0.04658063	-0.00328765
18	0.11551120	0.05873116	-0.00207583
19	0.10725129	0.07786994	-0.00160358
20	0.09817042	0.08595488	-0.00223823
21	0.08524070	0.09347782	-0.00360610
22	-0.10991804	0.00639345	0.00001342
23	-0.12938865	0.01618117	0.00062124
24	-0.14035907	0.02713363	0.00091558
25	-0.14747187	0.04581382	0.00200938
26	-0.14538407	0.05453520	0.00253135
27	-0.14027334	0.06386455	0.00322204
28	-0.13103650	0.07422367	0.00397965
29	0.15603344	-0.12670693	-0.01216975
30	-0.13651497	-0.10204184	-0.00276915
31	0.08065441	0.00199922	-0.00909365
32	-0.08534404	0.00257665	0.00809501
33	0.16866725	0.02260607	0.00376099
34	-0.15793062	0.00131838	0.00076433
35	0.06681698	0.09807971	-0.00394854
36	-0.11352394	0.08719213	-0.01038234

Procrustes sums of squares: 0.29625981136587026
Tangent sums of squares: 0.2915561189425964

Data matrices in this dataset:
- cochlea thickness, raw data
- cochlea thickness, centroid size
- cochlea thickness, Procrustes coordinates

New Procrustes fit:
Dataset 'cochlea thickness'

36 landmarks in 3 dimensions.
The dataset contains 40 observations, of which 36 are included for analyses.

Average shape:

Lmk.	Axis 1 (x)	Axis 2 (y)	Axis 3 (z)
1	0.18529530	-0.11118768	-0.00416073
2	0.20391004	-0.08669776	0.00037006
3	0.21727279	-0.06685790	0.00451012
4	0.22610274	-0.03325406	0.00838463
5	0.22225153	-0.01767620	0.00869686
6	0.21340667	-0.00147609	0.00844817
7	0.19599743	0.01472258	0.00636285
8	-0.15584483	-0.09489493	-0.00183817
9	-0.17070879	-0.08286031	-0.00089078
10	-0.18034456	-0.07067071	-0.00004078
11	-0.18570082	-0.05907163	0.00072665
12	-0.18546073	-0.03620004	0.00205215
13	-0.18079250	-0.02397126	0.00264352
14	-0.17236421	-0.01086471	0.00320690
15	0.10132228	0.01331252	-0.00808430

16	0.10990732	0.03186488	-0.00512532
17	0.11454977	0.04658063	-0.00328765
18	0.11551120	0.05873116	-0.00207583
19	0.10725129	0.07786994	-0.00160358
20	0.09817042	0.08595488	-0.00223823
21	0.08524070	0.09347782	-0.00360610
22	-0.10991804	0.00639345	0.00001342
23	-0.12938865	0.01618117	0.00062124
24	-0.14035907	0.02713363	0.00091558
25	-0.14747187	0.04581382	0.00200938
26	-0.14538407	0.05453520	0.00253135
27	-0.14027334	0.06386455	0.00322204
28	-0.13103650	0.07422367	0.00397965
29	0.15603344	-0.12670693	-0.01216975
30	-0.13651497	-0.10204184	-0.00276915
31	0.08065441	0.00199922	-0.00909365
32	-0.08534404	0.00257665	0.00809501
33	0.16866725	0.02260607	0.00376099
34	-0.15793062	0.00131838	0.00076433
35	0.06681698	0.09807971	-0.00394854
36	-0.11352394	0.08719213	-0.01038234

Procrustes sums of squares: 0.29625981136587026
Tangent sums of squares: 0.2915561189425964

Data matrices in this dataset:
- cochlea thickness, raw data
- cochlea thickness, centroid size
- cochlea thickness, Procrustes coordinates

Principal Component Analysis: PCA: CovMatrix, cochlea thickness, Procrustes coordinates

	Eigenvalues	% Variance	Cumulative %
1.	0.00201231	24.157	24.157
2.	0.00147914	17.756	41.913
3.	0.00104073	12.493	54.407
4.	0.00090420	10.855	65.261
5.	0.00049232	5.910	71.171
6.	0.00044573	5.351	76.522
7.	0.00036195	4.345	80.867
8.	0.00027900	3.349	84.217
9.	0.00022668	2.721	86.938
10.	0.00016353	1.963	88.901
11.	0.00014667	1.761	90.662
12.	0.00013721	1.647	92.309
13.	0.00008892	1.067	93.376
14.	0.00008387	1.007	94.383
15.	0.00006688	0.803	95.186
16.	0.00006097	0.732	95.918
17.	0.00005758	0.691	96.609
18.	0.00005269	0.633	97.242
19.	0.00003868	0.464	97.706
20.	0.00003264	0.392	98.098
21.	0.00002973	0.357	98.455
22.	0.00002636	0.316	98.771
23.	0.00001907	0.229	99.000

24.	0.00001782	0.214	99.214
25.	0.00001361	0.163	99.377
26.	0.00001198	0.144	99.521
27.	0.00001032	0.124	99.645
28.	0.00000792	0.095	99.740
29.	0.00000586	0.070	99.810
30.	0.00000396	0.048	99.858
31.	0.00000387	0.046	99.904
32.	0.00000307	0.037	99.941
33.	0.00000234	0.028	99.969
34.	0.00000150	0.018	99.987
35.	0.00000105	0.013	100.000

Total variance: 0.00833017

Variance of the eigenvalues: 0.0000000817217
Eigenvalue variance scaled by total variance: 0.00118
Eigenvalue variance scaled by total variance and number of variables: 0.12014
Note: throughout all calculations of eigenvalue variances, the dimensionality used was 101.
Please double-check because this dimensionality may not be appropriate for all situations.
Warning: There appear to be fewer PCs than dimensions in shape space.
This can have the following reasons, among others:
-- the degrees of freedom are less than the shape dimensionality (sample size, etc.).
-- there is complete integration in the data.
Interpret the results cautiously and check for reasons why this is the case.

Principal Component Coefficients

```
       PC1        PC2        PC3        PC4        PC5        PC6
PC7         PC8        PC9        PC10       PC11       PC12
PC13        PC14       PC15       PC16       PC17       PC18
PC19        PC20       PC21       PC22       PC23       PC24
PC25        PC26       PC27       PC28       PC29       PC30
PC31        PC32       PC33       PC34       PC35
   x1 -0.211341   0.048995  -0.029027   0.014099  -0.159889   0.063605
0.061789    0.045905   0.047188   0.152520   0.015049  -0.021286  -
0.145949   -0.038329  -0.013281  -0.172816  -0.057852   0.009233
0.016111   -0.046390   0.036503   0.056513  -0.157079  -0.140874
0.047262    0.089746   0.012020  -0.019041  -0.100023   0.165762  -
0.226303   -0.024229  -0.013849   0.025830  -0.062257
   y1 -0.022875  -0.101943   0.211958  -0.066711  -0.045225   0.080129
0.011798    0.038398  -0.259636   0.015329   0.046231   0.047876  -
0.002185   -0.085631   0.072319  -0.098444   0.004035  -0.140233
0.028750   -0.030726   0.201152   0.102010   0.123342   0.233381
0.077109   -0.008451   0.040760  -0.074038   0.209299  -0.342131
0.238270   -0.139960  -0.036986  -0.124782  -0.270942
   z1  0.010378  -0.048272  -0.000512   0.050458  -0.114802   0.165284
0.130701   -0.003798   0.038899  -0.018382   0.010026   0.042699
0.024760    0.081569   0.030578   0.131168   0.082600   0.079611  -
0.042069    0.108100  -0.033001   0.006801   0.109711  -0.154477
0.021968   -0.047541   0.073063   0.037295   0.087406  -0.037332  -
0.090965    0.081313  -0.075983  -0.007392   0.008366
   x2 -0.218008   0.074213  -0.016036  -0.034001  -0.113278   0.024625
0.057834    0.067588   0.053971   0.111428   0.007951  -0.021746  -
0.067531   -0.018330  -0.023904  -0.078603   0.070583  -0.076934
```

0.117122 0.016659 -0.117125 -0.167724 -0.048894 -0.046750
0.168140 -0.181785 0.018721 -0.081414 -0.020067 0.103238
0.156862 -0.124163 0.141568 0.015965 -0.027979
y2 -0.042634 -0.091476 0.155389 -0.113416 0.010919 0.035010
0.095988 0.045235 -0.096747 0.032875 -0.078212 -0.036883
0.071359 -0.008752 -0.112305 0.057708 -0.076779 -0.089823 -
0.053319 -0.069848 0.047659 0.131867 0.036972 0.121331 -
0.002158 0.034460 -0.012154 0.037379 0.047359 -0.040408
0.006735 0.110706 -0.207182 0.062813 0.153555
z2 0.008406 -0.032022 -0.029385 0.023587 -0.070064 0.123186
0.090640 -0.008189 0.022758 -0.025908 0.040094 -0.011429
0.042190 0.023487 0.074134 0.070190 0.046786 -0.041794
0.028484 0.017173 -0.088722 -0.031616 0.040237 -0.032725
0.045032 -0.020475 0.053476 -0.074395 0.052206 0.015135 -
0.010151 -0.135761 0.159515 0.035683 -0.037129
x3 -0.171327 0.063423 -0.030571 -0.054238 -0.062481 0.037816
0.132068 0.070971 0.082506 0.126811 -0.061860 0.028683
0.093584 -0.072375 -0.069009 -0.043997 0.014586 -0.091412
0.037100 -0.079770 -0.218598 -0.039756 -0.051090 0.116036
0.225316 -0.162487 -0.074439 -0.276135 0.081024 -0.087323
0.128722 0.128265 -0.109929 0.217220 -0.030527
y3 -0.081683 -0.065408 0.126547 -0.153500 0.018184 -0.024744
0.115280 0.049054 -0.011026 0.016784 -0.098610 -0.096921
0.046136 0.064728 -0.155679 0.125729 -0.026681 -0.080095 -
0.003531 0.001840 -0.020679 0.048260 0.059723 0.012128 -
0.013082 -0.014929 0.041849 0.178753 -0.032365 0.092793
0.198166 -0.090467 0.131151 -0.065670 -0.148783
z3 0.024474 -0.027579 -0.066245 -0.000581 -0.016580 0.098260
0.101009 -0.003888 0.025780 -0.017536 0.038248 -0.015742
0.096335 -0.039478 0.075961 -0.026419 -0.017947 -0.112706
0.008554 -0.071599 -0.099602 0.031933 -0.037525 0.023840
0.031859 0.075425 -0.000863 -0.122120 0.078291 -0.038615 -
0.001112 0.017857 0.080098 0.085304 -0.056358
x4 -0.139446 0.121008 -0.088478 -0.034207 0.037008 -0.035312
-0.038384 -0.016647 -0.039780 0.029868 -0.028075 -0.008824
-0.002242 0.012454 -0.071017 0.134192 0.040476 -0.091464
0.015260 -0.032818 -0.073959 0.008573 0.096768 0.155108 -
0.010708 -0.103630 -0.017120 -0.044407 -0.064207 -0.164883 -
0.031339 0.079566 -0.192418 -0.221050 0.006675
y4 -0.080363 -0.045459 0.065919 -0.189220 0.023956 -0.050705
0.177454 0.044512 0.112826 0.039311 -0.126800 -0.086121
0.085097 0.008090 -0.184724 0.137569 -0.036955 0.072361 -
0.014745 -0.013550 -0.142295 0.039523 0.010060 -0.032247 -
0.012595 0.062134 -0.075020 0.050747 0.003267 0.006230 -
0.118245 0.046577 0.016590 -0.024876 0.111397
z4 0.025466 0.013684 -0.106174 -0.031348 0.062247 0.030185
0.005217 -0.012955 -0.027860 -0.052814 0.080925 -0.091007
0.043938 -0.064450 0.016378 -0.087768 -0.056456 -0.157749
0.000374 -0.051923 -0.063204 0.035953 -0.038373 0.134841 -
0.011450 0.153259 -0.023143 0.002808 0.053652 0.014401 -
0.022817 -0.004228 -0.059933 0.043227 0.034491
x5 -0.129355 0.122939 -0.083828 -0.001725 0.053506 -0.026735
-0.103083 -0.032027 -0.081040 0.003023 0.020365 0.017810
0.009878 -0.034375 0.023956 0.163649 0.092060 -0.103232
0.011368 -0.031898 0.007490 0.007249 0.099354 0.022749 -
0.153769 -0.053423 0.041317 -0.057561 -0.085137 -0.142066 -
0.107650 0.127821 0.094051 -0.175066 -0.068930
y5 -0.068257 -0.022610 0.016225 -0.179503 0.030634 -0.074425
0.135587 0.028780 0.129518 0.032045 -0.065406 -0.085097
0.041788 0.015434 -0.109207 0.118565 -0.011320 0.112885 -

0.029704 -0.006485 -0.098838 0.034129 0.000807 -0.045807 -
0.065341 0.108351 -0.083973 0.047851 0.001472 0.003763 -
0.164850 0.005316 0.057064 -0.071429 -0.038203
z5 0.019666 0.023893 -0.108124 -0.042009 0.081702 0.012065
-0.036366 -0.008326 -0.052500 -0.052717 0.108943 -0.116307
0.027168 -0.092694 -0.013939 -0.116112 -0.043701 -0.151167
0.003192 -0.042644 -0.030680 0.042428 -0.026401 0.083133 -
0.052674 0.144658 -0.018227 0.013471 0.023999 0.028266 -
0.055558 0.028351 0.016792 -0.000139 -0.003026
x6 -0.108812 0.123759 -0.062194 0.017747 0.066374 -0.016443
-0.123079 -0.045106 -0.147628 -0.065117 -0.008002 0.012220
0.044900 -0.016763 0.062021 0.177206 0.108565 -0.044734 -
0.000685 0.021491 0.059419 -0.042796 0.035123 -0.015745 -
0.120698 0.074616 0.029300 0.025432 -0.053251 -0.069340 -
0.016009 0.058415 0.028107 -0.052123 0.049135
y6 -0.046172 0.005765 -0.031672 -0.146036 0.041207 -0.086612
0.063315 0.028696 0.103477 0.004378 0.055697 -0.074047 -
0.033208 0.028294 0.067322 0.071766 0.100032 0.105029 -
0.021689 0.000892 -0.014648 -0.057382 -0.052085 -0.047732 -
0.111587 0.145440 -0.071255 0.059321 -0.025768 -0.031516 -
0.119637 0.003891 0.017055 0.037181 0.019834
z6 0.011322 0.034540 -0.104844 -0.053775 0.089475 -0.002553
-0.072400 0.008293 -0.085486 -0.067234 0.120535 -0.146553
-0.008963 -0.109544 -0.079596 -0.124812 -0.025704 -0.102107
0.017655 -0.031267 0.005177 0.031056 -0.045266 0.040377 -
0.045667 0.124313 -0.035800 0.039372 -0.055404 0.023486 -
0.007867 0.027276 -0.026404 -0.002572 0.019978
x7 -0.088852 0.109293 -0.020027 0.024753 0.074440 -0.000715
-0.104462 -0.043453 -0.190272 -0.114660 -0.140643 0.033411
0.161006 0.022401 0.049090 0.190816 0.012020 0.056590 -
0.044021 0.084860 0.044386 -0.012701 0.020590 -0.004287 -
0.025166 0.039267 0.114178 0.050935 0.088543 0.001261
0.010471 -0.018311 0.050414 0.049111 0.110867
y7 -0.008190 0.040469 -0.076900 -0.112819 0.081448 -0.104192
-0.013038 0.053651 0.054513 -0.034923 0.181991 -0.037841
-0.072280 0.100138 0.282338 0.013554 0.248250 0.067504
-0.007610 0.059022 0.030813 -0.156529 0.013078 0.070702
-0.076105 0.067679 0.154336 0.021024 -0.077381 -0.028705
0.085805 -0.004559 0.055081 0.067844 0.126484
z7 -0.005278 0.036978 -0.092422 -0.064093 0.082424 -0.010688
-0.097887 0.041754 -0.085485 -0.071371 0.088270 -0.168041
-0.036764 -0.104428 -0.151866 -0.110889 -0.033161 -0.027191
0.023932 0.014952 0.012259 0.026788 -0.036433 -0.005336 -
0.015092 -0.024176 0.032183 0.033418 -0.151961 0.051277
0.068814 0.056311 -0.084098 -0.160465 -0.051531
x8 0.125742 0.021424 -0.126305 0.117991 0.053168 0.013151
0.002383 0.092310 -0.051606 0.077553 -0.174329 0.083152 -
0.208249 -0.023814 -0.005841 0.027455 0.047287 -0.152632
0.054481 0.045188 -0.156046 0.066801 0.009589 -0.012994 -
0.005323 0.038315 0.038539 0.122122 0.001628 0.034373
0.022721 -0.130186 -0.014889 -0.005410 -0.007486
y8 -0.194042 0.099495 0.026202 -0.004031 -0.013700 0.125587
-0.047574 0.105077 0.043799 -0.157033 0.072964 0.124898
0.028718 0.207894 -0.169326 -0.044833 -0.028555 0.032112
0.043386 -0.079900 -0.069182 -0.038806 -0.026164 0.014704 -
0.148291 -0.086258 -0.041012 -0.038605 0.095497 0.315651
0.008194 -0.250923 0.072908 -0.029658 -0.196575
z8 -0.011485 0.010151 0.048995 -0.040667 0.054780 -0.137765
-0.115011 -0.026732 -0.020844 0.034040 -0.036869 0.066556
-0.059975 0.072368 -0.006041 0.060755 -0.153028 -0.039481

0.011216 -0.002842 0.052599 -0.019892 -0.133230 0.033153
0.146044 0.109747 -0.063350 -0.065075 -0.062107 0.038916
0.029352 0.031530 -0.039211 0.031336 -0.037581
x9 0.095639 0.046579 -0.110146 0.110885 0.061132 0.055050
-0.001722 0.045850 -0.053564 0.077065 -0.100663 0.008823
-0.140097 0.153986 -0.104575 0.060569 -0.002075 -0.072219
-0.051331 -0.135733 0.046278 0.012540 -0.054940 -0.150525
-0.148042 -0.018156 0.076243 -0.063171 0.161982 0.048739
0.004880 0.140214 -0.057981 -0.043265 -0.122491
y9 -0.163579 0.094661 0.003703 -0.018408 0.013810 0.078398
-0.015849 0.111125 0.059668 -0.102398 0.005844 0.077823
0.039406 0.109121 -0.041131 -0.003287 -0.052153 -0.038082 -
0.035781 -0.096942 0.019693 -0.035792 -0.017179 -0.094430 -
0.084532 0.009751 -0.025896 0.002570 0.046149 0.008102
0.032321 0.165001 0.086145 0.040176 -0.040532
z9 -0.011786 0.013493 0.031322 -0.026411 0.022079 -0.144621
-0.105546 -0.032203 0.005606 0.052502 0.022653 0.046215
-0.016749 0.014196 -0.001394 0.037123 -0.118148 -0.041793
0.025312 -0.073972 0.023019 0.015412 0.018458 0.023230
0.077633 0.078833 0.009313 -0.044567 -0.027051 0.048279 -
0.122490 -0.018606 0.020345 -0.032512 -0.010986
x10 0.054059 0.056281 -0.098224 0.098146 0.063796
0.073047 -0.029543 0.036977 -0.043706 0.096910 -0.043144 -
0.017479 -0.074290 0.193291 -0.070686 0.048590 0.030570
0.020470 -0.045775 -0.154780 0.117121 -0.045287 -0.096812 -
0.124779 -0.071470 -0.050125 0.018950 -0.114124 0.070347 -
0.026076 0.021839 0.184306 0.006982 0.063506 -0.122219
y10 -0.149424 0.083047 -0.015603 -0.031426 0.033967
0.031981 -0.010228 0.133506 0.075273 -0.046450 -0.020739
0.069150 0.041407 -0.041415 0.110705 -0.010686 -0.001052 -
0.057565 -0.002000 -0.014797 -0.000363 -0.019177 0.026624 -
0.084239 0.098053 0.050847 -0.063167 0.128573 -0.074607 -
0.112548 0.098309 0.147325 0.095507 0.087895 -0.014975
z10 -0.012079 0.015042 0.017889 -0.015756 -0.005794 -
0.140695 -0.091594 -0.026041 0.023409 0.060992 0.065613
0.031492 0.011682 -0.017720 0.006023 -0.003700 -0.066317 -
0.055840 0.031310 -0.108032 -0.011944 0.018118 0.115436
0.036476 0.026401 0.037873 0.057257 -0.017375 0.036962
0.062059 -0.123376 -0.058926 0.037033 -0.061576 0.012838
x11 0.021467 0.055425 -0.077982 0.102442 0.062589
0.074391 -0.038707 0.062607 -0.037623 0.109503 -0.000477 -
0.006984 0.020818 0.103482 -0.022733 0.031648 0.028581
0.062408 0.005058 -0.130695 0.047018 -0.034919 -0.010865 -
0.097339 -0.056542 -0.011936 -0.069070 -0.045030 -0.175856 -
0.097958 -0.121546 -0.003469 -0.026109 -0.098245 -0.019100
y11 -0.124243 0.071416 -0.024846 -0.030840 0.047674 -
0.000033 0.004325 0.145377 0.078483 -0.012659 -0.037236
0.058999 0.052923 -0.141655 0.159411 -0.004027 0.013028 -
0.076730 0.018635 0.026430 -0.008310 0.015613 0.038555 -
0.097163 0.139336 0.056658 -0.051457 0.187459 -0.139734 -
0.069341 0.095433 0.057001 0.079960 -0.010910 0.019562
z11 -0.010673 0.015918 0.006903 -0.004873 -0.024878 -
0.129735 -0.078719 -0.022238 0.029110 0.068657 0.091848
0.017239 0.034433 -0.033110 0.000052 -0.034830 -0.022583 -
0.063276 0.031062 -0.123082 -0.021699 0.025582 0.151840
0.030250 -0.008721 0.006977 0.085404 0.018131 0.059299
0.056977 -0.120680 -0.088579 0.055770 -0.081130 0.005836
x12 -0.011494 0.053817 -0.069927 0.095213 0.040992
0.022778 -0.039411 0.114323 -0.010952 0.138474 0.076407
0.013248 0.167984 0.039388 0.041985 -0.012208 -0.026081

0.086969	0.015453	-0.032390	0.061832	0.041088	0.087014	
0.001163	0.160396	-0.019820	-0.076535	0.108317	-0.133711	-
0.192062	-0.044364	-0.204754	-0.117298	0.113140	0.223525	
y12	-0.056672	0.061072	-0.040503	-0.047298	0.066408	-
0.045576	0.033210	0.110904	0.090649	0.029643	-0.062308	
0.007447	0.035549	-0.145012	0.121663	0.008417	0.011250	-
0.091178	0.000083	0.064252	0.087712	0.053569	-0.016914	-
0.123748	0.111928	0.008710	0.025962	0.100225	0.010780	
0.036478	0.100242	0.062572	0.054722	-0.049593	-0.038125	
z12	-0.006894	0.017991	-0.009865	0.009603	-0.036960	-
0.092184	-0.052786	-0.002940	0.018575	0.073327	0.105173	-
0.000759	0.058562	-0.030238	-0.032227	-0.072882	0.060771	-
0.060381	0.011934	-0.068997	-0.025728	0.025510	0.108345	-
0.022058	-0.078408	-0.073744	0.077398	0.088583	0.090598	-
0.024628	-0.051145	-0.065227	0.091555	-0.015664	0.050751	
x13	-0.011364	0.057629	-0.061667	0.107439	0.029489	
0.022162	-0.026621	0.117105	-0.018168	0.120876	0.102529	
0.012397	0.195494	-0.020269	0.033467	-0.015023	-0.079424	
0.070411	-0.077648	-0.001719	0.032581	0.046009	0.060114	
0.118297	0.126875	-0.080431	-0.089340	0.031541	-0.167402	-
0.035704	-0.005506	-0.040593	-0.033718	0.098245	0.110784	
y13	-0.018267	0.054636	-0.036177	-0.065892	0.070241	-
0.061015	0.045540	0.081219	0.117003	0.053067	-0.068052	-
0.013831	0.026231	-0.114467	0.056635	-0.000864	0.023119	-
0.077705	-0.001975	0.099245	0.095408	0.063426	-0.028506	-
0.117035	0.049563	0.002722	0.102329	-0.003659	0.064283	
0.049375	0.033326	-0.004359	-0.031295	-0.099173	-0.067207	
z13	-0.003237	0.018817	-0.017297	0.015021	-0.030597	-
0.066737	-0.041119	0.005576	0.002215	0.064632	0.099478	-
0.006198	0.057790	-0.014315	-0.064356	-0.082248	0.103430	-
0.043211	-0.008120	-0.011510	-0.027060	0.025685	0.044167	-
0.059495	-0.118158	-0.117072	0.041839	0.122022	0.096887	-
0.102554	0.002865	-0.038748	0.082076	0.052609	0.013037	
x14	-0.009586	0.051382	-0.037508	0.093793	0.002353	
0.015934	-0.008598	0.111624	0.014865	0.135126	0.113374	
0.001959	0.229619	-0.051065	0.014932	-0.014686	-0.081093	
0.077476	-0.140655	0.095599	-0.040077	-0.017730	-0.007790	
0.219780	0.031057	-0.017698	-0.044093	-0.010283	-0.087408	
0.161465	0.092033	0.046258	0.059195	-0.010446	-0.038742	
y14	0.031453	0.047111	-0.035432	-0.083749	0.069637	-
0.086268	0.053932	0.045455	0.136195	0.067444	-0.064846	-
0.015860	0.018373	-0.087460	-0.039212	-0.021075	0.003148	-
0.047707	0.010726	0.148444	0.143114	0.115842	-0.020389	-
0.112336	-0.034571	-0.012174	0.206482	-0.176530	0.105733	-
0.033233	-0.159114	-0.047104	-0.074338	-0.116183	0.052297	
z14	0.001482	0.018972	-0.022860	0.017347	-0.017484	-
0.035037	-0.030168	0.018322	-0.018996	0.047905	0.084053	-
0.011645	0.042043	0.015857	-0.103161	-0.091128	0.149793	-
0.017359	-0.041067	0.073993	-0.031655	0.020468	-0.048236	-
0.102342	-0.161013	-0.156213	-0.001349	0.161206	0.123726	-
0.192679	0.115530	0.007159	0.040174	0.141676	-0.000962	
x15	0.245837	-0.127990	0.120720	-0.241732	-0.070771	
0.264009	-0.459777	0.246151	0.208576	0.065327	-0.053321	-
0.104618	-0.006348	0.022608	0.064074	-0.014407	-0.001754	
0.079859	0.109003	-0.063536	-0.030945	-0.048844	0.092925	-
0.004202	0.027664	0.019558	-0.005885	0.012263	0.099143	
0.029120	0.055180	0.136812	-0.074980	-0.014722	0.033869	
y15	-0.059021	0.049594	0.089326	0.373293	-0.027391	-
0.190456	0.177564	-0.147425	-0.043105	-0.201414	0.226118	
0.251300	-0.051135	-0.140463	-0.099129	0.002969	0.105666	

0.044815	0.035932	0.053763	-0.027157	0.190942	-0.032664	-
0.027564	0.079553	0.032921	-0.046245	-0.002782	-0.049790	
0.020671	-0.008161	0.212950	-0.040055	0.065452	-0.043609	
z15	-0.012968	-0.046706	0.083586	0.073585	0.067844	-
0.095568	0.065595	-0.017838	-0.000957	0.101076	-0.188164	
0.257483	0.086728	-0.161409	-0.134406	-0.129643	0.282711	
0.105475	0.099173	-0.220802	0.119101	-0.314643	0.108032	
0.050154	-0.061566	0.094918	0.004128	-0.021934	-0.002212	
0.139109	0.094727	0.091520	-0.077961	-0.028384	0.113307	
x16	0.142317	-0.069225	0.103871	0.008309	0.036823	-
0.023112	0.001077	-0.014400	0.134471	-0.050998	0.034178	
0.052720	0.076874	0.103236	-0.034339	-0.010582	0.178755	-
0.064263	-0.030072	-0.045673	-0.002157	0.167636	-0.069996	
0.019530	0.040049	-0.033374	-0.047071	-0.077844	-0.019120	
0.068671	0.062357	-0.170226	-0.040845	-0.035664	0.137105	
y16	0.064486	0.007163	0.015473	0.112024	-0.063552	
0.042048	-0.153567	-0.061874	0.087762	-0.098029	0.047294	
0.117042	-0.022647	-0.128455	0.000565	-0.021550	-0.073630	
0.045949	-0.081041	-0.027797	-0.089780	-0.051704	0.016611	-
0.023359	0.018444	-0.014177	0.028325	0.076993	0.049092	
0.048052	0.068542	0.165393	-0.084745	-0.069086	-0.059210	
z16	0.008609	-0.057788	0.053896	0.005563	0.066815	-
0.081698	-0.013871	-0.032290	0.009549	0.100264	-0.160446	
0.201881	0.094633	-0.026728	-0.011243	-0.089769	0.119266	
0.103383	0.109417	-0.100919	0.030879	-0.165758	-0.036141	-
0.000866	-0.013005	0.023489	-0.049428	0.019463	0.058136	-
0.015202	0.008658	0.039090	-0.018373	-0.073092	-0.060253	
x17	0.164924	-0.070560	0.113228	-0.009778	0.047964	-
0.054673	0.055982	0.019621	0.086091	-0.042720	0.022753	
0.046685	0.067219	0.071179	-0.065766	-0.086054	0.152916	
0.000409	-0.028443	-0.007305	-0.076479	0.122561	-0.060346	-
0.032734	0.102544	0.038635	-0.052460	0.040122	-0.020951	
0.055998	0.051617	-0.142967	0.004223	-0.111962	0.051062	
y17	0.079723	0.016352	-0.030136	0.086109	-0.025587	
0.046138	-0.116887	-0.145738	0.115198	-0.088904	-0.009272	
0.123972	0.039354	-0.047134	0.036523	-0.042522	-0.070411	-
0.064895	-0.194247	-0.044206	-0.076588	-0.063249	-0.019833	-
0.026044	-0.024291	-0.065724	-0.001903	0.058146	0.067265	
0.041394	0.077760	0.025863	-0.103650	-0.134040	0.057740	
z17	0.001448	-0.060222	0.027143	-0.015235	0.062334	-
0.094279	-0.035457	-0.051764	0.029143	0.097469	-0.106446	
0.195140	0.073896	0.063047	0.065366	-0.017822	0.015280	
0.090577	0.132015	-0.004556	-0.014561	-0.020691	-0.126232	-
0.010205	0.026346	-0.032123	-0.092983	0.063929	0.103079	-
0.114427	-0.057157	-0.037926	0.041937	-0.040287	-0.068630	
x18	0.176232	-0.069791	0.112262	-0.015209	0.057878	-
0.077718	0.080956	0.035085	0.053277	-0.044312	0.025891	-
0.005426	0.043171	0.039591	-0.108776	-0.080674	0.099125	
0.068138	-0.014712	-0.000335	-0.025078	0.102062	-0.051353	
0.002704	0.045661	0.075326	0.051484	-0.071635	-0.123077	
0.093361	-0.022524	-0.043609	-0.076355	0.044482	-0.066347	
y18	0.097982	0.025117	-0.057011	0.069151	0.005395	
0.044445	-0.084811	-0.184920	0.112282	-0.070253	-0.041762	
0.107391	0.078449	0.010827	0.047770	-0.072393	-0.077929	-
0.128300	-0.224178	-0.044666	-0.073988	-0.059283	-0.005068	-
0.033799	-0.022158	-0.094219	-0.061048	0.023289	0.023817	
0.078252	0.030314	-0.089663	-0.121672	-0.091638	0.078694	
z18	-0.007830	-0.062985	0.009903	-0.032017	0.059251	-
0.099780	-0.048858	-0.060888	0.038305	0.093757	-0.054591	
0.181556	0.046500	0.125532	0.119146	0.035231	-0.059281	

0.075240	0.118325	0.058001	-0.042961	0.060440	-0.159436	
0.008864	0.035797	-0.086345	-0.092491	0.049400	0.123086	-
0.136041	-0.105719	-0.059379	0.066720	0.017838	-0.027426	
x19	0.157093	-0.081020	0.116261	-0.001232	0.017517	-
0.079062	0.110152	0.089688	0.028224	-0.070897	0.061586	-
0.035345	-0.036223	-0.064306	-0.062094	-0.011237	0.012317	
0.122082	0.008570	0.007709	0.048170	0.005080	-0.055627	
0.013538	-0.130111	0.084879	0.172265	-0.064669	-0.151276	-
0.134416	0.035079	-0.067166	0.077265	0.171245	-0.172893	
y19	0.123232	0.036110	-0.091798	0.068539	0.023540	
0.048046	-0.048880	-0.170237	0.110895	-0.034568	-0.048564	
0.032816	0.066338	0.015740	0.030561	-0.060948	-0.100033	-
0.104217	-0.098508	-0.024399	0.046123	-0.024397	0.061586	-
0.015246	-0.053046	-0.094654	-0.074024	-0.026824	-0.070707	
0.030559	-0.009665	-0.164259	0.001635	0.173366	-0.060573	
z19	-0.030248	-0.065849	0.002002	-0.061413	0.056973	-
0.088223	-0.046806	-0.046409	0.009346	0.078537	0.027016	
0.125919	-0.026794	0.167008	0.121297	0.052774	-0.113886	
0.048268	0.016632	0.104732	-0.064124	0.136180	-0.062878	-
0.003300	0.022561	-0.105710	-0.026834	-0.038484	0.028712	-
0.083457	-0.078047	-0.010651	0.092595	-0.025203	0.003095	
x20	0.134941	-0.091375	0.128723	-0.000106	0.004744	-
0.073798	0.120324	0.088780	0.023788	-0.070211	0.059021	-
0.000372	-0.009505	-0.039602	-0.030808	0.011226	-0.061419	
0.067319	0.002508	-0.024319	0.003447	-0.048939	-0.067612	
0.007835	-0.131735	0.090360	0.181122	0.014110	-0.111566	-
0.170152	0.058213	-0.025427	0.096692	0.008789	-0.118617	
y20	0.131781	0.038015	-0.094339	0.073590	0.040701	
0.053970	-0.036838	-0.133932	0.097231	-0.021185	-0.052683	-
0.014058	0.073348	0.016159	-0.011104	-0.074452	-0.088794	-
0.018476	0.007944	0.004705	0.083076	0.017128	0.101360	-
0.021465	-0.068541	0.007840	-0.001650	-0.062469	-0.097552	-
0.079004	0.068463	-0.069558	0.153041	0.050811	-0.139086	
z20	-0.046605	-0.068324	0.002689	-0.072989	0.049280	-
0.075249	-0.037917	-0.029005	0.001186	0.073097	0.062541	
0.093845	-0.068376	0.146327	0.072087	0.048535	-0.110279	
0.026899	-0.047159	0.080642	-0.041804	0.131702	0.053985	-
0.014379	0.033053	-0.065738	0.046982	-0.088177	-0.061194	-
0.004194	-0.019972	0.018177	0.056262	-0.045886	0.001260	
x21	0.111552	-0.108553	0.143082	-0.004292	-0.022976	-
0.076437	0.133879	0.069252	0.015065	-0.071629	0.051570	
0.055252	0.007154	0.021901	0.032780	0.040617	-0.186043	-
0.086154	-0.043705	-0.053051	-0.021160	-0.165682	-0.101229	-
0.018484	-0.090784	-0.028833	0.042126	0.138710	-0.082160	-
0.108245	-0.027837	-0.026803	-0.024190	0.007992	0.052989	
y21	0.147195	0.030677	-0.091141	0.072662	0.059452	
0.053290	-0.028278	-0.114174	0.073690	-0.001015	-0.068521	-
0.043117	0.080899	0.048698	-0.059349	-0.089409	-0.118864	
0.067367	0.161165	0.031790	0.135306	-0.017392	0.121312	-
0.036302	-0.012606	0.067727	0.013898	-0.062963	-0.220542	-
0.083142	0.075074	-0.009452	0.059893	0.006688	-0.019894	
z21	-0.066872	-0.071006	0.009684	-0.086439	0.034920	-
0.059506	-0.022333	-0.007830	-0.002722	0.071043	0.097511	
0.060715	-0.125158	0.108255	-0.003049	0.035773	-0.111431	-
0.002756	-0.106104	0.024127	0.015505	0.072447	0.214539	-
0.024549	0.059539	0.000511	0.127847	-0.123200	-0.121406	
0.122194	0.032001	0.067506	-0.006898	-0.021580	0.003263	
x22	-0.058778	-0.173257	-0.271583	-0.017588	-0.106006	-
0.181360	0.031277	0.007616	0.052576	-0.223900	0.068195	-
0.035356	-0.062925	-0.086023	-0.017274	0.112884	-0.084200	

0.102433	0.100051	-0.161953	0.072783	0.051090	0.063255	
0.030446	0.107993	-0.102163	0.031544	-0.011227	0.072425	
0.079463	0.017995	-0.189146	-0.058236	0.072077	0.110118	
y22	-0.190941	-0.560682	-0.278565	0.149208	-0.295921	-
0.121393	-0.170862	-0.000024	0.044278	0.020068	-0.155669	-
0.101410	0.034602	-0.088776	0.017059	0.082305	0.016174	
0.041567	0.055794	-0.134736	0.071229	0.093625	-0.107427	
0.164996	-0.162051	-0.059433	0.042612	0.043247	-0.112668	
0.003764	-0.009033	0.081507	-0.001648	0.035623	-0.044086	
z22	0.038249	0.085042	0.027411	-0.027136	0.048276	
0.109433	0.122318	0.058946	-0.017031	-0.185689	-0.124960	-
0.064558	-0.139875	-0.090471	0.139873	-0.025840	-0.034084	
0.109008	-0.139138	0.017553	0.050541	0.115168	-0.054388	
0.101524	-0.031638	-0.198276	-0.162583	-0.106641	-0.065901	-
0.008586	-0.126074	0.107015	0.086404	-0.100731	0.029499	
x23	-0.005824	-0.057431	-0.032263	-0.024148	-0.230238	-
0.298927	-0.013354	-0.027575	-0.150443	-0.210008	-0.151262	-
0.178669	0.095605	0.150992	0.069396	-0.207908	0.073161	
0.002607	-0.095007	0.176427	-0.090364	-0.087035	0.197113	-
0.190834	0.164704	0.056898	-0.155000	-0.118696	0.000599	-
0.013807	0.019403	-0.000930	-0.056633	-0.139687	-0.074655	
y23	0.024779	-0.084787	0.026425	0.055583	-0.198553	-
0.115095	-0.062919	-0.024899	-0.223704	0.015259	-0.183422	-
0.168302	0.063487	0.200842	0.044405	-0.159364	0.144514	-
0.050333	-0.184808	0.030433	-0.169607	0.048235	-0.037747	-
0.045830	-0.038482	0.081957	-0.082113	-0.148283	0.010281	-
0.060351	0.029385	0.106069	0.095356	0.025413	0.063467	
z23	0.025331	0.087328	-0.010102	-0.004803	-0.005334	
0.116501	0.106597	0.014820	0.041804	-0.118503	-0.110662	-
0.041892	-0.082185	-0.120448	0.116886	0.069394	-0.047280	
0.114766	0.055844	-0.022357	0.042151	0.096021	-0.070699	
0.129808	-0.054872	-0.142305	-0.099459	-0.055552	-0.102583	
0.004491	0.027703	-0.014250	-0.101325	-0.059868	-0.001604	
x24	-0.020363	0.037451	0.026722	-0.070209	0.003669	-
0.041463	0.027574	-0.188289	0.034998	-0.098020	-0.039541	-
0.094362	-0.092546	0.040327	0.002241	-0.058529	0.048748	-
0.028323	0.056373	0.178196	0.086407	-0.239298	0.115095	
0.156521	0.176285	-0.140835	0.043246	-0.015204	-0.049069	
0.038877	-0.152633	-0.013997	-0.007507	-0.152367	-0.189763	
y24	0.049196	0.022181	0.010400	-0.000244	0.022401	
0.086755	0.004601	-0.112526	-0.105002	0.093625	-0.001619	-
0.054551	-0.094029	0.070747	-0.002435	0.033532	0.076712	
0.004739	-0.112609	-0.147915	-0.097997	0.072599	-0.088169	
0.125603	0.033768	-0.061500	0.113871	-0.064235	0.017638	-
0.010525	-0.036678	-0.020051	0.113257	0.050093	0.073082	
z24	0.019441	0.065947	-0.017708	0.006324	-0.062935	
0.100228	0.102077	-0.014138	0.006942	-0.063779	-0.105372	-
0.057819	0.020894	-0.067801	0.129379	0.031108	-0.041391	
0.107190	0.135059	-0.055578	-0.019996	0.107611	-0.060498	
0.070172	-0.082868	-0.020065	-0.006241	-0.045132	-0.062356	
0.057551	0.102302	-0.034226	-0.087640	-0.103740	-0.086476	
x25	-0.057758	0.017548	0.085265	-0.086430	0.015002	
0.074289	-0.019489	-0.264891	0.063507	-0.033434	0.014182	-
0.065205	-0.024961	0.007016	0.022425	-0.067275	0.059285	
0.015058	-0.011648	-0.031294	0.018133	-0.050836	-0.023756	
0.077112	0.089502	-0.008040	0.149578	0.216212	0.022177	-
0.060177	-0.126245	-0.001443	-0.038897	-0.006259	-0.189376	
y25	0.075777	0.030747	-0.020656	-0.021374	0.022150	
0.070547	0.030276	-0.092313	-0.139274	0.140307	0.052465	-
0.030809	-0.090204	-0.007460	0.020149	0.069685	0.045369	

0.098234	-0.154716	-0.216121	-0.144666	0.124737	-0.044706	-
0.031297	0.095022	-0.022410	0.019630	0.019030	-0.010827	-
0.022575	0.035165	-0.064373	0.031487	0.042811	-0.005607	
z25	0.020864	0.048478	-0.026334	0.027617	-0.088768	
0.118952	0.107815	-0.037259	-0.015431	-0.015756	-0.056199	-
0.054159	0.092431	0.004686	0.125054	0.003397	-0.030797	
0.048955	0.186325	-0.031743	-0.082897	0.072903	-0.034376	-
0.027521	-0.041477	0.109099	0.097514	0.003301	-0.033510	
0.102254	0.162744	-0.011454	-0.064042	-0.138775	0.000584	
x26	-0.065008	0.007579	0.088771	-0.100782	0.011317	
0.077997	-0.029360	-0.283406	0.054447	-0.010943	0.000860	-
0.044933	-0.016614	-0.019096	0.017217	-0.051771	0.063511	
0.056090	-0.006320	-0.105404	-0.020073	0.060308	-0.068935	
0.018128	0.074925	-0.029459	0.154880	0.134748	-0.029169	-
0.014255	-0.047578	0.090256	-0.076187	0.097692	-0.108640	
y26	0.086805	0.027011	-0.021176	-0.018094	0.014615	
0.042888	0.039178	-0.074288	-0.149391	0.161431	0.057679	-
0.033246	-0.091897	-0.037901	0.014297	0.070472	0.033664	
0.111084	-0.117662	-0.147247	-0.098309	0.054049	-0.002989	-
0.049012	0.095769	-0.003993	-0.064905	0.023349	-0.028028	-
0.007968	0.027559	-0.075152	0.007883	-0.034476	-0.041512	
z26	0.023787	0.037594	-0.019924	0.038684	-0.094790	
0.120721	0.104526	-0.033062	-0.018625	-0.011733	-0.027333	-
0.063340	0.107713	0.032160	0.105535	0.001968	-0.021640	-
0.000510	0.152592	-0.005721	-0.090755	0.022544	-0.013079	-
0.054579	-0.018666	0.135299	0.096913	0.021952	-0.017826	
0.081550	0.096751	0.004723	-0.091568	-0.060221	0.029147	
x27	-0.059284	0.000125	0.095195	-0.104584	0.010409	
0.064177	-0.037275	-0.296522	0.047844	0.004635	0.020931	-
0.039571	-0.025749	-0.086657	0.010304	-0.041593	0.040052	
0.036655	0.022438	-0.061399	-0.023041	0.045950	-0.040528	-
0.053940	0.068468	0.047999	0.048152	0.066748	-0.091381	
0.028240	-0.066856	0.178560	-0.049506	0.126073	0.039583	
y27	0.094652	0.017017	-0.013189	-0.001769	0.002170	
0.019702	0.043453	-0.047865	-0.152783	0.178607	0.064932	-
0.043586	-0.074011	-0.047408	-0.003634	0.076553	0.016624	
0.113811	-0.049204	-0.039474	-0.015628	-0.050565	0.027607	-
0.019650	0.024495	0.055023	-0.159586	0.013520	0.024656	
0.000659	0.003223	-0.140144	-0.017069	-0.166362	-0.039813	
z27	0.029705	0.022947	-0.006739	0.051078	-0.098766	
0.118699	0.100856	-0.021003	-0.016397	-0.023688	0.004515	-
0.084570	0.118933	0.061285	0.072754	0.007929	-0.006080	-
0.063229	0.083894	0.019757	-0.080241	-0.053457	0.013794	-
0.064301	-0.001135	0.145340	0.051849	0.023585	0.036161	
0.004780	-0.015244	0.008888	-0.082689	0.052038	0.024539	
x28	-0.054975	-0.008619	0.107487	-0.108159	0.000279	
0.050729	-0.037642	-0.295854	0.052297	0.029253	0.002531	-
0.057051	-0.044849	-0.134820	-0.027953	0.011138	0.045810	
0.042628	0.071561	0.039819	-0.026967	-0.010015	0.012152	-
0.048651	-0.075336	0.090094	-0.170469	-0.088890	0.038189	
0.018716	0.151447	-0.016276	0.244280	-0.105020	0.242232	
y28	0.101280	0.005138	-0.009682	0.023803	-0.008692	-
0.012503	0.053245	-0.005621	-0.165428	0.187498	0.083143	-
0.038547	-0.047585	-0.046994	-0.026451	0.073711	-0.040361	
0.114480	0.048185	0.069919	0.122897	-0.154836	0.056450	-
0.013683	-0.033537	0.164689	-0.240587	-0.003664	0.019900	
0.071095	-0.054360	-0.055781	-0.199220	-0.103693	0.043941	
z28	0.039203	0.005767	0.009937	0.062356	-0.098339	
0.110507	0.096912	0.003087	-0.014122	-0.059445	0.049652	-
0.105047	0.124994	0.091491	0.033877	0.018506	-0.001572	-

0.143567	-0.042119	0.050005	-0.068153	-0.140799	0.004895	-
0.064539	0.028509	0.153771	0.009942	0.042896	0.076551	-
0.115089	-0.210298	0.061669	-0.113846	0.186232	-0.022573	
x29	-0.206830	0.013082	0.002610	0.059083	-0.231115	
0.112692	0.062749	0.033562	-0.008982	0.154397	0.024262	
0.060007	-0.244944	0.003671	0.078316	-0.380468	-0.149820	
0.076179	-0.111516	0.052398	0.314222	0.081829	-0.100023	-
0.073344	-0.047412	0.186486	-0.040966	0.144373	0.154087	-
0.056634	-0.013033	-0.080952	0.029667	-0.072180	0.096777	
y29	-0.003513	-0.109221	0.256303	-0.025735	-0.002377	
0.088774	-0.113423	0.073421	-0.360862	-0.045405	0.096437	
0.007479	0.130501	-0.163629	0.106149	0.034645	0.070769	-
0.209385	0.197751	-0.043737	0.135089	-0.059044	-0.231129	-
0.286729	0.015816	-0.216229	0.101479	-0.229063	-0.157488	
0.164847	-0.182284	-0.065822	0.045251	0.017855	0.142932	
z29	-0.007903	-0.078923	0.040629	0.081888	-0.215057	
0.214269	0.132696	-0.025610	0.050040	0.015144	-0.007934	
0.090250	-0.040127	0.124560	0.056703	0.186400	0.082043	
0.149340	-0.040936	0.165130	0.140798	-0.015339	0.134367	-
0.103455	-0.004739	-0.144505	0.088955	0.005186	-0.140932	-
0.035297	0.106064	0.011507	-0.085858	-0.085947	0.067153	
x30	0.180369	-0.006869	-0.148000	0.118901	0.041226	-
0.041757	0.016578	0.113604	-0.051818	0.074558	-0.269659	
0.146936	-0.292929	-0.190295	0.085348	0.068449	0.051712	-
0.224812	0.078630	0.191717	-0.250289	0.041348	0.051107	
0.016652	-0.021830	0.116865	0.070985	0.081193	-0.027260	
0.066111	-0.028888	-0.073016	0.077460	0.058352	-0.019368	
y30	-0.195182	0.080217	0.023572	0.018765	-0.027830	
0.144098	-0.096899	0.134862	0.043314	-0.231795	0.004226	
0.206662	-0.075836	0.184522	-0.128936	-0.084749	0.037970	-
0.003133	0.115172	-0.029077	-0.129772	-0.033884	0.070521	
0.131652	-0.133894	0.209151	-0.041774	-0.155547	-0.161526	-
0.205002	-0.154615	-0.211416	-0.145251	0.007748	-0.004616	
z30	-0.009288	0.008055	0.066389	-0.056737	0.097018	-
0.124730	-0.127611	-0.015658	-0.056868	-0.006840	-0.093326	
0.075309	-0.119104	0.138236	-0.026229	0.095143	-0.157271	-
0.005978	-0.037066	0.110282	0.085250	-0.057675	-0.257583	
0.012110	0.117510	0.170345	-0.106088	-0.117523	-0.109241	
0.023487	0.249388	0.102549	-0.099813	0.162460	-0.108346	
x31	0.053633	-0.038527	0.172122	0.111561	0.016397	
0.019751	-0.070391	0.090587	0.087633	-0.091585	0.128865	-
0.008448	-0.005418	0.156610	0.072115	0.074963	0.203865	-
0.159885	0.153398	0.029612	0.178205	0.383661	0.150376	
0.065151	-0.016727	0.034595	-0.313364	0.116822	0.110236	
0.197976	-0.046394	0.176632	0.034058	0.010264	-0.058962	
y31	0.036119	-0.020181	0.151491	0.148680	-0.096329	
0.037111	-0.273964	0.107851	0.015029	-0.115563	0.143962	
0.021173	-0.174139	-0.172591	-0.123683	0.045444	-0.134866	
0.254630	0.108673	0.137335	-0.275522	-0.036309	0.012226	-
0.065603	0.196490	0.045398	0.142473	-0.077806	0.155198	-
0.138239	-0.069750	0.062346	0.027045	-0.070970	0.082878	
z31	-0.168754	0.035143	0.335714	0.435167	0.287786	-
0.110814	-0.016049	0.134075	0.147585	-0.038676	-0.193014	-
0.426636	-0.134355	-0.050091	0.123935	-0.039049	-0.040695	
0.110586	-0.111616	-0.158797	-0.011132	-0.087200	0.102603	
0.017709	-0.030876	-0.084929	-0.014018	-0.043621	0.103358	-
0.020656	-0.013266	-0.085218	0.040509	0.041477	0.055371	
x32	0.130563	0.099324	-0.237943	-0.137155	0.013410	-
0.104216	0.182136	0.056377	-0.089456	-0.263576	0.127152	
0.010396	-0.073835	0.057694	-0.046805	-0.005948	-0.116111	

0.138862	0.154660	-0.239915	0.064615	-0.028318	0.002607	-
0.075927	0.059558	-0.020952	-0.022624	-0.066443	0.285448	-
0.076787	0.158339	0.117454	0.116659	-0.062384	0.192726	
y32	-0.011798	0.022531	0.045003	0.115512	0.009538	
0.069733	0.045960	-0.081631	-0.045172	-0.001454	-0.082780	-
0.063655	-0.124179	0.290932	-0.131728	-0.033619	-0.012621	-
0.119853	0.162400	0.172115	0.056682	-0.088693	-0.176078	
0.385638	0.053736	-0.007487	0.170141	0.145444	0.022070	
0.056439	0.039928	0.051052	0.118971	-0.046917	0.402036	
z32	0.142301	0.006840	-0.014112	-0.243923	-0.104980	
0.126278	0.016258	0.151702	-0.147716	-0.179236	-0.201047	
0.219771	-0.078068	-0.163585	-0.074013	-0.157049	0.106328	
0.095223	-0.254912	0.006191	0.072221	-0.025834	0.102397	
0.173961	0.017524	-0.082924	-0.089770	0.032131	-0.071698	
0.045237	-0.220757	0.063264	0.216489	0.144333	0.061029	
x33	-0.154911	-0.123086	-0.060206	0.011956	0.276318	
0.129742	-0.056612	-0.043796	-0.264404	-0.160901	-0.184413	
0.041154	0.202910	-0.048175	-0.043160	0.075218	-0.143742	
0.199598	-0.089365	0.144747	0.074420	0.056462	0.073575	-
0.073688	0.069322	0.089081	0.114005	0.110426	0.233208	
0.169531	-0.026365	-0.155069	-0.041835	0.179501	-0.051488	
y33	0.018763	0.037143	-0.120852	-0.093895	0.090533	-
0.108224	-0.068267	0.064538	0.008204	-0.082054	0.157207	-
0.041136	-0.080078	0.091872	0.362526	-0.063087	0.198387	
0.156474	-0.051565	0.062297	0.012464	-0.129073	-0.184418	
0.096515	-0.047515	-0.063135	0.008517	-0.002961	0.069210	
0.142429	0.040629	-0.047467	-0.152785	0.119325	-0.067741	
z33	0.087484	0.336796	0.060398	-0.032332	-0.290976	-
0.165376	-0.177194	0.034446	-0.044179	-0.073218	-0.080300	-
0.141944	0.020483	-0.062246	-0.273420	0.122056	0.010592	
0.024557	0.028156	0.088272	0.063851	0.022473	-0.249940	-
0.077708	-0.050590	-0.207139	-0.103794	0.270419	-0.052762	-
0.057384	0.062319	-0.134684	-0.097879	0.059637	-0.029250	
x34	0.045966	0.104087	0.014379	0.076197	-0.111483	-
0.047544	0.007160	0.079743	0.054172	0.156526	0.116041	
0.025895	0.309556	-0.110213	-0.029870	0.002423	-0.090922	
0.119497	-0.187469	0.248023	-0.018809	-0.005987	-0.161000	
0.213720	-0.199502	0.059011	0.128593	-0.175016	0.118644	
0.106223	-0.017365	0.121956	0.147167	-0.156063	-0.037514	
y34	0.079119	0.068325	-0.048875	-0.078028	0.041319	-
0.106971	0.088938	0.027371	0.149041	0.061975	-0.099649	-
0.013097	-0.028366	-0.040825	-0.061566	-0.075104	0.027839	-
0.098110	-0.002529	0.118382	0.156725	0.077489	0.023359	
0.004539	-0.030298	-0.148775	0.148652	-0.171335	0.170497	-
0.048240	-0.100398	0.002687	-0.340694	0.111015	0.100632	
z34	-0.110633	-0.289762	-0.148200	-0.024311	0.345255	
0.146783	0.014879	0.039777	-0.071176	0.040712	0.163254	-
0.057008	-0.086214	0.019595	-0.255064	-0.239310	0.088728	
0.067779	-0.029421	0.264801	-0.129756	0.013177	0.035311	-
0.137329	-0.068240	-0.176231	-0.029984	0.045790	-0.108210	-
0.015050	0.144116	0.082666	-0.093499	-0.049392	0.037606	
x35	0.038336	-0.224594	0.116716	-0.017362	0.042958	-
0.034206	0.173487	0.077133	-0.011166	-0.087603	0.098683	
0.085759	-0.019620	0.045753	0.112940	0.018725	-0.302093	-
0.250202	-0.122580	-0.077053	-0.056851	-0.260461	-0.047328	-
0.054829	-0.084318	-0.156264	-0.089882	0.170801	-0.031664	
0.007156	-0.035052	0.072687	-0.047946	-0.162470	0.052585	
y35	0.162664	0.030456	-0.082666	0.065728	0.031776	
0.015716	0.015118	-0.092977	0.052215	0.037402	-0.069335	-
0.084498	0.065659	0.067002	-0.086800	-0.115803	-0.187190	-

0.002398	0.229719	0.049760	0.158378	-0.035199	0.115538	-
0.078254	0.080699	-0.108468	-0.056654	-0.057888	-0.056304	-
0.029009	-0.072606	0.119429	0.274877	0.129105	0.018189	
z35	-0.022936	0.117575	0.101086	-0.062512	-0.190921	-
0.123355	-0.038203	0.031772	-0.003972	0.065693	0.058628	
0.043073	-0.145860	0.060557	-0.057305	0.120897	-0.045881	-
0.057442	-0.211173	-0.083171	0.119113	-0.015417	0.291304	-
0.028915	-0.009475	0.061622	0.201925	-0.023289	-0.094329	
0.157953	0.177737	0.002169	-0.008615	0.182538	0.088634	
x36	-0.095353	-0.034466	0.114499	-0.105577	-0.032519	
0.017529	-0.019897	-0.220493	0.055112	0.046664	0.063013	
0.009165	-0.130949	-0.191074	0.055285	0.104011	-0.091355	-
0.064707	0.071806	0.184985	-0.065012	-0.050434	0.108444	-
0.034543	-0.396250	-0.012318	-0.268929	-0.184085	0.086076	-
0.024397	0.116331	-0.106470	-0.048480	0.264899	0.077321	
y36	0.111854	0.000304	-0.002717	0.049339	-0.066518	-
0.026155	0.107524	0.061413	-0.158411	0.158052	0.139295	-
0.077414	-0.057844	0.014987	-0.003995	0.053594	-0.038359	
0.055178	0.217105	0.080999	-0.050194	-0.171728	0.175733	
0.167386	0.004901	-0.129444	-0.106894	0.141732	0.101823	
0.171386	-0.133446	0.124826	-0.038290	0.178242	-0.205630	
z36	0.007857	-0.167553	-0.134731	0.101079	-0.000433	
0.167243	-0.012199	-0.002509	0.200114	-0.056323	0.127686	-
0.084490	-0.057541	-0.101458	-0.197707	0.320926	0.210287	-
0.169318	-0.199557	0.065800	0.247212	-0.208077	-0.158706	
0.018476	0.270554	0.160033	-0.139582	-0.107273	0.108577	-
0.076212	-0.028375	-0.112680	0.131360	-0.041832	-0.057651	

Canonical Variate Analysis: CVA ... cochlea thickness

Dataset: cochlea thickness
Classification criterion: subfam

Groups		Observations
1.	Ca	9
2.	Ce	13
3.	Di	3
4.	Pr	11

Variation among groups, scaled by the inverse of the within-group variation

	Eigenvalues	% Variance	Cumulative %
1.	6.13746041	50.357	50.357
2.	4.12544622	33.849	84.206
3.	1.92493133	15.794	100.000

Mahalanobis distances among groups:

	Ca	Ce	Di
Ce	5.3979		
Di	5.5600	5.7266	
Pr	5.2722	5.7029	5.8131

P-values from permutation tests (10000 permutation rounds) for Mahalanobis distances among groups:

	Ca	Ce	Di
Ce	<.0001		

Di	<.0001	0.0017	
Pr	<.0001	<.0001	0.0019

Procrustes distances among groups:

	Ca	Ce	Di
Ce	0.0544		
Di	0.0465	0.0685	
Pr	0.0404	0.0659	0.0400

P-values from permutation tests (10000 permutation rounds) for Procrustes distances among groups:

	Ca	Ce	Di
Ce	0.0324		
Di	0.8621	0.0341	
Pr	0.6585	0.0010	0.9288

Canonical coefficients:

	CV1	CV2	CV3
x1	-38.6999	-1.1839	-8.0196
y1	-6.4584	161.4704	-75.2231
z1	15.9027	-35.7584	-53.3071
x2	10.7416	17.9105	72.9448
y2	37.7187	9.7032	-10.2646
z2	-4.5036	5.1352	38.0151
x3	77.4022	148.4498	-13.4958
y3	-11.0921	27.4953	-39.2217
z3	13.0397	59.0796	11.3546
x4	30.6911	-52.5341	-59.5253
y4	12.0111	-75.0454	-9.5677
z4	-4.5073	19.9009	9.4701
x5	3.4582	-41.1281	-36.7964
y5	-23.6135	-53.5754	-54.4568
z5	-16.1548	13.8098	0.3529
x6	9.0317	-77.4997	15.0143
y6	-14.5124	-50.0908	-10.1933
z6	-3.3671	-5.3181	3.5726
x7	7.6543	-52.5748	51.2303
y7	16.9529	-56.6653	86.9409
z7	9.7695	-38.8240	-28.1175
x8	5.0217	-11.3564	-17.7710
y8	-108.9224	47.6082	-33.8194
z8	-3.0942	4.6955	5.6726
x9	14.2172	36.7351	-48.4368
y9	-13.3809	38.8168	-7.2609
z9	-23.4881	-15.8529	0.2007
x10	27.1735	81.0200	-22.0390
y10	18.4825	39.7221	-9.5172
z10	-30.5986	-15.9626	2.5165
x11	9.7462	-46.9226	-8.7930
y11	19.1321	-28.3893	2.3290
z11	-33.6801	-10.5743	-0.6039
x12	36.9358	-19.3324	56.1486
y12	24.4685	-16.3644	-3.1455
z12	-10.6348	12.9920	17.3555
x13	3.5298	25.9208	21.5713
y13	29.9854	-27.4213	-16.7067
z13	5.2069	54.5914	-1.6689

x14	-49.5013	22.3968	-12.1913
y14	52.2877	-64.3012	22.4540
z14	31.5091	96.3471	-14.7813
x15	13.8670	7.6497	-13.0307
y15	-14.0592	8.6244	-85.0010
z15	2.1860	-53.6259	28.3796
x16	38.1774	-62.9645	72.7373
y16	-23.7762	1.2402	-55.2924
z16	-13.7199	-0.5757	-27.1981
x17	11.1762	-73.4408	23.3467
y17	-5.0289	-48.3191	19.2607
z17	-21.1746	21.1736	-25.5849
x18	19.0171	7.3828	-35.7805
y18	1.1973	-40.6546	35.5527
z18	-21.8056	34.5831	-7.9927
x19	15.1672	95.2342	-26.0396
y19	5.6749	110.3013	3.8335
z19	-15.2204	9.2766	1.1285
x20	3.7497	24.4471	-1.7582
y20	9.5585	99.5981	-9.8177
z20	-5.4904	-4.2483	-6.1196
x21	-5.5349	-32.3907	39.9257
y21	35.1748	24.1974	12.0567
z21	-1.0059	-2.0248	-19.5745
x22	-3.5336	-9.3865	53.2849
y22	-6.4421	50.6023	-7.8603
z22	-21.9448	-17.8504	15.5751
x23	17.1852	-60.6946	-60.2756
y23	35.5886	-9.6065	39.3422
z23	2.0177	-14.6174	-4.7612
x24	-31.4062	20.4639	-63.7923
y24	13.0931	25.9920	78.1524
z24	-1.9868	-18.3616	-25.4689
x25	-19.9585	86.4829	-73.5422
y25	11.0017	23.8443	12.3423
z25	13.0972	-84.0046	13.9157
x26	19.9668	93.8547	-48.9201
y26	-7.0671	-7.6419	-16.5615
z26	20.1041	-72.7198	11.1408
x27	31.8318	25.1924	-8.4395
y27	-41.1164	-72.0542	-24.7697
z27	20.1292	-28.4375	-0.2033
x28	4.2056	-127.7739	138.9408
y28	-46.6400	-107.6518	-45.5717
z28	18.5580	36.4382	-41.1207
x29	-77.4917	7.2445	60.4881
y29	49.5718	-137.1097	156.4749
z29	30.1418	-81.6846	3.3070
x30	-0.1305	-16.2111	-28.6068
y30	-27.8658	-12.5908	-29.7934
z30	25.3438	54.9653	-8.8180
x31	-46.9350	41.5444	-118.2233
y31	-18.8173	-82.9689	10.1856
z31	11.0702	18.4920	69.4097
x32	9.3426	-46.8041	101.7120
y32	0.5266	-98.1744	208.6294
z32	-63.5528	76.6599	3.7805
x33	-39.6993	56.3824	-42.3962
y33	-28.3150	28.2311	-3.8019
z33	17.9294	5.5298	-18.0757

x34	-62.9990	-45.5892	15.9300
y34	90.1057	52.9875	-14.6289
z34	35.3629	-1.1638	-48.8020
x35	-40.2289	-75.1253	24.3217
y35	19.3567	94.3256	39.4940
z35	14.6167	34.0733	31.5833
x36	-3.1709	54.6010	0.2766
y36	-84.7808	143.8648	-164.5731
z36	9.9449	-56.1386	65.4676

New Procrustes fit:
Dataset 'first turn of the cochlea'

51 landmarks in 3 dimensions.
The dataset contains 40 observations, of which 36 are included for analyses.

Average shape:

Lmk.	Axis 1 (x)	Axis 2 (y)	Axis 3 (z)
1	-0.13512095	-0.09348533	0.00665219
2	-0.13872073	-0.08263326	0.00948012
3	-0.14206461	-0.07090141	0.01103930
4	-0.14502214	-0.05849464	0.01151261
5	-0.14758687	-0.04570653	0.01057155
6	-0.14903618	-0.03291715	0.00918801
7	-0.14974274	-0.02013277	0.00769776
8	-0.14911315	-0.00729983	0.00598446
9	-0.14748339	0.00546977	0.00375619
10	-0.14462276	0.01832549	0.00135589
11	-0.14071444	0.03125874	-0.00145489
12	-0.13553640	0.04421754	-0.00457447
13	-0.12880989	0.05693866	-0.00803475
14	-0.11660736	0.07492317	-0.01240032
15	-0.10245789	0.09055790	-0.01560625
16	-0.08678738	0.10388606	-0.01747387
17	-0.06987958	0.11523169	-0.01804284
18	-0.05169779	0.12487295	-0.01735470
19	-0.03187904	0.13239014	-0.01535616
20	-0.01040913	0.13777521	-0.01198563
21	0.01280243	0.14053070	-0.00732420
22	0.03746134	0.13977713	-0.00146259
23	0.06535621	0.13437757	0.00517337
24	0.08995468	0.12463847	0.01044197
25	0.11042135	0.11175149	0.01404142
26	0.12702742	0.09706183	0.01618380
27	0.14024169	0.08101447	0.01708565
28	0.15068405	0.06371069	0.01676834
29	0.15861400	0.04489412	0.01525119
30	0.16352935	0.02411216	0.01240544
31	0.16458428	0.00134753	0.00818556
32	0.16250928	-0.01646894	0.00392857
33	0.15790084	-0.03268432	-0.00008092
34	0.15125907	-0.04719683	-0.00369196
35	0.14282367	-0.05999652	-0.00692331
36	0.13293194	-0.07139946	-0.00979890
37	0.12124959	-0.08124194	-0.01227852
38	0.10787898	-0.08970566	-0.01441050
39	0.09260921	-0.09649433	-0.01619210

40	0.07549882	-0.10120038	-0.01750328
41	0.05921762	-0.10386415	-0.01661736
42	0.04372291	-0.10445454	-0.01488965
43	0.02919061	-0.10307179	-0.01244474
44	0.01562983	-0.10006946	-0.00941033
45	0.00307575	-0.09536728	-0.00575138
46	-0.00873255	-0.08922527	-0.00146335
47	-0.01986180	-0.08149235	0.00352276
48	-0.02995306	-0.07181613	0.00925083
49	-0.03869152	-0.06010309	0.01559786
50	-0.04563242	-0.04737672	0.02117524
51	-0.05001113	-0.03426340	0.02627687

Procrustes sums of squares: 0.4837676021435645
Tangent sums of squares: 0.46820256619629613

Data matrices in this dataset:
- first turn of the cochlea, raw data
- first turn of the cochlea, centroid size
- first turn of the cochlea, Procrustes coordinates

New Procrustes fit:
Dataset 'first turn of the cochlea'

51 landmarks in 3 dimensions.
The dataset contains 40 observations, of which 36 are included for analyses.

Average shape:

Lmk.	Axis 1 (x)	Axis 2 (y)	Axis 3 (z)
1	-0.13512095	-0.09348533	0.00665219
2	-0.13872073	-0.08263326	0.00948012
3	-0.14206461	-0.07090141	0.01103930
4	-0.14502214	-0.05849464	0.01151261
5	-0.14758687	-0.04570653	0.01057155
6	-0.14903618	-0.03291715	0.00918801
7	-0.14974274	-0.02013277	0.00769776
8	-0.14911315	-0.00729983	0.00598446
9	-0.14748339	0.00546977	0.00375619
10	-0.14462276	0.01832549	0.00135589
11	-0.14071444	0.03125874	-0.00145489
12	-0.13553640	0.04421754	-0.00457447
13	-0.12880989	0.05693866	-0.00803475
14	-0.11660736	0.07492317	-0.01240032
15	-0.10245789	0.09055790	-0.01560625
16	-0.08678738	0.10388606	-0.01747387
17	-0.06987958	0.11523169	-0.01804284
18	-0.05169779	0.12487295	-0.01735470
19	-0.03187904	0.13239014	-0.01535616
20	-0.01040913	0.13777521	-0.01198563
21	0.01280243	0.14053070	-0.00732420
22	0.03746134	0.13977713	-0.00146259
23	0.06535621	0.13437757	0.00517337
24	0.08995468	0.12463847	0.01044197
25	0.11042135	0.11175149	0.01404142
26	0.12702742	0.09706183	0.01618380
27	0.14024169	0.08101447	0.01708565

28	0.15068405	0.06371069	0.01676834
29	0.15861400	0.04489412	0.01525119
30	0.16352935	0.02411216	0.01240544
31	0.16458428	0.00134753	0.00818556
32	0.16250928	-0.01646894	0.00392857
33	0.15790084	-0.03268432	-0.00008092
34	0.15125907	-0.04719683	-0.00369196
35	0.14282367	-0.05999652	-0.00692331
36	0.13293194	-0.07139946	-0.00979890
37	0.12124959	-0.08124194	-0.01227852
38	0.10787898	-0.08970566	-0.01441050
39	0.09260921	-0.09649433	-0.01619210
40	0.07549882	-0.10120038	-0.01750328
41	0.05921762	-0.10386415	-0.01661736
42	0.04372291	-0.10445454	-0.01488965
43	0.02919061	-0.10307179	-0.01244474
44	0.01562983	-0.10006946	-0.00941033
45	0.00307575	-0.09536728	-0.00575138
46	-0.00873255	-0.08922527	-0.00146335
47	-0.01986180	-0.08149235	0.00352276
48	-0.02995306	-0.07181613	0.00925083
49	-0.03869152	-0.06010309	0.01559786
50	-0.04563242	-0.04737672	0.02117524
51	-0.05001113	-0.03426340	0.02627687

Procrustes sums of squares: 0.4837676021435645
Tangent sums of squares: 0.46820256619629613

Data matrices in this dataset:
- first turn of the cochlea, raw data
- first turn of the cochlea, centroid size
- first turn of the cochlea, Procrustes coordinates

Principal Component Analysis: PCA: CovMatrix, first turn of the cochlea, Procrustes coordinates

	Eigenvalues	% Variance	Cumulative %
1.	0.00762976	57.036	57.036
2.	0.00174557	13.049	70.084
3.	0.00122470	9.155	79.239
4.	0.00081830	6.117	85.357
5.	0.00049238	3.681	89.037
6.	0.00026754	2.000	91.037
7.	0.00024236	1.812	92.849
8.	0.00020825	1.557	94.406
9.	0.00018243	1.364	95.769
10.	0.00016446	1.229	96.999
11.	0.00007759	0.580	97.579
12.	0.00006254	0.468	98.046
13.	0.00005708	0.427	98.473
14.	0.00005091	0.381	98.854
15.	0.00004353	0.325	99.179
16.	0.00002349	0.176	99.355
17.	0.00001761	0.132	99.486
18.	0.00001379	0.103	99.589
19.	0.00001164	0.087	99.676
20.	0.00001022	0.076	99.753

21.	0.00000677	0.051	99.803
22.	0.00000605	0.045	99.849
23.	0.00000490	0.037	99.885
24.	0.00000397	0.030	99.915
25.	0.00000269	0.020	99.935
26.	0.00000226	0.017	99.952
27.	0.00000169	0.013	99.965
28.	0.00000127	0.010	99.974
29.	0.00000094	0.007	99.981
30.	0.00000073	0.005	99.987
31.	0.00000056	0.004	99.991
32.	0.00000043	0.003	99.994
33.	0.00000033	0.002	99.997
34.	0.00000028	0.002	99.999
35.	0.00000019	0.001	100.000

Total variance: 0.01337722

Variance of the eigenvalues: 0.0000004294497
Eigenvalue variance scaled by total variance: 0.00240
Eigenvalue variance scaled by total variance and number of variables:
0.35279
Note: throughout all calculations of eigenvalue variances, the dimensionality used was 146.
Please double-check because this dimensionality may not be appropriate for all situations.
Warning: There appear to be fewer PCs than dimensions in shape space.
This can have the following reasons, among others:
-- the degrees of freedom are less than the shape dimensionality (sample size, etc.).
-- there is complete integration in the data.
Interpret the results cautiously and check for reasons why this is the case.

Principal Component Coefficients

```
       PC1         PC2         PC3         PC4         PC5         PC6
PC7          PC8         PC9         PC10        PC11        PC12
PC13         PC14        PC15        PC16        PC17        PC18
PC19         PC20        PC21        PC22        PC23        PC24
PC25         PC26        PC27        PC28        PC29        PC30
PC31         PC32        PC33        PC34        PC35
   x1  0.080285  -0.047255  -0.027117  -0.221019  -0.095101   0.006086
     -0.016455   0.140261  -0.180742   0.215237   0.062528   0.049070
     -0.054770  -0.120946   0.086153  -0.160634   0.058737  -0.062968
     -0.032574   0.089942  -0.087851   0.127280   0.172166  -0.221008
0.040369   -0.001203   0.081216   0.152215  -0.004297   0.019944  -
0.166536   -0.012070  -0.011655  -0.066644   0.234396
   y1 -0.227267  -0.135292  -0.041699   0.145060   0.093096   0.068409
     -0.171382  -0.178779  -0.015529   0.062485   0.094566  -0.165654
     -0.116386  -0.042324  -0.322559  -0.114236  -0.109175  -0.006486
0.233383   -0.201308  -0.061651  -0.120640  -0.112259  -0.124029  -
0.144604   -0.036466   0.040399   0.093012  -0.058913  -0.068514  -
0.072379    0.046676  -0.061852  -0.028205   0.155917
   z1 -0.128042   0.087349   0.077111  -0.131449  -0.037832  -0.034475
0.083812   -0.281682  -0.114246   0.096523  -0.164855  -0.025127
0.026670    0.205277   0.020452  -0.089865  -0.115035  -0.053282
0.020865   -0.088010  -0.269509   0.122277   0.026346  -0.209946
0.152289   -0.125665  -0.089726  -0.192007   0.018106  -0.119740
0.038663   -0.055175   0.062913  -0.033382  -0.124783
```

x2 0.075002 -0.052667 -0.035399 -0.202071 -0.066855 0.014245
-0.020287 0.118653 -0.143960 0.190722 0.072319 0.012109
-0.052716 -0.115966 0.046557 -0.134141 0.014792 -0.094566
0.009524 0.062724 0.042660 0.054684 0.065882 -0.107784 -
0.024369 0.019289 0.074024 -0.006549 0.044175 -0.051652
0.121621 -0.131068 -0.174512 -0.084886 -0.078182
y2 -0.224100 -0.144365 -0.057871 0.148192 0.086396 0.032345
-0.120845 -0.123321 -0.046043 0.051093 0.074715 -0.075666
-0.069141 -0.046848 -0.157300 -0.079727 -0.018926 -0.048322
0.156626 -0.087710 -0.031854 -0.003005 -0.084748 -0.026688 -
0.092815 -0.074127 0.043819 -0.019200 0.013823 -0.016048 -
0.027642 -0.079316 -0.107820 -0.072092 0.031415
z2 -0.100598 0.064925 0.076783 -0.132319 -0.017242 -0.047681
0.081202 -0.234738 -0.097866 0.081700 -0.144008 0.020715
0.037271 0.145664 0.071520 -0.059917 -0.042745 -0.045020
0.050598 -0.060478 -0.169994 0.096020 0.029017 -0.074116
0.050461 -0.074524 -0.042716 -0.047722 -0.028563 0.101074
0.072590 -0.071800 0.042590 0.011017 0.014736
x3 0.067279 -0.051625 -0.049923 -0.177778 -0.041725 0.026335
-0.025610 0.085810 -0.129318 0.177447 0.044150 -0.028609
-0.069271 -0.101005 -0.056289 -0.102426 -0.009941 -0.077612
0.035563 -0.014452 0.053782 0.081354 -0.105941 0.049966 -
0.007630 0.017655 0.136517 0.063007 -0.090875 0.155441
0.027489 -0.100888 0.047743 0.014989 -0.126402
y3 -0.218416 -0.150032 -0.067602 0.154267 0.083569 0.005444
-0.090283 -0.072902 -0.077555 0.054716 0.032759 0.005535
-0.009682 -0.039585 0.019494 -0.029610 0.149280 -0.087174
0.043986 0.000543 -0.009067 0.112822 -0.003862 0.051322 -
0.010514 -0.055164 0.023556 -0.026855 -0.000907 0.091681
0.051170 -0.178413 0.003013 0.003483 -0.103135
z3 -0.073281 0.043730 0.075020 -0.126394 -0.001468 -0.058766
0.075568 -0.190219 -0.070709 0.057545 -0.123375 0.052738
0.040440 0.014809 0.106133 -0.045074 -0.008912 0.006089
0.025354 -0.003058 -0.019560 0.010110 0.025599 0.049229 -
0.084649 -0.000912 -0.011507 0.012431 0.010260 0.019458 -
0.012968 0.192337 -0.054466 0.024816 0.156645
x4 0.057311 -0.054666 -0.063019 -0.150829 -0.021913 0.048776
-0.041022 0.055447 -0.100311 0.138230 0.041332 -0.055595
-0.089101 -0.110816 -0.063630 -0.033763 -0.040032 -0.098998
0.030245 -0.021057 0.091396 -0.028101 -0.236976 0.135775 -
0.071462 -0.060003 -0.042694 -0.054232 -0.127004 0.103525 -
0.074119 0.042348 0.213299 0.094837 -0.165751
y4 -0.210958 -0.154214 -0.072331 0.166531 0.076309 -0.018002
-0.074875 -0.022718 -0.101020 0.053839 -0.003901 0.095395
0.049201 -0.075003 0.254179 0.028317 0.349079 -0.109199 -
0.123469 0.101187 0.039104 0.162866 0.168627 0.085100
0.050095 0.033108 -0.048860 -0.041504 0.016503 -0.015005
0.033007 0.034303 0.080495 0.019102 -0.032825
z4 -0.044688 0.021971 0.076986 -0.116453 0.022576 -0.067683
0.055976 -0.151385 -0.045611 0.050876 -0.098801 0.047088
0.038851 -0.070264 0.128006 -0.024590 0.000166 0.028280
0.058308 -0.000920 0.127296 -0.057358 0.001648 0.211823 -
0.215222 0.127590 0.119569 0.163180 -0.037903 0.025542 -
0.012161 0.202699 -0.151427 0.014750 -0.025116
x5 0.042298 -0.063344 -0.073088 -0.112372 0.002483 0.054238
-0.059067 0.045086 -0.089687 0.114594 0.018124 -0.079144
-0.032748 -0.036876 0.023465 0.039256 -0.065905 -0.078132
-0.009047 0.044351 0.039846 -0.058983 -0.121349 0.082741
-0.031091 0.120884 -0.123185 -0.210524 -0.083775 -0.248759
0.173724 0.052158 -0.119706 -0.044848 -0.004581

y5 -0.195750 -0.107550 -0.069584 0.103151 0.036942 -0.025287
0.006860 0.027465 -0.082185 0.056805 -0.032187 0.076004
0.034979 -0.064334 0.189140 0.046752 0.119734 -0.012271 -
0.131176 0.095639 0.039843 0.045920 0.108640 0.010414
0.020984 0.055280 -0.009235 -0.013618 -0.004670 -0.053137
0.019517 0.081693 -0.009845 -0.028708 0.049579
z5 -0.021918 -0.007625 0.090801 -0.105444 0.056920 -0.066716
0.040986 -0.096077 -0.016515 0.018264 -0.029772 0.015216
0.022404 -0.079623 0.053087 0.004148 0.038440 -0.025507
0.033915 -0.034753 0.143147 -0.132867 0.003654 0.117410 -
0.127704 0.017048 0.073094 0.141038 -0.045190 0.074130 -
0.046314 0.024068 0.002719 0.049743 -0.005833
x6 0.031481 -0.064889 -0.082879 -0.086162 0.028224 0.047934
-0.059073 0.050848 -0.068686 0.093628 0.001681 -0.052876
0.010409 -0.004983 0.029848 0.084168 -0.016463 -0.001678
0.017474 -0.018218 -0.083237 -0.089246 -0.048691 -0.016210 -
0.050379 0.000149 -0.270062 -0.093711 0.107726 -0.147530
0.083034 0.339527 -0.090687 -0.082527 -0.018517
y6 -0.178153 -0.065417 -0.065723 0.043210 0.004820 -0.031492
0.068407 0.074062 -0.061140 0.049231 -0.056405 0.058956
0.029561 -0.051735 0.111607 0.060493 -0.050494 0.047951 -
0.139040 0.078678 0.039574 -0.023319 0.022708 -0.016364
0.019375 0.029149 -0.003493 0.001463 -0.024703 0.005531
0.009904 0.022212 0.043004 0.026653 -0.026877
z6 -0.002202 -0.035673 0.095160 -0.090068 0.084041 -0.067485
0.028787 -0.042734 -0.004063 -0.013225 0.013468 -0.029124
0.022361 -0.073572 -0.002273 0.032338 0.038643 -0.055601 -
0.023426 0.002496 0.160852 -0.103176 -0.010669 0.123990 -
0.026169 0.031357 0.115455 0.035926 -0.061101 0.044689 -
0.045395 -0.176230 0.080312 -0.064873 0.027898
x7 0.018563 -0.055523 -0.090861 -0.063895 0.043944 0.034581
-0.070536 0.048399 -0.055581 0.055240 -0.009498 -0.039343
0.054954 0.026757 0.032421 0.109062 -0.031554 0.048680
0.013999 -0.073256 -0.109364 -0.045907 0.095283 0.032950
0.054592 -0.014212 -0.087451 0.028090 0.117901 -0.011175 -
0.037919 0.085122 0.028512 0.066756 0.202581
y7 -0.161838 -0.025871 -0.059271 -0.009985 -0.020828 -0.034577
0.116480 0.108557 -0.038437 0.039797 -0.069487 0.042634
0.023470 -0.034211 0.043966 0.067373 -0.161280 0.083129 -
0.135175 0.065780 0.046035 -0.074822 -0.040349 -0.043104
0.029847 0.027975 -0.005492 0.019940 -0.030105 0.019290
0.004298 -0.024710 0.002449 0.002449 -0.051595
z7 0.013066 -0.059689 0.099635 -0.075390 0.108271 -0.060034
0.020760 0.001897 0.016753 -0.032957 0.046555 -0.057891
0.015624 -0.064554 -0.040013 0.063160 0.064093 -0.057409 -
0.050931 0.023614 0.138304 -0.126800 -0.014727 0.037150
0.021287 0.000431 -0.010243 -0.046546 -0.004011 -0.002242 -
0.011351 -0.178634 0.056776 -0.062111 -0.032685
x8 0.007326 -0.038769 -0.102388 -0.052434 0.057176 0.022946
-0.086936 0.044431 -0.039809 0.038976 -0.057332 -0.029483
0.099071 0.074146 0.009779 0.106541 -0.012355 0.101683 -
0.036191 -0.106597 -0.058718 0.093010 0.032561 0.146068
0.055012 -0.105792 0.010252 -0.032316 0.263190 0.071525 -
0.068591 -0.109301 0.133444 0.248705 -0.080319
y8 -0.144864 0.010795 -0.051124 -0.057536 -0.041102 -0.033602
0.150071 0.131485 -0.017865 0.033554 -0.067693 0.023479
0.018555 -0.017702 -0.019502 0.058354 -0.201814 0.106991 -
0.124245 0.040303 0.037999 -0.085985 -0.100270 -0.040676
0.013286 0.004406 -0.036202 0.056683 0.022889 0.035664 -
0.050757 0.016867 0.014539 0.015740 -0.044246

z8 0.025557 -0.077999 0.100586 -0.055348 0.128007 -0.050009
-0.000226 0.038093 0.026333 -0.042010 0.073273 -0.068440
0.023159 -0.071533 -0.074350 0.071296 0.092914 -0.011842 -
0.069624 0.002847 0.095111 -0.106961 -0.054644 0.043590
0.049803 -0.045253 -0.144986 0.007462 0.174898 0.009686 -
0.130348 -0.050350 0.105018 0.035837 0.053458
x9 -0.003727 -0.019022 -0.109389 -0.046904 0.060998 0.022410
-0.068276 0.029647 0.000097 -0.009130 -0.080397 -0.038349
0.090876 0.119955 0.018606 0.142519 -0.041540 0.061669
0.025127 -0.079616 -0.047121 -0.034250 0.055924 0.052998
0.081008 0.071286 0.198043 0.011925 -0.054322 0.024321
0.020555 -0.121099 0.154784 0.057058 0.114962
y9 -0.127650 0.044005 -0.038666 -0.098146 -0.057721 -0.029672
0.169948 0.142935 0.004528 0.021572 -0.040031 -0.001091
0.009015 -0.001764 -0.053772 0.035085 -0.213194 0.100000 -
0.088688 0.041293 0.032128 -0.098117 -0.081014 -0.077077
0.007754 0.005064 -0.014730 0.034061 0.004648 0.024105
0.007336 0.000517 -0.050365 -0.018416 -0.016359
z9 0.031551 -0.092643 0.098422 -0.036352 0.134520 -0.040320
-0.007500 0.071377 0.042620 -0.051966 0.103804 -0.081530
0.007873 -0.064007 -0.049520 0.074371 0.053331 -0.001073 -
0.049825 0.033675 0.053456 -0.117482 0.018386 -0.076689
0.056082 -0.035164 -0.124909 -0.077145 0.069931 -0.079605
0.001325 -0.077012 -0.000686 0.015104 0.007455
x10 -0.016308 0.010420 -0.114883 -0.044856 0.063463
0.028598 -0.065060 0.013699 0.043571 -0.032390 -0.093435 -
0.055144 0.081079 0.153191 0.033614 0.134831 -0.044792
0.110712 0.017385 -0.014524 -0.067491 -0.114628 0.071763
0.059286 0.165787 0.093933 0.022420 0.047366 -0.142794 -
0.041802 -0.233204 -0.172479 -0.046907 -0.111515 0.016516
y10 -0.108529 0.072933 -0.023793 -0.132925 -0.069428 -
0.027518 0.172391 0.143835 0.023529 0.001283 0.002376 -
0.024886 0.000771 0.006179 -0.083590 0.005624 -0.162184
0.050400 -0.048816 0.022400 0.034032 -0.049047 -0.074311 -
0.056509 -0.029247 -0.007652 0.037680 -0.005455 0.032976
0.042158 0.084193 0.025546 0.015486 0.025998 -0.001688
z10 0.031831 -0.098383 0.093764 -0.015462 0.134592 -
0.018392 -0.029380 0.087855 0.050194 -0.055341 0.109139 -
0.085905 0.000701 -0.053706 -0.064386 0.056428 0.036737
0.020852 -0.040048 0.021856 0.049510 -0.058621 0.041080 -
0.141834 0.065110 0.003427 -0.031823 -0.120178 0.029456 -
0.124028 0.153958 -0.040217 -0.085410 0.059217 0.089688
x11 -0.024677 0.041120 -0.114155 -0.050412 0.060616
0.011353 -0.046348 0.006393 0.087006 -0.058154 -0.077958 -
0.042663 0.086713 0.142343 0.029955 0.060528 -0.005898
0.101767 0.048784 0.005368 0.017082 -0.113310 0.086694 -
0.031561 0.123112 -0.027529 0.008396 0.038175 -0.010365
0.058373 -0.032673 -0.000558 -0.144789 0.003206 0.070806
y11 -0.089903 0.099991 -0.007982 -0.161929 -0.074189 -
0.013034 0.154874 0.127921 0.041007 -0.025753 0.047322 -
0.061541 -0.009090 0.031068 -0.108272 -0.016345 -0.058919 -
0.001724 -0.011454 0.018037 0.003742 0.014412 -0.046028
0.013661 -0.005999 0.052066 0.050717 -0.038862 0.008434 -
0.008473 0.037901 -0.003684 0.080863 0.017906 0.019164
z11 0.031780 -0.104450 0.079494 0.007224 0.127435 -
0.004259 -0.042754 0.113530 0.051561 -0.056380 0.098494 -
0.074680 -0.013612 -0.024235 -0.044726 0.058836 0.008050
0.067563 -0.021145 0.041081 -0.022285 0.027639 0.009430 -
0.090624 0.057093 0.013108 -0.034678 -0.049842 -0.041737 -
0.059044 0.018457 -0.011269 -0.080835 0.032002 -0.006315

x12	-0.031137	0.076123	-0.114006	-0.058754	0.056045	-
0.002593	-0.041730	-0.015580	0.120081	-0.089372	-0.065389	-
0.066711	0.073019	0.142458	0.051868	-0.016181	0.033703	
0.071079	0.074432	-0.013230	0.105495	-0.125927	0.068514	-
0.022677	-0.022316	-0.102036	-0.033443	-0.027586	0.042342	
0.005588	-0.049835	0.149853	-0.025238	0.144075	0.003821	
y12	-0.071090	0.124679	0.012343	-0.185032	-0.076591	
0.003653	0.126890	0.101289	0.063987	-0.052029	0.112504	-
0.086415	-0.025240	0.046440	-0.123021	-0.049626	0.108229	-
0.070687	0.046485	0.023408	-0.032519	0.099878	0.006891	
0.055009	0.052210	0.096865	0.029096	0.002911	-0.002533	-
0.049592	-0.000811	-0.012180	0.023587	-0.041854	0.016568	
z12	0.025632	-0.103018	0.063345	0.028020	0.116705	
0.016656	-0.053878	0.137476	0.054239	-0.046215	0.073316	-
0.051827	-0.013289	0.007065	-0.044084	0.040113	-0.013949	
0.133931	0.008380	0.035918	-0.097241	0.151317	-0.003920	-
0.058162	0.059265	0.027598	0.039217	0.036497	-0.054933	
0.021523	0.004650	0.066705	-0.040969	-0.021094	-0.062379	
x13	-0.034854	0.118652	-0.103536	-0.075209	0.049488	-
0.008578	-0.043106	-0.048113	0.158682	-0.137397	-0.008605	-
0.081866	0.034234	0.139649	0.060772	-0.060630	0.122464	-
0.040054	0.122650	-0.050337	0.192575	-0.056182	0.077701	
0.008541	-0.044422	-0.074237	0.016913	-0.003816	-0.046266	-
0.022212	0.002197	0.029200	0.050794	-0.024585	-0.175742	
y13	-0.052352	0.144135	0.029985	-0.200544	-0.073841	
0.019724	0.091312	0.067997	0.086808	-0.071448	0.169616	-
0.118249	-0.035801	0.069793	-0.111773	-0.118309	0.297763	-
0.112842	0.115059	0.062911	-0.066301	0.135068	0.069204	
0.082370	0.059511	0.080496	-0.070348	-0.002670	0.038193	-
0.073377	-0.103784	0.117830	-0.038006	0.045154	0.132887	
z13	0.013574	-0.096272	0.042538	0.050986	0.102527	
0.043733	-0.066155	0.153942	0.056973	-0.024385	0.034859	-
0.031929	-0.009271	0.056188	-0.018151	0.008320	-0.045159	
0.228609	0.043657	0.036531	-0.189047	0.277952	0.001092	-
0.067612	0.022008	0.037127	0.128797	0.083741	-0.095365	
0.082834	0.074871	0.136564	-0.026232	0.053011	-0.124217	
x14	-0.024330	0.151362	-0.059967	-0.065126	0.061825	-
0.023066	-0.054021	-0.053068	0.137861	-0.168289	-0.048068	-
0.040516	0.014197	0.068772	-0.007115	-0.020609	0.099732	-
0.075086	0.084651	0.023028	0.167269	0.029196	0.020953	-
0.013880	0.073006	0.070082	0.003533	0.002192	-0.083453	
0.000379	0.182568	0.044151	0.022495	-0.167494	-0.059223	
y14	-0.009377	0.128054	0.076374	-0.145457	-0.057638	
0.045164	-0.012400	0.064058	0.038621	-0.006695	0.161795	-
0.026659	-0.064520	0.004920	-0.007330	-0.047720	0.148350	-
0.031160	0.062496	-0.051405	-0.113040	0.053560	0.084846	
0.062664	0.003604	-0.048410	0.020568	-0.010003	0.087471	
0.040289	-0.053654	-0.011593	0.003211	0.090958	-0.050124	
z14	-0.000179	-0.055240	0.006704	0.060393	0.054129	
0.081261	-0.063193	0.134120	0.081298	-0.004942	-0.018365	
0.041246	-0.063245	0.053846	0.011227	-0.061011	-0.071161	
0.093183	-0.004185	0.002243	-0.109992	0.126631	0.017134	-
0.012475	-0.031050	0.019618	0.047327	0.060763	-0.015925	
0.054307	-0.050554	0.192298	0.091954	-0.085384	-0.149819	
x15	-0.006078	0.174907	-0.007334	-0.044699	0.074298	-
0.047089	-0.081064	-0.045052	0.108030	-0.153829	-0.012007	
0.012133	-0.042348	-0.031401	0.030466	-0.040376	0.016403	-
0.037643	-0.007062	0.002143	0.079736	0.128138	0.023026	-
0.063022	-0.056215	-0.031502	-0.006680	0.011457	0.083055	
0.064471	0.120623	0.104355	-0.004015	-0.065730	-0.079001	

y15	0.023054	0.108050	0.104440	-0.097537	-0.046856	
0.074128	-0.097760	0.034840	0.000208	0.027783	0.119424	
0.018998	-0.066707	-0.016933	0.052257	0.044383	0.063933	-
0.006423	0.071317	-0.081474	-0.111997	-0.078224	0.041590	
0.082353	0.048370	-0.017999	0.001720	0.028419	-0.065359	-
0.010734	-0.075988	-0.140145	-0.033691	0.060792	-0.074242	
z15	-0.011838	-0.019983	-0.025484	0.062680	0.015890	
0.107716	-0.046344	0.114011	0.091868	0.002328	-0.074628	
0.102536	-0.075293	0.080011	-0.003865	-0.114514	-0.055865	-
0.033027	-0.041118	0.000920	-0.027910	0.052803	0.000190	
0.041564	-0.014251	0.009827	0.044858	0.069999	-0.033418	
0.067867	-0.040240	0.096907	0.084764	-0.140916	-0.011119	
x16	0.016665	0.189318	0.043503	-0.016286	0.087469	-
0.053736	-0.107430	-0.056539	0.059943	-0.144435	-0.010577	
0.038340	-0.068938	-0.094636	0.055325	-0.010387	-0.033295	-
0.019858	-0.038058	0.015685	0.027610	0.108617	-0.085606	-
0.046466	-0.111924	0.011338	-0.052694	0.106960	-0.041974	
0.041636	0.005115	-0.087914	-0.000751	0.013344	-0.007416	
y16	0.044732	0.083845	0.115747	-0.066487	-0.044453	
0.087353	-0.148598	0.029141	-0.016100	0.056138	0.098967	
0.062765	-0.068856	-0.033826	0.096473	0.087772	-0.021010	
0.013717	0.043583	-0.115675	-0.120088	-0.108938	0.127679	
0.064503	0.075454	-0.073676	0.010405	-0.058623	0.040884	-
0.007002	-0.016990	0.035374	-0.061358	-0.031222	-0.039492	
z16	-0.020253	0.011345	-0.049065	0.063080	-0.016022	
0.123506	-0.032276	0.093278	0.098777	0.013550	-0.102534	
0.137215	-0.077856	0.078774	0.000313	-0.152145	-0.045822	-
0.090304	-0.054203	-0.031006	0.013486	0.008922	-0.007130	
0.039096	-0.015532	-0.006818	0.023967	0.008936	0.034194	
0.011063	0.015146	0.048642	0.084018	-0.044535	0.079828	
x17	0.037782	0.194777	0.090735	0.010322	0.094615	-
0.057935	-0.113686	-0.041476	0.021838	-0.120294	-0.001416	
0.061703	-0.054266	-0.159276	0.049396	-0.004472	-0.095930	
0.013494	-0.070282	0.021331	-0.043406	0.121885	-0.043520	-
0.064473	-0.153797	-0.014374	-0.035653	-0.025573	-0.010975	
0.024680	-0.113644	-0.040990	0.027475	-0.000337	0.153894	
y17	0.060374	0.057004	0.108965	-0.041626	-0.042548	
0.102758	-0.182020	-0.000440	-0.033933	0.068788	0.061165	
0.079316	-0.082224	0.004046	0.136423	0.114012	-0.039750	-
0.001887	0.032756	-0.118268	-0.053968	-0.161006	0.049006	
0.086857	0.132343	-0.068279	-0.000780	0.090266	-0.044977	-
0.024757	0.154913	-0.024622	-0.059758	-0.026180	-0.181048	
z17	-0.024783	0.036340	-0.063229	0.062897	-0.045912	
0.130164	-0.016496	0.062352	0.089808	0.019070	-0.120043	
0.150970	-0.066948	0.077426	-0.002105	-0.148545	-0.025966	-
0.141040	-0.040873	-0.022562	0.033709	-0.030640	-0.025461	
0.053084	0.016100	0.003610	-0.003323	-0.000910	0.031989	-
0.020880	-0.029110	-0.063815	-0.002396	0.023284	0.127114	
x18	0.060002	0.189026	0.126631	0.044158	0.098862	-
0.049034	-0.113539	-0.030645	-0.021576	-0.086617	-0.014146	
0.076020	-0.037782	-0.170003	0.052868	-0.009175	-0.107163	
0.017631	-0.077047	0.050298	-0.100498	0.082587	-0.048994	-
0.038040	-0.101206	-0.026006	-0.020932	-0.042895	-0.020171	-
0.028012	-0.072817	-0.088687	-0.013855	-0.032217	-0.046803	
y18	0.067297	0.028056	0.092239	-0.033428	-0.052071	
0.108159	-0.181310	-0.021758	-0.042722	0.068898	0.044510	
0.069283	-0.078465	0.029851	0.127948	0.119419	-0.076696	
0.010237	0.003537	-0.140216	0.052296	-0.151508	0.015373	
0.068866	0.059765	-0.012520	0.001752	0.024954	-0.004892	-
0.013397	0.102948	0.115663	0.074605	-0.000896	0.245693	

z18	-0.025590	0.056995	-0.070449	0.060439	-0.068478	
0.128427	-0.003331	0.034031	0.076721	0.029673	-0.116077	
0.148100	-0.042575	0.049196	0.011124	-0.120677	-0.018533	-
0.150359	-0.008133	-0.011264	0.032956	-0.054371	-0.033170	
0.036800	0.037446	0.011928	-0.044421	-0.018142	0.059115	-
0.049694	0.028083	-0.130204	-0.094243	0.054435	0.003107	
x19	0.082165	0.170329	0.149648	0.081241	0.087180	-
0.025371	-0.084919	-0.020252	-0.070697	-0.047557	-0.020152	
0.077377	-0.013402	-0.135618	0.027650	-0.024746	-0.094646	
0.024550	-0.110906	0.006222	-0.048888	0.012554	-0.059813	-
0.029628	-0.018577	0.040560	0.002311	-0.068576	0.010915	
0.018576	0.057050	-0.009341	-0.017319	0.038760	0.081203	
y19	0.068829	-0.004005	0.068828	-0.033414	-0.050203	
0.095249	-0.183637	-0.033394	-0.035522	0.068385	0.015945	
0.018760	-0.024252	0.041374	0.121265	0.118091	-0.058069	
0.046334	0.060002	-0.015485	0.056957	0.010051	-0.004458	
0.016779	0.014930	-0.092382	-0.016782	0.042918	-0.022649	-
0.099921	-0.025457	-0.013265	0.165514	0.022508	-0.037272	
z19	-0.024357	0.072622	-0.072191	0.056412	-0.086344	
0.114720	0.014455	0.002064	0.057268	0.038922	-0.091033	
0.118254	0.001038	0.008640	0.025574	-0.071546	-0.021473	-
0.137960	0.035696	-0.010835	0.036173	-0.088798	-0.023467	
0.012196	-0.002431	0.015518	-0.066302	-0.071912	0.055219	-
0.016974	-0.007365	-0.110733	-0.095349	0.078270	0.018980	
x20	0.103137	0.138826	0.161231	0.122058	0.074350	
0.000221	-0.037870	-0.002113	-0.106419	0.007267	-0.019080	
0.045899	0.050064	-0.080963	-0.012950	-0.073725	-0.059538	
0.028571	-0.087395	0.009669	-0.026734	0.004459	-0.035161	-
0.012757	0.033881	0.041697	0.019061	-0.098731	0.023406	-
0.043382	0.027411	-0.030655	-0.003188	0.026575	0.056038	
y20	0.066278	-0.037081	0.035445	-0.042821	-0.056952	
0.091171	-0.166160	-0.062608	-0.047029	0.053182	-0.017402	-
0.020464	-0.004885	0.084689	0.102806	0.080781	-0.029561	
0.045812	0.068502	0.068336	0.180174	0.014919	-0.091361	-
0.077670	-0.029256	0.028762	-0.021382	0.055341	-0.058053	
0.063647	-0.061769	0.163217	0.036351	-0.132392	-0.066346	
z20	-0.020311	0.082563	-0.067175	0.048210	-0.099410	
0.091222	0.038929	-0.035120	0.029954	0.046281	-0.041744	
0.060368	0.077320	-0.046205	0.031915	0.012878	-0.014877	-
0.110837	0.101683	0.023013	0.015469	-0.060049	-0.026030	-
0.011886	-0.040059	0.019191	-0.067341	-0.042683	0.010218	
0.014519	-0.022667	-0.093414	-0.057508	0.078444	-0.004959	
x21	0.124736	0.092823	0.160249	0.166793	0.054822	
0.038000	0.030344	0.009013	-0.145225	0.073528	-0.015494	-
0.007466	0.139393	0.015559	-0.067738	-0.130215	0.006766	
0.018205	-0.035121	0.019329	0.039566	-0.021493	-0.027866	-
0.006407	0.104091	0.083508	0.021993	-0.082097	-0.008588	-
0.019203	-0.030230	0.011365	-0.033609	-0.063291	-0.024179	
y21	0.058876	-0.068120	-0.003968	-0.063737	-0.061306	
0.076749	-0.158422	-0.078727	-0.039758	0.010368	-0.043718	-
0.059969	-0.004142	0.135722	0.083954	0.014542	0.012813	
0.070277	0.042768	0.067947	0.258395	0.053048	-0.077015	-
0.206069	-0.059751	0.179372	0.018736	-0.096277	-0.015094	
0.189101	-0.026335	0.020091	0.064118	-0.065204	0.033072	
z21	-0.013725	0.086462	-0.054062	0.038990	-0.107255	
0.052119	0.063062	-0.070111	-0.002157	0.051521	0.032532	-
0.023686	0.176191	-0.119369	0.042712	0.111625	-0.005638	-
0.049495	0.162583	0.057580	-0.009416	-0.012006	-0.008331	-
0.062221	-0.095180	-0.010427	-0.035475	-0.017986	-0.022174	
0.000747	-0.041754	-0.016427	0.030394	0.052947	-0.015211	

x22	0.146618	0.034472	0.147264	0.213634	0.028645	
0.086930	0.114110	0.016306	-0.182275	0.140697	-0.014606	-
0.072410	0.255398	0.136016	-0.138740	-0.178512	0.091720	
0.000644	0.012704	0.016779	0.122875	-0.077688	-0.017629	
0.005233	0.180597	0.133394	0.053506	-0.018813	-0.058208	
0.040539	-0.081415	0.089897	0.030474	-0.052358	-0.056304	
y22	0.045833	-0.086445	-0.041371	-0.088506	-0.056601	
0.061365	-0.160940	-0.079118	-0.007243	-0.068156	-0.077433	-
0.082862	-0.028334	0.124018	0.027415	-0.031842	-0.020890	
0.033310	-0.047645	0.124403	0.192819	0.134540	-0.080692	-
0.163659	-0.033867	0.092089	0.007455	-0.242544	-0.074437	-
0.064855	-0.025594	-0.095305	-0.084431	0.190624	-0.053783	
z22	-0.002857	0.083604	-0.028844	0.030555	-0.107148	-
0.001623	0.076719	-0.101621	-0.046816	0.052406	0.117684	-
0.123179	0.271628	-0.190947	0.066472	0.210149	0.005435	
0.060842	0.203389	0.054196	-0.025420	0.024192	0.046289	-
0.148985	-0.075545	-0.030928	0.028471	0.014317	-0.011898	-
0.075406	-0.032030	-0.000324	0.052606	0.068792	0.037093	
x23	0.135985	-0.029566	0.102826	0.160771	-0.027860	
0.022117	0.067353	0.037644	-0.117612	0.097732	-0.043662	-
0.121778	0.122590	0.115387	-0.061897	-0.152615	0.041340	-
0.004610	0.037900	-0.000635	0.066646	-0.025465	0.035049	-
0.013898	-0.012030	-0.029944	-0.009635	0.045672	0.093463	
0.036161	-0.036161	0.067063	-0.034478	0.090686	-0.072652	
y23	0.029797	-0.080404	-0.078319	-0.105608	-0.035513	
0.034547	-0.161199	-0.060588	0.005903	-0.096040	-0.065489	-
0.082982	0.046051	0.032088	0.000928	-0.116382	-0.063123	
0.035242	-0.081490	0.070958	0.079224	0.150035	-0.065546	-
0.087374	-0.025122	-0.051248	0.050698	-0.063303	0.220039	-
0.067170	-0.147487	-0.045711	-0.035751	-0.006261	0.032593	
z23	-0.003831	0.050826	-0.030797	-0.011765	-0.080573	-
0.028585	0.058892	-0.102587	-0.059442	0.020868	0.038935	-
0.117771	0.176776	-0.138178	-0.020530	0.152750	0.024643	
0.068991	0.080459	0.022571	-0.010328	0.102516	0.028758	-
0.038985	-0.055892	-0.016518	0.007987	-0.034847	0.008633	
0.057008	0.031367	0.014888	0.046131	-0.058539	-0.016604	
x24	0.124253	-0.082735	0.057925	0.107610	-0.068376	-
0.025516	0.036946	0.042135	-0.055304	0.037529	-0.094619	-
0.165966	0.024287	0.093318	-0.003502	-0.066253	-0.020229	-
0.023567	0.007545	0.054704	0.018213	-0.005363	0.039327	
0.064620	-0.075471	-0.113067	-0.048178	0.134601	0.106193	-
0.070834	-0.103610	0.005489	-0.028991	0.120240	0.003189	
y24	0.018281	-0.058930	-0.096198	-0.096341	0.001029	
0.034795	-0.140191	-0.047969	-0.000005	-0.139095	-0.066371	-
0.068032	0.134922	-0.017398	-0.013423	-0.091504	-0.088613	-
0.004691	-0.148030	0.122272	-0.015282	0.061172	-0.028476	
0.106389	0.009625	-0.048508	0.010833	-0.005476	0.098135	-
0.108885	-0.240379	-0.045682	-0.068922	-0.027803	0.068323	
z24	-0.002779	0.019810	-0.026653	-0.046023	-0.050739	-
0.051485	0.029232	-0.106440	-0.078066	-0.011990	-0.033312	-
0.111399	0.072168	-0.068141	-0.063250	0.119419	0.032854	
0.086559	-0.078966	-0.014024	0.006942	0.120691	-0.023537	
0.094531	0.059343	0.030901	-0.058393	-0.013501	-0.005737	
0.057457	0.020575	-0.022631	0.007213	-0.173395	-0.030678	
x25	0.109682	-0.115404	0.019028	0.064766	-0.085494	-
0.060062	0.029045	0.051023	-0.003459	-0.011735	-0.128810	-
0.174880	-0.042585	0.074735	0.012400	0.015877	-0.027025	-
0.045881	0.020077	0.085277	-0.033772	-0.007664	0.076769	
0.077122	-0.108651	-0.062107	-0.095130	0.263877	-0.001190	-
0.050444	0.106911	-0.149955	-0.028592	-0.005063	0.088901	

y25	0.005601	-0.025045	-0.098839	-0.070302	0.045274		
0.031054	-0.103850	-0.019286	-0.014740	-0.137959	-0.024383	-
0.022617	0.189788	-0.063898	-0.036054	-0.122980	-0.050737	-
0.017099	-0.083853	0.088626	-0.071995	-0.034033	0.050600	
0.041858	-0.046914	0.008635	-0.038152	0.204895	-0.036402	-
0.005469	0.099875	-0.106601	-0.115542	-0.109645	0.093687	
z25	-0.002536	-0.003854	-0.016031	-0.065254	-0.022124	-
0.077885	0.006563	-0.100413	-0.093566	-0.020666	-0.057515	-
0.091625	-0.017188	-0.027591	-0.068669	0.030119	0.044355	
0.114800	-0.142270	-0.075243	0.025928	0.083221	-0.018109	
0.078891	0.026299	-0.008674	-0.024674	0.051886	0.012922	
0.013629	0.071837	-0.020698	-0.078772	-0.196011	0.031166	
x26	0.094693	-0.132328	-0.014020	0.038904	-0.086511	-
0.077869	0.020679	0.067420	0.018709	-0.043714	-0.121610	-
0.149480	-0.058099	0.034023	0.021389	0.017869	-0.033706	-
0.088258	0.033930	0.030369	-0.072492	-0.002928	0.136309	
0.055042	-0.183325	-0.080015	-0.038120	0.079053	0.073834	
0.045993	0.252414	-0.107649	-0.016879	0.084216	-0.051259	
y26	-0.002615	0.007904	-0.097275	-0.040848	0.083763	
0.038601	-0.080849	0.015549	-0.049955	-0.133601	0.017994	
0.011610	0.233514	-0.097135	-0.048389	-0.165449	-0.050034	-
0.065167	-0.052589	0.021581	-0.099952	-0.076846	0.100650	
0.038963	-0.058008	-0.008279	0.003802	0.027195	0.028947	
0.102612	0.183429	0.014300	-0.025132	0.083422	-0.109161	
z26	-0.002444	-0.020960	-0.005796	-0.074732	0.004559	-
0.094587	-0.011912	-0.086651	-0.102005	-0.022025	-0.049932	-
0.063516	-0.068113	-0.006576	-0.064577	-0.066895	0.063074	
0.120060	-0.156905	-0.122191	0.037739	0.020588	0.013131	
0.075298	0.010711	-0.097290	0.070942	0.004959	0.022113	
0.012838	0.103705	0.041530	-0.078886	0.003949	-0.003065	
x27	0.080659	-0.130871	-0.046416	0.017182	-0.075339	-
0.081477	0.025332	0.079298	0.025515	-0.066414	-0.127190	-
0.103324	-0.084970	0.013223	-0.008416	0.073705	-0.016909	-
0.076846	0.078982	-0.021477	-0.130939	0.031287	0.098209	
0.077812	-0.079802	0.065687	-0.079789	-0.132848	-0.127857	
0.027047	0.120305	0.042267	0.030859	-0.122323	0.077462	
y27	-0.006845	0.043420	-0.090772	-0.016727	0.115214	
0.047020	-0.048812	0.043104	-0.083156	-0.116894	0.020098	
0.032761	0.211603	-0.087955	-0.074521	-0.119812	-0.040504	-
0.062095	0.005653	-0.008756	-0.110043	-0.074279	0.046024	
0.053024	0.035841	0.072393	-0.068591	-0.052629	-0.115928	
0.025608	0.084620	0.098242	0.067330	0.007000	-0.031598	
z27	-0.001316	-0.032888	0.002189	-0.080208	0.033062	-
0.098594	-0.020247	-0.074617	-0.103078	-0.026660	-0.047031	-
0.036311	-0.109694	0.029448	-0.072270	-0.078977	0.072552	
0.110619	-0.146471	-0.119232	0.012273	-0.030347	0.001782	
0.096727	0.053143	-0.034886	0.070229	-0.004871	-0.078328	-
0.050884	0.021325	0.076796	0.007684	0.054342	-0.010184	
x28	0.064524	-0.121368	-0.071237	0.004277	-0.051290	-
0.087087	0.035598	0.057740	0.008231	-0.072543	-0.131326	-
0.041793	-0.053200	-0.016870	-0.012628	0.072724	0.015606	-
0.096978	0.063247	-0.094894	-0.133748	0.015227	-0.004483	-
0.010515	-0.120506	-0.017577	0.004573	-0.091724	-0.206891	
0.175737	-0.009871	-0.005339	0.059447	-0.051098	0.084535	
y28	-0.009817	0.076837	-0.073122	0.006994	0.142211	
0.052467	-0.009703	0.048811	-0.118073	-0.089085	0.002071	
0.041694	0.182385	-0.057475	-0.081290	-0.068001	-0.021576	-
0.072025	0.012532	-0.036146	-0.080183	-0.066658	0.002959	
0.016853	0.004205	-0.003780	-0.038841	0.047311	-0.133847	
0.074975	0.039518	0.041667	0.106607	0.086834	0.001650	

z28	-0.000788	-0.040263	0.012510	-0.079399	0.062743	-
0.099123	-0.021055	-0.059724	-0.099383	-0.020783	-0.035678	-
0.000716	-0.122141	0.056360	-0.071565	-0.095466	0.078266	
0.076263	-0.122230	-0.077336	-0.004161	-0.073639	0.012729	
0.061087	0.049298	-0.053331	0.074489	0.044866	-0.089467	-
0.038977	-0.005401	0.071373	0.038574	0.145989	0.021827	
x29	0.049300	-0.103664	-0.093468	-0.006943	-0.020659	-
0.086435	0.046042	0.042695	0.006318	-0.079849	-0.107707	
0.035329	-0.015148	-0.080416	-0.008158	0.071445	0.061294	-
0.129998	0.031550	-0.109337	-0.104230	-0.024007	-0.071530	-
0.061708	-0.040773	-0.055437	0.043473	-0.186885	0.081926	
0.093720	-0.043636	0.060113	0.197632	-0.087548	-0.095550	
y29	-0.008668	0.109072	-0.049207	0.030154	0.164364	
0.060213	0.034187	0.064225	-0.138272	-0.055777	-0.017390	
0.037778	0.100547	-0.017514	-0.083586	0.010527	-0.008749	-
0.080052	0.034478	-0.025730	-0.029555	-0.015367	0.005390	
0.014829	0.003989	-0.034244	-0.044790	0.036039	0.006163	
0.013409	0.041087	0.031615	0.130631	0.013839	-0.039640	
z29	-0.000246	-0.044207	0.022520	-0.073052	0.095852	-
0.090530	-0.016813	-0.039857	-0.085721	-0.013560	-0.015914	
0.033452	-0.124971	0.077546	-0.060628	-0.082909	0.045443	
0.022290	-0.088578	0.004074	-0.014246	-0.102911	0.043759	-
0.006649	0.033650	-0.037253	0.016381	0.002878	-0.003825	-
0.050752	-0.017022	0.026252	0.039041	0.054927	-0.006536	
x30	0.036068	-0.075333	-0.108846	-0.013760	0.014692	-
0.072744	0.066954	0.012328	0.005413	-0.079076	-0.095776	
0.087961	-0.020477	-0.111571	-0.011166	0.101162	0.101664	-
0.126509	-0.005971	-0.143925	-0.063388	-0.056123	-0.098165	-
0.054592	0.098244	0.079418	0.072052	-0.114029	-0.040016	-
0.005498	-0.171173	-0.080400	-0.029827	-0.124431	0.114656	
y30	-0.004236	0.143435	-0.014010	0.055657	0.182794	
0.074949	0.084706	0.075005	-0.156295	-0.007030	-0.058399	
0.011977	-0.025159	0.062297	-0.084682	0.119854	0.003708	-
0.064666	0.056689	-0.018101	0.038940	0.079082	0.013819	
0.028939	-0.021399	-0.035157	-0.031457	0.064836	0.007194	-
0.036625	0.046456	-0.029626	-0.036239	-0.041299	0.062654	
z30	0.001471	-0.043188	0.033266	-0.061279	0.128976	-
0.075694	-0.007385	-0.022142	-0.060949	-0.001716	0.001678	
0.068830	-0.122846	0.106634	-0.045173	-0.016236	-0.000554	-
0.040954	-0.025427	0.127316	-0.045115	-0.137718	0.096251	-
0.085083	-0.011120	0.055879	-0.033502	0.001161	0.005931	-
0.063805	-0.033317	-0.023543	0.027363	-0.034673	-0.027182	
x31	0.023139	-0.041119	-0.113732	-0.009110	0.072738	-
0.037372	0.089514	-0.006991	0.014160	-0.055710	-0.080143	
0.121598	-0.041508	-0.103288	0.002445	0.114315	0.099907	-
0.087214	-0.028848	-0.098784	-0.061354	-0.011364	-0.087409	-
0.005997	0.105154	0.078044	0.128810	-0.018921	-0.008752	-
0.068282	-0.216785	0.101673	-0.275713	0.049411	-0.191255	
y31	0.003550	0.179270	0.028840	0.084484	0.199662	
0.092568	0.139768	0.085120	-0.184236	0.051710	-0.110176	-
0.016725	-0.176050	0.160248	-0.080992	0.253349	0.028280	-
0.050074	0.088804	-0.007869	0.143046	0.204084	-0.011160	
0.025159	-0.053665	-0.052714	0.002938	0.043570	0.048416	-
0.023379	0.048632	-0.059594	-0.078710	-0.110786	0.109437	
z31	0.003581	-0.037093	0.046568	-0.039730	0.162497	-
0.051551	0.006812	-0.000995	-0.023469	0.016962	0.030347	
0.101199	-0.104036	0.130535	-0.020569	0.066530	-0.045963	-
0.112484	0.078484	0.275117	-0.088252	-0.166720	0.166305	-
0.158613	-0.118605	0.103690	-0.044872	0.017325	0.072378	-
0.012931	0.007847	-0.045676	0.161636	-0.006812	-0.127659	

x32	0.010541	-0.009902	-0.111601	-0.002720	0.103619	-
0.015084	0.113622	-0.007612	0.003149	-0.021806	-0.028105	
0.109608	-0.062340	-0.096250	0.027341	0.056444	0.064820	-
0.048254	0.081552	-0.096075	0.033308	-0.012177	-0.032878	-
0.172886	0.114021	-0.002859	0.132443	0.059550	-0.016037	-
0.072303	0.017765	0.152400	-0.148457	0.081859	0.009323	
y32	-0.002705	0.144460	0.034636	0.092287	0.120170	
0.056601	0.104379	0.073279	-0.075563	0.060209	-0.088947	-
0.086185	-0.136916	0.090890	0.002986	0.131008	0.052128	
0.020707	0.001818	-0.035192	0.049906	0.083755	-0.003524	
0.028558	-0.122236	-0.030385	0.015197	-0.009097	0.052191	-
0.001201	-0.067468	-0.062615	-0.065708	-0.010546	-0.018521	
z32	-0.000187	-0.052589	0.057324	-0.030313	0.145000	-
0.022023	0.022929	0.024689	0.013159	0.010381	0.049100	
0.042303	-0.036714	0.109216	0.063757	0.029687	-0.039810	-
0.115872	0.082436	0.178749	-0.065545	-0.099368	0.012972	-
0.113123	-0.090705	0.047635	-0.030711	0.010613	0.010094	
0.091239	-0.058514	0.001600	0.018457	0.019746	-0.003688	
x33	-0.002873	0.006240	-0.108764	0.010124	0.115544	
0.003292	0.116884	-0.020673	0.016771	0.019313	0.022256	
0.080315	-0.077239	-0.100379	0.051396	-0.014170	0.022533	
0.024269	0.090142	-0.060312	0.090832	0.032422	-0.010785	-
0.199177	0.032299	-0.077788	0.079031	0.175489	0.064513	-
0.151443	0.060047	0.074340	0.014158	0.078349	0.063980	
y33	-0.006351	0.110079	0.039743	0.094769	0.049467	
0.022596	0.076456	0.058273	0.014218	0.059691	-0.075414	-
0.143918	-0.102197	0.038528	0.065020	0.034735	0.072687	
0.066161	-0.059439	-0.052586	-0.041934	-0.018927	0.005315	
0.056393	-0.111020	0.047654	0.007253	-0.121306	0.000114	
0.047145	-0.091936	-0.066735	-0.045525	0.043832	-0.066469	
z33	-0.003243	-0.065293	0.066343	-0.020076	0.118887	
0.005670	0.034723	0.043199	0.046583	0.004658	0.062942	-
0.004290	0.023287	0.082490	0.127438	-0.011869	-0.032042	-
0.096599	0.077126	0.089094	-0.052681	-0.029617	-0.099830	-
0.043305	-0.061408	0.018402	0.011000	0.005490	-0.030064	
0.134394	-0.083776	0.024730	-0.067359	0.050538	0.013914	
x34	-0.016277	0.009873	-0.106350	0.023217	0.110041	
0.010325	0.108573	-0.042811	0.052228	0.057629	0.041446	
0.027151	-0.074057	-0.084942	0.072085	-0.076525	-0.005707	
0.121290	0.048877	-0.032218	0.097625	0.054015	-0.021325	-
0.071317	0.028668	-0.092715	-0.006953	0.053711	0.082939	-
0.048632	-0.023336	-0.061350	0.195897	0.196529	0.064404	
y34	-0.007951	0.082357	0.047987	0.093297	-0.005107	-
0.011040	0.058010	0.049450	0.073108	0.053375	-0.052865	-
0.168631	-0.078844	-0.011523	0.108662	-0.035887	0.078327	
0.062264	-0.069103	-0.059311	-0.099076	-0.099159	0.031475	
0.015467	-0.098515	0.096268	0.015276	-0.118111	-0.048342	
0.019500	-0.026240	-0.021539	-0.082581	-0.048534	-0.073453	
z34	-0.005436	-0.073038	0.072216	-0.011937	0.088691	
0.031183	0.041671	0.054631	0.073429	-0.002137	0.065131	-
0.034293	0.066112	0.056923	0.158351	-0.032158	-0.027048	-
0.085282	0.063277	0.012698	-0.048996	0.031304	-0.172255	
0.035677	-0.006303	0.010165	0.020840	-0.001047	-0.023001	
0.093685	-0.043530	0.023062	-0.084828	0.050896	0.012514	
x35	-0.033344	0.013196	-0.095333	0.025840	0.095879	
0.017125	0.111276	-0.072845	0.089477	0.090665	0.061923	-
0.022031	-0.094063	-0.070278	0.079717	-0.128488	-0.010953	
0.154913	0.033235	-0.034014	0.025554	0.015351	-0.074156	
0.009341	0.089118	0.024634	-0.150729	0.060776	0.095214	-
0.080186	0.069739	-0.081879	0.073328	0.005991	-0.162744	

y35	-0.006288	0.059734	0.057308	0.095986	-0.042352	-
0.046595	0.028202	0.049924	0.103935	0.047755	-0.035381	-
0.164079	-0.037490	-0.052013	0.131927	-0.080590	0.061354	
0.056286	-0.072887	-0.045706	-0.067598	-0.124273	0.100295	-
0.045103	-0.124886	0.021988	0.092582	-0.120514	-0.076457	
0.045464	-0.058679	0.057286	0.020387	-0.001745	0.134023	
z35	-0.006358	-0.075563	0.073821	-0.006360	0.058864	
0.049624	0.044610	0.063169	0.088721	-0.005482	0.055272	-
0.052031	0.093462	0.035525	0.164203	-0.030363	-0.030224	-
0.070574	0.049654	-0.045062	-0.034708	0.067892	-0.198440	
0.079215	0.044174	-0.005443	-0.009490	0.000754	-0.020016	
0.052370	-0.002621	0.012158	-0.040663	0.013502	0.078989	
x36	-0.050309	0.014416	-0.077425	0.036078	0.078127	
0.012757	0.089238	-0.091151	0.121096	0.119874	0.092553	-
0.043767	-0.082443	-0.059378	0.098178	-0.148547	-0.048852	
0.155594	0.054381	0.042703	0.024998	-0.009998	0.034926	
0.080403	0.062118	0.057656	-0.196415	0.053120	0.019544	
0.004813	-0.009205	-0.083946	0.017979	-0.172330	-0.051696	
y36	-0.003167	0.043030	0.067381	0.088445	-0.068845	-
0.086167	0.009686	0.052970	0.111123	0.032929	-0.041762	-
0.151979	-0.018070	-0.067973	0.104312	-0.089384	0.069575	
0.031492	-0.098550	-0.087233	-0.067661	-0.105876	0.010668	-
0.109421	-0.076604	-0.032755	0.120938	-0.118140	-0.012463	
0.001707	0.018626	0.067307	0.074513	0.141458	0.024805	
z36	-0.006707	-0.072508	0.073703	-0.002088	0.029548	
0.066756	0.044866	0.073204	0.095128	-0.008106	0.038413	-
0.047781	0.107148	0.007903	0.141131	-0.021766	-0.044257	-
0.059181	0.032015	-0.106797	-0.001599	0.090394	-0.172517	
0.084598	0.076351	-0.038575	0.024695	-0.025693	0.012196	-
0.000511	0.087736	-0.050754	0.008375	-0.031682	0.119551	
x37	-0.068984	0.019629	-0.049948	0.041445	0.061552	-
0.000509	0.057553	-0.084271	0.145199	0.145026	0.105994	-
0.054472	-0.063261	-0.045345	0.067739	-0.132459	-0.071143	
0.153040	0.028664	0.034915	-0.003068	-0.005532	0.104878	
0.092389	0.097582	0.086782	-0.150925	-0.051723	-0.070993	
0.086671	0.012980	-0.044090	0.048621	-0.089913	0.064658	
y37	0.002305	0.027068	0.078275	0.080570	-0.089791	-
0.129409	-0.011453	0.037905	0.097306	0.011961	-0.058865	-
0.117140	-0.000193	-0.064632	0.077731	-0.068773	0.058413	-
0.054157	-0.081206	-0.030875	-0.005443	-0.092178	-0.088663	-
0.124035	-0.055791	-0.115472	0.093981	0.036778	0.060627	-
0.083423	-0.001576	0.037773	0.005710	0.016931	-0.114318	
z37	-0.006162	-0.065267	0.069595	0.001853	-0.000852	
0.078505	0.037886	0.081958	0.095148	-0.009500	0.017615	-
0.030765	0.101984	-0.023254	0.106354	-0.005388	-0.058776	-
0.044866	0.018110	-0.149524	0.031190	0.108118	-0.102189	
0.061582	0.059446	-0.053270	0.055959	-0.027055	0.063984	-
0.052911	0.089293	-0.027551	0.012985	-0.069068	0.076428	
x38	-0.088602	0.027572	-0.010142	0.042482	0.034981	-
0.035766	0.009604	-0.070249	0.160444	0.162657	0.097194	-
0.046382	-0.027946	-0.031343	0.003151	-0.064658	-0.094625	
0.073019	-0.015200	0.061565	0.014662	0.011791	0.080346	
0.078946	0.039762	0.039064	-0.025937	-0.086953	-0.126298	
0.024881	-0.029523	0.008366	-0.013353	0.048911	0.024763	
y38	0.009496	0.009558	0.083657	0.074520	-0.097379	-
0.164210	-0.026695	0.023148	0.066785	-0.012701	-0.071536	-
0.069087	0.007921	-0.035486	0.051675	-0.044718	0.043711	-
0.095241	-0.019337	0.004014	0.019573	-0.071735	-0.057479	-
0.101113	0.139991	-0.123018	-0.033079	0.176951	0.136268	
0.051964	0.068580	-0.012580	0.069693	-0.261103	0.046815	

z38	-0.005390	-0.054832	0.061871	0.006190	-0.030167	
0.088994	0.025107	0.090092	0.086216	-0.010269	-0.012698	-
0.004571	0.093626	-0.048487	0.053516	0.001035	-0.059222	-
0.020819	0.008234	-0.167516	0.075204	0.101441	0.011895	
0.019441	0.038943	-0.045484	0.047203	-0.026584	0.088426	-
0.071951	0.059189	-0.018927	-0.031819	-0.058082	-0.004048	
x39	-0.109697	0.036280	0.038499	0.043116	0.003404	-
0.091127	-0.050384	-0.047339	0.159446	0.173343	0.062297	-
0.015461	0.022803	0.001627	-0.074509	0.036444	-0.107989	-
0.045705	-0.041632	0.114405	0.041525	0.026641	0.085535	
0.087490	0.065546	-0.065816	0.086103	-0.055131	-0.035219	
0.044494	0.020246	0.008602	-0.063294	-0.046491	0.088908	
y39	0.020461	-0.010838	0.085909	0.065481	-0.099009	-
0.186913	-0.041809	0.014484	0.043975	-0.040076	-0.061631	-
0.024732	-0.002475	-0.019856	0.003561	-0.022981	0.015981	-
0.124149	0.034664	-0.014360	0.035595	-0.021343	-0.060596	-
0.130865	0.216964	-0.025058	-0.111140	0.186787	-0.033176	-
0.014254	-0.047527	0.093336	0.113760	-0.088162	-0.070601	
z39	-0.003417	-0.040848	0.050045	0.008767	-0.063137	
0.094777	0.005265	0.088057	0.073674	-0.008467	-0.042985	
0.029939	0.072181	-0.076151	-0.033546	-0.002496	-0.059729	-
0.006514	0.001565	-0.186151	0.112637	0.067732	0.161094	-
0.040370	-0.013060	-0.008383	-0.006773	-0.001886	0.011884	-
0.116935	-0.044491	-0.032705	-0.012470	-0.064653	-0.116167	
x40	-0.130506	0.043592	0.094032	0.042251	-0.037499	-
0.160241	-0.127674	-0.012753	0.155932	0.177347	0.024739	
0.032924	0.076641	0.034134	-0.187776	0.163084	-0.128574	-
0.198772	-0.051886	0.165829	0.050900	0.070851	0.078840	
0.072636	0.088092	-0.141293	0.231900	-0.006647	-0.001983	-
0.009498	0.017085	0.094723	-0.092812	0.064782	-0.006388	
y40	0.033253	-0.036149	0.083793	0.054771	-0.089811	-
0.181862	-0.025428	-0.015175	0.007542	-0.057350	-0.048047	
0.011748	-0.013067	-0.001574	0.006860	-0.007050	-0.017172	-
0.082704	0.073083	0.012742	0.031390	0.019919	-0.026936	-
0.087194	0.279432	0.000504	-0.189706	-0.007518	-0.078715	
0.162294	-0.055417	0.001263	-0.113066	0.114962	0.062189	
z40	-0.000502	-0.022959	0.034161	0.008322	-0.095254	
0.095841	-0.010492	0.070524	0.050882	-0.000864	-0.072992	
0.067057	0.047022	-0.106019	-0.128951	-0.008978	-0.051841	
0.031839	-0.011179	-0.178004	0.134043	0.032142	0.358151	-
0.105266	-0.111594	0.030540	-0.074022	-0.014846	-0.087658	-
0.031714	-0.173949	-0.033400	-0.054201	-0.053030	-0.074501	
x41	-0.136036	0.017114	0.104720	0.025640	-0.062870	-
0.135323	-0.112704	0.011981	0.099601	0.115569	0.017002	
0.079805	0.070798	0.050989	-0.158112	0.108816	-0.041370	-
0.101086	-0.027597	0.072656	0.019083	0.042459	0.018144	
0.059863	0.002355	-0.018936	0.103309	-0.029421	0.035132	-
0.057549	0.017731	0.040416	-0.017141	0.037677	-0.041444	
y41	0.053521	-0.065552	0.072735	0.047156	-0.079584	-
0.138496	0.031757	-0.032054	-0.004626	-0.094580	-0.062132	
0.045837	-0.056472	-0.014159	0.024518	0.005085	-0.056577	-
0.002518	0.128148	-0.051049	0.026710	0.061720	-0.069371	-
0.014805	0.127965	0.122271	0.030418	0.115658	-0.220864	-
0.333963	0.020040	-0.079551	0.238331	0.100650	-0.044286	
z41	0.005194	0.004630	0.009221	0.021514	-0.101336	
0.109889	-0.022594	0.042001	0.043543	-0.001396	-0.108117	
0.033213	-0.015373	-0.122768	-0.127400	0.013782	0.008624	
0.056462	-0.004596	-0.063145	0.080565	0.021771	0.171612	-
0.072951	-0.061941	0.039570	-0.078963	0.021396	-0.113344	-
0.085082	-0.066738	0.035492	0.077685	-0.013094	-0.032165	

x42	-0.138590	-0.007275	0.112818	0.010722	-0.085676	-
0.105839	-0.092773	0.028863	0.051631	0.059066	0.012787	
0.118687	0.060118	0.067546	-0.120289	0.069547	0.027904	-
0.004088	-0.014359	-0.001135	-0.007256	0.042504	-0.027642	
0.060702	-0.061725	0.085990	-0.013327	-0.006302	0.021804	-
0.039381	0.007433	0.012347	0.062154	0.020956	-0.047708	
y42	0.073098	-0.087630	0.058350	0.033941	-0.064824	-
0.114943	0.062796	-0.043015	-0.009405	-0.108766	-0.042851	
0.064434	-0.029143	-0.006508	0.052324	-0.010657	-0.083653	
0.044721	0.134546	0.029957	-0.022710	0.123724	0.042369	
0.120498	0.053917	0.117359	-0.127314	0.092765	-0.183647	-
0.018508	-0.009666	-0.057605	-0.233827	0.276089	-0.034889	
z42	0.010462	0.028720	-0.016901	0.036748	-0.105832	
0.116523	-0.034997	0.017895	0.039013	0.001427	-0.119210	
0.011655	-0.056461	-0.120394	-0.120623	0.024793	0.054592	
0.062218	-0.001991	0.051903	0.027042	0.027030	0.092599	-
0.003654	-0.006202	-0.007663	-0.070575	0.032262	-0.061500	
0.006279	0.028025	-0.006643	-0.000543	0.079540	-0.052659	
x43	-0.136759	-0.027169	0.116586	0.002133	-0.101096	-
0.078043	-0.067204	0.041520	0.012074	0.004502	0.019327	
0.144932	0.056501	0.067968	-0.089607	0.037135	0.071183	
0.067493	0.006333	-0.041940	-0.025235	0.034400	-0.091042	
0.055599	-0.108292	0.125667	-0.089506	0.015527	0.090844	-
0.034528	0.002254	0.009586	0.059102	0.002101	0.004493	
y43	0.092407	-0.093223	0.037640	0.034345	-0.033935	-
0.095552	0.089562	-0.052872	-0.013427	-0.115315	-0.005252	
0.078624	-0.032225	-0.043042	0.027228	-0.035832	-0.118134	
0.035469	0.167709	0.067735	-0.010572	0.069406	-0.039936	
0.160785	-0.015886	0.087005	-0.007046	0.026782	0.191658	-
0.130317	0.022519	0.111573	-0.049262	0.033704	-0.031078	
z43	0.016665	0.050641	-0.045277	0.051686	-0.103709	
0.108123	-0.043583	-0.002602	0.024945	-0.001387	-0.113098	-
0.010999	-0.085381	-0.122244	-0.116624	0.039001	0.083609	
0.056163	0.004986	0.133198	-0.001809	-0.007740	-0.017932	
0.028875	0.047940	-0.007953	-0.047884	0.019456	0.030533	-
0.021887	0.056464	0.048293	0.063061	0.066242	0.021778	
x44	-0.130699	-0.042087	0.116443	-0.003920	-0.110447	-
0.044247	-0.038365	0.045480	-0.025234	-0.040178	0.027802	
0.156609	0.042596	0.067508	-0.064274	0.006741	0.100296	
0.116375	0.029977	-0.047916	-0.061605	0.011279	-0.101740	
0.051544	-0.127644	0.117374	-0.128359	0.008133	0.077792	-
0.020728	-0.071638	-0.055377	0.071074	-0.025723	-0.030008	
y44	0.110543	-0.090809	0.019931	0.027664	-0.012572	-
0.058204	0.097543	-0.077909	-0.013933	-0.091148	0.026091	
0.091602	-0.064087	-0.041722	-0.011277	-0.045713	-0.124648	
0.053872	0.156846	0.090919	-0.072027	0.027163	0.084848	
0.207908	-0.030342	0.065692	0.063809	-0.080312	0.145628	-
0.056269	-0.219303	-0.003822	0.101885	-0.122328	0.075864	
z44	0.024551	0.067774	-0.072473	0.063203	-0.102285	
0.090455	-0.049901	-0.013555	0.007790	-0.001209	-0.093795	-
0.020051	-0.109339	-0.104589	-0.100940	0.039426	0.094272	
0.058892	0.019999	0.172643	-0.042326	-0.029934	-0.067430	
0.055871	0.087916	-0.015993	-0.013613	-0.014072	0.061143	
0.040785	0.022687	0.037505	0.057609	0.081719	0.067216	
x45	-0.121243	-0.054691	0.110844	-0.002857	-0.120829	-
0.001187	-0.009708	0.033397	-0.057195	-0.088531	0.035947	
0.152133	0.026031	0.057100	-0.046449	-0.006378	0.110659	
0.125617	0.025149	-0.080772	-0.003105	-0.058400	-0.086254	-
0.039754	-0.086762	0.122043	-0.101457	-0.030816	0.103179	
0.038399	0.057322	-0.054517	-0.062346	-0.009061	0.008484	

y45	0.129972	-0.091827	0.000513	0.025790	-0.005437	-
0.022826	0.100797	-0.109248	0.003216	-0.075022	0.035248	
0.080677	-0.060982	-0.045278	-0.041180	-0.030428	-0.115225	-
0.005917	0.095928	0.015591	0.050815	-0.064025	0.098181	
0.080653	0.015227	0.059830	0.098554	-0.230737	0.183204	-
0.006486	0.092812	0.053002	-0.046266	-0.114232	-0.051087	
z45	0.031987	0.081184	-0.097613	0.074508	-0.099374	
0.061602	-0.054572	-0.022807	-0.005926	-0.002705	-0.062082	-
0.032085	-0.105529	-0.086678	-0.075146	0.039131	0.084859	
0.044920	0.019608	0.185038	-0.051808	-0.055325	-0.113841	
0.052074	0.090197	-0.024316	0.001803	-0.047323	0.035727	
0.028238	0.040907	0.045594	-0.003157	0.063620	0.037750	
x46	-0.109044	-0.062450	0.100766	0.003652	-0.125050	
0.049289	0.020602	0.020164	-0.071819	-0.126095	0.059917	
0.135033	-0.005377	0.052277	-0.026583	-0.006573	0.099207	
0.144493	0.036728	-0.088175	0.053677	-0.061428	-0.008697	-
0.117381	-0.018432	0.078780	0.006125	-0.006498	0.102392	
0.197220	0.146125	-0.032030	-0.051486	0.070893	0.113027	
y46	0.148945	-0.085838	-0.019519	0.022048	0.003672	
0.007169	0.099692	-0.122818	0.040343	-0.036536	0.070679	
0.085581	-0.067896	-0.029171	-0.069657	0.004990	-0.097743	
0.011258	0.046057	-0.037742	0.109049	0.012714	0.170385	-
0.010889	0.069274	0.043371	0.182299	-0.159918	0.135708	
0.215657	0.129261	0.011553	0.058157	0.067288	0.049310	
z46	0.039192	0.091676	-0.122712	0.084162	-0.097468	
0.023513	-0.061967	-0.025607	-0.019115	-0.002585	-0.008780	-
0.039273	-0.096856	-0.050190	-0.043258	0.045901	0.065024	
0.039779	0.020107	0.161621	-0.072334	-0.043168	-0.117811	
0.045998	0.110830	0.012373	0.028111	-0.005763	-0.011711	
0.067547	0.015687	0.027483	-0.003140	0.000723	0.018102	
x47	-0.094338	-0.063492	0.095739	0.012938	-0.121197	
0.099778	0.056662	-0.000123	-0.083142	-0.157926	0.076027	
0.095133	-0.025151	0.027952	0.000223	0.004684	0.059617	
0.134073	0.021317	0.006581	0.025030	-0.062543	0.012701	-
0.094373	0.062632	-0.061073	0.010172	0.056434	-0.045250	
0.165797	0.040903	-0.067478	-0.054970	-0.005933	0.097207	
y47	0.166083	-0.072907	-0.029323	0.012773	0.011558	
0.024137	0.095070	-0.135070	0.079344	0.015382	0.084298	
0.080706	-0.038984	-0.019234	-0.087141	0.045352	-0.058204	-
0.004307	-0.041793	-0.018705	0.039315	0.060534	0.115165	-
0.012552	0.096286	-0.012014	0.025972	0.021881	0.001785	
0.156990	0.024400	-0.032205	0.041948	0.022507	0.019270	
z47	0.046294	0.097844	-0.147363	0.094787	-0.097180	-
0.024453	-0.066824	-0.022799	-0.035141	-0.003796	0.051580	-
0.038934	-0.072207	-0.004222	-0.008455	0.038130	0.034243	
0.037501	0.007614	0.101256	-0.075388	-0.048016	-0.097082	
0.007307	0.095339	0.071830	0.072508	0.048130	0.023530	
0.060761	0.021502	-0.009871	-0.037553	-0.029424	0.012526	
x48	-0.077818	-0.062963	0.084455	0.021050	-0.106706	
0.145094	0.097084	-0.026607	-0.081495	-0.179038	0.086305	
0.044786	-0.040605	0.008469	0.032769	0.009242	0.022369	
0.063387	-0.001145	0.105575	-0.025390	-0.056543	-0.019878	
0.024464	0.032015	-0.265522	0.024283	-0.028129	-0.111339	
0.019842	-0.097800	0.007899	-0.070830	-0.101781	-0.141233	
y48	0.181782	-0.058735	-0.043242	-0.002668	0.023101	
0.025117	0.083627	-0.143999	0.130609	0.083811	0.081390	
0.083325	0.001404	0.011242	-0.100504	0.078859	0.005439	-
0.055925	-0.143543	-0.045912	-0.025931	0.062577	-0.010304	
0.018758	-0.014314	-0.074185	-0.115356	0.020629	-0.032909	
0.081535	-0.083437	0.048253	-0.044164	-0.023805	-0.110394	

z48	0.053078	0.100026	-0.172739	0.102447	-0.096140	-
0.086201	-0.070162	-0.015681	-0.053152	-0.003511	0.124288	-
0.034316	-0.028556	0.060441	0.031957	0.024946	-0.000698	
0.016255	-0.016066	-0.009020	-0.072481	-0.055633	-0.054880	-
0.029807	0.021261	0.147604	0.114778	0.086733	0.115395	
0.016380	0.024005	-0.035933	-0.031293	-0.033547	-0.056014	
x49	-0.058442	-0.060887	0.064966	0.034078	-0.094157	
0.185051	0.129308	-0.062973	-0.081203	-0.196132	0.097791	
0.000823	-0.048519	0.010018	0.063185	0.011562	-0.015842	
0.016425	-0.020257	0.161126	-0.027577	-0.096032	-0.000862	
0.018745	-0.013444	-0.346272	0.078805	-0.025068	-0.073123	
0.004707	-0.027488	-0.044495	-0.112444	-0.070510	-0.115326	
y49	0.196150	-0.039356	-0.060019	-0.017511	0.022520	
0.018483	0.053903	-0.156333	0.184338	0.162921	0.081733	
0.098064	0.052731	0.066313	-0.119534	0.122455	0.084281	-
0.067629	-0.248079	-0.123096	-0.047368	0.039947	-0.093267	-
0.093342	-0.100600	-0.019356	-0.263195	0.130772	-0.025590	
0.161649	0.005479	-0.006625	-0.101846	0.066990	0.002876	
z49	0.059844	0.098038	-0.197226	0.108326	-0.091039	-
0.157838	-0.070161	-0.001985	-0.071495	0.001087	0.207708	-
0.025309	0.030093	0.129495	0.074583	0.015916	-0.049056	-
0.006780	-0.035599	-0.152188	-0.070783	-0.059304	0.015903	-
0.070115	-0.067616	0.199809	0.141646	0.115133	0.174575	-
0.019955	-0.041257	-0.099310	-0.085552	-0.069492	-0.147811	
x50	-0.027738	-0.054935	0.036228	0.043837	-0.084866	
0.224312	0.129888	-0.155360	-0.012351	-0.140610	0.145029	-
0.055167	-0.029946	0.056607	0.032878	0.069808	-0.034769	-
0.102566	-0.192132	0.048476	-0.054843	-0.007547	0.008501	
0.019081	-0.039704	-0.036902	0.047812	0.087398	0.031560	-
0.093515	-0.095837	0.172897	0.149674	-0.076447	0.028043	
y50	0.190367	-0.035058	-0.051600	-0.031873	0.028398	-
0.009686	0.051055	-0.069208	0.166877	0.148481	0.004716	
0.180693	0.125345	0.083855	-0.070936	0.026608	0.160314	
0.101847	-0.066883	0.017205	-0.080472	-0.011857	-0.099186	-
0.042227	-0.137974	-0.094895	-0.018084	0.032524	-0.027746	-
0.053213	0.025688	0.000285	-0.021900	-0.082574	0.019910	
z50	0.052941	0.085855	-0.192310	0.122676	-0.094224	-
0.197633	-0.027959	0.072189	-0.118278	-0.038132	0.259090	
0.051980	0.008985	0.147834	0.053968	-0.020792	-0.061610	-
0.009405	-0.039633	-0.079668	0.066370	0.031756	0.032238	
0.017902	-0.080535	-0.039385	-0.014455	-0.015981	-0.046001	-
0.064731	-0.020729	0.004340	0.043032	-0.002564	0.020568	
x51	-0.007083	-0.044068	0.020087	0.057793	-0.083569	
0.251537	0.132636	-0.221087	0.040595	-0.098998	0.180635	-
0.110799	-0.029496	0.110821	0.016193	0.079150	-0.025969	-
0.181748	-0.319411	-0.008153	-0.060631	0.036836	-0.011608	-
0.043845	-0.059110	0.259302	0.110177	0.081784	-0.125024	-
0.177934	0.126399	-0.032605	0.098901	0.124177	0.059435	
y51	0.178008	-0.027077	-0.038634	-0.040556	0.032158	-
0.032904	0.040189	0.014476	0.136459	0.124914	-0.064837	
0.251327	0.176212	0.094253	-0.034373	-0.070266	0.233272	
0.269118	0.099038	0.157444	-0.088376	-0.061750	-0.121895	-
0.004167	-0.140918	-0.179858	0.213602	-0.072668	-0.148520	-
0.228000	0.134065	-0.133722	0.035381	-0.102884	0.086825	
z51	0.047712	0.071393	-0.187310	0.131819	-0.095750	-
0.237354	0.013342	0.136515	-0.155828	-0.069673	0.293152	
0.115281	-0.016879	0.156249	0.041849	-0.062048	-0.078249	-
0.010871	-0.034684	-0.043262	0.183529	0.084109	0.024360	
0.061465	-0.079010	-0.281665	-0.163951	-0.178792	-0.219979	-
0.029412	-0.036292	0.051929	-0.003151	0.008959	0.123070	

Canonical Variate Analysis: CVA ... first turn of the cochlea

Dataset: first turn of the cochlea
Classification criterion: subfam

Groups		Observations
1.	Ca	9
2.	Ce	13
3.	Di	3
4.	Pr	11

Variation among groups, scaled by the inverse of the within-group variation

	Eigenvalues	% Variance	Cumulative %
1.	10.79349363	68.531	68.531
2.	3.14731277	19.983	88.514
3.	1.80898674	11.486	100.000

Mahalanobis distances among groups:

	Ca	Ce	Di
Ce	6.9268		
Di	5.3144	8.8245	
Pr	4.5354	6.2485	5.8018

P-values from permutation tests (10000 permutation rounds) for Mahalanobis distances among groups:

	Ca	Ce	Di
Ce	<.0001		
Di	0.0031	0.0021	
Pr	<.0001	<.0001	0.0027

Procrustes distances among groups:

	Ca	Ce	Di
Ce	0.0849		
Di	0.0467	0.0940	
Pr	0.0405	0.1056	0.0430

P-values from permutation tests (10000 permutation rounds) for Procrustes distances among groups:

	Ca	Ce	Di
Ce	0.0212		
Di	0.7711	0.0564	
Pr	0.6314	0.0021	0.7815

Canonical coefficients:

	CV1	CV2	CV3
x1	69.0605	-91.5493	169.4932
y1	-33.7250	-139.2759	110.2264
z1	-40.0761	-17.6408	-59.5399
x2	116.8768	-10.5154	45.3731
y2	26.5665	-62.5164	71.8243
z2	-37.1828	13.9051	3.6876

x3	6.3038	32.5565	-48.4241
y3	34.7933	51.2710	-30.0114
z3	-33.3799	26.4093	82.3017
x4	-30.3847	72.3465	-133.5230
y4	60.9532	137.2210	0.4219
z4	37.6614	70.1513	33.6105
x5	11.3497	-36.0656	82.8769
y5	61.3920	49.6867	57.8565
z5	58.4805	67.8041	-2.6054
x6	12.6762	-31.3663	-56.4494
y6	26.4679	26.2396	-28.1382
z6	123.0285	72.6861	92.1116
x7	-71.5650	-47.0718	24.9251
y7	21.5577	-29.0154	-48.1429
z7	119.1867	35.0008	19.4132
x8	-165.3072	61.8772	-192.0552
y8	-18.9554	-58.7071	-84.6884
z8	-16.9586	-25.2187	-70.9701
x9	-37.8958	62.3297	92.8964
y9	4.6870	-71.3555	-41.9277
z9	35.0523	-42.0043	-41.4493
x10	54.7240	-144.6173	76.3026
y10	-14.6573	12.4117	-33.9603
z10	-24.7641	-42.6465	23.3732
x11	-33.1297	-62.8284	-13.6383
y11	-17.2123	24.9755	19.6467
z11	-33.2452	-84.9130	-27.4961
x12	-71.2113	62.8400	-94.2976
y12	-6.8656	-11.8828	43.1821
z12	-54.3271	-56.1462	-54.0321
x13	72.9758	163.8474	-74.7694
y13	-84.2634	-58.5412	48.9057
z13	-130.1977	-16.1397	-102.5588
x14	58.6836	123.8411	20.0081
y14	-16.1686	0.4564	-62.4803
z14	12.2746	45.9310	-61.6824
x15	26.3539	108.1282	-53.9356
y15	17.5265	-83.7043	-40.2387
z15	52.3374	43.9351	57.8949
x16	-10.5152	-33.7778	0.9200
y16	66.3415	-23.3348	-27.8511
z16	-5.1330	47.5069	88.1054
x17	16.4736	-58.8942	127.2546
y17	40.6686	7.5326	-139.4371
z17	-46.7861	-32.7406	100.8529
x18	67.2369	-51.7159	30.9189
y18	-55.4453	26.5992	104.8740
z18	-51.9295	-60.0924	9.7239
x19	-32.5314	-48.7306	53.3864
y19	-36.7312	82.8409	-52.0036
z19	-40.0231	-62.6323	9.3985
x20	-36.3672	-36.9537	52.9331
y20	-2.3762	53.2771	-31.4235
z20	-25.0508	-40.6016	-20.8517
x21	-14.9878	-39.7978	25.5251
y21	-49.7813	38.7288	77.4963
z21	12.4770	28.5314	-37.1766
x22	-54.6590	-5.9401	-25.0150
y22	-163.8855	-39.3884	-1.3431
z22	0.9944	46.3986	-35.5207

x23	-57.5679	26.4600	-105.6434
y23	-75.2619	-5.3254	24.7839
z23	58.6776	76.2561	-15.5392
x24	-36.8345	14.3949	-78.3092
y24	-9.9887	-57.2093	55.4535
z24	67.7829	5.4634	10.0434
x25	73.5424	20.5748	34.6803
y25	95.9498	-38.8227	94.2543
z25	85.6812	-18.7510	74.0182
x26	104.6305	133.7212	-42.3559
y26	49.4629	81.1417	-90.8484
z26	13.6183	33.4902	0.5934
x27	150.0876	48.8366	152.8873
y27	24.9811	20.0393	-11.5725
z27	-21.1780	9.2061	3.9367
x28	130.5373	12.0301	137.7244
y28	-13.8226	16.0501	-38.0892
z28	-62.3267	2.8257	-20.8648
x29	127.7332	119.2552	-5.1838
y29	12.8158	65.2259	-55.3228
z29	7.7802	6.6089	-8.9263
x30	73.8292	-127.2221	162.6321
y30	11.9995	1.0580	41.7628
z30	91.4030	-10.9302	28.3986
x31	-35.4907	-173.8572	-113.0264
y31	14.0785	20.1354	102.1140
z31	147.0623	98.4238	-39.1516
x32	-104.2481	-65.7977	-62.1502
y32	50.3530	-4.5461	37.2429
z32	39.5150	-1.5611	-4.5301
x33	-108.8082	16.5260	-47.4308
y33	69.1017	-32.3886	32.4835
z33	-44.0271	-48.5099	-12.3731
x34	-172.9287	45.7814	-77.9148
y34	147.8018	-40.1465	76.1838
z34	-104.5809	-56.5513	-22.3931
x35	-97.2316	-27.6029	-153.4535
y35	144.7734	26.8099	174.1413
z35	-128.4488	-50.8114	12.4133
x36	37.4076	-51.7235	-11.8430
y36	33.3803	59.9252	23.5071
z36	-105.3372	-0.8958	60.5218
x37	0.4190	-46.5343	44.3752
y37	74.8940	60.0909	-75.5069
z37	-56.4949	35.3468	55.6531
x38	-33.3010	-30.4454	2.6149
y38	121.2484	56.0225	-13.2094
z38	-11.0898	32.3993	13.7052
x39	32.2601	40.5787	32.5093
y39	-26.8866	-25.1002	-153.9143
z39	86.7817	12.0119	-12.1189
x40	-10.7993	105.8423	-98.3609
y40	-187.9449	-183.1966	-145.9673
z40	189.0099	-47.5151	50.6760
x41	-8.1963	62.1537	-50.6968
y41	-141.9719	4.0507	-17.3594
z41	88.7121	-1.3180	23.7035
x42	-20.9695	19.2013	-13.6313
y42	-232.4069	-191.6591	-122.4050
z42	4.5693	-5.7368	-68.7990

x43	-52.3487	-28.1566	24.4406
y43	-129.0985	21.5742	-60.6975
z43	-50.0367	26.1237	-60.7077
x44	-19.6222	-63.3787	35.8641
y44	47.1809	40.6729	127.4958
z44	-84.6221	9.4902	-53.6420
x45	-37.0299	-89.9537	42.5972
y45	67.3465	105.6658	41.5875
z45	-76.9782	-3.8800	-56.9555
x46	-95.1148	-43.8216	35.6506
y46	-52.8346	128.9821	-3.4790
z46	-53.4024	-24.5677	-42.0327
x47	-25.8663	-59.3301	26.8421
y47	-43.1761	12.1463	-51.2884
z47	-42.2424	-49.0412	-17.7545
x48	112.5296	21.7536	-64.5712
y48	10.3540	-55.4307	-107.8872
z48	-18.0365	-36.7836	-18.4465
x49	141.1137	69.1877	-53.7860
y49	-64.6491	-169.3749	-97.3112
z49	48.5747	-66.1610	-7.7556
x50	50.2641	77.7337	28.7544
y50	49.0733	8.3971	68.4986
z50	9.0542	26.3791	45.2670
x51	-72.1571	-14.1497	106.0789
y51	92.3597	141.6933	232.5620
z51	8.1403	31.5055	76.4705

New Procrustes fit:
Dataset 'cochlea mid line'

108 landmarks in 3 dimensions.
The dataset contains 40 observations, of which 36 are included for analyses.

Average shape:

Lmk.	Axis 1 (x)	Axis 2 (y)	Axis 3 (z)
1	-0.13961712	-0.04891933	-0.06501708
2	-0.13830880	-0.05155906	-0.05770046
3	-0.13579267	-0.05343166	-0.05041357
4	-0.13277277	-0.05420365	-0.04336858
5	-0.12994602	-0.05384852	-0.03636613
6	-0.12700110	-0.05239422	-0.02942302
7	-0.12438595	-0.05002226	-0.02234504
8	-0.12336136	-0.04632431	-0.01538577
9	-0.11915269	-0.04276146	-0.00679244
10	-0.11974742	-0.03470042	-0.00254143
11	-0.11757218	-0.02846314	0.00323452
12	-0.11547199	-0.02197627	0.00828862
13	-0.11329311	-0.01517063	0.01249849
14	-0.11107914	-0.00808114	0.01587298
15	-0.10882600	-0.00070658	0.01844793
16	-0.10659072	0.00696651	0.02032121
17	-0.10422842	0.01500103	0.02123440
18	-0.10165325	0.02336162	0.02112570
19	-0.09881901	0.03189506	0.02012242
20	-0.09585076	0.04038135	0.01807323
21	-0.09232732	0.04841612	0.01558475

22	-0.08843685	0.05614365	0.01286610
23	-0.08382407	0.06336536	0.00976588
24	-0.07877358	0.07013005	0.00617155
25	-0.07306929	0.07644511	0.00215734
26	-0.06678931	0.08238197	-0.00219497
27	-0.05986225	0.08781807	-0.00700003
28	-0.05222257	0.09249710	-0.01226698
29	-0.03979413	0.09813950	-0.01978257
30	-0.02681553	0.10175262	-0.02633783
31	-0.01348958	0.10354316	-0.03180866
32	0.00012014	0.10379656	-0.03621447
33	0.01413626	0.10266598	-0.03963844
34	0.02862107	0.09977583	-0.04202089
35	0.04359716	0.09511307	-0.04331603
36	0.05892761	0.08834914	-0.04350950
37	0.07412076	0.07906730	-0.04247252
38	0.08974133	0.06584319	-0.04054024
39	0.10167428	0.05119906	-0.03838804
40	0.10979210	0.03613915	-0.03632502
41	0.11475888	0.02136515	-0.03446429
42	0.11704509	0.00698045	-0.03277682
43	0.11705314	-0.00718681	-0.03133946
44	0.11485333	-0.02137947	-0.03013717
45	0.10996692	-0.03568882	-0.02911719
46	0.10189267	-0.04980751	-0.02821183
47	0.09372319	-0.05980663	-0.02777726
48	0.08468233	-0.06789089	-0.02715401
49	0.07512172	-0.07418239	-0.02632242
50	0.06517800	-0.07876997	-0.02531293
51	0.05493611	-0.08196274	-0.02413457
52	0.04426087	-0.08353467	-0.02274569
53	0.03313750	-0.08363754	-0.02116639
54	0.02154743	-0.08201643	-0.01942827
55	0.00969271	-0.07844352	-0.01751176
56	-0.00057347	-0.07381117	-0.01461363
57	-0.00949981	-0.06818641	-0.01157972
58	-0.01704314	-0.06170746	-0.00848794
59	-0.02333334	-0.05459782	-0.00533707
60	-0.02833697	-0.04681060	-0.00216970
61	-0.03228668	-0.03840419	0.00109894
62	-0.03514697	-0.02925883	0.00451014
63	-0.03658267	-0.01929730	0.00798928
64	-0.03640297	-0.00859708	0.01140214
65	-0.03496711	0.00198693	0.01395775
66	-0.03198610	0.01178726	0.01583848
67	-0.02804529	0.02077755	0.01727367
68	-0.02305460	0.02898948	0.01821965
69	-0.01671264	0.03632304	0.01849088
70	-0.00982486	0.04317496	0.01872483
71	-0.00138581	0.04904767	0.01806858
72	0.00816864	0.05414000	0.01703640
73	0.01910331	0.05777642	0.01545230
74	0.03154595	0.05940575	0.01484373
75	0.04319334	0.05872208	0.01421684
76	0.05363226	0.05631071	0.01367559
77	0.06308537	0.05252409	0.01324632
78	0.07153979	0.04735980	0.01303046
79	0.07930495	0.04081344	0.01290201
80	0.08618101	0.03258272	0.01281880
81	0.09177573	0.02240991	0.01282964

82	0.09553100	0.01049152	0.01287164
83	0.09690812	-0.00022042	0.01309746
84	0.09650337	-0.01024668	0.01337348
85	0.09475812	-0.01942937	0.01372050
86	0.09163204	-0.02777618	0.01413648
87	0.08739923	-0.03539819	0.01462508
88	0.08185451	-0.04232171	0.01514608
89	0.07507400	-0.04867499	0.01577190
90	0.06670978	-0.05410568	0.01642920
91	0.05679276	-0.05800029	0.01725216
92	0.04641544	-0.05967804	0.01902802
93	0.03691836	-0.05918779	0.02091115
94	0.02866861	-0.05731258	0.02293233
95	0.02157942	-0.05428494	0.02502496
96	0.01536083	-0.05027523	0.02732204
97	0.00991537	-0.04516868	0.02984258
98	0.00533623	-0.03875057	0.03267855
99	0.00226855	-0.03053148	0.03529436
100	0.00114704	-0.02059200	0.03714369
101	0.00313556	-0.00986485	0.03957626
102	0.00724134	-0.00118880	0.04116888
103	0.01195647	0.00481788	0.04210933
104	0.01654782	0.00891537	0.04279771
105	0.02138571	0.01176635	0.04351316
106	0.02782046	0.01253997	0.04423823
107	0.03537882	0.01212485	0.04534254
108	0.04370747	0.00722849	0.04421855

Procrustes sums of squares: 1.0221664666196177
Tangent sums of squares: 0.8627823692566696

Data matrices in this dataset:
- cochlea mid line, raw data
- cochlea mid line, centroid size
- cochlea mid line, Procrustes coordinates

New Procrustes fit:
Dataset 'cochlea mid line'

108 landmarks in 3 dimensions.
The dataset contains 40 observations, of which 36 are included for analyses.

Average shape:

Lmk.	Axis 1 (x)	Axis 2 (y)	Axis 3 (z)
1	-0.13961712	-0.04891933	-0.06501708
2	-0.13830880	-0.05155906	-0.05770046
3	-0.13579267	-0.05343166	-0.05041357
4	-0.13277277	-0.05420365	-0.04336858
5	-0.12994602	-0.05384852	-0.03636613
6	-0.12700110	-0.05239422	-0.02942302
7	-0.12438595	-0.05002226	-0.02234504
8	-0.12336136	-0.04632431	-0.01538577
9	-0.11915269	-0.04276146	-0.00679244
10	-0.11974742	-0.03470042	-0.00254143
11	-0.11757218	-0.02846314	0.00323452
12	-0.11547199	-0.02197627	0.00828862

13	-0.11329311	-0.01517063	0.01249849
14	-0.11107914	-0.00808114	0.01587298
15	-0.10882600	-0.00070658	0.01844793
16	-0.10659072	0.00696651	0.02032121
17	-0.10422842	0.01500103	0.02123440
18	-0.10165325	0.02336162	0.02112570
19	-0.09881901	0.03189506	0.02012242
20	-0.09585076	0.04038135	0.01807323
21	-0.09232732	0.04841612	0.01558475
22	-0.08843685	0.05614365	0.01286610
23	-0.08382407	0.06336536	0.00976588
24	-0.07877358	0.07013005	0.00617155
25	-0.07306929	0.07644511	0.00215734
26	-0.06678931	0.08238197	-0.00219497
27	-0.05986225	0.08781807	-0.00700003
28	-0.05222257	0.09249710	-0.01226698
29	-0.03979413	0.09813950	-0.01978257
30	-0.02681553	0.10175262	-0.02633783
31	-0.01348958	0.10354316	-0.03180866
32	0.00012014	0.10379656	-0.03621447
33	0.01413626	0.10266598	-0.03963844
34	0.02862107	0.09977583	-0.04202089
35	0.04359716	0.09511307	-0.04331603
36	0.05892761	0.08834914	-0.04350950
37	0.07412076	0.07906730	-0.04247252
38	0.08974133	0.06584319	-0.04054024
39	0.10167428	0.05119906	-0.03838804
40	0.10979210	0.03613915	-0.03632502
41	0.11475888	0.02136515	-0.03446429
42	0.11704509	0.00698045	-0.03277682
43	0.11705314	-0.00718681	-0.03133946
44	0.11485333	-0.02137947	-0.03013717
45	0.10996692	-0.03568882	-0.02911719
46	0.10189267	-0.04980751	-0.02821183
47	0.09372319	-0.05980663	-0.02777726
48	0.08468233	-0.06789089	-0.02715401
49	0.07512172	-0.07418239	-0.02632242
50	0.06517800	-0.07876997	-0.02531293
51	0.05493611	-0.08196274	-0.02413457
52	0.04426087	-0.08353467	-0.02274569
53	0.03313750	-0.08363754	-0.02116639
54	0.02154743	-0.08201643	-0.01942827
55	0.00969271	-0.07844352	-0.01751176
56	-0.00057347	-0.07381117	-0.01461363
57	-0.00949981	-0.06818641	-0.01157972
58	-0.01704314	-0.06170746	-0.00848794
59	-0.02333334	-0.05459782	-0.00533707
60	-0.02833697	-0.04681060	-0.00216970
61	-0.03228668	-0.03840419	0.00109894
62	-0.03514697	-0.02925883	0.00451014
63	-0.03658267	-0.01929730	0.00798928
64	-0.03640297	-0.00859708	0.01140214
65	-0.03496711	0.00198693	0.01395775
66	-0.03198610	0.01178726	0.01583848
67	-0.02804529	0.02077755	0.01727367
68	-0.02305460	0.02898948	0.01821965
69	-0.01671264	0.03632304	0.01849088
70	-0.00982486	0.04317496	0.01872483
71	-0.00138581	0.04904767	0.01806858
72	0.00816864	0.05414000	0.01703640

73	0.01910331	0.05777642	0.01545230
74	0.03154595	0.05940575	0.01484373
75	0.04319334	0.05872208	0.01421684
76	0.05363226	0.05631071	0.01367559
77	0.06308537	0.05252409	0.01324632
78	0.07153979	0.04735980	0.01303046
79	0.07930495	0.04081344	0.01290201
80	0.08618101	0.03258272	0.01281880
81	0.09177573	0.02240991	0.01282964
82	0.09553100	0.01049152	0.01287164
83	0.09690812	-0.00022042	0.01309746
84	0.09650337	-0.01024668	0.01337348
85	0.09475812	-0.01942937	0.01372050
86	0.09163204	-0.02777618	0.01413648
87	0.08739923	-0.03539819	0.01462508
88	0.08185451	-0.04232171	0.01514608
89	0.07507400	-0.04867499	0.01577190
90	0.06670978	-0.05410568	0.01642920
91	0.05679276	-0.05800029	0.01725216
92	0.04641544	-0.05967804	0.01902802
93	0.03691836	-0.05918779	0.02091115
94	0.02866861	-0.05731258	0.02293233
95	0.02157942	-0.05428494	0.02502496
96	0.01536083	-0.05027523	0.02732204
97	0.00991537	-0.04516868	0.02984258
98	0.00533623	-0.03875057	0.03267855
99	0.00226855	-0.03053148	0.03529436
100	0.00114704	-0.02059200	0.03714369
101	0.00313556	-0.00986485	0.03957626
102	0.00724134	-0.00118880	0.04116888
103	0.01195647	0.00481788	0.04210933
104	0.01654782	0.00891537	0.04279771
105	0.02138571	0.01176635	0.04351316
106	0.02782046	0.01253997	0.04423823
107	0.03537882	0.01212485	0.04534254
108	0.04370747	0.00722849	0.04421855

Procrustes sums of squares: 1.0221664666196177
Tangent sums of squares: 0.8627823692566696

Data matrices in this dataset:
- cochlea mid line, raw data
- cochlea mid line, centroid size
- cochlea mid line, Procrustes coordinates

Principal Component Analysis: PCA: CovMatrix, cochlea mid line, Procrustes coordinates

	Eigenvalues	% Variance	Cumulative %
1.	0.01066989	43.284	43.284
2.	0.00451822	18.329	61.613
3.	0.00208907	8.475	70.087
4.	0.00193676	7.857	77.944
5.	0.00121538	4.930	82.874
6.	0.00081837	3.320	86.194
7.	0.00069594	2.823	89.017
8.	0.00049694	2.016	91.033

9.	0.00041366	1.678	92.711
10.	0.00035174	1.427	94.138
11.	0.00021361	0.867	95.005
12.	0.00016803	0.682	95.686
13.	0.00015841	0.643	96.329
14.	0.00012748	0.517	96.846
15.	0.00011176	0.453	97.300
16.	0.00009345	0.379	97.679
17.	0.00008303	0.337	98.015
18.	0.00006735	0.273	98.289
19.	0.00006050	0.245	98.534
20.	0.00005655	0.229	98.763
21.	0.00005163	0.209	98.973
22.	0.00004194	0.170	99.143
23.	0.00003833	0.155	99.299
24.	0.00003272	0.133	99.431
25.	0.00002496	0.101	99.533
26.	0.00001965	0.080	99.612
27.	0.00001723	0.070	99.682
28.	0.00001623	0.066	99.748
29.	0.00001357	0.055	99.803
30.	0.00001215	0.049	99.852
31.	0.00001086	0.044	99.896
32.	0.00000926	0.038	99.934
33.	0.00000760	0.031	99.965
34.	0.00000540	0.022	99.987
35.	0.00000330	0.013	100.000

Total variance: 0.02465092

Variance of the eigenvalues: 0.0000004536157
Eigenvalue variance scaled by total variance: 0.00075
Eigenvalue variance scaled by total variance and number of variables: 0.23738
Note: throughout all calculations of eigenvalue variances, the dimensionality used was 317.
Please double-check because this dimensionality may not be appropriate for all situations.
Warning: There appear to be fewer PCs than dimensions in shape space.
This can have the following reasons, among others:
-- the degrees of freedom are less than the shape dimensionality (sample size, etc.).
-- there is complete integration in the data.
Interpret the results cautiously and check for reasons why this is the case.

Principal Component Coefficients

```
        PC1         PC2         PC3         PC4         PC5         PC6
PC7          PC8         PC9         PC10        PC11        PC12
PC13         PC14        PC15        PC16        PC17        PC18
PC19         PC20        PC21        PC22        PC23        PC24
PC25         PC26        PC27        PC28        PC29        PC30
PC31         PC32        PC33        PC34        PC35
   x1  0.120154  -0.122339  -0.009522   0.142006   0.022316  -0.012276
0.124496   -0.006213  -0.028629  -0.005222  -0.028088   0.064253
0.002975   -0.071767  -0.182386  -0.038784  -0.072028  -0.171537
0.054258   -0.055457   0.150946  -0.033217  -0.044704   0.010789
0.183752    0.090887   0.144077  -0.030544  -0.080632  -0.016668
0.036720   -0.115904  -0.079821   0.040059  -0.123337
```

y1 -0.021583 -0.054426 -0.025554 -0.067705 0.175458 -0.064270
0.071223 0.080704 0.004410 0.015266 -0.144859 -0.116111
0.075570 -0.164476 -0.015773 -0.045052 -0.156268 -0.011642 -
0.087518 0.090449 -0.117965 0.121476 -0.160655 -0.111453
0.097540 -0.111541 0.140903 -0.013006 -0.053870 0.013925
0.018115 0.092267 0.072395 -0.033499 -0.032168
z1 0.060443 -0.051941 0.021403 0.085207 0.051740 -0.061797
-0.066742 0.082196 -0.014382 -0.097926 -0.060086 -0.053543
-0.009585 -0.266404 -0.120528 -0.095473 0.227169 0.099433
-0.096539 -0.184721 0.076992 0.140727 -0.145699 -0.019356
-0.176445 -0.017045 -0.101702 -0.073755 0.071111 -0.007389
-0.127638 -0.172567 -0.008409 0.036226 -0.049328
x2 0.120236 -0.117837 -0.014824 0.138467 0.008240 -0.003958
0.108373 -0.006072 -0.047848 -0.009563 -0.039779 0.076237
0.002178 -0.023804 -0.136365 -0.022588 -0.071824 -0.144901
0.050920 -0.049222 0.142410 -0.050769 0.001726 0.043553
0.112987 0.001996 0.082364 -0.038546 -0.077408 0.015097 -
0.004110 -0.098460 -0.016810 -0.014445 0.032503
y2 -0.015030 -0.053101 -0.019523 -0.070049 0.179258 -0.075945
0.062896 0.079347 -0.010380 0.005238 -0.151421 -0.093665
0.037449 -0.144676 -0.009310 -0.029456 -0.125216 -0.019272 -
0.079709 0.055682 -0.094805 0.066089 -0.068629 -0.031347
0.033271 -0.066508 0.073245 0.015117 -0.071682 0.034953
0.005387 0.060954 0.027940 0.015556 0.026285
z2 0.063975 -0.033610 0.010319 0.074039 0.027103 -0.078715
-0.083915 0.077138 -0.048781 -0.097257 -0.048153 -0.026753
-0.030454 -0.179047 -0.078932 -0.070017 0.130751 0.100048
-0.057708 -0.154881 0.020785 0.046211 -0.055867 0.016607
-0.125509 -0.014559 -0.078981 -0.059671 0.056160 0.025429
-0.075940 -0.092749 -0.004859 -0.010935 -0.017121
x3 0.122216 -0.113642 -0.022447 0.142035 -0.003858 0.001008
0.100497 -0.007635 -0.050514 -0.022615 -0.033628 0.074822 -
0.003352 0.005147 -0.082224 -0.000807 -0.070904 -0.109707
0.030718 -0.028306 0.103003 -0.044867 0.004949 0.030389
0.063360 -0.024657 0.009904 -0.049222 -0.030773 0.040002
0.000495 -0.058502 0.050669 -0.057041 0.098444
y3 -0.009000 -0.046874 -0.014754 -0.075568 0.177125 -0.080910
0.053961 0.072843 -0.021524 0.000639 -0.151720 -0.064307
0.004620 -0.104779 -0.006996 -0.005712 -0.086866 -0.043027 -
0.056168 0.024084 -0.064219 0.017325 0.016851 0.022574 -
0.007404 -0.044321 -0.004798 0.015806 -0.040271 0.043784
0.004885 0.026092 0.007431 0.026163 0.019279
z3 0.070859 -0.023909 0.004405 0.069185 0.008734 -0.086558
-0.095274 0.070098 -0.070659 -0.092272 -0.030401 -0.000882
-0.039134 -0.101783 -0.051523 -0.047480 0.059226 0.080453
-0.016578 -0.126961 -0.019522 -0.017024 0.012939 0.035383
-0.064821 -0.017122 -0.065374 -0.040580 0.034911 0.019025
-0.033676 -0.020520 -0.004912 -0.022916 -0.025167
x4 0.121161 -0.109951 -0.026723 0.143924 -0.011168 0.008902
0.094616 -0.015234 -0.050343 -0.035518 -0.027447 0.064576
0.002561 0.013848 -0.041768 0.018055 -0.083524 -0.079503
0.011294 -0.007166 0.066703 -0.025899 0.004943 0.021515
0.009892 -0.023820 0.004149 -0.032260 -0.016454 0.047622
0.014476 -0.005848 0.066962 -0.072405 0.101044
y4 -0.005095 -0.035434 -0.011596 -0.084258 0.168627 -0.083385
0.044302 0.063140 -0.030435 -0.003751 -0.146567 -0.042831 -
0.018994 -0.062074 -0.004576 0.008330 -0.063738 -0.055509 -
0.033156 0.001205 -0.036504 -0.021897 0.078736 0.055059 -
0.043172 -0.032096 -0.041295 0.017023 -0.012463 0.045837
0.008124 -0.007077 -0.004394 0.015558 0.020069

z4 0.073566 -0.012096 -0.002484 0.060142 -0.008468 -0.087685
-0.101078 0.062509 -0.087759 -0.083327 -0.017360 0.024412
-0.043481 -0.033543 -0.028916 -0.032330 0.006252 0.061570
0.012288 -0.097944 -0.049885 -0.071145 0.064336 0.052967 -
0.018466 -0.032455 -0.056753 -0.027783 0.017668 0.013163 -
0.013739 0.035723 -0.004444 -0.042334 0.000338
x5 0.117997 -0.109307 -0.031049 0.143015 -0.019777 0.014523
0.090552 -0.012380 -0.051299 -0.044309 -0.011629 0.051951
0.005696 0.019352 -0.019577 0.022397 -0.089139 -0.044486 -
0.000751 0.011449 0.009445 0.017918 -0.013695 0.005281 -
0.027251 -0.001471 -0.009676 -0.014684 -0.005060 0.021958
0.017736 0.029753 0.052346 -0.058057 0.107974
y5 -0.000407 -0.026716 -0.008762 -0.094004 0.155779 -0.086492
0.035975 0.054337 -0.037792 -0.007587 -0.132302 -0.024864 -
0.036912 -0.027970 -0.003089 0.009625 -0.045130 -0.052463 -
0.015735 -0.012256 -0.018198 -0.043195 0.110944 0.071264 -
0.063795 -0.002009 -0.056000 0.011996 0.013604 0.024403
0.019689 -0.051081 -0.033617 0.024682 0.018086
z5 0.073701 0.001126 -0.009457 0.049161 -0.022070 -0.082455
-0.101044 0.052215 -0.099969 -0.073701 -0.008694 0.049980
-0.041748 0.021565 -0.000265 -0.020914 -0.033989 0.042797
0.027610 -0.071427 -0.062612 -0.102412 0.105307 0.068418
0.021207 -0.038392 -0.043966 -0.029388 0.009358 0.020593
0.004634 0.062660 0.008979 -0.032019 0.024492
x6 0.115552 -0.106467 -0.035485 0.141742 -0.019997 0.023202
0.092281 -0.015697 -0.048443 -0.054572 -0.001617 0.042186
0.016273 0.022942 0.012964 0.023174 -0.084604 -0.023706 -
0.017405 0.025336 -0.029056 0.025025 -0.002696 -0.009181 -
0.060472 0.010942 -0.027105 0.028367 0.019979 0.022349
0.036291 0.070917 0.024023 -0.039292 0.039860
y6 0.006297 -0.017613 -0.006986 -0.102377 0.142185 -0.086105
0.028085 0.040843 -0.044482 -0.013224 -0.113192 -0.009835 -
0.044657 0.009703 0.007437 0.007189 -0.038987 -0.046991
0.001405 -0.010798 -0.000382 -0.064026 0.134402 0.066700 -
0.076291 0.028474 -0.052925 0.011299 0.030012 0.007904
0.023789 -0.056131 -0.059622 0.013322 0.019482
z6 0.072378 0.011477 -0.014912 0.038969 -0.032316 -0.068856
-0.098688 0.044032 -0.103931 -0.060044 0.000210 0.069710
-0.032779 0.060225 0.023687 -0.014698 -0.057651 0.025220
0.031243 -0.046390 -0.069438 -0.107777 0.119072 0.078338
0.041583 -0.046710 -0.024929 -0.023645 -0.023451 0.013392
0.007991 0.085013 0.004964 -0.026220 0.056569
x7 0.109533 -0.101777 -0.038442 0.138708 -0.020386 0.031860
0.096649 -0.014030 -0.041918 -0.058326 0.007685 0.038330
0.020570 0.010736 0.047974 0.022402 -0.059388 0.003546 -
0.035589 0.034992 -0.055107 0.030583 0.003238 -0.007815 -
0.074635 0.016683 -0.062311 0.026827 0.048368 -0.000192
0.067813 0.069848 0.024948 0.016910 -0.056841
y7 0.014636 -0.010811 -0.004912 -0.109836 0.124946 -0.084778
0.021259 0.028201 -0.048040 -0.015389 -0.086513 0.008432 -
0.050860 0.041531 0.020753 -0.002479 -0.026543 -0.026477
0.010207 0.000596 0.016721 -0.080774 0.134003 0.052117 -
0.078049 0.057971 -0.052922 -0.013708 0.052573 -0.024155
0.011218 -0.068657 -0.054640 0.028831 0.009376
z7 0.069850 0.021488 -0.021128 0.027746 -0.037351 -0.049178
-0.093471 0.036487 -0.104392 -0.047239 0.012755 0.084194
-0.017187 0.091463 0.044627 -0.017107 -0.074875 0.003129
0.025472 -0.026292 -0.067669 -0.075875 0.115758 0.067727
0.052191 -0.044051 0.015703 0.005903 -0.052114 0.015238
0.012770 0.081305 -0.005573 -0.007837 0.067261

x8 0.052333 -0.023072 -0.057847 0.074709 -0.044245 0.035359
0.099515 -0.016831 -0.052363 -0.051850 -0.003743 0.016773 -
0.016878 0.013109 0.073013 0.013360 -0.091477 0.010806 -
0.030884 0.062557 -0.054201 -0.002031 0.026035 0.002375 -
0.088891 -0.017914 -0.051834 0.057781 0.012231 0.025034
0.071998 0.032768 0.062084 -0.055650 -0.003556
y8 0.037789 -0.021420 -0.000321 -0.102654 0.113250 -0.076853
0.014168 0.008032 -0.046352 -0.019972 -0.049571 0.028780 -
0.037338 0.077596 0.038982 -0.006703 -0.009089 -0.009832
0.012048 0.007977 0.025362 -0.083084 0.114170 0.024932 -
0.070189 0.082399 -0.011439 -0.005192 0.051603 -0.054824 -
0.004334 -0.053185 -0.068735 0.004529 -0.011545
z8 0.053573 0.045089 -0.028677 0.002292 -0.044308 -0.026018
-0.082369 0.026493 -0.097469 -0.032059 0.015648 0.096430
-0.001669 0.099507 0.070409 -0.039056 -0.075810 -0.011532
-0.003945 -0.002466 -0.048493 -0.041203 0.103468 0.044656
0.057927 -0.032724 0.032622 0.017055 -0.039930 0.013684
0.035091 0.052049 -0.010299 0.054055 0.043770
x9 0.109922 -0.107167 -0.040510 0.143391 -0.004141 0.064952
0.104943 -0.009459 -0.019905 -0.055231 0.041023 0.030120
0.055008 -0.006827 0.087701 0.025106 -0.006025 0.021960 -
0.050773 0.019893 -0.110265 0.086959 0.000741 0.010424 -
0.101657 0.006497 -0.022941 0.117939 0.007281 -0.081561
0.049302 0.118414 -0.069399 0.057764 -0.251131
y9 0.017433 0.028638 -0.008123 -0.150380 0.073092 -0.074719
0.006852 -0.015100 -0.052878 -0.014831 -0.028104 0.044366 -
0.049971 0.116344 0.058021 -0.047244 -0.037124 0.007333
0.015412 0.031233 0.063076 -0.110722 0.094812 -0.015466 -
0.050255 0.092107 0.048529 -0.021119 0.056767 -0.054644 -
0.001242 -0.092169 -0.026435 -0.029245 0.035781
z9 0.101849 -0.022584 -0.018210 0.057190 -0.013732 0.014806
-0.067799 0.022828 -0.068534 -0.029666 0.058234 0.111110
0.064846 0.096933 0.093067 -0.028768 -0.010638 -0.022674 -
0.039604 0.017766 -0.064214 0.077208 0.035559 0.003884
0.044581 0.010746 0.037182 0.040944 -0.046171 -0.035129
0.006076 0.095779 -0.069130 0.150682 -0.042172
x10 -0.023753 0.072854 -0.079117 -0.020545 -0.063600
0.055610 0.108605 -0.020449 -0.040437 -0.032949 -0.008442
0.008816 -0.024944 -0.021798 0.120194 -0.041189 -0.080328
0.035933 -0.054453 0.042409 -0.056360 0.025810 0.059950
0.018916 -0.055132 -0.028137 0.007769 0.141853 0.042726
0.033717 0.086647 -0.056659 0.032918 0.070706 -0.094725
y10 0.131993 -0.106712 0.021729 -0.040179 0.098624 -
0.045268 -0.001608 -0.029721 -0.015996 -0.030650 0.070827
0.082618 0.050022 0.150661 0.079017 -0.039216 0.043511
0.036818 0.001938 0.022186 0.024225 0.009197 -0.010903 -
0.088461 -0.039832 0.155465 0.109603 -0.009237 0.068939 -
0.136065 -0.063051 0.013705 -0.095460 0.055628 -0.080882
z10 -0.015589 0.135827 -0.053783 -0.080641 -0.062133
0.031200 -0.044049 0.007961 -0.084410 0.004157 0.009051
0.095570 0.007986 0.090802 0.116471 -0.086811 -0.076487 -
0.045509 -0.073832 0.085690 0.029486 0.051431 0.031507 -
0.029691 0.052092 0.013170 0.074736 0.005867 -0.050739 -
0.008504 0.060511 -0.059302 0.015812 0.108520 0.031234
x11 -0.027833 0.071810 -0.083748 -0.026068 -0.058564
0.048756 0.107299 -0.008372 -0.036657 -0.011817 0.005414 -
0.018544 -0.042394 -0.021327 0.126800 -0.029533 -0.053548
0.029624 -0.064152 0.012002 -0.066273 0.015577 0.054600 -
0.018345 -0.058186 -0.032795 -0.018794 0.095005 0.029051
0.005309 0.058444 -0.036044 0.020441 0.024350 -0.092652

y11	0.137792	-0.085732	0.007223	-0.054236	0.056924	-
0.042258	-0.008289	-0.032496	-0.016795	-0.020799	0.086587	
0.067143	0.047551	0.139153	0.072650	-0.037543	0.048089	
0.032698	-0.030930	-0.001223	0.041147	0.007776	-0.041377	-
0.118913	0.001588	0.117413	0.073116	-0.010989	0.055125	-
0.050356	-0.060955	-0.015204	-0.027417	0.072032	-0.056936	
z11	-0.014489	0.130658	-0.057980	-0.068117	-0.061364	
0.043891	-0.037809	0.009267	-0.080260	0.005998	-0.007925	
0.083297	0.032947	0.045644	0.061286	-0.048096	-0.066528	-
0.075411	-0.060016	0.087141	0.051808	0.045265	-0.005271	-
0.008677	0.035728	0.021029	0.060811	0.000216	-0.065632	
0.016690	0.046431	-0.031524	0.030800	0.048991	0.019571	
x12	-0.028141	0.071370	-0.087204	-0.028485	-0.057178	
0.049693	0.104803	-0.001315	-0.037590	0.009545	0.014181	-
0.031805	-0.041718	-0.036301	0.121353	-0.014777	-0.028937	
0.022386	-0.051398	-0.006254	-0.062167	0.001634	0.035402	-
0.046225	-0.048380	0.040478	-0.021311	0.053618	0.001558	-
0.006239	0.039012	-0.015711	-0.003953	0.017257	-0.057978	
y12	0.142308	-0.065802	-0.006400	-0.067444	0.018103	-
0.042202	-0.011525	-0.039434	-0.020188	-0.005113	0.094891	
0.053938	0.041739	0.125644	0.064521	-0.029820	0.048787	
0.028738	-0.057999	-0.022840	0.061065	0.005291	-0.066962	-
0.142722	0.018484	0.088661	0.022125	0.001927	0.016291	
0.010569	-0.029027	-0.026003	0.025118	0.046374	-0.019780	
z12	-0.015443	0.121699	-0.059316	-0.056812	-0.053974	
0.053106	-0.031983	0.015997	-0.068294	0.002699	-0.018024	
0.064451	0.044318	0.017569	0.021858	-0.019841	-0.051163	-
0.094498	-0.060434	0.087262	0.055489	0.038254	-0.030215	
0.008005	0.025604	-0.019950	0.047712	-0.013226	-0.041149	
0.028067	0.000805	-0.024048	0.028097	0.016879	-0.008443	
x13	-0.026891	0.069380	-0.089963	-0.033720	-0.051481	
0.046407	0.097406	0.004475	-0.025858	0.009281	0.031269	-
0.039404	-0.031187	-0.051909	0.110247	0.009814	0.003213	
0.016673	-0.031416	-0.026423	-0.062762	-0.000010	0.028702	-
0.064546	-0.050196	0.060555	-0.032204	-0.003958	-0.008790	-
0.012730	0.010651	-0.010879	0.014963	0.030384	-0.044519	
y13	0.145147	-0.048739	-0.018068	-0.078880	-0.013027	-
0.040887	-0.015403	-0.047430	-0.017678	0.003661	0.100250	
0.045252	0.038745	0.106021	0.050553	-0.016555	0.046236	
0.023971	-0.075342	-0.036256	0.080295	0.007323	-0.080477	-
0.150871	0.027986	0.041002	-0.004665	0.006331	-0.006674	
0.043718	-0.016163	-0.027383	0.065190	0.033311	-0.003197	
z13	-0.016910	0.111086	-0.059489	-0.044080	-0.045691	
0.062834	-0.023049	0.024495	-0.062554	0.015585	-0.029796	
0.046351	0.043761	-0.001470	-0.007168	-0.002891	-0.042087	-
0.108034	-0.061768	0.082193	0.055534	0.025647	-0.055142	
0.020815	0.025344	-0.019121	0.039151	-0.023849	-0.038251	
0.030364	-0.008771	-0.008161	-0.002479	-0.058555	-0.011914	
x14	-0.027870	0.070495	-0.091960	-0.042258	-0.050130	
0.043465	0.094799	0.010964	-0.016330	0.021503	0.039982	-
0.048846	-0.031295	-0.057775	0.092789	0.018480	0.026374	
0.025009	-0.022474	-0.019941	-0.057945	-0.015962	0.023593	-
0.071424	-0.034598	0.051658	-0.030395	-0.029062	-0.028205	-
0.038443	-0.045415	0.016066	-0.029075	-0.008308	-0.014967	
y14	0.147164	-0.034367	-0.028256	-0.085137	-0.038203	-
0.037602	-0.017174	-0.055889	-0.013723	0.015704	0.102487	
0.036633	0.034885	0.075484	0.032609	-0.004384	0.046298	
0.020568	-0.081159	-0.039188	0.083939	0.003516	-0.085112	-
0.136725	0.037315	0.000916	-0.026243	0.002008	-0.025655	
0.070585	-0.004178	-0.026187	0.081103	0.005876	0.010644	

z14	-0.018505	0.098331	-0.058368	-0.031200	-0.033316	
0.070186	-0.018520	0.031627	-0.057352	0.016050	-0.039598	
0.034868	0.044574	-0.024846	-0.024785	0.022368	-0.038553	-
0.123105	-0.052854	0.062501	0.057489	0.027532	-0.065240	
0.027254	0.016623	0.001089	0.032451	-0.018456	-0.012743	
0.029850	0.015677	-0.008586	0.002942	-0.057144	-0.049317	
x15	-0.027047	0.067970	-0.091221	-0.048376	-0.048244	
0.037811	0.090424	0.016018	-0.003636	0.034470	0.046885	-
0.049872	-0.036677	-0.062380	0.083709	0.020408	0.039460	
0.025316	-0.024982	-0.027046	-0.038394	-0.030675	0.011114	-
0.071518	0.003142	0.054364	-0.023420	-0.054673	-0.024295	-
0.023885	-0.052430	0.016296	-0.044751	-0.033410	-0.000598	
y15	0.147416	-0.021568	-0.037009	-0.089726	-0.057129	-
0.033686	-0.019618	-0.064696	-0.009575	0.029071	0.099199	
0.033638	0.029851	0.037174	0.009668	0.010484	0.044815	
0.016219	-0.075900	-0.040960	0.082537	-0.002389	-0.083266	-
0.103883	0.038657	-0.027531	-0.035232	0.001956	-0.031323	
0.082640	0.017737	-0.009813	0.089118	-0.018468	0.012918	
z15	-0.020609	0.084618	-0.055777	-0.018338	-0.017880	
0.077638	-0.013542	0.040721	-0.050354	0.012844	-0.043966	
0.018174	0.043108	-0.036987	-0.037843	0.047339	-0.031837	-
0.129723	-0.036092	0.057349	0.049758	0.028867	-0.069313	
0.024356	-0.002443	0.014111	0.036594	-0.020269	0.007655	
0.011792	0.001755	-0.003782	-0.001685	-0.048225	-0.049476	
x16	-0.024549	0.066224	-0.092245	-0.054057	-0.048483	
0.032132	0.081850	0.024499	0.007737	0.045735	0.058989	-
0.055774	-0.042473	-0.066162	0.065802	0.030016	0.062897	
0.040652	-0.012135	-0.031299	-0.026253	-0.033649	0.005498	-
0.074153	0.014455	0.058449	-0.023044	-0.072936	-0.030492	-
0.027515	-0.086003	0.025043	-0.022224	-0.058518	0.035571	
y16	0.146034	-0.010849	-0.044145	-0.090957	-0.069560	-
0.029600	-0.021224	-0.075817	-0.005768	0.042240	0.090547	
0.032333	0.019723	-0.008402	-0.018467	0.031304	0.038715	
0.010667	-0.057659	-0.033605	0.073317	-0.011232	-0.074642	-
0.056893	0.033218	-0.051690	-0.022366	0.011477	-0.035493	
0.067689	0.041377	0.006317	0.073318	-0.032809	-0.003642	
z16	-0.024212	0.067708	-0.049570	-0.006608	0.002019	
0.085340	-0.005313	0.049138	-0.036449	0.011179	-0.048222	
0.012400	0.043220	-0.046474	-0.037420	0.056938	-0.024861	-
0.136870	-0.031393	0.046107	0.034813	0.027325	-0.071432	
0.026430	0.011936	0.024122	0.041944	-0.059510	0.037106	-
0.014203	0.009435	-0.006182	-0.026677	-0.059785	-0.041091	
x17	-0.023104	0.063941	-0.089000	-0.061802	-0.045758	
0.024841	0.077124	0.030204	0.021343	0.058731	0.057309	-
0.054516	-0.051745	-0.073613	0.047730	0.030569	0.061441	
0.046520	-0.006375	-0.031444	-0.000332	-0.027758	-0.013399	-
0.056236	0.044228	0.047990	0.010855	-0.082186	-0.006103	-
0.050314	-0.120667	0.029165	-0.038923	-0.050834	0.061762	
y17	0.142886	-0.002854	-0.050049	-0.089848	-0.077090	-
0.024954	-0.024048	-0.085925	0.000685	0.051346	0.077830	
0.033352	0.012236	-0.064710	-0.048687	0.051512	0.034247	
0.003685	-0.024189	-0.022839	0.053773	-0.020309	-0.052532	
0.006673	0.018778	-0.078315	-0.003216	0.007042	-0.027450	
0.038425	0.075617	0.037049	0.058454	-0.062327	-0.022325	
z17	-0.026892	0.049672	-0.043407	0.006689	0.021359	
0.092054	-0.000426	0.058572	-0.024404	0.004632	-0.042109	
0.007609	0.034621	-0.041917	-0.035529	0.068346	-0.015282	-
0.126402	-0.020047	0.043489	0.017613	0.016802	-0.057934	
0.012384	0.014048	0.047264	0.036598	-0.058346	0.047175	-
0.027569	0.019191	-0.018253	-0.029286	-0.052132	-0.046881	

x18	-0.020474	0.057484	-0.084054	-0.064820	-0.047301	
0.011677	0.071839	0.034179	0.031535	0.057688	0.065142	-
0.044639	-0.062985	-0.092879	0.038447	0.027860	0.045061	
0.040311	0.017509	-0.012801	0.019444	-0.049592	-0.026050	-
0.046091	0.045987	0.018830	0.025377	-0.102622	0.007023	-
0.045151	-0.113417	-0.007822	-0.026505	-0.004321	0.093650	
y18	0.138071	0.003334	-0.054641	-0.086789	-0.075549	-
0.016958	-0.026525	-0.095116	0.010694	0.067010	0.058405	
0.039011	0.002947	-0.124858	-0.090167	0.071438	0.037724	
0.005379	0.014526	-0.010161	0.029688	-0.025935	-0.025434	
0.087565	-0.002328	-0.090556	0.042544	-0.004094	-0.013454	-
0.025350	0.095759	0.094866	0.013270	-0.090685	-0.058524	
z18	-0.029124	0.031576	-0.035566	0.018905	0.042812	
0.096603	0.006706	0.064140	-0.011391	-0.000936	-0.045439	-
0.004104	0.023633	-0.026831	-0.033252	0.083167	-0.018825	-
0.092889	-0.003574	0.042886	0.028157	0.026448	-0.021977	
0.000796	0.055828	0.076845	0.036756	0.026770	0.063228	-
0.043968	0.007047	-0.050091	-0.021997	-0.049714	-0.061712	
x19	-0.017573	0.049612	-0.078880	-0.069978	-0.042003	
0.000260	0.064277	0.032820	0.051066	0.066422	0.056725	-
0.032907	-0.063881	-0.119814	0.019025	0.024124	0.040194	
0.065275	0.036924	-0.006398	0.037774	-0.052931	0.004495	
0.000762	0.079443	0.001310	0.084494	-0.097660	0.044560	-
0.076551	-0.066393	-0.007433	-0.009727	0.029988	0.031718	
y19	0.132190	0.007832	-0.056775	-0.080399	-0.070297	-
0.010019	-0.027107	-0.106530	0.018936	0.078108	0.038719	
0.046305	-0.013654	-0.192000	-0.128191	0.101247	0.034530	-
0.001135	0.062839	0.013652	-0.003056	-0.035371	0.005566	
0.177734	-0.029059	-0.102109	0.083291	-0.011142	-0.001998	-
0.097539	0.116350	0.128326	-0.042943	-0.134688	-0.080060	
z19	-0.031609	0.012577	-0.024967	0.030730	0.065531	
0.102482	0.016170	0.073033	-0.001291	-0.007623	-0.033338	-
0.005216	0.008037	-0.004626	-0.010080	0.090843	-0.015087	-
0.073254	-0.003980	0.039644	0.027624	0.028469	-0.027680	-
0.031147	0.077869	0.092932	0.009618	0.044742	0.071913	-
0.053765	-0.023927	-0.099885	-0.053287	-0.034306	0.001346	
x20	-0.011711	0.048085	-0.070610	-0.060674	-0.043765	
0.006260	0.053898	0.040285	0.061600	0.075351	0.061218	-
0.015046	-0.060972	-0.086718	0.004731	-0.002893	0.025492	
0.061870	0.038342	-0.005293	0.028331	-0.011075	0.006050	
0.052934	0.080458	-0.011916	0.049159	-0.062325	0.093732	-
0.057927	-0.086071	0.041370	-0.021248	0.015336	-0.016879	
y20	0.125362	0.017207	-0.051449	-0.042043	-0.079236	
0.017482	-0.034643	-0.081848	0.007506	0.057311	0.017067	
0.009493	-0.019830	-0.135473	-0.090318	0.075851	0.001920	-
0.012030	0.088322	0.019365	-0.018624	0.000388	0.016961	
0.107847	-0.007539	-0.048526	0.036345	0.041922	0.008558	-
0.030402	0.070932	0.037299	-0.037827	-0.052312	-0.026427	
z20	-0.032649	-0.006910	-0.019734	0.029399	0.096186	
0.104246	0.029278	0.070268	0.004045	-0.006534	-0.000532	-
0.016809	0.018242	0.024436	0.001275	0.079199	0.006285	-
0.044057	0.025542	0.011912	0.021873	0.008067	-0.040313	-
0.050561	0.042939	0.080802	0.037155	0.024192	0.033128	-
0.046612	-0.009673	-0.056906	-0.005892	0.002008	0.009035	
x21	-0.008090	0.048206	-0.064993	-0.048269	-0.045816	
0.004342	0.042427	0.053100	0.066086	0.071257	0.070178	-
0.017604	-0.068452	-0.043318	-0.005120	-0.030069	0.018933	
0.051325	0.050467	0.004871	0.021413	-0.007501	0.020674	
0.076569	0.046651	0.005805	0.025470	-0.039352	0.101640	
0.003828	-0.048678	0.043558	-0.021043	0.024146	-0.027388	

y21	0.118552	0.023223	-0.044915	-0.008498	-0.084801	
0.044943	-0.038850	-0.061494	0.004416	0.043774	-0.006017	-
0.011184	-0.013239	-0.090309	-0.059846	0.050890	-0.027651	-
0.017675	0.103553	0.023880	-0.019288	0.038013	0.004588	
0.055982	0.019297	-0.039631	-0.010112	0.068760	0.021514	
0.001303	-0.015009	-0.017362	-0.032056	0.018118	0.032151	
z21	-0.031277	-0.025820	-0.013113	0.027063	0.118072	
0.106508	0.041817	0.063326	0.014632	0.000078	0.027787	-
0.011248	0.032057	0.035077	0.005747	0.067617	0.011118	-
0.021512	0.042100	-0.006025	0.027262	0.001959	-0.067808	-
0.045198	0.022470	0.026066	0.028279	0.019140	0.011026	-
0.051978	-0.041025	-0.020599	0.042536	0.005813	0.038791	
x22	-0.002978	0.048174	-0.058271	-0.033952	-0.048323	
0.002456	0.028158	0.063768	0.080032	0.060401	0.071316	-
0.019232	-0.061848	-0.004563	-0.012271	-0.034734	0.018170	
0.039863	0.064643	0.017752	0.009521	0.007626	0.008163	
0.097662	0.026961	0.002881	-0.018199	-0.039982	0.077672	
0.051296	-0.048829	0.025695	-0.021678	0.010439	0.004959	
y22	0.110979	0.027128	-0.040316	0.020240	-0.086132	
0.068383	-0.041768	-0.041526	-0.005868	0.037825	-0.025781	-
0.033920	-0.013701	-0.050066	-0.034840	0.016888	-0.052649	-
0.024915	0.109784	0.019694	-0.018152	0.059609	0.006610	
0.011439	0.023451	-0.016876	-0.021854	0.103089	0.049903	
0.015281	-0.055785	-0.031236	-0.028900	0.079512	0.035276	
z22	-0.030897	-0.041530	-0.010066	0.023227	0.135896	
0.106805	0.049437	0.057820	0.016608	0.010642	0.053090	-
0.007022	0.033972	0.045811	0.003530	0.042451	0.017483	-
0.000003	0.055788	-0.026298	0.029061	-0.008159	-0.068941	-
0.035990	-0.001686	0.011906	0.032673	0.032281	0.011426	-
0.053078	-0.026304	0.038831	0.069748	0.022230	0.018643	
x23	0.001132	0.047017	-0.053632	-0.013767	-0.052171	-
0.004373	0.016890	0.076335	0.091074	0.054490	0.068697	-
0.018361	-0.059653	0.023212	-0.021703	-0.050066	0.020278	
0.011967	0.065038	0.040889	0.037717	0.000314	-0.024353	
0.102066	0.001046	-0.003314	-0.031045	-0.054111	0.044686	
0.099949	-0.043091	-0.041689	-0.039533	0.048144	0.057557	
y23	0.103229	0.029134	-0.034422	0.043032	-0.082991	
0.089935	-0.043682	-0.025689	-0.014439	0.029027	-0.041704	-
0.060805	-0.011334	-0.015416	-0.007097	-0.002152	-0.071447	-
0.026489	0.116584	0.005194	-0.032972	0.064313	0.019084	-
0.016264	0.019402	0.012423	-0.036125	0.128717	0.058401	
0.020481	-0.064098	-0.020912	-0.015343	0.082737	0.004452	
z23	-0.030913	-0.055044	-0.007491	0.018276	0.149108	
0.103382	0.054693	0.047756	0.022106	0.014759	0.079949	-
0.002319	0.028208	0.048161	0.002674	0.030748	0.019822	
0.014264	0.062960	-0.041963	0.034818	-0.029408	-0.051571	-
0.016146	-0.016778	0.012014	0.026708	0.039803	0.003791	-
0.025474	0.007057	0.069672	0.081644	0.015126	-0.006250	
x24	0.006161	0.046248	-0.045019	0.002194	-0.056146	-
0.002758	-0.005153	0.090299	0.096863	0.050378	0.044818	-
0.024887	-0.045532	0.069862	-0.017669	-0.055916	0.009900	
0.009643	0.053163	0.041056	-0.009539	0.033778	-0.039206	
0.095475	0.000811	-0.024489	-0.040786	-0.032597	0.057458	
0.059469	-0.049492	0.001652	-0.008853	0.049805	0.058763	
y24	0.095072	0.029552	-0.030254	0.064521	-0.075601	
0.105495	-0.039458	-0.015487	-0.019506	0.026182	-0.049479	-
0.083046	-0.008149	0.000795	0.011433	-0.012512	-0.065780	-
0.027362	0.118856	-0.006649	-0.023359	0.051495	0.015771	-
0.026669	0.004927	0.043388	-0.058983	0.132505	0.034014	
0.031425	-0.080410	-0.036047	-0.015761	0.067398	0.009282	

z24	-0.029291	-0.064327	-0.007024	0.012319	0.154089	
0.100061	0.056455	0.034284	0.026501	0.026376	0.099242	
0.006609	0.021379	0.046871	-0.001803	0.021006	0.031675	
0.039302	0.063993	-0.051367	0.021587	-0.026974	-0.036945	
0.008654	-0.010444	0.002774	-0.005533	0.053185	0.005028	-
0.018563	0.015297	0.098981	0.084436	0.005694	-0.010868	
x25	0.010793	0.044569	-0.039156	0.023083	-0.058393	-
0.007017	-0.026470	0.101822	0.104749	0.047578	0.016832	-
0.033022	-0.032649	0.101877	-0.009373	-0.063173	0.009660	
0.001327	0.044752	0.024051	-0.010143	0.046752	-0.047418	
0.099656	-0.024683	-0.031238	-0.057454	-0.019071	0.042609	
0.034869	-0.026959	-0.001577	0.017283	0.040553	0.035328	
y25	0.084935	0.029628	-0.024544	0.078670	-0.065577	
0.119556	-0.032037	-0.006565	-0.025048	0.020161	-0.055336	-
0.109377	0.003992	0.015714	0.027464	-0.012587	-0.058524	-
0.027066	0.109314	0.003208	-0.031186	0.020881	-0.005973	-
0.049740	0.006909	0.053379	-0.047673	0.110773	-0.000604	
0.021122	-0.105712	-0.026877	-0.010887	0.042354	0.047675	
z25	-0.029487	-0.069197	-0.008689	0.003811	0.150467	
0.093196	0.055823	0.018389	0.030792	0.032248	0.118900	
0.013271	0.012528	0.043916	-0.011138	0.013928	0.031626	
0.050412	0.056586	-0.038569	0.011925	-0.028479	-0.031460	
0.015097	-0.005990	-0.008376	-0.026482	0.052887	0.000602	
0.002806	0.001725	0.100982	0.075544	0.002401	0.019198	
x26	0.014812	0.043710	-0.033647	0.045923	-0.057326	-
0.012810	-0.046180	0.111895	0.107430	0.045792	-0.015602	-
0.055617	-0.016531	0.131131	-0.002332	-0.052148	0.020947	-
0.010291	0.030894	0.024236	-0.012325	0.036565	-0.068626	
0.091876	-0.047259	-0.008403	-0.045835	-0.004610	-0.015605	
0.008387	-0.024177	0.001577	0.042560	0.023122	0.032524	
y26	0.076365	0.023308	-0.017637	0.090479	-0.055595	
0.131312	-0.025737	0.003540	-0.021783	0.011421	-0.057470	-
0.117960	0.021579	0.021995	0.043345	-0.027430	-0.056821	-
0.027211	0.096748	-0.015018	-0.034370	-0.006871	-0.024743	-
0.050430	-0.011736	0.013023	-0.060658	0.067091	-0.011295	-
0.013620	-0.078887	-0.010825	0.001055	0.001698	0.052563	
z26	-0.026802	-0.075581	-0.010681	-0.002919	0.139627	
0.084463	0.050723	0.001458	0.039870	0.035694	0.135632	
0.032854	-0.000299	0.029739	-0.016404	-0.005860	0.030753	
0.053405	0.047048	-0.034802	0.004003	-0.027064	-0.010452	
0.032752	-0.001490	-0.040127	-0.072499	0.042200	0.016703	
0.032758	0.037343	0.075095	0.060915	0.006088	0.032793	
x27	0.018381	0.041740	-0.026745	0.070141	-0.054964	-
0.019867	-0.064162	0.123004	0.110632	0.042573	-0.054699	-
0.076688	0.007074	0.152018	0.012402	-0.041334	0.031545	-
0.016339	0.004111	0.018439	-0.012642	0.014817	-0.096793	
0.087265	-0.058258	0.003053	-0.023741	-0.015414	-0.084721	-
0.049223	0.011155	-0.016630	0.056550	0.003103	0.007941	
y27	0.067389	0.016286	-0.013136	0.098809	-0.042512	
0.140367	-0.022462	0.009288	-0.021734	0.004477	-0.058652	-
0.136303	0.033473	0.026575	0.054892	-0.029319	-0.041434	-
0.034174	0.090209	-0.035874	-0.041659	-0.037517	-0.036893	-
0.053012	-0.062466	-0.018735	-0.066002	0.040043	-0.026845	-
0.038861	-0.063249	0.045799	0.013557	-0.057030	0.066196	
z27	-0.024018	-0.077125	-0.015823	-0.010487	0.121535	
0.073825	0.041467	-0.015146	0.047350	0.039540	0.153408	
0.047924	-0.025386	0.019089	-0.030938	-0.023927	0.025438	
0.046699	0.039296	-0.015109	-0.006603	-0.027313	0.011062	
0.045495	-0.006444	-0.064521	-0.119621	0.035887	0.022208	
0.085672	0.047739	0.038629	0.036777	-0.001134	0.075350	

x28	0.020923	0.038999	-0.018820	0.095307	-0.049539	-
0.026388	-0.085303	0.136100	0.114685	0.038525	-0.100757	-
0.109877	0.041217	0.169430	0.034008	-0.021990	0.053177	-
0.023709	-0.026616	0.000026	-0.025638	-0.009289	-0.133593	
0.077750	-0.082410	-0.007336	0.002222	-0.048104	-0.156365	-
0.125500	0.046963	-0.006277	0.073917	-0.036834	-0.005339	
y28	0.058508	0.009126	-0.010896	0.102082	-0.032092	
0.144023	-0.016243	0.010367	-0.024152	0.005674	-0.055169	-
0.127997	0.030484	0.026040	0.052882	-0.036961	-0.033351	-
0.037156	0.072714	-0.057921	-0.038612	-0.074334	-0.043666	-
0.038898	-0.101616	-0.000431	-0.070346	0.037927	-0.056506	-
0.076259	-0.019509	0.076843	0.051181	-0.103319	0.032896	
z28	-0.020907	-0.074628	-0.022918	-0.020117	0.095495	
0.060430	0.029205	-0.033280	0.053952	0.048967	0.168735	
0.071265	-0.058288	0.004706	-0.053198	-0.044431	0.018908	
0.039484	0.025816	0.011695	-0.021779	-0.027126	0.032636	
0.066516	-0.003983	-0.077273	-0.179258	0.034050	0.032934	
0.142729	0.063797	-0.008979	0.003960	-0.000748	0.114203	
x29	0.011979	0.035390	-0.003896	0.119831	-0.019730	-
0.027779	-0.104811	0.114729	0.109696	0.013171	-0.070526	-
0.090758	0.061284	0.124131	0.044258	-0.003646	0.052990	-
0.018312	-0.023748	0.037678	-0.021143	0.004332	-0.057257	
0.025858	-0.026603	-0.045608	0.018746	-0.054577	-0.080924	-
0.059131	0.024323	0.004288	0.045913	-0.049443	0.034113	
y29	0.042469	-0.002870	-0.000532	0.095351	-0.011745	
0.147526	-0.000418	-0.017773	-0.023874	0.012860	-0.013214	-
0.088196	0.006829	-0.017425	0.051082	-0.035300	0.017264	-
0.007719	0.042441	-0.073705	-0.059412	-0.058439	0.039013	-
0.009917	-0.016394	0.041984	-0.051226	-0.027256	0.007756	-
0.013505	0.046390	-0.008106	-0.006489	-0.112725	0.034706	
z29	-0.014251	-0.065479	-0.028180	-0.015952	0.042109	
0.036022	-0.002124	-0.032228	0.059156	0.054645	0.115804	
0.065014	-0.070527	0.016111	-0.060206	-0.058469	0.005106	
0.043363	0.028272	0.014486	-0.015577	0.015838	0.064310	
0.005028	0.008090	-0.062620	-0.084398	0.000759	-0.031225	
0.085131	0.076173	-0.062882	-0.020357	-0.041194	0.047713	
x30	0.002341	0.031748	0.011273	0.139691	0.013014	-
0.025917	-0.114975	0.087660	0.100153	-0.001097	-0.038945	-
0.068036	0.077488	0.069554	0.064050	0.025269	0.070752	-
0.008884	-0.028177	0.051594	-0.018731	0.006664	0.019447	-
0.001590	0.047675	-0.051453	0.016533	-0.059946	-0.012067	
0.003574	0.057291	-0.030955	0.012335	-0.066010	0.024192	
y30	0.032358	-0.016766	0.006629	0.080718	-0.007831	
0.148202	0.007538	-0.029543	-0.018068	0.002153	0.009237	-
0.043644	-0.006830	-0.036682	0.034434	-0.055309	0.024541	
0.004310	-0.000152	-0.074636	-0.059885	-0.035152	0.062576	-
0.005639	-0.006592	0.031722	-0.004530	-0.083893	0.055796	-
0.029990	0.042730	0.018954	-0.033677	-0.052617	0.004646	
z30	-0.007981	-0.055922	-0.032920	-0.011912	-0.007693	
0.015889	-0.032450	-0.025248	0.063431	0.053866	0.074364	
0.057836	-0.075113	0.030843	-0.076854	-0.068708	-0.013236	
0.029962	0.034493	0.026240	-0.007334	0.043877	0.058640	-
0.057659	-0.018526	-0.065196	-0.007148	-0.029169	-0.091470	
0.031460	0.043723	-0.083555	-0.034724	-0.050398	0.003722	
x31	-0.007587	0.027678	0.026562	0.151689	0.040974	-
0.018882	-0.121746	0.063829	0.089870	-0.017697	-0.008113	-
0.041011	0.089076	0.018180	0.077135	0.042061	0.072764	-
0.000378	-0.038443	0.058696	-0.011939	0.004794	0.073691	-
0.017594	0.105274	-0.059594	0.017285	-0.067989	0.054401	
0.041886	0.079811	-0.035455	-0.011649	-0.054708	-0.002795	

y31	0.027015	-0.023816	0.004540	0.065185	-0.013122	
0.143364	0.020449	-0.036645	-0.017263	0.008150	0.013614	-
0.025368	-0.023593	-0.036424	0.016180	-0.073961	0.048626	
0.013352	-0.012813	-0.062766	-0.062761	-0.005766	0.095708	
0.020283	0.009118	0.046203	0.021179	-0.100198	0.045542	-
0.011982	0.023504	0.013217	-0.036296	-0.115312	0.007569	
z31	-0.003673	-0.045549	-0.035211	-0.006630	-0.046423	-
0.001089	-0.057101	-0.018504	0.063987	0.052794	0.037261	
0.048640	-0.081954	0.038618	-0.086248	-0.071026	-0.021813	
0.028658	0.036219	0.033897	0.001261	0.057955	0.053021	-
0.091133	-0.023861	-0.048677	0.041682	-0.040782	-0.129056	
0.004996	0.036564	-0.088907	-0.054303	-0.050366	-0.027052	
x32	-0.016244	0.024479	0.039804	0.156979	0.061636	-
0.007239	-0.124575	0.041514	0.075547	-0.029457	0.022954	-
0.017934	0.090314	-0.023205	0.081956	0.049384	0.068163	
0.002957	-0.044580	0.065775	-0.000071	0.000402	0.107813	-
0.026096	0.131467	-0.062281	0.015849	-0.066702	0.084257	
0.058651	0.073800	-0.035793	-0.016571	-0.049373	-0.015073	
y32	0.023046	-0.032566	0.002329	0.045681	-0.026186	
0.132446	0.029623	-0.035171	-0.015347	0.011487	0.000473	
0.005757	-0.021020	-0.040334	0.007741	-0.087992	0.055212	
0.010771	-0.044732	-0.078253	-0.070943	0.010951	0.086777	
0.021497	0.009513	0.036157	0.072756	-0.141905	0.055631	-
0.016181	0.012288	0.011092	-0.093236	-0.044801	-0.009618	
z32	-0.001167	-0.035296	-0.034511	-0.001771	-0.075729	-
0.011010	-0.079406	-0.011633	0.062961	0.042227	0.006713	
0.043026	-0.079845	0.035174	-0.087173	-0.068638	-0.026556	
0.028190	0.033242	0.037866	0.015196	0.064410	0.046566	-
0.116368	-0.036939	-0.039334	0.069157	-0.040772	-0.139969	-
0.024626	0.039674	-0.067085	-0.059356	-0.049290	-0.050625	
x33	-0.024159	0.021021	0.051579	0.154798	0.073909	
0.007963	-0.122134	0.019658	0.057875	-0.035853	0.049702	
0.005602	0.083883	-0.060373	0.081837	0.042519	0.051685	
0.002128	-0.053825	0.063569	0.015171	-0.007121	0.121009	-
0.027767	0.138893	-0.057003	0.022417	-0.065201	0.096156	
0.057834	0.057862	-0.030406	-0.024097	-0.038323	-0.013964	
y33	0.023403	-0.036143	-0.002560	0.027138	-0.042527	
0.124568	0.045236	-0.026535	-0.014821	0.013818	-0.023906	
0.009426	-0.015919	-0.026923	-0.005816	-0.097904	0.051652	
0.006194	-0.063783	-0.071049	-0.064773	0.036470	0.074397	
0.021641	0.030913	0.056274	0.081038	-0.144457	0.038733	
0.001303	0.014395	-0.011243	-0.068934	-0.014141	-0.044508	
z33	-0.000561	-0.025061	-0.031607	0.004360	-0.094212	-
0.018083	-0.098592	-0.003059	0.061445	0.031325	-0.018162	
0.038860	-0.080356	0.022859	-0.080361	-0.058900	-0.020217	
0.039033	0.023945	0.039250	0.027928	0.061267	0.051869	-
0.120479	-0.042558	-0.024759	0.077157	-0.027161	-0.120413	-
0.036139	0.038998	-0.025751	-0.073935	-0.039273	-0.048252	
x34	-0.031528	0.017716	0.062557	0.143239	0.078390	
0.027105	-0.112557	-0.002279	0.036527	-0.037586	0.072307	
0.026240	0.066571	-0.092212	0.075555	0.024724	0.027965	-
0.001568	-0.061626	0.062667	0.035816	-0.021663	0.104358	-
0.012017	0.104043	-0.034187	0.016583	-0.060543	0.072934	
0.049696	0.031593	-0.013741	-0.023610	0.011501	0.006046	
y34	0.025293	-0.036805	-0.013394	0.009267	-0.052861	
0.110392	0.057912	-0.005568	-0.007733	0.017852	-0.053688	
0.025150	-0.023431	-0.012016	-0.011639	-0.093731	0.073534	
0.029564	-0.098852	-0.049897	-0.038128	0.036110	0.047693	
0.038005	0.000174	0.022293	0.026666	-0.101288	0.005300	
0.000991	0.037707	0.005530	-0.038017	0.028832	-0.053959	

z34	-0.000603	-0.013652	-0.025069	0.009313	-0.105670	-
0.020347	-0.112780	0.006989	0.057825	0.015976	-0.039602	
0.033285	-0.075695	0.007116	-0.069240	-0.042960	-0.014590	
0.051074	0.013385	0.040857	0.036804	0.054171	0.062636	-
0.116114	-0.026083	0.012820	0.079324	-0.013707	-0.084038	-
0.043950	0.047542	0.005232	-0.054277	-0.030811	-0.053194	
x35	-0.038048	0.014423	0.072962	0.123066	0.074677	
0.050357	-0.097248	-0.022700	0.008805	-0.031220	0.092129	
0.044687	0.039866	-0.115896	0.068138	0.002450	-0.010913	-
0.012416	-0.077966	0.048492	0.064504	-0.037027	0.070471	
0.000740	0.036070	-0.003077	0.010170	-0.053757	0.023593	
0.002762	-0.003188	0.002988	-0.004672	0.064352	0.029563	
y35	0.028242	-0.037454	-0.024818	-0.006930	-0.068151	
0.094751	0.071267	0.018475	-0.003458	0.016483	-0.090610	
0.035129	-0.001169	0.002941	-0.021684	-0.082332	0.064190	
0.033028	-0.130423	-0.043870	-0.033243	0.051735	0.033757	
0.032344	-0.003811	0.027725	0.032260	-0.078225	0.025977	-
0.013101	0.033158	0.016098	-0.008873	0.087953	-0.019891	
z35	-0.001518	-0.001237	-0.015789	0.013320	-0.108385	-
0.015990	-0.124503	0.022098	0.053654	-0.002936	-0.057442	
0.024836	-0.070661	-0.015429	-0.049306	-0.019495	0.002910	
0.066045	0.000015	0.049613	0.056554	0.023133	0.066645	-
0.093381	-0.023911	0.032477	0.048282	0.021449	-0.042593	-
0.034317	0.052429	0.061212	-0.023997	-0.001718	-0.060462	
x36	-0.044370	0.010494	0.082813	0.094606	0.065848	
0.075204	-0.073307	-0.042463	-0.021051	-0.020252	0.107642	
0.060865	0.009559	-0.127044	0.050435	-0.027674	-0.053631	-
0.022903	-0.090808	0.025450	0.088921	-0.053435	0.014489	
0.029154	-0.052714	0.037900	-0.003229	-0.065580	-0.054029	-
0.054678	-0.035408	0.027400	0.017708	0.113862	0.066478	
y36	0.032962	-0.035769	-0.040413	-0.018231	-0.079149	
0.071095	0.087246	0.047315	0.008738	0.012751	-0.137607	
0.037498	0.033917	0.036887	-0.043006	-0.063774	0.066968	
0.053211	-0.140930	-0.032885	-0.044954	0.066521	0.009641	
0.036881	0.026758	0.020202	0.010147	-0.065169	0.034866	
0.005427	0.044404	0.021645	0.009939	0.084505	0.020517	
z36	-0.003025	0.012554	-0.003906	0.015978	-0.101196	-
0.006571	-0.131092	0.037139	0.047002	-0.023967	-0.071629	
0.016962	-0.064942	-0.044913	-0.022401	0.013189	0.025575	
0.084867	-0.015606	0.061272	0.081820	-0.021125	0.071679	-
0.049960	-0.024491	0.055974	0.005549	0.085214	0.006600	-
0.019499	0.051577	0.113802	0.025074	0.045455	-0.066509	
x37	-0.050600	0.005623	0.091708	0.060313	0.054511	
0.099073	-0.048918	-0.061504	-0.047247	-0.010922	0.125625	
0.076625	-0.016847	-0.120408	0.032584	-0.064345	-0.093895	-
0.023291	-0.085333	0.006364	0.127680	-0.079687	-0.038237	
0.061576	-0.140885	0.053598	-0.014388	-0.057877	-0.138973	-
0.105379	-0.084235	0.041305	0.035379	0.158087	0.063499	
y37	0.039473	-0.031922	-0.060304	-0.022532	-0.083256	
0.037694	0.092061	0.079402	0.031247	0.003402	-0.182805	
0.034315	0.084230	0.088277	-0.046722	-0.040715	0.085067	
0.094498	-0.129827	-0.010481	-0.032333	0.074405	0.007618	
0.030297	0.094226	-0.054508	-0.031803	-0.003583	0.065157	
0.042194	0.031266	0.003141	0.026036	0.034091	-0.008090	
z37	-0.006391	0.026361	0.009918	0.017251	-0.082603	
0.007197	-0.126905	0.048262	0.035524	-0.047433	-0.080352	
0.015561	-0.052862	-0.083630	0.002171	0.054267	0.045848	
0.099124	-0.030281	0.060822	0.086088	-0.061655	0.059078	
0.026581	-0.027033	0.105719	-0.046333	0.138028	0.061037	
0.006702	0.052224	0.171502	0.060036	0.096839	-0.032439	

x38	-0.045661	0.004638	0.052746	0.029126	0.031396	
0.095722	0.004903	-0.045610	-0.037969	-0.015019	0.041975	
0.101857	-0.013736	-0.064389	0.048007	-0.081202	-0.068398	
0.034208	-0.087620	0.022620	0.122406	-0.069143	-0.039632	
0.076200	-0.100502	0.037267	-0.047389	-0.040763	-0.136610	-
0.033689	-0.058359	-0.051431	0.012975	0.046620	0.033256	
y38	0.041556	-0.025456	-0.050874	-0.021223	-0.080826	
0.006075	0.075478	0.095245	0.039439	0.011085	-0.142601	
0.016155	0.068654	0.108067	-0.065043	0.009084	0.049262	
0.079258	-0.121687	-0.011204	0.009790	0.027300	0.027666	
0.027467	0.047649	-0.050947	-0.012410	-0.004153	0.024433	
0.062880	-0.031711	-0.017357	0.030080	0.020366	0.020910	
z38	-0.012881	0.031330	-0.001149	0.004601	-0.079732	
0.008454	-0.114395	0.064448	0.016777	-0.053418	-0.069005	
0.040399	-0.011281	-0.086750	-0.010599	0.029831	0.013196	
0.062788	-0.007089	0.044931	0.071750	-0.060378	0.024131	-
0.025022	-0.036048	0.071360	-0.058069	0.097191	0.067261	-
0.005159	0.003054	0.098910	0.061784	0.081803	0.012779	
x39	-0.041061	0.002354	0.024793	-0.000035	0.013643	
0.090840	0.034929	-0.021103	-0.019369	-0.024063	-0.021602	
0.133484	0.007072	-0.022111	0.048113	-0.105938	-0.066132	
0.071735	-0.071879	0.029423	0.128845	-0.055810	-0.036756	
0.091288	-0.063781	-0.032162	-0.064244	-0.013689	-0.087255	-
0.007457	-0.055409	-0.105932	0.022324	-0.006071	-0.031428	
y39	0.041930	-0.018616	-0.033015	-0.017423	-0.069532	-
0.022769	0.043555	0.100466	0.050374	0.019127	-0.085759	
0.001840	0.065244	0.106777	-0.088546	0.040255	0.010609	
0.054944	-0.106654	-0.017906	0.036866	-0.022362	0.049780	
0.022449	0.011989	-0.047571	0.004566	-0.016514	0.021461	
0.020604	-0.088554	0.000151	0.040559	0.020789	0.020081	
z39	-0.019091	0.033490	-0.012199	-0.007268	-0.072396	
0.010073	-0.093079	0.074198	0.000279	-0.056444	-0.061239	
0.070119	0.033969	-0.089148	-0.015705	0.009376	-0.010914	
0.026044	0.014479	0.012429	0.048553	-0.050651	-0.005419	-
0.047627	-0.031638	0.037384	-0.056842	0.044576	0.078929	
0.006431	-0.022079	0.018049	0.034290	0.075412	0.045780	
x40	-0.033473	0.002870	0.004402	-0.021128	0.006267	
0.074640	0.046914	-0.001063	-0.008031	-0.023390	-0.057553	
0.133667	0.003188	0.011880	0.043706	-0.109800	-0.071246	
0.084758	-0.058208	0.024510	0.083988	-0.059586	-0.032210	
0.069386	-0.061262	-0.046876	-0.081028	-0.002081	-0.037230	
0.008399	-0.072302	-0.078891	-0.018425	-0.047218	-0.078984	
y40	0.042469	-0.010103	-0.015568	-0.010329	-0.052367	-
0.050976	0.013045	0.092198	0.050189	0.034138	-0.026884	-
0.022951	0.047002	0.095413	-0.096391	0.077913	-0.016135	
0.029998	-0.102237	-0.031317	0.021313	-0.068158	0.068478	
0.005578	-0.019244	0.007054	0.016620	-0.026266	0.006816	-
0.007276	-0.120429	0.032180	0.006579	0.028430	0.018132	
z40	-0.023768	0.035415	-0.020375	-0.017816	-0.060811	
0.013108	-0.069895	0.073785	-0.016471	-0.050802	-0.054114	
0.093969	0.065162	-0.088410	-0.015714	0.010629	-0.017418	
0.005895	0.021591	-0.018786	0.018023	-0.040839	-0.022416	-
0.055848	-0.008737	0.040697	-0.047583	0.024560	0.070363	
0.004761	-0.013516	-0.077196	0.007541	0.056569	0.052937	
x41	-0.025830	0.004437	-0.008728	-0.034001	0.002650	
0.052639	0.054618	0.007667	0.006404	-0.010206	-0.054081	
0.115909	-0.002522	0.037200	0.032290	-0.097464	-0.071592	
0.104019	-0.049205	0.023857	0.067623	-0.076674	-0.018168	
0.063189	-0.021181	-0.025330	-0.057814	-0.023269	-0.039294	-
0.008798	-0.081209	-0.056734	-0.024341	-0.125798	-0.014278	

y41	0.040651	-0.002523	0.000336	-0.002846	-0.037865	-
0.071087	-0.011843	0.081340	0.051026	0.046040	0.026899	-
0.037967	0.040728	0.079948	-0.094176	0.093394	-0.038790	
0.013411	-0.095191	-0.038456	0.016094	-0.097645	0.073023	
0.002321	-0.024719	0.035803	0.033205	-0.042514	-0.016911	-
0.030854	-0.136394	0.047420	0.002171	0.029973	0.048129	
z41	-0.026581	0.035990	-0.025172	-0.027576	-0.048782	
0.016471	-0.051290	0.070343	-0.027196	-0.042161	-0.050266	
0.109321	0.082476	-0.081779	-0.014946	0.019386	-0.011289	-
0.009303	0.028740	-0.049304	-0.013209	-0.039476	-0.033309	-
0.062817	0.007266	0.052027	-0.039637	0.017918	0.050746	-
0.014303	-0.011405	-0.137813	-0.000292	0.044998	0.058961	
x42	-0.019171	0.004473	-0.016015	-0.038254	-0.002744	
0.026290	0.049897	0.024652	0.020158	-0.011596	-0.039259	
0.089709	-0.014252	0.046171	0.032029	-0.114624	-0.099400	
0.077455	-0.037031	0.008743	0.040751	-0.070820	-0.019773	
0.049891	0.005365	-0.070263	-0.059723	-0.038191	0.015127	-
0.021458	-0.062609	0.009386	-0.000159	-0.127983	-0.009767	
y42	0.037126	0.004419	0.014280	0.004692	-0.023752	-
0.083203	-0.034652	0.071227	0.046470	0.052558	0.067118	-
0.043070	0.038261	0.058063	-0.080431	0.086243	-0.058233	-
0.007247	-0.086932	-0.043159	-0.002219	-0.105534	0.059980	-
0.006633	-0.030826	0.043563	0.037943	-0.044083	-0.021055	-
0.035558	-0.134853	0.055104	-0.011870	0.054213	0.035479	
z42	-0.028159	0.034407	-0.026029	-0.038060	-0.036819	
0.021496	-0.036130	0.069781	-0.031387	-0.031376	-0.046644	
0.121974	0.097938	-0.070307	-0.015982	0.020472	-0.006605	-
0.024875	0.034396	-0.073430	-0.035041	-0.037546	-0.032033	-
0.069170	0.014228	0.040698	-0.032971	0.008817	0.042201	-
0.033947	-0.000572	-0.144831	-0.006203	0.032871	0.065849	
x43	-0.010991	0.005294	-0.019186	-0.040493	-0.003418	
0.002689	0.036212	0.043031	0.021533	-0.005482	-0.010407	
0.080546	-0.015600	0.035210	0.021159	-0.086198	-0.109139	
0.050807	-0.032928	0.003448	0.020546	-0.077382	-0.024546	
0.015196	0.001189	-0.054594	-0.011087	-0.053030	0.047595	-
0.018651	-0.025689	0.015086	-0.022524	-0.042447	-0.025509	
y43	0.032360	0.010922	0.026981	0.012458	-0.009011	-
0.087277	-0.057241	0.060777	0.036791	0.057446	0.097993	-
0.043829	0.041800	0.033138	-0.062182	0.059554	-0.069635	-
0.028491	-0.071879	-0.038622	-0.032096	-0.100585	0.034040	-
0.018214	-0.037974	0.047451	0.032869	-0.035330	-0.019751	-
0.030378	-0.127559	0.045556	-0.028283	0.048506	0.013919	
z43	-0.028339	0.032184	-0.025303	-0.046689	-0.023740	
0.025370	-0.024034	0.066761	-0.030687	-0.014345	-0.041210	
0.125895	0.102898	-0.055196	-0.016570	0.022331	0.002652	-
0.038737	0.039187	-0.091343	-0.053346	-0.031791	-0.019932	-
0.074648	0.011998	0.032782	-0.032223	0.010856	0.025069	-
0.049617	0.000800	-0.115615	0.001541	-0.000864	0.062705	
x44	-0.004032	0.006346	-0.019261	-0.038722	0.002580	-
0.022317	0.016500	0.062985	0.026755	0.009245	0.011775	
0.058762	-0.022377	0.021673	0.012563	-0.071305	-0.116460	
0.026129	-0.017642	-0.012945	0.016763	-0.062182	-0.018088	-
0.010153	0.014868	-0.056412	0.064984	-0.022029	0.056218	
0.002003	-0.010719	0.068702	-0.044542	-0.040792	0.002943	
y44	0.025968	0.017183	0.039169	0.020551	0.005941	-
0.086560	-0.079059	0.048265	0.021520	0.062015	0.125520	-
0.042124	0.047530	0.003995	-0.033117	0.010376	-0.080043	-
0.050670	-0.056653	-0.029299	-0.076729	-0.078236	-0.007889	-
0.036407	-0.040450	0.041986	0.000595	-0.028820	-0.006332	-
0.024724	-0.109843	0.020204	-0.040332	0.019255	-0.018313	

z44	-0.026529	0.028369	-0.021689	-0.054900	-0.009990	
0.028974	-0.013992	0.064134	-0.026550	0.008568	-0.030788	
0.128347	0.106082	-0.039181	-0.019993	0.024190	0.013013	-
0.048359	0.036402	-0.102682	-0.065664	-0.014094	0.000718	-
0.081308	0.004209	0.022919	-0.039559	0.009008	0.019762	-
0.059264	0.003150	-0.065202	0.018645	-0.026780	0.048105	
x45	0.001716	0.004182	-0.011993	-0.031827	0.012048	-
0.044731	-0.015022	0.084533	0.026368	0.020170	0.036774	
0.040165	-0.022894	-0.001553	0.021203	-0.071540	-0.134882	-
0.024097	0.011378	-0.036571	-0.021097	-0.022037	-0.043042	-
0.046179	0.020883	-0.070927	0.079679	-0.004090	0.121130	-
0.001884	0.008443	0.097738	-0.077709	-0.030844	-0.043169	
y45	0.018600	0.023816	0.050379	0.030186	0.022286	-
0.080561	-0.100924	0.032984	0.001695	0.068223	0.153585	-
0.036744	0.053892	-0.031172	-0.002759	-0.052150	-0.081539	-
0.074418	-0.044649	-0.009131	-0.124956	-0.052133	-0.057856	-
0.056363	-0.040807	0.037381	-0.026035	-0.016103	0.006421	-
0.009945	-0.080306	-0.015358	-0.050410	-0.017179	-0.039352	
z45	-0.023535	0.023091	-0.014894	-0.062517	0.004481	
0.032260	-0.005819	0.062630	-0.017113	0.033909	-0.021362	
0.129309	0.102736	-0.020914	-0.026049	0.024313	0.030388	-
0.058222	0.028230	-0.102987	-0.074966	0.004940	0.036388	-
0.090429	-0.005204	0.004439	-0.040964	0.022033	0.019670	-
0.069114	0.016512	0.028423	0.039961	-0.056474	0.044855	
x46	0.006447	0.001057	-0.001366	-0.021605	0.029889	-
0.067625	-0.059869	0.105922	0.022803	0.051116	0.082494	
0.027395	-0.026447	-0.034821	0.041803	-0.095470	-0.142208	-
0.085619	0.028859	-0.055411	-0.067946	0.008910	-0.071219	-
0.080268	0.028481	-0.077808	0.075192	0.040697	0.165754	
0.000437	0.045446	0.089164	-0.108831	-0.048836	-0.041201	
y46	0.010171	0.031416	0.062676	0.041887	0.036566	-
0.070831	-0.119700	0.015076	-0.021395	0.062404	0.176263	-
0.035533	0.064681	-0.077775	0.026079	-0.114503	-0.087151	-
0.095725	-0.028396	0.015286	-0.178139	-0.014357	-0.119211	-
0.085283	-0.037707	0.028467	-0.027421	-0.023088	0.030105	
0.010834	-0.043929	-0.029911	-0.052527	-0.020203	-0.062849	
z46	-0.018977	0.015955	-0.004462	-0.069016	0.020549	
0.035667	-0.000921	0.058233	-0.003633	0.061266	-0.014139	
0.123788	0.090371	0.001082	-0.032996	0.028276	0.056300	-
0.061778	0.017288	-0.094449	-0.081931	0.017716	0.077514	-
0.102337	-0.016054	-0.006811	-0.037624	0.040260	0.009453	-
0.076564	0.034335	0.140645	0.067084	-0.090504	0.039867	
x47	0.013952	-0.001908	-0.002807	-0.024640	0.036899	-
0.067723	-0.069409	0.088630	0.015844	0.071622	0.056318	
0.016517	-0.055003	-0.017029	0.049876	-0.073408	-0.122763	-
0.060769	0.018900	-0.069339	-0.087246	0.014628	-0.070746	-
0.032633	0.041550	-0.015349	0.051747	0.046285	0.119428	
0.011276	0.047269	0.056543	-0.069076	-0.008332	-0.052310	
y47	0.005620	0.030523	0.056981	0.027855	0.033651	-
0.047170	-0.096647	-0.027026	-0.036394	0.048952	0.096274	-
0.010074	0.045012	-0.038815	0.006452	-0.105985	-0.032063	-
0.042254	-0.001874	0.045266	-0.122587	-0.027937	-0.089818	-
0.026665	-0.025841	0.013998	-0.036418	-0.035389	0.036791	-
0.010454	0.013067	-0.106400	0.003277	-0.051715	-0.035125	
z47	-0.013647	0.007440	-0.000141	-0.071247	0.030135	
0.049063	0.001038	0.045556	0.002110	0.079980	-0.005696	
0.102342	0.086490	0.010511	-0.032816	0.029063	0.043398	-
0.046765	0.017035	-0.073144	-0.066554	0.013199	0.048274	-
0.037550	-0.023173	-0.018750	-0.019657	-0.014607	0.022841	-
0.066578	0.032749	0.105828	0.079737	-0.026871	0.008507	

x48	0.020197	-0.006622	-0.004710	-0.031431	0.042447	-
0.063133	-0.071435	0.064435	0.007291	0.088031	0.019178	
0.015323	-0.080816	-0.003149	0.053526	-0.057508	-0.092448	-
0.038606	0.014419	-0.083895	-0.074297	0.013278	-0.062252	
0.007365	0.056102	0.016592	0.007173	0.056028	0.071107	
0.033774	0.052800	-0.013757	-0.032718	0.006655	-0.062755	
y48	0.000331	0.031434	0.052505	0.017837	0.028625	-
0.026393	-0.078449	-0.056827	-0.050020	0.031123	0.034998	
0.006306	0.034002	-0.003058	-0.011428	-0.099413	0.005006	
0.001487	0.018903	0.070703	-0.089667	-0.035558	-0.073797	
0.024650	-0.014142	0.003011	-0.032300	-0.059049	0.050705	-
0.047747	0.034347	-0.114333	0.058860	-0.082782	0.001093	
z48	-0.009006	-0.000605	0.004794	-0.070889	0.037579	
0.060740	0.002522	0.031128	0.007403	0.093211	0.000399	
0.078756	0.081146	0.017998	-0.035154	0.030462	0.032880	-
0.031451	0.017245	-0.050938	-0.055133	0.006728	0.025022	
0.017711	-0.026816	-0.027535	-0.006142	-0.054817	0.028383	-
0.058782	0.026650	0.069112	0.075452	0.020281	-0.002073	
x49	0.025062	-0.010894	-0.007678	-0.039529	0.045657	-
0.056041	-0.070139	0.038968	-0.002683	0.093678	-0.021018	
0.024349	-0.102529	0.012018	0.050985	-0.053158	-0.056478	-
0.022832	0.016863	-0.094209	-0.045656	0.011768	-0.060365	
0.046197	0.073029	0.033470	-0.038208	0.044850	0.035064	
0.043939	0.048040	-0.076239	0.025518	0.002514	-0.048845	
y49	-0.005021	0.035234	0.049590	0.012830	0.025927	-
0.010394	-0.065520	-0.076096	-0.059873	0.017241	-0.001190	
0.002508	0.033739	0.020430	-0.016175	-0.078869	0.028562	
0.036256	0.025000	0.093801	-0.087128	-0.034432	-0.046112	
0.048427	-0.011952	-0.005737	-0.006929	-0.050866	0.057885	-
0.073857	0.034959	-0.078130	0.052135	-0.104821	-0.007193	
z49	-0.005286	-0.008237	0.010127	-0.068120	0.042013	
0.069605	0.004462	0.018111	0.012873	0.100650	0.003904	
0.055133	0.078028	0.023201	-0.038349	0.025787	0.023330	-
0.018277	0.020840	-0.028188	-0.043533	0.003199	0.011088	
0.054300	-0.029715	-0.041613	0.009541	-0.080812	0.029823	-
0.041778	0.012938	0.044233	0.052009	0.045161	-0.017488	
x50	0.030409	-0.013613	-0.012665	-0.044714	0.049533	-
0.047056	-0.069825	0.017302	-0.015855	0.091843	-0.054201	
0.027297	-0.114474	0.026854	0.054479	-0.047661	-0.023946	-
0.016918	0.017522	-0.102654	-0.028214	0.014420	-0.049457	
0.067045	0.076359	0.040264	-0.058068	0.037956	0.022533	
0.043370	0.032440	-0.095237	0.043294	-0.013453	-0.052370	
y50	-0.012206	0.039907	0.050453	0.009645	0.025741	-
0.002397	-0.050099	-0.092770	-0.055586	0.005705	-0.012280	
0.005358	0.034993	0.026972	-0.029136	-0.055463	0.057685	
0.060802	0.024944	0.136133	-0.083271	-0.026230	-0.023362	
0.066702	-0.007222	0.007150	0.003956	-0.035413	0.018977	-
0.079580	0.028688	-0.077913	0.085260	-0.088477	-0.011546	
z50	-0.002388	-0.015179	0.016066	-0.063035	0.043322	
0.074694	0.008638	0.007716	0.019101	0.103127	0.005731	
0.036835	0.074530	0.024896	-0.041814	0.014177	0.013878	-
0.005914	0.026376	-0.006090	-0.034988	0.005462	0.003219	
0.076619	-0.034707	-0.048008	0.017480	-0.096480	0.029209	-
0.019976	-0.001967	0.024301	0.033973	0.065325	-0.039060	
x51	0.034893	-0.013585	-0.017959	-0.048541	0.056591	-
0.038705	-0.068655	-0.005984	-0.019229	0.082537	-0.074238	
0.030598	-0.116228	0.033289	0.055598	-0.039747	0.021713	-
0.019710	0.012928	-0.086841	-0.016992	0.027429	-0.032459	
0.069134	0.072648	0.044903	-0.074732	0.035912	0.000661	
0.034155	0.008065	-0.098303	0.070624	-0.036506	-0.051217	

y51	-0.019511	0.045081	0.049767	0.011850	0.021308		
0.002349	-0.035371	-0.096700	-0.062587	-0.009637	-0.014495		
0.002530	0.045805	0.040388	-0.037467	-0.050638	0.058600		
0.071923	0.038740	0.164094	-0.069139	-0.037330	-0.032952		
0.070913	-0.002930	-0.007192	0.042539	-0.035418	0.022313	-	
0.044843	0.024114	-0.059574	0.045391	-0.018310	0.019272		
z51	-0.000161	-0.021455	0.022337	-0.055950	0.042315		
0.076780	0.013057	-0.001989	0.025151	0.103676	0.008764		
0.015970	0.069522	0.026535	-0.047976	0.003328	0.004337		
0.010094	0.035790	0.016369	-0.032905	0.015853	0.003625		
0.081111	-0.035044	-0.046240	0.025631	-0.098112	0.026191		
0.005705	-0.024740	-0.002153	0.005857	0.064459	-0.046295		
x52	0.039226	-0.011221	-0.025402	-0.048153	0.066130	-	
0.030649	-0.066354	-0.028349	-0.018697	0.063262	-0.080610		
0.033498	-0.106867	0.038907	0.055179	-0.036898	0.069688	-	
0.036639	0.015653	-0.047563	-0.010184	0.039364	-0.019491		
0.055204	0.061053	0.033233	-0.072790	0.031413	-0.006909		
0.031517	-0.025170	-0.080751	0.080187	-0.038805	-0.031779		
y52	-0.028159	0.049497	0.051514	0.011204	0.014824		
0.006926	-0.024119	-0.094533	-0.068079	-0.025058	-0.019874		
0.010574	0.065558	0.032252	-0.036510	-0.037061	0.057875		
0.074944	0.016476	0.172032	-0.044935	-0.028168	-0.029071		
0.052576	-0.010432	-0.024954	0.087475	-0.016297	0.015040	-	
0.041984	0.013932	-0.035895	-0.005844	0.038417	0.002842		
z52	0.001328	-0.027510	0.028842	-0.047148	0.038674		
0.074620	0.019277	-0.011301	0.031994	0.103506	0.008095	-	
0.004857	0.062710	0.026422	-0.051680	-0.009116	-0.005483		
0.027699	0.045981	0.038852	-0.036248	0.029648	0.015589		
0.077650	-0.033298	-0.034024	0.022081	-0.083016	0.022942		
0.024833	-0.029980	-0.030014	-0.023865	0.039269	-0.039378		
x53	0.043114	-0.006218	-0.034854	-0.045719	0.078042	-	
0.021828	-0.063422	-0.051731	-0.013637	0.032775	-0.079126		
0.040944	-0.086172	0.039133	0.053280	-0.036618	0.123301	-	
0.067024	0.014564	0.011049	-0.002925	0.053891	-0.005604		
0.023242	0.033887	0.010912	-0.053850	0.028161	-0.010935		
0.025972	-0.068846	-0.038858	0.075244	-0.032657	-0.013355		
y53	-0.037762	0.051300	0.055410	0.007958	0.003106		
0.005668	-0.014057	-0.084653	-0.073527	-0.036166	-0.014917		
0.014692	0.077292	0.016992	-0.040836	-0.029685	0.037759		
0.075833	-0.000633	0.138277	-0.045300	-0.019187	-0.018507		
0.063364	-0.019174	-0.017446	0.084052	-0.034417	-0.015425	-	
0.018204	-0.017726	0.018081	-0.027654	0.014416	-0.005454		
z53	0.002115	-0.033241	0.035031	-0.036978	0.032873		
0.069048	0.025928	-0.020468	0.040182	0.103022	0.006717	-	
0.022147	0.054395	0.025776	-0.058180	-0.022082	-0.019033		
0.046381	0.056807	0.058217	-0.037891	0.043293	0.031267		
0.062358	-0.039838	-0.010221	0.011843	-0.058003	0.015736		
0.024709	-0.031584	-0.056728	-0.055723	0.017048	-0.028813		
x54	0.046046	0.000788	-0.045778	-0.041611	0.091798	-	
0.012469	-0.057363	-0.077060	-0.006101	-0.006949	-0.071613		
0.048815	-0.058988	0.039533	0.051045	-0.040722	0.181758	-	
0.105507	0.013812	0.084617	0.001465	0.069487	0.012177	-	
0.022576	0.002169	-0.023090	-0.028754	0.021127	-0.015453		
0.008678	-0.116463	0.024368	0.056704	-0.025276	0.010789		
y54	-0.048858	0.049970	0.063216	0.004802	-0.010922		
0.003948	0.002778	-0.070368	-0.080713	-0.034717	-0.007617		
0.001560	0.084109	0.019494	-0.037797	-0.025235	0.007873		
0.084116	-0.006159	0.099258	-0.044219	-0.016019	-0.006447		
0.064722	0.017086	-0.014054	0.038552	-0.035967	-0.030915	-	
0.034597	-0.000100	0.055617	-0.049974	0.050480	0.000886		

z54	0.002059	-0.038580	0.042221	-0.024890	0.024053		
0.060258	0.034611	-0.028398	0.046606	0.096708	0.001930	-
0.037035	0.045660	0.024612	-0.061975	-0.034114	-0.032434	
0.064122	0.065299	0.071217	-0.039550	0.058406	0.056365	
0.024344	-0.052224	0.016766	-0.004136	-0.031544	-0.001756	
0.012752	-0.038345	-0.081632	-0.087220	-0.009611	-0.031567	
x55	0.047755	0.008457	-0.057261	-0.036863	0.103359	
0.001123	-0.050841	-0.101706	0.000325	-0.048429	-0.057869	
0.056141	-0.027254	0.031757	0.044886	-0.044177	0.245482	-
0.146287	0.013280	0.159383	0.012240	0.080109	0.027345	-
0.075885	-0.023818	-0.046466	-0.020634	0.023997	-0.004872	-
0.013722	-0.163987	0.100541	0.053387	-0.037545	0.053927	
y55	-0.060365	0.046280	0.073796	-0.000723	-0.031653	
0.005442	0.012760	-0.043530	-0.095050	-0.017583	0.005738	-
0.009893	0.087173	-0.000258	-0.050264	-0.016198	-0.036880	
0.105417	-0.011905	0.018007	-0.035124	-0.011272	-0.011606	
0.083778	0.061444	0.035817	-0.024989	-0.032212	-0.020310	-
0.034141	0.008754	0.054119	-0.027832	0.034004	-0.007224	
z55	0.001431	-0.042888	0.049993	-0.010307	0.012481	
0.048131	0.044484	-0.032183	0.051153	0.086358	-0.005052	-
0.049675	0.037805	0.018330	-0.065097	-0.041097	-0.045787	
0.076024	0.072367	0.082096	-0.041930	0.070172	0.085271	-
0.028331	-0.071605	0.057806	-0.033099	0.003968	-0.019981	-
0.007470	-0.043743	-0.104010	-0.101887	-0.046409	-0.041922	
x56	0.037414	0.011064	-0.063154	-0.047178	0.088675	
0.013563	-0.045837	-0.105104	-0.007557	-0.054563	-0.049686	
0.019655	-0.003357	0.020412	0.000864	-0.057503	0.181684	-
0.117190	-0.007986	0.129727	-0.013597	0.039777	0.023650	-
0.060944	0.019269	-0.067448	-0.022497	0.039381	0.012408	-
0.014227	-0.098527	0.066314	-0.043943	-0.002814	0.040356	
y56	-0.068954	0.042947	0.089907	-0.002248	-0.039431	
0.007241	0.013821	-0.022798	-0.105380	-0.009689	0.013230	-
0.010715	0.073712	-0.001831	-0.038531	-0.017877	-0.053181	
0.114350	-0.009951	0.009877	-0.040580	0.008551	-0.022213	
0.063440	0.118071	-0.020524	-0.043937	0.025256	-0.015391	-
0.009003	0.011864	0.011827	-0.069023	0.046761	-0.025282	
z56	0.000419	-0.045750	0.059709	0.005400	0.006530	
0.024791	0.039516	-0.031759	0.061817	0.086266	-0.022872	-
0.029671	0.028801	0.003273	-0.030139	-0.052134	-0.050764	
0.077125	0.059186	0.083810	-0.023294	0.052076	0.063548	-
0.055531	-0.085303	0.031301	0.000752	0.044892	0.013802	
0.019890	-0.042693	-0.078963	-0.039062	-0.011462	-0.027493	
x57	0.026632	0.014221	-0.066934	-0.055266	0.073657	
0.024121	-0.042247	-0.104918	-0.009690	-0.051974	-0.044707	-
0.005827	0.021073	0.006935	-0.034468	-0.064216	0.144516	-
0.100170	-0.021096	0.094870	-0.011375	0.001993	0.032347	-
0.031659	0.018761	-0.042171	-0.057555	0.032246	0.028041	-
0.023197	-0.057322	0.051135	-0.072736	-0.029217	0.035495	
y57	-0.076200	0.041700	0.102634	-0.000974	-0.044870	
0.005668	0.017420	-0.002361	-0.105483	0.005922	0.016428	
0.003530	0.062282	-0.002030	-0.025556	-0.010375	-0.039938	
0.108391	-0.004834	-0.003681	-0.001072	0.018479	-0.015051	
0.059639	0.104693	-0.000806	-0.094039	0.041767	-0.009953	-
0.016911	-0.012637	0.018534	-0.024628	-0.033314	-0.034095	
z57	-0.000365	-0.047434	0.068639	0.017333	-0.000429	
0.002589	0.034247	-0.033891	0.072613	0.085872	-0.035988	-
0.013251	0.020617	-0.010385	-0.001119	-0.058859	-0.045246	
0.077130	0.048386	0.080785	-0.011741	0.038833	0.039907	-
0.082022	-0.092151	0.006503	0.033271	0.077186	0.027442	
0.043362	-0.044552	-0.064767	-0.000754	-0.009517	0.005079	

x58	0.016193	0.018142	-0.061816	-0.060322	0.061869	
0.024642	-0.037964	-0.100858	-0.010881	-0.046779	-0.032223	-
0.032860	0.034754	0.003628	-0.051500	-0.062218	0.097953	-
0.077767	-0.041958	0.058128	-0.000651	-0.029340	0.035656	-
0.023891	0.041079	-0.033943	-0.024447	0.068425	0.028963	
0.008135	-0.023665	0.044398	-0.085994	-0.042274	0.060697	
y58	-0.080499	0.040589	0.118470	0.003484	-0.046444	-
0.005682	0.024523	0.017888	-0.104603	0.019909	0.026312	
0.010245	0.040560	0.003623	-0.003439	-0.000552	-0.043595	
0.104763	-0.010308	-0.020956	0.026551	0.027252	-0.011190	
0.023578	0.125267	-0.012669	-0.056320	0.087935	-0.026970	
0.015035	-0.030359	0.021361	0.007087	-0.081605	0.011996	
z58	-0.000842	-0.047733	0.076277	0.026917	-0.007136	-
0.016833	0.033496	-0.034433	0.083080	0.082351	-0.048243	
0.004232	0.015870	-0.022075	0.023043	-0.061254	-0.043391	
0.070652	0.036571	0.075053	-0.002804	0.025255	0.019972	-
0.090360	-0.093436	-0.010085	0.055371	0.096116	0.042512	
0.050748	-0.040280	-0.037817	0.032539	0.002482	0.016969	
x59	0.005467	0.018755	-0.052270	-0.059004	0.047963	
0.026175	-0.033616	-0.092525	-0.012643	-0.041288	-0.014695	-
0.053936	0.047954	-0.004847	-0.060594	-0.064880	0.069538	-
0.052848	-0.046214	0.007271	-0.006775	-0.045439	0.042418	-
0.030423	0.045312	-0.027879	-0.033711	0.081397	0.023355	
0.000333	0.017322	0.027586	-0.065617	-0.079579	0.069765	
y59	-0.083020	0.035888	0.131997	0.012986	-0.049471	-
0.012784	0.033611	0.036171	-0.102314	0.028327	0.034139	
0.017209	0.022349	0.003152	0.013814	0.001861	-0.027116	
0.090670	0.002794	-0.045996	0.030200	0.044833	-0.001331	-
0.018346	0.123742	-0.020182	-0.068454	0.089117	-0.036745	
0.016669	-0.025667	0.009564	0.054587	-0.147242	0.025671	
z59	-0.001139	-0.046560	0.081874	0.034471	-0.012813	-
0.034400	0.035119	-0.036550	0.092650	0.079180	-0.057657	
0.019484	0.011516	-0.029172	0.042256	-0.058344	-0.037849	
0.059414	0.020649	0.068711	0.002468	0.012882	0.001155	-
0.083656	-0.084415	-0.014556	0.072141	0.101213	0.052542	
0.057182	-0.034769	-0.013089	0.046449	0.005417	0.040582	
x60	-0.005931	0.016965	-0.035978	-0.059661	0.033930	
0.026664	-0.026365	-0.087051	-0.019066	-0.032362	-0.006381	-
0.073018	0.068422	-0.002558	-0.070770	-0.066421	0.041303	-
0.016278	-0.046286	-0.016402	-0.005218	-0.031565	0.041605	-
0.024444	0.051232	0.014237	0.021514	0.084434	0.040751	-
0.002169	-0.011192	0.043904	-0.071971	-0.038298	0.098864	
y60	-0.085124	0.031186	0.144239	0.019825	-0.050469	-
0.017566	0.045537	0.048582	-0.104206	0.038393	0.029337	
0.026496	0.005273	0.012512	0.022327	-0.000169	-0.007664	
0.079081	0.014258	-0.043321	0.045743	0.067718	-0.000799	-
0.040607	0.118890	-0.007602	-0.028865	0.066813	-0.024110	
0.017128	-0.059216	0.024617	0.055904	-0.120894	0.040699	
z60	-0.000810	-0.042794	0.085746	0.040353	-0.016359	-
0.049856	0.037438	-0.036796	0.102639	0.076180	-0.063910	
0.035281	0.003670	-0.034328	0.060274	-0.048845	-0.026250	
0.043438	0.001910	0.057556	0.009773	-0.006391	-0.009611	-
0.070138	-0.071939	-0.032355	0.062171	0.104198	0.054937	
0.048209	-0.017153	0.000585	0.060636	0.004533	0.033484	
x61	-0.017838	0.014444	-0.016181	-0.058120	0.019644	
0.024100	-0.022286	-0.078373	-0.019549	-0.016051	-0.000370	-
0.097609	0.076422	-0.001355	-0.062743	-0.069212	0.036493	-
0.000927	-0.035311	-0.038946	-0.025000	-0.016250	0.039067	-
0.011276	0.045260	0.043442	0.016013	0.087822	0.021826	
0.038728	-0.010490	0.037768	-0.046329	-0.019914	0.148741	

y61	-0.086431	0.027557	0.153610	0.028674	-0.050687	-
0.022331	0.057696	0.060078	-0.100904	0.047712	0.021025	
0.029916	-0.020218	0.019279	0.033620	0.000416	0.026186	
0.042506	0.035616	-0.038912	0.047481	0.080409	0.001090	-
0.058017	0.098699	-0.007252	-0.037536	0.035622	-0.023922	
0.045219	-0.047588	0.025378	0.058846	-0.099118	0.052226	
z61	-0.000302	-0.037640	0.088306	0.043680	-0.019951	-
0.063157	0.043565	-0.038496	0.111632	0.070305	-0.070692	
0.050870	0.000421	-0.036950	0.075059	-0.036226	-0.013754	
0.025163	-0.019563	0.042739	0.013395	-0.024707	-0.015473	-
0.045117	-0.059183	-0.046281	0.056542	0.086144	0.059021	
0.033246	-0.005058	0.023982	0.053367	-0.000804	0.035116	
x62	-0.029188	0.013565	0.004977	-0.051371	0.010933	
0.025447	-0.025657	-0.066315	-0.021976	-0.001285	0.005679	-
0.103863	0.079565	-0.003686	-0.043332	-0.069521	0.031722	
0.017662	-0.023537	-0.067741	-0.005410	-0.019986	0.051365	
0.002967	0.021016	0.016256	0.003951	0.110961	0.024889	
0.055499	-0.001699	0.027688	-0.009092	0.023239	0.079986	
y62	-0.086046	0.025229	0.158989	0.040016	-0.048171	-
0.023894	0.070371	0.069785	-0.098673	0.053539	0.006340	
0.040714	-0.049709	0.027196	0.040430	0.001386	0.061288	-
0.004341	0.054178	-0.031704	0.062192	0.076622	0.010526	-
0.070644	0.061603	-0.018348	-0.030159	-0.006474	-0.001599	
0.059909	-0.022389	0.040123	0.033235	-0.061729	0.001232	
z62	0.000266	-0.031798	0.090343	0.043261	-0.025254	-
0.076409	0.055095	-0.043670	0.119655	0.064932	-0.075967	
0.062077	-0.001211	-0.033943	0.078882	-0.019491	-0.001519	
0.001852	-0.040322	0.031228	0.000680	-0.032171	-0.024317	-
0.012630	-0.037081	-0.027572	0.054663	0.056456	0.046135	
0.028942	0.005410	0.048497	0.015124	-0.022717	0.077932	
x63	-0.040030	0.014153	0.026321	-0.043658	0.001705	
0.023194	-0.024755	-0.045121	-0.027409	0.022304	0.008661	-
0.098747	0.071405	0.001658	-0.024763	-0.076376	0.039411	
0.026686	-0.017299	-0.088033	0.038963	-0.030174	0.050879	
0.006177	0.021858	-0.019857	0.036455	0.109091	0.035851	
0.030530	0.025856	-0.010221	0.025112	0.035096	-0.003777	
y63	-0.084325	0.023669	0.161423	0.051920	-0.044822	-
0.025959	0.087596	0.078585	-0.096027	0.058549	-0.012522	
0.055923	-0.083920	0.040394	0.038207	0.000658	0.102167	-
0.062796	0.071642	-0.017173	0.073231	0.073876	0.016953	-
0.085951	0.027409	-0.014047	-0.002257	-0.070099	0.037412	
0.063837	0.014342	0.052190	-0.001625	-0.020251	-0.041268	
z63	0.001337	-0.024598	0.091997	0.040926	-0.030454	-
0.086786	0.069220	-0.050023	0.127300	0.057470	-0.079845	
0.071637	0.000816	-0.029208	0.079102	0.001860	0.013314	-
0.025843	-0.059847	0.018075	-0.019125	-0.040928	-0.028391	
0.031416	-0.016843	-0.000795	0.044746	0.014099	0.017257	
0.024760	0.017719	0.080795	-0.045471	-0.047088	0.115612	
x64	-0.050489	0.013517	0.052185	-0.034205	-0.009672	
0.017370	-0.016210	-0.027143	-0.030444	0.043759	0.004956	-
0.085497	0.060632	0.010185	-0.018038	-0.072846	0.053952	
0.018575	-0.016310	-0.102779	0.055913	-0.025566	0.048739	
0.007580	0.008077	-0.004457	0.064847	0.081522	0.048895	
0.041170	0.051294	-0.002405	0.016071	0.058018	-0.062564	
y64	-0.080957	0.021641	0.162356	0.065797	-0.040855	-
0.027193	0.106579	0.083610	-0.091216	0.060350	-0.037340	
0.070023	-0.119417	0.054136	0.033196	-0.000128	0.152674	-
0.130820	0.090292	0.002004	0.074343	0.073169	0.026546	-
0.098378	-0.018264	0.002131	0.020675	-0.146718	0.079067	
0.079990	0.051589	0.079560	-0.045051	0.043688	-0.077925	

z64	0.002676	-0.015564	0.091421	0.036261	-0.033911	-
0.094835	0.082404	-0.052877	0.133075	0.050249	-0.076909	
0.078921	-0.000783	-0.022380	0.082182	0.023503	0.032868	-
0.049917	-0.075730	0.001809	-0.029046	-0.050235	-0.029486	
0.084206	0.007395	0.015190	0.025573	-0.032917	-0.012893	
0.000762	0.034887	0.093757	-0.097639	-0.078067	0.130970	
x65	-0.059212	0.011895	0.070225	-0.026790	-0.015030	
0.016586	-0.008677	-0.016142	-0.033143	0.057374	-0.000767	-
0.080665	0.037441	0.020962	-0.026338	-0.072871	0.060169	-
0.024103	-0.010169	-0.097658	0.072766	-0.024640	0.050047	
0.010328	0.007989	-0.043926	0.071272	0.062116	0.001129	
0.029669	0.072725	-0.000896	0.017800	0.040314	-0.093100	
y65	-0.070334	0.033480	0.126311	0.068043	-0.031843	-
0.024275	0.092089	0.061301	-0.059528	0.041202	-0.020313	
0.071477	-0.146019	0.048062	-0.020142	0.006613	0.116846	-
0.121419	0.049505	0.014501	0.016296	0.040696	-0.001696	-
0.061063	-0.034166	0.008867	-0.007669	-0.059391	0.049377	
0.013775	0.030436	0.052368	-0.053453	0.033667	-0.026323	
z65	0.007206	-0.005655	0.076184	0.026257	-0.034337	-
0.096991	0.072514	-0.075983	0.138532	0.041770	-0.057217	
0.048511	0.022728	-0.037833	0.081477	0.010557	0.025051	-
0.058309	-0.056600	-0.017219	-0.038063	-0.057964	-0.022972	
0.074224	0.028493	0.029520	-0.001486	-0.001729	-0.061800	-
0.019256	-0.014018	0.073083	-0.067466	-0.033899	0.065862	
x66	-0.061824	0.011306	0.080217	-0.017276	-0.016523	
0.015694	-0.009601	-0.020295	-0.032607	0.067671	-0.005392	-
0.074526	0.024548	0.029637	-0.039235	-0.067867	0.058957	-
0.047890	-0.006129	-0.102010	0.064195	-0.026379	0.014770	
0.002737	0.006284	-0.069684	0.051488	0.051236	-0.015272	-
0.007976	0.060273	0.010077	0.014610	0.068755	-0.089923	
y66	-0.059715	0.042601	0.092821	0.069095	-0.023717	-
0.023221	0.080037	0.042810	-0.028379	0.021372	-0.001132	
0.070252	-0.164808	0.039332	-0.063860	0.011459	0.082595	-
0.111455	0.012193	0.026642	-0.030514	0.008792	-0.017095	-
0.026641	-0.045527	0.015171	-0.026584	0.017341	0.016177	-
0.028144	0.010969	0.019314	-0.052809	0.030059	0.011270	
z66	0.009492	0.002715	0.062409	0.016631	-0.035522	-
0.098042	0.065898	-0.090346	0.136133	0.033407	-0.040661	
0.025906	0.037665	-0.047433	0.082566	-0.000588	0.019956	-
0.060972	-0.037663	-0.034060	-0.037488	-0.052160	-0.011555	
0.070003	0.049089	0.054388	-0.016164	0.014877	-0.094369	-
0.027937	-0.042364	0.046271	-0.037328	-0.007012	0.010325	
x67	-0.064383	0.014676	0.082149	-0.009723	-0.019437	
0.012711	-0.009815	-0.025950	-0.027553	0.070317	-0.006903	-
0.062325	0.008253	0.042977	-0.065552	-0.053719	0.048871	-
0.063580	-0.013603	-0.097282	0.060542	-0.029571	-0.004174	
0.003073	0.030103	-0.083506	0.058314	0.051373	-0.043920	-
0.006339	0.045947	-0.004334	0.044243	0.084595	-0.065205	
y67	-0.050517	0.050238	0.063691	0.068083	-0.015680	-
0.022161	0.070098	0.028207	-0.002480	0.003244	0.015014	
0.066812	-0.173344	0.026516	-0.090176	0.012459	0.054472	-
0.100203	-0.014105	0.033625	-0.071339	-0.017754	-0.026205	-
0.004395	-0.061523	0.023111	-0.050162	0.076719	-0.009768	-
0.066403	-0.006262	0.001731	-0.059870	0.021037	0.027368	
z67	0.011406	0.007895	0.051301	0.009119	-0.034971	-
0.095976	0.060167	-0.102987	0.129693	0.026659	-0.029386	
0.007052	0.051872	-0.055075	0.091125	-0.004895	0.017534	-
0.061445	-0.020316	-0.047803	-0.037626	-0.044645	-0.003206	
0.068828	0.051134	0.073382	-0.038765	0.027759	-0.109641	-
0.037798	-0.055876	0.032513	-0.037496	0.010718	-0.048424	

x68	-0.066091	0.018127	0.081611	-0.005281	-0.018540	
0.011025	-0.015132	-0.033820	-0.017457	0.068841	-0.004114	-
0.050109	-0.007547	0.042357	-0.092474	-0.042640	0.023432	-
0.077294	-0.030223	-0.105238	0.036944	-0.037282	-0.007656	
0.013888	0.004511	-0.093758	0.028944	0.084009	-0.076309	-
0.023063	0.045695	0.009541	0.031587	0.106669	-0.085923	
y68	-0.042114	0.056496	0.036920	0.066946	-0.008768	-
0.023051	0.064598	0.018433	0.018095	-0.013381	0.026173	
0.063078	-0.168993	0.015729	-0.100180	0.014125	0.041051	-
0.086399	-0.024935	0.046960	-0.091703	-0.033580	-0.035292	
0.006881	-0.053037	0.034974	-0.047609	0.099798	-0.017338	-
0.073830	-0.019235	-0.021718	-0.043114	0.010118	0.041855	
z68	0.012562	0.011349	0.041009	0.004630	-0.035179	-
0.093150	0.056942	-0.108986	0.115159	0.018462	-0.020985	-
0.008410	0.062490	-0.051809	0.096738	-0.001373	0.019926	-
0.057966	-0.000434	-0.052691	-0.029868	-0.017433	0.001606	
0.064644	0.065249	0.097575	-0.042700	0.016764	-0.090778	-
0.037623	-0.057730	-0.003248	-0.026468	0.013710	-0.079118	
x69	-0.068642	0.017803	0.079502	-0.006969	-0.027681	
0.007739	0.000366	-0.055658	-0.009658	0.052846	-0.022002	-
0.053424	0.010514	0.050468	-0.139928	-0.034648	-0.021456	-
0.099742	-0.025920	-0.090374	-0.028318	0.002938	-0.038790	
0.044221	-0.028790	0.042888	0.037657	0.036323	-0.069610	
0.028667	-0.018200	0.071350	-0.026281	0.130230	0.093314	
y69	-0.034098	0.062394	0.010481	0.066226	0.003036	-
0.024678	0.053223	0.016025	0.034920	-0.024404	0.042703	
0.069411	-0.164597	-0.003708	-0.090345	0.025031	0.041877	-
0.067634	-0.037964	0.057315	-0.082709	-0.063565	-0.012106	-
0.001471	-0.033547	-0.003507	-0.048129	0.130231	-0.025119	-
0.088816	0.001841	-0.047123	-0.007032	-0.007399	-0.010043	
z69	0.014463	0.016852	0.029866	0.006068	-0.026205	-
0.087985	0.039212	-0.101958	0.098731	0.015832	-0.003795	-
0.009738	0.054528	-0.052301	0.121290	0.005175	0.039422	-
0.041589	0.001749	-0.067855	0.012429	-0.018025	0.023070	
0.043780	0.091944	0.003303	-0.077810	0.051692	-0.083038	-
0.077513	-0.010894	-0.065133	0.021093	-0.001512	-0.193317	
x70	-0.066240	0.029592	0.064660	0.004249	-0.010123	
0.008317	-0.024693	-0.054536	-0.000204	0.053204	-0.001998	-
0.027045	-0.014489	0.039596	-0.129658	-0.009344	-0.001204	-
0.086754	-0.050818	-0.087299	0.003023	-0.026089	-0.025122	
0.027670	-0.024847	-0.045104	-0.039050	0.088899	-0.071568	-
0.035795	0.013159	-0.014733	0.041769	0.082913	-0.095249	
y70	-0.028350	0.062807	-0.010002	0.058850	0.005038	-
0.029348	0.058226	0.004674	0.048934	-0.043756	0.036361	
0.063477	-0.126443	-0.013921	-0.084542	0.026393	0.020634	-
0.051397	-0.024563	0.070872	-0.094252	-0.051067	-0.001681	
0.004571	-0.010011	0.045328	-0.008615	0.092543	-0.012662	-
0.030955	-0.016561	-0.009831	0.006425	-0.004449	0.041090	
z70	0.013116	0.012724	0.024634	-0.000368	-0.034327	-
0.084935	0.051944	-0.112669	0.072887	0.001373	-0.011663	-
0.030112	0.074363	-0.034117	0.103870	0.018304	0.010460	-
0.042641	0.020646	-0.060918	-0.023041	0.038154	0.020739	
0.061222	0.084417	0.106600	-0.041538	0.003138	-0.059946	-
0.018466	-0.026575	-0.023108	-0.050649	0.029754	-0.027988	
x71	-0.066037	0.035318	0.047880	0.004504	-0.004530	
0.002016	-0.020283	-0.076773	0.001790	0.039839	-0.009509	-
0.015005	0.003313	0.046159	-0.145243	0.026606	-0.027963	-
0.081012	-0.063398	-0.050016	-0.022693	-0.000799	-0.023964	
0.037474	-0.013833	0.073650	-0.034381	0.048750	-0.030067	
0.009813	-0.027280	0.026727	0.013997	0.070628	0.012170	

y71	-0.021380	0.066900	-0.030714	0.056715	0.014771	-
0.028846	0.042144	0.010636	0.070007	-0.057809	0.046308	
0.070579	-0.099880	-0.049245	-0.050371	0.021579	0.039492	-
0.030348	-0.007857	0.060859	-0.059326	-0.069339	0.033902	-
0.019477	0.000040	-0.049331	-0.026161	0.098768	-0.018869	-
0.038394	0.021168	-0.047231	0.094284	-0.025955	-0.134325	
z71	0.013804	0.014608	0.016930	0.003989	-0.027353	-
0.074437	0.034488	-0.099047	0.050736	-0.004265	0.000473	-
0.025241	0.060260	-0.022021	0.118215	0.026265	0.022456	-
0.024580	0.023040	-0.077560	0.013916	0.050880	0.027938	
0.049344	0.086835	0.001291	-0.085349	0.031476	-0.044162	-
0.037599	0.021328	-0.077913	-0.023084	0.022095	-0.124673	
x72	-0.063376	0.045152	0.022589	0.007887	0.009846	-
0.001400	-0.035359	-0.089074	0.014195	0.024215	-0.012290	
0.015107	0.026446	0.035238	-0.137284	0.057681	-0.034388	-
0.058278	-0.079059	-0.024129	-0.005431	0.010470	-0.008010	
0.027143	-0.011723	0.088130	-0.075723	0.040154	0.015528	
0.014751	-0.015830	0.028073	0.053745	0.024499	-0.050907	
y72	-0.016161	0.067276	-0.046970	0.049956	0.020016	-
0.031575	0.042832	0.009238	0.079630	-0.074473	0.056049	
0.062796	-0.069184	-0.073467	-0.025508	0.014891	0.044393	-
0.022612	0.021065	0.063657	-0.045390	-0.062916	0.076754	-
0.028948	0.046152	-0.051909	-0.018973	0.049381	0.011229	
0.003317	-0.002464	-0.032243	0.146265	-0.017821	-0.160665	
z72	0.012570	0.010549	0.012581	0.005537	-0.028695	-
0.065356	0.035385	-0.091698	0.016506	-0.014215	0.001409	-
0.028699	0.050757	0.004840	0.106346	0.042682	0.009374	-
0.014173	0.030717	-0.073442	0.005899	0.089890	0.024558	
0.058060	0.080263	0.007364	-0.088048	0.012904	-0.009112	-
0.002377	0.031315	-0.078417	-0.070091	0.039250	-0.022202	
x73	-0.060821	0.056070	-0.008203	0.007841	0.025050	-
0.005275	-0.047018	-0.106156	0.029360	-0.001014	-0.017197	
0.042026	0.063730	0.018157	-0.133880	0.085481	-0.049467	-
0.030884	-0.089483	0.017905	-0.002135	0.042414	0.026352	
0.027923	0.002260	0.144574	-0.100012	-0.001009	0.099902	
0.035894	-0.035545	0.061173	0.092680	-0.013097	-0.056781	
y73	-0.011308	0.059268	-0.055585	0.038749	0.012981	-
0.035680	0.057977	0.005152	0.075089	-0.088359	0.054649	
0.045606	-0.024276	-0.080245	-0.012521	0.023296	0.024889	-
0.015233	0.039189	0.045172	-0.072705	-0.036244	0.065481	-
0.040686	0.030177	0.048039	0.032482	-0.002396	0.010060	
0.076608	-0.031799	-0.019456	0.099147	0.015627	-0.027478	
z73	0.009984	0.001578	0.012685	0.006437	-0.033168	-
0.056019	0.044720	-0.083630	-0.024805	-0.023981	-0.002360	-
0.032814	0.039098	0.040334	0.077306	0.073128	-0.016748	-
0.006868	0.028231	-0.062122	-0.024024	0.138904	0.008219	
0.073278	0.031813	0.072914	-0.058717	-0.011471	0.014322	
0.052313	0.043738	-0.072353	-0.154683	0.058800	0.171509	
x74	-0.059310	0.055810	-0.019438	0.019732	0.015811	-
0.017587	-0.026310	-0.102888	0.031810	-0.027046	0.001589	
0.047278	0.030304	0.031089	-0.124080	0.102501	-0.040368	-
0.001261	-0.051616	0.001738	-0.034635	0.031568	0.007929	
0.001255	0.000690	0.122843	-0.042700	-0.008081	0.073690	
0.079212	-0.013120	0.007963	0.048832	0.014974	-0.037208	
y74	-0.005167	0.045003	-0.050353	0.026957	0.004647	-
0.032666	0.063258	0.038566	0.066055	-0.093702	0.059134	
0.038232	-0.012126	-0.066550	0.017250	-0.004318	0.047576	-
0.001956	0.058521	0.021343	-0.052135	-0.034882	0.063577	-
0.015908	0.023364	-0.062761	0.064324	0.000662	-0.027527	
0.018881	0.001978	-0.034831	0.115184	0.079780	-0.040763	

z74	0.007904	0.005024	0.007036	0.011034	-0.030474	-
0.053426	0.035752	-0.071974	-0.037566	-0.023901	-0.003455	-
0.027376	0.029495	0.045517	0.078127	0.080405	-0.009250	-
0.006407	0.062703	-0.038965	-0.004431	0.083703	-0.019034	
0.077247	0.028515	-0.004476	-0.027191	-0.026229	0.041752	
0.005442	-0.023080	-0.051599	-0.064773	0.023098	0.071698	
x75	-0.057368	0.052253	-0.028097	0.026670	0.003536	-
0.028990	-0.007540	-0.092455	0.038061	-0.055220	0.013221	
0.053660	0.015093	0.043543	-0.116489	0.099047	-0.038009	
0.022171	-0.014512	-0.018061	-0.054141	0.035920	-0.007559	-
0.014556	-0.016550	0.070015	0.003228	-0.024060	0.066710	
0.098139	0.005129	0.005124	0.021466	0.031797	-0.003152	
y75	0.000240	0.035432	-0.043357	0.015941	0.003157	-
0.025298	0.072864	0.051683	0.052292	-0.084642	0.055758	
0.015017	0.002673	-0.067930	0.029755	-0.003182	0.066804	
0.011175	0.052642	0.016385	-0.063023	-0.047138	0.062507	
0.001220	0.037573	-0.052168	0.083001	0.018661	-0.080704	
0.047534	0.001890	-0.049990	0.088474	0.091257	0.042354	
z75	0.006145	0.008671	0.003568	0.014302	-0.025264	-
0.050146	0.029161	-0.065531	-0.050086	-0.022747	-0.003468	-
0.030366	0.016063	0.042695	0.070117	0.094357	0.003072	-
0.005518	0.084348	-0.018916	-0.002031	0.030400	-0.041285	
0.079658	0.025295	-0.015170	0.019311	-0.030808	0.048598	-
0.010818	-0.077139	-0.046099	-0.003965	-0.009249	0.016915	
x76	-0.054390	0.048676	-0.033476	0.031586	-0.004204	-
0.040106	0.010673	-0.082712	0.037815	-0.071336	0.023957	
0.057811	0.003444	0.052272	-0.105981	0.103585	-0.032240	
0.041175	0.011442	-0.025859	-0.074786	0.033969	-0.021460	-
0.028712	-0.026047	0.043146	0.041360	-0.023980	0.047920	
0.102561	0.019779	-0.023237	-0.009823	0.048016	0.031108	
y76	0.005311	0.024341	-0.032731	0.003429	0.002978	-
0.020048	0.075137	0.070993	0.041602	-0.076724	0.049429	
0.012445	0.034132	-0.067331	0.044069	-0.014033	0.063553	
0.002713	0.049519	0.019126	-0.043036	-0.054998	0.050737	
0.006303	0.025673	-0.059390	0.080335	0.025898	-0.080450	
0.025434	0.024925	-0.057703	0.061648	0.144809	0.082494	
z76	0.004546	0.010996	0.001495	0.015116	-0.019788	-
0.045299	0.020303	-0.055129	-0.060126	-0.026012	-0.002965	-
0.029492	0.004456	0.040363	0.061847	0.091209	0.008138	-
0.006665	0.095780	-0.006209	0.004286	-0.004330	-0.061976	
0.082224	0.018235	-0.041556	0.054511	-0.030966	0.062010	-
0.022051	-0.105633	-0.029647	0.035881	-0.018589	-0.035628	
x77	-0.050667	0.043474	-0.036958	0.032115	-0.009696	-
0.049910	0.024992	-0.068032	0.032640	-0.081160	0.033440	
0.058078	0.002954	0.052490	-0.094326	0.090672	-0.024821	
0.050179	0.025956	-0.032115	-0.086632	0.042884	-0.029868	-
0.035678	-0.026007	0.010812	0.062792	-0.030504	0.039383	
0.102862	0.030200	-0.045142	-0.031573	0.058139	0.031639	
y77	0.009908	0.015859	-0.023614	-0.008709	0.003515	-
0.016834	0.072206	0.081852	0.027476	-0.059159	0.051754	-
0.006717	0.047432	-0.067767	0.056523	-0.027430	0.072283	-
0.003422	0.030575	0.018527	-0.020345	-0.044564	0.046819	
0.014431	0.036336	-0.050488	0.066530	0.032449	-0.086522	
0.022056	0.038408	-0.088022	0.029705	0.142125	0.052865	
z77	0.003742	0.013645	-0.000737	0.014679	-0.013334	-
0.039594	0.013421	-0.044030	-0.070533	-0.029113	0.000669	-
0.030628	-0.008413	0.038522	0.054843	0.085877	0.014173	-
0.002951	0.095477	0.001207	0.006020	-0.025463	-0.075850	
0.084005	0.006831	-0.056600	0.074315	-0.028286	0.060929	-
0.021261	-0.125217	-0.019277	0.064426	-0.025021	-0.071069	

x78	-0.045514	0.038981	-0.039204	0.031794	-0.011829	-
0.060841	0.038997	-0.050155	0.021467	-0.077154	0.044371	
0.049037	0.008284	0.050584	-0.087131	0.090878	-0.017437	
0.059620	0.042542	-0.024345	-0.092589	0.045123	-0.036972	-
0.041646	-0.033387	0.011274	0.073591	-0.030212	0.002624	
0.097106	-0.011976	-0.063949	-0.024479	0.036830	0.044807	
y78	0.014190	0.008618	-0.014301	-0.018170	-0.000295	-
0.014677	0.064602	0.105129	0.025170	-0.049745	0.053678	-
0.020967	0.071459	-0.052509	0.049835	-0.045015	0.047139	-
0.015816	0.043353	0.030203	0.032686	-0.040233	0.029194	
0.026261	0.000028	-0.074523	0.042426	0.017710	-0.059861	
0.017882	-0.015732	-0.029561	0.048609	0.130430	0.043472	
z78	0.002785	0.014758	-0.002794	0.010951	-0.008065	-
0.033731	0.004303	-0.031287	-0.077561	-0.037284	0.002857	-
0.024783	-0.012176	0.029673	0.049767	0.067790	0.018467	
0.001923	0.089220	-0.004857	0.011753	-0.027885	-0.084905	
0.088952	-0.004394	-0.074915	0.084575	-0.028568	0.067774	-
0.007222	-0.119558	0.010763	0.076548	-0.029427	-0.095467	
x79	-0.040354	0.032643	-0.039435	0.026515	-0.012800	-
0.068555	0.047602	-0.031245	0.015067	-0.078531	0.048164	
0.041941	0.025870	0.039502	-0.068831	0.073839	-0.000982	
0.059095	0.041050	-0.018905	-0.093826	0.056667	-0.043267	-
0.033476	-0.051670	0.005842	0.067808	-0.015717	-0.037157	
0.057273	-0.024307	-0.069514	-0.017237	0.008447	0.026821	
y79	0.017405	0.003697	-0.004105	-0.027028	-0.002446	-
0.012503	0.053096	0.109180	0.031125	-0.042191	0.053249	-
0.031020	0.083736	-0.043241	0.056591	-0.044074	0.043896	-
0.027506	0.032412	0.034489	0.075217	-0.028613	0.016505	
0.051715	-0.032352	-0.057009	0.026005	0.033996	-0.034072	
0.001820	0.005911	-0.019743	0.021286	0.097372	-0.003270	
z79	0.002582	0.016325	-0.005186	0.006698	-0.001991	-
0.026270	-0.003604	-0.016349	-0.082919	-0.047302	0.007955	-
0.022731	-0.020835	0.023463	0.042045	0.048479	0.025803	
0.006442	0.076301	-0.013358	0.006084	-0.020821	-0.093926	
0.091513	-0.026752	-0.069393	0.093796	-0.013585	0.049821	
0.018867	-0.114758	0.037421	0.084655	-0.029635	-0.087931	
x80	-0.034972	0.023490	-0.033973	0.017282	-0.015001	-
0.076192	0.060719	-0.010633	0.003355	-0.071994	0.041026	
0.042163	0.062924	0.020444	-0.034062	0.042227	0.012135	
0.062121	0.023930	-0.033402	-0.086634	0.078416	-0.037756	-
0.025399	-0.036158	-0.021414	0.038567	-0.026239	-0.055875	
0.001040	0.009278	-0.096656	0.012754	-0.025110	0.004218	
y80	0.021083	0.000412	0.009133	-0.033379	-0.006135	-
0.013502	0.045151	0.102796	0.035234	-0.024068	0.048575	-
0.039302	0.089773	-0.032676	0.066593	-0.021196	0.044918	-
0.028455	0.011070	0.031256	0.122084	-0.022109	0.014427	
0.074058	-0.038212	-0.009967	0.004884	0.040230	0.009482	-
0.000219	0.067338	-0.038522	-0.005340	0.031603	-0.030111	
z80	0.002872	0.017867	-0.007092	0.000880	0.005205	-
0.018780	-0.010146	-0.002304	-0.088838	-0.057267	0.016935	-
0.021977	-0.031041	0.018156	0.033588	0.028413	0.032288	
0.013898	0.047821	-0.021268	-0.009997	-0.001986	-0.100892	
0.095538	-0.043571	-0.048579	0.099989	0.010847	0.016804	
0.064650	-0.094017	0.058386	0.071981	-0.027756	-0.067178	
x81	-0.028279	0.013007	-0.030684	0.005921	-0.015159	-
0.083948	0.065193	0.010527	-0.014497	-0.063069	0.036155	
0.040923	0.087788	0.003554	-0.025205	0.007266	0.013595	
0.048614	0.017198	-0.010844	-0.098333	0.085494	-0.027054	-
0.002627	-0.029659	-0.034606	0.033506	-0.011366	-0.049565	-
0.023143	0.017452	-0.068806	-0.009701	-0.022109	-0.009123	

y81	0.025062	0.000832	0.018367	-0.035351	-0.008719	-
0.014547	0.027733	0.090363	0.043085	-0.007338	0.054511	-
0.050668	0.075680	-0.013418	0.040889	0.015887	0.034257	-
0.038768	0.010858	0.060235	0.159212	-0.028773	0.009479	
0.102052	-0.062494	0.075227	0.011703	0.064851	0.075793	
0.046867	0.082515	-0.024479	-0.063542	-0.032847	-0.015624	
z81	0.003791	0.019115	-0.007129	-0.006564	0.013113	-
0.010316	-0.017835	0.009593	-0.095737	-0.069075	0.023295	-
0.018688	-0.046306	0.008748	0.029437	0.004484	0.037446	
0.023617	0.008117	-0.033118	-0.036753	0.025065	-0.091633	
0.102809	-0.057491	-0.007135	0.117075	0.040947	-0.005969	
0.129919	-0.037786	0.071657	0.017404	0.008594	-0.054939	
x82	-0.019364	0.005316	-0.022420	-0.003608	-0.015914	-
0.091221	0.068353	0.037473	-0.028604	-0.040098	0.033872	
0.031492	0.110030	-0.004240	-0.028897	0.006203	0.034176	
0.041422	0.015173	0.014037	-0.058085	0.094462	-0.027063	
0.016440	-0.019840	-0.038200	0.027950	0.002755	-0.046126	-
0.048755	0.021276	-0.053313	0.026237	-0.056291	0.090019	
y82	0.028454	0.004707	0.030153	-0.035429	-0.013237	-
0.015995	0.007222	0.077118	0.058528	0.010658	0.059681	-
0.061264	0.063926	0.015248	0.010291	0.064242	0.022365	-
0.048160	0.012412	0.092353	0.227066	-0.037685	-0.003226	
0.126300	-0.099181	0.154631	0.007690	0.087114	0.150246	
0.084994	0.091589	-0.006525	-0.089285	-0.134032	0.005236	
z82	0.005396	0.019650	-0.006492	-0.015208	0.019649	
0.000287	-0.025446	0.022404	-0.096751	-0.081554	0.028747	-
0.016155	-0.057983	0.000601	0.017376	-0.023554	0.038148	
0.031528	-0.034938	-0.050719	-0.051831	0.066849	-0.080723	
0.109389	-0.075710	0.029429	0.112991	0.069785	-0.036102	
0.194497	0.024052	0.081009	-0.043456	0.034906	-0.010205	
x83	-0.009345	0.000901	-0.015682	-0.010305	-0.019318	-
0.095592	0.065542	0.060394	-0.034993	-0.035003	0.050938	
0.009599	0.122642	-0.007996	-0.039008	0.008811	0.031939	
0.022402	0.011892	0.028622	-0.005851	0.103699	-0.031644	-
0.006989	-0.065692	-0.015189	-0.002767	0.027387	-0.005661	-
0.059919	0.044623	0.010817	0.013648	-0.035802	0.032893	
y83	0.030193	0.004434	0.032272	-0.036773	-0.002241	
0.000857	-0.016035	0.049004	0.052363	0.024477	0.040026	-
0.055542	0.034934	-0.009226	0.011514	0.068232	-0.006257	-
0.047204	0.018500	0.077882	0.163004	-0.016540	-0.007480	
0.069828	-0.064338	0.083609	-0.030674	0.028088	0.063978	
0.078981	0.078324	-0.023063	-0.015685	-0.061582	-0.001182	
z83	0.008828	0.017065	-0.007679	-0.022480	0.024707	
0.010423	-0.024653	0.036852	-0.087696	-0.072529	0.034025	-
0.034188	-0.063406	0.007777	0.020197	-0.006901	0.039408	
0.011677	-0.027210	-0.007511	-0.034349	0.037659	-0.066277	
0.080245	-0.064957	0.026560	0.066994	0.035771	-0.075138	
0.169559	0.020284	0.061872	-0.073304	0.024636	0.005839	
x84	-0.001027	-0.003586	-0.013325	-0.013949	-0.022084	-
0.104203	0.058177	0.065012	-0.039501	-0.020193	0.059695	-
0.009302	0.109665	-0.017613	-0.046526	0.011162	0.032034	
0.029490	0.018069	0.057772	0.035651	0.101661	-0.030605	-
0.024312	-0.065576	0.024951	-0.055550	0.050511	-0.026745	-
0.042781	0.051504	0.036562	0.019909	-0.056874	0.093955	
y84	0.029596	0.005271	0.032561	-0.036874	0.008455	
0.015841	-0.035511	0.021001	0.047404	0.036074	0.020274	-
0.049193	0.007858	-0.028871	0.014884	0.070245	-0.034373	-
0.043825	0.023359	0.059817	0.104087	-0.000525	-0.006673	
0.024021	-0.025503	0.020575	-0.062331	-0.021124	-0.011807	
0.079220	0.067405	-0.041516	0.034128	-0.004168	-0.001979	

z84	0.012194	0.014464	-0.006474	-0.027492	0.027498	
0.017522	-0.022103	0.045573	-0.074517	-0.061976	0.037242	-
0.045804	-0.064263	0.012128	0.021372	0.007050	0.041841	-
0.000532	-0.020227	0.028567	-0.017130	0.012696	-0.052056	
0.050026	-0.045028	0.019875	0.028141	0.009562	-0.109263	
0.134070	0.010793	0.039772	-0.085123	0.010302	0.026091	
x85	0.006980	-0.007571	-0.010740	-0.017623	-0.016350	-
0.103493	0.047136	0.065401	-0.052984	-0.002929	0.050267	-
0.023471	0.097031	-0.028252	-0.034561	0.011954	0.017903	
0.023859	0.028596	0.069404	0.048327	0.089252	-0.017014	-
0.015995	-0.050863	0.044329	-0.079733	0.022503	-0.030814	-
0.016403	0.100497	0.057345	-0.029843	-0.052955	0.026421	
y85	0.027834	0.006911	0.033074	-0.035767	0.016919	
0.028998	-0.050640	-0.001741	0.046066	0.039657	0.006425	-
0.041740	-0.016214	-0.041046	0.015778	0.072912	-0.053349	-
0.038502	0.023803	0.042915	0.056687	0.015361	-0.009844	-
0.015988	-0.003556	-0.026152	-0.081112	-0.057269	-0.064118	
0.071927	0.046909	-0.053629	0.077609	0.044910	0.010044	
z85	0.015028	0.012023	-0.005301	-0.029721	0.030872	
0.022966	-0.019032	0.053437	-0.065199	-0.049503	0.041377	-
0.056998	-0.065735	0.019718	0.022913	0.018539	0.039035	-
0.014343	-0.011922	0.047792	-0.011475	-0.004006	-0.034700	
0.027328	-0.033643	0.013922	0.006080	-0.015021	-0.121397	
0.104902	0.015175	0.025341	-0.119291	0.001389	0.010323	
x86	0.014066	-0.011329	-0.005578	-0.021393	-0.010384	-
0.098474	0.033646	0.064128	-0.056819	0.009438	0.041595	-
0.034814	0.072353	-0.039007	-0.031769	0.013153	-0.000450	
0.029478	0.034707	0.073833	0.052810	0.085880	-0.007658	-
0.028165	-0.040467	0.070742	-0.103803	0.017599	-0.060728	-
0.009358	0.089193	0.045599	-0.054272	-0.028363	-0.003413	
y86	0.024600	0.009563	0.032894	-0.032738	0.023805	
0.038988	-0.059991	-0.022513	0.045753	0.039486	-0.005653	-
0.031878	-0.031349	-0.046046	0.020154	0.075003	-0.064048	-
0.034261	0.021507	0.025425	0.024510	0.030288	-0.013450	-
0.039814	0.011137	-0.067333	-0.082892	-0.083529	-0.084596	
0.064802	0.038607	-0.049748	0.101264	0.073669	0.018298	
z86	0.017348	0.009446	-0.002582	-0.030196	0.033903	
0.026468	-0.014189	0.057511	-0.052272	-0.038530	0.044199	-
0.066284	-0.065757	0.022452	0.018361	0.028072	0.036749	-
0.018077	-0.014460	0.053111	-0.009461	-0.012649	-0.020784	
0.010287	-0.020282	0.009622	-0.006813	-0.023200	-0.134957	
0.074480	0.020605	0.007449	-0.127357	-0.010017	-0.010901	
x87	0.020141	-0.014744	-0.000602	-0.023318	-0.002273	-
0.094768	0.018913	0.054862	-0.062743	0.021977	0.033284	-
0.034819	0.055167	-0.056716	-0.027744	0.019814	-0.016466	
0.016792	0.052834	0.083339	0.048301	0.090704	-0.004034	-
0.047881	-0.029746	0.055365	-0.113969	-0.026160	-0.052630	-
0.000473	0.064127	0.068823	-0.054439	-0.020367	-0.014686	
y87	0.020333	0.013511	0.032122	-0.027567	0.029409	
0.048701	-0.064242	-0.040012	0.049157	0.033633	-0.019435	-
0.021728	-0.051251	-0.038381	0.027050	0.072722	-0.065692	-
0.016220	0.009311	0.005352	0.006452	0.042761	-0.014325	-
0.041057	0.021016	-0.076729	-0.075680	-0.082483	-0.095794	
0.048459	0.038088	-0.045800	0.109832	0.095453	0.034474	
z87	0.019252	0.006514	-0.000234	-0.029151	0.036856	
0.028321	-0.009103	0.058751	-0.040732	-0.028469	0.049017	-
0.072853	-0.060413	0.024540	0.017901	0.036861	0.035462	-
0.017053	-0.018934	0.048415	-0.012762	-0.017339	-0.003037	-
0.005078	-0.001996	0.011322	-0.015041	-0.035453	-0.139243	
0.038537	0.020854	-0.004915	-0.133535	-0.022002	-0.010818	

x88	0.025496	-0.017152	0.007940	-0.023670	0.007341	-
0.084849	0.005097	0.043450	-0.062146	0.032575	0.015352	-
0.022873	0.032979	-0.058551	-0.028709	0.008439	-0.028276	
0.024529	0.069428	0.096485	0.057584	0.083940	0.007521	-
0.040636	-0.002061	0.042676	-0.107502	-0.049632	-0.056900	-
0.027306	0.017322	0.061109	-0.032151	0.027118	-0.012716	
y88	0.014732	0.018131	0.028274	-0.021156	0.034151	
0.054467	-0.064462	-0.057039	0.050263	0.025458	-0.032294	-
0.015929	-0.067046	-0.032477	0.043370	0.086115	-0.065868	-
0.003285	-0.005761	-0.025514	-0.013524	0.062085	-0.016703	-
0.043942	0.016863	-0.070676	-0.059452	-0.081566	-0.078222	
0.053287	0.051661	-0.016709	0.086159	0.076466	0.043069	
z88	0.020740	0.003335	0.003754	-0.026175	0.039817	
0.029006	-0.001935	0.055733	-0.028022	-0.019317	0.050815	-
0.078827	-0.055387	0.024366	0.011328	0.039645	0.035860	-
0.011292	-0.032349	0.034233	-0.020258	-0.014843	0.014201	-
0.009560	0.020595	0.013328	-0.024942	-0.030929	-0.140260	-
0.004533	0.032069	-0.013882	-0.104467	-0.024073	-0.008074	
x89	0.030406	-0.018036	0.013146	-0.021984	0.019513	-
0.076147	-0.008214	0.025245	-0.062449	0.047841	-0.010493	-
0.016707	-0.000483	-0.063529	-0.016520	0.017045	-0.033059	
0.038335	0.073660	0.087588	0.055440	0.093973	0.031233	-
0.033246	0.017250	0.048899	-0.113715	-0.068248	-0.062105	-
0.050039	-0.002722	0.073109	0.012970	0.012949	-0.001756	
y89	0.007348	0.023104	0.025054	-0.012486	0.036824	
0.061957	-0.062449	-0.071312	0.054845	0.006750	-0.041438	
0.002423	-0.072714	-0.018185	0.053057	0.080343	-0.073045	
0.007552	-0.006479	-0.046642	-0.020342	0.073260	-0.028793	-
0.033779	0.006546	-0.071878	-0.002252	-0.090484	-0.030132	
0.055501	0.025841	0.021926	0.025886	0.102276	0.055273	
z89	0.021964	0.000438	0.006749	-0.020879	0.042798	
0.028591	0.006109	0.052163	-0.014989	-0.010989	0.052756	-
0.084903	-0.049063	0.022458	0.002240	0.042223	0.035205	
0.001690	-0.050363	0.003704	-0.030150	-0.002036	0.031977	-
0.010444	0.039216	0.025683	-0.030315	-0.021458	-0.135674	-
0.041645	0.045352	-0.011709	-0.068639	-0.032033	0.006518	
x90	0.034412	-0.018001	0.017895	-0.016418	0.033270	-
0.060757	-0.022652	-0.000743	-0.063942	0.052171	-0.044010	
0.002831	-0.037589	-0.061464	0.001128	0.021411	-0.055262	
0.061625	0.089807	0.079484	0.038707	0.107691	0.059225	-
0.014766	0.042204	0.056927	-0.078522	-0.095249	-0.041346	-
0.067213	-0.036531	0.107824	0.048727	0.075976	-0.007590	
y90	-0.001935	0.028007	0.020335	-0.003742	0.039584	
0.063416	-0.056161	-0.082718	0.063001	-0.005565	-0.047545	
0.016792	-0.076526	-0.003282	0.062202	0.074356	-0.062203	
0.011116	-0.024805	-0.080212	-0.003843	0.078275	-0.044791	-
0.020764	-0.010339	-0.075629	0.039068	-0.086238	0.019082	
0.041565	-0.005838	0.047273	-0.037661	0.037629	0.079275	
z90	0.022919	-0.002490	0.010441	-0.013208	0.045559	
0.025777	0.016647	0.043177	-0.004280	-0.005821	0.050041	-
0.086475	-0.042973	0.017384	-0.009588	0.042837	0.027507	
0.018014	-0.073435	-0.037277	-0.043152	0.016950	0.048644	-
0.005754	0.063317	0.032942	-0.025569	0.003709	-0.116692	-
0.078757	0.061549	-0.006892	-0.019796	-0.028443	0.009250	
x91	0.036647	-0.016651	0.019583	-0.007441	0.049260	-
0.042679	-0.038600	-0.036227	-0.064419	0.054303	-0.085336	
0.033521	-0.082459	-0.061384	0.023429	0.021501	-0.074744	
0.084562	0.093002	0.057243	0.028275	0.132570	0.072356	
0.007341	0.056600	0.058500	-0.031840	-0.143641	0.000597	-
0.100821	-0.099380	0.168604	0.081442	0.105517	-0.006956	

y91	-0.011661	0.034886	0.020577	0.002529	0.040105	
0.063914	-0.042712	-0.084059	0.077359	-0.030752	-0.045606	
0.022107	-0.082261	0.025390	0.055618	0.065660	-0.041130	
0.026456	-0.049031	-0.069500	0.009679	0.064437	-0.030325	
0.016866	0.005413	-0.051311	0.046030	-0.036178	0.046347	
0.033545	0.020324	0.031638	-0.061807	0.024439	0.145608	
z91	0.024601	-0.003024	0.014509	-0.004174	0.044328	
0.025084	0.028674	0.026224	0.012350	-0.020869	0.043587	-
0.085243	-0.051106	0.011203	-0.028527	0.030348	0.036705	
0.059113	-0.138758	-0.058367	-0.050809	0.044561	0.047785	-
0.003944	0.076803	0.065197	-0.042154	0.026309	-0.079631	-
0.125417	0.132378	0.025247	0.076823	-0.022812	0.030219	
x92	0.055759	0.016669	0.037637	0.001707	0.059862	-
0.018108	-0.031625	-0.034960	-0.055427	0.063137	-0.071023	
0.017000	-0.083439	-0.041166	0.024496	0.058852	-0.033725	
0.112308	0.042903	0.026007	0.024958	0.075506	0.017162	-
0.005712	0.015666	0.060946	0.026956	-0.073262	0.004395	-
0.067780	-0.017280	0.075743	0.050587	0.057716	0.017490	
y92	-0.017860	0.039964	0.017347	-0.000956	0.034170	
0.065022	-0.027047	-0.060604	0.085717	-0.067695	-0.028102	
0.008030	-0.048363	0.030743	0.050265	0.083622	-0.034431	
0.007415	-0.069192	-0.074167	0.019202	0.049389	-0.051053	-
0.024031	-0.029284	-0.050401	0.000700	-0.027951	0.057492	
0.015152	-0.004103	0.004228	-0.075492	0.002337	0.062317	
z92	0.021466	-0.012323	0.013491	-0.002504	0.042904	
0.021296	0.024074	0.026558	0.017112	-0.034251	0.040647	-
0.088832	-0.071061	0.026228	-0.046765	0.030659	-0.001551	
0.065354	-0.124656	-0.013371	-0.036267	0.038136	0.055986	
0.010809	0.113051	0.072639	-0.020727	0.033885	-0.039502	-
0.061353	0.056742	-0.004002	0.078470	-0.053539	0.000353	
x93	0.070187	0.045737	0.052754	0.008150	0.069021	
0.001411	-0.021867	-0.032318	-0.048810	0.066083	-0.058294	-
0.001234	-0.086975	-0.026003	0.029990	0.102625	0.002436	
0.126305	-0.001348	0.000221	0.019950	0.020707	-0.023986	-
0.015833	-0.013866	0.083862	0.076261	-0.011559	0.011211	-
0.057906	0.058856	-0.013382	0.020359	0.022446	0.042596	
y93	-0.029744	0.030304	0.017411	-0.012366	0.026049	
0.058519	-0.017629	-0.051556	0.095178	-0.097669	-0.014325	
0.017815	-0.021980	0.033353	0.045736	0.078945	-0.041595	-
0.002545	-0.068791	-0.060011	0.030251	0.041122	-0.055792	-
0.030756	-0.028454	-0.037290	-0.033148	-0.024737	0.030584	
0.015294	-0.012211	0.018344	-0.070547	-0.008982	-0.035282	
z93	0.018221	-0.020355	0.011378	0.000286	0.038899	
0.016892	0.021512	0.024909	0.021980	-0.048048	0.033934	-
0.094408	-0.080841	0.037402	-0.061360	0.021353	-0.031525	
0.064954	-0.106792	0.027748	-0.015804	0.027226	0.053344	
0.007491	0.132443	0.059861	-0.002505	0.027967	-0.003974	-
0.011487	-0.006323	-0.027179	0.075883	-0.062064	-0.030832	
x94	0.079281	0.064123	0.067364	0.007987	0.073088	
0.017532	-0.014366	-0.026333	-0.036558	0.056866	-0.047504	-
0.011374	-0.082489	-0.013226	0.027506	0.126017	0.020755	
0.127222	-0.027180	-0.017592	0.019515	-0.018471	-0.053344	-
0.025707	-0.040237	0.081542	0.113581	0.033117	0.016621	-
0.065888	0.105318	-0.066090	-0.004935	0.011124	0.049606	
y94	-0.045194	0.016806	0.011201	-0.025976	0.014821	
0.052220	-0.016249	-0.036961	0.103378	-0.124138	-0.002284	
0.025103	0.004012	0.031225	0.024551	0.060643	-0.051967	-
0.015498	-0.064443	-0.053737	0.047214	0.046591	-0.056354	-
0.050199	-0.063507	-0.031965	-0.041918	-0.016215	0.020829	
0.008419	-0.011013	0.064865	-0.092141	0.016497	-0.115084	

z94	0.015444	-0.027760	0.008518	0.004642	0.033132	
0.011385	0.022281	0.021087	0.024348	-0.060181	0.023374	-
0.099256	-0.083934	0.043820	-0.068095	0.005079	-0.051981	
0.060978	-0.083125	0.058654	0.000603	0.011929	0.046137	
0.004167	0.149196	0.037846	-0.000479	0.016699	0.032483	
0.022010	-0.042973	-0.030870	0.065069	-0.058520	-0.052966	
x95	0.082301	0.073617	0.080368	0.000726	0.070642	
0.030458	-0.010495	-0.016542	-0.019892	0.032831	-0.043843	-
0.014610	-0.066672	-0.004086	0.022706	0.122865	0.025139	
0.119814	-0.029494	-0.038655	0.024522	-0.041950	-0.060731	-
0.025789	-0.055215	0.047427	0.131435	0.049912	0.024983	-
0.061332	0.138339	-0.083703	-0.023460	-0.006366	0.047550	
y95	-0.062155	0.000003	0.002206	-0.036068	0.001436	
0.042463	-0.017571	-0.026186	0.108793	-0.152010	0.000389	
0.016582	0.025966	0.029583	0.022770	0.035365	-0.054274	-
0.015802	-0.033771	-0.059718	0.053573	0.058351	-0.029389	-
0.035616	-0.051981	-0.037181	-0.079246	-0.042109	0.003684	
0.047070	0.000970	0.060357	-0.068165	-0.056429	-0.123869	
z95	0.013007	-0.033927	0.005245	0.008692	0.026370	
0.006095	0.024125	0.013357	0.023077	-0.066698	0.014082	-
0.094217	-0.081626	0.043830	-0.074676	-0.011003	-0.063160	
0.055270	-0.068795	0.079098	0.013795	-0.013975	0.019506	-
0.002685	0.136592	0.028815	0.002153	0.010171	0.050169	
0.019237	-0.066800	-0.019950	0.051176	-0.021744	-0.069784	
x96	0.081637	0.078502	0.090930	-0.011995	0.065784	
0.038522	-0.006240	-0.009882	-0.005046	0.003784	-0.045547	-
0.012401	-0.050287	0.007327	0.012879	0.110753	0.023765	
0.109564	-0.014643	-0.053888	0.036628	-0.053404	-0.060152	-
0.034992	-0.063547	0.002210	0.125176	0.063476	0.015210	-
0.050205	0.134140	-0.077801	-0.050132	-0.033628	0.029265	
y96	-0.077545	-0.017672	-0.008017	-0.046435	-0.005783	
0.029740	-0.018738	-0.022714	0.105722	-0.166738	0.001607	
0.021888	0.024284	0.030269	0.013032	0.008733	-0.058515	-
0.016124	-0.007510	-0.051839	0.070011	0.077357	-0.030414	-
0.014958	-0.053600	-0.036986	-0.097544	-0.036993	-0.007129	
0.033755	-0.019459	0.068063	-0.075626	-0.088214	-0.119006	
z96	0.009827	-0.039895	0.001127	0.013590	0.018932	-
0.000295	0.028157	0.003591	0.020678	-0.070517	0.004455	-
0.094856	-0.073719	0.040304	-0.072804	-0.020213	-0.060418	
0.048564	-0.048004	0.087244	0.016118	-0.042153	-0.006491	-
0.018148	0.135705	0.023169	-0.005208	-0.003294	0.063153	
0.020055	-0.071631	-0.008605	0.042953	0.003186	-0.075758	
x97	0.077270	0.079065	0.100827	-0.029099	0.059738	
0.046402	-0.001310	-0.001450	0.011794	-0.029804	-0.046606	-
0.008567	-0.032806	0.022264	0.001162	0.091409	0.021164	
0.102796	0.007024	-0.060266	0.018312	-0.047472	-0.037695	-
0.038044	-0.040370	-0.060780	0.091846	0.059371	0.002445	-
0.048611	0.049622	-0.038239	-0.055712	-0.073679	-0.007310	
y97	-0.094460	-0.038578	-0.019424	-0.053976	-0.012969	
0.016709	-0.021809	-0.018073	0.101690	-0.168363	0.008429	
0.019659	0.010227	0.037241	0.011963	-0.021786	-0.058497	-
0.012516	0.010748	-0.040153	0.049246	0.090987	-0.031368	
0.019601	-0.020862	-0.042961	-0.098403	-0.041564	-0.002496	-
0.009506	-0.055306	0.073132	-0.052847	-0.096053	-0.084751	
z97	0.006403	-0.045556	-0.003471	0.018710	0.008886	-
0.008274	0.032940	-0.011285	0.015664	-0.074167	-0.008463	-
0.090053	-0.061105	0.028744	-0.069272	-0.028991	-0.044543	
0.042462	-0.020940	0.085739	0.022343	-0.075023	-0.052280	-
0.040667	0.130086	0.028940	-0.012117	-0.007321	0.061521	
0.012909	-0.048446	0.004513	0.024122	0.044676	-0.066442	

x98	0.068179	0.073353	0.107026	-0.048786	0.048017	
0.045469	0.007004	0.005228	0.028591	-0.074344	-0.052845	-
0.001475	-0.007557	0.036068	-0.009457	0.075851	-0.005440	
0.084222	0.046526	-0.050791	0.032236	-0.055755	-0.018322	-
0.053266	-0.022596	-0.139334	0.042883	0.072671	-0.032857	-
0.059948	-0.013790	-0.001791	-0.038501	-0.064660	-0.008140	
y98	-0.114117	-0.063708	-0.035735	-0.059603	-0.025688	-
0.002108	-0.030749	-0.017078	0.090777	-0.162156	0.013133	
0.011274	-0.010057	0.040588	0.017062	-0.039338	-0.065136	-
0.013689	0.041551	-0.026834	0.045701	0.082658	-0.046367	
0.046101	0.000666	-0.025953	-0.066975	-0.017496	0.005071	-
0.074057	0.003358	0.042341	-0.005769	-0.045736	0.014943	
z98	0.002241	-0.051792	-0.007055	0.022990	-0.000857	-
0.016461	0.038800	-0.028341	0.014214	-0.073713	-0.019155	-
0.083972	-0.051469	0.014363	-0.065315	-0.045491	-0.008634	
0.036398	0.008542	0.079535	0.018785	-0.100516	-0.091224	-
0.055479	0.128408	0.045114	-0.018983	-0.020792	0.046771	-
0.008970	-0.039538	0.021165	0.000556	0.059806	-0.050303	
x99	0.051130	0.056331	0.110898	-0.074090	0.037675	
0.042655	0.010889	0.015068	0.046852	-0.127815	-0.054496	-
0.001025	0.001159	0.033396	-0.025527	0.064558	-0.025506	
0.037543	0.090680	-0.041726	0.046754	-0.052570	0.033681	-
0.070718	-0.003447	-0.123763	0.021811	0.044781	-0.087963	-
0.077898	-0.105781	-0.015484	-0.024473	-0.030612	-0.033773	
y99	-0.136372	-0.097347	-0.054384	-0.064873	-0.032975	-
0.018974	-0.053182	-0.012706	0.078936	-0.146896	0.029661	-
0.004783	-0.053764	0.035942	0.019510	-0.047377	-0.059949	-
0.024444	0.068061	-0.016229	0.039403	0.101403	-0.025097	
0.089380	0.011080	0.062982	0.007916	-0.014531	0.039934	-
0.168662	0.045082	-0.019372	0.065204	0.006709	0.081288	
z99	0.001320	-0.052280	-0.015534	0.032513	-0.023472	-
0.028600	0.051191	-0.051294	-0.001389	-0.061674	-0.054858	-
0.069559	-0.015463	0.003834	-0.050312	-0.103624	0.043753	
0.041366	0.065060	0.043691	0.009468	-0.159774	-0.212335	-
0.087360	0.116222	0.000634	-0.070592	-0.045623	0.049326	-
0.020475	0.083011	0.087405	-0.098623	0.121786	0.021812	
x100	0.027671	0.030219	0.109459	-0.100948	0.008468	
0.030685	0.002359	0.023847	0.061894	-0.167307	-0.059730	-
0.003197	0.016957	0.032132	-0.031271	0.018566	-0.032845	-
0.010479	0.180114	-0.049330	0.032608	-0.073335	0.041504	-
0.071381	0.005858	-0.125243	0.010613	0.011737	-0.065429	-
0.152138	0.001963	0.014835	-0.047739	-0.004972	-0.080076	
y100	-0.130270	-0.166659	-0.071583	-0.034213	-0.034298	-
0.033962	-0.074115	-0.009740	0.061243	-0.117171	0.046872	-
0.015265	-0.071538	0.040640	0.021677	-0.073301	-0.019243	-
0.019423	0.113181	-0.031828	-0.012565	0.105670	-0.058871	
0.136302	0.025718	0.134870	0.069570	-0.015985	0.076505	-
0.254294	0.192928	-0.009028	0.126633	0.086407	0.103306	
z100	-0.030864	-0.012324	-0.029119	0.000630	-0.044075	-
0.039144	0.063484	-0.072287	-0.011798	-0.054070	-0.069879	-
0.071025	-0.013734	-0.020128	-0.031706	-0.102183	0.025929	
0.023979	0.064992	0.059358	0.041870	-0.178742	-0.171632	-
0.111386	0.082742	-0.020320	-0.119940	-0.057081	0.055071	-
0.045443	0.075586	0.085053	-0.067255	0.140819	0.036674	
x101	0.004742	-0.041918	0.118092	-0.122135	-0.038015	
0.032182	-0.009451	0.018891	0.061423	-0.181091	-0.025788	-
0.014670	0.012986	0.019529	-0.029027	0.005510	-0.030949	-
0.026501	0.182557	-0.021643	-0.018201	-0.034374	0.042196	-
0.017554	0.015832	-0.008421	0.032217	-0.030944	-0.053048	-
0.053736	-0.003303	-0.026191	-0.019628	-0.021734	0.022709	

y101	-0.151643	-0.170039	-0.098481	-0.003780	-0.048956	-
0.017761	-0.087743	-0.012686	0.007272	-0.016365	0.028341	
0.000504	-0.070343	-0.004996	0.045347	-0.033580	0.013486	-
0.004012	0.006690	-0.017957	0.032477	0.045282	-0.068193	
0.052210	0.065460	0.020709	0.049484	0.057164	0.048753	-
0.151609	0.128547	-0.050172	0.016731	-0.009372	0.073564	
z101	-0.044460	0.018593	-0.038042	-0.002284	-0.054809	-
0.048928	0.056580	-0.087299	-0.039369	-0.019619	-0.062219	-
0.081239	-0.007312	-0.007672	-0.012162	-0.100820	0.018430	
0.012199	0.024199	0.011697	0.040268	-0.180230	-0.159129	-
0.129055	0.008488	-0.083800	-0.080820	-0.091449	0.038608	
0.038408	0.151494	0.050283	-0.034901	0.020934	0.060813	
x102	-0.021326	-0.101654	0.120649	-0.130442	-0.088679	
0.050070	-0.031424	0.014833	0.039492	-0.143040	-0.006112	-
0.030594	-0.001296	-0.032311	-0.000906	-0.009468	-0.016455	-
0.051490	0.115471	-0.013289	-0.032908	-0.052809	0.052010	-
0.033397	0.041591	0.006676	0.048737	-0.002686	-0.031013	
0.054600	-0.001827	0.004526	-0.019320	-0.055608	0.096367	
y102	-0.153604	-0.170130	-0.119640	0.035233	-0.042149	-
0.010715	-0.082179	-0.015073	-0.033691	0.059095	-0.004134	
0.018654	-0.040281	-0.033713	0.046933	-0.007240	0.034465	
0.014558	-0.058894	-0.013612	0.041103	0.010934	-0.093490	-
0.007958	0.051571	-0.005716	0.079778	0.075510	0.012588	-
0.052801	0.053095	-0.010960	-0.057115	-0.007154	0.078075	
z102	-0.046235	0.032136	-0.038391	0.001159	-0.054394	-
0.061904	0.060990	-0.099019	-0.050238	-0.006263	-0.039613	-
0.107669	-0.008604	0.009021	-0.000119	-0.064047	-0.023163	-
0.026024	-0.014642	-0.007553	0.017092	-0.117052	-0.083175	-
0.090843	-0.081233	0.002721	-0.025848	-0.141209	0.034673	
0.053248	0.126184	0.025995	0.058573	-0.019776	0.060325	
x103	-0.043102	-0.152759	0.116051	-0.129114	-0.113184	
0.059268	-0.046170	0.013605	0.014522	-0.105601	0.005450	-
0.037616	-0.008568	-0.056873	0.013185	0.010027	-0.009485	-
0.069951	0.035493	0.012538	-0.044259	-0.020875	0.079283	-
0.035236	0.024528	0.056225	0.088564	-0.006732	-0.002168	
0.100487	-0.055604	-0.001808	0.020853	-0.054899	0.014389	
y103	-0.156453	-0.149744	-0.138629	0.060459	-0.010166	-
0.014295	-0.072893	-0.016006	-0.040433	0.081369	-0.008886	
0.027496	-0.023595	-0.041446	0.038928	0.052185	0.016665	
0.022870	-0.075582	0.008854	0.046002	0.011994	-0.013656	
0.014625	0.003837	0.004621	0.101470	0.076759	-0.038953	-
0.022505	-0.085301	-0.000359	0.002595	-0.046574	-0.014200	
z103	-0.049222	0.045915	-0.036228	-0.000057	-0.047120	-
0.067436	0.055519	-0.099186	-0.044791	-0.005849	-0.002162	-
0.123302	-0.026450	0.011151	0.009541	-0.020070	-0.056955	-
0.058394	-0.039385	-0.008840	0.029939	-0.065816	0.043522	-
0.051506	-0.123587	0.045361	0.018984	-0.125560	0.031501	
0.029941	0.049968	-0.008702	0.152748	-0.064027	0.010602	
x104	-0.060958	-0.184984	0.100989	-0.120530	-0.125651	
0.053611	-0.052531	0.013239	-0.011018	-0.068610	0.001783	-
0.036922	-0.004998	-0.063502	0.024843	0.017144	0.002794	-
0.067824	0.004213	0.020508	-0.063026	-0.016001	0.068643	-
0.013994	0.000119	0.093472	0.097877	-0.026860	-0.006637	
0.136933	-0.043799	-0.010691	0.048792	-0.034431	-0.012786	
y104	-0.148846	-0.122669	-0.156458	0.086745	0.018254	-
0.024363	-0.059624	-0.023569	-0.048126	0.095444	-0.024025	
0.026722	0.001965	-0.026217	0.032552	0.067266	0.018417	
0.046352	-0.045319	-0.006396	0.044048	-0.014080	-0.015054	
0.015754	-0.040265	-0.011089	0.082320	0.082153	-0.096738	-
0.001116	-0.108253	0.031848	-0.021376	-0.073598	-0.016847	

z104	-0.045413	0.050731	-0.035144	0.006091	-0.042752	-
0.071293	0.050485	-0.096522	-0.048137	-0.002732	0.016820	-
0.132461	-0.032456	0.021671	0.019242	-0.010427	-0.060248	-
0.069044	-0.050015	-0.024088	0.048917	-0.037233	0.085598	-
0.049598	-0.139624	0.015616	0.037443	-0.093202	0.040178	
0.003894	0.030870	-0.021822	0.153397	-0.075875	-0.002823	
x105	-0.075933	-0.208592	0.081007	-0.105753	-0.125368	
0.044674	-0.053945	0.010919	-0.032269	-0.035617	-0.007654	-
0.039702	-0.001248	-0.056095	0.033137	0.025006	0.017626	-
0.059106	-0.005261	0.020097	-0.075232	-0.028289	0.052133	
0.001101	-0.042968	0.077421	0.078673	-0.021369	-0.039561	
0.136851	-0.044196	-0.024932	0.081623	-0.047165	-0.016206	
y105	-0.139303	-0.089398	-0.171447	0.105319	0.042074	-
0.034719	-0.054554	-0.035319	-0.048614	0.101312	-0.028278	
0.025502	0.017669	-0.011428	0.027983	0.066858	0.022261	
0.072069	0.005242	-0.025077	0.052842	-0.027756	-0.010019	
0.005361	-0.027722	-0.015129	0.058777	0.094496	-0.107613	
0.004451	-0.093394	0.030098	-0.081752	-0.066901	0.004955	
z105	-0.039743	0.047824	-0.034139	0.012402	-0.039082	-
0.076555	0.043196	-0.085333	-0.052204	-0.004669	0.035442	-
0.133301	-0.037249	0.031237	0.022984	0.000289	-0.058216	-
0.075775	-0.059021	-0.032164	0.066527	0.002586	0.121841	-
0.046720	-0.122276	-0.029745	0.042361	-0.059440	0.065180	-
0.039970	0.013731	-0.038557	0.131776	-0.072551	0.002190	
x106	-0.062816	-0.260861	0.057627	-0.053387	-0.102679	
0.033781	-0.055630	0.011262	-0.052277	-0.005333	-0.003538	-
0.042595	0.023139	-0.030480	0.042639	0.060079	0.061863	-
0.047838	-0.004306	0.018007	-0.092738	-0.032920	0.023226	
0.006301	-0.080305	0.043034	0.040021	-0.010642	-0.045275	
0.053648	-0.043245	-0.004050	0.061946	-0.003295	-0.053109	
y106	-0.158043	-0.003654	-0.190873	0.084974	0.051765	-
0.047282	-0.045350	-0.044355	-0.042673	0.103219	-0.045868	
0.016549	0.008642	0.002481	0.018338	0.045330	0.001208	
0.092383	0.063786	-0.042325	0.082558	-0.070157	0.014357	-
0.016004	0.033298	-0.004906	0.020390	0.076238	-0.097342	
0.037226	-0.048953	-0.019263	-0.096614	-0.066818	0.064742	
z106	-0.035417	0.039923	-0.031649	0.017156	-0.034362	-
0.078926	0.040488	-0.066195	-0.057480	-0.005433	0.054824	-
0.123967	-0.040077	0.036450	0.013759	0.009372	-0.058404	-
0.074928	-0.064674	-0.040671	0.073026	0.042771	0.130889	-
0.037392	-0.085279	-0.095833	0.015598	-0.034526	0.084253	-
0.100807	-0.001126	-0.047644	0.088972	-0.061892	0.064274	
x107	-0.083314	-0.259000	0.007385	-0.031467	-0.091552	
0.009343	-0.050536	0.007388	-0.084513	0.046632	-0.019656	-
0.037053	0.012092	0.018620	0.053951	0.147036	0.068911	-
0.024542	0.005782	0.046222	-0.090490	-0.111439	-0.006718	-
0.012356	-0.052769	-0.042573	-0.040647	-0.015093	-0.018123	-
0.028917	-0.008781	-0.056042	0.034287	0.071832	-0.031120	
y107	-0.135233	0.054891	-0.195863	0.107476	0.088567	-
0.053615	-0.032913	-0.054161	-0.008399	0.076810	-0.069352	
0.017381	0.036498	0.022909	-0.000076	0.022002	-0.006425	
0.076667	0.129401	-0.078944	0.108602	-0.080080	0.052683	-
0.084162	0.074818	0.030098	0.030225	0.018916	-0.082667	
0.106209	-0.045486	-0.007464	-0.092372	-0.007602	-0.046913	
z107	-0.011401	-0.001898	-0.019704	0.044603	-0.020440	-
0.072960	0.031945	-0.046257	-0.063675	-0.010414	0.077129	-
0.103050	-0.026277	0.028564	-0.012125	0.028593	-0.053949	-
0.072718	-0.072505	-0.036857	0.079195	0.112508	0.107949	-
0.024369	-0.037209	-0.196190	-0.005093	0.000654	0.094661	-
0.145597	-0.056137	-0.026907	-0.002092	0.020579	0.069331	

```
   x108    -0.105542  -0.215834  -0.072302  -0.006329  -0.066415  -
0.017959   -0.017059   0.001570  -0.130531   0.123622  -0.054692  -
0.005325    0.031240   0.108747   0.056135   0.325105   0.029009
0.017280   -0.029680   0.106158  -0.081531  -0.209976   0.004783  -
0.070551   -0.016536  -0.233604  -0.223948  -0.075297   0.100065  -
0.020628    0.098938  -0.108018  -0.145449   0.150151  -0.144385
   y108    -0.076744   0.115492  -0.179143   0.148489   0.169563  -
0.078042    0.002734  -0.066439   0.032760  -0.011668  -0.079675  -
0.039502    0.140456   0.071263  -0.026129  -0.146096   0.001567
0.032563    0.257727  -0.162242   0.075533  -0.010030   0.169091  -
0.057853   -0.093158   0.129244   0.062112  -0.107107  -0.071231
0.128105    0.131657   0.116128   0.047882   0.050274  -0.144760
   z108     0.006360  -0.028900   0.002730   0.054878  -0.007186  -
0.056606    0.010322  -0.025625  -0.079348  -0.002081   0.116306  -
0.106141   -0.023367  -0.020011  -0.036331  -0.054800  -0.036534  -
0.066604   -0.100046  -0.009270   0.144094   0.214657   0.152068
0.013458   -0.026749  -0.305090   0.032426   0.102598   0.131664  -
0.151079   -0.093813  -0.050207  -0.148917   0.144014   0.078164
```

Canonical Variate Analysis: CVA ... cochlea mid line

Dataset: cochlea mid line
Classification criterion: subfam

Groups		Observations
1.	Ca	9
2.	Ce	13
3.	Di	3
4.	Pr	11

Variation among groups, scaled by the inverse of the within-group variation

	Eigenvalues	% Variance	Cumulative %
1.	5.51806160	67.001	67.001
2.	2.14650957	26.063	93.064
3.	0.57123652	6.936	100.000

Mahalanobis distances among groups:

	Ca	Ce	Di
Ce	4.5796		
Di	3.6107	5.1605	
Pr	3.8801	5.1907	3.0145

P-values from permutation tests (10000 permutation rounds) for Mahalanobis distances among groups:

	Ca	Ce	Di
Ce	<.0001		
Di	0.8467	0.0018	
Pr	<.0001	<.0001	0.9995

Procrustes distances among groups:

	Ca	Ce	Di
Ce	0.1257		
Di	0.0771	0.1078	
Pr	0.0581	0.1379	0.0826

P-values from permutation tests (10000 permutation rounds) for Procrustes distances among groups:

	Ca	Ce	Di
Ce	0.0033		
Di	0.6884	0.0656	
Pr	0.8334	<.0001	0.4712

Canonical coefficients:

	CV1	CV2	CV3
x1	-58.2717	17.5038	-11.3995
y1	-18.4490	-1.4276	-23.7005
z1	-46.3008	32.7978	17.9728
x2	-14.6811	-10.2524	-6.1890
y2	-1.3656	2.3682	-16.6641
z2	-19.8016	8.3861	16.0637
x3	9.0612	-25.0756	-1.8956
y3	-0.4673	0.0671	-5.2613
z3	-7.6146	-3.9766	12.4654
x4	14.7127	-27.1437	-1.5489
y4	2.5228	-5.4786	4.0264
z4	9.8396	-17.6978	9.2696
x5	23.1621	-20.4004	-4.4825
y5	4.9422	-1.9934	9.8042
z5	21.8430	-23.2642	6.0819
x6	13.7896	-12.4086	-2.8939
y6	10.5499	-3.5539	13.2979
z6	30.6045	-25.6735	2.2127
x7	-7.4497	6.5873	-0.9946
y7	13.4786	1.8712	13.3402
z7	30.0515	-21.9289	-1.3024
x8	8.2595	-14.7783	2.1420
y8	11.2813	0.9415	13.0772
z8	19.1420	-4.1490	-3.3330
x9	-55.2457	29.1288	-1.1142
y9	25.6725	-7.8664	11.9844
z9	-9.8082	31.1771	-10.3569
x10	-11.9721	14.1475	2.0678
y10	-2.5431	26.3468	1.0136
z10	-2.8549	23.3002	-7.0954
x11	-13.7126	1.8828	5.9001
y11	-6.1295	21.7744	0.3588
z11	-13.0973	15.1102	-8.1764
x12	-5.3809	2.1555	3.8050
y12	-7.7263	12.3084	2.3641
z12	-22.8285	8.4598	-7.9035
x13	-3.2568	3.7093	2.0077
y13	-11.7933	6.3839	2.7021
z13	-27.2128	-4.5399	-5.1472
x14	6.7543	-5.1249	0.4703
y14	-13.9760	-1.3567	3.1103
z14	-38.3026	-0.1114	-3.4628
x15	7.6035	-10.4258	1.3745
y15	-16.7103	-5.9956	3.0556
z15	-34.8070	2.8906	-4.3861
x16	17.5680	-17.5298	0.0402
y16	-21.1042	-5.3570	1.8413
z16	-32.0455	1.8272	-2.9959

x17	25.1254	-15.3686	-3.3557
y17	-24.0472	-7.1047	1.2606
z17	-28.3135	4.2542	-2.1409
x18	34.7027	-6.0533	-6.8056
y18	-28.9592	-4.7303	-0.9638
z18	-21.0065	3.6096	-1.3324
x19	21.7304	7.0883	-8.8449
y19	-28.9241	-5.3365	-2.2001
z19	1.2689	3.7673	-0.2757
x20	12.2443	2.2841	-7.8562
y20	-2.6337	-0.4818	-3.3668
z20	5.3980	7.8700	-2.5575
x21	10.0177	2.4395	-4.9797
y21	22.4827	2.3172	-7.2181
z21	14.1951	2.7391	-5.4715
x22	15.3337	-2.8392	-3.2953
y22	32.6792	8.1740	-9.1095
z22	12.3771	4.4049	-6.5351
x23	20.3166	5.7818	-3.3317
y23	31.8443	4.4453	-7.2837
z23	6.1762	1.4405	-4.3568
x24	23.5459	4.6659	-5.6341
y24	33.3313	-0.7661	-4.4717
z24	7.2257	-2.6245	-3.3298
x25	12.8095	5.3453	-2.1222
y25	43.3878	-8.2111	-6.1385
z25	15.9788	-6.9581	-4.6279
x26	6.5575	5.6309	-2.0290
y26	39.7330	-16.3200	-2.4968
z26	17.0923	-8.3758	-1.2842
x27	-9.9629	10.1423	-0.4023
y27	39.8337	-29.0977	1.6866
z27	26.3589	-14.5656	1.1141
x28	-25.2392	10.2464	2.2220
y28	25.2366	-33.7545	4.8636
z28	34.5482	-17.6644	3.1916
x29	-4.9158	-1.9770	2.7719
y29	19.5034	-35.7812	12.7783
z29	10.6921	-17.0175	5.3313
x30	-2.9241	-8.9798	8.6116
y30	8.0285	-20.8663	8.9346
z30	-4.9876	-12.3097	2.6449
x31	-5.4776	-8.9753	10.8585
y31	3.5825	-34.6772	10.9649
z31	-15.3514	-5.8339	1.1985
x32	-5.5541	-10.7739	10.6773
y32	-7.3256	-15.9409	7.5350
z32	-22.0882	0.4862	0.5082
x33	-3.9775	-10.8316	8.5371
y33	-23.0094	-6.7462	4.7968
z33	-18.3956	6.3009	0.3949
x34	0.6358	-1.4547	2.4879
y34	-31.9105	10.0737	4.3416
z34	-15.5378	10.8358	-0.0122
x35	2.9278	9.5161	-4.9489
y35	-24.3795	24.5571	-1.4777
z35	-11.2997	19.0324	-0.3331
x36	5.1087	20.8745	-13.4563
y36	-15.0631	26.1264	-3.5210
z36	-4.6100	30.2651	-1.3458

x37	-0.0459	30.9521	-20.4485
y37	-16.3237	16.0637	1.4059
z37	11.1154	41.6320	-3.9286
x38	-9.5717	14.6885	-6.7766
y38	-9.3214	5.8941	-0.8556
z38	22.5412	32.3794	-5.9551
x39	-22.2212	7.1706	1.2112
y39	-4.6048	-0.5541	-5.6065
z39	27.0716	25.8350	-5.6356
x40	-23.2954	-1.3304	5.6649
y40	0.7610	-3.2609	-9.4726
z40	23.0795	21.7816	-3.3358
x41	-2.1278	-21.6329	5.5526
y41	10.4700	-7.7521	-13.7515
z41	21.1502	19.1046	-2.1890
x42	4.5085	-29.9699	4.3572
y42	9.4029	-4.5245	-16.1977
z42	20.5438	15.1754	-1.2287
x43	1.9791	-9.7587	-0.6426
y43	6.2883	-5.5755	-15.9530
z43	18.4132	5.5925	0.1576
x44	10.5662	-12.1688	-3.4137
y44	0.2030	-10.5936	-12.9509
z44	13.1743	-2.5267	1.1367
x45	3.4320	-10.0873	-2.2774
y45	-4.2495	-15.8921	-9.6148
z45	12.1208	-11.8656	1.6756
x46	5.6731	-15.3131	1.0098
y46	-8.7487	-13.4296	-9.6177
z46	11.1746	-21.3266	2.0930
x47	-0.6537	-5.9445	0.2602
y47	-4.4746	-11.6256	-0.9945
z47	-0.6083	-6.6884	-1.7035
x48	-9.6956	-1.7021	4.4770
y48	5.4470	-13.6153	2.1513
z48	-5.6699	3.8721	-4.7776
x49	-13.1315	-2.5217	8.7451
y49	7.8278	-14.6884	3.3687
z49	-10.3056	9.2118	-6.4290
x50	-17.1378	-5.7874	13.2358
y50	6.6914	-7.9906	-0.2984
z50	-15.2208	13.4665	-7.7665
x51	-19.7325	-9.9686	15.7870
y51	18.3754	6.7504	-5.8963
z51	-14.3567	12.2974	-8.3458
x52	-14.3276	-10.8234	15.3893
y52	12.1787	22.4432	-9.5166
z52	-8.7123	6.4794	-6.9480
x53	-8.2060	-8.9714	13.0754
y53	5.3352	13.7626	-10.6700
z53	-0.8395	2.4808	-5.8048
x54	0.7988	-6.7714	9.6259
y54	6.8873	16.9735	-12.7549
z54	3.9881	-2.1891	-3.8341
x55	16.3758	-9.2906	6.3347
y55	2.6010	8.8193	-12.1907
z55	7.5830	-8.3404	-1.2865
x56	14.8820	-2.5780	4.1740
y56	-0.3329	9.5549	-10.0098
z56	14.9013	-1.7797	-2.7617

x57	12.6501	-8.4832	5.7925
y57	-4.5812	-6.9501	-3.5858
z57	24.2472	-1.8456	-2.5614
x58	17.9606	-15.4395	4.6988
y58	4.9868	-22.1725	-1.6340
z58	27.3802	1.1843	-3.1449
x59	19.0773	-26.2464	6.9718
y59	5.5238	-38.5882	4.4692
z59	31.2836	2.3269	-3.4452
x60	31.0906	-20.7433	-0.2550
y60	9.8396	-33.3592	2.8179
z60	25.2591	3.7942	-1.3367
x61	43.4297	-22.6530	-2.3917
y61	9.6289	-28.7112	5.8134
z61	20.4314	4.0018	0.6457
x62	26.2183	-14.6731	-0.2344
y62	-6.5307	-16.1303	10.6077
z62	24.4548	0.2528	1.3758
x63	0.1573	-7.1805	4.2567
y63	-21.3790	-0.4821	14.8869
z63	25.6328	-3.5051	2.2582
x64	-20.2808	3.6484	6.7863
y64	-34.5094	19.7075	18.2815
z64	19.8947	-7.5522	5.0537
x65	-38.3824	1.7431	9.1642
y65	-16.2737	17.7885	10.9483
z65	8.2539	-4.4695	0.2846
x66	-39.5118	8.5193	6.3902
y66	-2.8217	15.8645	4.2216
z66	-3.3783	-2.9023	-2.1141
x67	-37.4064	9.5173	1.7119
y67	6.1662	13.4854	-0.2820
z67	-15.6294	-0.1034	-1.7908
x68	-44.1119	14.6088	-1.1123
y68	13.5218	10.4877	-3.7857
z68	-22.3593	0.5059	-0.1154
x69	6.0982	12.7242	-14.9974
y69	2.0001	8.2717	-2.0498
z69	-54.6317	0.6076	9.9873
x70	-45.9262	12.5253	-3.4033
y70	20.1876	3.6004	-8.4059
z70	-8.9406	0.7230	1.7635
x71	-12.4467	7.6115	-11.1131
y71	-25.5638	2.7363	-0.0323
z71	-38.2436	2.8138	12.1441
x72	-26.6017	3.9265	-7.5255
y72	-26.3666	-3.5733	-1.0939
z72	-11.5605	3.1427	11.6354
x73	-20.2759	-2.8817	-8.9656
y73	10.4557	-2.8810	-8.1632
z73	36.4150	5.1116	7.3087
x74	-11.7669	4.1529	-7.7722
y74	1.8407	11.1084	-7.3056
z74	19.1782	-2.3300	6.1854
x75	-0.0351	6.9494	-7.0204
y75	20.1456	10.0414	-9.2460
z75	11.5544	-8.4584	3.7983
x76	11.1485	11.3917	-6.8176
y76	28.5716	23.3690	-11.0676
z76	1.9171	-9.2090	2.1019

x77	11.1651	14.2135	-5.7444
y77	14.6036	25.9066	-7.5707
z77	-5.9629	-9.8612	1.0906
x78	16.0120	8.1513	-7.3502
y78	13.6935	20.2502	-9.4403
z78	-13.7406	-9.6901	1.5459
x79	8.5089	5.2123	-6.3700
y79	-3.1519	20.9780	-3.9360
z79	-14.8069	-9.4754	0.8951
x80	-5.3226	0.9534	-1.7142
y80	-17.3779	16.4929	5.7387
z80	-15.4567	-8.3388	0.3379
x81	-7.9048	2.7182	-3.0429
y81	-12.0085	9.2471	10.1408
z81	-19.0296	2.3466	0.7124
x82	13.7073	-5.1964	-4.3752
y82	-6.5256	-4.8954	16.3524
z82	-17.3502	10.0126	1.9976
x83	-0.2278	5.5771	-3.4466
y83	-10.5103	1.2148	9.0869
z83	-12.9667	8.3375	2.2750
x84	16.9790	1.0686	-3.9614
y84	-12.3388	4.4614	3.5683
z84	-6.8232	5.2448	1.9245
x85	-0.9473	6.2896	-0.2655
y85	-9.6812	7.5854	-1.4767
z85	-9.5660	4.4843	3.2984
x86	-6.3958	11.7067	-1.0267
y86	-8.0560	9.1603	-3.9176
z86	-15.3497	3.1305	4.1118
x87	-6.0340	10.1124	-2.4901
y87	-3.9314	12.3997	-4.8498
z87	-14.7431	-0.0685	4.4585
x88	0.0588	16.2929	-7.5325
y88	-2.1894	9.1648	-1.5128
z88	-14.5792	-1.9315	3.7373
x89	5.1364	9.7952	-7.3150
y89	5.7545	14.8848	-2.4738
z89	-12.0316	-5.8644	3.1346
x90	12.6926	18.1771	-12.6852
y90	11.3593	2.8929	3.3374
z90	-12.0172	-7.8285	1.8603
x91	20.3152	19.7523	-16.5886
y91	29.3106	1.6520	5.6016
z91	-11.3558	-3.0355	0.6007
x92	17.6398	20.7083	-7.0286
y92	6.2389	1.7245	9.4878
z92	-10.3752	-15.1789	-1.5214
x93	16.9394	23.5575	1.1153
y93	-20.1554	3.5188	9.3043
z93	-10.9014	-21.1053	-3.5200
x94	14.5100	28.0861	5.4932
y94	-40.8215	13.9352	7.1522
z94	-9.1326	-23.3570	-4.5720
x95	11.1154	26.0152	9.7784
y95	-45.3869	-5.8385	12.3781
z95	-7.8563	-14.1909	-7.8766
x96	6.0079	19.2143	13.4266
y96	-43.1350	-12.6349	12.9712
z96	-4.4257	-8.0368	-9.2995

x97	2.5474	1.3871	13.3481
y97	-29.5449	-19.2611	9.1690
z97	1.3172	5.7596	-10.6813
x98	9.2224	-6.7438	9.5571
y98	-0.2370	-8.5281	3.7975
z98	7.9729	13.8337	-11.0489
x99	6.7433	-9.0897	3.8148
y99	19.6217	4.1817	-1.6503
z99	29.2990	39.3509	-7.5654
x100	1.5647	2.3378	6.1578
y100	27.4255	30.9502	-5.7045
z100	35.9041	39.2962	-6.9444
x101	22.8584	-8.2884	1.7859
y101	9.9110	10.7647	3.7031
z101	22.8006	9.5071	6.4542
x102	35.2801	-24.9302	1.9601
y102	6.9653	11.0912	0.5736
z102	10.8091	-8.7759	6.3121
x103	5.4773	-26.0936	-0.3442
y103	-14.6992	-5.0608	-3.8813
z103	-5.3484	-30.2307	6.3294
x104	-8.7797	-19.2445	-1.6822
y104	-11.5474	-11.6193	-2.8065
z104	-10.5407	-36.7855	9.4222
x105	-14.0035	-21.1142	-0.4098
y105	2.8075	-10.1837	-0.3590
z105	-9.7033	-39.0850	11.5076
x106	-22.0446	-5.0865	-0.4462
y106	28.0726	-15.4965	3.4049
z106	5.8270	-39.8848	11.0898
x107	-7.6148	15.3919	-0.1189
y107	8.4655	-6.7532	4.4164
z107	5.9057	-22.2019	4.6490
x108	-28.3058	43.1226	11.6019
y108	-13.7601	-1.4698	10.1610
z108	5.3967	2.6779	1.4308

New Procrustes fit:
Dataset 'canaux'

111 landmarks in 3 dimensions.
The dataset contains 40 observations, of which 36 are included for analyses.

Average shape:

Lmk.	Axis 1 (x)	Axis 2 (y)	Axis 3 (z)
1	-0.06959037	0.07276733	0.04770251
2	-0.07363689	0.07341305	0.03983199
3	-0.07739021	0.07288406	0.03215236
4	-0.08082341	0.07136578	0.02485556
5	-0.08406735	0.06911955	0.01799937
6	-0.08721029	0.06625097	0.01161224
7	-0.09016420	0.06264987	0.00556711
8	-0.09296882	0.05834113	-0.00012627
9	-0.09563159	0.05325610	-0.00544067
10	-0.09807677	0.04738685	-0.01032458
11	-0.10054514	0.04104863	-0.01449572
12	-0.10258539	0.03451236	-0.01800539

13	-0.10432416	0.02782566	-0.02095404
14	-0.10569279	0.02093653	-0.02323331
15	-0.10687238	0.01394580	-0.02510138
16	-0.10752126	0.00667819	-0.02622521
17	-0.10788104	-0.00077784	-0.02673636
18	-0.10773192	-0.00844096	-0.02661657
19	-0.10718039	-0.01626130	-0.02582367
20	-0.10597395	-0.02322971	-0.02460692
21	-0.10449427	-0.02985878	-0.02298373
22	-0.10264348	-0.03615544	-0.02093140
23	-0.10033685	-0.04205866	-0.01838047
24	-0.09770400	-0.04763374	-0.01546666
25	-0.09482861	-0.05302579	-0.01216364
26	-0.09130479	-0.05786308	-0.00831076
27	-0.08750842	-0.06240046	-0.00403088
28	-0.08325542	-0.06644252	0.00069755
29	-0.07894898	-0.06966523	0.00606051
30	-0.07416243	-0.07219229	0.01156077
31	-0.06891575	-0.07400899	0.01713690
32	-0.06317222	-0.07513730	0.02279141
33	-0.05695148	-0.07548987	0.02848965
34	-0.05027863	-0.07512428	0.03436191
35	-0.04308271	-0.07411764	0.04025771
36	-0.03538902	-0.07243331	0.04623489
37	-0.02761767	-0.07118594	0.05284088
38	0.07765655	0.00649227	0.06973225
39	0.08150112	0.00590602	0.06242325
40	0.08424449	0.00484467	0.05477432
41	0.08665331	0.00354419	0.04732491
42	0.08880238	0.00211324	0.04009510
43	0.09071924	0.00051459	0.03306093
44	0.09236222	-0.00130737	0.02613395
45	0.09374085	-0.00336807	0.01931236
46	0.09458246	-0.00566618	0.01260881
47	0.09505178	-0.00815992	0.00605301
48	0.09500000	-0.01134526	-0.00025603
49	0.09441515	-0.01467504	-0.00611102
50	0.09301756	-0.01819928	-0.01142885
51	0.09130838	-0.02180632	-0.01634221
52	0.08895781	-0.02553908	-0.02072675
53	0.08607881	-0.02949808	-0.02462222
54	0.08272151	-0.03360186	-0.02811985
55	0.07875178	-0.03785582	-0.03108365
56	0.07433767	-0.04231919	-0.03360409
57	0.06839299	-0.04793002	-0.03600146
58	0.06222795	-0.05325623	-0.03744745
59	0.05589705	-0.05829503	-0.03809172
60	0.04949867	-0.06318630	-0.03809946
61	0.04296321	-0.06786374	-0.03730559
62	0.03623241	-0.07228290	-0.03578875
63	0.02928494	-0.07635498	-0.03342301
64	0.02214060	-0.08011981	-0.03018830
65	0.01489000	-0.08344640	-0.02596570
66	0.00747393	-0.08681203	-0.01966791
67	0.00083188	-0.08884344	-0.01266071
68	-0.00503848	-0.08974192	-0.00506727
69	-0.01054994	-0.08975678	0.00294795
70	-0.01526391	-0.08839173	0.01163778
71	-0.01931882	-0.08586254	0.02096600
72	-0.02266279	-0.08198114	0.03092851

73	-0.02553625	-0.07725582	0.04177315
74	-0.02761767	-0.07118594	0.05284088
75	-0.03801415	0.08081397	0.03185969
76	-0.03556153	0.08214516	0.02628206
77	-0.03249916	0.08311788	0.02104149
78	-0.02905020	0.08388630	0.01608895
79	-0.02529247	0.08442715	0.01135614
80	-0.02115063	0.08471084	0.00691992
81	-0.01664998	0.08468925	0.00276913
82	-0.01185079	0.08451568	-0.00118458
83	-0.00667098	0.08411025	-0.00488675
84	-0.00125682	0.08356808	-0.00851586
85	0.00459706	0.08255853	-0.01194192
86	0.01021521	0.08130990	-0.01520556
87	0.01575936	0.07961408	-0.01799225
88	0.02110934	0.07760700	-0.02049128
89	0.02639676	0.07515389	-0.02245230
90	0.03150129	0.07234493	-0.02412972
91	0.03649886	0.06910488	-0.02540139
92	0.04136687	0.06552105	-0.02627998
93	0.04609975	0.06153943	-0.02678684
94	0.05125362	0.05688224	-0.02706968
95	0.05594611	0.05229313	-0.02697374
96	0.06011471	0.04779005	-0.02624936
97	0.06384675	0.04333784	-0.02495256
98	0.06703947	0.03900965	-0.02312384
99	0.06982767	0.03472745	-0.02081778
100	0.07213897	0.03058136	-0.01801764
101	0.07395410	0.02647479	-0.01456032
102	0.07528093	0.02251381	-0.01062802
103	0.07644904	0.01813378	-0.00525526
104	0.07672283	0.01415212	0.00041199
105	0.07615181	0.01050370	0.00637045
106	0.07474792	0.00723044	0.01262545
107	0.07252927	0.00427452	0.01909245
108	0.06977927	0.00121270	0.02576819
109	0.06551252	-0.00034648	0.03306292
110	0.06064576	-0.00192887	0.04058851
111	0.05522574	-0.00396599	0.04787246

Procrustes sums of squares: 0.3935329086972655
Tangent sums of squares: 0.38852487864708196

Data matrices in this dataset:
- canaux, raw data
- canaux, centroid size
- canaux, Procrustes coordinates

New Procrustes fit:
Dataset 'canaux'

111 landmarks in 3 dimensions.
The dataset contains 40 observations, of which 36 are included for analyses.

Average shape:

Lmk. Axis 1 (x) Axis 2 (y) Axis 3 (z)

1	-0.06959037	0.07276733	0.04770251
2	-0.07363689	0.07341305	0.03983199
3	-0.07739021	0.07288406	0.03215236
4	-0.08082341	0.07136578	0.02485556
5	-0.08406735	0.06911955	0.01799937
6	-0.08721029	0.06625097	0.01161224
7	-0.09016420	0.06264987	0.00556711
8	-0.09296882	0.05834113	-0.00012627
9	-0.09563159	0.05325610	-0.00544067
10	-0.09807677	0.04738685	-0.01032458
11	-0.10054514	0.04104863	-0.01449572
12	-0.10258539	0.03451236	-0.01800539
13	-0.10432416	0.02782566	-0.02095404
14	-0.10569279	0.02093653	-0.02323331
15	-0.10687238	0.01394580	-0.02510138
16	-0.10752126	0.00667819	-0.02622521
17	-0.10788104	-0.00077784	-0.02673636
18	-0.10773192	-0.00844096	-0.02661657
19	-0.10718039	-0.01626130	-0.02582367
20	-0.10597395	-0.02322971	-0.02460692
21	-0.10449427	-0.02985878	-0.02298373
22	-0.10264348	-0.03615544	-0.02093140
23	-0.10033685	-0.04205866	-0.01838047
24	-0.09770400	-0.04763374	-0.01546666
25	-0.09482861	-0.05302579	-0.01216364
26	-0.09130479	-0.05786308	-0.00831076
27	-0.08750842	-0.06240046	-0.00403088
28	-0.08325542	-0.06644252	0.00069755
29	-0.07894898	-0.06966523	0.00606051
30	-0.07416243	-0.07219229	0.01156077
31	-0.06891575	-0.07400899	0.01713690
32	-0.06317222	-0.07513730	0.02279141
33	-0.05695148	-0.07548987	0.02848965
34	-0.05027863	-0.07512428	0.03436191
35	-0.04308271	-0.07411764	0.04025771
36	-0.03538902	-0.07243331	0.04623489
37	-0.02761767	-0.07118594	0.05284088
38	0.07765655	0.00649227	0.06973225
39	0.08150112	0.00590602	0.06242325
40	0.08424449	0.00484467	0.05477432
41	0.08665331	0.00354419	0.04732491
42	0.08880238	0.00211324	0.04009510
43	0.09071924	0.00051459	0.03306093
44	0.09236222	-0.00130737	0.02613395
45	0.09374085	-0.00336807	0.01931236
46	0.09458246	-0.00566618	0.01260881
47	0.09505178	-0.00815992	0.00605301
48	0.09500000	-0.01134526	-0.00025603
49	0.09441515	-0.01467504	-0.00611102
50	0.09301756	-0.01819928	-0.01142885
51	0.09130838	-0.02180632	-0.01634221
52	0.08895781	-0.02553908	-0.02072675
53	0.08607881	-0.02949808	-0.02462222
54	0.08272151	-0.03360186	-0.02811985
55	0.07875178	-0.03785582	-0.03108365
56	0.07433767	-0.04231919	-0.03360409
57	0.06839299	-0.04793002	-0.03600146
58	0.06222795	-0.05325623	-0.03744745
59	0.05589705	-0.05829503	-0.03809172
60	0.04949867	-0.06318630	-0.03809946

61	0.04296321	-0.06786374	-0.03730559
62	0.03623241	-0.07228290	-0.03578875
63	0.02928494	-0.07635498	-0.03342301
64	0.02214060	-0.08011981	-0.03018830
65	0.01489000	-0.08344640	-0.02596570
66	0.00747393	-0.08681203	-0.01966791
67	0.00083188	-0.08884344	-0.01266071
68	-0.00503848	-0.08974192	-0.00506727
69	-0.01054994	-0.08975678	0.00294795
70	-0.01526391	-0.08839173	0.01163778
71	-0.01931882	-0.08586254	0.02096600
72	-0.02266279	-0.08198114	0.03092851
73	-0.02553625	-0.07725582	0.04177315
74	-0.02761767	-0.07118594	0.05284088
75	-0.03801415	0.08081397	0.03185969
76	-0.03556153	0.08214516	0.02628206
77	-0.03249916	0.08311788	0.02104149
78	-0.02905020	0.08388630	0.01608895
79	-0.02529247	0.08442715	0.01135614
80	-0.02115063	0.08471084	0.00691992
81	-0.01664998	0.08468925	0.00276913
82	-0.01185079	0.08451568	-0.00118458
83	-0.00667098	0.08411025	-0.00488675
84	-0.00125682	0.08356808	-0.00851586
85	0.00459706	0.08255853	-0.01194192
86	0.01021521	0.08130990	-0.01520556
87	0.01575936	0.07961408	-0.01799225
88	0.02110934	0.07760700	-0.02049128
89	0.02639676	0.07515389	-0.02245230
90	0.03150129	0.07234493	-0.02412972
91	0.03649886	0.06910488	-0.02540139
92	0.04136687	0.06552105	-0.02627998
93	0.04609975	0.06153943	-0.02678684
94	0.05125362	0.05688224	-0.02706968
95	0.05594611	0.05229313	-0.02697374
96	0.06011471	0.04779005	-0.02624936
97	0.06384675	0.04333784	-0.02495256
98	0.06703947	0.03900965	-0.02312384
99	0.06982767	0.03472745	-0.02081778
100	0.07213897	0.03058136	-0.01801764
101	0.07395410	0.02647479	-0.01456032
102	0.07528093	0.02251381	-0.01062802
103	0.07644904	0.01813378	-0.00525526
104	0.07672283	0.01415212	0.00041199
105	0.07615181	0.01050370	0.00637045
106	0.07474792	0.00723044	0.01262545
107	0.07252927	0.00427452	0.01909245
108	0.06977927	0.00121270	0.02576819
109	0.06551252	-0.00034648	0.03306292
110	0.06064576	-0.00192887	0.04058851
111	0.05522574	-0.00396599	0.04787246

Procrustes sums of squares: 0.3935329086972655
Tangent sums of squares: 0.38852487864708196

Data matrices in this dataset:
- canaux, raw data
- canaux, centroid size
- canaux, Procrustes coordinates

Principal Component Analysis: PCA: CovMatrix, canaux, Procrustes coordinates

	Eigenvalues	% Variance	Cumulative %
1.	0.00346150	31.183	31.183
2.	0.00176331	15.885	47.067
3.	0.00130645	11.769	58.836
4.	0.00073030	6.579	65.415
5.	0.00066725	6.011	71.426
6.	0.00051934	4.678	76.105
7.	0.00039661	3.573	79.677
8.	0.00032233	2.904	82.581
9.	0.00027707	2.496	85.077
10.	0.00024115	2.172	87.249
11.	0.00019359	1.744	88.993
12.	0.00017643	1.589	90.583
13.	0.00015672	1.412	91.995
14.	0.00014059	1.267	93.261
15.	0.00012089	1.089	94.350
16.	0.00008272	0.745	95.095
17.	0.00007574	0.682	95.778
18.	0.00006693	0.603	96.380
19.	0.00005833	0.525	96.906
20.	0.00005527	0.498	97.404
21.	0.00005288	0.476	97.880
22.	0.00003730	0.336	98.216
23.	0.00003358	0.302	98.519
24.	0.00003136	0.282	98.801
25.	0.00002464	0.222	99.023
26.	0.00001920	0.173	99.196
27.	0.00001657	0.149	99.345
28.	0.00001472	0.133	99.478
29.	0.00001286	0.116	99.594
30.	0.00001187	0.107	99.701
31.	0.00001087	0.098	99.799
32.	0.00000869	0.078	99.877
33.	0.00000637	0.057	99.934
34.	0.00000423	0.038	99.972
35.	0.00000307	0.028	100.000

Total variance: 0.01110071

Variance of the eigenvalues: 0.0000000559013
Eigenvalue variance scaled by total variance: 0.00045
Eigenvalue variance scaled by total variance and number of variables: 0.14834
Note: throughout all calculations of eigenvalue variances, the dimensionality used was 326.
Please double-check because this dimensionality may not be appropriate for all situations.
Warning: There appear to be fewer PCs than dimensions in shape space.
This can have the following reasons, among others:
-- the degrees of freedom are less than the shape dimensionality (sample size, etc.).
-- there is complete integration in the data.
Interpret the results cautiously and check for reasons why this is the case.

Principal Component Coefficients

```
        PC1         PC2         PC3         PC4         PC5         PC6
PC7          PC8         PC9         PC10        PC11        PC12
PC13         PC14        PC15        PC16        PC17        PC18
PC19         PC20        PC21        PC22        PC23        PC24
PC25         PC26        PC27        PC28        PC29        PC30
PC31         PC32        PC33        PC34        PC35
   x1  0.001795  -0.041739  -0.006518   0.009151  -0.021355   0.077451
     -0.121497  -0.037383  -0.018485  -0.033263  -0.119524   0.145766
     -0.093901   0.038078  -0.054167   0.115744  -0.010969  -0.030242
     -0.219760   0.035538   0.073118  -0.002766  -0.035422   0.034653
     -0.088817   0.000123   0.015499  -0.030410  -0.025515  -0.062629
     -0.032837   0.053970   0.064304  -0.109399  -0.081718
   y1  0.006943  -0.055698   0.090129  -0.056214   0.024333  -0.071111
0.016964    0.042601  -0.079956   0.037858  -0.046874  -0.014315  -
0.045533   -0.014457   0.013217  -0.058822   0.009579  -0.026022  -
0.019776    0.005578   0.116769   0.062872  -0.051039   0.140365  -
0.121349   -0.054439  -0.075806   0.103102  -0.056222  -0.049455
0.010796   -0.005254  -0.070789   0.045524   0.072973
   z1  0.016399   0.005356  -0.020247  -0.025229   0.061037  -0.013128
0.006145   -0.067387   0.117637  -0.073345   0.006221  -0.022921  -
0.150618   -0.009999   0.144614  -0.049642  -0.037278   0.029382  -
0.095354   -0.068894  -0.010694  -0.004445   0.096569   0.050863  -
0.120136   -0.157837  -0.072072   0.002344  -0.125147  -0.013727
0.027304    0.095786   0.048153  -0.023329  -0.069438
   x2 -0.010470  -0.042707  -0.013701   0.009428  -0.019147   0.087784
     -0.120024  -0.012776  -0.007752  -0.029230  -0.115771   0.139498
     -0.078395   0.027573  -0.070497   0.087192  -0.018286  -0.022491
     -0.164818   0.023152   0.079215   0.025964  -0.044683   0.030533
     -0.057293   0.011708   0.030861  -0.047122   0.004635  -0.036375
     -0.015807   0.049896   0.069121  -0.070267  -0.067059
   y2  0.005427  -0.055984   0.081389  -0.064882   0.047989  -0.065603
     -0.018631   0.032373  -0.067889   0.039512  -0.084094  -0.001967
     -0.053852  -0.001862   0.037488  -0.057682  -0.033257  -0.022166
     -0.040337  -0.005721   0.097903   0.028218  -0.029008   0.090442
     -0.103394  -0.034416  -0.065123   0.115071  -0.005876  -0.038687
     -0.070526   0.007025  -0.121742   0.118841   0.063550
   z2  0.017038   0.013233  -0.029651  -0.039541   0.059035  -0.014252
     -0.002413  -0.085816   0.118922  -0.070095  -0.016128  -0.013653
     -0.111105  -0.026895   0.127756  -0.042712  -0.066839   0.017155
     -0.065014  -0.059935  -0.019451   0.003012   0.048601   0.055591
     -0.098201  -0.098951  -0.048590  -0.028709  -0.096151   0.001228
0.002907    0.038514   0.044800  -0.040096  -0.021407
   x3 -0.022801  -0.042991  -0.021503   0.009656  -0.014382   0.094411
     -0.115566   0.008442   0.004629  -0.022355  -0.106383   0.131698
     -0.060746   0.018579  -0.083393   0.061087  -0.026781  -0.018051
     -0.112158   0.006693   0.078799   0.039254  -0.041397   0.024079
     -0.038000  -0.004645   0.014128  -0.043482   0.042986  -0.019873
     -0.011510   0.055743   0.063651  -0.022760  -0.001569
   y3  0.006679  -0.053166   0.076351  -0.067019   0.060163  -0.059598
     -0.040080   0.016642  -0.053309   0.035913  -0.111430   0.001068
     -0.059620   0.002675   0.041118  -0.056784  -0.055144  -0.010806
     -0.040707  -0.011928   0.090905   0.046342  -0.021451   0.038900
     -0.051516   0.006412  -0.025055   0.101765  -0.010963  -0.008117
     -0.110749  -0.029043  -0.087137   0.120725  -0.043375
   z3  0.018888   0.021692  -0.035907  -0.048252   0.051602  -0.013192
     -0.000661  -0.098835   0.114201  -0.059657  -0.029153  -0.008150
     -0.075877  -0.045638   0.109718  -0.045150  -0.083368   0.015141
```

-0.039675 -0.062420 -0.004920 0.033553 -0.006142 0.053862
-0.062580 -0.059044 -0.019033 -0.074338 -0.096391 0.030020
0.014431 0.007007 0.078929 -0.067230 0.007796
x4 -0.033948 -0.041190 -0.027643 0.008315 -0.012762 0.096550
-0.105697 0.022108 0.020838 -0.016560 -0.096368 0.118565
-0.048119 0.014264 -0.091554 0.035964 -0.034277 -0.016043
-0.051618 -0.005985 0.077587 0.033597 -0.024171 0.012163
-0.008576 -0.003772 0.018278 -0.028701 0.050490 -0.012337
-0.040435 0.064275 0.056867 0.001344 0.004384
y4 0.008006 -0.053276 0.065167 -0.069796 0.081432 -0.048455
-0.064085 0.011830 -0.054811 0.034786 -0.127940 0.018954
-0.050760 -0.007718 0.043500 -0.049993 -0.058098 0.006882
-0.065936 -0.011910 0.064398 0.071618 -0.036367 -0.004893
-0.013975 -0.006948 -0.028237 0.042965 0.032713 0.006168
-0.043615 -0.049746 -0.060099 0.112635 -0.085485
z4 0.021768 0.029282 -0.041079 -0.054400 0.042363 -0.009633
0.003647 -0.108741 0.108850 -0.049894 -0.038807 -0.002514 -
0.042271 -0.060893 0.093871 -0.043146 -0.095074 0.010469 -
0.021047 -0.064051 0.009665 0.054472 -0.050256 0.051745 -
0.048772 -0.017162 0.003579 -0.094819 -0.085893 0.051950
0.020859 -0.025245 0.080013 -0.059465 0.068201
x5 -0.042551 -0.040669 -0.033436 0.006001 -0.002642 0.096927
-0.099175 0.029810 0.033435 -0.022548 -0.088291 0.107488
-0.032751 0.006535 -0.104985 0.020561 -0.038324 0.004519
-0.020216 -0.021072 0.069475 0.023993 -0.006093 -0.001516
-0.001235 0.003515 0.006893 -0.028532 0.043548 -0.019627
-0.025466 0.077933 0.072745 0.047732 0.044461
y5 0.006868 -0.046955 0.054191 -0.070526 0.078601 -0.036653
-0.068111 0.006864 -0.052381 0.060467 -0.134110 0.024080
-0.043156 -0.007717 0.058872 -0.063267 -0.067319 -0.026766
-0.034654 -0.011296 0.061341 0.096166 -0.047737 -0.038415
0.030820 -0.025542 0.002357 0.044741 0.037837 0.025508 -
0.056276 -0.096658 -0.093987 0.063719 -0.061613
z5 0.025718 0.034661 -0.045412 -0.058141 0.032329 -0.007851
0.009648 -0.114732 0.102099 -0.043109 -0.042189 0.006461 -
0.014264 -0.070944 0.072379 -0.037420 -0.096477 0.002898
0.000035 -0.050240 0.014354 0.060663 -0.071331 0.046626 -
0.028717 0.020219 0.007196 -0.103246 -0.064841 0.056196
0.015094 -0.038854 0.079638 -0.061001 0.067688
x6 -0.052301 -0.037687 -0.037231 0.000646 0.001854 0.095685
-0.091353 0.040887 0.039090 -0.011097 -0.075196 0.096860
-0.022841 0.005514 -0.101110 -0.005839 -0.035717 -0.008838
0.032409 -0.015245 0.062175 0.023072 -0.005297 -0.017536
0.030817 0.009409 0.026621 -0.006616 0.074443 -0.006119 -
0.019607 0.059347 0.056423 0.035574 0.038844
y6 0.012596 -0.042860 0.038398 -0.068011 0.085655 -0.023977
-0.064473 -0.011274 -0.045351 0.051287 -0.142657 0.030091
-0.015068 -0.022681 0.050247 -0.051790 -0.063725 0.006799
-0.053636 -0.035129 0.042797 0.084172 -0.020218 -0.068462
0.020564 -0.027208 -0.029761 0.018435 -0.033782 0.005154 -
0.047743 -0.046355 -0.063703 0.068324 -0.023060
z6 0.030250 0.037819 -0.046687 -0.058595 0.018592 -0.005901
0.016539 -0.114500 0.092928 -0.036842 -0.036397 0.014910
0.006155 -0.075590 0.049507 -0.023955 -0.093048 -0.003416
0.017852 -0.034336 0.022704 0.064686 -0.088882 0.033125 -
0.014843 0.059465 0.015264 -0.099985 -0.039285 0.057438
0.029920 -0.057444 0.070641 -0.047617 0.058624
x7 -0.059434 -0.033766 -0.040881 -0.004675 0.008885 0.094319
-0.079345 0.045396 0.045260 -0.006865 -0.059216 0.083682
-0.005303 0.001297 -0.101023 -0.022240 -0.029671 -0.007403

0.065720 -0.016906 0.063025 0.004112 -0.003590 -0.024668
0.044282 0.004888 0.028344 0.010817 0.064322 -0.000726 -
0.020252 0.053383 0.023959 0.025448 0.022257
y7 0.016066 -0.038523 0.024255 -0.064874 0.083110 -0.014609
-0.061822 -0.022697 -0.046976 0.050506 -0.139816 0.037362
0.003354 -0.032219 0.045278 -0.050782 -0.046533 0.005771 -
0.039002 -0.039946 0.004324 0.088477 -0.003698 -0.102525
0.034349 -0.011957 -0.027634 -0.001584 -0.024220 0.006712 -
0.012978 -0.045750 -0.028053 0.048004 0.000372
z7 0.036086 0.040623 -0.048043 -0.056151 0.004699 -0.002138
0.029302 -0.112100 0.084893 -0.027959 -0.029642 0.022539
0.026163 -0.077692 0.031007 -0.012000 -0.082880 -0.007350
0.030648 -0.012814 0.032451 0.058653 -0.089976 0.013777 -
0.005180 0.097329 0.021712 -0.078617 -0.022044 0.054309
0.035128 -0.059419 0.046179 -0.028441 0.045320
x8 -0.064506 -0.028778 -0.043974 -0.010483 0.013815 0.089787
-0.061169 0.039974 0.052921 -0.004280 -0.044564 0.074847
0.003472 0.002861 -0.100373 -0.038866 -0.013479 -0.008252
0.109968 -0.027724 0.064932 -0.008096 0.014545 -0.028083
0.055947 0.000497 0.025003 0.031198 0.028304 -0.019844 -
0.019743 0.028514 0.020075 0.003477 0.054481
y8 0.019394 -0.033503 0.008710 -0.061404 0.080145 -0.002952
-0.058835 -0.025718 -0.053477 0.055042 -0.128322 0.036197
0.040059 -0.046366 0.040811 -0.049138 -0.027236 0.002761 -
0.043538 -0.026695 -0.035327 0.073973 -0.010798 -0.139931
0.035602 0.009757 -0.023687 -0.019971 -0.008278 0.025060
0.033655 -0.024033 -0.029061 0.014848 -0.038447
z8 0.044219 0.041928 -0.047442 -0.050543 -0.012787 0.002260
0.047736 -0.107844 0.076787 -0.021189 -0.016622 0.031516
0.037610 -0.074865 0.009264 0.003754 -0.061928 -0.013407
0.050845 0.005646 0.043151 0.043155 -0.068907 -0.003327
0.013340 0.128964 0.024101 -0.037995 -0.004558 0.038232
0.033887 -0.058467 0.032560 -0.003232 0.042870
x9 -0.069933 -0.022671 -0.047024 -0.015025 0.020023 0.083563
-0.044280 0.031721 0.058314 0.003770 -0.029074 0.061063
0.019351 0.003726 -0.101040 -0.052260 0.000183 -0.012714
0.136183 -0.031444 0.049944 -0.028193 0.025524 -0.038205
0.056899 -0.011299 0.030855 0.033710 -0.005097 -0.033537
0.001866 0.021315 -0.008081 -0.016749 0.074548
y9 0.026022 -0.028054 -0.007547 -0.060423 0.074434 0.008729
-0.043135 -0.032822 -0.062337 0.048591 -0.107743 0.037988
0.073187 -0.063022 0.034759 -0.047397 0.013532 -0.003063 -
0.018935 -0.017873 -0.061253 0.057937 0.000289 -0.165914
0.047991 0.032779 -0.023682 -0.017313 -0.009969 0.028180
0.072470 -0.017863 -0.022815 -0.033207 -0.048661
z9 0.051721 0.042721 -0.046725 -0.039981 -0.033966 0.007546
0.067380 -0.098561 0.066426 -0.007039 -0.000342 0.041479
0.050237 -0.068865 -0.014169 0.023652 -0.035027 -0.016270
0.055381 0.031746 0.039908 0.019437 -0.043789 -0.032802
0.009058 0.154127 0.023887 0.003069 0.023592 0.024395
0.047927 -0.032335 0.000805 0.041064 0.024694
x10 -0.074521 -0.016517 -0.047013 -0.021439 0.028121
0.076101 -0.031177 0.022674 0.061254 0.008568 -0.011922
0.046683 0.032320 0.005486 -0.091210 -0.065324 0.007756 -
0.023196 0.156884 -0.040651 0.042437 -0.041392 0.033444 -
0.040556 0.063955 -0.014328 0.009465 0.048231 -0.048810 -
0.032670 0.028634 -0.008659 -0.027188 -0.052200 0.075437
y10 0.034067 -0.020845 -0.027346 -0.057431 0.061600
0.020915 -0.016384 -0.040090 -0.073165 0.049451 -0.085567
0.041934 0.116175 -0.082772 0.017138 -0.044414 0.072510 -

0.003992	0.005646	0.001878	-0.103035	0.028557	0.015506	-
0.194636	0.040709	0.046657	-0.003345	-0.012033	-0.015343	
0.011960	0.116460	0.017889	-0.011556	-0.079747	-0.021138	
z10	0.059835	0.041199	-0.042756	-0.025784	-0.055524	
0.012885	0.087745	-0.086005	0.054522	0.002398	0.026745	
0.048263	0.054340	-0.058067	-0.034330	0.043818	-0.006828	-
0.021911	0.063758	0.055550	0.042961	-0.001645	-0.006492	-
0.059903	0.012112	0.184137	0.004660	0.056743	0.060286	
0.007414	0.068277	-0.020377	-0.032718	0.079399	-0.027916	
x11	-0.075053	-0.009957	-0.045587	-0.022510	0.030772	
0.064290	-0.015408	0.013797	0.061359	0.015790	-0.005316	
0.032435	0.020934	0.009727	-0.064236	-0.049188	-0.010935	-
0.028023	0.145769	-0.035340	0.042543	-0.012704	-0.001327	-
0.013748	0.050520	-0.028253	-0.007007	0.033061	-0.064838	-
0.020907	0.047281	-0.003053	-0.085485	-0.031214	0.073280	
y11	0.037045	-0.017648	-0.050649	-0.047135	0.056257	
0.021985	-0.005651	-0.057682	-0.072776	0.041335	-0.059832	
0.031658	0.130748	-0.067400	0.011680	-0.028635	0.077742	-
0.023985	0.025874	0.019281	-0.103951	-0.009957	0.022362	-
0.126653	0.014822	0.032552	-0.026149	0.011525	-0.034042	-
0.052853	0.063569	0.053672	0.019274	-0.083202	-0.035764	
z11	0.061187	0.038903	-0.037546	-0.014346	-0.063942	
0.016967	0.086511	-0.084057	0.053309	0.008738	0.029144	
0.047040	0.024863	-0.035046	-0.035091	0.040956	-0.023602	-
0.026960	0.033744	0.068751	0.070913	-0.009679	0.006801	-
0.054303	-0.001461	0.123091	0.001782	0.072290	0.063704	-
0.026530	0.027026	-0.005660	-0.042373	0.064557	-0.019205	
x12	-0.075181	-0.005875	-0.043378	-0.023674	0.030844	
0.053027	-0.007023	0.002147	0.065814	0.027074	-0.000811	
0.020074	0.019205	0.022669	-0.038007	-0.032428	-0.025368	-
0.039090	0.141276	-0.027226	0.014323	-0.013498	-0.019877	
0.009750	0.047909	-0.015017	-0.024317	0.028000	-0.091771	-
0.038748	0.030443	-0.055065	-0.099712	-0.017503	0.012878	
y12	0.038730	-0.013551	-0.070305	-0.035803	0.050610	
0.023199	0.009637	-0.068070	-0.072798	0.032342	-0.037183	
0.021924	0.134629	-0.060994	0.009768	-0.014387	0.074753	-
0.031751	0.033198	0.034390	-0.079384	-0.027457	0.016394	-
0.067284	-0.013135	0.001550	-0.042075	0.019862	-0.036580	-
0.080963	0.046464	0.117413	0.007845	-0.078257	0.004591	
z12	0.062093	0.034576	-0.030497	-0.005103	-0.072324	
0.021067	0.080661	-0.078257	0.051967	0.019374	0.034920	
0.041986	0.002248	-0.006254	-0.034529	0.038511	-0.026992	-
0.031526	0.016509	0.064956	0.077323	-0.019900	0.017999	-
0.050149	-0.003407	0.070722	0.003918	0.076697	0.052671	-
0.070371	-0.009216	-0.025123	-0.031079	0.064139	-0.047670	
x13	-0.073186	-0.000158	-0.037627	-0.026051	0.035986	
0.041022	0.004418	-0.002603	0.059352	0.031795	0.001809	
0.009596	0.010629	0.027068	-0.020372	-0.019718	-0.029377	-
0.051584	0.131149	-0.011236	0.009023	0.001383	-0.036933	
0.014056	0.045496	-0.004544	-0.045666	0.018022	-0.077988	-
0.022710	0.012357	-0.048019	-0.113668	-0.006895	-0.053584	
y13	0.038668	-0.011002	-0.088098	-0.025592	0.042805	
0.024412	0.021468	-0.076309	-0.066526	0.028282	-0.017875	
0.016075	0.138701	-0.049622	0.010222	0.000462	0.068340	-
0.035709	0.042033	0.040243	-0.068349	-0.055300	0.018892	-
0.014848	-0.033009	-0.025461	-0.048492	0.030864	-0.052804	-
0.115782	0.032221	0.125860	0.009560	-0.067227	0.033487	
z13	0.063630	0.031606	-0.021971	0.000379	-0.074196	
0.022366	0.075329	-0.068638	0.044805	0.025202	0.038345	
0.036604	-0.019526	0.015105	-0.038350	0.037757	-0.026871	-

0.038465	-0.001811	0.070760	0.082807	-0.020640	0.026929	-
0.055975	-0.004547	0.028889	-0.001332	0.072803	0.061518	-
0.077919	-0.054656	-0.006882	-0.025652	0.057283	-0.081058	
x14	-0.071043	0.004231	-0.030686	-0.022679	0.035258	
0.035408	0.012970	-0.001100	0.057591	0.052398	-0.001207	
0.002815	0.007734	0.028054	0.006220	-0.007604	-0.031110	-
0.052670	0.106054	-0.023030	0.009956	0.001368	-0.071186	
0.037564	0.057052	-0.009699	-0.044520	-0.000603	-0.096664	
0.010224	0.001606	-0.060904	-0.100771	0.006450	-0.041304	
y14	0.037902	-0.007231	-0.103043	-0.018826	0.036792	
0.022750	0.031998	-0.078755	-0.061002	0.019315	0.003266	
0.007829	0.137633	-0.038584	0.008939	0.012081	0.057414	-
0.037681	0.052673	0.057064	-0.058846	-0.066779	0.030272	
0.016337	-0.050954	-0.045265	-0.053391	0.022928	-0.047487	-
0.133922	0.020867	0.124266	0.002497	-0.048309	0.007673	
z14	0.063439	0.026726	-0.012332	0.008891	-0.075299	
0.025479	0.067843	-0.055829	0.040937	0.038586	0.035678	
0.034277	-0.030359	0.034925	-0.035977	0.036201	-0.022515	-
0.043633	-0.025403	0.051136	0.083663	-0.039687	0.023363	-
0.047123	0.005433	0.000729	0.004066	0.076782	0.044761	-
0.075179	-0.098596	-0.010448	-0.021924	0.049198	-0.061200	
x15	-0.068984	0.007973	-0.023079	-0.020856	0.039394	
0.027593	0.025504	-0.003053	0.051261	0.057451	-0.006500	-
0.006323	-0.005977	0.024967	0.022730	-0.008174	-0.022740	-
0.053579	0.087048	-0.015331	0.012538	0.026751	-0.073169	
0.039669	0.064196	-0.016926	-0.051386	-0.010378	-0.085138	
0.026709	0.012506	-0.047312	-0.102754	-0.028639	-0.042484	
y15	0.036632	-0.002792	-0.114808	-0.012383	0.028889	
0.020433	0.041729	-0.072127	-0.055617	0.019224	0.021331	
0.008691	0.135344	-0.026427	0.010870	0.026288	0.047881	-
0.039060	0.054295	0.055537	-0.040074	-0.094586	0.029600	
0.042311	-0.058410	-0.062997	-0.040882	0.009610	-0.051246	-
0.121658	-0.003970	0.105496	0.014782	0.004147	0.001516	
z15	0.062546	0.019852	-0.003290	0.012682	-0.069723	
0.028746	0.060321	-0.042687	0.036142	0.041329	0.031634	
0.022057	-0.043768	0.045755	-0.033093	0.021168	-0.018460	-
0.044005	-0.045837	0.042773	0.072646	-0.028798	0.045494	-
0.055189	0.017591	-0.033999	-0.001210	0.086164	0.040189	-
0.086218	-0.095290	0.008967	-0.047729	-0.009670	-0.047282	
x16	-0.064023	0.011495	-0.012929	-0.019297	0.038526	
0.029131	0.032543	-0.001872	0.041523	0.075545	-0.009653	-
0.008348	-0.015373	0.013911	0.050545	-0.010882	-0.015670	-
0.050522	0.073450	-0.015078	0.015947	0.018441	-0.097094	
0.050683	0.094110	-0.001200	-0.048425	-0.042145	-0.092276	
0.062447	0.038318	-0.031979	-0.074114	-0.010435	-0.055338	
y16	0.033785	0.001524	-0.123784	-0.006033	0.022102	
0.013592	0.050816	-0.056912	-0.047170	0.018674	0.033832	
0.007187	0.127639	-0.012110	0.013910	0.040296	0.040610	-
0.033657	0.040966	0.052266	-0.019883	-0.098005	0.036987	
0.047830	-0.060940	-0.091305	-0.021807	-0.013118	-0.038642	-
0.093593	-0.026293	0.060683	0.010715	0.023947	-0.013271	
z16	0.063289	0.012507	0.008033	0.014265	-0.063350	
0.037517	0.047891	-0.028908	0.026851	0.052510	0.033458	
0.013096	-0.049842	0.051404	-0.023571	0.007290	-0.012935	-
0.045683	-0.049460	0.027648	0.053604	-0.046802	0.043500	-
0.052409	0.047120	-0.038366	-0.000693	0.065860	0.015222	-
0.067291	-0.095142	0.016555	-0.062987	0.007353	-0.047936	
x17	-0.062222	0.016760	-0.000788	-0.014820	0.039001	
0.024114	0.044586	-0.000610	0.031941	0.085744	-0.015431	-
0.016395	-0.024381	0.007586	0.059667	-0.015269	0.002802	-

0.040613	0.049055	-0.011746	0.030677	0.033019	-0.110562	
0.044597	0.100176	-0.030367	-0.038835	-0.057958	-0.066071	
0.110408	0.049410	0.019726	-0.038920	-0.027945	-0.056691	
y17	0.031187	0.005445	-0.130037	-0.003152	0.013740	
0.009444	0.058690	-0.035479	-0.040848	0.022863	0.046251	
0.011682	0.114092	-0.002160	0.022957	0.048350	0.029575	-
0.031598	0.031156	0.040771	0.001520	-0.109112	0.043406	
0.048904	-0.050196	-0.107637	0.003501	-0.053979	-0.038379	-
0.041353	-0.025046	0.004079	0.016843	0.076333	-0.023666	
z17	0.058989	0.006373	0.020969	0.016607	-0.054696	
0.040471	0.035744	-0.015336	0.019757	0.058065	0.032443	-
0.001937	-0.051131	0.061302	-0.025292	-0.011328	0.002420	-
0.043137	-0.067280	0.005839	0.039009	-0.045465	0.040038	-
0.060051	0.048898	-0.078530	0.004171	0.067506	0.022687	-
0.028997	-0.086671	0.040173	-0.064224	-0.013096	-0.047655	
x18	-0.060010	0.016468	0.008850	-0.008384	0.035726	
0.023485	0.054500	-0.009820	0.025244	0.094199	-0.023570	-
0.015986	-0.025209	-0.000208	0.069982	-0.011205	0.001886	-
0.035270	0.048542	0.004516	0.018578	0.023828	-0.103913	
0.056131	0.081633	-0.015889	-0.067992	-0.047936	-0.052603	
0.091332	0.055622	0.020311	0.008873	-0.019601	-0.065015	
y18	0.027578	0.009744	-0.132971	-0.000973	0.005484	
0.002656	0.065581	-0.006133	-0.033360	0.028899	0.055402	
0.017363	0.097332	0.008739	0.031353	0.054862	0.018611	-
0.027938	0.014578	0.025220	0.027463	-0.114546	0.048942	
0.031433	-0.033259	-0.128926	0.035921	-0.102931	-0.024022	
0.036192	-0.027128	-0.068119	0.027803	0.134464	-0.041002	
z18	0.054775	-0.002965	0.032546	0.017893	-0.043072	
0.046839	0.024934	-0.004328	0.009826	0.064400	0.031183	-
0.014435	-0.045394	0.065593	-0.017739	-0.028666	0.013550	-
0.036295	-0.063080	-0.021544	0.009144	-0.043503	0.037229	-
0.065682	0.043750	-0.091840	-0.015698	0.066611	0.017018	-
0.014337	-0.057415	0.043690	-0.062675	-0.014929	-0.061106	
x19	-0.058465	0.016950	0.020313	0.001192	0.038330	
0.019355	0.056981	-0.013150	0.023960	0.097407	-0.035911	-
0.025973	-0.018532	0.007198	0.077511	0.003084	0.005644	-
0.014231	0.016182	0.023679	0.019704	0.038411	-0.092847	
0.054413	0.082805	-0.022732	-0.058421	-0.015931	-0.006530	
0.053434	-0.000703	0.015600	0.027580	-0.048775	-0.066230	
y19	0.023109	0.014121	-0.132830	0.000116	-0.002433	-
0.005178	0.072496	0.028387	-0.024527	0.036926	0.061710	
0.024909	0.077213	0.021470	0.042283	0.063124	0.004709	-
0.019974	-0.006871	0.005527	0.056721	-0.115471	0.056377	
0.003849	-0.010668	-0.147103	0.074949	-0.167180	0.003752	
0.126244	-0.026228	-0.166549	0.040847	0.202766	-0.069812	
z19	0.048804	-0.011759	0.045694	0.019290	-0.024331	
0.050099	0.008542	0.010206	0.004031	0.069374	0.023150	-
0.033979	-0.028186	0.076513	-0.012609	-0.037056	0.024452	-
0.023612	-0.076743	-0.053394	-0.021421	-0.025921	0.032429	-
0.077128	0.058569	-0.097975	-0.014349	0.067201	0.025564	
0.003213	-0.053223	0.051300	-0.088990	-0.055314	-0.066253	
x20	-0.058651	0.017255	0.029000	0.008106	0.038059	
0.020081	0.056748	-0.020077	0.030999	0.086064	-0.034836	-
0.027257	-0.013832	0.011343	0.087138	0.017809	0.008702	-
0.009588	0.010269	0.040801	-0.005317	0.022908	-0.048937	
0.053669	0.062722	-0.000590	-0.033859	0.003090	0.001146	
0.008734	-0.003836	0.003086	0.031920	-0.055561	-0.059765	
y20	0.023572	0.015526	-0.131357	0.000589	-0.003493	-
0.000465	0.073325	0.048214	-0.018548	0.048208	0.061924	
0.027881	0.059627	0.012379	0.029765	0.048239	0.001962	-

0.001324	-0.039458	0.008263	0.032868	-0.060980	0.053648	
0.027595	0.017018	-0.086723	0.056397	-0.094408	0.026643	
0.157471	-0.041189	-0.126293	0.019713	0.108203	-0.026442	
z20	0.043119	-0.023747	0.056015	0.017182	-0.010764	
0.058807	-0.011367	0.007386	-0.011103	0.069200	0.024277	-
0.047446	-0.013493	0.087912	-0.011358	-0.022813	0.015681	-
0.014983	-0.056422	-0.053385	-0.049815	-0.027084	0.011706	-
0.072005	0.052776	-0.072828	0.009560	0.042178	0.023333	
0.027925	-0.030745	0.015476	-0.069184	-0.054114	-0.031547	
x21	-0.057884	0.016327	0.038445	0.012612	0.040406	
0.018103	0.065823	-0.026728	0.027987	0.077155	-0.033911	-
0.030253	-0.012719	0.011824	0.084959	0.020951	0.016217	-
0.010561	0.004067	0.048725	-0.011395	0.014584	-0.006186	
0.044839	0.037047	-0.023290	-0.033837	0.024401	0.024120	-
0.035062	-0.012069	0.014586	0.068134	-0.040975	-0.028889	
y21	0.023084	0.015125	-0.128101	0.000845	-0.005693	
0.002638	0.075011	0.063462	-0.017152	0.057016	0.063426	
0.025190	0.043825	0.007632	0.018079	0.029781	-0.002331	
0.012502	-0.068715	0.010289	0.014227	-0.010295	0.044430	
0.046484	0.032512	-0.043967	0.025452	-0.028344	0.042122	
0.166536	-0.048170	-0.084004	0.015777	0.036152	0.008128	
z21	0.037487	-0.036258	0.066072	0.015060	-0.000221	
0.064429	-0.023727	0.003946	-0.031257	0.070630	0.025971	-
0.060798	0.000338	0.091847	-0.014832	-0.016484	0.011052	-
0.011324	-0.043165	-0.050748	-0.068615	-0.025189	-0.007552	-
0.066662	0.033231	-0.066757	0.008422	0.030658	0.028602	
0.038313	-0.006433	-0.000412	-0.030454	-0.055835	0.009826	
x22	-0.058404	0.017475	0.048697	0.019007	0.046779	
0.017522	0.070233	-0.034556	0.026128	0.065365	-0.032579	-
0.031773	-0.009307	0.009962	0.088630	0.021464	0.023171	-
0.004983	-0.002812	0.051547	-0.006975	0.018207	0.025147	
0.042994	0.024935	-0.031537	-0.013993	0.008763	0.054966	-
0.048923	-0.014148	0.023607	0.079363	-0.007603	0.021050	
y22	0.022035	0.015545	-0.123181	0.002808	-0.006084	
0.007720	0.073858	0.075253	-0.014789	0.062101	0.063645	
0.017255	0.027803	0.006668	0.011810	0.010849	-0.005612	
0.030357	-0.092245	0.013074	0.006925	0.035979	0.031968	
0.064348	0.050861	-0.014874	0.010979	0.001233	0.057690	
0.180601	-0.051436	-0.043273	0.005493	0.000198	0.044625	
z22	0.031368	-0.046250	0.075297	0.015800	0.009813	
0.071616	-0.036441	0.000570	-0.044942	0.068296	0.025094	-
0.073072	0.012003	0.088735	-0.014063	-0.008920	0.004848	-
0.003295	-0.024899	-0.046850	-0.082266	-0.021169	-0.019582	-
0.056051	0.024421	-0.056485	0.022469	-0.000245	0.030028	
0.064108	-0.001441	-0.009922	-0.000757	-0.019770	0.048561	
x23	-0.059677	0.017197	0.057973	0.024203	0.048148	
0.014457	0.071589	-0.044424	0.024028	0.056547	-0.028993	-
0.038621	0.003297	0.014167	0.104369	0.030072	0.024586	-
0.000851	-0.004111	0.057697	-0.013886	0.009384	0.050279	
0.035742	-0.011650	-0.027880	0.014809	0.004350	0.058811	-
0.086872	0.011362	-0.012879	0.086607	0.004974	0.069466	
y23	0.020692	0.015105	-0.117424	0.003955	-0.008800	
0.012333	0.069376	0.082493	-0.011574	0.061586	0.062791	
0.000726	0.014169	0.017130	0.013547	0.000911	-0.008657	
0.044392	-0.104226	0.023399	-0.004968	0.061636	0.021326	
0.073128	0.048046	0.014323	0.005967	0.027658	0.067396	
0.159244	-0.032620	-0.027675	0.008102	-0.037620	0.098524	
z23	0.025063	-0.056435	0.082479	0.017141	0.014663	
0.076276	-0.045163	-0.003566	-0.055809	0.066578	0.023732	-
0.083981	0.025317	0.083655	-0.001615	0.005694	-0.000866	

0.009435	-0.002160	-0.038577	-0.095083	-0.020611	-0.022308	-
0.048967	-0.000042	-0.043059	0.040875	-0.028664	0.023639	
0.057371	0.030654	-0.039056	0.025282	-0.017695	0.106429	
x24	-0.059734	0.016227	0.067282	0.027881	0.055092	
0.011597	0.072851	-0.051330	0.016914	0.045826	-0.032319	-
0.030753	0.009431	0.010498	0.102272	0.037232	0.018439	-
0.003586	-0.000007	0.057572	-0.009951	-0.000908	0.082429	
0.021937	-0.018887	-0.026269	0.042789	0.028719	0.068080	-
0.086012	0.004261	-0.034301	0.076174	0.018794	0.046665	
y24	0.018899	0.013854	-0.110191	0.006297	-0.009288	
0.017240	0.060634	0.087002	-0.009967	0.055601	0.058248	-
0.013446	0.000508	0.029543	0.007981	-0.007747	-0.019614	
0.051713	-0.112877	0.036331	-0.009703	0.071430	0.011316	
0.076326	0.054753	0.046391	0.004602	0.062581	0.082693	
0.139047	-0.023044	-0.021136	0.004173	-0.070032	0.096542	
z24	0.019187	-0.066863	0.090679	0.019665	0.020941	
0.080977	-0.054756	-0.004894	-0.067032	0.065144	0.016515	-
0.085673	0.038467	0.067022	-0.001949	0.017801	-0.013233	
0.012686	0.011931	-0.036094	-0.094856	-0.030679	-0.022496	-
0.042220	0.005975	-0.020623	0.052354	-0.030171	0.023843	
0.070127	0.030111	-0.040593	0.051014	-0.020598	0.099459	
x25	-0.058698	0.016498	0.077782	0.028947	0.060928	
0.008424	0.079340	-0.059955	0.007023	0.036468	-0.030790	-
0.021380	0.019924	0.006859	0.106082	0.024276	0.022797	-
0.005367	0.000767	0.034490	-0.006170	0.004611	0.099703	
0.006249	-0.035053	-0.026894	0.040923	0.060053	0.067500	-
0.087695	-0.003198	-0.031654	0.068901	0.038913	0.018053	
y25	0.018440	0.013130	-0.100541	0.005798	-0.010923	
0.024066	0.055132	0.087107	-0.011448	0.045558	0.058505	-
0.033320	-0.012187	0.049037	0.009994	-0.031652	-0.019364	
0.057878	-0.112282	0.035350	-0.015447	0.085873	-0.007834	
0.073145	0.052326	0.060015	-0.016359	0.095506	0.074270	
0.097853	-0.010599	0.002950	0.013375	-0.060511	0.073559	
z25	0.014652	-0.076939	0.097348	0.022248	0.024114	
0.084603	-0.056741	-0.006468	-0.076993	0.063070	0.011678	-
0.083352	0.051603	0.046086	0.001550	0.020418	-0.024147	
0.018449	0.031792	-0.037429	-0.097815	-0.021610	-0.014894	-
0.028492	0.002693	-0.006615	0.041128	-0.026008	0.005970	
0.076032	0.008787	-0.029937	0.069754	0.008936	0.081825	
x26	-0.060053	0.013423	0.084425	0.033414	0.062280	
0.002624	0.075348	-0.070558	0.004241	0.031163	-0.040392	-
0.007629	0.039987	0.000531	0.110976	0.034825	0.001636	-
0.005104	0.011774	0.020537	-0.022366	0.015137	0.124136	
0.013972	-0.049401	-0.015023	0.059648	0.057740	0.038612	-
0.074890	-0.051657	-0.049779	0.023089	0.084673	0.008579	
y26	0.016080	0.010104	-0.092864	0.009066	-0.015129	
0.029902	0.038315	0.079114	-0.005119	0.029345	0.049792	-
0.060081	-0.016809	0.074576	0.016967	-0.031127	-0.039862	
0.063053	-0.091567	0.052863	-0.037886	0.100513	-0.010609	
0.090333	0.039286	0.086806	0.003926	0.078980	0.040093	
0.048409	-0.036197	0.004985	-0.017851	-0.002552	0.052420	
z26	0.008812	-0.087600	0.100817	0.027719	0.022563	
0.086451	-0.059905	-0.008731	-0.084866	0.061372	0.006178	-
0.077902	0.062356	0.017299	0.012488	0.032774	-0.038148	
0.028404	0.054107	-0.031654	-0.098730	-0.004370	0.002148	-
0.008791	-0.004325	0.014862	0.037916	-0.039647	-0.027110	
0.074039	-0.015852	-0.017918	0.076283	0.042463	0.063995	
x27	-0.060890	0.008597	0.094339	0.033104	0.067287	-
0.005509	0.079866	-0.072335	-0.008257	0.029087	-0.042775	
0.012214	0.050711	-0.012092	0.110848	0.035040	-0.011749	

0.006466	0.018871	0.008981	-0.013045	0.031282	0.134028	-
0.006252	-0.074607	-0.029924	0.064892	0.074436	0.021340	-
0.035052	-0.085128	-0.057400	0.012262	0.098050	-0.018872	
y27	0.012096	0.006881	-0.079149	0.012836	-0.019161	
0.031656	0.031529	0.073929	-0.004377	0.012240	0.042190	-
0.084738	-0.031259	0.100541	0.017665	-0.041743	-0.051070	
0.077613	-0.088852	0.076359	-0.026467	0.096642	-0.034105	
0.079110	0.006525	0.092140	0.000931	0.077836	0.059971	-
0.011468	-0.006324	0.018650	0.015857	-0.036835	0.010123	
z27	0.002695	-0.097078	0.105567	0.036065	0.018367	
0.084789	-0.055527	-0.011478	-0.089258	0.059527	-0.004424	-
0.063710	0.071808	-0.020071	0.017700	0.040328	-0.048683	
0.043164	0.069235	-0.028224	-0.089217	0.003115	0.024052	
0.009067	-0.007887	0.034814	0.016930	-0.031370	-0.031623	
0.070316	-0.025248	-0.000318	0.091622	0.022963	0.042177	
x28	-0.061254	0.005503	0.103405	0.034490	0.074232	-
0.014652	0.084763	-0.070877	-0.028532	0.037855	-0.041618	
0.040326	0.066621	-0.037583	0.120692	0.027420	-0.017474	
0.013958	0.019971	-0.028649	0.008134	0.051778	0.127696	-
0.032771	-0.075147	-0.046642	0.045474	0.062303	-0.016906	
0.031833	-0.083160	-0.039468	-0.021916	0.103540	-0.016881	
y28	0.008383	0.005249	-0.065010	0.019423	-0.021546	
0.032137	0.021726	0.069251	-0.009017	0.002810	0.033587	-
0.105181	-0.037364	0.121156	0.030940	-0.048107	-0.049889	
0.083828	-0.089235	0.083702	-0.014422	0.077262	-0.067302	
0.053637	-0.010392	0.094913	-0.031990	0.042415	0.060102	-
0.075016	0.047339	0.046622	0.028626	-0.067755	0.019543	
z28	-0.002553	-0.106412	0.108782	0.044601	0.013527	
0.082987	-0.049155	-0.012369	-0.091053	0.057975	-0.014492	-
0.047280	0.081557	-0.065145	0.030183	0.047695	-0.059902	
0.050021	0.080895	-0.031756	-0.075838	0.014896	0.048089	
0.031989	-0.002239	0.054641	-0.009271	-0.033048	-0.050989	
0.073083	-0.047232	0.028357	0.091302	0.014109	0.009568	
x29	-0.053194	0.007535	0.096640	0.023958	0.076522	-
0.028718	0.087596	-0.040811	-0.036970	0.023863	-0.008381	
0.066814	0.077778	-0.041457	0.116508	0.004741	-0.014932	
0.007156	-0.032822	0.002541	0.008970	0.022046	0.092372	-
0.019882	-0.066618	-0.015526	0.045001	0.055050	0.012963	
0.034066	-0.029618	-0.022613	-0.057731	0.087415	0.015560	
y29	0.000894	-0.007265	-0.034435	0.040381	-0.033068	
0.028550	0.015879	0.032783	-0.014434	-0.006536	-0.000771	-
0.105333	-0.028149	0.105089	0.056539	-0.006023	-0.052628	
0.118895	-0.034665	0.054549	-0.014795	0.014480	-0.027534	
0.055405	-0.017592	0.094552	-0.004518	0.060275	-0.004466	-
0.067139	0.047956	0.021792	0.025840	0.024520	0.010036	
z29	-0.004399	-0.105467	0.091334	0.048043	0.013538	
0.067880	-0.011417	-0.001307	-0.087304	0.040063	0.001822	-
0.010935	0.082385	-0.094984	-0.004398	0.011141	-0.027751	
0.038595	0.046028	0.010269	-0.015971	-0.014707	0.008845	
0.075255	-0.001836	0.065729	-0.042036	-0.042652	-0.021018	
0.021097	-0.013881	0.009634	0.043402	-0.013285	-0.024595	
x30	-0.044323	0.010069	0.086514	0.014583	0.080430	-
0.047840	0.086617	-0.011619	-0.051732	0.008798	0.026736	
0.094707	0.079345	-0.041879	0.107604	-0.021133	-0.000024	
0.000230	-0.080724	0.031513	0.012612	0.014836	0.043017	-
0.007263	-0.049355	-0.009265	0.018772	0.025538	0.035665	
0.033242	0.016933	-0.003040	-0.041491	0.044739	0.011963	
y30	-0.005183	-0.018318	-0.010187	0.053344	-0.040586	
0.013205	0.006946	0.007287	-0.025992	-0.018310	-0.014720	-
0.087830	-0.024979	0.094188	0.067903	0.013871	-0.034041	

0.126389	0.005634	0.027438	-0.009647	-0.017353	-0.019823	
0.060915	-0.025929	0.085403	-0.014793	0.069170	-0.031219	-
0.064696	0.058334	-0.021861	0.084132	0.045319	-0.024304	
z30	-0.005580	-0.102003	0.073632	0.052298	0.010211	
0.050398	0.019760	0.010146	-0.077410	0.022532	0.014630	
0.020458	0.079083	-0.114248	-0.030525	-0.021342	-0.002350	
0.028595	0.020504	0.035654	0.032242	-0.026705	-0.025838	
0.110833	-0.001403	0.064577	-0.053131	-0.057480	0.006794	-
0.013148	0.012904	-0.006120	-0.015299	-0.036532	-0.016556	
x31	-0.034155	0.015126	0.073991	0.010168	0.084260	-
0.064243	0.085183	0.013520	-0.064008	-0.005468	0.051437	
0.116855	0.074505	-0.042130	0.088521	-0.035669	0.013971	-
0.002672	-0.118054	0.052832	0.017017	0.011638	0.003089	-
0.000704	-0.027613	-0.003498	0.009881	-0.017036	0.045983	
0.044693	0.046265	0.038977	-0.049311	-0.013077	0.016662	
y31	-0.008474	-0.026438	0.009873	0.069541	-0.046303	-
0.006671	0.006869	-0.009778	-0.029197	-0.030709	-0.023191	-
0.068131	-0.026819	0.071827	0.057788	0.021100	-0.006572	
0.120079	0.027206	0.019665	0.002072	-0.021811	-0.001113	
0.055310	-0.008498	0.068234	0.010148	0.030090	-0.063993	-
0.055020	0.031805	0.012014	0.075825	0.013620	-0.036486	
z31	-0.006922	-0.096991	0.055331	0.054386	0.005285	
0.031646	0.044476	0.018853	-0.065798	0.004034	0.024168	
0.045028	0.074637	-0.119194	-0.044966	-0.047553	0.017623	
0.018834	0.007199	0.051894	0.063111	-0.031996	-0.045957	
0.130691	0.001377	0.056067	-0.072633	-0.056551	0.015004	-
0.044972	0.030588	-0.016943	-0.043861	-0.044851	-0.012931	
x32	-0.023342	0.021573	0.058266	0.006404	0.086232	-
0.076094	0.077208	0.030313	-0.075168	-0.021472	0.066333	
0.133990	0.065264	-0.033457	0.065029	-0.035186	0.018394	-
0.002801	-0.138750	0.056257	0.011298	0.004440	-0.023644	
0.002023	-0.014178	0.002991	-0.004700	-0.042746	0.047458	
0.054747	0.077624	0.057142	-0.040793	-0.064991	0.003088	
y32	-0.007488	-0.033659	0.024753	0.081758	-0.049206	-
0.024631	0.006388	-0.024939	-0.033120	-0.049436	-0.032057	-
0.052353	-0.025650	0.051632	0.060379	0.035357	0.022061	
0.110554	0.035116	0.007093	0.008641	-0.026981	0.002728	
0.057516	0.017593	0.052150	-0.023486	-0.008704	-0.113609	-
0.049700	0.021829	0.052773	0.077317	-0.052193	-0.098313	
z32	-0.008243	-0.090102	0.036452	0.055826	-0.000814	
0.012100	0.062861	0.027726	-0.052280	-0.013726	0.029926	
0.063415	0.068400	-0.112747	-0.045194	-0.070262	0.034586	
0.011773	0.002416	0.065160	0.083138	-0.032111	-0.055704	
0.138321	0.005702	0.043129	-0.079574	-0.046725	0.010554	-
0.065343	0.026871	-0.016736	-0.063952	-0.037047	-0.008482	
x33	-0.011315	0.029926	0.039287	0.004092	0.087313	-
0.084931	0.065312	0.038451	-0.083548	-0.038412	0.069957	
0.144388	0.053176	-0.016668	0.035489	-0.025424	0.021264	-
0.006846	-0.143656	0.061904	-0.015456	-0.018899	-0.043362	
0.003456	-0.014785	0.028431	-0.023737	-0.035945	0.051769	
0.039582	0.072045	0.069346	-0.037910	-0.112682	0.026533	
y33	-0.005116	-0.034579	0.037310	0.086831	-0.055379	-
0.040763	0.001720	-0.039443	-0.044899	-0.066901	-0.036915	-
0.041718	-0.029190	0.041125	0.052167	0.042889	0.045966	
0.081203	0.044671	-0.005451	0.006748	-0.035363	0.028163	
0.044583	0.004143	0.084303	0.007380	-0.019364	-0.108642	-
0.060585	-0.015911	0.063673	0.078792	-0.105103	-0.038008	
z33	-0.010190	-0.081309	0.017208	0.055445	-0.007054	-
0.006920	0.073814	0.040089	-0.038159	-0.029050	0.037786	
0.077589	0.057847	-0.090069	-0.037488	-0.090377	0.041271	

0.014105	0.010148	0.059327	0.104777	-0.014570	-0.062638	
0.133017	0.015320	0.006733	-0.072703	-0.050315	-0.004835	-
0.053503	0.034004	-0.016487	-0.058536	-0.015955	-0.018174	
x34	0.001924	0.038940	0.015303	0.001023	0.089608	-
0.087180	0.050714	0.042072	-0.093462	-0.050815	0.072117	
0.142742	0.030443	0.001063	0.017559	-0.017942	0.017991	
0.003684	-0.127302	0.043618	-0.030383	-0.006959	-0.050872	-
0.004752	0.006453	0.010564	-0.034582	-0.054270	0.028945	
0.047631	0.077395	0.063129	-0.025847	-0.070047	-0.022620	
y34	-0.001136	-0.032325	0.040487	0.081424	-0.059392	-
0.049968	0.001516	-0.051050	-0.057880	-0.066910	-0.025497	-
0.039001	-0.040730	0.015037	0.054103	0.025364	0.072202	
0.037194	0.059856	-0.045423	0.036546	-0.001799	0.053977	
0.023200	0.013510	0.076904	0.036910	-0.078605	-0.078598	-
0.023091	0.020629	0.039573	0.090148	-0.057773	-0.055176	
z34	-0.013416	-0.071397	-0.002794	0.056708	-0.015972	-
0.031477	0.077627	0.050339	-0.014895	-0.047097	0.034114	
0.092324	0.053860	-0.047230	-0.036214	-0.091470	0.043069	
0.005263	0.019386	0.058400	0.104554	-0.025118	-0.059860	
0.116231	0.007581	-0.011050	-0.072310	-0.008152	-0.015153	-
0.055554	0.007672	-0.018336	-0.055274	-0.041764	-0.004464	
x35	0.015390	0.048772	-0.010550	0.001041	0.092792	-
0.089369	0.026025	0.040606	-0.097928	-0.062814	0.063700	
0.144912	0.014020	0.023507	-0.017897	0.007677	0.015698	
0.001679	-0.103459	0.016240	-0.067705	-0.019131	-0.064633	-
0.016192	0.022843	0.018379	-0.040997	-0.045993	0.006399	
0.033283	0.035099	0.050708	-0.006149	-0.034551	0.003912	
y35	0.003497	-0.033252	0.038249	0.072981	-0.053671	-
0.061746	-0.007860	-0.046951	-0.046088	-0.062314	-0.010150	-
0.008980	-0.029335	-0.002620	0.035024	0.013830	0.123516	-
0.011600	0.057172	-0.078879	0.051041	-0.005242	0.052586	-
0.009237	0.014753	0.082068	0.080762	-0.087836	-0.027923	
0.041906	0.037643	0.001155	0.081251	-0.004001	0.004615	
z35	-0.016212	-0.057032	-0.024451	0.055569	-0.026472	-
0.054458	0.076367	0.059719	0.002964	-0.064935	0.031532	
0.086840	0.039985	0.005619	-0.015494	-0.089363	0.035012	
0.001033	0.039900	0.040534	0.103006	0.011364	-0.043276	
0.079952	0.004343	-0.064105	-0.072508	0.015872	-0.050588	-
0.043552	-0.009396	-0.015650	-0.018108	-0.057468	-0.006351	
x36	0.030694	0.061010	-0.041392	0.002646	0.099027	-
0.087124	0.000263	0.030400	-0.102140	-0.077473	0.050795	
0.135343	-0.007245	0.054290	-0.066981	0.051523	0.011624	-
0.002034	-0.057668	-0.014392	-0.110009	-0.041156	-0.059076	-
0.033943	0.023004	0.032935	-0.050125	-0.033225	-0.004971	
0.015815	-0.007917	0.005870	0.034228	0.013451	0.007458	
y36	0.011725	-0.021423	0.020981	0.059680	-0.033569	-
0.072059	-0.012513	-0.041408	-0.028732	-0.076432	0.010668	-
0.014388	-0.019879	0.029201	-0.003416	0.029949	0.168029	-
0.101254	0.038666	-0.134480	0.079124	0.000389	0.040064	-
0.072291	-0.015110	0.070325	0.044018	-0.035904	0.000120	
0.026253	0.015726	-0.127603	0.068338	-0.025698	-0.033651	
z36	-0.020284	-0.044111	-0.041967	0.054053	-0.045617	-
0.079784	0.069320	0.069240	0.022347	-0.071520	0.026873	
0.086503	0.025335	0.054957	0.020030	-0.098855	0.026268	
0.005279	0.070716	0.019405	0.096497	0.074312	-0.009181	
0.038340	0.028304	-0.100514	-0.025850	0.015042	-0.088329	-
0.000379	-0.010222	0.032071	0.026563	-0.015177	-0.005136	
x37	0.044813	0.073821	-0.076381	0.010849	0.098817	-
0.076484	-0.027603	0.022601	-0.103006	-0.091037	0.031248	
0.117403	-0.027738	0.098252	-0.094981	0.099726	-0.023937	-

0.013914	-0.008040	-0.051227	-0.137063	-0.077731	-0.043947	-
0.051167	0.020173	0.048434	-0.088899	0.016290	-0.077626	
0.013874	-0.065519	-0.007751	0.062278	0.091925	-0.047841	
y37	0.017463	-0.011438	0.004341	0.061284	-0.030254	-
0.073076	-0.002763	-0.021844	-0.016931	-0.089617	0.044829	-
0.011637	-0.003454	0.070936	0.017415	0.013074	0.161556	-
0.202839	-0.001147	-0.161759	0.137598	-0.011165	0.037914	-
0.111777	-0.035213	0.117493	-0.037124	-0.018942	-0.008423	
0.046397	-0.002809	-0.113020	-0.024017	-0.068446	-0.067931	
z37	-0.020588	-0.025553	-0.061884	0.046975	-0.060389	-
0.113965	0.047738	0.074215	0.040399	-0.086462	0.016868	
0.088476	-0.002735	0.102577	0.052203	-0.103959	0.037401	
0.016779	0.106618	-0.011218	0.041195	0.132967	0.043939	-
0.021756	0.054343	-0.167513	0.058108	0.034737	-0.101378	
0.023144	-0.044308	0.057550	0.109872	0.031487	0.063930	
x38	-0.024633	0.018881	0.025393	-0.019368	0.058979	-
0.173549	0.022096	0.009381	0.134900	-0.011481	-0.046781	-
0.077364	0.057939	0.051899	-0.134140	-0.081629	0.120854	
0.128096	-0.030104	0.038262	-0.045550	-0.135548	-0.041912	
0.046805	0.038422	-0.000161	0.157068	0.049928	-0.168673	
0.048148	0.048688	-0.157414	0.012817	-0.027295	-0.060126	
y38	0.039063	0.074786	-0.040821	-0.067338	-0.039233	
0.044527	-0.043652	0.021184	-0.078501	-0.056856	-0.037695	
0.102809	0.031078	0.000353	0.025573	-0.055826	-0.103374	
0.097896	0.117212	-0.027598	0.078922	0.040165	-0.010480	
0.008852	-0.012416	-0.106269	0.251274	0.089350	0.073751	
0.018933	0.186236	0.011958	0.191467	-0.061590	-0.159223	
z38	-0.035564	-0.015035	-0.016483	-0.011641	0.008374	
0.033409	0.107854	-0.039444	-0.052544	-0.056521	0.014362	-
0.063007	-0.042116	-0.007015	-0.074257	-0.148782	-0.100029	-
0.282135	0.072482	-0.147206	-0.039724	0.105598	0.065641	
0.108123	-0.022316	-0.009314	-0.079242	-0.077309	0.311013	
0.047137	0.008320	0.067856	0.057245	0.000546	-0.000552	
x39	-0.021889	0.015798	0.018514	-0.021247	0.069758	-
0.163077	0.019771	0.013685	0.124036	-0.000980	-0.054098	-
0.077114	0.057308	0.044855	-0.122820	-0.084824	0.063515	
0.091583	-0.013535	-0.017501	-0.011135	-0.082566	0.008380	
0.082732	0.075430	-0.014757	0.095503	0.025254	-0.039532	
0.046266	-0.001891	-0.085141	-0.015043	0.025020	0.017985	
y39	0.048118	0.081134	-0.043606	-0.076098	-0.033687	
0.035176	-0.033898	0.019202	-0.079940	-0.051115	-0.041414	
0.098977	0.009359	0.007574	0.034761	-0.049252	-0.121260	
0.073367	0.119181	-0.010530	0.035485	-0.007238	0.005907	
0.017600	-0.009472	-0.059633	0.154648	0.054015	0.071873	-
0.007180	0.096548	-0.018856	0.120394	-0.083210	-0.083963	
z39	-0.025984	-0.027547	-0.011814	-0.023816	0.043456	
0.039592	0.129285	-0.049712	-0.066981	-0.069789	-0.009853	-
0.062191	-0.061938	0.017598	-0.100017	-0.107320	-0.075887	-
0.223478	0.047337	-0.042070	-0.048726	0.084632	0.071840	
0.089565	0.008214	-0.023879	-0.034904	-0.063711	0.198523	
0.037866	-0.015844	0.040581	0.047124	-0.013962	0.002971	
x40	-0.021468	0.018384	0.013792	-0.016105	0.059170	-
0.158511	0.014630	0.025305	0.115596	0.016610	-0.077078	-
0.062586	0.066105	0.040256	-0.131023	-0.058157	0.059221	
0.067389	-0.013932	-0.029046	-0.033002	-0.057635	-0.041723	
0.079610	0.055212	0.077016	0.076053	0.022275	-0.025172	
0.066714	-0.012548	0.014490	-0.026816	-0.039712	0.091004	
y40	0.054718	0.086311	-0.041263	-0.077128	-0.027989	
0.026410	-0.026184	0.018287	-0.075988	-0.046800	-0.047570	
0.089777	-0.014735	0.012505	0.042935	-0.050804	-0.113839	

0.064872	0.109386	-0.007165	0.009028	-0.038877	0.019902	
0.010828	0.002724	-0.022006	0.083983	0.032099	0.068775	-
0.017223	0.033314	-0.053953	0.057597	-0.086202	0.000396	
z40	-0.018636	-0.035545	-0.006494	-0.030647	0.066716	
0.040962	0.141539	-0.058061	-0.073188	-0.080361	-0.035417	-
0.052292	-0.073224	0.038204	-0.126438	-0.058636	-0.035896	-
0.161498	0.018155	0.053025	-0.068194	0.057681	0.027803	
0.051829	0.015876	-0.007852	0.012180	-0.026920	0.067456	
0.017831	-0.036421	0.006628	0.045281	-0.074048	0.036797	
x41	-0.019523	0.010933	0.004608	-0.002845	0.058972	-
0.146118	0.012009	0.012573	0.110919	0.031896	-0.091772	-
0.066642	0.069945	0.034766	-0.110648	-0.060703	0.051575	
0.059118	-0.033051	-0.010367	-0.005927	-0.052868	-0.014566	
0.060659	0.047221	0.065536	0.020877	0.048483	0.086891	-
0.024672	-0.021741	-0.016419	0.023626	0.057952	0.081201	
y41	0.060502	0.089320	-0.038218	-0.076957	-0.023425	
0.019918	-0.016611	0.016808	-0.069414	-0.042375	-0.055768	
0.079249	-0.033982	0.015433	0.056214	-0.051746	-0.102812	
0.058940	0.088940	0.002103	-0.013505	-0.060419	0.044350	
0.001753	0.016206	-0.000096	0.024821	0.025776	0.059514	-
0.029499	-0.016671	-0.088179	0.008106	-0.048096	0.037496	
z41	-0.012184	-0.044370	-0.002877	-0.035018	0.085818	
0.044560	0.148070	-0.070648	-0.074720	-0.085707	-0.051277	-
0.047450	-0.079739	0.053185	-0.136358	-0.026129	-0.009664	-
0.102866	-0.007143	0.122353	-0.063831	0.030619	0.017432	
0.011492	0.021944	-0.027511	0.031718	0.002610	-0.013161	-
0.022327	-0.046153	-0.051994	0.042973	-0.073959	0.001642	
x42	-0.017992	0.008784	-0.004277	0.002009	0.049950	-
0.131692	0.005886	0.013709	0.095804	0.038567	-0.104981	-
0.052299	0.074685	0.029313	-0.093441	-0.048123	0.044103	
0.046840	-0.015146	0.004463	-0.012129	-0.027357	-0.017840	
0.040778	0.027263	0.072593	0.019627	0.031800	0.068462	-
0.032945	-0.024217	0.006549	0.036512	0.094734	0.041650	
y42	0.065644	0.092197	-0.033628	-0.075248	-0.021383	
0.018793	-0.008042	0.018648	-0.063563	-0.037188	-0.062320	
0.071113	-0.046249	0.015459	0.063941	-0.053967	-0.088868	
0.046409	0.074785	0.005722	-0.032893	-0.081085	0.052354	
0.002708	0.027897	0.021748	-0.021320	0.017259	0.036077	-
0.036190	-0.038110	-0.096996	-0.034995	-0.034635	0.072057	
z42	-0.007404	-0.051004	0.001631	-0.039540	0.095292	
0.048598	0.148632	-0.078526	-0.073783	-0.087645	-0.060114	-
0.036285	-0.076983	0.061070	-0.138642	0.002152	0.009595	-
0.050661	-0.023255	0.157999	-0.059253	0.014886	0.002602	-
0.014133	0.026761	-0.025461	0.052342	0.014799	-0.089974	-
0.028616	-0.047551	-0.070459	0.033106	-0.071282	-0.005322	
x43	-0.015055	0.007073	-0.011417	0.005214	0.036632	-
0.117782	0.006663	0.011216	0.077104	0.050040	-0.108865	-
0.049545	0.082122	0.023870	-0.061935	-0.032603	0.045806	
0.037079	-0.022010	0.017083	0.000753	-0.015973	-0.012402	
0.018138	0.005027	0.050355	0.011192	-0.008303	0.081387	-
0.043025	-0.020370	0.002211	0.053998	0.075895	-0.024858	
y43	0.069978	0.092817	-0.027125	-0.072008	-0.020934	
0.021308	0.000669	0.021000	-0.055854	-0.031136	-0.068365	
0.058684	-0.049034	0.016802	0.072223	-0.053481	-0.073188	
0.034290	0.058111	0.001208	-0.045227	-0.089475	0.054692	
0.001250	0.041094	0.039315	-0.060826	0.008359	0.017609	-
0.039582	-0.058074	-0.092515	-0.069247	-0.026567	0.065044	
z43	-0.003906	-0.056497	0.007124	-0.042774	0.096172	
0.052551	0.145075	-0.084434	-0.068922	-0.084759	-0.061618	-
0.028511	-0.064227	0.063778	-0.129028	0.025451	0.022111	-

0.003721	-0.038479	0.160853	-0.042865	0.005336	-0.006238	-
0.034747	0.033792	-0.020853	0.059451	0.014917	-0.130239	-
0.025334	-0.047330	-0.076005	0.020740	-0.056349	-0.022077	
x44	-0.010770	0.005491	-0.017088	0.007684	0.028717	-
0.104862	0.004414	0.015601	0.057468	0.051177	-0.116089	-
0.039623	0.070175	0.017417	-0.032025	-0.011246	0.041244	
0.018619	-0.014565	0.021971	0.026682	0.012965	-0.011428	-
0.008404	-0.023357	0.031243	0.000864	-0.012355	0.087349	-
0.028611	-0.024691	0.049953	0.083900	0.004445	-0.016235	
y44	0.074504	0.092621	-0.019190	-0.067943	-0.020284	
0.027832	0.010423	0.025722	-0.047877	-0.026499	-0.073239	
0.046277	-0.052369	0.015937	0.075482	-0.054824	-0.053144	
0.015235	0.040113	-0.010064	-0.044460	-0.086770	0.044991	
0.000323	0.057044	0.050790	-0.087506	0.005455	-0.001892	-
0.038719	-0.067869	-0.060395	-0.074952	-0.034024	0.071139	
z44	-0.001273	-0.061089	0.013931	-0.044568	0.091984	
0.056298	0.136043	-0.087125	-0.058032	-0.081671	-0.058307	-
0.017637	-0.048801	0.060994	-0.115285	0.041456	0.026920	
0.039218	-0.044525	0.133705	-0.015550	0.005889	-0.015801	-
0.051978	0.037812	-0.004199	0.057750	0.026564	-0.134912	-
0.007891	-0.043738	-0.048176	0.016161	-0.028207	0.005990	
x45	-0.007661	0.002375	-0.023770	0.011611	0.019623	-
0.094676	0.004756	0.018071	0.041997	0.041891	-0.122958	-
0.029237	0.054645	0.013084	-0.006981	0.000295	0.034385	
0.013456	-0.002914	0.031869	0.053932	0.015255	0.026755	-
0.037826	-0.056150	0.012760	-0.005865	-0.010981	0.115184	-
0.014725	-0.010179	0.066047	0.074976	-0.034763	-0.068859	
y45	0.078278	0.090997	-0.010688	-0.061866	-0.022321	
0.037834	0.021258	0.030695	-0.035849	-0.024476	-0.076281	
0.031465	-0.052403	0.012938	0.073956	-0.060678	-0.030066	-
0.001547	0.019406	-0.027742	-0.033141	-0.084185	0.038595	
0.002698	0.082096	0.053471	-0.090273	0.002041	-0.020294	-
0.028910	-0.059227	-0.025421	-0.071118	-0.020597	0.057529	
z45	0.000116	-0.064423	0.020762	-0.043715	0.080908	
0.060383	0.122180	-0.090134	-0.040781	-0.075239	-0.049289	-
0.006226	-0.026043	0.055540	-0.094341	0.050278	0.022204	
0.076980	-0.043349	0.075194	0.019718	0.009626	-0.024018	-
0.063781	0.041796	0.015610	0.044267	0.030629	-0.115354	
0.019639	-0.035582	-0.004811	-0.009770	0.015639	0.019160	
x46	-0.004372	-0.000376	-0.025052	0.018296	0.013711	-
0.077252	0.005847	0.015469	0.033001	0.035787	-0.124866	-
0.031397	0.037971	0.023118	0.012674	0.011207	0.027455	-
0.001355	-0.001713	0.032132	0.075197	0.008763	0.055895	-
0.050878	-0.048206	-0.014476	-0.014636	-0.037940	0.118737	
0.015186	-0.019739	0.046471	0.049569	-0.065899	-0.068952	
y46	0.081339	0.088069	0.000559	-0.051768	-0.023878	
0.052039	0.032961	0.035120	-0.019259	-0.020143	-0.081088	
0.011467	-0.045438	0.012463	0.068787	-0.067096	-0.002105	-
0.020379	-0.003133	-0.053535	-0.020158	-0.065550	0.022919	
0.010940	0.118300	0.047739	-0.083759	-0.010501	-0.039040	-
0.009414	-0.046104	0.008529	-0.060095	-0.008360	0.046792	
z46	-0.000138	-0.065797	0.028837	-0.040716	0.062549	
0.067490	0.103660	-0.091586	-0.016082	-0.065216	-0.033489	
0.005886	0.007023	0.046855	-0.066527	0.052407	0.008833	
0.112186	-0.038508	-0.015657	0.060861	0.018211	-0.034971	-
0.066689	0.050401	0.040808	0.024422	0.023797	-0.071297	
0.054954	-0.028952	0.045268	-0.039025	0.076600	0.053319	
x47	-0.002284	-0.001612	-0.024736	0.028180	0.005867	-
0.056565	0.003595	0.009960	0.027113	0.034080	-0.123282	-
0.029817	0.036556	0.035156	0.033795	0.015845	0.006414	-

0.007884	-0.004914	0.018080	0.079458	-0.016716	0.067640	-
0.063784	-0.036631	-0.032645	-0.008715	-0.055055	0.103999	
0.029530	-0.023108	0.030388	0.035838	-0.099539	-0.042087	
y47	0.083465	0.084149	0.014004	-0.040465	-0.026345	
0.070824	0.044695	0.039982	-0.003569	-0.012962	-0.083533	-
0.009591	-0.028601	0.011396	0.061236	-0.074025	0.023460	-
0.034809	-0.028705	-0.081603	-0.004477	-0.036045	-0.007836	
0.022590	0.154127	0.047627	-0.065874	-0.017391	-0.059907	
0.004753	-0.015318	0.063174	-0.031399	0.011673	0.036328	
z47	-0.001934	-0.066443	0.037165	-0.035857	0.036518	
0.074380	0.077626	-0.090215	0.012910	-0.051429	-0.011001	
0.019345	0.048156	0.030754	-0.027770	0.048538	-0.009483	
0.140740	-0.030814	-0.137127	0.110523	0.032490	-0.041117	-
0.066426	0.056196	0.069669	-0.005053	0.007426	-0.006395	
0.103767	-0.017680	0.108496	-0.070283	0.154267	0.090719	
x48	0.002272	-0.003320	-0.020177	0.034321	-0.002100	-
0.030905	0.005298	0.010528	0.021567	0.015683	-0.127247	-
0.035968	0.030577	0.047838	0.042937	0.012340	-0.003693	-
0.020986	0.002105	0.000373	0.070379	-0.033450	0.088962	-
0.068443	-0.001563	-0.027651	-0.039899	-0.051610	0.065810	
0.055447	-0.016785	0.038499	0.023073	-0.099527	-0.012853	
y48	0.087040	0.073158	0.018189	-0.039896	-0.016458	
0.076364	0.038929	0.049252	0.007894	-0.008847	-0.078508	-
0.017204	-0.014142	0.007079	0.043504	-0.063854	0.049931	-
0.048875	-0.023582	-0.055367	-0.008030	-0.032588	-0.023839	
0.038677	0.099738	0.040317	-0.015273	-0.020078	-0.073208	
0.013453	-0.015035	0.100129	-0.025055	0.035745	0.031999	
z48	0.001017	-0.073913	0.029349	-0.052999	0.020027	
0.062742	0.060146	-0.067522	0.018897	-0.050919	-0.000349	
0.031474	0.028635	0.028596	-0.010607	0.042246	-0.003254	
0.133549	-0.027796	-0.108293	0.077004	-0.003305	-0.028125	-
0.046510	0.022486	0.056820	0.010307	-0.009500	0.021128	
0.093734	0.015889	0.059886	-0.054930	0.120431	-0.012197	
x49	0.006246	-0.008921	-0.016406	0.037230	-0.013481	-
0.007667	0.004464	0.014384	0.020903	0.006013	-0.110640	-
0.044432	0.031339	0.062273	0.051466	0.016439	-0.012562	-
0.029413	0.017495	0.009716	0.049990	-0.050031	0.077250	-
0.054170	-0.006648	-0.006355	-0.050554	-0.031074	0.025596	
0.053206	-0.004087	0.033076	0.005149	-0.070435	0.009689	
y49	0.089457	0.060766	0.022250	-0.039767	-0.007863	
0.080229	0.032861	0.058650	0.019792	-0.003884	-0.070556	-
0.020567	-0.000886	0.003730	0.026011	-0.052488	0.068798	-
0.059026	-0.018571	-0.032477	-0.012610	-0.025309	-0.041878	
0.058441	0.048564	0.038856	0.033716	-0.018527	-0.076563	
0.015574	-0.011864	0.128568	-0.011183	0.073918	0.002338	
z49	0.003458	-0.079358	0.021843	-0.066984	0.005453	
0.049892	0.043801	-0.048762	0.023970	-0.049963	0.005905	
0.040902	0.011218	0.023557	0.002798	0.035357	0.004478	
0.123150	-0.028068	-0.084369	0.049191	-0.037534	-0.014590	-
0.033464	-0.004751	0.043408	0.015719	-0.026210	0.046398	
0.085959	0.045227	0.016428	-0.038314	0.075926	-0.078089	
x50	0.011737	-0.014981	-0.014486	0.033739	-0.019030	
0.011673	0.001544	0.017657	0.023586	-0.011586	-0.108731	-
0.038995	0.027392	0.076341	0.046373	0.027774	-0.004141	-
0.046565	0.024920	0.026463	0.041348	-0.080846	0.067023	-
0.050497	-0.016212	0.018237	-0.067504	-0.041978	0.021688	
0.071636	-0.005103	0.080535	0.004055	-0.077202	0.018239	
y50	0.091873	0.046631	0.026913	-0.040050	0.000704	
0.081397	0.028931	0.068911	0.032607	-0.001949	-0.064948	-
0.022742	0.009991	-0.000627	0.013552	-0.043241	0.088230	-

0.065659	-0.011501	-0.013412	-0.011924	-0.016126	-0.053105	
0.071882	0.003240	0.033831	0.057130	-0.023187	-0.068927	
0.012092	-0.016888	0.163056	-0.007172	0.094406	-0.025876	
z50	0.004720	-0.082454	0.014140	-0.077985	-0.008418	
0.035588	0.028752	-0.034155	0.027426	-0.044100	0.017003	
0.045450	-0.001437	0.014702	0.014474	0.027664	0.004830	
0.115827	-0.030705	-0.066056	0.016172	-0.063812	-0.006695	-
0.019838	-0.021970	0.030024	0.025421	-0.026655	0.055028	
0.076913	0.068260	-0.029538	-0.025114	0.040775	-0.128159	
x51	0.017060	-0.027888	-0.015521	0.036729	-0.022538	
0.030333	-0.010217	0.019113	0.015950	-0.016315	-0.093077	-
0.042766	0.024223	0.081334	0.061195	0.022711	-0.010904	-
0.051093	0.003147	0.037356	0.031726	-0.124505	0.030777	-
0.054026	-0.010988	0.024028	-0.062141	-0.034360	0.015310	
0.086913	0.033487	0.007905	0.014434	-0.070224	-0.018178	
y51	0.093446	0.031662	0.030933	-0.038846	0.007371	
0.079953	0.027506	0.078875	0.039186	-0.000323	-0.057657	-
0.027831	0.017071	-0.005278	-0.002370	-0.033950	0.095434	-
0.065592	-0.003674	-0.006366	-0.017251	-0.007493	-0.066809	
0.076667	-0.037547	0.021742	0.090672	-0.022105	-0.066787	
0.016903	-0.005000	0.144275	0.003329	0.102971	-0.035142	
z51	0.005318	-0.082960	0.007564	-0.087282	-0.019270	
0.020305	0.018347	-0.023692	0.035620	-0.039686	0.026375	
0.048894	-0.011238	0.006351	0.018953	0.020146	0.008171	
0.105377	-0.026051	-0.053037	0.000028	-0.076771	0.009697	-
0.001904	-0.033405	0.013269	0.022663	-0.028960	0.069104	
0.056971	0.068603	-0.035871	-0.008881	0.023999	-0.128421	
x52	0.022278	-0.039315	-0.017397	0.037504	-0.029388	
0.046055	-0.010864	0.014935	0.023396	-0.018637	-0.071796	-
0.046647	0.029083	0.090365	0.058390	0.016840	-0.019440	-
0.043758	0.014329	0.040228	0.026296	-0.141081	0.016103	-
0.037940	-0.012823	-0.018219	-0.057739	-0.029809	0.018228	
0.078038	0.048642	-0.024944	0.027290	-0.042847	0.011640	
y52	0.093724	0.015472	0.036448	-0.041957	0.011778	
0.074217	0.023042	0.089511	0.042563	0.000395	-0.043577	-
0.029041	0.022485	-0.007461	-0.027552	-0.019319	0.095846	-
0.062218	0.007230	-0.010338	-0.009441	-0.000281	-0.074231	
0.068487	-0.084094	0.012246	0.123800	-0.021146	-0.038900	
0.028080	0.006119	0.098733	0.009250	0.089554	-0.039988	
z52	0.005273	-0.083049	0.001481	-0.092699	-0.026113	
0.004203	0.006932	-0.014228	0.039236	-0.035354	0.032086	
0.052262	-0.024143	-0.006775	0.031648	0.011238	0.014683	
0.088935	-0.040121	-0.038373	-0.016199	-0.088746	0.011885	
0.007824	-0.038681	0.020125	0.006439	-0.024580	0.071647	
0.036112	0.067566	-0.037486	-0.007500	0.000580	-0.127753	
x53	0.030265	-0.054365	-0.020650	0.036234	-0.030515	
0.060455	-0.018099	0.016198	0.029664	-0.032736	-0.056453	-
0.040258	0.022328	0.094719	0.069626	0.021721	-0.024535	-
0.048114	0.010457	0.052306	0.015318	-0.157807	-0.011735	-
0.020184	-0.019839	0.000977	-0.025537	-0.015286	0.045525	
0.096996	0.059250	-0.032553	-0.028099	-0.040504	-0.024433	
y53	0.094408	-0.002293	0.040327	-0.042449	0.016388	
0.065425	0.026229	0.099249	0.047329	-0.001797	-0.033575	-
0.032509	0.027485	-0.015547	-0.035912	-0.013671	0.096508	-
0.056970	0.011371	-0.004344	-0.011564	0.007430	-0.085280	
0.065931	-0.105785	0.003514	0.115316	-0.015152	-0.028268	
0.006704	0.006855	0.088024	0.006751	0.076277	-0.041267	
z53	0.002447	-0.080224	-0.003505	-0.095244	-0.035420	-
0.014795	-0.001500	-0.011369	0.045217	-0.022379	0.041438	
0.050131	-0.026964	-0.018085	0.026522	0.001281	0.016016	

0.077810	-0.043882	-0.033619	-0.027395	-0.093066	0.016888	
0.009316	-0.044781	0.001803	-0.019769	-0.013642	0.060883	-
0.001308	0.064664	-0.043933	0.035171	-0.010272	-0.061640	
x54	0.033804	-0.070384	-0.023129	0.035376	-0.037931	
0.065259	-0.025930	0.016149	0.040604	-0.032704	-0.029900	-
0.037279	0.029108	0.096805	0.071244	0.011744	-0.035320	-
0.040300	0.002630	0.060288	0.009739	-0.159530	-0.053789	-
0.017438	-0.024185	-0.013681	-0.033978	-0.015529	0.023083	
0.083827	0.074065	-0.031970	-0.031265	-0.019109	0.007662	
y54	0.095031	-0.021625	0.043626	-0.044244	0.018756	
0.054589	0.027415	0.107738	0.050971	-0.005322	-0.021295	-
0.033152	0.029841	-0.020192	-0.051890	-0.001065	0.090495	-
0.047074	0.020440	-0.007893	-0.011091	0.016970	-0.089829	
0.055229	-0.132340	-0.010162	0.116872	-0.007873	0.003561	-
0.010530	0.004317	0.034039	-0.007514	0.038514	-0.043998	
z54	0.001933	-0.076657	-0.009621	-0.094750	-0.038091	-
0.028557	-0.005978	-0.010266	0.048735	-0.015991	0.046167	
0.048304	-0.034056	-0.031395	0.027979	-0.002969	0.022218	
0.062634	-0.048204	-0.029782	-0.035271	-0.094345	0.018590	
0.021755	-0.044313	-0.002839	-0.027624	0.013155	0.072857	-
0.026450	0.049887	-0.039541	0.044405	-0.021347	-0.018692	
x55	0.039514	-0.089821	-0.027508	0.034567	-0.042329	
0.068563	-0.027447	0.013442	0.052532	-0.035824	-0.000253	-
0.031158	0.026080	0.095022	0.071263	0.013499	-0.039791	-
0.037219	0.005380	0.072931	-0.003962	-0.159997	-0.093200	-
0.005429	-0.054569	-0.029049	-0.020422	0.001186	0.041799	
0.049103	0.064162	-0.038007	-0.042956	-0.004119	0.018550	
y55	0.094779	-0.042395	0.047320	-0.046803	0.020546	
0.040042	0.030383	0.117662	0.053633	-0.011223	-0.009776	-
0.034146	0.031573	-0.027048	-0.064895	0.008558	0.074876	-
0.034183	0.025963	-0.017998	-0.011337	0.025575	-0.093658	
0.039754	-0.144119	-0.021200	0.102659	-0.002596	0.037028	-
0.030647	0.001743	-0.024818	-0.022863	-0.007934	-0.043936	
z55	-0.000620	-0.070829	-0.015107	-0.092261	-0.039680	-
0.044769	-0.014533	-0.011925	0.055356	-0.005359	0.049004	
0.044074	-0.033447	-0.042682	0.024297	-0.015622	0.021303	
0.050675	-0.061110	-0.028026	-0.034856	-0.092274	0.015424	
0.027187	-0.026979	-0.001581	-0.055419	0.042740	0.058301	-
0.050704	0.043431	-0.027819	0.044981	-0.028659	0.069641	
x56	0.046433	-0.111617	-0.032763	0.031666	-0.045399	
0.066397	-0.033927	0.009847	0.070389	-0.043128	0.026545	-
0.019749	0.028008	0.097577	0.059451	0.007363	-0.049030	-
0.027410	0.008422	0.086970	-0.019028	-0.151331	-0.128362	
0.001397	-0.071652	-0.028497	-0.027921	0.057893	0.057672	-
0.007341	0.044897	-0.047430	-0.100164	-0.008378	0.072819	
y56	0.094391	-0.063757	0.050486	-0.048841	0.020407	
0.024121	0.036339	0.127144	0.052503	-0.014302	0.006400	-
0.037536	0.030204	-0.034645	-0.071092	0.015107	0.060107	-
0.012803	0.028586	-0.030795	-0.001792	0.040995	-0.105965	
0.020627	-0.142568	-0.049305	0.087939	-0.013499	0.070177	-
0.044890	0.016034	-0.083880	-0.027020	-0.054816	-0.049268	
z56	-0.004669	-0.063312	-0.020954	-0.085813	-0.040458	-
0.059782	-0.021706	-0.017678	0.059197	0.008658	0.055948	
0.034285	-0.031062	-0.061193	0.025621	-0.017451	0.021299	
0.030938	-0.071613	-0.028013	-0.034303	-0.093268	0.015692	
0.034731	-0.022226	-0.012672	-0.058440	0.047067	0.053978	-
0.065865	0.031248	-0.008419	0.086559	-0.005966	0.141384	
x57	0.051723	-0.125746	-0.042698	0.028485	-0.055178	
0.066339	-0.041193	0.012760	0.067075	-0.039284	0.031146	
0.005166	0.039225	0.075032	0.073997	0.027431	-0.042335	-

0.046224	0.008830	0.064528	-0.009281	-0.077640	-0.107619	
0.001632	-0.054170	-0.024907	-0.005919	0.057035	0.018462	-
0.014862	0.016405	-0.039734	-0.036761	0.037196	0.038295	
y57	0.090439	-0.073588	0.052910	-0.050040	0.013200	
0.000016	0.045621	0.132181	0.047104	-0.003200	-0.004965	-
0.036908	0.005536	-0.056134	-0.066083	0.051046	0.040329	-
0.015639	0.010802	-0.026688	0.004314	0.053387	-0.023690	-
0.004749	-0.091704	-0.025760	0.017627	-0.015050	0.030400	-
0.050165	0.022602	-0.086565	-0.023111	-0.077109	0.013242	
z57	-0.008060	-0.046567	-0.028497	-0.078291	-0.037390	-
0.067274	-0.036014	-0.021716	0.053111	0.029234	0.038971	
0.037162	-0.033287	-0.071720	0.032754	-0.029223	0.035525	
0.010725	-0.071587	-0.023146	-0.036586	-0.076100	0.020898	
0.007968	-0.012923	0.005490	-0.053000	0.065189	0.009836	-
0.046769	-0.005831	0.026581	0.074264	-0.011817	0.150304	
x58	0.058913	-0.136740	-0.048989	0.020062	-0.059641	
0.056824	-0.044758	0.015237	0.061607	-0.026816	0.031904	
0.023678	0.035518	0.052399	0.078856	0.038239	-0.037415	-
0.060249	-0.001013	0.041267	-0.013030	-0.012504	-0.046533	
0.009687	-0.041048	-0.004863	0.004571	0.056189	-0.025007	-
0.004726	-0.021267	-0.021144	-0.015204	0.021372	0.083262	
y58	0.083938	-0.080303	0.052341	-0.043616	0.005030	-
0.015328	0.051458	0.133725	0.045015	-0.001353	-0.012049	-
0.035380	-0.005158	-0.070464	-0.055832	0.075302	0.020587	-
0.015685	0.003218	-0.013620	0.017574	0.065056	0.009526	-
0.034328	-0.053560	-0.025487	-0.031728	-0.020097	0.000320	-
0.071740	0.026377	-0.084464	0.024536	-0.045693	0.011588	
z58	-0.014221	-0.028001	-0.037182	-0.070954	-0.030785	-
0.068471	-0.049559	-0.024062	0.051918	0.042438	0.028876	
0.033584	-0.030596	-0.081741	0.039277	-0.029340	0.050838	-
0.003687	-0.064201	-0.015442	-0.033330	-0.068254	-0.009379	-
0.016099	-0.009063	0.015549	-0.038732	0.072213	-0.002309	-
0.042664	-0.031734	0.029122	0.079903	0.026917	0.060249	
x59	0.064434	-0.144392	-0.055654	0.015958	-0.063190	
0.047491	-0.048731	0.015453	0.054376	-0.017211	0.032269	
0.037232	0.038735	0.035047	0.081796	0.045533	-0.029297	-
0.070808	-0.006604	0.030581	-0.018376	0.046343	-0.005815	
0.013275	-0.035522	0.013725	0.010037	0.048686	-0.059329	-
0.013050	-0.054280	-0.007694	0.014087	0.023815	0.081404	
y59	0.077257	-0.084667	0.051613	-0.038688	0.001723	-
0.030643	0.054192	0.131694	0.046183	0.001207	-0.013509	-
0.036679	-0.017175	-0.083416	-0.049314	0.088521	-0.002305	-
0.009808	-0.004535	-0.008859	0.026706	0.067206	0.038750	-
0.056104	-0.013732	-0.024814	-0.063484	-0.023085	-0.017351	-
0.077401	0.027788	-0.078267	0.049732	-0.030000	0.022844	
z59	-0.019386	-0.009826	-0.045364	-0.067406	-0.021898	-
0.066076	-0.061660	-0.024003	0.053339	0.053880	0.024840	
0.025734	-0.031416	-0.090630	0.036566	-0.030656	0.052747	-
0.018309	-0.062348	-0.013820	-0.024253	-0.069128	-0.034169	-
0.032753	0.002101	0.017114	-0.003258	0.082102	-0.001830	-
0.034579	-0.043049	0.025208	0.070083	0.036856	0.003444	
x60	0.069401	-0.148823	-0.062349	0.012199	-0.064314	
0.035532	-0.055196	0.013811	0.049684	-0.007063	0.035652	
0.046409	0.043804	0.019878	0.080580	0.048111	-0.028467	-
0.072735	-0.011900	0.020179	-0.027873	0.089986	0.025678	
0.014227	-0.027276	0.024102	0.014260	0.047070	-0.079978	-
0.027073	-0.085604	-0.010106	0.031007	0.045975	0.090420	
y60	0.070365	-0.086867	0.052000	-0.031228	0.000668	-
0.044062	0.053376	0.122600	0.046526	-0.002098	-0.011226	-
0.041208	-0.025700	-0.093843	-0.045237	0.089049	-0.016565	-

0.004361	-0.009820	-0.003173	0.031573	0.056655	0.056295	-
0.079847	0.033805	-0.026473	-0.080947	-0.030226	-0.024487	-
0.053337	0.038589	-0.054756	0.062879	-0.040262	0.017596	
z60	-0.024593	0.008564	-0.048714	-0.060803	-0.015390	-
0.062018	-0.073940	-0.026423	0.048053	0.055803	0.018826	
0.008781	-0.029749	-0.103104	0.036149	-0.032547	0.062048	-
0.032648	-0.059795	-0.006106	-0.025045	-0.085241	-0.059973	-
0.050776	0.046605	0.010484	0.013367	0.066477	-0.003069	
0.011666	-0.043182	0.045638	0.047852	-0.002908	-0.039282	
x61	0.073156	-0.148435	-0.067227	0.009025	-0.061914	
0.017575	-0.062045	0.005661	0.043968	0.000829	0.042697	
0.047648	0.047362	0.004773	0.074169	0.038904	-0.026225	-
0.063551	-0.021017	0.005847	-0.047920	0.123261	0.048953	
0.022776	0.000819	0.053331	0.018656	0.023632	-0.096105	-
0.037004	-0.098909	-0.006573	0.022689	0.053055	0.042727	
y61	0.064762	-0.089062	0.048710	-0.021519	0.001592	-
0.051381	0.048361	0.112925	0.049006	-0.005673	-0.008820	-
0.042574	-0.032442	-0.096799	-0.046883	0.087713	-0.026029	
0.000285	-0.004317	0.012403	0.043881	0.040809	0.061024	-
0.112638	0.057728	-0.061618	-0.088027	-0.022432	-0.006149	-
0.032401	0.039625	-0.039871	0.080356	-0.054611	0.054639	
z61	-0.028543	0.026451	-0.054981	-0.059245	-0.006053	-
0.055204	-0.083167	-0.018416	0.045170	0.062231	0.017275	-
0.011082	-0.043136	-0.111566	0.037427	-0.028113	0.067653	-
0.044344	-0.054529	0.004750	-0.013477	-0.085383	-0.057487	-
0.050930	0.055150	-0.018212	0.026384	0.058181	-0.016921	
0.040815	-0.042294	0.035010	0.020357	-0.005356	-0.018049	
x62	0.078972	-0.145549	-0.072745	0.006498	-0.055779	-
0.000445	-0.069505	-0.001537	0.035666	0.010112	0.050764	
0.043736	0.046827	-0.009614	0.058065	0.023003	-0.018264	-
0.046848	-0.029273	0.000682	-0.060592	0.138935	0.062505	
0.019596	0.034377	0.051098	0.024354	0.000257	-0.087228	-
0.037918	-0.110853	0.011228	0.022365	0.037920	0.009612	
y62	0.055208	-0.087923	0.046708	-0.008715	0.002633	-
0.059800	0.037660	0.097151	0.055753	-0.014465	-0.001104	-
0.044718	-0.025660	-0.096292	-0.050685	0.073208	-0.046847	
0.007372	0.000845	0.027488	0.041408	0.026600	0.049614	-
0.119265	0.078456	-0.059591	-0.080017	-0.025420	0.005476	-
0.011387	0.039912	-0.045442	0.039206	-0.044038	0.060333	
z62	-0.034859	0.047148	-0.058075	-0.055891	0.002239	-
0.043024	-0.088024	-0.013744	0.041724	0.061345	0.009969	-
0.035498	-0.036335	-0.116342	0.035253	-0.021759	0.059392	-
0.061643	-0.050999	0.017618	-0.005746	-0.079597	-0.065488	-
0.034470	0.049862	-0.006028	0.048354	0.031308	-0.010165	
0.050858	-0.059378	0.009216	-0.024300	-0.000140	0.006842	
x63	0.084003	-0.139344	-0.079377	0.005941	-0.047669	-
0.020104	-0.079191	-0.012959	0.026369	0.019104	0.059467	
0.037088	0.049871	-0.018573	0.035897	0.001124	-0.011620	-
0.026573	-0.034117	0.000805	-0.077484	0.141931	0.060609	
0.013109	0.075419	0.049188	0.040150	-0.035362	-0.057922	-
0.039231	-0.109343	0.015402	-0.002229	0.020045	-0.035031	
y63	0.045699	-0.083011	0.046802	0.005352	0.007820	-
0.069493	0.026897	0.076004	0.067410	-0.025289	0.012343	-
0.058622	-0.024805	-0.094458	-0.049487	0.043082	-0.061469	
0.024692	0.011211	0.046508	0.034629	-0.002024	0.040047	-
0.120914	0.102618	-0.072730	-0.068072	-0.025441	0.020705	
0.012300	0.034353	-0.014987	0.031506	-0.013782	0.068238	
z63	-0.040354	0.067919	-0.059883	-0.050609	0.009396	-
0.026227	-0.084709	-0.009585	0.036414	0.051658	0.004126	-
0.072490	-0.028120	-0.119589	0.040682	-0.014902	0.048499	-

0.075836	-0.042292	0.038192	0.016508	-0.072176	-0.067819	-
0.022699	0.049097	-0.026192	0.075588	-0.005644	-0.000690	
0.038227	-0.064831	0.013638	-0.005746	0.004547	0.026369	
x64	0.089281	-0.128875	-0.085165	0.005655	-0.036255	-
0.043207	-0.087227	-0.026709	0.016733	0.028559	0.071590	
0.020999	0.048279	-0.025878	0.010020	-0.030322	-0.000268	-
0.000618	-0.038596	0.004781	-0.097493	0.132843	0.053358	
0.012547	0.119562	0.047624	0.054874	-0.088474	-0.013048	-
0.045590	-0.097380	0.009637	-0.040361	-0.000526	-0.093541	
y64	0.035599	-0.076787	0.045213	0.019964	0.016057	-
0.073022	0.016151	0.052081	0.078800	-0.043644	0.023842	-
0.064279	-0.021760	-0.087460	-0.050843	0.010610	-0.074734	
0.046830	0.024013	0.064486	0.009354	-0.026850	0.014768	-
0.125639	0.118532	-0.099686	-0.056461	0.003892	0.035677	
0.045231	-0.000068	0.056532	0.028365	0.014211	0.031863	
z64	-0.045915	0.087783	-0.061143	-0.050299	0.019048	-
0.004593	-0.073625	-0.000325	0.026904	0.035698	-0.003600	-
0.107538	-0.026949	-0.123128	0.042073	0.003506	0.038756	-
0.086360	-0.038627	0.061503	0.021048	-0.056632	-0.060566	-
0.007850	0.030761	-0.048807	0.082093	-0.032862	-0.002672	
0.010481	-0.095296	0.038237	0.012736	-0.006230	0.028331	
x65	0.093868	-0.115256	-0.090216	0.005496	-0.022294	-
0.067851	-0.094017	-0.043591	0.006871	0.037811	0.086280	-
0.001391	0.046220	-0.030603	-0.022000	-0.065282	0.011834	
0.031772	-0.046703	0.011208	-0.126487	0.112617	0.038977	
0.009559	0.165293	0.045493	0.063088	-0.149832	0.043399	-
0.066345	-0.077314	-0.002057	-0.095264	-0.031688	-0.167561	
y65	0.020929	-0.071625	0.041520	0.034684	0.025375	-
0.076308	-0.005426	0.027729	0.089409	-0.058480	0.044204	-
0.065018	-0.008032	-0.070081	-0.059335	-0.028919	-0.091942	
0.067081	0.043390	0.070866	-0.007304	-0.057637	-0.008429	-
0.136870	0.108299	-0.115080	-0.024410	0.032924	0.039351	
0.073349	-0.004222	0.045882	0.019638	-0.009834	0.014230	
z65	-0.055498	0.106145	-0.063808	-0.053730	0.027944	
0.017818	-0.065525	0.014517	0.015460	0.021848	-0.005272	-
0.139884	-0.021102	-0.119392	0.037256	0.030784	0.022185	-
0.105869	-0.040648	0.086476	0.031672	-0.043284	-0.033062	
0.014385	-0.017973	-0.058187	0.082579	-0.057271	-0.017532	-
0.040461	-0.102271	-0.028294	0.047696	-0.051154	0.065698	
x66	0.087510	-0.085166	-0.098249	-0.001405	-0.012535	-
0.053802	-0.096802	-0.070312	-0.051007	0.047831	0.074476	-
0.074520	0.005487	-0.052343	-0.020983	-0.042583	0.015420	
0.045438	-0.006174	0.016368	-0.009030	0.097884	0.033379	
0.026839	0.094537	0.011237	0.029419	-0.076643	0.024287	-
0.024509	-0.001654	0.000402	-0.066726	0.012635	-0.124771	
y66	-0.002916	-0.052891	0.032372	0.042084	0.047338	-
0.056133	-0.035389	-0.001487	0.086529	-0.070495	0.048932	-
0.081655	0.012722	-0.045727	-0.045665	-0.059592	-0.120359	
0.050908	0.039700	0.064749	-0.020383	-0.051965	0.022588	-
0.079673	0.045519	-0.068504	-0.020759	0.032724	-0.013673	
0.028642	-0.021224	0.037232	0.022640	-0.001882	-0.054969	
z66	-0.058117	0.115271	-0.065727	-0.030673	0.020303	
0.021200	-0.045425	0.048692	0.015902	0.000568	-0.032856	-
0.137725	-0.016732	-0.095951	0.034293	0.093641	0.006726	-
0.084944	-0.031339	0.082827	0.007765	0.001025	-0.016792	-
0.009700	-0.005829	-0.021036	0.052157	-0.049890	-0.019696	-
0.037063	-0.024861	-0.033202	0.006826	0.003737	-0.047783	
x67	0.082652	-0.058156	-0.104177	-0.004922	-0.001952	-
0.045580	-0.095685	-0.087239	-0.094471	0.051560	0.058999	-
0.126608	-0.029379	-0.064927	-0.031471	-0.021377	0.018015	

0.053227	0.030478	0.033478	0.077635	0.084784	0.040411	
0.036209	0.028207	-0.008609	0.008586	0.000277	0.017752	
0.019113	0.040597	0.000898	-0.051059	0.011669	-0.076703	
y67	-0.021717	-0.037920	0.020648	0.049264	0.067826	-
0.040150	-0.061672	-0.019443	0.091981	-0.084868	0.052736	-
0.079575	0.031575	-0.019069	-0.055007	-0.089215	-0.137824	
0.043740	0.029540	0.050117	-0.049392	-0.038291	0.057107	-
0.041431	-0.028998	-0.025703	-0.002284	0.035712	-0.024184	
0.035242	-0.018276	0.025878	-0.010955	-0.011540	-0.053245	
z67	-0.059133	0.118633	-0.070936	-0.010563	0.018966	
0.019209	-0.031967	0.074927	0.028776	-0.028789	-0.054241	-
0.114740	-0.009595	-0.069416	0.011835	0.134676	-0.013951	-
0.063711	-0.027597	0.087197	-0.044283	0.039893	0.003654	-
0.031909	-0.025743	0.012182	0.027196	-0.028478	0.005431	-
0.024436	0.021926	-0.031644	-0.057833	0.008040	-0.040647	
x68	0.073891	-0.032185	-0.106194	-0.003092	0.006071	-
0.039137	-0.094609	-0.099965	-0.123650	0.054115	0.054494	-
0.155929	-0.041956	-0.068469	-0.030001	0.004428	0.004130	
0.057640	0.041327	0.057952	0.130262	0.069967	0.019356	
0.057392	-0.041437	-0.012086	-0.039023	0.022638	-0.014902	
0.026890	0.075735	-0.034978	-0.067108	-0.015282	-0.044482	
y68	-0.038473	-0.019630	0.010925	0.058052	0.087559	-
0.028803	-0.078671	-0.047867	0.094603	-0.102152	0.061692	-
0.065376	0.056038	0.000023	-0.043244	-0.095497	-0.165675	
0.035972	0.000730	0.040383	-0.076807	-0.024991	0.038835	-
0.006525	-0.100938	0.025159	-0.016438	0.010361	-0.009349	
0.005935	-0.011964	0.039244	-0.079901	-0.033910	-0.112177	
z68	-0.062559	0.117273	-0.073602	0.009364	0.014600	
0.014663	-0.020211	0.086020	0.039652	-0.052875	-0.058797	-
0.091879	0.013147	-0.042687	0.011856	0.163121	-0.040506	-
0.040764	-0.034606	0.091741	-0.092320	0.071546	-0.001882	-
0.036548	-0.056479	0.057786	-0.012840	-0.034697	0.001673	-
0.037511	0.067394	-0.047207	-0.111813	0.031130	-0.045028	
x69	0.069795	-0.006510	-0.106739	-0.005305	0.022874	-
0.037616	-0.091825	-0.099296	-0.141364	0.038024	0.043552	-
0.162350	-0.063481	-0.061749	-0.043863	0.022660	0.006637	
0.053138	0.056907	0.043488	0.165727	0.031995	0.011056	
0.050123	-0.072026	-0.023037	-0.078476	0.069434	0.018178	
0.039651	0.091752	0.015612	-0.012852	-0.018009	-0.000702	
y69	-0.046167	-0.005731	0.003964	0.065034	0.098002	-
0.020660	-0.095066	-0.058730	0.097369	-0.120593	0.060145	-
0.054956	0.065515	0.018325	-0.044236	-0.117215	-0.153381	
0.009761	-0.022303	-0.005850	-0.088346	-0.040385	0.031607	-
0.007664	-0.142202	0.051895	-0.062895	-0.018679	0.011848	-
0.031474	-0.001505	0.076975	-0.060873	-0.010531	-0.071915	
z69	-0.059966	0.110481	-0.075159	0.023506	0.012991	
0.004843	-0.008085	0.100270	0.044857	-0.077801	-0.064546	-
0.068041	0.014979	-0.018687	0.003504	0.162587	-0.038935	-
0.024578	-0.023374	0.074752	-0.101665	0.088153	0.009555	-
0.053334	-0.062183	0.057688	-0.027578	-0.016722	0.016666	-
0.024013	0.084829	0.000727	-0.099203	0.057408	-0.014767	
x70	0.064040	0.016880	-0.104861	-0.005110	0.039078	-
0.033133	-0.075900	-0.090476	-0.161078	0.015468	0.037220	-
0.150004	-0.078503	-0.049933	-0.053097	0.031473	0.014207	
0.047694	0.053834	0.037635	0.167281	0.025861	0.004730	
0.028814	-0.083614	-0.034824	-0.069441	0.075613	-0.008901	
0.076625	0.052592	0.011200	-0.013111	0.012266	0.067765	
y70	-0.048454	0.004176	-0.003001	0.067398	0.092751	-
0.018084	-0.084058	-0.064958	0.075029	-0.134269	0.061895	-
0.053106	0.063898	0.030015	-0.025917	-0.121892	-0.114561	-

0.018483	-0.029045	-0.036926	-0.065219	-0.020881	0.019345	-
0.013982	-0.173603	0.066435	-0.027297	-0.022862	-0.008389	
0.001795	-0.064126	0.070298	-0.107161	0.058615	0.041455	
z70	-0.056028	0.097531	-0.073589	0.033415	0.003476	-
0.005280	0.007729	0.107808	0.045524	-0.090372	-0.061115	-
0.042941	0.012795	0.002252	0.007570	0.141135	-0.034369	-
0.008397	-0.018198	0.060357	-0.095114	0.118925	0.017876	-
0.066583	-0.052363	0.034943	-0.026067	-0.014364	0.016367	-
0.004145	0.098064	0.014523	-0.088955	0.068034	-0.009533	
x71	0.060131	0.034205	-0.101381	-0.001415	0.053359	-
0.044005	-0.067222	-0.075838	-0.171086	-0.003436	0.026046	-
0.121547	-0.091165	-0.036365	-0.066195	0.029358	0.002539	
0.037335	0.052133	0.013337	0.137724	0.011536	-0.022970	
0.005370	-0.065309	-0.028773	-0.032906	0.087284	-0.011012	
0.088193	0.023044	0.015619	0.031286	0.039073	0.056097	
y71	-0.041130	0.007088	-0.003972	0.067178	0.073361	-
0.028613	-0.072201	-0.060405	0.042778	-0.127178	0.065043	-
0.066398	0.048037	0.036508	-0.021793	-0.125773	-0.064450	-
0.057198	-0.028801	-0.060181	-0.027547	-0.030741	-0.006742	-
0.009381	-0.161246	0.066007	0.021817	-0.030228	0.023449	
0.024764	-0.092796	0.028720	-0.059209	0.025703	0.063181	
z71	-0.050033	0.075887	-0.072138	0.040899	-0.008683	-
0.025743	0.017010	0.108089	0.048405	-0.095410	-0.052440	-
0.009687	0.009792	0.024263	0.015456	0.105583	-0.032277	
0.007615	0.001761	0.041178	-0.073992	0.141142	0.021243	-
0.064218	-0.029281	0.003188	-0.025774	-0.014258	0.006281	
0.007179	0.104346	0.031639	-0.058297	0.071497	-0.037139	
x72	0.057494	0.047933	-0.095863	-0.001867	0.074327	-
0.057865	-0.057526	-0.057191	-0.166039	-0.027699	0.020673	-
0.058064	-0.094774	-0.006736	-0.091087	0.038339	0.006293	
0.023405	0.051391	-0.007434	0.075786	-0.014453	-0.046085	-
0.006748	-0.021956	-0.026045	-0.006516	0.080642	-0.007579	
0.046163	-0.011216	0.012411	0.064474	0.052495	0.046861	
y72	-0.026474	0.003954	-0.007407	0.056236	0.056412	-
0.047509	-0.057894	-0.058974	0.010623	-0.113575	0.062617	-
0.045403	0.020983	0.046706	-0.026884	-0.100924	0.013268	-
0.096398	-0.016893	-0.092206	0.023955	-0.033916	-0.043649	
0.004366	-0.084447	0.051846	0.077026	-0.011530	0.046689	
0.011536	-0.076425	-0.028985	-0.012361	-0.022790	0.029542	
z72	-0.042527	0.047982	-0.069049	0.046922	-0.026063	-
0.048287	0.026288	0.102217	0.054976	-0.098157	-0.037556	
0.019198	0.013348	0.052437	0.024196	0.059530	-0.029981	
0.015221	0.025739	0.031207	-0.048518	0.156079	0.046718	-
0.055128	-0.014433	-0.038329	-0.044470	-0.019101	-0.022524	
0.011066	0.096930	0.044476	-0.010302	0.077521	-0.023538	
x73	0.052205	0.060009	-0.088268	-0.001436	0.102034	-
0.061123	-0.047753	-0.034655	-0.140764	-0.056680	0.028163	
0.020538	-0.066117	0.031864	-0.110561	0.065393	-0.001078	
0.010950	0.018591	-0.034844	-0.042969	-0.018632	-0.051107	-
0.028519	0.009611	-0.002907	-0.044556	0.033151	-0.013017	-
0.019236	-0.052759	-0.015782	0.083694	0.132808	0.094797	
y73	-0.004231	-0.004321	-0.010201	0.045339	0.039356	-
0.055386	-0.035605	-0.070170	0.004818	-0.095196	0.056311	-
0.037076	0.005916	0.043760	-0.033065	-0.042190	0.079342	-
0.153256	-0.040755	-0.132925	0.057868	0.013438	-0.013270	-
0.036502	-0.009706	0.046535	0.021071	-0.010254	0.068256	-
0.068768	-0.023821	-0.133210	0.066986	0.075910	0.027230	
z73	-0.034057	0.014169	-0.063328	0.053569	-0.053395	-
0.080160	0.041259	0.099995	0.052759	-0.094292	-0.020091	
0.054450	0.016345	0.090220	0.051481	-0.002804	-0.002443	

0.011721	0.066514	0.017402	0.016118	0.129782	0.048267	-
0.038348	-0.003230	-0.059801	-0.022291	0.010754	-0.080450	
0.051487	0.035839	0.045216	-0.000262	0.035979	-0.037735	
x74	0.044813	0.073821	-0.076381	0.010849	0.098817	-
0.076484	-0.027603	0.022601	-0.103006	-0.091037	0.031248	
0.117403	-0.027738	0.098252	-0.094981	0.099726	-0.023937	-
0.013914	-0.008040	-0.051227	-0.137063	-0.077731	-0.043947	-
0.051167	0.020173	0.048434	-0.088899	0.016290	-0.077626	
0.013874	-0.065519	-0.007751	0.062278	0.091925	-0.047841	
y74	0.017463	-0.011438	0.004341	0.061284	-0.030254	-
0.073076	-0.002763	-0.021844	-0.016931	-0.089617	0.044829	-
0.011637	-0.003454	0.070936	0.017415	0.013074	0.161556	-
0.202839	-0.001147	-0.161759	0.137598	-0.011165	0.037914	-
0.111777	-0.035213	0.117493	-0.037124	-0.018942	-0.008423	
0.046397	-0.002809	-0.113020	-0.024017	-0.068446	-0.067931	
z74	-0.020588	-0.025553	-0.061884	0.046975	-0.060389	-
0.113965	0.047738	0.074215	0.040399	-0.086462	0.016868	
0.088476	-0.002735	0.102577	0.052203	-0.103959	0.037401	
0.016779	0.106618	-0.011218	0.041195	0.132967	0.043939	-
0.021756	0.054343	-0.167513	0.058108	0.034737	-0.101378	
0.023144	-0.044308	0.057550	0.109872	0.031487	0.063930	
x75	0.023136	-0.008199	-0.025167	-0.011569	-0.032656	-
0.024813	-0.022692	-0.039978	0.009588	0.017131	-0.018354	
0.120046	-0.105576	-0.023239	-0.028665	-0.050489	0.160386	
0.064992	0.051094	-0.023793	-0.186270	0.077140	-0.012613	-
0.031271	-0.129206	-0.149989	0.071389	-0.059001	0.119632	-
0.031714	-0.052174	-0.123700	-0.091943	-0.124173	0.169073	
y75	-0.018784	-0.005035	0.082397	-0.040135	0.040939	-
0.082621	0.017256	0.002745	0.057613	0.059448	0.073142	
0.057919	-0.078166	0.026174	-0.011264	0.142753	-0.096803	
0.010826	0.118994	0.034327	0.083025	-0.033607	-0.071365	
0.008695	-0.064888	-0.049284	-0.024516	-0.068684	0.024105	-
0.021212	-0.066468	0.027295	-0.086304	-0.002581	-0.032124	
z75	-0.041354	-0.031799	0.014817	-0.037657	0.112716	
0.039690	-0.129826	0.077570	-0.036100	0.004893	0.027108	-
0.027692	-0.035519	0.114302	0.012285	-0.014146	0.026687	-
0.129584	-0.144649	-0.055973	0.000883	-0.034055	0.044307	
0.126434	0.108818	-0.077922	-0.026642	0.045587	0.006407	-
0.120369	0.293841	-0.054278	0.127152	0.137839	-0.011792	
x76	0.034010	-0.015802	-0.006211	-0.003415	-0.062496	-
0.039559	0.005973	-0.042764	0.002719	0.027277	-0.022957	
0.080404	-0.126748	-0.020932	-0.021454	-0.058503	0.120606	
0.032443	0.081764	0.014637	-0.195688	0.050137	-0.015941	-
0.031359	-0.103813	-0.079766	0.075365	-0.008281	0.059974	-
0.020673	0.044427	-0.108844	-0.062304	-0.007427	0.105982	
y76	-0.018796	0.000273	0.084166	-0.044620	0.057427	-
0.075461	-0.006193	0.000634	0.052554	0.061624	0.077361	
0.064098	-0.069901	0.026844	-0.013664	0.167351	-0.084198	
0.004295	0.101635	0.013740	0.058981	-0.034569	-0.050203	
0.042201	-0.029199	-0.022598	-0.028273	-0.081987	0.041176	-
0.037309	-0.063988	0.028045	-0.066540	0.017406	0.000133	
z76	-0.046737	-0.020481	0.001257	-0.029411	0.106112	
0.044759	-0.120740	0.059133	-0.035935	-0.029193	0.020884	-
0.018230	-0.023108	0.086896	0.024600	0.001548	0.052130	-
0.060435	-0.073927	-0.049006	-0.017169	-0.011694	0.028145	
0.099446	0.083769	-0.082060	0.030111	0.048686	-0.000122	-
0.094415	0.191769	-0.083726	0.034368	0.081397	-0.005274	
x77	0.043901	-0.018638	0.014764	-0.004065	-0.088364	-
0.045183	0.030494	-0.033887	-0.003863	0.041093	-0.028380	
0.041081	-0.157818	-0.015363	-0.006259	-0.046093	0.086157	

0.017436	0.085648	0.033793	-0.159077	0.026596	-0.023065	-
0.039588	-0.087075	-0.019601	0.059156	-0.008195	0.026242	
0.005214	0.098365	-0.045547	-0.054190	0.083360	0.066622	
y77	-0.019612	0.003705	0.082170	-0.043607	0.070417	-
0.070542	-0.025203	-0.004620	0.047299	0.054953	0.086235	
0.069690	-0.053028	0.023427	-0.015165	0.170234	-0.071989	-
0.006884	0.088399	0.003054	0.029863	-0.033589	-0.031088	
0.066752	0.011448	-0.009135	-0.041324	-0.078997	0.028446	-
0.046231	-0.058709	0.011443	-0.018760	-0.029196	-0.021168	
z77	-0.050836	-0.012229	-0.007394	-0.022954	0.092963	
0.048805	-0.107181	0.049208	-0.035992	-0.056767	0.011269	-
0.017707	-0.023000	0.062176	0.044560	0.021847	0.071209	-
0.004409	-0.013171	-0.034307	-0.007945	0.004123	0.021606	
0.069697	0.057464	-0.070835	0.058399	0.055130	0.000702	-
0.066715	0.093469	-0.079246	-0.038766	0.032004	-0.046893	
x78	0.050642	-0.021003	0.034419	-0.011995	-0.109376	-
0.058717	0.044464	-0.027291	-0.006600	0.049595	-0.016680	
0.024509	-0.167124	-0.013943	-0.005771	-0.047327	0.062491	-
0.000974	0.068865	0.043846	-0.119191	0.012739	-0.032520	-
0.044577	-0.069944	0.008813	-0.015628	-0.034639	0.030737	-
0.003785	0.127321	0.046181	-0.030353	0.106939	0.016640	
y78	-0.020469	0.007381	0.077752	-0.038155	0.079501	-
0.060261	-0.036878	-0.009451	0.036794	0.043425	0.086516	
0.062300	-0.047525	0.024028	-0.007170	0.173544	-0.062188	-
0.017277	0.081046	-0.003713	0.000958	-0.030366	-0.019726	
0.071389	0.038821	0.015934	-0.018327	-0.048253	0.010991	-
0.030485	-0.047189	-0.007840	0.010125	-0.024970	0.005581	
z78	-0.055088	-0.007315	-0.013387	-0.021473	0.078404	
0.046146	-0.097683	0.042728	-0.035103	-0.076481	0.010197	-
0.005843	-0.019245	0.037363	0.054506	0.028624	0.093791	
0.040561	0.022017	-0.021480	-0.000526	0.020172	0.014793	
0.040866	0.033009	-0.063842	0.041343	0.031396	0.027253	-
0.062945	0.019684	-0.037218	-0.091048	-0.013877	-0.068193	
x79	0.055285	-0.018703	0.049844	-0.013524	-0.119485	-
0.069542	0.051033	-0.027732	-0.011480	0.046140	-0.012701	
0.003140	-0.172811	-0.006638	-0.010898	-0.035258	0.026492	-
0.018477	0.071825	0.041618	-0.102404	-0.003785	-0.032833	-
0.043055	-0.059559	0.031534	-0.047794	-0.029372	0.008837	
0.012457	0.098996	0.075914	-0.022685	0.087044	-0.009075	
y79	-0.020684	0.009641	0.073349	-0.034281	0.081361	-
0.048694	-0.045125	-0.009430	0.033604	0.034101	0.088599	
0.059759	-0.038990	0.020482	0.005355	0.163647	-0.045167	-
0.025728	0.064491	0.003615	-0.015145	-0.026621	-0.007452	
0.082818	0.061705	0.037709	0.005963	-0.029252	0.009186	-
0.037222	-0.008292	0.000749	0.017503	-0.003046	0.027019	
z79	-0.058856	-0.004025	-0.016988	-0.015482	0.065707	
0.041810	-0.088649	0.036467	-0.035132	-0.091208	0.004877	-
0.002782	-0.021277	0.015879	0.057346	0.039180	0.109278	
0.074495	0.059085	-0.015080	-0.001698	0.027557	0.010268	
0.018786	0.011802	-0.049807	0.027132	0.016534	0.036244	-
0.028456	-0.063093	-0.023187	-0.123459	-0.064701	-0.050717	
x80	0.060100	-0.014683	0.059111	-0.016086	-0.124768	-
0.078915	0.049820	-0.025387	-0.015000	0.039412	-0.012325	-
0.013234	-0.165086	0.000117	-0.006904	-0.026652	-0.001998	-
0.023583	0.069192	0.047945	-0.098133	-0.028435	-0.028927	-
0.023528	-0.038126	0.044574	-0.051023	-0.013831	-0.005874	
0.011234	0.089406	0.082425	-0.017330	0.090283	-0.053233	
y80	-0.022738	0.012084	0.072050	-0.028716	0.079466	-
0.036503	-0.047512	-0.012772	0.030518	0.023899	0.090614	
0.054932	-0.034738	0.018672	0.008989	0.156414	-0.029086	-

0.037322	0.056471	-0.000440	-0.016837	-0.013957	-0.002011	
0.077144	0.068230	0.048777	0.022069	-0.018285	0.008645	-
0.025626	-0.007076	0.010032	0.025605	0.003010	0.069425	
z80	-0.060892	-0.003746	-0.021272	-0.010851	0.049878	
0.034957	-0.082368	0.039033	-0.034406	-0.097206	-0.003893	-
0.001250	-0.025766	-0.003838	0.060352	0.040203	0.119290	
0.099744	0.084589	0.001109	-0.011089	0.015037	0.016722	
0.011418	0.003857	-0.039129	0.013059	0.004642	0.045904	-
0.011390	-0.094024	-0.010770	-0.136079	-0.071206	-0.069880	
x81	0.062041	-0.005185	0.069263	-0.017998	-0.125028	-
0.078985	0.049730	-0.020492	-0.025921	0.029469	-0.003236	-
0.023777	-0.150673	0.006748	0.008597	-0.023778	-0.017081	-
0.021383	0.042165	0.030767	-0.073979	-0.018637	-0.044319	-
0.006769	-0.002667	0.047701	-0.012356	-0.045695	-0.028010	
0.028236	0.084247	0.083638	-0.011548	0.087368	-0.083305	
y81	-0.024405	0.013241	0.068246	-0.022413	0.075625	-
0.029279	-0.048059	-0.016717	0.031206	0.012823	0.089907	
0.045181	-0.029112	0.016617	0.004155	0.140357	-0.015745	-
0.045268	0.059534	0.004188	-0.021176	-0.008379	0.010214	
0.064265	0.066445	0.040458	0.014420	0.006110	0.014037	-
0.013059	-0.015778	0.024524	0.045925	-0.026595	0.094430	
z81	-0.063001	-0.004440	-0.021399	-0.004975	0.031018	
0.030688	-0.073349	0.048053	-0.038500	-0.095939	-0.007843	
0.001834	-0.035846	-0.021264	0.069279	0.031067	0.133927	
0.114439	0.085358	0.007693	-0.005313	0.017346	0.004540	
0.004911	0.008583	-0.006533	0.022925	-0.033646	0.045077	
0.014402	-0.092622	0.017992	-0.137015	-0.040685	-0.054406	
x82	0.061864	0.006711	0.075826	-0.017995	-0.118953	-
0.080814	0.047951	-0.021318	-0.032686	0.009839	-0.000710	-
0.035267	-0.120330	0.018420	0.013709	-0.005951	-0.040347	-
0.028459	0.026725	0.012330	-0.060575	-0.025285	-0.031148	
0.028033	0.011541	0.031055	0.014250	-0.042911	-0.032415	
0.024720	0.023490	0.053952	-0.004352	0.031864	-0.047790	
y82	-0.025937	0.013911	0.064297	-0.017031	0.068723	-
0.016808	-0.054140	-0.016733	0.033300	-0.000300	0.092235	
0.042342	-0.031640	0.011644	0.010316	0.113510	0.006260	-
0.041842	0.050196	0.020056	-0.019537	-0.001506	0.003021	
0.043736	0.064827	0.044214	0.034741	0.021592	0.016063	
0.013593	0.004595	0.038603	0.033933	-0.016407	0.042578	
z82	-0.065283	-0.007569	-0.018790	0.004976	0.009461	
0.020367	-0.057573	0.054951	-0.041485	-0.088976	-0.020481	-
0.002977	-0.041033	-0.033853	0.067718	0.029219	0.139941	
0.105832	0.089575	0.014545	0.000926	-0.002754	0.014911	
0.008971	0.007401	0.017253	-0.003457	-0.044187	0.063838	
0.038021	-0.097494	0.043624	-0.105189	-0.034723	0.037069	
x83	0.060716	0.020281	0.078496	-0.021267	-0.105006	-
0.079654	0.037705	-0.024462	-0.041524	-0.019848	0.005089	-
0.038255	-0.082043	0.031496	0.018878	0.012463	-0.059754	-
0.029425	0.006976	-0.000274	-0.058274	-0.021357	-0.014546	
0.061876	0.010382	0.004978	0.057189	-0.044389	-0.035558	
0.008348	-0.056335	-0.017267	-0.025274	-0.021700	-0.071251	
y83	-0.028698	0.014450	0.062125	-0.008187	0.055526	-
0.005819	-0.052480	-0.017636	0.036295	-0.009857	0.089926	
0.033221	-0.033259	0.004542	0.015582	0.085731	0.025634	-
0.037862	0.051222	0.028721	-0.003647	0.007989	-0.010161	
0.021199	0.072053	0.046865	0.049210	0.044017	0.017768	
0.041661	0.015393	0.085953	0.034298	-0.021602	0.054022	
z83	-0.065568	-0.014582	-0.015901	0.012056	-0.011772	
0.009971	-0.045326	0.064661	-0.045622	-0.082048	-0.029506	-
0.005986	-0.050563	-0.043762	0.061035	0.018812	0.141553	

0.084764	0.081207	0.038813	-0.003210	-0.026958	0.038749	
0.013402	-0.007989	0.037123	-0.029665	-0.058499	0.079007	
0.044422	-0.076959	0.041250	-0.084536	0.013728	0.057526	
x84	0.057236	0.037903	0.079713	-0.025146	-0.084808	-
0.074614	0.022124	-0.026900	-0.050493	-0.052617	0.014270	-
0.039221	-0.028302	0.042886	0.031580	0.031835	-0.082344	-
0.023969	-0.014535	-0.033332	-0.040101	-0.004642	-0.006299	
0.095781	0.026252	-0.039125	0.125722	-0.052499	-0.046716	
0.002021	-0.138792	-0.092298	-0.041576	-0.095297	-0.075660	
y84	-0.030935	0.014742	0.060023	0.003239	0.039231	
0.005955	-0.047130	-0.020322	0.038673	-0.021676	0.085324	
0.024676	-0.042670	-0.000497	0.018548	0.050277	0.060928	-
0.034521	0.040280	0.054523	0.004790	0.006063	-0.024445	-
0.002825	0.051782	0.041689	0.061708	0.070717	0.034202	
0.079887	0.016420	0.124886	0.038481	-0.012032	0.040087	
z84	-0.066210	-0.022954	-0.009904	0.017890	-0.033523	
0.001354	-0.034192	0.078593	-0.048702	-0.066449	-0.034717	-
0.014210	-0.056649	-0.053833	0.065301	0.002241	0.123702	
0.054961	0.077822	0.046600	0.016366	-0.034453	0.057343	
0.010220	-0.003487	0.052944	-0.046064	-0.073762	0.075067	
0.070509	-0.030373	0.045416	-0.028825	0.124294	0.090708	
x85	0.057204	0.044302	0.077670	-0.020045	-0.073502	-
0.059049	0.016461	-0.033838	-0.040021	-0.080047	0.017744	-
0.049507	-0.005678	0.021788	0.035712	0.044745	-0.052655	-
0.002535	-0.036009	-0.034497	-0.002132	0.001136	0.009103	
0.065906	0.042318	-0.032558	0.117350	-0.046775	-0.009929	-
0.030469	-0.073858	-0.031690	-0.062259	-0.098034	0.004023	
y85	-0.036032	0.016159	0.058288	0.013194	0.032049	
0.016876	-0.042770	-0.024685	0.043673	-0.012808	0.085949	
0.027270	-0.046221	-0.004608	0.017687	0.022711	0.068224	-
0.036442	0.042107	0.059720	-0.008885	0.028703	-0.018915	-
0.016752	0.040459	0.027810	0.029064	0.100072	0.037439	
0.098828	0.020835	0.097522	0.017414	-0.027828	0.000945	
z85	-0.068412	-0.024379	-0.004367	0.017482	-0.045953	-
0.024001	-0.023756	0.081833	-0.055246	-0.047480	-0.045598	-
0.011734	-0.036556	-0.040153	0.040202	-0.000511	0.081237	
0.026665	0.054182	0.025063	0.006930	-0.051565	0.074150	
0.032827	-0.013409	0.073036	-0.062659	-0.031584	0.031778	
0.037184	-0.066565	0.014845	0.012773	0.039820	0.070171	
x86	0.057330	0.051438	0.078076	-0.016806	-0.063817	-
0.042628	0.009196	-0.035432	-0.030934	-0.101299	0.021946	-
0.059335	0.017905	0.000996	0.037422	0.050392	-0.029613	
0.008986	-0.051005	-0.039850	0.032862	0.010127	0.018016	
0.033645	0.066521	-0.030837	0.109318	-0.039946	0.012535	-
0.052875	-0.006662	0.033990	-0.073795	-0.071407	0.058876	
y86	-0.042006	0.015956	0.055096	0.027112	0.024803	
0.025612	-0.036526	-0.034904	0.044634	-0.001092	0.083967	
0.034222	-0.054362	-0.007344	0.018396	-0.002326	0.079727	-
0.035502	0.032883	0.068944	-0.026911	0.039515	-0.029141	-
0.027164	0.020729	0.021981	0.001721	0.110235	0.031632	
0.107187	-0.009887	0.058661	0.005694	-0.054413	-0.049819	
z86	-0.069326	-0.022134	0.003370	0.011860	-0.058157	-
0.044259	-0.021104	0.093796	-0.060694	-0.033026	-0.052834	-
0.012916	-0.014605	-0.032160	0.017971	-0.004821	0.037516	-
0.005100	0.047233	0.002946	0.000819	-0.044072	0.092954	
0.037736	-0.011845	0.079261	-0.054054	-0.001939	-0.009885	
0.027944	-0.051238	0.021778	0.058803	0.027618	0.029165	
x87	0.058213	0.058532	0.077404	-0.012703	-0.055972	-
0.026175	0.004061	-0.040900	-0.018182	-0.118437	0.022841	-
0.062738	0.035896	-0.015538	0.031753	0.056450	-0.010496	

0.017258	-0.053281	-0.035484	0.042374	0.013630	0.030646	
0.011679	0.071334	-0.026264	0.096482	-0.019792	0.026350	-
0.077492	0.018592	0.061827	-0.103240	-0.054703	0.096833	
y87	-0.049900	0.014911	0.051470	0.040174	0.018242	
0.031479	-0.034605	-0.034946	0.042785	0.006499	0.082536	
0.036826	-0.056012	-0.007789	0.022576	-0.022177	0.077825	-
0.039295	0.019872	0.073494	-0.026826	0.042073	-0.028011	-
0.028159	0.009163	0.012810	-0.027985	0.118191	0.034636	
0.118425	0.002124	0.031336	0.037815	-0.063374	-0.089841	
z87	-0.068004	-0.020055	0.008553	0.008604	-0.068461	-
0.061792	-0.017332	0.091261	-0.062461	-0.019456	-0.063323	-
0.011977	0.000947	-0.024929	-0.010362	-0.000269	0.003578	-
0.029390	0.046059	-0.002672	-0.023410	-0.052796	0.108237	
0.041247	-0.018566	0.092766	-0.038203	0.039656	-0.051965	-
0.000435	-0.092961	0.007572	0.024928	0.015898	0.016445	
x88	0.058573	0.066875	0.079030	-0.007032	-0.050041	-
0.007735	-0.000382	-0.041157	-0.009905	-0.123696	0.030960	-
0.065837	0.051146	-0.030770	0.034069	0.048751	0.006610	
0.027080	-0.066877	-0.049413	0.066885	0.022894	0.014641	-
0.005599	0.094381	-0.039618	0.079568	-0.022244	0.035352	-
0.071892	0.066825	0.082245	-0.088419	-0.028495	0.098906	
y88	-0.059365	0.012984	0.045376	0.053269	0.009630	
0.037308	-0.028167	-0.034109	0.036685	0.008901	0.079454	
0.041307	-0.062492	-0.008700	0.020948	-0.033464	0.078909	-
0.044098	0.015818	0.084131	-0.026211	0.041415	-0.007561	-
0.025197	-0.013592	0.010362	-0.039309	0.118105	0.014340	
0.112219	-0.007327	-0.008507	0.039542	-0.061088	-0.072758	
z88	-0.065870	-0.016002	0.014455	0.009280	-0.076595	-
0.077219	-0.017610	0.087869	-0.065010	0.003363	-0.064725	-
0.013086	0.017923	-0.021369	-0.016665	-0.012818	-0.030467	-
0.029452	0.014594	-0.034471	-0.016095	-0.045444	0.073631	
0.040286	0.013453	0.075261	-0.030764	0.039857	-0.054165	-
0.003562	-0.036701	0.011072	0.037397	0.002475	-0.049418	
x89	0.059201	0.074193	0.080074	-0.002463	-0.045442	
0.008747	-0.004564	-0.043353	0.000009	-0.131085	0.034228	-
0.061849	0.062198	-0.039776	0.024615	0.046753	0.017489	
0.021846	-0.063881	-0.042897	0.063495	0.023301	0.015894	-
0.017000	0.093351	-0.040352	0.039881	0.009440	0.043438	-
0.075431	0.066332	0.081021	-0.073761	-0.006300	0.098490	
y89	-0.068763	0.009132	0.038050	0.066903	0.001100	
0.040332	-0.027624	-0.032318	0.030554	0.014061	0.073731	
0.041372	-0.067115	-0.014154	0.024160	-0.052432	0.070806	-
0.040000	0.006474	0.088291	-0.023114	0.040843	0.001589	-
0.031771	-0.016887	0.010096	-0.030902	0.098176	-0.002924	
0.099592	0.005642	-0.028248	0.050368	-0.043026	-0.084265	
z89	-0.063003	-0.011878	0.019044	0.008044	-0.082448	-
0.093336	-0.016906	0.078983	-0.067023	0.012052	-0.071693	-
0.006004	0.030643	-0.010542	-0.041629	-0.000366	-0.064397	-
0.053990	-0.003165	-0.038324	-0.029400	-0.061262	0.066020	
0.041498	0.015831	0.071389	-0.041972	0.078920	-0.045597	-
0.030050	-0.046015	-0.004996	0.002319	-0.021554	-0.048932	
x90	0.059833	0.082298	0.082587	0.002344	-0.042536	
0.027240	-0.008115	-0.041902	0.006265	-0.129996	0.043564	-
0.057021	0.069755	-0.048770	0.022360	0.033663	0.031093	
0.015608	-0.060127	-0.045779	0.061550	0.032862	0.000789	-
0.030199	0.094651	-0.052082	0.001869	0.024788	0.035624	-
0.057212	0.070784	0.078143	-0.035960	0.014821	0.077117	
y90	-0.079263	0.003085	0.027686	0.080999	-0.007980	
0.038465	-0.023172	-0.030542	0.021926	0.017999	0.060678	
0.044677	-0.072345	-0.020896	0.022224	-0.069865	0.056920	-

0.034943	-0.007590	0.092954	-0.016513	0.036734	0.009261	-
0.038320	-0.012662	0.016412	-0.016128	0.070399	-0.026267	
0.051745	0.006068	-0.052541	0.022908	-0.021059	-0.061585	
z90	-0.059011	-0.004665	0.025742	0.006609	-0.086237	-
0.101728	-0.021791	0.070882	-0.074119	0.023307	-0.059068	-
0.005771	0.043079	-0.005022	-0.043258	0.008310	-0.086518	-
0.059626	-0.025605	-0.060696	-0.025656	-0.053941	0.041700	
0.027395	0.025306	0.050585	-0.025169	0.088837	-0.028221	
0.001085	0.008647	0.010373	0.023933	-0.037719	-0.061379	
x91	0.060354	0.090276	0.084262	0.008288	-0.042501	
0.046123	-0.012685	-0.039973	0.015325	-0.122908	0.052990	-
0.047792	0.076304	-0.054380	0.017867	0.017523	0.039967	
0.008533	-0.045754	-0.043666	0.046054	0.041151	-0.016824	-
0.027819	0.084902	-0.064867	-0.039236	0.038854	0.013830	-
0.032589	0.054978	0.036386	-0.002645	0.038902	0.042791	
y91	-0.090659	-0.003753	0.016741	0.094342	-0.015310	
0.032796	-0.020891	-0.026695	0.009472	0.020895	0.044087	
0.045557	-0.076546	-0.027137	0.013251	-0.083556	0.031000	-
0.032721	-0.022482	0.085962	-0.003883	0.021769	0.022885	-
0.052738	0.001437	0.026502	0.007425	0.045313	-0.035673	
0.008363	0.033942	-0.055192	0.014168	0.014021	-0.030408	
z91	-0.053352	0.002306	0.030029	0.008828	-0.092783	-
0.104681	-0.031396	0.061648	-0.075566	0.034473	-0.041967	-
0.007257	0.057171	-0.003545	-0.034560	0.023168	-0.094999	-
0.059172	-0.048047	-0.057002	-0.017411	-0.054090	-0.004493	
0.037089	0.027429	0.018192	-0.001226	0.052409	-0.032017	
0.012505	0.027160	-0.018860	0.000839	-0.043287	-0.032375	
x92	0.060980	0.098208	0.086948	0.013398	-0.043051	
0.065235	-0.014962	-0.035980	0.022848	-0.113145	0.063244	-
0.035536	0.078899	-0.055938	0.009177	-0.001482	0.046504	-
0.007346	-0.020839	-0.037936	0.019614	0.047383	-0.033399	-
0.021820	0.061969	-0.081206	-0.097482	0.067818	-0.007847	-
0.004326	0.023014	-0.011640	0.046629	0.072958	-0.004348	
y92	-0.104194	-0.012325	0.003861	0.110607	-0.026158	
0.024779	-0.020879	-0.020281	-0.005306	0.027086	0.027288	
0.044815	-0.076003	-0.035740	0.011980	-0.087720	-0.003390	-
0.027415	-0.052334	0.079162	0.012971	-0.008156	0.020725	-
0.058145	0.016611	0.050048	0.040031	-0.001796	-0.049159	-
0.046391	0.084201	-0.070728	-0.004621	0.058892	0.015616	
z92	-0.046593	0.009795	0.035308	0.007477	-0.091919	-
0.106629	-0.044817	0.048796	-0.077740	0.033561	-0.029875	-
0.004315	0.062883	0.003307	-0.036416	0.030160	-0.101575	-
0.064019	-0.043032	-0.048989	-0.013025	-0.031157	-0.019918	
0.024146	0.034188	-0.022463	0.003765	0.046679	-0.028168	
0.019807	0.027599	-0.018341	-0.019533	-0.012348	0.002596	
x93	0.060984	0.107056	0.089957	0.019094	-0.046279	
0.084836	-0.018355	-0.029879	0.029498	-0.097587	0.073517	-
0.022404	0.079677	-0.053224	0.000746	-0.016204	0.049543	-
0.025276	0.012792	-0.034634	-0.017543	0.051850	-0.060552	-
0.004865	0.029217	-0.098839	-0.166038	0.100284	-0.041977	
0.038520	-0.020270	-0.078520	0.100224	0.119008	-0.072539	
y93	-0.117314	-0.022308	-0.009514	0.125136	-0.032423	
0.013915	-0.021472	-0.013147	-0.023985	0.027543	0.004867	
0.040656	-0.083221	-0.043128	0.001846	-0.086921	-0.044532	-
0.021600	-0.077890	0.068555	0.040441	-0.039471	0.032739	-
0.073045	0.040712	0.061705	0.094299	-0.055803	-0.038406	-
0.103292	0.123675	-0.076130	-0.027833	0.136069	0.085993	
z93	-0.039172	0.019055	0.039980	0.009979	-0.097550	-
0.103674	-0.056867	0.036807	-0.077052	0.042134	-0.008961	
0.006938	0.078743	0.006249	-0.024236	0.021183	-0.093068	-

0.062905	-0.040495	-0.035744	-0.014221	-0.008999	-0.073961	
0.014761	0.032135	-0.050089	0.011283	0.028519	-0.058776	
0.016372	0.066205	-0.036123	-0.040948	-0.034921	-0.005231	
x94	0.052121	0.103382	0.082971	0.043611	-0.039451	
0.092655	-0.026282	-0.009164	0.020799	-0.085562	0.047018	-
0.006262	0.064899	-0.069829	-0.008923	-0.032895	0.029492	
0.000542	0.005599	-0.010178	-0.022057	0.042545	-0.040983	
0.012727	0.026113	-0.045961	-0.139128	0.067467	-0.022844	
0.026545	-0.013422	-0.121871	0.084851	0.069642	-0.054715	
y94	-0.118657	-0.021214	-0.013788	0.120240	-0.046465	
0.011879	-0.005154	-0.018252	-0.039965	0.026149	0.010693	
0.020290	-0.061475	-0.036222	0.007101	-0.073304	-0.054654	-
0.013063	-0.062629	0.053094	0.043601	-0.034170	-0.012858	-
0.073462	0.046713	0.030821	0.101608	-0.092884	-0.044211	-
0.093213	0.084930	-0.049789	-0.024384	0.101083	0.073436	
z94	-0.026814	0.030538	0.047455	-0.002684	-0.093285	-
0.090516	-0.060570	0.024744	-0.072348	0.034294	-0.001054	
0.018248	0.086512	0.007857	-0.028987	0.030050	-0.073160	-
0.067609	-0.013274	-0.002798	-0.027597	-0.006836	-0.083439	-
0.006862	0.001021	-0.062681	0.010701	0.042332	-0.015380	
0.002205	0.057104	0.007369	-0.042772	-0.025213	-0.032911	
x95	0.043039	0.098091	0.074292	0.062365	-0.033726	
0.097685	-0.030187	0.008172	0.015998	-0.075136	0.023571	
0.005827	0.046156	-0.085036	-0.021234	-0.039829	0.011616	
0.016769	0.002239	0.011442	-0.032299	0.025967	-0.020335	
0.025690	0.018469	-0.000949	-0.104314	0.047719	-0.004337	-
0.002498	-0.017529	-0.143033	0.040634	0.022961	-0.016073	
y95	-0.117441	-0.019281	-0.018061	0.111422	-0.055927	
0.010694	0.008825	-0.023084	-0.048966	0.017663	0.008063	
0.005761	-0.049384	-0.030161	0.008966	-0.054726	-0.052029	-
0.010177	-0.050598	0.039685	0.043207	-0.037368	-0.044206	-
0.073949	0.053561	0.005634	0.112026	-0.103733	-0.018385	-
0.088543	0.047311	-0.011408	-0.032438	0.056810	0.062716	
z95	-0.015342	0.044094	0.055692	-0.005395	-0.089457	-
0.077995	-0.056345	0.014855	-0.072480	0.044391	0.018013	
0.018757	0.104799	0.007525	-0.033518	0.007424	-0.061594	-
0.054394	0.007747	0.007037	-0.006420	0.020314	-0.090601	-
0.010523	-0.027420	-0.095642	-0.008408	0.020557	-0.015891	
0.024729	0.043374	0.013693	0.002753	0.007711	-0.032988	
x96	0.033495	0.094176	0.064773	0.077345	-0.028163	
0.100835	-0.030502	0.025910	0.013182	-0.059990	0.008658	
0.012064	0.029818	-0.098887	-0.030673	-0.042454	-0.001420	
0.031384	-0.012663	0.015920	-0.029348	0.004535	-0.006899	
0.042955	0.009131	0.025383	-0.076884	0.026587	0.009030	-
0.010946	-0.013791	-0.153961	0.026662	-0.016068	-0.003249	
y96	-0.117871	-0.016458	-0.022213	0.103774	-0.067678	
0.009275	0.018938	-0.029227	-0.052860	0.016388	0.008836	-
0.008002	-0.031739	-0.023828	0.013396	-0.038291	-0.051748	-
0.001843	-0.046132	0.017750	0.042334	-0.035404	-0.079046	-
0.066955	0.051198	-0.017621	0.105287	-0.110710	-0.006559	-
0.082108	0.024911	-0.003781	-0.029162	0.030032	0.044678	
z96	-0.002277	0.055095	0.060919	-0.006492	-0.081244	-
0.063502	-0.053735	0.003216	-0.061189	0.043122	0.016590	
0.031829	0.105341	0.017764	-0.044019	-0.004997	-0.050353	-
0.039266	0.054525	0.030197	-0.019254	0.044065	-0.068317	-
0.007692	-0.045417	-0.100877	0.015760	-0.000816	-0.016795	
0.026631	-0.005586	0.027005	-0.001875	0.017441	0.020614	
x97	0.024624	0.087529	0.055785	0.086104	-0.022746	
0.100948	-0.024929	0.041376	0.008921	-0.046132	0.003371	
0.015227	0.007246	-0.110447	-0.040736	-0.043478	-0.012538	

0.041459	-0.017146	0.015288	-0.027766	-0.002991	0.003488	
0.049709	0.001521	0.037267	-0.044786	0.006453	0.007612	-
0.014756	-0.018424	-0.140824	0.025550	-0.020540	-0.000697	
y97	-0.116473	-0.012814	-0.025694	0.093401	-0.074934	
0.008329	0.029839	-0.035111	-0.056265	0.010188	0.003847	-
0.017258	-0.021712	-0.017056	0.007762	-0.021383	-0.042144	
0.006294	-0.033282	-0.003513	0.034490	-0.026539	-0.100783	-
0.063683	0.050099	-0.041306	0.096326	-0.107757	0.012932	-
0.064750	-0.004688	0.022559	-0.020462	0.012072	0.030804	
z97	0.007743	0.066974	0.064346	-0.008265	-0.076172	-
0.050268	-0.060780	-0.000424	-0.052439	0.049163	0.012971	
0.043294	0.113349	0.026806	-0.036329	-0.009635	-0.043321	-
0.028777	0.062033	0.052033	-0.024224	0.047230	-0.054269	-
0.019733	-0.072238	-0.067169	0.015089	-0.006716	-0.021046	
0.043969	-0.012690	0.031732	-0.013590	0.007088	-0.001617	
x98	0.015160	0.078524	0.044502	0.088439	-0.017606	
0.098771	-0.020036	0.054052	0.003617	-0.030139	0.003904	
0.017270	-0.012906	-0.120140	-0.046747	-0.039472	-0.028201	
0.046710	-0.011964	0.015055	-0.034138	-0.017533	0.021500	
0.049134	-0.009803	0.043287	-0.016774	-0.018404	-0.007681	-
0.002684	-0.041556	-0.119376	0.020278	-0.000697	-0.024664	
y98	-0.115884	-0.009298	-0.029471	0.083170	-0.083594	
0.007157	0.036882	-0.042489	-0.054246	0.008419	-0.001005	-
0.023576	-0.005830	-0.010237	0.006249	-0.005259	-0.035324	
0.019572	-0.024965	-0.024831	0.019116	-0.014892	-0.121626	-
0.053045	0.039176	-0.055379	0.069289	-0.096051	0.020366	-
0.054755	-0.028734	0.028653	-0.017319	0.005773	0.017792	
z98	0.019532	0.080257	0.069374	-0.007652	-0.063505	-
0.038381	-0.058274	-0.004018	-0.038018	0.051554	0.013835	
0.046479	0.107695	0.036140	-0.038791	-0.022021	-0.023037	-
0.017071	0.057422	0.052125	-0.003382	0.065194	-0.043777	-
0.017670	-0.074273	-0.044004	0.027104	-0.000714	-0.002863	
0.038318	-0.002861	0.042289	0.010399	-0.016885	0.044270	
x99	0.005398	0.070419	0.035859	0.086915	-0.011431	
0.095266	-0.007595	0.065819	-0.001316	-0.015596	0.008385	
0.014409	-0.030640	-0.129131	-0.055106	-0.032829	-0.026833	
0.050412	-0.022698	0.000755	-0.032449	-0.027523	0.041532	
0.031193	-0.004960	0.026775	0.005804	-0.033714	-0.012996	
0.014150	-0.065127	-0.080115	0.019782	-0.034124	-0.032788	
y99	-0.114685	-0.004129	-0.032979	0.071429	-0.089107	
0.006290	0.045481	-0.050459	-0.049635	0.005649	-0.012652	-
0.026399	0.006103	-0.001613	-0.003940	0.009990	-0.022526	
0.031844	-0.018696	-0.048296	0.001681	0.001129	-0.134272	-
0.042235	0.026548	-0.067311	0.035050	-0.070236	0.040097	-
0.042822	-0.048810	0.041973	-0.004439	0.002154	0.019070	
z99	0.031170	0.090239	0.069504	-0.006899	-0.055868	-
0.027056	-0.061589	-0.007475	-0.020742	0.060846	0.016107	
0.048517	0.098432	0.051189	-0.033405	-0.029950	-0.027548	-
0.006462	0.063035	0.062916	0.009573	0.064137	-0.024916	-
0.003456	-0.078818	0.002594	0.033642	-0.008947	-0.023892	
0.031810	0.009407	0.022853	0.013414	0.020185	0.051263	
x100	-0.005883	0.058848	0.022838	0.079557	-0.007471	
0.086791	0.009408	0.074811	-0.006566	0.005725	0.021102	
0.009266	-0.048367	-0.138265	-0.058329	-0.021821	-0.034932	
0.047919	-0.022085	-0.026187	-0.033666	-0.039403	0.041525	
0.017152	-0.025422	0.006722	0.032675	-0.054369	-0.037351	
0.018293	-0.067373	-0.022502	0.025859	0.010403	-0.057617	
y100	-0.114047	0.000974	-0.036327	0.060016	-0.096755	
0.005425	0.047908	-0.058881	-0.041289	0.006468	-0.022328	-
0.028060	0.021227	0.006306	-0.007918	0.026344	-0.007489	

0.049521	-0.018893	-0.070440	-0.023126	0.019344	-0.150326	-
0.022281	0.004406	-0.062902	-0.014666	-0.031746	0.047008	-
0.028335	-0.056312	0.020805	0.002899	-0.007674	0.018019	
z100	0.046211	0.101397	0.072804	-0.007067	-0.039830	-
0.015685	-0.065742	-0.003797	-0.006355	0.063308	0.017722	
0.045941	0.076083	0.065606	-0.031360	-0.037717	-0.010674	
0.001379	0.043006	0.083951	0.037830	0.053552	0.019132	-
0.007694	-0.065978	0.050235	0.045092	-0.002011	0.000468	
0.034152	-0.009605	0.016702	0.019236	-0.022766	0.050005	
x101	-0.015794	0.049930	0.011566	0.066995	-0.004895	
0.080133	0.030769	0.080510	-0.011706	0.024121	0.035577	
0.002087	-0.070114	-0.137411	-0.061940	-0.011062	-0.026538	
0.034798	-0.032223	-0.061081	-0.031528	-0.063288	0.049387	
0.005483	-0.028560	-0.017964	0.023087	-0.045157	-0.040416	
0.024292	-0.056159	0.056912	0.062571	0.027553	-0.079542	
y101	-0.113446	0.006676	-0.039534	0.047673	-0.100188	
0.002799	0.052305	-0.068480	-0.028776	0.006003	-0.036400	-
0.027890	0.034712	0.012838	-0.019984	0.043835	0.008857	
0.068476	-0.015689	-0.089235	-0.052329	0.046578	-0.152467	-
0.005900	-0.021480	-0.069028	-0.066961	0.011687	0.056327	-
0.012127	-0.077573	0.018434	0.012803	0.000275	0.033265	
z101	0.058618	0.109348	0.073673	-0.004544	-0.023143	-
0.009225	-0.072014	0.001798	0.009676	0.074002	0.026819	
0.040226	0.055020	0.076548	-0.028000	-0.041084	-0.010068	
0.018520	0.026090	0.109646	0.072206	0.055758	0.057502	-
0.024023	-0.054909	0.091758	0.083112	-0.013853	-0.015267	
0.036177	-0.030765	-0.004602	0.008453	-0.019222	0.046448	
x102	-0.028630	0.038197	-0.001584	0.052011	-0.001011	
0.064412	0.055136	0.089316	-0.014348	0.044694	0.051273	-
0.009307	-0.087137	-0.136383	-0.073995	0.006233	-0.034453	
0.024800	-0.037826	-0.087789	-0.012254	-0.062076	0.061209	-
0.004376	-0.047111	-0.034104	0.048239	-0.057329	-0.061890	
0.056830	-0.070234	0.132649	0.062644	0.036819	-0.079831	
y102	-0.111367	0.012534	-0.042542	0.033163	-0.103448	
0.003442	0.052085	-0.081718	-0.013580	0.006288	-0.048712	-
0.023261	0.046711	0.019001	-0.026241	0.061723	0.036704	
0.089592	-0.015196	-0.122154	-0.089977	0.056104	-0.150944	
0.006627	-0.050228	-0.077911	-0.133131	0.062443	0.069611	-
0.001153	-0.077569	-0.012854	0.024243	0.027080	0.021254	
z102	0.075224	0.116228	0.073661	-0.005797	-0.006276	
0.003484	-0.076054	0.005820	0.025393	0.086304	0.039883	
0.029172	0.023901	0.088694	-0.006220	-0.050455	0.011712	
0.026323	-0.002654	0.114549	0.098730	0.013101	0.098784	-
0.040511	-0.024469	0.132041	0.088335	-0.001836	-0.010318	-
0.010555	-0.015768	-0.022496	0.037504	-0.028796	0.012858	
x103	-0.041395	0.022559	-0.019532	0.025656	0.003947	
0.055021	0.077229	0.094767	-0.029265	0.046530	0.060556	-
0.004022	-0.072589	-0.114549	-0.058877	0.009246	-0.028788	
0.023271	-0.034793	-0.096457	0.006445	-0.047853	0.053289	-
0.043475	-0.078713	-0.026797	0.043027	0.026109	-0.062371	
0.040920	-0.036770	0.116447	0.050133	-0.017545	-0.016796	
y103	-0.100204	0.023195	-0.030514	0.009172	-0.111237	
0.008397	0.029767	-0.092944	0.015366	0.017961	-0.056204	-
0.012410	0.052789	0.018811	-0.033568	0.080422	0.046919	
0.066984	-0.026929	-0.102653	-0.061556	0.056891	-0.093025	
0.014374	-0.049081	-0.026883	-0.088328	0.063404	0.036419	-
0.000185	-0.052255	-0.040936	0.016942	-0.008383	-0.016146	
z103	0.080223	0.114202	0.055867	0.026978	0.023444	
0.010867	-0.062332	0.020482	0.027686	0.095384	0.043875	
0.012501	0.010714	0.099630	-0.000813	-0.054802	0.012638	

0.052048	-0.007428	0.055842	0.086528	0.016119	0.041691	-
0.064529	-0.049613	0.087984	0.033304	-0.060768	-0.027793	-
0.029870	-0.034062	-0.039388	0.026153	-0.011041	-0.000881	
x104	-0.055782	0.007523	-0.032746	-0.003177	0.001124	
0.046241	0.082298	0.096837	-0.032911	0.047388	0.070057	-
0.002971	-0.051424	-0.087788	-0.037650	0.021668	-0.040958	
0.014549	-0.023759	-0.121571	0.024622	-0.025141	0.048477	-
0.061675	-0.107986	0.014829	0.063599	0.067836	-0.097461	
0.041748	0.011950	0.033012	0.008713	-0.026218	0.019351	
y104	-0.089243	0.032924	-0.019394	-0.008008	-0.112717	
0.013172	0.016844	-0.101655	0.039433	0.029446	-0.057205	-
0.008612	0.056374	0.021049	-0.054999	0.077704	0.053901	
0.059331	-0.034789	-0.076813	-0.040240	0.048828	-0.049490	
0.020770	-0.032363	-0.020223	-0.076178	0.054133	0.013295	-
0.024646	-0.036311	-0.024519	0.008497	-0.027057	-0.041326	
z104	0.084954	0.108288	0.037129	0.056966	0.052864	
0.014055	-0.039371	0.033856	0.025919	0.102019	0.046145	-
0.000476	-0.006389	0.099367	-0.001921	-0.065752	0.025556	
0.077835	-0.010895	0.025395	0.073280	0.006064	-0.010796	-
0.083539	-0.050232	0.024201	-0.039476	-0.091499	-0.019462	-
0.074443	-0.053492	-0.000992	0.032829	-0.018938	-0.014219	
x105	-0.068037	-0.001072	-0.039828	-0.033706	-0.001583	
0.040352	0.084387	0.092807	-0.031622	0.049022	0.076566	-
0.011807	-0.026085	-0.055716	-0.019380	0.018584	-0.046783	
0.010644	-0.014998	-0.123739	0.033305	-0.014771	0.047539	-
0.071533	-0.124358	0.050656	0.055330	0.083099	-0.110262	
0.006531	0.036836	0.008385	-0.042571	-0.044927	0.041194	
y105	-0.079223	0.040369	-0.010228	-0.024269	-0.109597	
0.017346	0.003065	-0.102407	0.052686	0.036404	-0.052396	
0.002894	0.044982	0.017044	-0.072754	0.074736	0.057332	
0.048115	-0.044108	-0.057701	-0.027571	0.041988	-0.009205	
0.039288	-0.004279	-0.015444	-0.044749	0.046745	0.006197	-
0.008620	-0.022623	-0.045285	0.024416	-0.037370	-0.031291	
z105	0.087634	0.099519	0.018247	0.083933	0.076781	
0.015134	-0.022717	0.042984	0.023317	0.106350	0.047935	-
0.001560	-0.025018	0.091943	-0.006489	-0.065320	0.031593	
0.090175	-0.008562	-0.014710	0.061345	0.008808	-0.043277	-
0.088542	-0.046385	-0.012697	-0.074290	-0.101103	-0.008955	-
0.087093	-0.048449	0.002256	0.044920	-0.015601	0.009604	
x106	-0.079781	-0.004518	-0.041327	-0.067326	0.000055	
0.035251	0.087251	0.085128	-0.033346	0.043699	0.088094	-
0.027048	-0.010975	-0.026089	-0.005672	0.014152	-0.049808	-
0.005621	-0.006253	-0.130145	0.038524	-0.003471	0.038681	-
0.087637	-0.119738	0.053847	0.063265	0.091277	-0.111569	
0.014058	0.041191	0.000223	-0.071275	-0.069050	0.066346	
y106	-0.070074	0.044982	-0.002365	-0.037981	-0.107646	
0.023654	-0.017024	-0.095833	0.064085	0.048894	-0.046707	
0.019176	0.038526	0.014674	-0.087875	0.061373	0.062841	
0.054658	-0.044711	-0.029666	-0.020396	0.031318	0.034142	
0.063104	0.018911	-0.002291	-0.046714	0.033262	-0.000420	-
0.030296	-0.010659	-0.068305	0.045298	-0.050065	-0.051261	
z106	0.088357	0.087434	-0.001160	0.110966	0.091835	
0.013448	-0.009946	0.045287	0.025798	0.112561	0.044479	-
0.000658	-0.033465	0.078195	-0.002241	-0.057789	0.028752	
0.094113	-0.003615	-0.047742	0.041158	0.008897	-0.068058	-
0.077555	-0.042938	-0.027893	-0.100078	-0.100463	-0.014448	-
0.111678	-0.032291	-0.014353	0.040730	-0.011820	0.018125	
x107	-0.091724	-0.006425	-0.038939	-0.102408	-0.005799	
0.031609	0.081560	0.072918	-0.031929	0.046455	0.101981	-
0.037656	0.003559	0.000160	0.006682	-0.006617	-0.048355	

0.002375	-0.006316	-0.116926	0.026267	0.007990	0.030411	-
0.091311	-0.101667	0.073394	0.049735	0.064441	-0.080733	-
0.026853	0.018745	-0.019916	-0.062417	-0.085206	0.048167	
y107	-0.060570	0.052298	0.008211	-0.049012	-0.098619	
0.028792	-0.029701	-0.080750	0.068685	0.045025	-0.039757	
0.027618	0.026406	0.018287	-0.108488	0.052697	0.069014	
0.047181	-0.032447	-0.020887	-0.014104	0.021279	0.081068	
0.071995	0.010094	-0.010175	-0.024092	0.030463	-0.038532	-
0.010704	0.038257	-0.017590	0.003727	-0.038930	-0.019687	
z107	0.089143	0.074024	-0.019732	0.134117	0.104304	
0.009238	0.005078	0.040879	0.028120	0.109328	0.034480	
0.003750	-0.045594	0.061250	-0.005691	-0.043145	0.025742	
0.083549	0.007120	-0.080792	0.023920	0.010622	-0.064748	-
0.059539	-0.036244	-0.036692	-0.082425	-0.072816	-0.010230	-
0.111838	-0.005320	0.005819	0.033274	-0.015832	0.038272	
x108	-0.108298	0.003919	-0.035760	-0.154219	-0.008478	
0.034075	0.083607	0.073791	-0.025992	0.031140	0.138425	-
0.054837	0.027666	0.009873	0.021101	-0.024168	-0.053051	
0.052426	0.028965	-0.111484	-0.012092	0.001939	-0.014364	-
0.078762	-0.019154	0.069838	0.077068	0.150240	0.041888	-
0.102160	-0.034334	0.040981	0.001970	-0.096454	-0.100336	
y108	-0.042283	0.033315	0.021373	-0.025711	-0.092839	
0.027184	-0.064508	-0.113283	0.049773	0.099581	-0.030661	
0.045154	-0.005593	0.073825	-0.123913	0.077706	0.082043	-
0.046143	-0.113222	-0.005223	0.025009	0.068956	0.222331	
0.100416	-0.093262	-0.017131	-0.084689	-0.128654	-0.196076	
0.150074	0.145353	-0.012255	-0.145716	-0.114312	0.155273	
z108	0.090727	0.047463	-0.039839	0.170310	0.104606	
0.001909	0.010818	0.010438	0.026070	0.136654	0.031998	
0.013328	-0.052142	0.059720	0.003039	0.005188	0.018697	
0.037721	-0.013496	-0.119716	0.002899	0.034257	-0.018869	-
0.011157	-0.045636	-0.025306	-0.062708	-0.058863	-0.049268	-
0.052413	0.052218	0.020407	-0.045639	-0.087305	0.034075	
x109	-0.118796	0.002006	-0.018544	-0.188573	-0.018536	
0.032266	0.049239	0.055618	-0.028722	0.040702	0.141954	-
0.065511	0.024242	0.069344	0.020215	-0.044362	-0.016576	
0.020888	0.016037	-0.071975	0.008836	0.043934	0.017985	-
0.066894	-0.028811	0.077459	-0.003682	-0.013315	-0.018917	-
0.087562	0.020865	0.019033	0.015867	-0.056936	-0.028331	
y109	-0.035386	0.045617	0.022824	-0.039754	-0.074607	
0.033233	-0.050170	-0.087569	0.052785	0.067842	-0.008722	
0.040266	0.011330	0.031563	-0.124449	0.023536	0.027396	
0.004351	-0.068741	0.013140	0.001895	0.000762	0.211508	
0.095215	-0.057123	-0.052159	-0.053091	-0.064062	-0.083200	
0.100868	0.091997	-0.014332	-0.105633	0.025514	0.062950	
z109	0.087793	0.032061	-0.060372	0.186051	0.104242	-
0.008701	0.032921	0.000859	0.037932	0.121886	0.008473	
0.018920	-0.041611	0.016950	0.006966	0.015736	-0.008514	
0.028438	0.026213	-0.140215	-0.022541	0.008415	-0.010349	
0.023255	-0.003095	-0.006618	-0.021089	-0.002634	-0.002177	-
0.071855	0.066959	-0.000988	-0.034166	-0.008112	-0.011422	
x110	-0.130689	0.009724	0.000807	-0.234658	-0.024142	
0.031850	0.019819	0.045572	-0.024310	0.032738	0.150633	-
0.077516	0.027230	0.112664	0.017007	-0.072684	0.022150	
0.009397	0.028770	-0.012925	0.018476	0.062953	0.029161	-
0.054114	-0.008627	0.076166	-0.075772	-0.119034	-0.012200	-
0.087617	0.021033	-0.007774	0.049860	0.045015	-0.022503	
y110	-0.024237	0.052571	0.028870	-0.046191	-0.047185	
0.040191	-0.033061	-0.071876	0.043960	0.041439	0.002783	
0.021658	0.008575	0.011131	-0.150046	-0.023248	-0.013638	

0.059419	-0.023268	0.003711	-0.018340	-0.026406	0.199512	
0.070567	0.010984	-0.088408	-0.033190	-0.081571	0.073180	
0.046084	0.040646	0.053431	-0.038656	0.062086	-0.019571	
z110	0.089184	0.009302	-0.074607	0.201236	0.110947	-
0.021989	0.059163	-0.029027	0.044453	0.122412	-0.040226	
0.019589	-0.020769	-0.018610	-0.001125	0.065899	-0.022577	
0.014956	0.086475	-0.174876	-0.053155	0.019437	-0.001944	
0.050915	0.058196	0.059288	0.007773	-0.004156	0.126732	-
0.031370	0.051633	-0.009747	-0.013486	0.010979	-0.051528	
x111	-0.145285	0.024092	0.028213	-0.283385	-0.034972	
0.039887	-0.003798	0.032128	-0.016657	0.024258	0.166682	-
0.093301	0.030248	0.143708	0.001675	-0.105740	0.055666	
0.020790	0.029888	0.039860	0.012446	0.066049	0.028337	-
0.035492	0.040101	0.074299	-0.123841	-0.258688	0.044822	-
0.122685	-0.013766	-0.025908	0.128852	0.100494	-0.054232	
y111	-0.018378	0.058461	0.025477	-0.047233	-0.028389	
0.048600	-0.042237	-0.041018	0.035389	0.011840	0.029311	
0.013272	-0.014178	0.026903	-0.149477	-0.087874	-0.032159	
0.067965	-0.002300	0.018489	-0.050925	-0.037612	0.228379	
0.088213	0.073992	-0.148045	-0.024086	-0.044668	0.046297	-
0.029468	0.042026	0.112328	-0.124987	0.087873	-0.064710	
z111	0.082286	-0.012935	-0.103600	0.223359	0.091998	-
0.033006	0.067011	-0.052806	0.063783	0.093271	-0.050602	
0.040696	-0.043376	-0.062186	0.008594	0.076691	-0.046448	-
0.051679	0.079490	-0.184725	-0.076092	0.002430	0.106404	
0.145816	0.110069	0.075679	0.157277	0.173363	0.052571	-
0.059390	0.144347	0.031339	-0.099093	-0.011625	-0.024145	

Canonical Variate Analysis: CVA ... canaux

Dataset: canaux
Classification criterion: subfam

Groups		Observations
1.	Ca	9
2.	Ce	13
3.	Di	3
4.	Pr	11

Variation among groups, scaled by the inverse of the within-group variation

	Eigenvalues	% Variance	Cumulative %
1.	5.03001015	53.375	53.375
2.	2.32732137	24.696	78.071
3.	2.06653351	21.929	100.000

Mahalanobis distances among groups:

	Ca	Ce	Di
Ce	3.9345		
Di	5.9511	6.5881	
Pr	4.1925	5.0271	5.6198

P-values from permutation tests (10000 permutation rounds) for Mahalanobis distances among groups:

	Ca	Ce	Di
Ce	<.0001		

Di	0.0001	0.0009	
Pr	0.0001	<.0001	0.0016

Procrustes distances among groups:

	Ca	Ce	Di
Ce	0.0706		
Di	0.0610	0.0881	
Pr	0.0429	0.0766	0.0493

P-values from permutation tests (10000 permutation rounds) for Procrustes distances among groups:

	Ca	Ce	Di
Ce	0.0272		
Di	0.7040	0.0288	
Pr	0.5948	0.0012	0.8146

Canonical coefficients:

	CV1	CV2	CV3
x1	-23.3948	-9.5753	33.3983
y1	-20.3696	30.4091	-16.2218
z1	7.3789	-3.3450	9.2214
x2	-12.6351	-7.0544	28.3645
y2	-12.2524	38.0592	-27.1023
z2	-1.6346	0.1998	2.5953
x3	-12.0904	12.2543	17.6272
y3	12.0456	13.0142	-16.8450
z3	-6.3979	-0.8068	5.1125
x4	-4.5687	13.9276	11.4494
y4	21.4348	4.1445	-7.4579
z4	-14.4796	8.8483	-0.3858
x5	1.9915	23.1632	4.4480
y5	6.4389	8.1375	-12.2638
z5	-14.2920	5.6457	-0.4547
x6	4.0982	19.3174	2.5748
y6	1.4969	15.1884	-13.2487
z6	-10.3300	1.7488	-0.9123
x7	7.7379	13.2175	-0.2253
y7	-3.6599	14.4900	-9.8096
z7	-6.8592	-1.8899	-2.3594
x8	1.5466	17.0607	-5.6301
y8	-1.5723	-2.0176	-1.0938
z8	-4.1590	-2.4302	-3.9206
x9	-3.7054	16.2325	-10.7070
y9	-6.7678	-14.1694	4.8319
z9	2.1194	-4.2858	-4.8100
x10	-6.8414	10.5168	-13.3501
y10	-19.9746	-18.7635	10.5076
z10	14.5201	-13.7536	-3.6800
x11	-7.8746	12.6663	-21.8315
y11	-16.5735	-23.9911	12.5416
z11	5.6340	-1.7143	-3.8022
x12	5.6338	-2.0631	-24.5006
y12	-25.6477	-13.5282	8.5491
z12	10.4717	-0.8925	-1.8261
x13	15.1820	-14.2867	-21.0803
y13	-27.3775	-7.3575	4.1514
z13	12.2209	-0.7604	2.9190

x14	14.7274	-11.3372	-19.7066
y14	-17.9630	-12.2577	2.2109
z14	6.5409	6.6317	3.5720
x15	5.3100	-11.7726	-14.7478
y15	-4.7638	-11.4858	0.2566
z15	-8.2850	9.6351	4.8167
x16	10.3713	-18.2338	-9.9452
y16	3.7482	-11.2414	0.9318
z16	-4.9097	6.4979	0.5265
x17	5.5000	-15.3878	3.2892
y17	18.9157	-12.5885	-0.7544
z17	-7.1263	7.8819	6.7261
x18	8.5753	-16.8438	7.8337
y18	36.8139	-13.0950	-0.2043
z18	-2.0967	3.7384	8.2507
x19	1.0964	-10.2404	15.3823
y19	59.6348	-15.4125	1.0323
z19	-6.3025	0.3018	11.5152
x20	-0.9485	-13.4025	14.6591
y20	33.1739	-9.3667	6.6557
z20	-6.3458	1.6247	8.2143
x21	-4.7298	-3.0000	16.3350
y21	12.5427	-1.8261	11.8956
z21	-9.7932	8.3884	8.0098
x22	-9.0068	9.4793	12.7883
y22	-2.1137	7.4457	12.9100
z22	-7.0392	14.6085	3.4798
x23	-14.3292	17.6163	7.2020
y23	-18.7321	19.5351	11.2965
z23	-13.6032	22.1152	-2.3160
x24	-7.2914	10.4404	7.7033
y24	-23.4144	18.7154	12.8914
z24	-9.9404	14.6282	0.3331
x25	0.7423	5.7339	9.1969
y25	-17.3028	18.6238	12.8308
z25	-0.7437	9.0169	-1.2742
x26	9.0013	0.9911	-1.7784
y26	-3.9487	14.2640	-1.7584
z26	7.4054	1.3382	-6.6436
x27	15.3924	-1.3525	-1.1058
y27	-0.0396	8.8461	5.7401
z27	7.6002	-8.0795	-3.6504
x28	12.7690	0.3638	-3.5858
y28	-8.6509	14.1873	5.4434
z28	9.5152	-20.6477	-2.8511
x29	2.7817	3.5528	-3.9490
y29	11.8393	7.2414	-6.0983
z29	6.9691	-29.3306	-0.3571
x30	-3.6305	2.2322	5.2360
y30	26.6516	-2.5069	-1.4310
z30	-2.4858	-27.6227	-2.9018
x31	-16.1239	0.4139	12.1876
y31	19.8314	-10.9769	0.7085
z31	-6.6016	-24.1536	-4.6702
x32	-20.8992	-6.1044	20.0353
y32	16.3800	-30.7280	9.4890
z32	-8.1430	-18.2149	-7.5471
x33	-33.5750	-3.0420	21.1095
y33	-8.9240	-25.4241	8.2350
z33	-0.3723	-13.3017	-7.0158

x34	-14.1819	-10.3847	19.9606
y34	1.1507	-34.1112	9.1479
z34	-5.4805	-3.5783	-5.0603
x35	-10.9263	-1.1794	12.8017
y35	-1.4243	-21.2223	7.7703
z35	-4.0970	6.3883	-1.0564
x36	1.8738	2.0273	7.0784
y36	1.7323	-21.0508	14.5716
z36	7.2794	14.2584	0.9958
x37	30.3420	-6.0319	-0.1961
y37	-8.6411	-27.3520	11.9785
z37	7.5073	40.8319	1.4399
x38	47.8489	-30.3671	21.6464
y38	48.8026	-45.4183	47.8180
z38	5.3168	-26.7556	9.8103
x39	36.5790	-7.0632	9.9357
y39	20.7206	-28.5847	23.8576
z39	-0.2906	-23.2022	7.0635
x40	3.4408	2.9081	10.9734
y40	-4.3167	-7.1750	2.8420
z40	-20.2604	-12.4767	6.6160
x41	17.6263	18.1898	9.7128
y41	-12.4535	6.1442	-13.9011
z41	-15.2034	-11.1057	6.7247
x42	26.4404	11.7801	8.2766
y42	-22.6578	13.9649	-26.0777
z42	-14.2623	-8.4389	5.2213
x43	30.6725	-1.1575	16.1514
y43	-24.9760	12.6345	-31.2778
z43	-8.7000	-7.9264	5.1667
x44	9.4901	3.4015	24.8068
y44	-31.4315	13.8477	-30.3584
z44	-8.7138	4.0761	3.8797
x45	7.1586	-7.1484	31.1357
y45	-27.7147	9.2102	-25.8689
z45	-2.9513	12.5201	1.3026
x46	-1.8887	-7.5051	28.6279
y46	-22.9572	4.6407	-19.1516
z46	3.4047	25.5760	-3.6830
x47	-12.7184	-4.5178	25.1961
y47	-15.1244	-0.2676	-9.0890
z47	12.5427	39.0250	-9.2064
x48	-17.8097	-1.8996	17.6740
y48	-10.6205	-2.7681	-5.4509
z48	29.1296	10.1616	1.1571
x49	-16.1393	1.0287	6.8298
y49	2.5277	-10.1507	-0.0960
z49	35.9859	-9.9507	9.6961
x50	-21.1985	0.8443	6.5911
y50	10.4947	-15.1213	4.4575
z50	42.1143	-26.3203	15.4201
x51	-8.9408	-9.3829	8.3005
y51	16.7657	-17.5691	7.5826
z51	39.9396	-27.7851	16.8385
x52	-4.3672	1.1553	3.7084
y52	19.6706	-18.5061	11.9389
z52	32.8243	-29.2388	16.7408
x53	1.4177	-9.7037	1.5980
y53	17.9884	-16.0712	12.3017
z53	19.4170	-11.7285	14.3549

x54	0.7368	2.9428	-4.8341
y54	13.9919	-14.3011	12.9676
z54	7.2603	-1.1598	12.4748
x55	0.5027	11.7700	-9.0087
y55	8.3744	-11.8105	12.8170
z55	-12.4817	20.7324	4.7812
x56	-12.9879	32.6110	-21.8366
y56	4.6156	-9.0639	14.8811
z56	-22.6123	38.0367	2.2225
x57	0.9360	25.2763	-15.8575
y57	-15.2972	1.4412	7.0589
z57	-32.3508	39.9925	-0.1983
x58	-13.0528	28.9664	-16.6171
y58	-10.9142	5.5450	5.8386
z58	-12.4979	23.8354	4.5912
x59	-14.5622	25.1535	-14.8395
y59	-10.5143	10.0891	3.7320
z59	-1.6116	11.2034	8.7254
x60	-13.5098	26.5834	-17.1380
y60	-10.6674	7.8284	5.9395
z60	-2.5722	-3.2159	14.3167
x61	-2.9549	9.6665	-16.4956
y61	-19.9497	21.8344	6.7452
z61	-9.5323	0.9048	8.9743
x62	1.0949	-0.3567	-11.9891
y62	-18.0839	23.3509	-0.6115
z62	-15.7628	2.0075	-0.5792
x63	7.5494	-13.8372	-8.8595
y63	-11.3232	27.7306	-4.8783
z63	-18.3941	4.3040	-1.9752
x64	16.7403	-31.7040	-6.3130
y64	2.2209	26.5961	-2.7062
z64	-23.1450	4.1928	-1.6588
x65	27.0419	-53.3482	-3.9474
y65	9.2156	23.3088	-1.1255
z65	-35.5960	7.0493	-3.4598
x66	23.9466	-37.4202	-7.0839
y66	29.2000	-3.6115	-3.0908
z66	-3.8327	-18.9334	-2.4729
x67	11.9556	-22.7597	-8.0606
y67	32.5600	-13.8033	-7.7016
z67	-4.1799	-16.9043	-9.1924
x68	-1.6069	-14.3532	-14.2563
y68	38.0433	-33.6050	-10.0982
z68	1.9233	-17.6675	-18.9819
x69	-10.5221	1.5884	-6.9181
y69	33.8174	-23.6320	-12.2453
z69	2.9458	-5.3444	-18.5659
x70	-21.4458	22.1728	-14.1959
y70	14.8066	6.6144	-30.7646
z70	6.1960	1.3395	-16.4352
x71	-11.7610	20.8166	-8.2116
y71	3.3409	8.4064	-20.5067
z71	16.9926	0.2627	-10.0785
x72	-4.0023	19.0688	-3.5214
y72	0.3451	-3.3009	-2.0190
z72	21.5108	9.1378	-7.8870
x73	4.5812	34.6563	-13.7137
y73	14.2971	5.1621	1.4939
z73	19.9606	8.2427	-3.0449

x74	30.3420	-6.0319	-0.1961
y74	-8.6411	-27.3520	11.9785
z74	7.5073	40.8319	1.4399
x75	-70.7555	57.0086	-17.0739
y75	-1.1712	14.7099	-11.1162
z75	50.3553	-15.2346	15.7795
x76	-34.7283	34.0518	-21.7501
y76	-1.4457	15.0590	-11.6464
z76	32.7538	-10.2313	2.8506
x77	-14.3743	23.2941	-25.0913
y77	0.4771	0.2112	-1.7040
z77	23.1046	-14.3902	-3.1846
x78	-4.4220	15.0118	-18.9160
y78	-0.2251	-1.1834	-1.0545
z78	9.3458	-11.4650	-6.6571
x79	-3.1947	7.0234	-16.5029
y79	0.6703	-2.5550	-1.9507
z79	-9.4891	-3.3013	-11.5426
x80	8.5561	-7.1513	-13.7031
y80	-7.1342	5.3831	-3.5275
z80	-10.2568	-6.3573	-13.6940
x81	16.7913	-19.4810	-8.4930
y81	-16.7484	11.6286	-0.3167
z81	-11.5261	-3.6283	-17.4874
x82	4.9262	-17.5908	-6.0789
y82	-6.8384	1.3677	3.9147
z82	-29.6338	15.5860	-21.2071
x83	4.0681	-28.0326	-3.8260
y83	-12.3209	6.6295	6.2521
z83	-26.6782	18.5865	-24.9381
x84	-1.1927	-35.4297	0.5172
y84	-12.3868	9.7744	11.3722
z84	-11.6618	26.3373	-29.8867
x85	-20.0880	-15.7161	-2.2537
y85	-9.0880	5.0739	12.2858
z85	-15.8987	11.9383	-18.2450
x86	-28.1218	-0.8837	-4.5570
y86	-7.0695	-1.9753	14.6028
z86	-2.7115	-5.5609	-7.6945
x87	-35.4324	10.3619	-10.4296
y87	1.1047	-9.1093	21.6417
z87	-2.1174	-13.8236	-11.6800
x88	-29.9758	14.7299	-7.0337
y88	-2.3562	-5.3841	16.5575
z88	14.0512	-31.3307	1.0459
x89	-26.2182	20.0697	-7.0321
y89	3.9013	-9.3086	14.5007
z89	9.2371	-31.9242	0.1582
x90	-17.1241	21.0801	-2.5855
y90	1.1282	-5.6809	3.0689
z90	13.0986	-35.8628	11.8162
x91	-3.5862	18.7235	-1.0049
y91	1.6210	-2.2669	-5.3919
z91	6.7008	-28.5143	8.8322
x92	14.4757	15.8434	1.4515
y92	1.3814	1.2785	-17.3044
z92	3.3569	-12.8573	1.5266
x93	39.6994	8.9904	3.6620
y93	1.3179	12.7085	-32.9703
z93	0.2574	-11.8798	0.2328

x94	29.0442	1.7623	0.1086
y94	-2.4503	11.4860	-28.9903
z94	2.1193	-10.3262	3.6474
x95	11.5498	0.3290	-8.0270
y95	-10.3313	10.7611	-22.9070
z95	9.9321	-1.3469	6.5184
x96	2.7561	-5.5247	-7.8170
y96	-10.8957	9.3092	-17.1038
z96	0.4102	13.6143	-2.3141
x97	1.2309	-9.7411	-7.8038
y97	-11.5034	10.0713	-10.5146
z97	1.2504	7.9285	-1.6385
x98	8.2933	-18.7356	-9.0386
y98	-9.4679	11.6218	-7.1195
z98	-10.2751	18.2326	2.3612
x99	2.1793	-23.0195	-2.7858
y99	-9.5027	17.4792	-2.2079
z99	-2.5363	17.2536	-3.3904
x100	13.1298	-28.3109	-0.7873
y100	-8.8868	21.5711	1.1990
z100	-10.5543	13.9960	2.4198
x101	19.5075	-31.7301	10.1612
y101	-9.2863	32.2379	2.9498
z101	-9.8233	11.9307	0.2414
x102	19.5061	-32.5480	15.3357
y102	0.1012	37.3277	5.6092
z102	-4.3726	2.0261	7.0752
x103	-2.2179	-13.8789	16.0368
y103	2.5209	15.6722	12.0186
z103	0.8013	10.3152	2.1295
x104	-6.4936	-9.4477	7.3162
y104	4.7000	4.6910	15.4940
z104	-1.2249	21.1044	3.5181
x105	-13.4679	-5.6214	-1.3619
y105	4.4919	-0.4126	18.6427
z105	-4.8363	32.3889	2.9203
x106	-21.6762	0.4343	-4.6437
y106	9.9408	-10.1875	21.2486
z106	-4.9878	34.2741	-0.3720
x107	-19.2996	-0.3004	-4.2792
y107	5.9187	-10.2527	14.7585
z107	-8.6167	32.2440	0.1124
x108	15.1297	-23.9394	20.1646
y108	-52.4488	5.9360	-9.9044
z108	-22.5891	9.2487	2.4342
x109	7.6386	-6.8593	4.7572
y109	-2.1415	-1.9025	-10.2456
z109	5.5560	-9.7324	1.7405
x110	24.8518	5.7925	-6.7574
y110	27.1703	-10.6747	2.4742
z110	20.4097	-32.2668	10.5971
x111	44.4816	7.4969	-5.4702
y111	39.3807	-19.1608	-12.1347
z111	13.3845	-61.7704	5.6428

New Procrustes fit:
Dataset 'endolymphatic sac vs first turn'

38 landmarks in 3 dimensions.

The dataset contains 40 observations, of which 36 are included for analyses.

Average shape:

Lmk.	Axis 1 (x)	Axis 2 (y)	Axis 3 (z)
1	-0.01741777	0.05771991	-0.06633901
2	-0.00736983	0.05098244	-0.07523462
3	0.00435096	0.04515466	-0.08219976
4	0.01578437	0.03929954	-0.08734108
5	0.02669780	0.03294833	-0.09047084
6	0.03693627	0.02577452	-0.09164302
7	0.04689540	0.01800968	-0.09094052
8	0.05646687	0.00953199	-0.08835301
9	0.06547064	0.00016760	-0.08378930
10	0.07384659	-0.00988438	-0.07722438
11	0.08128518	-0.01973829	-0.06750190
12	0.08738504	-0.02863274	-0.05700909
13	0.09227991	-0.03626016	-0.04583719
14	0.09648225	-0.04227216	-0.03408693
15	0.10011491	-0.04649268	-0.02171479
16	0.10323982	-0.04895154	-0.00864216
17	0.10625248	-0.04937978	0.00515989
18	0.10953794	-0.04782164	0.01954178
19	0.11322454	-0.04437550	0.03414432
20	0.11704791	-0.03514102	0.05067870
21	0.12031850	-0.02472713	0.06497117
22	0.12280422	-0.01321558	0.07712215
23	0.12459723	-0.00055940	0.08706046
24	0.12525590	0.01309127	0.09491377
25	0.12488178	0.02794079	0.10063953
26	0.12310437	0.04381144	0.10447144
27	0.11985283	0.06069005	0.10630313
28	0.11467780	0.07837820	0.10635742
29	-0.13040548	0.00328977	0.00027654
30	-0.15455642	0.00197101	0.00813705
31	-0.17912707	0.00095307	0.01357550
32	-0.20212237	-0.00074695	0.01833940
33	-0.22322537	-0.00299112	0.02363936
34	-0.24313647	-0.00549876	0.02769063
35	-0.26274699	-0.00781073	0.02992779
36	-0.27987929	-0.01147130	0.03163242
37	-0.29659134	-0.01505481	0.03236287
38	-0.31221313	-0.01868860	0.03138231

Procrustes sums of squares: 0.2074615771642196
Tangent sums of squares: 0.20570421296574365

Data matrices in this dataset:
- endolymphatic sac vs first turn, raw data
- endolymphatic sac vs first turn, centroid size
- endolymphatic sac vs first turn, Procrustes coordinates

New Procrustes fit:
Dataset 'endolymphatic sac vs first turn'

38 landmarks in 3 dimensions.

The dataset contains 40 observations, of which 36 are included for analyses.

Average shape:

Lmk.	Axis 1 (x)	Axis 2 (y)	Axis 3 (z)
1	-0.01741777	0.05771991	-0.06633901
2	-0.00736983	0.05098244	-0.07523462
3	0.00435096	0.04515466	-0.08219976
4	0.01578437	0.03929954	-0.08734108
5	0.02669780	0.03294833	-0.09047084
6	0.03693627	0.02577452	-0.09164302
7	0.04689540	0.01800968	-0.09094052
8	0.05646687	0.00953199	-0.08835301
9	0.06547064	0.00016760	-0.08378930
10	0.07384659	-0.00988438	-0.07722438
11	0.08128518	-0.01973829	-0.06750190
12	0.08738504	-0.02863274	-0.05700909
13	0.09227991	-0.03626016	-0.04583719
14	0.09648225	-0.04227216	-0.03408693
15	0.10011491	-0.04649268	-0.02171479
16	0.10323982	-0.04895154	-0.00864216
17	0.10625248	-0.04937978	0.00515989
18	0.10953794	-0.04782164	0.01954178
19	0.11322454	-0.04437550	0.03414432
20	0.11704791	-0.03514102	0.05067870
21	0.12031850	-0.02472713	0.06497117
22	0.12280422	-0.01321558	0.07712215
23	0.12459723	-0.00055940	0.08706046
24	0.12525590	0.01309127	0.09491377
25	0.12488178	0.02794079	0.10063953
26	0.12310437	0.04381144	0.10447144
27	0.11985283	0.06069005	0.10630313
28	0.11467780	0.07837820	0.10635742
29	-0.13040548	0.00328977	0.00027654
30	-0.15455642	0.00197101	0.00813705
31	-0.17912707	0.00095307	0.01357550
32	-0.20212237	-0.00074695	0.01833940
33	-0.22322537	-0.00299112	0.02363936
34	-0.24313647	-0.00549876	0.02769063
35	-0.26274699	-0.00781073	0.02992779
36	-0.27987929	-0.01147130	0.03163242
37	-0.29659134	-0.01505481	0.03236287
38	-0.31221313	-0.01868860	0.03138231

Procrustes sums of squares: 0.2074615771642196
Tangent sums of squares: 0.20570421296574365

Data matrices in this dataset:
- endolymphatic sac vs first turn, raw data
- endolymphatic sac vs first turn, centroid size
- endolymphatic sac vs first turn, Procrustes coordinates

Principal Component Analysis: PCA: CovMatrix, endolymphatic sac vs first turn, Procrustes coordinates

Eigenvalues	% Variance	Cumulative %

1.	0.00224870	38.261	38.261
2.	0.00102278	17.402	55.663
3.	0.00072682	12.367	68.030
4.	0.00040254	6.849	74.879
5.	0.00032980	5.611	80.490
6.	0.00027993	4.763	85.253
7.	0.00020836	3.545	88.799
8.	0.00015692	2.670	91.469
9.	0.00010354	1.762	93.230
10.	0.00007098	1.208	94.438
11.	0.00005584	0.950	95.388
12.	0.00004936	0.840	96.228
13.	0.00004178	0.711	96.939
14.	0.00003603	0.613	97.552
15.	0.00003275	0.557	98.109
16.	0.00002081	0.354	98.463
17.	0.00001987	0.338	98.801
18.	0.00001817	0.309	99.111
19.	0.00001125	0.191	99.302
20.	0.00000927	0.158	99.460
21.	0.00000772	0.131	99.591
22.	0.00000591	0.101	99.692
23.	0.00000441	0.075	99.767
24.	0.00000377	0.064	99.831
25.	0.00000326	0.056	99.886
26.	0.00000166	0.028	99.915
27.	0.00000149	0.025	99.940
28.	0.00000097	0.017	99.956
29.	0.00000062	0.011	99.967
30.	0.00000045	0.008	99.975
31.	0.00000043	0.007	99.982
32.	0.00000034	0.006	99.988
33.	0.00000031	0.005	99.993
34.	0.00000026	0.004	99.998
35.	0.00000014	0.002	100.000

Total variance: 0.00587726

Variance of the eigenvalues: 0.0000000631057
Eigenvalue variance scaled by total variance: 0.00183
Eigenvalue variance scaled by total variance and number of variables: 0.19732
Note: throughout all calculations of eigenvalue variances, the dimensionality used was 107.
Please double-check because this dimensionality may not be appropriate for all situations.
Warning: There appear to be fewer PCs than dimensions in shape space.
This can have the following reasons, among others:
-- the degrees of freedom are less than the shape dimensionality (sample size, etc.).
-- there is complete integration in the data.
Interpret the results cautiously and check for reasons why this is the case.

Principal Component Coefficients

PC1	PC2	PC3	PC4	PC5	PC6
PC7	PC8	PC9	PC10	PC11	PC12
PC13	PC14	PC15	PC16	PC17	PC18
PC19	PC20	PC21	PC22	PC23	PC24

PC25 PC26 PC27 PC28 PC29 PC30
PC31 PC32 PC33 PC34 PC35
x1 -0.072273 0.205976 0.014688 -0.044931 -0.189409 -0.197825
-0.172331 0.195011 0.076351 -0.105937 0.240942 0.017682
-0.158934 -0.263028 -0.098927 -0.250376 -0.082544 0.016588
-0.003666 0.007789 -0.035617 -0.090931 -0.018927 0.013909
-0.097371 0.024844 -0.294537 -0.226623 -0.046853 0.083370
0.135670 -0.049870 -0.251558 0.156458 -0.090324
y1 0.041958 0.014613 -0.181436 0.002756 -0.032861 0.084172
-0.048539 -0.158689 0.216499 0.121379 -0.273444 0.061716
-0.026652 -0.125064 0.125084 -0.174808 0.052777 -0.026373
0.335982 -0.187929 -0.296029 -0.017827 -0.047068 -0.071473 -
0.155288 0.003451 -0.275943 0.018043 -0.024902 0.002835 -
0.037268 0.147560 -0.138424 0.068504 0.063254
z1 -0.105651 0.010403 0.008691 -0.133153 0.124435 -0.194042
0.101162 -0.143606 -0.053552 -0.143387 -0.151245 0.039734 -
0.128626 0.086434 -0.257380 0.131916 0.229727 0.231188 -
0.002017 0.098894 -0.010424 0.151472 -0.116518 0.108333
0.078010 0.102808 -0.014079 -0.201779 0.017607 -0.223877
0.023464 0.222537 -0.070658 -0.051479 0.151889
x2 -0.101193 0.173349 -0.040760 0.005821 -0.136481 -0.183449
-0.140045 0.181032 -0.016082 -0.155969 0.136422 -0.043700
-0.058972 -0.159056 -0.086046 -0.186591 -0.114889 0.052409
-0.020505 0.071786 -0.059850 0.168484 -0.010124 -0.093223
0.141761 -0.054034 0.004764 0.235231 0.053612 -0.237045 -
0.018794 -0.058385 0.220784 -0.336512 -0.001816
y2 0.031304 -0.000525 -0.202611 -0.049195 -0.033915 0.071142
-0.044433 -0.097268 0.175051 -0.099603 -0.127076 -0.026275
0.009403 -0.026163 0.008124 -0.130248 -0.046361 -0.021917
0.144249 -0.066435 -0.187251 0.171321 -0.024909 -0.052805
0.046702 -0.126785 -0.062693 0.172252 -0.025125 -0.065521
0.005079 0.077697 0.172948 -0.136595 -0.120403
z2 -0.095625 0.008451 0.049733 -0.137651 0.103210 -0.165696
0.053376 -0.146993 -0.034527 -0.099909 -0.082191 0.055419 -
0.083354 -0.012953 -0.074086 0.067715 0.148967 0.114662
0.047645 0.052030 0.039241 0.045528 -0.073895 0.047827
0.166390 0.078586 -0.081939 0.149570 -0.118478 0.052861
0.035558 0.023182 0.022689 0.098045 -0.079400
x3 -0.118568 0.146130 -0.080922 0.054610 -0.107766 -0.174120
-0.099201 0.104198 -0.116750 -0.083998 -0.014847 -0.037504
-0.004594 -0.044086 -0.007840 -0.073125 -0.076976 0.040536
-0.010569 0.054242 -0.004685 0.071127 0.050776 -0.111406
0.128541 0.079742 0.220170 -0.009744 0.040790 0.141420
0.017810 0.290845 0.168213 0.140277 0.118826
y3 0.024225 -0.005127 -0.218243 -0.078890 -0.052338 0.049711
-0.045593 -0.055152 0.107169 -0.190549 -0.064163 -0.055346
0.005683 0.068321 -0.047145 -0.017287 -0.101845 -0.037972
0.035623 0.015229 -0.039876 0.127305 0.105821 -0.074780
0.097590 0.053927 0.211957 -0.134323 0.139460 0.177332
0.021187 0.232132 0.112269 0.056755 0.105625
z3 -0.096117 0.006561 0.073023 -0.137107 0.103548 -0.152050
0.010134 -0.147501 -0.033974 -0.057765 -0.034493 0.080395 -
0.030626 -0.065811 0.008979 0.025697 0.075785 0.041747
0.086339 0.034733 0.091119 0.049760 -0.045474 0.036072
0.086347 0.000640 -0.091246 0.068020 -0.110168 0.060144
0.004169 -0.194765 0.027095 -0.011721 -0.099541
x4 -0.127855 0.117675 -0.102174 0.098073 -0.073713 -0.146092
-0.047062 0.045211 -0.174602 0.013479 -0.092876 0.021598
0.029788 0.039020 0.063341 -0.044314 -0.012418 0.066282 -
0.010459 -0.012726 -0.007538 -0.000587 -0.009363 -0.083407

0.023320 0.038185 0.091497 0.004336 -0.091241 0.102576 -
0.120209 -0.101127 -0.059403 0.234233 0.038495
y4 0.024498 -0.011981 -0.206963 -0.088340 -0.065092 0.045243
-0.017156 -0.026946 0.037218 -0.175615 0.001567 -0.009881
-0.005003 0.123399 -0.045933 -0.005805 -0.069838 -0.011404
-0.063043 -0.013690 0.035569 0.025472 0.048471 -0.017876
0.028372 0.037872 0.075207 -0.041132 -0.023270 0.028301 -
0.161931 -0.336823 -0.132477 0.187656 0.062300
z4 -0.100775 0.003282 0.092051 -0.133278 0.100740 -0.130516
-0.023454 -0.142559 -0.017764 -0.018042 0.012226 0.088981
0.008107 -0.103220 0.075545 -0.001130 0.008086 -0.028729
0.096394 0.028582 0.089673 0.003491 0.027345 0.000411 -
0.008958 -0.033753 -0.034891 -0.016440 0.000535 0.086709 -
0.022700 -0.114795 0.037274 -0.068536 -0.104411
x5 -0.133154 0.091123 -0.120607 0.123347 -0.036664 -0.111801
-0.014395 -0.007253 -0.193902 0.070195 -0.150237 0.047058
0.066266 0.082706 0.094685 -0.004634 0.025039 0.092936
0.025878 -0.014626 0.017949 -0.007128 -0.035927 -0.044452 -
0.023353 -0.073317 0.053793 -0.087785 -0.119042 0.073364 -
0.079972 -0.069043 -0.139609 -0.018355 0.060401
y5 0.028036 -0.017533 -0.183346 -0.102554 -0.071613 0.049275
-0.000795 -0.026902 -0.014023 -0.145520 0.036373 0.006718
0.011186 0.116292 -0.041947 0.001256 -0.047840 0.031906 -
0.081178 0.019175 0.107370 -0.014497 0.012413 0.040393 -
0.003863 -0.110115 0.006262 -0.157311 -0.079023 0.045858 -
0.077713 -0.209364 -0.225920 -0.121703 0.037558
z5 -0.106991 -0.004393 0.101535 -0.123311 0.095574 -0.100667
-0.049072 -0.134601 -0.003250 0.007295 0.042175 0.082564
0.044504 -0.111668 0.113481 -0.024694 -0.045822 -0.079003
0.079245 0.005061 0.083889 -0.048592 0.061902 -0.005699 -
0.057486 -0.047297 0.008906 -0.033641 0.100563 0.074046 -
0.005077 0.022872 0.031275 -0.020112 0.002626
x6 -0.131911 0.063682 -0.130013 0.134723 -0.000712 -0.067991
0.005045 -0.029537 -0.167916 0.115600 -0.160178 0.056438
0.069026 0.106214 0.098774 0.026261 0.031454 0.099085
0.065598 -0.012851 0.031494 -0.021259 -0.033234 -0.025303 -
0.047672 -0.018794 0.008258 0.119482 0.037337 0.067341 -
0.008575 0.010953 -0.058930 -0.158524 -0.171848
y6 0.035969 -0.024242 -0.144808 -0.112537 -0.077427 0.060909
0.012346 -0.013053 -0.033933 -0.088316 0.075671 0.002674
0.005981 0.083872 -0.022020 -0.001009 -0.034542 0.051922 -
0.072653 0.036235 0.139970 -0.094349 -0.007502 0.076083 -
0.015602 -0.025232 0.046864 0.192417 0.065152 0.046951
0.023668 -0.035462 -0.043287 -0.116019 -0.198308
z6 -0.115350 -0.012759 0.103985 -0.104010 0.087157 -0.063902
-0.071721 -0.124380 0.014800 0.015373 0.060861 0.064843
0.081345 -0.102787 0.132533 -0.054486 -0.077607 -0.114839
0.030509 -0.020845 0.041157 -0.073452 0.084395 -0.015119 -
0.064826 -0.082262 0.035223 0.008170 0.085139 0.018790 -
0.025541 -0.008254 -0.011989 -0.004916 0.130131
x7 -0.125617 0.044623 -0.134070 0.137661 0.034128 -0.028758
0.006472 -0.052078 -0.135858 0.130795 -0.131032 0.083777
0.070474 0.102677 0.074801 0.048259 0.033316 0.074373
0.024234 -0.024566 0.069248 -0.076549 0.022119 -0.023550 -
0.084820 -0.031887 -0.033749 0.046613 0.055869 -0.054778
0.040587 0.023373 0.031921 -0.004494 -0.054010
y7 0.043902 -0.020534 -0.096531 -0.108992 -0.080828 0.051854
0.008647 -0.015808 -0.087055 -0.016453 0.114403 0.035987
0.016023 0.027124 -0.019253 0.013376 -0.020829 0.033382 -
0.124650 0.014086 0.163927 -0.155425 0.047033 0.062791 -

0.078631 -0.037241 -0.002596 0.118905 -0.001841 -0.016965
0.109967 0.104808 0.131242 0.155777 -0.061572
z7 -0.127381 -0.023127 0.096849 -0.074587 0.077254 -0.025743
-0.086740 -0.108087 0.024873 0.017024 0.064439 0.037157
0.109295 -0.061995 0.116847 -0.067808 -0.095968 -0.133691 -
0.003972 -0.035465 -0.004858 -0.071050 0.073083 0.006924 -
0.064292 -0.098464 0.049228 0.017949 0.100885 0.036485 -
0.023894 0.045349 0.021671 0.020964 0.064456
x8 -0.114773 0.028171 -0.128533 0.129966 0.066291 0.020680
-0.005156 -0.057276 -0.059591 0.108279 -0.079000 0.086401
0.052704 0.103825 0.036443 0.031311 0.008424 0.046694
0.004062 0.000526 0.084498 -0.088614 -0.014085 0.046739 -
0.039531 -0.051633 -0.106737 -0.027293 0.074173 -0.049386
0.152966 -0.034648 0.023492 -0.024980 0.061103
y8 0.051040 -0.014400 -0.036471 -0.092831 -0.081840 0.046861
-0.003762 -0.007946 -0.117946 0.041714 0.119843 0.035092
0.016151 -0.010353 0.005854 0.002414 -0.047631 0.028334 -
0.088609 0.046009 0.110618 -0.127401 -0.029102 0.054404 -
0.030931 -0.018327 -0.057609 -0.010924 -0.033348 -0.006008
0.167118 0.067429 0.058627 0.038535 0.062694
z8 -0.141798 -0.035747 0.080368 -0.037494 0.065559 0.013148
-0.093739 -0.086408 0.032692 0.002224 0.053417 -0.001205
0.130115 0.002491 0.063584 -0.074803 -0.101205 -0.128622 -
0.054788 -0.050860 -0.059896 -0.050499 0.057239 -0.029036 -
0.059424 -0.063161 0.046909 0.028996 0.061799 -0.011675 -
0.041776 0.069179 0.009432 0.022743 -0.009097
x9 -0.101225 0.012483 -0.119742 0.111828 0.091435 0.069637
-0.023883 -0.055463 0.037470 0.078548 0.006008 0.077904
0.022831 0.091599 -0.017995 0.047537 -0.037224 0.005055 -
0.002106 0.019034 0.108103 -0.066496 -0.015254 0.115472
0.028888 -0.049230 -0.149796 -0.140768 0.123785 -0.134477
0.072151 -0.098908 0.227973 0.100820 0.041921
y9 0.054283 -0.006394 0.029364 -0.063051 -0.088279 0.032389
-0.017169 -0.007059 -0.158630 0.141791 0.103797 0.031054
0.020698 -0.069152 0.043454 0.041155 -0.074575 0.018567 -
0.019208 0.065867 0.018544 -0.060915 -0.046068 -0.017354
0.043349 0.030851 -0.020731 -0.161410 -0.001919 -0.167413
0.011746 -0.033212 0.161166 0.030210 0.029017
z9 -0.158248 -0.048290 0.055509 0.007173 0.055081 0.052899
-0.093668 -0.059790 0.040678 -0.028120 0.030267 -0.048453
0.145238 0.094939 -0.028434 -0.081416 -0.088414 -0.105555 -
0.124324 -0.056891 -0.128411 -0.011995 0.006704 -0.063352 -
0.044624 0.002124 0.036598 0.018681 -0.030940 -0.076577
0.004761 0.022026 -0.051133 0.050506 -0.059766
x10 -0.090128 -0.006971 -0.100962 0.093334 0.119521
0.107604 -0.031757 -0.042664 0.151469 0.048948 0.095926
0.064620 -0.023935 0.081303 -0.079073 0.090716 -0.077571 -
0.031049 -0.075248 -0.014607 0.063331 -0.016059 0.018578
0.115718 0.050744 -0.024700 -0.166431 0.016817 0.043886 -
0.118185 -0.006087 0.088643 0.114561 0.057819 -0.116190
y10 0.052589 0.001351 0.108086 -0.014519 -0.086914
0.002585 -0.011141 -0.008375 -0.201874 0.288045 0.046552
0.042260 0.025478 -0.152815 0.116491 0.094433 -0.081106
0.037703 0.010445 0.022798 -0.159010 -0.035017 -0.018705 -
0.171964 0.088272 0.111110 0.085251 -0.037246 -0.054939 -
0.317399 -0.108547 0.026513 0.008001 -0.019774 0.077228
z10 -0.176998 -0.060252 0.024709 0.054504 0.040608
0.097574 -0.089489 -0.028512 0.054168 -0.072326 0.004177 -
0.110010 0.156958 0.212696 -0.148137 -0.083382 -0.055591 -
0.059343 -0.224226 -0.048322 -0.211781 0.028440 -0.084269 -

0.087342	0.011310	0.092813	0.035170	-0.064262	-0.091154	-
0.129601	-0.038261	-0.000669	-0.077068	0.122778	0.007565	
x11	-0.056258	-0.011656	-0.060024	0.060629	0.119780	
0.109452	-0.039039	-0.053993	0.157458	0.093007	0.124733	
0.045979	-0.058524	0.020748	-0.051098	0.110253	-0.097403	-
0.074752	-0.066823	0.020288	-0.018587	0.010536	-0.008985	
0.021413	0.162436	0.075810	-0.080020	-0.049022	-0.050505	
0.034375	-0.060197	-0.034484	-0.163394	-0.161980	-0.053851	
y11	0.025284	0.000525	0.156726	0.042847	-0.052649	
0.015880	-0.011441	0.004520	-0.123548	0.246980	0.064339	-
0.015177	0.021630	-0.056614	0.024074	0.046866	-0.064732	
0.051272	0.003645	-0.007432	-0.131239	0.032780	-0.082420	-
0.096795	0.093494	0.105644	0.031330	-0.070351	-0.049669	-
0.032316	-0.041993	-0.020713	-0.125242	-0.077716	-0.083343	
z11	-0.168998	-0.067788	-0.006948	0.005317	-0.007958	
0.158536	-0.050356	0.026042	-0.013727	-0.058355	0.016053	-
0.119506	0.070031	0.119593	-0.073808	-0.022182	-0.035051	-
0.002105	-0.072263	-0.043346	-0.125548	0.016950	-0.056426	-
0.025648	-0.018268	0.082798	0.009609	-0.022087	-0.093256	-
0.056762	0.093036	-0.054887	-0.050139	-0.060958	-0.009516	
x12	-0.035585	-0.019631	-0.032736	0.028638	0.115465	
0.089655	-0.030605	-0.054753	0.147961	0.101291	0.136523	
0.059003	-0.059609	-0.047757	-0.026581	0.124255	-0.054933	-
0.079372	-0.015356	0.018003	0.014856	0.053508	-0.004874	-
0.004394	0.128426	0.165172	0.077326	-0.000993	0.006969	
0.154660	0.033374	0.073444	-0.059494	0.000490	-0.003040	
y12	-0.005852	0.001774	0.185690	0.086776	-0.028405	
0.011394	-0.000621	0.023655	-0.063049	0.188568	0.068821	-
0.031326	0.039285	-0.003192	-0.037390	0.020119	-0.006528	
0.071299	0.019468	-0.044165	-0.042878	0.102964	-0.079160	-
0.011035	0.005885	0.105879	0.031995	-0.008770	0.037372	
0.199274	0.093656	0.062572	-0.044811	-0.019651	-0.061312	
z12	-0.150841	-0.067996	-0.028460	-0.036120	-0.048738	
0.197674	-0.013226	0.063930	-0.061703	-0.044585	0.029043	-
0.133681	-0.006795	0.052327	-0.022510	0.032145	-0.021174	
0.038717	0.043806	-0.043266	-0.048258	0.016360	-0.045133	-
0.007689	-0.007826	0.071809	0.018414	-0.022454	-0.075441	
0.031107	0.089432	-0.050632	-0.014102	-0.119236	-0.033250	
x13	-0.021701	-0.026400	-0.014538	0.008102	0.118116	
0.070224	-0.002815	-0.055620	0.128338	0.096212	0.140640	
0.023428	-0.070297	-0.058229	-0.043270	0.096456	-0.043412	-
0.047099	-0.017779	0.060293	0.042174	0.075758	0.005136	-
0.040892	0.120889	0.084474	0.004539	0.119183	-0.117586	
0.113897	-0.087230	0.097817	-0.034351	-0.047962	0.144955	
y13	-0.037170	0.006515	0.194940	0.124354	-0.000885	
0.011552	0.021100	0.040787	-0.015407	0.129298	0.050387	-
0.055974	0.024128	0.054155	-0.110279	-0.033197	0.021397	
0.097258	0.003258	-0.023244	0.006070	0.119094	-0.034665	
0.017836	-0.020582	0.028636	-0.072312	0.076053	0.006796	
0.208555	0.024345	0.015477	-0.029934	-0.057345	0.086318	
z13	-0.126793	-0.063805	-0.043167	-0.070815	-0.081045	
0.220195	0.019093	0.091838	-0.090344	-0.029720	0.029468	-
0.124884	-0.067506	-0.014552	0.014043	0.069558	-0.002142	
0.062813	0.117672	-0.045122	0.008030	0.015560	-0.033698	
0.011024	-0.009434	0.037164	0.021020	-0.018235	0.008474	
0.052826	0.009604	-0.038533	0.013250	-0.053958	-0.003691	
x14	-0.012994	-0.039279	-0.007608	-0.005029	0.108243	
0.061048	0.019128	-0.029662	0.111261	0.077752	0.132913	
0.025642	-0.058348	-0.099475	-0.026094	0.068401	-0.007787	-
0.003277	0.015860	0.058490	0.053735	0.105416	0.046798	-

0.054063	0.070244	-0.031995	0.046208	0.131512	-0.067489	
0.070658	-0.087121	-0.104752	0.036735	0.115575	0.073246	
y14	-0.065276	0.007830	0.188383	0.152348	0.013402	
0.016329	0.036425	0.070185	0.025879	0.070411	0.021807	-
0.046449	0.011052	0.066203	-0.130881	-0.069370	0.048207	
0.108649	0.011984	-0.026658	0.026392	0.124773	0.043727	
0.044852	-0.037897	-0.069770	-0.057824	0.086453	0.070218	
0.147897	-0.012634	-0.083067	0.057654	0.048047	0.051832	
z14	-0.096628	-0.056126	-0.052066	-0.095550	-0.105766	
0.221179	0.047106	0.103349	-0.101544	-0.010870	0.020927	-
0.102045	-0.108093	-0.063200	0.033658	0.079454	0.008139	
0.075957	0.138007	-0.023069	0.031961	0.016396	-0.013904	
0.038819	-0.026984	0.004347	0.009375	-0.009014	0.026496	
0.011599	-0.037005	0.041223	0.046211	0.003093	0.002869	
x15	-0.007683	-0.049419	-0.007269	-0.021381	0.099921	
0.045655	0.046088	-0.007013	0.100859	0.035195	0.115294	
0.036484	-0.050347	-0.127085	0.013641	0.016852	0.002266	
0.043638	0.034908	0.014210	0.009175	0.092634	0.005211	
0.024366	-0.055514	-0.205870	0.141415	0.044810	-0.113847	-
0.015943	0.210266	0.000179	-0.020500	-0.005230	-0.017647	
y15	-0.088348	0.012112	0.171884	0.160665	0.022505	
0.018345	0.051855	0.094401	0.064965	0.010774	-0.015068	-
0.023166	-0.022799	0.064291	-0.112163	-0.096837	0.044912	
0.104072	0.015184	-0.043245	0.005664	0.095449	0.057594	
0.090372	-0.078333	-0.183615	-0.009675	0.031545	0.056458	
0.030248	0.062441	-0.009047	0.025224	0.005490	-0.051542	
z15	-0.059465	-0.045026	-0.054801	-0.112357	-0.123152	
0.203710	0.068212	0.102534	-0.096551	0.016000	-0.000028	-
0.064872	-0.123775	-0.090251	0.035444	0.066083	0.020436	
0.075274	0.117762	0.011391	0.039725	0.018530	0.017159	
0.033056	-0.025656	-0.006777	-0.035559	0.005215	0.055676	
0.000069	-0.086754	0.049104	0.027128	0.077300	-0.006746	
x16	-0.011535	-0.053805	-0.013882	-0.034723	0.095783	
0.028578	0.082420	0.001679	0.071746	-0.021890	0.105643	
0.003332	-0.029981	-0.157074	0.031185	-0.017656	0.051867	
0.067872	0.028606	0.005867	0.003157	0.037033	-0.086129	
0.076741	-0.180515	-0.302306	0.214230	-0.028288	-0.145161	-
0.135868	-0.058640	-0.034827	-0.018861	-0.082926	0.228586	
y16	-0.107873	0.021775	0.145411	0.151836	0.027441	
0.016768	0.073694	0.108140	0.085834	-0.037520	-0.043672	-
0.011802	-0.062449	0.044729	-0.088255	-0.101159	0.052299	
0.060333	0.018946	-0.022716	0.013179	0.041321	0.064824	
0.092161	-0.077111	-0.214506	0.025131	0.001187	0.051450	-
0.070444	-0.100495	0.028522	0.027444	0.041521	-0.003173	
z16	-0.013864	-0.030036	-0.050034	-0.123279	-0.133581	
0.165486	0.085470	0.086960	-0.067509	0.059113	-0.031997	-
0.010861	-0.114638	-0.094025	0.020907	0.032804	0.020377	
0.068181	0.064227	0.030213	0.027196	0.020066	0.052498	
0.016917	-0.012107	-0.000120	-0.083866	0.041041	0.094207	
0.005733	-0.070432	0.049818	0.003693	0.089658	-0.049728	
x17	-0.010984	-0.058789	-0.023358	-0.058450	0.094153	
0.003907	0.119459	0.008927	0.054181	-0.027161	0.082431	-
0.030344	-0.026074	-0.169593	-0.011334	-0.051637	0.090823	
0.069999	-0.018249	-0.003114	-0.026410	-0.049191	-0.104870	-
0.016301	-0.247262	0.110867	0.087367	-0.221621	-0.059174	-
0.062058	0.093016	-0.009652	0.011813	0.023961	-0.136458	
y17	-0.117429	0.033216	0.111423	0.121074	0.030006	
0.012779	0.100108	0.114342	0.100141	-0.053884	-0.076266	
0.005486	-0.106617	0.032003	-0.060059	-0.089979	0.046090	-
0.002578	0.013989	-0.003073	0.046467	-0.043983	0.068728	

0.019165	-0.035640	-0.036958	-0.004347	-0.047480	0.019462	-
0.103464	-0.056445	0.003049	0.009258	0.096979	0.012262	
z17	0.040500	-0.010253	-0.038179	-0.125382	-0.138163	
0.108518	0.100754	0.050346	-0.022831	0.105503	-0.062433	
0.050584	-0.077163	-0.064877	-0.006733	-0.019665	0.019259	
0.044739	-0.031776	0.028694	0.003630	0.027494	0.041202	
0.009960	0.024250	-0.088917	-0.051454	0.094419	0.036602	
0.033109	-0.016668	0.004963	-0.015570	0.036766	0.113050	
x18	-0.005759	-0.058564	-0.023663	-0.065744	0.080803	-
0.018174	0.157969	-0.007783	0.030210	-0.019297	0.053673	-
0.049108	0.020913	-0.133776	-0.044802	-0.068798	0.124746	
0.060559	-0.053187	-0.036478	-0.086057	-0.075990	-0.036370	-
0.041125	-0.157463	0.189162	-0.003288	-0.203752	0.054216	-
0.008043	-0.346713	-0.010833	0.204072	0.054219	-0.124445	
y18	-0.118776	0.048721	0.071837	0.075775	0.023173	
0.007690	0.129643	0.108391	0.107265	-0.056017	-0.112248	
0.036920	-0.139017	0.019610	0.011896	-0.051201	0.032234	-
0.100506	0.033178	0.016273	0.074024	-0.145544	0.089246	-
0.033745	0.073434	0.048087	0.036689	-0.034172	-0.008014	-
0.051612	-0.110568	0.022851	0.076734	0.068341	0.012322	
z18	0.102552	0.013160	-0.019950	-0.119896	-0.138078	
0.028067	0.112922	0.000981	0.039971	0.155786	-0.098538	
0.119515	-0.027843	-0.017588	-0.054267	-0.088086	0.015944	
0.021103	-0.156562	0.029853	-0.020487	0.021428	0.000485	-
0.023161	0.037294	-0.105988	-0.006685	0.085888	-0.048415	
0.084139	0.169321	-0.043839	-0.099105	-0.076584	0.095462	
x19	0.004242	-0.052490	-0.004381	-0.088383	0.075466	-
0.059710	0.185267	-0.027722	0.023383	0.008313	0.006514	-
0.066671	0.043487	-0.027818	-0.082637	-0.111609	0.115920	
0.003931	-0.083507	-0.061646	-0.083486	-0.150759	-0.032317	-
0.051370	-0.177679	0.255132	0.132194	0.125195	0.224333	
0.102415	0.057112	0.051276	0.021183	-0.011747	0.035201	
y19	-0.114335	0.068078	0.022319	0.005553	0.012853	-
0.004585	0.157863	0.093430	0.114823	-0.043342	-0.167539	
0.084344	-0.182168	-0.007288	0.102235	-0.022373	-0.018603	-
0.213233	0.035152	0.044964	0.072491	-0.264177	0.093003	-
0.105710	0.216908	0.119898	0.109185	-0.092589	-0.138404	-
0.010804	0.089165	-0.080228	0.007335	-0.151778	-0.132740	
z19	0.166885	0.037086	-0.000449	-0.101858	-0.127530	-
0.062062	0.125120	-0.054804	0.105925	0.195074	-0.129760	
0.183543	0.039170	0.030214	-0.119746	-0.157891	0.034977	
0.006115	-0.324327	0.043677	-0.056505	0.040456	-0.022246	-
0.103931	0.094687	-0.154011	-0.028976	-0.045981	0.016058	
0.043883	-0.026176	-0.003465	0.025434	0.076318	-0.107677	
x20	0.002408	-0.060920	0.003921	-0.113331	0.030632	-
0.056622	0.155311	-0.019312	-0.033090	-0.009641	-0.000242	-
0.121038	0.027418	0.043316	-0.055331	-0.172940	0.049890	-
0.022488	-0.010636	-0.040457	-0.028656	-0.018114	-0.097266	-
0.046007	0.046897	0.000488	-0.122006	0.085156	0.203439	-
0.106081	0.005064	-0.096740	-0.135953	-0.113431	0.030340	
y20	-0.080679	0.048050	-0.010662	-0.026280	-0.016486	-
0.058470	0.137617	0.095483	0.094327	-0.059170	-0.081939	
0.062886	-0.043392	0.033909	0.103014	0.026761	-0.037969	-
0.162302	0.047790	0.039902	0.064194	-0.118939	-0.041234	-
0.054323	0.078036	0.088166	0.019462	-0.008643	-0.021381	-
0.012160	0.067777	0.076068	-0.114804	-0.139441	0.109147	
z20	0.184512	0.018365	0.013194	-0.045631	-0.075679	-
0.081321	0.067828	-0.022698	0.092722	0.138274	-0.047135	
0.118353	0.076055	0.060120	-0.107218	-0.096453	0.008997	
0.018801	-0.098765	0.011923	-0.014846	-0.019341	0.053491	-

0.034844	0.051355	-0.021018	0.079175	-0.026492	-0.060052	
0.028374	0.000110	-0.017731	0.104649	0.012500	-0.050465	
x21	0.000771	-0.064321	0.029786	-0.099729	-0.010889	-
0.065814	0.124723	-0.008941	-0.078861	-0.001299	0.020988	-
0.136137	0.035725	0.040766	-0.007161	-0.085304	0.100761	-
0.062109	0.012623	0.011529	-0.085763	-0.018998	0.034585	
0.051401	0.117245	0.038795	0.040610	0.146514	0.030918	
0.084470	0.007413	0.015346	-0.195358	-0.074145	0.131005	
y21	-0.050227	0.034050	-0.034839	-0.047100	-0.034752	-
0.106088	0.114717	0.082607	0.069563	-0.056632	-0.007684	
0.054038	0.080694	0.026150	0.112793	0.091498	-0.027059	-
0.104198	0.033024	0.033497	0.018567	-0.002812	-0.056677	
0.002938	0.004239	0.027459	-0.028147	0.041775	-0.027811	
0.040532	0.013400	0.049507	-0.046170	-0.036638	0.087856	
z21	0.196113	0.000211	0.021900	-0.003700	-0.031431	-
0.085688	0.020318	0.004953	0.077404	0.075407	0.005443	
0.058577	0.087041	0.094750	-0.104089	-0.074134	-0.041420	
0.033758	0.064607	-0.020033	0.051644	-0.043044	0.052444	-
0.020245	0.041022	0.003453	0.037937	-0.084093	-0.017707	-
0.057414	-0.045445	-0.008212	0.098663	0.010085	0.002897	
x22	-0.000009	-0.068260	0.058131	-0.097872	-0.043009	-
0.069854	0.083869	-0.013925	-0.106034	-0.000496	0.027092	-
0.172216	0.054375	0.053098	0.011015	-0.020559	0.140271	-
0.112731	0.035765	-0.040647	-0.131371	-0.019441	0.132776	-
0.017655	0.076016	-0.120279	0.070829	0.117379	0.002065	-
0.068517	0.152040	-0.109157	-0.112339	0.090611	-0.067264	
y22	-0.021715	0.021397	-0.050656	-0.058124	-0.043425	-
0.135336	0.083751	0.059852	0.044374	-0.052700	0.042851	
0.040814	0.159943	0.017476	0.101752	0.141722	-0.009435	-
0.062089	0.013142	0.004968	-0.011433	0.069902	-0.046066	
0.030840	-0.055654	-0.004084	-0.035704	0.091859	-0.049275	-
0.046907	-0.037575	-0.038442	0.020598	0.111811	0.001499	
z22	0.201515	-0.013730	0.029474	0.038046	0.004830	-
0.081646	-0.014364	0.031495	0.061134	0.017728	0.037803	
0.017983	0.078207	0.111736	-0.083799	-0.060802	-0.083620	
0.043655	0.177657	-0.012051	0.092923	-0.047451	0.020200	
0.028276	0.040959	0.054673	0.034290	-0.128542	0.001970	
0.002729	-0.087115	0.057220	0.036476	-0.084520	0.066462	
x23	0.002362	-0.074084	0.092589	-0.100702	-0.070320	-
0.047175	0.041386	-0.002273	-0.085221	-0.018964	-0.002830	-
0.177351	0.056104	0.093244	0.031623	0.014957	0.074234	-
0.092665	0.072479	-0.052278	-0.022831	-0.043860	0.170037	-
0.011823	0.103288	-0.069590	-0.011022	-0.035321	-0.017908	-
0.000058	0.138777	0.132894	0.087573	0.108450	-0.223805	
y23	0.005203	0.007156	-0.056665	-0.061168	-0.039051	-
0.134055	0.037723	0.023879	0.020261	-0.062942	0.063231	
0.016880	0.187415	0.025514	0.084563	0.137549	-0.023134	-
0.018875	0.000783	0.003485	-0.020535	0.112785	-0.080571	
0.046371	-0.085836	-0.016959	-0.050384	0.020152	-0.023157	-
0.040360	-0.031258	-0.019764	0.016968	0.105934	-0.021070	
z23	0.200059	-0.021131	0.033501	0.078114	0.033080	-
0.081985	-0.032250	0.052533	0.033937	-0.026624	0.059214	-
0.010397	0.065606	0.094972	-0.055389	-0.034524	-0.071741	
0.033311	0.225593	-0.012661	0.078388	-0.026965	0.012298	
0.045483	0.022982	0.043842	0.026491	-0.088860	-0.006340	-
0.066303	-0.068393	-0.034014	-0.024357	-0.069944	0.071433	
x24	-0.000182	-0.074851	0.134188	-0.081598	-0.093337	-
0.018773	-0.016154	-0.003908	-0.080860	-0.029577	-0.023707	-
0.129955	0.044112	0.081345	0.024705	0.063492	0.026955	-
0.100719	0.023527	-0.016435	-0.012999	-0.022627	0.180872	-

0.012353	0.102449	-0.103577	-0.135394	-0.016898	-0.122694	-
0.132519	-0.224227	0.080520	0.016874	0.055453	0.146745	
y24	0.029158	-0.001327	-0.051792	-0.050415	-0.028099	-
0.118105	-0.018942	-0.019811	-0.011071	-0.065576	0.071305	
0.001436	0.174420	0.004975	0.068415	0.126105	-0.015422	
0.002065	-0.025115	0.004615	-0.035284	0.118432	-0.070414	
0.030088	-0.088097	0.013364	-0.076018	0.040979	-0.014376	-
0.048402	-0.035751	-0.025931	0.029988	0.071816	-0.011754	
z24	0.194623	-0.026370	0.036905	0.107573	0.053603	-
0.073084	-0.036041	0.068874	0.013847	-0.064688	0.051157	-
0.041006	0.040753	0.083727	-0.016469	-0.017485	-0.064418	
0.010682	0.241520	-0.011166	0.072399	-0.005773	-0.001433	
0.052306	0.002674	0.072797	0.015847	-0.060255	0.050908	
0.021708	0.112207	0.039818	-0.079021	-0.088194	-0.075411	
x25	-0.003205	-0.087472	0.173480	-0.063507	-0.104457	
0.021644	-0.063585	-0.001373	-0.042839	-0.054265	-0.071421	-
0.043055	0.039025	0.093070	0.012895	0.079315	-0.031384	-
0.058370	-0.001030	0.036282	0.027013	0.004417	0.112165	
0.021590	0.068129	-0.145464	-0.198962	-0.187907	0.020521	
0.017225	0.006035	0.092582	0.027399	-0.127996	0.018535	
y25	0.051915	-0.011412	-0.038468	-0.031140	-0.003189	-
0.075242	-0.086320	-0.076946	-0.043566	-0.077881	0.052171	-
0.014870	0.112202	0.004906	0.038782	0.072384	-0.028395	
0.026332	-0.033629	0.032374	-0.031761	0.103261	-0.097794	
0.023826	-0.058071	0.027549	-0.085751	-0.065240	0.100855	
0.029053	0.075871	0.040071	0.014017	-0.111611	-0.082089	
z25	0.182530	-0.021055	0.038163	0.136953	0.061463	-
0.071171	-0.023511	0.080924	-0.013940	-0.087499	0.034565	-
0.059104	0.013988	0.022935	0.038122	0.034345	-0.015146	-
0.029839	0.177077	-0.033578	0.035515	-0.005342	0.048126	
0.030791	-0.040661	0.076815	0.035698	0.123791	-0.070305	-
0.060921	-0.004113	-0.014088	-0.081200	0.119266	-0.046432	
x26	-0.007221	-0.091363	0.207141	-0.056461	-0.128634	
0.050410	-0.092078	0.000130	-0.012001	-0.065093	-0.115144	
0.046117	-0.003760	0.074731	0.014864	0.063035	-0.087532	-
0.011086	-0.009531	0.026488	0.025146	0.015599	0.094563	
0.009720	-0.024563	-0.065948	-0.121702	-0.126397	-0.076363	
0.176138	-0.092619	0.076687	-0.038763	-0.111767	-0.079338	
y26	0.071792	-0.015745	-0.019553	0.000841	0.028699	-
0.028369	-0.151582	-0.146298	-0.090474	-0.074827	0.023134	-
0.027765	0.010011	-0.025050	0.004761	0.009495	-0.013750	
0.049646	-0.041902	0.016987	-0.023145	0.052522	-0.040406	
0.006831	-0.042694	0.029621	-0.066916	-0.065355	0.069431	
0.086261	0.043137	0.045505	0.001086	-0.104886	-0.027228	
z26	0.164400	-0.014293	0.041797	0.163468	0.067391	-
0.051559	-0.006927	0.086227	-0.037164	-0.122779	-0.004990	-
0.065952	-0.020100	-0.022842	0.089960	0.069668	0.033739	-
0.080073	0.042651	-0.009480	0.001710	0.011110	0.029504	
0.026712	-0.048810	0.032986	0.015564	0.126999	-0.038461	-
0.096024	0.031471	-0.062211	-0.048409	0.115557	0.012626	
x27	-0.011220	-0.097007	0.236765	-0.047990	-0.155929	
0.097002	-0.124072	0.010100	0.044162	-0.102742	-0.165236	
0.176676	0.013256	0.023068	0.012782	0.034381	-0.068694	
0.023216	0.024170	-0.012179	0.039070	0.048132	-0.073983	
0.032448	-0.038978	0.057564	-0.003695	-0.002351	-0.074015	
0.127221	-0.018385	-0.147634	0.149501	0.077914	0.029960	
y27	0.089701	-0.017381	0.007066	0.044331	0.068315	
0.034140	-0.224668	-0.226856	-0.141663	-0.073857	-0.025991	-
0.033867	-0.120054	-0.069540	-0.037996	-0.067239	0.020865	
0.062735	-0.036280	-0.007029	-0.004943	-0.031890	0.030064	-

0.003414	0.026727	-0.000373	0.002105	-0.028340	0.015757	
0.069593	0.036427	0.021567	0.026549	-0.010151	0.011987	
z27	0.140692	-0.004830	0.043703	0.185304	0.065698	-
0.024869	0.019356	0.084314	-0.063438	-0.143488	-0.066506	-
0.066232	-0.086898	-0.075729	0.156890	0.107008	0.058391	-
0.134432	-0.154112	0.022898	-0.047650	0.015810	-0.020267	-
0.004511	-0.078508	0.005608	-0.021093	0.096901	-0.000326	
0.021850	0.024275	-0.006407	-0.029587	0.029588	-0.055681	
x28	-0.015333	-0.101826	0.259573	-0.046626	-0.189132	
0.121320	-0.147859	0.038364	0.128368	-0.121346	-0.230090	
0.313232	-0.000846	0.013533	0.038999	0.000196	-0.134049	
0.045407	0.029332	0.025052	0.069049	0.074349	-0.313493	
0.045399	-0.097215	0.210067	0.210818	0.149743	0.111448	-
0.199376	0.109123	-0.068528	-0.040090	0.074449	-0.015696	
y28	0.105808	-0.016807	0.037303	0.095533	0.114277	
0.107913	-0.299845	-0.314210	-0.192270	-0.065586	-0.092435	-
0.034932	-0.288951	-0.111956	-0.082773	-0.163057	0.056680	
0.060743	-0.030855	-0.028149	0.016114	-0.122538	0.092416	-
0.012995	0.110249	-0.029642	0.127968	0.074456	-0.047933	-
0.094968	-0.037009	-0.092095	-0.050171	0.089880	0.015683	
z28	0.112504	0.006415	0.047654	0.202528	0.058692	
0.032327	0.048456	0.063028	-0.089803	-0.185815	-0.148746	-
0.061016	-0.162818	-0.122729	0.231155	0.081700	0.086938	-
0.189570	-0.391704	0.077485	-0.100548	0.049669	-0.166763	-
0.028751	-0.039648	-0.056084	-0.162272	-0.087037	0.072645	
0.094170	0.001402	0.029338	0.127282	-0.119408	0.045548	
x29	0.147205	0.435608	0.068348	-0.181204	0.039644	
0.012009	0.011641	-0.051662	-0.014287	0.095855	-0.145222	-
0.173431	-0.136941	0.049434	-0.004242	0.141507	-0.146154	-
0.111437	-0.036178	-0.359539	-0.001921	0.056337	-0.029498	
0.230172	-0.104950	-0.080491	0.301868	-0.131689	0.188963	
0.010481	0.000656	-0.088080	-0.002987	-0.090582	-0.050249	
y29	0.000211	-0.012537	-0.071542	-0.061855	0.157685	
0.030288	-0.171692	0.256628	-0.069047	0.077968	-0.207976	
0.055887	0.225013	-0.272749	-0.331321	0.174125	0.138663	-
0.190162	0.019629	0.002689	-0.019296	0.023877	0.219150	
0.017675	0.025683	0.065091	-0.074105	-0.048243	0.196515	-
0.059650	0.034915	-0.158525	0.012939	-0.129553	0.018351	
z29	-0.037245	0.036794	-0.066946	0.113170	-0.250082	-
0.108340	-0.119966	0.011945	0.083014	-0.009827	0.221396	
0.282610	-0.146807	0.014310	0.061795	0.208257	0.227360	
0.026075	-0.154514	-0.400840	-0.110000	-0.023439	0.143126	
0.280297	0.040045	0.036251	0.030870	0.048780	0.008263	-
0.071764	-0.141001	0.191499	-0.139586	-0.062842	-0.009774	
x30	0.152071	0.360486	0.072176	-0.132311	0.084754	
0.074133	0.026875	-0.057265	0.021294	0.020971	-0.042820	-
0.128501	-0.098959	0.155450	-0.026980	0.090493	-0.081424	-
0.008500	0.019654	0.082106	-0.096741	0.042198	0.064202	-
0.018690	-0.176598	0.139947	-0.195654	0.046154	-0.185002	
0.047761	-0.040040	-0.094472	0.107899	0.066260	0.041056	
y30	0.007000	-0.030756	-0.046022	-0.081364	0.146545	
0.007769	-0.180568	0.217175	-0.026220	0.075053	-0.119985	
0.023882	0.125897	-0.160697	-0.152658	0.070775	0.144824	-
0.101239	-0.069558	-0.018425	0.064413	-0.008216	0.021009	
0.114284	0.017901	0.064284	0.024127	0.111379	0.030259	
0.063097	-0.068217	-0.012136	-0.087544	0.130794	0.029062	
z30	-0.025709	0.074010	-0.046704	0.084011	-0.171445	-
0.070705	-0.102057	-0.069417	0.164026	0.042199	0.135252	
0.028242	-0.122365	0.148922	-0.018185	0.251866	0.243424	
0.038724	0.085357	0.050679	-0.241426	-0.049477	0.211685	-

0.266098	-0.148352	0.070473	0.039313	0.018658	0.024233	
0.058527	0.203137	-0.312978	0.247787	-0.077158	0.128816	
x31	0.152722	0.253670	0.066741	-0.048525	0.078519	
0.135441	0.010927	-0.020081	0.018611	-0.062760	-0.015738	-
0.021076	0.043691	0.013453	-0.056810	0.099626	-0.061891	
0.013325	-0.018401	0.191302	0.051857	0.007303	-0.004106	-
0.074512	-0.045740	0.038696	-0.160145	0.197438	-0.063469	-
0.132535	-0.084267	0.149169	-0.111543	0.011718	-0.008398	
y31	0.014549	-0.030447	-0.024437	-0.106012	0.136100	-
0.021649	-0.149763	0.168263	0.018659	0.080673	-0.019272	-
0.023673	0.016763	0.044834	0.007344	-0.018428	0.166168	-
0.013938	-0.027189	-0.017155	-0.018631	-0.043492	-0.087032	
0.054631	-0.074616	-0.062450	0.091702	-0.036300	-0.360587	
0.120389	0.086033	0.102823	0.079956	0.005074	0.045266	
z31	-0.021421	0.080291	-0.035682	0.115320	-0.114875	-
0.016423	-0.104946	-0.060925	0.163891	0.067697	-0.021704	-
0.123317	-0.025611	-0.042468	-0.135504	0.224738	0.029795	
0.022702	0.040769	0.389054	0.079302	-0.085009	-0.020606	-
0.262108	-0.125307	-0.205098	0.152172	-0.016542	0.209125	-
0.051099	-0.095215	0.129784	-0.271875	0.089098	-0.270757	
x32	0.151068	0.146313	0.043288	0.008114	0.045722	
0.161041	0.003555	0.015831	-0.009808	-0.118763	0.037597	
0.068682	0.154857	-0.009729	0.009931	0.000977	0.066714	
0.059525	0.002387	-0.008466	-0.093138	-0.186341	-0.031556	
0.050988	0.118397	-0.064190	0.013196	0.125512	-0.016935	
0.005575	0.027901	0.119644	0.037665	0.023113	0.006609	
y32	0.016176	-0.026821	0.003627	-0.103112	0.130367	-
0.022856	-0.113550	0.115226	0.055729	0.088378	0.019423	-
0.041080	-0.069564	0.120594	0.114773	-0.015612	0.128008	
0.021766	-0.018714	0.061987	0.035308	0.052083	-0.069757	-
0.106774	-0.107573	-0.103115	0.112192	-0.081353	-0.211037	-
0.030931	0.064220	0.137095	0.025633	-0.062039	-0.066204	
z32	-0.009475	0.071888	-0.039215	0.102053	-0.103264	-
0.014325	-0.086690	-0.007978	0.090757	0.031128	-0.025048	-
0.204035	0.145326	-0.060938	-0.099079	-0.036622	-0.007296	
0.093649	0.009032	0.087347	-0.081938	-0.539749	-0.264236	
0.288821	0.075985	0.007478	-0.021942	0.114259	-0.098435	
0.093777	-0.068514	-0.009619	0.132581	-0.100884	0.093599	
x33	0.143043	0.049048	0.014445	0.054393	0.034224	
0.161114	0.021849	0.015459	0.020496	-0.132286	0.035972	
0.084827	0.167537	-0.043827	0.101278	-0.001790	0.130478	
0.093598	-0.004672	-0.034105	-0.001008	-0.012703	0.039230	-
0.088884	0.127672	-0.086147	0.057266	-0.072026	0.037763	
0.041922	0.100559	0.041771	-0.092362	0.092457	-0.006125	
y33	0.016649	-0.022035	0.027231	-0.095358	0.099728	-
0.032000	-0.081101	0.081037	0.044168	0.080274	0.082619	-
0.029371	-0.108553	0.184477	0.144596	-0.077499	0.072666	
0.037075	-0.031200	0.054322	-0.003472	-0.033304	-0.050831	-
0.002689	-0.014741	-0.004712	0.028960	0.001171	-0.009534	-
0.130410	-0.066444	0.024000	-0.002935	-0.110421	-0.123699	
z33	-0.012170	0.077559	-0.059675	0.105608	-0.067390	
0.014935	-0.022587	-0.032829	0.127367	0.050687	-0.095469	-
0.221558	0.130979	-0.154873	0.103478	0.013366	0.026737	
0.034372	-0.042114	0.045508	0.246266	0.070299	-0.100686	-
0.068779	0.012199	-0.055390	0.002364	-0.108489	0.005309	
0.023082	0.094712	-0.014872	0.009746	0.009290	0.250012	
x34	0.133757	-0.043853	-0.007845	0.074470	0.016135	
0.133505	0.028355	0.019297	0.019485	-0.126199	0.033449	
0.077479	0.144353	-0.059595	0.106919	-0.042549	0.160482	
0.117113	0.024575	-0.071396	-0.015107	0.008713	0.058199	-

0.072735	0.168612	-0.002177	-0.027623	-0.184078	0.050172	-
0.150188	0.238714	-0.062102	-0.091811	0.013527	0.098800	
y34	0.013934	-0.018236	0.040058	-0.064292	0.065980	-
0.031803	-0.032228	0.026772	0.028142	0.054985	0.108820	-
0.024990	-0.136857	0.176456	0.161754	-0.072718	0.042537	
0.043660	-0.027627	0.076130	0.013324	-0.040107	-0.011725	
0.004422	-0.010915	-0.017271	-0.022480	0.060150	0.233250	-
0.061397	-0.141499	-0.057221	-0.021047	-0.008102	0.051633	
z34	-0.008821	0.079249	-0.077544	0.085960	-0.014605	
0.027801	0.007175	-0.015536	0.132743	0.095996	-0.046328	-
0.211054	0.138045	-0.141491	0.136257	-0.094491	0.047035	
0.073736	-0.089762	-0.225167	0.280750	0.220328	-0.065480	-
0.041944	0.180898	0.049664	-0.007325	-0.007546	-0.093049	-
0.034540	-0.174989	-0.093888	0.035944	-0.049518	-0.122821	
x35	0.117888	-0.084953	-0.057522	0.073192	-0.096943	
0.094404	-0.046738	-0.023416	-0.135641	-0.010719	0.147751	
0.155390	-0.020268	0.134418	-0.016518	-0.196636	0.280243	-
0.286456	0.095222	0.136740	0.087762	0.057749	-0.150832	-
0.038791	-0.119476	0.024962	0.025065	-0.017999	0.102401	
0.103567	-0.223036	-0.043155	0.013575	-0.135432	0.058423	
y35	0.014668	-0.051612	0.068151	-0.003546	0.105083	-
0.042758	0.091173	-0.002207	0.147648	-0.034442	0.015177	-
0.108939	-0.015465	-0.031144	0.226498	0.042291	-0.111673	
0.344071	-0.103010	-0.091926	-0.016920	-0.071701	0.101752	-
0.048967	0.151499	0.107106	-0.083096	0.013655	0.166095	
0.036510	0.053719	-0.069981	-0.033063	0.073433	-0.087851	
z35	-0.010203	0.110776	-0.105810	0.054661	-0.030436	
0.058182	-0.008857	-0.026225	-0.102781	0.178727	0.089573	-
0.015254	-0.095995	0.100187	0.025032	-0.246413	0.099754	-
0.336629	0.066298	0.041662	0.146456	0.223293	-0.063661	
0.151883	0.066600	0.167389	-0.056544	-0.022270	-0.019706	-
0.114724	0.199782	0.044603	0.042639	0.175517	-0.123004	
x36	0.117360	-0.219965	-0.054743	0.080394	-0.020169	-
0.016167	0.012475	0.022421	0.021387	-0.046616	-0.015998	
0.024002	0.038353	-0.051747	0.066478	-0.048198	0.064789	
0.104598	-0.008230	0.014483	0.021114	0.030873	0.095379	-
0.004094	-0.018997	0.077582	0.036810	0.010928	-0.050230	
0.132259	-0.168114	0.037694	0.068449	-0.080133	-0.182325	
y36	0.003982	-0.001442	0.050408	0.060351	-0.024962	-
0.014418	0.109224	-0.118114	-0.000493	-0.007457	0.138378	-
0.039086	-0.073770	0.043151	0.011836	0.009881	0.007134	-
0.053467	0.038298	-0.045460	0.074348	0.013980	-0.138820	-
0.077357	0.070838	0.014303	-0.074313	-0.012970	0.165934	
0.079871	0.011723	-0.027734	0.036995	0.095465	0.185372	
z36	-0.001729	0.022437	-0.104144	0.050753	0.133610	
0.048162	0.073057	0.076141	-0.074246	0.047138	-0.058353	
0.113107	0.032627	-0.001595	0.187488	-0.198045	-0.211231	
0.159334	-0.086331	0.374067	-0.191032	0.135867	0.392721	
0.341165	-0.134819	0.161915	0.069637	-0.029783	-0.010958	
0.000665	0.010409	-0.013654	-0.073407	-0.072165	0.068546	
x37	0.100337	-0.299165	-0.091355	0.071819	-0.026865	-
0.131663	-0.009066	0.017324	0.045032	0.064375	-0.079535	-
0.074629	-0.094180	-0.046346	-0.008607	0.028051	-0.101790	
0.002251	-0.014279	0.011763	-0.040239	0.004864	0.018280	
0.044386	0.066841	-0.029157	0.024160	-0.000054	-0.051444	-
0.040542	0.041336	-0.104946	0.114356	0.051030	-0.068923	
y37	-0.017691	0.011031	0.032316	0.147389	-0.086021	
0.008919	0.214417	-0.188547	-0.044508	-0.037049	0.080453	
0.007563	-0.001454	-0.055842	-0.074367	0.021074	-0.081287	-
0.079879	0.011273	0.066203	-0.057565	0.014415	-0.012292	

0.066142	0.014222	-0.024300	0.007600	0.025747	-0.012191	-
0.036159	0.017318	0.021114	-0.054207	0.018710	0.155254	
z37	0.040769	-0.019748	-0.062041	0.005581	0.170799	
0.016305	0.066445	0.118220	-0.178720	-0.078922	0.028458	
0.159246	-0.060718	0.052953	-0.161728	-0.042600	-0.197263	
0.081510	-0.094486	-0.338193	0.242910	-0.082892	0.104767	-
0.420192	-0.236857	0.043820	-0.125063	0.123534	-0.090326	-
0.032665	0.026663	0.120521	-0.014882	-0.100255	0.101934	
x38	0.091134	-0.383292	-0.118550	0.039384	-0.064307	-
0.274475	-0.036971	0.007988	0.053820	0.156202	-0.144360	-
0.191031	-0.239725	0.001203	-0.093012	0.100385	-0.260598	-
0.096878	-0.058469	-0.050156	0.033272	0.000614	-0.037715	
0.074568	-0.113098	-0.000705	-0.051621	0.048608	0.006299	-
0.045096	0.083654	0.038507	-0.056733	0.037361	0.107544	
y38	-0.028461	0.019098	0.022821	0.228244	-0.153127	
0.031830	0.330607	-0.274584	-0.122936	-0.121353	0.033633	
0.048332	0.103708	-0.204820	-0.223651	0.064553	-0.112908	-
0.142660	0.049379	-0.031065	-0.007287	0.030400	0.077966	
0.063954	-0.121326	0.003156	0.086655	-0.036026	-0.206726	-
0.009268	0.012434	0.003388	0.037403	-0.087305	-0.179229	
z38	0.040642	-0.010183	-0.100928	0.019086	0.260885	-
0.008904	0.103675	0.208214	-0.196581	-0.035653	0.079049	
0.213591	-0.103656	0.042288	-0.078639	0.080792	-0.228759	-
0.028375	0.153876	-0.033397	-0.330274	-0.014233	-0.295677	-
0.046626	0.219839	-0.281912	0.013121	-0.077070	0.097024	
0.147564	-0.054441	-0.065523	0.051170	0.133313	-0.092752	

Canonical Variate Analysis: CVA ... first turn vs vestibular aqueduct

Dataset: endolymphatic sac vs first turn
Classification criterion: subfam

Groups		Observations
1.	Ca	9
2.	Ce	13
3.	Di	3
4.	Pr	11

Variation among groups, scaled by the inverse of the within-group variation

	Eigenvalues	% Variance	Cumulative %
1.	8.01595925	58.144	58.144
2.	3.67331910	26.645	84.788
3.	2.09712771	15.212	100.000

Mahalanobis distances among groups:

	Ca	Ce	Di
Ce	3.6253		
Di	7.7981	7.2875	
Pr	6.1481	6.0515	7.0760

P-values from permutation tests (10000 permutation rounds) for Mahalanobis distances among groups:

	Ca	Ce	Di
Ce	<.0001		
Di	0.0043	0.0007	

Pr	0.0001	<.0001	0.0016

Procrustes distances among groups:

	Ca	Ce	Di
Ce	0.0273		
Di	0.0488	0.0530	
Pr	0.0581	0.0579	0.0595

P-values from permutation tests (10000 permutation rounds) for Procrustes distances among groups:

	Ca	Ce	Di
Ce	0.7094		
Di	0.5976	0.1435	
Pr	0.0342	0.0001	0.0601

Canonical coefficients:

	CV1	CV2	CV3
x1	-27.0652	115.6062	-96.6832
y1	-83.9978	134.1331	50.1140
z1	-144.5923	-183.6329	2.0961
x2	157.9728	-149.2253	71.2798
y2	59.2479	56.5795	-10.8071
z2	35.6291	-49.3886	-25.6590
x3	10.3670	-4.0075	46.8331
y3	1.0382	32.7572	-6.2374
z3	83.5621	-16.1507	-83.5978
x4	22.9034	29.8525	87.0873
y4	77.4724	1.1862	108.4640
z4	31.7428	25.2795	-105.5942
x5	-7.5267	-13.6683	-34.2994
y5	26.9590	-47.8591	-100.3795
z5	10.1599	15.4803	-33.5887
x6	-4.2484	-13.1803	-28.7120
y6	33.7637	-30.9738	-17.5468
z6	41.8788	-5.8559	50.6273
x7	-11.9349	-13.6204	40.4828
y7	-10.6822	33.7891	27.6202
z7	19.4763	30.5078	44.7006
x8	53.9285	-61.6694	29.7867
y8	19.6569	-11.5519	-22.6177
z8	-14.3863	65.4065	50.5992
x9	49.6117	-64.9612	15.2506
y9	-15.9032	-21.8847	-25.4648
z9	-24.1210	105.8837	71.2958
x10	-7.8303	-7.2354	-29.8449
y10	-39.8199	-32.0024	95.7669
z10	-56.2624	87.0121	123.5003
x11	31.5063	-21.6142	-121.2720
y11	-21.3694	11.4778	-32.3451
z11	-8.9661	73.7140	7.6593
x12	34.9175	30.5732	-48.3278
y12	3.2248	35.5930	-29.2464
z12	-1.7706	50.7335	-72.8423
x13	49.5478	-58.7716	-13.9274
y13	39.8418	-57.0344	14.3552
z13	-5.6785	-9.3240	-44.0090
x14	99.5368	-43.9860	77.2881

y14	57.1078	-64.8808	72.9286
z14	-41.6219	-29.4921	-30.7562
x15	68.8601	57.1887	-16.2876
y15	9.2589	-14.8751	17.9721
z15	-50.3920	-51.5297	7.4101
x16	-25.1131	-37.4331	35.7126
y16	-52.6705	-49.3588	39.1614
z16	-33.6291	-52.8402	32.1346
x17	-177.7503	192.5329	-75.9769
y17	-54.9964	-15.8698	36.1314
z17	59.2285	-95.1099	75.6508
x18	-272.2021	123.8787	-62.2551
y18	-45.1016	-4.9501	-29.3916
z18	127.2765	-40.4139	31.0611
x19	-84.1439	54.2117	167.0384
y19	31.9298	92.3252	-233.9239
z19	40.8942	-7.2415	25.4470
x20	56.4043	-182.3824	148.5505
y20	39.9159	-50.4460	-47.1990
z20	1.9226	63.1951	-39.6192
x21	23.5916	-108.1107	59.0317
y21	39.9982	-24.6807	-6.5732
z21	-21.1533	10.7791	-34.4531
x22	50.0106	7.6865	71.8837
y22	29.9679	23.8329	63.6089
z22	-52.3477	-30.4442	-89.2348
x23	-54.1952	93.4512	-132.7792
y23	5.5228	28.9447	48.5831
z23	-26.6895	-45.2722	-42.0013
x24	-61.2737	-82.1614	-37.9963
y24	4.2168	22.0078	59.7425
z24	-17.6461	10.6697	-81.8720
x25	-18.6900	-64.2930	-178.1623
y25	-5.1738	2.5818	-37.6705
z25	-21.2223	51.9324	49.1837
x26	-58.3293	58.3250	-213.7648
y26	-18.5141	5.9061	-51.8294
z26	15.0168	21.4701	95.3191
x27	66.1770	83.6182	-17.1036
y27	-7.6566	8.6395	-30.1909
z27	-4.5280	45.5904	31.8948
x28	89.8720	7.1225	265.3010
y28	8.6481	-20.6260	66.3397
z28	3.6514	-27.9497	-50.4441
x29	35.4656	102.5526	-66.1463
y29	14.1273	-50.6818	-0.2221
z29	-158.2430	98.7357	-119.7546
x30	-11.7169	129.3031	-9.6662
y30	-17.9807	-29.8028	78.7511
z30	84.4941	114.2572	72.3565
x31	-9.7013	-101.7955	109.3692
y31	-92.0953	164.8175	-162.0073
z31	-62.0534	-155.4859	144.5393
x32	0.6212	-25.9724	17.6929
y32	-96.2076	86.8611	-89.3958
z32	-22.1605	-66.3488	-86.3094
x33	29.8033	-27.6776	21.8426
y33	-83.5251	-10.5973	-10.6816
z33	131.4043	-160.8008	48.7966
x34	55.6218	-60.7008	27.3951

y34	1.2822	-160.6321	171.5999
z34	73.9190	-55.1022	-88.1334
x35	-136.9649	-88.8846	-1.4494
y35	106.2313	-38.8839	80.9523
z35	-22.0957	58.3486	-28.5883
x36	-124.1035	96.5026	-134.2741
y36	103.3644	-158.3098	114.7778
z36	-107.0869	136.3341	2.7095
x37	49.5912	49.3553	-30.4771
y37	28.0109	-55.7502	37.8544
z37	23.7544	57.5016	67.2174
x38	56.4795	-0.4100	57.5791
y38	-95.0929	210.2191	-240.9933
z38	112.6358	-40.4479	22.2581

New Procrustes fit:
Dataset 'angles between vestibular aqudect and first turn'

4 landmarks in 3 dimensions.
The dataset contains 40 observations, of which 36 are included for analyses.

Average shape:

Lmk.	Axis 1 (x)	Axis 2 (y)	Axis 3 (z)
1	0.18440990	-0.26663638	-0.01951883
2	0.62170240	0.18923769	-0.00608989
3	-0.14721429	-0.03416426	0.04741036
4	-0.65889802	0.11156295	-0.02180163

Procrustes sums of squares: 0.20652831545340927
Tangent sums of squares: 0.20470596918983677

Data matrices in this dataset:
- angles between vestibular aqudect and first turn, raw data
- angles between vestibular aqudect and first turn, centroid size
- angles between vestibular aqudect and first turn, Procrustes coordinates

New Procrustes fit:
Dataset 'angles between vestibular aqudect and first turn'

4 landmarks in 3 dimensions.
The dataset contains 40 observations, of which 36 are included for analyses.

Average shape:

Lmk.	Axis 1 (x)	Axis 2 (y)	Axis 3 (z)
1	0.18440990	-0.26663638	-0.01951883
2	0.62170240	0.18923769	-0.00608989
3	-0.14721429	-0.03416426	0.04741036
4	-0.65889802	0.11156295	-0.02180163

Procrustes sums of squares: 0.20652831545340927
Tangent sums of squares: 0.20470596918983677

Data matrices in this dataset:
- angles between vestibular aqudect and first turn, raw data
- angles between vestibular aqudect and first turn, centroid size
- angles between vestibular aqudect and first turn, Procrustes coordinates

Principal Component Analysis: PCA: CovMatrix, angles between vestibular aqudect and first turn, Procrustes coordinates

	Eigenvalues	% Variance	Cumulative %
1.	0.00302170	51.664	51.664
2.	0.00127907	21.869	73.533
3.	0.00072796	12.446	85.980
4.	0.00058314	9.970	95.950
5.	0.00023688	4.050	100.000

Total variance: 0.00584874

Variance of the eigenvalues: 0.0000009702390
Eigenvalue variance scaled by total variance: 0.02836
Eigenvalue variance scaled by total variance and number of variables: 0.17727
Note: throughout all calculations of eigenvalue variances, the dimensionality used was 5.
Please double-check because this dimensionality may not be appropriate for all situations.

Principal Component Coefficients

	PC1	PC2	PC3	PC4	PC5
x1	-0.131718	-0.541839	0.029683	-0.358493	-0.451557
y1	-0.028985	-0.340985	-0.422118	0.583975	-0.073410
z1	0.028732	-0.023751	-0.307741	-0.156036	0.124859
x2	0.374523	0.071683	-0.101666	0.357193	0.210218
y2	0.136580	0.506303	0.023338	-0.223843	-0.022584
z2	-0.004618	-0.037222	-0.004493	-0.063525	0.124218
x3	-0.726614	0.258709	-0.006709	0.076344	0.351894
y3	0.067098	-0.367466	0.601823	-0.104783	0.442958
z3	-0.123168	0.167079	0.514537	0.435844	-0.464626
x4	0.483809	0.211446	0.078692	-0.075043	-0.110555
y4	-0.174693	0.202148	-0.203044	-0.255349	-0.346964
z4	0.099054	-0.106105	-0.202303	-0.216283	0.215548

Canonical Variate Analysis: CVA ... angle between vestibular aqueduct and first turn cochlea

Dataset: angles between vestibular aqudect and first turn
Classification criterion: subfam

Groups		Observations
1.	Ca	9
2.	Ce	13
3.	Di	3
4.	Pr	11

Variation among groups, scaled by the inverse of the within-group variation

	Eigenvalues	% Variance	Cumulative %
1.	1.24901063	81.918	81.918
2.	0.19890030	13.045	94.963
3.	0.07680598	5.037	100.000

Mahalanobis distances among groups:

	Ca	Ce	Di
Ce	1.0775		
Di	1.7616	2.3673	
Pr	2.0208	2.4364	1.4049

P-values from permutation tests (10000 permutation rounds) for Mahalanobis distances among groups:

	Ca	Ce	Di
Ce	0.3718		
Di	0.3600	0.0227	
Pr	0.0016	0.0001	0.3561

Procrustes distances among groups:

	Ca	Ce	Di
Ce	0.0252		
Di	0.0545	0.0671	
Pr	0.0766	0.0829	0.0418

P-values from permutation tests (10000 permutation rounds) for Procrustes distances among groups:

	Ca	Ce	Di
Ce	0.6277		
Di	0.2608	0.0453	
Pr	0.0020	0.0004	0.4142

Canonical coefficients:

	CV1	CV2	CV3
x1	11.3392	2.8011	11.9158
y1	-0.7480	17.9295	21.7092
z1	-6.2266	11.2302	-9.8408
x2	-10.5347	1.5887	1.5804
y2	-11.2112	-5.2578	-7.9591
z2	0.5866	0.5793	-7.0291
x3	11.9912	2.6306	-16.0303
y3	15.8958	-18.9693	-19.8616
z3	10.1431	-19.5412	31.7440
x4	-12.7957	-7.0204	2.5341
y4	-3.9367	6.2976	6.1115
z4	-4.5031	7.7317	-14.8741

New Procrustes fit:
Dataset 'first turn vs second turn orientation'

55 landmarks in 3 dimensions.

The dataset contains 40 observations, of which 36 are included for analyses.

Average shape:

Lmk.	Axis 1 (x)	Axis 2 (y)	Axis 3 (z)
1	-0.14740773	-0.08735086	0.07911699
2	-0.14818085	-0.08385919	0.06644871
3	-0.14694992	-0.07904466	0.05434608
4	-0.14382348	-0.07426191	0.04292995
5	-0.13919728	-0.07056657	0.03187152
6	-0.13289189	-0.06745158	0.02126520
7	-0.12540482	-0.06543416	0.01063493
8	-0.11676278	-0.06594525	-0.00102326
9	-0.10565379	-0.06263104	-0.01086586
10	-0.09400990	-0.06899945	-0.02093053
11	-0.08139720	-0.07019177	-0.02815332
12	-0.06869268	-0.07168819	-0.03410061
13	-0.05574490	-0.07327457	-0.03852613
14	-0.04259251	-0.07491307	-0.04152421
15	-0.02926975	-0.07669746	-0.04306858
16	-0.01572331	-0.07869561	-0.04336564
17	-0.00186844	-0.08068927	-0.04194602
18	0.01224403	-0.08257120	-0.03869160
19	0.02649565	-0.08416969	-0.03387599
20	0.04037900	-0.08551132	-0.02742462
21	0.05382984	-0.08564811	-0.02036154
22	0.06695992	-0.08509112	-0.01298222
23	0.07968534	-0.08311541	-0.00507251
24	0.09183522	-0.08017939	0.00354022
25	0.10360100	-0.07600351	0.01271983
26	0.11506559	-0.07066619	0.02243113
27	0.12603344	-0.06403828	0.03276367
28	0.13619398	-0.05584054	0.04364152
29	0.15020247	-0.04095841	0.05893644
30	0.16193177	-0.02391836	0.07206764
31	0.17163085	-0.00515244	0.08285162
32	-0.04925585	0.22470740	0.02148416
33	-0.05822458	0.20994826	0.01769559
34	-0.06528844	0.19349418	0.01397544
35	-0.07009830	0.17515346	0.01045960
36	-0.07218845	0.15506109	0.00719153
37	-0.07127938	0.13673522	0.00273022
38	-0.06799872	0.11969563	-0.00151276
39	-0.06254693	0.10409465	-0.00543418
40	-0.05537621	0.08988758	-0.00908296
41	-0.04635772	0.07711041	-0.01239045
42	-0.03571091	0.06545173	-0.01555850
43	-0.02322917	0.05488053	-0.01863668
44	-0.00857270	0.04586044	-0.02143201
45	0.00818792	0.03870430	-0.02369836
46	0.02528937	0.03346682	-0.02451137
47	0.04205173	0.03098679	-0.02427667
48	0.05819990	0.03054519	-0.02347968
49	0.07378706	0.03220575	-0.02200867
50	0.08877372	0.03650801	-0.01951269
51	0.10350786	0.04192693	-0.01713942
52	0.11751869	0.05036791	-0.01339942
53	0.13109186	0.06100765	-0.00919293
54	0.14334591	0.07474903	-0.00439077

55	0.15385647	0.09200959	-0.00153178

Procrustes sums of squares: 1.176182627220216
Tangent sums of squares: 0.883651189143264

Data matrices in this dataset:
- first turn vs second turn orientation, raw data
- first turn vs second turn orientation, centroid size
- first turn vs second turn orientation, Procrustes coordinates

New Procrustes fit:
Dataset 'first turn vs second turn orientation'

55 landmarks in 3 dimensions.
The dataset contains 40 observations, of which 36 are included for analyses.

Average shape:

Lmk.	Axis 1 (x)	Axis 2 (y)	Axis 3 (z)
1	-0.14740773	-0.08735086	0.07911699
2	-0.14818085	-0.08385919	0.06644871
3	-0.14694992	-0.07904466	0.05434608
4	-0.14382348	-0.07426191	0.04292995
5	-0.13919728	-0.07056657	0.03187152
6	-0.13289189	-0.06745158	0.02126520
7	-0.12540482	-0.06543416	0.01063493
8	-0.11676278	-0.06594525	-0.00102326
9	-0.10565379	-0.06263104	-0.01086586
10	-0.09400990	-0.06899945	-0.02093053
11	-0.08139720	-0.07019177	-0.02815332
12	-0.06869268	-0.07168819	-0.03410061
13	-0.05574490	-0.07327457	-0.03852613
14	-0.04259251	-0.07491307	-0.04152421
15	-0.02926975	-0.07669746	-0.04306858
16	-0.01572331	-0.07869561	-0.04336564
17	-0.00186844	-0.08068927	-0.04194602
18	0.01224403	-0.08257120	-0.03869160
19	0.02649565	-0.08416969	-0.03387599
20	0.04037900	-0.08551132	-0.02742462
21	0.05382984	-0.08564811	-0.02036154
22	0.06695992	-0.08509112	-0.01298222
23	0.07968534	-0.08311541	-0.00507251
24	0.09183522	-0.08017939	0.00354022
25	0.10360100	-0.07600351	0.01271983
26	0.11506559	-0.07066619	0.02243113
27	0.12603344	-0.06403828	0.03276367
28	0.13619398	-0.05584054	0.04364152
29	0.15020247	-0.04095841	0.05893644
30	0.16193177	-0.02391836	0.07206764
31	0.17163085	-0.00515244	0.08285162
32	-0.04925585	0.22470740	0.02148416
33	-0.05822458	0.20994826	0.01769559
34	-0.06528844	0.19349418	0.01397544
35	-0.07009830	0.17515346	0.01045960
36	-0.07218845	0.15506109	0.00719153
37	-0.07127938	0.13673522	0.00273022
38	-0.06799872	0.11969563	-0.00151276

39	-0.06254693	0.10409465	-0.00543418
40	-0.05537621	0.08988758	-0.00908296
41	-0.04635772	0.07711041	-0.01239045
42	-0.03571091	0.06545173	-0.01555850
43	-0.02322917	0.05488053	-0.01863668
44	-0.00857270	0.04586044	-0.02143201
45	0.00818792	0.03870430	-0.02369836
46	0.02528937	0.03346682	-0.02451137
47	0.04205173	0.03098679	-0.02427667
48	0.05819990	0.03054519	-0.02347968
49	0.07378706	0.03220575	-0.02200867
50	0.08877372	0.03650801	-0.01951269
51	0.10350786	0.04192693	-0.01713942
52	0.11751869	0.05036791	-0.01339942
53	0.13109186	0.06100765	-0.00919293
54	0.14334591	0.07474903	-0.00439077
55	0.15385647	0.09200959	-0.00153178

Procrustes sums of squares: 1.176182627220216
Tangent sums of squares: 0.883651189143264

Data matrices in this dataset:
- first turn vs second turn orientation, raw data
- first turn vs second turn orientation, centroid size
- first turn vs second turn orientation, Procrustes coordinates

Principal Component Analysis: PCA: CovMatrix, first turn vs second turn orientation, Procrustes coordinates

	Eigenvalues	% Variance	Cumulative %
1.	0.01251844	49.584	49.584
2.	0.00370521	14.676	64.259
3.	0.00305327	12.094	76.353
4.	0.00174346	6.906	83.258
5.	0.00115498	4.575	87.833
6.	0.00086061	3.409	91.242
7.	0.00039903	1.580	92.822
8.	0.00035145	1.392	94.214
9.	0.00030293	1.200	95.414
10.	0.00029247	1.158	96.573
11.	0.00018724	0.742	97.314
12.	0.00015457	0.612	97.926
13.	0.00010580	0.419	98.345
14.	0.00008373	0.332	98.677
15.	0.00006865	0.272	98.949
16.	0.00004285	0.170	99.119
17.	0.00003910	0.155	99.274
18.	0.00003298	0.131	99.404
19.	0.00003094	0.123	99.527
20.	0.00002562	0.101	99.628
21.	0.00001984	0.079	99.707
22.	0.00001673	0.066	99.773
23.	0.00001341	0.053	99.826
24.	0.00001077	0.043	99.869
25.	0.00000854	0.034	99.903
26.	0.00000567	0.022	99.925
27.	0.00000398	0.016	99.941

```
 28.   0.00000381   0.015      99.956
 29.   0.00000345   0.014      99.970
 30.   0.00000264   0.010      99.980
 31.   0.00000143   0.006      99.986
 32.   0.00000123   0.005      99.991
 33.   0.00000115   0.005      99.995
 34.   0.00000088   0.003      99.999
 35.   0.00000035   0.001     100.000
```

Total variance: 0.02524718

Variance of the eigenvalues: 0.0000011480473
Eigenvalue variance scaled by total variance: 0.00180
Eigenvalue variance scaled by total variance and number of variables: 0.28638
Note: throughout all calculations of eigenvalue variances, the dimensionality used was 158.
Please double-check because this dimensionality may not be appropriate for all situations.
Warning: There appear to be fewer PCs than dimensions in shape space.
This can have the following reasons, among others:
-- the degrees of freedom are less than the shape dimensionality (sample size, etc.).
-- there is complete integration in the data.
Interpret the results cautiously and check for reasons why this is the case.

Principal Component Coefficients

```
        PC1         PC2         PC3         PC4         PC5         PC6
PC7          PC8         PC9         PC10        PC11        PC12
PC13         PC14        PC15        PC16        PC17        PC18
PC19         PC20        PC21        PC22        PC23        PC24
PC25         PC26        PC27        PC28        PC29        PC30
PC31         PC32        PC33        PC34        PC35
   x1 -0.036763   0.118691  -0.123822   0.108737  -0.156199  -0.015228
0.098606   -0.042797   0.264686  -0.055150   0.094524  -0.018537
0.240818    0.132339   0.034756  -0.106289   0.170325  -0.207057
0.138871   -0.212035  -0.250526  -0.087036  -0.034516   0.169026
0.027754   -0.000081  -0.040664   0.024568  -0.065725  -0.273216
0.065915   -0.033134  -0.072610  -0.079464   0.003417
   y1 -0.151678   0.151872   0.085356   0.002077   0.008935   0.007779
     -0.076474   0.023135  -0.083825  -0.158023   0.227843  -0.043343
     -0.134849  -0.094959   0.135777  -0.207265   0.167778  -0.184582
     -0.073654   0.116876   0.005589   0.075214   0.210267  -0.014232
     -0.099409   0.013787   0.036739   0.078952   0.044355  -0.042340
0.142891    0.231021   0.078555  -0.038871   0.056047
   z1  0.001022  -0.041607   0.009470   0.041072   0.108197  -0.021908
     -0.051732  -0.158736  -0.231456   0.027007  -0.069188   0.188330
0.323763   -0.285775   0.071616  -0.178737  -0.078548   0.079868  -
0.148322   -0.271455  -0.015402   0.086248  -0.036552   0.040684  -
0.060576    0.006704   0.001753   0.000243  -0.104501  -0.101560  -
0.065626    0.099209   0.023219  -0.042833   0.033123
   x2 -0.043585   0.108779  -0.124454   0.088865  -0.167956   0.006769
0.050219   -0.064342   0.223004  -0.082427   0.049754   0.017078
0.149466    0.038889   0.019071  -0.071630   0.109549  -0.075381
0.096268   -0.099330  -0.119047  -0.049989  -0.062123   0.044975
0.011904   -0.030417   0.045308   0.027102   0.079866   0.064871
0.013513    0.118936  -0.002012  -0.002073  -0.036003
```

y2 -0.150344 0.141183 0.095382 -0.001125 0.027191 0.018157
-0.087355 0.017305 -0.085526 -0.134084 0.161717 -0.012927
-0.082928 -0.081008 0.093469 -0.151683 0.152616 -0.096550
-0.107885 0.135356 0.035928 -0.021362 0.037431 -0.037521
0.012312 -0.035158 0.060460 -0.082711 0.097513 0.087755
0.020001 -0.036699 -0.086091 -0.131614 -0.082143
z2 0.009426 -0.011624 -0.010061 0.060459 0.109555 -0.043714
-0.007825 -0.115854 -0.167885 0.071903 0.011176 0.145112
0.226360 -0.134325 0.052756 -0.125827 -0.028416 0.026554 -
0.053765 -0.140063 0.024838 0.045644 -0.002696 -0.017774 -
0.005144 -0.020464 0.010593 -0.058985 -0.016895 0.001633 -
0.005189 -0.002938 -0.034608 0.026903 -0.037585
x3 -0.053757 0.099822 -0.119305 0.072391 -0.171402 0.020146
0.012550 -0.075762 0.158751 -0.091734 0.021974 0.044304
0.059304 -0.025611 0.001464 -0.031624 0.045726 0.022747
0.036928 -0.018005 0.000029 0.005927 -0.094732 -0.084786 -
0.013830 0.021917 0.015775 -0.062221 0.067674 0.086997 -
0.044929 0.066023 -0.003844 0.041057 -0.051597
y3 -0.152979 0.142138 0.108068 0.002995 0.032189 0.010284
-0.072741 0.018563 -0.084956 -0.101103 0.082328 -0.015180
-0.028896 -0.045387 0.034266 -0.049179 0.115416 -0.046230
-0.097729 0.089193 0.006391 -0.047082 -0.081559 -0.006215
0.019358 -0.036836 0.025902 -0.073250 0.033007 0.038587 -
0.035741 -0.123178 -0.121035 -0.027455 -0.133268
z3 0.015819 0.010736 -0.025378 0.075289 0.103514 -0.060680
0.026641 -0.081272 -0.108945 0.103719 0.058940 0.106170
0.142603 -0.015850 0.022509 -0.058225 0.007533 0.006380
0.015284 -0.014919 0.043221 0.001994 0.013119 -0.047190 -
0.011331 -0.026749 0.029783 -0.073814 0.055079 0.090789
0.000014 -0.044776 -0.051993 0.010864 -0.075838
x4 -0.058474 0.081293 -0.111907 0.056370 -0.164775 0.032871
-0.005453 -0.084195 0.103202 -0.094466 -0.006767 0.072546
-0.004302 -0.072881 0.004547 -0.009713 0.003428 0.078651
-0.015071 0.030547 0.094796 0.013029 -0.085294 -0.124776
-0.023993 0.038388 -0.000617 -0.073292 0.079681 0.092006
-0.118025 -0.051158 0.046504 0.015007 0.082507
y4 -0.150941 0.146590 0.113470 0.009167 0.038707 0.001133
-0.038036 0.019242 -0.072808 -0.077399 0.019962 -0.010321
0.022197 -0.017230 -0.012912 0.020383 0.070563 -0.016699 -
0.074404 0.034317 -0.013500 -0.062246 -0.098488 0.037315
0.023562 -0.034806 -0.000507 0.020759 0.009124 0.031240 -
0.071672 -0.140808 -0.016549 0.093286 0.109372
z4 0.020542 0.031838 -0.037578 0.083736 0.093374 -0.071789
0.058416 -0.049106 -0.056380 0.124029 0.090292 0.066911
0.074555 0.072254 -0.002796 -0.004899 0.027980 -0.026860
0.064606 0.069650 0.056152 -0.029585 0.057726 -0.061137 -
0.027847 -0.010782 0.001319 -0.036187 0.097780 0.126787
0.005225 -0.084877 -0.007101 0.053127 0.036149
x5 -0.061441 0.062458 -0.107182 0.040420 -0.149190 0.035500
-0.024510 -0.086314 0.059288 -0.095378 -0.040018 0.078058
-0.035514 -0.084963 0.016155 0.013500 -0.058322 0.094087
-0.027655 0.060540 0.099878 0.025126 -0.022834 -0.121181
-0.034572 0.031265 -0.025641 -0.054929 0.048091 0.095644
-0.060346 -0.111779 -0.001567 0.039258 0.001210
y5 -0.144621 0.149670 0.115694 0.018110 0.046523 -0.010142
-0.018250 0.023406 -0.054279 -0.063484 -0.035218 -0.017913
0.076350 0.020325 -0.035262 0.094603 -0.012395 -0.002423 -
0.025637 -0.035072 -0.068531 -0.047651 -0.093435 0.052479
0.045826 -0.023450 0.000516 0.097561 -0.022807 0.011024 -
0.052580 -0.150568 -0.011246 0.153796 0.073490

z5 0.023194 0.053247 -0.047897 0.086527 0.081457 -0.079165
0.076625 -0.010652 -0.006430 0.129442 0.107035 0.024992
0.025365 0.138436 -0.008894 0.049698 0.018983 -0.045685
0.097272 0.096908 0.051821 -0.027828 0.101498 -0.057486 -
0.021046 -0.015366 0.015455 0.018758 0.092051 0.129973
0.042443 -0.113542 -0.000739 0.018579 0.070506
x6 -0.066747 0.043957 -0.102200 0.028302 -0.130138 0.039250
-0.025317 -0.094877 0.004840 -0.080380 -0.075800 0.088657
-0.064416 -0.096114 0.022919 0.003667 -0.090371 0.097945
-0.044414 0.069746 0.127228 0.036477 0.016139 -0.098272
-0.044613 0.006636 -0.000231 -0.006130 0.005034 0.054392
-0.014651 -0.116621 0.063877 0.002521 0.054120
y6 -0.137830 0.152116 0.114023 0.030065 0.047499 -0.022601
0.011073 0.017959 -0.043486 -0.043793 -0.079346 -0.017830
0.090305 0.036710 -0.059383 0.118219 -0.051124 0.033667
0.022137 -0.065655 -0.047991 -0.020822 -0.077013 0.039922
0.019189 0.005327 -0.000009 0.093785 -0.065733 -0.038759 -
0.044203 -0.038212 0.051736 0.094697 0.009553
z6 0.022750 0.071161 -0.055155 0.083489 0.062280 -0.081886
0.088631 0.016705 0.026445 0.120078 0.105779 -0.011577 -
0.021598 0.167081 -0.016312 0.078014 0.005377 -0.061404
0.095155 0.112372 0.055344 -0.020276 0.120378 -0.049489 -
0.046382 0.004454 0.005688 0.034392 0.028769 0.062965
0.037629 -0.089484 0.034737 0.005539 0.030828
x7 -0.071596 0.027635 -0.097053 0.016696 -0.104977 0.033873
-0.029797 -0.095783 -0.046486 -0.052803 -0.099216 0.082130
-0.084872 -0.086318 0.002467 0.003339 -0.103137 0.090161
-0.039268 0.100289 0.093123 0.028553 0.053033 -0.070678
-0.020216 -0.030358 -0.031446 -0.002715 -0.114380 0.032325
0.050907 0.002659 0.089417 0.107836 -0.212861
y7 -0.126050 0.152731 0.111863 0.043359 0.045761 -0.039180
0.033501 0.011738 -0.025419 -0.022455 -0.101103 -0.024449
0.085767 0.044807 -0.102434 0.129551 -0.074356 0.086066
0.076139 -0.056462 -0.043710 0.000680 -0.043090 -0.006704
0.003359 0.021751 -0.085122 0.023641 -0.121827 -0.085584
0.030190 0.117275 0.048281 0.145287 -0.137465
z7 0.022703 0.085351 -0.058115 0.073348 0.039646 -0.084393
0.088266 0.042850 0.057225 0.100267 0.099796 -0.040658 -
0.066780 0.158747 -0.033504 0.102053 -0.013900 -0.030608
0.058404 0.132255 0.033069 0.000666 0.091345 -0.044988 -
0.041729 0.000397 -0.006354 0.014671 -0.103470 -0.004589
0.079228 0.038327 0.070887 0.068043 -0.154847
x8 -0.071712 -0.003932 -0.090016 0.006475 -0.073104 0.030869
-0.018787 -0.103643 -0.104819 -0.021560 -0.117177 0.089503
-0.094678 -0.072114 -0.001662 -0.007253 -0.073315 0.061222
-0.039346 0.086531 0.088066 0.014082 0.085555 0.012876
0.026377 -0.097470 -0.010370 0.066913 -0.089262 0.024166
0.015103 0.026228 0.021464 0.009086 0.016026
y8 -0.064008 0.051098 0.124549 0.056195 0.039310 -0.044614
0.037891 -0.041038 0.003933 -0.033145 -0.097635 0.019759
0.070765 0.024743 -0.069034 0.125922 -0.022757 0.068830
0.075415 -0.065785 0.073544 -0.049881 -0.108398 0.060764
0.013686 -0.081856 -0.107940 0.114832 -0.089339 -0.046068 -
0.026872 0.089790 0.041976 0.087621 0.055808
z8 0.053207 0.025565 -0.046742 0.054664 0.011550 -0.073236
0.063740 0.029821 0.083106 0.041365 0.090725 -0.029178 -
0.097284 0.101047 0.004889 0.112178 0.015388 -0.037798 -
0.057034 0.110089 0.080311 -0.034343 -0.017038 0.056455 -
0.025557 -0.086505 -0.006804 0.105606 -0.142415 -0.009177 -
0.017141 0.070716 0.045111 0.044548 0.002787

x9 -0.101486 0.025371 -0.093747 -0.001394 -0.025695 0.020057
0.002902 -0.073534 -0.167308 0.042479 -0.134589 0.060719 -
0.077749 -0.023802 -0.018551 -0.035928 -0.080656 0.025278
0.034781 0.084261 -0.008125 0.017176 0.212687 0.043769
0.115017 -0.056446 0.029779 0.081459 -0.069442 -0.018028
0.052968 0.009402 -0.018361 -0.116804 0.054899
y9 -0.145326 0.247646 0.089518 0.087625 0.048386 -0.063275
0.093037 0.046479 -0.000403 0.041969 -0.103770 -0.061901
0.055230 0.034753 -0.171725 0.159495 -0.230154 0.199814
0.252887 -0.142383 -0.069530 0.121073 0.194975 -0.249647
0.102220 0.152882 -0.080862 -0.147912 0.079058 -0.025456
0.030553 0.128955 0.060268 -0.288148 0.069599
z9 0.034764 0.061959 -0.049104 0.033372 -0.010785 -0.063749
0.046667 0.065674 0.098139 0.003142 0.062892 -0.052834 -
0.114711 0.028160 0.015902 0.111000 -0.032691 0.030147 -
0.109226 0.074105 0.048997 0.005876 0.002287 0.038029
0.027499 -0.032637 0.019089 0.074178 -0.151742 -0.074195 -
0.058822 0.077229 0.038231 -0.169352 0.035816
x10 -0.095644 -0.023121 -0.088843 -0.015447 0.024758
0.006878 0.019706 -0.075884 -0.222903 0.100625 -0.155143
0.048905 -0.038174 0.048804 -0.024627 -0.078382 -0.015098 -
0.108810 0.084151 0.128374 -0.068831 -0.042967 0.225560
0.203805 0.127980 -0.009545 -0.000418 0.154339 -0.036137 -
0.045645 0.052658 -0.017532 -0.144991 -0.020928 0.079635
y10 0.088460 -0.198117 0.133428 0.075505 0.028415 -
0.065465 0.056173 -0.166979 0.089368 -0.046604 -0.054571
0.108133 0.013905 -0.012398 -0.003246 -0.006451 0.146665 -
0.012648 0.158111 0.132271 0.324920 -0.123541 -0.068527
0.217268 -0.046035 -0.071439 -0.154735 0.006033 0.114347 -
0.115496 -0.031497 0.178694 0.020829 0.021476 -0.086936
z10 0.038261 0.040204 -0.043718 -0.001226 -0.037958 -
0.047808 0.010899 0.079929 0.109190 -0.060792 0.007960 -
0.059841 -0.115797 -0.061801 0.027030 0.098558 -0.063945
0.055832 -0.208285 0.046623 0.078204 0.011394 -0.129605
0.105909 0.020086 0.066361 -0.009105 0.056057 -0.129863 -
0.041882 -0.158431 0.133647 -0.090351 -0.204009 0.046244
x11 -0.106402 -0.047553 -0.067144 -0.023176 0.047275 -
0.011885 0.003897 -0.072398 -0.189819 0.092347 -0.133213 -
0.007190 -0.010213 0.097142 0.008052 -0.018190 0.060403 -
0.077766 0.053385 0.070633 -0.056874 -0.005912 0.108632
0.128377 0.037524 0.059064 -0.035375 0.045022 -0.047678 -
0.025319 -0.063130 0.001376 -0.067598 -0.045966 0.041674
y11 0.093823 -0.172460 0.111644 0.085617 0.017668 -
0.077203 0.043412 -0.155347 0.089226 -0.029909 -0.065209
0.099753 -0.020764 -0.014854 -0.013315 0.023278 0.122737
0.034741 0.103585 0.034773 0.213227 -0.068332 -0.016937
0.084237 -0.037406 -0.074420 -0.072180 -0.039935 0.053255 -
0.095709 -0.077319 -0.069759 0.041686 -0.028811 0.064131
z11 0.036353 0.027373 -0.049337 -0.019066 -0.038801 -
0.049703 -0.020709 0.076997 0.070766 -0.030425 -0.069668 -
0.066111 -0.129656 -0.063883 0.009626 0.091554 -0.031729
0.035006 -0.143378 -0.001308 0.014369 0.052645 -0.085341
0.115586 0.014887 0.078075 -0.080074 -0.066978 -0.084283 -
0.052028 -0.167735 -0.012942 -0.036207 -0.130396 0.051594
x12 -0.116296 -0.069759 -0.046615 -0.031917 0.070155 -
0.030482 -0.006379 -0.073783 -0.155854 0.077532 -0.099215 -
0.043155 0.012626 0.132141 0.031905 0.043424 0.125828 -
0.053664 0.004649 0.023257 -0.037321 0.021870 0.032224
0.023792 -0.061392 0.070462 -0.000812 0.035483 -0.027038 -
0.024868 -0.023230 0.069521 -0.050927 -0.018060 0.006441

y12	0.094998	-0.147543	0.097089	0.095788	0.017685	-
0.079089	0.044891	-0.145707	0.087732	-0.018121	-0.047508	
0.096536	-0.043464	-0.018985	-0.027597	0.013423	0.089660	
0.025329	0.029940	-0.008032	0.088856	-0.021317	0.057486	-
0.005858	-0.057077	0.012101	0.042526	0.058508	0.040550	-
0.062250	0.036472	-0.013954	-0.036111	0.016361	0.072626	
z12	0.030198	0.014414	-0.047527	-0.037734	-0.032013	-
0.048940	-0.036810	0.063459	0.040817	-0.020506	-0.112819	-
0.056253	-0.128101	-0.068222	-0.007402	0.082335	0.005195	-
0.003906	-0.113074	-0.032347	-0.029282	0.060784	-0.014399	
0.057898	-0.023845	0.076764	-0.047562	-0.002523	0.024871	-
0.123897	0.014708	0.000282	-0.095631	0.049291	0.112632	
x13	-0.124098	-0.088267	-0.032753	-0.037803	0.085360	-
0.047873	-0.005644	-0.061192	-0.127051	0.064646	-0.061359	-
0.080987	0.031909	0.153680	0.045250	0.079764	0.148250	-
0.016551	-0.035519	0.009321	-0.000878	0.023299	-0.044818	-
0.029877	-0.066559	0.085582	-0.050561	0.013059	-0.012612	-
0.011764	-0.037573	-0.006090	0.023387	-0.000042	-0.005554	
y13	0.093873	-0.126997	0.080120	0.101944	0.011438	-
0.079427	0.053244	-0.122965	0.075747	-0.003637	-0.032982	
0.064023	-0.065664	-0.017014	-0.039874	0.001063	0.027572	
0.036100	-0.024581	-0.015925	0.024506	0.002795	0.072549	-
0.074254	-0.002621	0.122598	-0.017522	0.154867	0.024673	-
0.043121	0.059224	-0.077316	-0.089511	0.040180	-0.027871	
z13	0.022644	0.001248	-0.046702	-0.055239	-0.030335	-
0.045645	-0.033429	0.063999	0.009195	-0.011873	-0.144379	-
0.066619	-0.122373	-0.060557	-0.026850	0.055420	-0.001012	-
0.029100	-0.073843	-0.024839	-0.004396	0.044579	0.012522	
0.062265	0.041624	0.137072	-0.144947	0.011733	0.066708	-
0.106392	0.047980	-0.064776	-0.037756	0.208327	0.024005	
x14	-0.129696	-0.100965	-0.020359	-0.040026	0.098503	-
0.067270	-0.003140	-0.046220	-0.091631	0.050884	-0.018862	-
0.096519	0.042978	0.148855	0.043046	0.097655	0.152329	
0.017674	-0.059564	-0.020524	0.009953	0.035306	-0.102756	-
0.062503	-0.080175	0.026648	-0.044922	-0.037714	-0.003585	
0.023065	-0.034450	0.010579	0.072335	-0.071251	-0.065960	
y14	0.093697	-0.112488	0.065560	0.103830	0.010661	-
0.083102	0.047889	-0.104587	0.067647	0.014668	-0.021906	
0.053775	-0.075425	-0.015347	-0.035130	-0.007122	-0.013411	
0.036030	-0.069196	-0.051495	-0.042867	0.041281	0.067214	-
0.082209	0.048894	0.060129	0.069679	0.108740	0.023573	
0.021179	0.082927	-0.057342	-0.040871	-0.107697	-0.147307	
z14	0.017188	-0.017242	-0.041781	-0.070966	-0.023820	-
0.047531	-0.036875	0.064656	-0.012582	0.000813	-0.160527	-
0.057397	-0.115729	-0.055631	-0.024710	0.033643	0.010424	-
0.041528	-0.042151	-0.051877	-0.034824	0.034790	-0.036153	
0.067363	0.070365	0.040309	-0.117188	-0.014114	0.113821	-
0.043470	0.056933	-0.058159	0.019430	0.102953	-0.081317	
x15	-0.132807	-0.109085	-0.012093	-0.040820	0.106887	-
0.085066	-0.001005	-0.027360	-0.056088	0.033767	0.027581	-
0.099566	0.050167	0.123509	0.028784	0.103652	0.152364	
0.048743	-0.074615	-0.030377	0.017086	0.036979	-0.127826	-
0.076253	-0.107876	0.013763	-0.035083	-0.060074	0.004832	
0.015874	-0.035113	0.023493	0.072481	-0.063311	-0.003414	
y15	0.091693	-0.095806	0.051535	0.099575	0.008804	-
0.088490	0.034332	-0.086804	0.055339	0.032389	-0.018991	
0.037917	-0.074435	-0.012038	-0.032780	-0.018931	0.000189	
0.020657	-0.097190	-0.049212	-0.096249	0.081620	0.049975	-
0.079660	0.020819	0.061190	0.069395	0.038223	0.023367	
0.030780	0.091448	-0.018815	-0.071954	-0.117196	0.061176	

z15	0.009839	-0.032897	-0.036367	-0.087814	-0.017113	-
0.051378	-0.037190	0.063952	-0.025066	0.002351	-0.163639	-
0.047170	-0.099750	-0.056465	-0.031141	0.008303	0.051602	-
0.044397	0.008504	-0.042290	-0.049420	0.026233	-0.053070	
0.037024	0.054963	-0.014000	-0.086248	-0.062233	0.157526	-
0.074697	0.109500	-0.002838	-0.010250	0.052535	-0.016442	
x16	-0.133814	-0.113356	-0.006595	-0.037958	0.110855	-
0.101533	0.005040	-0.003299	-0.017754	0.011834	0.079618	-
0.092522	0.044604	0.082153	-0.000761	0.104627	0.118789	
0.082598	-0.094245	-0.052721	0.009930	0.029890	-0.100228	-
0.079757	-0.099670	-0.015165	-0.025309	-0.045523	0.024256	
0.015826	-0.032919	0.034731	0.056040	-0.048578	-0.019388	
y16	0.086711	-0.084363	0.042085	0.094742	0.009654	-
0.087894	0.014121	-0.062733	0.045095	0.050028	-0.017971	
0.002684	-0.077391	0.003254	-0.029885	0.000577	-0.021132	
0.004127	-0.135503	-0.060516	-0.128298	0.097758	0.022809	-
0.111375	0.032044	0.041479	0.094316	0.017343	0.063348	
0.033391	0.012617	0.019292	-0.060007	-0.119064	-0.077254	
z16	0.000060	-0.050971	-0.025230	-0.102603	-0.011256	-
0.048517	-0.035442	0.063918	-0.030105	-0.011097	-0.158617	-
0.038412	-0.090539	-0.052985	-0.030733	0.017265	0.059379	-
0.061065	0.029108	-0.079102	0.001477	-0.019688	-0.036419	-
0.002542	0.050050	-0.066182	-0.008049	-0.062461	0.187143	-
0.019586	0.082817	0.136994	0.059105	0.003243	-0.112930	
x17	-0.132175	-0.111944	-0.005438	-0.033268	0.109588	-
0.117186	0.014220	0.024721	0.026546	-0.013188	0.133029	-
0.071650	0.039424	0.022442	-0.041120	0.077361	0.059686	
0.114347	-0.096220	-0.055835	0.005840	0.008118	-0.052016	-
0.034410	-0.047986	-0.042372	-0.016811	-0.022311	0.011330	
0.003049	-0.019933	-0.010645	0.007436	0.006178	0.042042	
y17	0.082295	-0.072649	0.029760	0.084855	0.009678	-
0.090761	-0.005473	-0.049708	0.038207	0.069964	-0.008455	-
0.010047	-0.067520	0.011119	-0.013724	0.011026	-0.011669	-
0.055042	-0.141240	-0.046246	-0.169715	0.104536	0.017418	-
0.047550	0.036099	0.026436	0.133861	-0.112745	0.081050	
0.089594	-0.038973	-0.041247	0.020075	-0.079413	0.046541	
z17	-0.007561	-0.069321	-0.016630	-0.116267	-0.005948	-
0.050143	-0.023189	0.055014	-0.016504	-0.021641	-0.130829	-
0.020164	-0.071742	-0.050635	-0.027089	0.000332	0.073138	-
0.060311	0.062471	-0.077616	0.000766	-0.078142	-0.043074	-
0.026378	0.047570	-0.092098	0.085122	-0.099062	0.184127	
0.018760	0.060123	0.061012	0.063031	-0.022125	0.013596	
x18	-0.128015	-0.105906	-0.007768	-0.025607	0.103580	-
0.132196	0.025872	0.059217	0.072651	-0.043419	0.189983	-
0.036408	0.026855	-0.057474	-0.097422	0.033537	-0.020254	
0.135171	-0.097241	-0.053374	0.006042	-0.031534	0.042526	
0.029110	0.029519	-0.092265	0.014811	0.019753	0.002444	-
0.005827	0.025890	0.004525	-0.016661	0.073103	0.010217	
y18	0.076746	-0.058955	0.022664	0.068891	0.006060	-
0.097955	-0.030326	-0.038631	0.023563	0.065908	-0.026829	-
0.038806	-0.045184	0.025716	-0.015471	-0.016921	0.006331	-
0.082023	-0.143173	-0.045560	-0.143736	0.087669	-0.051759	
0.033799	0.059280	0.046045	-0.010255	-0.041536	-0.104813	
0.277262	0.068697	-0.038086	-0.024440	0.076519	0.043949	
z18	-0.015603	-0.083783	-0.007414	-0.124893	0.000346	-
0.053797	-0.013186	0.054938	-0.005937	-0.035198	-0.089261	-
0.007929	-0.053546	-0.027175	-0.026199	-0.032813	0.087912	
0.042829	0.108579	-0.076128	-0.082184	-0.152324	-0.078954	-
0.063277	0.065340	-0.009205	0.149675	0.069031	0.039940	
0.083681	0.158478	-0.093650	0.084424	-0.079620	-0.025827	

x19	-0.121816	-0.096145	-0.013645	-0.016431	0.093587	-
0.147855	0.044294	0.094000	0.118477	-0.074395	0.253345	
0.009770	0.001843	-0.157764	-0.176277	-0.016532	-0.114486	
0.165161	-0.090174	-0.046538	-0.010796	-0.058463	0.174223	
0.102615	0.107377	-0.170737	0.009470	0.076982	0.010338	-
0.026977	0.077451	-0.007363	-0.095664	0.129952	0.027148	
y19	0.070756	-0.048468	0.009125	0.053073	0.003954	-
0.102530	-0.041783	-0.014296	0.027333	0.075743	-0.036450	-
0.057642	-0.018154	0.009376	-0.029633	-0.070807	0.013970	-
0.106872	-0.135307	0.005187	-0.127860	0.053688	0.002637	
0.078659	0.058360	0.007521	-0.086601	-0.190375	-0.070079	
0.164035	0.015869	0.071757	0.043059	0.255611	-0.043493	
z19	-0.024348	-0.097936	0.001780	-0.132493	0.003229	-
0.055529	0.009994	0.063288	0.027616	-0.060952	-0.045251	
0.017268	-0.028723	-0.040569	-0.024590	-0.088822	0.062628	
0.110898	0.125245	-0.074276	-0.083186	-0.236874	-0.024717	-
0.127380	0.066762	-0.007310	0.221475	0.113937	0.020250	-
0.026262	0.039857	-0.047092	0.097589	-0.046842	-0.047187	
x20	-0.111522	-0.070495	0.026508	0.007954	0.092150	-
0.095474	0.019798	0.072896	0.094229	-0.046568	0.176197	
0.019245	-0.026289	-0.133889	-0.132373	-0.012549	-0.138987	
0.011082	-0.034019	-0.051333	0.022461	-0.009637	0.117857	
0.082680	0.044562	-0.038678	-0.052078	0.051020	0.059015	-
0.050829	0.025682	0.000795	-0.078831	0.069463	0.000075	
y20	0.062349	-0.044900	0.000743	0.037745	0.011936	-
0.106202	-0.049590	0.019015	0.010999	0.077299	0.002699	-
0.035780	-0.004551	-0.032505	-0.033188	-0.070891	-0.021593	-
0.084588	-0.003493	0.061420	-0.133395	0.016950	-0.001922	
0.056690	0.080421	-0.016929	0.114262	-0.265125	-0.085457	-
0.006745	-0.114775	-0.086039	0.117423	0.080238	0.005264	
z20	-0.029727	-0.097206	0.036168	-0.133195	-0.003257	-
0.039949	0.007341	0.050890	0.056084	-0.076210	-0.026302	
0.055910	0.006742	-0.009574	-0.043940	-0.088333	0.025517	
0.059489	0.154472	-0.004809	-0.032913	-0.091673	-0.028760	-
0.101973	0.017025	0.001024	0.202493	0.019115	-0.058385	
0.012982	-0.020266	-0.055349	-0.003783	-0.028553	0.025950	
x21	-0.101860	-0.044323	0.060971	0.029275	0.090275	-
0.051790	-0.000694	0.060881	0.073830	-0.016521	0.113337	
0.027653	-0.036514	-0.107793	-0.090214	-0.010132	-0.145536	-
0.113354	0.018307	-0.029243	0.051790	0.000108	0.017608	
0.074906	0.048610	0.058178	-0.070888	-0.010628	0.067466	
0.005227	0.038988	-0.055040	-0.036974	0.003021	-0.055255	
y21	0.055994	-0.041509	-0.001361	0.026962	0.005551	-
0.100350	-0.070688	0.044941	-0.011697	0.062887	0.005192	-
0.040270	-0.022040	-0.052182	-0.001972	-0.088559	-0.058810	-
0.040858	0.052019	0.037345	-0.099183	-0.028329	0.021084	
0.021826	-0.048679	-0.058033	0.145391	-0.095270	-0.128021	-
0.098861	-0.168060	0.031805	0.179941	0.048430	0.023936	
z21	-0.031188	-0.096270	0.064102	-0.126994	-0.014882	-
0.019477	0.002107	0.033688	0.073546	-0.085333	-0.026494	
0.075311	0.017975	0.026767	-0.038296	-0.101071	-0.013689	
0.032511	0.123850	0.016441	-0.015544	0.014927	0.020843	-
0.093133	-0.087724	0.024349	0.127783	0.001799	-0.066444	-
0.022270	-0.041364	0.089853	-0.006051	0.043681	0.047923	
x22	-0.091399	-0.022751	0.089044	0.048906	0.086287	-
0.010773	-0.022877	0.048479	0.061792	0.006281	0.056079	
0.037602	-0.047026	-0.083737	-0.044818	-0.013671	-0.139785	-
0.195966	0.059454	-0.021367	0.067204	-0.007347	-0.019132	
0.056480	0.022774	0.136795	-0.009864	-0.058444	0.060289	-
0.012289	0.015121	-0.071418	0.029437	-0.014931	-0.096115	

y22	0.049844	-0.038840	0.003346	0.014496	-0.004566	-
0.091162	-0.073030	0.072874	-0.034109	0.066330	0.024679	-
0.047250	-0.021597	-0.062098	0.022909	-0.066055	-0.113336	-
0.014400	0.079200	0.051091	-0.064164	-0.035588	-0.064001	
0.001003	-0.044218	-0.094991	0.013535	0.049801	-0.104685	-
0.070635	-0.093245	0.033200	0.091667	-0.087046	0.131996	
z22	-0.033222	-0.088979	0.087707	-0.120758	-0.025609	-
0.004642	0.008191	0.016255	0.085078	-0.079332	-0.017691	
0.085590	0.035637	0.058273	-0.035986	-0.088891	-0.044285	
0.005672	0.086481	0.042817	0.003334	0.103156	-0.000309	-
0.061132	-0.101828	0.030760	-0.022024	-0.013524	-0.075483	
0.058512	-0.041009	0.079445	-0.052345	0.026813	0.108966	
x23	-0.079879	-0.003325	0.110867	0.065564	0.079310	
0.023146	-0.038927	0.035808	0.052954	0.023077	0.005019	
0.039864	-0.063186	-0.073390	-0.001163	-0.020150	-0.114828	-
0.227446	0.068064	-0.014647	0.091196	-0.006647	-0.034966	
0.034606	-0.022503	0.163949	-0.007351	-0.024886	0.024498	-
0.017866	0.001234	-0.017847	0.086882	0.002864	0.068896	
y23	0.046010	-0.033353	0.014945	0.005877	-0.023068	-
0.087543	-0.085579	0.099933	-0.052545	0.058671	0.053004	-
0.049905	-0.008050	-0.029135	0.049997	-0.078526	-0.088332	-
0.019547	0.081715	0.067323	-0.040419	-0.098937	-0.124162	
0.007291	-0.101714	-0.127884	-0.033714	0.124969	-0.167419	
0.220778	0.116694	0.105908	-0.006401	-0.083565	0.191891	
z23	-0.034934	-0.077830	0.106484	-0.110925	-0.033039	
0.012419	0.011995	-0.005480	0.097491	-0.062341	-0.009471	
0.094423	0.055855	0.093663	-0.024037	-0.066473	-0.067410	-
0.022192	0.063886	0.046772	-0.007572	0.138995	-0.007688	-
0.034121	-0.068635	0.045128	-0.057648	-0.072220	-0.073625	
0.087063	-0.031941	0.014629	-0.107744	-0.024304	-0.089891	
x24	-0.068793	0.012209	0.128610	0.078758	0.072893	
0.053480	-0.045794	0.023979	0.052110	0.044727	-0.028957	
0.044096	-0.081342	-0.067413	0.025846	0.010831	-0.094934	-
0.226282	0.076303	-0.017198	0.075742	0.005605	-0.040799	-
0.021917	-0.033061	0.151553	0.057683	-0.059340	0.056910	-
0.061217	-0.024667	0.030561	0.081286	-0.038483	0.030841	
y24	0.042496	-0.031256	0.026392	-0.006371	-0.031046	-
0.054064	-0.094199	0.107899	-0.055604	0.075529	0.041713	-
0.026629	0.035465	-0.017184	0.074150	-0.037767	-0.137585	
0.004258	0.132980	0.057025	-0.042678	-0.058491	-0.124868	
0.070031	-0.009957	-0.046205	-0.056946	0.040365	-0.098913	-
0.001216	-0.019421	-0.112628	-0.008422	-0.108831	-0.167521	
z24	-0.034000	-0.059888	0.116169	-0.096957	-0.042905	
0.025889	0.016497	-0.028544	0.101286	-0.045390	-0.007389	
0.086960	0.070438	0.116967	0.006410	-0.065527	-0.064000	-
0.033117	0.013191	0.052438	0.008055	0.135070	-0.007523	
0.011759	-0.052108	0.053040	-0.118922	-0.014349	-0.123857	
0.153472	-0.017569	-0.036224	-0.082462	-0.008489	-0.017762	
x25	-0.054353	0.021186	0.141135	0.089593	0.064021	
0.078408	-0.043089	0.008890	0.053857	0.065593	-0.053513	
0.043522	-0.080959	-0.049546	0.063254	0.032408	-0.054846	-
0.193395	0.030552	-0.043061	0.044579	0.004146	-0.082619	-
0.014254	-0.030725	0.078540	0.078348	-0.040654	0.041732	
0.000637	-0.039275	0.120573	0.088473	-0.043778	-0.028010	
y25	0.040361	-0.028303	0.042518	-0.016078	-0.050511	-
0.025015	-0.106178	0.119074	-0.049412	0.078758	0.031583	-
0.015000	0.048528	-0.005529	0.089542	-0.013920	-0.127723	
0.021179	0.156810	0.108080	0.008330	-0.074683	-0.064903	
0.003364	0.017174	0.010313	-0.027820	-0.023841	-0.051325	-
0.121353	-0.017250	-0.254846	-0.078175	-0.065328	-0.054644	

z25	-0.032906	-0.036322	0.118658	-0.079825	-0.051993	
0.039591	0.020915	-0.055162	0.104680	-0.017019	-0.002129	
0.071533	0.067018	0.111490	0.026397	-0.056512	-0.055586	-
0.024978	-0.017192	0.062749	0.011825	0.111318	0.023606	
0.016459	-0.023389	0.045694	-0.127047	-0.012931	-0.108122	
0.086920	0.012560	-0.058899	-0.083070	-0.010408	0.017983	
x26	-0.042909	0.035500	0.148203	0.095850	0.054124	
0.095436	-0.043543	0.006673	0.063863	0.084921	-0.080540	
0.033441	-0.069831	-0.037554	0.093802	0.032907	0.004471	-
0.094639	0.020879	-0.008537	0.029692	-0.025455	-0.114912	-
0.018455	0.029781	0.008888	0.065704	-0.035848	-0.035040	-
0.004079	0.063927	0.028050	0.024515	-0.031216	-0.039292	
y26	0.039808	-0.025617	0.060901	-0.020922	-0.074606	
0.011471	-0.104005	0.114212	-0.045398	0.084475	0.039141	-
0.000292	0.053389	0.015784	0.114513	0.040584	-0.114089	
0.022169	0.087408	0.059667	0.025661	-0.055432	0.003887	-
0.051429	-0.018840	0.016254	0.028239	-0.007011	0.059642	-
0.090724	-0.095844	-0.027693	-0.097449	0.006073	0.009317	
z26	-0.029425	-0.011043	0.114747	-0.057962	-0.059344	
0.054676	0.032539	-0.088889	0.101509	0.018569	0.008043	
0.051239	0.064731	0.099695	0.066119	-0.016605	-0.055735	-
0.029603	-0.081674	0.028156	-0.010408	0.069267	0.053777	
0.036259	0.021589	0.007549	-0.065972	0.010326	-0.060089	
0.058980	-0.037696	-0.036543	0.017073	-0.054420	0.015605	
x27	-0.030769	0.044215	0.151726	0.101072	0.040902	
0.113079	-0.042428	0.005670	0.081364	0.098422	-0.104458	
0.026890	-0.070472	-0.017314	0.128532	0.050594	0.054846	
0.039201	-0.027679	-0.016583	0.029336	-0.062455	-0.133150	-
0.040665	0.080478	-0.048967	0.099069	0.018085	-0.099739	
0.030659	0.109218	-0.054122	-0.005569	-0.028495	0.075041	
y27	0.041846	-0.021442	0.080921	-0.026029	-0.101167	
0.046754	-0.097071	0.100166	-0.036461	0.096695	0.048053	
0.006869	0.077097	0.028077	0.134469	0.077037	-0.055838	
0.040476	0.031658	0.057380	0.003767	-0.035490	0.089888	-
0.061558	-0.027682	-0.049547	-0.006088	-0.041615	0.135328	-
0.076757	-0.030376	0.193425	-0.157594	0.106880	-0.029200	
z27	-0.024641	0.020514	0.102122	-0.035343	-0.065573	
0.067244	0.053515	-0.128679	0.098396	0.064053	0.015882	
0.020118	0.061024	0.074847	0.093350	0.017152	-0.044593	-
0.011494	-0.138089	0.016458	-0.038564	0.006829	0.116341	
0.046627	0.094439	-0.026882	-0.000354	-0.019061	0.012957	-
0.055991	-0.041277	-0.048442	0.091196	-0.053175	-0.005259	
x28	-0.018741	0.046031	0.152896	0.100356	0.027581	
0.120438	-0.051043	0.008478	0.096146	0.093307	-0.116370	
0.034325	-0.057623	-0.006757	0.159108	0.079848	0.133848	
0.171670	-0.061550	0.010326	0.012370	-0.085818	-0.070055	-
0.095600	0.112811	-0.121090	0.180133	0.105733	-0.151134	
0.004622	0.166434	0.031928	-0.111300	0.089588	0.044514	
y28	0.047184	-0.015415	0.100431	-0.027107	-0.130829	
0.094271	-0.077290	0.080089	-0.010889	0.130717	0.052788	-
0.003009	0.094647	0.052516	0.158838	0.125429	-0.009230	
0.121040	-0.053480	0.022406	-0.028396	-0.028354	0.119888	-
0.061754	0.011806	-0.075800	-0.063392	-0.104071	0.213135	-
0.069442	0.020236	0.195217	-0.189471	0.122148	-0.001175	
z28	-0.018540	0.057240	0.083001	-0.009576	-0.072221	
0.078768	0.079372	-0.173000	0.091354	0.115113	0.016842	-
0.020459	0.052767	0.044875	0.123831	0.046224	-0.026095	
0.007672	-0.207042	0.003102	-0.055379	-0.080371	0.187648	
0.061254	0.194882	-0.075411	0.052574	-0.030190	0.103567	-
0.171744	-0.072416	-0.103611	0.189366	-0.030005	0.061561	

x29	0.005848	0.045583	0.133665	0.091895	0.025227	
0.100573	-0.027450	0.040839	0.069171	0.039796	-0.118928	
0.013549	-0.087949	-0.004939	-0.053042	-0.067810	0.134850	
0.150777	-0.026273	0.053691	-0.126155	0.023946	-0.009352	-
0.077573	-0.079849	-0.095430	-0.028902	0.035492	0.047165	-
0.011855	0.042687	0.030226	-0.182161	-0.034724	0.004207	
y29	0.064448	-0.005605	0.108188	-0.012829	-0.160675	
0.144220	-0.026398	0.077709	-0.053760	0.105016	0.052315	-
0.049748	0.098336	0.043195	0.025443	0.058427	0.010124	
0.059954	-0.060746	-0.059708	0.071158	-0.009955	0.022868	
0.034192	0.057692	-0.031689	0.033547	0.017819	0.063606	
0.017758	-0.004267	-0.027949	0.103185	0.075677	-0.013240	
z29	-0.003709	0.090332	0.021982	0.031393	-0.058137	
0.043397	0.131250	-0.187497	0.013953	0.125970	0.046111	-
0.079259	-0.037809	-0.073941	0.050931	0.051236	-0.012261	
0.013176	-0.047786	-0.006819	-0.056700	-0.080800	0.014240	
0.064165	0.052183	-0.033985	0.055493	0.011556	-0.016945	-
0.014444	-0.023919	-0.001732	0.077214	0.002775	-0.026402	
x30	0.022316	0.040950	0.114389	0.077564	0.029044	
0.083041	-0.023278	0.071931	0.054122	-0.003689	-0.129672	
0.004915	-0.048806	0.045862	-0.161343	-0.175514	0.120388	
0.167029	0.023843	0.150944	-0.198802	0.063531	0.032415	
0.078151	-0.022937	-0.029957	-0.100960	0.018878	0.028141	
0.018370	-0.053676	-0.097396	0.080774	0.043311	-0.054845	
y30	0.085419	0.004306	0.108779	0.004378	-0.184357	
0.179390	0.037334	0.058499	-0.102093	0.075823	0.075290	-
0.104880	0.038711	-0.017714	-0.167601	-0.004349	0.071945	
0.007377	-0.065149	-0.146991	0.109462	0.067992	0.006822	-
0.012685	-0.101163	-0.016588	-0.016043	0.081157	0.050904	
0.038004	0.046356	-0.002663	0.007846	-0.067461	-0.007581	
z30	0.007653	0.120783	-0.035141	0.061754	-0.045434	
0.004146	0.176098	-0.200682	-0.045614	0.137129	0.056899	-
0.116833	-0.115357	-0.185200	-0.033163	0.035156	0.001960	
0.009691	0.096292	0.013170	-0.078633	-0.068669	-0.087580	
0.043591	-0.094437	-0.047460	0.014657	-0.050845	-0.015300	
0.012924	-0.002172	0.085277	-0.053476	0.084672	-0.001002	
x31	0.032293	0.025850	0.102322	0.064426	0.033274	
0.064415	-0.036261	0.082526	0.058766	-0.036888	-0.131616	
0.011680	-0.021157	0.079156	-0.213975	-0.246176	0.082290	
0.160191	0.040241	0.200833	-0.252442	0.077755	0.038171	
0.108041	-0.039415	-0.124851	-0.097480	-0.020284	0.118215	
0.022168	-0.268898	0.054198	0.045157	-0.034569	-0.008000	
y31	0.108080	0.016179	0.100807	0.019495	-0.195304	
0.207940	0.098339	0.045918	-0.152098	0.041689	0.091162	-
0.158180	-0.000430	-0.069494	-0.343807	-0.076319	0.144571	-
0.025556	-0.011474	-0.163457	0.135195	0.136571	0.002694	
0.084170	-0.131437	0.087973	-0.065119	0.179383	-0.091536	
0.059347	0.166326	-0.089955	0.172616	0.002427	0.003923	
z31	0.013913	0.144145	-0.082473	0.083140	-0.030342	-
0.036993	0.206196	-0.205776	-0.089480	0.147337	0.055554	-
0.128873	-0.163471	-0.255294	-0.078472	0.008608	0.005508	-
0.003958	0.197953	0.015389	-0.101703	-0.089079	-0.178353	-
0.013807	-0.163962	-0.087940	-0.036052	-0.091257	0.000541	
0.084019	-0.064346	0.005895	-0.221132	0.029247	-0.020679	
x32	0.023147	-0.125215	-0.050896	-0.065190	-0.033926	
0.179053	-0.024718	0.118633	0.017881	0.150793	0.097392	
0.140171	-0.016297	-0.161037	0.000414	0.158335	0.104136	-
0.030578	-0.009092	-0.070403	-0.156479	-0.129726	0.070783	-
0.063822	-0.165289	0.124328	-0.115374	0.021678	-0.155830	-
0.089700	-0.060812	-0.186076	-0.177910	-0.054397	-0.144424	

y32	-0.027667	-0.052476	-0.136496	-0.058885	-0.071813	-
0.036258	-0.307137	-0.145667	0.133566	-0.028236	-0.029688	-
0.061332	0.174450	-0.152911	-0.037154	0.188728	0.081326	-
0.066237	0.073236	0.135482	-0.078841	0.060397	0.103288	
0.031375	-0.040990	-0.020859	0.010602	0.066428	-0.099496	
0.038185	0.160624	-0.014935	0.209776	-0.041224	-0.028696	
z32	-0.075052	0.110974	0.090786	-0.190839	-0.095266	-
0.100118	0.027931	0.052908	0.032603	0.067508	-0.115009	
0.112701	-0.010980	0.017077	-0.031213	0.012654	-0.068282	-
0.209275	-0.103452	-0.126415	0.011352	-0.053938	0.015897	-
0.079547	-0.194580	-0.347104	-0.116107	-0.020940	-0.012564	
0.043414	0.018466	-0.032810	-0.058774	-0.044428	0.145253	
x33	0.027897	-0.114479	-0.046910	-0.055348	-0.026876	
0.173210	0.024931	0.104037	-0.005241	0.139805	0.096694	
0.146825	-0.024012	-0.087399	-0.021984	0.103330	0.063483	-
0.021609	-0.032102	-0.039376	-0.099169	-0.148171	0.072900	
0.007416	-0.112077	0.066243	-0.115960	-0.064553	-0.022226	
0.037596	-0.034034	0.000929	-0.034843	-0.062704	-0.062110	
y33	-0.046540	-0.048484	-0.140518	-0.052802	-0.079619	-
0.024719	-0.277292	-0.122543	0.107554	-0.004362	-0.012299	-
0.029692	0.105284	-0.114919	-0.052586	0.176395	0.052468	-
0.063552	0.054319	0.091701	-0.076391	0.065512	0.050012	-
0.059264	-0.014917	-0.011531	-0.020585	0.032209	-0.028013	-
0.019497	0.058692	0.019692	0.025406	0.010485	0.079446	
z33	-0.071609	0.087406	0.092744	-0.176841	-0.099969	-
0.075379	0.021703	0.030148	0.014741	0.055989	-0.073811	
0.082012	0.005990	0.030028	-0.042251	0.004684	-0.025981	-
0.132109	-0.090172	-0.069483	0.045446	-0.045303	0.055293	-
0.030014	-0.101873	-0.192230	-0.015065	0.004609	0.001128	
0.030027	0.050897	-0.036334	0.021082	-0.020017	-0.099917	
x34	0.034560	-0.102848	-0.042800	-0.049481	-0.019015	
0.159979	0.064356	0.093940	-0.018006	0.112943	0.087348	
0.128134	-0.019850	-0.035724	-0.023730	0.056942	0.001080	
0.005657	-0.024216	0.010154	-0.082108	-0.123956	0.015478	
0.005236	-0.019647	0.045542	-0.064787	0.049780	0.060691	
0.094356	-0.116563	0.054299	0.136981	-0.114524	0.180262	
y34	-0.065372	-0.046419	-0.140955	-0.038508	-0.093744	-
0.009268	-0.222547	-0.096480	0.057750	0.030511	0.016936	
0.008314	0.035715	-0.035768	-0.080367	0.117641	0.021319	-
0.065242	0.031274	0.027289	-0.025125	0.042034	0.013470	-
0.069705	0.018793	-0.006448	-0.033658	-0.012628	0.014765	-
0.011379	0.004889	0.046755	-0.066395	0.055520	0.021283	
z34	-0.067401	0.063144	0.091424	-0.160156	-0.101090	-
0.047832	0.014558	0.006299	-0.002230	0.045744	-0.028436	
0.043028	0.020337	0.031728	-0.052104	-0.003398	0.010568	-
0.040914	-0.060262	-0.011746	0.059895	-0.033536	0.048953	
0.023258	-0.012575	-0.017760	0.053187	0.046109	0.001287	
0.017518	0.001104	-0.013836	0.082472	-0.034947	-0.162501	
x35	0.044762	-0.086365	-0.038567	-0.044644	-0.005143	
0.139594	0.094244	0.070899	-0.031337	0.090467	0.057338	
0.098531	-0.016620	0.002336	-0.002376	0.005592	-0.062115	
0.007552	-0.026663	0.001297	-0.066453	-0.074901	-0.045641	-
0.018809	0.058531	-0.030161	-0.055968	-0.011977	0.082871	
0.079400	-0.001782	0.056852	0.242538	0.065375	0.137014	
y35	-0.084048	-0.047478	-0.138214	-0.018201	-0.114704	
0.007825	-0.147387	-0.065376	-0.008061	0.062976	0.054754	
0.045285	-0.038644	0.073427	-0.117926	0.024494	-0.023804	-
0.052354	0.019434	-0.029324	0.035152	0.003861	-0.045007	-
0.043704	0.096639	0.001559	-0.002968	0.060599	0.061638	
0.024776	-0.122173	0.047248	-0.062908	-0.021632	0.025046	

z35	-0.062524	0.037447	0.085882	-0.138959	-0.097649	-
0.018929	0.007280	-0.012376	-0.019842	0.039755	0.022179	
0.004568	0.032091	0.011242	-0.057089	-0.005366	0.049527	
0.042677	-0.017125	0.026887	0.057782	-0.036113	0.026708	
0.077160	0.031824	0.163211	0.126262	0.021105	-0.019462	
0.019676	-0.004059	0.027262	0.026247	0.041831	-0.110380	
x36	0.056949	-0.067692	-0.033003	-0.041792	0.024106	
0.121330	0.104296	0.050956	-0.016340	0.047669	0.045126	
0.036554	0.006293	0.019420	0.006199	0.002297	-0.102551	-
0.013977	-0.024743	0.054299	-0.053552	0.014083	-0.074195	-
0.068888	0.093223	-0.044195	0.049068	0.037314	0.023262	-
0.005032	0.002883	0.073351	0.172060	0.231073	-0.057150	
y36	-0.101246	-0.049349	-0.132597	0.007114	-0.136209	
0.023656	-0.056884	-0.035586	-0.089299	0.097789	0.090385	
0.083183	-0.123824	0.203853	-0.159062	-0.094665	-0.076756	-
0.029571	0.001659	-0.108762	0.120425	-0.024225	-0.140971	-
0.000191	0.196029	-0.018574	0.063291	0.103149	0.091255	
0.044348	-0.201801	0.072353	-0.074259	0.010511	-0.012374	
z36	-0.056950	0.010644	0.074619	-0.114913	-0.086835	
0.011634	-0.001179	-0.022378	-0.035719	0.032684	0.073582	-
0.034945	0.042712	-0.024430	-0.063952	0.001359	0.095625	
0.117786	0.042787	0.070850	0.024144	-0.051755	-0.001070	
0.137362	0.060628	0.356063	0.203429	-0.059353	-0.018189	
0.037859	-0.008855	0.072178	-0.081626	0.101536	0.146119	
x37	0.071839	-0.044253	-0.034273	-0.047601	0.049052	
0.115457	0.088822	0.043466	0.011106	0.020894	0.003812	
0.000756	0.058899	0.007607	-0.006008	-0.007833	-0.086225	-
0.034190	0.049659	0.014520	-0.015925	0.103496	-0.074401	
0.073729	0.098938	-0.031669	-0.071460	-0.157895	-0.042008	
0.042840	0.233061	0.131709	0.039176	-0.020152	-0.078881	
y37	-0.105497	-0.051962	-0.129570	0.020296	-0.109774	
0.023311	-0.004520	-0.007581	-0.059051	0.058161	0.055918	
0.067833	-0.103737	0.175536	-0.062950	-0.106180	-0.065248	
0.010283	-0.004263	-0.095373	0.071112	0.008732	-0.115795	
0.036148	0.110797	-0.038496	0.040475	-0.004664	0.036999	-
0.019402	-0.026043	0.013933	-0.020654	-0.025788	-0.047220	
z37	-0.047338	-0.023980	0.064160	-0.083467	-0.054833	
0.034524	0.002317	-0.040645	-0.041368	0.000057	0.097055	-
0.062366	0.020739	-0.033923	-0.001579	0.004340	0.044154	
0.113274	0.068703	0.067454	-0.016990	0.026631	-0.031873	
0.115294	0.065565	0.211534	0.069204	-0.063956	0.019921	
0.003929	0.053248	0.035970	-0.094273	-0.023039	0.104449	
x38	0.086120	-0.022881	-0.033029	-0.053025	0.064668	
0.100335	0.064626	0.039687	0.023594	0.012485	0.001037	-
0.025174	0.074992	0.006055	-0.025249	0.010514	-0.072971	-
0.017522	0.031370	0.099671	0.048682	0.135106	-0.071448	-
0.015545	0.104869	-0.086718	0.083966	-0.127598	-0.151557	
0.005857	0.238018	0.130269	-0.124851	0.036123	-0.034375	
y38	-0.106542	-0.052997	-0.125709	0.029868	-0.082417	
0.023843	0.038893	0.019149	-0.027375	0.025819	0.028067	
0.052936	-0.088560	0.148676	0.015299	-0.115880	-0.058905	
0.053646	-0.009419	-0.062127	0.038539	0.041105	-0.098506	
0.028528	0.033986	-0.034029	0.027010	-0.064445	-0.023592	-
0.062074	0.108287	-0.015769	-0.044842	-0.024721	-0.061202	
z38	-0.037333	-0.052399	0.054018	-0.049892	-0.029539	
0.053558	0.001583	-0.059414	-0.049342	-0.027387	0.116277	-
0.078372	0.000619	-0.050158	0.050135	0.005750	0.007286	
0.110164	0.084356	0.094718	-0.036572	0.071546	-0.049756	
0.057861	0.061180	0.091604	0.011972	-0.055470	-0.008961	-
0.045585	0.050500	0.014792	-0.018167	0.035352	0.043836	

x39	0.099237	0.001428	-0.031029	-0.069503	0.066183		
0.080935	0.034875	0.021756	0.023224	0.016819	-0.010133	-	
0.066781	0.104752	-0.023467	-0.040228	0.022447	-0.009375	-	
0.058085	0.018714	0.052620	0.109238	0.158674	-0.051110		
0.067694	0.032896	-0.104471	0.148824	-0.062353	-0.014857		
0.042677	0.057822	0.051043	-0.007380	-0.021972	0.205007		
y39	-0.103309	-0.051868	-0.121428	0.031394	-0.058647		
0.026576	0.073395	0.038746	-0.006318	0.005240	0.003780		
0.032400	-0.070603	0.111565	0.077309	-0.111135	-0.039119		
0.067338	-0.022145	-0.047676	0.038450	0.057336	-0.074349		
0.057127	-0.068392	-0.020488	0.026329	-0.098062	-0.033243	-	
0.078820	0.121635	-0.028161	-0.003517	-0.016396	0.106439		
z39	-0.027870	-0.074182	0.043069	-0.021227	-0.009379		
0.071672	-0.002499	-0.069035	-0.055891	-0.050057	0.122039	-	
0.095377	-0.018274	-0.055844	0.077693	0.011815	-0.005043		
0.085174	0.082070	0.073060	-0.048014	0.105623	-0.058178		
0.019925	0.025502	-0.012361	-0.040633	-0.038077	0.031713	-	
0.037111	0.007399	-0.012908	0.040030	0.005172	0.065386		
x40	0.112167	0.030873	-0.025245	-0.080751	0.069950		
0.057358	0.005314	0.006298	0.027901	0.015390	-0.025898	-	
0.115031	0.105934	-0.021829	-0.061833	0.026586	0.013091	-	
0.052526	0.017408	0.028673	0.131028	0.184584	-0.046014		
0.022409	0.053283	-0.115154	0.126451	0.061234	0.026233	-	
0.089848	-0.013689	-0.150082	-0.151250	-0.100497	0.063280		
y40	-0.095508	-0.047671	-0.114663	0.029152	-0.033571		
0.028923	0.097419	0.054969	0.013686	-0.011249	-0.023737	-	
0.000536	-0.056670	0.075339	0.113949	-0.095370	-0.020104		
0.081331	-0.020753	-0.038420	0.034353	0.074796	-0.035065		
0.056252	-0.105961	-0.018767	0.032195	-0.079497	-0.044717	-	
0.131119	0.160006	-0.099355	-0.007823	-0.017743	0.048511		
z40	-0.019864	-0.086458	0.032249	0.005519	0.009432		
0.087991	-0.009532	-0.072166	-0.055892	-0.068260	0.115361	-	
0.113661	-0.035403	-0.049794	0.087360	0.011742	-0.018303		
0.065735	0.083523	0.050231	-0.043920	0.122459	-0.055741	-	
0.031033	0.039896	-0.083045	-0.099600	0.002973	0.032682	-	
0.073983	-0.002767	-0.073537	0.070489	-0.043773	-0.058888		
x41	0.125383	0.058463	-0.025126	-0.093865	0.078226		
0.035948	-0.018944	-0.019583	0.027266	0.019844	-0.027414	-	
0.123358	0.112412	-0.004196	-0.067274	0.010221	-0.020904	-	
0.085300	-0.008760	0.027867	0.071725	0.128510	-0.015626		
0.066393	0.044545	-0.106177	0.193174	0.050112	0.096483	-	
0.046817	-0.123026	-0.104004	-0.038949	-0.048747	-0.086335		
y41	-0.084338	-0.039621	-0.110763	0.018674	-0.003418		
0.031592	0.122292	0.062324	0.033213	-0.019677	-0.051497	-	
0.025983	-0.026078	0.045267	0.144397	-0.078584	-0.051378		
0.052433	-0.031991	-0.024731	-0.002221	0.064708	0.011458		
0.093218	-0.145041	-0.001247	0.077983	-0.052335	-0.001419	-	
0.109204	0.082519	-0.098858	0.069562	0.018873	-0.034145		
z41	-0.011265	-0.092663	0.018966	0.028451	0.031173		
0.100491	-0.010451	-0.073034	-0.049627	-0.087534	0.095850	-	
0.117110	-0.033289	-0.027470	0.099911	-0.000788	-0.060495		
0.021362	0.065411	0.031252	-0.084608	0.101756	-0.048759	-	
0.041748	0.027189	-0.106817	-0.089331	0.071274	0.077873	-	
0.053567	-0.042364	-0.056047	0.097140	-0.055796	-0.077674		
x42	0.138486	0.084444	-0.022045	-0.105213	0.078372		
0.010205	-0.051147	-0.044567	0.032617	0.031927	-0.022448	-	
0.120855	0.083876	0.019651	-0.067319	-0.001403	-0.057243	-	
0.060606	-0.015584	-0.008723	0.055502	0.096333	-0.064231	-	
0.009928	-0.001765	-0.042534	0.103181	0.106950	0.130342	-	
0.027401	-0.088966	-0.039955	-0.159058	0.008492	-0.079850		

y42	-0.070132	-0.029169	-0.106719	0.002521	0.024405	
0.033484	0.133145	0.070206	0.057502	-0.013991	-0.079912	-
0.048131	-0.006005	0.016984	0.158041	-0.047081	-0.080298	
0.040064	-0.020871	-0.039965	-0.024022	0.074653	0.014398	
0.051288	-0.185687	0.043090	0.060882	0.040648	0.063813	-
0.059002	0.008125	-0.027492	-0.032253	0.060990	-0.037284	
z42	-0.002548	-0.094094	0.006223	0.050310	0.048536	
0.111912	-0.018921	-0.064454	-0.039864	-0.102705	0.070890	-
0.118410	-0.033808	-0.001726	0.097697	-0.005689	-0.083579	-
0.026711	0.050600	-0.019773	-0.093377	0.083436	-0.040915	-
0.088441	0.006043	-0.105567	-0.105060	0.147926	0.089213	-
0.040934	-0.038062	0.004672	0.019955	-0.044357	-0.042431	
x43	0.149741	0.108555	-0.016424	-0.109484	0.072182	-
0.010347	-0.083668	-0.071883	0.026508	0.037073	-0.004261	-
0.101419	0.036418	0.039053	-0.077869	-0.042583	-0.064706	-
0.008057	-0.025324	-0.006507	0.078661	0.023078	-0.057514	-
0.052890	-0.026283	0.028676	0.054346	0.072150	0.067474	-
0.024462	-0.017104	-0.002388	-0.064573	-0.003771	0.022632	
y43	-0.053622	-0.017173	-0.100829	-0.013389	0.048014	
0.045384	0.135950	0.080790	0.072355	-0.003944	-0.103204	-
0.078253	0.003427	-0.030537	0.122352	-0.024540	-0.058751	
0.029729	0.010169	-0.027399	0.036734	0.053371	0.057610	
0.014038	-0.144536	0.084862	0.060819	0.052587	0.045736	
0.012933	-0.041784	-0.002524	-0.002519	0.064648	-0.042891	
z43	0.005454	-0.090236	-0.005117	0.075348	0.058476	
0.128405	-0.032625	-0.047113	-0.030493	-0.110053	0.041738	-
0.124366	-0.041417	0.019293	0.056534	-0.025181	-0.057588	-
0.056314	0.040292	-0.052425	-0.045488	0.029365	0.001206	-
0.130256	0.045323	-0.076881	-0.093648	0.128209	0.001420	-
0.022288	-0.035552	0.034355	0.026311	-0.008184	-0.020736	
x44	0.159161	0.130142	-0.009350	-0.111192	0.060910	-
0.037237	-0.127481	-0.108762	0.010901	0.041150	0.036107	-
0.067320	-0.013269	0.075429	-0.066339	-0.105384	-0.064412	
0.045825	-0.057226	-0.000520	0.046966	-0.071317	-0.023184	-
0.051146	-0.091648	0.050546	0.008347	-0.001938	0.006319	-
0.084559	0.038548	0.003336	-0.011815	-0.004262	-0.004861	
y44	-0.035631	-0.005012	-0.092981	-0.031367	0.068170	
0.054832	0.116217	0.083271	0.082642	0.011894	-0.107158	-
0.110917	0.021232	-0.070391	0.079332	-0.019892	0.007875	
0.014365	0.024125	-0.018821	0.071315	0.007983	0.141777	
0.016830	-0.103269	0.031848	0.085476	-0.008649	0.000289	
0.082132	-0.106037	0.011726	-0.038146	0.052538	-0.084757	
z44	0.013601	-0.082332	-0.017247	0.099492	0.063772	
0.141364	-0.053420	-0.024276	-0.019340	-0.115948	0.006218	-
0.127636	-0.041313	0.050187	0.002336	-0.044107	-0.012273	-
0.099427	0.018913	-0.092756	0.002137	-0.045085	0.059270	-
0.159733	0.071641	-0.035933	-0.049025	0.057199	-0.102709	-
0.007366	-0.064586	0.039584	-0.032896	0.077628	-0.019859	
x45	0.167658	0.151519	-0.000868	-0.113248	0.046048	-
0.069366	-0.166470	-0.150941	-0.008429	0.042957	0.085183	-
0.014471	-0.078248	0.130282	-0.036931	-0.168627	-0.104753	
0.117064	-0.089008	0.015236	-0.007543	-0.154851	-0.018025	-
0.068011	-0.197249	0.128011	-0.061963	-0.020773	-0.017070	-
0.186804	0.117931	0.069179	0.124963	0.008800	-0.063082	
y45	-0.014365	0.009152	-0.084774	-0.056049	0.088402	
0.054987	0.098775	0.075215	0.086457	0.020634	-0.100497	-
0.125823	0.044425	-0.097277	0.054084	-0.005003	0.028812	
0.002647	0.037339	0.003015	0.073918	-0.007320	0.198183	
0.027345	-0.118775	0.027928	0.108082	0.049972	0.011018	
0.087968	-0.138642	0.058479	0.034206	-0.020635	-0.174982	

z45	0.021273	-0.066081	-0.030055	0.119221	0.068512	
0.146428	-0.063716	-0.000689	-0.004513	-0.124037	-0.035951	-
0.110866	-0.029879	0.094297	-0.042461	-0.066891	0.009892	-
0.140175	-0.014423	-0.133493	0.033508	-0.109677	0.093655	-
0.188371	0.067924	0.074673	0.016888	0.038477	-0.149501	-
0.007413	-0.076547	0.082847	-0.072497	0.137746	-0.025420	
x46	0.156498	0.128097	0.011647	-0.088341	0.017982	-
0.081069	-0.130199	-0.093574	-0.029468	-0.000556	0.083955	
0.023863	-0.100530	0.103207	0.031575	-0.090274	-0.052853	
0.100047	-0.008147	-0.060053	-0.004105	-0.058839	0.018666	-
0.016269	-0.109185	0.051939	-0.037200	-0.023179	-0.021705	-
0.112768	0.046564	0.017550	0.061142	0.027290	-0.008232	
y46	0.011303	0.038792	-0.084242	-0.079256	0.110386	
0.051763	0.048947	0.029126	0.088533	0.029006	-0.065417	-
0.108675	0.035389	-0.066051	0.041749	-0.082017	0.033415	
0.037131	0.012309	-0.015773	0.100631	-0.062315	0.137628	-
0.011237	-0.057470	-0.047234	-0.032866	0.024590	-0.024451	
0.094387	-0.134028	0.063380	0.055032	-0.010972	0.089684	
z46	0.032353	-0.041670	-0.037834	0.132931	0.066081	
0.133217	-0.101563	0.000099	-0.017546	-0.127617	-0.028975	-
0.068966	-0.011169	0.099022	-0.065738	-0.015245	-0.019691	-
0.065072	-0.018386	-0.077661	0.031006	-0.125206	0.100662	-
0.066239	-0.021422	0.005108	0.026772	-0.044123	-0.076877	-
0.025925	-0.029131	0.030878	-0.084143	0.098311	0.085556	
x47	0.143524	0.102053	0.022707	-0.065243	-0.010442	-
0.091839	-0.089314	-0.037740	-0.048248	-0.040411	0.074753	
0.047956	-0.111927	0.073197	0.092030	-0.011955	-0.001840	
0.070216	0.060005	-0.118340	-0.012426	0.021476	0.047096	
0.030745	-0.037937	0.022928	-0.009213	-0.018044	-0.036058	-
0.015528	-0.000668	-0.013353	0.013308	0.060753	-0.027876	
y47	0.030418	0.058523	-0.080306	-0.099120	0.124841	
0.055880	0.028782	-0.002594	0.083910	0.029205	-0.037635	-
0.078529	0.015994	-0.039328	0.025393	-0.093020	0.051292	
0.045013	-0.008078	-0.044355	0.070506	-0.102682	0.081240	-
0.029110	0.039895	0.001325	-0.058758	-0.049713	-0.088665	
0.059698	0.014156	-0.009043	-0.013368	-0.031771	0.051425	
z47	0.037157	-0.014687	-0.045415	0.142230	0.062215	
0.118197	-0.118210	0.014727	-0.027771	-0.120643	-0.024102	-
0.028209	0.002718	0.093271	-0.078440	0.033847	-0.032627	
0.000334	-0.017970	-0.056092	0.034405	-0.115578	0.090692	
0.024815	-0.066090	-0.016434	0.033974	-0.118812	-0.043660	-
0.002709	0.039690	-0.052522	-0.071574	0.021603	0.105061	
x48	0.131129	0.076451	0.031394	-0.042996	-0.036778	-
0.101854	-0.050513	0.006579	-0.064622	-0.073427	0.060701	
0.059751	-0.111515	0.044591	0.136824	0.048778	0.036420	
0.044215	0.109950	-0.153133	-0.013583	0.091088	0.069940	
0.063297	0.017981	-0.007089	0.010702	0.008175	-0.028451	
0.053274	-0.039330	-0.015640	0.005689	0.050275	-0.037259	
y48	0.045870	0.070442	-0.072336	-0.111661	0.130698	
0.054547	0.014278	-0.018477	0.064904	0.009542	-0.010058	-
0.047018	0.015710	-0.022716	0.023073	-0.096205	0.074704	
0.028239	-0.039942	-0.055987	0.052074	-0.130368	0.019487	-
0.007816	0.082541	0.006447	-0.151772	-0.147023	-0.002913	
0.056771	-0.032932	0.052173	-0.071577	-0.011489	0.066078	
z48	0.041602	0.005335	-0.050893	0.148333	0.055970	
0.100529	-0.131778	0.033227	-0.036347	-0.113922	-0.017654	
0.001429	0.021325	0.083483	-0.074029	0.063423	-0.031759	
0.031931	-0.019190	-0.042471	0.031625	-0.102999	0.065353	
0.105538	-0.096411	-0.042504	0.019474	-0.166496	0.023377	
0.013303	0.018690	-0.096335	-0.060499	-0.007973	0.039053	

x49	0.119318	0.051854	0.037654	-0.021519	-0.062413	-
0.110244	-0.015817	0.037571	-0.076601	-0.096274	0.045052	
0.055335	-0.097031	0.023773	0.163247	0.084619	0.054634	
0.016487	0.136544	-0.165035	-0.018875	0.114764	0.087819	
0.081067	0.035935	-0.016521	0.013509	0.035493	-0.032379	
0.091159	-0.063421	-0.001736	-0.014215	0.039648	-0.015024	
y49	0.059388	0.077308	-0.067918	-0.120640	0.133468	
0.053315	0.012403	-0.016714	0.052391	-0.019913	0.002594	-
0.006941	0.001368	-0.029281	0.031031	-0.068725	0.093523	
0.047521	0.007737	-0.061329	0.086780	-0.088858	0.021139	-
0.008591	0.163242	0.033923	-0.167880	-0.103976	-0.000320	
0.052041	0.015012	-0.046970	0.097926	-0.054123	0.057089	
z49	0.043998	0.022598	-0.057942	0.149595	0.049029	
0.078349	-0.132201	0.058762	-0.037636	-0.096316	-0.015022	
0.031767	0.033325	0.057910	-0.063054	0.080079	-0.002542	
0.069616	0.000699	-0.040037	0.050199	-0.063788	0.036562	
0.144369	-0.069824	-0.043330	0.043772	-0.153646	0.069419	
0.053976	0.070993	-0.104906	0.038002	-0.053945	0.000582	
x50	0.107555	0.027027	0.040906	0.001912	-0.090130	-
0.114274	0.013764	0.060799	-0.090323	-0.111232	0.032535	
0.033416	-0.069844	0.004551	0.144356	0.089630	0.080329	
0.008848	0.128914	-0.157521	0.006874	0.119158	0.103634	
0.069193	0.041259	-0.043772	0.021088	0.005505	-0.033871	
0.105601	-0.067983	-0.022218	-0.001300	0.038750	-0.042056	
y50	0.072857	0.083833	-0.066195	-0.133122	0.150664	
0.031923	0.053163	-0.043438	0.056861	-0.069097	-0.010423	
0.070846	0.025778	0.014216	0.154296	-0.039786	-0.073408	-
0.009430	-0.005682	0.013442	-0.069174	-0.089321	-0.134578	
0.049935	0.111508	0.154503	-0.131114	0.211179	0.111286	
0.065817	0.029674	-0.016442	-0.010445	0.045460	0.131907	
z50	0.046552	0.040638	-0.068775	0.141365	0.057727	
0.034876	-0.101740	0.065036	-0.024725	-0.092315	-0.026044	
0.091674	0.071855	0.056287	0.034410	0.081511	-0.111709	
0.051673	-0.035300	0.007402	-0.059697	-0.039156	-0.057374	
0.186683	-0.151373	0.050257	0.079076	0.119058	0.169542	
0.114390	0.069115	0.014658	0.042312	-0.004924	0.054383	
x51	0.096572	0.002971	0.039967	0.020403	-0.111280	-
0.121349	0.055786	0.063004	-0.095607	-0.123405	0.005597	
0.014324	-0.013610	-0.005522	0.138006	0.073602	0.036090	-
0.026863	0.071487	-0.103885	-0.026243	0.069175	0.065910	
0.041765	-0.008672	-0.020453	0.019988	0.028398	0.029368	
0.073457	-0.077957	0.040788	-0.017300	0.066020	0.093224	
y51	0.078723	0.076793	-0.052608	-0.117691	0.121796	
0.055502	0.036619	-0.012041	0.008460	-0.078275	0.031403	
0.065068	-0.043519	-0.006294	0.028663	0.028155	0.096592	
0.022851	0.060789	-0.033648	0.061771	-0.023621	0.001087	-
0.029972	0.223587	0.028912	-0.173702	-0.014863	-0.098881	
0.057407	0.013452	-0.119507	0.016990	-0.131277	-0.100553	
z51	0.043895	0.051200	-0.071028	0.144697	0.033232	
0.025422	-0.113792	0.115503	-0.035082	-0.034684	0.000798	
0.077226	0.045962	0.011626	-0.041765	0.070605	0.063338	
0.077693	0.007406	-0.000544	0.075575	-0.006890	0.002433	
0.124632	0.005266	-0.017189	0.030041	-0.093700	0.004740	
0.060096	0.053219	-0.019708	0.075708	-0.155382	-0.070078	
x52	0.084093	-0.023986	0.042210	0.045954	-0.143437	-
0.111037	0.076943	0.085351	-0.102938	-0.108788	-0.004606	-
0.047840	0.022656	-0.020958	0.042534	0.031159	0.093545	-
0.016595	0.045353	-0.040691	0.083243	-0.000219	0.097389	-
0.055371	0.085471	-0.033159	-0.044531	-0.104782	-0.059604	-
0.027240	-0.032204	-0.023052	0.018859	-0.063843	0.076972	

y52	0.086013	0.069897	-0.050929	-0.112778	0.115041	
0.043690	0.082660	-0.037837	-0.029528	-0.132237	0.024727	
0.132380	-0.018331	0.008845	0.054112	0.086645	-0.011710	-
0.042378	-0.009859	-0.002228	-0.103963	0.015153	-0.073572	-
0.001370	0.023382	0.050638	-0.038285	0.090391	0.023926	
0.105202	-0.052004	0.005515	-0.003678	0.051478	0.005011	
z52	0.042899	0.063522	-0.081991	0.131652	0.033724	-
0.022168	-0.071707	0.125122	-0.018758	-0.009230	-0.004318	
0.119345	0.076648	-0.004545	0.023239	0.029001	0.002779	
0.045734	-0.046619	0.029029	-0.015163	0.029711	-0.059937	
0.105784	-0.052195	0.038855	0.048142	0.135334	0.068218	
0.077455	-0.001744	0.100804	0.046188	-0.031993	-0.106113	
x53	0.072538	-0.050424	0.039294	0.068252	-0.166227	-
0.109860	0.106196	0.084971	-0.108192	-0.108219	-0.025549	-
0.107073	0.075098	-0.029329	-0.019160	-0.039345	0.049534	-
0.054704	-0.020047	0.039332	0.089248	-0.064556	0.024727	-
0.137888	0.070853	0.012088	-0.050491	-0.158613	-0.097605	-
0.106040	-0.080996	-0.054437	0.052214	0.011809	0.112432	
y53	0.088533	0.054717	-0.044063	-0.093793	0.085855	
0.053245	0.117586	-0.025379	-0.070700	-0.156170	0.029490	
0.162319	0.004023	0.016974	-0.023399	0.142159	0.024832	-
0.040741	-0.027927	0.075167	-0.076171	0.010112	-0.027864	-
0.054574	0.007765	0.005499	0.051670	0.009155	-0.009029	
0.017876	0.041617	-0.028074	-0.013330	0.010980	-0.018183	
z53	0.038413	0.071269	-0.089068	0.121385	0.018974	-
0.054622	-0.040700	0.160939	-0.009560	0.051498	0.007740	
0.119724	0.076568	-0.042214	0.011969	-0.031449	0.084690	
0.029380	-0.055768	0.047576	0.029278	0.061198	-0.045333	
0.005024	0.041570	0.038847	0.001860	0.147634	-0.020120	
0.000858	-0.074107	0.115332	0.049854	-0.013365	-0.111734	
x54	0.061700	-0.069826	0.029531	0.079119	-0.165190	-
0.123439	0.149446	0.051406	-0.096484	-0.129732	-0.074272	-
0.143236	0.176505	-0.003538	0.020473	-0.033145	-0.113474	-
0.091800	-0.133347	0.153039	-0.028597	-0.134832	-0.038986	-
0.143966	-0.098476	0.159836	0.017608	0.133848	0.052789	-
0.000920	-0.000251	-0.089289	0.028320	0.039301	0.078947	
y54	0.089474	0.029851	-0.037391	-0.064052	0.045090	
0.063537	0.170365	-0.001320	-0.115126	-0.176729	0.034800	
0.184511	0.028342	0.014114	-0.134313	0.125036	0.019772	-
0.075655	-0.043380	0.184016	-0.066314	0.001669	-0.046143	-
0.118257	-0.001307	-0.051338	0.153755	-0.142792	-0.080590	-
0.172281	-0.068397	-0.069644	0.037193	0.166324	-0.097697	
z54	0.030886	0.073683	-0.092545	0.108789	0.000183	-
0.084096	-0.004438	0.200868	0.002217	0.125665	0.018666	
0.104097	0.070143	-0.089129	-0.016888	-0.120437	0.199422	-
0.004831	-0.052848	0.066780	0.084339	0.090572	-0.053560	-
0.132511	0.197677	0.066039	-0.032092	0.040168	-0.154394	-
0.143154	-0.094341	0.125170	-0.017456	-0.016935	-0.086906	
x55	0.056059	-0.074510	0.024861	0.077172	-0.159024	-
0.135107	0.072983	0.024093	-0.073101	-0.109847	-0.113077	-
0.234977	0.215996	-0.001753	0.069034	-0.069074	-0.151734	
0.013074	-0.118972	0.138891	0.017348	-0.295820	-0.018468	
0.031336	0.137346	0.044137	-0.133605	-0.017827	0.052184	
0.190781	0.137074	0.059265	-0.092199	-0.128414	-0.194872	
y55	0.098126	0.018925	-0.015339	-0.049611	0.017212	
0.027094	0.184109	-0.012124	-0.153746	-0.239697	-0.002849	
0.168614	-0.004514	0.025364	-0.150729	0.154557	-0.003055	-
0.121230	-0.108242	0.118595	-0.135849	-0.053044	-0.039758	-
0.008656	-0.057680	-0.011680	0.029507	-0.043603	0.066726	-
0.166979	0.086747	0.196938	-0.064196	-0.216241	0.038544	

```
   z55      0.021936   0.097732  -0.106271   0.088569   0.015141  -
0.120983    0.013593   0.205240   0.012496   0.147626  -0.019345
0.102417    0.082632  -0.070641   0.032029  -0.118253   0.137960  -
0.035426    0.026864   0.116012   0.057463   0.165964  -0.041289  -
0.239331    0.035392   0.036261  -0.182095   0.093827   0.162197  -
0.139744    0.158216  -0.136201   0.020199   0.113270   0.180662
```

	Eigenvalues	% Variance	Cumulative %
1.	0.00135409	16.268	16.268
2.	0.00098794	11.869	28.138
3.	0.00074144	8.908	37.045
4.	0.00063923	7.680	44.725
5.	0.00053060	6.375	51.100
6.	0.00049512	5.948	57.048
7.	0.00045969	5.523	62.571
8.	0.00039896	4.793	67.365
9.	0.00036897	4.433	71.797
10.	0.00028589	3.435	75.232
11.	0.00027046	3.249	78.482
12.	0.00024860	2.987	81.468
13.	0.00023376	2.808	84.277
14.	0.00019938	2.395	86.672
15.	0.00018538	2.227	88.899
16.	0.00017293	2.078	90.977
17.	0.00012651	1.520	92.497
18.	0.00012325	1.481	93.978
19.	0.00011175	1.343	95.320
20.	0.00010724	1.288	96.609
21.	0.00008507	1.022	97.631
22.	0.00007953	0.956	98.586
23.	0.00006070	0.729	99.316
24.	0.00005697	0.684	100.000

Canonical Variate Analysis: CVA ...

Dataset: first turn vs second turn orientation
Classification criterion: subfam

Groups		Observations
1.	Ca	9
2.	Ce	13
3.	Di	3
4.	Pr	11

Variation among groups, scaled by the inverse of the within-group variation

	Eigenvalues	% Variance	Cumulative %
1.	8.21075762	55.891	55.891
2.	4.18698023	28.501	84.392
3.	2.29287630	15.608	100.000

Mahalanobis distances among groups:

	Ca	Ce	Di
Ce	5.0871		
Di	6.7840	8.0333	
Pr	5.8391	6.2469	5.9810

P-values from permutation tests (10000 permutation rounds) for Mahalanobis distances among groups:

	Ca	Ce	Di
Ce	<.0001		
Di	0.0001	<.0001	
Pr	<.0001	<.0001	<.0001

Procrustes distances among groups:

	Ca	Ce	Di
Ce	0.1053		
Di	0.0695	0.1283	
Pr	0.0599	0.1386	0.0806

P-values from permutation tests (10000 permutation rounds) for Procrustes distances among groups:

	Ca	Ce	Di
Ce	0.0193		
Di	0.7015	0.0288	
Pr	0.7519	0.0009	0.5166

Canonical coefficients:

	CV1	CV2	CV3
x1	-130.0787	-79.3512	107.6012
y1	-66.9253	-6.6480	53.1733
z1	-132.1383	33.4085	68.5139
x2	13.6696	62.0136	6.8111
y2	46.7546	15.3819	16.7539
z2	-42.2318	37.3581	23.1299
x3	53.7914	113.2982	-48.0940
y3	51.7787	21.1952	-26.6513
z3	49.1997	38.4915	-9.8036
x4	68.6924	63.9603	-42.8718
y4	6.0521	-43.9065	-11.7000
z4	80.3234	-1.7815	-15.9950
x5	71.3602	46.9228	-48.2837
y5	-3.0629	-53.8500	-27.5210
z5	84.0647	-53.3319	1.0146
x6	39.6221	-13.3667	-20.1191
y6	-2.0735	-22.0567	-29.9776
z6	76.8934	-62.9645	-7.6436
x7	50.5690	47.9073	-73.6382
y7	-10.1262	64.6383	-46.0247
z7	64.7271	-7.0331	-50.8426
x8	-37.7955	-48.7052	27.2730
y8	-36.8329	-8.2453	18.0771
z8	-1.4230	-62.2400	-5.1073
x9	-76.4544	-114.3430	85.2095
y9	6.4075	51.5898	93.2250
z9	-30.5462	-88.6480	49.8745
x10	-116.3826	-143.7003	71.1006
y10	43.5731	49.0521	-19.4681
z10	-53.9469	-18.6102	21.3908
x11	-37.1230	-46.9901	24.9664
y11	19.5229	-37.0684	27.5044
z11	-29.2911	-9.0401	11.2188

x12	-10.1577	18.0452	-3.2875
y12	-41.4857	-30.1837	14.3671
z12	-101.8245	-13.3609	9.0189
x13	54.3582	34.1224	-30.9582
y13	-18.6325	-9.5535	-29.6195
z13	-51.1986	23.7729	-63.2296
x14	100.2140	58.6347	-23.0830
y14	21.6880	-6.2720	-15.7989
z14	10.6537	42.5509	-53.8550
x15	84.8395	55.9870	-9.0012
y15	-51.8831	-28.3353	42.4157
z15	-25.3492	39.6760	-4.8366
x16	79.0356	53.0426	-3.4108
y16	3.6784	29.1025	-4.5054
z16	17.2552	94.2422	-24.9510
x17	27.5084	0.6565	20.1608
y17	10.6034	2.4764	-4.6661
z17	3.1993	47.5003	0.5444
x18	-15.9457	-38.9754	27.8680
y18	-16.0881	23.4582	-18.2731
z18	34.6527	-13.5879	-9.5558
x19	-86.8724	-107.8239	72.7635
y19	-14.6865	93.4415	-61.8026
z19	22.5400	-6.6138	-27.6203
x20	-60.6532	-49.3134	24.7503
y20	-7.4092	-10.8641	-38.5203
z20	-14.7897	-9.2208	-7.7014
x21	-8.2779	-12.0671	-25.5778
y21	-27.4129	-18.4184	-23.2443
z21	-43.2253	38.1087	-10.0080
x22	17.3188	15.3403	-78.8281
y22	-48.6583	-66.0752	59.4387
z22	-38.0915	38.1891	21.8251
x23	-28.3372	1.3951	-62.1018
y23	-55.8571	-47.6566	98.1143
z23	23.7619	66.0389	-7.7230
x24	-24.5714	28.2803	-68.2339
y24	63.3342	-25.0816	-4.2242
z24	23.8607	23.6223	7.1503
x25	-6.5031	65.1792	-67.7899
y25	26.4745	-82.1728	4.7382
z25	-0.3979	-14.7516	27.2802
x26	-27.7604	18.6240	-27.1083
y26	9.2316	-7.5399	-13.2331
z26	22.1350	-42.3257	25.4709
x27	-58.3090	-48.4683	23.5821
y27	-18.1811	62.3625	2.1740
z27	23.5866	-80.2159	22.3199
x28	-108.6191	-48.1485	37.2658
y28	-14.8991	66.2078	32.9290
z28	16.1713	-150.1579	31.7230
x29	-35.0261	30.4188	66.4067
y29	57.8232	-25.0735	-32.6509
z29	2.1881	-34.1233	-16.5939
x30	91.3974	8.3328	-6.5719
y30	14.6356	-4.6072	14.6891
z30	-50.3001	58.4059	-22.5495
x31	103.7898	46.7442	27.6332
y31	6.3132	-46.2844	-19.9527
z31	-45.3244	105.0859	-5.0142

x32	-16.8141	-18.3244	-0.9983
y32	59.9801	-94.5128	27.1978
z32	-92.9235	-74.0757	145.5000
x33	26.5415	21.3398	1.7817
y33	-13.7347	-51.7035	36.1561
z33	8.9361	-30.9895	27.8970
x34	49.2146	-31.9646	21.2481
y34	-20.1577	9.2444	-0.4090
z34	67.1663	-7.6242	-47.8486
x35	50.1388	-3.4938	-19.0161
y35	10.5006	14.1319	-25.3874
z35	41.8636	25.8822	-102.2091
x36	40.1426	38.2364	-99.8283
y36	14.8609	43.4003	-78.5831
z36	-46.5838	29.3128	-103.4909
x37	-28.3554	33.7768	29.3935
y37	-2.2960	15.1609	-31.0285
z37	-33.5670	4.3579	-17.2238
x38	-114.0787	-7.2043	46.0984
y38	-35.3498	5.2241	2.6285
z38	-13.7504	2.8269	-10.3550
x39	-77.7250	-75.2911	66.5809
y39	-73.6564	-29.2199	43.2397
z39	14.8983	-6.7132	21.1063
x40	-48.6931	-116.3752	66.9654
y40	-67.0510	-49.2146	46.1367
z40	58.4177	-9.8400	27.3779
x41	46.5598	-56.5851	-22.9164
y41	-4.8256	-27.5281	-8.0034
z41	82.1040	-4.8399	17.9671
x42	25.7185	12.3447	-39.2316
y42	0.2770	32.2189	-33.4879
z42	40.3227	10.6305	32.2571
x43	5.6261	9.8487	-27.7225
y43	24.1001	55.1471	-55.6186
z43	0.3507	16.6421	28.7154
x44	0.7619	13.3168	-23.7528
y44	27.5045	61.9045	-40.8814
z44	-54.8693	34.7634	7.9981
x45	27.4391	46.2309	-56.5673
y45	81.3291	63.0183	-51.9860
z45	-91.6489	69.8512	-37.9493
x46	0.2221	21.8897	-15.7855
y46	9.4807	17.4547	39.3657
z46	-90.4340	1.5933	13.3839
x47	-0.6789	27.9578	-11.9829
y47	-33.4447	2.1381	51.6191
z47	-59.9069	-44.2636	41.3826
x48	8.0494	26.8848	-1.0782
y48	-32.7858	59.6182	50.2071
z48	1.2307	-37.6466	29.6158
x49	-1.6721	21.3988	14.5509
y49	26.3830	13.5468	40.3194
z49	58.0425	-40.8368	17.8629
x50	11.6100	21.3350	16.0763
y50	21.3627	7.2331	-30.0313
z50	82.4489	-17.8962	-38.7318
x51	-24.8833	17.1026	23.3315
y51	51.1883	-9.6079	35.7219
z51	74.3312	-26.0327	22.7365

x52	-41.4504	-29.6630	77.3351
y52	44.2085	37.4514	-40.2736
z52	86.5882	34.6232	-50.3017
x53	-55.7576	-20.0356	35.7378
y53	21.6847	-18.0250	-15.6617
z53	49.1904	39.4357	-39.7037
x54	41.0114	-19.9858	-62.7168
y54	4.5925	-17.1384	-80.5061
z54	-58.4454	42.6912	-2.8595
x55	89.7739	49.6077	-1.9361
y55	-67.8076	-38.9556	69.5004
z55	-18.8960	-30.2962	39.4282

New Procrustes fit:
Dataset 'angles between first and second turn of cochlea'

4 landmarks in 3 dimensions.
The dataset contains 40 observations, of which 36 are included for analyses.

Average shape:

Lmk.	Axis 1 (x)	Axis 2 (y)	Axis 3 (z)
1	-0.60702438	-0.19207557	-0.01967511
2	0.29107027	-0.31326124	0.07388396
3	-0.04103752	0.51708406	0.03541596
4	0.35699163	-0.01174725	-0.08962482

Procrustes sums of squares: 0.6297515807324856
Tangent sums of squares: 0.42540006226740024

Data matrices in this dataset:
- angles between first and second turn of cochlea, raw data
- angles between first and second turn of cochlea, centroid size
- angles between first and second turn of cochlea, Procrustes coordinates

New Procrustes fit:
Dataset 'angles between first and second turn of cochlea'

4 landmarks in 3 dimensions.
The dataset contains 40 observations, of which 36 are included for analyses.

Average shape:

Lmk.	Axis 1 (x)	Axis 2 (y)	Axis 3 (z)
1	-0.60702438	-0.19207557	-0.01967511
2	0.29107027	-0.31326124	0.07388396
3	-0.04103752	0.51708406	0.03541596
4	0.35699163	-0.01174725	-0.08962482

Procrustes sums of squares: 0.6297515807324856
Tangent sums of squares: 0.42540006226740024

Data matrices in this dataset:
- angles between first and second turn of cochlea, raw data

- angles between first and second turn of cochlea, centroid size
- angles between first and second turn of cochlea, Procrustes coordinates

Principal Component Analysis: PCA: CovMatrix, angles between first and second turn of cochlea, Procrustes coordinates

	Eigenvalues	% Variance	Cumulative %
1.	0.00798741	65.717	65.717
2.	0.00226273	18.617	84.334
3.	0.00077203	6.352	90.685
4.	0.00070514	5.802	96.487
5.	0.00042698	3.513	100.000

Total variance: 0.01215429

Variance of the eigenvalues: 0.0000081297707
Eigenvalue variance scaled by total variance: 0.05503
Eigenvalue variance scaled by total variance and number of variables: 0.34395
Note: throughout all calculations of eigenvalue variances, the dimensionality used was 5.
Please double-check because this dimensionality may not be appropriate for all situations.

Principal Component Coefficients

	PC1	PC2	PC3	PC4	PC5
x1	-0.298401	0.431724	-0.200082	0.054913	0.157914
y1	-0.371458	-0.358660	-0.124595	0.230662	0.051894
z1	0.060830	0.039753	-0.067321	0.165517	-0.085633
x2	-0.461038	-0.392962	0.426533	-0.024845	-0.083535
y2	0.227281	-0.240901	-0.089919	-0.516779	-0.409803
z2	-0.049819	-0.126460	-0.053826	-0.295019	0.509293
x3	0.460685	0.137640	0.434425	-0.232167	0.030498
y3	-0.234492	0.623608	-0.028071	-0.158546	-0.090071
z3	-0.041968	-0.033020	-0.104565	-0.278525	0.120034
x4	0.298753	-0.176402	-0.660876	0.202099	-0.104877
y4	0.378670	-0.024047	0.242585	0.444664	0.447980
z4	0.030957	0.119727	0.225713	0.408027	-0.543695

Canonical Variate Analysis: CVA ... angle between 1st and 2nd turns

Dataset: angles between first and second turn of cochlea
Classification criterion: subfam

Groups		Observations
1.	Ca	9
2.	Ce	13
3.	Di	3
4.	Pr	11

Variation among groups, scaled by the inverse of the within-group variation

Eigenvalues% Variance Cumulative %

1.	0.29791648	59.756	59.756
2.	0.12041555	24.153	83.909
3.	0.08021980	16.091	100.000

Mahalanobis distances among groups:

	Ca	Ce	Di
Ce	1.1073		
Di	1.7617	1.2746	
Pr	0.7774	1.0016	1.5614

P-values from permutation tests (10000 permutation rounds) for Mahalanobis distances among groups:

	Ca	Ce	Di
Ce	0.2707		
Di	0.2175	0.5064	
Pr	0.8403	0.3200	0.3287

Procrustes distances among groups:

	Ca	Ce	Di
Ce	0.0316		
Di	0.0456	0.0446	
Pr	0.0487	0.0613	0.0471

P-values from permutation tests (10000 permutation rounds) for Procrustes distances among groups:

	Ca	Ce	Di
Ce	0.4601		
Di	0.5248	0.5079	
Pr	0.8255	0.1501	0.8109

Canonical coefficients:

	CV1	CV2	CV3
x1	-0.9691	2.9376	-8.0000
y1	5.3546	9.4545	-3.5177
z1	7.8567	2.6504	1.3858
x2	-1.2554	-3.3111	-1.5521
y2	-11.4995	1.6059	24.0627
z2	-20.6990	-3.3579	-11.8204
x3	-6.8139	-16.3753	-0.6160
y3	-3.4011	-2.5852	0.5527
z3	-12.7739	0.1251	0.8385
x4	9.0384	16.7488	10.1680
y4	9.5460	-8.4752	-21.0978
z4	25.6162	0.5823	9.5962

New Procrustes fit:
Dataset 'centroid-size'

686 landmarks in 3 dimensions.

The dataset contains 40 observations, of which 37 are included for analyses.

Average shape:

Lmk.	Axis 1 (x)	Axis 2 (y)	Axis 3 (z)
1	-0.04019379	0.02607325	-0.00836638
2	-0.02468057	-0.01012978	-0.02623661
3	-0.02613617	-0.01169092	-0.02727685
4	-0.02803587	-0.01254339	-0.02844157
5	-0.03003845	-0.01290077	-0.02948996
6	-0.03208044	-0.01289319	-0.03044531
7	-0.03421610	-0.01261323	-0.03131256
8	-0.03650340	-0.01192963	-0.03213873
9	-0.03894313	-0.01088893	-0.03288741
10	-0.04144339	-0.00932976	-0.03354864
11	-0.04394490	-0.00725142	-0.03407908
12	-0.04659243	-0.00435646	-0.03453453
13	-0.04862632	-0.00127557	-0.03457688
14	-0.05016677	0.00177655	-0.03425389
15	-0.05118980	0.00479364	-0.03356004
16	-0.05184638	0.00776567	-0.03258749
17	-0.05209121	0.01073313	-0.03126163
18	-0.05189916	0.01370367	-0.02958736
19	-0.05118797	0.01668152	-0.02754906
20	-0.04978782	0.01951626	-0.02519534
21	-0.04774596	0.02176219	-0.02209023
22	-0.04529597	0.02319932	-0.01924350
23	-0.04270318	0.02393862	-0.01662513
24	-0.04001204	0.02402242	-0.01422837
25	-0.03727124	0.02352597	-0.01195115
26	-0.03427045	0.02233895	-0.00994382
27	-0.03107728	0.02036840	-0.00824496
28	-0.02774002	0.01779257	-0.00666179
29	-0.02428107	0.01450487	-0.00539564
30	-0.02260898	0.01283072	-0.00506779
31	-0.02120255	0.01117090	-0.00514125
32	-0.02012074	0.00953236	-0.00561803
33	-0.01933528	0.00792778	-0.00646866
34	-0.01884341	0.00634979	-0.00768777
35	-0.01860769	0.00477621	-0.00924913
36	-0.01857466	0.00318725	-0.01113838
37	-0.01875954	0.00150974	-0.01337378
38	-0.01943088	0.00009495	-0.01602543
39	-0.02045263	-0.00027457	-0.01764531
40	-0.02163258	-0.00052159	-0.01920345
41	-0.02261562	-0.00107280	-0.02063066
42	-0.02339427	-0.00191409	-0.02190157
43	-0.02395464	-0.00305215	-0.02301518
44	-0.02434738	-0.00446854	-0.02401140
45	-0.02449344	-0.00619873	-0.02483509
46	-0.02447862	-0.00818458	-0.02548324
47	-0.01950838	-0.01445070	-0.02642088
48	-0.02193968	-0.01553371	-0.02775769
49	-0.02441994	-0.01626305	-0.02898183
50	-0.02689806	-0.01664101	-0.03006757
51	-0.02933144	-0.01672056	-0.03104412
52	-0.03169576	-0.01654433	-0.03195491
53	-0.03404220	-0.01607958	-0.03275442
54	-0.03634762	-0.01535298	-0.03348900

55	-0.03863031	-0.01438686	-0.03418383
56	-0.04087153	-0.01308937	-0.03474489
57	-0.04228205	-0.01080715	-0.03457678
58	-0.04487248	-0.00993848	-0.03571342
59	-0.04658900	-0.00819389	-0.03597150
60	-0.04817816	-0.00633069	-0.03615229
61	-0.04963230	-0.00438375	-0.03624560
62	-0.05088795	-0.00228855	-0.03616265
63	-0.05196169	-0.00008099	-0.03595518
64	-0.05285123	0.00226101	-0.03557077
65	-0.05361028	0.00469363	-0.03509924
66	-0.05412830	0.00687229	-0.03444115
67	-0.05443999	0.00898676	-0.03367513
68	-0.05457488	0.01104586	-0.03281137
69	-0.05452485	0.01304845	-0.03184194
70	-0.05428353	0.01498449	-0.03076581
71	-0.05391726	0.01691531	-0.02962183
72	-0.05329487	0.01873536	-0.02830878
73	-0.05248088	0.02049658	-0.02690074
74	-0.05148359	0.02218466	-0.02539005
75	-0.05014055	0.02367179	-0.02386636
76	-0.04868322	0.02496308	-0.02223271
77	-0.04711925	0.02602158	-0.02047790
78	-0.04544779	0.02686789	-0.01859172
79	-0.04366857	0.02746805	-0.01659049
80	-0.04176791	0.02783577	-0.01445876
81	-0.03971786	0.02795972	-0.01211183
82	-0.03763309	0.02798084	-0.00977505
83	-0.03542483	0.02813590	-0.00736796
84	-0.02469204	0.00508896	0.02175974
85	-0.02659415	0.00354426	0.02310298
86	-0.02891670	0.00256809	0.02440083
87	-0.03124698	0.00190369	0.02547143
88	-0.03351013	0.00138793	0.02627825
89	-0.03574330	0.00104061	0.02691500
90	-0.03797191	0.00086125	0.02736116
91	-0.04020382	0.00082448	0.02765488
92	-0.04244924	0.00103029	0.02776346
93	-0.04468672	0.00141700	0.02767907
94	-0.04731274	0.00207557	0.02715591
95	-0.04964290	0.00300058	0.02634874
96	-0.05168649	0.00410575	0.02527937
97	-0.05339986	0.00543146	0.02395871
98	-0.05488940	0.00687240	0.02242472
99	-0.05615455	0.00848508	0.02066289
100	-0.05716301	0.01025216	0.01864683
101	-0.05789662	0.01223183	0.01639613
102	-0.05826960	0.01439435	0.01393211
103	-0.05816493	0.01699436	0.01087253
104	-0.05746290	0.01924509	0.00810453
105	-0.05619312	0.02117344	0.00571482
106	-0.05451242	0.02272788	0.00362107
107	-0.05240233	0.02391413	0.00181385
108	-0.04990825	0.02475203	0.00015872
109	-0.04689627	0.02526302	-0.00116941
110	-0.04337667	0.02539026	-0.00216109
111	-0.03939094	0.02516532	-0.00252448
112	-0.03566201	0.02345406	-0.00200618
113	-0.03233956	0.02182252	-0.00099466
114	-0.02939221	0.02030627	0.00012429

115	-0.02682178	0.01884685	0.00119411
116	-0.02461066	0.01742259	0.00231094
117	-0.02270305	0.01601186	0.00358512
118	-0.02112241	0.01459409	0.00517699
119	-0.02005788	0.01336951	0.00722897
120	-0.01989219	0.01284472	0.00936220
121	-0.02059791	0.01295176	0.01085722
122	-0.02155025	0.01312693	0.01232752
123	-0.02239488	0.01295503	0.01377297
124	-0.02310612	0.01245246	0.01519834
125	-0.02361273	0.01146738	0.01657531
126	-0.02401317	0.01028762	0.01791328
127	-0.02425018	0.00882833	0.01918885
128	-0.02425867	0.00686147	0.02041154
129	-0.01876141	0.00319276	0.02391194
130	-0.02092590	0.00230692	0.02521625
131	-0.02329298	0.00156149	0.02617140
132	-0.02563341	0.00091134	0.02704722
133	-0.02795440	0.00034754	0.02781468
134	-0.03025240	-0.00012069	0.02849910
135	-0.03254261	-0.00050607	0.02911845
136	-0.03483967	-0.00079722	0.02965783
137	-0.03715086	-0.00099217	0.03005269
138	-0.03945387	-0.00108383	0.03032133
139	-0.04181054	-0.00092900	0.03042259
140	-0.04404924	-0.00065906	0.03042115
141	-0.04616489	-0.00023669	0.03016693
142	-0.04815510	0.00029984	0.02971393
143	-0.05001715	0.00095176	0.02907882
144	-0.05175562	0.00176175	0.02825660
145	-0.05339695	0.00269441	0.02727240
146	-0.05491298	0.00376111	0.02609108
147	-0.05631667	0.00496181	0.02474998
148	-0.05777110	0.00652096	0.02304808
149	-0.05894157	0.00813521	0.02127817
150	-0.05985060	0.00979222	0.01943883
151	-0.06055023	0.01148973	0.01756018
152	-0.06099791	0.01323328	0.01562252
153	-0.06118445	0.01499460	0.01359866
154	-0.06109683	0.01677731	0.01149553
155	-0.06070816	0.01858562	0.00930512
156	-0.05998708	0.02039662	0.00705370
157	-0.05867301	0.02250191	0.00473805
158	-0.05693192	0.02423362	0.00263205
159	-0.05488745	0.02568037	0.00071032
160	-0.05256023	0.02689124	-0.00108702
161	-0.04984301	0.02775063	-0.00268109
162	-0.04679071	0.02832948	-0.00411687
163	-0.04334602	0.02853506	-0.00535620
164	-0.03951309	0.02855421	-0.00647550
165	-0.03542483	0.02813590	-0.00736796
166	-0.02488827	-0.01809223	-0.01132516
167	-0.02582807	-0.01938256	-0.01118322
168	-0.02706335	-0.02043641	-0.01055226
169	-0.02826432	-0.02134544	-0.00971461
170	-0.02943414	-0.02215131	-0.00867608
171	-0.03063705	-0.02284252	-0.00740910
172	-0.03184999	-0.02348836	-0.00590151
173	-0.03308117	-0.02402234	-0.00414239
174	-0.03429744	-0.02447192	-0.00215719

175	-0.03558534	-0.02469944	0.00007009
176	-0.03648488	-0.02474938	0.00168589
177	-0.03732678	-0.02465839	0.00328735
178	-0.03818735	-0.02306170	0.00467446
179	-0.03880636	-0.02403085	0.00638250
180	-0.03943093	-0.02350047	0.00788256
181	-0.03999204	-0.02283949	0.00936520
182	-0.04046875	-0.02204647	0.01082479
183	-0.04088356	-0.02110545	0.01226312
184	-0.04123011	-0.02000902	0.01367011
185	-0.04144001	-0.01865929	0.01522605
186	-0.04146402	-0.01726713	0.01658064
187	-0.04131347	-0.01589813	0.01776107
188	-0.04095719	-0.01445603	0.01869544
189	-0.04043510	-0.01301700	0.01948036
190	-0.03971455	-0.01152481	0.02000944
191	-0.03878875	-0.00998743	0.02030918
192	-0.03767755	-0.00843349	0.02043032
193	-0.03637264	-0.00683757	0.02029805
194	-0.03443667	-0.00481449	0.01989956
195	-0.03261864	-0.00299314	0.01922001
196	-0.03089276	-0.00144925	0.01822763
197	-0.02927835	-0.00009389	0.01702958
198	-0.02778169	0.00105067	0.01559763
199	-0.02638973	0.00202877	0.01393432
200	-0.02510286	0.00272141	0.01200509
201	-0.02384126	0.00330651	0.00987486
202	-0.02276138	0.00366964	0.00750813
203	-0.02208929	0.00346818	0.00556460
204	-0.02158844	0.00298745	0.00363952
205	-0.02119910	0.00236068	0.00184925
206	-0.02083638	0.00158379	0.00018921
207	-0.02046399	0.00063564	-0.00136089
208	-0.02012655	-0.00051206	-0.00286516
209	-0.01984796	-0.00184961	-0.00430506
210	-0.01977243	-0.00338417	-0.00566593
211	-0.02017913	-0.00500755	-0.00664105
212	-0.02094850	-0.00649850	-0.00672344
213	-0.02189565	-0.00797688	-0.00670760
214	-0.02279807	-0.00944939	-0.00681782
215	-0.02349604	-0.01091076	-0.00714100
216	-0.02396975	-0.01234906	-0.00769442
217	-0.02425927	-0.01378919	-0.00846293
218	-0.02437605	-0.01525689	-0.00946170
219	-0.02442561	-0.01676491	-0.01059120
220	-0.02280881	-0.01991476	-0.01636761
221	-0.02424890	-0.02110805	-0.01566785
222	-0.02563703	-0.02217217	-0.01477154
223	-0.02696066	-0.02313481	-0.01369155
224	-0.02825407	-0.02401987	-0.01251239
225	-0.02947793	-0.02479352	-0.01117838
226	-0.03069594	-0.02548092	-0.00975657
227	-0.03186881	-0.02610666	-0.00821353
228	-0.03299316	-0.02664463	-0.00653125
229	-0.03410512	-0.02713068	-0.00474110
230	-0.03518223	-0.02746499	-0.00281555
231	-0.03624369	-0.02768946	-0.00095253
232	-0.03723718	-0.02772698	0.00090130
233	-0.03819515	-0.02762620	0.00270711
234	-0.03904395	-0.02732609	0.00452067

235	-0.03986368	-0.02687294	0.00629538
236	-0.04061011	-0.02622096	0.00806154
237	-0.04130156	-0.02542420	0.00979867
238	-0.04192746	-0.02444158	0.01151281
239	-0.04250837	-0.02321302	0.01336366
240	-0.04298404	-0.02197085	0.01505602
241	-0.04327016	-0.02065905	0.01658414
242	-0.04337078	-0.01928805	0.01797373
243	-0.04329691	-0.01787233	0.01918761
244	-0.04307573	-0.01641412	0.02026946
245	-0.04269627	-0.01491079	0.02119480
246	-0.04211559	-0.01334555	0.02195347
247	-0.04140050	-0.01174545	0.02257606
248	-0.04037348	-0.00988821	0.02311980
249	-0.03918089	-0.00806553	0.02333936
250	-0.03787188	-0.00626209	0.02330014
251	-0.03643745	-0.00450576	0.02298035
252	-0.03489194	-0.00277548	0.02240497
253	-0.03332904	-0.00094211	0.02168790
254	-0.03140034	0.00059518	0.02044853
255	-0.02936035	0.00216628	0.01902626
256	-0.02749967	0.00390636	0.01746773
257	-0.01437015	-0.00884076	0.00106321
258	-0.01378287	-0.00911586	0.00170756
259	-0.01310930	-0.00940964	0.00224040
260	-0.01231133	-0.00961351	0.00260428
261	-0.01146395	-0.00977596	0.00287782
262	-0.01064647	-0.01003156	0.00299501
263	-0.00971593	-0.01032588	0.00296068
264	-0.00877591	-0.01064936	0.00288736
265	-0.00761919	-0.01086478	0.00314888
266	-0.00666552	-0.01134311	0.00219721
267	-0.00541184	-0.01140526	0.00172314
268	-0.00430690	-0.01155825	0.00123791
269	-0.00339640	-0.01164911	0.00065591
270	-0.00262487	-0.01174326	-0.00000726
271	-0.00201008	-0.01188826	-0.00073522
272	-0.00152945	-0.01203335	-0.00157958
273	-0.00118481	-0.01209500	-0.00254035
274	-0.00104485	-0.01213453	-0.00358397
275	-0.00111318	-0.01211892	-0.00467414
276	-0.00183363	-0.01239378	-0.00549659
277	-0.00266963	-0.01251305	-0.00614939
278	-0.00353419	-0.01256271	-0.00663699
279	-0.00445144	-0.01259805	-0.00692172
280	-0.00540280	-0.01259119	-0.00704053
281	-0.00641990	-0.01253439	-0.00700940
282	-0.00743849	-0.01244139	-0.00676826
283	-0.00857987	-0.01240503	-0.00639555
284	-0.00969844	-0.01227900	-0.00585889
285	-0.01074656	-0.01192087	-0.00531268
286	-0.01171102	-0.01154175	-0.00470003
287	-0.01249282	-0.01116866	-0.00402923
288	-0.01315792	-0.01079434	-0.00332593
289	-0.01369109	-0.01044697	-0.00257132
290	-0.01409430	-0.01008246	-0.00174436
291	-0.01434442	-0.00973085	-0.00084816
292	-0.01441613	-0.00938511	0.00010162
293	0.02608995	0.01944433	0.01589818
294	0.02834013	0.01826428	0.01822532

295	0.03014761	0.01721400	0.01988751
296	0.03269419	0.01477895	0.02145456
297	0.03362389	0.01325117	0.02144902
298	0.03445390	0.01141340	0.02096555
299	0.03495286	0.00913059	0.01968311
300	0.03726842	0.01685872	-0.01061084
301	0.03937701	0.01619731	-0.01090181
302	0.04108544	0.01549707	-0.01107143
303	0.04227021	0.01456069	-0.01099977
304	0.04354043	0.01232431	-0.01043417
305	0.04377097	0.01095019	-0.00994402
306	0.04360401	0.00932627	-0.00924027
307	0.02063801	0.00412504	-0.01860356
308	0.02113106	0.00246103	-0.01983663
309	0.02172831	0.00100267	-0.02053722
310	0.02238367	-0.00022952	-0.02081672
311	0.02390022	-0.00221114	-0.02030108
312	0.02480569	-0.00308015	-0.01955797
313	0.02585638	-0.00387898	-0.01841012
314	0.03193328	0.00457889	0.01068977
315	0.03350590	0.00343425	0.01187094
316	0.03463135	0.00237488	0.01258189
317	0.03546603	0.00139287	0.01289866
318	0.03650266	-0.00061084	0.01250655
319	0.03675770	-0.00175430	0.01180381
320	0.03683797	-0.00304016	0.01071245
321	0.03846835	0.00602761	-0.00647736
322	0.04089649	0.00445100	-0.00689413
323	0.04244731	0.00366880	-0.00734310
324	0.04365489	0.00189783	-0.00720965
325	0.04373575	0.00096983	-0.00693044
326	0.04359323	-0.00009129	-0.00650055
327	0.04313312	-0.00138576	-0.00586854
328	0.02826071	-0.00277658	-0.01144729
329	0.02854069	-0.00444732	-0.01315944
330	0.02907037	-0.00586290	-0.01397366
331	0.03017197	-0.00771834	-0.01424686
332	0.03078545	-0.00839519	-0.01385197
333	0.03151008	-0.00902715	-0.01315454
334	0.03235682	-0.00957962	-0.01199984
335	-0.01207978	-0.00151945	0.01822383
336	-0.00998259	-0.00043367	0.01594557
337	-0.00788071	0.00110107	0.01314038
338	-0.00506400	0.00177183	0.01188027
339	-0.00195794	0.00140724	0.01345324
340	0.00043750	0.00236446	0.01288709
341	0.00287788	0.00356013	0.01240506
342	0.00526281	0.00505963	0.01182641
343	0.00760871	0.00677742	0.01137725
344	0.00995550	0.00849386	0.01145188
345	0.01159860	0.00918310	0.01150754
346	0.01319527	0.00991166	0.01146533
347	0.01473363	0.01062916	0.01134199
348	0.01622823	0.01127428	0.01112026
349	0.01766628	0.01190288	0.01077294
350	0.01906444	0.01254342	0.01034881
351	0.02044157	0.01314914	0.00982113
352	0.02173924	0.01378969	0.00909474
353	0.02297211	0.01441133	0.00828228
354	0.02504921	0.01498828	0.00673071

355	0.02682191	0.01537953	0.00508157
356	0.02832926	0.01559925	0.00338700
357	0.02952814	0.01561794	0.00162484
358	0.03047961	0.01545390	-0.00013322
359	0.03123852	0.01512536	-0.00190711
360	0.03176475	0.01458165	-0.00370499
361	0.03194420	0.01372837	-0.00548224
362	0.03182191	0.01298421	-0.00751970
363	0.03126613	0.01185952	-0.00912977
364	0.03056086	0.01126289	-0.01048786
365	0.02970830	0.01035369	-0.01154694
366	0.02871697	0.00951899	-0.01229309
367	0.02762904	0.00868176	-0.01275909
368	0.02644342	0.00781750	-0.01300499
369	0.02514808	0.00690613	-0.01299755
370	0.02372906	0.00598061	-0.01272739
371	0.02216477	0.00516973	-0.01206611
372	0.02052764	0.00335982	-0.00988084
373	0.01924990	0.00247625	-0.00772680
374	0.01857657	0.00167791	-0.00564644
375	0.01836586	0.00123809	-0.00373185
376	0.01869930	0.00072014	-0.00190301
377	0.01962830	0.00014197	-0.00009616
378	0.02093794	-0.00021919	0.00172912
379	0.02320101	-0.00030898	0.00346974
380	0.02534491	0.00118532	0.00436814
381	0.02810094	0.00127258	0.00412229
382	0.02967291	0.00227062	0.00355277
383	0.03097030	0.00279842	0.00281328
384	0.03177113	0.00305483	0.00186401
385	0.03233819	0.00298033	0.00085957
386	0.03279650	0.00265416	-0.00017926
387	0.03323512	0.00194897	-0.00143420
388	0.03278156	0.00116751	-0.00305891
389	0.03133425	0.00070246	-0.00425400
390	0.03068832	-0.00057768	-0.00427878
391	0.02979158	-0.00153491	-0.00378021
392	0.02925419	-0.00186236	-0.00322667
393	0.02942612	-0.00226509	-0.00285825
394	0.02999055	-0.00283164	-0.00241277
395	0.03114878	-0.00381530	-0.00210482
396	0.03262977	-0.00497475	-0.00167552
397	0.03565082	-0.00578356	-0.00127447
398	0.00190122	0.01411120	0.01974349
399	0.00416286	0.01349926	0.01959726
400	0.00667185	0.01348167	0.01941644
401	0.00913115	0.01375987	0.01904011
402	0.01150846	0.01431523	0.01850395
403	0.01388448	0.01497912	0.01776087
404	0.01630068	0.01587489	0.01678255
405	0.01874432	0.01694295	0.01564197
406	0.02127030	0.01807349	0.01435106
407	0.02391995	0.01919525	0.01288623
408	0.02641788	0.02005549	0.01063844
409	0.02861425	0.02059283	0.00832434
410	0.03052207	0.02079284	0.00595596
411	0.03209798	0.02072899	0.00349785
412	0.03332830	0.02041115	0.00100561
413	0.03422625	0.01987226	-0.00159873
414	0.03479463	0.01907480	-0.00426263

415	0.03497291	0.01797807	-0.00695859
416	0.03488602	0.01679669	-0.00988079
417	0.03381432	0.01542632	-0.01206432
418	0.03260139	0.01409977	-0.01390007
419	0.03123447	0.01284570	-0.01540149
420	0.02976465	0.01158945	-0.01653188
421	0.02816124	0.01039473	-0.01731595
422	0.02643342	0.00919044	-0.01773598
423	0.02455087	0.00802368	-0.01780829
424	0.02256179	0.00682813	-0.01750173
425	0.02054650	0.00555361	-0.01680413
426	0.01774009	0.00395057	-0.01438009
427	0.01570321	0.00256079	-0.01186515
428	0.01437116	0.00139787	-0.00930497
429	0.01369346	0.00051672	-0.00669291
430	0.01363063	-0.00001650	-0.00401091
431	0.01438019	-0.00042188	-0.00126067
432	0.01584510	-0.00056853	0.00155380
433	0.01805173	-0.00050633	0.00443319
434	0.02084403	-0.00003180	0.00742593
435	0.02340718	0.00112840	0.00831166
436	0.02569078	0.00227130	0.00885167
437	0.02775109	0.00330509	0.00902548
438	0.02961877	0.00415566	0.00882458
439	0.03125113	0.00486518	0.00824718
440	0.03267100	0.00547216	0.00730520
441	0.03387544	0.00595027	0.00599018
442	0.03489630	0.00626758	0.00428755
443	0.03573291	0.00635458	0.00222791
444	0.03609788	0.00620517	0.00039922
445	0.03628015	0.00592374	-0.00128971
446	0.03630993	0.00558471	-0.00285223
447	0.03617186	0.00515030	-0.00427244
448	0.03588771	0.00460761	-0.00555253
449	0.03540866	0.00399754	-0.00669678
450	0.03475213	0.00331569	-0.00771317
451	0.03393444	0.00253274	-0.00859649
452	0.03294537	0.00166773	-0.00933074
453	0.03181404	0.00075520	-0.00970612
454	0.03073214	-0.00002902	-0.00985621
455	0.02975426	-0.00078070	-0.00976278
456	0.02882774	-0.00143397	-0.00944831
457	0.02797290	-0.00203415	-0.00887879
458	0.02720065	-0.00260177	-0.00803010
459	0.02659354	-0.00320380	-0.00688083
460	0.02617811	-0.00384507	-0.00546336
461	0.02601360	-0.00455910	-0.00384920
462	0.02684751	-0.00433831	-0.00201772
463	0.02777802	-0.00434434	-0.00060512
464	0.02872768	-0.00440146	0.00048779
465	0.02976100	-0.00448991	0.00121731
466	0.03077422	-0.00441388	0.00182068
467	0.03205825	-0.00455710	0.00180708
468	0.03394185	-0.00482885	0.00122355
469	0.03581642	-0.00584205	-0.00026279
470	-0.01525698	-0.00454298	0.01266612
471	-0.01445817	-0.00617333	0.01222797
472	-0.01339028	-0.00766540	0.01173173
473	-0.01209161	-0.00893738	0.01136343
474	-0.01072610	-0.01015632	0.01125428

475	-0.00921643	-0.01125057	0.01132436
476	-0.00762457	-0.01230170	0.01159500
477	-0.00596498	-0.01335300	0.01209849
478	-0.00416323	-0.01429407	0.01276083
479	-0.00223848	-0.01511905	0.01359960
480	-0.00041944	-0.01557427	0.01466008
481	0.00130113	-0.01587645	0.01578453
482	0.00298294	-0.01593815	0.01692770
483	0.00459716	-0.01579787	0.01810482
484	0.00614187	-0.01546473	0.01933664
485	0.00763579	-0.01493858	0.02060612
486	0.00908343	-0.01414266	0.02189660
487	0.01047438	-0.01309208	0.02321040
488	0.01182835	-0.01179879	0.02448626
489	0.01306422	-0.01026368	0.02567936
490	0.01430946	-0.00862983	0.02666431
491	0.01556080	-0.00695856	0.02753091
492	0.01683934	-0.00521632	0.02815756
493	0.01809480	-0.00338643	0.02862647
494	0.01937673	-0.00147500	0.02890071
495	0.02067908	0.00052722	0.02898679
496	0.02199118	0.00260965	0.02886433
497	0.02330411	0.00476722	0.02847881
498	0.02528388	0.00776488	0.02751930
499	0.02723717	0.01041737	0.02614284
500	0.02918830	0.01269161	0.02441044
501	0.03116237	0.01462136	0.02239672
502	0.03317285	0.01623441	0.02009137
503	0.03522297	0.01751936	0.01744644
504	0.03730023	0.01844998	0.01443157
505	0.03927500	0.01893343	0.01097341
506	0.04115127	0.01895155	0.00712926
507	0.04275185	0.01843566	0.00247504
508	0.04356065	0.01747669	-0.00192471
509	0.04361684	0.01621737	-0.00587622
510	0.04314669	0.01481100	-0.00934809
511	0.04215855	0.01326705	-0.01237311
512	0.04076690	0.01163765	-0.01505913
513	0.03893031	0.00989665	-0.01744088
514	0.03656840	0.00798750	-0.01947631
515	0.03362284	0.00589638	-0.02102109
516	0.03099421	0.00426158	-0.02180367
517	0.02844308	0.00269958	-0.02206239
518	0.02603472	0.00123626	-0.02185096
519	0.02379336	-0.00012244	-0.02122930
520	0.02170488	-0.00140637	-0.02031325
521	0.01975060	-0.00261319	-0.01901670
522	0.01791562	-0.00374361	-0.01734660
523	0.01621601	-0.00479478	-0.01529637
524	0.01469186	-0.00574548	-0.01284771
525	0.01372349	-0.00666676	-0.01038867
526	0.01312936	-0.00741047	-0.00792384
527	0.01288751	-0.00803514	-0.00556302
528	0.01288519	-0.00846763	-0.00315325
529	0.01324545	-0.00879688	-0.00087484
530	0.01385632	-0.00898901	0.00140428
531	0.01477153	-0.00908661	0.00365888
532	0.01600809	-0.00898293	0.00588104
533	0.01762233	-0.00869592	0.00799691
534	0.01939233	-0.00815070	0.00995219

535	0.02126946	-0.00741870	0.01150431
536	0.02318733	-0.00658458	0.01272653
537	0.02514979	-0.00563394	0.01362840
538	0.02717725	-0.00452160	0.01413402
539	0.02924743	-0.00340043	0.01447153
540	0.03138252	-0.00205852	0.01433092
541	0.03359340	-0.00062562	0.01389419
542	0.03582070	0.00090998	0.01294473
543	0.03814684	0.00207247	0.01146176
544	0.04007583	0.00297255	0.00966352
545	0.04160634	0.00361509	0.00771161
546	0.04282657	0.00403254	0.00563628
547	0.04374816	0.00420961	0.00344855
548	0.04438765	0.00418719	0.00108000
549	0.04469043	0.00393789	-0.00148783
550	0.04453449	0.00340272	-0.00425258
551	0.04387992	0.00256727	-0.00709327
552	0.04296652	0.00159992	-0.00936600
553	0.04177182	0.00056928	-0.01123528
554	0.04044535	-0.00048646	-0.01274216
555	0.03898325	-0.00158712	-0.01388993
556	0.03740551	-0.00271437	-0.01472874
557	0.03566260	-0.00387486	-0.01523349
558	0.03377330	-0.00509317	-0.01545161
559	0.03169738	-0.00633992	-0.01524670
560	0.02953015	-0.00759518	-0.01453126
561	0.02763752	-0.00891471	-0.01322876
562	0.02616855	-0.00998372	-0.01166004
563	0.02512062	-0.01083816	-0.00999141
564	0.02442955	-0.01152071	-0.00828588
565	0.02404299	-0.01208265	-0.00651582
566	0.02395580	-0.01252746	-0.00465284
567	0.02423194	-0.01282933	-0.00265849
568	0.02496660	-0.01281289	-0.00056025
569	0.02617110	-0.01255771	0.00143818
570	0.02804298	-0.01193091	0.00333762
571	0.02992764	-0.01115600	0.00448063
572	0.03155268	-0.01044983	0.00505411
573	0.03290955	-0.00983373	0.00521855
574	0.03417912	-0.00929963	0.00509115
575	0.03549119	-0.00882148	0.00457981
576	0.03690277	-0.00836600	0.00338478
577	0.03773534	-0.00790258	0.00101855
578	-0.00464144	-0.00599742	-0.01380416
579	-0.00280013	-0.00541174	-0.01296169
580	-0.00080541	-0.00530892	-0.01203860
581	0.00090212	-0.00536639	-0.01088391
582	0.00235174	-0.00558505	-0.00948066
583	0.00343787	-0.00583122	-0.00779593
584	0.00414608	-0.00619745	-0.00576148
585	0.00469463	-0.00673751	-0.00356003
586	0.00501702	-0.00741867	-0.00112369
587	0.00537711	-0.00840588	0.00138541
588	0.00640728	-0.00996733	0.00368095
589	0.00785121	-0.01136460	0.00577225
590	0.00937845	-0.01241926	0.00771090
591	0.01095014	-0.01305286	0.00955739
592	0.01254641	-0.01324700	0.01134680
593	0.01423502	-0.01306444	0.01305388
594	0.01599072	-0.01248041	0.01471231

595	0.01780931	-0.01148671	0.01631891
596	0.01967714	-0.01006515	0.01785495
597	0.02155873	-0.00823119	0.01908091
598	0.02329239	-0.00627908	0.02003766
599	0.02490642	-0.00426316	0.02068812
600	0.02645422	-0.00223745	0.02100629
601	0.02798005	-0.00023048	0.02096512
602	0.02951270	0.00175572	0.02055923
603	0.03111765	0.00369168	0.01977448
604	0.03285804	0.00551280	0.01864152
605	0.03470711	0.00719123	0.01720754
606	0.03681891	0.00865298	0.01468668
607	0.03868428	0.00970510	0.01209768
608	0.04026569	0.01038570	0.00944675
609	0.04153519	0.01070329	0.00668668
610	0.04248762	0.01067589	0.00385875
611	0.04310459	0.01029712	0.00092240
612	0.04337047	0.00962046	-0.00212257
613	0.04321366	0.00867906	-0.00530133
614	0.04257652	0.00753599	-0.00857573
615	0.04159458	0.00629327	-0.01103072
616	0.04033191	0.00508318	-0.01309272
617	0.03891296	0.00382773	-0.01476128
618	0.03728194	0.00257374	-0.01603713
619	0.03550388	0.00125753	-0.01691432
620	0.03354812	-0.00009183	-0.01740619
621	0.03142440	-0.00148644	-0.01751567
622	0.02912633	-0.00293651	-0.01724597
623	0.02667540	-0.00445648	-0.01658504
624	0.02456995	-0.00589669	-0.01530331
625	0.02280251	-0.00708604	-0.01378160
626	0.02141666	-0.00811241	-0.01206127
627	0.02039746	-0.00897016	-0.01015067
628	0.01977361	-0.00968368	-0.00804365
629	0.01954743	-0.01030750	-0.00574288
630	0.01971798	-0.01080379	-0.00324200
631	0.02027013	-0.01120547	-0.00052290
632	0.02120330	-0.01134213	0.00236399
633	0.02258919	-0.01117236	0.00454386
634	0.02403017	-0.01074473	0.00637335
635	0.02553293	-0.01012675	0.00782727
636	0.02712993	-0.00937038	0.00890775
637	0.02881977	-0.00853091	0.00962894
638	0.03061560	-0.00757868	0.00998909
639	0.03252442	-0.00652231	0.00998590
640	0.03457546	-0.00542535	0.00965356
641	0.03671193	-0.00418749	0.00894745
642	0.03829828	-0.00343227	0.00788953
643	0.03964142	-0.00282533	0.00670927
644	0.04074559	-0.00237906	0.00540041
645	0.04160994	-0.00208650	0.00396996
646	0.04224964	-0.00196391	0.00242652
647	0.04264925	-0.00198106	0.00074336
648	0.04284743	-0.00219413	-0.00105848
649	0.04276036	-0.00251561	-0.00300531
650	0.04240747	-0.00296619	-0.00506571
651	0.04182445	-0.00355324	-0.00662138
652	0.04111826	-0.00419763	-0.00794152
653	0.04026250	-0.00485730	-0.00901195
654	0.03930799	-0.00558822	-0.00983876

655	0.03824011	-0.00635386	-0.01042577
656	0.03706585	-0.00718955	-0.01077677
657	0.03579441	-0.00810055	-0.01088642
658	0.03442271	-0.00907640	-0.01075658
659	0.03292539	-0.01008123	-0.01037442
660	0.03175632	-0.01100151	-0.00942149
661	0.03080667	-0.01175138	-0.00840262
662	0.03008503	-0.01235589	-0.00732956
663	0.02961108	-0.01284886	-0.00621123
664	0.02938081	-0.01320273	-0.00504615
665	0.02936235	-0.01341475	-0.00382484
666	0.02959211	-0.01349267	-0.00254634
667	0.03005903	-0.01341122	-0.00121789
668	0.03073753	-0.01316650	0.00017093
669	0.03183169	-0.01276149	0.00092252
670	0.03281941	-0.01228432	0.00144262
671	0.03377060	-0.01184664	0.00176484
672	0.03464744	-0.01138373	0.00187452
673	0.03547696	-0.01089368	0.00176851
674	0.03628189	-0.01033572	0.00141918
675	0.03707150	-0.00964506	0.00077684
676	0.03761620	-0.00860920	-0.00015398
677	-0.00909638	0.01389013	0.00007162
678	-0.01262522	0.01689383	-0.00149735
679	-0.01643150	0.01951586	-0.00295792
680	-0.02002979	0.02197323	-0.00419971
681	-0.02329629	0.02439344	-0.00529067
682	-0.02644548	0.02656275	-0.00617810
683	-0.02949680	0.02853531	-0.00685835
684	-0.03252491	0.03014723	-0.00732685
685	-0.03537769	0.03169408	-0.00759349
686	-0.03816149	0.03290959	-0.00772531

Procrustes sums of squares: 0.3134047093632342
Tangent sums of squares: 0.31047273223643096

Data matrices in this dataset:
- all, raw data
- centroid-size, centroid size
- centroid-size, Procrustes coordinates

Principal Component Analysis: PCA: CovMatrix, centroid-size, Procrustes coordinates

	Eigenvalues	% Variance	Cumulative %
1.	0.00128871	14.943	14.943
2.	0.00087746	10.174	25.117
3.	0.00068253	7.914	33.031
4.	0.00060678	7.036	40.067
5.	0.00055093	6.388	46.455
6.	0.00043345	5.026	51.481
7.	0.00037052	4.296	55.777
8.	0.00034355	3.983	59.761
9.	0.00032661	3.787	63.548
10.	0.00031234	3.622	67.170
11.	0.00028679	3.325	70.495
12.	0.00022412	2.599	73.094

13.	0.00019769	2.292	75.386
14.	0.00018256	2.117	77.503
15.	0.00017619	2.043	79.546
16.	0.00015841	1.837	81.383
17.	0.00015210	1.764	83.146
18.	0.00014271	1.655	84.801
19.	0.00012776	1.481	86.282
20.	0.00011957	1.386	87.669
21.	0.00011711	1.358	89.027
22.	0.00010252	1.189	90.215
23.	0.00009396	1.090	91.305
24.	0.00009189	1.066	92.371
25.	0.00007776	0.902	93.272
26.	0.00007342	0.851	94.124
27.	0.00006816	0.790	94.914
28.	0.00006612	0.767	95.680
29.	0.00006218	0.721	96.401
30.	0.00005930	0.688	97.089
31.	0.00005347	0.620	97.709
32.	0.00004638	0.538	98.247
33.	0.00004367	0.506	98.753
34.	0.00003895	0.452	99.205
35.	0.00003665	0.425	99.630
36.	0.00003192	0.370	100.000

Total variance: 0.00862424

Variance of the eigenvalues: 0.0000000022402
Eigenvalue variance scaled by total variance: 0.00003
Eigenvalue variance scaled by total variance and number of variables:
0.06181
Note: throughout all calculations of eigenvalue variances, the
dimensionality used was 2051.
Please double-check because this dimensionality may not be appropriate
for all situations.
Warning: There appear to be fewer PCs than dimensions in shape space.
This can have the following reasons, among others:
-- the degrees of freedom are less than the shape dimensionality (sample
size, etc.).
-- there is complete integration in the data.
Interpret the results cautiously and check for reasons why this is the
case.

Principal Component Coefficients

```
       PC1         PC2         PC3         PC4         PC5         PC6
PC7          PC8         PC9         PC10        PC11        PC12
PC13         PC14        PC15        PC16        PC17        PC18
PC19         PC20        PC21        PC22        PC23        PC24
PC25         PC26        PC27        PC28        PC29        PC30
PC31         PC32        PC33        PC34        PC35        PC36
   x1 -0.007271   0.008589   0.026604  -0.001769   0.023207  -0.026624
     -0.017365   0.013569  -0.033570  -0.013666   0.006643   0.009409
0.024907   -0.003662   0.034095  -0.022599   0.007986   0.002943
0.015597    0.000327  -0.061750   0.019126  -0.030788  -0.049535
0.006659   -0.042855  -0.012433  -0.017414  -0.036233  -0.034180
0.025683   -0.030192  -0.028828   0.009047   0.019308  -0.000790
   y1 -0.029885  -0.004832   0.020534  -0.018617   0.022553  -0.031042
0.014734    0.005921   0.028766   0.025425  -0.037368   0.010215   -
0.008868    0.024007  -0.013343  -0.010463   0.000250   0.009850
```

0.004845 -0.014874 0.021771 0.016492 -0.017275 0.014199 -
0.013933 -0.010531 0.010479 -0.032815 0.041900 0.010935 -
0.003486 0.016783 -0.015946 0.003643 -0.018729 0.036592
z1 0.009055 0.014193 -0.008475 -0.002641 0.013973 -0.007906
-0.060222 -0.041744 0.018953 -0.013812 -0.002205 -0.040463
0.020705 0.022312 -0.000155 0.020296 0.025733 0.004975 -
0.004235 0.030682 -0.047170 0.012376 -0.005222 -0.005101
0.025010 -0.027224 -0.007482 0.030153 -0.015542 0.015520 -
0.020588 -0.009095 -0.020415 0.006632 -0.015081 0.022338
x2 -0.028800 0.006514 0.012145 -0.026591 0.006771 -0.022388
-0.015631 0.024120 -0.002732 -0.016692 -0.008112 -0.010667
0.007679 -0.021217 -0.017429 0.030953 0.014373 -0.035651 -
0.001882 -0.012280 0.015601 0.011542 -0.005662 0.019679 -
0.006476 0.005222 0.025025 0.008694 -0.022624 0.006228 -
0.041720 -0.003334 0.022591 0.034999 0.004634 -0.009101
y2 0.014970 0.004841 -0.005998 -0.012517 0.032066 0.031111
-0.023954 -0.017170 0.007932 -0.031317 -0.001944 0.018634
-0.012112 0.004315 -0.016139 -0.010713 -0.001893 0.007087
0.034163 -0.033674 0.026514 0.020592 0.003238 -0.004025 -
0.003925 -0.009485 0.013892 0.004520 -0.002917 -0.001434 -
0.051053 0.034722 -0.006470 -0.052956 -0.022292 0.011228
z2 0.024890 -0.004673 -0.007898 0.023602 0.007059 -0.017864
-0.004164 0.014596 -0.019959 -0.012366 0.002825 0.013517
0.008899 -0.022889 -0.032130 0.002823 0.017718 0.015942 -
0.003628 -0.014089 0.015751 0.021679 0.003044 -0.000736
0.014579 -0.018393 -0.003804 -0.005784 0.001583 -0.033993
0.012072 -0.004750 0.004691 0.002160 0.020608 -0.008721
x3 -0.030853 0.009452 0.008366 -0.028058 0.011093 -0.013537
-0.018845 0.018015 -0.007107 -0.013141 -0.015411 -0.025426
0.002508 -0.020344 -0.024492 0.025447 0.013602 -0.029193 -
0.001311 -0.010430 0.022988 0.012099 -0.011397 0.019029 -
0.014947 0.006067 0.019950 0.018237 -0.012189 0.002458 -
0.023291 -0.015058 0.030442 0.017772 0.002726 -0.015292
y3 0.016279 0.003916 0.000262 -0.010503 0.033731 0.032614
-0.020872 -0.017467 0.002696 -0.034212 0.000675 0.024904
-0.016769 0.000584 -0.005064 -0.016370 0.007855 0.007461
0.024968 -0.022053 0.031715 0.016719 0.007996 -0.007410 -
0.005564 -0.001124 0.024080 0.002490 -0.007694 -0.007157 -
0.027495 0.051893 -0.016576 -0.052550 -0.021810 0.019467
z3 0.028496 -0.008747 -0.011777 0.024423 0.008623 -0.018602
-0.007094 0.012871 -0.022731 -0.006223 -0.003087 0.014199
-0.000136 -0.019502 -0.028390 0.000797 0.016719 0.015991
-0.007736 -0.024923 0.021785 0.017486 0.001464 -0.001321
0.008554 -0.013187 -0.007096 0.000520 0.002556 -0.021472
0.011327 -0.005729 0.014769 -0.006913 0.018339 0.000428
x4 -0.029943 0.011239 0.006059 -0.027759 0.015071 -0.009290
-0.021395 0.014755 -0.011262 -0.013243 -0.018127 -0.038935
-0.002496 -0.019684 -0.024738 0.020797 0.018367 -0.024582
-0.003888 -0.002817 0.036069 0.010681 -0.010516 0.013969
-0.017987 0.010744 0.023745 0.030014 -0.004487 -0.001325
0.006714 -0.008480 0.029627 0.001061 0.007586 -0.003010
y4 0.014064 0.002047 0.006960 -0.009499 0.033100 0.035670
-0.014902 -0.018713 -0.003120 -0.034816 0.003795 0.035944
-0.016438 -0.006704 0.003040 -0.018950 0.010249 0.010233
0.020672 -0.019335 0.028488 0.013613 0.013596 -0.008641 -
0.007981 0.000747 0.024346 -0.006039 -0.012882 -0.013422 -
0.028639 0.050781 -0.015279 -0.045784 -0.026929 0.010978
z4 0.034443 -0.012494 -0.015289 0.026468 0.010735 -0.019395
-0.009779 0.011187 -0.024909 -0.001190 -0.006126 0.015397
-0.007975 -0.018685 -0.024230 -0.003356 0.017951 0.014035

-0.013710 -0.028853 0.029761 0.012860 0.003328 -0.000762
0.001890 -0.005523 -0.003676 0.004005 0.000996 -0.008003
0.022432 -0.000582 0.021601 -0.017696 0.018805 0.013262
x5 -0.030916 0.011545 0.005595 -0.026891 0.019346 -0.005070
-0.021897 0.011988 -0.016999 -0.013560 -0.019554 -0.047918
-0.006674 -0.020012 -0.024294 0.017179 0.022148 -0.017724
-0.005469 0.001992 0.045255 0.009784 -0.007269 0.010736
-0.021448 0.013040 0.027248 0.034610 0.002737 -0.004368
0.023731 -0.008924 0.035382 -0.010195 0.008938 0.002415
y5 0.012459 0.002515 0.012332 -0.008830 0.029654 0.036974
-0.009257 -0.018214 -0.005312 -0.035711 0.006300 0.040878
-0.017173 -0.010199 0.010634 -0.017926 0.013377 0.007338
0.018219 -0.015925 0.025393 0.009842 0.012687 -0.006290 -
0.010724 0.004360 0.017358 -0.010261 -0.016622 -0.025585 -
0.027061 0.052472 -0.024464 -0.043173 -0.022274 0.007567
z5 0.039301 -0.014951 -0.017817 0.027983 0.011819 -0.018868
-0.012025 0.008809 -0.024827 0.003982 -0.008802 0.017712
-0.013448 -0.018444 -0.021383 -0.006354 0.016007 0.012431
-0.016907 -0.031884 0.034472 0.010286 0.003428 -0.001890
-0.004577 -0.002525 -0.002227 0.006858 0.001726 0.001904
0.026100 0.001621 0.028823 -0.024150 0.016929 0.021455
x6 -0.032125 0.011594 0.006249 -0.024762 0.022541 -0.000931
-0.022940 0.009331 -0.023467 -0.013814 -0.020004 -0.053446
-0.011023 -0.019531 -0.024328 0.013905 0.023321 -0.013351
-0.007759 0.006920 0.047502 0.010615 -0.006648 0.012526
-0.024090 0.013581 0.030772 0.039355 0.005804 -0.001529
0.038902 -0.011825 0.035902 -0.018699 0.009505 0.006322
y6 0.013945 0.001936 0.015393 -0.009157 0.026201 0.038439
-0.003827 -0.016658 -0.007647 -0.035488 0.009133 0.044105
-0.015310 -0.005091 0.014483 -0.022477 0.014921 0.008554
0.012239 -0.013711 0.024844 0.005473 0.011555 -0.010745 -
0.012547 0.003693 0.016501 -0.010500 -0.020193 -0.021521 -
0.020250 0.057273 -0.037795 -0.044696 -0.026965 0.001083
z6 0.042889 -0.015440 -0.019279 0.029342 0.012736 -0.018605
-0.013049 0.006012 -0.022862 0.008017 -0.011034 0.017449
-0.018587 -0.017743 -0.016976 -0.007009 0.015158 0.011787
-0.016576 -0.034842 0.038682 0.005579 0.003526 -0.004926
-0.011216 0.003116 -0.003989 0.005670 0.006237 -0.001252
0.020682 0.005023 0.036695 -0.027333 0.016373 0.032064
x7 -0.033225 0.011243 0.007714 -0.023405 0.025830 0.002177
-0.023565 0.007709 -0.029589 -0.014994 -0.019463 -0.057916
-0.014643 -0.016083 -0.023900 0.009787 0.025087 -0.006193
-0.009469 0.011059 0.050390 0.008436 -0.007359 0.010906
-0.025724 0.016189 0.035802 0.042346 0.009144 -0.002418
0.047257 -0.013535 0.031480 -0.024555 0.008731 0.013412
y7 0.012910 0.002864 0.018970 -0.009288 0.023430 0.041096
0.004225 -0.015547 -0.007556 -0.032101 0.008240 0.049462 -
0.011338 -0.005308 0.017175 -0.026381 0.012226 0.009707
0.003666 -0.011486 0.022971 0.007187 0.006310 -0.010441 -
0.009706 0.004180 0.015204 -0.013306 -0.024401 -0.027781 -
0.020938 0.050491 -0.042025 -0.044021 -0.025668 -0.008786
z7 0.046573 -0.015031 -0.019807 0.032185 0.013756 -0.017165
-0.014046 0.002749 -0.021089 0.011989 -0.012625 0.017501
-0.022711 -0.015527 -0.013312 -0.008411 0.012375 0.009541
-0.016702 -0.035702 0.038744 0.001573 0.001876 -0.006131
-0.016750 0.006592 -0.000750 0.005584 0.008241 0.001221
0.015983 0.007249 0.040139 -0.031002 0.016937 0.036166
x8 -0.034835 0.010488 0.010787 -0.022318 0.029397 0.004423
-0.024289 0.006487 -0.035761 -0.016222 -0.017513 -0.059544
-0.018406 -0.010780 -0.022739 0.005613 0.027069 0.001623

-0.010896 0.014194 0.049076 0.005745 -0.007856 0.007495
-0.026800 0.018290 0.043083 0.042584 0.010903 -0.005565
0.049664 -0.013262 0.021832 -0.027017 0.008033 0.025855
y8 0.013556 0.004059 0.022467 -0.004801 0.015458 0.037229
0.009151 -0.008918 -0.006128 -0.033168 0.022815 0.058626 -
0.015429 -0.004528 0.022491 -0.013898 0.016369 -0.006535
0.004418 -0.007560 0.013622 0.005574 0.008912 -0.011840 -
0.008596 0.004046 0.012478 -0.018947 -0.022062 -0.031167 -
0.023525 0.046933 -0.039094 -0.041677 -0.021160 -0.010223
z8 0.049527 -0.015210 -0.019701 0.034163 0.014963 -0.014043
-0.015329 -0.002311 -0.018598 0.017251 -0.014976 0.017637
-0.024998 -0.011501 -0.009405 -0.008373 0.008038 0.009516
-0.018269 -0.035152 0.036962 -0.001091 -0.001606 -0.007221
-0.019670 0.005961 0.001125 0.004916 0.008761 0.002480
0.010177 0.008726 0.041032 -0.033505 0.019663 0.037070
x9 -0.036713 0.009601 0.015872 -0.020230 0.033401 0.005532
-0.024086 0.004933 -0.043051 -0.018804 -0.015628 -0.058707
-0.022111 -0.006589 -0.020178 0.001064 0.027551 0.011522
-0.012766 0.017181 0.043801 0.002515 -0.006173 0.005210
-0.026452 0.020819 0.049480 0.040465 0.009731 -0.007204
0.043578 -0.013863 0.012185 -0.024878 0.003644 0.040443
y9 0.014401 0.003891 0.023286 -0.006555 0.009364 0.035095
0.020461 -0.004574 -0.005215 -0.033107 0.024512 0.061358 -
0.010478 -0.002824 0.022917 -0.015106 0.013896 -0.007816
0.002049 -0.003442 0.015861 0.004225 0.011149 -0.017592 -
0.008286 -0.000677 0.002927 -0.021964 -0.020744 -0.036903 -
0.026639 0.043370 -0.045693 -0.038711 -0.022916 -0.012882
z9 0.052714 -0.014164 -0.018687 0.037913 0.016092 -0.010640
-0.014861 -0.007382 -0.016177 0.020101 -0.016334 0.016194
-0.025819 -0.006662 -0.005340 -0.010911 0.004512 0.009180
-0.017424 -0.031501 0.036952 -0.004333 -0.004354 -0.009541
-0.024183 0.006395 0.005636 0.003878 0.009645 0.002207
0.004217 0.012464 0.036256 -0.031487 0.019165 0.036629
x10 -0.038041 0.008084 0.020937 -0.018076 0.036627
0.005319 -0.022504 0.005569 -0.049666 -0.021864 -0.011107 -
0.055150 -0.025303 0.000511 -0.017599 -0.004752 0.028757
0.022665 -0.016175 0.019268 0.037932 -0.002494 -0.005318
0.002643 -0.023520 0.025554 0.061421 0.037136 0.006606 -
0.007448 0.037479 -0.011746 -0.000695 -0.017302 0.005617
0.052908
y10 0.015412 0.006151 0.022716 -0.003176 -0.001596
0.030244 0.031293 0.003204 -0.002241 -0.033426 0.035752
0.067331 -0.009164 -0.005605 0.022078 -0.006727 0.011016 -
0.023663 0.002642 0.000171 0.007940 0.006539 0.015981 -
0.009845 -0.005861 -0.000479 -0.008547 -0.028196 -0.016650 -
0.038831 -0.026643 0.031029 -0.035022 -0.031708 -0.014120 -
0.025794
z10 0.055137 -0.011558 -0.017086 0.041636 0.016447 -
0.006102 -0.013858 -0.013348 -0.012238 0.023529 -0.017369
0.014547 -0.024934 -0.004297 -0.001768 -0.012289 -0.000732
0.007917 -0.016081 -0.026930 0.033085 -0.006360 -0.008073 -
0.009054 -0.026193 0.005470 0.008633 0.001026 0.011311 -
0.001046 -0.003515 0.015072 0.034796 -0.027685 0.023796
0.031870
x11 -0.038934 0.006254 0.026288 -0.015925 0.038900
0.005985 -0.020917 0.007417 -0.058134 -0.025287 -0.007443 -
0.049558 -0.025772 0.003738 -0.014288 -0.009737 0.026035
0.030845 -0.018303 0.020862 0.032741 -0.005517 -0.007366
0.002432 -0.022535 0.027729 0.071438 0.029838 0.001557 -

0.002080 0.032787 -0.009463 -0.014927 -0.006828 0.006080
0.062647
y11 0.016697 0.008209 0.020603 -0.002209 -0.013705
0.026641 0.041718 0.012528 0.002460 -0.033206 0.043386
0.070756 -0.004776 -0.009612 0.018601 0.002897 0.005660 -
0.042055 0.004662 0.001916 0.006699 0.010553 0.017139 -
0.005539 -0.005091 -0.004643 -0.024926 -0.034906 -0.008578 -
0.036402 -0.022761 0.019127 -0.026861 -0.030754 -0.009037 -
0.040429
z11 0.056709 -0.007496 -0.014872 0.044907 0.016068 -
0.000668 -0.012023 -0.019917 -0.006297 0.025882 -0.018024
0.010988 -0.022288 -0.002409 -0.000490 -0.014135 -0.007825
0.006086 -0.014279 -0.021117 0.028140 -0.007692 -0.012436 -
0.011004 -0.027071 0.002510 0.010710 -0.002768 0.015313 -
0.006479 -0.012148 0.018218 0.032360 -0.023114 0.028437
0.026816
x12 -0.033971 0.006467 0.025458 -0.009709 0.037631
0.008161 -0.011574 0.014837 -0.056949 -0.030312 -0.002609 -
0.041192 -0.027652 0.001645 -0.013469 -0.007153 0.015572
0.022856 -0.006829 0.021947 0.016196 -0.006914 0.000613
0.009360 -0.023807 0.012514 0.041277 0.012809 0.008373 -
0.002603 0.013884 -0.003676 -0.020325 -0.000796 0.005375
0.049554
y12 0.011751 0.008914 0.021948 -0.004513 -0.027880
0.019092 0.051667 0.011665 -0.000061 -0.029777 0.047886
0.062400 0.005113 -0.019783 0.018196 -0.000800 -0.004309 -
0.036389 -0.007203 -0.009988 0.011116 0.002764 0.017942 -
0.006987 -0.002435 -0.000129 -0.040102 -0.026866 -0.008741 -
0.039740 -0.010364 0.005310 -0.018562 -0.022765 -0.008329 -
0.031857
z12 0.054485 -0.006225 -0.011920 0.049790 0.009551
0.008694 -0.010063 -0.016798 -0.004030 0.023750 -0.016771
0.014123 -0.017107 -0.002012 0.004171 -0.008833 -0.018401 -
0.000858 -0.008771 -0.014764 0.021136 -0.005007 -0.009165 -
0.008470 -0.024571 -0.001477 0.009755 -0.006196 0.011190 -
0.019376 -0.015475 0.016501 0.025493 -0.013595 0.023716
0.015795
x13 -0.029596 0.007818 0.026449 -0.002705 0.035357
0.009341 -0.002446 0.022239 -0.057728 -0.034374 0.002048 -
0.032471 -0.024901 -0.003456 -0.012508 -0.005341 0.006013
0.013456 0.005621 0.018938 -0.000129 -0.011313 0.005223
0.016586 -0.025973 0.004746 0.019139 -0.007449 0.015122
0.004763 -0.000353 -0.001650 -0.017106 0.008702 0.006932
0.035982
y13 0.007490 0.011349 0.024816 -0.005545 -0.038598
0.009236 0.058334 0.012812 -0.001248 -0.026133 0.050223
0.054239 0.017329 -0.024885 0.016047 -0.003540 -0.006968 -
0.034988 -0.011887 -0.018829 0.014863 -0.007978 0.015746 -
0.012469 -0.000301 0.008773 -0.042269 -0.024650 -0.003842 -
0.036356 0.000683 -0.006423 -0.008748 -0.013212 -0.005315 -
0.020796
z13 0.052242 -0.003839 -0.009238 0.054250 0.002337
0.015360 -0.006728 -0.014522 -0.003412 0.020169 -0.013425
0.015974 -0.012121 -0.003155 0.008025 -0.006616 -0.026678 -
0.009187 -0.005550 -0.010201 0.016146 -0.004345 -0.007674 -
0.008746 -0.021477 -0.002492 0.012486 -0.012717 0.011991 -
0.025188 -0.016604 0.010047 0.019995 -0.004681 0.022916
0.012837
x14 -0.025707 0.006238 0.026303 -0.001877 0.030512
0.012764 0.003727 0.025056 -0.058781 -0.032191 0.001819 -

0.026445	-0.021355	-0.006448	-0.013073	-0.007990	-0.004385	
0.018621	0.010901	0.013477	-0.008698	-0.014049	0.008628	
0.027110	-0.033997	-0.004050	0.003576	-0.014083	0.016772	-
0.000147	-0.004851	0.001088	-0.016570	0.016848	0.003999	
0.023462						
y14	0.003658	0.011327	0.025776	-0.010591	-0.048836	
0.001714	0.061820	0.011005	-0.001368	-0.019454	0.049683	
0.043777	0.023637	-0.026934	0.012557	-0.007435	-0.009099	-
0.025964	-0.019893	-0.026690	0.023276	-0.015675	0.014840	-
0.013246	-0.004017	0.013834	-0.041548	-0.014694	-0.002209	-
0.038788	0.014330	-0.013700	-0.001085	-0.007125	-0.006645	-
0.012033						
z14	0.050091	-0.002952	-0.006223	0.054855	-0.005058	
0.021711	-0.004425	-0.014100	-0.003512	0.019401	-0.014233	
0.015653	-0.008441	-0.003486	0.008108	-0.005698	-0.033917	-
0.011279	-0.005217	-0.006630	0.015071	-0.005191	-0.005380	-
0.007383	-0.023663	-0.006495	0.017867	-0.009203	0.009334	-
0.035523	-0.013733	0.008075	0.017340	0.000934	0.017125	
0.010556						
x15	-0.021029	0.006849	0.027949	0.000193	0.022146	
0.010286	0.009028	0.031503	-0.059835	-0.029663	0.009925	-
0.024297	-0.020277	-0.009989	-0.008354	-0.003273	-0.006782	
0.012231	0.012530	0.005585	-0.015027	-0.021481	0.012595	
0.032737	-0.033044	-0.002347	-0.006428	-0.020413	0.023421	-
0.005327	-0.008247	0.002886	-0.011554	0.023560	0.002947	
0.015659						
y15	0.000305	0.011394	0.026322	-0.014535	-0.057858	-
0.006386	0.062897	0.009196	-0.001134	-0.013973	0.051987	
0.032824	0.025381	-0.029246	0.012239	-0.007079	-0.008112	-
0.020347	-0.026769	-0.033249	0.028590	-0.021718	0.013947	-
0.013600	-0.004944	0.019653	-0.038689	-0.007573	-0.000103	-
0.038412	0.023846	-0.020432	0.006269	-0.001494	-0.007165	-
0.002991						
z15	0.048982	-0.001461	-0.002243	0.055688	-0.012444	
0.024815	-0.001967	-0.012270	-0.005181	0.018407	-0.012702	
0.013621	-0.004881	-0.002966	0.008513	-0.001160	-0.037809	-
0.016643	-0.005678	-0.002964	0.013994	-0.008222	-0.002968	-
0.005934	-0.023752	-0.005980	0.023712	-0.005127	0.009900	-
0.039836	-0.008890	0.005138	0.017866	0.005302	0.015299	
0.010393						
x16	-0.016596	0.007264	0.029778	0.001148	0.014012	
0.010334	0.013643	0.033622	-0.061376	-0.025467	0.012410	-
0.019737	-0.015796	-0.009077	-0.007295	-0.001956	-0.008565	
0.015332	0.016016	-0.003538	-0.023186	-0.028064	0.009067	
0.043079	-0.030243	-0.004834	-0.018078	-0.023942	0.020218	-
0.011525	-0.007260	0.002104	-0.010982	0.029354	0.004614	
0.001520						
y16	-0.002699	0.011809	0.027281	-0.017007	-0.062848	-
0.013554	0.062006	0.007894	0.000011	-0.007560	0.048144	
0.022867	0.029691	-0.026487	0.006284	-0.008078	-0.007022	-
0.014531	-0.026110	-0.035256	0.028356	-0.025914	0.010583	-
0.009372	-0.005972	0.019525	-0.035317	-0.001599	-0.003282	-
0.036623	0.033480	-0.026616	0.012959	0.003663	-0.009726	
0.000220						
z16	0.048132	-0.000496	0.000492	0.055714	-0.019934	
0.027747	0.001086	-0.012837	-0.007775	0.017098	-0.011682	
0.011836	-0.001324	-0.001685	0.008136	0.002815	-0.041000	-
0.015739	-0.007475	-0.001820	0.013427	-0.010854	-0.006572	-
0.003058	-0.023252	-0.007007	0.025189	-0.002310	0.008745	-

0.040552 -0.003147 -0.003749 0.013004 0.009592 0.014875
0.009012
x17 -0.012589 0.006178 0.031936 0.002835 0.004415
0.009862 0.015847 0.035902 -0.067131 -0.016371 0.012974 -
0.015705 -0.015967 -0.010760 -0.006740 -0.000628 -0.009524
0.013745 0.011583 -0.011915 -0.025078 -0.034617 0.008738
0.051589 -0.026246 -0.011370 -0.012480 -0.026366 0.010250 -
0.013647 0.001711 0.001907 -0.005515 0.029284 0.006855 -
0.014256
y17 -0.005738 0.010810 0.027345 -0.020112 -0.066557 -
0.019533 0.058144 0.005256 0.000966 -0.001484 0.044258
0.011515 0.030034 -0.023003 0.001945 -0.007621 -0.005691 -
0.007004 -0.026184 -0.035425 0.026746 -0.026927 0.007131 -
0.003902 -0.008290 0.015712 -0.026137 0.005425 -0.009618 -
0.030648 0.044028 -0.028937 0.016973 0.007435 -0.014520
0.003645
z17 0.047675 -0.000293 0.004014 0.054994 -0.027208
0.028645 0.003704 -0.012823 -0.012199 0.018444 -0.012623
0.008109 0.001103 -0.000987 0.005853 0.006102 -0.042303 -
0.014726 -0.010296 0.001091 0.014370 -0.014778 -0.008980
0.000775 -0.025520 -0.007792 0.031649 0.004649 0.006811 -
0.040284 0.004342 -0.010341 0.012785 0.012574 0.015540
0.006958
x18 -0.009130 0.003931 0.032658 0.004871 -0.008545
0.008120 0.018303 0.038968 -0.071989 -0.008163 0.015716 -
0.014784 -0.016967 -0.010331 -0.006687 0.001775 -0.012289
0.012773 0.008400 -0.018754 -0.027482 -0.039621 0.002556
0.052978 -0.020706 -0.020941 -0.011646 -0.021083 -0.001708 -
0.011249 0.011334 0.008951 -0.002936 0.027593 0.004088 -
0.031264
y18 -0.008596 0.009275 0.026927 -0.023800 -0.068129 -
0.024803 0.052036 0.002325 0.002663 0.005579 0.038028 -
0.001063 0.028743 -0.016484 -0.004010 -0.006856 -0.002647
0.002518 -0.023504 -0.033560 0.024795 -0.025807 0.002859
0.003817 -0.010876 0.008963 -0.015813 0.010609 -0.017781 -
0.024248 0.054651 -0.031026 0.019407 0.009837 -0.021186
0.009036
z18 0.047570 -0.000047 0.007114 0.053665 -0.034831
0.027768 0.006963 -0.013296 -0.017285 0.019808 -0.013736
0.001792 0.004914 0.000742 0.001544 0.008164 -0.042238 -
0.010180 -0.013499 0.004613 0.016041 -0.019204 -0.016045
0.003998 -0.027482 -0.007986 0.037233 0.013991 0.005628 -
0.035989 0.011233 -0.021817 0.010682 0.017082 0.014660
0.006494
x19 -0.006511 0.002032 0.035215 0.004290 -0.021169
0.004895 0.017988 0.042376 -0.075621 0.003431 0.013081 -
0.018196 -0.012990 -0.011650 -0.004090 0.000844 -0.010899
0.014538 0.002393 -0.028425 -0.026520 -0.048518 -0.004963
0.054683 -0.013405 -0.022658 -0.005293 -0.014728 -0.012797 -
0.021580 0.029858 0.007210 -0.005853 0.026548 0.004314 -
0.035270
y19 -0.010734 0.007669 0.026499 -0.026334 -0.066082 -
0.029788 0.043518 0.000015 0.005067 0.013769 0.028675 -
0.012620 0.027329 -0.005494 -0.013297 -0.005764 0.001633
0.011728 -0.016583 -0.026804 0.020844 -0.022493 -0.002183
0.014180 -0.014055 -0.001656 -0.000181 0.016575 -0.027338 -
0.014828 0.063003 -0.031475 0.022596 0.011512 -0.029895
0.008533
z19 0.047707 -0.000860 0.009760 0.050425 -0.042798
0.026372 0.010476 -0.015366 -0.023761 0.020272 -0.014140 -

0.006619	0.006906	0.001598	-0.003962	0.010295	-0.040654	-
0.002496	-0.019554	0.005810	0.020751	-0.023531	-0.023506	
0.007568	-0.028545	-0.006328	0.044205	0.024953	0.003897	-
0.028516	0.021229	-0.034817	0.009371	0.018810	0.013212	
0.009998						
x20	-0.002538	-0.003242	0.037511	0.002121	-0.034724	
0.001074	0.017949	0.043580	-0.080718	0.011904	0.015754	-
0.023684	-0.004924	-0.007111	-0.006561	-0.000250	-0.013903	
0.022693	-0.006634	-0.038155	-0.019703	-0.050244	-0.015180	
0.055216	-0.004087	-0.022751	0.013407	-0.001197	-0.025615	-
0.026442	0.056616	0.003337	-0.003617	0.023221	-0.001444	-
0.050965						
y20	-0.013108	0.007172	0.025878	-0.029167	-0.061877	-
0.033963	0.032183	-0.003192	0.008252	0.024040	0.016097	-
0.025535	0.023034	0.005590	-0.020041	-0.004529	0.009444	
0.020669	-0.006272	-0.019481	0.011807	-0.019962	-0.007475	
0.028464	-0.018562	-0.015204	0.016151	0.021132	-0.040897	-
0.011170	0.067481	-0.024989	0.024154	0.013380	-0.035162	
0.004465						
z20	0.047505	-0.001310	0.012465	0.045747	-0.049741	
0.023990	0.012734	-0.017806	-0.031727	0.021919	-0.016951	-
0.016062	0.007640	0.002123	-0.010223	0.012875	-0.035881	
0.006931	-0.026828	0.005735	0.026132	-0.030076	-0.030855	
0.012680	-0.032014	-0.006213	0.049468	0.035912	0.002897	-
0.014540	0.035012	-0.044925	0.006361	0.017051	0.014017	
0.022659						
x21	-0.003856	-0.009114	0.043142	0.006106	-0.042190	
0.001021	0.019652	0.045026	-0.079896	0.015467	0.017791	-
0.018639	0.001602	-0.010914	0.001242	-0.005519	-0.004892	
0.016424	-0.013736	-0.026055	-0.009736	-0.034930	-0.022379	
0.028210	0.002878	-0.007058	0.015918	0.002541	-0.033002	-
0.012329	0.042352	-0.009027	0.004120	0.020741	-0.007796	-
0.016099						
y21	-0.019672	0.005880	0.020940	-0.029921	-0.039943	-
0.035655	0.021528	-0.007847	0.020675	0.026057	-0.000672	-
0.019904	0.019246	0.007765	-0.019312	-0.004920	0.019879	
0.011020	0.003384	-0.006467	0.007159	-0.006324	-0.000776	
0.019618	-0.023643	-0.006619	0.013655	0.012731	-0.031401	
0.004417	0.039285	-0.009550	0.014717	0.008903	-0.018796	
0.013965						
z21	0.040141	-0.000445	0.010321	0.037802	-0.053964	
0.021116	0.008865	-0.021513	-0.017333	0.025409	-0.021222	-
0.018996	0.012556	0.000914	-0.010001	0.019445	-0.026162	
0.009340	-0.036492	0.011618	0.026261	-0.016910	-0.034302	
0.005875	-0.017600	0.008998	0.028362	0.034681	-0.003049	-
0.004210	0.014518	-0.037426	0.008595	0.012177	-0.001114	
0.017292						
x22	-0.008221	-0.014357	0.047449	0.007557	-0.047359	-
0.000047	0.020500	0.041223	-0.075894	0.014744	0.017168	-
0.014000	0.004840	-0.017799	0.012529	-0.018348	0.004379	
0.010185	-0.022193	-0.008722	-0.007977	-0.021467	-0.018524	
0.005758	0.008596	0.010291	0.017998	0.001251	-0.052770	-
0.001489	0.022775	-0.003902	0.022972	0.009980	-0.014700	
0.003482						
y22	-0.022235	0.005458	0.017402	-0.028606	-0.021886	-
0.038201	0.011108	-0.010008	0.030198	0.026713	-0.014203	-
0.016831	0.017324	0.012812	-0.022091	-0.004100	0.024534	
0.003857	0.015088	-0.001371	0.002319	0.004178	0.001976	
0.016994	-0.029480	-0.004854	0.014544	0.010996	-0.016404	

0.009461 0.023390 -0.005289 -0.000925 0.009941 0.001169
0.009033
z22 0.034036 0.000417 0.008409 0.031390 -0.054864
0.017891 0.005166 -0.023673 -0.005767 0.027666 -0.023485 -
0.021561 0.017247 0.001107 -0.011592 0.028006 -0.018282
0.012525 -0.041682 0.014674 0.028043 -0.004731 -0.042180
0.002846 -0.004346 0.019265 0.006642 0.034640 -0.002722
0.010339 -0.002317 -0.039916 0.005829 0.012597 -0.009508
0.021704
x23 -0.011409 -0.018367 0.050130 0.007696 -0.050438 -
0.003965 0.021531 0.035821 -0.066404 0.012309 0.014150 -
0.009662 0.009711 -0.023285 0.017759 -0.023764 0.008558
0.004286 -0.024554 0.005218 -0.010231 -0.003079 -0.020831 -
0.011019 0.016859 0.020176 0.020590 0.001233 -0.064345
0.012734 0.004931 -0.005520 0.038236 0.003727 -0.017473
0.018372
y23 -0.024314 0.004208 0.014338 -0.025874 -0.006390 -
0.037735 0.002996 -0.010416 0.033600 0.027907 -0.022345 -
0.013716 0.013400 0.014604 -0.019153 -0.005784 0.029766 -
0.001239 0.022345 -0.000611 0.001065 0.005755 0.005627
0.010698 -0.033878 -0.001289 0.010261 0.014478 -0.002883
0.005520 0.007310 -0.000435 -0.019184 0.010578 0.015233
0.009781
z23 0.029013 0.001225 0.007200 0.026516 -0.055667
0.013985 0.000828 -0.026161 0.001148 0.028459 -0.024815 -
0.024239 0.020640 0.000176 -0.010378 0.034286 -0.008920
0.016490 -0.048295 0.013914 0.030108 -0.002438 -0.045671
0.003375 0.003250 0.027014 -0.010985 0.030362 0.000179
0.016874 -0.014366 -0.039060 -0.001033 0.013508 -0.015747
0.022764
x24 -0.012229 -0.021563 0.050500 0.008277 -0.053425 -
0.010399 0.022878 0.027630 -0.057581 0.011820 0.010634 -
0.006016 0.015827 -0.030693 0.023424 -0.024625 0.010221
0.000363 -0.025739 0.013414 -0.013594 0.005663 -0.026869 -
0.022807 0.019137 0.026232 0.018484 0.005873 -0.061773
0.024329 -0.010124 -0.010206 0.041213 0.001554 -0.012828
0.028313
y24 -0.028320 0.004423 0.013662 -0.022583 0.011193 -
0.033317 -0.005137 -0.006919 0.037365 0.027446 -0.028732 -
0.007806 0.008390 0.023282 -0.017903 -0.009109 0.036545 -
0.009610 0.031177 0.005994 0.001943 0.014797 0.007717
0.004005 -0.027509 0.002837 0.011234 0.014781 -0.003523 -
0.000953 -0.004966 0.005673 -0.026653 0.005655 0.014279
0.008445
z24 0.022851 0.003605 0.008077 0.022027 -0.052157
0.011510 -0.004787 -0.029060 0.009966 0.026467 -0.026764 -
0.027691 0.020247 0.004810 -0.011995 0.040724 0.003584
0.015605 -0.051565 0.016646 0.033266 0.006680 -0.047173
0.005627 0.014708 0.033885 -0.017416 0.020307 -0.007138
0.022523 -0.025165 -0.033410 -0.002800 0.007523 -0.028299
0.015993
x25 -0.011770 -0.020673 0.049141 0.008318 -0.054053 -
0.015126 0.023085 0.018855 -0.046032 0.011638 0.003169 -
0.004432 0.019880 -0.031402 0.025092 -0.017472 0.013783 -
0.005507 -0.028871 0.018358 -0.015289 0.005927 -0.035351 -
0.023625 0.021997 0.037091 0.017138 0.005524 -0.059232
0.034911 -0.021387 -0.013973 0.035912 -0.017516 -0.011312
0.032854
y25 -0.033361 0.001129 0.010817 -0.020280 0.023180 -
0.031237 -0.014578 -0.004052 0.036512 0.024618 -0.025118 -

0.000014	-0.000545	0.027346	-0.009913	-0.016582	0.035044	-
0.007502	0.029277	0.011733	-0.001686	0.026587	0.021745	-
0.008245	-0.031515	0.002593	0.007416	0.008515	0.002786	-
0.006307	-0.001800	0.004756	-0.026320	0.023701	0.021001	
0.016359						
z25	0.015526	0.002906	0.007063	0.016490	-0.050482	
0.004718	-0.010328	-0.037769	0.016506	0.023426	-0.022634	-
0.032786	0.018911	0.002072	-0.012748	0.039935	0.013973	
0.014444	-0.049596	0.021603	0.028484	0.019213	-0.042191	
0.001186	0.017944	0.032194	-0.023419	0.016880	-0.010125	
0.032422	-0.037116	-0.027012	0.001097	0.012535	-0.034481	
0.011610						
x26	-0.012004	-0.020056	0.047539	0.009002	-0.056472	-
0.022629	0.021096	0.003598	-0.030467	0.014160	-0.005875	-
0.005462	0.019817	-0.028091	0.021725	-0.004362	0.017866	-
0.009814	-0.026507	0.022924	-0.019057	0.011216	-0.040132	-
0.026967	0.025521	0.037098	0.012635	0.013430	-0.051821	
0.040545	-0.032975	-0.018378	0.031100	-0.024926	-0.016707	
0.029064						
y26	-0.036589	0.001982	0.011879	-0.016239	0.040016	-
0.027061	-0.019673	0.009865	0.028980	0.022481	-0.024194	
0.010708	0.005539	0.037021	-0.009037	-0.030904	0.029557	-
0.017203	0.037361	0.023747	-0.013665	0.027950	0.029792	-
0.015152	-0.021757	0.002319	0.009737	0.006217	0.002992	-
0.022533	-0.006263	0.005633	-0.019640	0.033355	0.020741	
0.019201						
z26	0.009793	0.004939	0.008728	0.014654	-0.039949	-
0.003388	-0.013601	-0.041121	0.016634	0.014726	-0.022407	-
0.034418	0.028377	0.001647	-0.016813	0.031024	0.022336	
0.002171	-0.043921	0.032583	0.020572	0.021792	-0.038039	
0.006663	0.021012	0.036042	-0.015710	0.001233	-0.017730	
0.035626	-0.042264	-0.016305	0.001603	0.016701	-0.042416	
0.004781						
x27	-0.011681	-0.018441	0.045238	0.011710	-0.057907	-
0.032929	0.017986	-0.016020	-0.014726	0.015486	-0.017315	-
0.007141	0.016150	-0.026183	0.016887	0.010240	0.021971	-
0.016649	-0.025943	0.025790	-0.019384	0.014158	-0.044245	-
0.024540	0.028013	0.034395	0.013228	0.020049	-0.042565	
0.041416	-0.040530	-0.020899	0.020314	-0.022828	-0.026457	
0.021923						
y27	-0.038001	0.000394	0.010940	-0.017771	0.047586	-
0.018112	-0.022701	0.024032	0.018235	0.016670	-0.011047	
0.020442	-0.001289	0.036367	0.002880	-0.042600	0.017760	-
0.009680	0.032891	0.020681	-0.021040	0.030149	0.033616	-
0.015373	-0.017289	-0.000803	0.005160	0.007300	0.002406	-
0.032388	-0.000813	-0.003946	-0.014539	0.034728	0.034919	
0.019781						
z27	0.007303	0.004529	0.010806	0.008811	-0.031141	-
0.009108	-0.016707	-0.047706	0.012862	0.002186	-0.016303	-
0.033929	0.031633	-0.002440	-0.014619	0.029099	0.025495	
0.002524	-0.043524	0.030950	0.011999	0.023576	-0.038486	
0.020632	0.025078	0.033310	-0.011381	-0.011025	-0.025456	
0.043717	-0.041586	-0.012251	0.001061	0.016510	-0.034864	-
0.011199						
x28	-0.010064	-0.014736	0.041328	0.013299	-0.057615	-
0.045245	0.013952	-0.037726	0.003926	0.015868	-0.030403	-
0.010939	0.011236	-0.025586	0.012079	0.032311	0.026590	-
0.022804	-0.024642	0.023553	-0.020409	0.010058	-0.050107	-
0.012288	0.029220	0.028009	0.014828	0.026063	-0.028384	

0.040339 -0.046551 -0.019994 0.004118 -0.023327 -0.031134
0.005704
y28 -0.040683 -0.000636 0.009244 -0.016056 0.053288 -
0.007413 -0.023438 0.041051 0.007557 0.009833 0.003015
0.029068 -0.010285 0.038468 0.018027 -0.051985 0.005333 -
0.001971 0.026851 0.013170 -0.021603 0.028621 0.041452 -
0.018839 -0.014565 -0.003124 -0.005009 0.005592 0.001788 -
[truncated: 441,155 more chars]
